# Recent Advances in Heterocyclic Nanographenes and Other Polycyclic Heteroaromatic Compounds

**DOI:** 10.1021/acs.chemrev.1c00449

**Published:** 2021-12-01

**Authors:** Arseni Borissov, Yogesh Kumar Maurya, Liliia Moshniaha, Wai-Shing Wong, Marika Żyła-Karwowska, Marcin Stępień

**Affiliations:** Wydział Chemii, Uniwersytet Wrocławski, ul. F. Joliot-Curie 14, 50-383 Wrocław, Poland

## Abstract

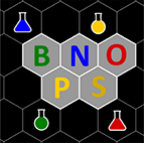

This review surveys recent progress in the chemistry of polycyclic heteroaromatic molecules with a focus on structural diversity and synthetic methodology. The article covers literature published during the period of 2016–2020, providing an update to our first review of this topic (*Chem. Rev.***2017**, *117* (4), 3479–3716).

## 1 Introduction

### 1.1 Structure and Scope

**Figure 1:**
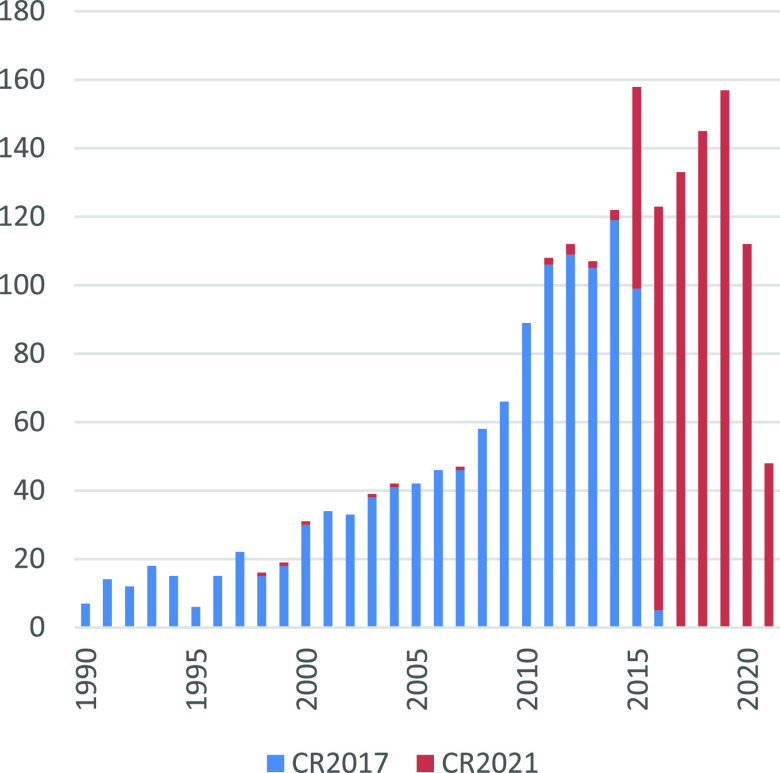
Partial citation timeline of CR2017 and the new references included in the present Review.

Since the publication of the first part of this review (denoted CR2017),^1^ the field of heterocyclic nanographenes and related polycyclic heteroaromatic systems (PHAs) has grown substantially. CR2017 surveyed relevant research published until the end of 2015 (over 1600 references). Soon after publication, it became apparent that, to keep up with the rapid progress of the area, an update might need to be prepared in the next few years. The present review covers relevant literature published since late 2015 until March 2021 and includes close to 800 references (Figure 1). Again, we mainly focus on atomically precise synthetic methods. Accordingly, the present update covers solution syntheses of small molecules and structurally well-defined polymers consisting of extensively fused subunits. Atomically resolved on-surface chemistry is also included.

The scope of the present update retains the selection criteria adopted in CR2017. Briefly, the material is restricted to ring frameworks containing at least (a) one heteroatom, (b) one peri fusion point, (c) five fused rings, and (d) 20 π-conjugated atoms. By way of convention, all tri- and divalent atomic centers are considered to be π-conjugated, and no attempt was generally made to quantify the extent of p-orbital overlap. Curved aromatics are within the scope of the review; however, some highly twisted rings with evidently interrupted π-conjugation were omitted.

The classification of ring frameworks used in this review reflects the decreasing extent of benzenoid (graphene-like) fusion in the PHAs. However, because the review covers a range of research topics and subfields that are interrelated in a complex fashion, it was not always possible to classify such diverse material in a chemically relevant way. Thus, while some of these fields are presented in a single subsection, others may be discussed in more than one place. In such cases, we provide cross references to assist the reader in finding related work.

The present review generally retains the section structure of CR2017. This should help the reader in appreciating the progress in individual areas and in locating background information for the newest results. In some cases, the lowest hierarchy level was modified to better reflect the ongoing research in the field.

To keep the review as concise as possible, we only cite relevant original papers that have not been included in the first part of this review. It should also be noted that the work reviewed herein was often based on earlier (or parallel) developments of related carbocyclic systems or smaller heterocyclic molecules that do not fit into the scope of this review. The corresponding papers are generally not included in this review, but they may be easily identified in the cited references.

### 1.2 Recent Developments

**Chart 1:**
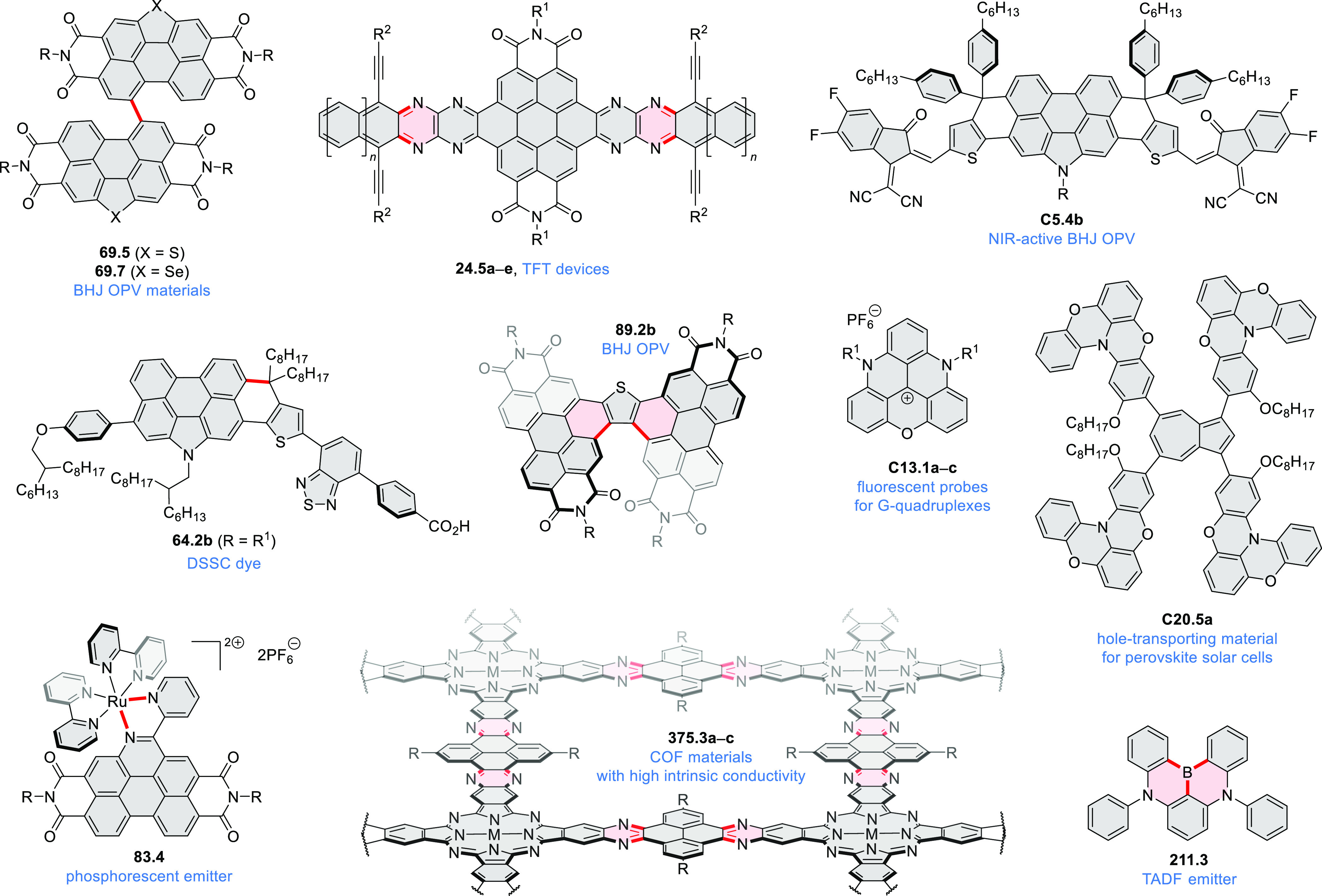
Examples of PHA-Based Materials

The most recent developments of PHA chemistry have been motivated by advances in synthetic methodology and by the application potential of heteroatom-doped π-conjugated systems. Representative recent examples of PHA-based materials are highlighted in Chart 1 (for more information, check the corresponding sections). Several bay-annulated perylene diimide (PDI) derivatives, often with nonplanar cores, have emerged as high-performance materials for bulk-heterojunction photovoltaics (**69.5,7**, **C5.4b**, and **89.2b**) and as effective DSSC dyes (**64.2b**, cf. Sections 3.2 and 3.3). The use of a π-extended PDI as a functional ligand yielded a highly efficient phosphorescent emitter **83.4**. Progress in azaacene chemistry (Sections 2.5.1 and 4.6) provided access to solution-processable nitrogen-doped nanoribbons. Some of these molecules are remarkably long (cf. Scheme 158) and were investigated, e.g., as semiconductors for TFT devices (**24.5a**–**e**).

Exploration of boron-doped PHAs has produced many notable methodological advances (Sections 2.3, 3.1, 3.4, 4.1.1, 4.2, 4.3, 4.5, 4.6, 5.1, and5.3.1, 7.3.1). Compound **211.3**, an ultrapure-blue emitter based on thermally activated delayed fluorescence, simultaneously highlights the use of B*-*doping and the utility of “triangular” fusion in designing new materials. **C20.5a**, combining four oxygen-bridged triphenylamine subunits, contains a similar fusion pattern and was employed as a hole-transporting material for perovskite solar cells. The related heteratriangulenes (Section 4.1), available in a variety of doping patterns, are often emissive and can be used, e.g., for bioimaging applications (**C13.1a**–**c**).

PHA motifs have been incorporated into several types of covalent organic frameworks (COFs, e.g. Scheme 260, Section 6.1.6; Scheme 375, Section 7.5.5; and Scheme 393, Section 7.7.2). COFs **375.2a**–**c**, notable for their high intrinsic conductivity, additionally illustrate the progress in macrocyclic PHAs, which is surveyed in Section 7. This area is notable for the development of several unprecedented fusion motifs and creation of some remarkable 3D-fused structures. A variety of new heteroatom-doped circulenes and coronoids have been developed (Section 6.1), including systems with significant positive or negative curvature.

### 1.3 Ring-Forming Reactions

**Scheme 1:**
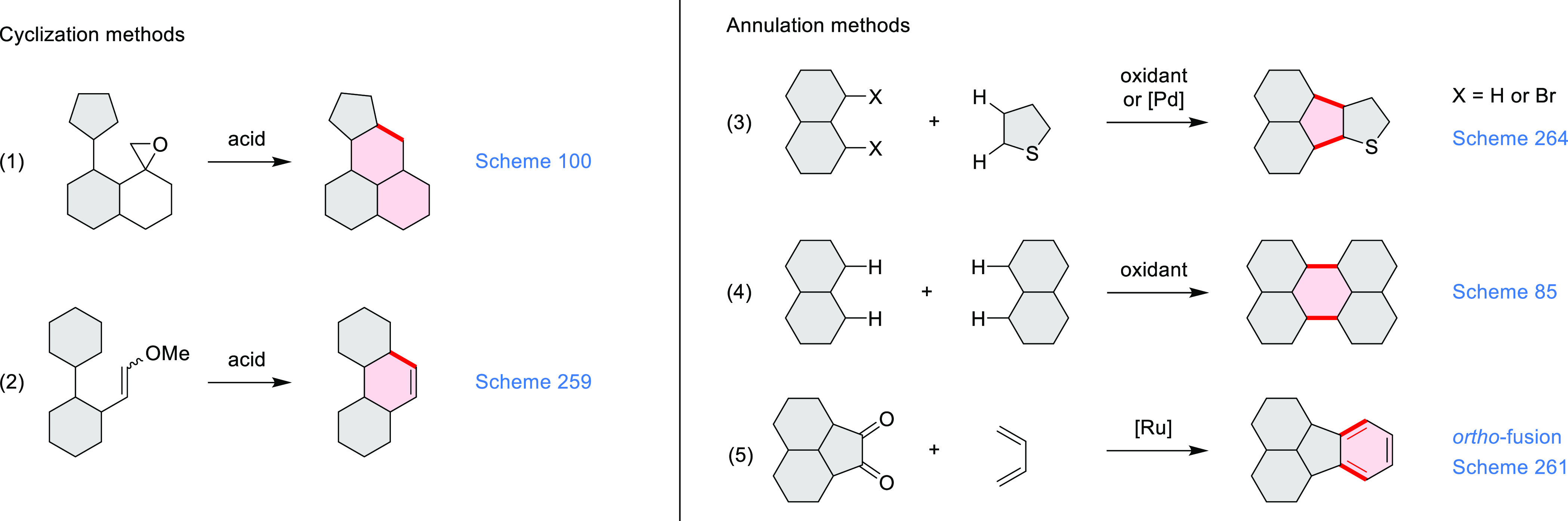
Carbocycle Formation via Cyclization and Annulation Reactions π-Conjugation is implicit in shaded rings. Numbers correspond to representative schemes and charts.

**Scheme 2:**
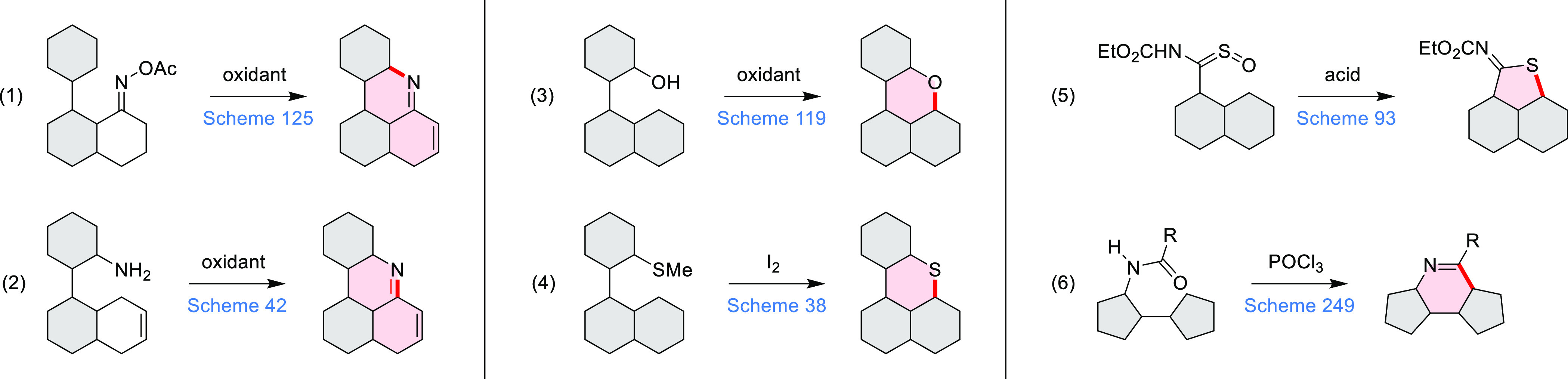
Heterocycle Formation via Cyclization Reactions π-Conjugation is implicit in shaded rings. Numbers correspond to representative schemes and charts.

**Scheme 3:**
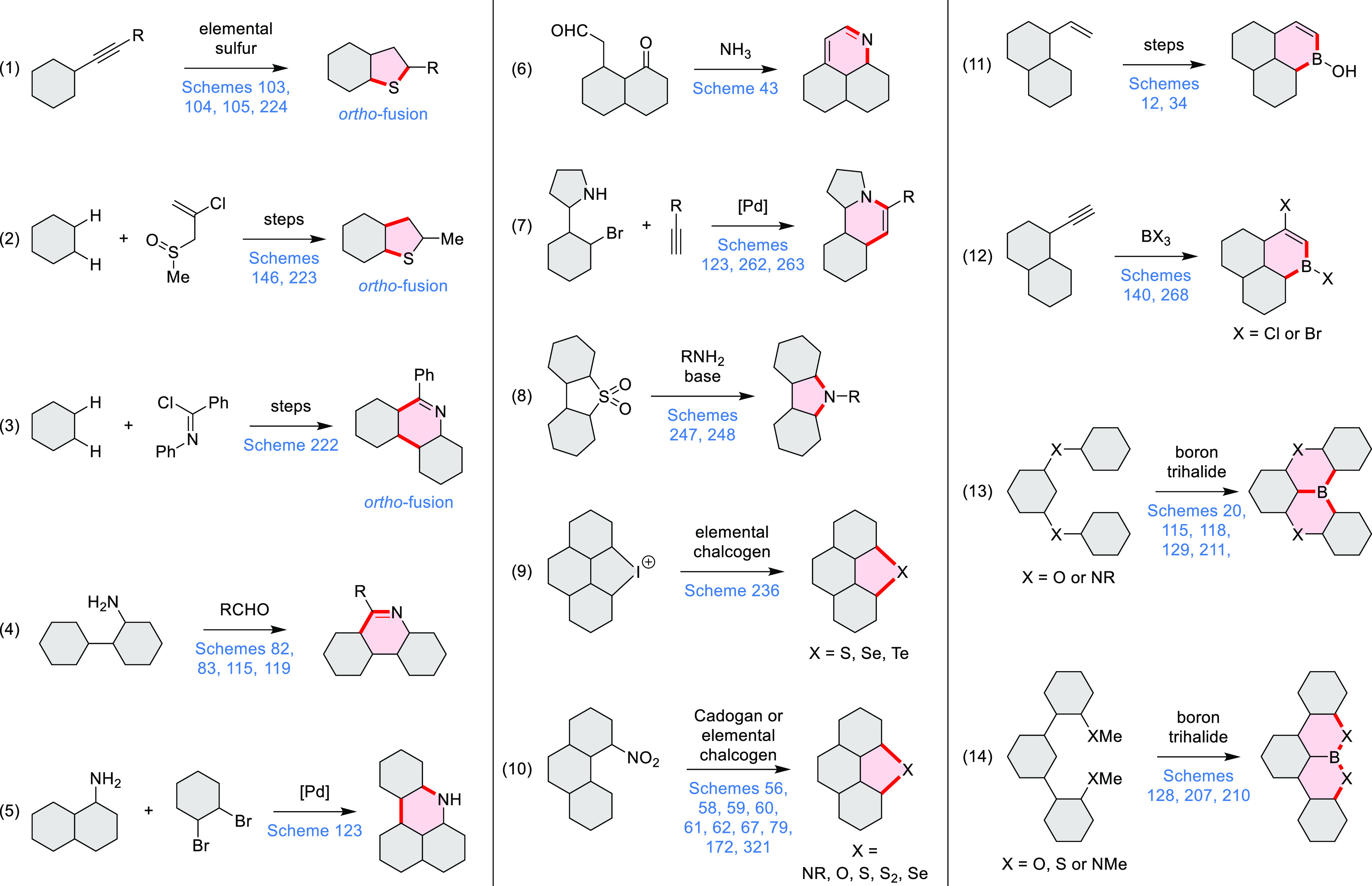
Heterocycle Formation via Annulation Reactions π-Conjugation is implicit in shaded rings. Numbers correspond to representative schemes and charts.

The synthetic chemistry of PAHs relies primarily on efficient ring-forming reactions. These transformations are necessary not only to make heterocyclic rings but also to build fused carbocyclic units and create macrocycles. The core methodology of PHA synthesis was summarized in the original review (CR2017, Section 1.4). Below we provide a brief summary of notable recent developments (Schemes 1, 2, and 3).

Closures of six- and five-membered carbocyclic rings involve a variety of approaches, both conventional and transition-metal-catalyzed ones. The utility of electrophilic cyclizations has been extended by introduction of new reactive electrophile units, such as the epoxide ring and the 2-methoxyvinyl group (Scheme 1, entries 1 and 2, cf. Schemes 100 and 259). Annulations involving unfunctionalized components, such as the recently reported direct pentannulations of thiophene (cf. Scheme 264) and the oxidative self-dimerization of an electron-rich naphthalene (cf. Scheme 85), can achieve a rapid increase in molecular complexity from readily available starting materials. Ruthenium-catalyzed benzannulations involving 1,3-butadiene, such as the one shown in Scheme 261, provide similar advantages in the construction of *ortho*-fused benzene rings.

Several oxidative cyclization methods for cove-region heterocycle formation have been recently described (Scheme 2). They include oxidative transformations of oximes and amines (entries 1 and 2, cf. Schemes 125 and 42), phenols (entry 3, cf. Scheme 119), and thioethers (entry 4, cf. Scheme 38), yielding, respectively, N*-*, O-, and S*-*doped substructures. Electrophilic heterocyclizations have been used to close both five- and six-membered rings. In one example, the acid-catalyzed dehydrative cyclization of thioamide *S-*oxide generates a *peri*-fused sulfur-containing heterocycle (entry 5, cf. Scheme 93). *peri*-Fused pyridine rings have been closed using an intramolecular variant of the Vilsmeier–Haack reaction (entry 6, cf. Scheme 249).

Examples of heterocycle-forming annulation reactions are collected in Scheme 3. *ortho*-Thienannulation protocols have been developed for alkynyl-substituted arenes (entries 1 and 2, cf. Schemes 103, 104, 105, and 224) and unfunctionalized arenes (Schemes 146 and 223). Several methods for construction of *ortho*- or *peri*-fused pyridines have been reported, some involving functionalized arenes (e.g., entry 3, cf. Scheme 222). In alternative approaches, aminoarenes were condensed with aldehydes (entry 4, cf. Schemes 82, 83, 119, and 242) or with 1,2-dibromobenzene under palladium catalysis (entry 5, cf. Scheme 123). *peri*-Fused pyridine rings were also formed via the condensation of a 1,5-dicarbonyl-containing arene with ammonia (entry 6, cf. Scheme 43) or via Sonogashira coupling followed by intramolecular hydroamination of alkyne (entry 7, Schemes 262, 123, and 263).

Conversions of reactive heterocycles containing heavier heteroelements have found use as a strategy toward nitrogen- and chalcogen-containing rings. In one approach, the dibenzothiophene *S*,*S-*dioxide moiety was transformed to the corresponding carbazole moiety via the S_N_Ar mechanism (entry 8, cf. Schemes 247 and 248), whereas the five-membered cyclic iodonium moiety was converted to the fused thiophene, selenophene, and tellurophene rings (entry 9, cf. Scheme 236). Nitroarenes play an important role in the classical Cadogan syntheses of indoles and carbazoles and have also been applied for the formation of oxygen-, sulfur-, and selenium-containing rings in perylenoids (entry 10, cf. Schemes 56, 58, 59, 60, 61, 62, 67, 79, 172, and 321).

Borylative annulations have emerged as an important method to generate *peri*-fused boron-containing six-membered rings from olefin-substituted (entry 11, cf. Schemes 138 and 34) and alkyne-substituted precursors (entry 12, Schemes 140 and 268). This strategy was also extended to produce double annulations, leading to concomitant formation of three C–B bonds (entry 13, cf. Schemes 20, 211, 115, 129, and 118) or to borylation of both aromatic carbon atoms and heteroatom substituents (entry 14, cf. Schemes 207, 210, and 128).

## 2 Coronenoids

Cyclodehydrogenation of hexaarylbenzenes (HABs) and their heterocyclic analogues remains the most popular route toward hexa-*peri*-fused coronenes. Complete fusion is occasionally difficult to achieve, and products of partial ring fusion are often reported. For completeness, these species are discussed in Section 2, even though they do not contain a coronene substructure. Likewise, edge-expanded coronenoids containing seven-membered rings are also included below.

### 2.1 Edge-Doped Aza- and Oxacoronenes

#### 2.1.1 Mono- and Diazacoronenes

**Scheme 4:**
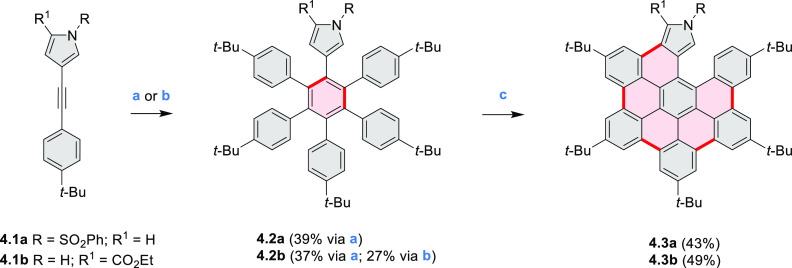
Synthesis of a HBC-Like System Containing a Pyrrole Ring Reagents and conditions: (a)^2^ 2,3,4,5-tetrakis(4-*tert*-butylphenyl)cyclopenta-2,4-dien-1-one, toluene, 200 °C, 18 h for **4.1a** 72 h for **4.1b**; (b) 2,3,4,5-tetrakis(4-(*t*-butyl)phenyl)cyclopenta-2,4-dien-1-one, THF, dioxane, Co_2_(CO)_8_, 160 °C, 15 min, microwave, 350 W; (c) FeCl_3_, DCM, MeNO_2_, 0 °C.

In 2017, Jux et al. reported a synthetic route toward pyrrole-containing HBC analogues (Scheme 4).^2^ The Diels–Alder reactions of each alkyne **4.1a**,**b** at 195–200 °C in a sealed tube in toluene under an argon atmosphere led to the formation of **4.2a**,**b**. Conversion of these compounds into HBC systems was then attempted under dehydrogenative cyclization conditions (DCM/MeNO_2_/FeCl_3_), leading to quintuply fused structures **4.3a**,**b**. The fully fused HBC analogues could not be obtained under those conditions. **4.3a** showed a helicene-like shape of the molecules in the solid state. The UV–vis spectra of **4.3a**,**b** contain a broad, intense absorption around 360 nm, and due to the lower molecular symmetry, the π → π* transition could be observed as an absorption band around 420 nm. The fluorescence of both [5]helicenes **4.3a**,**b** is similar to HBC derivatives, with two maxima at around 470 and 500 nm. Cyclovoltammetric measurements revealed two reversible oxidation waves at +0.85 V/+0.80 V and +1.29 V/+1.22 V (vs SHE) for **4.3a**/**4.3b**, respectively.

**Scheme 5:**
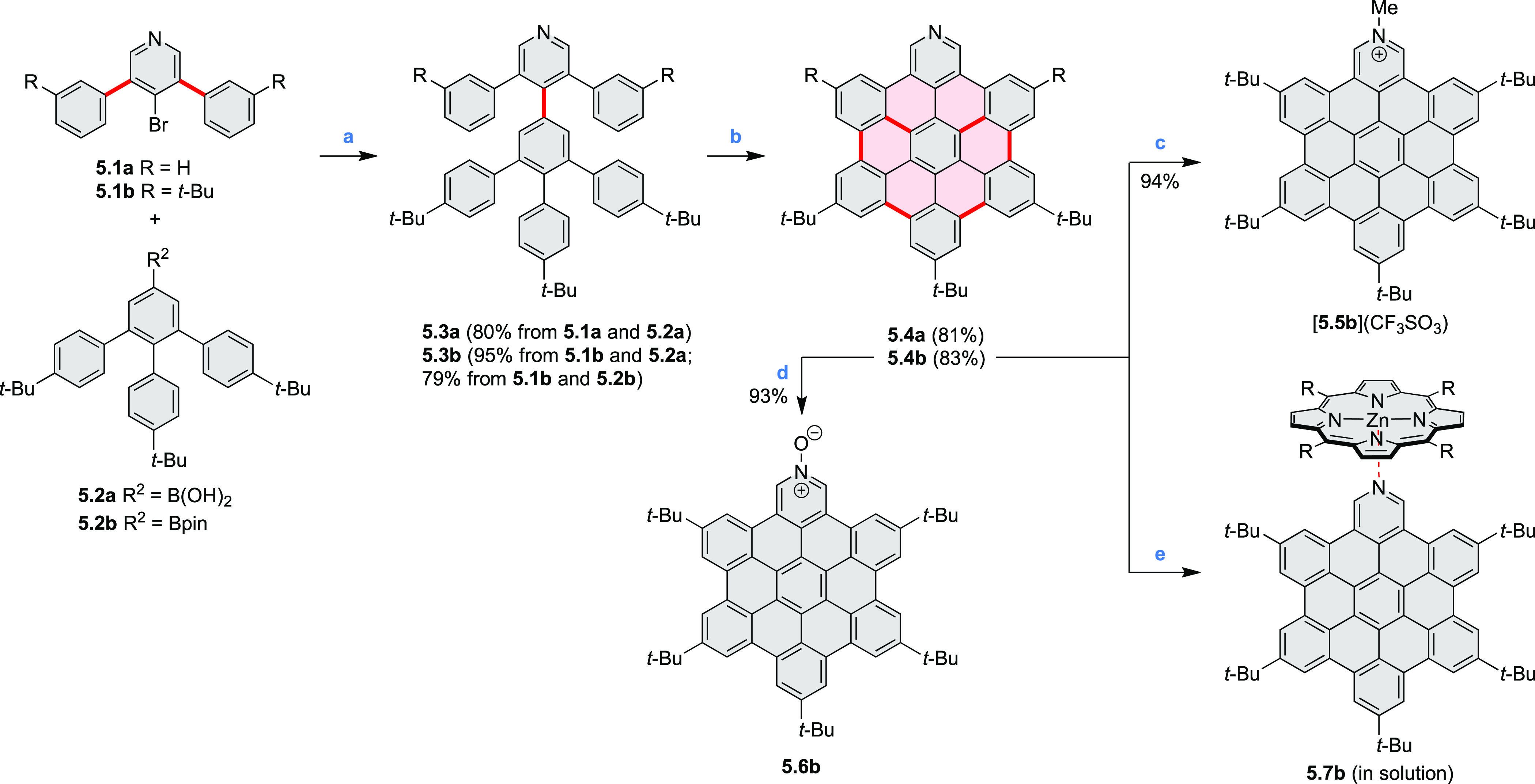
Synthesis of π-Extended Pyridines Reagents and conditions: (a)^3^ Cs_2_CO_3_ (2 equiv), 10 mol % of Pd(PPh_3_)_4_, THF/H_2_O (4:1), 17–24 h, 80 °C, N_2_; (b) **5.4a**: DDQ (7 equiv), TfOH (14 equiv), DCM, 1 h, 0 °C, N_2_; **5.4b**: DDQ (7 equiv), TfOH (14 equiv), DCM, 4 h, −50 °C to −20 °C, N_2_; (c) (1) MeI (excess), CH_3_CN, 2 h, rt, N_2_, (2) Ag(OTf) (2.1 equiv), 15 min, rt, N_2_; (d) *m*-CPBA (1 equiv), CHCl_3_, 24 h, 0 °C to rt; (e) tetrakis(4-*tert*-butylphenyl)porphyrinato zinc (1 equiv), C_6_D_6_, rt.

The Jux group further reported a solution for synthesis of pyridine analogues of hexa-*peri*-hexabenzocoronene (Scheme 5).^3^ A key feature of their strategy was the early formation of the C–C bonds at the 3 and 5 positions of the pyridine (**5.1a**,**b**, red bonds). These bonds are otherwise unreactive and difficult to close under oxidative conditions. Compounds **5.1a**,**b** and **5.2a**,**b** were obtained via multistep procedures starting from 4-aminopyridine and *para*-nitroaniline, respectively. Formation of the pseudo-HAB precursors **5.3a**,**b** was achieved via a Suzuki reaction. The final step was carried out under oxidative cyclodehydrogenation conditions with DDQ and triflic acid in DCM, with yields up to 83%. The more soluble product **5.4b** was further derivatized via metal coordination, *N*-alkylation, and *N*-oxidation. Methylation at the nitrogen atom yielded a pyridinium ion (**5.5b**), which was isolated as its triflate salt, whereas *m*-CPBA oxidation produced the corresponding *N-*oxide **5.6b**. **5.4b** was shown to act as an apical ligand toward tetrakis(4-*tert*-butylphenyl)porphyrinato zinc in solution. Formation of the corresponding complex **5.7b** was demonstrated by a very large upfield shift of the 2,6-pyridine proton signals from 10.54 to 4.60 ppm, caused by the shielding effect of the aromatic ring current of the porphyrin.

**Scheme 6:**
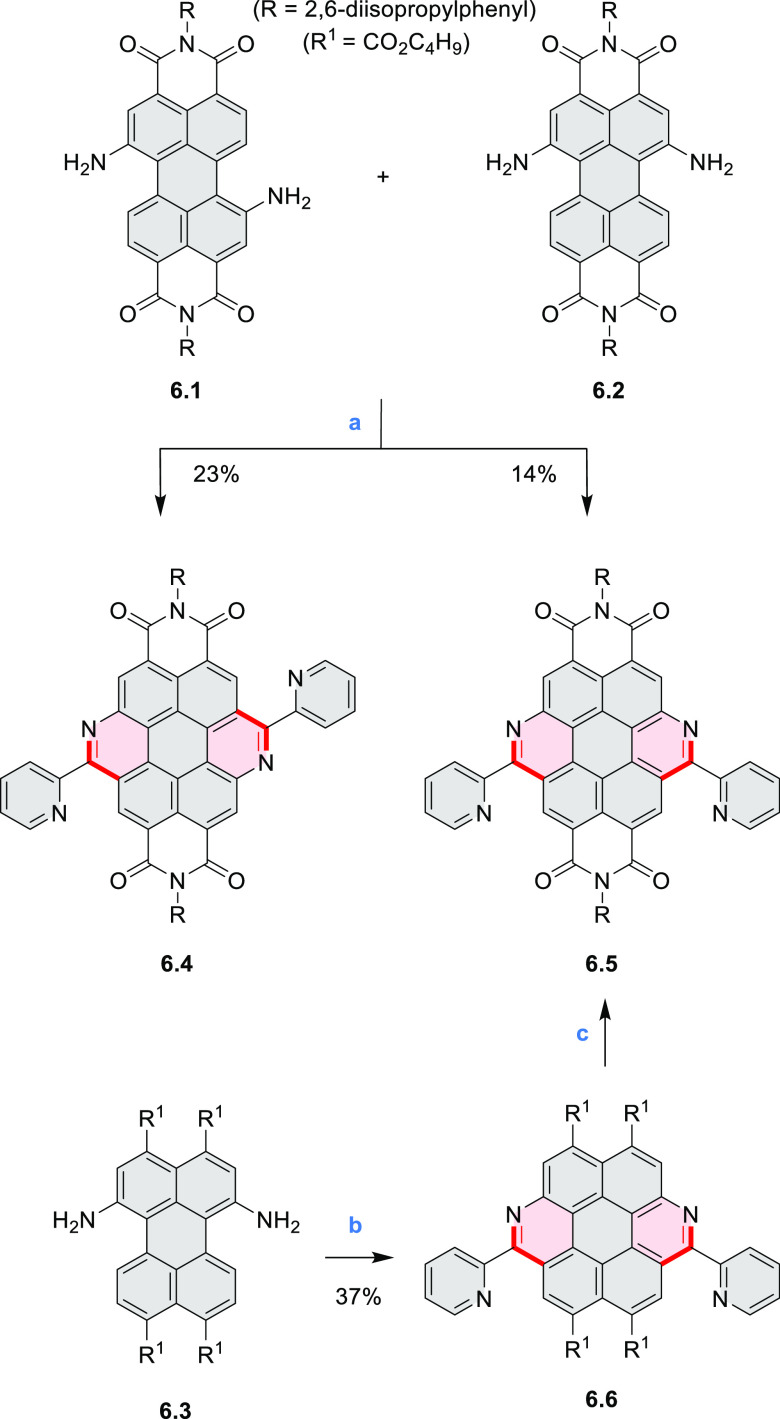
Synthetic Routes to Diazacoronene Diimides and Tetraesters Reagents and conditions: (a)^4^ picolinaldehyde, dry DMF, TFA, molecular sieves 3 Å, 110 °C, N_2_ to O_2_, 21 h; (b) picolinaldehyde, dry DMF, TFA, molecular sieves 3 Å, 110 °C, N_2_ to O_2_, 18 h; (c) (1) H_2_SO_4_, AcOH, reflux, 17 h, (2) RNH_2_, imidazole, pyridine, 120 °C, 6 h.

Diimide and tetraester diazacoronenes were synthesized via the Pictet–Spengler reaction of 1,6- and 1,7-diaminoperylenes with picolinaldehyde in 2016 by Würthner et al. (Scheme 6).^4^ Starting from a 3:2 regioisomer mixture of 1,7- and 1,6-diamino PDIs **6.1**–**2**, azabenzannulated products **6.4**–**5** were obtained and separated by column chromatography (for singly azabenzannulated analogues, see Scheme 82, Section 3.1.1). Yields of these syntheses were limited by purification problems. Similarly, the bisazabenzannulated perylene tetraester **6.6** was synthesized from the isomerically pure 1,6-diaminoperylene tetraester **6.3** in 37% yield. After hydrolysis of **6.6**, the corresponding dianhydride was converted into variously functionalized bisazabenzannulated perylene diimides, such as **6.5** (Scheme 6). The spectroscopic data reveal a hypsochromic shift of the S_0_–S_1_ transition band, which is less pronounced in comparison to the parent PDI. The oscillator strengths and the intensities of the S_0_–S_2_ transitions strongly increase with core extension because the S_0_–S_2_ transition dipole moments are aligned along the laterally elongated molecular axes. Therefore, the spectra of **6.4**–**6** are not predominated by the lowest-energy S_0_–S_1_ transitions but by higher-energy absorption bands.

**Scheme 7:**
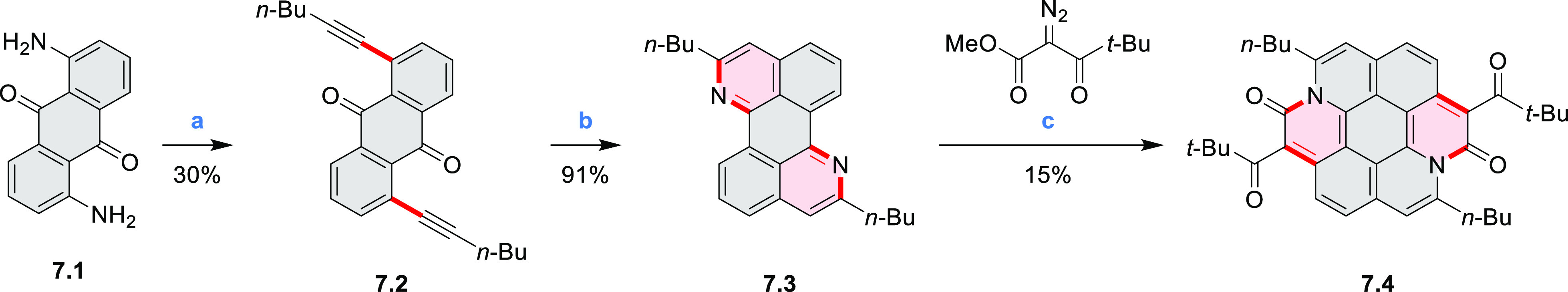
Synthetic Route to Bis(amide)-Containing Coronene Reagents and conditions: (a)^6^ (1) NaNO_2_, H_2_SO_4_, (2) KI, water, (3) hex-1-yn-1-yl copper, pyridine; (b) urea, DMF; (c) [Rh^III^Cp·(MeCN)_3_](SbF_6_)_2_, TFE, 120 °C, 14 h, (2) DMAP, TFE, 140 °C, 14 h.

A soluble amide-containing coronene **7.4** was recently reported by Yamaguchi and Glorius et al.^5^ The fused framework of **7.4**, containing two fused lactam rings, was obtained by 2-fold C–H activation of diazaperylene precursor **7.3** (Scheme 7), which was synthesized in two steps from the commercially available **7.1**. **7.4** exhibited a far more red-shifted absorption band (λ_abs_ = 673 nm in DCM), in comparison to the diazacoronene **6.4** (λ_abs_ = 485 nm in DCM). **7.4** showed a deep green color and an intense NIR emission with the λ_em_ of 686 nm and a high fluorescence quantum yield of 0.64 in DCM.

**Scheme 8:**
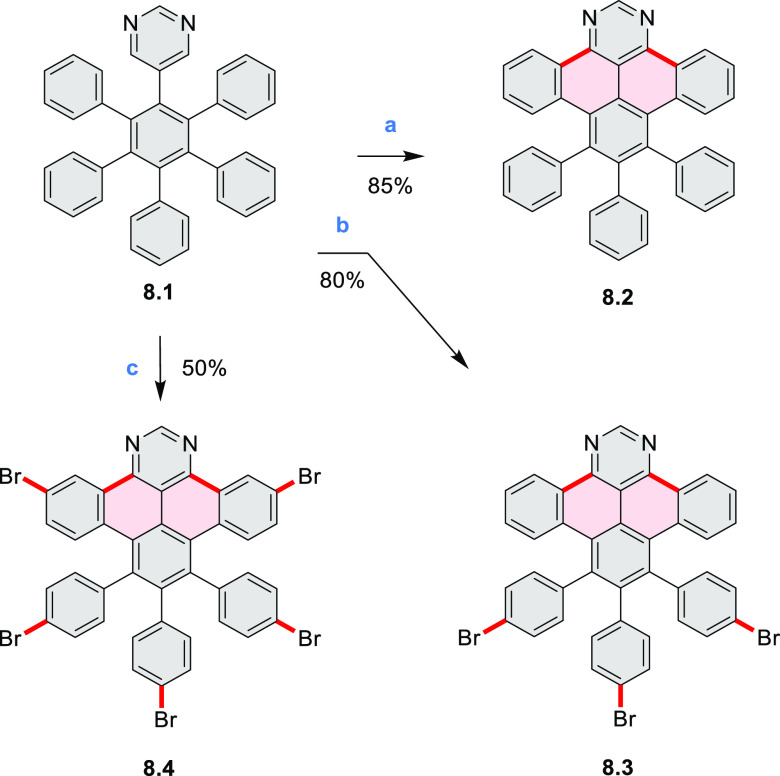
Pyrimidine-Containing Nanographenes Reagents and conditions: (a)^6^ Br_2_, toluene, 90 °C, 2 h; (b) Br_2_, rt, 25 min; (c) Br_2_, CHCl_3_, reflux, 3 h.

The possibility to induce intramolecular bond formation at the pyrimidine ring of **8.1** by using dibromine was explored by Draper and co-workers (Scheme 8).^6^ The pyrimidine-containing precursor **8.1** was reacted in neat dibromine at rt to yield different proportions of **8.2**, **8.3**, and **8.4**, depending on the reaction time (5 min to 5 h). Performing the reaction in toluene at 90 °C resulted in the formation of only **8.2** in 85% yield, while refluxing in chloroform was successful in driving the reaction to higher yields of the pentabrominated **8.4**. The use of bromine electrophiles was subsequently found to be an efficient method for oxidative coupling of the more electron-rich hexapyrrolylbenzenes (see below).

#### 2.1.2 Dioxacoronenes

**Scheme 9:**
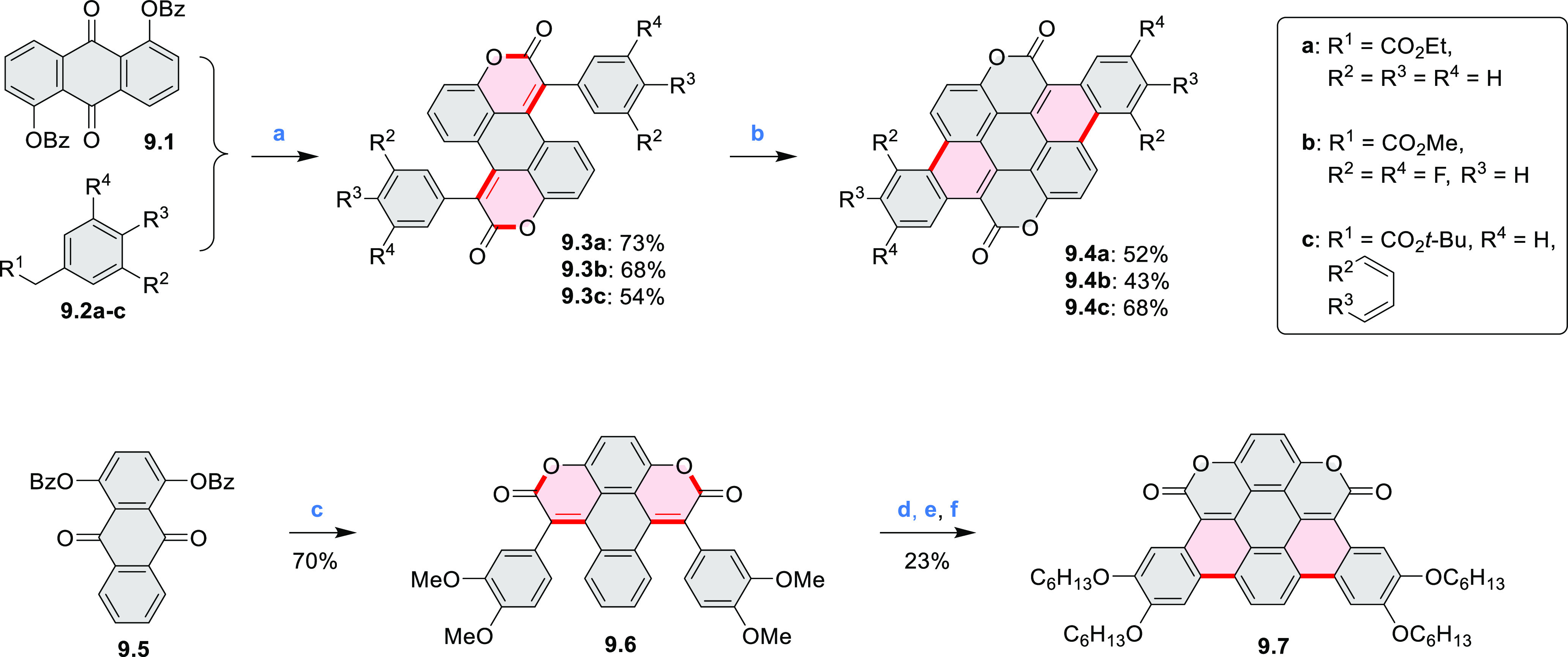
Synthesis of π-Extended Biscoumarins Reagents and conditions: (a)^7^ K_2_CO_3_, DMSO, 100 °C; (b) *h*ν, THF, rt; (c)^8^ K_2_CO_3_, DMSO, 100 °C; (d) BBr_3_, DCM, −40 °C to rt; (e) C_6_H_13_Br, K_2_CO_3_, DMF, 60 °C; (f) FeCl_3_, DCM, rt.

New π-extended biscoumarins were reported in 2017 by Gryko and Głowacki et al. (Scheme 9).^7^ The synthesis, based on a previously developed strategy, involved initial Knoevenagel condensation of **9.1** and **9.2a**–**c**. The resulting **9.3a**–**c** were subjected to Mallory photocyclization, to produce the fused targets **9.4a**–**c**. Compound **9.7** was obtained from **9.5** by using a similar synthetic strategy (Scheme 9).^8^ The methoxy groups of the intermediate **9.6** were replaced with −OC_6_H_13_, and the final ring fusion was achieved by oxidation with FeCl_3_. Photophysical properties of **9.7** were almost identical to its regioisomers **9.4a**–**c**. These compounds exhibit vibronically resolved absorptions and high fluorescence quantum yields of up to 90%.

#### 2.1.3 Triazacoronenes

**Scheme 10:**
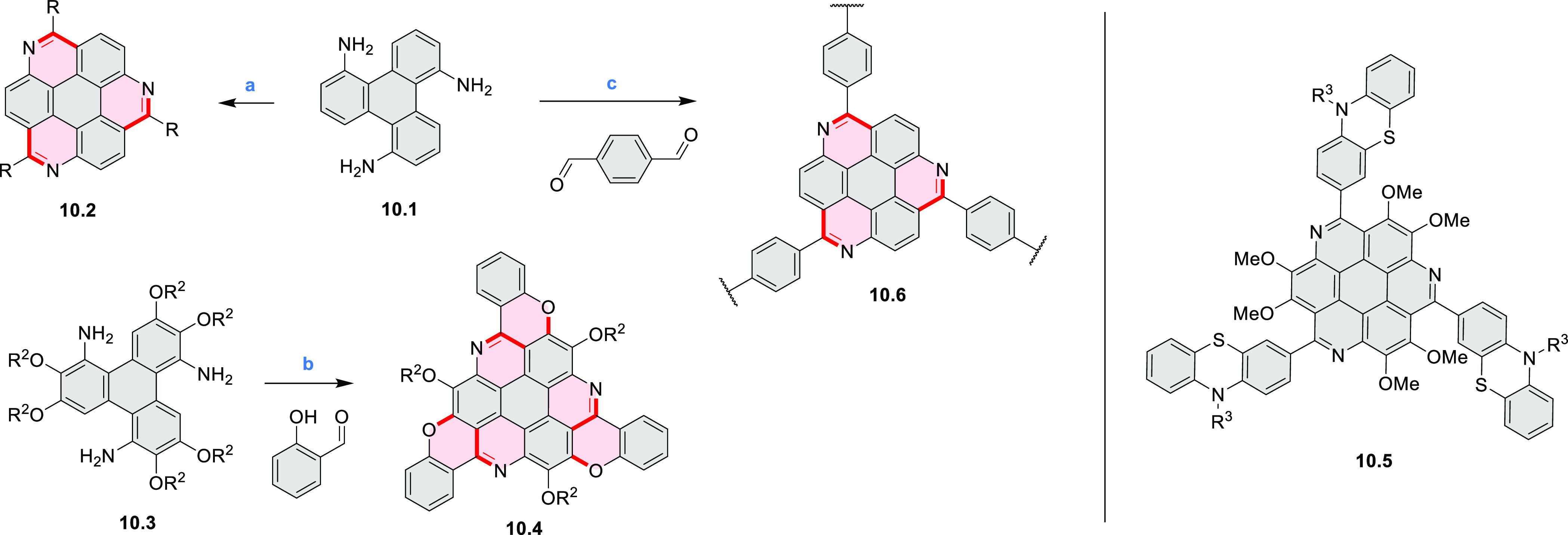
1,5,9-Triazacoronene Derivatives Reagents and conditions: (a)^9−12^ various aldehydes, DMSO, 130–150 °C, in Ar, then in air; (b)^10^ (1) DMF or NMP, TfOH, 120 °C, 12 h, (2) 6 equiv of K_2_CO_3_, 120 °C, 12 h; (c)^12^ DMF/dioxane (10:1 v/v), AcOH, TfOH.

1,5,9-Triazacoronene (TAC) derivatives were explored by Wei et al. as potential materials for electrogenerated chemiluminescence (ECL).^9−11^ Tris(phenothiazine)-substituted **10.5** was prepared through a triflic-acid-catalyzed 3-fold Pictet–Spengler cyclization and subsequent oxidative aromatization.^9^ Absorption and fluorescence emission spectra of **10.5** revealed that its electronic properties were affected by intramolecular charge transfer from phenothiazine donors to TAC acceptors in the excited state while showing no charge-transfer interaction in the ground state. π-Extended triazacoronene derivatives **10.4** containing three *peri*-fused benzopyran units were synthesized in one pot with yields of up to 87%.^10^ The synthetic approach involved a tandem triflic-acid-catalyzed 3-fold Pictet–Spengler cyclization and a K_2_CO_3_-catalyzed *ipso*-aromatic substitution. In the solid state, **10.4** forms sandwich-type trimeric assemblies stabilized by stacking interactions between the sterically unhindered π surfaces. In 2020, it was reported that the Pictet–Spengler cyclization for synthesizing TACs from the triphenylene triamine and aldehydes proceeds not only under acidic but also under neutral or even alkaline conditions.^11^ Under optimized conditions (Scheme 10), a wide variety of TAC derivatives (**10.2**) could be synthesized, with yields reaching 94%.^11^ Acid-catalyzed conditions were used by Coskun et al. to synthesize conjugated microporous polymers (CMPs) **10.6** from 1,5,9-triaminetriphenylene **10.1** and terephthalaldehyde.^12^ The optical band gap and surface area of the resulting CMPs were found to correlate with the strength of the acid catalyst.

#### 2.1.4 Tetra- and Hexaazacoronenes

**Scheme 11:**
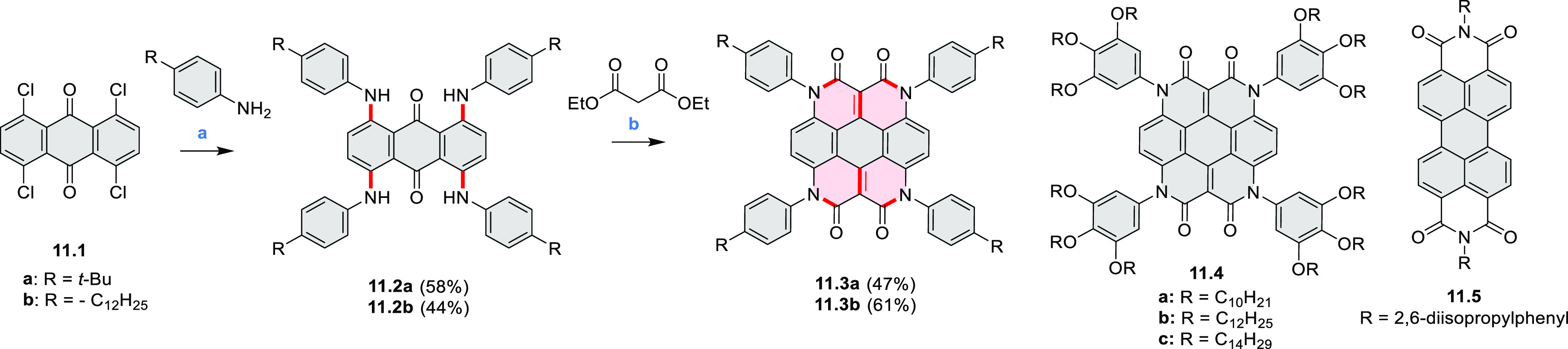
From Anthraquinone to a Heteracoronene Reagents and conditions: (a)^13^ Pd_2_(dba)_3_, BINAP, Cs_2_CO_3_, toluene, 105 °C, 24 h; (b) CH_3_CO_2_K, DMF, microwave, 170 °C, 30 min.

In 2017, Li et al. reported tetralactam coronenoids **11.3a**,**b**, which were obtained from the commercially available anthraquinone **11.1** via 4-fold Buchwald–Hartwig amination with a 4-alkylaniline followed by microwave-assisted Knoevenagel condensation of the intermediate **11.2** with diethyl malonate (Scheme 11).^13^ These disclike tetraazacoronenes exhibited optical properties similar to PDIs but had higher LUMO (−3.6 eV) and HOMO (−5.8 eV) levels than those of perylene orange **11.5** (LUMO = −3.8 eV, HOMO = −6.1 eV). Although **11.3** had nearly no fluorescence in solution, it revealed strong photoluminescence in the solid state. **11.3** exhibits high thermal stability (up to 515 °C) and photostability comparable with PDI dyes. Discotic liquid crystals **11.4a**–**c** were designed by introducing wedge-shaped side groups with alkyl tails of different lengths at the periphery of the tetraazacoronene core.^14^ A high hole mobility μ_h_ of 8.84 cm^2^ V^–1^ s^–1^ was determined for **11.4a**, whereas **11.4c** showed an electron mobility μ_e_ of 3.59 cm^2^ V^–1^ s^–1^.

**Scheme 12:**
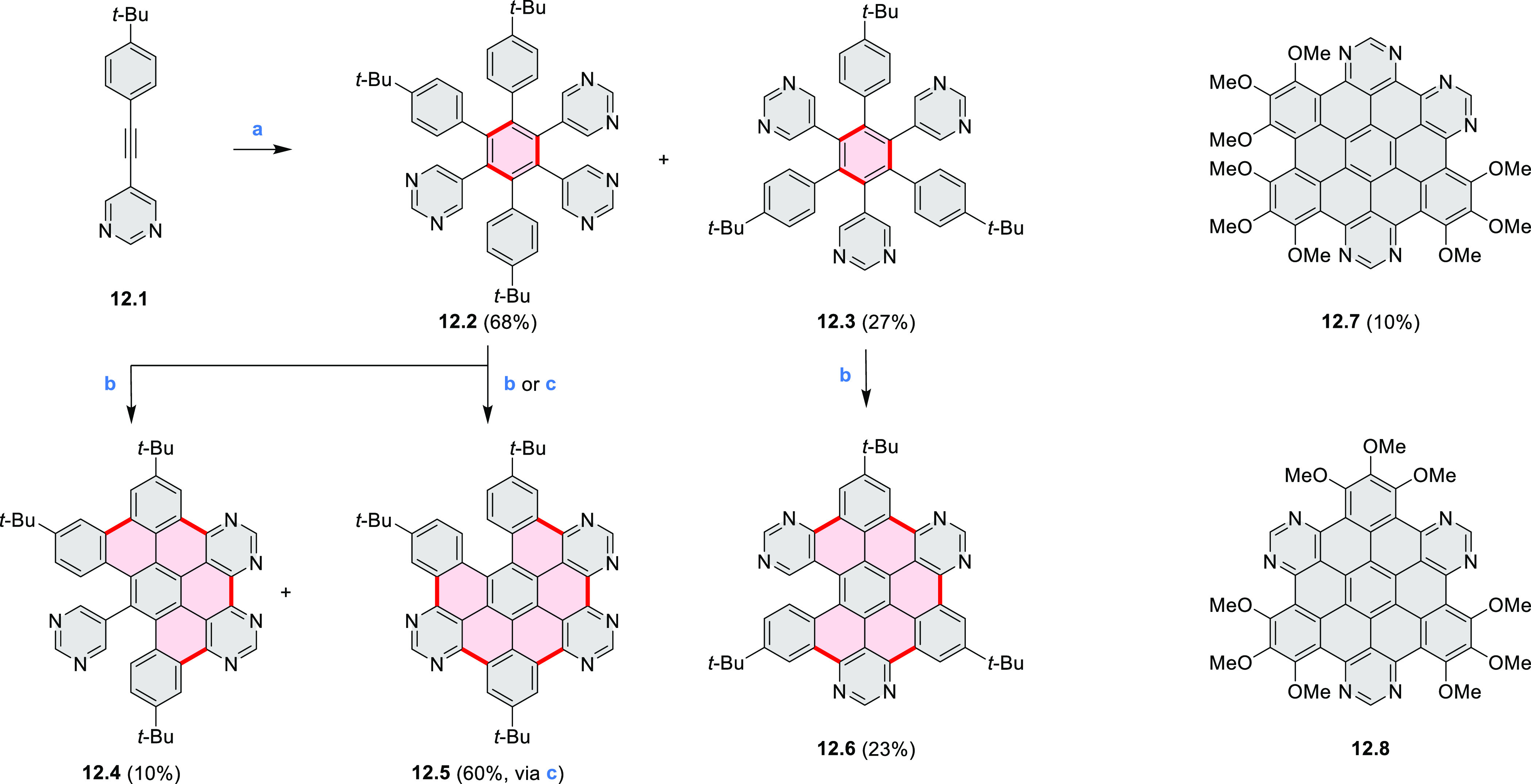
Synthesis of Pyrimidine-Containing Hexaaza-HBCs Reagents and conditions: (a)^15^ Co_2_(CO)_8_, dioxane, 115 °C, 24 h, under N_2_; (b) FeCl_3_, CH_3_NO_2_, 298 K, Ar bubbling, 72 h; (c) DDQ, MeSO_3_H or CF_3_SO_3_H, DCM.

The Draper group reported two isomeric types of hexaaza-HBCs, differing in their N*-*doping pattern (Scheme 12).^15^ The initial hexaarylbenzenes were obtained as regioisomer mixtures in a dicobalt octacarbonyl-catalyzed cyclotrimerization reaction. For the *tert*-butyl-substituted precursor **12.2**, cyclodehydrogenation using FeCl_3_ as the oxidant yielded **12.4** and **12.5** in yields of 10% and 20%, respectively, whereas the DDQ/H^+^-mediated reaction produced **12.5** as the major product. Incomplete ring fusion was similarly observed in the oxidation of **12.3**, which yielded **12.6**. Fully cyclized products **12.7** and **12.8** were however successfully obtained upon treating the more electron-rich methoxy-substituted HABs with FeCl_3_.

### 2.2 Internally Doped Azacoronenes

**Scheme 13:**
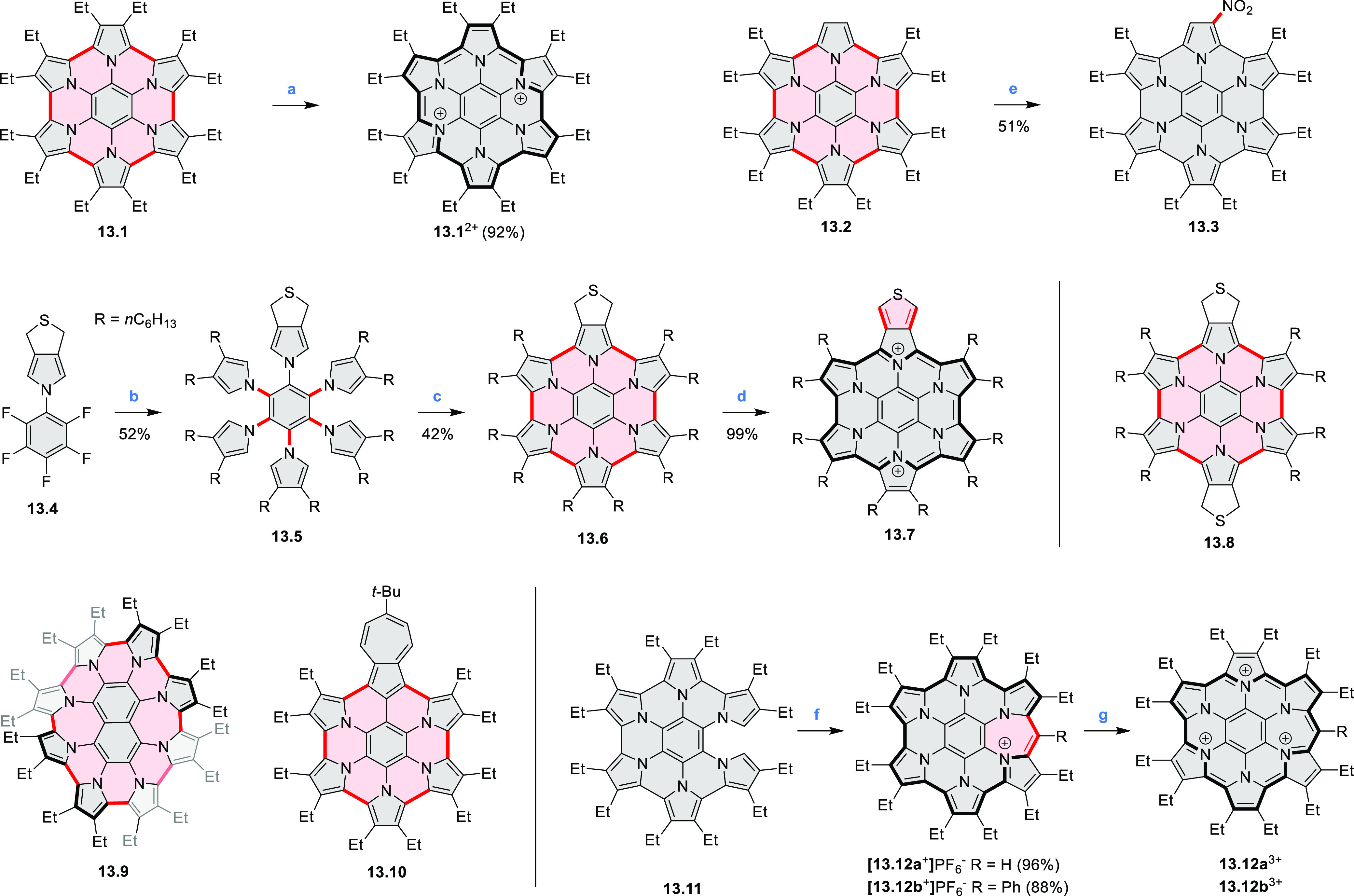
Synthesis and Structures of Peripherally Fused Azacoronenes and Their Analogues Reagents and conditions: (a)^16^ NOSbF_6_ (2.0 equiv) and DCM, rt, 10 min; (b)^18^ 3,4-dihexylpyrrole, NaH, DMF, rt; (c) FeCl_3_, CH_3_NO_2_, rt; (d) I_2_ (excess), reflux, under N_2_ flow; (e)^17^ AgNO_2_ (9.7 equiv), DCM, rt; (f)^21^ DMF or *N*,*N-*dimethylbenzamide, POCl_3_, KPF_6_, DCE; (g) NOSbF_6_ or BAHA, DCM.

Hexapyrrolohexaazacoronenes (HPHACs) are typically synthesized in two steps, namely, via the S_N_Ar reaction of hexafluorobenzene with the corresponding pyrrole followed by oxidative cyclodehydrogenation of the resulting hexapyrrolylbenzene (Scheme 13; cf. CR2017, section 2.2). Variants of this method provide access to a range of structurally diverse molecules. The use of ethyl substitution introduced by Uno and Takase et al. provides access to more electron-rich and potentially more reactive HPHAC derivatives, such as **13.1**,^16^ which is easily oxidizable to the typical globally aromatic dication **13.1**^2+^ containing a 22 π-electron conjugation pathway. According to an MCD analysis and DFT calculations, the NIR absorption band observed for the dication but absent in the spectrum of the parent cyclo[6]pyrrole macrocycle is attributed to a CT transition from the central benzene to the peripheral pyrrole moieties. Oxidation of β-unsubstituted HPHACs **13.2** with silver(I) nitrite did not result in reversible formation of the corresponding dication nor in oxidative dimerization that could be expected on the basis of the known reactivity of corrole or porphyrin chemistry. Instead, the nitrated derivative **13.3** was obtained, displaying redox properties similar to the parent system.^17^

In 2019, Uno and Takase et al. described the preparation of 1,3-dihydrothieno[3,4-*a*]- and 1,3,8,10-tetrahydrodithieno-[3,4-a;3′,4′-*m*]-HPHACs (**13.6** and **13.8**, respectively) by successive S_N_Ar reactions of hexafluorobenzene with (1) 1,3-dihydrothieno[3,4-*c*]pyrrole and (2) 3,4-dihexylpyrrole, followed by oxidative coupling.^18^ Upon oxidation with diiodine, **13.6** formed a dehydrogenated dicationic species **13.7**, which was not isolated in its neutral form. The dication was stable, and its NMR spectrum was indicative of global diatropicity, consistent with a peripheral aromatic pathway. The formation of a mixed-valence dimer consisting of the neutral **13.6** and its radical cation was observed in a CV measurement performed at high concentration and slow scan speed. **13.10**, a HPHAC analogue containing an azulene moiety replacing one of the pyrroles, was synthesized in three steps from a Bpin-substituted azulene derivative, using FeCl_3_ oxidation in the ultimate step.^19^ Similarly to the parent HPHAC, **13.10** displayed stable oxidized forms, and its dication was isolated and characterized. Structural and theoretical data demonstrated the existence of a 22π-electron conjugation encompassing the azacoronene core and a tropylium-like conjugation in the outer seven-membered ring.

**13.9**, a nonplanar core-expanded HPHAC analogue containing two N*-*doped seven-membered rings, was obtained from the commercially available octafluoronaphthalene and 3,4-diethylpyrrole via S_N_Ar and oxidative coupling reactions.^20^ X-ray diffraction analyses revealed the distorted structures of both the neutral **13.9** and dication [**13.9**]^2+^[PF_6_^–^]_2_ (prepared with AgPF_6_). In spite of the twisted structure, the nucleus-independent chemical shift (NICS) data of **13.9**^2+^ indicated a significant aromaticity increase in the dicationic state, while the anisotropy of the induced current density (ACID) plot demonstrated an amplified current density of the peripheral pathway. These results are consistent with global Hückel aromaticity in the dicationic state, corresponding to peripheral 30 π-electron conjugation.

Peripheral expansion of HPHACs by inserting additional bridges or rings into the rim of the π-conjugated framework leads to systems with significantly modified electronic properties (CR2017, section 2.2). This approach was followed in the synthesis of an antiaromatic expanded azacoronene cation [**13.12**]^+^ reported by the Uno group in 2019.^21^ Synthesis of the partially fused **13.11** was carried out under standard conditions while controlling the amount of oxidant employed. The Vilsmeier–Haack reaction of **13.11** using DMF and POCl_3_ gave an excellent yield of the intramolecularly cyclized [**13.12a**]^+^, rather than α-formyl derivatives of **13.11**. The antiaromatic monocation [**13.12**]^+^ was readily transformed into the aromatic trication [**13.12a**]^3+^.

**Chart 2:**
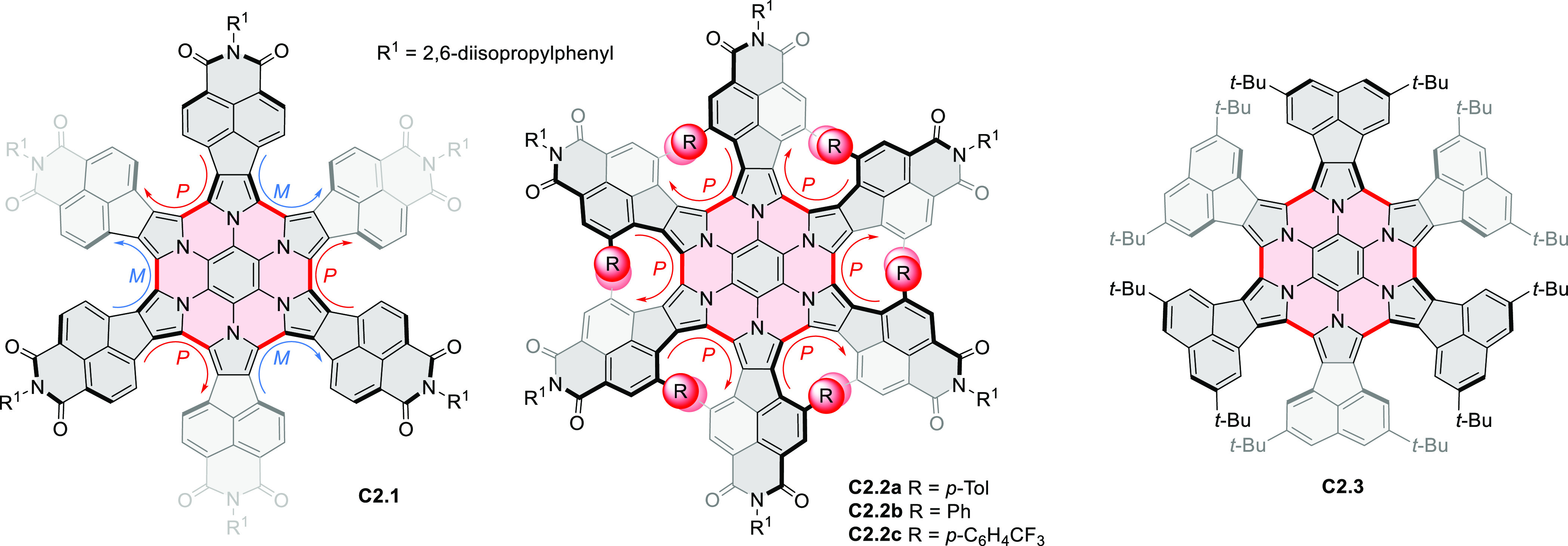
Radially π-Extended Pyrrole-Fused Azacoronenes

Radial π-extension of HPHACs is conveniently achieved by employing β–β-fused pyrrole building blocks. Our group reported the first such system, the large electron-deficient heterocycle **C2.1**, which was prepared from the naphthalenemonoimide (NMI)-fused pyrrole **308.1b**(22) (Section 7.1) following the standard two-step procedure (Chart 2).^23^ An XRD analysis of **C2.1** revealed a “monkey saddle” conformation, with alternating handedness of the peripheral helicene fragments. The electronic absorption spectrum of **C2.1** showed an intense band in the visible region, with a vibronic pattern characteristic of many rylene imide derivatives. **C2.1** exhibits considerable solvatochromism in solution, its color changing from purple in toluene, through bluish in DCM, to bluish gray in methanol. While the oxidation behavior of **C2.1** was similar to that of its HPHAC parent, the new ring system revealed an exceptional ability to consecutively accept ten electrons at easily accessible potentials, yielding anions with small electronic band gaps and panchromatic UV–vis–NIR absorption. Efficient aerial reoxidation of the reduced nanographenoid indicated its resistance to decomposition.

Molecular propellers **C2.2a**–**c**, chiral analogues of the snowflake-shaped **C2.1**, were obtained from sterically hindered hexapyrrolylbenzenes (HPBs), which were treated with bromine electrophiles, *N-*bromosuccinimide (NBS), and dibromine as oxidative coupling agents.^24,25^ Ferric chloride, generally an effective oxidant in the syntheses of HPHAC derivatives, applied to the sterically congested precursors of **C2.2a**–**c**, produced only mixtures of ill-defined, possibly polymeric products. Subsequent screening of various halogen electrophiles and reaction conditions showed the superior performance of NBS in lactic acid, producing **C2.2a**–**c** in 85–88% yields with excellent chemo- and stereoselectivities. Specifically, in contrast to the parent **C2.1** the helical sections in **C2.2a**–**c** are homochiral. These propeller HPHACs, the first examples of chiral nanographene analogues with deeply embedded nitrogen atoms, possess small band gaps, near panchromatic absorption, and multiredox behavior.

**C2.3**, an electron-rich analogue of **C2.1**, with *tert*-butyl groups replacing the peripheral imide moieties, was synthesized by Uno’s team.^26^ In that synthesis, CaH_2_ was found to effectively promote the complete S_N_Ar reaction of C_6_F_6_ with the bulky 2,5-di-*tert*-butyl-8*H*-acenaphtho[1,2-*c*]pyrrole. Like other azacoronene derivatives, **C2.3** was stable in its oxidized forms, and the NICS calculations demonstrated the existence of global aromaticity in the dication.

**Scheme 14:**
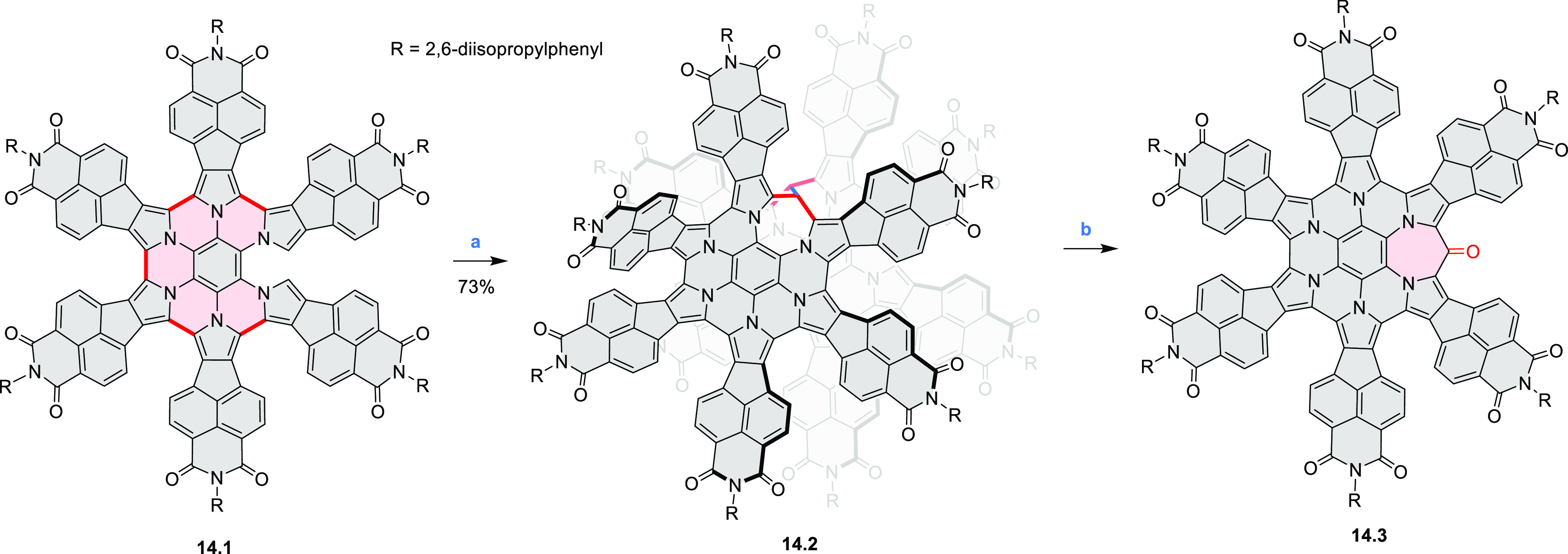
Synthesis of an Azacoronene Nanosandwich Reagents and conditions: (a)^27^ 6 equiv of 10-camphorsulfonic acid, 4 equiv of paraformaldehyde, CHCl_3_, pressure tube, 90 °C, 17 h; (b) toluene, 365 nm irradiation, air.

A dimeric naphthalimide–azacoronene hybrid linked via a pair of methylene bridges was described by our group in 2020 (Scheme 14).^27^ Compound **14.2** was formed in a reaction of the partially oxidized HPB **14.1** with paraformaldehyde in the presence of 10-camphorsulfonic acid as the catalyst. In an oxygen-free toluene solution, the **14.2** dimer undergoes photodissociation into a radical monomer. The radical exhibits extensive spin delocalization in its 139-electron π system and spontaneously dimerizes back to a stable σ-dimer and, in the presence of oxygen, is further oxidized to a stable ketone **14.3**. Photoinduced switching between the radical and its σ-dimer was found to rely on homolytic cleavage of a weak C(sp^3^)–C(sp^3^) bond, but its thermodynamics were controlled by a balance between π-conjugative stabilization, internal strain, and nonbonding interactions. The latter contribution had a decisive influence on the overall energetics of dimer formation and cleavage.

**Scheme 15:**
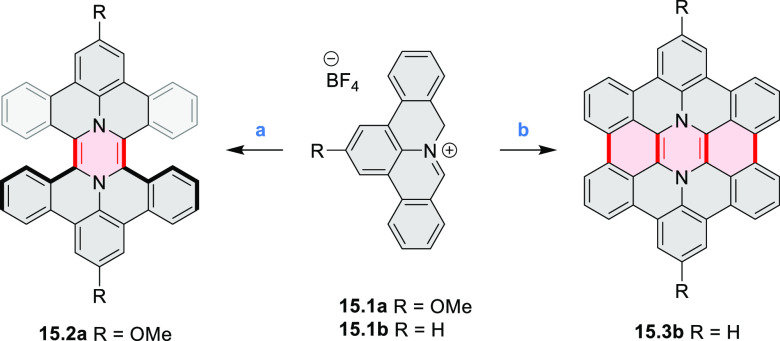
Synthesis of Diaza-HBP and Internally N*-*Doped Diaza-HBC Reagents and conditions: (a)^28^ (1) tributylamine, DMSO, 190 °C, Ar, (2) DDQ, C_2_D_2_Cl_4_, 100 °C; (b) in vacuo on the Ag(111) surface, 270 °C.

The majority of internally doped azacoronenes is based on the HPHAC design. A different approach to internal doping was proposed by Müllen and co-workers, who obtained pyrazine-containing nanographenes via dimerization of dibenzo-9*a*-azaphenalene (DBAP) (Scheme 15).^28^ A DBAP salt **15.1a** was dimerized by treatment with a large excess of tributylamine at 190 °C and oxidized with excess DDQ in dry C_2_D_2_Cl_4_, forming the hexabenzoperylene **15.2a**. Attempts to oxidize the latter product directly to diaza-HBC derivatives resulted in insoluble solids, which were not characterized. By changing the strategy to on-surface synthesis, small quantities of **15.3b** were detected after depositing **15.1** on Ag(111) by molecular beam evaporation and annealing to 270 °C. **15.3b** was characterized by STM and FM-AFM; combined scanning probe data and theoretical investigations indicated that **15.3b** remained neutral on the surface, retaining the 8π-electron state of the pyrazine ring.

### 2.3 B- and BN-Doped Coronenes

#### 2.3.1 Diboracoronenes

**Scheme 16:**
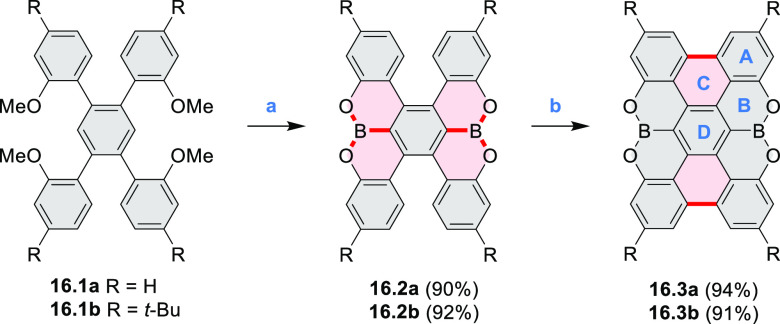
Synthesis of OBO-Doped *peri-*Tetracenes via Bistetracenes Reagents and conditions: (a)^29^ BBr_3_, *o*-DCB, rt to 150 °C, 12 h; (b) DDQ, TfOH, DCM, 0 °C to rt.

A synthesis of stable OBO-doped nanographenes was described in 2016 by Feng and Müllen et al.^29^ Treatment of hexabromobenzene with 2-methoxyphenylmagnesium bromide in THF provided **16.1a**,**b**, which were then heated in *o*-dichlorobenzene with BBr_3_ at 150 °C, to furnish the OBO-doped helical bistetracenes **16.2** with excellent yields (Scheme 16, see also Scheme 51, Section 3.1.2). Bistetracenes **16.2a**,**b** exhibited good stability and strong fluorescence (Φ = 61% for **16.2a** and 52% for **16.2b**) in comparison to their air-sensitive and nonfluorescent hydrocarbon analogue bistetracene. Single-crystal X-ray analysis revealed a double hetero[5]helicene structure with a highly twisted benzene ring. The cyclodehydrogenation of **16.2a**,**b** in the presence of DDQ/TfOH cleanly transformed the twisted bistetracene analogues into the planar nanographenes **16.3a**,**b**. In contrast to the unstable all-carbon *peri*-tetracene, the OBO-doped analogues **16.3a**,**b** displayed excellent stability under ambient conditions. Compound **16.3b** shows blue fluorescence with a quantum yield of 27%, with the emission spectrum being almost the mirror image of the low-energy absorption band. The Stokes shift was as small as 7 nm, indicating the rigid structure of this nanographene molecule. According to NICS calculations, the A and D rings in OBO-doped *peri*-tetracenes (Scheme 16) are highly aromatic. Ring B is nonaromatic, and ring C exhibits low aromaticity. These results are consistent with the Clar sextet formulation of **16.3a**,**b** and explain their different properties relative to their hydrocarbon parent.

**Scheme 17:**
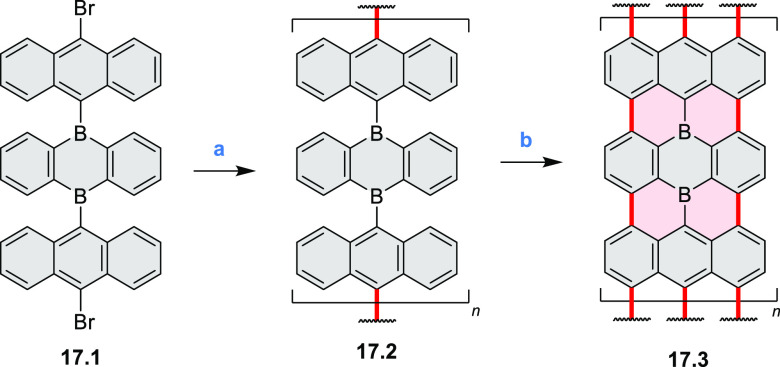
Bottom-Up Synthesis of Substitutionally Boron-Doped Graphene Nanoribbons Reagents and conditions: (a)^30,31^ Au(111), 200–220 °C; (b) Au(111), 300–400 °C.

Atomically precise introduction of group III dopant atoms into bottom-up fabricated semiconducting armchair graphene nanoribbons (AGNRs) was described by Fischer in 2015^30^ and further studied by Garcia-Lekue, Corso, and Pascual et al.^31^ Nanoribbon **17.3** was obtained in a two-step on-surface reaction of **17.1** (Scheme 17). A clean Au(111) single-crystal surface was precovered with precursor **17.1** and annealed to 200–220 °C for ∼5 min to activate its Ullmann-like coupling and polymerization into structure **17.2**. Further annealing to 300–400 °C (∼3 min) induced a cyclodehydrogenation reaction, leading to the completely planarized GNR **17.3**. Scanning tunneling microscopy (STM) topography revealed a characteristic modulation of the local density of states along the backbone of **17.3** that is superimposable with the expected position and concentration of dopant B atoms.

#### 2.3.2 BN-Containing Systems

**Scheme 18:**
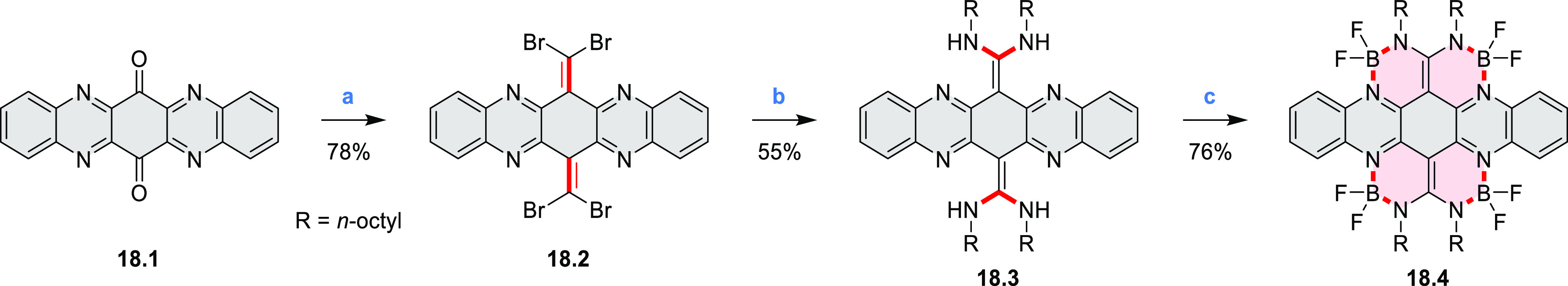
Coronenoid Containing Four NBN Units Reagents and conditions: (a)^32^ CBr_4_, PPh_3_, DCM, −50 to 25 °C; (b) R-NH_2_, *t*-BuONa, Pd_2_(dba)_3_, dppf, toluene, 120 °C; (c) BF_3_·Et_2_O, Et_3_N, DCM, 50 °C.

Heteracoronene **18.4** containing four embedded NBN fragments was described by Dou, Liu, and Wang (Scheme 18).^32^ Because of the presence of tetrahedral BF_2_ fragments, the system is not fully conjugated, yet it can be viewed as an example of potentially useful heterocyclization. Treatment of **18.1** with a mixture of CBr_4_/PPh_3_ in DCM afforded the tetra-brominated 6,13-dimethylene-6,13-dihydroquinoxalino[2,3-*b*]phenazine **18.2** as a yellow solid. **18.2** was then subjected to a Pd-catalyzed alkylamination reaction to give the alkylaminated precursor **18.3**. Finally, cyclization of **18.3** with BF_3_·Et_2_O/Et_3_N led to the simultaneous formation of four B–N rings, to produce **18.4** as a bluish violet solid. The compound displayed intense red fluorescence in toluene (Φ = 50%).

**Scheme 19:**
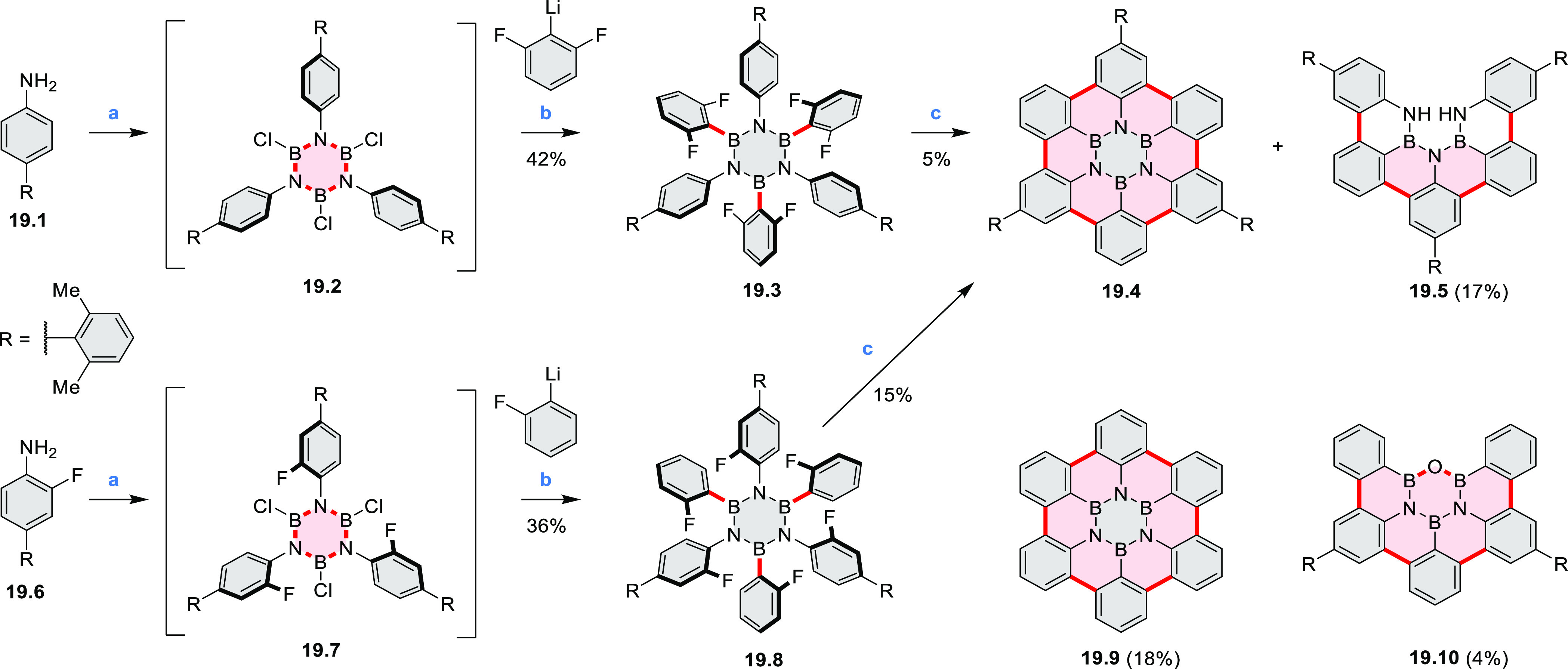
Synthesis of a BN-Doped Hexa-*peri-*hexabenzocoronene Reagents and conditions: (a)^33^ BCl_3_, toluene, reflux; (b) THF, −84 °C to rt; (c) [*i*-Pr_3_Si][CB_11_H_6_Cl_6_], Me_2_SiMes_2_, PhCl, 110 °C, Schlenk line.

A solution synthesis of a BN-doped HBC analogue in which the central benzene ring was replaced by a borazine core was described by Bonifazi and co-workers.^33^ The hexaaryl-substituted borazine precursor **19.3** was obtained in a reaction of 4-xylyl aniline **19.1** with BCl_3_ followed by addition of (2,6-difluorophenyl)lithium (Scheme 19). Borazine **19.3** was planarized into the target hexa-*peri-*hexabenzoborazinocoronene **19.4** in a reaction with [*i*-Pr_3_Si][CB_11_H_6_Cl_6_] and Me_2_SiMes_2_. Along with **19.4**, the partially fused BN derivative **19.5** was obtained as the major product (17% yield), suggesting that the ring closures proceed sequentially with the last aryl fusion likely being the rate-determining step. **19.4** exhibited strong blue-violet singlet emission and green phosphorescence.^34^ Higher cyclization yields were subsequently achieved for precursors containing singly fluorinated aryl groups (Scheme 19).^35^ In particular, **19.8** afforded borazino-coronene **19.4** in 15% yield, along with an unexpected cleavage product **19.10**. The insoluble unsubstituted **19.9**, previously reported in an on-surface synthesis (CR2017, section 2.3.2), was also obtained via the same silane-based protocol.^35^

**Scheme 20:**
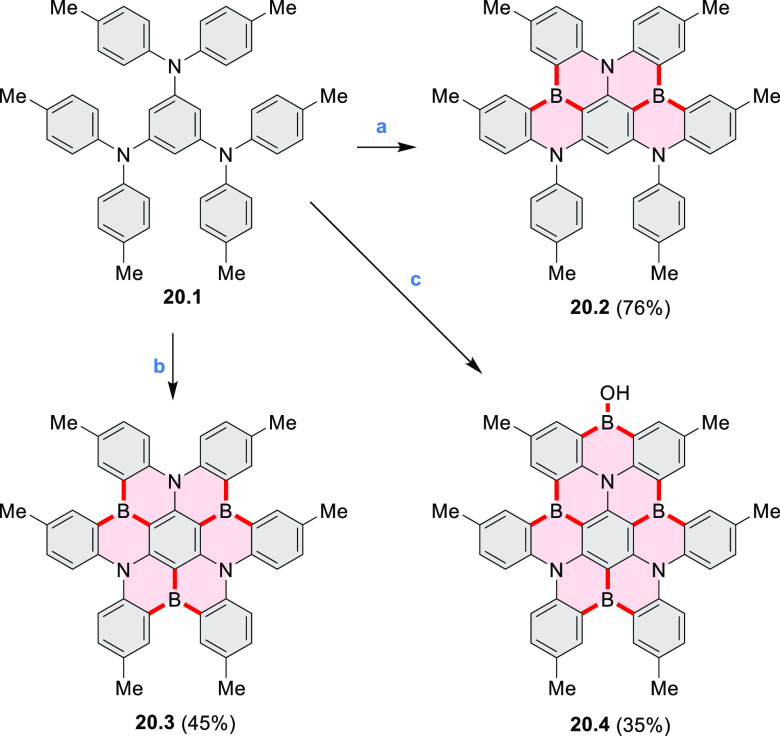
One-Shot Multiple Borylation toward BN-Doped Nanographenes Reagents and conditions: (a)^31^ BI_3_, Ph_3_B, *o*-DCB, reflux, 20 h; (b) BI_3_, Ph_3_B, 1,2,4-trichlorobenzene, 200 °C, 20 h; (c) BI_3_, *o*-DCB, reflux, 12 h.

A synthesis of BN-doped hexa-*cata*-hexabenzocoronene **20.3** was reported by Hatakeyama et al. in 2017 (Scheme 20).^36^**20.1** was obtained via the palladium-catalyzed C–N coupling between 1,3,5-tribromobenzene and di-*p*-tolylamine and subjected to one-shot quadruple borylation in the presence of 12 equiv of BI_3_, to afford the quadruply borylated **20.4** in 35% isolated yield and triple borylation compound **20.3** in only 3% yield. Other boron sources, like BCl_3_ and BBr_3_, did not give desired borylation products. In the presence of 5.0 equiv of BI_3_ and 2.0 equiv of Ph_3_B, selective double borylation took place under reflux in *o*-DCB to give **20.2** in 76% isolated yield. Triple borylation could be achieved at a more elevated temperature to give **20.3** as the main product (45% isolated yield). **20.2**, **20.3**, and **20.4** showed deep-blue fluorescence (at 488, 466, and 475 nm, respectively) and small energy differences between the excited singlet and triplet states (0.15–0.18 eV).

### 2.4 *peri-*Condensed Coronenes

**Scheme 21:**
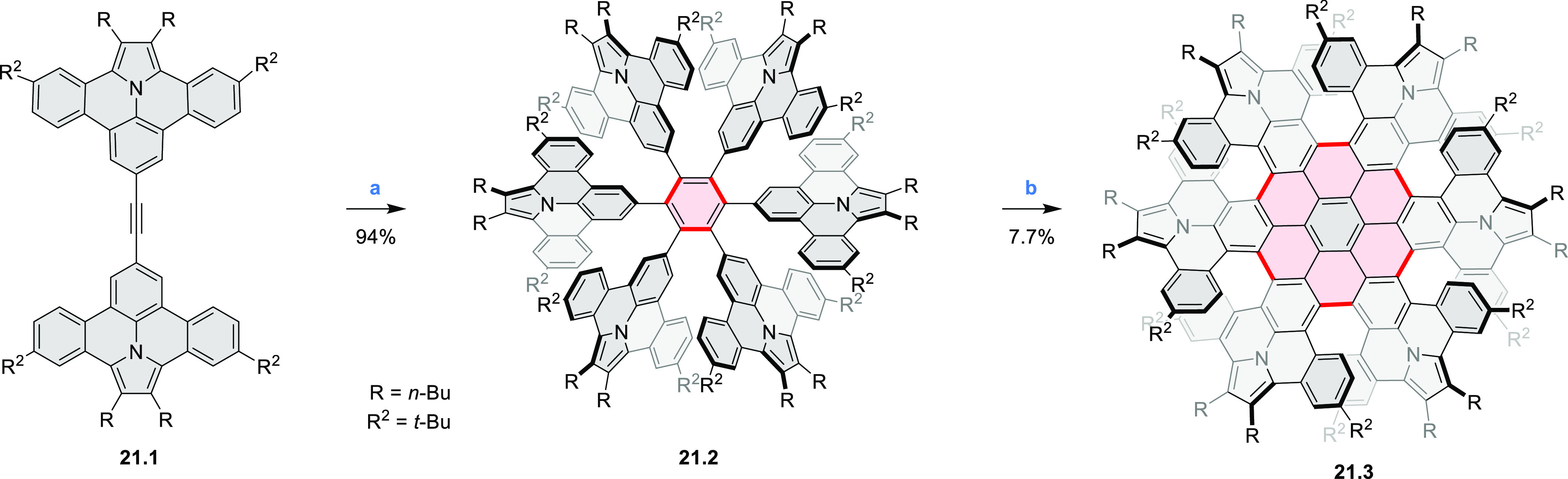
Propeller-Shaped Nanographene Reagents and conditions: (a)^37^ Co_2_(CO)_8_, dioxane, 120 °C; (b) *h*ν, CHCl_3_.

The propeller-shaped, nitrogen-doped hexapole [7]helicene **21.3** was reported by Wang and co-workers in 2019 (Scheme 21).^37^ A [2 + 2 + 2] cyclotrimerization of alkyne precursor **21.1**, catalyzed by Co_2_(CO)_8_ in dioxane at 120 °C, produced the hexaarylbenzene precursor **21.2** containing six dibenzoullazine arms. Extensive screening of cyclodehydrogenation conditions established that photooxidation of **21.2** in chloroform under an aerobic atmosphere was a practical method of preparing **21.3** in modest yield (ca. 8%). Unsuccessful attempts to obtain **21.3** via chemical oxidation indicated that the radical cation of the electron-rich dibenzoullazine unit in **21.2** is probably stable, which prevents it from undergoing further transformation. Although the detailed mechanism of the photochemical conversion of **21.2** into **21.3** was not clarified, control experiments indicated that both oxygen and chloroform were essential. No photoreaction was observed when a solution of **21.2** in chloroform was irradiated under a nitrogen atmosphere or when DCM was used as the solvent.

**Scheme 22:**
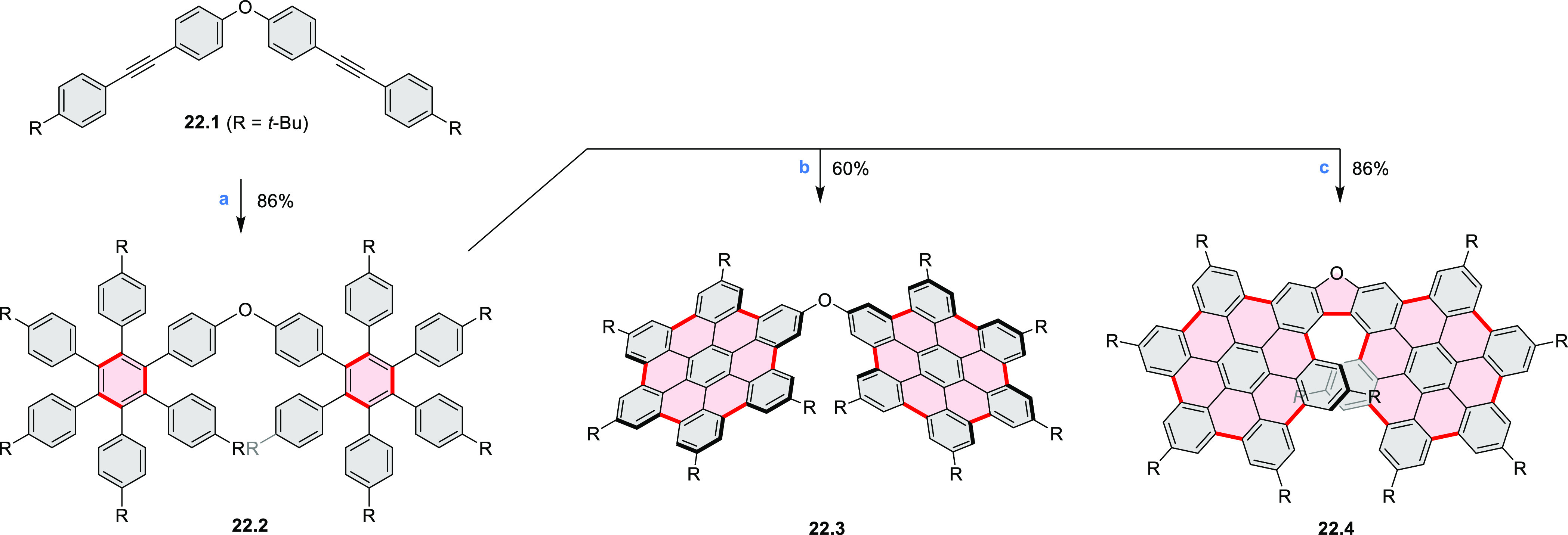
Synthesis of Oxa[7]superhelicene Reagents and conditions: (a)^38^ 2,3,4,5-tetrakis(4-*tert*-butylphenyl)cyclopenta-2,4-dien-1-one (2.5 equiv), toluene, N_2_, 23 h, 220 °C (pressure flask); (b) anhydrous FeCl_3_ (35 equiv), MeNO_2_, DCM, N_2_, 25 min at 0 °C, 20 h at rt; (c) DDQ (15 equiv), triflic acid (30 equiv), DCM, N_2_, 25 min at 0 °C, 20 h at rt.

A π-extended “superhelicene” containing two HBC units was synthesized by Jux and co-workers (Scheme 22).^38^ Their four-step synthesis started with diphenyl ether, which was first converted into its 4,4′-dibrominated derivative by aromatic halogenation and subjected to double Sonogashira coupling with 4-*tert*-butylphenylacetylene to provide **22.1**. A Diels–Alder reaction of **22.1** with 2.5 equiv of 2,3,4,5-tetrakis[4-(*tert*-butyl)phenyl]cyclopenta-2,4-diene-1-one yielded **22.2** which was reacted with DDQ and triflic acid in DCM to produce the helical compound **22.4**. The closure of the furan ring is most probably the final step of the cyclodehydrogenation cascade, as shorter reaction times (e.g., 2 h) furnished mixtures of helicene **22.4** and the incompletely cyclized **22.3**. Changing the reagents to FeCl_3_ in nitromethane/DCM allowed us to avoid the closure of the furan ring, thus forming **22.3** selectively. **22.3** was sensitive to light and transformed into helicene **22.4** through photocyclization, causing minor amounts of **22.4** to always be observed during photophysical measurements. **22.4** showed an almost 10^3^-fold amplification of photoluminescence dissymmetry factors *g*_PL_ of a π-extended superhelicene when embedded in an achiral conjugated polymer matrix, from approximately 3 × 10^–4^ in solution to 0.15 in a blend film in the solid state.^39^

**Scheme 23:**
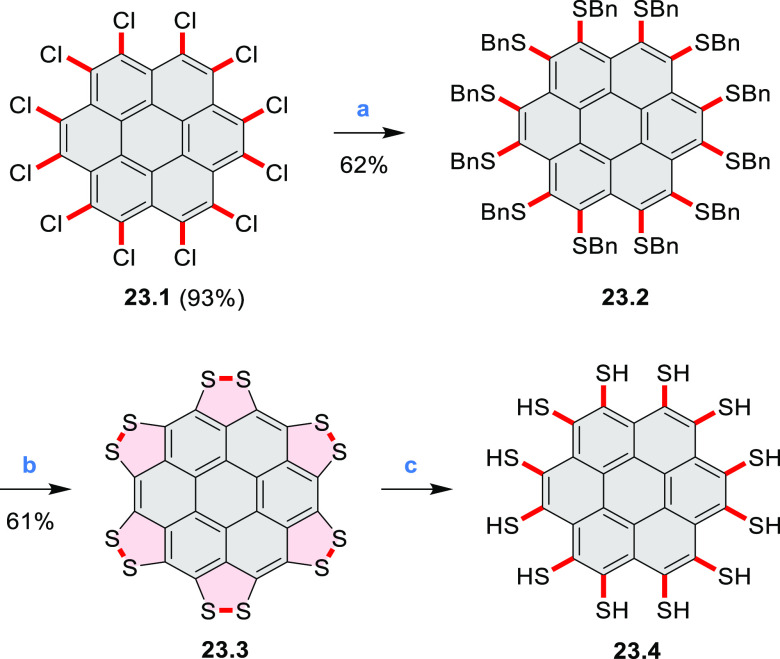
Persulfurated Coronene Reagents and conditions: (a)^40^ phenylmethanethiol, NaH, DMI, 0 °C to rt, 16 h; (b) (1) Li, THF, MeOH, NH_3_, −78 °C to rt, 4 h, (2) HCl/H_2_O_2_/water, rt; (c) NaBH_4_.

A persulfurated coronene “sunflower” **23.3** was reported in 2017 by Feng and Müllen et al. (Scheme 23).^40^ Its synthesis was carried out starting from coronene, which was chlorinated to the dodecachloro derivative **23.1**. Nucleophilic replacement of all peripheral chloro substituents was achieved using lithium benzylthiolate at rt, to afford dodecakis(benzylthio)coronene **23.2** as a red powder in 62% yield. After reductive cleavage of the protective benzyl groups under Birch conditions, the resulting dodecalithium species was treated with aqueous hydrogen chloride and hydrogen peroxide to afford the desired product **23.3** as a dark-red solid. Because of the low solubility of **23.3**, its characterization by NMR or XRD was not possible. The structure was studied by mass spectrometry, IR, and Raman spectroscopy and by scanning tunneling microscopy (STM). Compound **23.3** could be reduced with sodium borohydride to afford the more readily soluble perthiolated coronene **23.4**.

**Chart 3:**
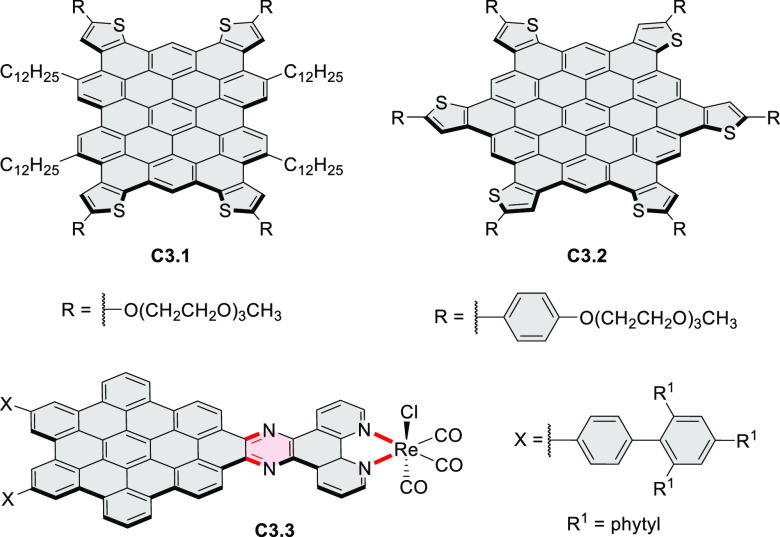
Peripherally Fused HBC Systems^41,42^

Thiophene-fused extended HBCs with the proposed structures **C3.1** and **C3.2** were reported by Jin et al. (Chart 3).^41^ These structures were prepared by FeCl_3_ oxidation of the corresponding hexaarylbenzenes, which were assembled using either the cobalt-catalyzed cyclotrimerization or the Diels–Alder cycloaddition route. The formation of **C3.1** and **C3.2** was validated only by mass spectrometry.

A well-defined nanographene–Re complex **C3.3** was synthesized by Li and co-workers.^42^ The ligand was obtained via condensation reaction of 1,10-phenanthroline-5,6-diamine with the corresponding coronene-diketone derivative (new bonds indicated in red, Chart 3). The ligand was then treated with an excess of Re(CO)_5_Cl in hot toluene to yield **C3.3**. The nanographene-containing complex showed a significantly less negative potential for electrocatalytic CO_2_ reduction as well as visible-light-driven photocatalytic CO_2_ reduction without the need for a photosensitizer.

### 2.5 *ortho*-Condensed Coronenoids

#### 2.5.1 Coronenoids Fused to Azaheterocycles

**Scheme 24:**
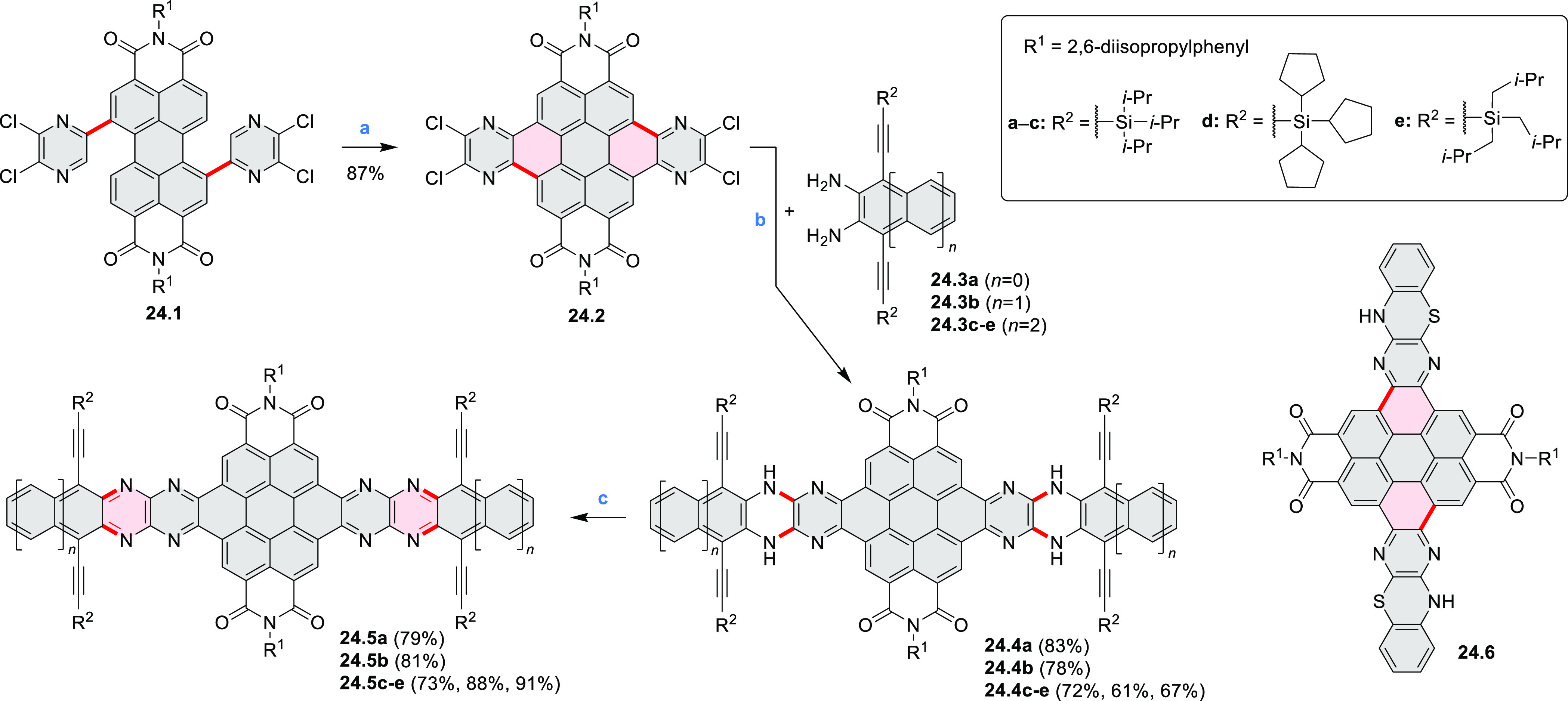
Coronene-Containing *N*-Heteroarenes Reagents and conditions: (a)^43^ I_2_, *hν*, rt; (b) Pd(dba)_2_, RuPhos, DIPEA, CHCl_3_, 60 °C; (c) MnO_2_, CHCl_3_, rt.

Three *N-*heteroarenes with azaacene units fused to a coronene nucleus were described by Bunz et al. (Scheme 24).^43^ Green light irradiation of a chloroform solution of **24.1** with a catalytic amount of iodine yielded the key coronene precursor **24.2**. Palladium-catalyzed coupling of this compound with *o*-diaminoarenes **24.3a**–**e** produced **24.4a**–**e** in good yields (up to 83%). Finally, the dihydropyrazine species **24.4a**–**e** were oxidized with MnO_2_ at rt. The resulting **24.5a**–**c** showed decreased solubility, as a consequence of increased π-stacking. The most soluble **24.5e** derivative was used for fabrication of a proof-of-concept thin-film transistor, yielding electron mobilities of 8 × 10^–4^ cm^2^ V^–1^ s^–1^ in polycrystalline films. This relatively low charge carrier mobility was attributed to the small domain size and polycrystalline nature of the fabricated films. A related phenothiazine-fused PDI **24.6** was synthesized by Aratani and Yamada et al.^44^ The latter system was prepared via DDQ/TfOH-mediated oxidation of the corresponding phenothiazine-linked PDI.

#### 2.5.2 Thieno-Fused Coronenoids

**Scheme 25:**
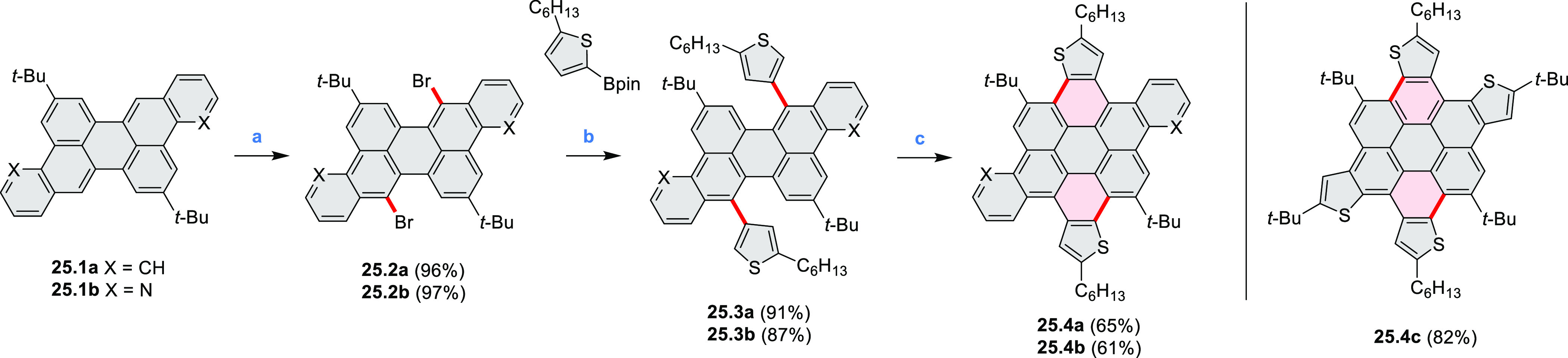
*cata*-Condensed Heteroannulated Coronenes Reagents and conditions: (a)^45^ 2.2 equiv of NBS, DCM, rt, 1 h; (b) 5 mol % of Pd_2_(dba)_3_, 7.5 mol % of *t*-Bu_3_P·HBF_4_, THF, K_2_CO_3_, 80 °C 16 h; (c) I_2_, *h*ν, propylene oxide, cyclohexane, 4 h.

A strategy toward tetraheteracoronenes derived from soluble diarenoperylenes was presented by Mastalerz et al. in 2018 (Scheme 25).^45^**25.1a**,**b** were selectively brominated at the 8,16 positions by treating 2.2 equiv of NBS in DCM at rt for 1 h. The aryl bromides **25.2a**,**b** were then appended with thienyl units via Suzuki–Miyaura cross-coupling, leading to **25.3a**,**b**. Finally, photocyclization of these compounds in the presence of catalytic I_2_ and propylene oxide as an acid scavenger provided the extended coronenes **25.4a**,**b** in 61–65% yields. Compound **25.4c** was obtained in the same manner, starting from a thiophene-substituted perylene.

**Scheme 26:**
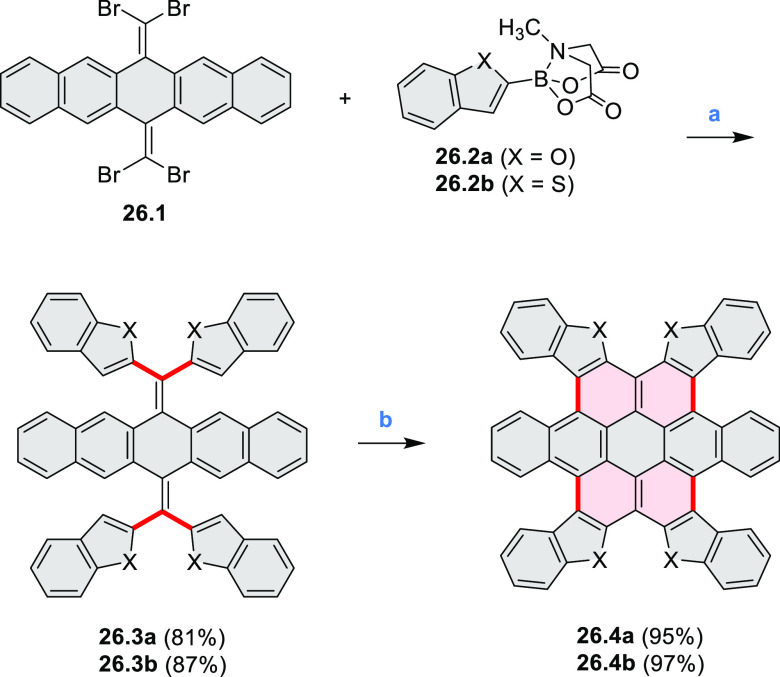
Synthesis of O*-* and S*-*Doped Contorted Coronenes Reagents and conditions: (a)^46^ Pd(OAc)_2_, SPhos, K_3_PO_4_, dioxane/water, 60 °C; (b) *h*ν, I_2_, toluene, 2-methyloxirane.

Benzofuran- and benzothiophene-fused analogues of *c*-HBC **26.4a**,**b** were synthesized by Loo et al. in 2015 (Scheme 26).^46^**26.1** was obtained via Corey–Fuchs reaction using isopropyl phosphite with tetrabromomethane and dibenzoanthraquinone. In the Suzuki–Miyaura coupling step, *N-*methyliminodiacetic acid (MIDA) boronates **26.2a**,**b** were used instead of boronic acid derivatives. Their gradual conversion to the boronic acid counterparts during the reaction allowed the coupling to compete favorably with boronic acid decomposition, thus leading to higher synthetic yields, particularly in the case of the 2-benzofuranyl derivative **26.2a**. Intermediates **26.3a**,**b** were then subjected to ring closing via photocyclization in toluene, leading to **26.4a**,**b**. Unlike in the syntheses of many *c*-HBC derivatives, chemical oxidation was not necessary to effect complete ring closure of the precursors to yield **26.4a**,**b**, likely because peripheral congestion was reduced by introduction of five-membered rings. Such benzofuran- and benzothiophene-containing *c*-HBC derivatives showed stronger visible-light absorption, in comparison with *c*-HBC, which was attributed to a simultaneous decrease in molecular symmetry and an increase in conjugation relative to the parent *c*-HBC compound.

**Scheme 27:**
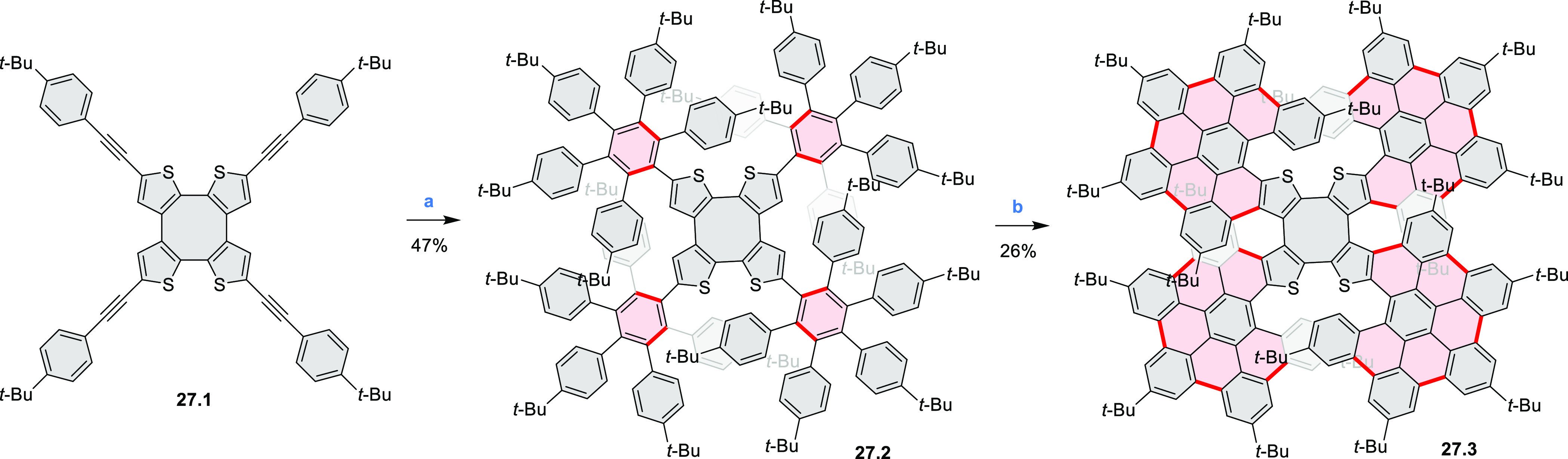
Sulfur-Doped Three-Dimensional Nanographenes Reagents and conditions: (a)^47^ 2,3,4,5-tetrakis(4-*tert*-butylphenyl)cyclopenta-2,4-dien-1-one, MW, 300 °C; (b) FeCl_3_, MeNO_2_, DCM, 0 °C.

Three-dimensional S*-*doped nanographenes featuring a cyclooctatetraene core **27.3** were reported by the groups of Molina-Ontoria, Guldi, and Martín (Scheme 27).^47^ The initial tetraalkyne **27.1** was prepared via Sonogashira coupling from a tetrabrominated cyclic tetrathiophene, which was obtained via an oxidative dimerization of 2,2′-dibromo[3,3′]bithiophene. Compound **27.1** was subjected to a microwave-assisted [4 + 2] cycloaddition with 2,3,4,5-tetrakis[4-(1,1-dimethylethyl)phenyl]2,4-cyclopentadien-1-one to give rise to **27.2**. Finally, the treatment of the latter species with FeCl_3_ produced the corresponding fully cyclodehydrogenated **27.3** in moderate yield. Two different crystal polymorphs of **27.3** were crystallographically characterized. **27.3** underwent triplet energy transfer to C_60_, but in contrast to its all-benzene analogue, it showed no electron transfer to TCNE.

**Scheme 28:**
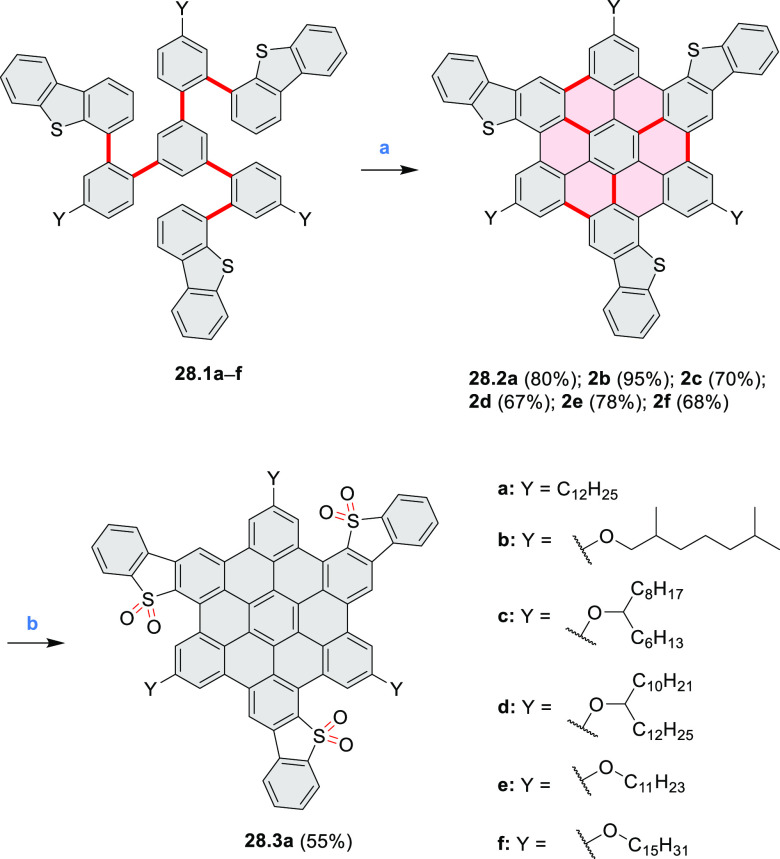
Tris(benzothiophene)-Fused Hexa-*peri-*hexabenzocoronenes Reagents and conditions: (a)^48^ FeCl_3_, DCM, MeNO_2_, rt, 1 h; (b) *m*-CPBA, THF, rt, 1 h.

A series of trisbenzothieno[1,2:7,8:13,14]hexa-*peri*-hexabenzocoronenes were synthesized by Pisula and Feng et al. (Scheme 28).^48^ Compounds **28.2a**–**f** bearing alkyl or alkoxy substituents were synthesized via an intramolecular oxidative cyclodehydrogenation reaction of triarylbenzenes **28.1a**–**f**. The products were well soluble in common organic solvents, including DCM, toluene, and THF. Unlike its alkoxy analogues, the alkyl-substituted **28.2a** could be selectively oxidized with *m*-CPBA to produce the triple sulfone **28.3**, which was obtained as a red powder. The HOMO level of a methoxy analogue of **28.2b**–**c** increased to −4.98 eV relative to the alkyl-substituted **28.2a** (−5.06 eV), while the LUMO level also increased from −1.80 eV (**28.2a**) to −1.74 eV (**28.2b**,**c**). The HOMO/LUMO levels of **28.3a** (−5.58/–2.34 eV) decreased compared with those of **28.2a** (−5.06/–1.80 eV), reflecting the conversion of the electron-rich benzothiophene ring into an electron-poor thiophene-*S*,*S-*dioxide unit.

**Chart 4:**
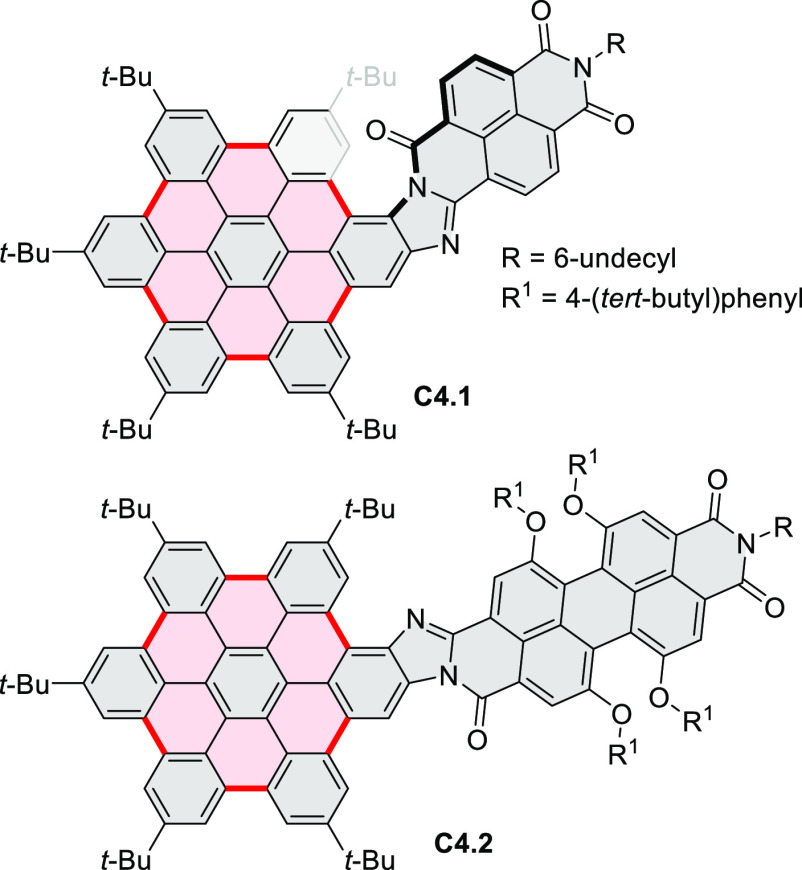
Ryleneimide–HBC Hybrids

Highly π-extended donor–acceptor hybrids consisting of a perylene or naphthalene diimide fused to a hexabenzocoronene core (e.g., **C4.1**–**2**, Chart 4) were described in 2020 by Guldi and Hirsch et al.^49^ The hexaphenylbenzenes were obtained from the corresponding brominated 1,2-ryleneimidebenzimidazole in a sequence consisting of palladium-catalyzed Sonogashira cross-coupling, Diels–Alder reaction, and FeCl_3_-mediated oxidative coupling. Linearly fused systems such as **C4.2** showed enhanced π conjugation, leading to red-shifted absorption and fluorescence. All conjugates revealed a CT character in the ground and excited states. For (PDI–HBC)s, the CT character of the ground state was reflected in a slight hypsochromic shift of the PDI absorption.

## 3 Perylenoids

### 3.1 Heteraperylenoids

#### 3.1.1 Monoheteraperylenoids

**Scheme 29:**
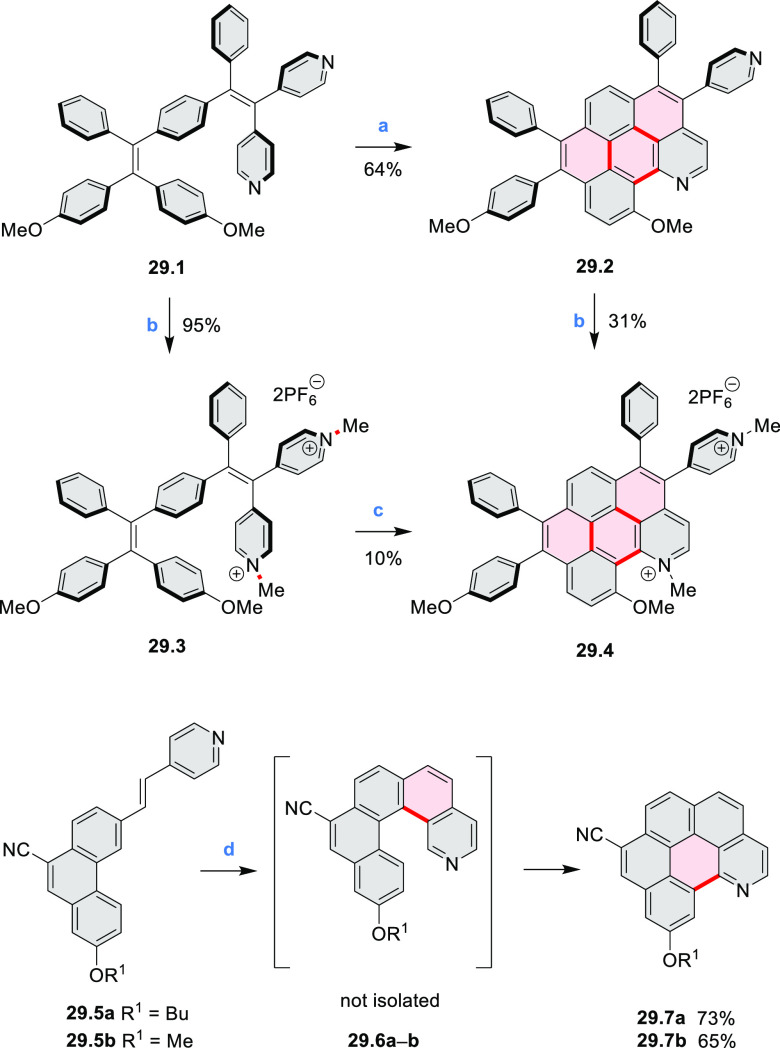
Synthesis of N*-*Doped Benzo[*ghi*]perylene and Its Dication Reagents and conditions: (a)^50^ I_2_, propylene oxide, *hν* (500 W high-pressure mercury vapor lamp), rt, 2 h, 64%; (b) (1) CH_3_I, MeCN, reflux, overnight, (2) KPF_6_, acetone, 2 h, 95%; (c) MeOH, *hν* (500 W high-pressure mercury vapor lamp), O_2_, 10%; (d)^51,52^ iodine, propylene oxide, toluene, *hν*.

Azaperylene **29.2** was obtained by Tang et al. via highly regioselective 3-fold photocyclodehydrogenation of the tetraphenylethylene derivative **29.1** (Scheme 29).^50^ In contrast to most PAHs, azaperylene **29.2** did not suffer from strong fluorescence quenching in the solid state and exhibited high emission quantum yields of 49% in solution and 21% as a solid. Dimers bound through π–π interactions were revealed in the solid-state structure of **29.2**, but further π-stacking was prevented by steric crowding. The dimeric nature of **29.2** was proposed to be responsible for the prominent red-shift of its emission in the solid state (λ_em_ = 566 and 609 nm) compared to that in solution (λ_em_ = 476 nm). Methylation of the pyridine units in **29.1** with iodomethane followed by ion exchange with potassium hexafluorophosphate led to **29.3** in 95% yield. This compound underwent photocyclodehydrogenation to **29.4** upon UV irradiation in the presence of oxygen. **29.4** could also be synthesized by dimethylation of **29.2**. While **29.3** was weakly emissive in solution, **29.4** displayed emission at 580 nm with a quantum yield of 18% in a PBS-buffered aqueous medium. The transformation from **29.3** to **29.4** was performed in HeLa cells upon UV irradiation, which resulted in a strong increase in fluorescence that could be observed through yellow or red channels.

A similar synthetic approach was employed by Raouafi and Aloui in their synthesis of N*-*doped benzo[*ghi*]perylenes **29.7a**,**b** (Scheme 29). Compounds **29.7a**,**b** were obtained via double oxidative photocyclization of π-extended stilbene derivatives **29.5a**,**b**. In the course of this reaction, the intermediate helicenes **29.6a**,**b** were not isolated. Target perylenes **29.7a**,**b** were well-soluble in common organic solvents and exhibited absorption and emission in the visible region.

**Scheme 30:**
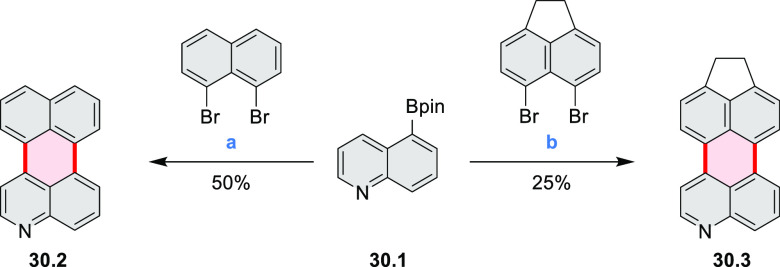
Synthesis of Azaperylenes Through Intramolecular C–H Activation Reagents and conditions: (a)^53^ [Pd_2_(dba)_3_]·CHCl_3_, P(*m*-tolyl)_3_, Cs_2_CO_3_, 1-chloronaphthalene, 160 °C, 16 h, 50%; (b) Pd(PPh_3_)_2_Cl_2_, Cs_2_CO_3_, mesitylene, 120 °C, 16 h, 25%.

Würthner and co-workers reported the synthesis of 3-azaperylenes through a cross-coupling methodology. In the reaction of 5-quinolineboronic ester **30.1** with 1,8-dibromonaphthalene at high temperature, the initial Suzuki coupling was followed by cyclization through C–H activation to provide the 3-azaperylene **30.2** (Scheme 30).^53^ This represents the first synthesis of unsubstituted 3-azaperylene, which was previously only isolated from natural sources. A different set of optimized conditions allowed us to perform this coupling with the more electron-rich 5,6-dibromoacenaphthalene to give **30.3**. Several nonheterocyclic analogues were prepared along with these examples. The N*-*doping was observed to cause a red-shift of emission (main peak at 457 nm in **30.2** and 488 nm in **30.3**) and an increase in Stokes shifts.

**Scheme 31:**
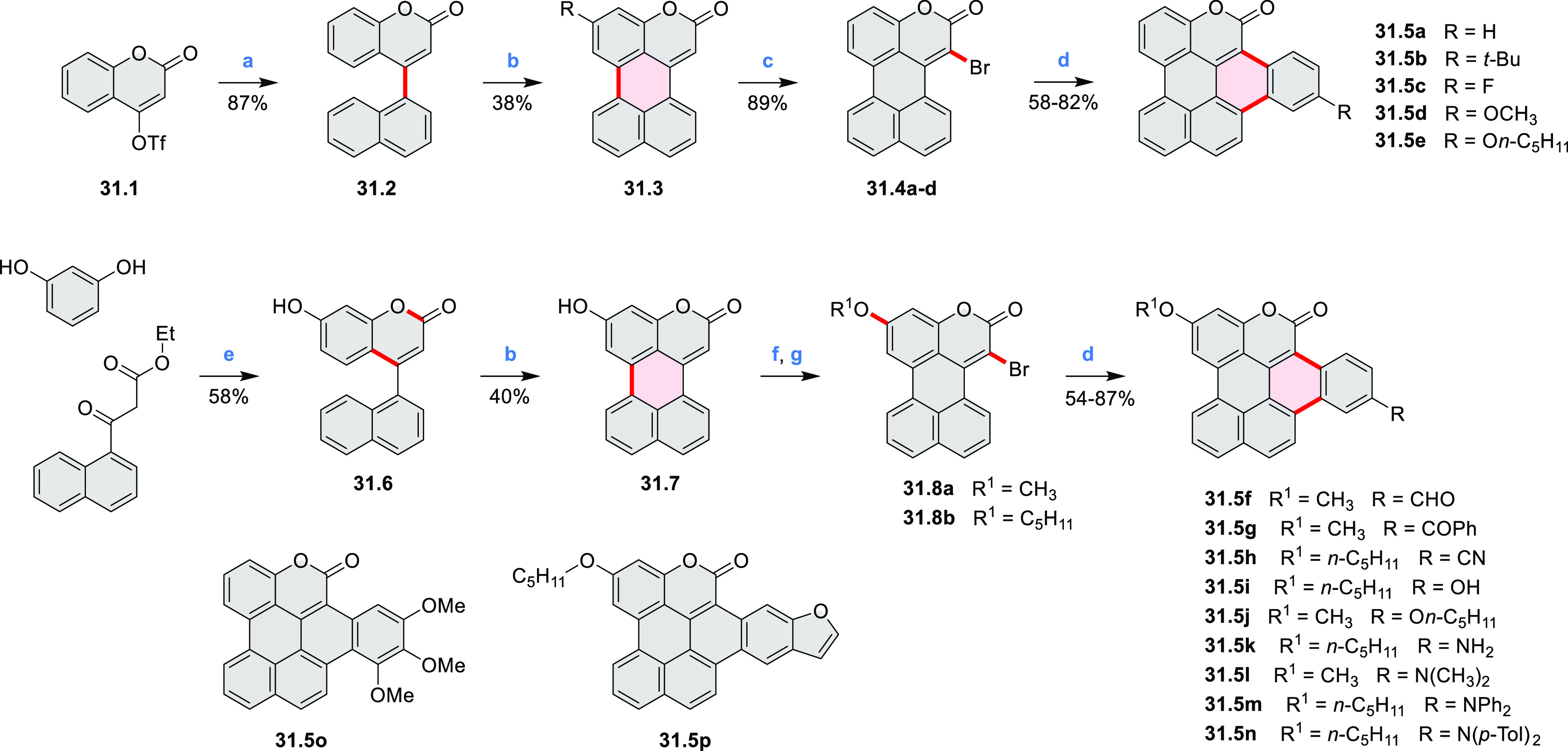
Synthesis of Extended Perylenoids Containing a Coumarin Substructure Reagents and conditions: (a)^55^ 1-naphthylboronic acid, Pd(PPh_3_)_4_, K_2_CO_3_, 4:1 toluene/H_2_O, 90 °C, 2 h, 87%; (b) AlCl_3_, NaCl, 140 °C, 4 h, 38%; (c) NBS, Bz_2_O_2_, DCM, 30 °C, 12 h, 89%; (d) RB(OH)_2_, Pd(PPh_3_)_2_Cl_2_, K_3_PO_4_, 5:1 EtOH/H_2_O, 90 °C, 8 h, then 5:1 EtOH/H_2_O, air, *hν* (blue LED), rt, 3 h, 54–87%; (e) MeSO_3_H, rt, 16 h, 58%; (f) MeI, K_2_CO_3_, DMF, rt, 5 h, 85% or 1-bromopentane, Na_2_CO_3_, KI, DMF, reflux, 16 h, 48%; (g) NBS, Bz_2_O_2_, CHCl_3_, rt, 1 h, 82–84%.

Dehydrogenative cyclization of 4-naphthylcoumarins via a variant of the Scholl reaction^54^ was used by the group of Zhang to synthesize perylenoid structures **31.3** and **31.7** (Scheme 31).^55^ These compounds were then selectively brominated and derivatized under optimized Suzuki coupling conditions. Irradiation of unprocessed Suzuki reaction mixtures with blue LED light under air resulted in an efficient electrocyclization and dehydrogenation, leading to the fused products **31.5a**–**p**. The final compounds were intensely fluorescent, with emission color tunable from blue to red depending on the substitution pattern. Peak emission wavelength varied from 424 nm in **31.5b**,**c** to 570–605 nm in the electron-rich compounds **31.5k**–**n**. These analogues also had the highest fluorescence quantum yields (**31.5l**: 67.9%, **31.5m**: 75.2%, **31.5l**: 53.0%).

**Scheme 32:**
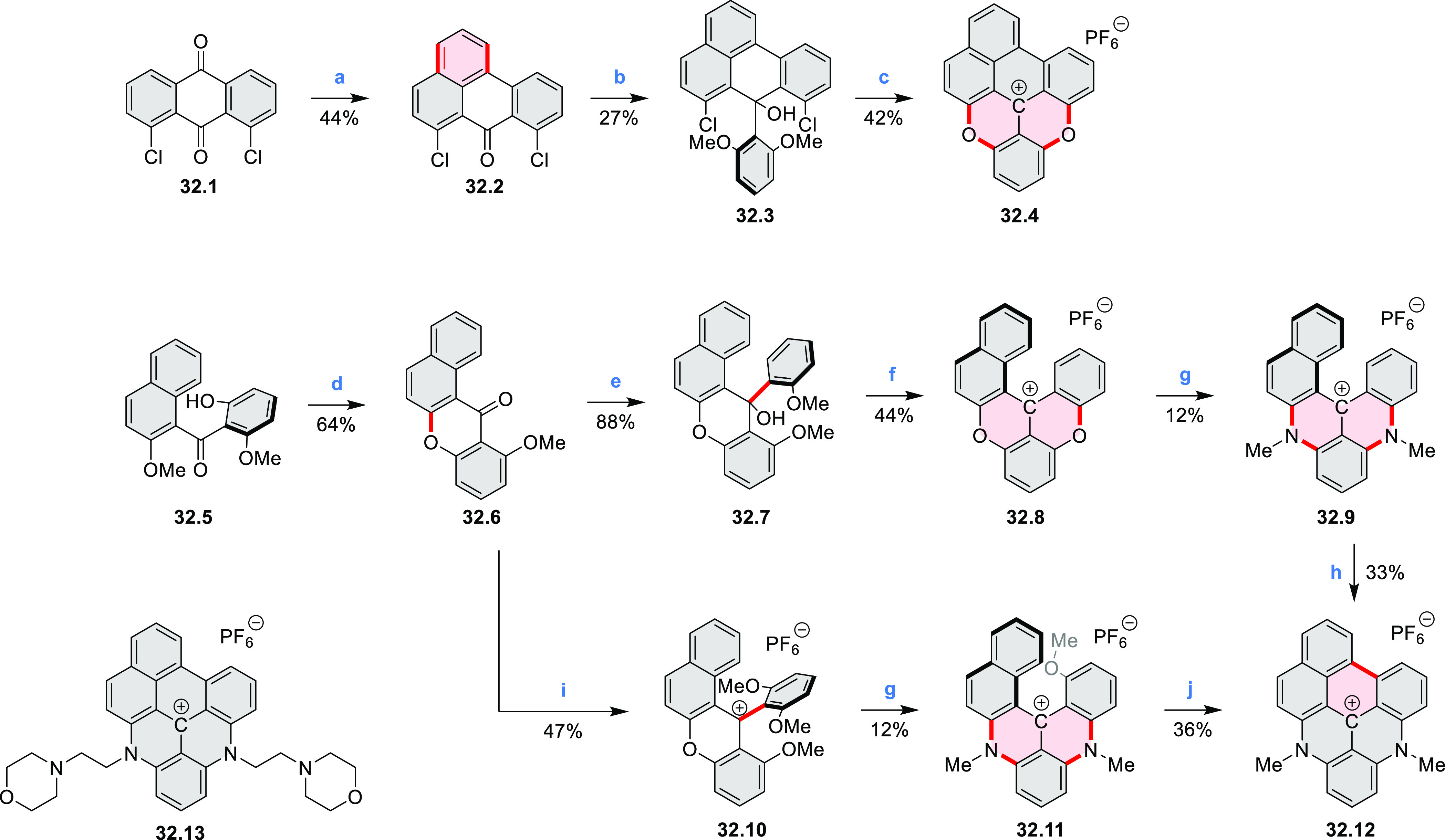
Synthesis of π-Extended Triangulenium Dyes Reagents and conditions: (a)^56^ (1) Al, H_2_SO_4_, 25 °C, 18 h, (2) glycerol, H_2_SO_4_, 125 °C, 3.5 h; (b) *m*-dimethoxybenzene, TMEDA, *n*-BuLi, 1:1 benzene/Et_2_O, rt, 1 h; (c) (1) 48% HBr_(aq)_, AcOH, reflux, 48 h, (2) 0.2 M KPF_6(aq)_; (d) neat, 225 °C, 5 h; (e) Li, *o*-bromoanisole, benzene/Et_2_O, reflux, 30 min; (f) pyridine hydrochloride, 190 °C, 5 min; (g) methylamine, PhCO_2_H, NMP, 90–95 °C, 18 h; (h) PPA, 110 °C, 30 h; (i) (1) *m*-dimethoxybenzene, TMEDA, *n*-BuLi, 1:1 benzene/Et_2_O, reflux, 2 days, (2) 6 M HCl_(aq)_, 0.2 M KPF_6(aq)_; (j) PPA, 110 °C, 1 h.

Diheteratriangulenium salts with a π-extension resulting in a monoheteraperylenoid substructure were evaluated as fluorescent dyes by Laursen and co-workers (Scheme 32).^56^ These compounds are extended analogues of a larger series of triangulenium fluorophores (see Chart 13 and Scheme 122, Section 4.1). Different synthetic pathways were suitable for dioxa and diaza variants. Addition of lithiated *o*-dimethoxybenzene to the dichlorobenzanthrone **32.2**, followed by a one-step dehydration, demethylation, and nucleophilic aromatic substitution, provided **32.4**. Unlike other dioxatriangulenium examples, **32.4** could not be transformed into its diaza analogues in reactions with primary amines. In contrast, condensation of carbenium salts **32.8** and **32.10** with methylamine gave the diazahelicenes **32.9** and **32.11**, respectively. Both compounds were cyclized to **32.12** via heating in polyphosphoric acid. Emission spectra of the resulting heteraperylenoids were red-shifted relative to their triangulenium analogues lacking the π extension. Compound **32.4** had its main emission peak at 595 nm (Φ = 78%), while for **32.12** a weaker red luminescence at 652 nm (Φ = 30%) was observed. While the solubility of these compounds prevented their use in cell imaging, the morpholine-appended compound **32.13** was successfully introduced into cells. It displayed emission enhancement upon DNA binding, which was attributed primarily to deaggregation.^57^

**Scheme 33:**
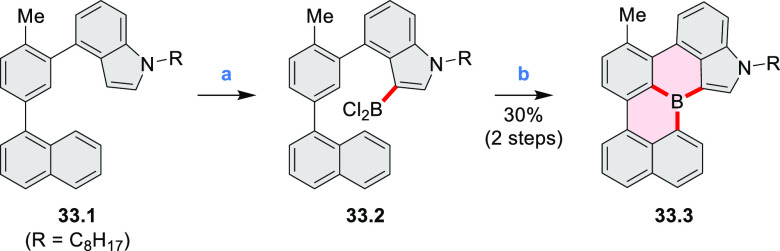
Indole-Fused Boraperylene Prepared by Electrophilic Borylation Reagents and conditions: (a)^58^ BCl_3_, 2,4,6-tri(*tert*-butyl)pyridine, AlCl_3_, DCM, 1 h; (b) 2,4,6-tri(*tert*-butyl)pyridine, 2,6-dichloropyridine, AlCl_3_, DCM, rt, 18 h.

An electrophilic borylation reaction was used to introduce boron as a fusion point in the indole-fused boraperylene **33.3** (Scheme 33).^58^ This was done through a two-step, one-pot sequence from **33.1**, where the 3-position of indole was borylated first, followed by further intramolecular borylation. Highly electrophilic borenium salts derived from chloroboranes and dichloropyridine in the presence of AlCl_3_ are thought to be the electrophilic species responsible for the second step of this process.^59^ To prevent indole oligomerization caused by strong acid, an excess of 2,4,6-tri(*tert*-butyl)pyridine was added as a noncoordinating base. Compound **33.3** was obtained in 30% yield, partly because of its vulnerability to protodeborylation, which caused significant product loss during chromatographic purification.

#### 3.1.2 Diheteraperylenoids

**Scheme 34:**
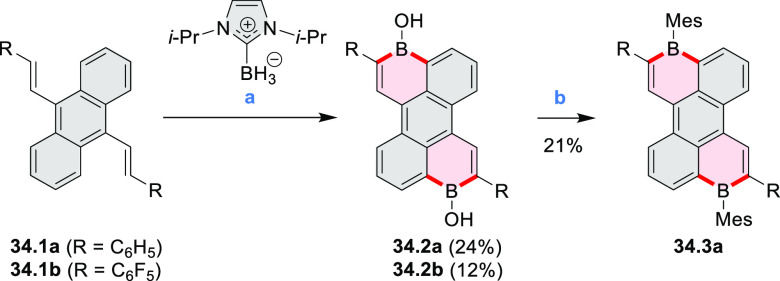
Synthesis of Boron-Doped Perylene Reagents and conditions: (a)^60,61^ (1) HNTf_2_, chlorobenzene, rt, 90 min then **34.1a** added, 110 °C, 5 h or **34.1b** added, 160 °C, 24 h, (2) TEMPO, 80 °C, 36 h for **34.1a** or 24 h for **34.1b**, (3) hydrolytic workup (b) (1) BBr_3_, DCM, rt, 24 h, (2) MesMgBr, toluene, rt.

In 2017, Würthner and co-workers used a new one-pot strategy in the synthesis of a stable 3,9-diboraperylene as its corresponding borinic acid **34.2a** (Scheme 34).^60^ The sequence consists of alkene hydroboration followed by C–H borylation with an NHC-borenium ion. The doubly boron-doped **34.2a** exhibits absorbance in the visible region, with the lowest-energy maximum at 561 nm and bright fluorescence in CHCl_3_ solution (λ_max_ = 603 nm, Φ = 63%). The electron-deficient analogue **34.2b** showed an even higher fluorescence quantum yield (λ_max_ = 603 nm, Φ = 0.95). Cyclic voltammetry studies performed on **34.2a** showed two reversible one-electron reductions at moderate potentials of −1.30 and −1.64 eV vs Fc^+^/Fc in DMSO. These two reduction potentials were anodically shifted relative to those of perylene. The *B-*hydroxyl groups in **34.2a** were subsequently replaced with *B-*mesityl groups, yielding **34.3a**.^61^ The B–C bonds in **34.2a** were also used as reactive handles for Suzuki coupling, giving a saddle-shaped hydrocarbon with two seven-membered rings.^62^**34.3a** was implemented in organic thin-film transistors (OTFTs), exhibiting n-type charge-carrier mobilities of 3 × 10^–3^ cm^2^ V^–1^ s^–1^. It was also used as an acceptor in combination with donor polymers in bulk-heterojunction solar cells with power conversion efficiencies of up to 3%.^61^ Moreover, this C–H borylation method was expanded to other polyaromatic boronic acids including triangulene (Chart 12, Section 4.1) and pyrenoid systems (Scheme 140, Section 4.3).

**Scheme 35:**
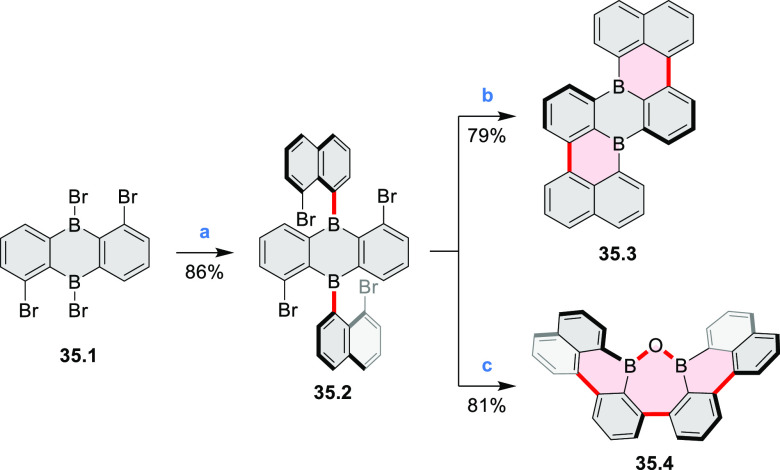
Synthesis of a Boron-Doped Tetrabenzopentacene Reagents and conditions: (a)^63^ 1,8-dibromonaphthalene, 1 equiv of *n*-BuLi, Et_2_O, 0 °C – rt, 45 min, then **35.1**, rt, overnight, 86%; (b) Ni(cod)_2_, cod, 2,2′-bipyridyl, pyridine, rt, 24 h, 79%; (c) Ni(cod)_2_, cod, 2,2′-bipyridyl, THF, rt, 24 h, 81%.

Wagner and co-workers reported the synthesis of a boron-doped tetrabenzopentacene **35.3** and oxadiborepin **35.4** from a single starting material (Scheme 35).^63^ First, a single lithium–halogen exchange of 1,8-dibromonaphthalene followed by condensation with **35.1** led to **35.2**, which was obtained as a mixture of atropisomers. Intramolecular Yamamoto coupling of **35.2** in pyridine led to the expected product **35.3**. However, performing the same reaction in THF provided the oxadiborepin **35.4** in high yield. The authors proposed that **35.3** undergoes a Ni-catalyzed transformation to **35.4** upon introduction of oxygen and moisture while quenching the reaction with a stream of air. The absence of this transformation in pyridine was attributed to its interaction with the Lewis-acidic boron atoms.

**Scheme 36:**
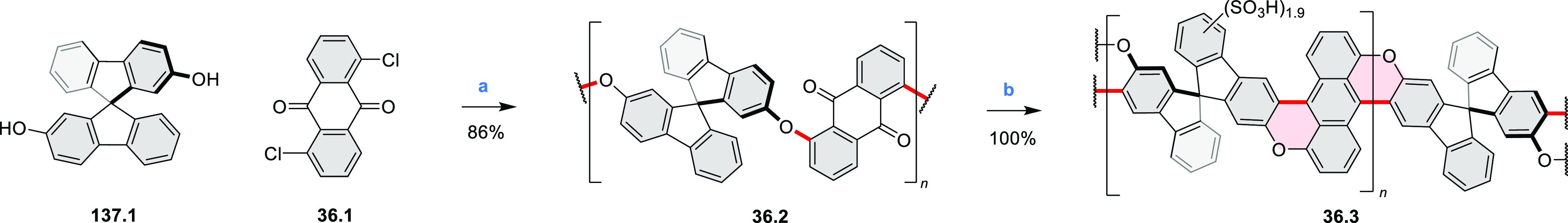
Synthesis of a Coil-Shaped Dioxaperylenoid Polymer Reagents and conditions: (a)^64^ K_2_CO_3_, diphenyl sulfone, 210 °C, 5 h; (b) (1) H_2_SO_4_, 160 °C, 6 h, (2) tetrabutylammonium iodide, 120 °C, 6 h.

A two-step procedure developed by Takata and co-workers provided access to two types of helical polymers containing either a dioxaperylene or dioxapyrene substructure (cf. Scheme 137, Section 4.3).^64^ Polycondensation of **137.1** with the anthraquinone spacer **36.1** efficiently gave the polymeric precursor **36.2** in 86% yield (Scheme 36). A subsequent intramolecular cyclization reaction of **36.2** upon treatment with H_2_SO_4_ quantitatively afforded a screw-shaped helical polymer **36.3**. The product was partially sulfonated, bearing an average of 1.9 sulfonic acid groups per monomer on its fluorene moieties. This caused **36.3** to be soluble in water and DMSO.

**Scheme 37:**
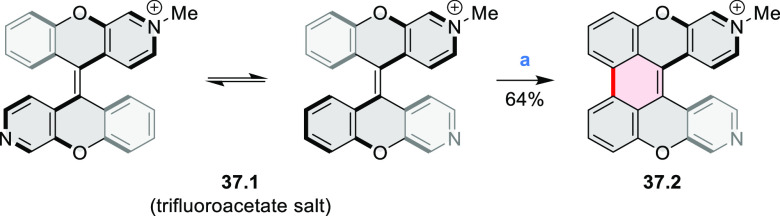
Photocyclization of Monomethylated Diazaxanthilidene Reagents and conditions: (a)^65^ H_2_O, *hν* (fluorescent light bulb), 3 days, 64%.

A 6π electrocyclization and oxidation upon prolonged irradiation with visible light converted the methylated diazaxanthilidene **37.1** into compound **37.2** that contained a dioxaperylene substructure (Scheme 37).^65,66^ This compound had red-shifted absorption and emission peaks (λ_abs_ = 499 nm, λ_em_ = 597 nm in H_2_O) compared to **37.1** (λ_abs_ = 410 nm, λ_em_ = 536 nm). Compound **37.1** and its dimethylated analogue showed good cell permeability and low cytotoxicity and were studied as dyes for bioimaging. Both dyes were observed to localize in lysosomes. Photocyclization of **37.1** to **37.2** was performed in live HeLa cells upon irradiation with 405 nm laser light. This transformation could be conveniently detected because of the significant difference in emission wavelengths of **37.1** to **37.2**. These features allowed labeling of individual cells in densely populated samples.

**Scheme 38:**
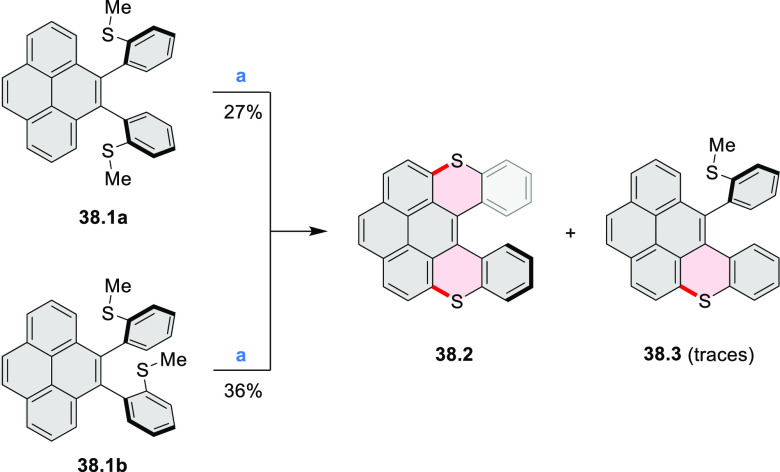
Synthesis of a Sulfur-Doped Perylenoid Reagents and conditions: (a)^67^ I_2_, CHCl_3_, 1 h at 70 °C, 1 h at 80 °C, 22 h at 90 °C.

In 2017, Liu and Fang developed an efficient five-step route toward a series of π-extended S-doped perylene (Scheme 38) and pyrene derivatives (Scheme 138, Section 4.3).^67^ The bis(*o*-methylthio)-substituted precursor for the final annulation step exists as a pair of atropisomers **38.1a** and **38.1b**, which can be cleanly separated using normal silica gel chromatography. When treated with I_2_ under the same conditions, **38.1a** and **38.1b** were converted into the same product **38.2** in 27% and 36% yield, respectively, along with a trace amount of the half-closed **38.3**. Crystal structures of **38.2** showed that the two terminal phenyl groups were bent symmetrically to the opposite sides of the pyrene plane, resulting in a helical structure with *C*_2_ symmetry. The dihedral angle between the helicene blades was 50.3° in the monoclinic polymorph and 62.4° in the triclinic one.

**Scheme 39:**
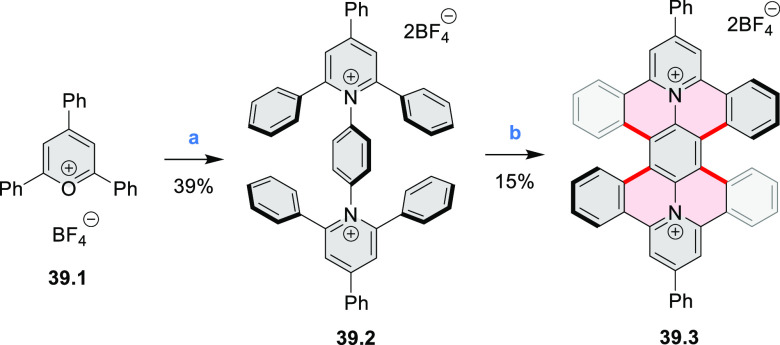
Synthesis of a Diazonia Derivative of Hexabenzoperylene Reagents and conditions: (a)^68^*p*-phenylenediamine, DMF, 150 °C, 20 h, 39%; (b) 2:5 MeOH/DCM, air, *hν* (450 W high-pressure mercury vapor lamp), 14 h, 15%.

Embedding of two pyridinium rings in the hexabenzoperylene framework of **39.3** was achieved by Sato and co-workers in a simple two-step procedure (Scheme 39).^68^ First, condensation of a triphenylpyrylium salt **39.1** with *p*-phenylenediamine gave the dipyridinium product **39.2**, which was photocyclized by UV irradiation in MeOH/DCM solution in the presence of air, providing **39.3** in 15% yield. This compound had a twisted double helicene structure and was determined to exist as a mixture of enantiomers (*meso* form was not found). **39.3** exhibited yellow luminescence in solution (λ_max_ = 548 nm, Φ = 22%), while the solid-state emission was much weaker (Φ = 0.4%) and red-shifted. **39.3** was also found to undergo four reversible reductions in voltammetric experiments.

**Scheme 40:**
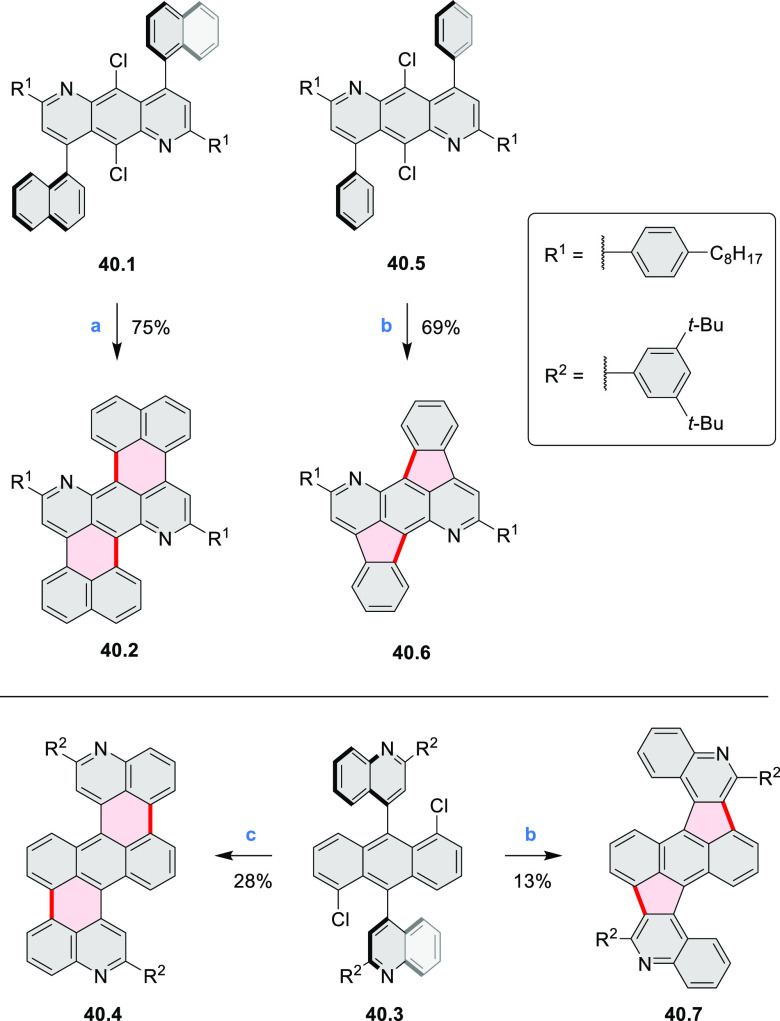
Synthesis of N*-*Doped Tetrabenzopentacenes and N*-*Doped Rubicenes Reagents and conditions: (a)^69^ KOH, *o*-xylene, reflux; (b) Pd(OAc)_2_, PCy_3_, DBU, DMA, 150 °C; (c) KOH, quinoline, 180 °C.

The preparation of previously unknown N*-*containing tetrabenzopentacenes **40.2** and **40.4** was reported by Gorodetsky and co-workers (Scheme 40).^69^ The final cyclization steps involved a base-mediated cyclodehydrohalogenation to form C–C bonds between the acene core and the pendant naphthyls or quinolines of **40.1** and **40.3**, respectively. Moreover, when precursor **40.3** or **40.5** was reacted under Heck-type coupling conditions, N*-*doped rubicene derivatives **40.7** and **40.6** were obtained, respectively. **40.2** displayed absorption peaks at 555, 600, and 650 nm, while the absorption features of **40.4** were slightly blue-shifted (543, 588, and 638 nm). Rubicene derivatives **40.6** and **40.7** showed blue-shifted absorption compared to tetrabenzopentacene derivatives. Within the N*-*doped derivatives, tetrabenzopentacenes, or rubicenes, the electronic properties were found be influenced by the precise location of the nitrogen dopants; in particular, **40.4** and **40.7** featured a lower-lying LUMO and HOMO than, respectively, **40.2** and **40.6**. From electrochemical measurements, HOMO and LUMO energies were calculated to be −5.23, −3.58 and −4.94, −3.50 eV, for **40.4** and **40.2**, respectively, in agreement with a computational study (−5.13, −3.11 vs −4.89, −2.87 eV).

**Scheme 41:**
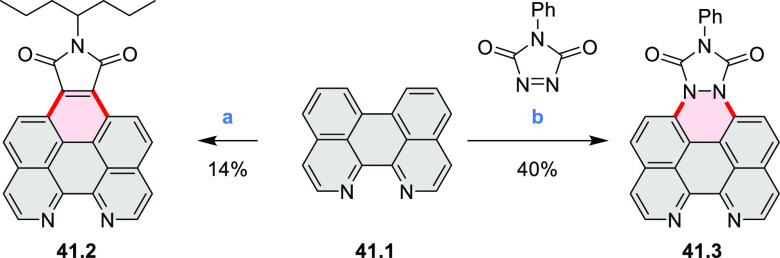
Synthesis of 7,8-Diazabenzo[*ghi*]peryleneimide and 1,2-Diazonia-7,8-diazabenzo[*ghi*]peryleneimide Reagents and conditions: (a)^70^ (1) maleic anhydride, *p*-chloranil, 220 °C, (2) 4-heptylamine, 150 °C; (b) *p*-chloranil, 150 °C.

Two planar N*-*doped benzo[*ghi*]perylene derivatives, namely, 7,8-diazabenzo[*ghi*]peryleneimide **41.2** and 1,2-diazonia-7,8-diazabenzo[*ghi*]peryleneimide **41.3**, were synthesized to study the effects of nitrogen lone pairs on the electronic structure of N*-*doped aromatics (Scheme 41).^70^**41.2** was obtained in 14% yield from 1,12-diazaperylene **41.1**, which was subjected to a Diels–Alder reaction with maleic anhydride followed by imidization with 4-heptylamine. **41.3** was similarly prepared via the Diels–Alder reaction of **41.1** but with 4-phenyl-1,2,4-triazoline-3,5-dione. Nitrogen atoms connected to the perylene core in **41.3** significantly increased its HOMO level, resulting in a DFT HOMO–LUMO gap of 2.63 eV, smaller than the value of 3.45 eV determined for **41.2**. This change caused a significant red-shift of the spectral features of **41.3**: in particular, its emission band was in the 570–780 nm range, while **41.2** emitted at 420–550 nm.

**Scheme 42:**
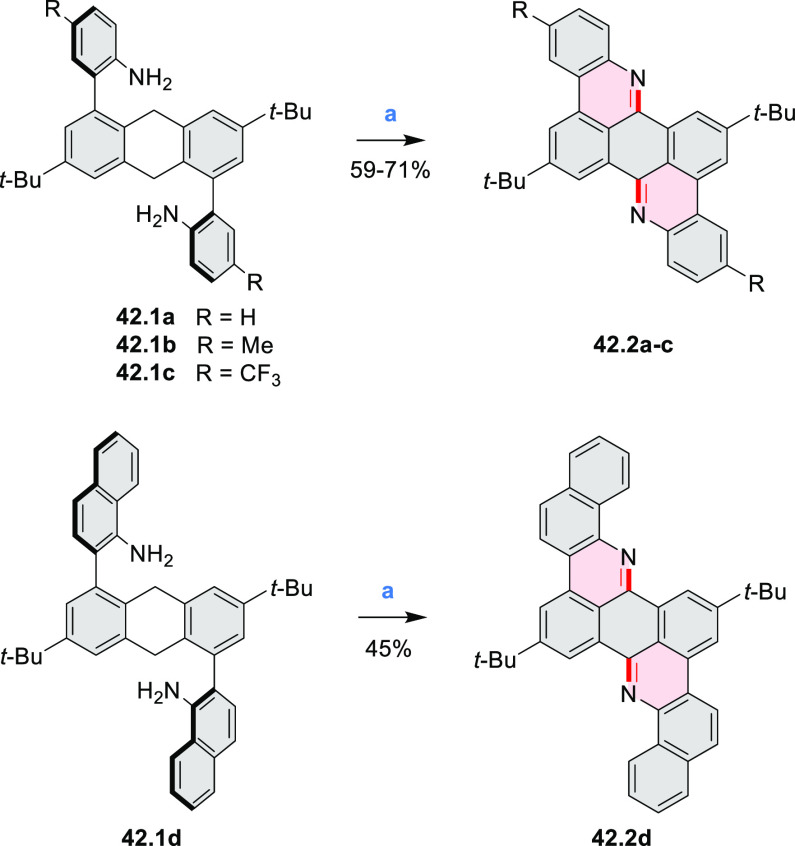
Synthesis of Diazaperylenoids via Metal-Free C(sp^3^)–H Aminations with Unprotected Anilines Reagents and conditions: (a)^71^*t*-BuOK, O_2_, DMF, 120 °C.

In 2017, Alabugin and co-workers developed a direct method for intramolecular C–H amination under mild conditions in the presence of KO*t*-Bu and molecular oxygen in DMF.^71^ The scope of this methodology was demonstrated by the synthesis of several quinolines and 9-azaphenanthrenes, as well as the dibenzo- and dinaphthoperylenes **42.2a**–**d** (Scheme 42). The reaction is thought to proceed via radical anion intermediates formed by deprotonation of the NH_2_ group followed by hydrogen atom abstraction from the adjacent methylene bridge by the DMF radical.

**Scheme 43:**
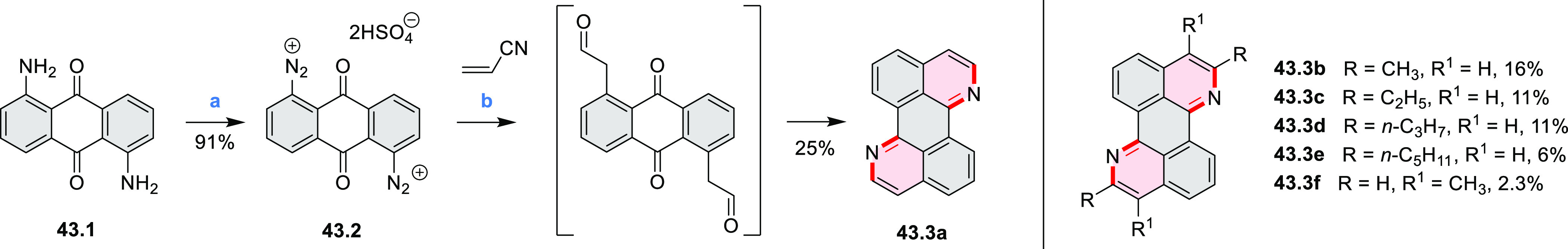
Synthesis of 1,7-Diazaperylene and Its Dialkyl Derivatives Reagents and conditions: (a)^72^ NOHSO_4_, conc. H_2_SO_4_, rt, 1 h, 91%; (b) CuCl, H_2_SO_4_, *t*-BuOH, 55–60 °C, then 10:1 25% NH_3_(aq)/CHCl_3_, rt, 24 h, 25%.

A scalable synthesis of 1,7-diazaperylene and its alkyl derivatives (**43.3a**–**f**, Scheme 43) was reported by Langhals and co-workers.^72^ Diazotization of 1,5-diaminoanthraquinone **43.1** provided the isolable and stable double diazonium salt **43.2**. The subsequent copper-catalyzed Meerwein arylation reaction with acrylonitrile provided the corresponding dialdehyde, which was used without purification in a condensation with NH_3_. The use of *t*-BuOH as the main solvent combined with H_2_O and H_2_SO_4_ was important to avoid the reduction of **43.2** to anthraquinone through hydrogen abstraction from a solvent molecule. Using acrylonitrile derivatives with a terminal alkene function furnished dialkylated 1,7-diazaperylenes, albeit in decreased yields. A very low yield was also obtained with an internal alkene (crotononitrile), leading to **43.3f**.

**Scheme 44:**
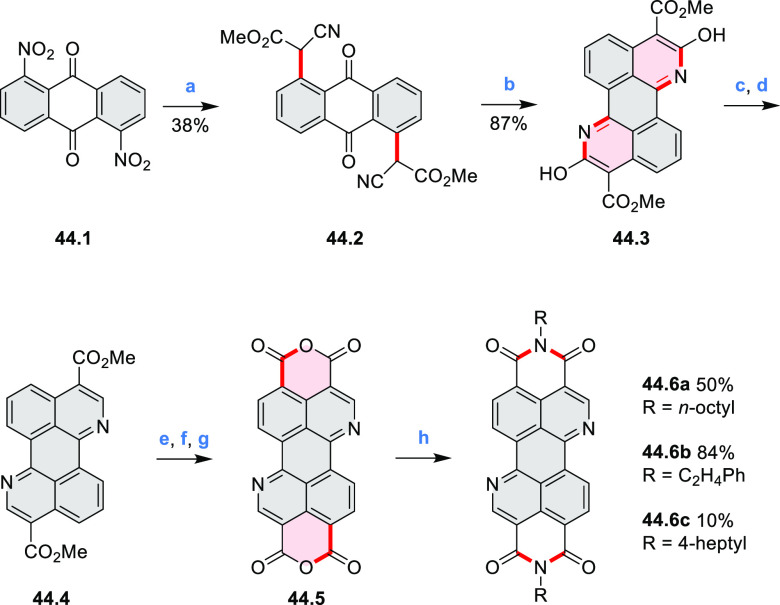
Synthesis of 1,7-Diazaperylene Diimides Reagents and conditions: (a)^73^ methyl cyanoacetate, KO*t*-Bu, DMSO, 50 °C, 3 h, 38%; (b) H_2_SO_4_, rt, 30 min, 87%; (c) Tf_2_NPh, DMAP, Et_3_N, DCM, −20 °C to rt, 2 h, 22%; (d) Pd(PPh_3_)_4_, HCOOH, Et_3_N, DMF, 80 °C, 2 h, 83%; (e) NBS, H_2_SO_4_, 50 °C, 2.5 h, 47%; (f) 2,4,6-trichlorophenyl formate, Pd(OAc)_2_, Xantphos, Et_3_N, toluene, 100 °C, 12 h, 22%; (g) TsOH·H_2_O, *o*-dichlorobenzene, 120 °C, 24 h, 89%; (h) RNH_2_, EtCOOH, *o*-dichlorobenzene, 150 °C, 11–38 h.

The synthesis of 2,7-diazaperylene diimides was recently described by Okamoto and co-workers (Scheme 44).^73^ The diazaperylene core in **44.3** was formed through condensation of the nitrile-functionalized anthraquinone **44.2**. The hydroxyl groups were then removed to afford the diester **44.4**. A challenging functionalization of this highly electron-deficient compound through bromination under harsh conditions and Pd-catalyzed carbonylation led to the dianhydride **44.5**. Finally, diimides **44.6a**–**c** were prepared through condensation with amines. In the solid state, C–H···N hydrogen-bonding contacts were observed, which, along with π-stacking interactions, governed the aggregation patterns. These compounds were successfully applied as n-type organic semiconductors with high electron mobility.^73−75^ Notably, their high chemical stability allowed the use of photolithography for patterning.^73^

**Scheme 45:**
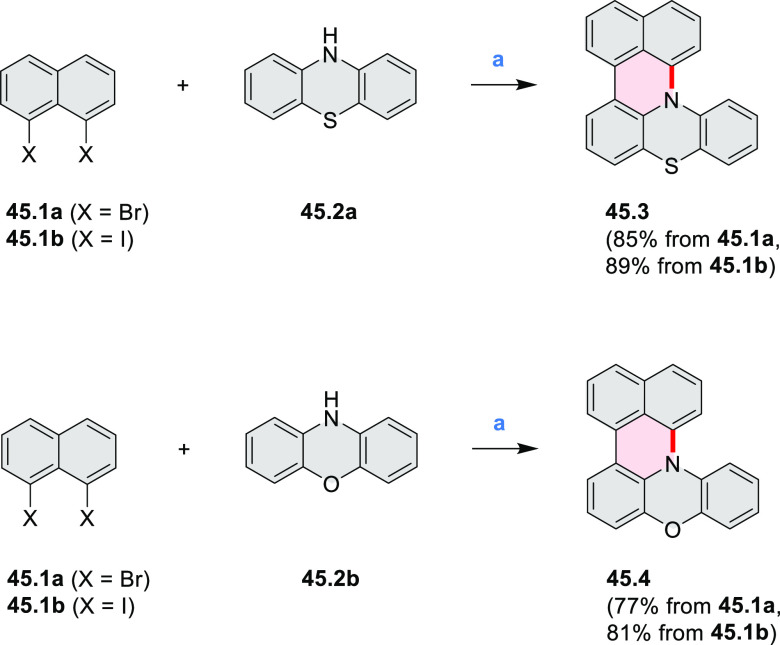
Synthesis of N,S*-* and N,O*-*Containing Benzoperylene Derivatives Reagents and conditions: (a)^76^*t*-BuONa, Pd(OAc)_2_, Pd_2_(dba)_3_, Cy_3_P, (*t*-Bu)_3_P, toluene, 90 °C, 10 h.

In 2016, Li and co-workers developed an efficient N–H/C–H one-pot coupling method for the preparation of benzo[*kl*]acridines, including the N,S- and N,O-containing benzoperylenes **45.3** and **45.4**, respectively (Scheme 45).^76^ This strategy is based on the reaction of 1,8-dibromonaphthalene **45.1a** or 1,8-diiodonaphtalene **45.1b** with a secondary aromatic amine. The reaction was proposed to proceed via Buchwald–Hartwig amination followed by intramolecular C–H arylation. The catalyst system used here was essentially a mixture of Pd sources and ligands known to be effective for these two transformations (Pd_2_(dba)_3_/(*t*-Bu)_3_P for the amination and Pd(OAc)_2_/Cy_3_P for the C–H arylation). The same palladium-catalyzed domino approach gave easy access to other benzo[*kl*]acridine derivatives (Scheme 183, Section 5.1.1; Scheme 294, Section 6.4.2). High yields were obtained using **45.2a**,**b** and other cyclic and acyclic diarylamines; carbazoles however were not suitable substrates.

**Scheme 46:**
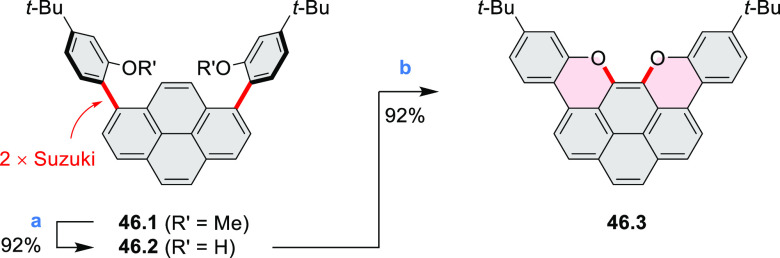
π-Extended Dioxaperylenoid Reagents and conditions: (a)^77^ BBr_3_, DCM, 0 °C to rt, overnight; (b) CuO, nitrobenzene, air, 200 °C, overnight.

In 2020, Bonifazi et al. reported the synthesis of oxygen-doped nanographenes, including the π-extended dioxaperylenoid **46.3** (Scheme 46).^77^ The important precursor **46.1** was obtained from the cross-coupling of 1,8-diiodopyrene and the appropriate arylboronic acid. Compound **46.1** was demethylated using BBr_3_ to give **46.2**. Subsequently, a 2-fold oxidative Pummerer cyclization using CuO in nitrobenzene at 200 °C furnished the target nanographene **46.3** in a high overall yield. Mixed-valence crystals containing the oxidized form of **46.3** were obtained using electrocrystallization with *n*-Bu_4_ClO_4_/THF as the electrolyte. The asymmetric unit [(**46.3**)_3_(ClO_4_)_2_·THF·0.5H_2_O] of the mixed-valence complex contains three independent molecules of **46.3** and two perchlorate anions. The molecules of **46.3** stack at distances of 3.21–3.42 Å, and they have similar bond lengths, indicating charge delocalization over the stacks. The authors also reported the construction of other oxygen-doped polyaromatic skeletons using the oxidative Pummerer reaction (see Scheme 119, Section 4.1, and Scheme 141, Section 4.3).

**Scheme 47:**
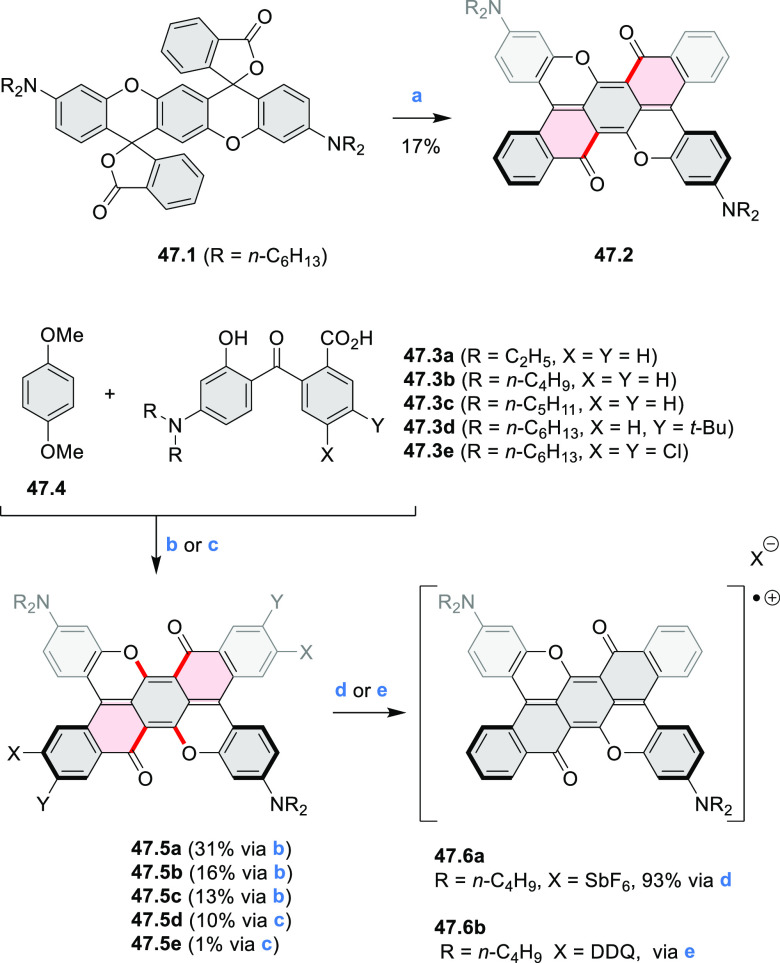
Synthesis of Bridged Tetraaryl-*p*-quinodimethanes Reagents and conditions: (a)^78^ conc. H_2_SO_4_, 140 °C, 3 h; (b) conc. H_2_SO_4_, 75 to 100 °C, 48 h, then 160 °C, 2 h (6 h for **47.5a**); (c) conc. H_2_SO_4_, 95 to 130 (150) °C, 78 (75) h (**47.5e**); (d)^79^ AgSbF_6_, DCM, rt; (e) DDQ, DCM, rt.

New stable derivatives of Thiele’s hydrocarbon, possessing a bridged tetraaryl-*p*-quinodimethane structure, were reported by Tanioka, Muranaka, Uchiyama, and co-workers.^78^**47.2** was obtained in 17% yield by treatment of *iso*-aminobenzopyranoxanthene **47.1** with concentrated sulfuric acid at high temperature (Scheme 47). Analogues bearing shorter *N-*alkyl chains **47.5a**–**c** were synthesized via a one-step reaction from the corresponding 2-(4-dialkylamino-2-hydroxybenzoyl)benzoic acids **47.3a**–**c** and *p*-dimethoxybenzene **47.4**. A *tert*-butyl-substituted derivative **47.5d** and chloro-substituted **47.5e** were also prepared in a similar manner, with the yields of 10% and 1%, respectively. Despite their quinodimethane substructure, **47.2** and **47.5a**–**c** showed no evidence of diradical character. Their closed-shell nature was attributed to the presence of a strong electron donor and acceptor moieties, favoring zwitterionic resonance forms over the diradical ones. The chemical oxidation of **47.5c** proceeded smoothly with an equivalent amount of silver hexafluoroantimonate in DCM, providing the radical cation salt **47.6a** in 93% yield after recrystallization (Scheme 47).^79^ The oxidation of **47.5c** was also achieved using DDQ in DCM. The resulting radical cation salts were stable in air and showed no evidence of dimerization in the solid state. **47.6a** had an intense NIR absorption with peaks at 927, 1007, 1094, and 1303 nm. Thin films of **47.6b** displayed high electric conductivity of 7.7 × 10^–3^ S cm^–1^.

**Scheme 48:**
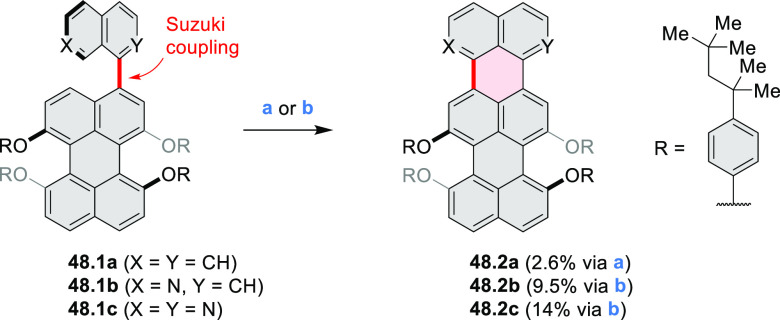
Synthesis of Azaterrylene Derivatives Reagents and conditions: (a)^80^ (1) Sc(OTf)_3_, DDQ, xylene, 165 °C, 1 day, (2) N_2_H_2_·H_2_O, rt, 1 h; (b) (1) potassium, xylene, 130 °C, 16–17 h, (2) air, 4 h.

Cyclizations of naphthyl-, azanaphthyl-, or 2,7-naphthyridinyl-substituted perylenes **48.1a**–**c** were reported by Hasobe and co-workers to produce terrylenes **48.2a**–**c** (Scheme 48).^80^ Oxidative coupling conditions were used for the naphthyl-containing substrate **48.1a**; this approach is however known to fail with electron-deficient N*-*doped compounds. Thus, cyclization of **48.1b**–**c** was performed under the radical anion coupling conditions (CR2017, Section 3.1), i.e., via treatment with potassium in xylene followed by air bubbling (cf. Scheme 84, Section 3.3). Electrochemical measurements revealed that the first one-electron reduction and oxidation potentials became positively shifted with the increasing number of nitrogens in the terrylene core (*E*_red1_ = −1.45, −1.28, and −1.12 V and *E*_ox1_ = 0.54, 0.66, and 0.75 V, respectively, in the series **48.2a**–**c**). The HOMO–LUMO gaps remained similar (2.39, 2.37, and 2.35 eV for **48.2a**–**c**, respectively), corresponding to an ca. 40 nm red-shift of absorption and emission features in **48.2c** compared to **48.2a**. Protonation of the N*-*doped terrylenes with TFA in DCM solution produced new NIR absorption bands: a broad peak between 600 and 820 nm for **48.2b** and a sharp peak at 800 nm for **48.2c**.

#### 3.1.3 Tri-, Tetra-, and Hexaheteraperylenoids

**Scheme 49:**
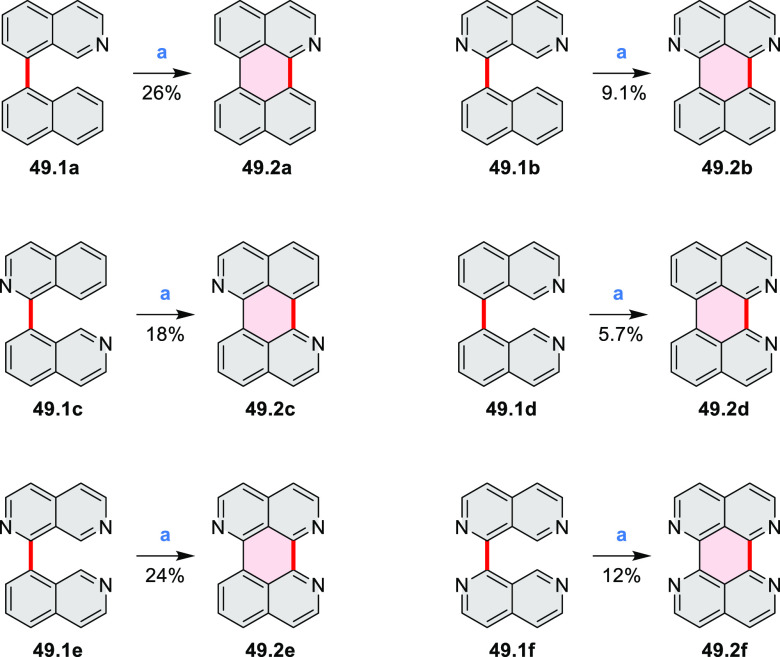
Synthesis of Azaperylenes via Radical Anion Coupling Reagents and conditions: (a) (1) 10–12 equiv of K, DME, rt, or DME, 100 °C for **49.2f**, or toluene, 95 °C for **49.2e**, ca. 1 day, (2) air, 4 h.

Electrochemical and photophysical properties of azaperylenes containing from 1 to 4 nitrogen atoms were compared in a study by Sato, Hasobe, and co-workers.^81^ The new diazaperylene **49.2b** and triazaperylene **49.2e** were prepared along with previously known compounds (Scheme 49). A common synthetic methodology based on radical anion coupling was used in all cases. Precursors **49.1a**–**f** were obtained via Suzuki or Negishi-type coupling reactions using derivatives of naphthalene, quinoline, and 2,7-naphthyridine as building blocks. The final cyclization was performed by treatment with K metal followed by exposure to air, providing **49.2a**–**f** in low yields. It was found that increasing the number of embedded nitrogens resulted in lower HOMO as well as LUMO energies, with only a small increase of the band gap. The emission quantum yield was significantly lowered in the triazaperylene **49.2e** (32%) and tetraazaperylene **49.2f** (0.013%) compared to **49.2a**–**d** (69–81%). This was explained by stabilization of energy levels of the nonemissive excited states corresponding to an n−π* transition. In the series of azaperylenes, compound **49.2d** had the highest proton affinity, which was attributed to intramolecular proton exchange between N atoms positioned in the bay region. Introduction of further N atoms in **49.2e**–**f** led to a lower proton affinity.

**Scheme 50:**
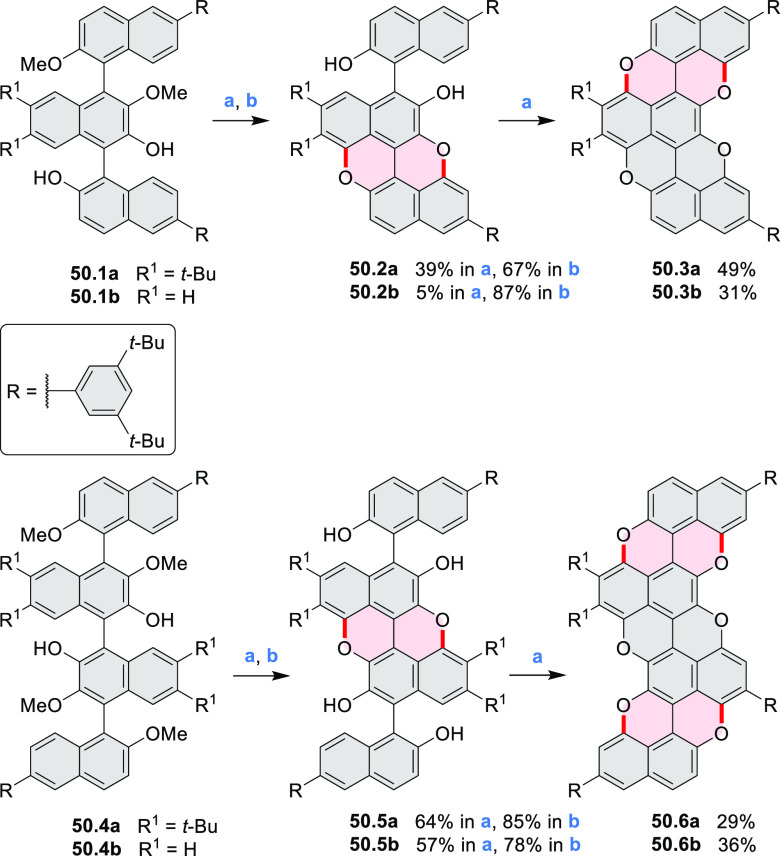
Synthesis of O*-*Doped Benzorylenes Reagents and conditions: (a)^82^ CuI, pivalic acid, DMSO, 130–145 °C; (b) BBr_3_, DCM, 0 °C.

In 2016, Bonifazi and co-workers described the preparation of O*-*doped benzo-fused rylenes by using a stepwise planarization strategy involving the formation of C–O bonds through an intramolecular oxidative coupling (Scheme 50).^82^ Intramolecular etherification of the quaternaphthalenes **50.4a** and **50.4b** by treatment with CuI and PivOH in DMSO afforded **50.5a**,**b** as mixtures of atropisomers. Demethylation of methoxy groups with BBr_3_ followed by the second ring-closure reaction led to the formation of the hexaoxa derivatives **50.6a** and **50.6b** in 29% and 36% yield, respectively. A similar sequence of transformations applied to **50.1a**,**b** yielded **50.3a**,**b**. Single-crystal X-ray diffraction analysis showed that the tetraoxa derivative **50.3b** forms face-to-face π–π stacks in the solid state.

**Scheme 51:**
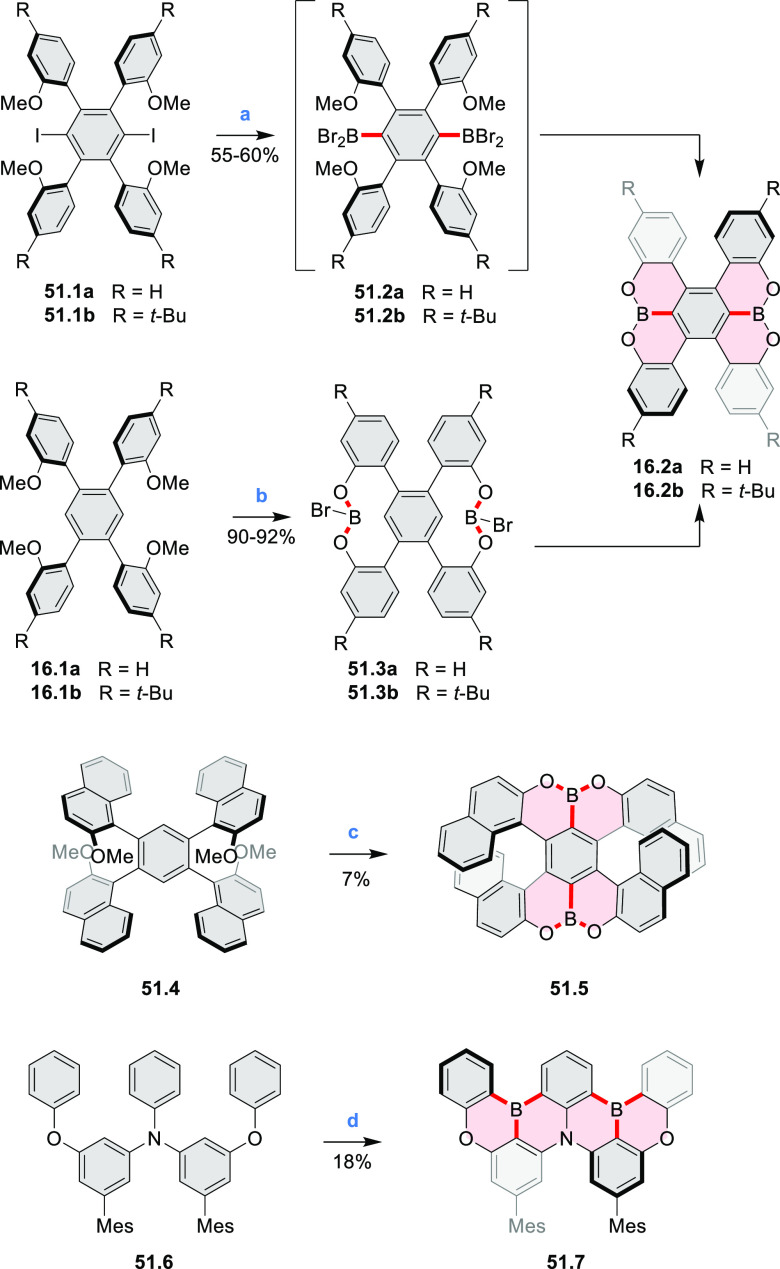
Synthesis of BO- and BNO-Doped Perylenoids Reagents and conditions: (a)^83^ (1) *t*-BuLi, chlorobenzene, −45 °C, 4 h or −45 to 0 °C, 3 h, (2) BBr_3_, (3) 40 °C, 24 h; (b)^29^ (1) BBr_3_, *o*-dichlorobenzene, 150 °C, 12 h; (c)^84^ BBr_3_, *o*-dichlorobenzene, 180 °C, 24 h; (d)^86^ 6 equiv of BI_3_, 1,2,4-trichlorobenzene, 180 °C, 20 h.

In 2016, Hatakeyama et al. reported a two-step synthesis of double [5]helicenes possessing two boronate substructures at the ring junction (Scheme 51).^83^ The annulation synthetic step consisted of lithium–halogen exchange of **51.1a**,**b** followed by trapping of the resulting aryllithium with boron tribromide to give the diborylated intermediates **51.2a**,**b**. These readily underwent boron-assisted demethylative cyclization at 40 °C providing **16.2a** and **16.2b** in 55% and 60% yield, respectively (Scheme 51). Shortly afterward, a more efficient synthesis of the same compounds was presented by Feng, Müllen, and co-workers (Scheme 51).^29^ Heating a solution of **16.1a**,**b** in *o*-dichlorobenzene at 150 °C in the presence of BBr_3_ gave the corresponding OBO-doped bistetracenes **16.2a**,**b** in 92% and 90% yield, respectively. The reaction was thought to proceed via demethylation to form the intermediates **51.3a**,**b** followed by intramolecular C–H borylation. Moreover, compounds **16.2a**,**b** were converted into OBO-doped peritetracenes by cyclodehydrogenation (Scheme 16, Section 2.3). The same methodology was also used to prepare the double [7]heterohelicene **51.5**.^84^ The low yield obtained in this example was attributed to the significant increase in steric strain upon cyclization. Electron-withdrawing character of the OBO fragments incorporated into **16.2a** allowed us to obtain its dianionic form upon treatment with Na or K in the presence of 18-crown-6.^85^ A related synthesis described by Hatakeyama and co-workers employed double borylation of the electron-rich compound **51.6** with BI_3_ to provide the fully fused product **51.7** through the formation of four new rings. Of the three [4]helicenes in this compound, the lateral ones had dihedral angles of 21.0° and 24.8° in the crystal structure, while the central helicene had a greater twist of 41.4°.

Blue luminescence of the products (**16.2a**: 405, 430 nm, Φ = 68%; **16.2b**: 411, 436 nm, Φ = 65%) was a very good match for pure RGB blue which is desirable in OLEDs.^83^ Compound **16.2b** had an increased barrier to epimerization compared to **16.2a** and was separated into enantiomers, allowing the detection of CPL with a dissymmetry factor of 1.7 × 10^–3^ in the optically pure material. Emission of the double [7]helicene **51.5** was weaker and red-shifted relative to the [5]helicene analogues, with a peak at 487 nm and a quantum yield of 26%.^84^ OLED devices prepared from **51.7** combined with charge-transporting polymers displayed emission at 505 nm, which was close to a pure green color.

**Scheme 52:**
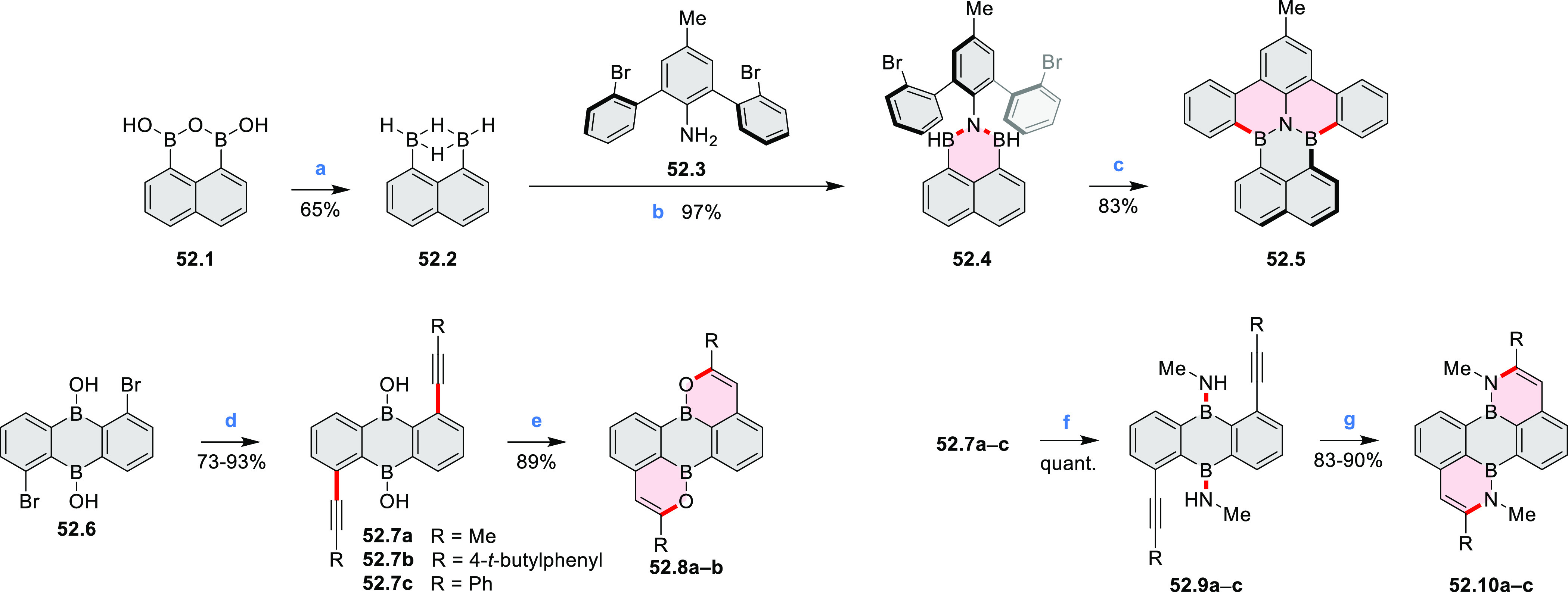
Synthesis of BN- and BO-Doped Perylenoids Reagents and conditions: (a)^87^ LiAlH_4_, Et_2_O, 0 °C to rt, 12 h, then Me_3_SiCl, −78 °C to rt, 12 h; (b) C_6_H_6_, 50 °C, 4.5 h; (c) Mg, THF, 50 °C, 2.5 h; (d)^88^ R–CC–SnR′_3_, Pd(P*t*-Bu_3_)_2_, toluene, 80 °C, 4 h, then rt, 14 h; (e) 10–20 mol % of (Ph_3_P)Au(NTf_2_), DCM, 60 °C, 4 h; (f) TMS_2_NMe, C_6_D_6_, 120 °C, 2 days; (g) 25 mol % of (Ph_3_P)Au(NTf_2_), DCM, 60 °C, 2 days.

The BNB-doped perylenoid **52.5** (Scheme 52) was reported by Wagner and co-workers together with smaller phenalenoid analogs with BNB or BOB doping.^87^ Reduction of the 1,8-naphthalenediboronic anhydride **52.1** provided the diborane **52.2**, which was then condensed with the aniline **52.3**, giving **52.4**. Treatment of this compound with Mg or K metal as a reducing agent resulted in B–C coupling, providing **52.5**. This compound had a twisted geometry and was a rare example of a cryptoracemate (a racemic mixture that forms chiral crystals). The same authors also reported a synthesis of bis-BO- and bis-BN-doped perylenoids (Scheme 52).^88^ The key transformation was the Au-catalyzed cyclization of alkynes with adjacent OH or NH functions. In this manner, borinic acids **52.7a**,**b** were converted into the tetraheteraperylenoids **52.8a**,**b** in 89% yield for both analogues. Aminoboranes **52.9a**–**c**, obtained via condensation of **52.7a**–**c** with TMS_2_NMe, were also cyclized by this methodology. Somewhat harsher conditions were needed, but the products **52.10a**–**c** were obtained in similarly high yields as **52.8a**,**b**.

**Scheme 53:**
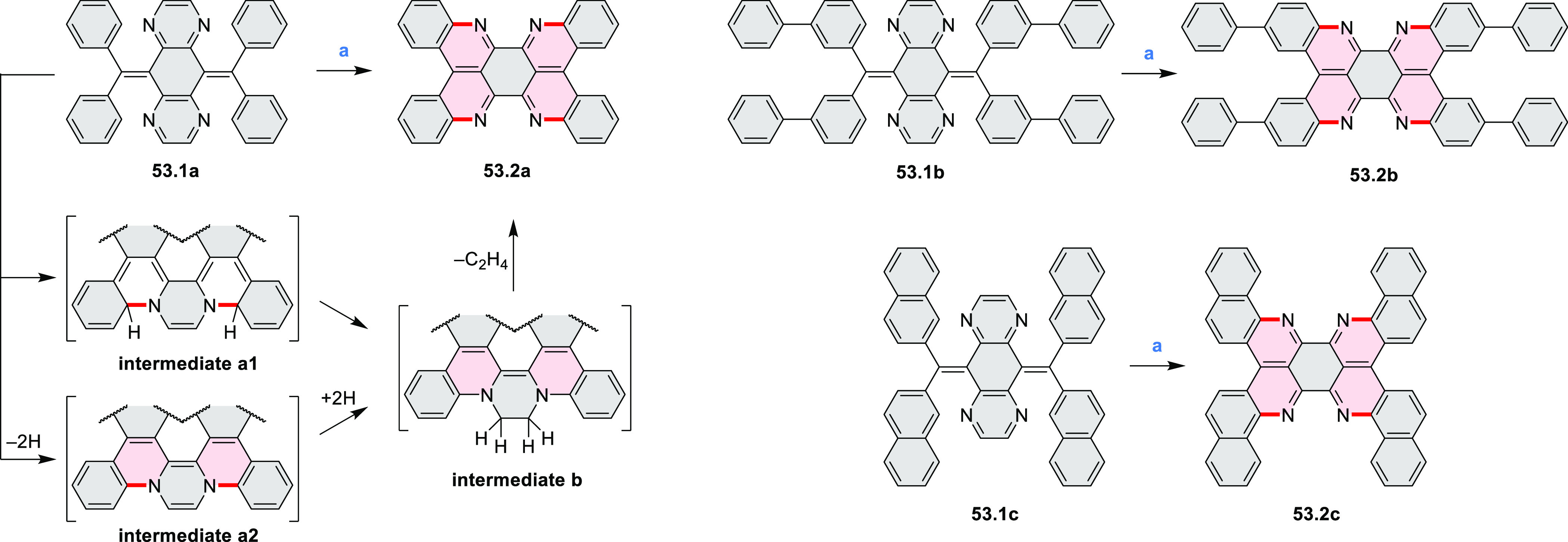
Formation of Tetraazaperylenoids on the Gold Surface Reagents and conditions: (a)^89^ Thermal deposition on the Au(111) surface in a vacuum and then annealing at 470–550 K.

Thermal annealing of tetraazaanthraquinodimethanes **53.1a**–**c** on the Au(111) surface caused a rearrangement with elimination of ethylene to form **53.2a**–**c** (Scheme 53).^89^ This process formed four new pyridine rings while breaking two pyrazine rings, a process named “heterocyclic segregation” by the authors. The transformation was proposed to proceed with a transfer of hydrogen atoms from the lateral phenyl rings to the pyrazine vinylene bridge. This transfer could plausibly be intramolecular (via intermediate **a1**) or mediated by the Au surface (via intermediate **a2**). Both types of intermediates formed from **53.1a** were detected after annealing at decreased temperatures (370–470 K). The products and intermediates were analyzed on the surface using STM, and no preparative synthesis was attempted.

**Scheme 54:**
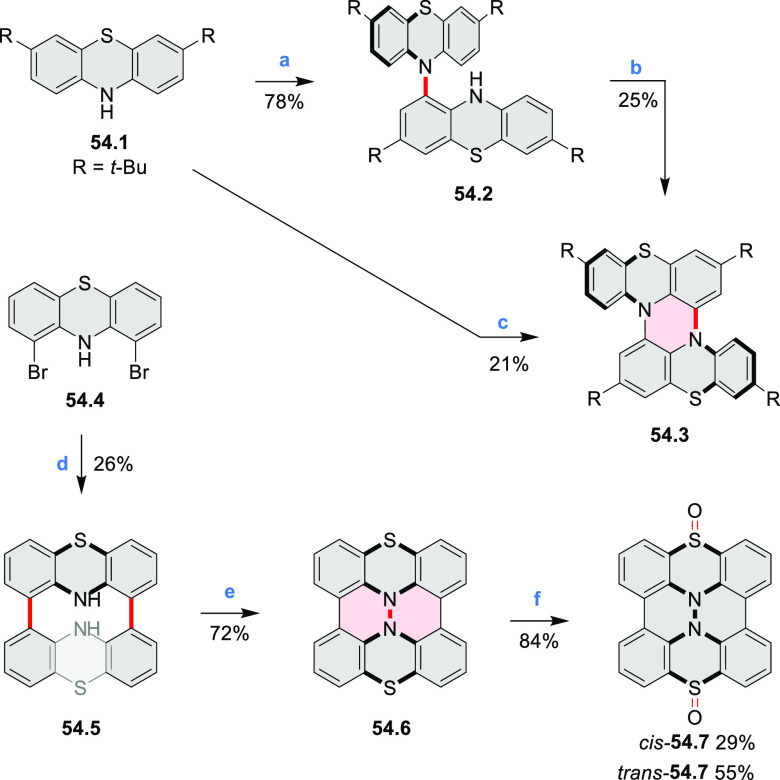
Synthesis of Phenothiazine Dimers Reagents and conditions: (a)^90^ DDQ, CHCl_3_, rt, 7 h; (b) DDQ, Sc(OTf)_3_, CHCl_3_, reflux, 23 h; (c) (1) DDQ, DCM, rt, 2 h, (2) DDQ, Sc(OTf)_3_, reflux, 18 h; (d)^91^ Ni(cod)_2_, cod, 2,2′-bipyridyl, THF, 60 °C, 24 h; (e) DDQ 3.1 equiv, CHCl_3_, rt, 24 h, then N_2_H_4_·H_2_O; (f) *m*-CPBA, CHCl_3_, rt, 40 min.

An N,S-doped dibenzoperylene derivative with a double hetero[4]helicene structure was synthesized via dimerization of phenothiazine (Scheme 54).^90^ The synthetic route started with oxidative homocoupling of **54.1** using DDQ to provide **54.2**. Then, oxidation of **54.2** by a combination of DDQ and scandium triflate resulted in intramolecular C–N bond formation and gave the fused phenothiazine dimer **54.3**. These two steps could also be performed in one pot. The enantiomers of **54.3** were much more stable toward racemization than other double [4]helicenes, presumably as a consequence of the highly bent structure of phenothiazine. Upon oxidation with BAHA, **54.3** was converted into a crystalline radical cation salt, which was stable under ambient atmosphere and lighting. The radical cation possessed a NIR absorption band at 1500 nm.

Another kind of phenothiazine dimer was obtained by Yamamoto homocoupling of the dibromophenothiazine **54.4** followed by DDQ oxidation to form the central N–N bond, providing **54.6**.^91^ This compound was further oxidized to the disulfoxide **54.7** upon treatment with *m*-CPBA, resulting in a *cis*/*trans* mixture due to the sulfoxide geometry. The respective disulfone could also be formed using an excess of *m*-CPBA but was not characterized due to its low solubility. **54.6**–**7** had a “butterfly” structure with a ridge formed by the hydrazine substructure. **54.6** was nonemissive, while the two isomers of **54.7** displayed emission close to 540 nm. Compound *cis*-**54.7** had a stronger emission (Φ = 15%) than its *trans* isomer (Φ = 5%).

**Scheme 55:**
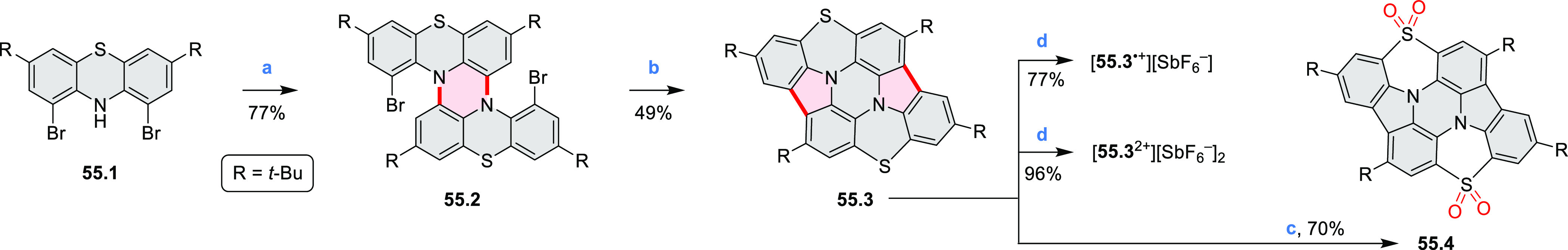
Synthesis of Phenothiazine-Containing Buckybowls Reagents and conditions: (a)^92^ CuI, K_2_CO_3_, 18-crown-6, *o*-dichlorobenzene, 180 °C, 48 h; Pd(OAc)_2_, PCy_3_·HBF_4_, K_2_CO_3_, DMA, 170 °C, 48 h; (c) 30% H_2_O_2_, AcOH, CHCl_3_, 60 °C, 12 h; (d) AgSbF_6_ (1 or 2 equiv), dry DCM, rt, 20 min.

In 2021, Zhang et al. described the synthesis of the thiazine-fused buckybowl **55.3** and its derivatives (Scheme 55).^92^ The synthesis began with the copper(I)-catalyzed cyclodimerization of the dibromophenothiazine **55.1**. The same transformation under Buchwald–Hartwig conditions only led to a complex product mixture. The resultant compound **55.2** was then cyclized under palladium catalysis to give the target buckybowl **55.3** in 49% yield. Upon oxidation by H_2_O_2_, the corresponding bis(*S*,*S-*dioxide) **55.4** was obtained in 70% yield. Chemical oxidation of **55.3** could take place using 1 or 2 equiv of AgSbF_6_ to give the radical cation **55.3**^•+^ and the dication **55.3**^2+^, respectively. According to single-crystal XRD data, the bowl depth of the neutral compound **55.3** (0.59 Å) was deeper than that of the radical cation (0.37 Å), respectively, while the dication assumed a planar geometry.

### 3.2 [*ghi*]Heteroannulated Perylenoids: Five-Membered Rings

**Scheme 56:**
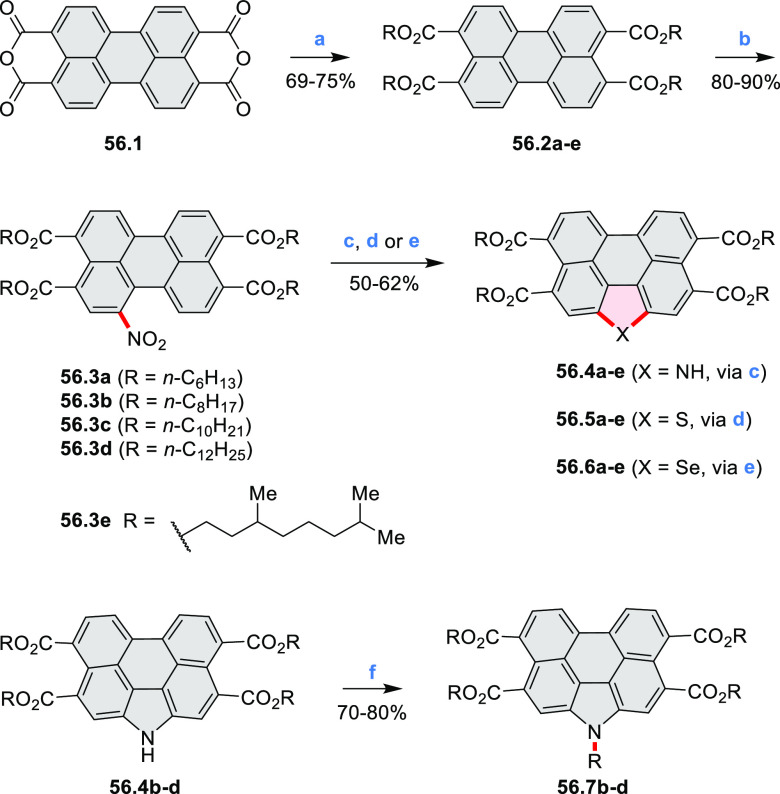
Synthesis of Heteroatom-Annulated Perylene Tetraesters Reagents and conditions: (a)^95^ KOH, H_2_O, 70 °C, 0.5 h, then acidified to pH 8–9 with 1 M HCl, then Aliquat 336, KI, RBr, reflux, 12 h, 69–75%; (b) NaNO_2_, HNO_3_, DCM, 0 °C, 1 h, 80–90%; (c)^93,95,96^ P(OEt)_3_, reflux, 4 h, 55–62%; (d)^94,96^ sulfur powder, NMP, 70 °C, 0.5 h, then 180 °C, 17 h, 50–60%; (e)^95,96^ selenium powder, NMP, 70 °C, 0.5 h, then 180 °C, 17 h, 52–58%; (f) NaH, RBr, THF, reflux, 17 h, 70–80%.

In a series of articles by Achalkumar and co-workers, the commercially available perylene dianhydride **56.1** was derivatized into tetraesters **56.2a**–**e** via hydrolysis followed by S_N_2 esterification (Scheme 56). NaNO_2_-mediated nitration of these intermediates afforded the 6-nitro derivatives **56.3a**–**e** in high yields, in contrast to the low-yielding nitration of unsubstituted perylene. The nitro group was then used as a reactive handle for various annulations (see CR2017, Section 3.2 for earlier related work). The Cadogan reaction with triethyl phosphite provided N-annulated perylenes (NAPs) **56.4a**–**e**, which were further derivatized via *N*-alkylation to afford **56.7b**–**d**.^93^ Treatment of **56.3a**–**e** with elemental S or Se in NMP afforded, respectively, S-annulated **56.5a**–**e**^94^ and Se-annulated **56.5a**–**e**.^95^ Moderate yields of 50–60% were generally observed in all annulation reactions. The products were luminescent, having green emission in THF solutions of **56.4b**–**d** and **56.7b**–**d** (492–495 nm), light blue in **56.5a**–**d** (461–464 and 481–482 nm), and bluish green in **56.6a**–**d** (weak and broad emission peaking at 476 nm). The heteroannulated compounds were generally capable of self-assembly into π-stacked columns, giving rise to columnar hexagonal liquid crystalline phases. This behavior was disrupted in the N*-*alkylated **56.7b**–**d**, where the additional alkyl chain was thought to disfavor self-assembly. In **56.4e**, **56.5e**, and **56.5e**, which were functionalized with branched tetrahydrogeranyl chains, the temperature range of the hexagonal columnar mesophase was especially broad (from −50 to 285 °C for **56.4e**).^96^ These liquid crystalline materials were used as electroluminescent layers in OLED devices either in neat form or as dopants in a polyvinyl carbazole matrix. The latter OLED variant provided a higher emission intensity.

**Scheme 57:**
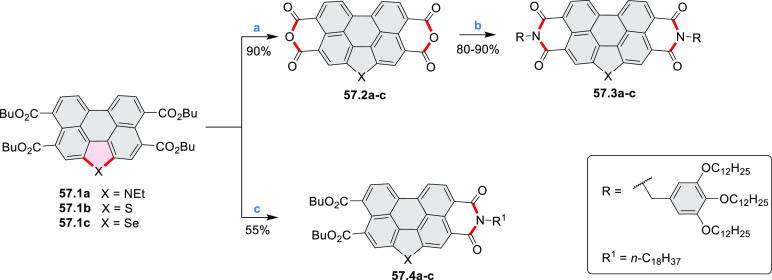
Synthesis of N*-*Annulated Perylene Monoimides and Diimides Reagents and conditions: (a) *p*-toluenesulfonic acid monohydrate, toluene, 100 °C, 30 h, 90%; (b)^98^ tris(*n*-dodecyloxy)benzylamine (2.05 equiv), zinc acetate, imidazole, 165 °C, microwave, 30 min, 80–90%; (c) *n*-octadecylamine (1.1 equiv), imidazole, 165 °C, microwave, 35 min, 55%.

Achalkumar and co-workers also investigated the conversion of heteroannulated perylene tetraesters into mono- and diimides (Scheme 57).^97,98^ Both transformations were effected by microwave heating in imidazole in the presence of 1.1 or 2.05 equiv of the respective amine. Diimides **57.3a**–**c** were prepared via the anhydride intermediates **57.2a**–**c**, while monoimides **57.4a**–**c** were made directly from the tetraesters **57.1a**–**c**. A possibility of elaborating monoimides into unsymmetrically substituted diimides was discussed but only realized experimentally with a nonannulated perylene core. Self-assembly into a hexagonal columnar mesophase as seen in tetraester analogues (Scheme 56) was also observed in the diimides **57.3a** and **57.3b**. In **57.3c** an additional oblique columnar phase was detected. Solution luminescence of the monoimides and diimides was red-shifted in comparison to the tetraesters, with two main emission peaks in the 500–600 nm range.

**Scheme 58:**
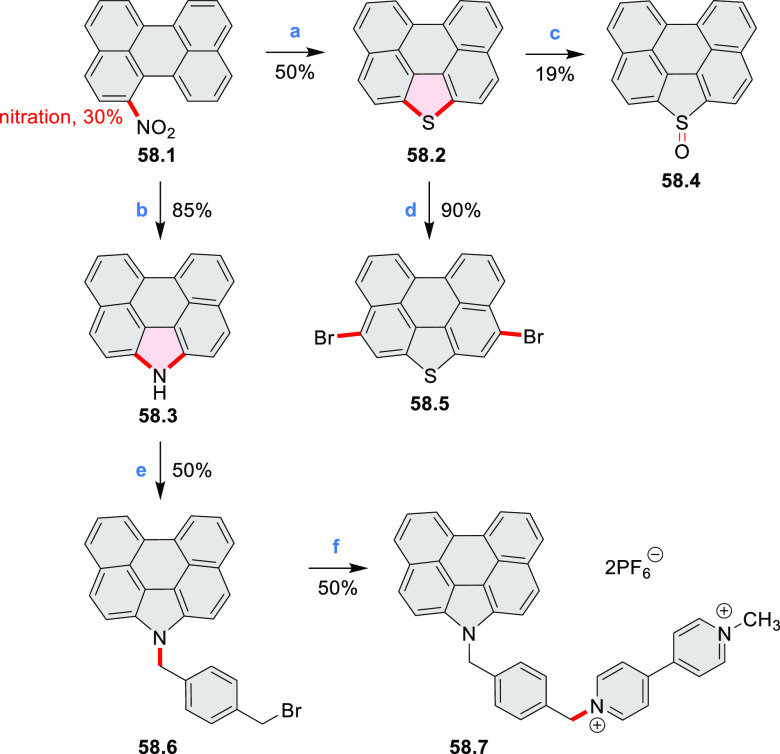
Synthesis of Unsubstituted N*-* and S*-*Annulated Perylenes Reagents and conditions: (a)^99^ sulfur powder, NMP, 70 °C, then **58.1**, 180 °C, 10 h, 50%; (b)^101^ P(OEt)_3_, reflux, 2 h, 85%; (c) *m*-CPBA, DCM, −78 to 5 °C, 19%; (d)^100^ NBS, 1:1 CHCl_3_/AcOH, rt, 5 h; (e)^102^ α,α′-dibromo-*p*-xylene, KOH, KI, THF, reflux, 2 days, 50%; (f) *N-*methyl-4,4′-bipyridinium hexafluorophosphate, MeCN, reflux, 12 h, 50%.

Simple examples of introducing [*ghi*]heteroannulation into unsubstituted perylenes include bay nitration followed by Cadogan reaction to provide N*-*annulated **58.3** or treatment with elemental sulfur, leading to S*-*annulated **58.2** (Scheme 58). In particular, the Cadogan reaction is significantly more efficient with the otherwise unsubstituted 1-nitroperylene **58.1** than with perylene tetraesters or perylene diimides (PDIs). The resulting compounds can be derivatized by 3,10-dihalogenation as shown in the example of **58.5**. The S*-*annulated **58.2** could also be oxidized to sulfoxide upon treatment with *m*-CPBA.^99^ The sulfoxide **58.4** undergoes deoxygenation upon irradiation with UV or violet light (420 nm). While photodeoxygenation of related sulfoxides is known to generate atomic oxygen, the mechanism of this process in the case of **58.5** is currently unclear. The dibrominated **58.5** was observed to self-assemble on the Ag(111) surface, forming three distinct networks, all of which contained pores flanked by six molecules of **58.5**.^100^ This ordering was driven by σ-hole interactions, with Br···Br halogen bonding contacts as well as a weaker S···Br chalcogen bonding.

The simple N*-*annulated perylene **58.3** formed a complex with diiodine, which was crystallized out of a benzene solution.^101^ The crystal structure revealed polymeric chains of I atoms adjacent to columnar stacks of **58.3**. These chains were nearly linear with distances of 3.054–3.174 Å between adjacent I atoms. These values are significantly larger than the I–I covalent bond length (2.70 Å) and were thought to imply that the I atoms have a partial negative charge. A similar polyiodide is believed to be present in the iodine–starch complex, as implied by the similarities in low-wavenumber Raman signals. Alkylation on the pyrrolic N atom of **58.3** led to a conjugate with a positively charged viologen unit.^102^ This compound was nonemissive, while blue emission with a 435 nm maximum was present in its *N-*benzyl counterpart. This effect was attributed to photoinduced electron transfer from the N*-*annulated perylene to the viologen segment. This charge-separated state recombined in approximately 20 ps.

**Scheme 59:**
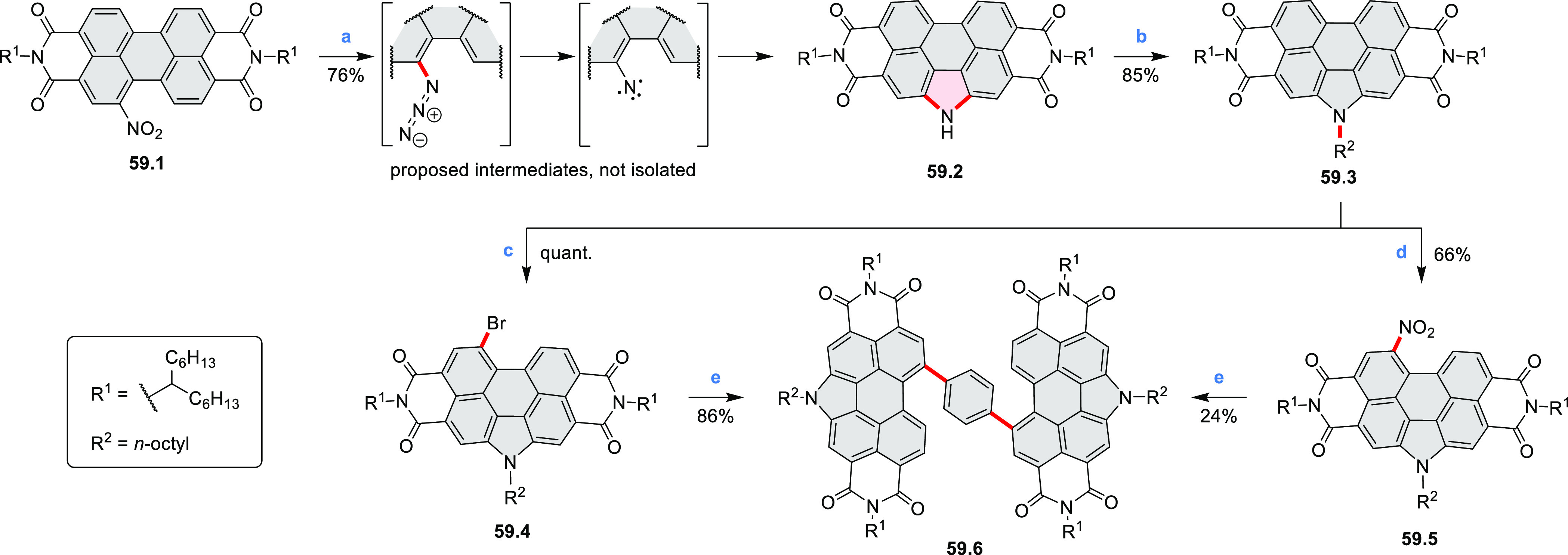
Preparation of a NAP Diimide Dimer via Suzuki–Miyaura Coupling Using Nitro and Bromo Derivatives Reagents and conditions: (a)^103^ NaN_3_, 1:1 DMF/THF, rt, 18 h, 76%; (b) 1-bromooctane, K_2_CO_3_, KI, DMF, 90 °C, 18 h, 85%; (c) Br_2_, DCM, rt, 30 min, quant.; (d) HNO_3_ (fuming), DCM, rt, 5 min, 66%; (e) 1,4-benzenediboronic acid bis(pinacol) ester, Pd(PPh_3_)_4_, K_2_CO_3_, 9:1 dioxane/H_2_O, 110 °C, microwave heating, 2 h.

N-Annulation of bay-nitrated PDIs can be accomplished by a simple treatment with NaN_3_ at rt (Scheme 59),^103^ which presents a valuable alternative to the Cadogan reaction using triethyl phosphite or PPh_3_ under much harsher conditions. Indeed, **59.2** was obtained in 76% yield, which is an improvement over 50–70% efficiencies generally seen using the Cadogan approach with PDIs or perylene tetraesters. The authors propose a displacement of the nitro group followed by spontaneous decomposition of the resulting azido derivative to nitrene as the reaction mechanism. As no bay-azido-substituted PDIs are known, this mechanistic hypothesis is likely to be correct. Another unusual transformation demonstrated in this work is the use of the bay-NO_2_ substituent as a reactive handle for Suzuki cross-coupling. While this reaction was inefficient with **59.5**, providing the bis-PDI **59.6** in only 24% yield, much better results were obtained with nonannulated PDIs. This difference is attributed to the reactivity of the NO_2_ group being decreased by the additional electron density from the pyrrolic ring.

**Scheme 60:**
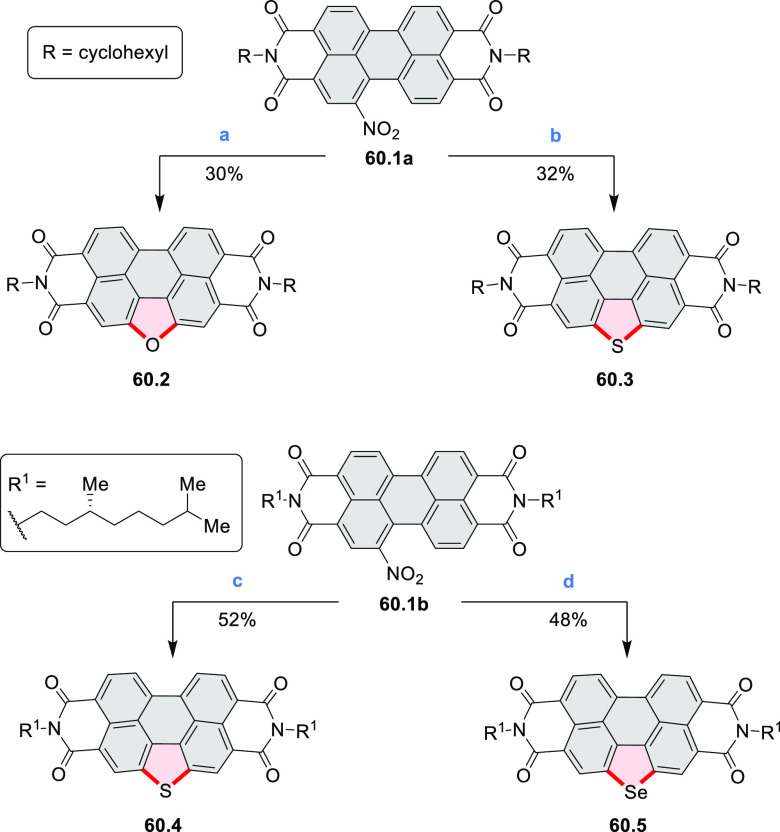
O-, S-, and Se-Annulation of Perylene Diimides Reagents and conditions: (a)^104,105^ NMP, O_2_, 180 °C, 5 h, 30%; (b)^106^ sulfur powder, NMP, 130 °C, 12 h, 32%; (c)^107^ sulfur powder, NMP, 70 °C then **60.1b**, 190 °C, 3 h, 52%; (d) selenium powder, NMP, 70 °C then **60.1b**, 190 °C, 3 h, 48%.

As an alternative to the synthetic sequence shown in Scheme 57, the heteroatom annulation can be done after diimide formation (Scheme 60). Bay-nitrated PDI **60.1a** was converted into the O-annulated derivative **60.2** in 30% yield by heating in NMP under an oxygen atmosphere.^104,105^ Additionally, the S*-*annulated analogue **60.3** was prepared using a similar methodology.^104,106^ Spectroscopic and electrochemical data of this compound were reported and compared with several nonannulated analogues.^106^ The O*-*annulated compound **60.2** displayed slightly red-shifted absorption and emission (λ_abs_ = 510 nm, λ_em_ = 522 nm) in comparison to **60.3** (λ_abs_ = 500 nm, λ_em_ = 515 nm). Self-assembly into a needle-like morphology was reported for **60.2**, while **60.3** formed nanosheets.^104^

S- and Se-annulation was performed on the diimide **60.1b** functionalized with optically pure (*R*)-tetrahydrogeranyl chains in similar yields as shown above for perylene tetraesters.^107^ Aggregation of these compounds occurred in 2:3 CHCl_3_/MeOH, and the resulting CD features had opposite signs in **60.5** and **60.4**. This chiroptical difference translated into macroscopic features of dried samples, where **60.4** formed 200 nm wide ribbons with a left-handed twist, while **60.5** produced 100 nm wide right-handed ribbons. Conductivity of these fibers was sensitive to the presence of NH_3_ gas via formation of donor–acceptor complexes, resulting in up to 340-fold current response for **60.4** in the presence of 100 ppm of NH_3_.

**Scheme 61:**
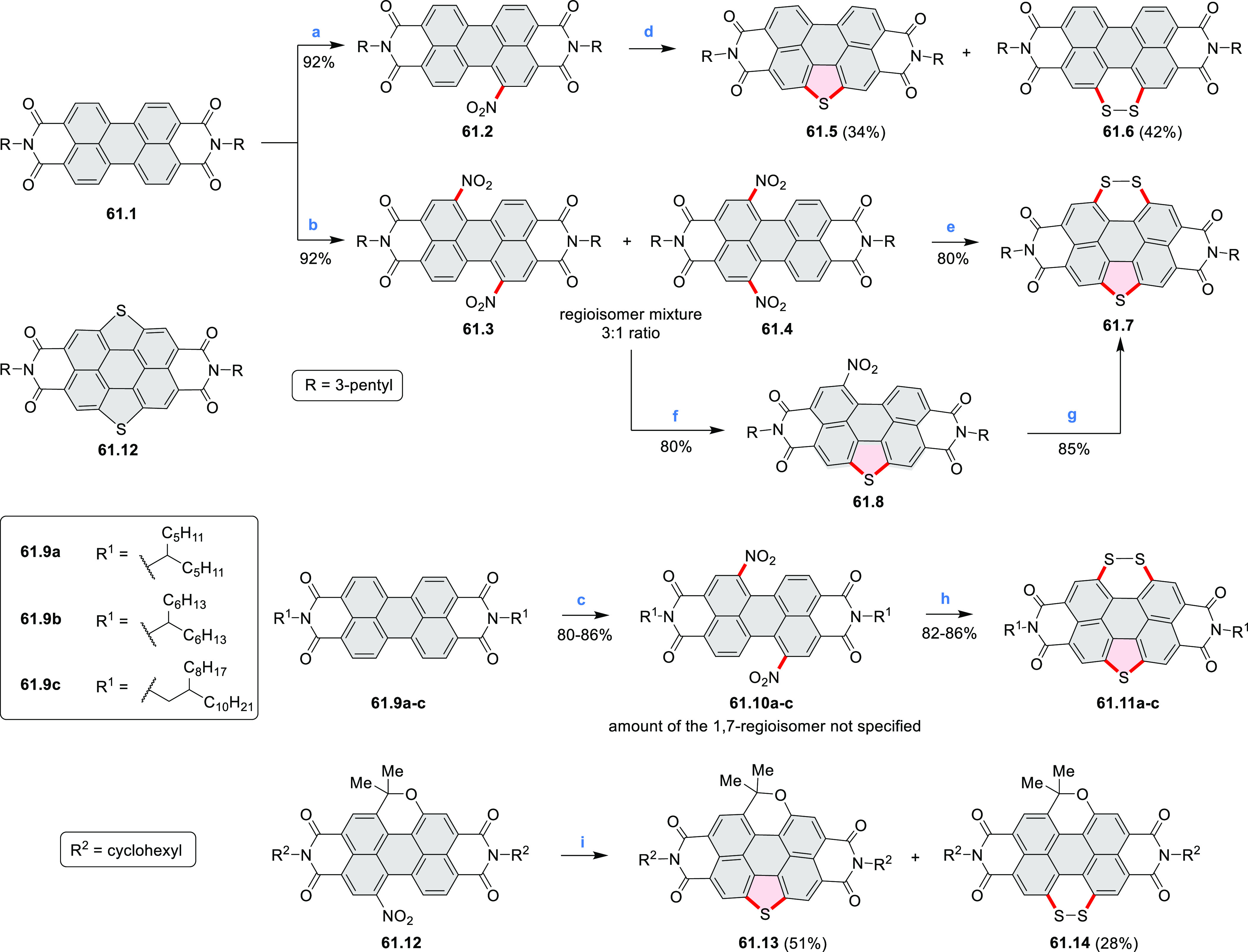
Synthesis of S*-* and S_2_-Annulated Perylene Diimides Reagents and conditions: (a)^108^ HNO_3_, CAN, DCM, 25 °C, 2 h, 92%; (b)^108^ HNO_3_, CAN, DCM, 25 °C, 48 h, 92%; (c)^109^ HNO_3_ (fuming), DCM, 0 °C, 20 min then rt, 12 h, 80–86%; (d)^108^ sulfur powder, DMF, 120 °C, 8 h; (e)^108^ sulfur powder, DMF, 120 °C, 6 h, 80%; (f)^108^ sulfur powder, DMF, 80 °C, 2 h, 80%; (g)^108^ sulfur powder, DMF, 120 °C, 3 h, 85%; (h)^109^ sulfur powder, NMP, 190 °C, 4 h, 82–86%; (i)^110^ sublimed sulfur, NMP, 110 °C, 10 h.

Perylene diimides such as **61.1** and **61.9a**–**c** can be nitrated at the *bay* positions to mono- or dinitro derivatives (Scheme 61). In the latter case, a mixture of 1,6- and 1,7-regioisomers is obtained, as demonstrated by **61.3** and **61.4**, which formed in a 3:1 ratio.^108^ S-Annulation of the dinitro derivatives resulted in the formation of **61.7** and **61.11a**–**c**,^109^ each with a sulfide and a disulfide bridge across the two perylene bays. A disulfide-bridged compound could also be formed from the mononitro derivative **61.2** by performing the S*-*annulation under relatively gentle conditions (120 °C rather than 180–190 °C), resulting in a separable mixture of **61.5** and S_2_-annulated **61.6**. An even lower reaction temperature allowed us to obtain the singly annulated nitro derivative **61.8**. Additionally, **61.12**, annulated with two five-membered rings, was prepared according to a previously reported method.

Compared to **61.12**, **61.7** and **61.11c** have an additional broad absorption band at 500–700 nm, which was tentatively assigned to a charge-transfer transition involving the electron-rich disulfide. Similar spectroscopic differences were seen between compounds **61.13** and **61.14**, where the absorption maximum of **61.14** was red-shifted to 654 nm, relative to 548 nm in **61.13**.^110^ Compound **61.14** was additionally nonemissive, while **61.13** displayed an orange fluorescence. A lack of luminescence was also reported in the case of the S/S_2_-annulated **61.7**.

**Scheme 62:**
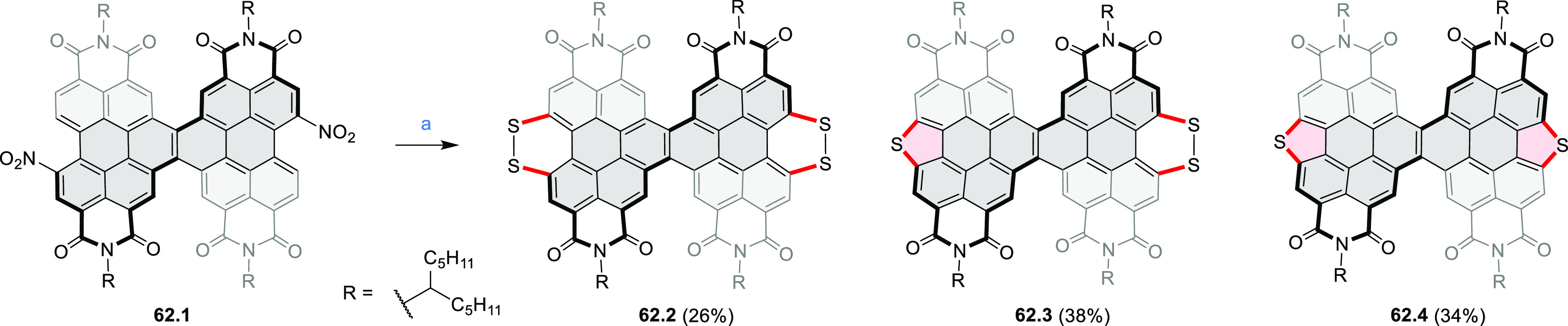
Synthesis of Sulfur-Annulated Fused Perylene Diimides Reagents and conditions: (a)^111^ sulfur powder, NMP, 70 °C then **62.1**, 200 °C, 20 min.

A dinitro derivative of a fused perylene diimide dimer, **62.1**, was efficiently converted into a mixture of S- and S_2_-annulated products under similar conditions as shown above (Scheme 62).^111^ At longer reaction times, product mixtures enriched in **62.4** were obtained. As in the compounds with a single perylene core, introduction of S_2_ bridges caused a decrease of the optical band gap, giving rise to new absorption features in the 600–700 nm range. DFT calculations showed that the highest HOMO amplitude is present on the S_2_ bridges in **62.2** and **62.3**, in contrast to a very low HOMO amplitude on the S bridges in **62.4**. This observation implies that the aromaticity of S*-*annulated rings is absent in S_2_-annulated analogues, where the sulfur atoms simply act as electron-rich substituents.

**Scheme 63:**
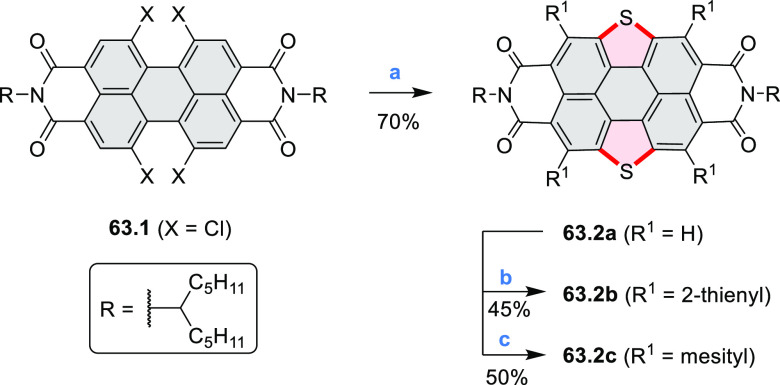
Synthesis of Dithiophene-Annulated Tetraarylperylenes Reagents and conditions: (a)^112^ Pd(PPh_3_)_4_, bis(tributyltin)sulfide, toluene, reflux, 10 h; (b) 2-thienylmagnesium bromide, THF, 0 °C to rt, 12 h; (c) mesitylmagnesium bromide, THF, 0 °C to rt, 12 h.

1,4-Addition of Grignard reagents was demonstrated by Wudl and Zheng et al. as an effective catalyst-free method to directly functionalize the 2,5,8,11-positions of bay-annulated perylene diimides with aryl substituents (Scheme 63).^112^ The S*-*heterocyclic annulated **63.2a** was prepared in 70% yield via a Stille-type coupling reaction between **63.1** and bis(tributyltin)sulfide. The reaction of **63.2a** with the corresponding Grignard reagent, 2-thienylmagnesium or 2-mesitylmagnesium bromide, afforded tetrasubstituted S*-*containing perylene diimides, **63.2b** and **63.2c**, respectively, in reasonable yields. Preliminary studies on OSCs utilizing these molecules as nonfullerene acceptors showed PCE values up to 5.07% for the thienyl-substituted **63.2b**.

**Scheme 64:**
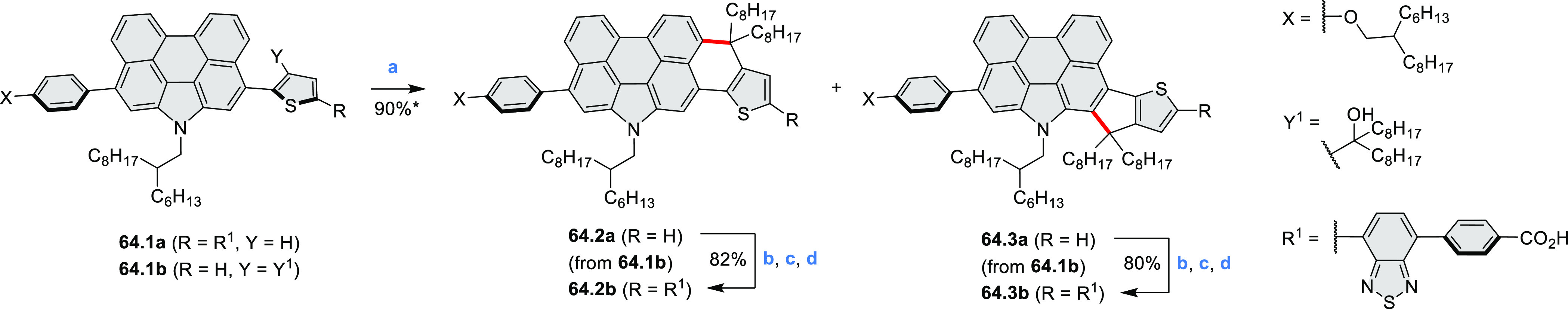
Synthesis of N*-*Annulated Thienoperylene Dyes Reagents and conditions: (a)^113^ Amberlyst 15, toluene, reflux, 12 h; (b) *t*-BuLi, THF, −78 °C, 1 h, then chlorotrimethylstannane, −78 °C to rt, 24 h; (c) butyl 4-(7-bromobenzo[*c*][1,2,5]thiadiazol-4-yl)benzoate, Pd(PPh_3_)_2_Cl_2_, toluene, reflux, 12 h; (d) KOH, 3:1 THF/H_2_O, reflux, 8 h, then HCl. *64.2a and **64.3a** were obtained in 1:2 ratio.

In 2015, Wang and co-workers designed and synthesized two D–A organic dyes consisting of electron-rich N*-*annulated 6*H*-thieno[3′,2’:5,4]-benzo[*cd*]perylene or its isomer 13*H*-thieno[2′,3′:3,4]cyclopenta[*b*]perylene, linked to an electron acceptor, 4-(benzo[*c*]-[1,2,5]thiadiazol-7-yl)benzoic acid (Scheme 64).^113^ Thiophene-substituted **64.1b** underwent intramolecular Friedel–Crafts cyclization in the presence of Amberlyst 15 acting as a solid acid catalyst to concurrently afford **64.2a** and **64.3a** in 30% and 60% yield, respectively. The electron-rich compounds **64.2a** and **64.3a** were treated with *tert*-butyllithium and chlorotrimethylstannane to generate the corresponding stannanes, which were cross coupled with butyl 4-(7-bromobenzo[*c*]-[1,2,5]thiadiazol-4-yl)benzoate to yield **64.2b** and **64.3b** after the KOH-mediated hydrolysis. The final compounds were tested as dyes for DSSCs. The best results were obtained with **64.2b**, with a PCE of up to 12% when irradiated with 100 mW cm^–2^, AM 1.5G simulated sunlight. This was the first example of a metal-free organic dye reaching such a high PCE without any coadsorbate.

**Scheme 65:**
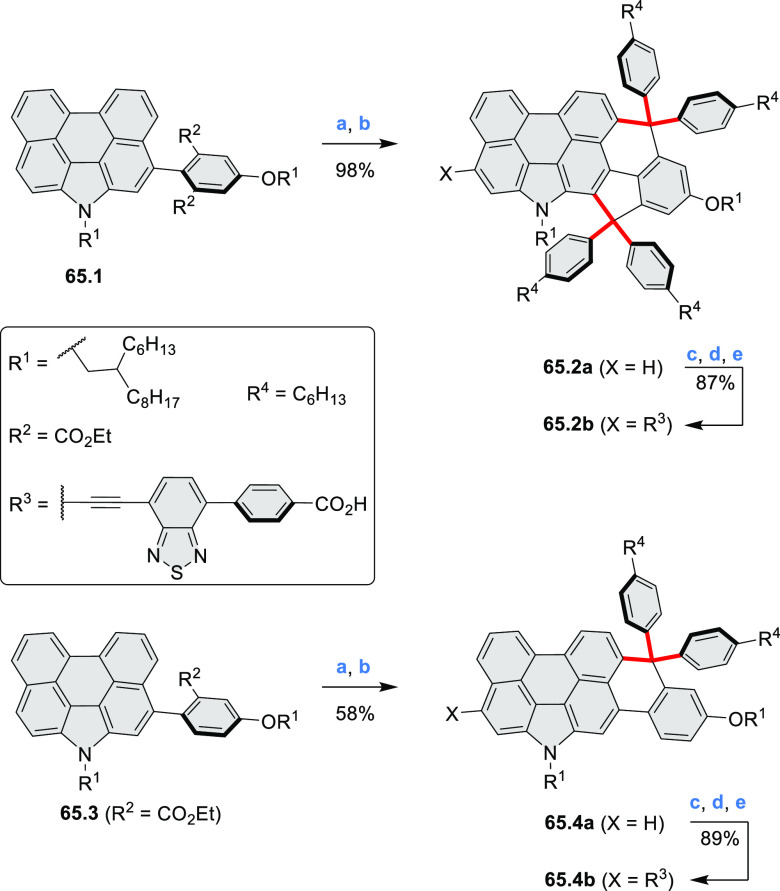
Synthesis of N*-*Annulated Perylene Dyes Reagents and conditions: (a)^114^ 4-hexylphenylmagnesium bromide, THF, reflux, overnight; (b) Amberlyst 15, toluene, reflux, overnight; (c) NBS, THF, 0 °C, 30 min; (d) butyl 4-(7-ethynylbenzo[*c*][1,2,5]thiadiazol-4-yl)benzoate, Pd_2_(dba)_3_, P(*t*-Bu)_3_, Cs_2_CO_3_, dioxane, reflux, 5 h; (e) (1) KOH, 3:1 THF/H_2_O, reflux, 8 h, (2) phosphoric acid.

In 2017, Wang et al. developed another D–A dye based on a NAP decorated with 4-(benzo[*c*]-[1,2,5]thiadiazol-7-yl)benzoic acid.^114^ As presented in Scheme 65, N*-*annulated perylene **65.1** was subjected to the addition reaction with (4-hexylphenyl)magnesium bromide, generating a tertiary alcohol, which was cyclized into **65.2a** in 98% yield in the presence of Amberlyst 15 sulfonic acid resin. **65.2a** was brominated and derivatized in a Sonogashira coupling with butyl 4-(7-ethnylbenzo[*c*]thiadiazol-4-yl)benzoate. Finally, this precursor underwent hydrolysis in the presence of potassium hydroxide followed by acidification with H_3_PO_4_ to afford the target product **65.2b**. Compound **65.4b**, acting as a control dye, was synthesized by an analogous approach. DSSCs containing **65.2b** adsorbed on TiO_2_ particles reached a PCE of up to 12.6% with no coadsorbent. A slightly lower efficiency (11.7%) was achieved with **65.4b**, which was attributed to the inhibition of charge carrier recombination by the additional 4-hexylphenyl groups in **65.2b**.

**Chart 5:**
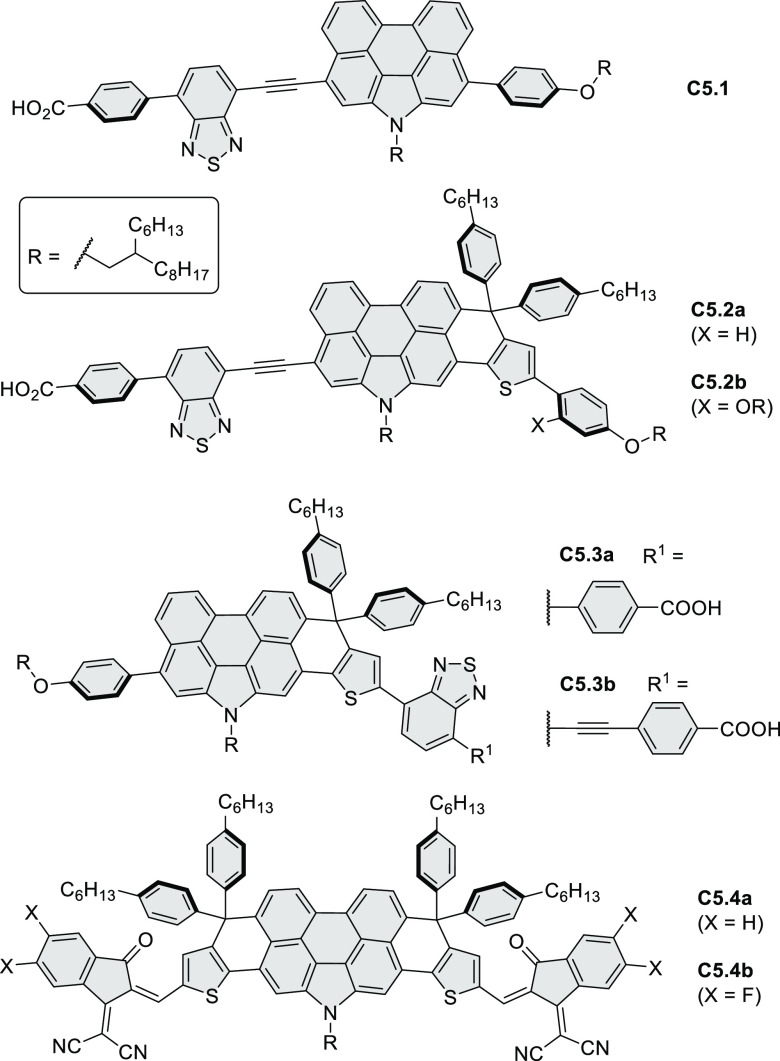
N*-*Annulated Perylene Dyes

In their further research on donor–acceptor organic dyes, Wang and co-workers reported two more molecules with a NAP-based unit as the central module of the electron donor and ethynylbenzothiadiazole-benzoic acid as the electron acceptor, **C5.2a** and **C5.2b**,^115^ along with the previously reported model dye **C5.1** (Chart 5). The thiophene-fused compounds **C5.2a** and **C5.2b** were more electron rich, corresponding to a higher HOMO energy and decreased HOMO–LUMO gap compared to **C5.1**. An additional alkoxy substituent in **C5.2b** resulted in a further decrease in the energy gap attributed to conformational rigidification by an O···S chalcogen bonding interaction. DSSC devices containing these dyes in combination with TiO_2_ particles displayed high PCE values of 11.5% with **C5.2a** and 12.4% with **C5.2b** under AM 1.5G irradiation. The same group also described compounds **C5.3a** and **C5.3b** where the benzothiadiazole acceptor unit was attached to the fused thiophene.^116^ The additional ethynyl linker in **C5.3b** resulted in a decreased HOMO–LUMO gap and red-shifted absorption and NIR emission peaks. These dyes were incorporated into transparent DSSCs that partially transmitted visible light in the 600–750 nm range. The devices achieved PCE values of 10.1% with **C5.3a** and 10.3% with **C5.3b**, which were considered high for transparent cells.

The synthetic approach shown in Scheme 65 was further extended to prepare symmetrically derivatized NAPs **C5.4a** and **C5.4b**.^117^ In this case, very narrow HOMO–LUMO gaps of 1.74 eV for **C5.4a** and 1.70 eV for **C5.4b** were achieved, which allowed the absorption bands to extend into the NIR region. Bulk heterojunction organic photovoltaic cells were prepared using these compounds and the PTB7-Th polymer as the donor material. Remarkably, the cells were able to capture NIR radiation with wavelengths of up to 1000 nm. Compound **C5.4b** afforded much higher electron mobility and external quantum efficiency values, allowing us to achieve a PCE of 10.21% under a simulated solar irradiation of 100 mW/cm^2^.

**Scheme 66:**
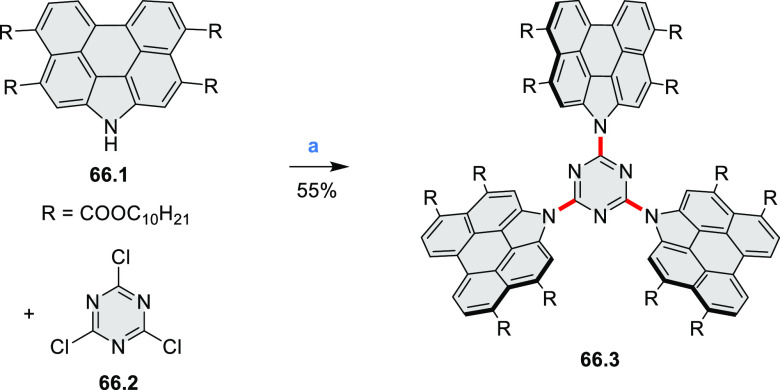
Synthesis of a NAP–Triazine Hybrid Reagents and conditions: (a)^118^*n*-BuLi, THF, reflux, 5 h.

A star-shaped molecule with a central electron-deficient triazine ring connected to three electron-rich NAP subunits was designed as a prospective emissive layer in OLEDs.^118^ To obtain the target **66.3**, the NAP tetraester **66.1** was treated with cyanuric chloride **66.2** in the presence of *n*-BuLi as a base (Scheme 66). The star-shaped **66.3** exhibits bright green fluorescence with a high quantum yield. When applied in green OLED devices, **66.3** displayed an external quantum efficiency of 1.76%, which was improved to 2.57% by embedding it in a polyvinylcarbazole matrix.

**Scheme 67:**
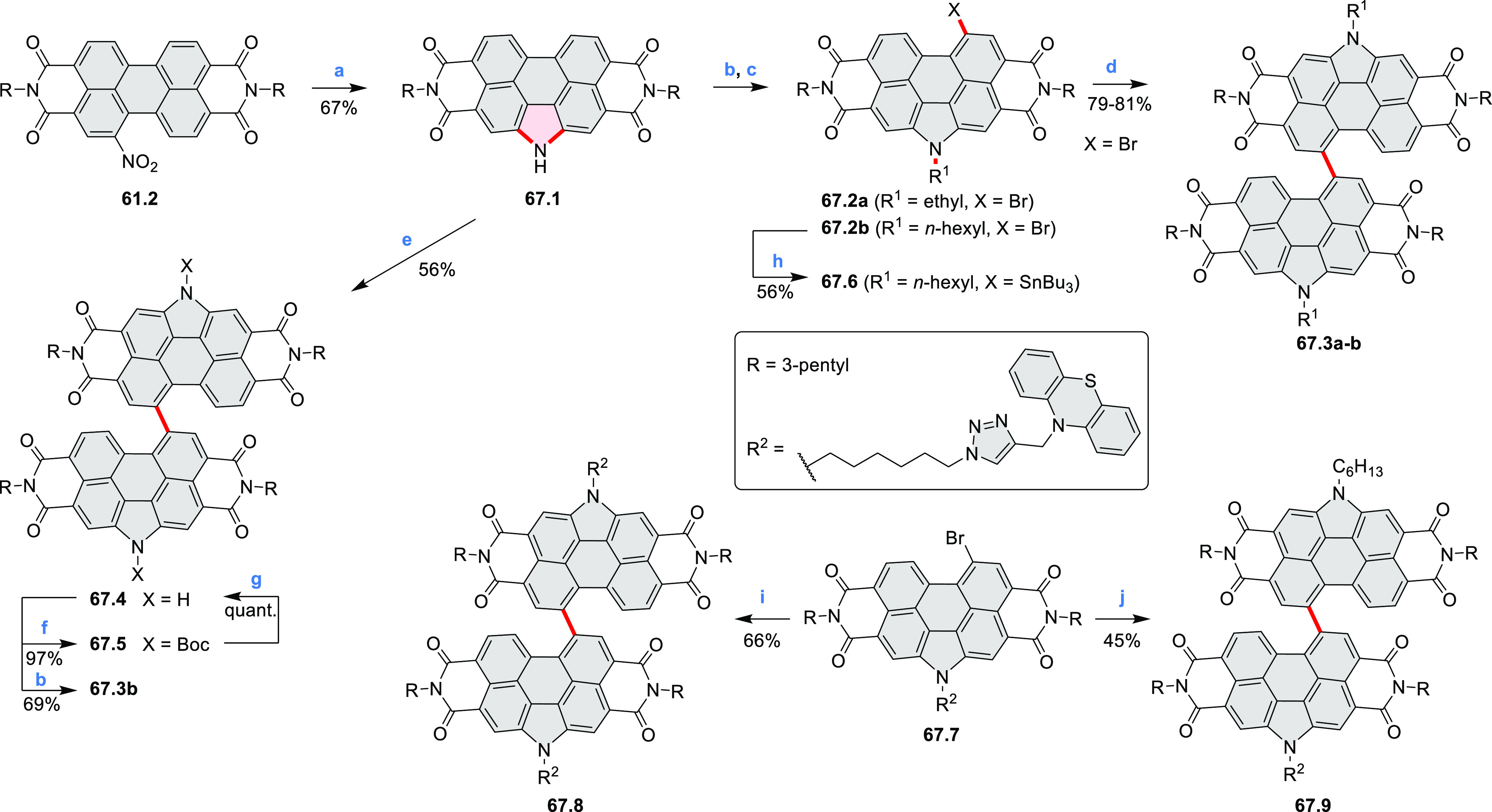
Synthesis of N*-*Annulated Perylene Diimide Dimers Reagents and conditions: (a)^119^ PPh_3_, DMF, 150 °C, 21 h, 67%; (b) *n*-hexyl bromide, K_2_CO_3_, DMF, 120 °C, 18 h, 89–96% for **67.2a**–**b**; (c) Br_2_, DCM, rt, 2 h, 94–98%; (d) Pd(dba)_2_, Zn dust, DMF, 100 °C, 3.5 h, 79–81%; (e)^120^ Br_2_ (neat), rt, 1 h, 93%, then Pd(dba)_2_, Zn dust, DMF, 120 °C, 30 min, 56%; (f) Boc_2_O, DMAP, K_2_CO_3_, DMF, 80 °C, 24 h, 97%; (g) 200 °C, 1 h, quant. or 180 °C in thin films; (h)^121^ hexabutylditin, SiliaCat DPP-Pd, toluene, 100 °C, 1 h, 63%; (i) Zn dust, Pd_2_(dba)_3_, DMF, 100 °C, 2.5 h, 66%; (j) **67.6**, Pd(PPh_3_)_4_, toluene, 180 °C, microwave, 10 min, 45%.

N*-*Annulated perylene diimide dimers such as **67.3a**,**b** were obtained by Negishi-type homocoupling of aryl bromide precursors (Scheme 67). An alternative Cadogan annulation procedure using PPh_3_ in DMF rather than neat P(OEt)_3_ was used in the synthetic sequence.^119^ Dimerization could also be performed using **67.1** to provide **67.4** with free pyrrolic NH groups.^120^ While the low solubility of **67.4** in organic solvents impaired its processability, it could be Boc protected to **67.5**, which was highly soluble, similarly to the *N*-alkylated analogues. This compound was then deprotected upon thermal annealing of a thin film. The unprotected **67.4** interacted with bases; with DBU it was deprotonated to a dianion with a strongly red-shifted absorption. Apart from homodimers, a perylene dimer **67.9** with unsymmetric N-substitution was prepared via a Stille coupling of the stannylated **67.6** with **67.7**.^121^ These donor–acceptor conjugates were characterized by quenching of the PDI emission (**67.3b**: 588 nm in solution, 633 nm in film),^122^ attributed to charge transfer.

Compared with their monomeric analogues, the perylene diimide dimers showed decreased aggregation in thin films. This led to an improved film morphology which corresponded to higher PCE values of bulk heterojunction OSC devices that used these materials as electron acceptors.^122^ PCE values in the range of 5–7% were reported for compounds **67.3a**,**b** in blends with PTB7-Th and TTFQx-T1 donor polymers.^119,120^ Additionally, N*-*annulation decreased the electron affinity of the perylene core, leading to higher open-circuit voltages in OSCs.^122^

**Chart 6:**
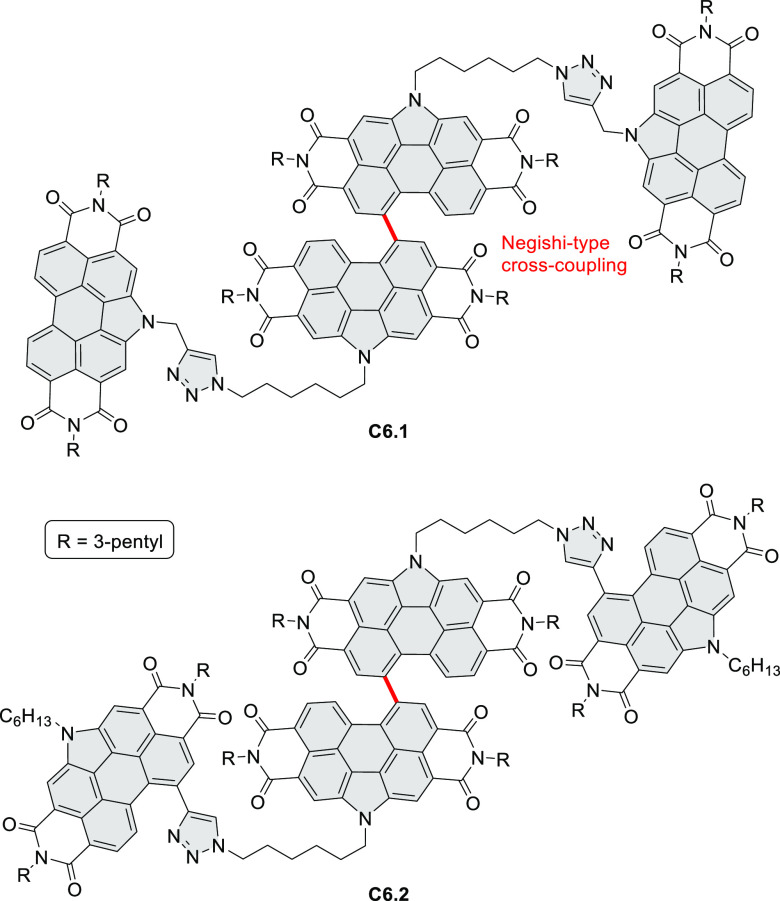
N*-*Annulated PDI Tetramers with Dimeric Cores

A similar Negishi-type homocoupling of aryl bromides was used in the final step toward the tetrameric N*-*annulated PDIs **C6.1**–**2** (Chart 6).^123^ The reaction was performed using Pd_2_(dba)_3_ and Zn dust at 100 °C in DMA, providing **C6.1** and **C6.2** in 81% and 32% yield, respectively. The triazole linkages were formed via CuAAC prior to the final homocoupling step. While the spectral features of **C6.2** were essentially a sum of its building blocks (monomeric and dimeric N*-*annulated PDI), compound **C6.1** displayed signs of folding. OPV devices including **C6.1**–**2** as coacceptors alongside PC_61_BM achieved a PCE of 7.0–7.2% under simulated sunlight and up to 13.7% under LED light irradiation, demonstrating their utility in powering small devices via indoor light recycling.

**Scheme 68:**
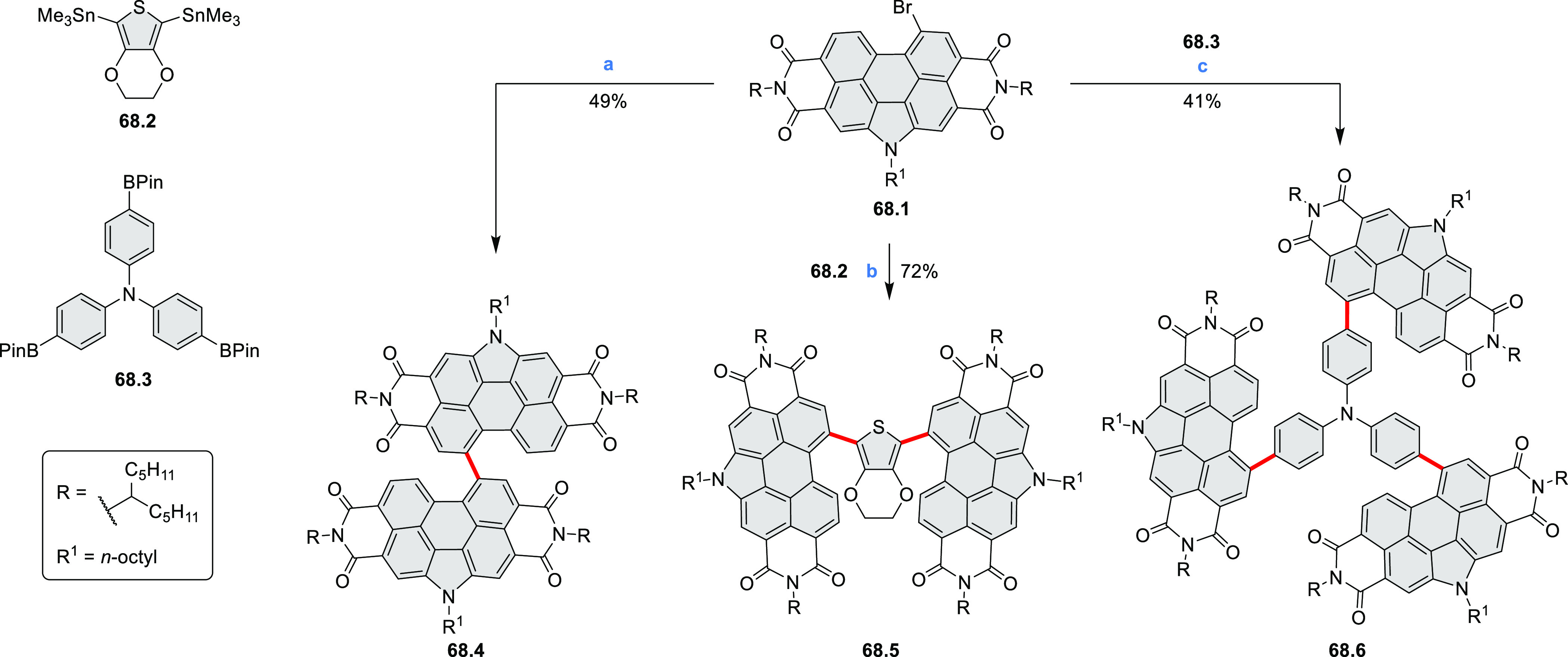
Preparation of N*-*Annulated PDI Conjugates via Cross-Coupling Reactions Reagents and conditions: (a)^124^ B_2_pin_2_, Pd(dppf)Cl_2_, NaOAc, 10:3/1 DMF/toluene/H_2_O, 70 °C, overnight, 49%; (b) Pd(PPh_3_)_4_, toluene, 100 °C, 24 h, 72%; (c) Pd(PPh_3_)_4_, K_2_CO_3_, 2:1 toluene/H_2_O, reflux, 2 days, 41%.

N-Annulated PDI dimer **68.4** was evaluated for OSC applications alongside compounds **68.5** and **68.6** containing electron-rich core subunits (Scheme 68).^124^ The N-annulation was carried out similarly as shown in Scheme 67, and the final compounds were prepared via Suzuki or Stille cross-coupling reactions. In particular, the homocoupling of **68.1** to provide **68.4** in 49% yield is notable as an alternative to the Negishi-type approach shown above. OSC devices containing these compounds in blends with a PTB7-Th donor polymer had PCEs of up to 5.29% for **68.4**, which was in agreement with the results observed with the structurally similar **67.3a**,**b**. Compounds **68.5** and **68.6** displayed lower PCE values but higher open-circuit voltage (up to 1.14 V for **68.5**) due to the higher LUMO energy.

**Scheme 69:**
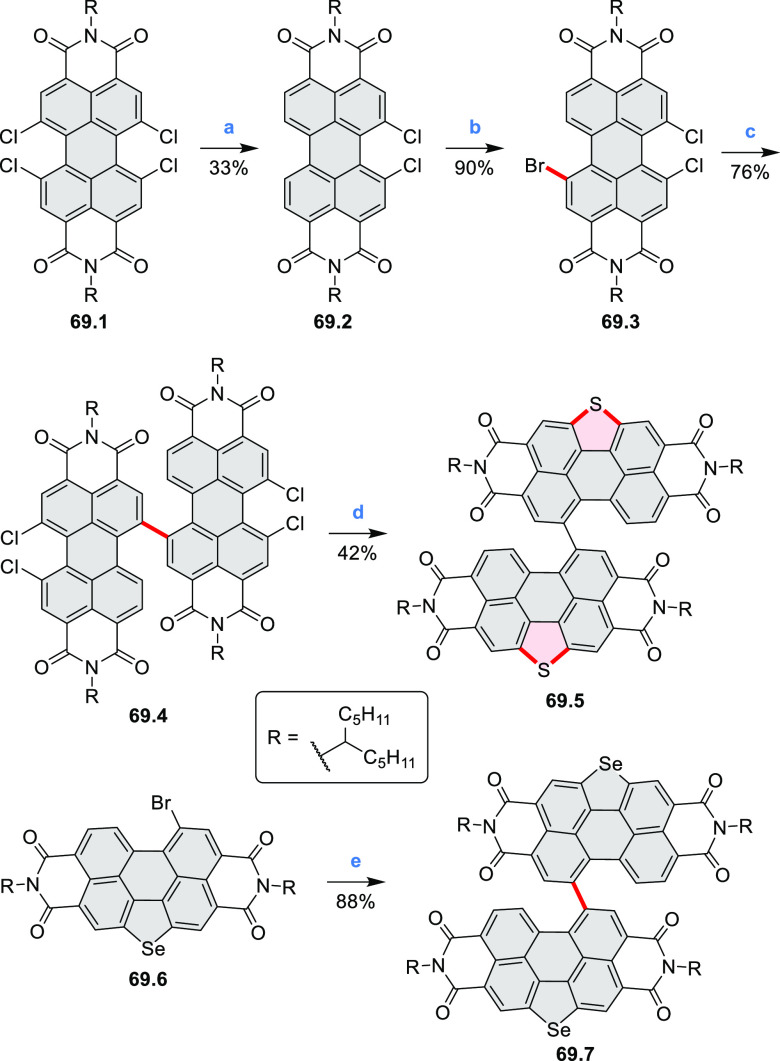
Synthesis of S- and Se-Annulated Perylene Diimide Dimers Reagents and conditions: (a)^125^ CuI, l-proline, 110 °C, 24 h, 33%; (b) Br_2_, H_2_SO_4_, rt, 2 days, 90%; (c) Cu, DMSO, 65 °C, 12 h, 76%; (d) (Bu_3_Sn)_2_S, Pd(PPh_3_)_4_, toluene, reflux, 12 h, 42%; (e)^126^ Zn dust, Pd_2_(dba)_3_, DMF, 55 °C, 1 h, 88%.

Synthesis of an S*-*annulated perylene diimide dimer was accomplished via an unusual synthetic route with no recourse to bay nitration. Partial dehalogenation of the tetrachloro-PDI **69.1** followed by bromination provided **69.3** (Scheme 69).^125^ This compound underwent Ullmann-type homocoupling under surprisingly mild conditions to the dimeric **69.4**. Finally, the S*-*annulation was performed via a Stille-type cross-coupling with bis(tributyltin)sulfide, giving the target **69.5**. A Se-annulated analogue **69.7**(126) was also prepared using the same annulation approach as shown above in Scheme 56, with a final reductive dimerization step similar to that in Scheme 67. Compounds **69.5** and **69.7** displayed similar spectroscopic properties with visible absorption in the 400–600 nm range in thin films. Bulk heterojunction OSCs formulated with **69.5** and **69.7** in tandem with the PDBT-T1 donor polymer and 1,8-diiodooctane additive achieved a PCE of 7.2% and 8.4%, respectively. With **69.7**, the OSCs possessed remarkably high fill factors approaching 70%.

**Scheme 70:**
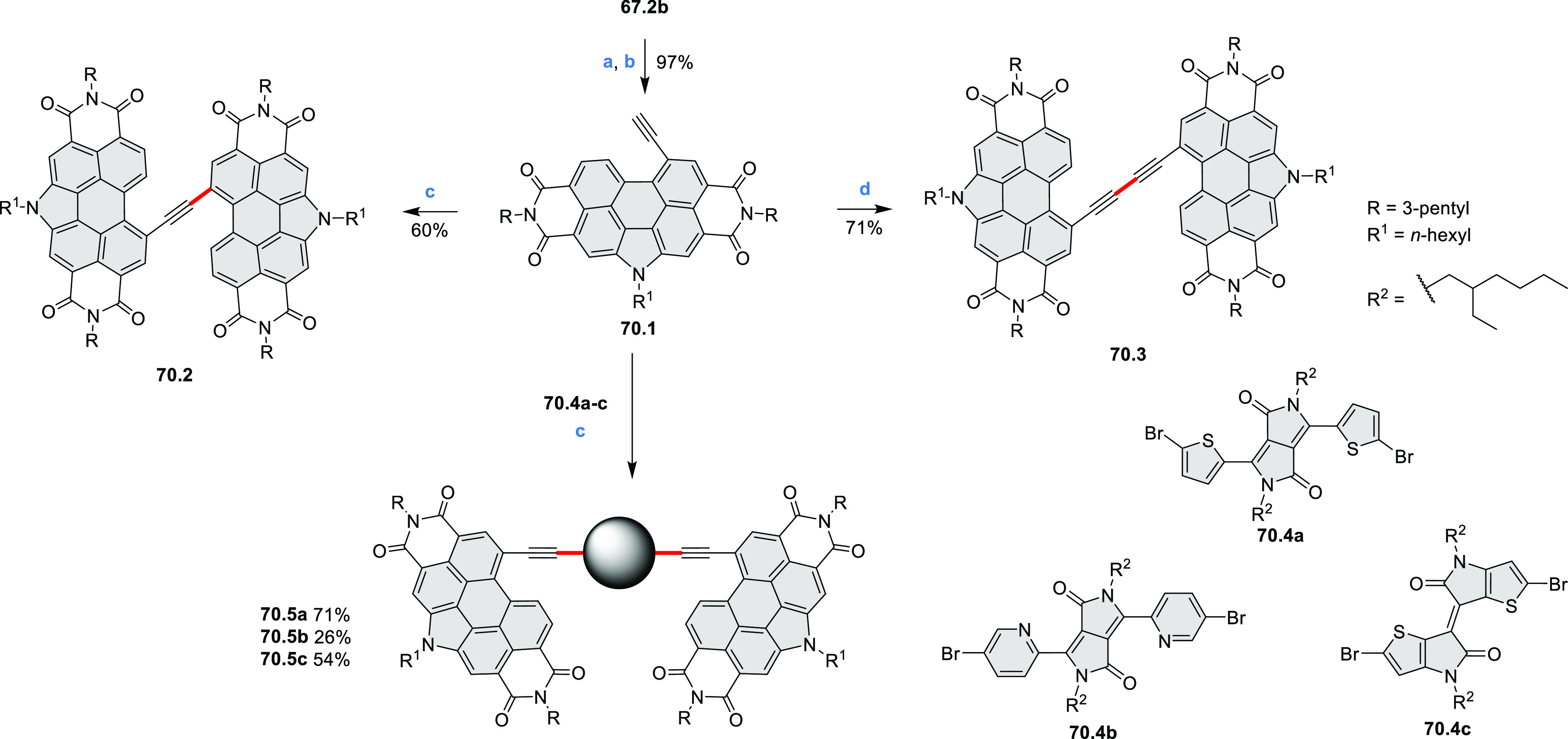
Synthesis of N*-*Annulated Perylene Diimide Dimers with Acetylene Linkers Reagents and conditions: (a)^127^ TMS-acetylene, Pd(PPh_3_)_4_, CuI, (*i*-Pr)_2_NH, 40 °C, 24 h, then SiliaMetS DMT, DCM, rt, 4 h, 98%; (b) K_2_CO_3_, 1:1 CHCl_3_/MeOH, rt, 10 min, 99%; (c) Pd(PPh_3_)_4_, CuI, 1:10 (*i*-Pr)_2_NH/toluene, 60 °C, 0.3–5 h; (d) Pd(PPh_3_)_4_, CuI, K_2_CO_3_, 1:1 CHCl_3_/MeOH, rt, air, 3 h, 71%.

Acetylene linkers can be introduced into N*-*annulated PDI dimers and PDI–dye conjugates using Sonogashira coupling (Scheme 70).^127,128^ In the preparation of **70.1** via cross-coupling with TMS–acetylene, thorough removal of Pd residues with a dimercaptotriazine (DMT) resin was important to achieve high yields. **70.1** was subsequently used as a building block in the synthesis of the homodimer **70.3** via oxidative alkyne homocoupling and in Sonogashira syntheses of **70.2**(127) and **70.5a**–**c**.^128^ Compared to **67.3b**, the acetylene-spaced dimers **70.2** and **70.3** have a decreased steric strain, which allows them to adopt a conformation with coplanar PDI units. This greatly strengthens their aggregation and reduces solubility. Such a behavior is detrimental to the morphology of their blends with donor polymers for OSC applications; as a result, compounds such as **67.3b** perform much better. Increased freedom of intramolecular rotation, which is facilitated by the presence of acetylene spacers, was also present in the donor–acceptor conjugates **70.5a**–**c**. In this case, the accessibility of coplanar conformations results in intense charge–transfer absorption bands which extend into the NIR range (up to approximately 900 nm in **70.5c**). Overall panchromatic absorption across the visible spectrum is thus achieved.

**Scheme 71:**
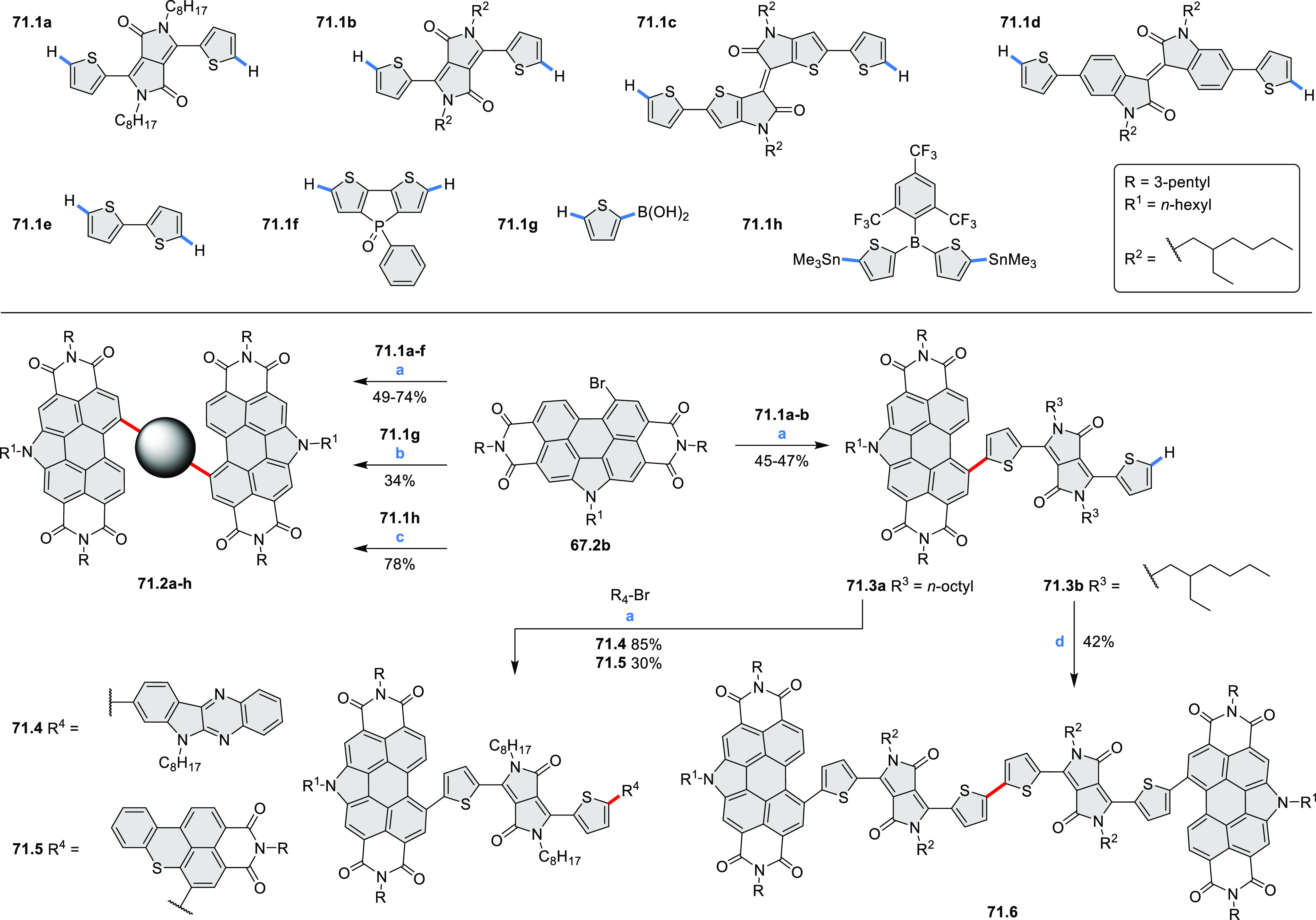
Preparation of Thiophene-Linked Dimeric N*-*Annulated PDIs via Direct Heteroarylation Reagents and conditions: (a)^129^ SiliaCat DPP-Pd, PivOH, K_2_CO_3_, DMA, 80–90 °C, 24 h (or microwave, 80 °C, 4 h for **71f**), 49–74%; (b)^136^ SiliaCat DPP-Pd, PivOH, Cs_2_CO_3_, DMA, 80 °C, 18 h, 34%; (c) Pd_2_(dba)_3_, P(*o*-Tol)_3_, toluene, 80 °C, 36 h, 78%; (d)^135^ Pd(OAc)_2_, pivalic acid, K_2_CO_3_, *N*,*N’*-dimethylacetamide, 80 °C, 24 h.

Palladium-catalyzed C–H arylation of thiophenes in the presence of catalytic pivalate was extensively used by Welch and co-workers to prepare conjugates of N*-*annulated PDI **67.2b** with various thiophene-containing units (Scheme 71).^129−136^ This methodology provided symmetrically substituted **71.1a**–**g** in a single step in moderate yields. Additionally, unsymmetrical **71.4**(129) and **71.5**(133) could be prepared via two consecutive steps. A product of a single arylation (**71.3b**) was also elaborated into the dimeric **71.6** via thiophene homocoupling.^135^ As the borane functionality was reactive under the direct arylation conditions, Stille coupling was used instead to prepare **71.2h**.^136^ In contrast to the analogues with ethynyl spacers (Scheme 70), these compounds adopt twisted conformations wherein the PDI is out of the plane of the adjacent thiophene ring. As a result, the low-energy charge transfer absorption is much weaker in **71.2b**(130) than in its ethynyl-linked counterpart **70.4a**. Further conformational control was realized using fused (**71.2f**)^134^ or twisted (**71.2h**)^136^ core subunits. Additionally, solvent vapor annealing of thin films could cause molecular reorganization, causing a remarkable enhancement of low-energy absorption of the unsymmetrical **71.4**.^129^ Strong absorptions seen in the 600–800 nm range for **71.1c**(130) and **71.6**(135) were presumably due to the planarity of their bithiophene units, resulting in greater π-electron delocalization. When tested as acceptor materials in bulk heterojunction OSC devices, the best results were obtained with **71.2a** (PCE of 5.6%).^131^ However, the conjugates shown here generally did not exceed the performance of the structurally simpler N-annulated PDI dimers (Scheme 67).

**Scheme 72:**
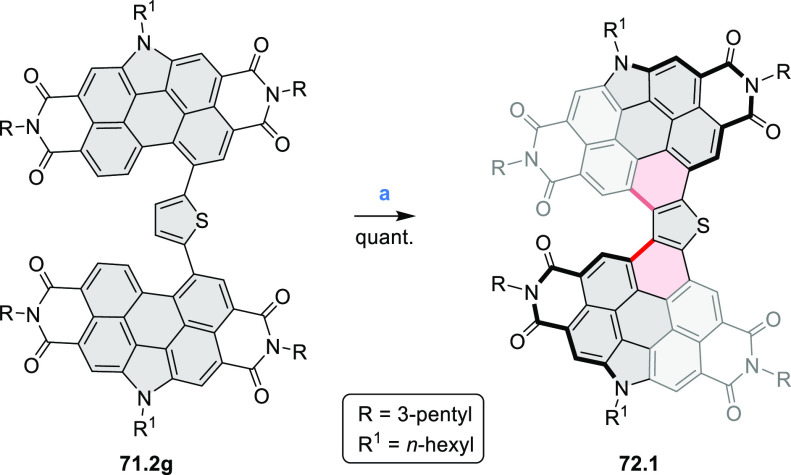
Preparation of an N-Annulated PDI Dimer with a Fused Thiophene Bridge Reagents and conditions: (e)^137^ FeCl_3_, MeCN, toluene, 50 °C, 1 min, quant.

Compound **71.2 g** was converted into the fused product **72.1** in a fast and efficient oxidation with FeCl_3_ (Scheme 72).^137^ Short reaction time was in this case essential for good results, as impurities were formed after longer periods of up to 30 min. OSCs prepared with **72.1** blended with PTB7-Th possessed unusually high open-circuit voltage in the range of 1.05–1.15 V. However, PCE values of up to only 2.78% could be obtained.

**Scheme 73:**
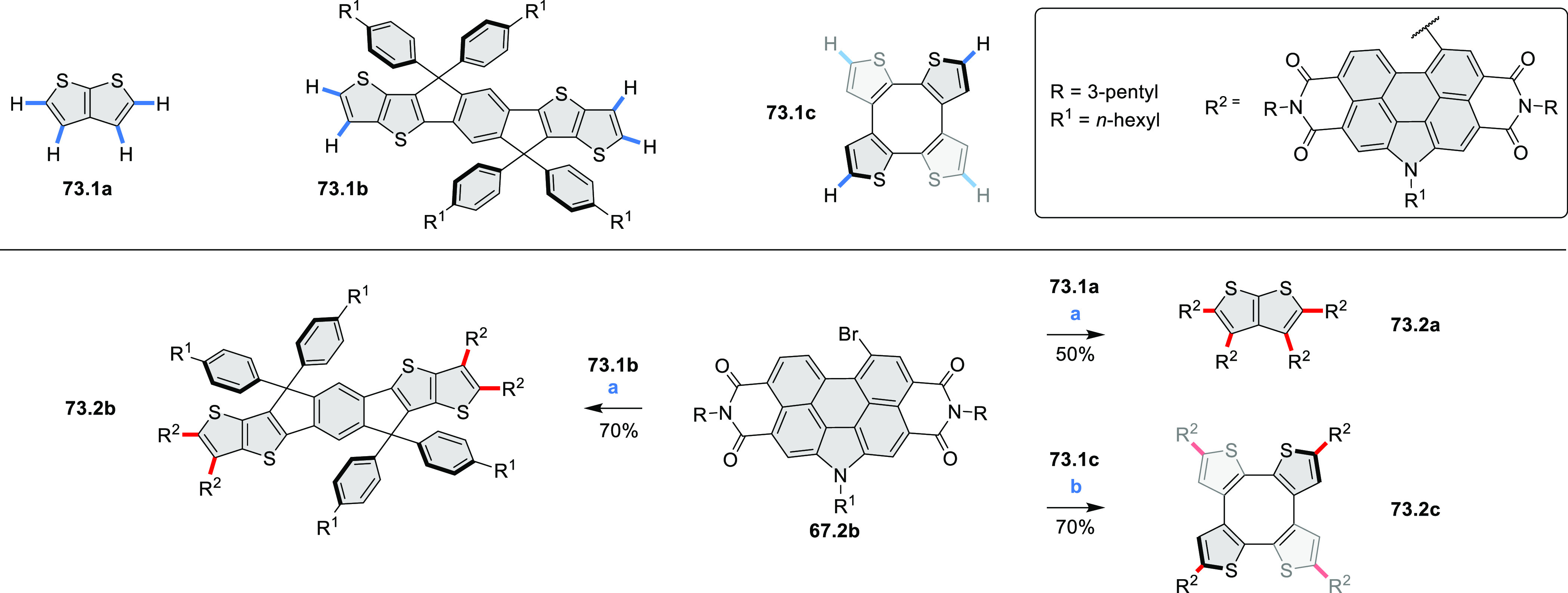
Direct Heteroarylation Leading to Tetrameric N*-*Annulated PDI Conjugates Reagents and conditions: (a)^139^ SiliaCat DPP-Pd, PivOH, K_2_CO_3_, DMA, 120 °C, 24 h, 70%; (b)^140^ SiliaCat DPP-Pd, AcOH, Cs_2_CO_3_, DMA, 120 °C, 24 h, 70%.

When the thienothiophene-based building blocks **73.1a** and **73.1b** were subjected to C–H arylation, their terminal thiophene rings reacted at both the α and β positions, yielding the highly sterically congested compounds **73.2a**(138) and **73.2b**,^138,139^ each bearing four N*-*annulated PDI units (Scheme 73). Preparation of these compounds was chromatography-free and selective: **73.2b** was obtained in 70% yield with only the trisubstituted compound as a major byproduct. DFT calculation showed that the PDIs are orthogonal to the core subunits of **73.2b**, which adopts a butterfly-shaped structure. A low-energy emission (650–900 nm) was observed, indicating an unusually large Stokes shift. When applied as an acceptor material in OSCs, **73.2b** reached moderate PCE values of up to 3.41%, reflecting the low electron mobility of this material in comparison with the hole mobility.

Another sterically congested tetrakis PDI) conjugate prepared by this methodology was **73.2c**, where a saddle-shaped structure was achieved through introduction of a cyclooctatetrathiophene core.^140^ In this case, using catalytic acetate instead of pivalate allowed us to achieve a higher yield. This improvement was attributed to decreased steric strain in the concerted palladation–deprotonation step, which is responsible for the C–H activation of thiophenes. OSC devices formulated with **73.2c**, a PTB7-Th donor polymer, and a 1-chloronaphthalene additive displayed PCEs of up to 4.26%, with improved fill factor and short-circuit current in comparison to **73.2b**.

**Scheme 74:**
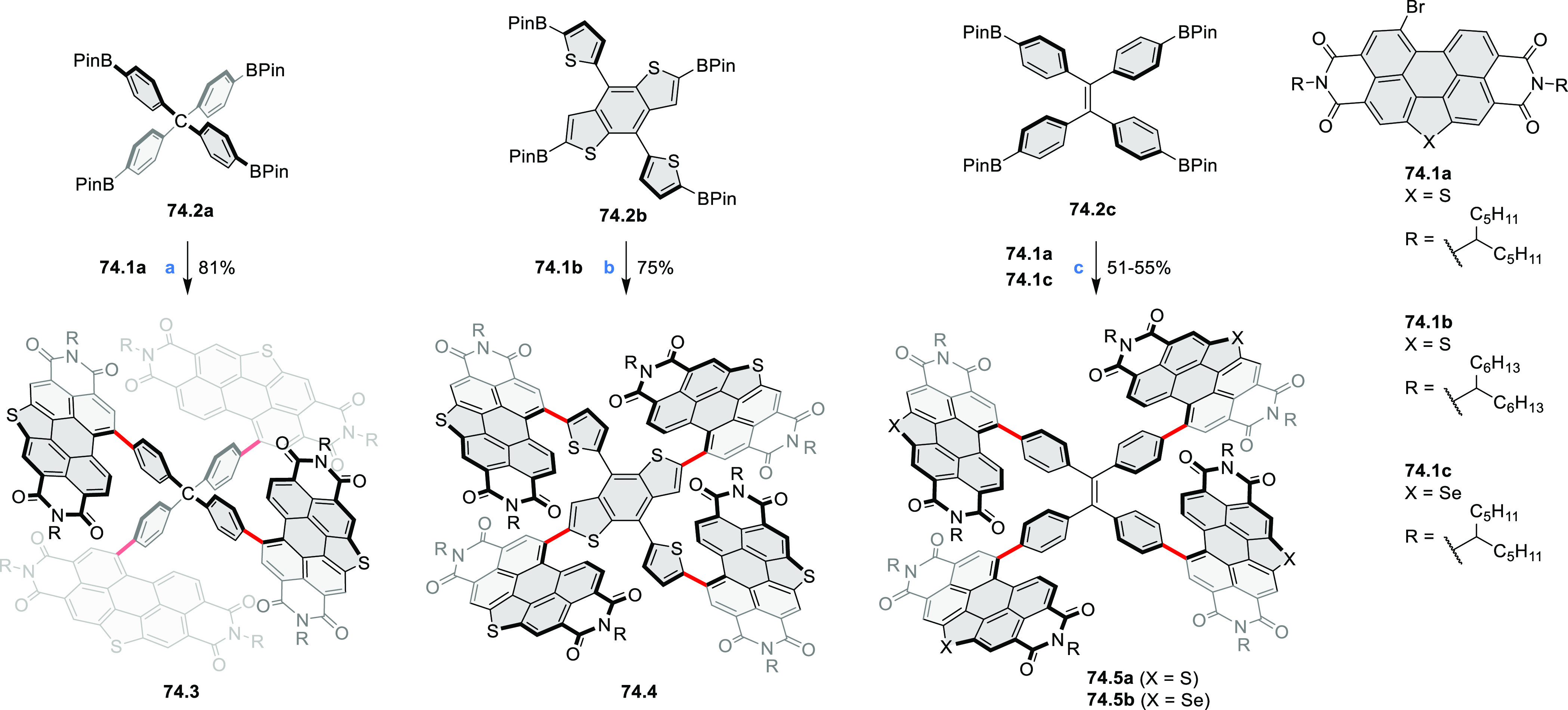
Synthesis of Three-Dimensional Thiophene-Annulated PDI Dyes Reagents and conditions: (a)^141^ Pd(PPh_3_)_4_, K_2_CO_3_, THF, reflux, 2 days, 81%; (b)^142^ Pd_2_(dba)_3_, P(*p*-C_6_H_4_OMe)_3_, K_2_CO_3_, THF/H_2_O, 16 h, reflux, 75%; (c)^143^ Pd(PPh_3_)_4_, K_2_CO_3_, 2:1 THF/H_2_O, 80 °C, 2 days, 51–55%.

Similarly to N*-*annulated perylene diimides, their S- and Se-annulated analogues can be efficiently brominated to 6-bromo derivatives^126^ such as **74.1a**–**c** and further derivatized via cross-coupling reactions. A series of compounds with four S- or Se-annulated PDI units located on different core synthons were prepared in this manner via Suzuki coupling in the final step (Scheme 74).^141−143^ In all cases, the S- or Se-annulation was introduced in earlier steps similarly as shown above in Scheme 60. The sterically constrained structures of these molecules were intended to prevent aggregation via π-stacking and lead to improved morphology and phase separation in blends with donor polymers. OSCs built using **74.3** combined with the PBDT-TS1 polymer and diphenyl ether additive reached a PCE of 6.17%.^141^ Importantly, **74.3** greatly outperformed its analogue lacking the S*-*annulation (3.62%). A similar trend in PCE was observed, with **74.4** performing better than a nonannulated counterpart (8.28% vs 7.16%).^142^ In both cases, the improvement was attributed to balanced carrier mobilities as well as to the increase of LUMO energies caused by the electron-donating character of the S atom. Among dyes derived from tetraphenylethylene, the Se-containing **74.5b** displayed a higher maximum PCE of 7.63% compared to 6.85% for **74.5a**.^143^ Compound **74.5b** performed better across the full range of parameters, showing remarkably high open-circuit voltage (1.078 V) and fill factor (68.8%) as well as well-balanced carrier mobility (μ_h_ = 3.21 × 10^–4^ cm^2^ V^–1^ s^–1^, μ_e_ = 1.37 × 10^–4^ cm^2^ V^–1^ s^–1^).

**Scheme 75:**
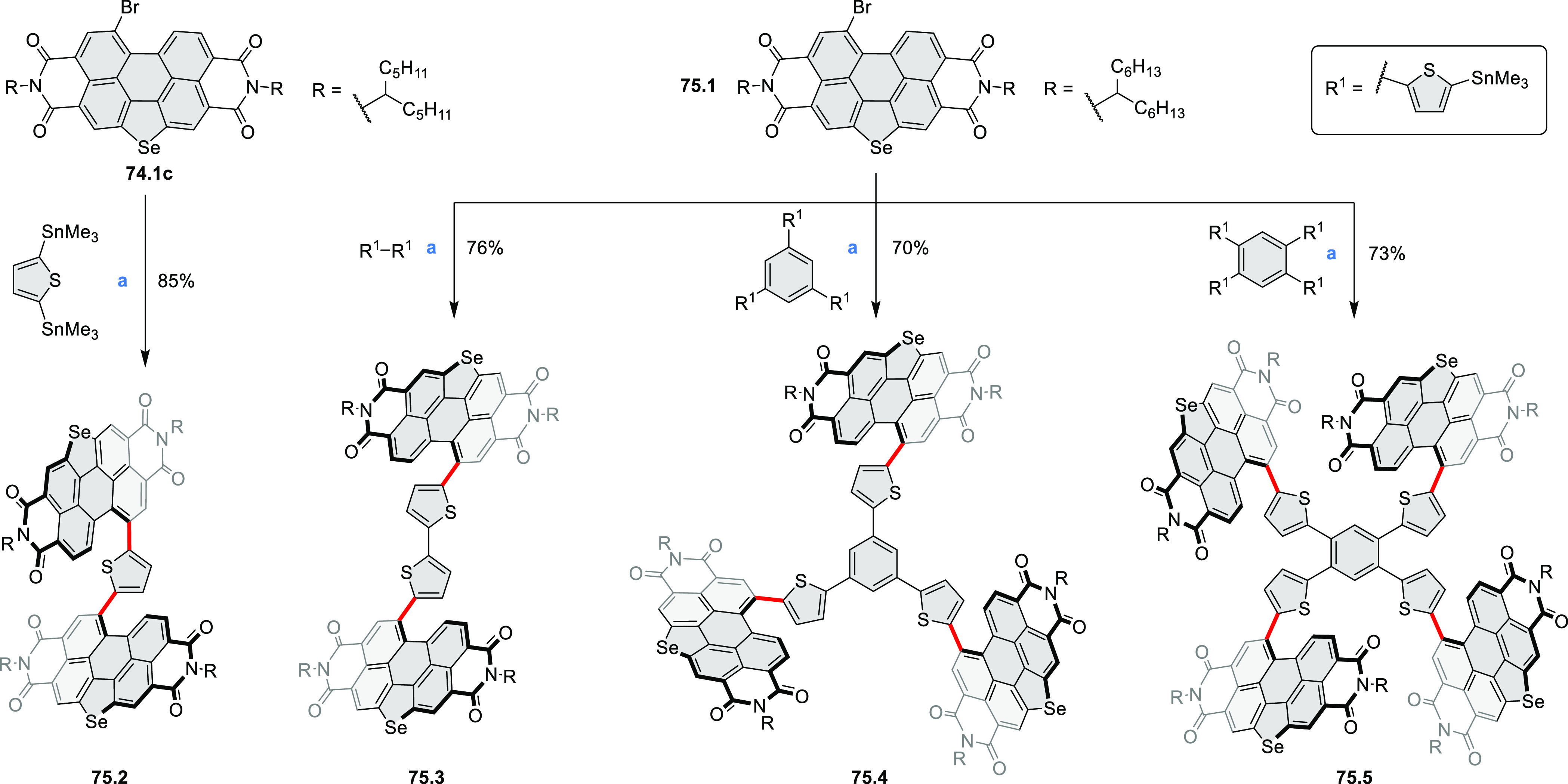
Synthesis of Acceptors with Two, Three, and Four Se-Annulated Peryleneimide Units Reagents and conditions: (a)^144,145^ Pd_2_(dba)_3_, P(*o*-tolyl)_3_, toluene, reflux, 24 h, 70–85%.

Chen, He, and co-workers used Stille coupling to efficiently append Se-annulated peryleneimide units onto thiophene-based cores to provide **75.3**–**5** (Scheme 75).^144^ Compound **75.4** was found by DFT calculations to have the greatest steric constraints, preferentially adopting a conformation where two of its PDI units are nearly orthogonal. This feature was thought to disfavor aggregation and was found to be beneficial for the performance of organic photovoltaics based on combinations of these materials with the PBDB-T thiophene-based donor polymer. Electron mobility, hole mobility, and short-circuit current were the highest with **75.4**, resulting in the best PCE value (**75.3**: 4.10%, **75.4**: 5.82%, **75.5**: 5.10%). A related compound, **75.2**, likewise had a twisted geometry, with an ca. 119° angle between PDI planes according to molecular modeling.^145^ Although not directly comparable to **75.3**–**5** because of the use of different donor polymers, OSCs based on **75.2** displayed a lower performance, with PCE of up to 2.53%.

**Scheme 76:**
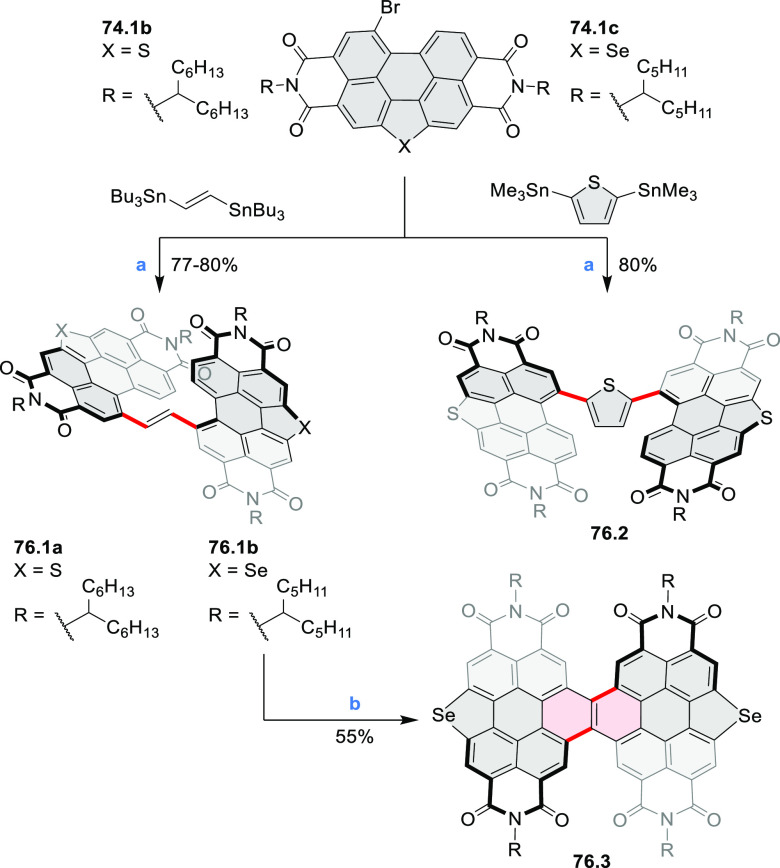
Synthesis of Vinylene- and Thiophene-Bridged S- and Se-Annulated PDI Dimers Reagents and conditions: (a)^146^ Pd(PPh_3_)_2_Cl_2_, toluene, reflux, 24 h, 77–80%; (b)^147^ I_2_, toluene, *hν* (Hg lamp), 36 h, 55%.

Double S- or Se-annulated PDIs linked via a vinyl or a thiophene bridge were prepared using a similar approach based on Stille coupling (Scheme 76). Likewise, **76.1a**,**b** and **76.2** adopt a twisted structure which prevents aggregation and helps to form smooth films upon spin coating.^146^ These compounds were used as interfacial materials in perovskite solar cells, positioned as an interlayer between the perovskite absorbing layer and the fullerene-based electron transport layer. The presence of the interlayer improved charge transport and inhibited charge recombination, resulting in better device performance (no interlayer: PCE 17.53%, FF 77.50%; **76.2** interlayer: PCE 20.41%, FF 83.86%). The Se-containing **76.1b** was further converted into the fused dimer **76.3** via UV irradiation in the presence of catalytic I_2_.^147^ This fused product was predicted by DFT modeling to adopt a highly twisted geometry with a nearly 35° angle between the PDI planes. The absorption spectrum of **76.3** has maxima at 471 and 509 nm, which are blue-shifted relative to the precursor **76.1b** (483 nm, 517 nm). HOMO and LUMO levels were estimated to be similar in both compounds. When used in OSC devices alongside the PBDB-T donor polymer, **76.3** enabled higher and more balanced carrier mobilities compared to **76.1b**. This corresponded to higher PCE values of up to 7.41% for **76.3**, achieved with a 1,8-diiodooctane additive.

**Scheme 77:**
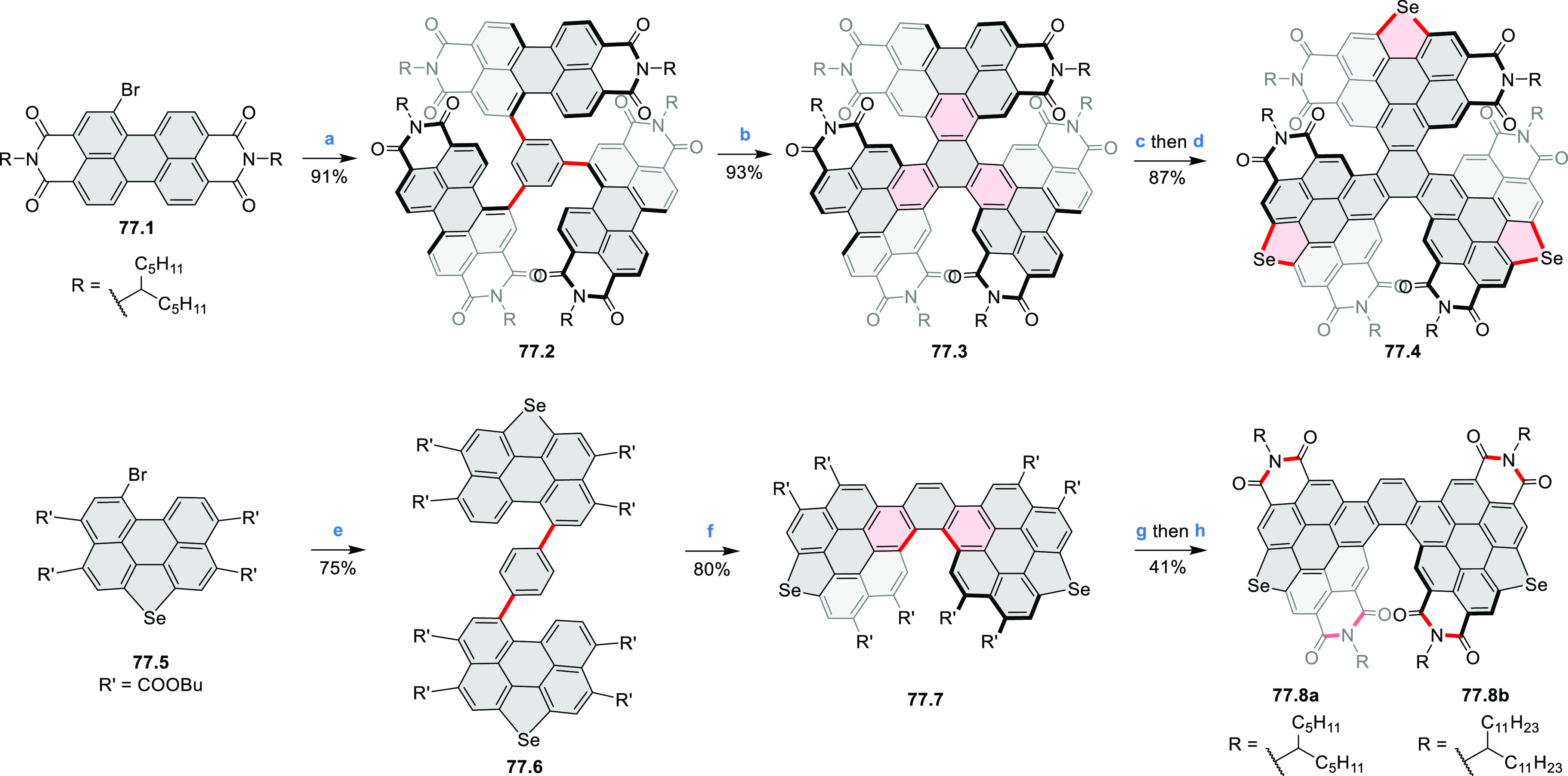
Synthesis of Benzene-Fused Se-Annulated PDI Dimers and Trimers Reagents and conditions: (a)^148^ 1,3,5-benzenetriboronic acid tris(pinacol)ester, Pd(PPh_3_)_4_, Na_2_CO_3_, 2:1 THF/H_2_O, reflux, 48 h, 91%; (b) I_2_, toluene, *hν* (Hg lamp), rt, 24 h, 93%; (c) HNO_3_ (fuming), DCM, rt, 12 h, quant.; (d) selenium powder, NMP, 190 °C, 12 h, 87%; (e)^149^ Pd(PPh_3_)_4_, K_2_CO_3_, 2:1 THF/H_2_O, 85 °C, 13 h, 75%; (f) I_2_, DCM, *hν* (sunlight), rt, 8 h, 80%; (g) ClSO_3_H, rt, 4 h, quant.; (h) 6-undecylamine or 12-tricosylamine, imidazole, 150 °C, 4 h, 41%.

UV irradiation in the presence of catalytic I_2_ was also used to achieve the fusion of three PDI blocks to a common benzene core, leading to the propeller-shaped **77.3** (Scheme 77).^148^ This compound was then subjected to late-stage nitration, providing a mixture of bay-nitrated isomers, followed by Se annulation to provide **77.4**. These molecules are highly twisted because of steric crowding, especially in **77.4** where the Se annulation further rigidifies the PDI. In the solid-state structure of **77.4**, pairs of Se···O chalcogen bonding contacts with a distance close to 3.0 Å were observed between adjacent molecules. OSCs built using **77.3** or **77.4** blended with the PDBT-T1 donor polymer displayed higher open-circuit voltage for **77.4** (1.00–1.01 V vs 0.96–0.97 V for **77.3**), reflecting the higher LUMO level of **77.4**. Blend films with **77.4** also displayed higher carrier mobilities, which were otherwise well-balanced with both compounds (μ_e_/μ_h_ = 1.3–1.5). These favorable properties resulted in PCE values reaching 8.28% for **77.3** and 9.28% for **77.4** in the presence of a 1,8-diiodooctane additive. A similar approach was also used in the preparation of dimeric benzene-fused PDIs **77.8a**,**b**.^149^ In this case, **77.6** was exposed to sunlight in the presence of I_2_, providing the fused **77.7** regioselectively despite its steric congestion. Perylene tetraesters were then converted into PDIs in the final steps. In films of **77.8a** blended with PBDB-T, μ_e_ was higher by an order of magnitude than for **77.8b**, implying that long alkyl chains of **77.8b** impeded charge transport. This difference was reflected in a much better OSC performance of **77.8a** (PCE up to 7.41%, *J*_SC_ = 11.34 mA/cm^2^) compared to **77.8b** (PCE up to 4.57%, *J*_SC_ = 8.28 mA/cm^2^).

**Scheme 78:**
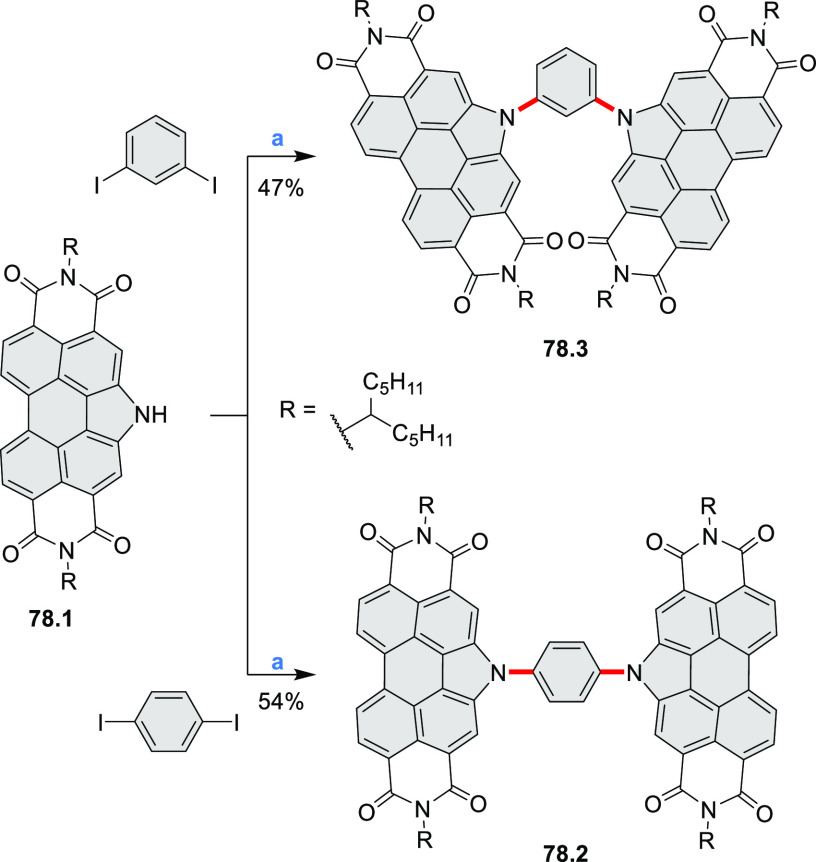
Synthesis of Phenylene-Bridged Isomeric N*-*Annulated Perylene Diimide Dimers Reagents and conditions: (a)^150^ Pd(OAc)_2_, *t*-BuOK, P(*t*-Bu)_3_, toluene, 110 °C, o/n, 47–54%.

N-Annulated PDI chromophores were appended to a phenylene spacer via Buchwald–Hartwig amination (Scheme 78).^150^ The isomeric products **78.2**–**3** displayed nearly identical UV–vis spectra and electrochemical behavior. In the solid state, however, the slightly convex PDI units were nearly parallel in **78.2** but close to orthogonal in **78.3**. Additive-free OSCs containing blends of these compounds with the PTB7-Th donor polymer performed better for **78.3**, in line with several reports of superior properties of PDI-based acceptor materials with a twisted structure. The use of a 1-chloronaphthalene additive enabled greater optimization for **78.2**, leading to a PCE of 5.01%, compared to 4.15% for **78.3** with a 1,8-diiodooctane additive. These results were explained by demonstrating a significantly faster symmetry-breaking charge separation in **78.2** than in **78.3**, attributed to better conjugation through the *para*-phenylene bridge.^151^ This process was shown to be particularly favorable in polar solvents (THF and acetone), where charge separation became exergonic.

**Scheme 79:**
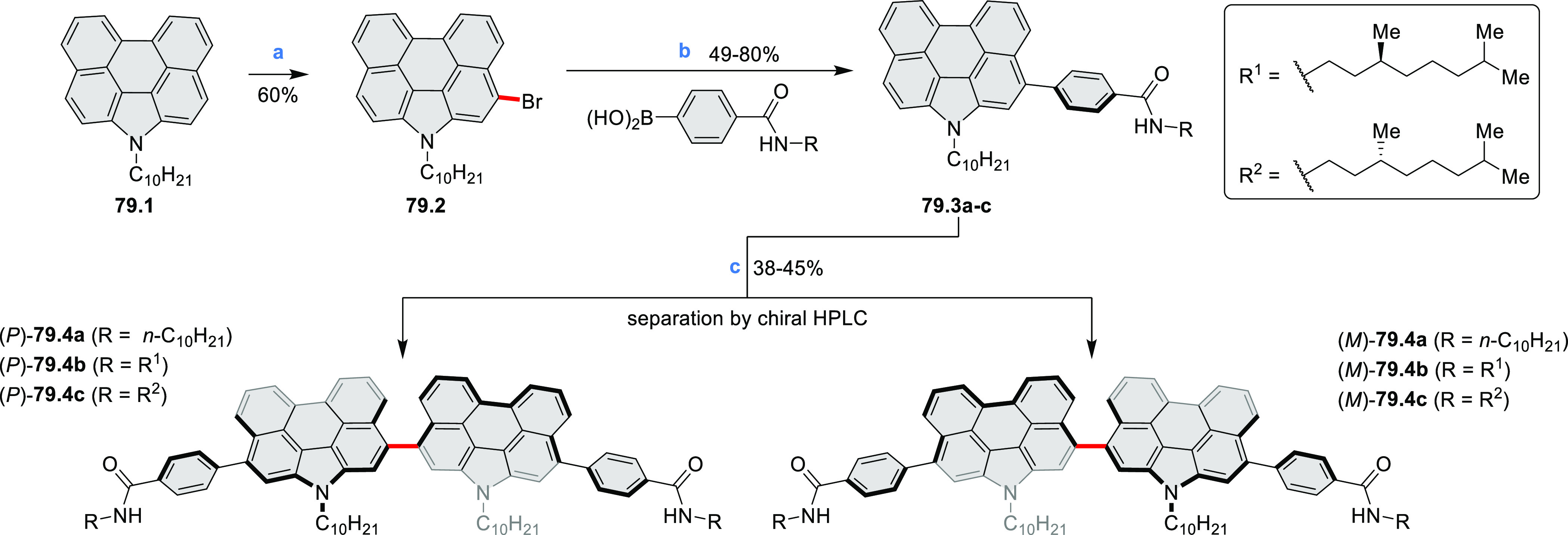
Chiral Twin N-Annulated Perylene Carboxamides Reagents and conditions: (a)^152^ NBS, DMF, 0 °C, 30 min, 60%; (b) Pd(PPh_3_)_4_, K_2_CO_3_, 19:1 THF/water, reflux, 40 h, 49–80%; (c) Sc(OTf)_3_, DDQ, toluene, rt, 22–48 h, 38–45%.

N-Annulated perylene **79.1** (prepared via the Cadogan reaction similarly as shown in Scheme 56) was selectively monobrominated to the 3-bromo derivative **79.2**, which was then derivatized via Suzuki coupling to **79.3a**–**c** (Scheme 79).^152^ These fragments were oxidatively homocoupled at the 10 position in a process enabled by the electron-donating character of the pyrrolic ring. In the dimers **79.4a**–**c**, rotation around the central C–C bond was sufficiently restricted to permit resolution of atropisomers by HPLC. These compounds underwent self-assembly via a combination of π-stacking and hydrogen-bonding interactions between the amide groups. The aggregation occurred upon addition of cyclohexane to a DCM solution and was complete in 19:1 cyclohexane/DCM. A preference for homochiral aggregation was observed, which was determined by the axial chirality of the biperylenyl core, overriding the point chirality of the tetrahydrogeranyl chains.

**Chart 7:**
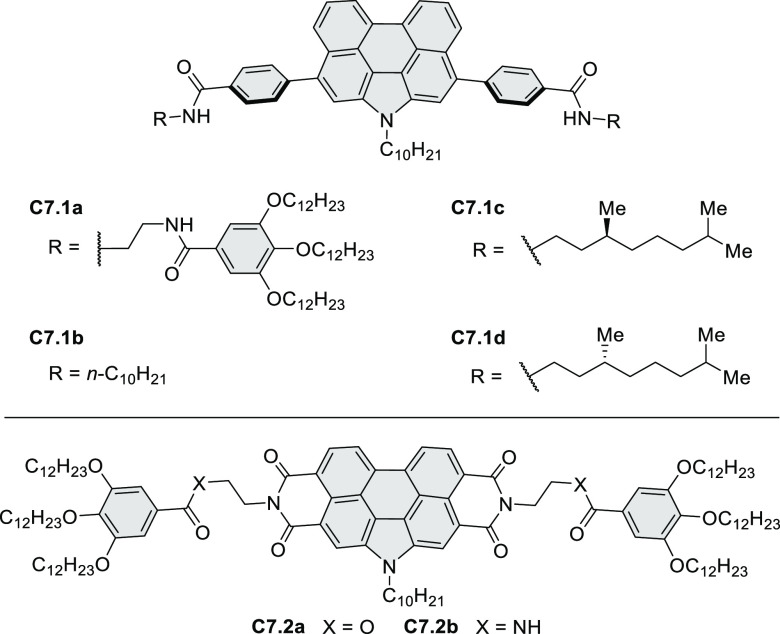
N*-*Annulated Perylenes with Amide-Bound Side Chains

Compounds **C7.1a**–**d** (Chart 7), obtained using similar synthetic methods, formed aggregates in nonpolar solvent, analogous to those observed for **79.4a**–**c**. In the case of **C7.1a**, intramolecular hydrogen bonding between adjacent amide groups caused aggregation in toluene to proceed with a lag period of 10–40 min, followed by the formation of a distinct intermediate and final aggregates.^153^ The lag period could be eliminated upon seeding with the intermediate aggregate, indicating that it possessed active sites for supramolecular polymerization. The formation of fibrillar aggregates in methylcyclohexane was also reported for compounds **C7.1b**–**d**.^154^ Columnar assembly with NAP units oriented in the same direction in adjacent monomers was identified. A mixture of *M*- and *P*-type aggregates was always obtained, regardless of point chirality in the alkyl side chains. The helicity of these structures was postulated to spontaneously interconvert via intrastack stereomutation. This flexible chirality was used to produce CD response in solution upon mechanical agitation, with opposite CD spectra being obtained with clockwise and counterclockwise stirring. Aggregation properties were also studied for the related **C7.2a**,**b**, functionalized with trialkoxyphenyl groups.^155^ Analog **C7.2a** with ester-appended lateral groups formed a single type of fibrillar aggregate with an absorption spectrum red-shifted relatively to the monomeric species. The presence of amide linkers in **C7.2b** enabled intra- and intermolecular hydrogen bonding, resulting in a more complex behavior reminiscent of that outlined above for **C7.1a**. Overall, four types of fibrillar and nanosheet aggregates were formed by **C7.2b** under different conditions.

**Chart 8:**
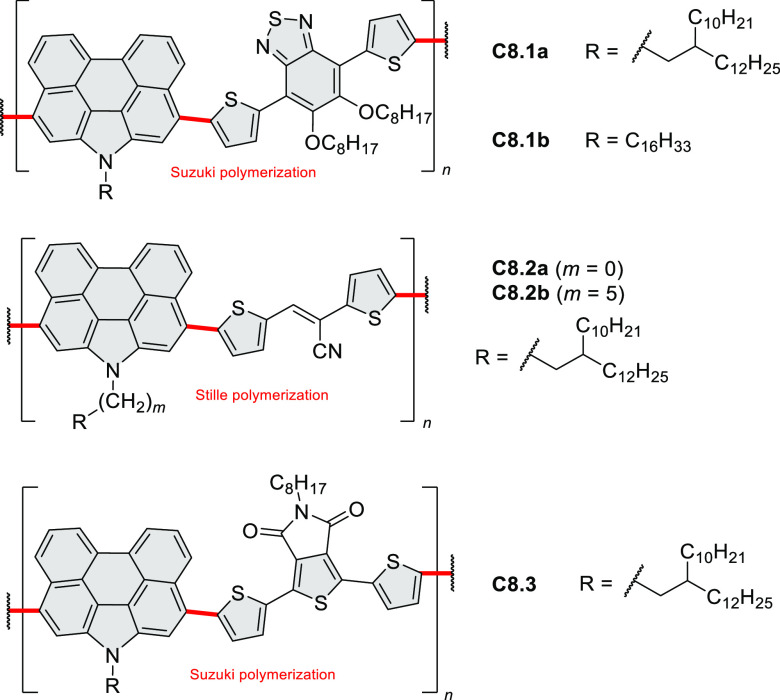
Polymers Containing N*-*Annulated Perylene Units

N-Annulated perylenes were incorporated as electron-rich donor units into push–pull copolymers (Chart 8). These systems were evaluated as dyes for organic photovoltaics in combination with the fullerene-based acceptor PC_71_BM (**C8.1a**,**b** and **C8.2a**,**b**) or for green-selective photodiodes (**C8.3**). The polymers were prepared via Pd-catalyzed Suzuki or Stille polymerization reactions from the respective diboronic acid or dibromide precursors. In materials **C8.1a** and **C8.1b**, the presence of a branched alkyl chain resulted in a slight broadening (ca. 20 nm) of the visible absorption band in the 400–600 nm range.^156^ The photovoltaic performance of these polymers was inferior to an analogue containing carbazole donor units, with PCEs of 2.4–2.5% (5.8% for the carbazole-based material). This decrease was attributed mainly to a lower yield of charge-separated excited states.

In copolymers **C8.2a**,**b**, the longer alkyl chain caused **C8.2b** to adopt a more planar conformation in the solid state, resulting in slightly red-shifted absorption and emission spectra.^157^ In photovoltaic devices with films composed of polymer and PC_71_BM in a 1:4 weight ratio, **C8.2b** afforded a higher PCE of 4.80%, compared to 3.63% for **C8.2a**. A planar heterojunction photodiode was prepared with spin-coated films of ZnO and polymer **C8.3**.^158^ The ICT absorption of **C8.3** in the amorphous solid film (538 nm with 138 nm fwhm) produced a green-selective response of the device. At a reverse bias voltage (−5 V), the photodiode was characterized by a low dark current of 0.68 nA/cm^2^ and a strong response to 550 nm light with a detectivity of 1.04 × 10^12^ Jones.

### 3.3 [*ghi*]Heteroannulated Perylenoids: Six- and Seven-Membered Rings

**Scheme 80:**
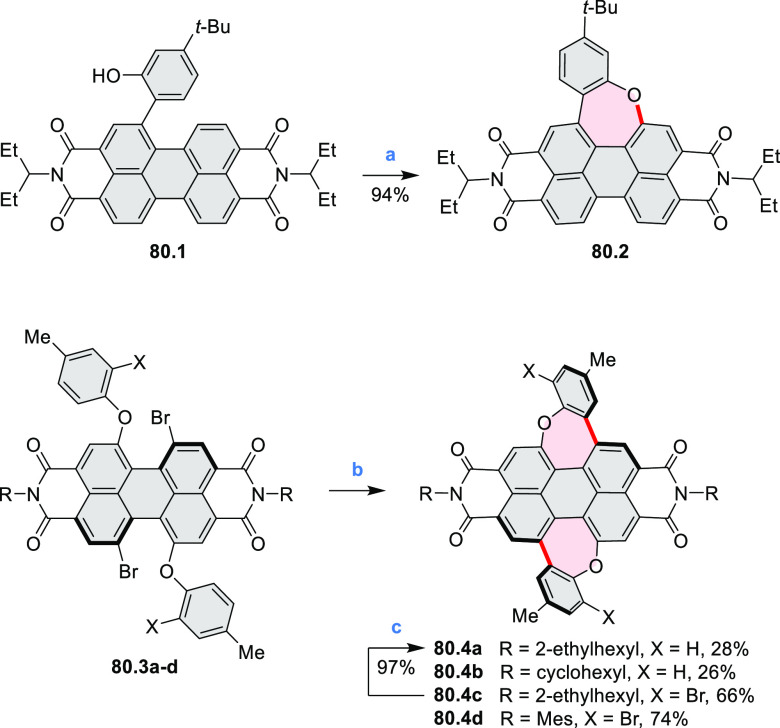
Synthesis of Benzoxepine-Fused Perylene Diimides Reagents and conditions: (a)^159^ Cu(OAc)_2_, Cs_2_CO_3_, PivOH, DMSO, 140 °C, overnight, 94%; (b)^160^ Pd(OAc)_2_, PCy_3_·HBF_4_, K_2_CO_3_, DMA, 100–120 °C, 2–4 h (c) Pd(PPh_3_)_4_, Cs_2_CO_3_, paraformaldehyde, 1:1 DMF/toluene, 80 °C, 17 h, 97%.

[*ghi*]Benzoxepine-fused perylene diimide **80.2** was obtained via Cu-mediated ring closure of the phenol-substituted PDI precursor **80.1** (Scheme 80).^159^ Smaller ring fusion was also possible by this method, leading to analogues with five- and six-membered rings (Scheme 141, Section 4.3). Fusion of the benzoxepine ring in **80.2** led to a hypsochromic shift and a quenching of the emission quantum yield by 16% compared to the parent PDI. Another method to access the benzoxepine-fused PDI framework is the Pd-catalyzed intramolecular cyclization to effect the fusion of a pendant phenyl ether substituent (Scheme 80).^160^ C–H activation at the *ortho* position of this substituent enables its coupling to a bay-brominated PDI core. This method was applied on PDIs **80.3a**–**d** and was reported to be unsuccessful when the respective perylene tetraesters were used instead. While the cyclized compounds **80.4a**,**b** were obtained in relatively low yields, a greater efficiency was attained with the brominated substrates **80.3c**,**d**, leading to **80.4c**,**d**. The Br substituents on the electron-rich phenyl ether groups were not affected under the reaction conditions. Subsequent dehalogenation of **80.4c** was thus a more efficient way of preparing **80.4a** than its direct synthesis from **80.3a**. The cyclized products had a curved geometry with a 22° twist angle in the perylene core. This resulted in good solubility in organic media even in the absence of large imide-bound solubilizing substituents.

**Scheme 81:**
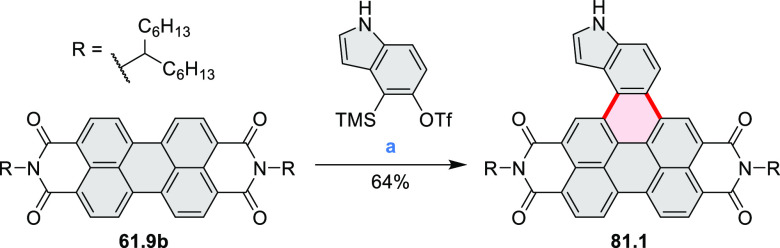
Benzyne Cycloaddition to Perylene Diimide Reagents and conditions: (a)^161^ CsF, 1:1 toluene/MeCN, 80 °C, 36 h, 64%.

Diels–Alder cycloaddition with benzynes was used to prepare PDIs fused with various aromatics, including the indole-fused molecule **81.1** (Scheme 81).^161^ Benzyne intermediates were obtained by diazotization of anthranilic acid derivatives or by desilylation of *o*-trimethylsilyl aryl triflates. The indole-fused **81.1** was characterized by higher HOMO and LUMO levels and much lower extinction coefficients compared to analogues fused with carbocyclic units.

**Scheme 82:**
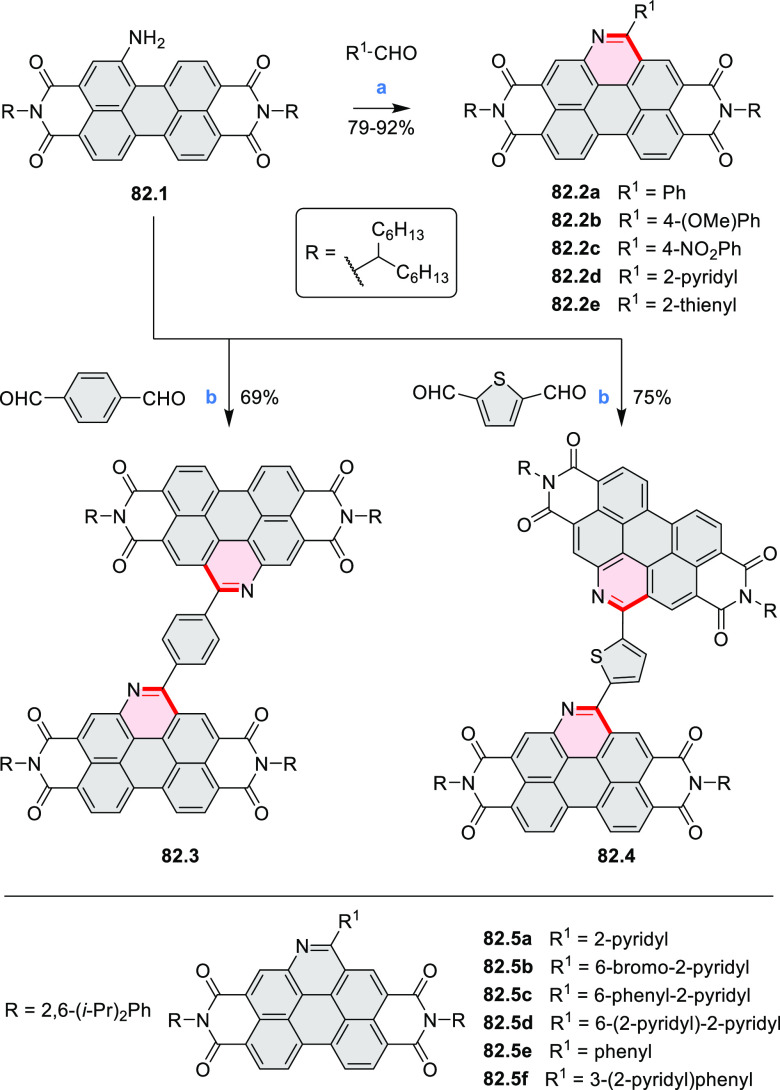
Azabenzannulation of PDI by Cyclization with an Imine Reagents and conditions: (a)^162^ (1) 3 equiv of R^1^CHO, TFA, DCM, reflux, 3 h, (2) DCM, rt, *hν* (white LED), 1 h (12 h for **82.2c**), (3) DDQ, DCM, rt, 2 min; (b) (1) 0.33 equiv of dialdehyde, TFA, toluene, reflux, 3 h, (2) DCM, rt, *hν* (white LED), 12 h, (3) DDQ, DCM, rt, 2 min.

Goujon, Hudhomme, and co-workers described the preparation of azabenzannulated PDI derivatives in a one-pot condensation–photocyclization–oxidation sequence from 1-aminoPDI **82.1** and various aryl aldehydes (Scheme 82).^162^ In the first step, an imine was formed from these starting materials in the presence of TFA. After concentrating the reaction mixture under vacuum, the crude imine was redissolved in DCM and subjected to visible-light irradiation, resulting in photocyclization to a secondary amine intermediate. This species was rapidly oxidized with DDQ, providing the azabenzannulated products **82.2a**–**e** in high yields. The dimeric products **82.3**–**4** were prepared in the same manner from the respective dialdehydes. The resulting compounds were emissive, with moderate to high quantum yields. Electron-rich groups on the nitrogen-containing ring resulted in increased HOMO levels and narrower band gaps. Thus, **82.2b**, **82.2e**, and the dimeric **82.4** were notable for broad and red-shifted emission bands (552, 541, and 572 nm, respectively). On the other hand, electron-deficient substituents in **82.2c**,**d** had little effect on the spectral properties. An earlier report by Würthner and co-workers included a similar methodology used to prepare **82.5a**–**f**, although with lower yields compared to the protocol described above.^4^ The synthesis of related perylene dianhydrides, anhydride imides, and tetraesters was also presented, as well as double azabenzannulations to provide diazacoronenes (see Scheme 6, Section 2.1.1).

**Scheme 83:**
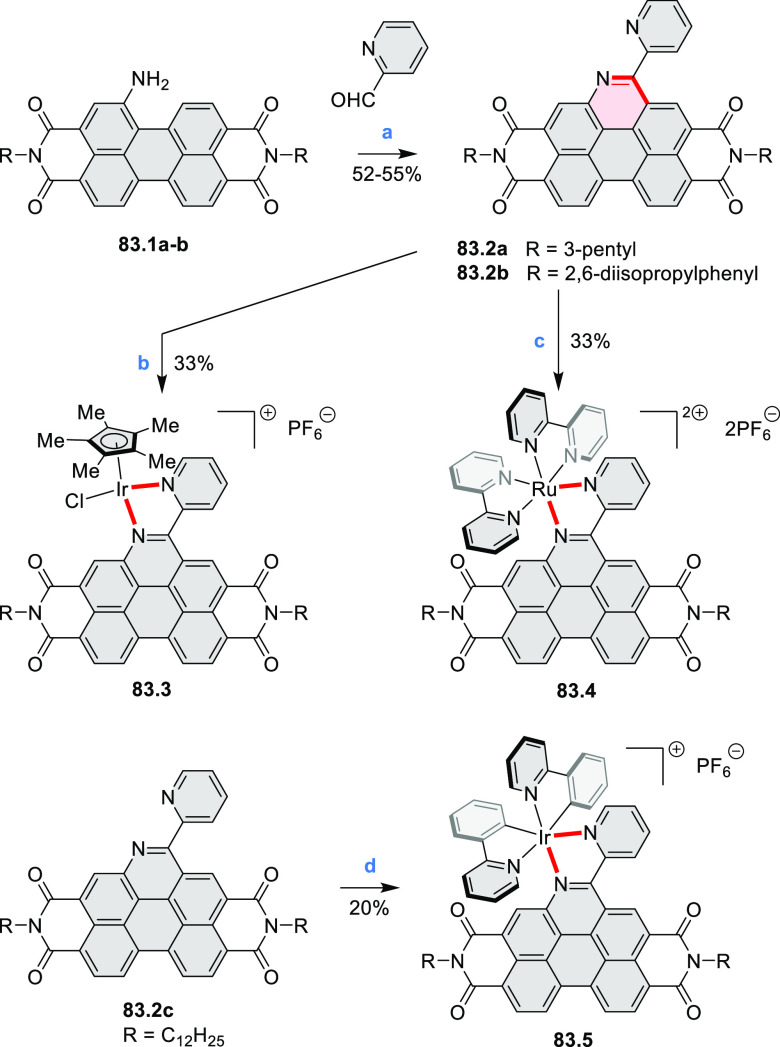
Coordination Chemistry of a Fused Perylenoid Ligand Reagents and conditions: (a)^164^ TfOH, DMF, 110 °C, 2–3 h, 52–55%; (b) [Cp*IrCl_2_]_2_, 40 °C, 18 h, then NH_4_PF_6_, EtOH, 67%; (c) Ru(bpy)_2_Cl_2_, LiCl, AgClO_4_, 20:7:1 CHCl_3_/EtOH/Et_3_N, 65 °C, 42 h, 69%; (d)^165^ [(ppy)_2_IrCl]_2_, 5:1 DCM/MeOH, 80 °C, 24 h, then NH_4_PF_6_, 2 h, 20%.

Dehydrogenative condensation of amino-substituted PDIs with 2-formylpyridine according to a previously reported method^163^ led to ligands **83.2a**–**c** (Scheme 83). These compounds were then used to prepare the Ir(III) and Ru(II) complexes **83.3**–**5**. Phosphorescence in the NIR region (700–1100 nm) was observed with **83.3** and **83.4**.^164^ The ruthenium complex **83.4** had a particularly high phosphorescence quantum yield of 11% with a 4.2 μs lifetime. Low quantum yield (<1%) with a 33 μs lifetime was recorded for **83.3**. For compound **83.5** a strong aggregation-induced dependence of the emission spectrum on concentration was reported.^165^ Strong NIR emission with peaks at 736 and 824 nm was seen at high concentration (10^–2^ M) but not in dilute solution (10^–5^ M). Emission at 758 nm was also seen in films prepared from neat **83.5** as well as in a blend with PMMA.

**Scheme 84:**
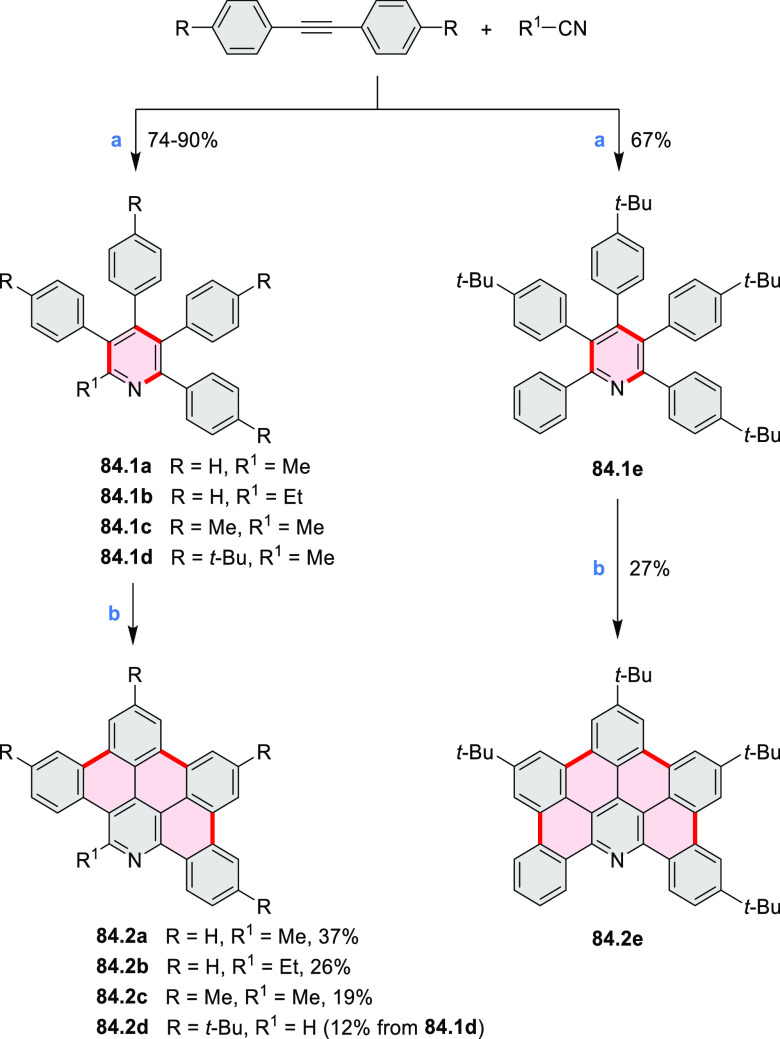
Mechanochemical Cyclodehydrogenation of Polyarylpyridines Reagents and conditions: (a)^166^ CoI_2_, 1,3-bis(diphenylphosphino)propane, Zn, NMP, or DMA, 80 °C, 24 h; (b) K metal, ball milling (30 Hz), rt, 2 h.

Wang, Yoshikai, and co-workers reported the synthesis of pentasubstituted pyridines via Co-catalyzed [2 + 2 + 2] cyclotrimerization between two alkynes and a nitrile (Scheme 84).^166^ The reaction was proposed to proceed via an initial coupling of two alkyne molecules into a cobaltacyclopentadiene, followed by the insertion of nitrile. This process required cobalt(0) complexes, which formed in situ in the presence of Zn dust. Among the compounds prepared by this methodology, the tetra- and pentaarylpyridines **84.1a**–**e** were subsequently cyclized into the N*-*doped perylenoid PAHs **84.2a**–**e**. This cyclodehydrogenation was achieved via an unusual method of solvent-free ball milling of **84.1a**–**e** with K metal. Attempts to effect the same transformation under oxidative coupling conditions (e.g., with FeCl_3_ in MeNO_2_/DCM) failed, apparently because of the electron-deficient and basic character of pyridine. In the case of **84.1d**, an unexpected demethylation occurred, providing **84.2d** in 12% yield.

**Scheme 85:**
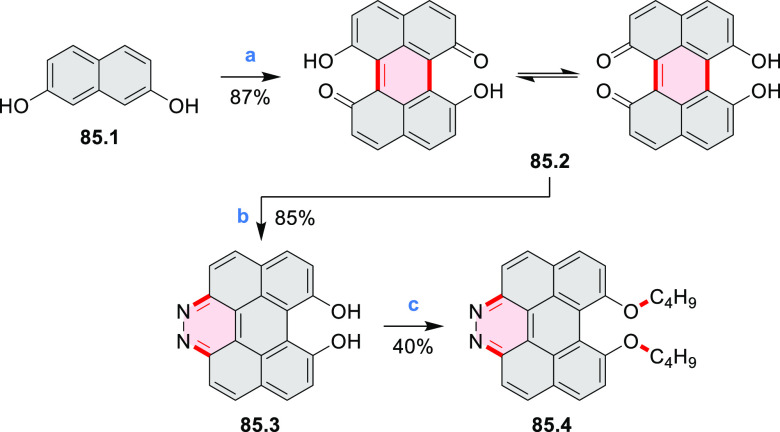
Synthesis of Pyridazine-Fused Perylenes Reagents and conditions: (a)^167^ KMnO_4_, 1:9 MeOH/H_2_O, rt, 12 h, 87%; (b) N_2_H_4_·H_2_O, EtOH, rt, overnight, 85%; (c) *n*-BuBr, NaOH, DMF, 60 °C, overnight, 40%.

A pyridazine-fused perylene **85.3** was prepared in a short and efficient sequence from dihydroxynaphthalene **85.1** (Scheme 85).^167^ First, oxidation in a dilute KMnO_4_ solution formed the perylene ring system in **85.2**. This reaction was proposed to proceed via a diradical intermediate. While the trans tautomer of **85.2** was predicted to be more stable than the cis form, their interconversion was fast at rt, and thus the condensation product **85.3** was efficiently formed from the cis tautomer and hydrazine. The product was further derivatized by O*-*alkylation to provide **85.4**. The crystal structure of **85.3** revealed a twisted geometry of the perylene core with a dihedral angle close to 30°. The twist arises due to the steric clash between the alkoxy substituents. Compound **85.3** displayed a broad red emission peaking at 628 nm, while a narrower emission blue-shifted to 521 nm was seen in **85.4**. Large Stokes shifts were observed in both cases.

**Scheme 86:**
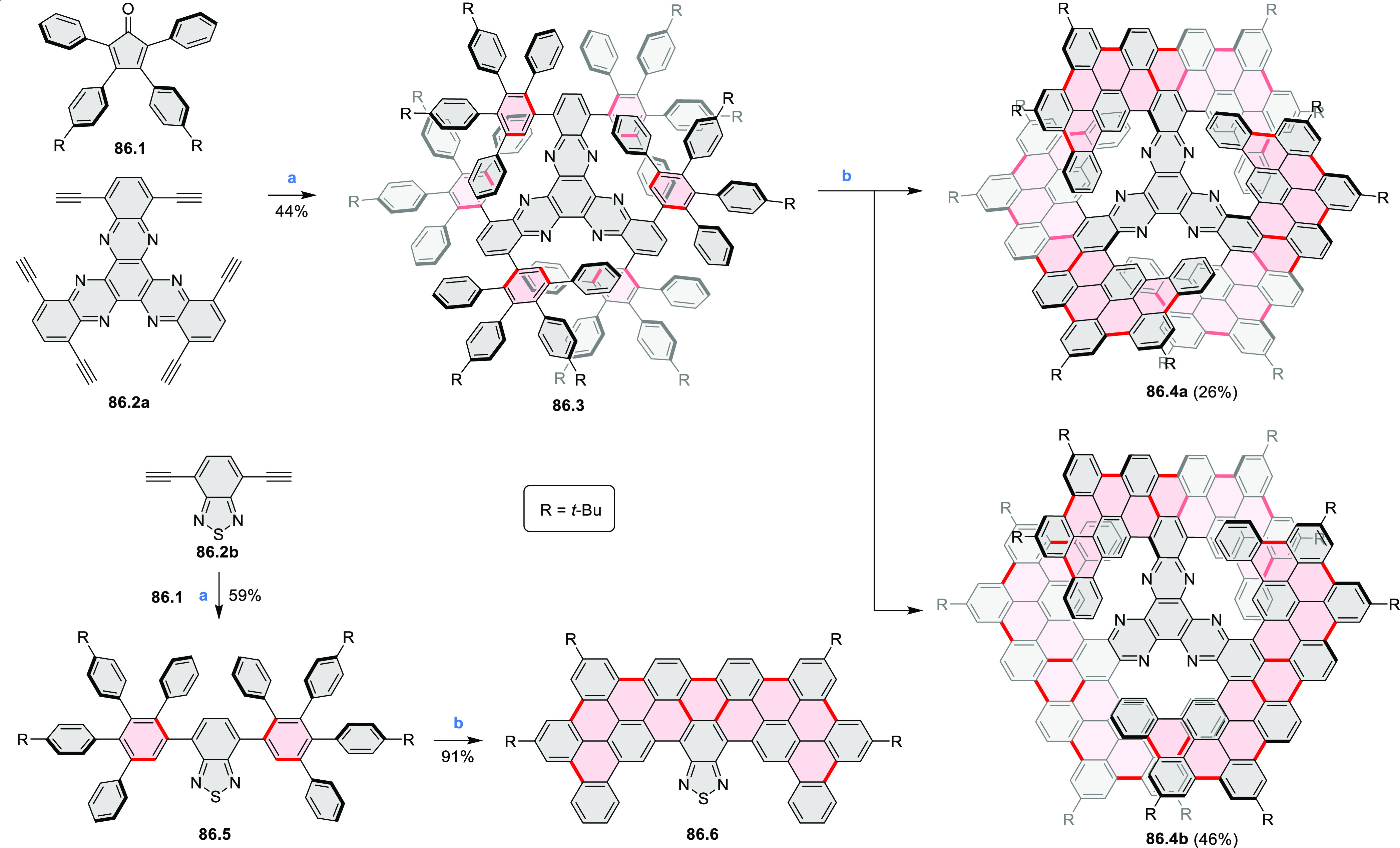
Synthesis of Twisted N-Doped Nanographenes Reagents and conditions: (a)^168^*o*-xylene, 80 °C, 30 min, then reflux, 12 h; (b) DDQ, TfOH, DCM (high dilution), 0 °C to rt, 4 h.

Inverse electrons demand Diels–Alder cycloaddition between cyclopentadienone **86.1** and alkynes **86.2a**,**b**, providing the sterically crowded products **86.3** and **86.5** (Scheme 86).^168^ Multiple fusions of the closely positioned phenyl groups in these molecules were then accomplished via oxidative coupling with DDQ and TfOH. These conditions were chosen as optimal, as incomplete dehydrogenation was observed when using FeCl_3_ in MeNO_2_. The fused product **86.6** was thus obtained from **86.5** in 91% yield. Compound **86.3** was converted into a mixture of diastereomers **86.4a**,**b** in 72% overall yield, which is an excellent result considering that 27 C–C bonds were formed in a single step. The diastereomers were successfully separated via silica gel chromatography; enantiomers of **86.4a** were also resolved by semipreparative chiral HPLC. While the planar compound **86.6** was highly insoluble, **86.4a**,**b** had good solubility in several organic solvents because their twisted structures prevented aggregation. The propeller-shaped *D*_3_-symmetric **86.4a** had a higher solubility than the *C*_2_-symmetric **86.4b**, which was observed to aggregate in DCM at micromolar concentrations. In the CD spectra of the pure enantiomers of **86.4a**, a strong Cotton effect was present with a peak Δε of 762 and −768 M^–1^ cm^–1^, respectively, which was attributed to the high rigidity of the extended chiral structure.

**Scheme 87:**
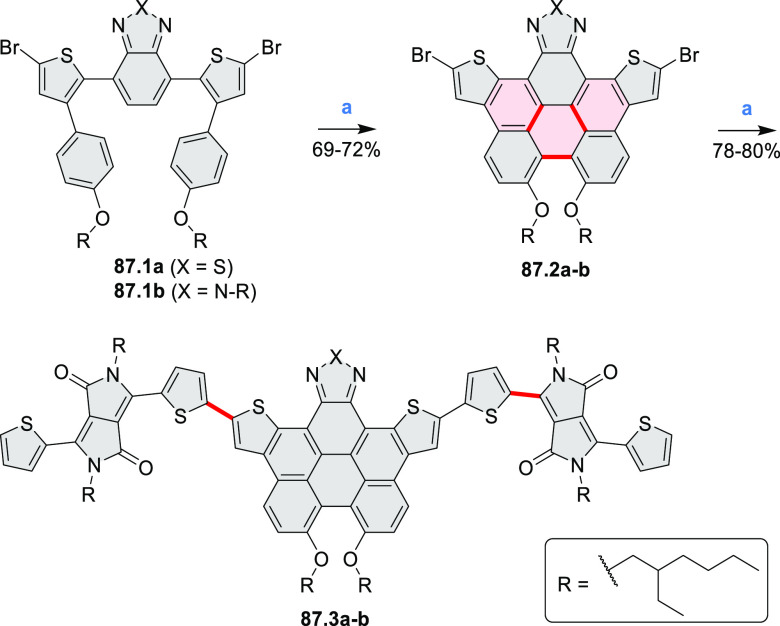
Synthesis of Thiadiazole- and Triazole-Fused Perylenes Reagents and conditions: (a)^169^ FeCl_3_, MeNO_2_, 0 °C, 3 h, 69–72%; (b) ArB(OH)_2_, Pd(PPh_3_)_4_, K_2_CO_3_, 2:1 toluene/H_2_O, 85 °C, overnight, 78–80%.

Oxidative coupling was successfully employed in the cyclization of compounds **87.1a**,**b**, which were readily assembled via cross-coupling reactions, into **87.2a**,**b** (Scheme 87).^169^ When FeCl_3_ was used as the oxidant, yields close to 70% were achieved for the formation of three C–C bonds. These cyclized products were then elaborated into acceptor-fused donor–acceptor triads through cross-coupling with diketopyrrolopyrrole units. The resulting products **87.3a**,**b** had an intense absorption band in the 500–800 nm range, which was absent in **87.2a**,**b**. The triazole-containing **87.3b** was more electron rich and had a slightly larger band gap than **87.3a**. Compound **87.3b** also had nearly two times higher extinction coefficients in the 550–650 nm range. OSCs built using **87.3a**,**b** blended with the PC_71_BM fullerene-based acceptor achieved PCEs of up to 2.88% with **87.3b**.

**Scheme 88:**
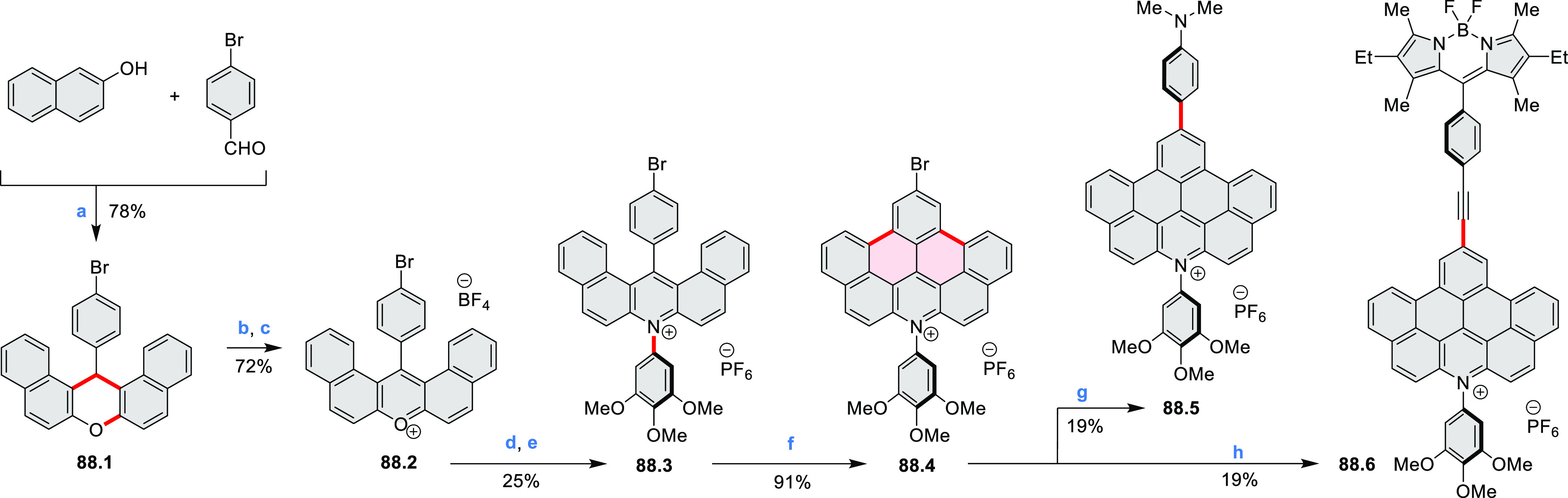
Synthesis of Expanded Acridinium Cations Reagents and conditions: (a)^170^ 1:25 conc. HCl_(aq)_/AcOH, reflux, 36 h, 78%; (b) PbO_2_, AcOH, reflux, 6.5 h, 77%; (c) HBF_4_, 1:2 AcOH/MeCN, rt, 24 h, 93%; (d) 3,4,5-trimethoxyaniline, NMP, molecular sieves, reflux, 8 h, 38%; (e) MnO_2_, HCl_(g)_, Ac_2_O, reflux, 1 h, 66%; (f) 1:1 MeCN/H_2_O, *hν* (sunlight), rt, 91%; (g) 4-(dimethylamino)phenylboronic acid, Pd(PPh_3_)_4_, Na_2_CO_3_, 5:8 DME/DMF, 95 °C, 24 h, 19%; (h)^171^ alkyne, Pd(PPh_3_)_4_, CuI, Et_3_N, DMF, 80 °C, 30 h, 19%.

π-Extended acridinium dyes with a perylenoid substructure were prepared, as shown in Scheme 88.^170^ Condensation of 2-naphthol with an aldehyde led to the dibenzoxanthene **88.1**. This compound was oxidized to the xanthenium salt **88.2**. The pyrylium oxygen atom in this compound could be displaced by an aniline, leading to the dibenzoacridinium **88.3**. This product readily cyclized to **88.4** upon exposure to sunlight without any catalyst or oxidant.^170^ A similar cyclization of **88.2** was also reported to be possible, allowing us to switch the order of steps. Finally, donor–acceptor compounds **88.5** and **88.6**(171) were obtained via cross-coupling reactions. Charge transfer upon photoexcitation was reported for these compounds with the expanded acridinium subunit acting as an acceptor.

Perylene diimides containing S_2_-annulated bays^108,110,111^ were obtained and studied along with their analogues containing five-membered thiophene annulations and are discussed in Section 3.2 (Schemes 61 and 62).

**Scheme 89:**
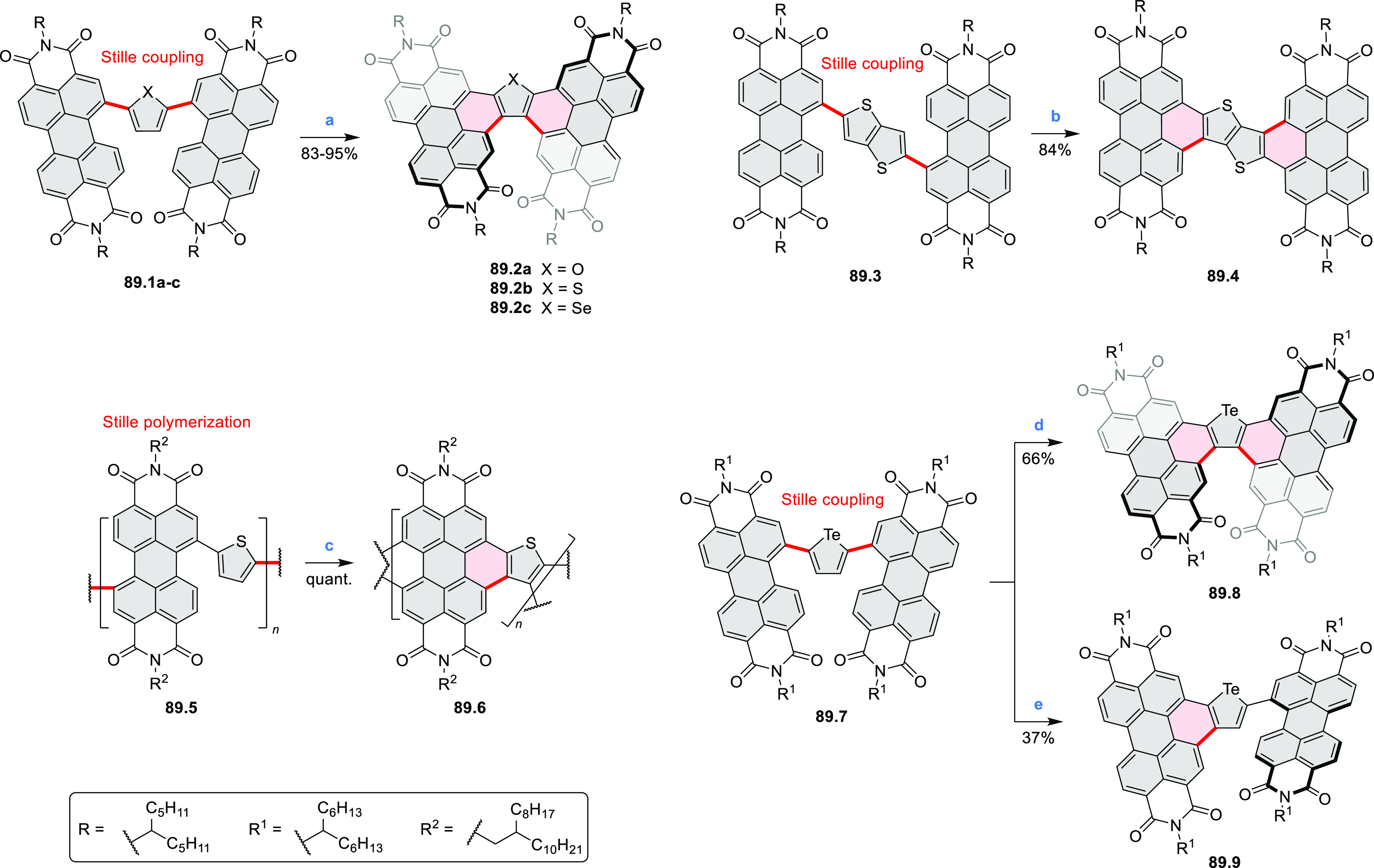
Synthesis of Perylene Diimides Fused to Five-Membered Heterocycles Reagents and conditions: (a)^172^ FeCl_3_, MeNO_2_, reflux, 3 h; (b)^175^ I_2_, toluene, *hν* (incandescent lamp), air, rt, 24 h, 84%; (c)^174^ I_2_, DCM, *hν* (xenon lamp), rt, 10 h, quant.; (d)^173^ FeCl_3_, MeNO_2_/chlorobenzene, 130 °C, 3 days, 66%; (e) FeCl_3_, MeNO_2_/toluene, 90 °C, 8 h, 37%.

Fusion of two PDI units to a common heterocyclic ring was reported by Jen and co-workers (Scheme 89).^172^ Oxidation with FeCl_3_ was used to effect double bay cyclization, to produce products **89.2a**–**c** with a furan, thiophene, or selenophene core, respectively. A tellurophene analogue **89.8** and its singly fused analogue **89.9** could also be prepared using the same method, with a selectivity dependent on reaction conditions.^173^ Alternatively, bay cyclizations could be achieved by visible or near-UV irradiation in the presence of I_2_. The latter approach was used for the postpolymerization modification to afford **89.6**,^174^ as well as to prepare **89.4** with a thienothiophene core.^175^ Compound **89.2b** was also obtained by photocyclization in 88% yield, comparable to that achieved with FeCl_3_ oxidation. The resulting compounds **89.2a**–**c** and **89.8** adopt a twisted structure, where the angle between the two PDI planes is predicted to increase with the bulk of the central heteroatom.^172,173^ Similar twist angles were reported for the DFT-optimized structures of **89.2b** (23°)^172^ and the polymer **89.6** (24.4°).^174^ The fused compounds had higher LUMO levels and larger band gaps than their heterocycle-bridged precursors, which corresponded to shorter wavelengths of absorption onset. Similar HOMO and LUMO energies were reported for compounds with different heterocyclic cores (**89.2a**–**c** and **89.8**). Ring fusion was generally observed to increase electron mobility in donor–acceptor blend films, which translated to the better performance of OSC devices. PCE significantly improved with ring fusion in the tellurophene series (**89.7**: 1.45%, **89.9**: 3.26%, **89.8**: 7.52%), where **89.8** notably outperformed the fullerene-based acceptor PC_61_BM.^173^

**Chart 9:**
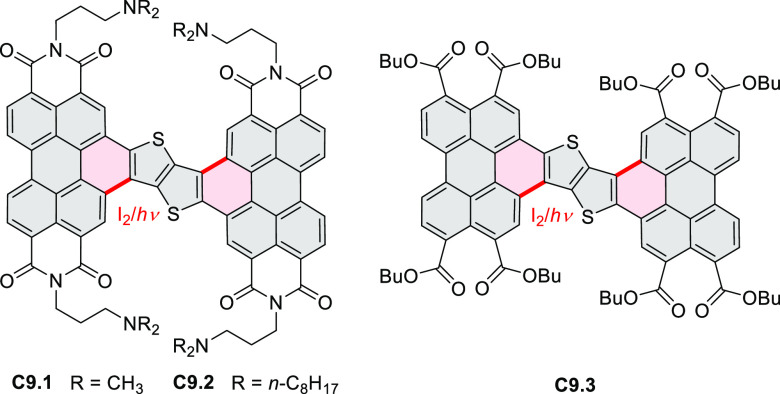
Thienothiophene-Fused Perylene Diimides and Perylene Tetraesters

Irradiation in the presence of oxygen with catalytic I_2_ provided access to further examples of thienothiophene-fused dimeric PDIs **C9.1**–**2** and a dimeric perylene tetraester **C9.3** (Chart 9).^176^ The planarity of these molecules facilitated the formation of self-assembled monolayers on the HOPG surface. **C9.1** formed rows of molecules with contacts along the unsubstituted perylene edges. Its short alkylamine chains occupied the space between the rows. Longer chains in **C9.2** entirely occupied the areas of contact between adjacent molecules arranged in parallel rows with greater intermolecular distances than in **C9.1**. The dimeric perylene tetraester **C9.3** formed a honeycomb pattern with 4 nm voids surrounded by six molecules.

**Chart 10:**
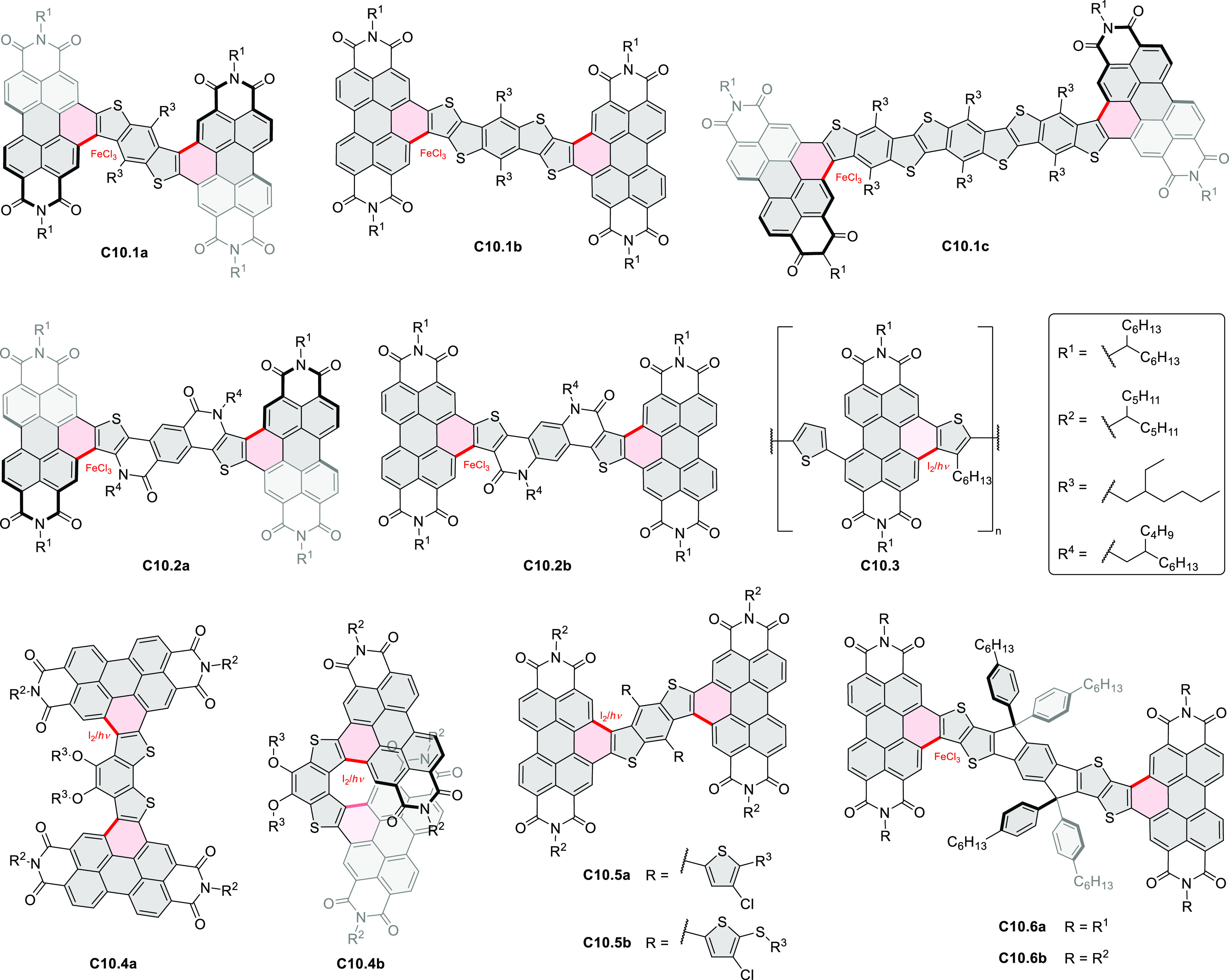
Further Examples of Dimeric Thiophene-Fused Perylene Diimides

Methods of introducing ring fusion summarized in Scheme 89 were used to synthesize several dimeric fused PDIs with extended thiophene-based cores (Chart 10). In most of these examples, the [*ghi*] cyclization was performed in the last synthetic step. The only exception was the polymer **C10.3**, where a fused monomer was prepared prior to polymerization. The respective method of annulation (oxidative cyclization with FeCl_3_ or irradiation in the presence of catalytic I_2_) is indicated for each molecule. In the **C10.1a**–**c** series, compound **C10.1b** was predicted by DFT to be nearly planar.^177^ In contrast, **C10.1a** and **C10.1c** were expected to be twisted because of a steric clash between the PDIs and core-bound alkyl groups. Extension of the electron-rich core resulted in increased HOMO and LUMO energies and a decreased band gap. In **C10.1b** and **C10.1c**, an absorption band at 600–650 nm was present, which was assigned to intramolecular charge transfer, highlighting their acceptor–donor–acceptor architecture. Core extension also resulted in weaker and red-shifted emission bands and in a strong increase of a two-photon absorption cross section. OSCs prepared with **C10.1a**–**c** blended with PTB7-Th displayed higher *V*_OC_ and PCE values than their nonfused analogues.^178^ A slight increase of *V*_OC_ with core extension was also observed. The highest PCE of 6.06% was reached for the planar compound **C10.1b**.

Similarly, the optimized structure of **C10.2a** was twisted, while its isomer **C10.2b** was predicted to be planar.^179^**C10.2a** had a strongly red-shifted low-energy absorption band compared to **C10.2b**. Its emission was also red-shifted and much weaker (λ_em_ = 647 nm, Φ_F_ = 0.042), in contrast to the bright emission of the planar isomer **C10.2b** (λ_em_ = 547 nm, Φ_F_ = 0.52). A slightly better performance in OSCs was observed for **C10.2b** (PCE = 4.97%) compared to **C10.2a** (PCE = 4.79%). Significant differences in geometry were also evident between the isomeric compounds **C10.4a** and **C10.4b**.^180^ The more sterically congested **C10.4b** had partially overlapping PDI units with a 42.6° dihedral angle between them. Conversely, a smaller tilt of 12.7° was predicted for **C10.4a**, which displayed a higher band gap and lower HOMO energy than **C10.4b**. OSCs built using blends of these compounds with PTB7-Th and a 1-chloronaphthalene additive achieved PCEs of up to 4.34% for **C10.4b** and 2.89% for **C10.4a**. The main improvement observed for the twisted isomer **C10.4b** was the short-circuit current (*J*_SC_ = 10.27 mA cm^–2^ and *J*_SC_ = 6.79 mA cm^–2^ for **C10.4a**). Compounds **C10.5a**,**b** had planar conjugated backbones, but their pendant thiophene groups decreased the tendency to aggregate.^181^**C10.5b** contained additional thioether functions, which resulted in a higher HOMO level and a decreased band gap as determined by cyclic voltammetry. Absorption spectra of the two analogues were broadly similar. OSCs using these compounds as acceptors had high open-circuit voltage values of up to 1.14 V, with a better overall performance recorded for **C10.5a**.

Polymer **C10.3** proved to be a more efficient acceptor in OSCs compared to its analogue with nonfused PDI units as well as the one in which both PDI bays were fused.^182^ Open-circuit voltages of up to 1.03 V were obtained with the PBDB-T donor, while the highest PCE (4.60%) was achieved with PTB7-Th. Compounds **C10.6a**,**b** were reported in OSC applications by several groups.^183,184^ A good photovoltaic performance was achieved with a PCE of up to 7.33%, a *V*_OC_ close to 1 V, and a high *J*_SC_ of 13.24 mA cm^–2^. A later study of the photophysical properties of **C10.6b** in blends with PTB7-Th revealed delayed photoluminescence indicative of nongeminate charge recombination.^184^ This effect was proposed to be an indicator for fast screening of photovoltaic blends.

**Scheme 90:**
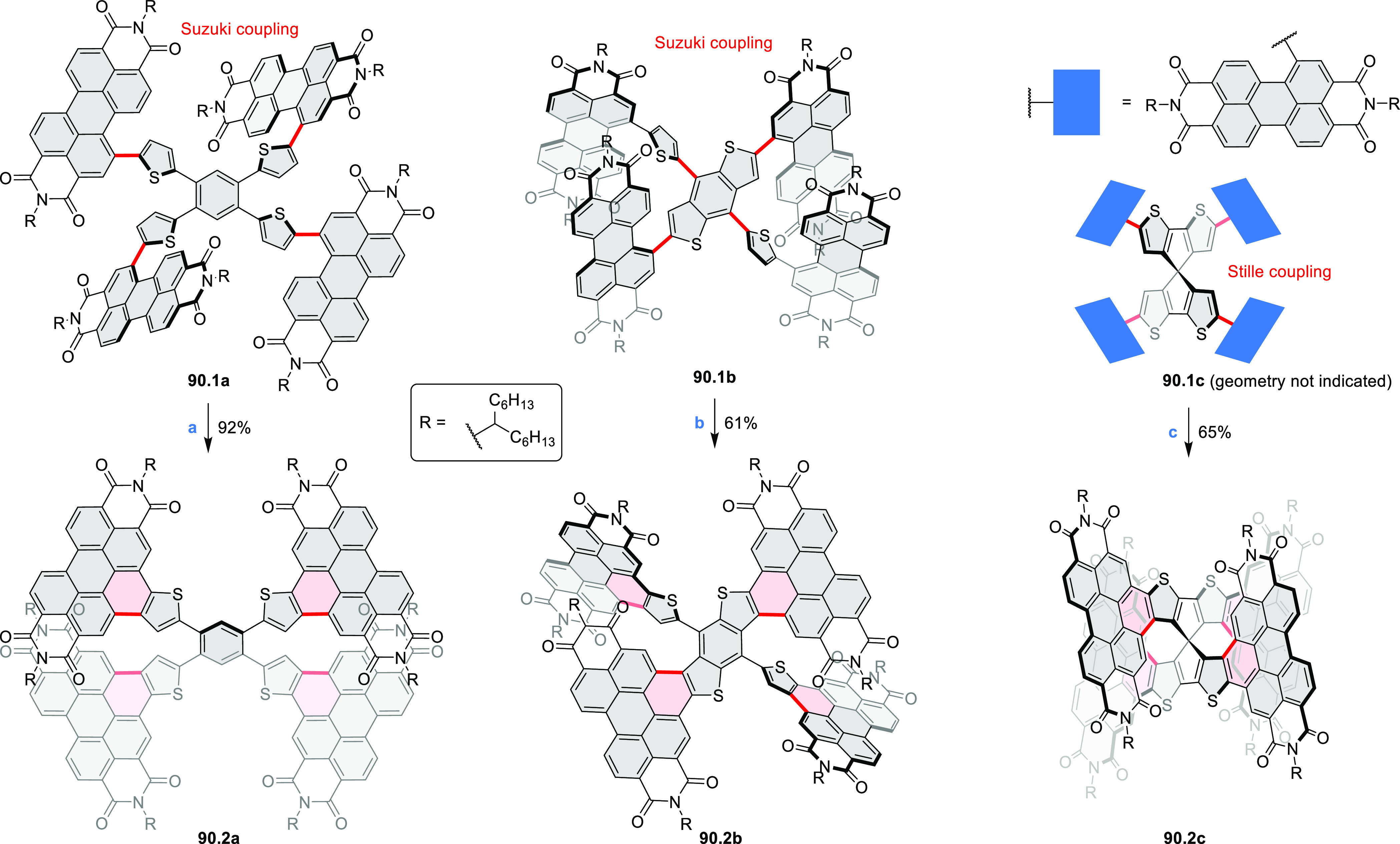
Synthesis of Tetrameric Thiophene-Fused PDIs Reagents and conditions: (a)^185^ FeCl_3_, 25:1 toluene/MeNO_2_, rt, 2 h, 92%; (b)^186^ FeCl_3_, 2:1 toluene/MeNO_2_, 0 °C to rt, 5 h, 61%; (c)^187^ FeCl_3_, 2:1 toluene/MeNO_2_, 110 °C, 5 h, 65%.

The above method of preparing thiophene-fused PDIs was further extended to sterically congested targets with four PDIs connected to thiophene-based cores (Scheme 90).^185−187^ The requisite precursors, **90.1a**–**c**, were prepared via cross-coupling reactions. Conformational freedom in this series of compounds decreases from **90.1a**, through **90.1b** with a partially fused core, to **90.1c**, in which a fully rigid spirocyclic core was introduced. Fused final products **90.2a**–**c** were then obtained upon cyclodehydrogenation with FeCl_3_, which led to significant changes in molecular geometry. While in **90.1a** adjacent PDI units were nearly orthogonal, compound **90.2a** adopted a “double-decker” structure, characterized by staggered though largely coplanar PDIs and a tilted central benzene ring. In **90.2b**, two PDIs were integrated into the fully fused section of the molecule, to which the remaining fused PDIs were orthogonal. Compound **90.2c** had a very rigid x-shaped structure with two orthogonal fully conjugated π-systems.

Compared to **90.1a**, its fused analogue **90.2a** had higher HOMO and LUMO levels, decreased band gap, and higher extinction coefficients across the entire visible spectrum.^185^ OSCs containing blends of **90.2a** with a P3TEA donor polymer achieved a remarkably high PCE of 10.37% and open-circuit voltage of 1.13 V, significantly improving upon the performance of the PC_71_BM fullerene-based acceptor (PCE = 7.71%, *V*_OC_ = 0.90 V). A much lower performance (PCE = 6.67%, *V*_OC_ = 1.05 V) was also recorded for the nonfused precursor **90.1a**. Using the finely tuned donor material P3TAE^188^ enabled further increase of *V*_OC_ to 1.20 V, albeit with a lower PCE of 7.83%. Different changes in energy levels were observed between **90.1b** and **90.2b**, where the latter had a greater band gap.^186^ This resulted in a shorter wavelength of absorption onset but was compensated by higher absorption in the 300–500 nm range. As in the previous case, the fused compound **90.2b** produced more efficient OSCs in blends with the PTB7-Th donor (**90.1b**: PCE 5.16%, *V*_OC_ 0.85 V; **90.2b**: PCE 7.56%, *V*_OC_ 0.92 V). In contrast to the other fused tetrameric compounds, **90.2c** had a much lower band gap of 1.87 eV, compared to 2.16 eV in **90.2a** and 2.46 eV in **90.2b**.^187^ Combined with PTB7-Th in OSCs, **90.2c** achieved a PCE up to 8.75%, with a *V*_OC_ of 0.90 V and a very high short-circuit current (*J*_SC_) of 16.6 mA cm^–2^.

**Scheme 91:**
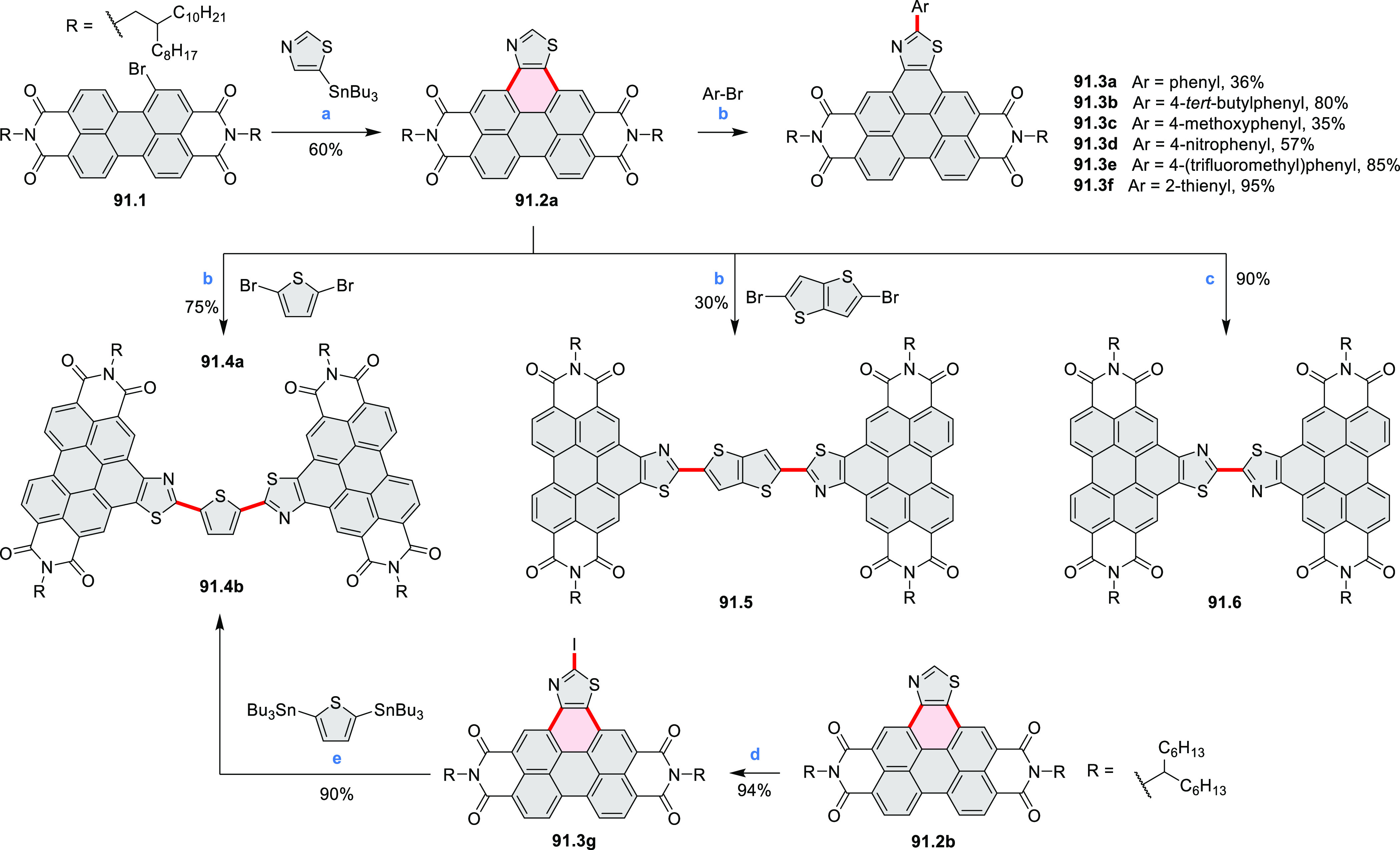
Synthesis and C–H Arylation of Thiazole-Annulated PDI Reagents and conditions: (a)^189^ Pd(PPh_3_)_4_, CuI, 90 °C, 4 h, then I_2_, *hν* (254 nm), rt, 3 h; (b) bis(chloro-di-*tert*-butylphosphine)palladium dichloride, Cu(Xantphos)I, K_2_CO_3_, DMF, 135 °C, overnight; (c) Ag_2_O, Cu(Xantphos)I, K_2_CO_3_, DMF, 135 °C, overnight, 90%; (d)^190^ KO*t*-Bu, toluene, rt, 30 min, 94%; (e) Pd(PPh_3_)_4_, toluene, 110 °C, overnight, 90%.

A thiazole-fused perylene diimide was prepared via Stille coupling of the brominated PDI **91.1** followed by UV irradiation of the Stille reaction mixture in the presence of I_2_ with no prior purification or workup (Scheme 91).^189^ The product **91.2a** was further functionalized through C–H arylation of its thiazole ring under Pd/Cu-catalyzed conditions. A particularly high-yielding cross-coupling occurred with 2-bromothiophene to provide **91.3f**. This methodology was extended to the synthesis of bridged dimers, providing the thiophene-bridged **91.4a** in 75% yield and thienothiophene-bridged **91.5** with a lower efficiency. The synthesis of analogues with dithienothiophene and 2,2′-bithiophene cores was not successful. Compound **91.2a** also underwent oxidative homocoupling in the presence of Ag_2_O and Cu(I) catalyst to form **91.6** in 90% yield. Additionally, a free radical iodination initiated by KO*t*-Bu with pentafluoroiodobenzene as the source of iodine was developed.^190^ This method was used to make **91.3 g**, which was elaborated via Stille coupling into the thiophene-bridged dimer **91.4b**.

**Scheme 92:**
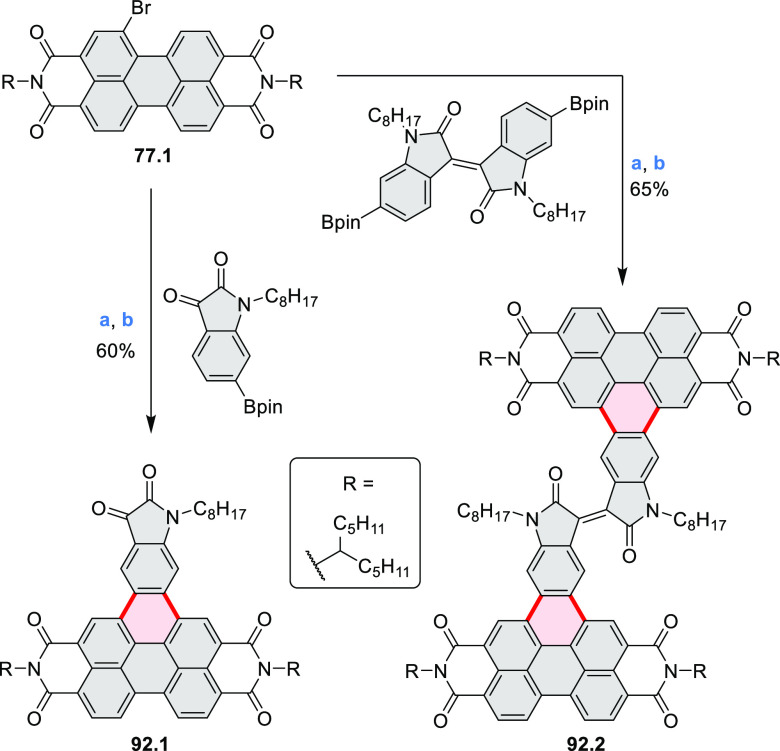
Synthesis of Isatin- and Isoindigo-Fused Perylene Diimides Reagents and conditions: (a)^191^ Pd_2_(dba)_3_, P(*t*-Bu)_3_·HBF_4_, K_3_PO_4_, THF, 80 °C, 12 h; (b) I_2_, CHCl_3_, air, *hν* (sunlight), 25 °C, 24 h; (c) I_2_, toluene, air, *hν* (blue LED), 90 °C, 12 h (flow reactor).

Photocyclization in the presence of I_2_ was also used to prepare isatin- and isoindigo-fused PDIs (Scheme 92).^191^ Exposure to sunlight was used in the synthesis of **92.1**, whereas the preparation of **92.2** was effected under blue (450 nm) LED irradiation in a flow reactor. In contrast to the heterocycle-fused compounds in Scheme 89, oxidation with FeCl_3_ or DDQ was not successful in the case of **92.2**. Compound **92.1** was further elaborated into dimeric structures via condensation reactions with the isatin ketone group. The resulting compounds were used to build bottom-gate/bottom-contact OFET devices that displayed on/off current ratios up to 2 × 10^6^.

### 3.4 [*cd*]Heteroannulated Perylenoids

**Scheme 93:**
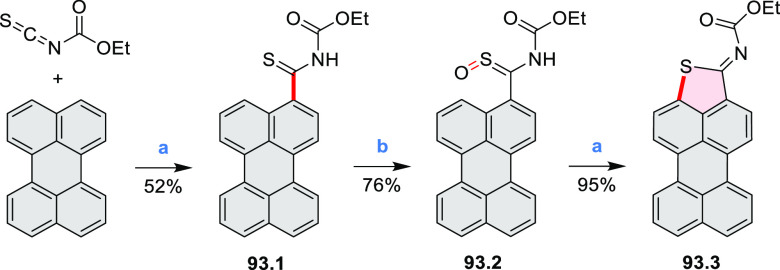
Thiophene Imine-Fused Perylene Reagents and conditions: (a)^192^ TfOH, DCM, rt; (b) oxone, 1:1 MeCN/H_2_O, rt, 1 h.

Perylene was selectively derivatized in a 3-position in an S_E_Ar reaction with ethoxycarbonyl isothiocyanate in the presence of triflic acid to provide **93.1** (Scheme 93, see Scheme 142, Section 4.4, for related fused pyrenes).^192^ Oxidation to thioamide *S-*oxide **93.2** and subsequent cyclization under acidic conditions provided **93.3** in very high yield (95%). The thiophene-imine-fused perylene **93.3** was strongly emissive in DCM solution, with the emission maximum at 606 nm and a quantum yield of 54%.

**Scheme 94:**
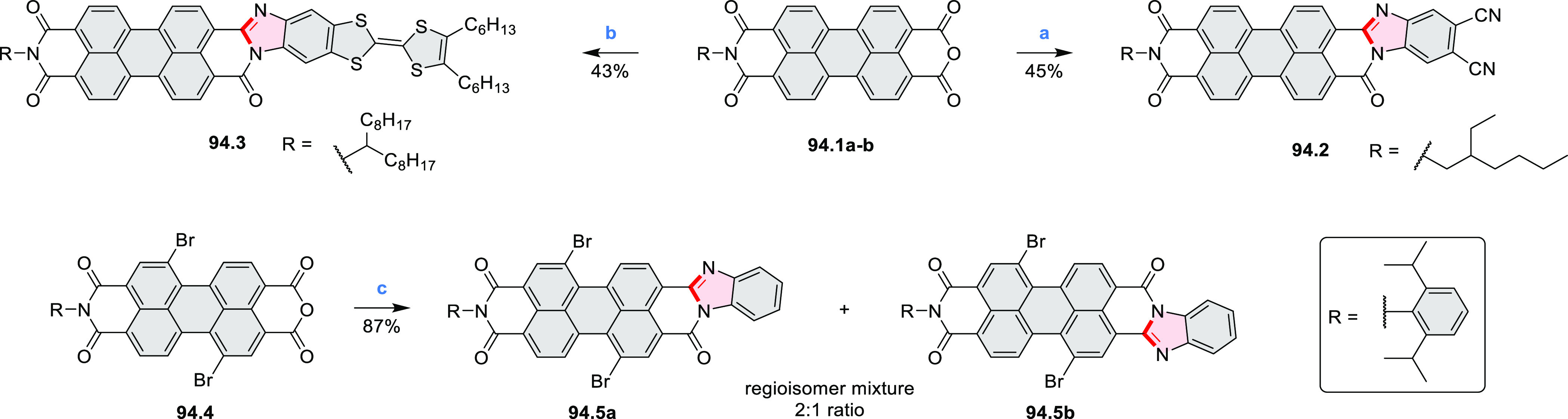
Synthesis of Benzimidazole-Fused Peryleneimides Reagents and conditions: (a)^193^ 4,5-diaminophthalonitrile, Zn(OAc)_2_, quinoline, 150 °C, 18 h, 45%; (b)^194^ 1,2-diaminoarene, imidazole, pyridine, 130 °C, 23 h, 43%; (c)^195^*o*-phenylenediamine, propionic acid, reflux, 3 h, 87%.

Condensation of perylenetetracarboxylic acid anhydride imides with 1,2-diaminoarenes leads to benzimidazole-fused peryleneimides. Yuksel et al. reported the dinitrile derivative **94.2** (Scheme 94, see also Scheme 130, Section 4.2).^193^ The compound was fluorescent, with emission peaks at 535, 582, and 629 nm in CHCl_3_. A peryleneimide–tetrathiafulvalene conjugate with a fused benzimidazole linker **94.3** was prepared by Guldi and Liu et al. through condensation in pyridine in the presence of imidazole.^194^ Compound **94.3** underwent binding to graphene sheets and was used to improve the ultrasonic exfoliation of graphite, providing dispersions with higher optical density and greater stability. Signs of graphene p-doping were present, reflecting the electron-deficient character of peryleneimide. Grozema and Jager et al. reported the condensation between **94.4** and 1,2-diaminobenzene in propionic acid.^195^ A significantly higher yield of 87% was obtained, implying that Brønsted acid catalysis may be more effective for this reaction. The product was a 2:1 mixture of regioisomers **94.5a**,**b**.

**Scheme 95:**
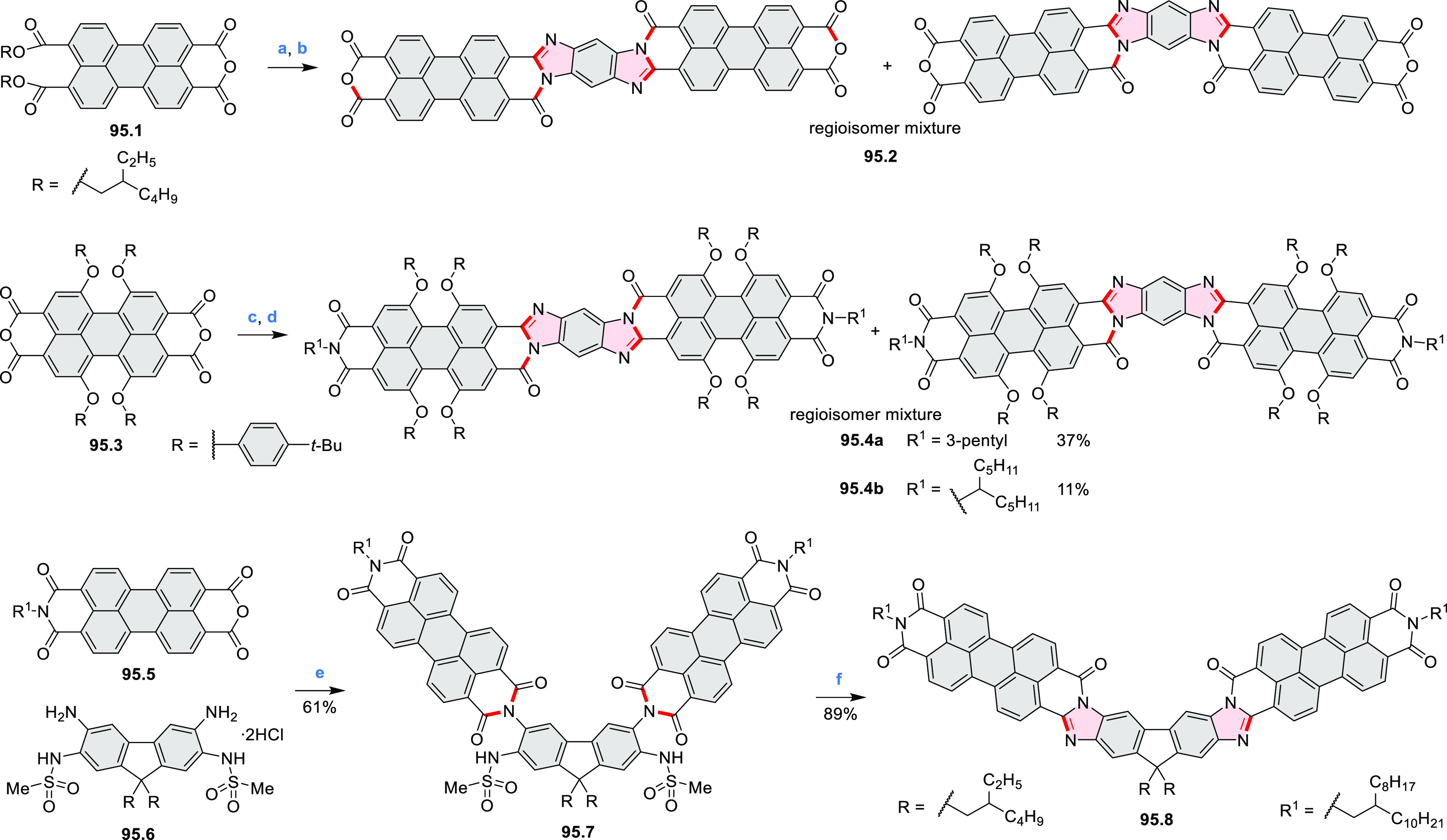
Synthesis of Dimeric Benzimidazole-Fused Perylenes Reagents and conditions: (a)^196^ 1,2,4,5-tetraaminobenzene tetrahydrochloride, pyridine, reflux, overnight, 41%; (b) TsOH·H_2_O, toluene, 110 °C, 24 h, 95%; (c)^197^ 1,2,4,5-tetraaminobenzene, ZnCl_2_, quinoline, 170 °C, 18 h, 21%; (d) R^1^NH_2_, Zn(OAc)_2_, imidazole, 140 °C, 2 h; (e)^198^ Zn(OAc)_2_, quinoline, 145 °C, 14 h, 61%; (f) 1:1 H_2_SO_4_/propionic acid, 150 °C, 5 h, 89%.

Condensations of 1,2,4,5-benzenetetramine with perylene anhydrides in 1:2 stoichiometry provide access to benzodiimidazole-fused PDI dyads (Scheme 95). This approach was applicable to monoanhydride **95.1** as well as the dianhydride **95.3**, with subsequent steps converting the diester function into anhydrides to provide **95.2**(196) and anhydrides into imides to produce **95.4a**,**b**.^197^ Compound **95.2** was prepared along with the naphthalene-based analogue **130.2** (Scheme 130, Section 4.2). In both condensations with benzenetetramine, mixtures of regioisomers were obtained, which were not separated.

A single regioisomer **95.8** could however be prepared via a stepwise condensation as reported by Schönamsgruber and Hirsch.^198^ The fluorene-2,3,6,7-tetramine derivative **95.6** with the amino groups on C2 and C7 protected as sulfonamides was used as the key starting material. Initial condensation involving the free amino groups on **95.6** formed the diimide **95.7**. Further condensation could proceed upon cleavage of sulfonamide groups under acidic conditions, providing **95.8**. Aside from enabling regioselectivity, this method had a higher overall yield than direct condensations with 1,2,4,5-benzenetetramine. Compound **95.8** displayed an absorption maximum at 614 nm and a NIR emission peak at 708 nm, strongly red-shifted relative to monomeric perylene diimides.^198^ A strong NIR two-photon absorption of up to 1110 GM was measured in the 1200–1400 nm range, suggesting an application in optical power-limiting materials. In the case of **95.4a**, an absorption peak at 650 nm was observed, which was further red-shifted to 773 nm upon protonation of its basic imidazole rings.^197^ Its broad NIR emission with maxima at 744 and 915 nm was significantly quenched by protonation.

**Scheme 96:**
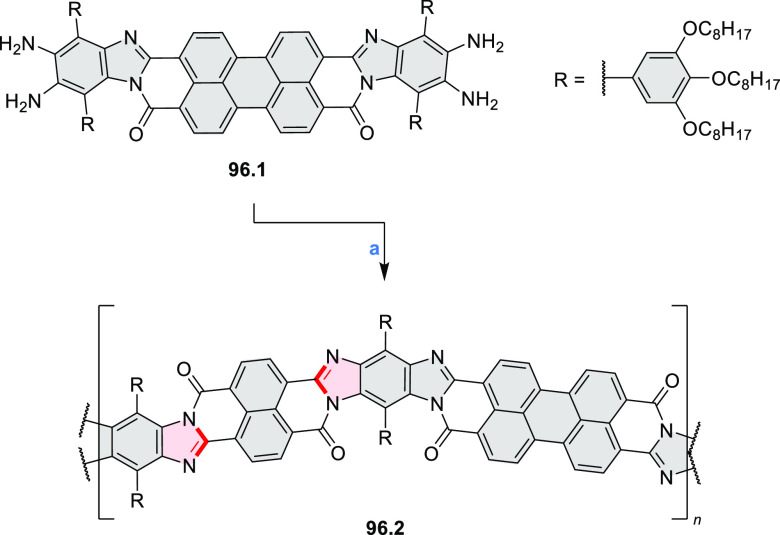
Polycondensation of **96.1** Reagents and conditions: (a)^199^ naphthalene-1,4,5,8-tetracarboxylic acid dianhydride, *m*-cresol, 5 h at 80 °C then 32 h at 200 °C.

Condensation of the air-stable tetraamine **96.1** with naphthalene-1,4,5,8-tetracarboxylic acid dianhydride was used to obtain the conjugated polymer **96.2** (Scheme 96).^199^ The same approach also gave access to an NDI analogue **126.4** (Scheme 126, Section 4.2). The absorption spectrum of **96.2** in DCM solution covers the entire visible region and extends into the NIR, with two intense peaks at 697 and 758 nm and at 711 and 803 nm in the thin film. In a blend with the P3HT donor polymer, **96.2** was used to construct bulk heterojunction photodetectors, showing high detectivity in the NIR range (1.6 × 10^10^ Jones at 800 nm).

**Scheme 97:**
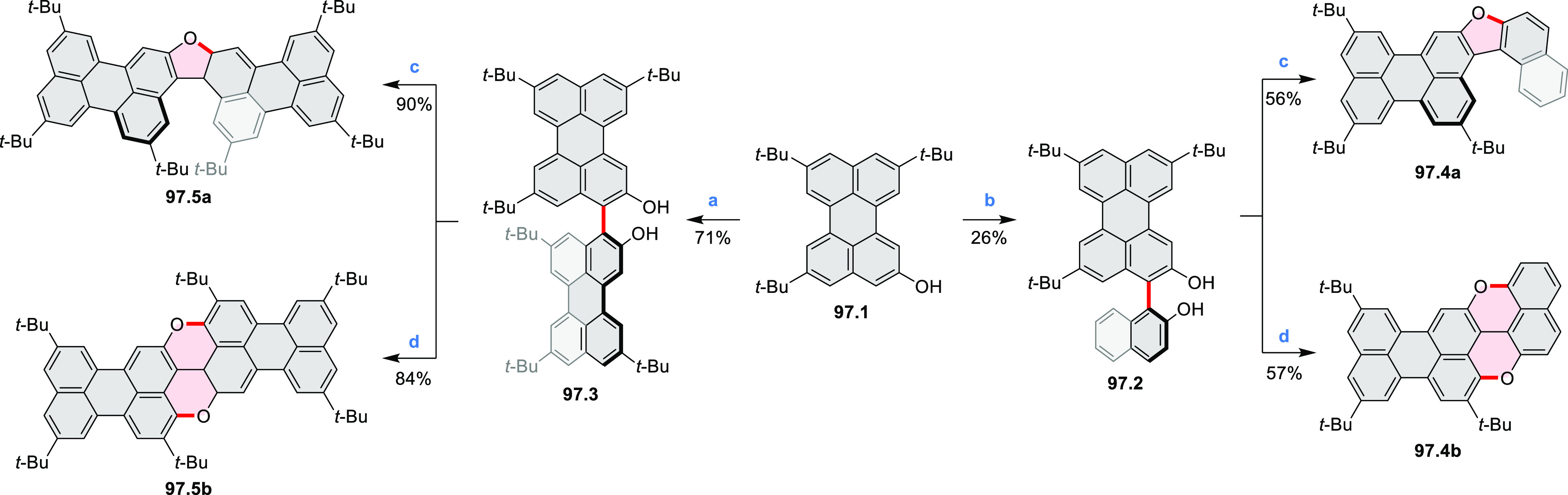
Synthesis of Thiophene-Fused Perylenoids Through Dehydrogenative Photocyclizations Reagents and conditions: (a) [Cu(OH)(Cl)(TMEDA)], DCM, air, rt, 1 h, 71%; (b) 2-naphthol, [Cu(OH)(Cl)(TMEDA)], DCM, air, rt, 2 h, 26%; (c) TsOH, toluene, reflux, 4 h; (d) CuI, PivOH, DMSO, 140 °C, 2 h.^200^

Bonifazi and co-workers reported syntheses of furan- and pyranopyran-fused perylenes from common precursors.^200^ The Cu-catalyzed aerobic dimerization of perylenol **97.1** provided **97.3**, while the cross-coupling of **97.1** with 2-naphthol gave **97.2** along with the homocoupled products **97.3** and binaphthol (Scheme 97). Upon treatment with TsOH, compounds **97.2**–**3** were condensed into the furan-fused derivatives **97.4a** and **97.5a**, respectively. It was also possible to cyclize **97.2** and **97.3** into the corresponding pyranopyran-fused derivatives **97.4b** and **97.5b**. This oxidative etherification was effected in the presence of CuI and pivalic acid in DMSO, according to the methodology previously developed in the group (see Scheme 50).^82^ The furan-fused compounds had nonplanar structures with interplanar angles close to 17°, while the pyranopyran-fused analogues were planar. All compounds **97.1**–**5** were strongly emissive, with quantum yields of 50–88%. The wavelengths of absorption and emission peaks increased on going from the nonfused precursors **97.2**–**3**, through furan-fused, to pyran-fused compounds, a change attributed mainly to higher HOMO energies caused by O-annulation. Additionally, spectral features of biperylenol-derived systems were red-shifted relative to the respective perylenol–naphthol analogues. Tuning of the emission wavelength in the range between 463 nm in **97.1** and 649 nm in **97.5b** was thus demonstrated.

**Scheme 98:**
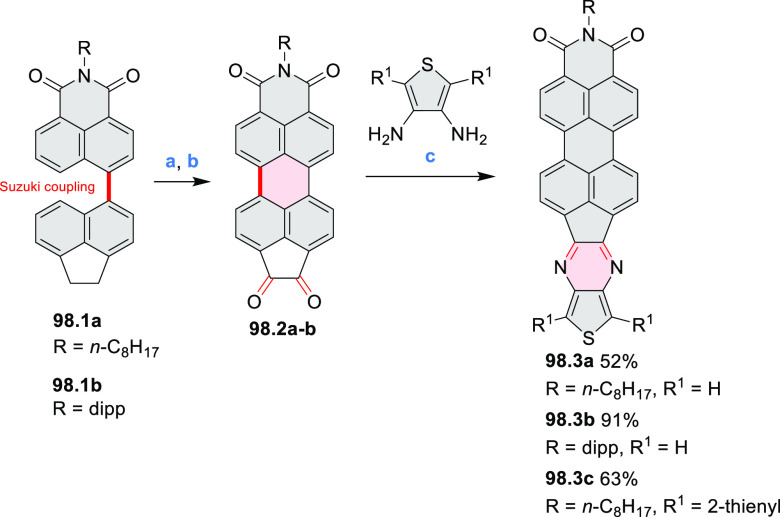
Synthesis of Pyridazine-Bridged Peryleneimide-thiophene Assemblies Reagents and conditions: (a)^201^ AlCl_3_, chlorobenzene, reflux, 6 h, 80% from **98.1a** or 20% from **98.1b**; (b) benzeneseleninic anhydride, chlorobenzene, 130 °C, overnight, 78–95%; (c) TsOH, CHCl_3_, 50 °C, overnight.

Peryleneimides linked to a thiophene or terthiophene through a fused pyrazine ring were prepared in a condensation reaction from acenaphthene-derived diketones **98.2a**,**b** (Scheme 98).^201^ The terthiophene-containing **98.3c** displayed panchromatic absorption with a broad low-energy band peaking at 668 nm and extending up to ca. 900 nm. This band, which was absent in **98.3a**,**b**, was assigned to an intramolecular charge transfer absorption. Compounds **98.3a**–**c** were studied as n-type semiconductors for OFET devices, with **98.3a** showing an electron mobility (μ_e_) of up to 1.5 × 10^–4^ cm^2^ V^–1^ s^–1^.

**Scheme 99:**
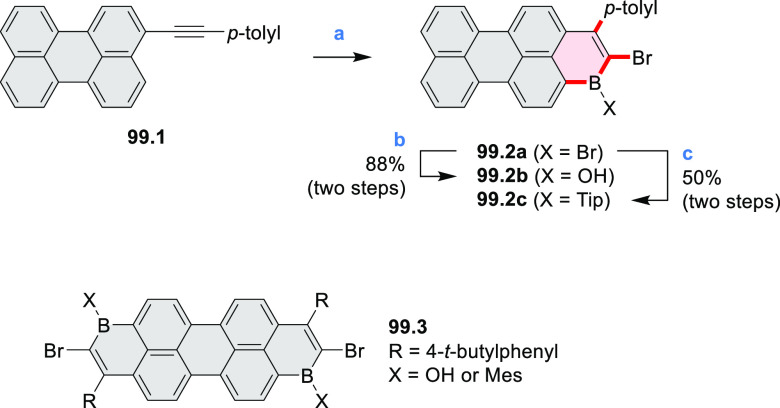
*peri*-Fused Perylenoids Bearing One or Two Boron Atoms Reagents and conditions: (a)^202^ BBr_3_, 2,4,6-tri-*tert*-butylpyridine, *o*-dichlorobenzene, heptane, rt, 24 h; (b) DCM, air, 1 h, then aq. HCl; (c) TipMgBr, *o*-dichlorobenzene, 48 h.

In 2020, Ingleson and Zysman-Colman et al. reported a bromoboration–electrophilic borylation sequence for the preparation of the boron-containing perylenoids **99.2**–**3** (Scheme 99).^202^ For instance, 3-(*p*-tolylethynyl)perylene (**99.1**) was treated with BBr_3_ and 2,4,6-tri-*tert*-butylpyridine to bring about the 1,1-bromoboration of the alkyne unit, followed by borylative cyclization with C4 of the perylene core. When the resulting compound **99.2a** was stirred in untreated DCM under air, the B–Br bond was hydrolyzed to give **99.2b** in 88% yield. Alternatively, **99.2a** was reacted with 2,4,6-triisopropylphenylmagnesium bromide (TipMgBr) to give **99.2c** in 50% yield. Similarly, the doubly *peri*-fused perylenoids **99.3** were obtained from the corresponding 3,9-dialkynyl-substituted perylene precursor.

### 3.5 *ortho*-Heteroannulated Perylenoids

**Scheme 100:**
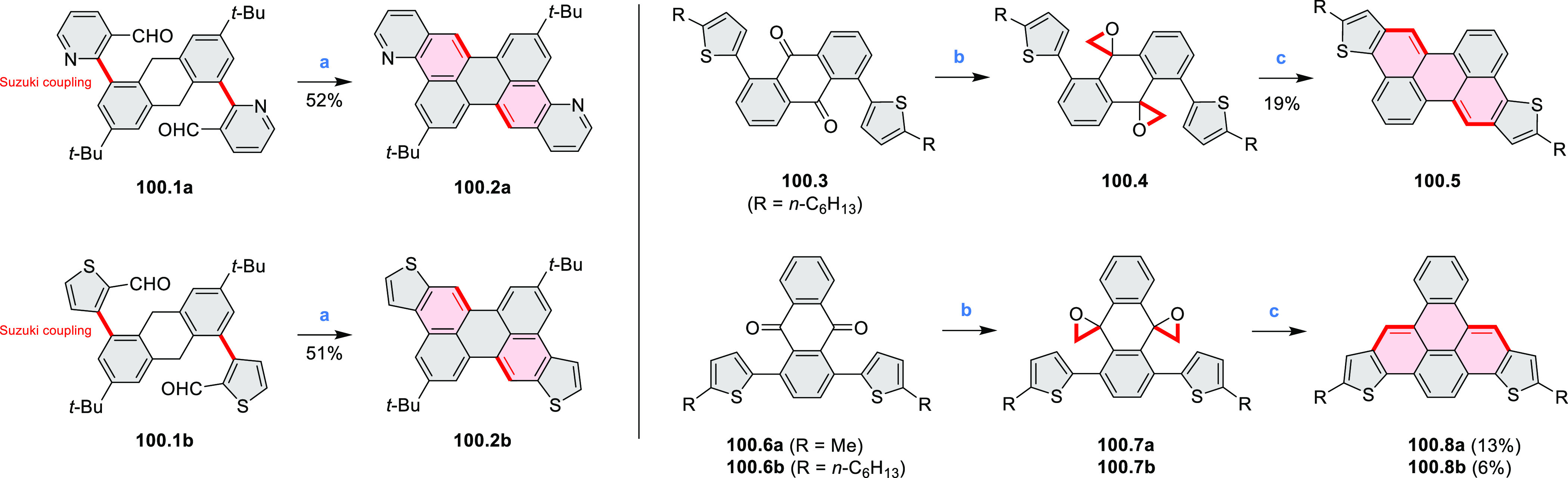
Syntheses of Pyridine- and Thiophene-Fused Perylenes Reagents and conditions: (a)^203^ KO*t*-Bu, THF, 60 °C, 16 h; (b)^204^ Me_3_SI, sodium hydride, DMSO/THF, 50 °C, 1 h; (c) SnCl_2_, 1,2-dichloroethane, 60 °C, 12 h.

Mastalerz et al. reported a base-promoted double cyclization of dihydroanthracenes bearing *o*-formylaryl substituents to yield the corresponding doubly fused perylenes, including the pyridine- and thiophene-fused examples **100.2a**,**b** (Scheme 100).^203^ Several nonheterocyclic analogues were reported along with the compounds shown here, with yields of 45–79%. The transformation was accomplished by treatment of **100.1a**,**b** with KO*t*-Bu in THF solution, giving **100.2a**,**b** in satisfactory yields. The products were soluble in organic solvents despite the large and planar π-surfaces. Compound **100.2a** was strongly luminescent (λ_em_ = 446 nm, Φ = 67%), while **100.2b** had a weaker and red-shifted emission (λ_em_ = 477 nm, Φ = 24%).

Related thieno-fused perylene structures were accessed via a procedure consisting of a Corey–Chaykovsky reaction followed by dehydrative cycloaromatization that was developed by Kakiuchi et al. (Scheme 100).^204^ The reaction of anthraquinone **100.3** with trimethylsulfonium iodide and sodium hydride at 50 °C gave the corresponding diepoxide **100.4**. The use of SnCl_2_ as a catalyst for the dehydrative cycloaromatization was found to be successful, and this two-step protocol gave the desired dithiophene-fused perylene **100.5** in 19% yield. The synthesis of thiophene-containing pyrenoids **100.8a**,**b** was performed in a similar fashion.

**Scheme 101:**
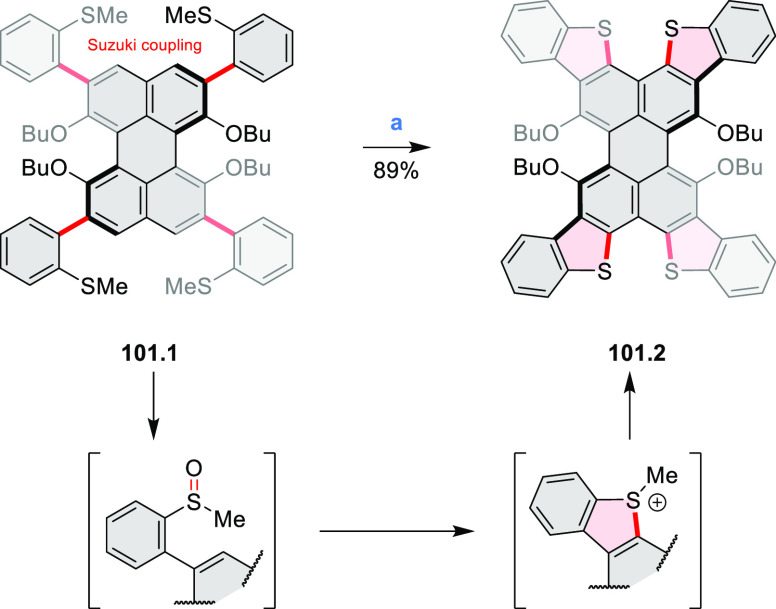
Synthesis of a Benzothiophene-Fused Perylene via Oxidative Cyclization Reagents and conditions: (a)^205^ I_2_, 4:3:4 CHCl_3_/AcOH/propylene oxide, 55 °C, 12 h, 89%.

A perylene extended through fusion with four benzothiophenes was prepared via an oxidative cyclization of the precursor **101.1** bearing *o*-(methylmercapto)phenyl groups (Scheme 101).^205^ Based on a previously known methodology, the reaction was initiated by oxidation of thioethers to sulfoxides upon treatment with I_2_. The sulfoxide underwent an acid-mediated cyclization and condensation to form a methylsulfonium intermediate. Finally, this species was demethylated by a base (AcO^–^ or I^–^) to complete the process. Propylene oxide was added to the reaction medium to scavenge excess HI formed during thioether oxidation, thus preventing the cleavage of butyl ethers. Under optimized conditions, the 4-fold cyclization to **101.2** proceeded in a high yield of 89%. Compound **101.2** was strongly twisted, with interplanar angles of 46.7° and 57.0° between its terminal benzene rings observed in the solid-state structure. A green emission at 504 nm with a quantum yield of 68% was seen in DCM solution of **101.2**. Enantiomers of this compound were separated using chiral HPLC, leading to the observation of CPL with a dissymmetry factor of 1.09 × 10^–3^ in the optically pure samples.

**Scheme 102:**
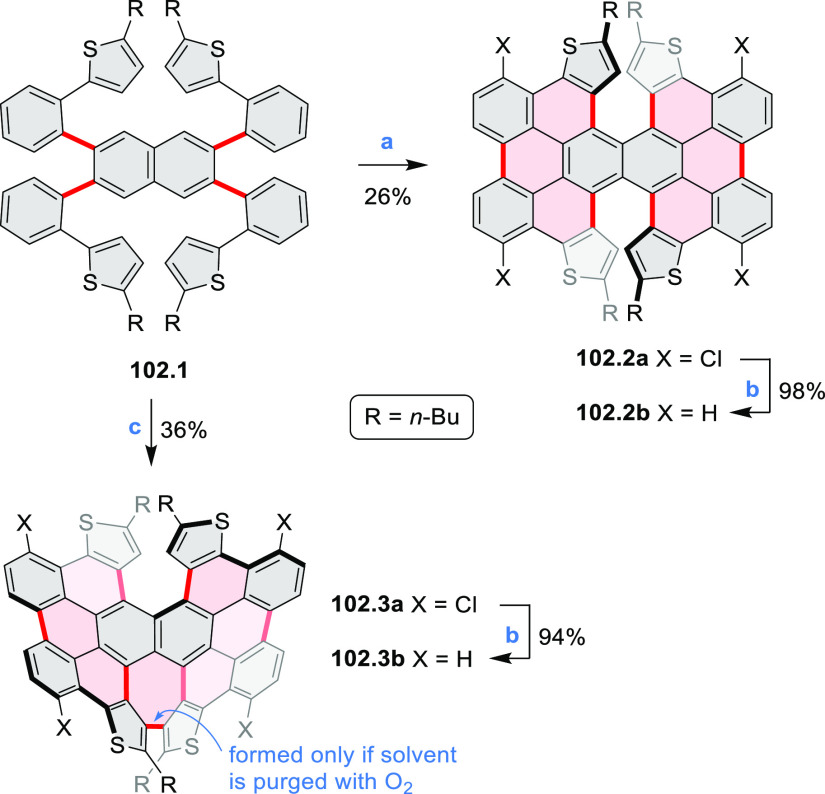
Synthesis of π-Extended S-Doped Double [6]Helicenes Reagents and conditions: (a)^206^ MoCl_5_ (20 equiv), DCM (5 mM), rt, 40 min; (b) Pd/C, HCO_2_H, Et_3_N, pyridine, 130 °C, 25–72 h; (c)^207^ MoCl_5_, O_2_-purged dry DCM, rt, 27 h.

The synthesis of π-extended dithia[6]helicene **102.2** reported by Itami et al.^206^ demonstrates the utility of *ortho*-phenylene-linked precursors for the synthesis of sterically encumbered nanographenes (Scheme 102). When the quadruply substituted naphthalene **102.1** was treated with an excess of MoCl_5_ (20 equiv), the tetrachlorinated **102.2a** was produced in 26% yield. Lowering the proportion of the oxidant (12 to 16 equiv) did not afford well-defined products. It was assumed that chlorination took place immediately after cyclization, and the introduced chlorine atoms served to cap the reactive sites of intermediate **102.2**, corresponding to the most nucleophilic positions of perylene. A subsequent palladium-catalyzed dechlorination of **102.2** quantitatively furnished the halogen-free product **102.2b**. A single-crystal X-ray diffraction analysis revealed that the configuration of the double helicene corresponds to the pair of enantiomers (*P*,*P*)-**102.2b** and (*M*,*M*)-**102.2b**. The meso isomers, (*P*,*M*)-**102.2a** or (*P*,*M*)-**102.2b**, were not detected. It was shown in subsequent work that the formation of the more extensively fused product **102.3a** was possible under somewhat more forced conditions.^207^ The additional formation of a seven-membered ring in **102.3a** occurred when the reaction mixture was additionally purged with O_2_ during MoCl_5_ oxidation.

**Scheme 103:**
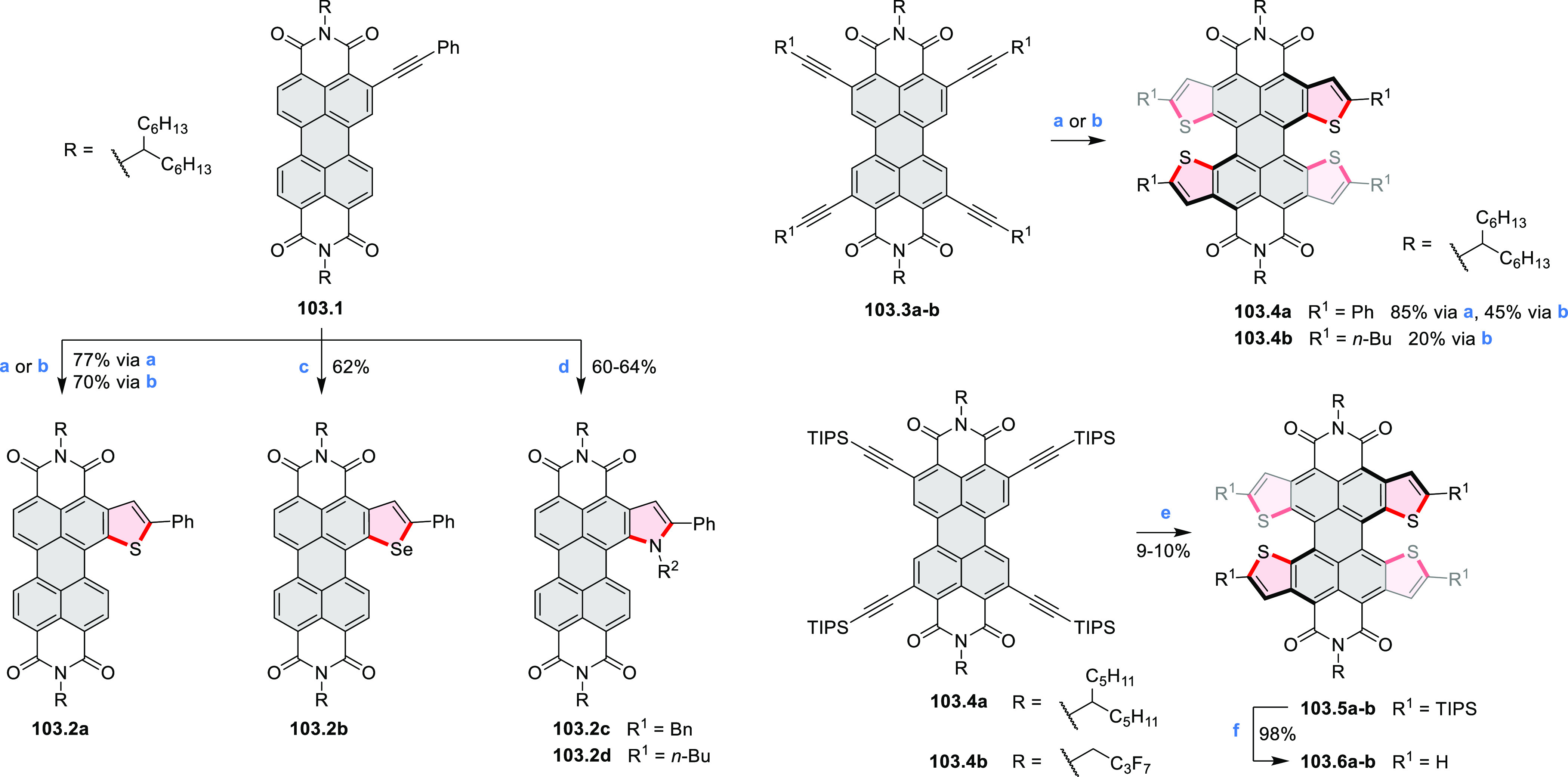
Heteroannulation of Alkynyl-Substituted Perylene Diimides Reagents and conditions: (a)^208^ sulfur powder, DMA, 140 °C, 20–48 h, 77–85%; (b) K_2_S, DMF, 80 °C, 20–48 h; (c) selenium powder, DMA, 140 °C, 36 h, 62%; (d) benzylamine or *n*-butylamine, KO*t*-Bu, toluene, 4 Å MS, 120 °C, 18 h, 60–64%; (e)^210^ sulfur powder, 10:1 DMF/H_2_O, 90 °C, 45 h, 9–10%; (f) TBAF, THF, rt, 1 h, 98%.

Perylene diimides [*a*]-fused with thiophenes, selenophenes, and pyrroles were prepared using an ethynylarene heteroannulation approach (Scheme 103).^208^ The phenylethynyl-substituted PDI **103.1** was converted into the thiophene-fused **103.2a** via treatment with elemental sulfur at 140 °C, based on an earlier methodology.^209^ Alternatively, K_2_S, Na_2_S·9H_2_O, or KSAc were used as the source of sulfur to decrease the reaction temperature to 80 °C while retaining yields above 50%. Similarly, heating **103.1** with Se in DMA provided the selenophene-fused product **103.2b**, and treatment with primary amines in the presence of a base resulted in the pyrrole-fused **103.2c**,**d**. All annulations are thought to proceed via nucleophilic attack on the distal carbon atom of the alkyne, assisted by its conjugation with the electron-deficient PDI core. Analogous conditions were also used for the cyclization of tetraalkynes **103.3a**,**b**. While the synthesis of **103.4a** was efficient using elemental sulfur, the *n*-butyl-substituted analogue **103.4b** could only be obtained upon treatment with K_2_S at a lower temperature (80 °C). A relatively low reaction temperature of 90 °C was also employed when using sulfur in 10:1 DMF/H_2_O medium, although low yields were observed for the TIPS-functionalized quadruply cyclized products **103.5a**,**b**.^210^ These derivatives were then desilylated to afford **103.6a**,**b**. These fused PDIs were determined by X-ray crystallography to be strongly twisted, with an ca. 53° interplanar angle between the blades of the [5]helicene substructure. Despite the large twist angle, enantiomers of **103.6a** were observed to interconvert on the time scale of minutes at rt.

**Scheme 104:**
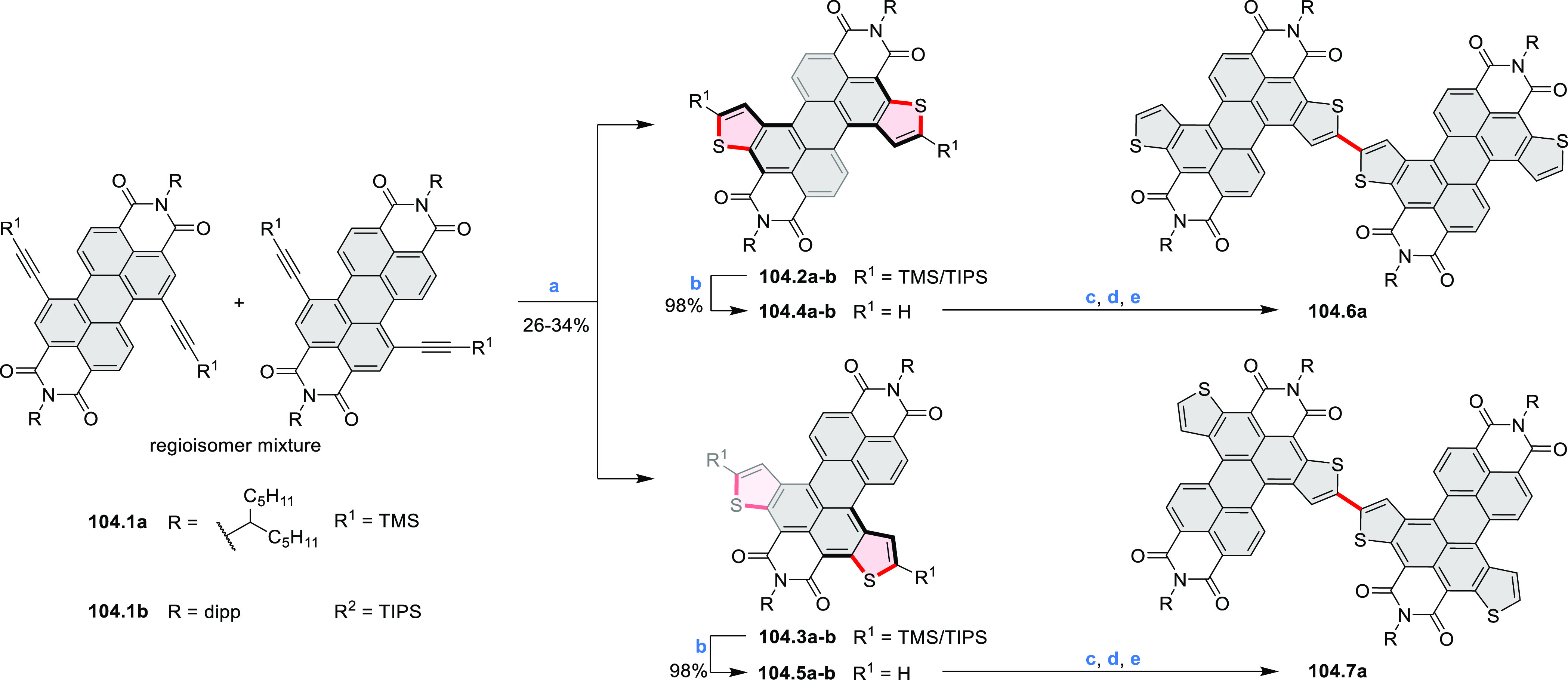
Synthesis of Perylenodithiophenes and Their Dimers Reagents and conditions: (a)^211^ sulfur powder, 10:1 DMF/H_2_O, 90 °C, 12 h, 26–34%; (b) K_2_CO_3_, 10:1 THF/MeOH, rt, 3 h, 98%; (c) NBS, 20:1 DCM/AcOH, 55 °C, 79–80%; (d) Cu powder, DMSO, 90 °C, 10 h; (e) PdCl_2_(dppf), TMEDA, NaBH_4_, THF, 55 °C, 3 h, 45–66% (two steps).

The same method was used for the cyclization of PDIs **104.1a**,**b** bearing two alkynyl substituents in the bay positions (Scheme 104).^211^ The starting materials were used as mixtures of 1,6- and 1,7-disubstituted regioisomers derived from mixed 1,6- and 1,7-dibromo PDIs. Isomeric fused products **104.2**–**3** were thus obtained in modest yield and separated by chromatography. These were further processed by desilylation to **104.4**–**5**. Finally, dimers **104.6a**–**7a** were prepared via double α-bromination of **104.4**–**5** at the fused thiophene moieties, dimerization through Ullmann coupling, and dehalogenation to remove unreacted thienyl bromides. In the solid-state structures of both **104.4b** and **104.5b**, slightly twisted geometries were observed, with interplanar angles of 23–26°. Dimers **104.6a**–**7a** had narrower band gaps and red-shifted absorption spectra, with the low-energy π–π* absorption at 705 and 708 nm, respectively, in contrast to those of the monomeric **104.4a**–**5a** (respectively, 638 and 654 nm).

**Scheme 105:**
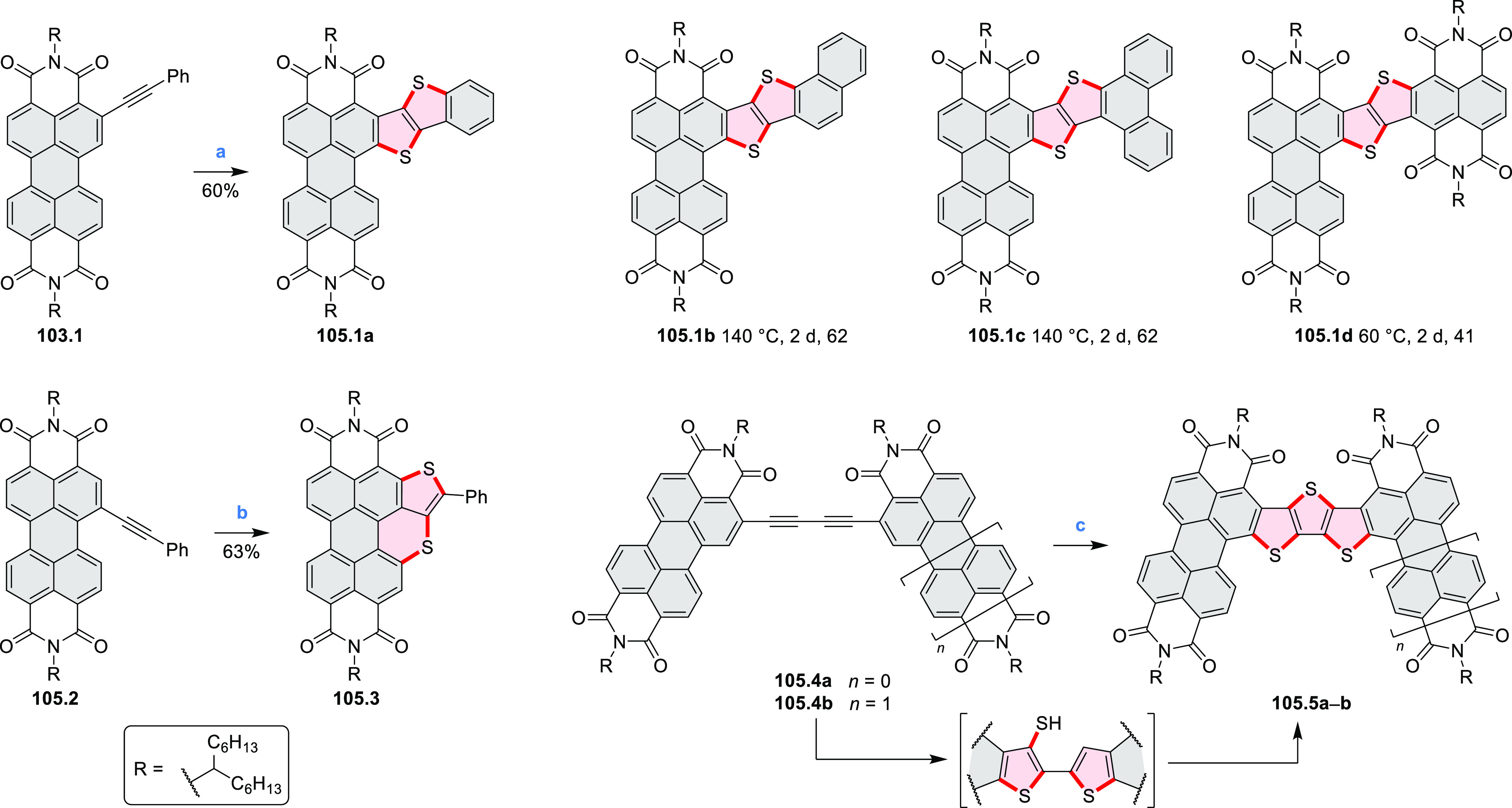
Stitching Thienannulations of Alkynyl-Substituted PDIs Reagents and conditions: (a)^212^ K_2_S, DMF, 170 °C, 12 h, 60%; (b) K_2_S, DMF, 40 °C, 1 h, 63%; (c) K_2_S, DMF, **105.5a**: 80 °C, 2 days, 61%, **105.5b**: 25 °C, 15 h, then 140 °C, 2 days, 79%.

Treating *ortho*-(arylethynyl)-substituted PDIs with K_2_S in DMF at even higher temperatures (140–170 °C) than shown above in Scheme 103 led to a stitching thienannulation, providing the thienothiophene-fused structures **105.1a**–**c** (Scheme 105),^212^ rather than products of a single annulation such as **103.2a**. The same reaction proceeded at a lower temperature for a more electrophilic substrate (alkyne-bridged PDI–NDI conjugate), providing **105.1d**. This process is thought to begin with a nucleophilic addition of S_3_^•–^ to alkynes. The formation of S_3_^•–^ upon addition of K_2_S to DMF was confirmed by its characteristic absorption at 617 nm. The bay-phenylethynyl substrate **105.2** led instead to the thienobenzothiopyran **105.3**, apparently because of the more electrophilic nature of PDI compared to a phenyl group. Triple thienannulations of butadiyne-bridged substrates were also demonstrated, providing the dithienothiophene-fused **105.5a**,**b**. Lateral thiophene rings in these products readily formed at ambient or slightly elevated temperatures, while closure of the central ring required harsher conditions. The 2-thieno-3-mercaptothiophene intermediates were isolated by running the reaction at 25–40 °C. Apart from the perylenoid products shown here, several NDI-derived analogues were reported, demonstrating the versatility of this methodology for electron-deficient aromatics.

**Scheme 106:**
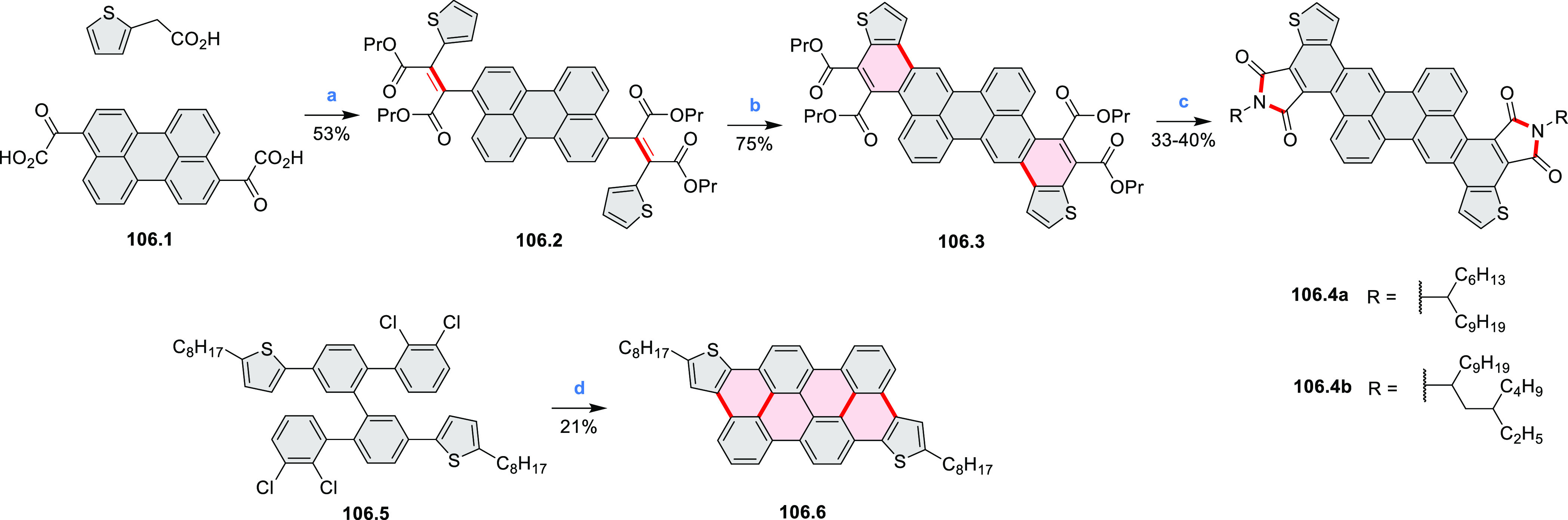
Synthesis of Thiophene-Fused Perylenoids Through Dehydrogenative Photocyclizations Reagents and conditions: (a)^213^ Ac_2_O, Et_3_N, THF, reflux, 16 h, then 1-bromopropane, DBU, 1:10 PrOH/THF, reflux, 10 h, 53%; (b) I_2_, 1:13 dioxane/toluene, *hν* (incandescent lamp), reflux, 6 days, 75%; (c) amine hydrochloride, imidazole, *o*-dichlorobenzene, reflux, 16 h, 33–40%; (d)^214^ Na_2_CO_3_, 77:1 acetone/H_2_O, rt, *hν* (medium pressure mercury lamp), 5 h, 21%.

A perylene derivative **106.3** fused with two benzothiophene motifs was prepared via dehydrogenative cyclization of **106.2** upon irradiation in the presence of I_2_ (Scheme 106).^213^ The diester functions of **106.3** were then converted into imides via treatment with primary amines and imidazoles in refluxing *o*-dichlorobenzene. These compounds were able to form liquid crystalline mesophases thanks to their tendency to self-assemble. The use of doubly branched alkyl chains in **106.4b** led to a much wider temperature window for the mesophase. A cyclodehydrochlorination reaction upon irradiation under basic conditions was used to prepare **106.6** by forming four C–C bonds in a single step.^214^ Several nonheterocyclic PAHs as well as 2-azatriphenylene were prepared by the same method, achieving higher overall yields and yields per cyclization than in the case of **106.6**. The relatively low efficiency with a thiophene-containing substrate was attributed to a greater likelihood of rearrangement reactions.

**Scheme 107:**
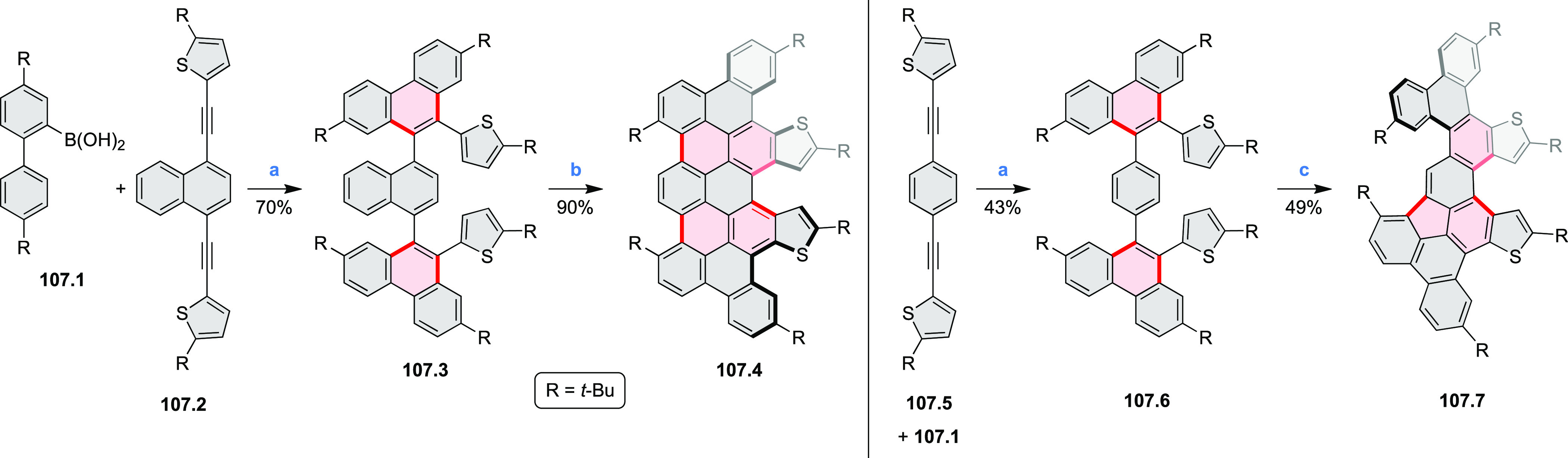
Synthesis of Thiophene-Fused Perylenoids R = *t*-Bu. Reagents and conditions: (a)^215^ [Rh(Cp*)Cl_2_]_2_, AgOAc, DMF, 100 °C, 16 h, 70%; (b) FeCl_3_, 1:2 MeNO_2_/DCM, rt, 1 h, 90%; (c) MeSO_3_H, DDQ, DCM, 0 °C, 2 h, 49%.

PAHs with a picene substructure, including the perylenoid **107.4**, were prepared via an oxidative coupling cyclization (Scheme 107).^215^ Sterically congested precursors **107.3** and **107.6** were prepared in a rhodium-catalyzed double annulation reaction from the corresponding dialkynes (**107.2** and **107.5**) and the biphenyl boronic acid **107.1**. The subsequent oxidation of **107.3** with FeCl_3_ as an oxidant provided the quadruply cyclized, *C*_2_-symmetrical product **107.4** in 90% yield. Compound **107.6** upon treatment with DDQ and MsOH led to the triply cyclized, desymmetrized product **107.7** in 49% yield, in which one five-membered ring was formed. The other side of the molecule did not cyclize in the same manner due to steric repulsion. Crystal structures of **107.4** and **107.7** revealed a twisted geometry in both products, with 37–39° interplanar angles between the thiophene-containing blades.

**Scheme 108:**
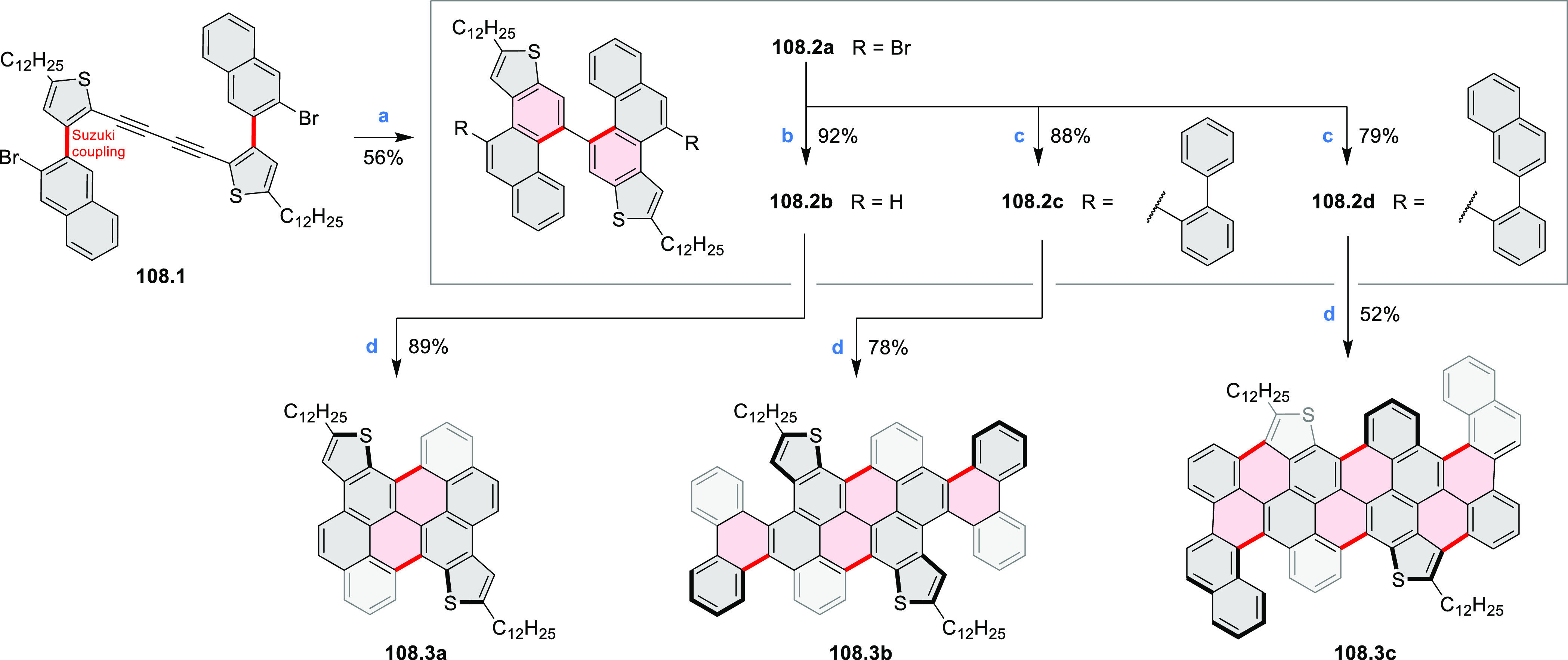
Cyclodehydrogenation toward Thiophene-Fused Nanographenes Reagents and conditions: (a) PtCl_2_, toluene, 100 °C, 20 h; (b) *n*-BuLi, THF, −78 °C, 1 h; (c) RB(pin), Pd(PPh_3_)_4_, K_2_CO_3_, toluene/EtOH/H_2_O, 90–100 °C, 24 h; (d) DDQ, TfOH, DCM, −35 °C for **108.3a** or 0 °C for **108.3b**,**c**, 30–90 min.

A similar cyclodehydrogenation was used to prepare thiophene-fused nanographenes **108.3a**,**b** that contained multiple instances of [4]- and [5]helicene substructures (Scheme 108). The bi(thienophenanthryl) precursors were assembled in two steps, starting with a Pt-catalyzed cyclization of the diyne **108.1** to provide **108.2a**. This compound was then converted into the cyclodehydrogenation precursor **108.2b** by dehydrogenation and into **108c**,**d** by Suzuki coupling. The final cyclodehydrogenation was conducted using the DDQ oxidant in the presence of TfOH, giving **108.3a**–**c** in moderate to high yields, which corresponded to 90–94% yield per cyclization.

Dihedral angles in **108.3a**–**c** were estimated by DFT calculations to be 15–19° in thieno[4]helicenes, 24.1° in the carbo[4]helicenes of **108.3b**, and 33–34° in thieno- and carbo[5]helicenes of **108.3c**. All [4]helicene substructures readily underwent inversion at rt, while the helicity of [5]thienohelicenes in **108.3b** was reported to be configurationally stable. Greater π-extension corresponded to decreased HOMO–LUMO gaps and red-shifted spectral features (**108.3a**: λ_max_ = 460 nm, Φ = 4.5%; **108.3b**: λ_max_ = 505 nm, Φ = 13.8%; **108.3c**: λ_max_ = 569 nm, Φ = 3.8%).

**Scheme 109:**
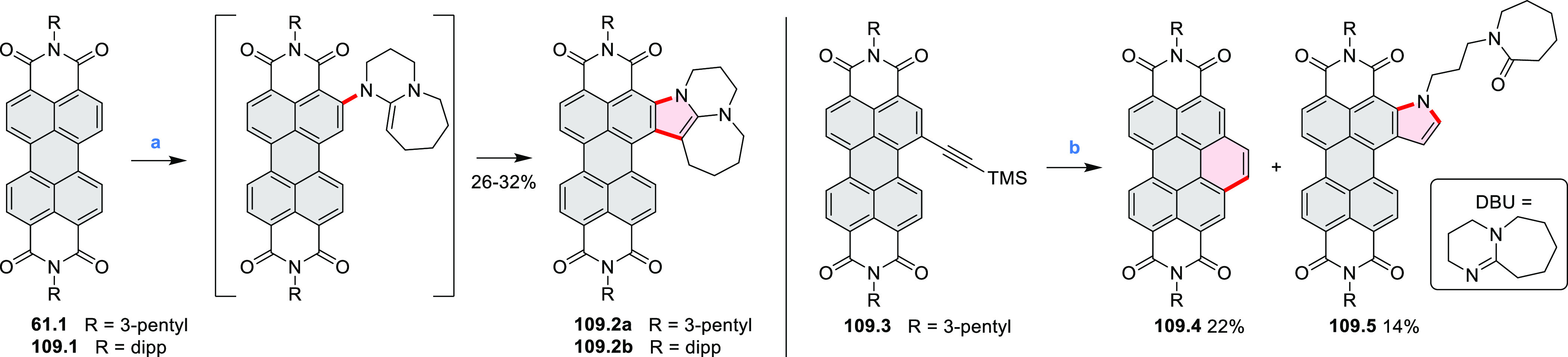
Reactions of PDIs and *bay*-Alkynyl PDI with DBU Reagents and conditions: (a)^216^ DBU, toluene, 140 °C, 24 h, 26–32%; (b)^217^ DBU, toluene, 120 °C, overnight.

Reaction of perylene diimides with DBU was reported to produce fused products **109.2a**,**b**, containing a 5–6–7 ring system (Scheme 109).^216^ This transformation was proposed to proceed through a nucleophilic attack of the imine nitrogen of DBU on the *ortho* position of the PDI followed by dehydrogenation. Another nucleophilic addition of the enediamine carbon at the bay position with further loss of hydrogen was proposed to complete the process. Subjecting the bay-alkynyl-substituted PDI **109.3** to similar reaction conditions provided the pyrrole-annulated **109.5** in 14% yield.^217^ The DBU-derived caprolactam ring remained appended to the pyrrolic N atom of **109.5**. The benzannulated PDI **109.4** was isolated alongside **109.5**.

Compound **109.2a** had a broad absorption band extending into the NIR region (580–950 nm), which was thought to include π–π* as well as charge transfer transitions.^216^ Stabilization of the charge-transfer excited state in polar solvents caused a bathochromic shift of this absorption band (ca. 70 nm difference between toluene and DMSO). Quenching of this low-energy absorption and strong enhancement of the emission peaks at 532 and 569 nm occurred upon protonation. In **109.5**, the electron-donating character of the fused pyrrole caused a strong red-shift of the PDI absorption features to a 625 nm maximum.^217^ A red emission (λ_em_ = 654 nm, Φ = 25%) was also observed. Overall, pyrrole fusion of PDIs provides a way of achieving panchromatic absorption.

**Scheme 110:**
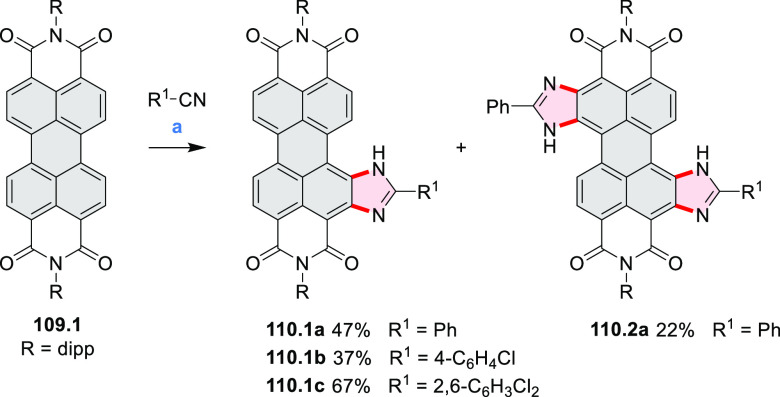
Synthesis of Imidazole-Fused PDIs Reagents and conditions: (a)^218^ NaNH_2_, respective benzonitrile used as solvent, 150–170 °C, aeration, 0.5–3 h.

Imidazole-fused PDIs were accessed via the reaction of **109.1** with NaNH_2_ and benzonitriles which were used as both reagent and solvent (Scheme 110).^218^ Using unsubstituted benzonitrile provided a mixture of single and double annulation products **110.1a** and **110.2a**, whereas with chlorinated benzonitriles only the singly annulated **110.1b**,**c** were obtained. In analogy to the pyrrole-annulated systems (Scheme 109), PDIs extended with imidazole rings had strongly red-shifted spectral features. Further bathochromic shifts occurred upon deprotonation of the imidazoles with a strong base (tetraoctylammonium hydroxide) in THF solution. Unlike the weakly emissive pyrrole-fused PDIs, **110.1a**–**c** displayed luminescence quantum yields of 98–99% (λ_em_ = 584–597 nm). **110.2a** had a slightly weaker deep red emission (λ_em_ = 584–597 nm, Φ = 85%). Imidazolide anions had weak emission peaks red-shifted into the NIR region, with the longest wavelength observed for the dianion of **110.2a** (λ_em_ = 802 nm, Φ = 5%). These properties were used to demonstrate colorimetric and fluorescent sensing of pH and CO_2_.

**Scheme 111:**
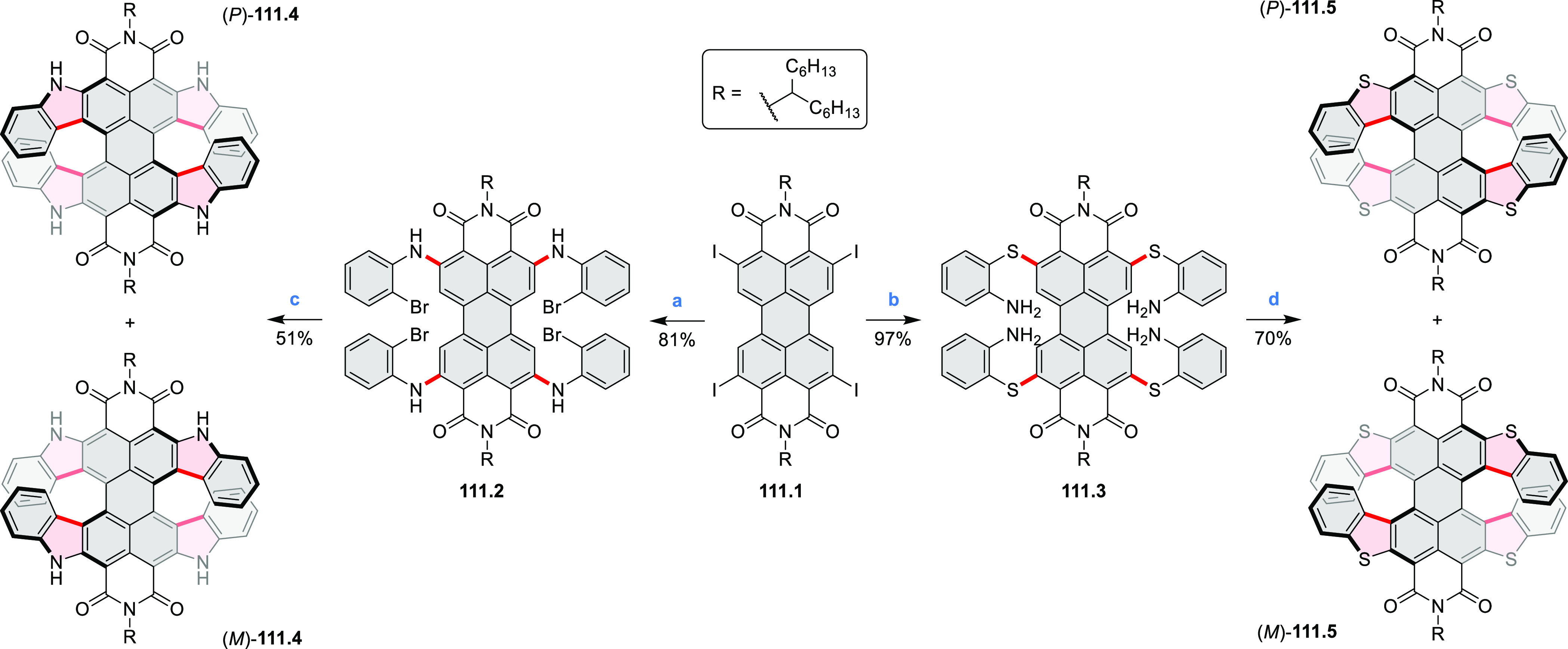
Synthesis of Tetraindole- and Tetra(benzothiophene)-Fused PDIs Reagents and conditions: (a)^219^*o*-bromoaniline, KO*t*-Bu, neat, 120 °C, 24 h, 81%; (b) *o*-aminothiophenol, CuI, l-proline, Cs_2_CO_3_, CTAB, 3:1 toluene/H_2_O, reflux, overnight, 97%; (c) Pd(OAc)_2_, P(*t*-Bu)_3_·HBF_4_, Cs_2_CO_3_, DMF, 150 °C, 24 h, 51%; (d) isopentyl nitrite, 1:1 DCM/AcOH, 0 °C, 1 h, then hydroquinone, rt, 30 min, 70%.

Double helicenes **111.4**–**5** were obtained via fusion of four indole or benzothiophene moieties to a PDI core (Scheme 111).^219^ First, the tetraiodo PDI **111.1** (accessible from unsubstituted PDI)^220^ was functionalized with *o*-bromoaniline via an S_N_Ar reaction or with *o*-aminothiophenol via Cu-catalyzed cross-coupling to provide **111.2**–**3**. Compound **111.2** was then cyclized using Pd-catalyzed intramolecular C–H activation, while for **111.3** the cyclization was effected by diazotization and subsequent reduction with hydroquinone. The products **111.4**–**5** had a high barrier to epimerization (ca. 65 kcal mol^−1^ as determined by DFT) and were separated into their *P* and *M* enantiomers by chiral HPLC.

Absorption features of **111.4**–**5** were heavily red-shifted relative to the parent PDIs, with the lowest-energy absorption band at 737 nm in **111.4**. Notably, the CD features of pure enantiomers extended into the NIR region. Consequently, OFET devices prepared using (*P*)-**111.4** and (*M*)-**111.4** displayed differences in photocurrent when illuminated by left- or right-handed circularly polarized light at 635 or 730 nm.

**Scheme 112:**
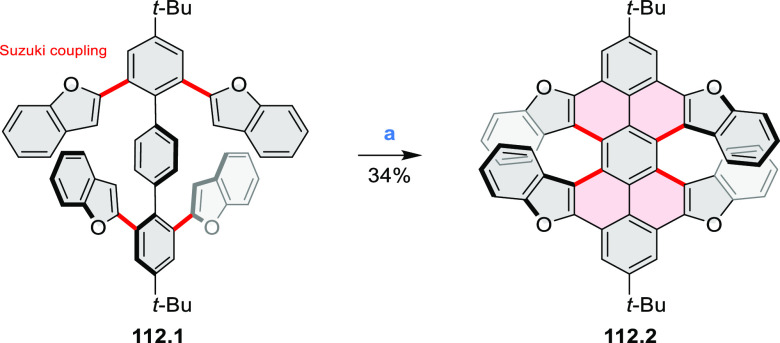
Synthesis of a Double Tetraoxa[7]helicene Reagents and conditions: (a)^221^ DDQ, TfOH, DCM, 0 °C, 2 h.

Another example of a double [7]heterohelicene is **112.2** with four benzofurans fused to a dibenzoperylene core (Scheme 112).^221^ This compound was prepared in 34% yield from the tetrakis(benzofuranyl)terphenyl **112.1** upon cyclodehydrogenation with DDQ in the presence of TfOH. The product **112.2** had ca. 30° interplanar angles between helicene blades in the solid state. It was configurationally stable, with a 50.5 kcal mol^−1^ barrier to interconversion estimated by DFT calculations, and was separated into (*P*,*P*) and (*M*,*M*) enantiomers. Compound **112.2** had a lowest-energy absorption band at 488 nm and a bright emission (λ_max_ = 511 nm, Φ = 71%) in DCM solution. Upon treatment with AgBF_4_, it was reversibly converted into a radical cation, which displayed panchromatic absorption with a broad NIR band at 770–1200 nm.

## 4 Pyrenoids

### 4.1 Triangulenes

Heteroatom-doped triangulenes can be conveniently classified as either heteroatom- or carbon-centered (Sections 4.1.1 and 4.1.2, respectively), depending on the element located at the center of the fused framework. Other heteroatoms are usually located at some of the three central positions along the zigzag edges of the triangulene parent. In the absence of additional fusion, pyrene is the largest leading substructure present in the triangulene core. However, several *peri*-fused triangulenes have been discussed in Sections 2 and 3, e.g., **4.3a**,**b**, **12.4**–**6**, **19.10**, **20.4**, **27.3**, **32.12**, and **84.2e**.

#### 4.1.1 Heteroatom-Centered Heteratriangulenes

**Scheme 113:**
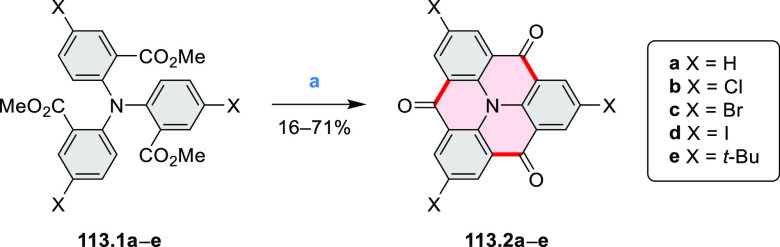
Azatriangulenetriones from Triarylamine Precursors Reagents and conditions: (a)^222^ (1) KOH, MeOH/H_2_O, (2) SOCl_2_, DMF, then SnCl_4_, DCM.

In 2020, Hamzehpoor and Perepichka reported a series of emissive azatriangulenetrione derivatives **113.2a**–**e** (Scheme 113).^222^ Compounds **113.2a**–**e** were obtainable from the 3-fold intramolecular Friedel–Crafts acylation of the corresponding triester precursors **113.1a**–**e**. In the solid state, the unsubstituted azatriangulenetrione **113.2a** exhibits phosphorescence at 538 nm with a lifetime of 28.6 ms and a high photoluminescence quantum yield (PLQY) of 42%. The solids of the trihalo-substituted congeners **113.2b**–**d** also show phosphorescence within 560–589 nm, albeit with shorter lifetimes and lower PLQYs. This trend observed from the four emitters was ascribed to the heavy atom effect. In stark contrast, the tri-*t*-butyl-substituted derivative **113.2e** fluoresces at 495 nm with a lifetime of 10.0 ns with PLQY of 11% in the solid state. The single-crystal structures of all five compounds indicate the formation of π-stacks. Contrary to the coaligned, stacking molecules observed for **113.2a**–**d**, the molecules of **113.2e** are rotated by 60° relative to the neighbors along the stack to accommodate the bulk of *t*-butyl groups. This work demonstrated that the solid-state emission properties could be tuned by the crystal packing structures, which in turn could be controlled by the substituents on the π-skeleton.

**Chart 11:**
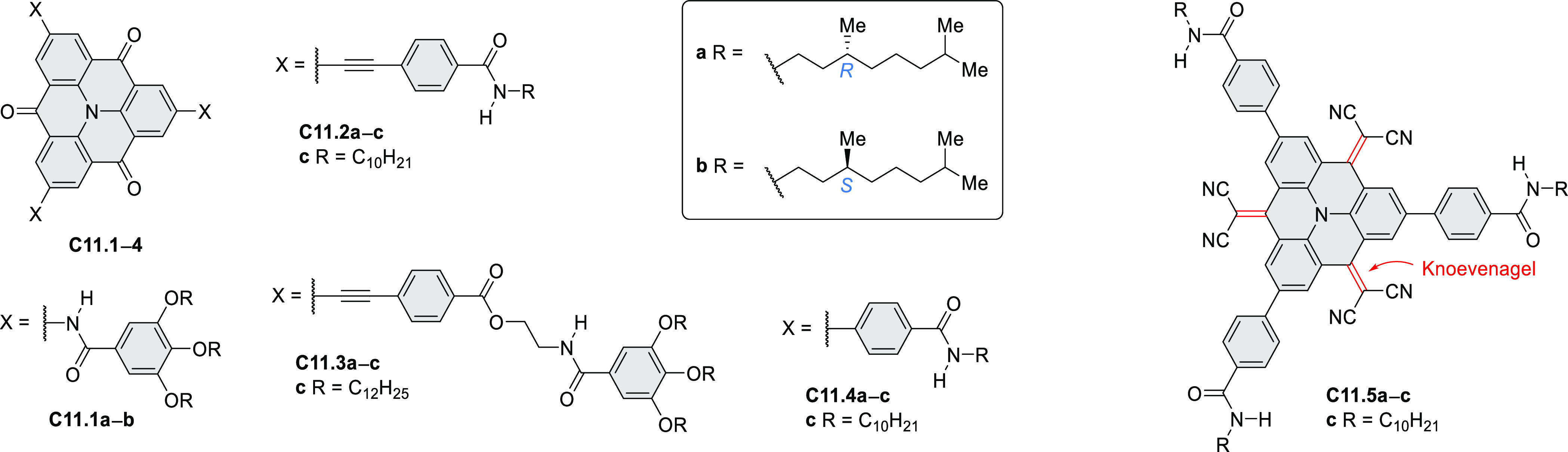
Benzamide-Functionalized Azatriangulenetrione Derivatives

Enantiomeric azatriangulenetriones **C11.1a**,**b**, reported in 2016 by Meijer and Schmidt et al., possess trialkoxy-substituted benzamide side chains, with each alkoxy group in either the *R* or *S* configuration (Chart 11).^223^ The self-assembly process of each enantiopure sample dissolved in *o*-dichlorobenzene was monitored by CD spectroscopy at an initial temperature of 80 °C, and two different aggregation states could be obtained upon cooling. The kinetically trapped state **A** was achieved by direct cooling to 7 °C, whereas the thermodynamic state **B** was reached by undercooling to −5 °C and then returning to 7 °C. The two states were stable for hours. States **A** and **B** were proposed to arise via isodesmic and nucleation–elongation processes, respectively. Notably, mixing the aggregates **A** and **B** of the same enantiomer resulted in the complete conversion from **A** into **B**, as revealed by CD and UV–vis spectroscopy. On the other hand, when **C11.1a** in state **A** was mixed with **C11.1b** in state **B**, the CD signal stayed midway between those of the parent solutions, indicating the absence of state conversion. This work demonstrated the enantioselective nature of the self-assembling processes for the reported molecules.

Chiral and achiral trisubstituted azatriangulenetriones prepared in subsequent years unveiled the pathway complexity of their supramolecular assembling processes. The trialkynyl derivatives **C11.2a**–**c**^224^ and **C11.3a**–**c**^225^ were synthesized by Sánchez and Casado et al., while the triaryl derivatives **C11.4a**–**c** were explored by Sánchez and Ortí et al.^226^ On the basis of CD spectroscopic evidence, all these azatriangulenetriones were proposed to form self-assembled helical stacks in the solution phase. Tris(dicyanovinylidene)-substituted azatriangulenes **C11.5a**–**c** were obtained by Knoevenagel condensation of **C11.4a**–**c** with malononitrile in the presence of TiCl_4_ and pyridine.^794^ DFT calculations suggested the presence of *C*_1_- and *C*_3_-symmetrical conformations for compounds **C11.5a–c**, with the *C*_1_ conformation being less stable by 2.5 kcal mol^−1^ and accessible through a barrier of 5.4 kcal mol^−1^. As shown spectroscopically, the monomeric species with different molecular symmetries were found to self-assemble into different aggregates, which coexisted in freshly prepared solutions. The interconversion between the two aggregates is unfavorable at rt because of the steric effects of the dicyanovinylidene groups within the assembly. Equilibration to the *C*_3_-based assembly could be achieved by heating the solution followed by cooling. The overall process involves deaggregation and the conformation flipping of monomeric **C11.5** from *C*_1_ to *C*_3_ symmetry at a high temperature, followed by reassembly upon cooling.

**Scheme 114:**
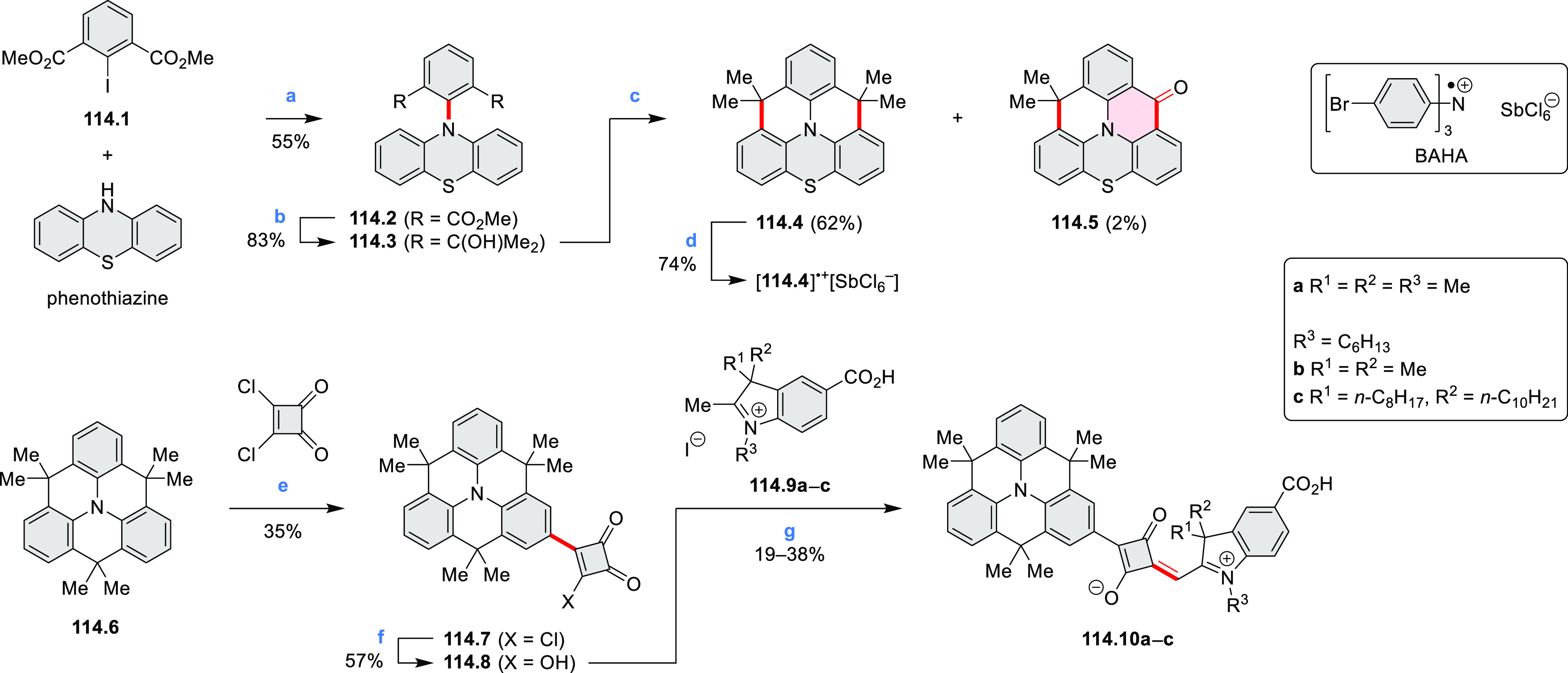
Azatriangulene Derivatives Bearing Peripheral sp^3^-Hybridized Carbon Atoms Reagents and conditions: (a)^227^ Cu, CuI, K_2_CO_3_, *n*-Bu_2_O, 150 °C, 48 h; (b) MeMgBr, toluene, 110 °C, 15 h; (c) 85% H_3_PO_4_, rt, 4 h; (d) tris(4-bromophenyl)ammoniumyl hexachloroantimonate (BAHA), DCM, N_2_, rt, 1 h; (e)^228^ benzene, 80 °C, 24 h; (f) AcOH, H_2_O, HCl, 100 °C, 12 h; (g) benzene/*n*-BuOH/pyridine, Dean–Stark apparatus, reflux, 5 h.

In 2020, Kato et al. reported the preparation of the sulfur-containing azatriangulene analogues **114.4**–**5** (Scheme 114).^227^ The intermediate **114.2** was obtainable from the Ullmann-type reaction between dimethyl 2-iodoisophthalate (**114.1**) and phenothiazine. Compound **114.2** was then treated with MeMgBr to give the diol **114.3**. Subsequent H_3_PO_4_-promoted cyclization yielded the target molecule **114.4** (62% yield) and the side product **114.5** (2%). Upon one-electron oxidation by BAHA, compound **114.4** could be converted to its radical cation **114.4**^•+^ as the SbCl_6_^–^ salt, which was characterized by the UV–vis–NIR and ESR spectroscopy. The three-line splitting observed in the ESR spectrum of [**114**^•+^][SbCl_6_^–^] results from the hyperfine coupling of the unpaired electron with the ^14^N nucleus, with a coupling strength of 0.72 mT.

In 2016, Nithyanandhan et al. reported the zwitterionic dyes **114.10a**–**c** comprising an azatriangulene donor moiety (Scheme 114). The synthesis involved the condensation of the known azatriangulene **114.6** with squaryl dichloride, followed by hydrolysis of the C–Cl bond to afford compound **114.8**. Subsequent condensation with the indolinium salts **114.9a**–**c** provided the target sensitizers **114.10a**–**c**. Upon evaluation of the dye-sensitized solar cell performance, compound **114.10c** displayed the highest power conversion efficiency (PCE) of 6.73% and an open-circuit voltage (*V*_OC_) of 0.53 V without any coadsorbent.

**Scheme 115:**
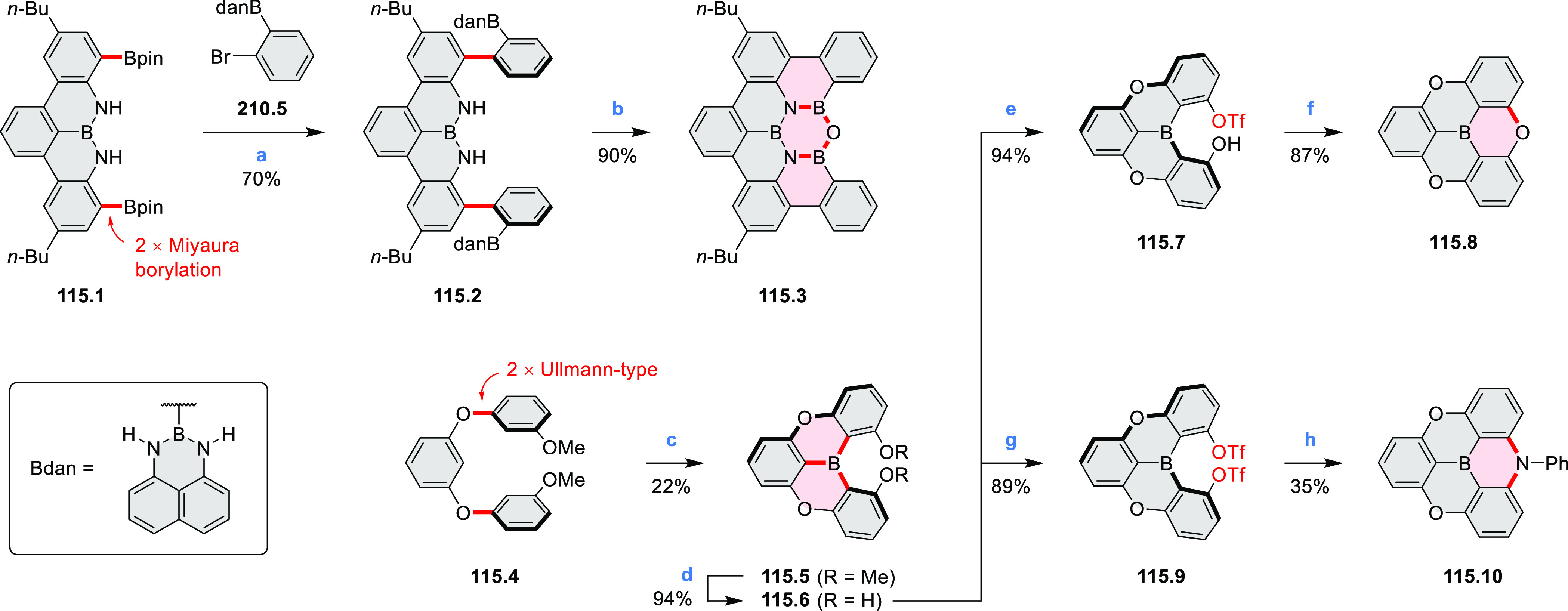
Syntheses of Boron-Centered, Oxygen-Containing Heteratriangulenes Reagents and conditions: (a)^229^ Pd(PPh_3_)_4_, K_2_CO_3_, toluene/EtOH/H_2_O, reflux, 18 h; (b) aq. H_2_SO_4_ (2 M), THF, 50 °C, 3 days; (c)^230^ (1) *n*-BuLi, TMEDA, THF, 0 °C, 0.9 h, then rt, 4 h, (2) BF_3_·Et_2_O, THF/benzene, −40 °C, 0.4 h, then rt, 2 h, then 87 °C, 21 h; (d) BBr_3_, DCM, −78 °C, 0.9 h, then rt, 2 h; (e) Tf_2_O (2.1 equiv), *i*-PrNEt_2_ (1.2 equiv), DCM, −78 °C, 1.25 h, then rt, 3 h; (f) DBU, DMF, microwave, 240 °C; (g)^231^ Tf_2_O (2.2 equiv), *i*-PrNEt_2_ (3.0 equiv), DCM, −78 °C, 1.25 h, then rt, overnight; (h) aniline, LiN(SiMe_3_)_2_, 1,4-dioxane, 10 °C, then 150 °C, 20 h.

In 2020, Fingerle and Bettinger reported the synthesis of the nanographene **115.3** incorporating a boroxadizine (B_3_N_2_O) unit (Scheme 115).^229^ The diboronate ester **115.1** could be prepared from the known compound **210.2** in two steps. Using similar procedures outlined in Scheme 210 (Section 5.3.1), the target molecule **115.3** could be obtained. The UV–vis spectrum of **115.3** contains three broad absorption maxima within 330–370 nm. These peaks are blue-shifted relative to the lowest-energy absorption of the all-carbon analogue (dibenzo[*fg*,*ij*]phenanthro[9,10,1,2,3-*pqrst*]pentaphene). The small Stokes shift (221 cm^–1^) of **115.3** suggested molecular rigidity in the excited state. The NICS(1) value calculated for the boroxadizine ring in **115.3** is −1.0 ppm, reflecting the nonaromatic character of the B_3_N_2_O unit.

In two reports, Kitamoto, Oi et al. described syntheses of boron-centered heteratriangulenes **115.8** and **115.10** (Scheme 115).^230,231^ Among the three attempted synthetic routes, the successful one involved the precursor **115.4**, which was derived from 2-fold Ullmann-type coupling between resorcinol and 3-iodoanisole. 3-Fold *ortho*-directed lithiation followed by treatment with boron trifluoride etherate afforded the annulated product **115.5** in 22% yield. This strategy ensured that the two methoxy groups would remain intact during borylation. Subsequent double demethylation with boron tribromide furnished the diol **115.6**. Controlled esterification with Tf_2_O led to singly (**115.7**) and doubly (**115.9**) triflated products in good yield. For the monotriflate **115.7**, an intramolecular S_N_Ar reaction using DBU as the base smoothly produced the boratriangulene **115.8** bearing three peripheral oxygen atoms. For the bis(triflate) **115.9**, a double S_N_Ar reaction with aniline in the presence of LiHMDS produced the boratriangulene **115.10** bearing two peripheral oxygen atoms and one phenylamino group in 35% yield. Both **115.8** and **115.10** were shown by single-crystal XRD analysis to possess a flat triangulene skeleton in the solid state.

**Scheme 116:**
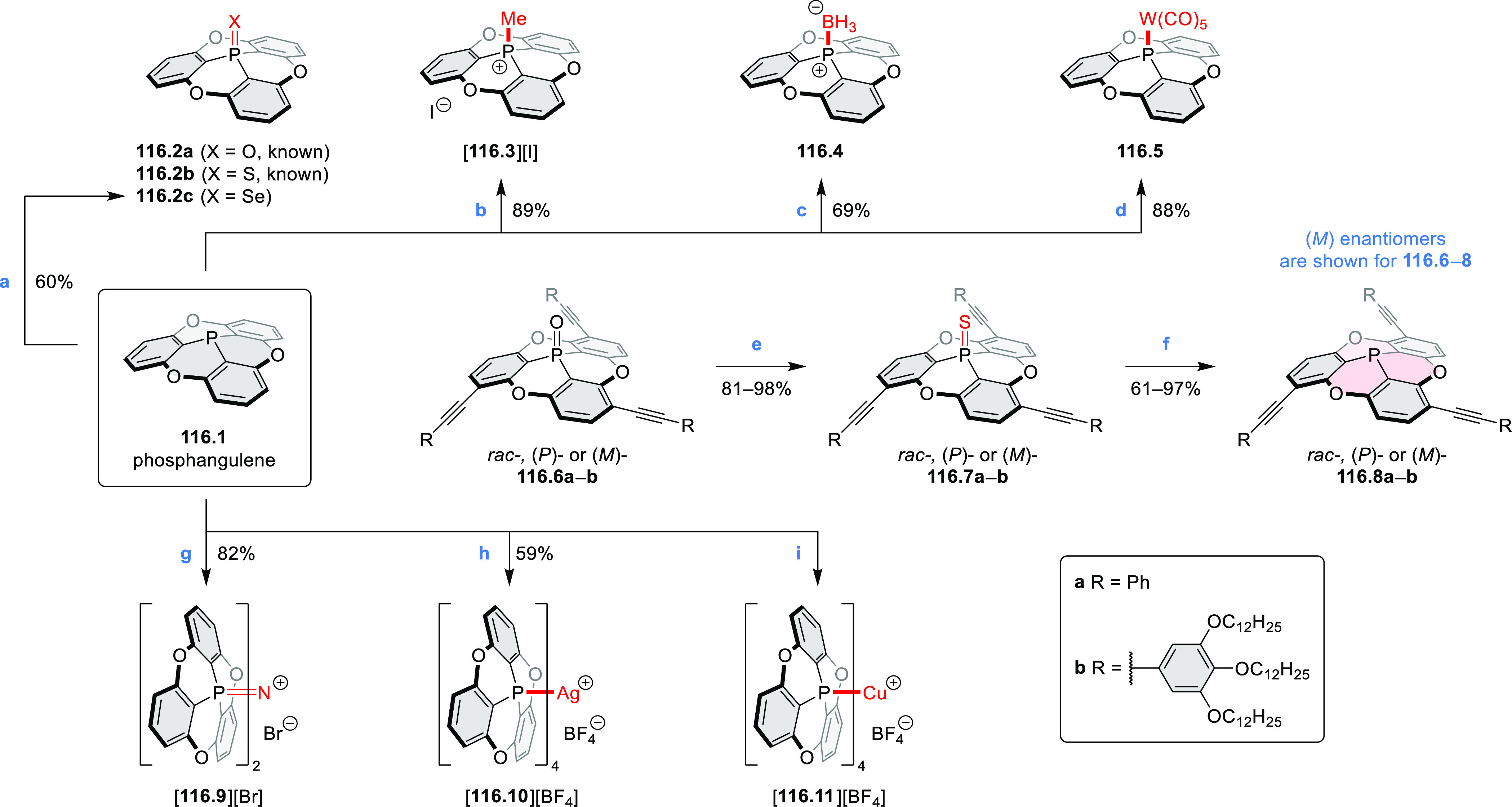
Phosphangulene Derivatives Reagents and conditions: (a)^232^ Se, CDCl_3_, rt, 25 days; (b) MeI, CDCl_3_, 55 °C, 54 h; (c) BH_3_·Me_2_S, CDCl_3_, rt, 10 min; (d) W(CO)_6_, THF, preirradiated with a high-pressure mercury lamp (400 W) at rt for 30 min, then rt, 10 min; (e)^233,234^ Lawesson’s reagent, toluene, reflux. 12–16 h; (f) P(NMe_2_)_3_, toluene, reflux, 15–24 h; (g)^237^ (1) Br_2_, 1,1,2,2-tetrachloroethane, 25 °C, 2 h, (2) NH_2_OH·HCl, reflux, 20 h; (h)^238^ AgBF_4_, DCM, 25 °C, 1 h; (i) [Cu(MeCN)_4_]BF_4_, DCM/MeCN, 25 °C, 12 h.

Yamamura and Nabeshima reported the X-ray structures and computational results on six “phosphangulene” derivatives bearing various axial substitutions (Scheme 116).^232^ In addition to the known phosphangulene oxide (**116.2a**) and sulfide (**116.2b**), the authors prepared the selenide (**116.2c**), *P*-methylphosphonium iodide [**116.3**][I], borane adduct (**116.4**), and phosphine tungsten pentacarbonyl complex (**116.5**) from phosphangulene (**116.1**). According to the XRD and DFT data of the six derivatives, the phosphine cone angle of the tungsten complex **116.5** was the smallest, while that of the phosphonium cation **116.3**^+^ was the largest. Natural bond orbital (NBO) analysis on the optimized structures showed that the bowl depth increased with the s-orbital character of the phosphorus atom. The heteronuclear coupling constants (^1^*J*_P–Se_, ^1^*J*_P–C_, and ^1^*J*_P–W_) for **116.2c**, [**116.3**][I], and **116.5** were noticeably larger than those of the corresponding triphenylphosphine adducts, i.e., Ph_3_PS, Ph_3_PMe^+^I^–^, and Ph_3_PW(CO)_5_. This difference was consistent with the increased s-orbital character in these phosphangulene adducts.

In 2015, Yamamura, Sukegawa, and Nabeshima explored the synthesis and supramolecular properties of the tris(arylethynyl)-substituted phosphangulenes **116.8a**,**b**.^233^ The known phosphangulene oxide **116.6a** was treated with Lawesson’s reagent to give the corresponding sulfide **116.7a** in good yield. Desulfurization with tris(dimethylamino)phosphine furnished the target molecule **116.8a**. This reaction sequence was performed starting with the racemic sample and with both enantiopure samples of **116.6a** with retention of configuration. In the cocrystal of *rac*-**116.8a** with C_60_, the formation of a 2:1 complex was observed. Specifically, the fullerene was encapsulated by one (*P*)-**116.8a** molecule and one (*M*)-**116.8a**, resulting in an achiral cavity. In contrast, the oxide **116.6a** and sulfide **116.7a** each formed a complex with C_60_ in 4:1 ratio, to give a capsule-like structure. This difference in host–guest behavior was attributed to the deeper bowl depth of the **116.8a** relative to the corresponding oxide and sulfide. In 2016, the same group reported a similar phosphangulene analogue **116.8b** bearing nine long alkoxy chains in racemic and enantiopure forms.^234^ The formation of a columnar liquid crystalline phase for *rac*-, (*P*)-, and (*M*)-**116.8b** was supported by DSC and XRD analyses and by CD spectroscopy for the enantiopure samples.

Wuest et al. reported X-ray crystal structures of various polymorphs of phosphangulene chalcogenides **116.2a**–**c**^235^ and those of the various solvates originating from the cocrystallization of **116.2a**–**c** with C_60_ or C_70_.^236^ Furthermore, they characterized the crystal structures of the bis(phosphangulene)iminium salts (including [**116.9**][Br])^237^ and the silver(I) (**116.10**^+^) and copper(I) (**116.11**^+^) complexes featuring a metal center that is tetracoordinated by phosphangulene (Scheme 116).^238^

**Scheme 117:**
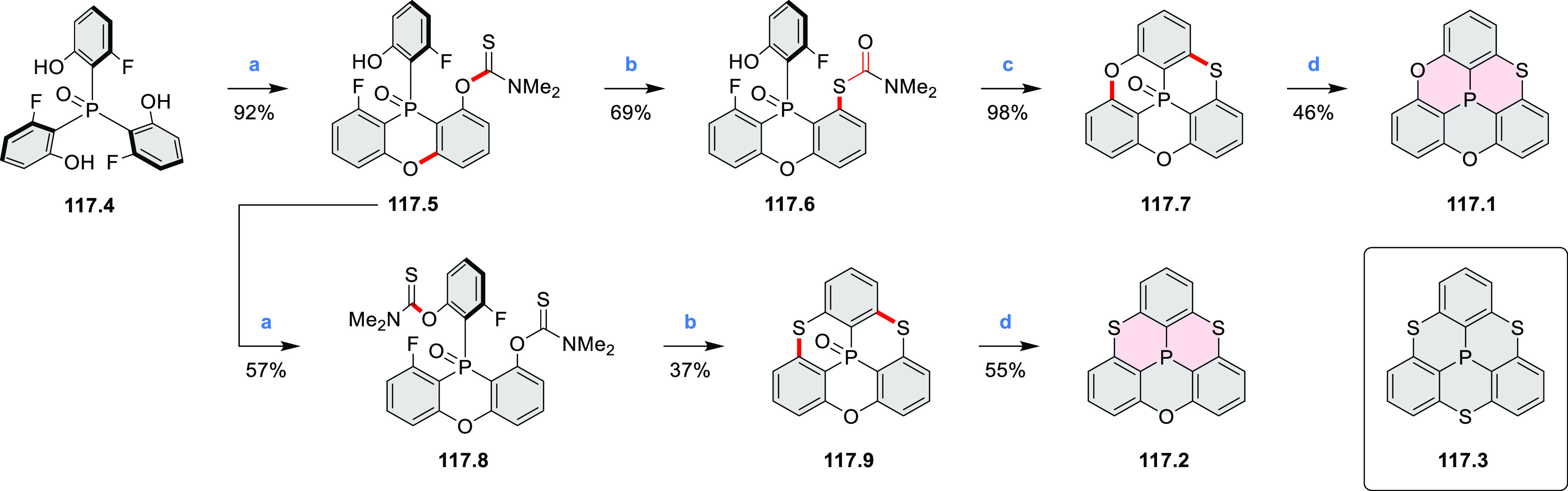
Synthesis of Phosphorus-Centered, Sulfur-Containing Heteratriangulenes Reagents and conditions: (a)^239^ dimethylthiocarbamoyl chloride, DABCO, DMF, rt, 30 min, then 75 °C, 2–17 h; (b) 240 °C, argon, 2–5 h; (c) *t*-BuOK, DMF, 140 °C, 21 h; (d) SiHCl_3_, Et_3_N, toluene, 120 °C, 61–72 h.

In 2016, Yamamura, Hasegawa, and Nabeshima reported three heteratriangulenes **117.1**–**3** that are formed by conceptually replacing one, two, or three oxygen atoms in phosphangulene (**116.1**) with sulfur atoms (Scheme 117).^239^ The synthesis begins with the treatment of the known triarylphosphine oxide **117.4** with dimethylthiocarbamoyl chloride using DABCO as the base. The product **117.5** containing one new *O-*thiocarbamate group and one cyclized phosphaxanthene unit was isolated in 92% yield. Newman–Kwart rearrangement of **117.5** followed by alkoxide-promoted cyclization gave the doubly cyclized product **117.7** in 68% yield over two steps. Finally, the phosphine oxide **117.7** could be reduced using trichlorosilane to the target phosphine **117.1** in 46% yield. The other two target molecules **117.2**–**3** were obtained using similar reactions. X-ray crystal structures of **117.1**–**3** revealed a progressive decrease of their bowl depths (1.88, 1.68, and 1.46 Å, respectively), reflecting the increasing circumference of these molecules caused by the introduction of sulfur atoms.

**Scheme 118:**
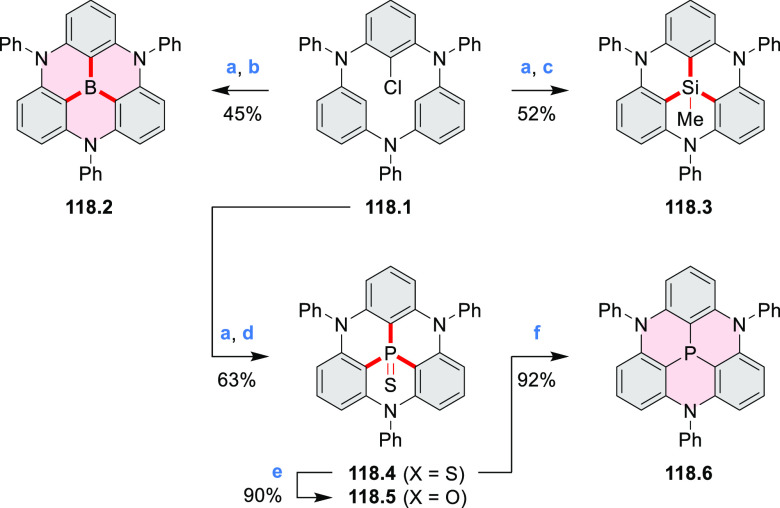
Divergent Synthesis of the Heteroatom-Centered 4,8,12-Triazatriangulenes Reagents and conditions: (a)^240^*t*-BuLi, *t*-butylbenzene, −45 °C, then 50 °C, 30 min to 2 h; (b) BBr_3_, *i*-Pr_2_NEt, −45 °C, 1 h, then 165 °C, 14 h; (c) MeSiCl_3_, *t*-butylbenzene, 150 °C, 18 h; (d) (1) PCl_3_, toluene, 50 °C, 2 h, (2) S_8_, *o*-dichlorobenzene, 110 °C, 12 h; (e) *m*-CPBA, DCM, −30 °C, 1 h; (f) PEt_3_, *o*-xylene, 120 °C, 2 days.

In 2017, the Hatakeyama group developed synthetic routes to boron-, phosphorus-, and silicon-centered 4,8,12-triazatriangulenes.^240^ These syntheses relied on efficient incorporation of heteroatoms into the macrocyclic precursor through electrophilic C–Li and C–H substitution (Scheme 118). The starting macrocycle **118.1** was in turn obtained in four steps starting from 1-bromo-2,3-dichlorobenzene. Postsynthetic modification of **118.4** by desulfurization with PEt_3_ gave the corresponding phosphine **118.6** in 92% yield, whereas oxidation with *m*-CPBA led to the corresponding phosphine oxide **118.5** in 90% yield. Unlike the bowl-shaped structures of **118.3**–**6**, the boron-centered **118.2** adopts a planar geometry. The fluorescence spectrum of **118.2** showed a sharp and strong emission band at λ = 399 nm with Φ = 0.54. Notably, the fwhm of this peak (26 nm) is one of the smallest values observed for organic light-emitting materials. This fwhm value in combination with a small energy difference between the S_1_ and T_1_ states (Δ*E*_ST_ = 0.21 eV) make **118.2** a promising candidate as an OLED material.

#### 4.1.2 Carbon-Centered Heteratriangulenes

**Chart 12:**
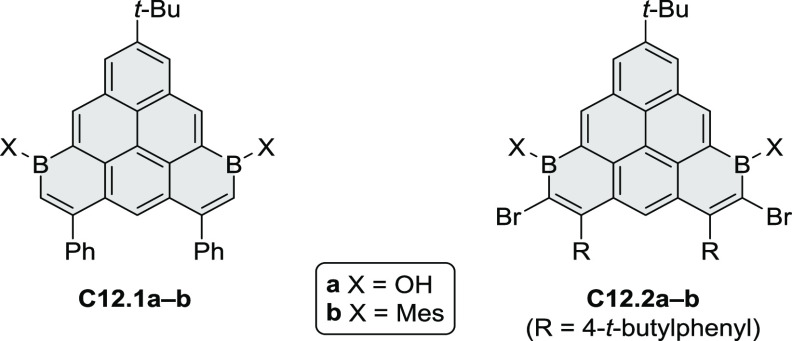
Diboratriangulenes

Diboratriangulenes **C12.1a**,**b** were obtained by employing the C–H borylation strategy reported by Würthner et al. (Chart 12, cf. Scheme 34, Section 3.1.2).^61^ The borinic acid **C12.1a** was synthesized from its diolefin precursor, and upon stepwise treatment with BBr_3_ and MesMgBr, it was converted into the dimesityl-substituted derivative **C12.1b**. Both compounds were stable under ambient conditions. The related diboratriangulenes **C12.2a**,**b** were prepared according to the bromoboration–electrophilic C–H borylation protocol reported by Ingleson and Zysman-Colman et al. (cf. Scheme 99, Section 3.4).^202^ The authors attributed the lower photoluminescence quantum yield of **C12.2b** (5.9%) relative to that of **C12.1b** (34%) to the heavy atom effect in the bromine-containing compound.

**Scheme 119:**
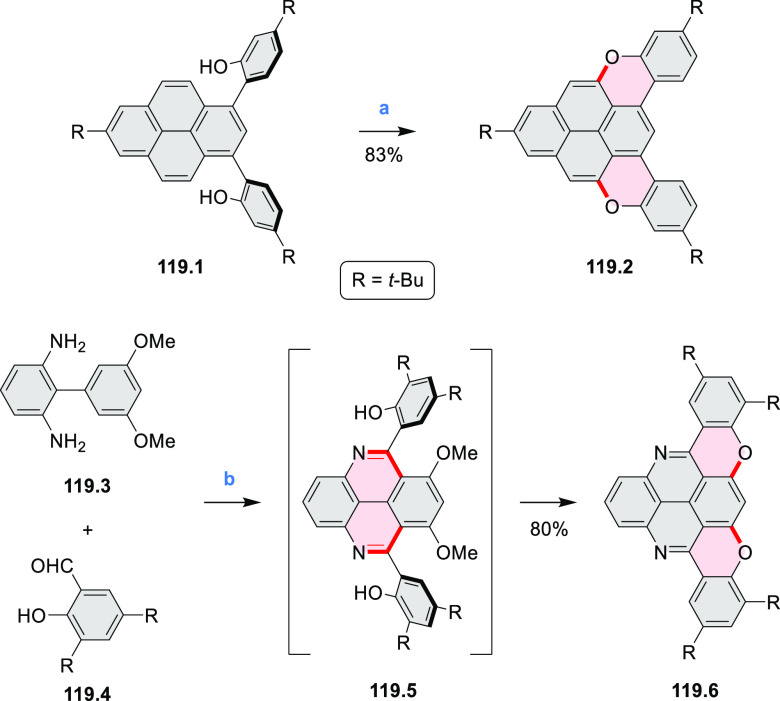
π-Extended Dioxa- and Diazadioxatriangulene Reagents and conditions: (a)^77^ CuO, nitrobenzene, air, reflux, overnight; (b)^241^ TFA, toluene, O_2_, 100 °C, 16 h.

The oxidative Pummerer cyclization employed by Bonifazi et al. for the synthesis of an oxygen-doped perylenoid (see Scheme 46, Section 3.1.2) was also used to prepare the π-extended dioxatriangulene **119.2** (Scheme 119).^77^ A series of π-extended N,O-doped systems, including the triangulene **119.6**, was reported in 2018 by Mastalerz et al.^241^ The synthesis involved the Pictet–Spengler reaction of biphenyldiamine **119.3** with the substituted salicylaldehyde **119.4** in 1:2 stoichiometry in the presence of TFA and O_2_. The intermediate **119.5** was not isolated but spontaneously underwent a thermally induced *ipso*-substitution to give the desired tetraheteratriangulene **119.6** in 80% yield. In contrast, the same *ipso*-substitution applied to other starting materials required higher reaction temperatures (>180 °C, see Scheme 205, Section 5.2.2). Compound **119.6** emits at 538 nm when dissolved in chloroform and at 433 nm when dissolved in THF, with the quantum yields of 8% and 30%, respectively. The effect was caused by protonation of **119.6** in the slightly acidic chloroform. Addition of K_2_CO_3_ to the chloroform solution gave an emission spectrum which resembled that obtained in THF.

**Scheme 120:**
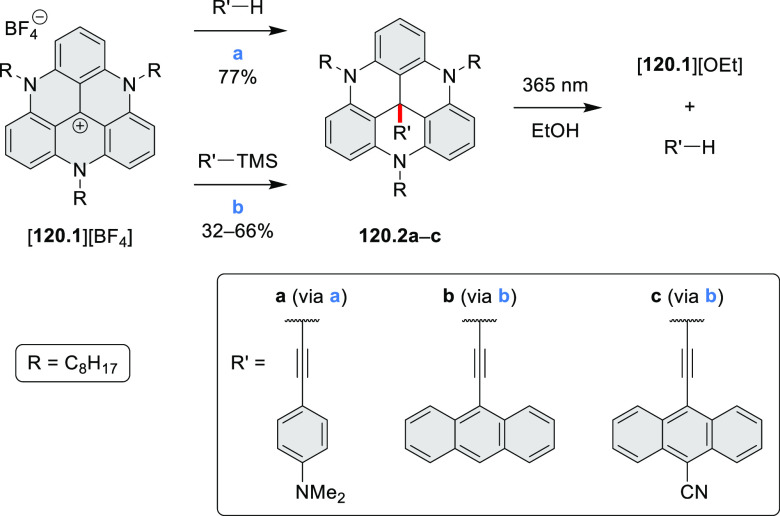
Synthesis of the Triazatriangulene Derivatives Bearing Different Axial Groups at the Central Carbon Atom Reagents and conditions: (a)^242^*n*-BuLi, THF, 0 °C, 1 h then rt, 1 h, (2) [**120.1**][BF_4_], rt, overnight; (b) (1) KOH powder, THF, 0 °C, 30 min, (2) [**120.1**][BF_4_], 0 °C.

In 2015, Matsuda et al. reported photochemical cleavage of triazatriangulene derivatives **120.2a**–**c** substituted with an apical arylethynyl group at the central carbon (Scheme 120).^242^ These derivatives were prepared from the reaction between the known triazatriangulenium salt [**120.1**][BF_4_] and acetylide anions generated via deprotonation or desilylation. Upon irradiation (365 nm) of **120.2a**–**c** dissolved in ethanol, the photoinduced C–C bond cleavage could be detected by the yellow fluorescence at 562 nm and the red color of the resulting cation [**120.1**]^+^. This transformation was also corroborated by the emergence of ^1^H resonances corresponding to [**120.1**]^+^, when **120.2a**–**c** dissolved in ethanol-*d*_6_ was irradiated. Under these conditions, the acetylenic resonance of the expected arylacetylene product was not observable presumably because of H/D exchange.

**Scheme 121:**
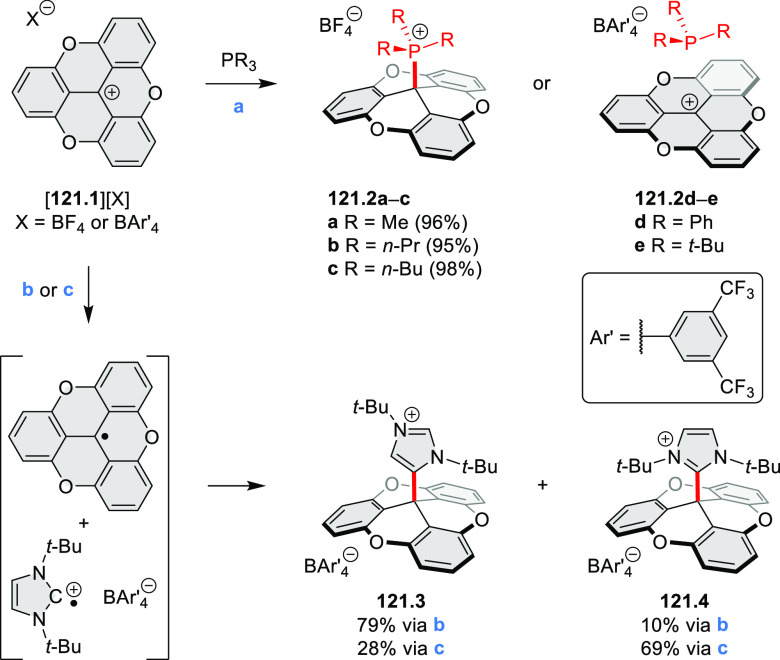
Lewis Acid–Base Chemistry of a Trioxatriangulenium Cation Reagents and conditions: (a)^243^ MeCN (for **a**–**c**), CDCl_3_ or CD_3_CN (for **d**), CD_2_Cl_2_ or CD_3_CN (for **e**), rt, 5 min; (b) 1,3-di-*t*-butylimidazol-2-ylidene, toluene, rt, 1 h; (c) 1,3-di-*t*-butylimidazol-2-ylidene, toluene, −78 °C, 30 min.

Lewis-acid reactivity of the known trioxatriangulenium cation **121.1**^+^ toward phosphines and an N*-*heterocyclic carbene as Lewis bases was explored by Gianetti et al. (Scheme 121).^243^ When [**121.1**][BF_4_] was treated with PMe_3_, P(*n*-Pr)_3_, or P(*n*-Bu)_3_, the corresponding stable Lewis adducts **121.2a**–**c** could be isolated in good yield. The ^31^P NMR spectrum of **121.2a** shows a resonance at 39.7 ppm, indicative of a phosphonium species. In addition, the structure of **121.2a** was confirmed by single-crystal XRD analysis. In contrast, when [**121.1**][BAr′_4_] was exposed to the weakly basic PPh_3_ or the bulky P(*t*-Bu)_3_ in a deuterated solvent, no evidence for the formation of Lewis adducts was observed by ^1^H and ^31^P NMR spectroscopy. In the UV–vis absorption spectrum of an equimolar mixture of [**121.1**][BAr′_4_] and P(*t*-Bu)_3_ in DCM, an extra weak absorption band emerged, with maxima at 576 and 626 nm, besides the major bands of [**121.1**]^+^ at 458 and 483 nm. On the basis of absorption spectroscopy and DOSY data, the authors postulated the dynamic existence of the frustrated encounter complexes **121.2d**,**e** in the solution state. Furthermore, when [**121.1**][BAr′_4_] was mixed with 1,3-di-*t*-butylimidazol-2-ylidene, single-electron transfer occurred, to generate the trioxatriangulenyl radical **121.1**^•^, observable by EPR spectroscopy. The C4-adduct **121.3** and C2-adduct **121.4** were isolated as products of this reaction. The former regioisomer was the major product at rt, and the regioselectivity was reversed when the reaction was performed at −78 °C.

**Chart 13:**
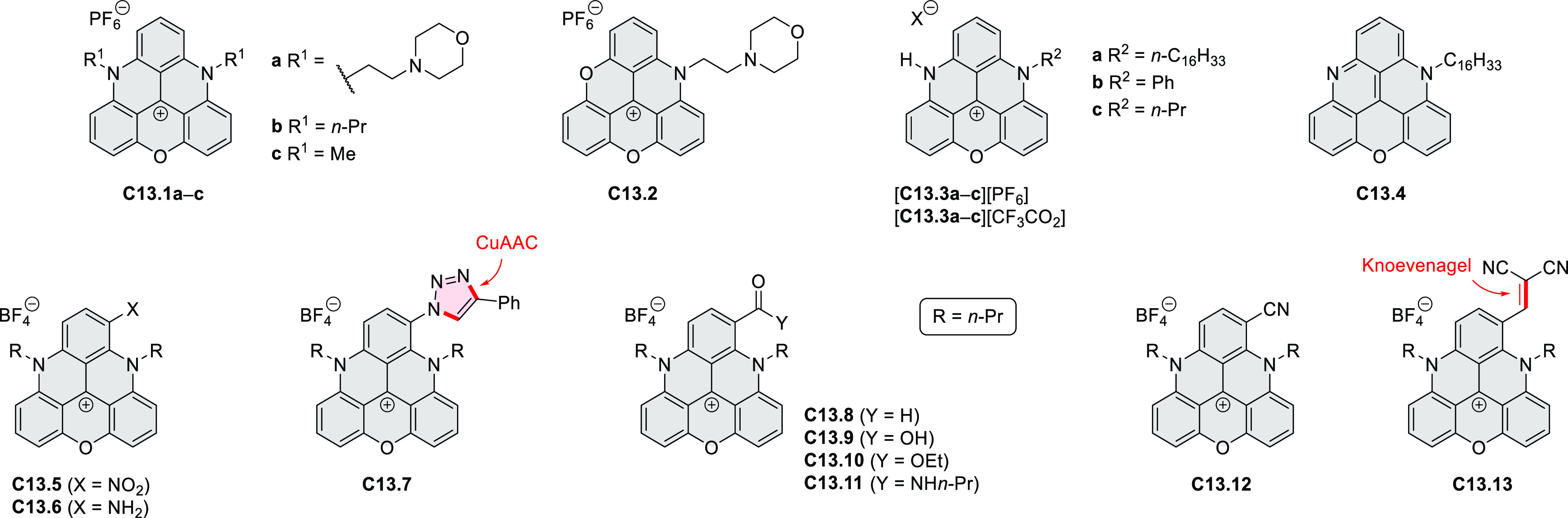
Diazaoxa- and Azadioxatriangulenium Salts

Vilar and Kuimova et al. studied heteratriangulenium cations **C13.1**–**2** as optical probes for G-quadruplexes (Chart 13). The introduction of 2-(4-morpholinyl)ethyl and propyl groups at the nitrogen atoms in **C13.1**–**2** was achieved using established methods. In the presence of DNA, the emission of **C13.1a** was enhanced and bathochromically shifted, whereas the emission of **C13.2** was quenched.^244^ The former compound was then shown to bind with single- and double-stranded DNAs, as well as G-quadruplex DNA, with log *K*_a_ of 5.7–6.1 determined using emission titration experiments. The fluorescence lifetime of **C13.2** observed *in vitro* is longer for G-quadruplexes than for double- and single-stranded DNAs. Compound **C13.2** was found to permeate into cells and localize mainly in the nucleus. Further photophysical and computational investigations suggested the importance of the morpholine moiety for the fluorescence enhancement upon binding with DNA.^245^ In particular, the propyl-substituted derivative **C13.1b** displayed a reversed fluorescence selectivity for double-stranded DNA over G-quadruplexes.

Lacour, Gruenberg, Vauthey, and Bakker et al. reported a series of diazaoxatriangulenium cations [**C13.3a**–**c**]^+^ as the hexafluorophosphate and trifluoroacetate salts (Chart 13).^246^ The free NH group rendered these three cations pH-sensitive (p*K*_a_ = 7.3–8.4). In particular, the hexadecyl-substituted cation **C13.3a**^+^ and its deprotonated form **C13.4** emit fluorescence in the red part of the visible spectrum with quantum yields of 14–16% and lifetimes of 7.7–7.8 ns. Cellular studies with the neutral **C13.4** demonstrated selective imaging of late endosomes in HeLa cells. The lipophilic dodecyl side chain was found to be crucial for achieving such selectivity.

In 2018, Lacour et al. reported nine cationic diazaoxatriangulenium-based dyes **C13.5**–**13** substituted with one electron-donating or electron-withdrawing group at one of the peripheral carbons (Chart 13).^247^ The nitro and formyl species (**C13.5** and **C13.8**, respectively) were derived directly from the known *N*,*N*′-unfunctionalized precursor via appropriate substitution reactions and further used as starting materials for the other seven derivatives. The substituents not only influenced the redox potentials but also tuned the optical properties relative to the unfunctionalized triangulenium. The absorption maxima of **C13.5**–**13** span a range of 528–640 nm in acetonitrile, exhibiting variable fluorescence in the yellow–red range of the visible spectrum. For instance, the emission maxima of the 1,2,3-triazole **C13.7** and the nitrile **C13.12** were observed at 607 and 556 nm, respectively.

**Scheme 122:**
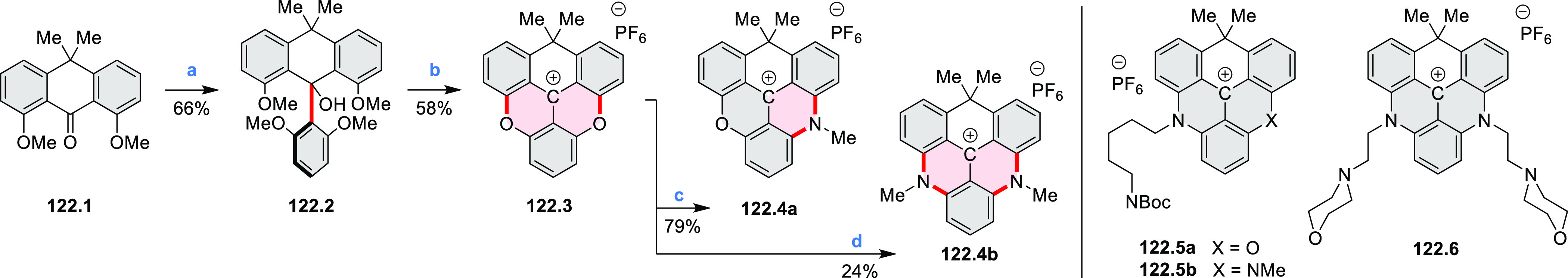
Diheteratriangulenium Dyes with an Isopropylene Bridge Reagents and conditions: (a)^248^*m*-dimethoxybenzene, TMEDA, *n*-BuLi, 1:1 benzene/Et_2_O, 0 °C to rt, 3 h; (b) pyridine hydrochloride, 190–200 °C; (c) 2 equiv of methylamine, PhCO_2_H, NMP, 70 °C, 3 days; (d) 60 equiv of methylamine, PhCO_2_H, 1:1 EtOH/NMP, reflux, 5 days.

Laursen and co-workers reported further analogues of the above compounds, in which one of the peripheral heteroatoms was replaced with an sp^3^ carbon bridge (Scheme 122).^248^ The triangulenium structure was assembled through an addition of *ortho*-lithiated *m*-dimethoxybenzene to **122.1**, followed by condensation in molten pyridine hydrochloride. The resulting dioxatriangulenium salt **122.3** could be further transformed into the azaoxa and diaza analogues **122.4a**,**b** by treatment with methylamine. Other primary amines such as *N-*Boc-cadaverine were incorporated in a similar manner, leading to compounds **122.5a**,**b** and **122.6**.^57^ Introduction of nitrogen atoms caused a bathochromic shift of emission peaks, while the quantum yields increased slightly (**122.3**: λ_max_ = 535 nm, Φ = 54%; **122.4a**: λ_max_ = 584 nm, Φ = 57%; **122.4b**: λ_max_ = 624 nm, Φ = 61%). Additionally, emission of **122.4b** was red-shifted by ca. 50 nm compared to its closest oxygen-bridged analogue **C13.1c**. A further red-shift could be achieved by π-extension (see Scheme 32, Section 3.1.1). The triangulenium salts were introduced into cells for fluorescence imaging, where **122.4b** localized in the nucleus and displayed good photostability. The appended morpholine units in **122.6** resulted in emission quenching due to photoinduced electron transfer. Emission enhancement occurred in an acidic environment or upon binding to DNA.

### 4.2 Azapyrenoids

**Scheme 123:**
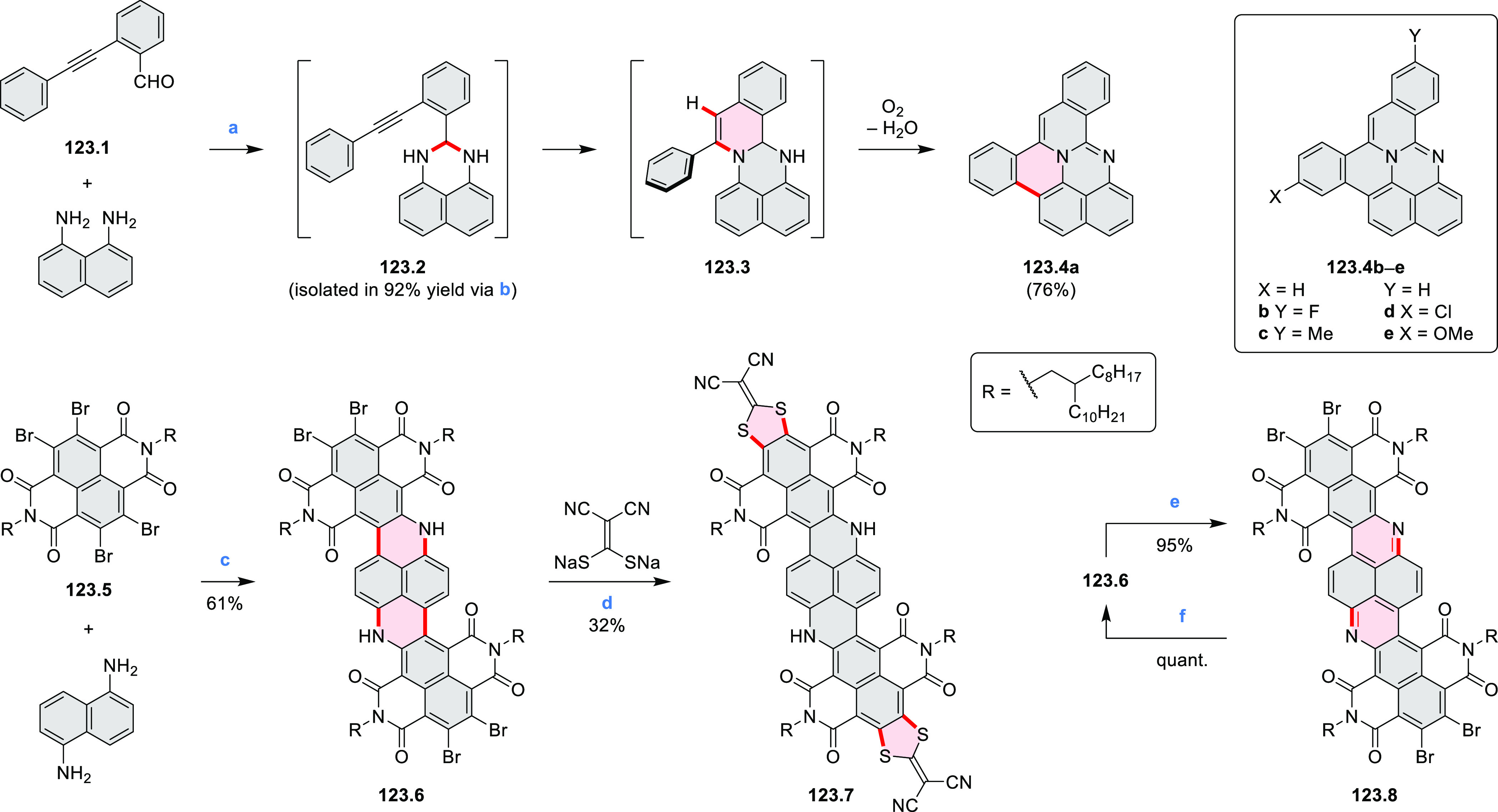
Construction of π-Extended Diazapyrenoids by Annulation Reagents and conditions: (a)^249^ Cu(OAc)_2_ (20 mol %), Cs_2_CO_3_ (1.0 equiv), dioxane, O_2_, 100 °C; (b) dioxane, rt, 6 h; (c)^250^ K_2_CO_3_, THF, 85 °C, 8 h; (d) THF, 80 °C, 36 h; (e) *p*-phenylenediamine, CHCl_3_, rt, several seconds; (f) PbO_2_, CHCl_3_, 45 °C, 5 h.

In 2017, Feng, Liu, and Wang reported a copper-catalyzed domino tricyclization reaction for the construction of the heptacyclic dibenzo[1,2:7,8]quinolizino[3,4,5,6-*kla*]perimidine (**123.4a**) from 2-(phenylethynyl)benzaldehyde (**123.1**) and naphthalene-1,8-diamine (Scheme 123).^249^ The optimized conditions involved heating a dioxane solution of the two reactants in the presence of Cu(OAc)_2_, Cs_2_CO_3_, and O_2_ gas, giving **123.4a** in 76% yield. Stirring the two reactants in the absence of any reagents at rt afforded a high yield of the aminal **123.2**, which was thus proposed as an intermediate for the domino reaction. Presumably, **123.2** undergoes intramolecular hydroamination to produce **123.3**, which is then dehydrogenated to give the target molecule. The functional group tolerance of this transformation was examined, and a library of substituted derivatives including **123.4b**–**e** were isolated in 67–81% yield.

In 2015, Zhang and Pei et al. reported the diazapyrenoids **123.6**–**8** that are fused with two naphthalene diimide units (Scheme 123).^250^ The authors found that 1,5-naphthalenediamine reacted with the tetrabromo NMI derivative **123.5** in a 1:2 stoichiometry to yield the doubly annulated product **123.6**. Notably, this conversion did not necessitate palladium catalysis, and only a weak base (K_2_CO_3_) was required. **123.6** was further annulated with sodium 1,1-dicyanoethylene-2,2-dithiolate to form compound **123.7**. Besides, the two NH groups in **123.6** could be oxidized by *p*-phenylenediamine to give **123.8**. In the UV–vis–NIR absorption spectra, compound **123.6** showed a broad band between 600 and 1200 nm, while the corresponding band for compound **123.7** is bathochromically shifted by roughly 100 nm (log ε ≈ 5). The oxidized derivative **123.8** absorbs in the NIR range much less intensely (log ε ≈ 3). The narrower band gap of **123.7** in comparison with **123.6** was ascribed to a lower LUMO level (−4.72 eV vs −4.35 eV, respectively), as determined by CV measurements.

**Scheme 124:**
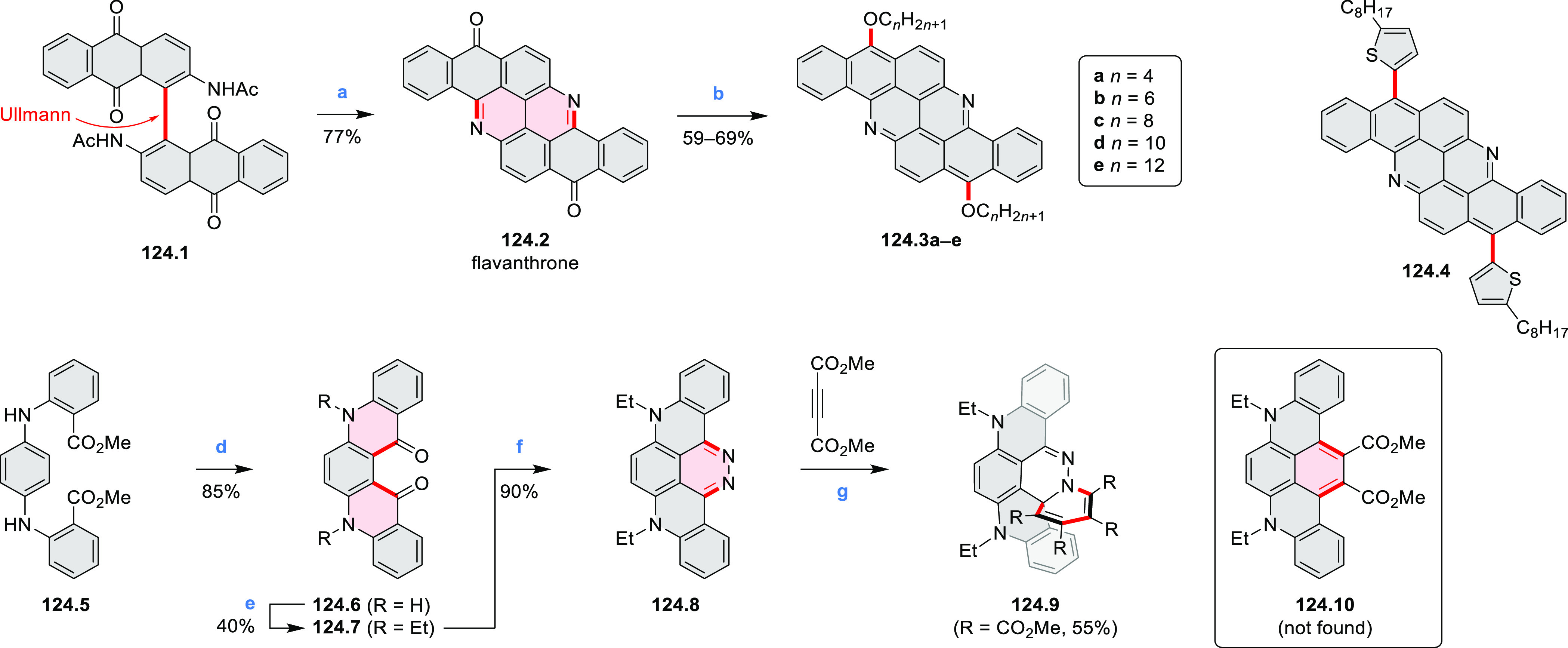
Diaza- and Tetraazapyrenoids from Condensation of Ketone Precursors Reagents and conditions: (a)^251^ aq. HCl, reflux, 24 h; (b) (1) Na_2_S_2_O_4_, NaOH, H_2_O, 60 °C, 1 h, (2) C_*n*_H_2*n*+1_Br, Aliquat 336, toluene, 90 °C, 24 h; (c)^252^ (1) 2-octylthiophene and *n*-BuLi (premixed in Et_2_O at −78 °C for 1.5 h), −78 °C, then rt, overnight, (2) SnCl_2_·H_2_O, rt, 3 h; (d)^253^ MsOH, 150 °C, 16 h; (e) (1) NaH, DMF, rt, 2 h, (2) EtI, rt, 24 h; (f) N_2_H_4_·H_2_O, EtOH, 130 °C, 72 h; (g) toluene, 120 °C, 48 h.

In 2016, Monkman and Bujak et al. reported flavanthrone-derived dyes **124.3a**–**e** for application in organic electronics (Scheme 124).^251^ Flavanthrone **124.2** (see CR2017, Section 4.2 for historical developments of its chemistry) was obtained through the acid-mediated 2-fold cyclization of compound **124.1**, which in turn was obtained via Ullmann reaction of the bromo-substituted precursor. Next, **124.2** was reduced by Na_2_S_2_O_4_ in the presence of NaOH, followed by alkylation with alkyl bromides under phase-transfer conditions in one pot. The five dialkoxy-substituted products **124.3a**–**e** were isolated in 59–69% yield. STM images showed that the dioctyloxy-substituted derivative **124.3c** self-assembled in monolayers deposited on a highly oriented pyrolytic graphite (HOPG) surface. Among compounds **124.3a**,**c**,**e**, which show electroluminescence in a guest/host-type OLED, **124.3c** displayed a luminance value approaching 1900 cd m^–2^ and the highest luminous efficiency (>3 cd A^–1^). Later, the dithienyl-substituted analogue **124.4** was studied by Bujak et al. as a potential luminophore.^252^

A selective synthesis of the fused phthalazine derivative **124.8** (Scheme 124) was developed by Zhu and Zhang.^253^ Upon heating the known diester **124.5** in methanesulfonic acid, the 2-fold Friedel–Crafts acylation took place regioselectively to give the angularly fused product **124.6** rather than the isomeric, linearly fused quinacridone skeleton. Compound **124.6** was ethylated at both NH groups and condensed with hydrazine to yield the tetraazapyrenoid **124.8** in 36% over two steps. A [4 + 2] cycloaddition–elimination sequence involving the pyridazine ring in **124.8** and dimethyl acetylenedicarboxylate was envisaged to provide compound **124.10**. However, only the [2 + 2 + 2] cycloadduct **124.9** was isolated in this reaction.

**Scheme 125:**
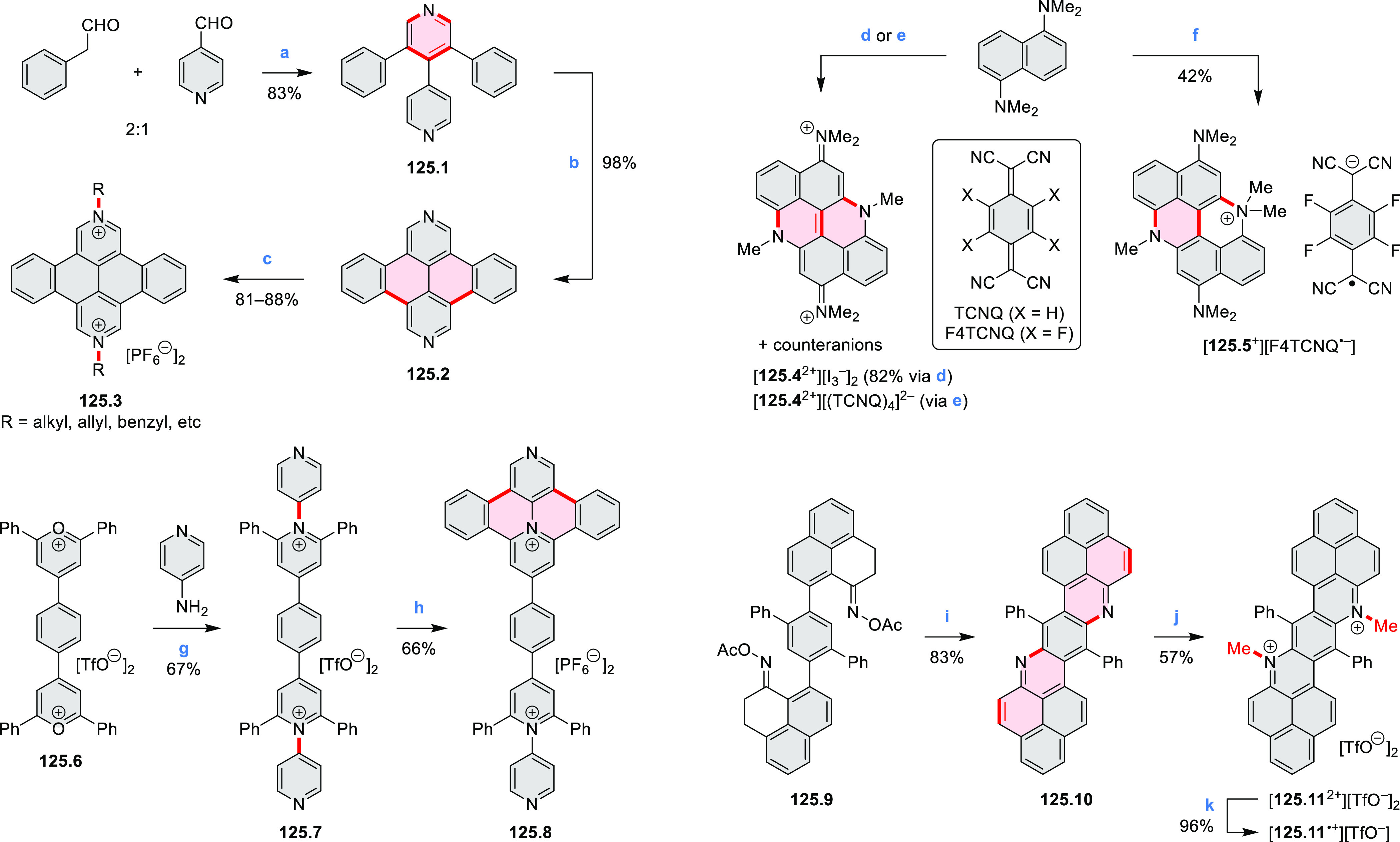
Cationic Azapyrenoids Reagents and conditions: (a)^254^ (1) KOH, EtOH, 0 °C, rt, 26 h, (2) NH_2_OH·HCl, reflux, 2 h; (b) 5% HCl, *hν* (300 nm), air, rt, 24 h; (c) (1) MeI or RBr, DMF, 100 °C, 48 h, (2) KPF_4_, H_2_O, rt; (d)^255^ I_2_, pyridine/1,4-dioxane, rt, 12 h; (e) TCNQ, MeCN, reflux, 20 h; (f) 2,3,5,6-tetrafluoro-7,7,8,8-tetracyanoquinodimethane, MeCN, reflux, overnight; (g)^256^ (1) DMF, argon, 60 °C, 2 h, (2) toluene, Dean–Stark apparatus, 140 °C, 6 h; (h) (1) MeCN, O_2_, *hν* (300–400 nm), 14 h, (2) NH_4_PF_6_; (i)^257^ Fe(acac)_3_, AcOH, 90 °C, 12 h; (j) MeOTf, 1,2-DCE, 50 °C, 16 h; (k) Et_3_N, MeCN/DCM, argon, rt, 2 h.

In 2017, Wei and Li et al. reported a family of viologens (*N*,*N*′-disubstituted 4,4′-bipyridinium salts) in which the pyridyl rings are connected by two *ortho*-phenylene linkages (Scheme 125).^254^ First, phenylacetaldehyde and pyridine-4-carboxaldehyde underwent base-catalyzed condensation in a 2:1 stoichiometry to give a 1,5-dialdehyde intermediate, which was then condensed with hydroxylamine to generate the new pyridine ring in **125.1**. Owing to the electron-poor nature of **125.1**, subsequent cyclodehydrogenation did not take place when DDQ was used as the oxidant. Instead, the Mallory photocyclization worked smoothly to furnish the doubly cyclized product **125.2** in 98% yield. Reactions of **125.2** with various alkyl halides, followed by salt metathesis, gave ten viologens **125.3** in the form of hexafluorophosphate salts in 81–88% yield. All new viologens showed intense blue emission in the range of 421–453 nm.

In 2020, Wallis et al. showed that the diiminium species **125.4**^2+^ can be obtained by oxidative dimerization of 1,5-bis(dimethylamino)naphthalene (Scheme 125).^255^ This transformation is comprised of two types of bond formation, namely, the intermolecular C–C coupling of the carbon atoms *para* to the dimethylamino groups and the intramolecular C–N coupling that generates the new hexagonal rings in **125.4**^2+^. The bis(triiodide) salt [**125.4**^2+^][I_3_^–^]_2_ was obtained with diiodine as the oxidant at rt, whereas [**125.4**^2+^][(TCNQ)_4_]^2–^ was obtained using tetracyanoquinodimethane (TCNQ) as the oxidant in refluxing acetonitrile. Interestingly, 1,5-bis(dimethylamino)naphthalene was oxidized by 2,3,5,6-tetrafluoro-7,7,8,8-tetracyanoquinodimethane (F4TCNQ) to give the monocationic quaternary ammonium salt [**125.5^+^**][F4TCNQ^•–^]. All three salts were unambiguously confirmed by X-ray crystallography. In particular, the negative charge carried by TCNQ in the solid state of [**125.4**^2+^][(TCNQ)_4_]^2–^ was estimated from bond length measurements. Additionally, the IR spectrum of this salt exhibits two C≡N stretching frequencies at 2185 and 2154 cm^–1^.

Hromadová, Kolivoška, and Valášek et al. reported the cationic diazapyrenoid **125.8** in which one positively charged nitrogen center occupies an interior position of the π-skeleton (Scheme 125).^256^ The known bis(pyrylium) salt **125.6** was doubly condensed with 4-aminopyridine in DMF, followed by azeotropic removal of water. The resulting bis(pyridinium) salt **125.7** was then subjected to Mallory photocyclization, yielding the doubly cyclized product **125.8** with one diazapyrenoid unit. Further ring closures of compound **125.8** could not be achieved either by altering the Mallory reaction conditions or by FeCl_3_-mediated oxidation. Both **125.7** and **125.8** show a reversible two-electron reduction wave centered at −1.0 V, implying a limited effect on the LUMO levels imposed by the double cyclization. This observation was reflected in DFT data obtained for **125.7** and **125.8** (*E*_LUMO_ = −3.78 and −3.75 eV, respectively).

Chen et al. reported the radical cation **125.11**^•+^ consisting of two azapyrenoid moieties (Scheme 125, cf. Scheme 136).^257^ The key step in the synthesis involves the electrophilic cyclization and dehydrogenative aromatization of the bis(*O-*acetyl oxime) **125.9** in the presence of iron(III) acetylacetonate. The resulting polycyclic arene **125.10** was isolated in 83% yield. 2-Fold methylation of **125.10** with methyl triflate gave the dicationic species **125.11**^2+^ as the triflate salt. Single-electron reduction of this dication with triethylamine quantitatively afforded the radical cation salt [**125.11**^•+^][TfO^−^] which possessed appreciable air stability (>7 days) in the solid form. This salt was characterized by X-ray crystallography and EPR spectroscopy in the solution and solid states. On photoexcitation, solutions of [**125.11**^•+^][TfO^−^] showed red fluorescence with a quantum yield of 9.3%. Upon dispersion in a PMMA film, [**125.11**^•+^][TfO^−^] fluoresces with a higher quantum yield (19.3%), suggesting an inhibition of the radiationless decay of the excited state. The salt dissolved in DCM showed excellent photostability, with a half-life of 9.5 × 10^4^ s under irradiation at 350 nm.

**Scheme 126:**
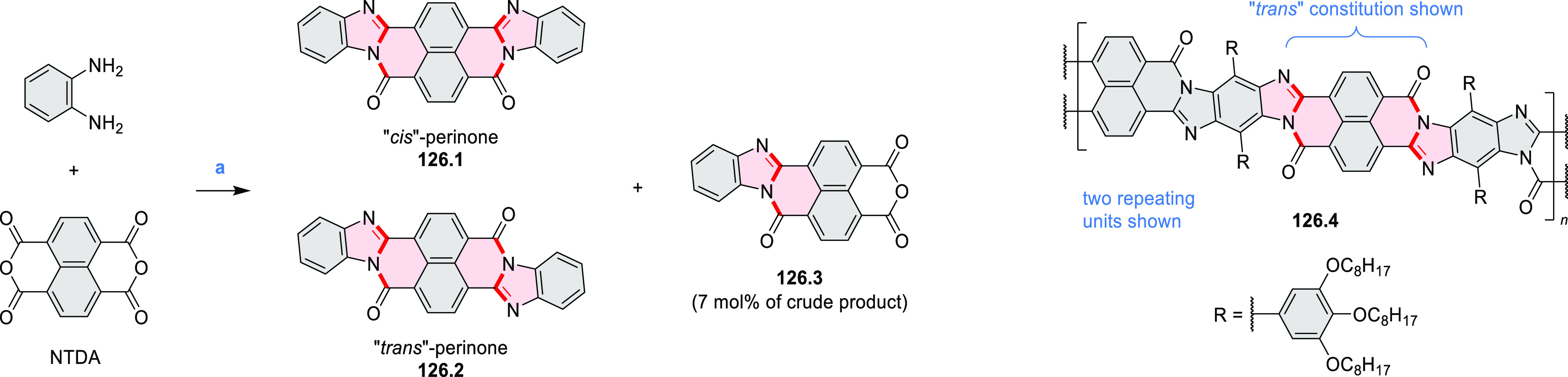
Benzimidazole-Fused Carbonyl-Containing Pyrenoids Reagents and conditions: (a)^258^ H_2_O, 180–250 °C, autoclave or microwave reactor.

In 2018, Unterlass et al. reported a green hydrothermal synthesis of perinones **126.1**–**2** (Scheme 126, cf. CR2017, Section 4.2).^258^ In their protocol, *o*-phenylenediamine and NTDA in a molar ratio of 2:1 were suspended in deionized water in a nonstirred batch autoclave and heated at 200 °C for 16 h. Purification and NMR analysis confirmed the formation both the “*cis*”- and “*trans*”-isomers of perinone (**126.1**–**2**) in a 2:3 ratio. The singly condensed side product **126.3** was also identified, and its emergence could not be avoided under various screened conditions. The reaction time could be shortened to 12 min when the mixture was stirred under microwave heating at 200 °C. The perinone-based conjugated ladder polymer **126.4** was prepared by Wang and Qiao et al. in addition to its perylene-derived congener (see Scheme 96, Section 3.4) and evaluated in bulk heterojunction photodetectors.^199^

**Scheme 127:**
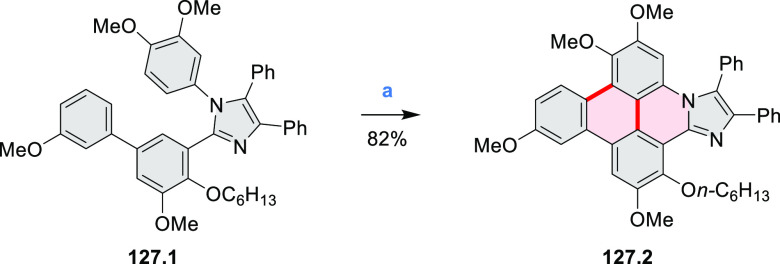
Imidazole-Fused Pyrene via Oxidative Coupling Reagents and conditions: (a)^259^ PIFA, BF_3_·Et_2_O, toluene, rt.

Tetraaryl-substituted imidazole **127.1** bearing electron-donating groups was used by the Gryko group as a precursor to the fully conjugated benzo[*e*]pyrene scaffold **127.2** (Scheme 127).^259^ For this transformation, (bis(trifluoroacetoxy)iodo)benzene was used as the oxidant, which was shown to work well for a range of *ortho*-fused imidazole targets. In the synthesis of **127.2**, 2.5 equiv of the oxidant was sufficient to achieve complete reaction, and no partially fused products were observed, indicating that the first oxidative coupling facilitated the ultimate fusion. The emission spectrum recorded for **127.2** shows three maxima at 400, 422, and 444 nm. The fluorescence quantum yield in acetonitrile was determined to be 32%.

**Scheme 128:**
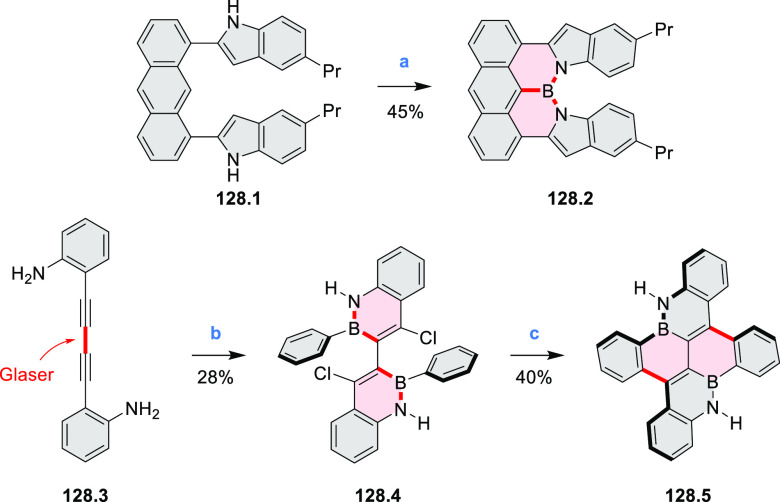
B,N-Doped Pyrenoids Reagents and conditions: (a)^260^ BBr_3_, *i*-Pr_2_EtN, *o*-DCB, 180 °C; (b)^261^ PhBCl_2_, Et_3_N, 1,2,4-trichlorobenzene, reflux, 36 h; (c) Pd(OAc)_2_, PCy_3_, Cs_2_CO_3_, *o*-xylene, reflux, overnight.

An NBN-doped pyrene **128.2** was obtained via electrophilic C–H borylation by Wakamiya and Wang in 2018 (Scheme 128).^260^ This double annulation was also used for construction of related phenalenoid systems (see Scheme 209, Section 5.3.1). Irreversible oxidation peaks were observed for **128.2** in cyclic voltammetry; however, the attempts to construct an NBN-containing seven-membered ring from **128.2** were unsuccessful, which was ascribed to the instability of the anthracene moiety toward oxidants. **128.2** has small band gap of ca. 2.5 eV resulting from a high-lying HOMO and a low-lying LUMO, making the compound potentially suitable for OPV applications.

In 2020, Park and Shin et al. reported the preparation of B_2_N_2_-ixene (**128.5**, Scheme 128).^261^ The key precursor, 2,2′-(buta-1,3-diyne-1,4-diyl)dianiline (**128.3**), was obtained from the Glaser coupling of 2-ethynylaniline. Treatment of **128.3** with PhBCl_2_ and Et_3_N brought about the chloroboration of alkyne followed by B–N bond formation to yield compound **128.4** in 28% yield. Next, palladium-catalyzed cyclization involving the phenyl groups and the C–Cl bonds afforded the target B_2_N_2_-ixene (**128.5**) in 40% yield. In the solid state, **128.5** showed a contorted structure, in which the four peripheral benzene rings deviated from planarity. The molecules packed into a 1D columnar pattern with a short intermolecular distance of 3.685 Å, which may have resulted from favorable dipolar interactions.

**Scheme 129:**
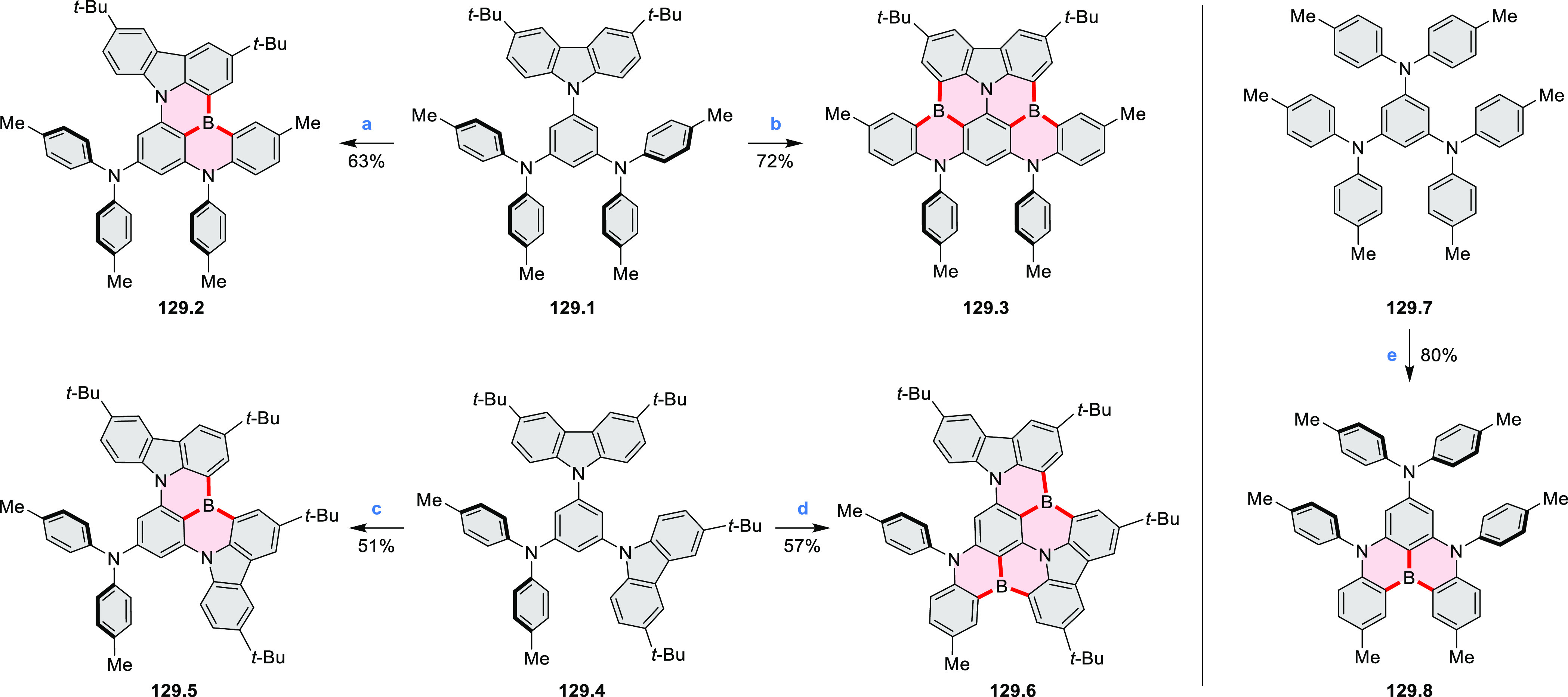
Synthesis of BN-Doped Pyrenoids Reagents and conditions: (a)^262^ 2 equiv of BBr_3_, toluene, reflux, 20 h; (b) 4 equiv of BBr_3_, chlorobenzene, reflux, 20 h; (c) 12 equiv of BBr_3_, benzene, reflux, 20 h; (d) 24 equiv of BBr_3_, chlorobenzene, reflux, 20 h; (e) 1 equiv of BBr_3_, *o*-dichlorobenzene, 170 °C, 20 h.

Electrophilic borylation with BBr_3_ was used by Hatakeyama and co-workers to prepare various BN-doped polycyclic aromatics (Scheme 129).^262^ In **129.1**, boron was selectively introduced at the central benzene, *ortho* to the carbazolyl substituent, where the highest HOMO amplitude was predicted by DFT. The product of single borylation (**129.2**) was obtained with 2 equiv of BBr_3_ in refluxing toluene. With a greater excess of BBr_3_ and higher temperature, double borylation to **129.3** was also accomplished. The starting material **129.4** bearing two carbazolyl groups reacted in a similar manner, providing **129.5**–**6**, respectively. For compound **129.7** with three diarylamine substituents, relatively harsh conditions (170 °C) were needed to establish a single *peri* fusion through borylation, giving **129.8** in 80% yield. All fused products shown here, as well as their differently substituted variants, were characterized by strong, narrowband photoluminescence, with quantum yields of 80–88% and fwhm of 26–39 nm in the PMMA matrix. Emission maxima were found in the 453–504 nm range, making these borylated derivatives suitable for fabrication of deep blue, sky blue, and green OLEDs. Products of double borylation (**129.3** and **129.6**) displayed red-shifted emission relative to their singly borylated counterparts, which was attributed to π-extension.

**Scheme 130:**
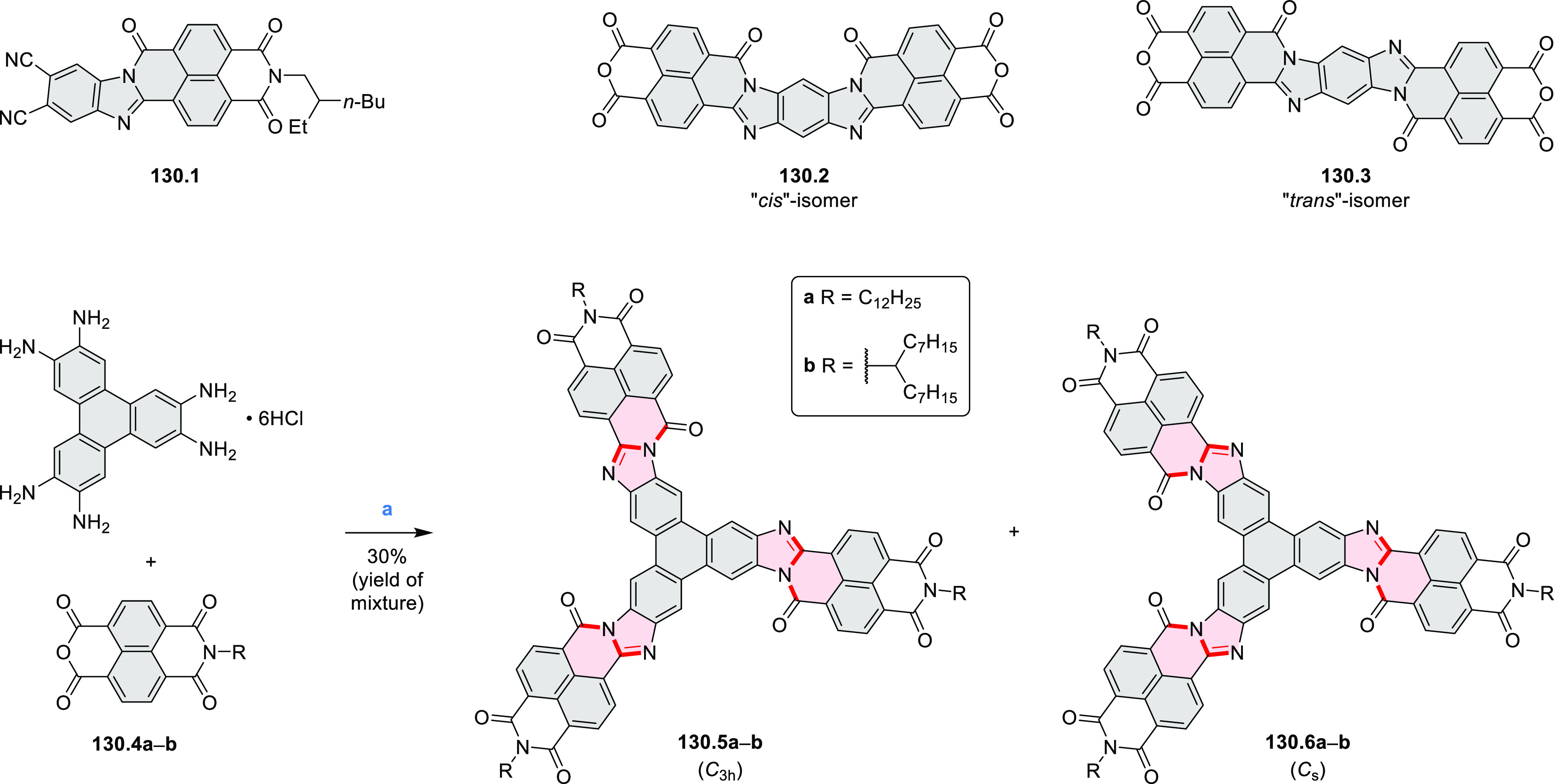
Benzimidazole-Fused π-Systems Derived from Naphthalene Monoimide Monoanhydride Reagents and conditions: (a)^263,264^ (1) Et_3_N, quinoline, rt, 30 min, (2) **130.2a**/**b**, Zn(OAc)_2_, 180 °C, overnight.

Condensations of NTDA and related monoimides with aromatic oligoamines provided access to a variety of perinone analogues, such as the dicyano-substituted **130.1**(193) or the isomeric dianhydrides **130.2**–**3**^196^ (Scheme 130, for syntheses of the corresponding perylene-based analogues **94.2** and **95.2**; see Section 3.4). Liu, Ma, and Navarro et al. explored the disk-shaped triphenylene-tris(naphthaleneimidazole)s **130.5**–**6** as n-type semiconductors (Scheme 130).^263,264^ These compounds were synthesized through the condensation of 2,3,6,7,10,11-triphenylenehexaamine with the naphthalene monoimide monoanhydrides **130.4a**,**b**. In each case, the *C*_3*h*_-symmetrical (**130.5**) and the *C*_*s*_-symmetrical (**130.6**) products were obtained as a mixture. In OFET devices, the average and maximum electron mobilities for the annealed thin films of the dodecyl analogues (**130.5a**,**6a**) were 1.0 × 10^–3^ and 1.3 × 10^–3^ cm^2^ V^–1^ s^–1^, respectively. Both values are at least 1 order of magnitude larger than those (8.5 × 10^–5^ and 1.3 × 10^–4^ cm^2^ V^–1^ s^–1^) of the branched alkyl analogues (**130.5b**,**6b**). The linear alkyl side chains were believed to facilitate molecular organization, thereby enhancing electron transport in the thin films.

**Scheme 131:**
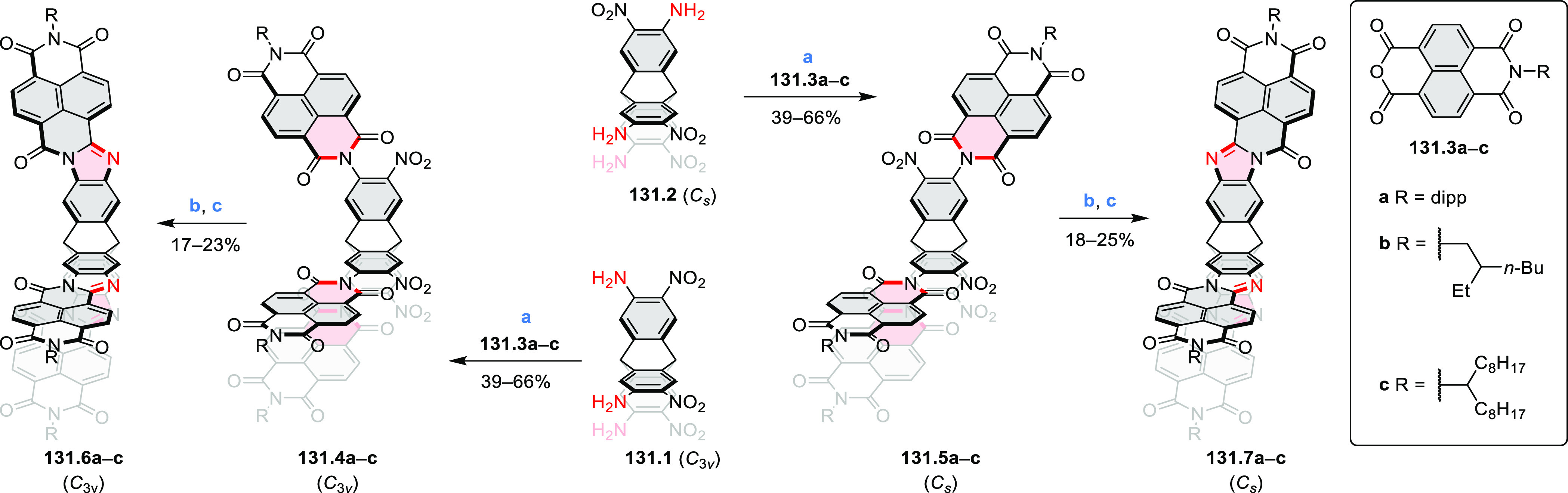
Synthesis of *C*_*s*_- and *C*_3*v*_-Symmetric π-Extended Triptycene Derivatives Reagents and conditions: (a)^265,266^ Zn(OAc)_2_·2H_2_O, quinoline, 180 °C, 24 h; (b) SnCl_2_·2H_2_O, EtOAc, 85 °C, 12 h; (c) DMF (for **a**) or Zn(OAc)_2_·2H_2_O, quinoline (for **b** and **c**), 140 °C, 72 h.

Mastalerz and Vaynzof et al. reported the design and synthesis of triptycene-triaroylenimidazoles (TTAIs) **131.6**–**7**, which constitute a class of three-dimensional acceptor molecules (Scheme 131).^265,266^ The synthetic pathway is based on the *C*_3*v*_-symmetrical triaminotrinitro-substituted triptycene **131.1** and its *C*_*s*_-symmetrical isomer **131.2**. Both molecules were obtained from the corresponding triptycenetriamines via an acetylation–nitration–deacetylation sequence. Compounds **131.1**–**2** underwent 3-fold condensation with **131.3a**–**c** in quinoline at 180 °C in the presence of Zn(OAc)_2_ to give **131.4a**–**c** (*C*_3*v*_) and **131.5a**–**c** (*C*_*s*_) in 39–66% yield. The nitro groups in these compounds were then reduced by SnCl_2_ to amino groups, and then the crude products were subjected to intramolecular condensation to furnish the target TTAIs **131.6a**–**c** (*C*_3*v*_) and **131.7a**–**c** (*C*_*s*_) with different symmetries. This stepwise condensation approach does not result in the formation of isomers (compare with Scheme 126). Organic solar cells were fabricated using each TTAI and the donor polymer PTB7 in 1:1 ratio. The photovoltaic performance of the TTAIs was found to be insensitive to molecular symmetry, but it was dependent on the solubilizing groups. For the *C*_3*v*_-symmetrical TTAIs (**131.6a**−**c**), the maximum PCEs were 0.90%, 2.97%, and 2.76%, respectively. The *C*_*s*_-symmetrical series (**131.7a**–**c**) exhibited the same trend. The high open-circuit voltage values (*V*_OC_) determined for these photovoltaic devices were 0.15–0.2 V higher than those for PTB7–fullerene-based devices.

### 4.3 Other Heterapyrenoids

**Scheme 132:**
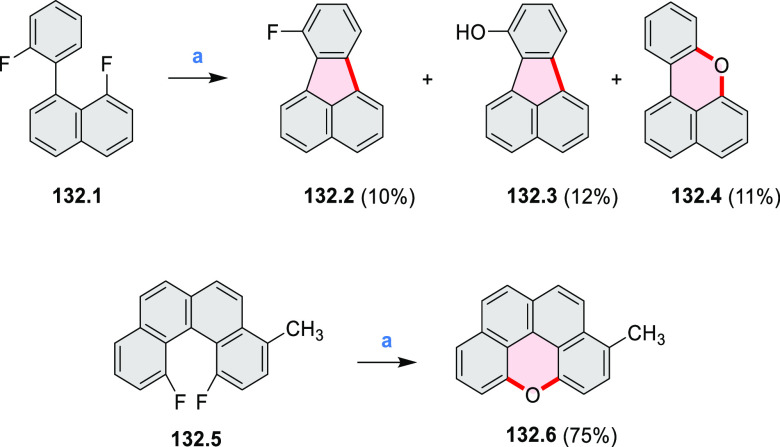
Fused Xanthenes via Alumina-Promoted Oxodefluorination Reagents and conditions: (a)^267^ γ-Al_2_O_3_, 190 °C, 12 h.

Fluorinated biphenyls can be converted into dibenzofurans and fused xanthenes using an alumina-promoted oxodefluorination reported by Akhmetov et al. in 2020 (Scheme 132).^267^ The reaction occurred on the surface of activated γ-Al_2_O_3_ and did not require other reagents. An attempt to convert **132.1** into **132.4** using an analogous approach showed low selectivity with pentannulation products being formed alongside the target xanthene derivative. However, **132.5** with two fluorine atoms in the cove region was efficiently transformed into **132.6**, providing access to the elusive naphtho[2,1,8,7-*klmn*]xanthene core.

**Scheme 133:**
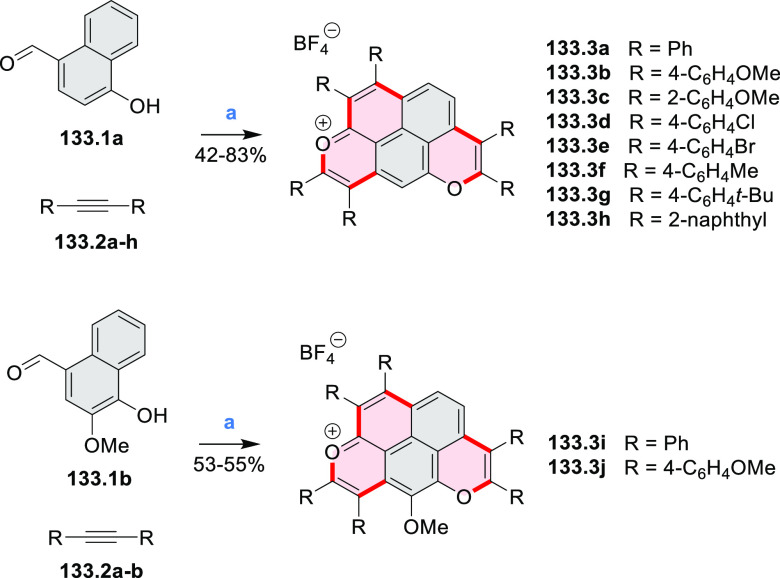
Synthesis of Pyrylium-Fused Oxapyrenes Reagents and conditions: (a)^268^ [Cp*RhCl_2_]_2_, Cu(OAc)_2_, NaBF_4_, THF, 150 °C, 24 h.

A single-step triple annulation was used to prepare pyrylium-containing oxapyrenoids **133.3a**–**j** from simple precursors (Scheme 133).^268^ The products were obtained in moderate to high yields from 4-formyl-1-naphthols **133.1a**,**b** and symmetric diarylalkynes **133.2a**–**h** in the presence of a rhodium catalyst and Cu(OAc)_2_ acting as a stoichiometric oxidant. The multistep mechanism proposed for this transformation consists of three distinct cyclizations. Each of these stages involves an O*-*directed C–H activation by the rhodium catalyst, followed by insertion of alkyne into the resulting rhodacycle. Remarkably, different products were obtained by simply using NH_4_BF_4_ as an additive instead of NaBF_4_. The ammonium nitrogen was incorporated into the products in the place of the aldehyde-derived oxygen, leading to pyridinium-fused oxaphenalenes (Scheme 196, Section 5.1.3). Compounds **133.3a**–**j** as well as phenalenoids **196.1a**,**b** and **196.2a**–**d** were emissive, with luminescence quantum yields of 35–69%. Emission wavelength in this series of compounds varied from 470 nm in **196.1a** to 623 nm in the most electron-rich pyrenoid **133.3j**. A selection of pyrylium and pyridinium products were tested as fluorescent probes for use in cell cultures. These compounds permeated into cells and localized in the mitochondria of HepG2 cells. Compound **133.3a** displayed the best performance in terms of high photostability and low cytotoxicity.

**Scheme 134:**
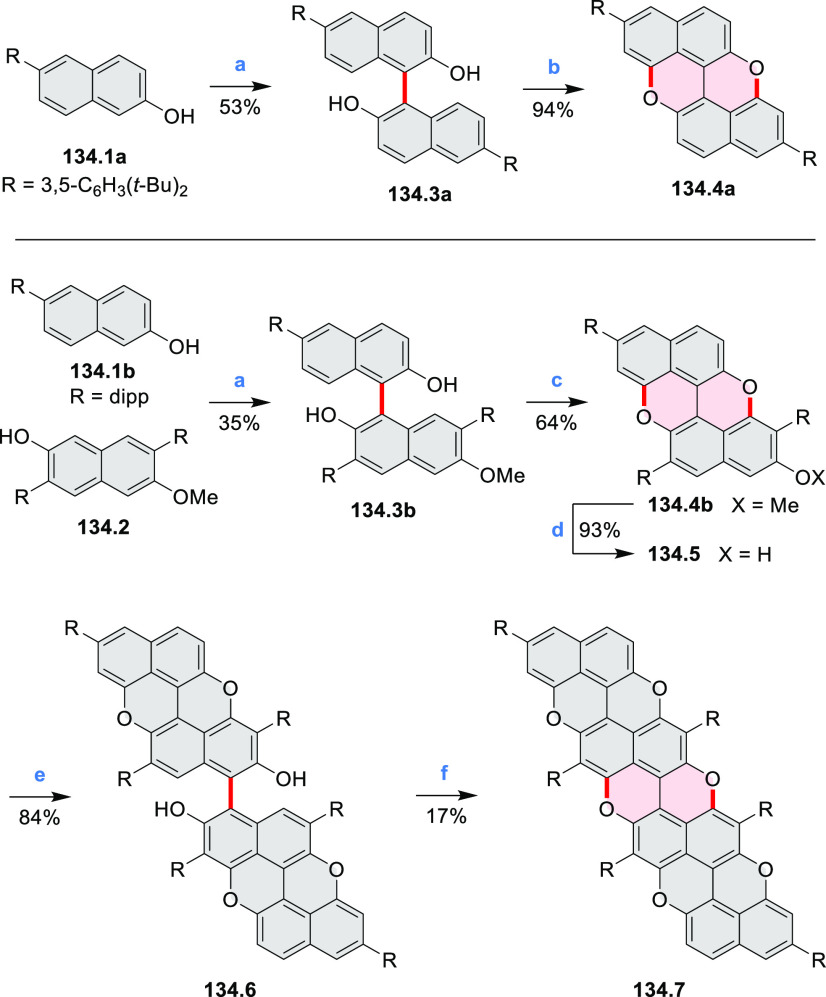
Synthesis of Zig-Zag O-Doped *peri*-Acenoacenes Reagents and conditions: (a)^269^ CuCl_2_, 1-phenylethan-1-amine, MeOH, DCM, 0 °C to rt, 2 h; (b) CuI, pivalic acid, DMSO, 130 °C, 2 h; (c)^82^ CuCl, K_2_CO_3_, *N-*methylimidazole, 120 °C, 20 h, 64%; (d) *n*-dodecanethiol, NaOH, NMP, 130 °C, 4 h, 93%; (e) Cu-TMEDA, DCM, rt, 5 min; (f) CuCl, K_2_CO_3_, *N-*methylimidazole, 120 °C, 20 h.

In 2018, Bonifazi reported the first synthesis of a chemically and thermally stable, O*-*doped zigzag-edged nanoribbon **134.7** (Scheme 134).^269^ A similar strategy was employed as in the synthesis of O*-*doped benzorylenes such as **134.4a** (see Scheme 50, Section 3.1.3).^82^ The synthetic procedure started with a C–C oxidative heterodimerization of **134.1b** and **134.2**, which gave the racemic binaphthol **134.3b** in 35% yield. Surprisingly, oxidative O*-*annulation of **134.3b** following a procedure successfully applied in the synthesis of **134.4a**(82) failed, yielding the desired product in only 7% yield along with extensive degradation. However, under alternative conditions with a catalytic amount of CuCl, K_2_CO_3_, and *N-*methylimidazole in *m*-xylene at 120 °C, the *peri*-xanthenoxanthene (PXX) **134.4b** was obtained in 64% yield and was subsequently demethylated using *n*-dodecanethiol to provide **134.5**. This compound was then homodimerized into **134.6** in the presence of a Cu–TMEDA catalyst. The final oxidative ring-closure reaction gave the desired zigzag nanoribbon **134.7** in 17% yield. The Cu(I)-mediated cyclization methodology shown here is an alternative to the earlier approach using a Hg(II) oxidant.^270^ Photophysical and electrochemical data showed that the extension of the **134.4** PXX core into the molecular ribbon **134.7** reduced the band gap. This change was caused by an increase of the HOMO energy from −5.14 eV in **134.4** to −4.74 eV in **134.7**, which is a desirable feature for prospective p-type organic semiconductors. Compound **134.7** had a yellow emission (λ_max_ = 540 nm, Φ = 38%), which was red-shifted relative to **134.4b** (λ_max_ = 457 nm, Φ = 48%).

**Scheme 135:**
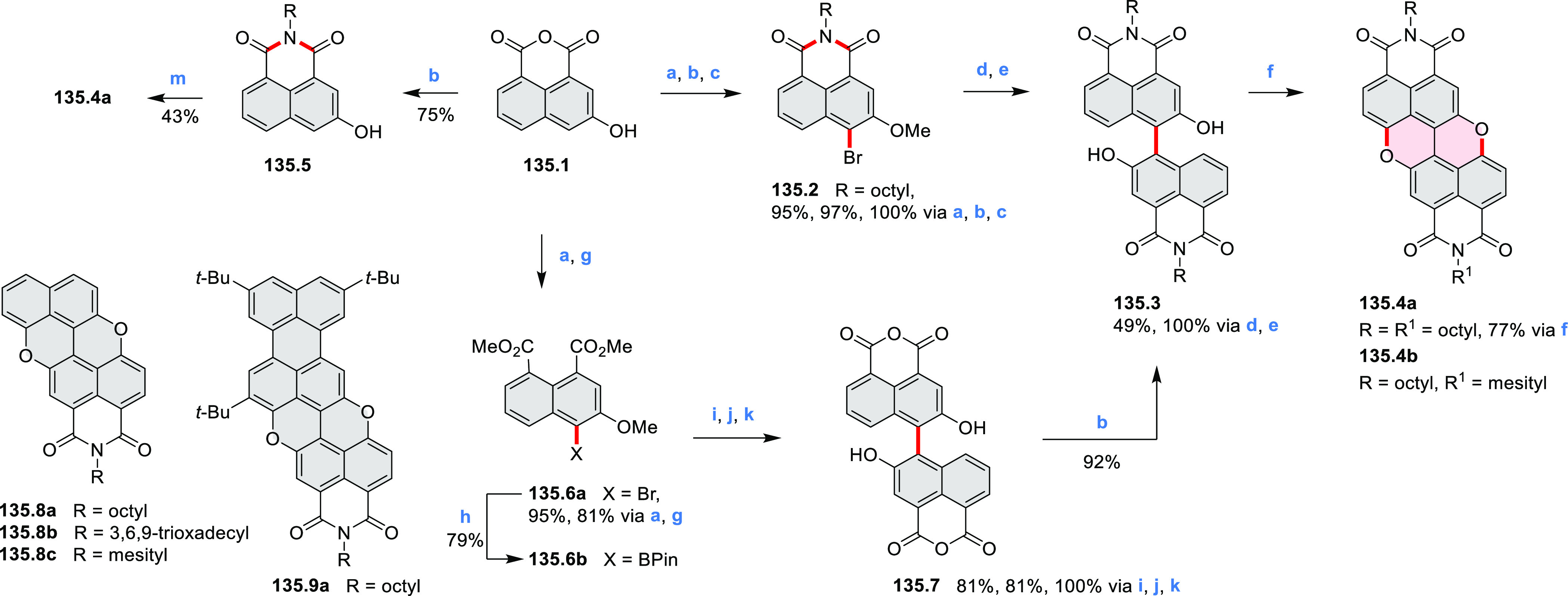
Synthetic Strategies Toward the Preparation of *peri*-Xanthenoxanthene Imides Reagents and conditions: (a)^271,272^ Br_2_, dioxane, reflux, 2.5 h; (b) *i*-Pr_2_NEt, *n*-octylamine, dioxane, reflux, 16–24 h; (c) K_2_CO_3_, CH_3_I, MeCN, reflux, 4 h; (d) Cs_2_CO_3_, B_2_Pin_2_, Pd(dba)_2_, SPhos, dioxane, reflux, 18 h; (e) BBr_3_, DCM, 0 °C to rt, 16 h; (f) pivalic acid, CuI, DMSO, 120 °C, 5 h; (g) DBU, MeI, MeOH, reflux, 18 h; (h) B_2_Pin_2_, KOAc, Pd(PPh_3_)_2_Cl_2_, 1,4-dioxane, reflux, 16 h; (i) **135.6a**, **135.6b**, K_3_PO_4_, Pd(dba)_2_, SPhos, 1,4-dioxane, H_2_O, reflux; (j) (1) KOH, *i*-PrOH, reflux, 13 h, (2) HCl(aq.), AcOH, reflux, 24 h; (k) HBr(aq.), AcOH, 126 °C, 24 h; (m) CuCl, DMSO, 120 °C, 24 h, air.

Frontier orbital energy levels of the O*-*doped anthranthrene core (**134.4a**, Scheme 134) can be tuned by introducing electron-withdrawing imide groups (Scheme 135).^271^ Regioselective bromination of the 1,8-naphthalic anhydride hydroxyl derivative **135.1** (95%) followed by imidization (97%) and methylation (100%) gave **135.2** in an overall excellent yield. **135.2** was then subjected to in situ Miyaura borylation and Suzuki coupling, providing **135.3** after demethylation. The final ring closure was achieved by a Cu-promoted oxidative etherification, which gave good yields despite the presence of electron-withdrawing imide groups. The same approach was utilized in the synthesis of **135.8a** bearing one *N-*octylimide functionality. Excited-state redox potentials of **135.4a** and **135.8a** were comparable to those of organometallic photocatalysts such as [Ru(bpy)_3_]^2+^ and [Ir(ppy)_3_]. These compounds were thus successfully used to promote a photoredox dehalogenation of several alkyl and aryl halides in the presence of DIPEA as a stoichiometric reducing agent.

To further investigate the synthetic methods toward the *peri*-xanthenoxanthene diimide **135.4a**, a direct oxidative Pummerer dimerization was examined (Scheme 135).^272^ The homocoupling of **135.5** was done in up to 43% yield in the presence of CuCl in DMSO under air. An alternative pathway toward **135.4a** and related compounds was also described in the same study. In this approach, imidization was performed in the penultimate step (such as from the dimeric anhydride **135.3**), allowing for convenient introduction of various N*-*substituents. Compound **135.4b** with differentially substituted imides was also prepared. Moreover, monoimides **135.8a**–**c** and the *peri*-π-extended analogue **135.9a** were obtained by the same approach.

**Scheme 136:**
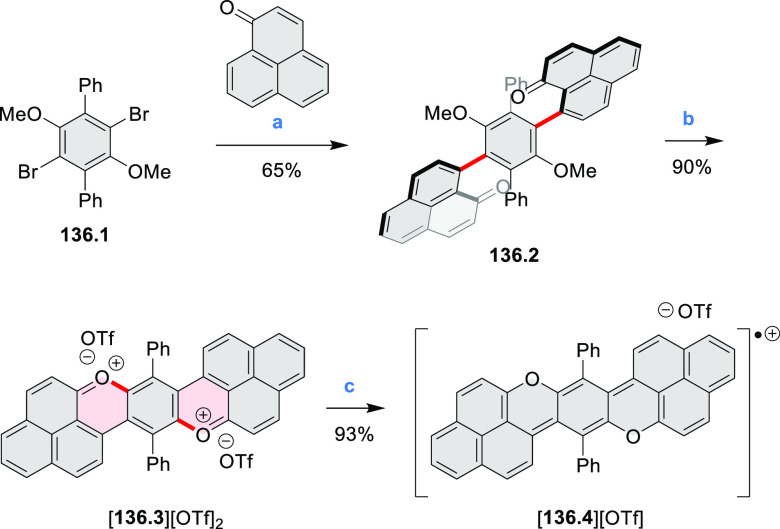
Synthesis of π-Radical Cation **[136.4]**^**•**^^+^ Reagents and conditions: (a) *s*-BuLi, TMEDA, THF, −78 °C, 45 min, then phenalenone, −78 °C to rt, overnight;^273^ (b) TfOH, 1,1,2,2-tetrachloroethane, 120 °C, 12 h; (c) Na_2_S_2_O_4_, MeCN, rt, 2 h.

In 2019, Chen and co-workers reported the synthesis of a bisphenalenyl radical cation [**136.3**]^•+^ (Scheme 136).^273^ Treatment with triflic acid caused demethylation of the methoxy groups in **136.2**, which was followed by cyclization to generate the dipyrylium salt [**136.3**]OTf_2_ after stirring in tetrachloroethane at 120 °C for 12 h. Reduction of the dication [**136.3**]^2+^ in the presence of either strong reducing reagents (i.e., Na, K, and Zn) or mild ones (e.g., sodium dithionite) provided the π-radical cation [**136.3**]^•+^, while the corresponding neutral species could not be obtained. The electronically stabilized π-radical cation [**136.3**]^•+^ shows multiple short intermolecular contacts (<3.4 Å) in its X-ray crystal structure. DFT simulations revealed that these close π–π interactions enabled intermolecular spin–spin coupling, implying that [**136.3**]^•+^ achieved electrostatically enhanced intermolecular covalent bonding interactions in two dimensions. Single-crystal devices fabricated from [**136.3**]^•+^ demonstrated average electrical conductivities of 1.31 × 10^–2^ S/cm.

**Chart 14:**
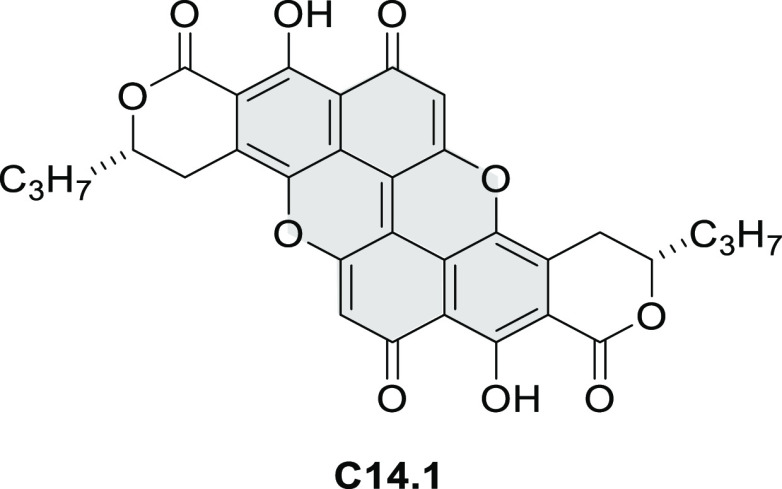
Xylindein

The natural product xylindein **C14.1** from the wood-staining fungi, *Chlorociboria aeruginosa* (Chart 14, see CR2017, Section 4.3), is of potential interest as a fluorescent probe as well as an organic semiconductor. Its problematic purification was addressed by Remcho, who developed a centrifugal partition chromatography (CPC) method utilizing a new biphasic solvent system for the preparative-scale isolation and purification of **C14.1**.^274^ Electronic properties of **C14.1** for potential use in optoelectronic devices were explored by Ostroverkhova and co-workers (Chart 14).^275,276^ Aggregation of xylindein in THF was detected in the 8–180 μM concentration range by the emergence of a NIR absorption band at 720 nm. This band was thought to arise from a combination of hydrogen bonding and π-stacking interactions. Photoresponsive conductive films were prepared from xylindein and from its blends with PMMA or crystalline nanocellulose.

**Scheme 137:**
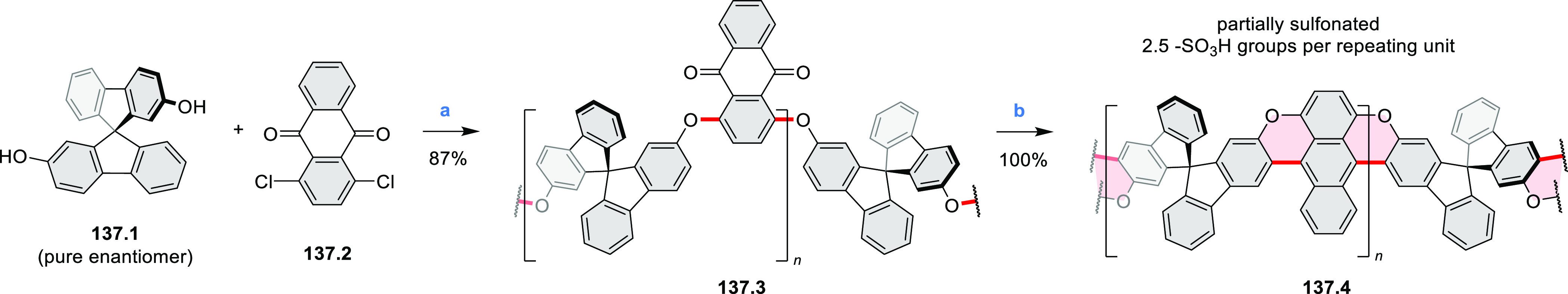
Synthesis of a Screw-Shaped Polymer Reagents and conditions: (a)^64^ K_2_CO_3_, diphenyl sulfone, 210 °C, 5 h; (b) (1) H_2_SO_4_, 160 °C, 6 h, (2) tetrabutylammonium iodide, 120 °C, 6 h.

In 2015, Takata and co-workers developed a homochiral helical polymer consisting of alternating spirobifluorene and dioxapyrenoid moieties (Scheme 137).^64^ First, the poly(aryl ether) precursor **137.3** was synthesized through polycondensation of the optically pure spirobifluorene **137.1** and the anthraquinone spacer **137.2**. Next, a postpolymerization cyclization was done using concentrated H_2_SO_4_ followed by addition of an iodide salt as a reducing agent. In contrast to related polymers with rigid structures, the resulting **137.4** was highly soluble in water because of incidental introduction of sulfonyl groups into the polymer backbone. The sulfonation ratio was estimated to be 2.5 per repeating according to elemental analysis. The isomeric polymer **36.3** with a dioxaperylene substructure was synthesized using the same approach (see Scheme 36, Section 3.1.2).

**Scheme 138:**
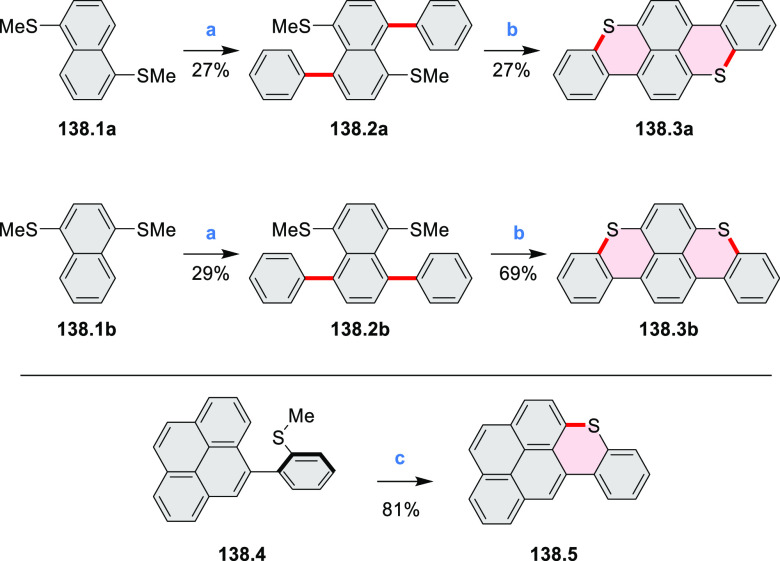
Synthesis of Dithiapyrenoids and Pyrene-Containing Thioxanthene Reagents and conditions: (a)^277^ phenylboronic acid neopentylglycol ester, [Cp*Rh(MeCN)_3_][SbF_6_]_2_, Ag_2_O, *t*-AmOH, 120 °C, 48 h; (b) (1) *m*-CPBA, DCM, rt, (2) TfOH, 1,2-DCE, rt, (3) pyridine, water; (c)^67^ I_2_, CHCl_3_, 1 h at 70 °C, 1 h at 80 °C, 22 h at 90 °C.

Miura and co-workers developed a new rhodium-catalyzed C–H activation method for a direct arylation of naphthalene derivatives bearing thioether groups.^277^ The coupling products **138.2a**,**b** were readily transformed into the corresponding S*-*doped polyaromatics **138.3a**,**b** (Scheme 138). This process involved oxidation of the thioethers to sulfoxides upon treatment with *m*-CPBA, followed by cyclization in the presence of acid. The same approach was used to prepare sulfur-containing π-extended phenalenoids (Scheme 197, Section 5.1.3). In a related example, the pyrene-containing thioxanthene **138.5** was obtained via a new strategy of extending the pyrene core from its K region (Scheme 138).^67^ The final ring annulation was achieved by treating the *o*-methylthio precursor **138.4** with an excess of iodine in chloroform at an elevated temperature. A product of double cyclization was similarly obtained (Scheme 38, Section 3.1.2). **138.5** was almost perfectly planar in the solid state, with a torsion angle of only 1.43° between the pyrene and benzene fragments. The molecules of **138.5** were arranged into columnar stacks with a π–π distance of 3.554 Å.

**Scheme 139:**
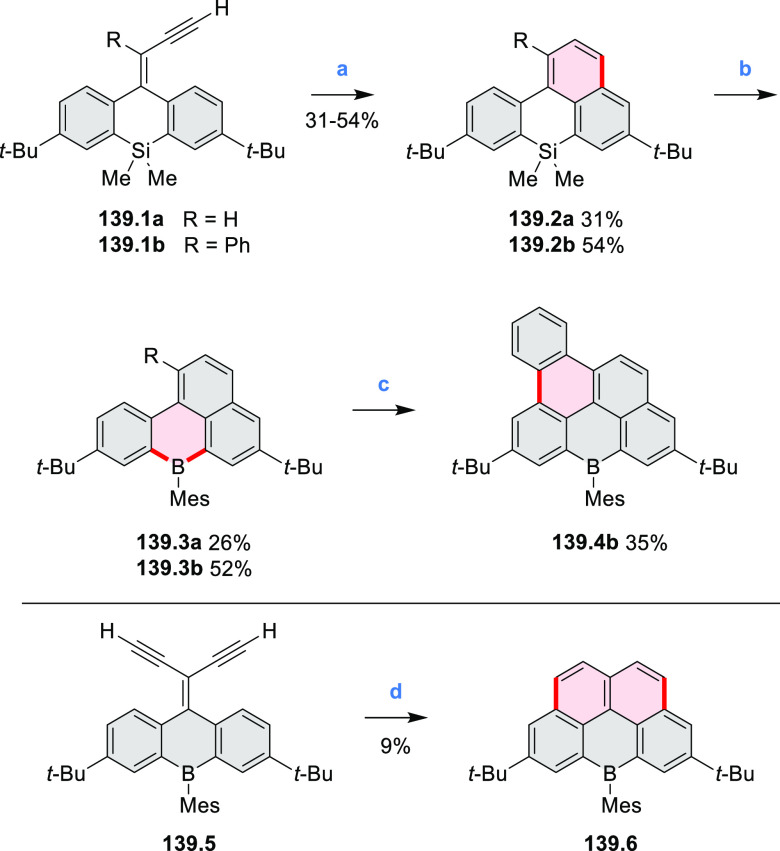
Synthesis of Polycyclic Conjugated Arylboranes Reagents and conditions: (a)^278^ PPh_3_Ru(cymene)Cl_2_, NH_4_PF_6_, 1,2-DCE, 75 °C; (b) (1) neat BBr_3_, rt, (2) MesMgBr, THF, rt; (c) I_2_, *hν*, toluene, rt; (d) PPh_3_Ru(cymene)Cl_2_, NH_4_PF_6_, 1,2-DCE, 80 °C.

A series of boron-containing polycyclic aromatic hydrocarbons were synthesized through the ruthenium-catalyzed annulation of arylborane and -silane enynes as the key step (Scheme 139).^278^ A single cyclization was performed to furnish the silicon-containing 7*H*-benzo[*de*]anthracene derivatives **139.2a**,**b** (see also Scheme 193, Section 5.4.1). Double cyclization of an enediyne was also possible, providing the boron-doped 6*H*-benzo[*cd*]pyrene **139.6**. Si/B exchange was achieved by treating **139.2a**,**b** with excess neat BBr_3_ at rt followed by mesitylation. **139.3b** readily undergoes a light-induced conversion to the fully planarized **139.4b** upon irradiation with UV light in the presence of iodine. All the B*-*doped products proved to be benchtop-stable and showed deep blue photoluminescence with quantum yields of 79–91%.

**Scheme 140:**
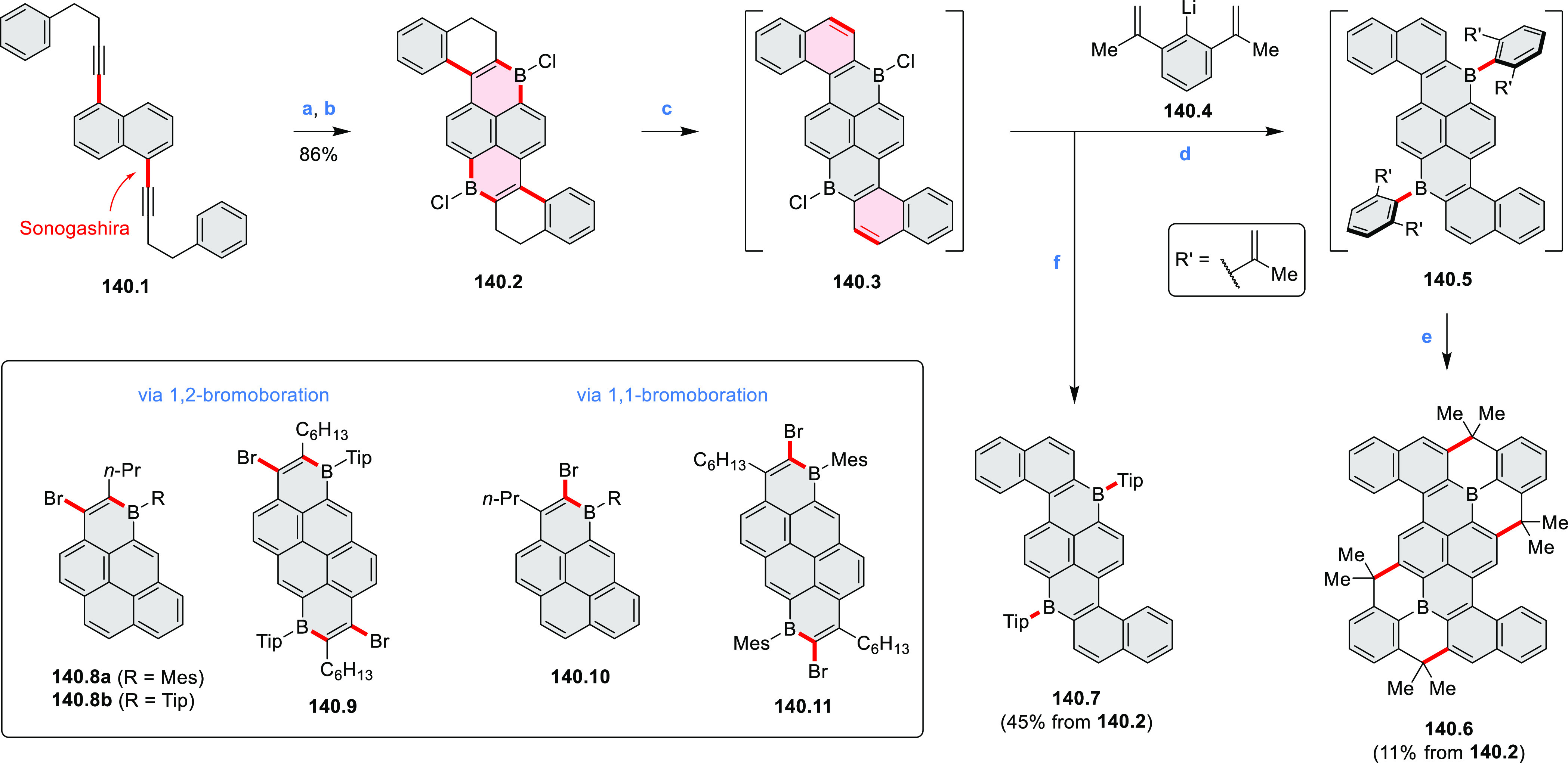
π-Extended Bora- and Diborapyrenes from Alkynes Reagents and conditions: (a)^279^ (1) BCl_3_, 2,4,6-tri-*tert*-butylpyridine, DCM, 5 min, (2) AlCl_3_, 15 min; (b) AlCl_3_, 2,6-dichloropyridine, DCM, 18 h; (c) [Ph_3_C][BF_4_], 2,4,6-tri-*tert*-butylpyridine, DCE, 75 °C, 120 h; (d) toluene, rt, overnight; (e) Sc(OTf)_3_, DCE, 75 °C, 16 h; (f) TipMgBr, toluene, overnight.

In 2017, Ingleson et al. described the preparation of π-extended diborapyrene derivatives **140.5**–**6** (Scheme 140).^279^ In the synthesis, the dialkynyl-substituted naphthalene **140.1** was treated with BCl_3_ and 2,4,6-tri-*tert*-butylpyridine to initiate electrophilic cyclization involving the phenyl groups. The resulting BCl_2_-containing intermediate was reacted with stoichiometric amounts of AlCl_3_ and 2,6-dichloropyridine to induce the C–H borylative cyclization with the naphthalene core to give the diborapyrene derivative **140.2** in 86% yield. Subsequent dehydrogenative aromatization with trityl tetrafluoroborate yielded compound **140.3**, which then reacted with either the aryllithium **140.4** or 2,4,6-triisopropylphenylmagnesium bromide (TipMgBr) to give **140.5** and **140.7**, respectively. The former product **140.5** could undergo 4-fold electrophilic cyclization to give **140.6**. In subsequent work, Ingleson and Zysman-Colman et al. reported the boron-doped π-extended pyrenes **140.8**–**11** through a bromoboration–electrophilic borylation sequence on the corresponding monoalkynyl- and dialkynyl-substituted pyrenes (for details, see Scheme 99, Section 3.4).^202^ A bromine-free analogue of compound **140.9** was also prepared using an alternative approach from an olefinic precursor, as reported by Würthner et al. (cf. Scheme 34, Section 3.1.2).^61^

**Scheme 141:**
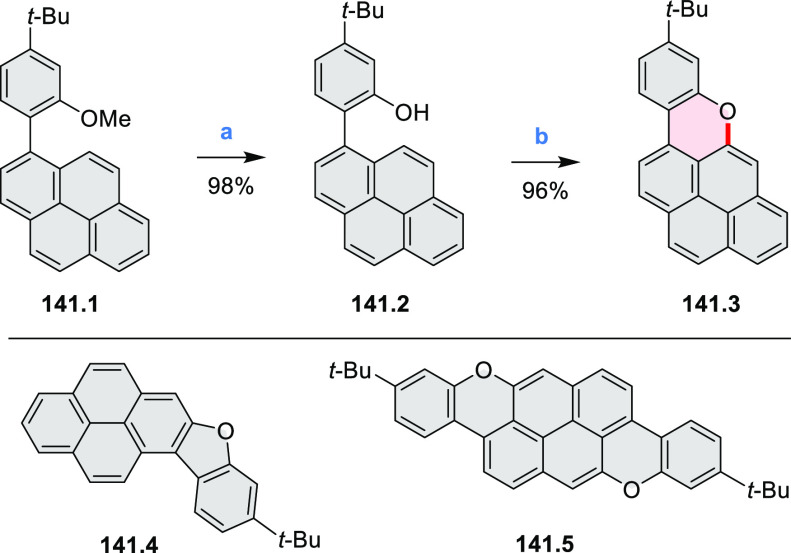
Oxygen-Doped Pyrenoids via Oxidative Pummerer Cyclization Reagents and conditions: (a)^159^ BBr_3_, DCM, 0 °C to rt, overnight; (b) CuO, nitrobenzene, 240 °C, overnight.

The Pummerer oxidative annulation reaction was utilized to extend PAHs through the formation of an intramolecular C–O bond with a suitable phenol substituent (Scheme 141).^159^ The pyrane-containing **141.3** was synthesized by treatment of **141.2** with copper(II) oxide in boiling nitrobenzene. Depending on the topology of the phenol-substituted precursor, five- and seven-membered rings could be obtained (furans and oxepines, respectively, cf. **141.4** and Scheme 80, Section 3.3). The annulations led to substantial changes in the photophysical and electrochemical properties relative to the nonfused precursors. Fusion with the pyrane ring in **141.3** caused a strong bathochromic shift and enhancement of quantum yields (**141.1**: λ_em_ = 384 nm, Φ = 25%; **141.3**: λ_em_ = 450 nm, Φ = 100%). Conversely, the oxepin annulation had a detrimental effect on the emission properties of **80.2** (Scheme 80, Section 3.3). The same cyclization approach was used for the preparation of the π-extended pyrenoid **141.5** bearing two oxygen atoms (for its perylenoid isomer **46.3**, see Scheme 46, Section 3.1.2).^77^

### 4.4 [*cd*]-Heterofused Pyrenoids

**Scheme 142:**
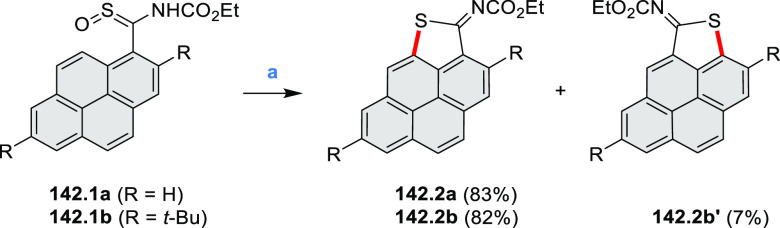
Triflic-Acid-Promoted Cyclization of Thioamide *S-*Oxides Reagents and conditions: (a)^192^ TfOH, DCM, rt.

In 2018, Zakrzewski and co-workers developed a TfOH-promoted cyclization method of polycyclic aromatic *N*-ethoxycarbonylthioamide *S*-oxides, which provides pyrenes with a [*cd*]fused thiophene imine unit (Scheme 142, see Scheme 93, Section 3.4, for perylene analogues).^192^ The proposed reaction mechanism involves the intermediacy of an electrophilic sulfur species, either a protonated iminosulfenic acid or an iminosulfenium cation. These species may attack either the *peri*- or *ipso*-position of the arene, leading to regioisomeric products like **142.2b** and **142.2b′**. Generation of sulfenium-type electrophiles in this reaction was also supported by isolation in a small amount of an intermolecular coupling product. In contrast to **142.2a** and **142.2b′**, which exhibit very weak emission (Φ_f_ < 0.01), **142.2b** fluoresces with a quantum yield of 64% in a DCM solution.

### 4.5 [*a*]-Heterofused Pyrenoids

**Scheme 143:**
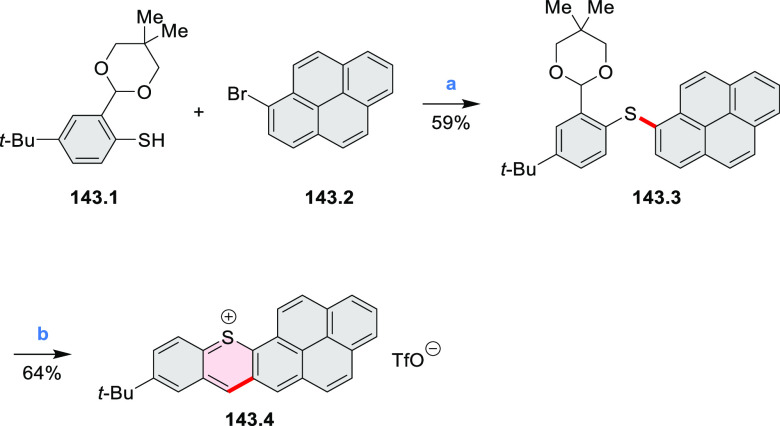
Synthesis of Thiopyrylium-Fused Pyrene Reagents and conditions: (a)^280^ Cu_2_O, 2,4,6-trimethylpyridine, 150 °C, 48 h, 59%; (b) TfOH, DCM, rt, 2 h, 64%.

In 2021, Nagahora et al. reported a synthesis of thiopyrylium-fused aromatics, including the pyrene derivative **143.4** (Scheme 143).^280^ Diaryl thioether **143.3** with a cyclic-acetal-protected aldehyde function was prepared in an S_N_Ar-type reaction in the presence of Cu_2_O in trimethylpyridine. Treatment of this compound with TfOH resulted in acetal cleavage and condensation, leading to the thiopyrylium-containing **143.4**. Compared to analogues that contained smaller aromatic systems, **143.4** had a much smaller HOMO–LUMO gap, resulting in an additional weak and broad NIR absorption band with a maximum at 686 nm.

**Scheme 144:**
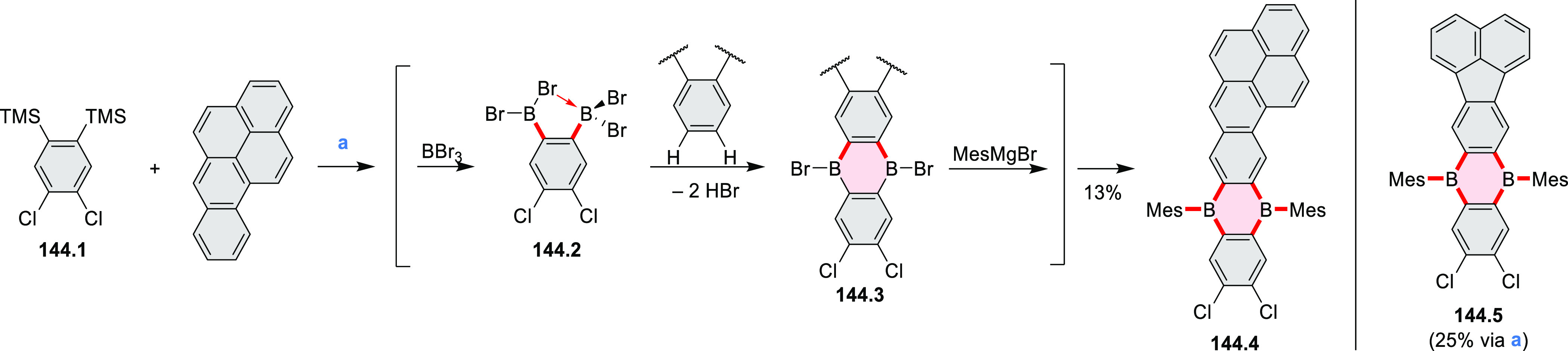
Vicinal Diborylation Reaction toward Doubly Boron-Doped Polycyclic Arenes Reagents and conditions: (a)^281^ (1) BBr_3_, *n*-hexane, ampule flame-sealed under vacuum, 120 °C, 2.5 days, (2) MesMgBr, toluene/THF, 0 °C, then rt, overnight.

A vicinal electrophilic diborylation reaction reported by the Wagner group provides access to doubly boron-doped PAHs such as the benzopyrene-containing **144.4** (Scheme 144).^281^ The latter system was obtained by treating 4,5-dichloro-1,2-bis(trimethylsilyl)benzene **144.1** with BBr_3_ in the presence of benzo[*a*]pyrene, followed by addition of mesitylmagnesium bromide. The transformation was proposed to proceed through a highly electrophilic diborylated intermediate **144.2** formed from **144.1** in a reaction with BBr_3_. Other B*-*doped aromatics were synthesized in this manner, including the fluoranthene-fused **144.5**. Compound **144.4** exhibited intense photoluminescence with a maximum at 503 nm and a quantum yield of 82% in cyclohexane solution. The emission maximum position depended on solvent polarity and was bathochromically shifted by about 100 nm in acetonitrile.

**Scheme 145:**
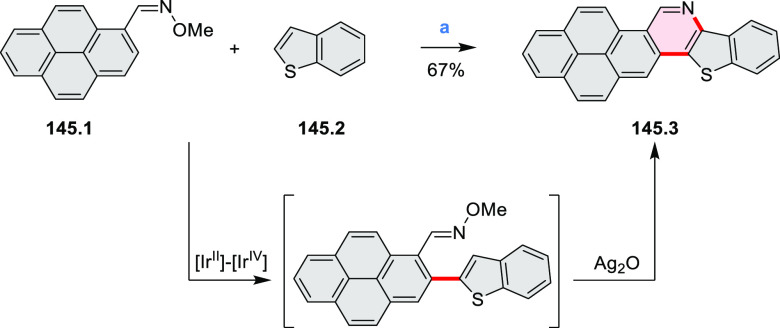
Synthesis of a Benzo[4,5]thieno[3,2-*b*]pyridine-Fused Pyrene Reagents and conditions: (a)^282^ [Cp*IrCl_2_]_2_, AgSbF_6_, Ag_2_O, Zn(OTf)_2_, hexafluoroisopropanol, 140 °C, 24 h.

Synthesis of heterofused pyrene derivative **145.3** was achieved by an iridium-catalyzed direct fusion of pyrene-1-oxime methyl ether **145.1** and benzothiophene **145.2**, which is a successful example of a cascade C–H/C–H cross-coupling/cyclization strategy (Scheme 145).^282^ The reaction was proposed to begin with an oxime-directed C–H activation of **145.1** followed by C–H arylation of benzothiophene in an [Ir^II^]–[Ir^IV^] catalytic cycle with Ag_2_O as the terminal oxidant. The resulting cross-coupled intermediate underwent cyclization via two consecutive one-electron oxidations by Ag_2_O.

**Scheme 146:**
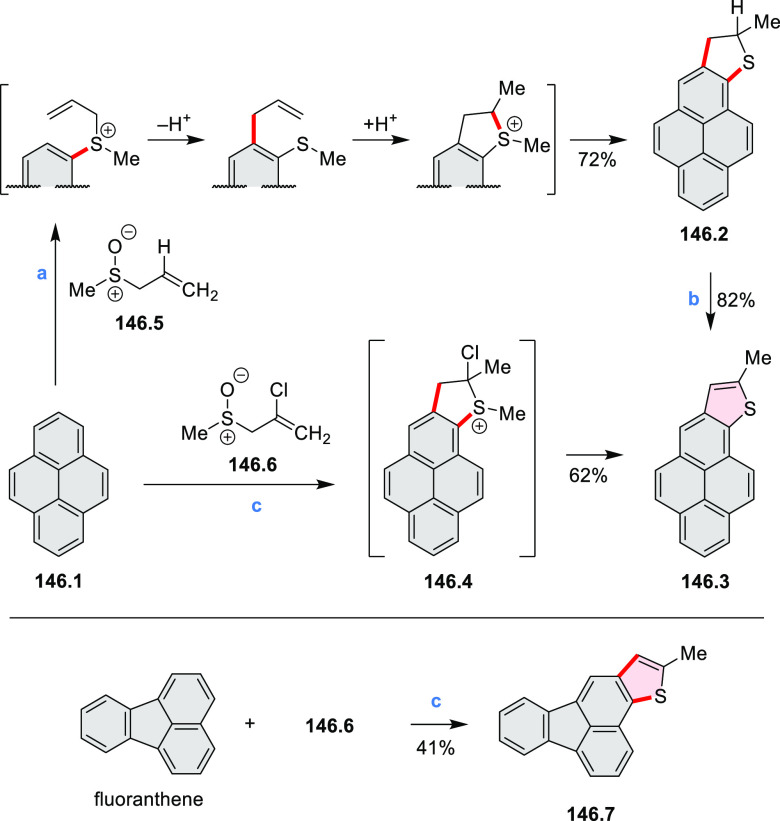
One-Pot Synthesis of an [*a*]-Thiophene-Fused Pyrene Reagents and conditions: (a)^284^ (1) **146.5**, triflic anhydride, DCE, −30 to 90 °C, MW heating, (2) Et_3_N, 0 to 50 °C; (b) DDQ, toluene, rt to 80 °C; (c) (1) **146.6**, triflic anhydride, DCE, −30 to 90 °C, MW heating, (2) Et_3_N, 0 to 50 °C.

The majority of the recently reported [*a*]hetero-fused pyrenoids contains a thiophene ring (see Scheme 141 for related furan derivatives and ref (283) for systems containing phosphole and silole rings). In 2019, Procter and co-workers developed a one-pot approach for the synthesis of dihydrothiophene-fused arenes proceeding through a sequence of an intermolecular interrupted Pummerer reaction between an activated sulfoxide **146.5** and nonprefunctionalized pyrene **146.1**, followed by Claisen-type [3,3]-sigmatropic rearrangement and cyclization (Scheme 146).^284^ The oxidation/aromatization of the 2,3-dihydrobenzothiophene **146.2** by treatment with DDQ in hot toluene led to the thiophene-fused **146.3**. Alternatively, using the chlorinated starting material **146.6** produced **146.3** in 62% yield without the final oxidative step. In that case, intermediate **146.4** was formed, which was converted to **146.3** by demethylation and HCl elimination. This transition-metal-free thienannulation was also utilized to obtain various thiophene-fused systems, e.g., ones containing fluoranthene (**146.7**) and corannulene (Scheme 223, Section 6.1.1).

**Scheme 147:**
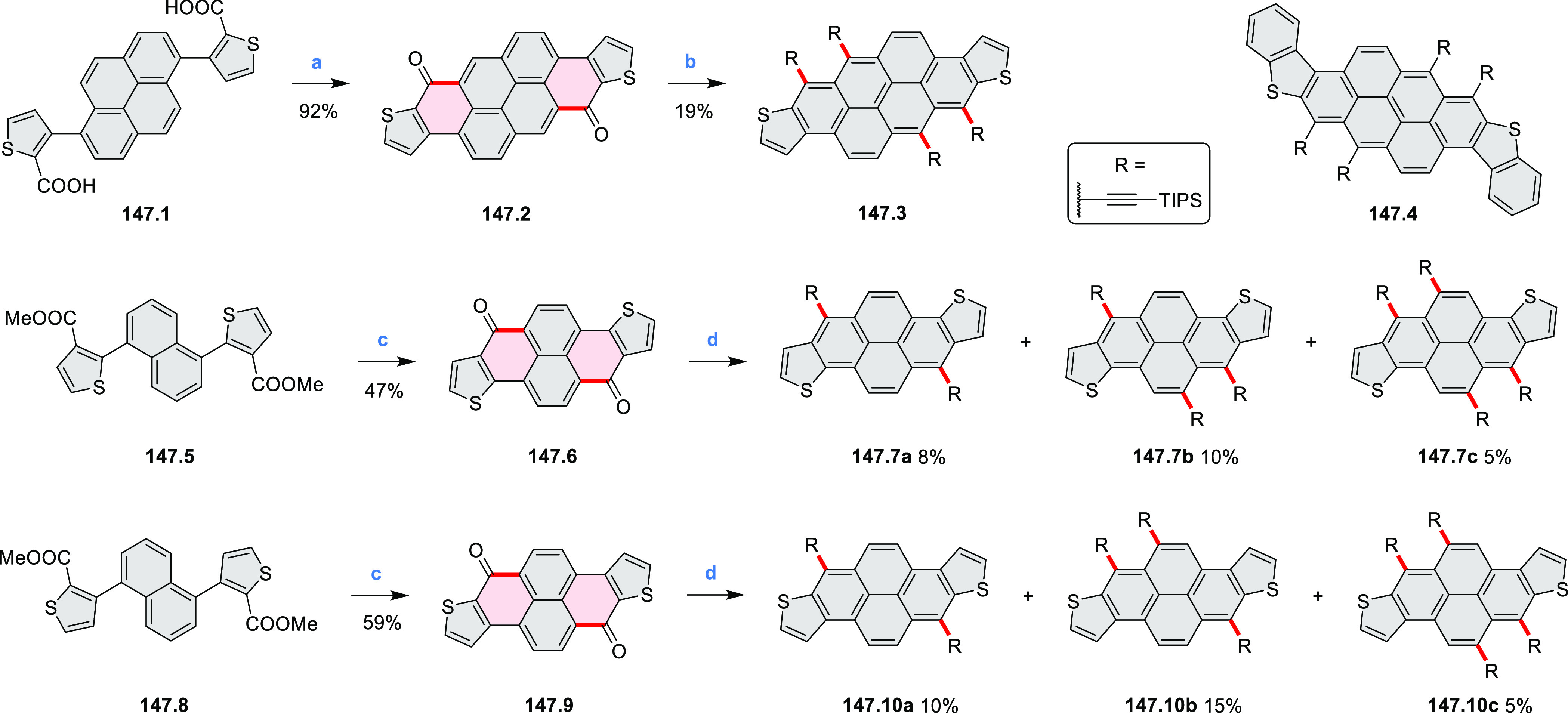
Synthesis of TIPS-Substituted Thieno-Fused Anthanthrenes and Pyrenes Reagents and conditions: (a)^285^ polyphosphoric acid, 90–120 °C, 24 h; (b) (1) triisopropylsilylacetylene, *n*-BuLi, THF, 0 °C, 1 h then **147.2**, rt, 6 h, (2) SnCl_2_·2H_2_O, HCl, rt, 3 h; (c)^286^ TfOH, MsOH, 140 °C, 4 h; (d) (1) triisopropylsilylacetylene, *n*-BuLi, Et_2_O, 0 °C, 1 h, then **147.6** or **147.9** in THF, rt, overnight, (2) SnCl_2_·2H_2_O, HCl, rt, 1 h.

In 2018, Sun and Chen reported a synthesis of the two anthanthrene derivatives fused with thiophene and benzothiophene, **147.3** and **147.4**, respectively (Scheme 147).^285^ Their synthetic route started with intramolecular Friedel–Crafts cyclization of 1,6-bisthienylopyrene **147.1**, providing the key intermediate **147.2** in high yield. Treatment of this compound with lithium alkynide prepared from TIPS-acetylene resulted in 1,4- and 1,2-addition to its α,β-unsaturated ketone substructures. After subsequent treatment with SnCl_2_, the tetrasubstituted product **147.3** was obtained in 19% yield. **147.4** bearing two additional benzene rings was synthesized using a similar approach. Both products exhibited a red emission at 662–663 nm. An additional vibronic NIR emission band was observed at 715 nm in **147.3** and 723 nm in **147.4**. Likewise, two isomeric pyrenodithiophenediones **147.6** and **147.9** were converted into their TIPS-ethynyl functionalized derivatives **147.7a**–**c** and **147.10a**–**c**. In this case, mixtures of derivatives with two, three, and four TIPS-ethynyl substituents were obtained.^286^ Increasing the number of appended alkynes lowered the LUMO energy levels while having little impact on the HOMO energy. These changes were reflected in bathochromic shifts of absorption bands.

**Scheme 148:**
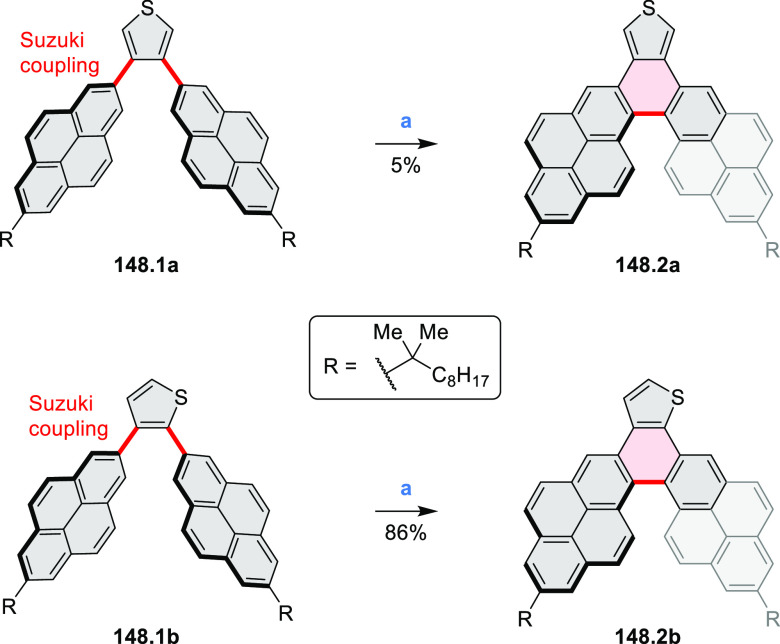
Synthesis of a Dithiophene-Fused Benzopyrene Reagents and conditions: (a)^287^ FeCl_3_, MeNO_2_, 0 °C, 10 min for **148.2a** or 1 h for **148.2b**.

Oxidative coupling with FeCl_3_ was used to convert two isomeric bis(pyren-2-yl)thiophenes **148.1a**,**b** into the fully fused compounds **148.2a**,**b** (Scheme 148).^287^ Attempted cyclization on thiophenes carrying three and four pyren-2-yl groups was not successful. While compound **148.2b** with a [*b*]fused thiophene was obtainable in high yield, its [*c*]fused isomer **148.2a** was unstable and could only be obtained in 5% yield. The stable isomer **148.2b** was analyzed by single-crystal X-ray diffraction, revealing a 39° angle between its [5]helicene blades.

### 4.6 Pyrazine-Fused Systems (Pyrazaacenes)

**Scheme 149:**
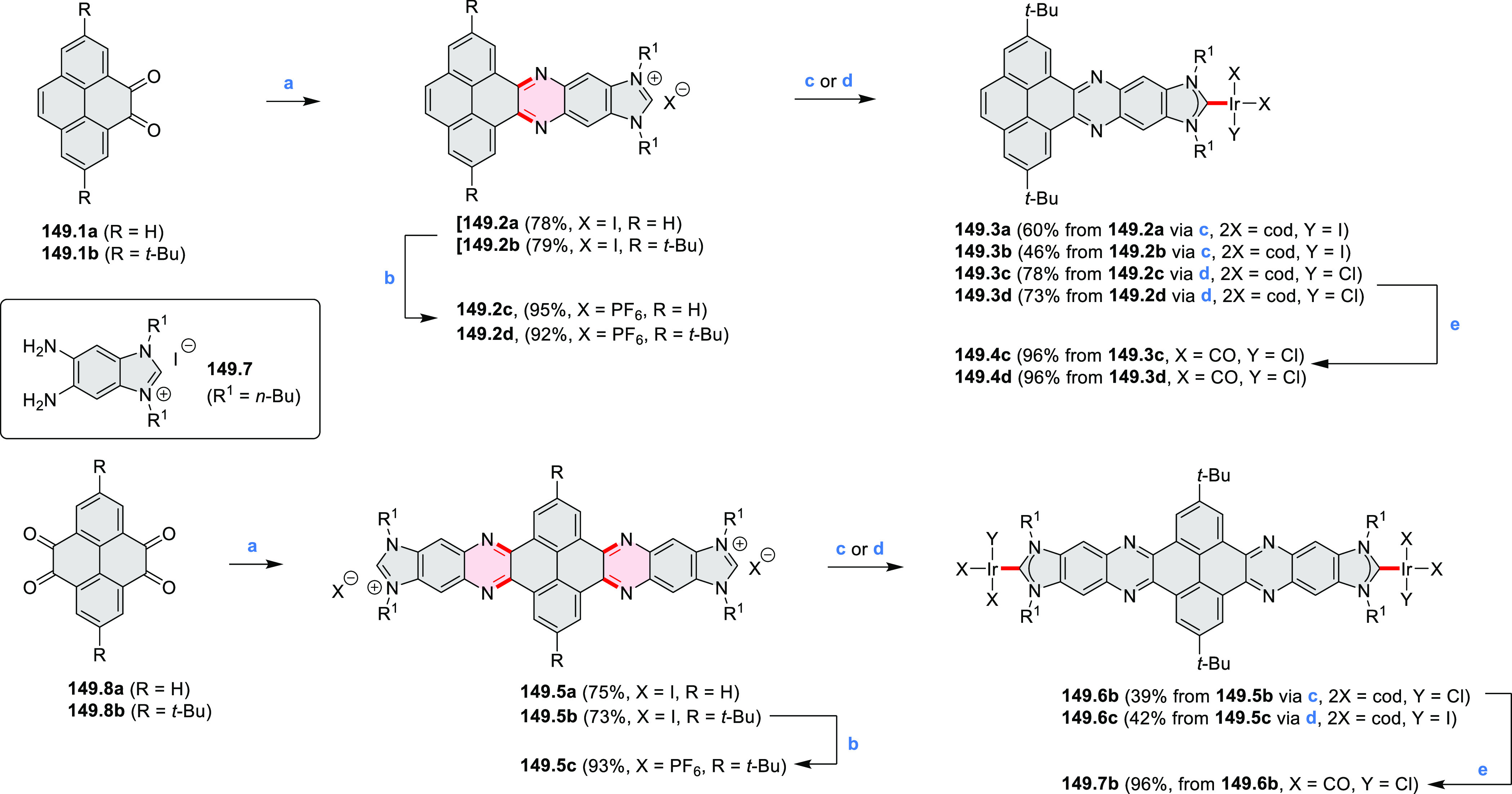
Synthesis of Iridium Complexes Containing Phenanthro[4,5-*abc*]phenazino[11,12-d]imidazol-2-ylidene Reagents and conditions: (a)^288,289^**149.7**, MeOH, reflux, overnight; (b) NH_4_PF_6_, MeOH/DCM (3:1), rt, overnight; (c) potassium *tert*-butoxide, NaI (in the case of **149.3a**,**b**), [IrCl(cod)]_2_, THF, rt, overnight; (e) CO, DCM, 0 °C, 20 min; (d) potassium *tert*-butoxide, [IrCl(cod)]_2_, KI (in case of **149.6c**), THF, rt, overnight.

Condensation of pyrene diones and tetraones, such as **149.1** and **149.8** (Scheme 149), with aromatic amines provides a very general approach toward large N-doped “pyrazaacenes”, usually with ribbon-like structures (cf. CR2017, Section 4.6, for earlier developments and Scheme 260, Section 6.1.6, for related 2D materials). Pyrazaacenes can be tailored to perform a variety of functions; for instance, they can be elaborated into ligands. Poyatos and Peris described the preparation of NHC ligands containing fused phenanthro[4,5-*abc*]-phenazine units (for acenaphtho analogues see Chart 25, Section 6.2.1).^288^ First, azolium salts **149.2a**,**b** were prepared by condensation of diketone **149.1a**,**b** and 1,3-dibutyl-5,6-diaminobenzimidazolium iodide **149.1** in yields of 78 and 79%, respectively. The coordination of **149.2a**–**d** to iridium was then achieved by deprotonation with potassium *tert*-butoxide and subsequent addition of [IrCl(cod)]_2_. Furthermore, treatment of **149.3c**,**d** with carbon monoxide in methylene chloride afforded the related carbonyl complexes **149.4c**,**d** in excellent yields. Addition of π-stacking additives such as pyrene and hexafluorobenzene was investigated for its possible effect on the electron-donating character of the ligands.

Janus-like bis-N*-*heterocyclic carbene (bis-NHC) ligands **149.5a**,**b** were obtained by the same group by following a similar direct condensation approach with the corresponding pyrene-4,5,9,10-tetraone **149.8a**,**b**.^289^ Anion metathesis of **149.5b** with ammonium hexafluorophosphate allowed the preparation of the corresponding PF_6_ salt, **149.5c**, in 93% yield. Due to their higher solubility, the di-*tert*-butyl-substituted bisazoliums **149.5b** and **149.5c** were chosen for coordination experiments. The reaction of the bis-azolium salt **149.5c** with [IrCl(cod)]_2_ produced the di-iridium complex **149.6c** in 42% yield. In the absence of the KI additive, the reaction of **149.5b** afforded the chlorine analogue complex **149.6b**. Treatment of **149.6b** with carbon monoxide in DCM gave the tetra-carbonyl species **149.7b**. The X-ray crystal structure of **149.6c** revealed a metal-to-metal distance of 22.4 Å.

**Scheme 150:**
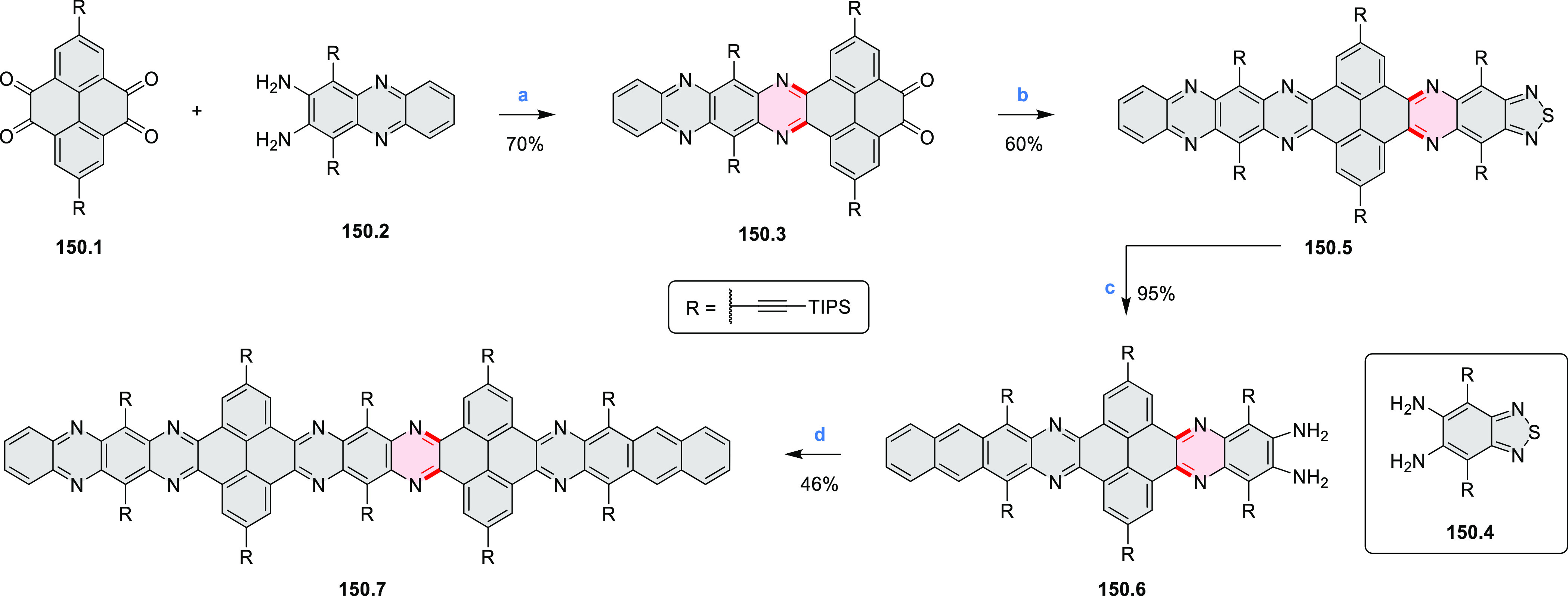
Synthesis of a Pyrene-Fused *N-*Heteroacene Reagents and conditions: (a)^290^ (1) CHCl_3_/AcOH (2:1), 80 °C, 36 h, (2) MnO_2_, DCM; (b) CHCl_3_/AcOH (2:1), 80 °C, 30 h; (c) LiAlH_4_, THF, 0 °C to rt, 14 h; (d) CHCl_3_/AcOH (4:1), 80 °C, 48 h.

In 2017, Zhu and Zhang successfully synthesized a pyrene-containing nitrogen-rich nanoribbon with 15 six-membered rings linearly annulated in one row.^290^ Compound **150.3** was obtained in a good yield of 70% through the reaction between diamine **150.2** and an excess amount of tetraketone **150.1** (Scheme 150). Both substrates were substituted by triisopropylsilyl groups to increase their solubility and crystallizability. Next, the condensation of **150.3** and **150.4** followed by reductive ring opening was performed yielding **150.6**. Finally, the target compound **150.7** was prepared by condensing diketone **150.3** with diamine **150.6** in a mixture of chloroform and AcOH under reflux conditions. The single X-ray crystal structure of **150.7** shows a slightly twisted structure at the two pyrene units which might stabilize this large azaacene system by releasing the strain resulting from structure packing. The optical band gap of **150.7** was found to be 1.85 eV, and this compound exhibits emission maxima at 661 nm with a fluorescence quantum yield of 3%. The low Φ_f_ value might be attributed to the self-absorption effect and intersystem crossing.

**Scheme 151:**
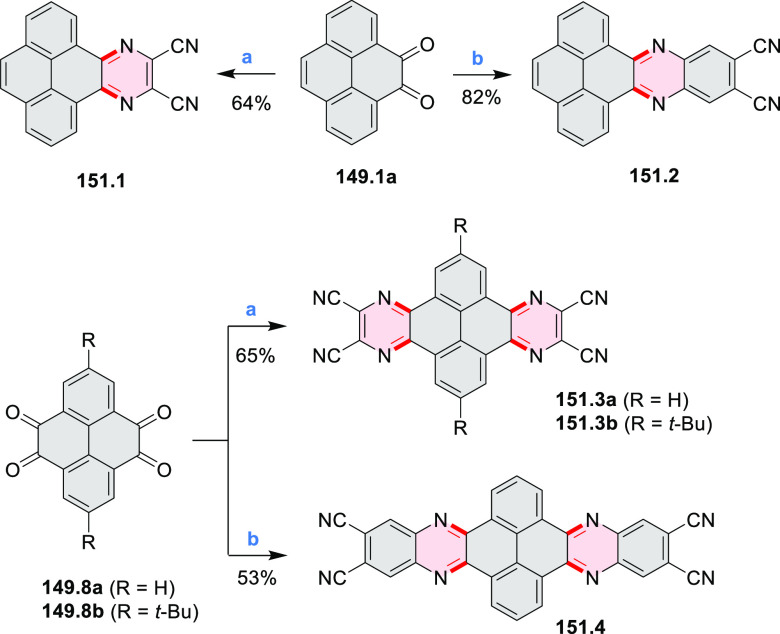
Synthesis of Di- and Tetracyanopyrazines Reagents and conditions: (a)^291^ 2,3-diaminomaleonitrile, EtOH/AcOH (1:1), 80 °C, 11–16 h; (b) 4,5-diaminophthalonitrile, EtOH/AcOH (1:1), 80 °C, 11–16 h.

In their search for solid-state electron acceptors, the Mastalerz group revisited four previously known di- and tetracyano-substituted pyrene-fused pyrazaacenes and synthesized a new derivative **151.2**.^291^ Except **151.1**, single crystals from all known compounds were grown for the first time. **151.1** and **151.2** were prepared by condensing pyrenedione **149.1a** with 2,3-diaminomaleonitrile and 4,5-diaminophthalonitrile, respectively, under acidic conditions (Scheme 151). Tetracarbonitriles **151.3a**,**b** and **151.4** were obtained similarly from the corresponding pyrenetetraones **149.8a**,**b**. The electron-deficient nature of these compounds and low-lying LUMOs (up to −3.9 eV) makes them suitable as electron-transport materials. X-ray crystallographic analyses revealed that all the structures formed face-to-face columnar stacks with small π–π distances (ca. 3.29–3.51 Å; average: 3.37 Å). Calculated charge transfer integrals for electron transport (69–76 meV for **151.3b**, 101–172 meV for **151.2**, 108 meV for **151.4**, and 113–252 meV for **151.1**) and small reorganization energies (91–256 meV) indicated that those four compounds might be useful as n-type semiconductors.

**Scheme 152:**
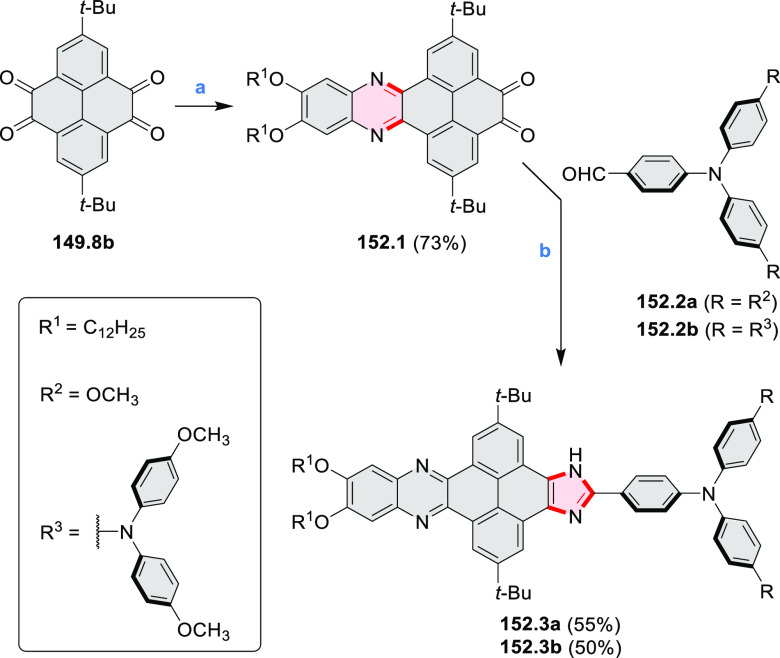
Synthesis of Unsymmetrically Fused Pyrazaacenes Reagents and conditions: (a)^292^ 1,2-diamino-4,5-didodecyloxybenzene, AcOH, CHCl_3_, reflux; (b) AcOH, reflux.

Asymmetric pyrazine–pyrene–imidazole systems with a D−π–A structure were similarly synthesized (Scheme 152).^292^ In the second step, the Debus–Radziszewski condensation was employed, furnishing the target imidazoles **152.3a** and **152.3b** in moderate yields. The color of **152.3a** and **152.3b** changed from light yellow to brown upon protonation with trifluoroacetic acid in DCM solution. Thin films of **152.3b** showed reversible electrochromic behavior accompanied by color alteration from yellow to green under different bias voltages, observable by in situ UV–vis and NIR spectroscopy.

**Scheme 153:**
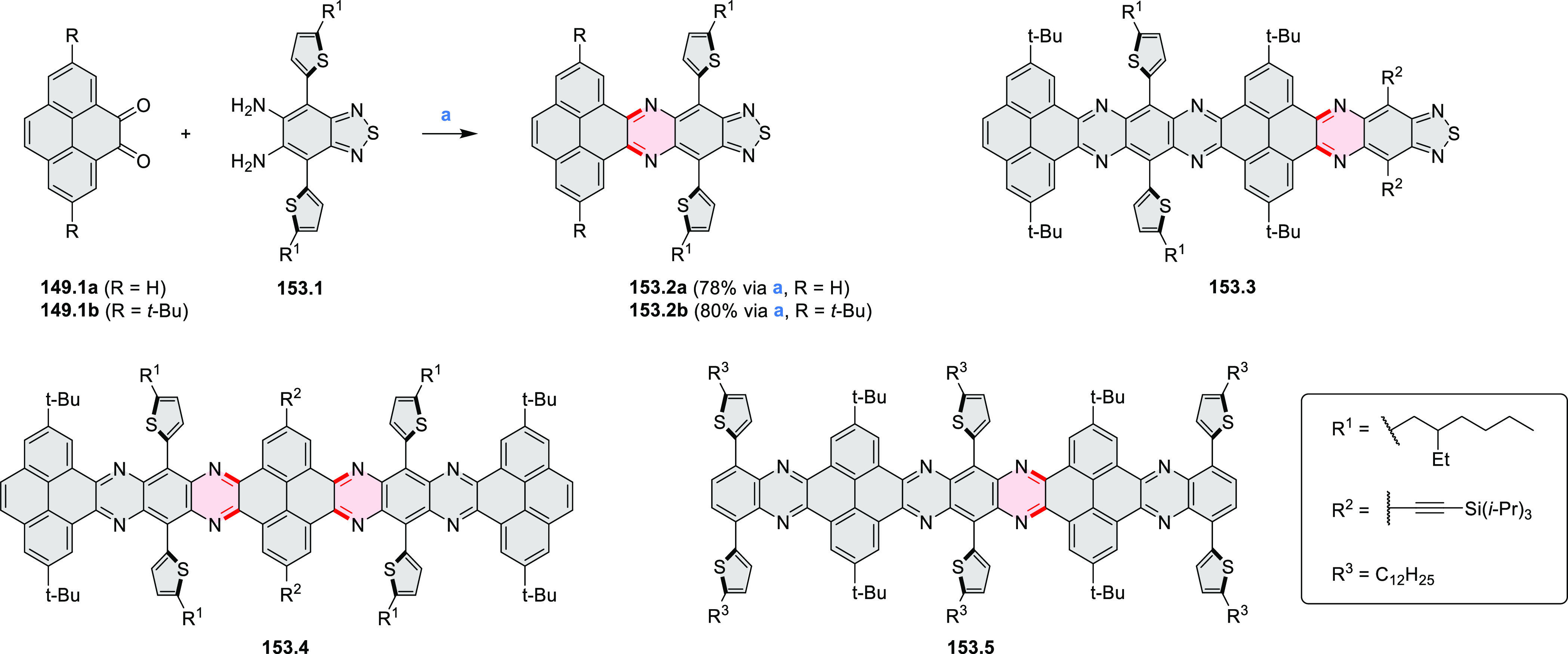
Synthesis of Thiophene-Substituted Pyrazine-Fused Pyrenoids Reagents and conditions: (a)^293^ AcOH, 100 °C, 24 h.

Stepwise annulation protocols were employed in the syntheses of thienyl-substituted pyrazaacenes, such as the [1,2,5]thiadiazolo[3,4-*g*]quinoxalines, reported by Zhang et al.^293^ Here, the key annulation step was performed between pyrene-4,5-dione **149.1a** or 2,7-di-*tert*-butylpyrene-4,5-dione **149.1b** and thiadiazole-fused diamine **153.1**, providing the target compound **153.2a** and **153.2b**, respectively (Scheme 153). In OFET investigations, **153.2a** and **153.2b** showed typical p-type characteristics with mobilities of up to 0.05 and 0.0055 cm^2^ V^–1^ s^–1^ and on/off current ratios of 1 × 10^6^ and 1 × 10^4^, respectively. Because of bulkier substitution, compound **153.2b** adopted looser packing in the solid state, and the larger π– π distances reduced the transport performance. A related thiadiazole-based charge-transfer system, **153.3**, showed an excellent nonvolatile tristate memory behavior, with a ternary device yield as high as 78% and retention stability longer than 10^4^ s.^294^ The ribbon-like **153.4** with 12 linearly fused aromatic six-membered rings was well soluble in chlorinated solvents but could also be easily crystallized, showing a zigzag packing motif in the solid state.^295^ Single-crystal organic field effect transistors fabricated from **153.4** through an organic ribbon mask technique exhibited hole mobility of up to μ_h_ = 8.1 × 10^–3^ cm^2^ V^–1^ s^–1^. The related nanoribbon **153.5** was shown by Lee and co-workers to form organogels with hydrocarbon solvents and select halogenated solvents, with the lowest critical gelation concentration of 1.2 mM in hexadecane.^296^**153.5** showed a relatively stabilized *E*_LUMO_ of −3.74 eV due to the electron-deficient nature of the core and reduced band gap of 1.55 eV as a result of intramolecular charge transfer between the core and axial thiophene.

**Scheme 154:**
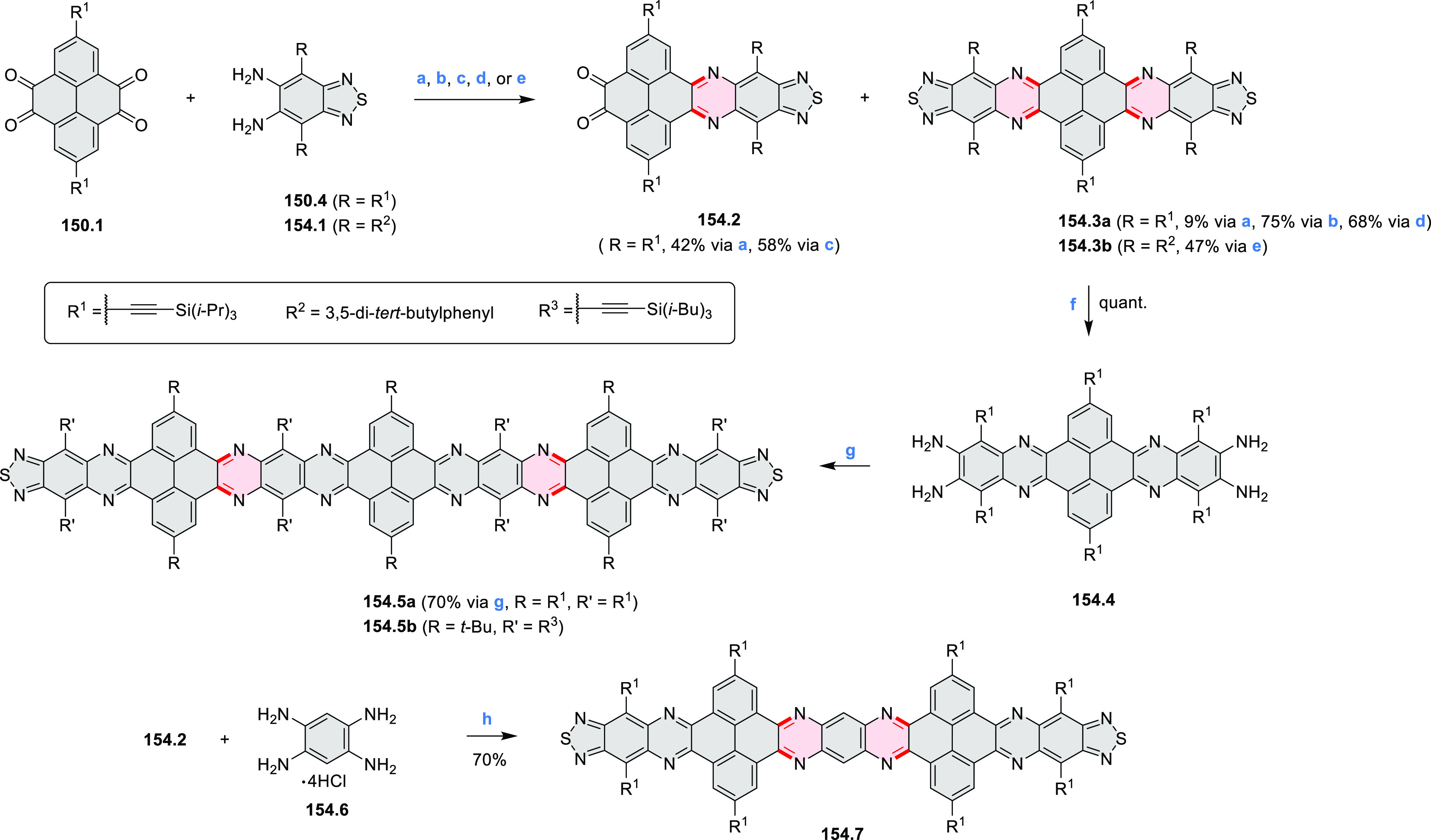
Synthesis of Thiadiazoloquinoxaline-Containing Long Pyrene-Fused *N-*Heteroacenes Reagents and conditions: (a)^299^**150.1**/**150.4** (1:1), CHCl_3_/AcOH (2:1), reflux, 1 day; (b)^298^**150.1**/**150.4** (1:2), CHCl_3_/AcOH (1:1), 80 °C, 1 day; (c)^298^**150.1**/**150.4** (1:1), CHCl_3_/AcOH (1:1), reflux, 1 day; (d)^297^**150.1**/**150.4** (1:2.9), AcOH, 132 °C, overnight; (e)^297^**150.1**/**154.1** (1:3), AcOH, reflux, overnight; (f)^299^ LiAlH_4_, THF, 0 °C to rt, overnight; (g)^299^**154.2**, CHCl_3_/AcOH (1:1), reflux, 1 day; (h)^299^ CHCl_3_/AcOH (1:1), reflux, 1 day.

Thiadiazole-capped pyrazaacenes were obtained in several laboratories via stepwise condensations summarized in Scheme 154 and investigated as organic semiconductors.^297−299^ In particular, **154.3a** and **154.3b** were used as LED emitters, showing red–NIR emission bands, centered at 658 and 721 nm, respectively.^297^**154.3a** was also explored as an active material in resistive memory devices.^298^ Charge carrier transport properties in the homologous series consisting of **154.3a**, **154.5a**, and **154.7** were evaluated in “top contact bottom gate” FETs.^299^ In particular, devices based on a **154.3a** single crystal exhibited an average electron mobility of about 6 × 10^–4^ cm^2^ V^–1^ s^–1^, with an ON/OFF ratio of 2000 and a threshold voltage (*V*_th_) of 25 V. For a single crystal of **154.7**, the average mobility was higher than that of **154.3a**, with an electron mobility of approximately 5 × 10^–3^ cm^2^ V^–1^ s^–1^, an ON/OFF ratio of 500, and a threshold voltage (*V*_th_) of 20 V. The highly soluble variant **154.5b** substituted with *tert*-butyl and TIBS groups was used for OFET device fabrication.^300^ Thin films of **154.5b** deposited from solution show an n-type behavior and a maximum electron mobility of μ_e_^max^ = 1.4 × 10^–4^ cm^2^ V^–1^ s^–1^ without any device optimization.

**Chart 15:**
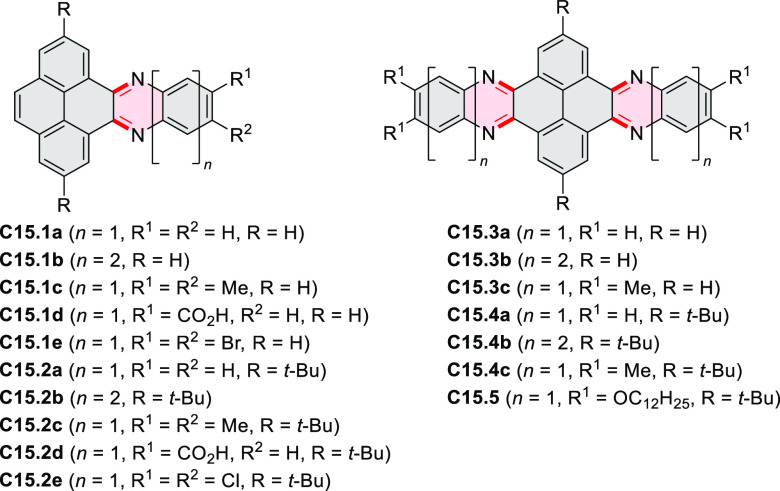
Pyrene-Fused Azaacenes

A mechanochemical synthesis under solvent-free ball-milling conditions was reported as an efficient route to **C15.1a**–**e**, **C15.2a**–**e**, **C15.3a**–**c**, and **C15.4a**–**c** (Chart 15).^301^ Di- or tetraketone pyrenes were used as precursors along with the appropriate 1,2-diaminoarenes, and the reactions took 3 to 4 h under ambient laboratory conditions with *p*-toluenesulfonic acid as the catalyst. In 2018, thin films of two pyrazaacenes, **C15.4c** and its dodecyloxy-substituted derivative **C15.5**, on silicon/native silica (Si/SiO_2_)_native_ and fused silica substrates were analyzed with respect to their microstructural and anisotropic optical properties (Chart 15).^302^ Because of solubility differences, thin films of **C15.4c** were obtained by organic molecular beam deposition in ultrahigh vacuum conditions, while **C15.5** layers were produced from solution by spin coating. The two films exhibited different textures, each with strongly uniaxial anisotropy. The molecules of **C15.4c** were arranged parallel to the surface, while the cores of **C15.5** were cofacially packed and tilted with respect to the surface normal. In another report, Tegeder et al. investigated the adsorption geometry and electronic properties of **C15.3a** and its 2,11-di-*tert-*butyl analogue **C15.4a** as a function of coverage on Au(111).^303^ Both molecules adopted a planar geometry with respect to the substrate in both a single monolayer and thin films (up to 10 monolayers thick). In contrast, in the crystal structures, a tilt of up to 82° was observed between molecular planes in neighboring stacks.

**Scheme 155:**
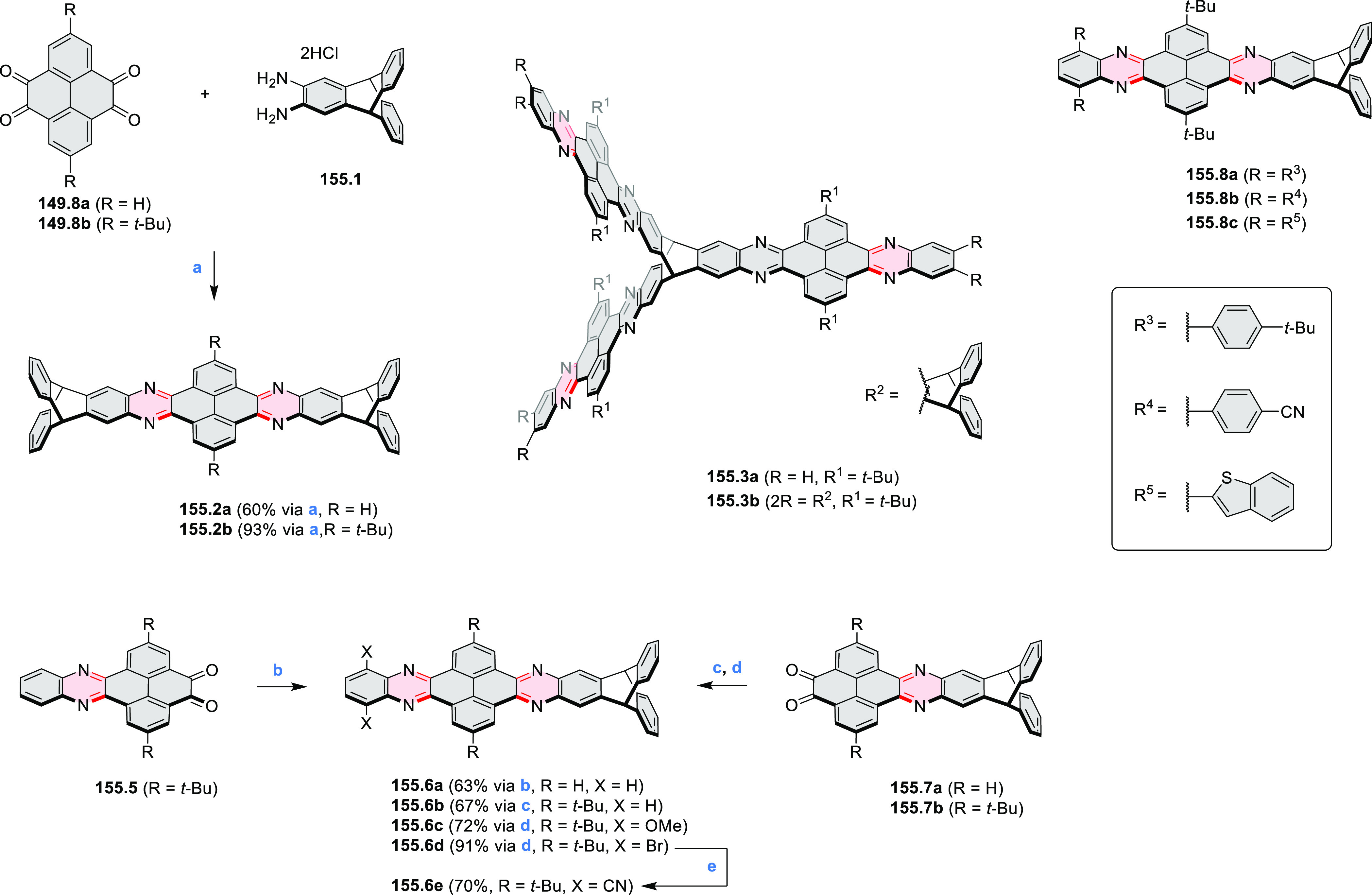
Synthesis of Triptycene End-Capped Quinoxalinophenanthrophenazines Reagents and conditions: (a)^304^ KOAc, CHCl_3_, AcOH, 70 °C, 16 h; (b)^306^**155.1**, KOAc, CHCl_3_, AcOH, 70 °C, 16 h; (c)^306^*o*-phenylenediamine, CHCl_3_, AcOH, 70 °C, 16 h; (d)^307^ appropriate *o*-phenylenediamine, CHCl_3_, AcOH, 70 °C, 16 h; (e) NMP, CuCN, 180 °C 15 h.

Further work on pyrazaacene–triptycene hybrids was reported by Mastalerz and co-workers. Triptycenylene end caps were, for instance, introduced to enhance the solubility of quinoxalinophenanthrophenazines **155.2a**,**b** (Scheme 155).^304^ In this way, the formation of LC phases was avoided, a behavior often caused by functionalization with long alkyl and alkoxy chains. Compounds **155.2a**,**b** were synthesized in 60% and 93% yield, respectively, by condensing diammonium triptycene dichloride **155.1** with pyrene tetraketones **149.8a**,**b**. In 2015, Mastalerz described four polymorphs of triptycene-based **155.3b**, an organic molecule of intrinsic microporosity (OMIM), which showed an unusual gas sorption behavior with a very large hysteresis (Scheme 155).^305^ The material could not be activated by solvent exchange; however, by thermal treatment, high specific surface areas of up to 350 m^2^ g^–1^ could be generated reproducibly. Quinoxalinophenanthrophenazines **155.6a**,**b** with triptycenylene units were used as the model compounds to get a deeper impact of the dispersion interaction of the *tert*-butyl groups and the triptycenylene units on the formation of the unusual packing motif of **155.3a**.^306^ The monotriptycenylene-substituted **155.6a** and **155.6b** were obtained from the annulation reactions between either **155.7a** or **155.5** with the corresponding *o*-phenylene diamine in 67% and 63% yield, respectively. Well-balanced dispersion contributions of *tert*-butyl groups and one triptycenylene unit at the periphery of the π plane both seemed to affect packing motifs, leading **155.6a** and **155.6b** to adopt a brick-wall arrangement of π planes in the solid state.

To study the effect of substitution on crystal packing, the Mastalerz group obtained 17 single-crystal solvates of four QPP structures (**155.6c**–**e** and **155.6b**).^306,307^ All crystal structures formed the cofacial π–π dimers and the degree of overlap, and the arrangement of these dimers to each other depended on solvents, substituents, and crystallization conditions. For some crystalline structures of **155.6b**, a high degree of LUMO–LUMO overlap and high transfer integrals were found. Related investigations were performed on three triptycene-end-capped QPP derivatives **155.8a**–**c** bearing aromatic substituents at the peripheral phenylene units.^308^ Absorption spectra of these films indicated increased π stacking tendency of **155.8b** and **155.8c** relative to **155.8a**. The formation of π-dimers occurred to a different degree in all crystal structures, showing that the directing capability of the triptycene end capping was also pronounced in the presence of larger aromatic substituents that could disturb the packing by competing π-stacking interactions (e.g., in **155.8c**). The group also reported charge-transfer (CT) complexes based on **155.6c** with six small electron-deficient molecules.^309^

**Chart 16:**
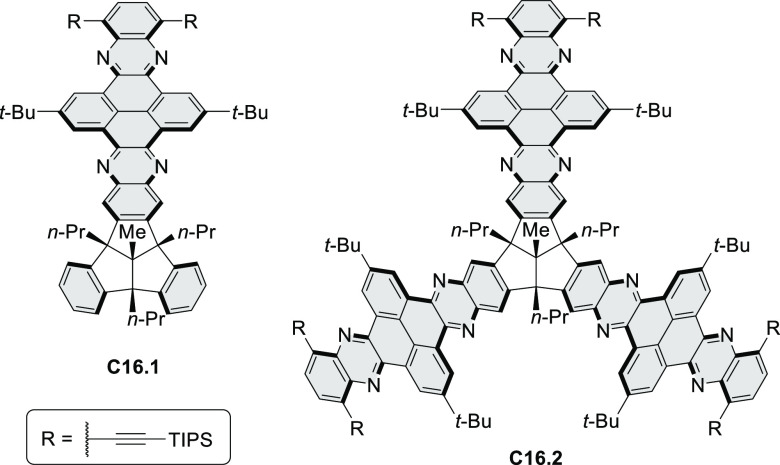
Quinoxalino–Phenanthrophenazine-Fused Tribenzotriquinacenes

In 2021, Mastalerz et al. reported the synthesis and properties of the π-extended tribenzotriquinacene (TBTQ) derivatives **C16.1**–**2** bearing one and three quinoxalinophenanthrophenazine units (Chart 16).^310^ These two species were obtained in two steps from the corresponding diamino- and hexamino-substituted TBTQs, respectively. Both compounds **C16.1**–**2** had similar absorption and emission profiles. In particular, compound **C16.2** exhibited solvatofluorochromism, with the emission maximum becoming increasingly red-shifted in the order of hexane, toluene, THF, chloroform, and DCM.

**Scheme 156:**
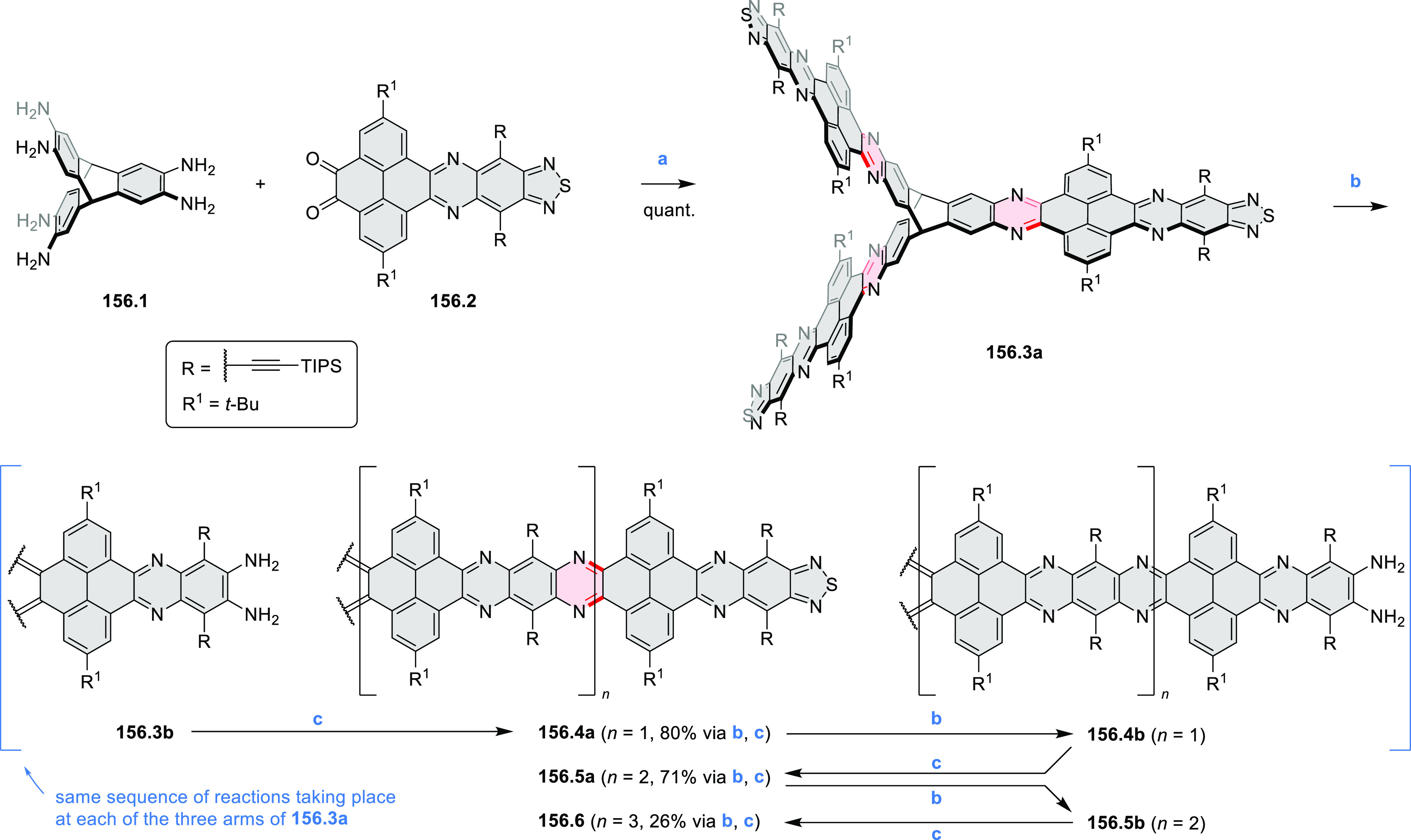
Synthesis of Triptycene-Based Three-Dimensional Pyrene-Fused *N*-Heteroacenes Reagents and conditions: (a)^311^ KOAc, AcOH, chlorobenzene, reflux, 2 days; (b) LiAlH_4_, THF, 0 °C to rt; (c) **156.2**, KOAc, AcOH, chlorobenzene, reflux, 2 days; (d) (1) LiAlH_4_, THF, 0 °C to rt, (2) **156.2**, KOAc, AcOH, chlorobenzene, reflux, 2 days.

In 2019, Hu and Baumgarten reported triptycene-based three-dimensionally extended pyrazaacenes (**156.3**–**6**) obtained by an iterative approach (Scheme 156).^311^**156.3a** was synthesized by the condensation of the intermediate diketone **156.2** and hexamine triptycene hydrochloride salt [**156.1**][6HCl]. To further increase the length of nanoribbons to **156.4**–**6**, the thiadiazole units were reduced by a large excess of LiAlH_4_ in THF to the corresponding diamine moieties, which were then condensed with **156.2**. The diameters of **156.3**–**6** were calculated to be 3.66, 6.06, 8.48, and 10.88 nm, respectively. Compared to their linear counterparts (**154.5a**, 18 rings, only soluble in hot *o*-dichlorobenzene, Scheme 154),^299^**156.3**–**6** (**156.6** with 22 rings) all showed good solubility in DCM, THF, chlorobenzene, and tetrachloroethane.

**Scheme 157:**
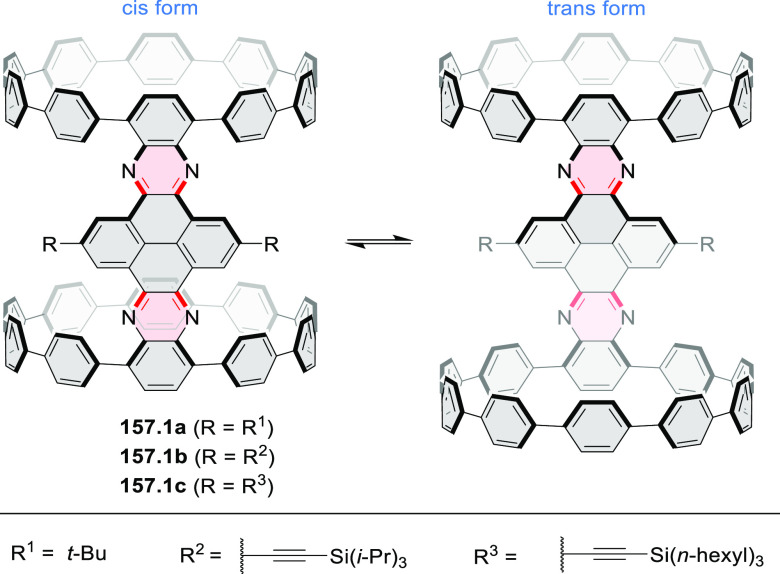
Dimeric Cycloparaphenylenes with a Rigid Aromatic Linker Conformations interconvert at rt.

In 2021, Sun et al. reported the synthesis of three [10]CPP dimers **157.1a**–**c** with a rigid pyrazaacene linker (Scheme 157).^312^ The combined VT-NMR spectroscopic analysis and theoretical analysis indicated rapid interconversion of cis and trans conformers at rt, with trans conformers favored by 2.3 kcal mol^–1^ and an energy barrier of 9.0 kcal mol^–1^ for the *cis*–*trans* interconversion. Compound **157.1c** in DCM exhibited three absorption maxima at 284, 340, and 429 nm and a broad absorption in the range of 440–550 nm. This compound exhibited a large effective Stokes shift of 276 nm, emitting at 616 nm with a quantum yield of up to 80%. A host–guest binding study of **157.1c** revealed that it could bind two C_60_ molecules with *K*_1_ = 4.15 ± 0.58 × 10^5^ M^–1^ and *K*_2_ = 3.63 ± 0.44 × 10^3^ M^–1^.

**Scheme 158:**
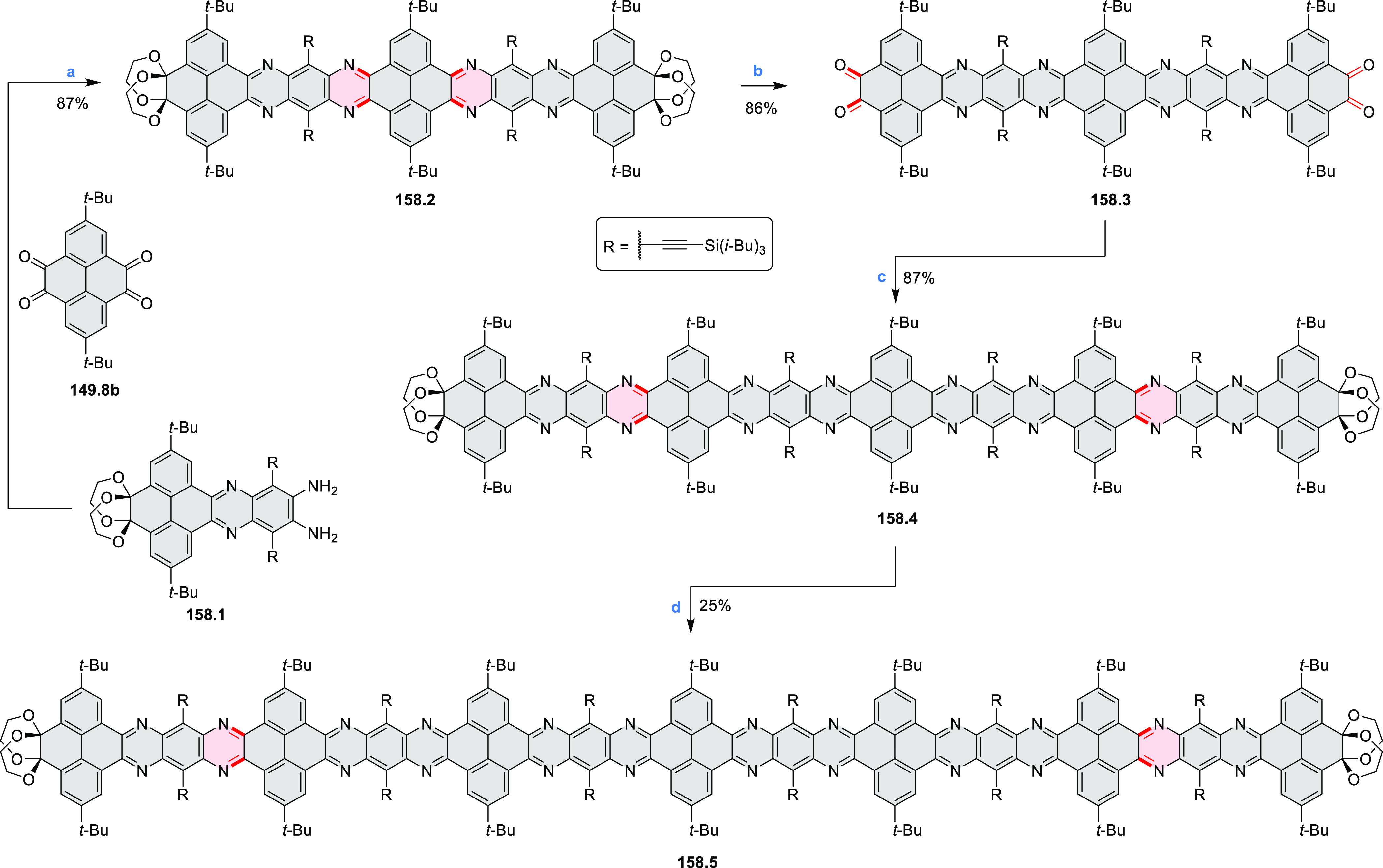
Iterative Synthesis of Pyrazine-Containing Nanoribbons Reagents and conditions: (a)^313^ CHCl_3_, AcOH, reflux, 48 h; (b) TFA, water, rt; (c) CHCl_3_, AcOH, reflux, 4 days; (d) (1) TFA, water, rt, (2) CHCl_3_, AcOH, reflux, 3 days.

In 2018, Melle-Franco and Mateo-Alonso reported on an iterative assembly of a small molecular building block into pyrazaacene nanoribbons containing up to 30 conjugated linearly fused rings (Scheme 158).^313^ The selectivity of individual iterations was achieved using diacetal-protected *o*-dione functionalities, which were unmasked before each consecutive condensation step. In the last completed iteration, performed without isolating the intermediate tetraone, the 7.7 nm long tetradecabenzodotriacontacene **158.5** was obtained in a yield of 25%. The solubility of these nanoribbons could be ensured by introduction of relatively small *tert*-butyl and tri-isobutylsilyl substituents. Electronic spectra showed the expected bathochromic shift and an increase of absorptivity in the series **158.2**, **158.4**, and **158.5**. The photoluminescence spectra in chloroform were almost superimposable over the whole series of nanoribbons; however, the quantum yields of **158.2** (0.27), **158.4** (0.14), and **158.5** (0.11) decreased with increasing lengths. Pseudophotoconductivities were found to be nearly length-invariable for this family of N*-*doped graphene nanoribbons.

**Scheme 159:**
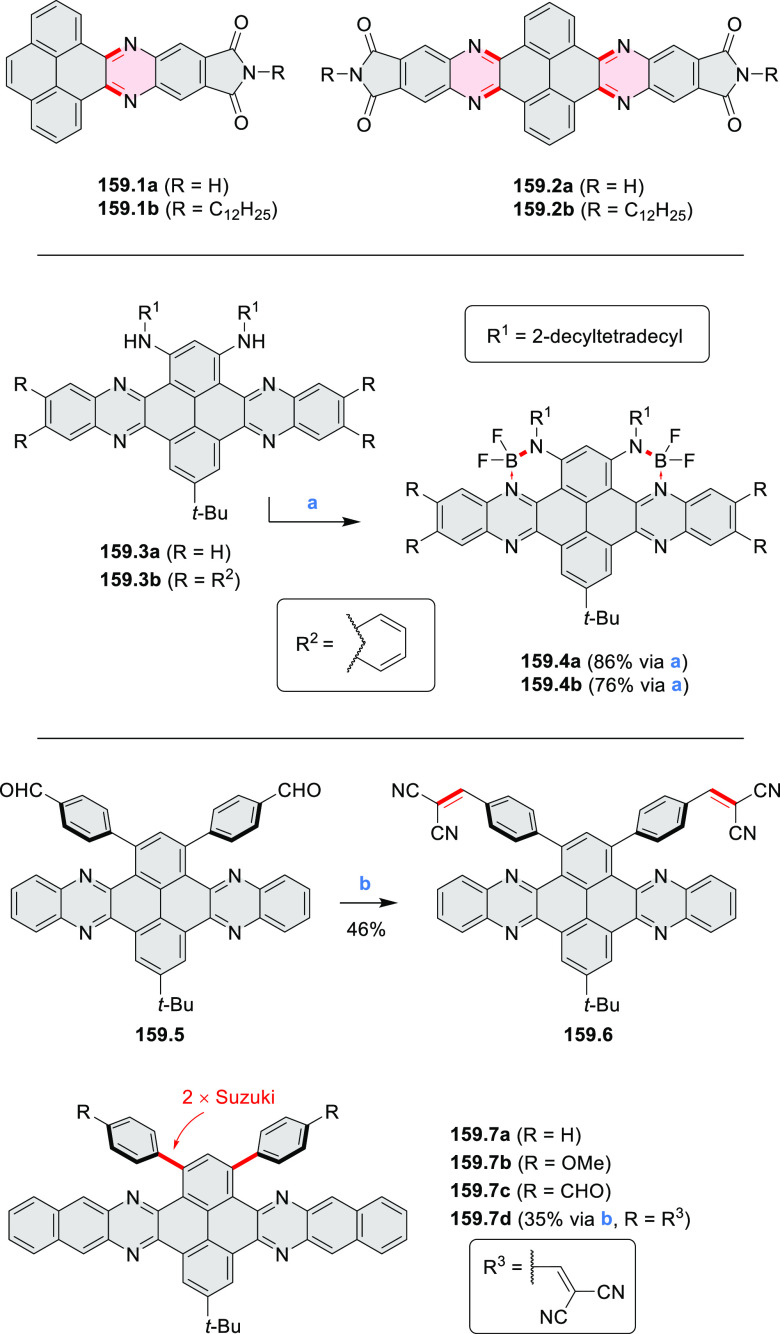
Synthesis of Azaacenes Containing BN Units Reagents and conditions: (a)^315^ BF_3_·Et_2_O, Et_3_N, DCM, 50 °C; (b)^316^ malononitrile, Al_2_O_3_, toluene, reflux, 12 h.

Electronic properties of pyrazaacenes can be fine-tuned by ring fusion and introduction of substituents (Scheme 159). In one recent example, pyrene–phenazine imides **159.1a**,**b** and **159.2a**,**b** were synthesized by annulation of an appropriate diaminophthalimide and the corresponding di- or tetraketone (Scheme 159).^314^ These systems self-assemble into high aspect ratio crystalline tapes and might be usable as n-channel semiconductors with charge carrier mobilities ranging from 0.18 up to 4.1 cm^2^ V^–1^ s^–1^ for **159.2b** and **159.1a**, respectively. The luminescence of the N*-*alkylated imides, **159.1b** and **159.2b**, was quenched in the presence of acid. Pyrene-fused azahexacene **159.4a** and azaheptacene **159.4b** containing boron–nitrogen units were synthesized by treatment of either **159.3a** or **159.3b** with BF_3_·Et_2_O and triethylamine in DCM at 50 °C (Scheme 159).^315^**159.4a** and **159.4b** exhibited low-lying LUMO energy levels and high electron affinities, thus demonstrating their n-type character. As a result, solution-processed OFET devices based on **159.4b** exhibited unipolar n-type characteristics with an electron mobility of up to 0.21 cm^2^ V^–1^ s^–1^. A lowering of LUMO energies correlating with the electron-withdrawing character of substituents was also demonstrated for perylene-functionalized acenes **159.5**–**7** and **159.7a**–**c**.^316^

**Scheme 160:**
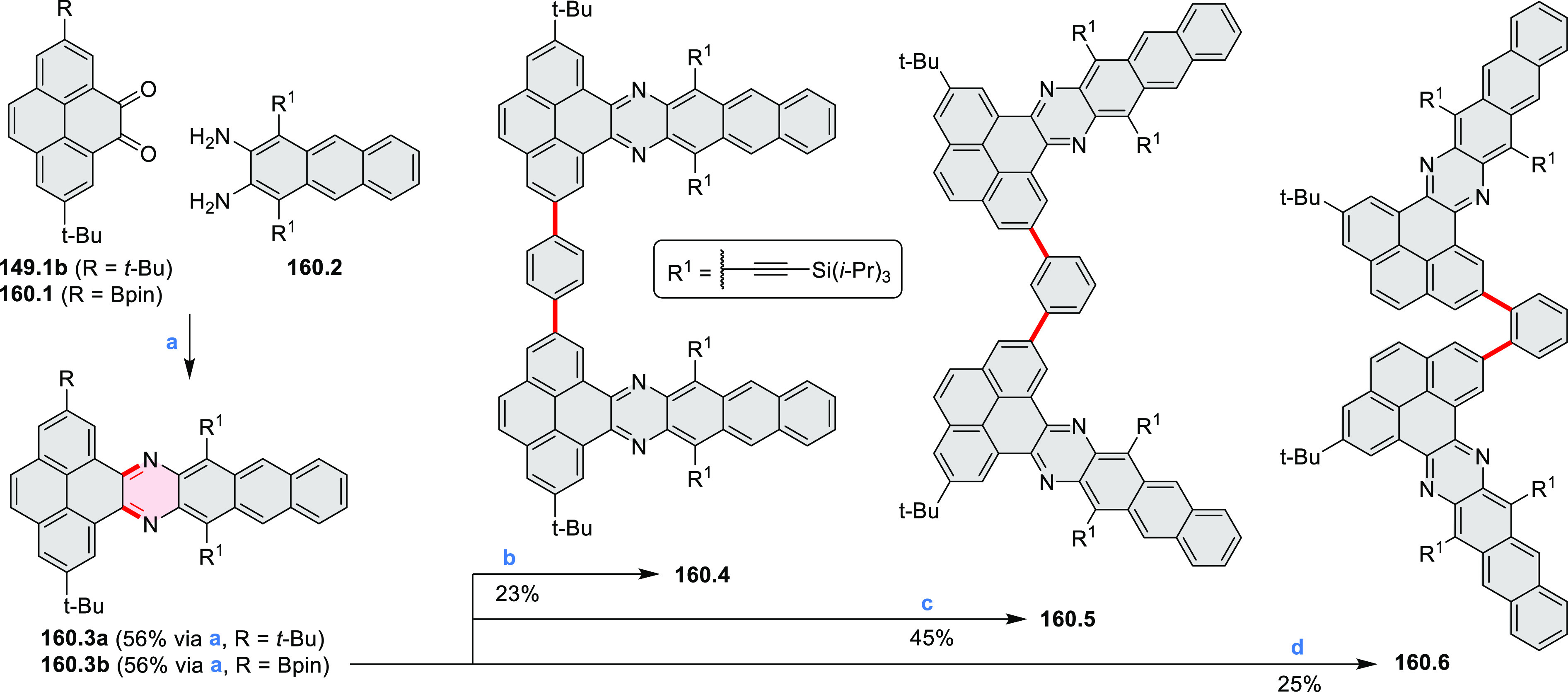
Synthesis of Pyrene-Fused Azaacene Dimers Reagents and conditions: (a)^317^ CHCl_3_, AcOH, reflux; (b) *p*-diiodobenzene, PdCl_2_(dppf), K_3_PO_4_, THF, H_2_O, 80 °C, 11 min, microwave heating; (c) *m*-diiodobenzene, PdCl_2_(dppf), K_3_PO_4_, THF, H_2_O, 80 °C, 11 min, microwave heating; (d) *o*-diiodobenzene, PdCl_2_(dppf), K_3_PO_4_, THF, H_2_O, 80 °C, 11 min, microwave heating.

Three isomeric pyrazaacene dimers that undergo singlet fission with triplet quantum yields as high as 125% were developed by Franko, Guldi, and Mateo-Alonso (Scheme 160).^317^ Cyclocondensation of dione **160.1** and anthracenediamine **160.2** yielded the asymmetric dibenzodiazahexacene **160.3b** with a boronate ester that was further engaged in cross-coupling reactions (Scheme 160). The dimers were obtained via microwave-assisted Suzuki reaction between the precursor **160.3b** with *ortho*-, *meta*-, and *para*-diiodobenzene, affording, respectively, the desired **160.4**, **160.5**, and **160.6**. Pyrazaacene monomer **160.3a** and dimers **160.4**–**6** were all found to fluoresce in the red to NIR region with the quantum yields of 25%, 19%, 20%, and 13%, respectively, recorded in toluene. The optical HOMO–LUMO gaps (*E*_gap_ ≈ 1.8 eV) and electrochemical HOMO–LUMO gaps (*E*_gap_ ≈ 1.8 eV) were found to be nearly identical for all the pyrazaacene derivatives and, according to computational results, met the energetic requirements (2T_1_=S_1_) for singlet fission (SF) in dimers. A detailed investigation of the excited-state dynamics using a kinetic model fit, from femtosecond (fs-TAS) and nanosecond transient absorption (ns-TAS) data, showed that **160.6** underwent SF with ^1^(T_1_T_1_) triplet quantum yields (TQYs) of 125% in toluene. The lower TQYs of 92% found in benzonitrile indicated that a change in solvent polarity affected the energetic levels of excited-state species. TQYs of ^1^(T_1_T_1_) were found to be lower in value for **160.4** and **160.5** with 82% and 70% in toluene. The observed high TQYs in the *o*-dimer (**160.6**) were ascribed to the close spatial proximity between the two dibenzoazahexacenes, leading to an enhanced electronic communication in comparison with the *m*-dimer (**160.5**) or *p*-dimer (**160.4**).

**Scheme 161:**
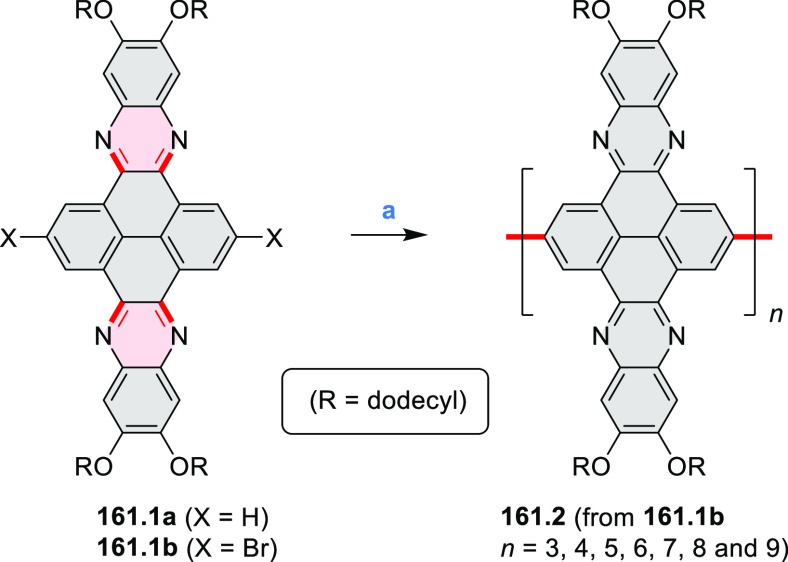
Synthesis of Poly(2,11-diquinoxalinopyrene) Reagents and conditions: (a)^318^ (1) bis(1,5-cyclooctadiene) nickel(0), 1,5-cyclooctadiene, 2,2′-bipyridine, DMF, 85 °C, 30 min, (2) **161.1b** in toluene, 12–48 h, (3) bromobenzene, 4 h.

Homopolymerization of an alkoxy-substituted pyrazaacene was reported by Ling and Mo et al. (Scheme 161).^318^ The poly(2,11-diquinoxalinopyrene) was obtained by subjecting the bromo derivative **161.1b** to Yamamoto reaction conditions. By varying the reaction time, it was possible to obtain oligomers with variable lengths and molar weights of up to 7300 Da. The oligomers showed limited band gap dependence on their length, indicating that the conjugation between subunits was relatively weak.

**Scheme 162:**
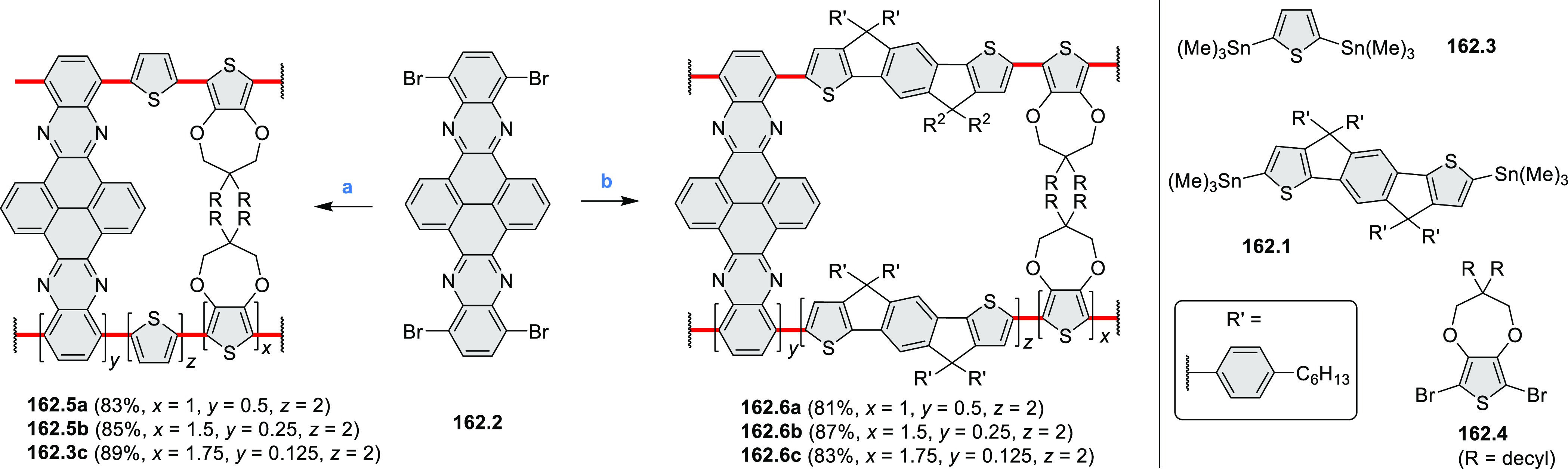
Synthesis of Thiophene- and Pyrazaacene-Based Oligomers Reagents and conditions: (a)^319^ Pd(PPh_3_)_2_Cl_2_, toluene, 100 °C, 48 h, **162.2**/**162.3**/**162.4** were used in molar ratios of 0.5:2:1, 0.25:2:1.5, and 0.125:2:1.75 for **162.5a**, **162.5b**, and **162.5c**, respectively; (b)^320^ Pd(PPh_3_)_2_Cl_2_, toluene, 100 °C, 48 h, **162.2**/**162.1**/**162.4** were used in molar ratios of 0.5:2:1, 0.25:2:1.5, and 0.125:2:1.75 for **162.6a**, **162.6b**, and **162.6c**, respectively.

Electrochromic copolymers with the general structures **162.5**–**6** were obtained by Stille cross-coupling of the pyrazaacene **162.2** with dibromothiophene **162.4** and one of the distannanes **162.1** or **162.3** in varying molar ratios (Scheme 162).^319^ All three polymers **162.5a**–**c** had band gaps smaller than 1.85 eV and satisfactory thermal stability. The polymers showed electrochromic switching with high optical contrast and fast response time in both the visible and near-infrared regions. For **162.5c**, the optical contrasts were 53.35% at 540 nm and 66.49% at 1325 nm, with coloration efficiencies of 163 cm^2^ C^–1^ at 540 nm and 205 cm^2^ C^–1^ at 1325 nm. A similar performance was obtained for **162.6a**–**c**, which had somewhat larger band gaps of ca. 2.0 eV.

**Scheme 163:**
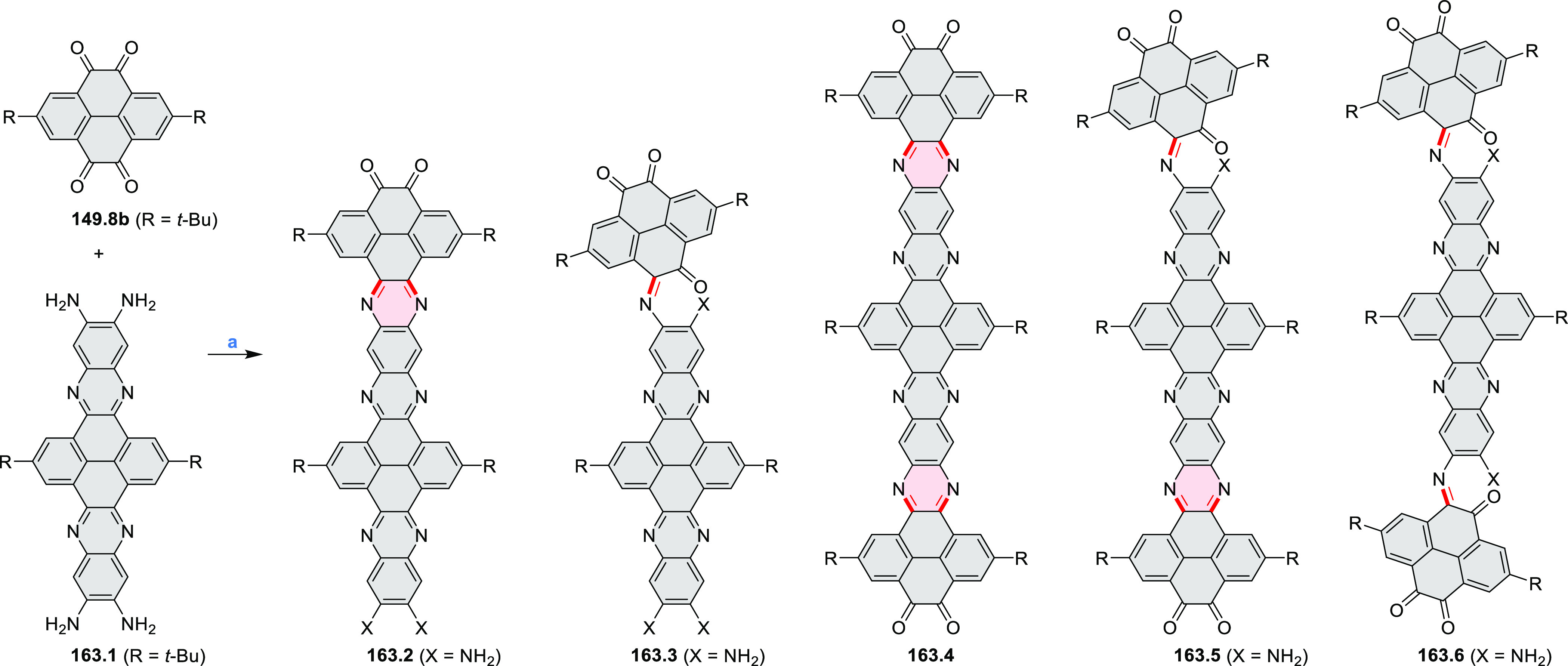
Synthesis of Pyrene-Fused Pyrazaacenes on Metal Surfaces Reagents and conditions: (a)^321^ Ag(111), *T*_STM_ = rt to 510 K, *U*_s_ = 2.22 V, *I*_t_ = 0.09 nA.

Papageorgiou, Reichert, and Mateo-Alonso investigated the reaction of tetraketone **149.8b** and the tetraamine **163.1** for the in situ formation of pyrene-fused pyrazaacene-based oligomers on three close-packed coinage metal surfaces (Au, Ag, and Cu) under ultrahigh vacuum conditions (Scheme 163).^321^ They found that, in contrast to the reaction on Ag(111), the reactants desorb from the Au(111) surface before reacting, whereas they decompose on the Cu(111) surface during thermal treatment. To promote cyclocondensation, **149.8b** and **163.1** were deposited onto the Ag(111) surface at rt and subsequently annealed gradually to 510 K. The simplified classification of the occurring oligomers includes straight **163.2** and bent **163.3** dimers and diketone-terminated trimers **163.4**–**6** (Scheme 163). Formation of tetramers and longer oligomers was also observed. To investigate the effect of stoichiometry, the tetraketone to tetraamine ratio was tuned from ca. 1:5 to 4:1. For every investigated stoichiometry and a given oligomer length, the straight oligomer type was preferred to the corresponding bent type.

**Chart 17:**
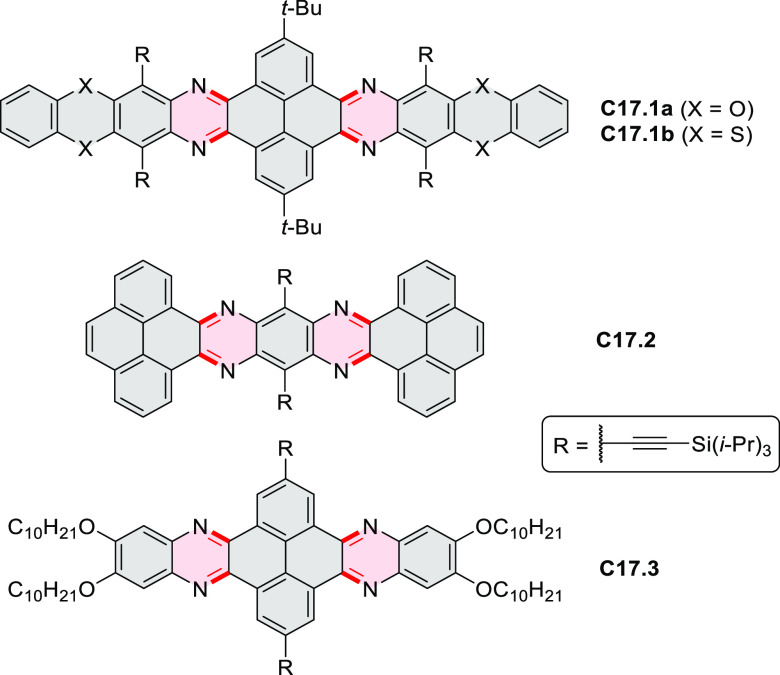
Symmetric Heteroacenes and Pyrazine-Fused Pyrenoids

In 2019, Li and co-workers synthesized tetraazatetraoxadecacene **C17.1a** and tetraazatetrathiadecacene **C17.1b**, heteroacenes with ten linearly fused rings containing, respectively, embedded O/N or S/N atoms (Chart 17).^322^ Crystal structures showed that the O*-*containing **C17.1a** exhibited linear conformations, while the S*-*containing **C17.1b** was noticeably bent. **C17.1a** showed a fluorescence quantum yield of 0.55 in DCM, which was more than 10 times higher than that of **C17.1b** (Φ_f_ = 0.05). Upon simple reprecipitation, **C17.1a** and **C17.1b** self-assembled into microwires and microrods, respectively, which demonstrated distinctive nonlinear optical properties depending on the orientation of transition dipoles. The synthesis of pyrene-fused tetraazaheptacene **C17.2** consisting of two terminal pyrene units and a central tetraazaanthracene core substituted by two solubilizing triisopropylsilylethynyl (TIPS) groups was described by Hueso and Mateo-Alonso et al.^323^**C17.2** exhibited a strong absorption in the UV–vis region with the lowest-energy transition at 576 nm and an orange to red photoluminescence, which is centered at 620 nm and exhibits a shoulder at 666 nm. The high stability of **C17.2** allowed the preparation of thin films via sublimation. Charge transport studies on thin films of **C17.2** show a p-type semiconducting behavior for heptacene **C17.2**. Hole mobilities are in the range of 0.2 × 10^–6^ cm^2^ V^–1^ s^–1^. Symmetric donor–acceptor *N-*heteroacene **C17.3** possessing electron-donating decyloxy substituents and pyrazine rings as acceptor units were investigated by Li, Lu, and Zhang as materials for memory devices.^298^

**Chart 18:**
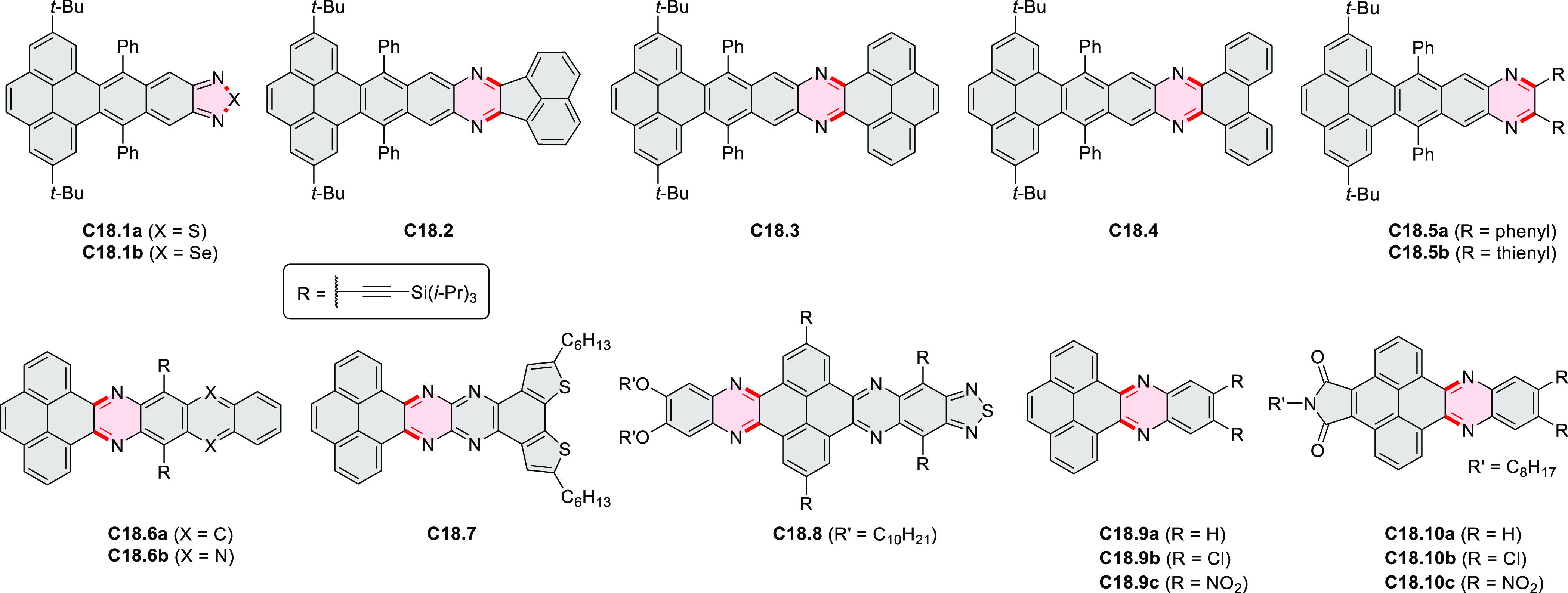
Unsymmetrical Pyrazaacenes

As shown above, synthetic methods available in pyrazaacene chemistry provide access to various nanoribbon structures with nonequivalent termini. This possibility was also explored by Xiao and co-workers, who reported a family of π-extended systems **C18.1**–**5** with the two ends capped, respectively, with a pyrene moiety and a variety of ring systems (Chart 18).^324^ In particular, compound **C18.1a** was obtained by reacting the appropriate diamine derivative with thionyl chloride in the presence of triethylamine in DCM, while compound **C18.1b** was prepared through the reaction between the same diamine and selenium oxide in hot ethanol. All these compounds exhibit weak fluorescence (Φ_f_ = 0.024–0.13), with emissions ranging from the yellow through orange up to red region. Spectral features of derivatives containing pyrazine rings, i.e., **C18.2**–**5**, were significantly affected by addition of a strong protic acid. Further examples of similar designs include hexacenes **C18.6a**,**b**,^325^ the thiophene-fused system **C18.7**,^326^ and pyrazaacenes **C18.8**–**10**, which were successfully employed in memory devices.^298,327^

**Chart 19:**
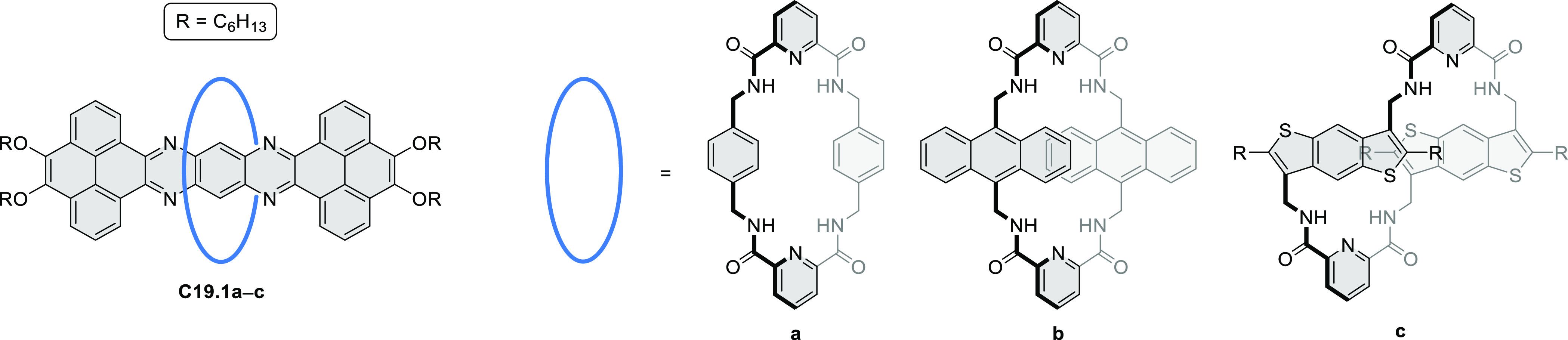
Mechanically Interlocked Nitrogenated Nanographenes

In 2020, Mateo-Alonso et al. reported a series of rotaxanes **C19.1a**–**c** consisting of a nitrogenated nanographene and a tetraamide-based macrocycle (Chart 19).^328^ The synthesis was accomplished using the clipping strategy, in which the macrocycle formation was assisted by hydrogen bonding between the sp^2^-hybridized nitrogen atoms on the nanographene and amide groups. The structure of **C19.1b** was confirmed by X-ray crystallography. All three mechanically interlocked systems showed improved photostability under UV irradiation relative to the isolated nanographene.

### 4.7 Other [*e*]Fused Pyrenoids

#### 4.7.1 Pyrrole- and Indole-Containing Systems

**Scheme 164:**
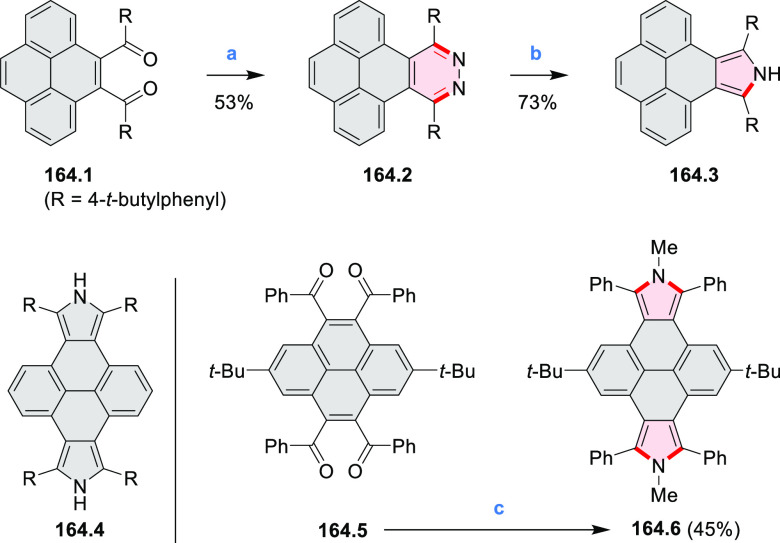
Synthesis of the Pyridazine- and Pyrrole-Extended Pyrenes Reagents and conditions: (a)^329^ N_2_H_5_OH, pyridine, water, reflux, 3 h; (b) Zn, DCM, AcOH, rt, 3 h; (c)^330^ HCOONH_3_CH_3_, 145 °C, 24 h.

Benzoylpyrenes **164.1** and **164.5**, obtained via an oxidative ring opening of cyclopentadienone units, were used by Mastalerz et al. for the synthesis of hetero-[*c*]-annulated pyrenes (Scheme 164; for related systems, see Schemes 170, 173, and 177, in subsequent subsections).^329^ Pyrrole rings, in particular, were obtained in a two-step approach consisting of condensation with hydrazine hydrate followed by reductive ring contraction with zinc dust, yielding **164.3** and **164.4**. These two compounds exhibit blue emission with quantum yields of 37 and 40%, respectively. An alternative method, due to Liu and Zhang, involved heating with molten methylammonium formate, yielding the N*-*methylated derivative **164.6** in moderate yield (45%, Scheme 164; cf. Schemes 173 and 177 below).^330^ Fluorescence emission **164.6** was blue-shifted relative to the starting dibenzoylpyrene **164.5** as well as the chalcogen-containing analogues (**173.3**, Scheme 173, and **177.3**–**4**, Scheme 177).

#### 4.7.2 Imidazole-Containing Systems

**Scheme 165:**
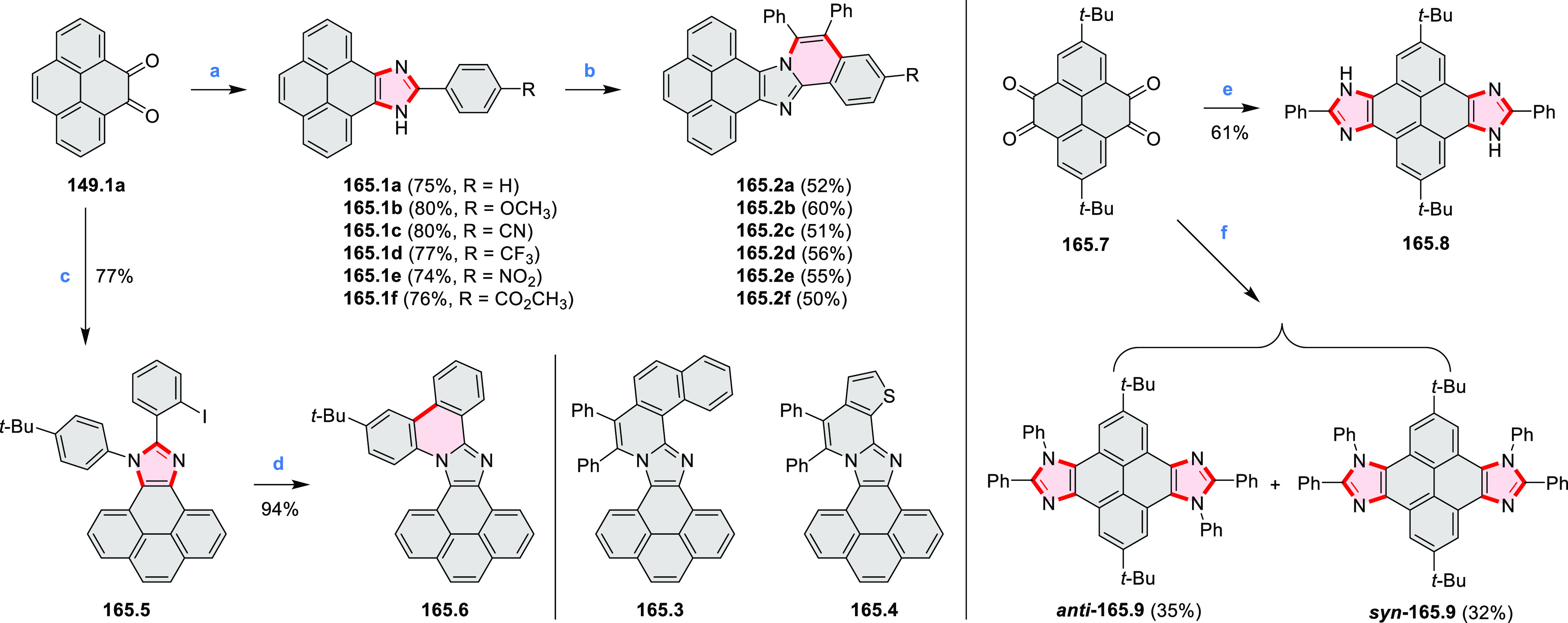
Syntheses of Pyrenoimidazole Derivatives Reagents and conditions: (a)^334^ 4-R-benzaldehyde (R = H, OCH_3_, CN, CF_3_, NO_2_, or CO_2_CH_3_), NH_4_OAc, AcOH, reflux, 24 h; (b) [RuCl_2_(*p*-cymene)]_2_, 1-adamantanecarboxylic acid, Cu(OAc)_2_, K_2_CO_3_, diphenylacetylene, 120 °C, 16 h; (c)^333^ 4-*tert*-butylaniline, 2-iodobenzaldehyde, NH_4_OAc, AcOH, 110 °C, 5 h; (d) *hν*, 48–72 h; (e)^331^ benzaldehyde, NH_4_OAc, dioxane, 120 °C, 3 days; (f)^332^ aniline, benzaldehyde, NH_4_OAc, AcOH, reflux, 2 h.

Pyrene diones and tetraones such as **149.1a** and **165.7** provide straightforward access to pyreno-fused mono- and diimidazoles, which can be used as handles for further ring fusion or metal coordination. The Debus–Radziszewski reaction used in these syntheses (Scheme 165) is efficient enough to be used in COF syntheses (cf. Scheme 393, Section 7.7.1),^331^ but it will produce regioisomers for N*-*substituted diimidazoles (e.g., *anti*-**165.9** and *syn*-**165.9**).^332^ π-Extension of such imidazoles via light-induced direct arylation in the solid state was reported in 2016 by Skonieczny and Gryko.^333^ Photochemical direct arylation was then performed via evaporation of a solution of **165.5** on a quartz glass followed by irradiation with a UV lamp, to produce **165.6**. The same approach was also applied to acenaphthylene-1,2-dione (Scheme 262, Section 6.2.1). Fusion of the additional rings in **165.6** led to a dramatic increase of fluorescence quantum yield from less than 0.0001 for **165.5** to 0.75 for **165.6**. Oxidative annulation of imidazoles **165.1a**–**f** with diphenylacetylene in the presence of [RuCl_2_(*p*-cymene)]_2_ as a catalyst was found to produce the isoquinolino-fused products **165.2a**–**f**.^334^ The same reaction sequence was employed to obtain naphthalene and thiophene analogues **165.3** and **165.4**, respectively. **165.2e** showed distinct solvatofluorochromism, which was attributed to intramolecular charge transfer due to the electron-withdrawing NO_2_ group.

**Scheme 166:**
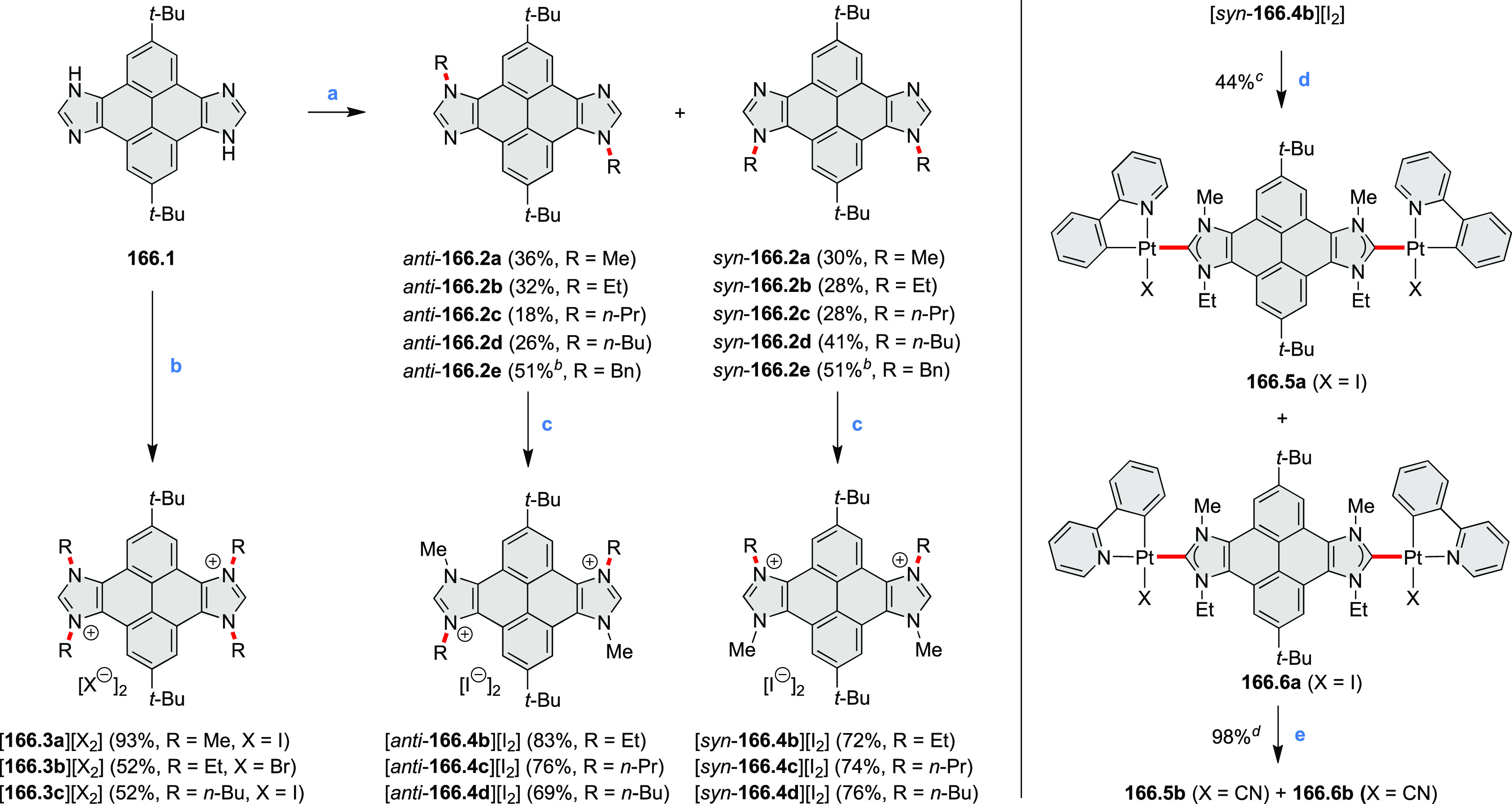
Pyrene-Fused Imidazolium Cations and Their NHC Complexes Reagents and conditions: (a)^335^ (1) NaOH, DMSO, rt, 2 h, (2) RBr or RI (2 equiv), rt for 30 min, then 37 °C, overnight; (b) (1) NaOH, DMSO, rt, 2 h, (2) RBr or RI (excess), rt for 30 min, then 37 °C, overnight; (c) MeI (excess), 100 °C, 24 h; (d) [Pt(ppy)(μ-Cl)]_2_, (ppy = 2-phenylpyridinate), NaOAc, NaI, DMSO, 100 °C, overnight; (e) (1) AgBF_4_, DCM, 1 h, (2) NaCN, rt, overnight. For **166.2e***anti* and *syn* isomers were obtained as a mixture in 1:1 ratio and were not separated. Total yield is given. Isomers **166.5a** and **166.6a** were obtained as a mixture in 6:4 ratio and were not separated. Total yield is given. **166.5b** and **166.6b** were obtained as a mixture in 1:1 ratio and were not separated. Total yield is given.

One- or two-step alkylation of **166.1** was used by Poyatos and Peris et al. to obtain the tetraalkyldiimidazolium salts **166.3**–**4** (Scheme 166).^335^ Intermediates **166.2a**–**e** were obtained as mixtures of the anti and syn regioisomers, which could usually be separated and converted into the corresponding tetraalkyl targets. The reaction between [{Pt(ppy)(μ-Cl)_2_}_2_] and [*syn*-**166.4b**][I_2_] performed in the presence of sodium acetate and sodium iodide produced a mixture of the corresponding bis-*N-*heterocyclic carbene complexes **166.5a** and **166.6a**, as two isomers were obtained in 6:4 molar ratio. Iodide abstraction with a silver salt and subsequent reaction with NaCN afforded a mixture of the isomeric complexes **166.5b** and **166.6b**, which contain strong-field CN^–^ auxiliary ligands. All the compounds **166.2**–**6** showed emissions in the range of 379–423 and 370–420 nm for the neutral bisazole **166.2a**–**e** and dicationic bisazolium compounds **166.3**–**6**, respectively, with quantum yields in the range of Φ_f_ = 0.20–0.55. Both the emissions and the quantum yields were only slightly sensitive to the nature of the bisazoles and their substituents.

**Scheme 167:**
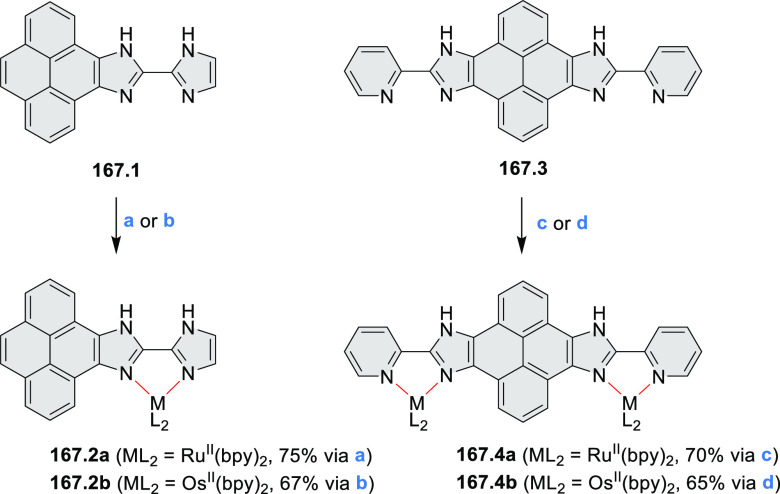
Synthesis of the Pyrene-Biimidazole-Based Ru(II) and Os(II) Complexes Reagents and conditions: (a)^336^ (1) *cis*-[Ru(bpy)_2_Cl_2_]·2H_2_O, AgClO_4_, EtOH, reflux, 1 h, (2) **167.1**, reflux, 10 h; (b) (1) *cis*-Os(bpy)_2_Cl_2_, EtOH, water, reflux, 40 h, (2) NaClO_4_, rt; (c)^338^ (1) *cis*-[Ru(bpy)_2_Cl_2_]·2H_2_O, ethylene glycol, reflux, overnight, (2) NaClO_4_, water, rt; (d) (1) *cis*-Os(bpy)_2_Cl_2_, ethylene glycol, reflux, overnight, (2) NaClO_4_, water, rt.

Two mononuclear ruthenium(II) and osmium(II) complexes containing a pyrene-fused biimidazole ligand were designed by Baitalik et al. as cyanide ion sensors for aqueous media.^336^ The monometallic complexes **167.2a**,**b** were synthesized by reacting **167.1** with the appropriate metal precursor (Scheme 167). Both complexes showed high selectivity toward cyanide ions in aqueous media in the presence of an excess of other anions, with measurable responses in absorption and emission spectra as well as in voltammetric measurements. Analogous binuclear complexes **167.4a**,**b** were found to interact with CT-DNA via an intercalative binding mode with intrinsic binding constants on the order of 10^5^ M^–1^.^337,338^

**Scheme 168:**
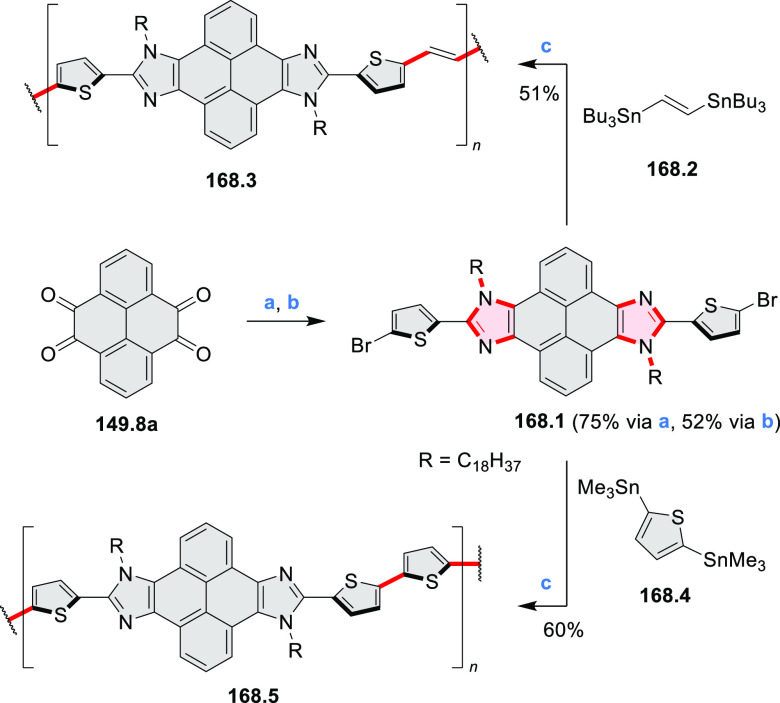
Synthesis of Pyrenodiimidazole Polymers Reagents and conditions: (a)^339^ 5-Bromothiophene-2-carbaldehyde, NH_4_OAc, AcOH, reflux, 10 h; (b) 1-bromooctadecane, DMF, 100 °C, 12 h; (c) Pd(PPh_3_)_4_, THF, 65 °C, 16–20 h.

Pyrenodiimidazole **149.8a** was copolymerized with vinylene **168.2** and thiophene **168.4** by Stille coupling reaction to furnish two conjugated polymers, **168.3** and **168.5** (Scheme 168).^339^ The two polymers exhibited coplanar backbones, similar LUMO energy levels at −3.7 eV, and uniformly delocalized HOMOs with low energies of ca. −5.7 eV. X-ray diffraction and grazing-incident X-ray diffraction measurements demonstrated that **168.5** adopted a highly ordered structure, reflected in an enhanced hole mobility of 0.015 cm^2^ V^–1^ s^–1^ in organic thin-film transistors. In contrast, the thin film of **168.3** was disordered with a hole mobility of up to 2.22 × 10^–3^ cm^2^ V^–1^ s^–1^.

**Scheme 169:**
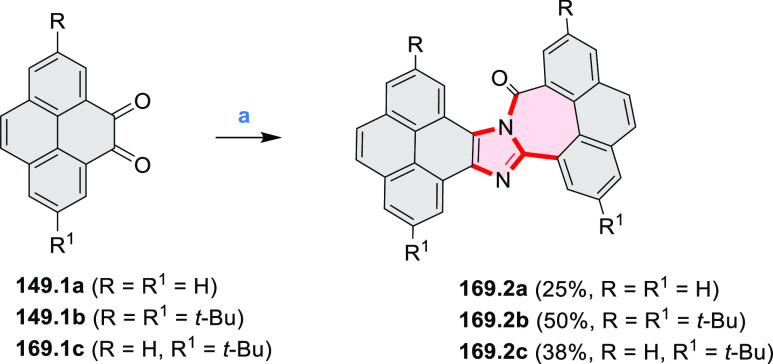
Self-Condensation of Pyrene-4,5-dione Reagents and conditions: (a)^340^ NH_4_OAc, AcOH, 100 °C.

When a mixture of pyrene-4,5-dione **149.1a**, ammonium acetate, and glacial acetic acid was heated at 100 °C for 10 h, self-condensation was observed, resulting in the formation of **169.2a**, 6*H*-phenanthro[4,5-*cde*]pyreno[4′,5′:4,5]imidazo[1,2-*a*]azepin-6-one in 25% yield (Scheme 169).^340^ To address the solubility problem of **169.2a**, *tert*-butyl-substituted pyrene-4,5-diones **149.1b** and **169.1c** were subjected to the same conditions to afford analogous products with both improved solubility and yields. **169.2b** exhibited solvato-, mechano-, and acidofluorochromic properties, presumed to originate from excimer/monomer equilibria.

#### 4.7.3 Other Nitrogen-Containing Systems

**Scheme 170:**
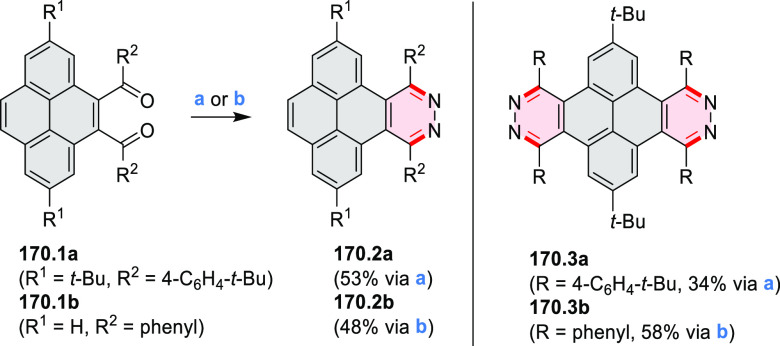
Synthesis of the Pyridazine-Extended Pyrenes Reagents and conditions: (a)^329^ N_2_H_5_OH, pyridine, water, reflux, 3 h; (b)^341^ N_2_H_5_OH, MeOH, 70 °C, 72 h.

Pyridazines **170.2a**,**b** were obtained by direct condensation of corresponding benzoylpyrenes with hydrazine hydrate (Scheme 170).^329^ Both **170.2a** and **170.3a** underwent reductive contraction to yield the corresponding pyrrole analogues (Scheme 164, Section 4.7.1). **170.2b** and **170.3b** exhibited moderate two-photon absorption cross-section values, reaching 454 GM in the case of **170.3b**.^341^

**Scheme 171:**
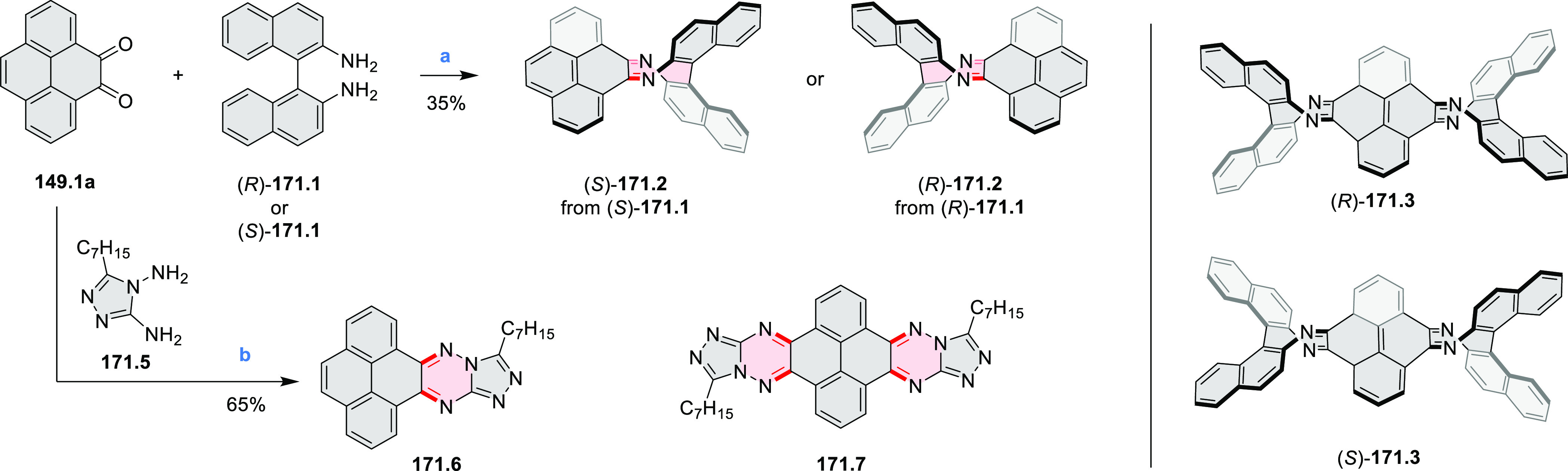
Synthesis of Chiral Pyrene-Based Compounds Reagents and conditions: (a)^343^ (*R*)- or (*S*)-(±)-2,2′-diamino-1,1′-binaphthalene, CF_3_COOH, toluene, 60 °C, 3 h; (b)^342^ AcOH, reflux, overnight.

Structural variety can be created by condensing pyrene di- and tetraones with untypical aromatic diamines. A recent example is provided by condensations with 3,4-diamino-5-heptyl-1,2,4-triazole **171.5**, which yielded the triazolo–triazines **171.6**–**7** (Scheme 171, cf. Scheme 262, Section 6.2.1, for an acenaphthylene analogue).^342^ LUMO energy levels were calculated to be −3.77 eV and −3.95 eV for **171.6** and **171.7**, respectively, indicating their potential utility as n-type semiconductors. Condensations with pure enantiomers of 2,2′-diamino-1,1′-binaphthalene **171.1** furnished the chiral systems **171.2**–**3** containing twisted 1,4-diazocine rings.^343^ The CD spectra of **171.2**–**3** demonstrated chiral induction between the chiral binaphthalene group(s) and the pyrene chromophore.

**Scheme 172:**
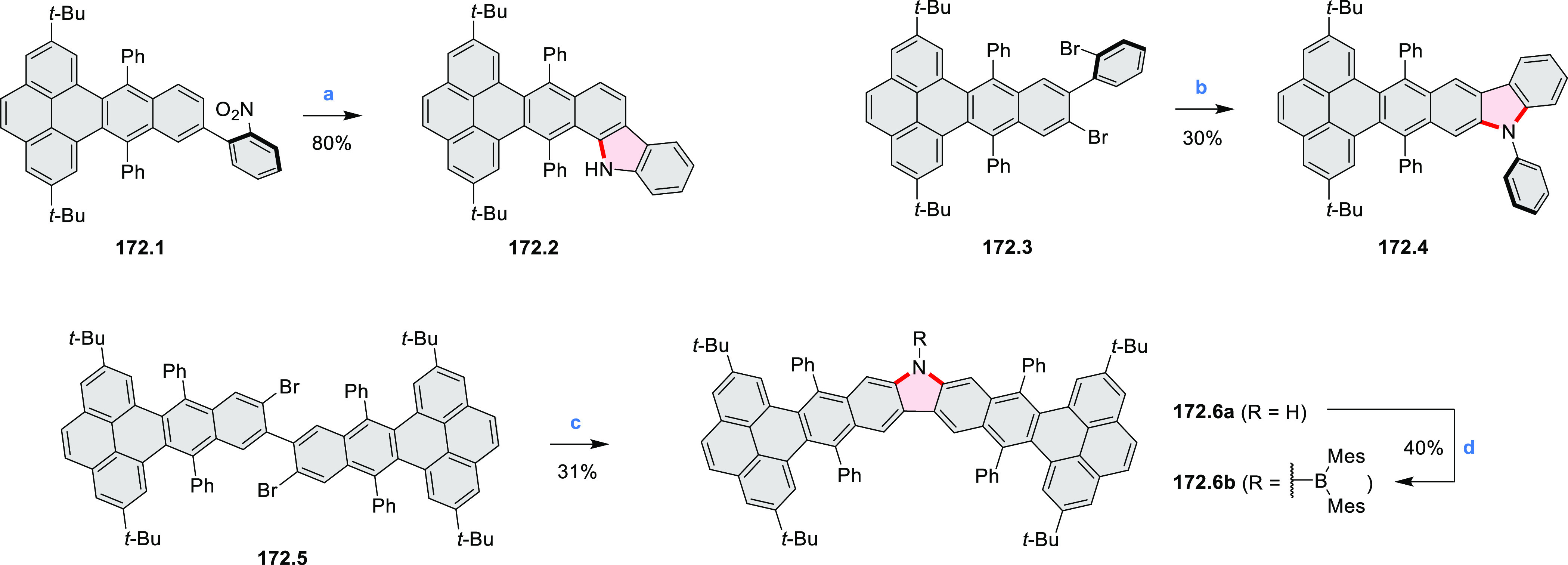
Synthesis of Unsymmetrical Azatwistacenes Reagents and conditions: (a)^344^ P(OEt)_3_, *o*-DCB, 160 °C, 24 h; (b) aniline, Pd(OAc)_2_, PCy_3_, *t*-BuOK, toluene, reflux, 48 h; (c)^345^ NaN_3_, CuI, *N*,*N*′-dimethylethylenediamine, DMSO, 120 °C, 48 h; (d) (1) *n*-BuLi, THF, −78 °C, 2 h, (2) dimesitylboron fluoride, rt, 24 h.

Azatwistacenes **172.2** and **172.4** containing fused carbazole moieties were synthesized using respectively the Cadogan reaction and a Pd-catalyzed annulative amination (Scheme 172, for a furan derivative see Scheme 175, Section 4.7.4).^344^ The former reaction was regioselective, producing exclusively the angularly fused isomer **172.2**. The related bent twistacene **172.6a** was synthesized from the respective dibromo precursor using a Cu-catalyzed amination reaction.^345^ Compound **172.2** displayed blue fluorescence in DCM solution with maxima peaks at 455 and 475 nm and a quantum yield of 0.29, while **172.4** showed the bathochromic shift to 481 and 510 nm, resulting in green emission (Φ_f_ = 0.32). Twistacenes **172.2** and **172.4** were tested as dopants in multilayered OLED devices and exhibited promising electroluminescent performance. **172.6a** was found to be solvatofluorochromic, its fluorescence changing from green to yellow with increasing solvent polarity. In contrast, the dimesitylboryl derivative **172.6b** emitted blue light with no significant solvent dependence.

#### 4.7.4 Oxygen-Containing Systems

**Scheme 173:**
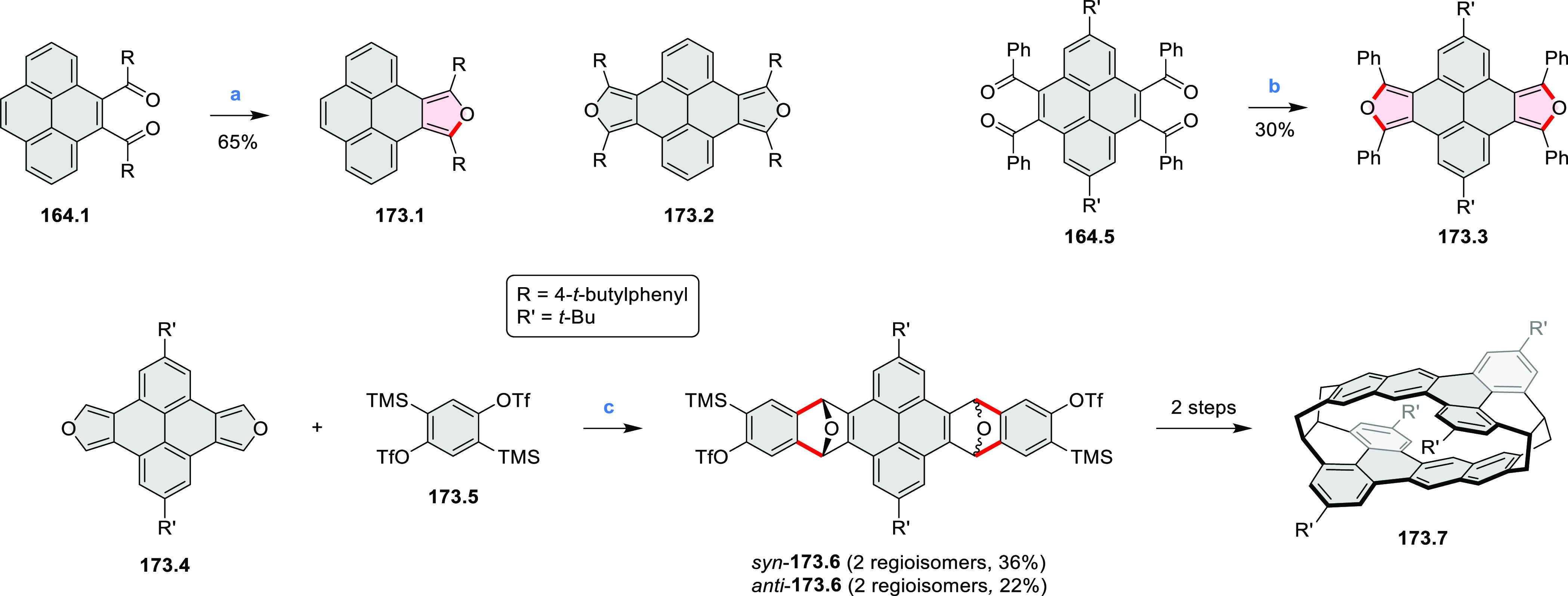
Synthesis and Reactivity of Furan-Extended Pyrenes Reagents and conditions: (a)^329^ LiAlH_4_, Et_2_O, rt, 30 min; (b)^330^ NaBH_4_, MeOH, rt, overnight; (c)^347^ CsF, MeCN/THF, 40 °C, overnight.

Pyrenoids with K-region-fused furans **173.1**–**3** were recently accessed via LiAlH_4_ or NaBH_4_ reduction of the corresponding benzoyl derivatives followed by aqueous acidic workup (Scheme 173).^329,330^ This method complements other synthetic transformations of pyrene ketones leading to [*c*]heteroannulated products (Schemes 164, 170, and 177). **173.1** and **173.2** show blue emission with the quantum yields of 0.16 and 0.24, respectively. The furan rings in such furan-fused pyrenes can act as dienes in Diels–Alder reactions. For example, as reported by Miao et al., pyrenodifuran **173.4** reacted with the benzyne generated *in situ* from **173.5** in a 1:2 stoichiometry to give **173.6** as an isomeric mixture. The regioisomeric mixture of *syn*-**173.6** could be isolated and was then converted to the hydrogenated zigzag nanobelt **173.7** in two steps. The first fully conjugated zigzag nanobelt, reported in 2021 by Itami and Segawa et al., was synthesized using a similar synthetic approach.^346^

**Scheme 174:**
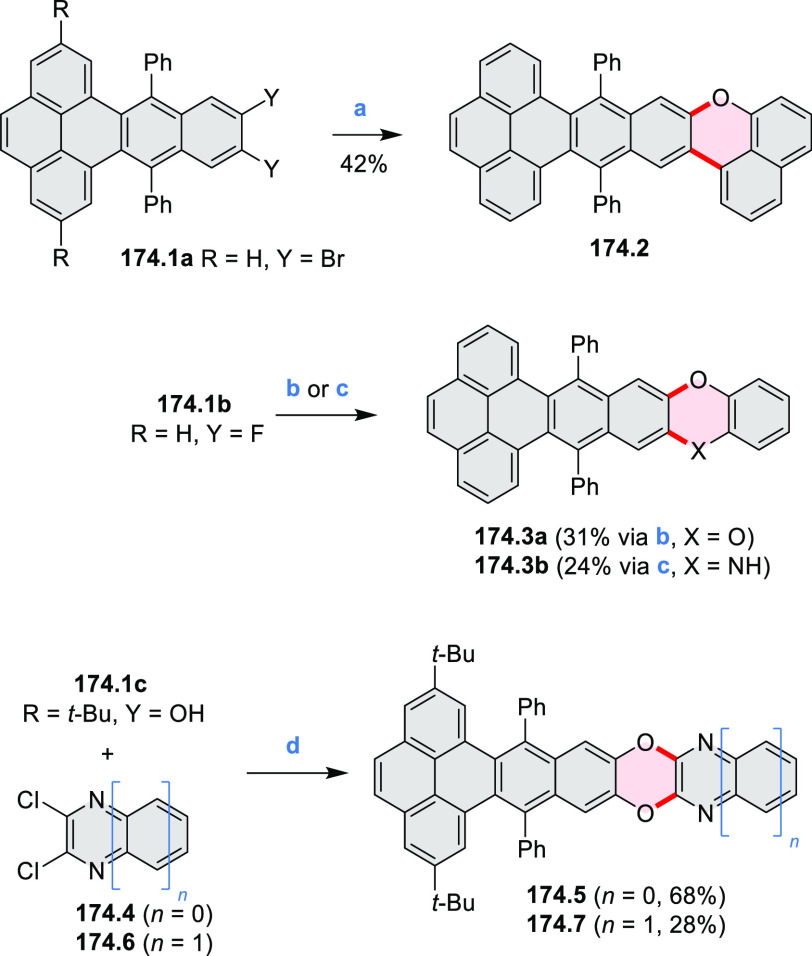
Synthesis of Oxygen- and Nitrogen-Doped Twistacenes Reagents and conditions: (a)^348^ 1-naphthol, PPh_3_, Cs_2_CO_3_, Pd(OAc)_2_, DMF, 140 °C, 24 h; (b)^349^ catechol, NaH, NMP, 205 °C, 12 h; (c) 2-aminophenol, NaH, NMP, 205 °C, 12 h; (d)^350^ K_2_CO_3_, DMF, 110 °C.

Laterally π-extended twistacenes (also known as twistarenes) consisting of 9,14-diphenyldibenzo[*de*,*qr*]tetracene and a fused heterocyclic subunit have been explored as functional dyes for various applications. A pyran-containing system **174.2** was synthesized in 42% yield via a one-step Pd-catalyzed annulation between **174.1** and 1-naphthol (Scheme 174).^348^ Under controlled conditions, **174.2** assembled into various morphologies (nanowires or nanospheres), as evidenced by SEM, UV–vis absorption, and emission data. The fabricated electroluminescent devices based on **174.2** had a maximum brightness of 4355 cd m^–2^ (bias voltage at 9.0 V) with CIE coordinates of (0.14, 0.53). **174.3a** and **174.3b** were obtained by annulative nucleophilic substitution of **174.1b** with catechol or 2-aminophenol, respectively (Scheme 174).^349^ The same approach proved to be effective in the syntheses of their sulfur-containing analogues (Scheme 180, Section 4.7.5). Photodetector devices fabricated by using compound **174.3b** and single-walled carbon nanotubes as the donor and acceptor, respectively, produced a steady photocurrent upon irradiation of a halogen lamp, displaying good stability and photosensitivity.

Dioxa derivative **174.5** and its more π-extended analogue **174.7** were also obtained using nucleophilic annulations, this time with the twistarene building block **174.4** acting as a nucleophile (Scheme 174).^350^**174.7** adopted a reclining chair conformation in the solid state, rather than the usual twisted structure. Self-assembly of **174.5** and **174.7** was investigated via the surfactant-assisted reprecipitation method. It was shown that **174.5** could self-assemble into nanobelts, and **174.7** formed nanowires in the presence of cetyltrimethylammonium bromide.

**Scheme 175:**
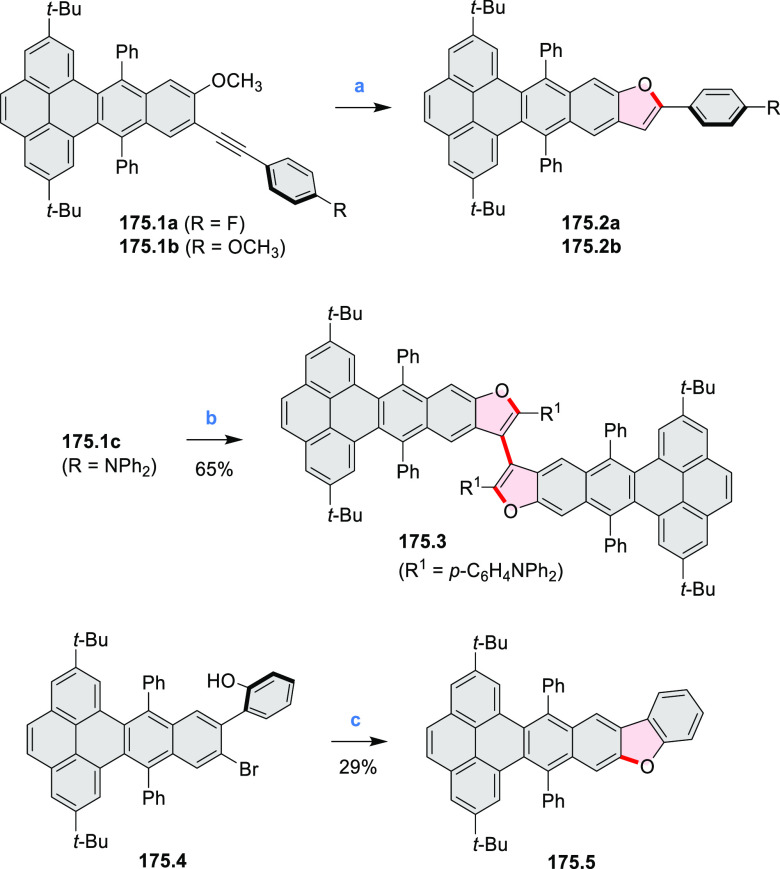
Synthesis of Oxatwistacene Reagents and conditions: (a)^351^ (1) I_2_, DCM, (2) *n*-BuLi, THF; (b) I_2_, DCM; (c)^344^ Pd(OAc)_2_, K_3_PO_4_, 2-di-*t*-butylphosphino-2′-methylbiphenyl, toluene, 100 °C, 24 h.

Benzofuran-fused twistacene **175.5** was synthesized by the intramolecular O*-*arylation of **175.4** (Scheme 175, for its nitrogen analogues, see Scheme 172, Section 4.7.3).^344^ Compound **175.5** exhibited blue fluorescence in DCM solution (λ_max_^em^ = 456 and 479 nm, QY = 38%). The related twistacenes **175.2a**,**b** were synthesized by diiodine-induced cyclization of the corresponding precursors **175.1a**,**b** followed by dehalogenation with *n*-BuLi (Scheme 175).^351^ When the triphenylamine-substituted **175.1c** was reacted under the same I_2_-mediated conditions, the dimeric **175.3** formed in 65% yield. Twistacenes **175.2a**,**b** and **175.3** as well as their precursors **175.1a**–**c** emitted blue light (QY = 0.26–0.49). Additionally, **175.2a**,**b** and dimeric **175.3** showed an increase of two-photon absorption cross sections relative to the starting twistacenes **175.1a**–**c**, which was attributed to the introduction of electron-rich furan units.

**Scheme 176:**
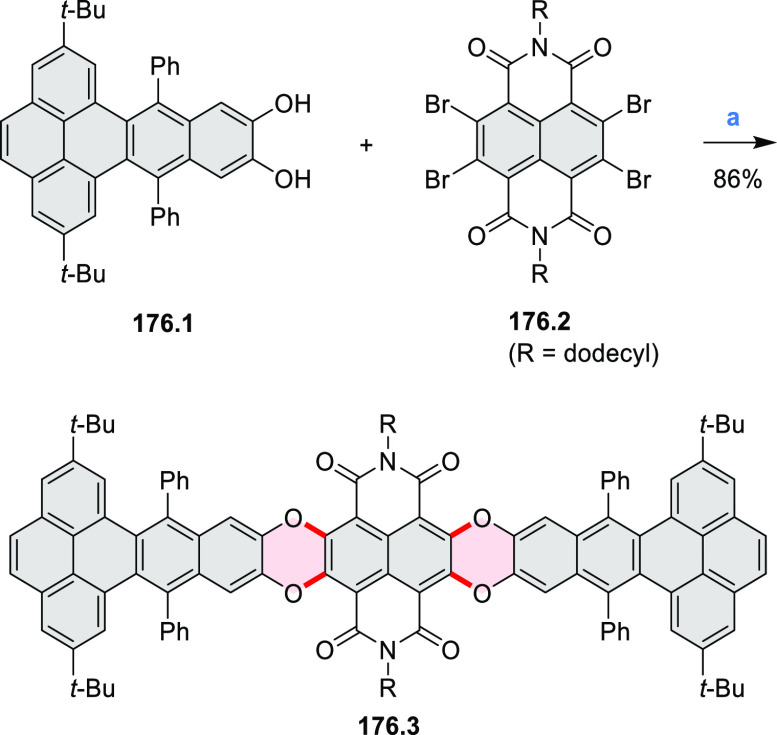
Synthesis of a Twelve-Ring Twistheteroacene Reagents and conditions: (a)^352^ K_2_CO_3_, DMF.

A donor–acceptor twistarene **176.3** containing 12 linearly fused rings was reported by Xiao and Zhang et al. (Scheme 176).^352^ In the final step, **176.1** was treated with 4,5,9,10-tetrabromo-2,7-didodecylbenzo[*lmn*][3,8]phenanthroline-1,3,6,8-tetraone **176.2** in the presence of potassium carbonate to afford the desired **176.3** as a dark red solid. **176.3** was thermally stable up to 436 °C, had absorption maxima at 510 and 538 nm, and emitted weak red fluorescence (Φ_f_ up to 1.9% in toluene). A solution-processed memory device based on **176.3** had an ON/OFF current ratio of 103.46:1 and a threshold voltage of −2.44 V.

#### 4.7.5 Sulfur-Containing Systems

**Scheme 177:**
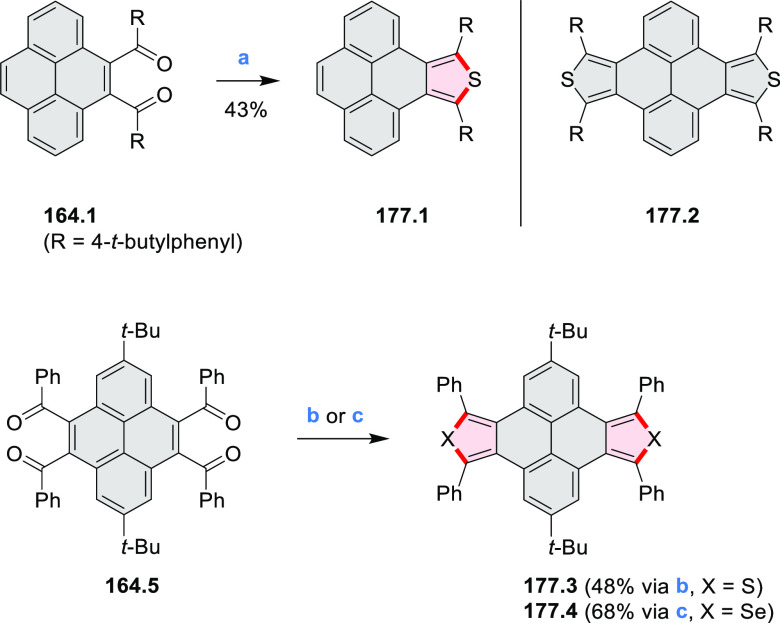
Synthesis of Thiophene and Selenophene Extended Pyrenes Reagents and conditions: (a)^329^ P_4_S_10_, NaHCO_3_, MeCN, reflux, 20 h; (b)^330^ Lawesson’s reagent, reflux, overnight, in the dark; (c) Woollins’ reagent, reflux, 2 h, in the dark.

Many of the sulfur-containing [*e*]fused derivatives discussed below were obtained as S*-*doped analogues of systems discussed in preceding subsections. For instance, the thiophene-fused pyrenes **177.1** and **177.2** were obtained by the Mastalerz group from the previously mentioned benzoylpyrenes in a reaction with sodium bicarbonate and phosphorus pentasulfide (Scheme 177).^329^ The longest absorption wavelength of the monofused system **177.1** was bathochromically shifted by 30 nm in comparison with **177.2**. This effect was explained by the lower π-conjugation in **177.2**, on the basis of additional XRD evidence. Liu and Zhang obtained the related **177.3** and its selenium analogue **177.4** using, respectively, Lawesson’s and Woollins’ reagents (Scheme 177).^330^**177.3**, **177.4**, and the oxygen analogue **173.3** all exhibited the same HOMO energy level of −5.29 eV, lower than the HOMO of −5.06 eV for the nitrogen analogue **164.6**.

**Scheme 178:**
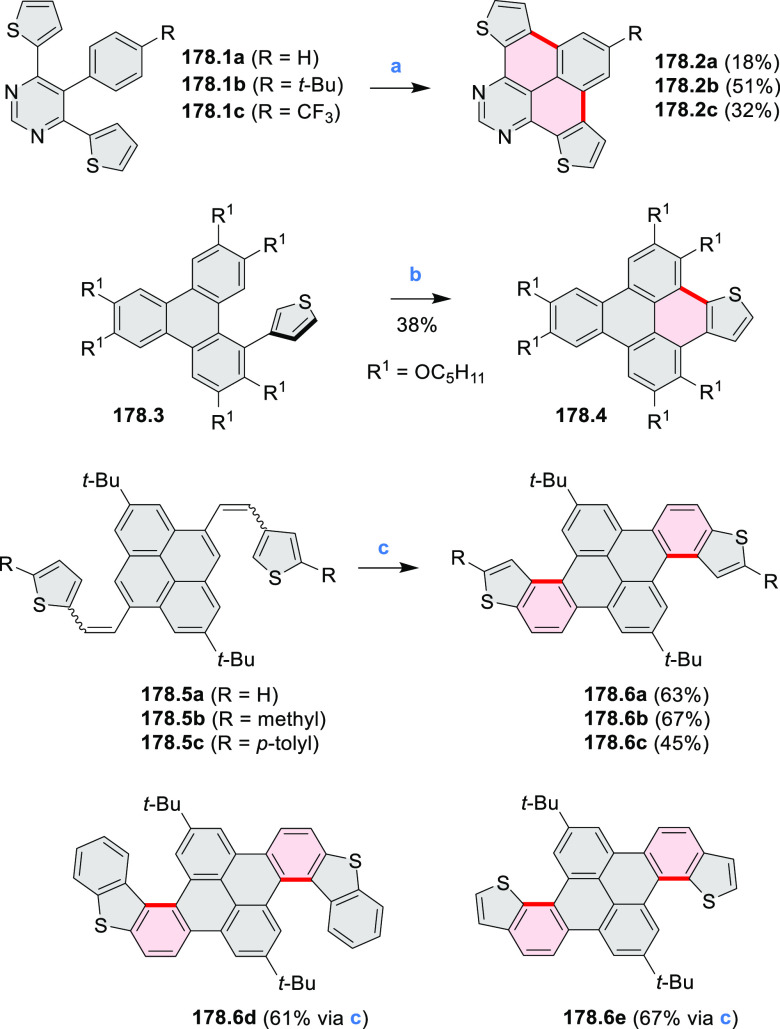
Synthesis of Thiophene- and Benzothiophene-Fused [*e*]-Pyrene Reagents and conditions: (a)^353^ I_2_, MeCN, UV light irradiation, rt, 130 h (**178.1a**), 30 h (**178.1b**), 150 h (**178.1c**); (b)^354^ iron(III) chloride, nitromethane, 0 °C, 1.5 h; (c)^355,356^ I_2_, benzene, *hν.*

Several thieno-fused pyrenoids were obtained via cyclization of thienyl-containing substituents. Verbitskiy et al. used oxidative photocyclization to obtain **178.2a**–**c** in moderate yields (Scheme 178).^353^ The reactivity of precursors depended on substitution (R, *t*-Bu < H < CF_3_), correlating with the electron-withdrawing character of the R group. Compounds **178.2a**–**c** were somewhat more fluorescent (Φ = 0.1–0.13) than their pyrimidine parents **178.1a**–**c** (Φ = 0.05–0.07). Further examples include the mesomorphic fused triphenylene **178.4** obtained by FeCl_3_-mediated cyclodehydrogenation^354^ and bishelicenes **178.6a**–**e**, prepared using Mallory photocyclization.^355,356^**178.6a**,**b** were investigated as active layers in p-type OFET top contact devices, exhibiting unusual electronic stability.

**Scheme 179:**
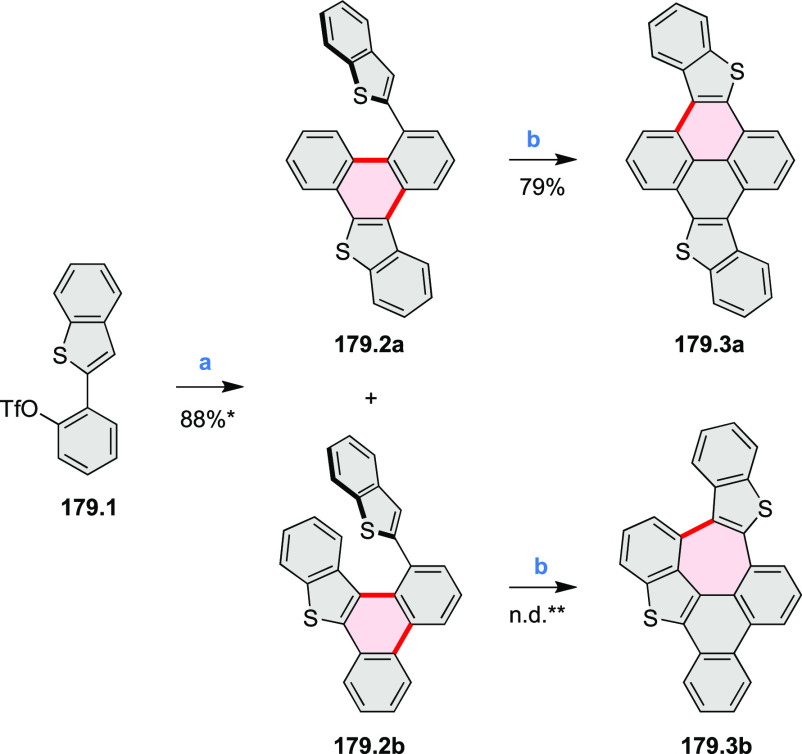
Annulative Dimerization of Phenylene Triflate Reagents and conditions: (a)^357^ PdCl_2_, P(*n*-Bu)_3_·HBF_4_, K_2_CO_3_, pivalic acid, cyclopentyl methyl ether, 140 °C, 20 h; (b) FeCl_3_, DCM, 0 °C, 2.5 h. * Obtained as a mixture of two regioisomers **179.2a** and **179.2b** in 4:1 ratio (overall yield). ** n.d. = not determined. Obtained as a complex mixture.

In 2020, Murakami and Itami reported a synthetic route to thiophene-fused PAHs by a palladium-catalyzed annulative dimerization of phenylene triflate through 2-fold inter- and intramolecular C–H activation.^357^ Optimized conditions utilized palladium chloride, tributylphosphonium tetrafluoroborate, K_2_CO_3_, and pivalic acid in cyclopentyl methyl ether at 140 °C. The benzothiophene-substituted phenylene triflate **179.1** was converted into **179.2a** and **179.2b** in 88% yield as a 4:1 mixture of regioisomers (Scheme 179). The reaction of **179.2b** resulted in a complex mixture containing **179.3b**, whereas the regioisomer **179.2a** reacted smoothly to give the bisbenzothiophene extended pyrene derivative **179.3a** in 79% yield.

**Scheme 180:**
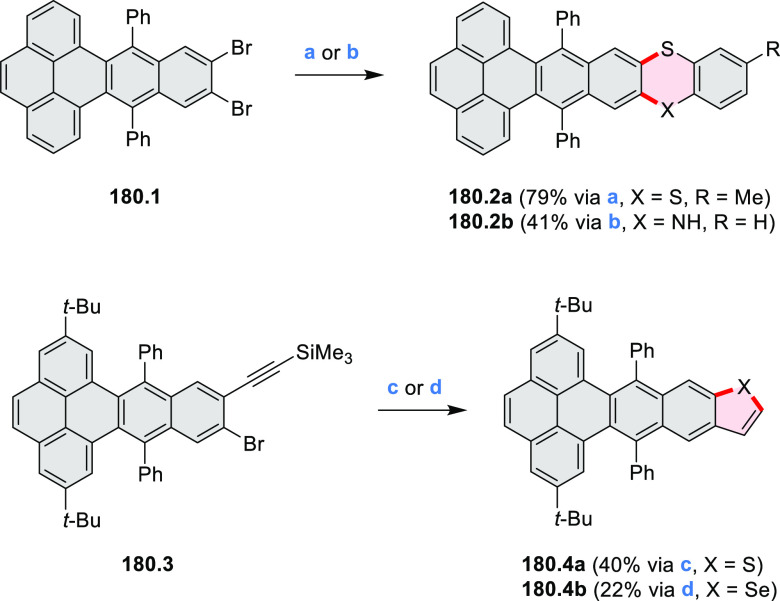
Synthesis of Sulfur-, Selenium-, and Nitrogen-Doped Twistacenes Reagents and conditions: (a)^349^ toluene-3,4-dithiol, K_2_CO_3_, DMF, 100 °C, 1 h; (b) 2-aminobenzenethiol, K_2_CO_3_, DMF, 130 °C, 1 h; (c)^358^ Na_2_S·9H_2_O, NMP, 195 °C, overnight; (d) (1) Se, sodium borohydride, EtOH, 0 °C, 40 min, (2) **180.3** in NMP, then 190 °C, overnight.

π-Extension of twistacenes to include sulfur-containing rings was achieved using methods described in preceding sections. Specifically, the S*-* and N*-*doped **180.2a** and **180.2b** were obtained by nucleophilic cyclization of the dibromo building block **180.1** (Scheme 180).^349^**180.2a** had the expected twisted structure in the solid state, with an additional bend at the 1,4-dithiine ring. **180.2b** deposited on single-wall carbon nanotubes produced a steady photocurrent upon irradiation of a halogen lamp. Terminal thiophene and selenophene rings in **180.4a**,**b** were synthesized through cyclization of the alkyne precursor **180.3** with Na_2_S·9H_2_O or selenium and sodium borohydride, respectively.^358^**180.4a** and **180.4b** emitted blue fluorescence with quantum yields of 0.39 and 0.04, respectively, and self-assembled into nanoparticles upon reprecipitation from a mixture of THF and water.

**Scheme 181:**
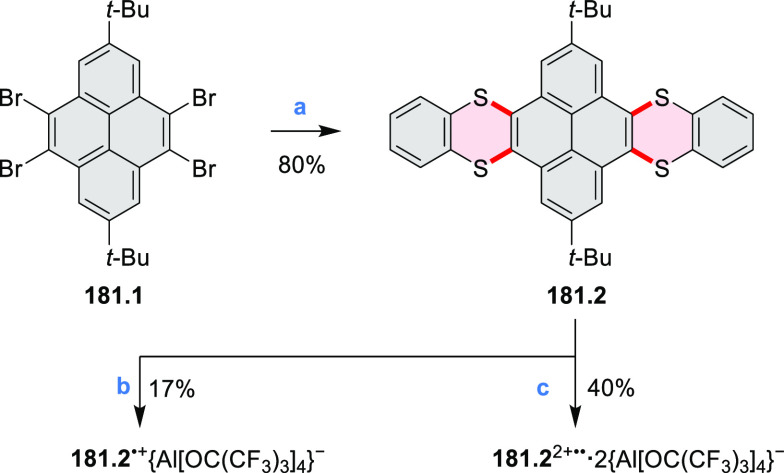
S*-*Doped Hexacene via Nucleophilic Substitution Reagents and conditions: (a)^359^ 1,2-benzenedithiol, K_2_CO_3_, DMF, reflux, 12 h; (b) NO{Al[OC(CF_3_)_3_]_4_} (1 equiv), toluene, rt, 12 h; (c) NO{Al[OC(CF_3_)_3_]_4_} (2 equiv), DCM, rt, 12 h.

Condensation of 2,7-di-*tert*-butyl-4,5,9,10-tetrabromopyrene **181.1** with 1,2-benzenedithiol in the presence of potassium carbonate was used by Wang et al. to prepare the S*-*doped hexacene **181.2** (Scheme 181).^359^ Chemical oxidation of **181.2** was carried out with NO{Al[OC(CF_3_)_3_]_4_} at different molar ratios, affording the yellow-green radical cation salt **181.2**^•+^{Al[OC(CF_3_)_3_]_4_}^−^ and green diradical dication salt **181.2**^2+••^·2{Al[OC(CF_3_)_3_]_4_}^−^, respectively. Both salts were isolated as crystals that were thermally stable under anaerobic conditions at rt. The spin density in **181.2**^•+^ is delocalized on one sulfur-doped ring and the central pyrene unit, while that in **181.2**^2+••^ is delocalized on the whole molecule. The diradical dication **181.2**^2+••^ featured a robust triplet ground state, representing the first example of a high-spin sulfur-containing hydrocarbon diradical (with no nitrogen atoms incorporated).

## 5 Phenalenoids

**Scheme 182:**
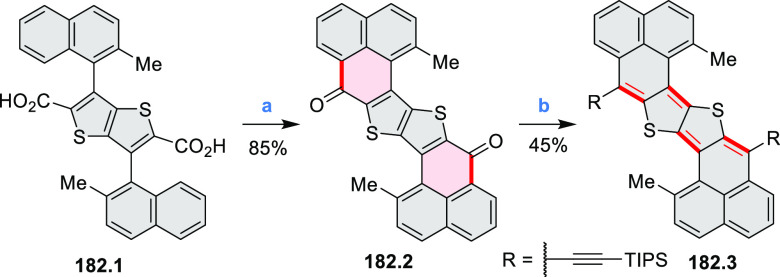
Synthetic Route of Bisphenaleno-thieno[3,2-*b*]thiophene Reagents and conditions: (a)^360^ (1) SOCl_2_, dry DCM, reflux, (2) AlCl_3_, dry DCM, 0 °C to rt, overnight; (b) (1) (triisopropylsilyl)ethynyllithium, THF, 0 °C to rt, (2) SnCl_2_, toluene, rt, 12 h.

Most systems discussed in this section contain at least one heteroatom incorporated into the phenalene substructure (see CR2017, Section 5 for scope definition and earlier examples). A unique example of an extended phenalene containing an *ortho*-fused heterocycle is the pro-aromatic bisphenaleno-thieno[3,2-*b*]thiophene (**182.3**) reported by Kim and Chi et al. in 2015 (Scheme 182).^360^ It was synthesized from diacid **182.1**, which was converted into the corresponding acyl chloride and subjected to double Friedel–Crafts acylation, to afford the desired diketone **182.2** in 85% yield. Addition of triisopropylsilyl ethynyl lithium to **182.2**, followed by SnCl_2_ reduction, gave the target **182.3** in 45% yield. Transient absorption spectra of **182.3** exhibited a ground-state bleach signal at around 690 nm and a weak excited-state absorption band in the 450–600 nm region. **182.3** displayed amphoteric redox behavior with two reversible oxidation waves (*E*_1/2_^ox^ = −0.03, +0.44 V vs Fc^+^/Fc) and two reversible reduction waves (*E*_1/2_^red^ = −1.73, −1.38 V vs Fc^+^/Fc).

### 5.1 Monoheteraphenalenes

#### 5.1.1 Azaphenalenes

**Scheme 183:**
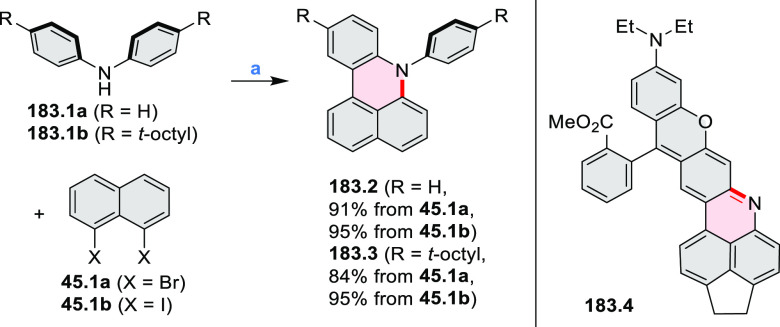
Syntheses of Azabenzophenalenoids Reagents and conditions: (a)^76^*t*-BuONa, Pd(OAc)_2_, Pd_2_(dba)_3_, Cy_3_P, (*t*-Bu)_3_P, toluene, 90 °C, 10 h.

The 1-azaphenalene motif, which also appears in some larger fused frameworks (cf. Schemes 45, Section 3.1.2, and 294, Section 6.4.2), can be assembled in several ways, e.g., using the previously discussed tandem cross-coupling annulation reported by Li et al. (**183.2**, Scheme 183).^76^ In this synthesis, Buchwald–Hartwig amination was presumed to precede the arylation step. An alternative approach, employing nucleophilic aromatic substitution in the ring-forming step, was used by Zeng et al. to prepare the NIR fluorescent rhodamine dye **183.4**.^361^**183.4** showed a near-infrared emission at 830 nm, significantly red-shifted in comparison with conventional rhodamine dyes.

**Scheme 184:**
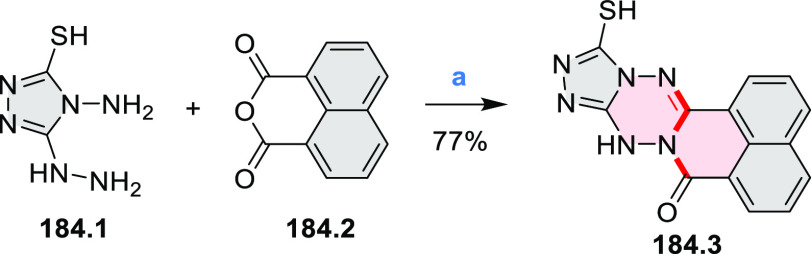
Reactivity of 4-Amino-5-hydrazineyl-4*h*-1,2,4-triazole-3-thiol Reagents and conditions: (a)^362^ glacial acetic acid, reflux, 4 h.

The reactivity of 4-amino-5-hydrazineyl-4*h*-1,2,4-triazole-3-thiol **184.1** toward cyclic anhydrides was studied by El-Shaieb et al. in 2019 (Scheme 184).^362^ Depending on the anhydride, several types of heterocyclic structures were obtained, including cyclic hydrazides and triazoloterazine derivatives. The most complex ring system, the pentacyclic **184.3**, was obtained in the reaction with 1,8-naphthalic anhydride **184.2**.

**Scheme 185:**
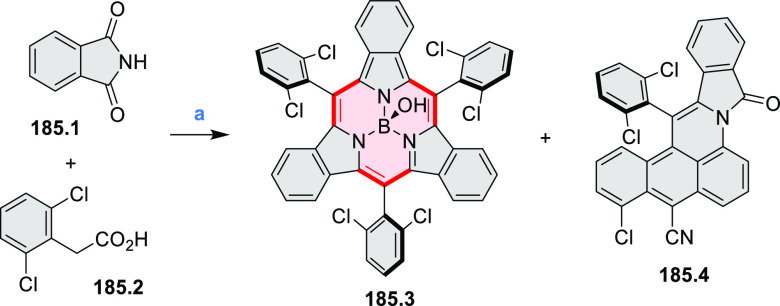
Byproduct Formation in the Synthesis of a Tribenzosubporphyrin Reagents and conditions: (a)^363^ B(OH)_3_, 360 °C.

An unexpected formation of the blue azaphenalenoid **185.4** was observed by Furuta and Shimizu et al. during the synthesis of *meso*-2,6-dichlorophenyl-substituted tribenzosubporphyrin (**185.3**) in a reaction of phthalimide (**185.1**) and 2,6-dichlorophenylacetic acid (**185.2**, Scheme 185).^363^ No similar product was isolated in the reaction using phenylacetic acid, indicating potential relevance of the chloro substituents in **185.2**. The mechanism of this unusual transformation remains to be elucidated.

**Scheme 186:**
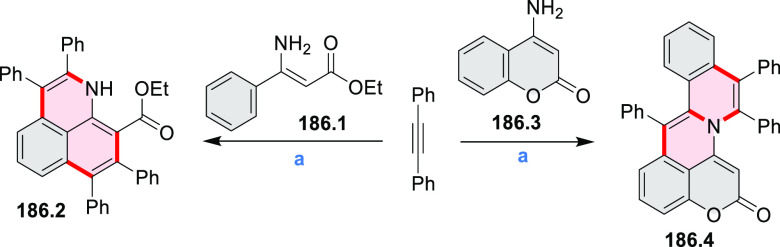
Synthesis of Nitrogen-Containing Phenalenoids via Rhodium-Catalyzed Multiple C–H Bond Activation and Oxidative Annulation Reagents and conditions: (a)^364^ [Cp*RhCl_2_]_2_, Cu(OAc)_2_, DMF, 100 °C, Ar, 24 h.

Several nitrogen-containing phenalenoids were obtained using a rhodium-catalyzed oxidative annulation of β-enamino esters with internal alkynes, described by Sun et al. (Scheme 186).^364^ For instance, diphenylacetylene and the unprotected β-enamino ester **186.1** could be reacted in the presence of [Cp*RhCl_2_]_2_ and Cu(OAc)_2_ to afford **186.2** in 73% yield. When 4-aminocoumarins were used as substrates in the same reaction, extended systems, such as **186.4**, were formed. This double annulation was shown to work with a variety of alkynes, β-enamino esters, and 4-aminocoumarins.

**Scheme 187:**
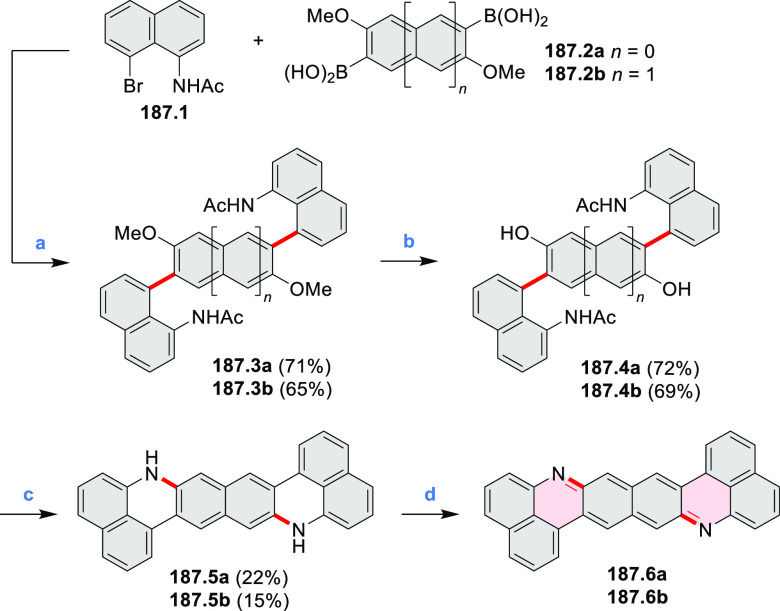
N-Doped Heptazethrene and Octazethrene Reagents and conditions: (a)^365^ Pd(PPh_3_)_4_, 2 M Na_2_CO_3_, 1,2-dimethoxyethane, 115 °C, 48 h; (b) BBr_3_, dry DCM, rt, 48 h; (c) hydrazine monohydrate (80%), DMSO/*i*-PrOH, 140 °C, 72 h; (d) PbO_2_, dry DCM, rt, 30 min.

Assembly of 1-azaphenalene fragments was the key step in the synthesis of N*-*doped heptazethrene and octazethrene diradicaloids reported by Wu and co-workers in 2019 (Scheme 187).^365^ Suzuki coupling between 1-acetamido-8-bromonaphthalene (**187.1**) and diboronic acids **187.2a**,**b** gave the corresponding intermediates **187.3a**,**b**. Demethylation of **187.3a**,**b** with boron tribromide afforded diols **187.4a**,**b**, which were further subjected to hydrazine-mediated deprotection and cyclization to give precursors **187.5a**,**b**. Chemical oxidation of **187.5a**,**b** by lead(IV) oxide in dry DCM gave the respective dehydrogenated products **187.6a**,**b**, which were, however, too unstable to be isolated. **187.6a** exhibited a major absorption band with maximum (λ_max_) at 598 nm and a weak shoulder peak at 660 nm. **187.6b** displayed a similar band structure to **187.6a** with λ_max_ = 644 nm together with a shoulder peak at 728 nm.

**Scheme 188:**
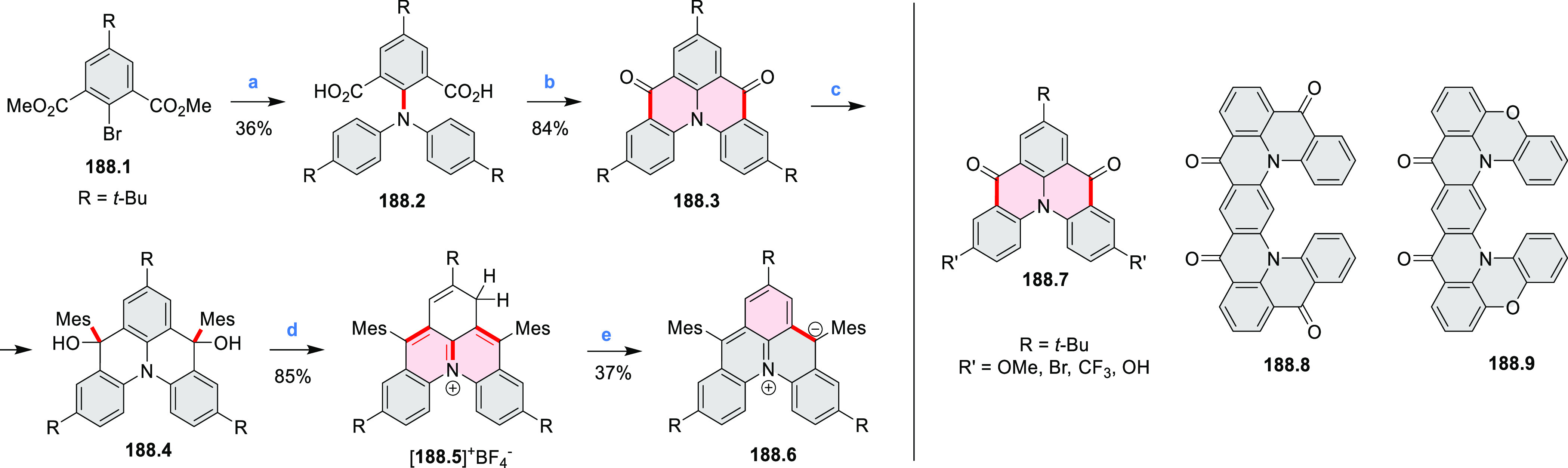
Polycyclic Heterahelicenes Reagents and conditions: (a)^366^ (1) bis(4-*tert-*butylphenyl)amine, Cu, 18-crown-6, K_2_CO_3_, 200 °C, 24 h, (2) NaOH, EtOH, 100 °C, 9 h; (b) (1) (COCl)_2_, DMF, DCM, 45 °C, 1 h, (2) SnCl_4_, 45 °C, 12 h; (c) MesLi, THF, −15 °C to rt, 18 h; (d) Et_3_SiH, HBF_4_·Et_2_O, DCM, rt; (e) NaH, THF/hexane, rt, 4 h.

In 2019, Arikawa, Shimizu, and Shintani et al. reported the synthesis of a polycyclic zwitterion azoniadibenzo[*a*,*j*]phenalenide **188.6**.^366^ The synthesis of **188.6** was accomplished in six steps from 2-bromo-5-(*tert-*butyl)-1,3-bis(methoxycarbonyl)benzene **188.1** (Scheme 188). The amination of **188.1** with bis(4-*tert-*butylphenyl)amine gave **188.2**, and hydrolysis of its methyl esters followed by 2-fold intramolecular Friedel–Crafts acylation resulted in **188.3**. Reaction of **188.3** with mesityllithium gave the diol **188.4**, which was then reduced by Et_3_SiH/HBF_4_·Et_2_O to give the cation **188.5**. Deprotonation of **188.5** using NaH in THF/hexane in a degassed sealed tube gave **188.6**, which was isolated by recrystallization under an inert atmosphere. **188.6** is stable at ambient temperature in a degassed solution or in the solid state under argon. The negative charge of **188.6** delocalized over the periphery of the main core, and a positive charge localized near the nitrogen center. **188.6** has an open-shell singlet ground state, which was demonstrated using NMR, ESR studies, and DFT calculations.

Methoxy-, bromo-, CF_3_-, and hydroxy-substituted bridged triarylamine helicenes (**188.7**), analogous to **188.3**, were synthesized by Shang and Yamamoto et al.^367^ Triarylamine helicenes **188.7** were synthesized from **188.1** in a similar to **188.3** manner (Scheme 188). The final cyclizations were carried out in neat sulfuric acid under ambient conditions, providing the products in 65–98% yield. Among the four derivatives, methoxy **188.7** showed the highest photoluminescence quantum yield (38%) and longest lifetime (13.98 ns), while the hydroxy-substituted **188.7** showed solvent- and pH-dependent luminescence in alkaline conditions. All four derivatives had low oxidation potentials ranging from −1.37 to −1.00 V.

*cis*-Quinacridone derivatives structurally related to **188.7** were reported as delayed fluorescence luminogens by Yasuda et al. in 2021.^368^**188.8**–**9** were synthesized in two or three steps, starting with diethyl 4,6-dibromoisophthalate and Buchwald–Hartwig amination. The resulting arylamine-functionalized isophthalates were then subjected to intramolecular Friedel–Crafts acylation. Deep-blue OLEDs based on **188.8** achieved a high maximum external electroluminescence (EL) quantum efficiency η_ext_ of 19.0% with a narrow fwhm of 37 nm.

**Scheme 189:**
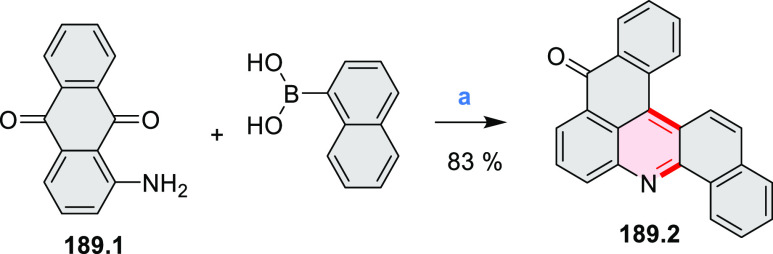
Synthesis of a Ceramidonine via a Copper Trifluoroacetate-Mediated Relay Reaction Reagents and conditions: (a)^369^ Cu(OTf)_2_, 1,1,2,2-tetrachloroethane (TCE), 100 °C, 60 h.

A synthesis of acridines from *o*-aminoaryl ketones and arylboronic acids was developed by Zhang and Zhang et al., who used copper(II)-mediated relay reactions that involved intermolecular Chan–Lam cross-coupling and subsequent intramolecular cyclization.^369^ In particular, the fused ceramidonine **189.2** was synthesized from 1-aminoanthracene-9,10-dione **189.1** and 1-naphthylboronic with Cu(OTf)_2_ (1.2 equiv) as the activator (Scheme 189).

**Scheme 190:**
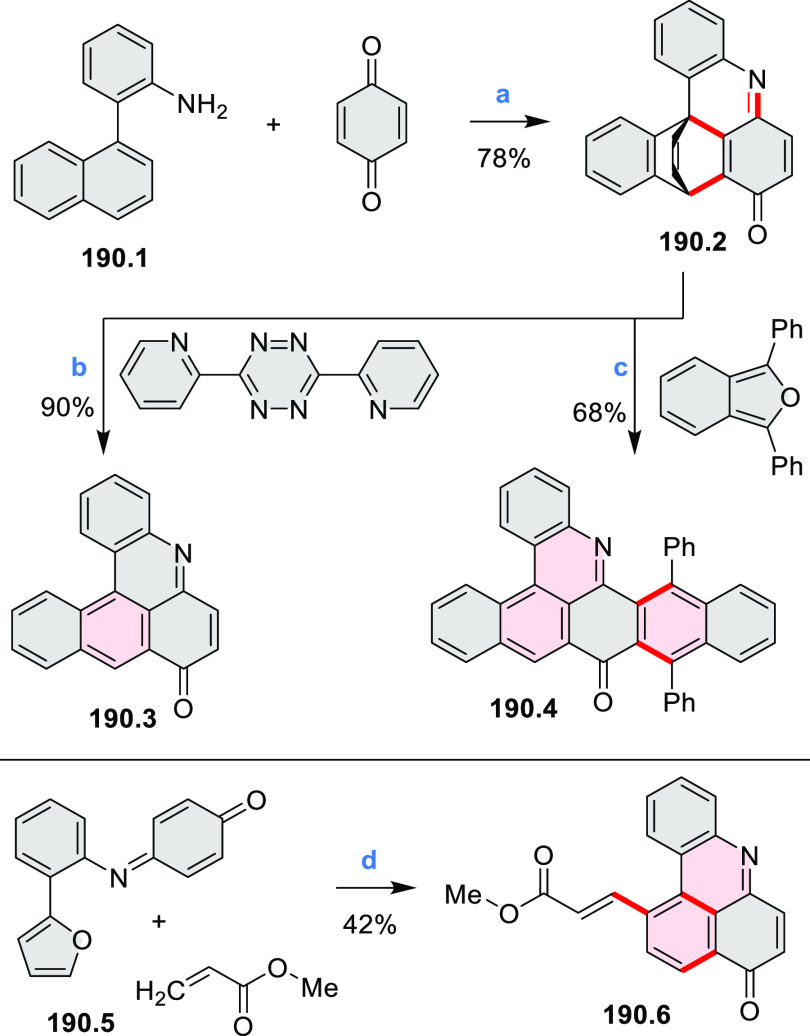
Synthesis of Planar Acridinone Heterocyclics Reagents and conditions: (a)^370^ Cu(OAc)_2_ (2 equiv), AcOH (1.8 equiv), *o*-DCB, 140 °C, under air, 24 h; (b) DCM, 80 °C, reflux, overnight; (c) (1) toluene, 180 °C, 16 h, (2) *p*-TsOH, 8 h, 180 °C; (d) 10 mol % of Pd(AcO)_2_, Cu(AcO)_2_, O_2_, TFE, 120 °C, 24 h.

A transformation of iptycene **190.2** into planar acridinones **190.3**–**4** was reported by Chuang et al. in 2017 (Scheme 190).^370^ The starting **190.2** was obtained in a reaction between **190.1** and *p*-benzoquinone in the presence of Cu(OAc)_2_ as the oxidant. The inverse electron-demand Diels–Alder reaction between 3,6-di(pyridin-2-yl)-1,2,4,5-tetrazine and **190.2** yielded the fully planarized compound **190.3**. The cycloaddition of **190.2** and the electron-rich 1,3-diphenylisobenzofuran occurred at the outer double bond and, after subsequent acid-catalyzed aromatization, produced the π-extended product **190.4**. A competition experiment between methyl acrylate and the iminoquinone moiety in **190.5** carried out in the presence of 10 mol % of Pd(OAc)_2_ and Cu(OAc)_2_/O_2_ as the catalyst and the oxidant, respectively, resulted in *ortho* C–H activation and insertion of methyl acrylate at position 3 of the furan moiety, followed by [4 + 2] cycloaddition of the iminoquinone, to yield **190.6**.

**Scheme 191:**
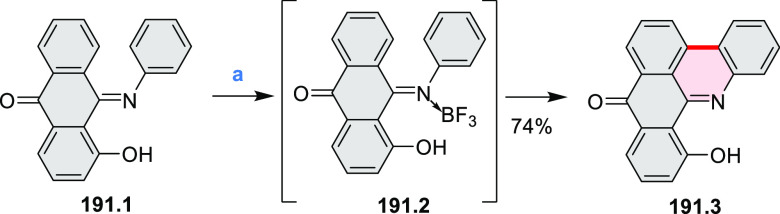
π-Extended Acridones Reagents and conditions: (a)^371^ BF_3_·Et_2_O, *hν*, rt, 1 h.

As part of their research on anthraquinone imine dyes for DSSCs, Sundermeyer and co-workers reported in 2016 a four-step synthesis of benzoacridinone **191.3** (Scheme 191).^371^ The requisite precursor **191.1** had to be obtained via a monoacetal intermediate, to avoid selectivity problems. **191.1** was then photolyzed in a Mallory-type reaction, yielding **191.3**. In the latter reaction boron trifluoride was used as a Lewis-acid activator to promote the 6π electrocyclization of the imide.

#### 5.1.2 Boraphenalenes

**Scheme 192:**
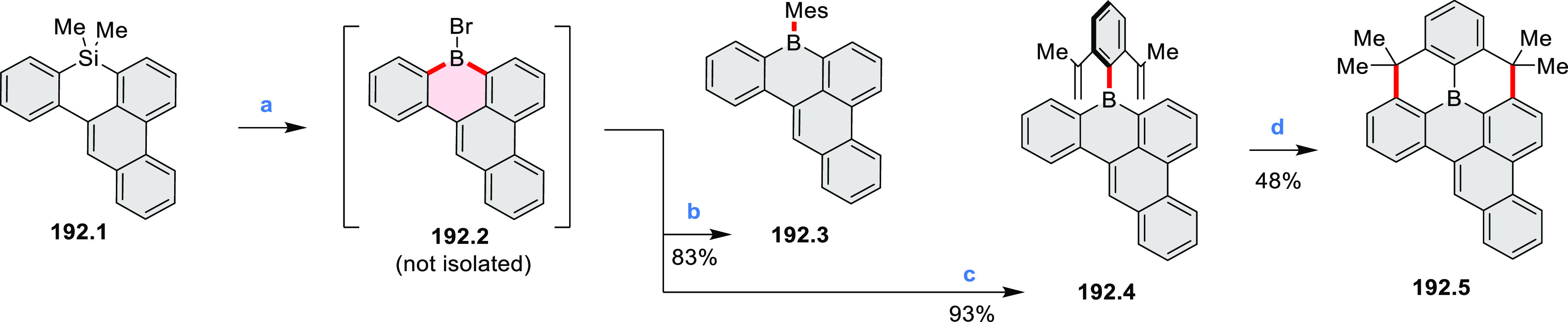
Synthetic Routes to 8-Borabenzo[*gh*]tetraphenes Reagents and conditions: (a)^372^ excess neat BBr_3_, 25 °C; (b) 1.5 equiv of MesLi, toluene/THF, 0 °C; (c) 1.1 equiv of [2,6-bis(propen-2-yl)phenyl]lithium, toluene/THF, 25 °C; (d) 1 equiv of Sc(OTf)_3_, 1,2-DCE, reflux temperature.

A route to 8-borabenzo[*gh*]tetraphenes was developed by Yamaguchi and Wagner et al. (Scheme 192).^372^ Compound **192.1** undergoes smooth Si/B exchange with BBr_3_ at rt to afford the highly reactive bromoborane **192.2**. This intermediate was not isolated but immediately converted into the stable triarylboranes **192.3** and **192.4** by addition of an appropriate aryllithium reagent. An intramolecular, Sc(OTf)_3_-mediated Friedel–Crafts cyclization carried out on **192.4** gave the planarized boron-doped PAH **192.5**. **192.3** and **192.5** had similar electronic properties; in particular, both were strongly fluorescent, with quantum yields of 85% and 89%, respectively.

**Scheme 193:**
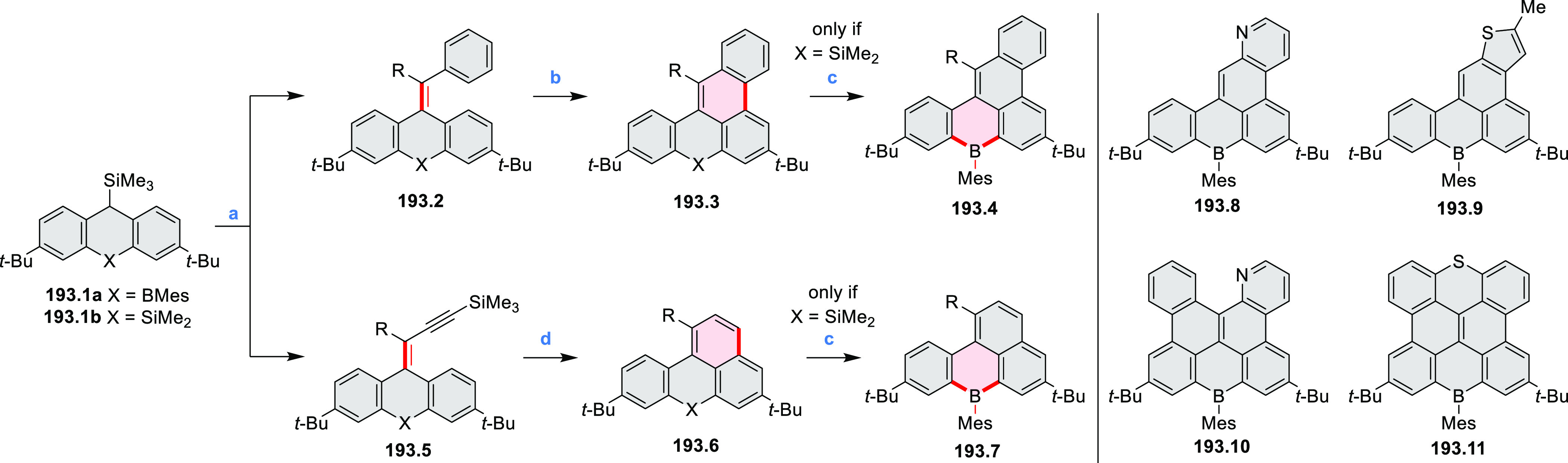
Synthetic Routes to Boron-, Nitrogen-, and Sulfur-Doped PAHs Reagents and conditions: (a)^373^*n*-BuLi, Et_2_O; (b) UV irradiation (Hg medium pressure lamp), I_2_, propylene oxide, cyclohexane; (c) (1) excess neat BBr_3_, (2) MesMgBr, toluene/THF; (d) (1) [*n*-Bu_4_N]F, THF, (2) (PPh_3_)Ru(cymene)Cl_2_, NH_4_PF_6_, (CH_2_Cl)_2_.

A synthetic approach to B*-*doped PAHs containing additional N and S centers was described by Wagner’s group in 2016 (Scheme 193).^373^ The strategy generally relied on the tricyclic starting material **193.1a** to circumvent destructive side reactions between BBr_3_ and the Lewis basic heteroatoms during the Si/B exchange. For similar reasons, the stilbene-type photocyclization was performed to set up the polycyclic framework and omitted the Ru-catalyzed enyne benzannulation approach. Using **193.1a** and pyridine-2-aldehyde, the preparation of the monopyridine derivative **193.8** was achieved in 2 steps and 35% yield. Similarly, the use of phenyl(pyridin-2-yl)methanone yielded the extended congener **193.10**. Photocyclization of a thienyl substituent was also possible, with the formation of PAH **193.9**, while pyrrole- and furan-containing precursors proved to be inert under UV–vis irradiation. Thioxanthen-9-one was sufficiently electrophilic toward the lithiated **193.1a** to furnish the Peterson olefination product, which could be photocyclized to give **193.11**. A similar synthetic approach to form **193.7** through the Ru-catalyzed cyclization of aryl enynes was also described by the Wagner group.^278^

**Scheme 194:**
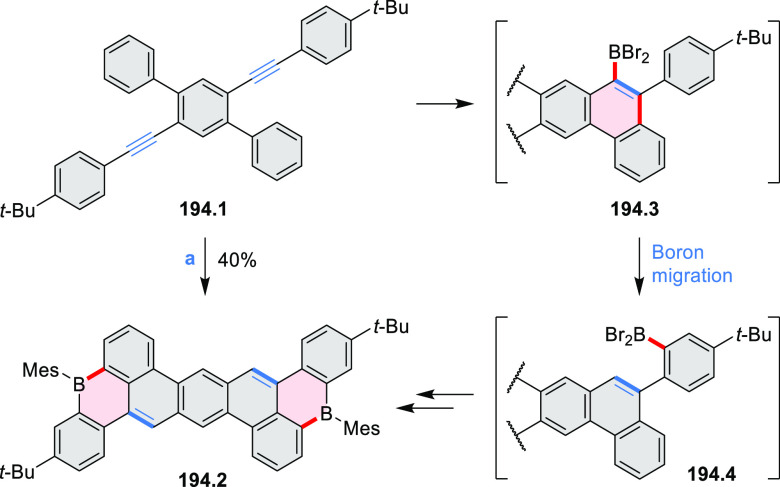
One-Pot Synthetic Routes to B-Doped PAHs Reagents and conditions: (a)^374^ (1) BBr_3_, TBP, TCB, 200 °C, 24 h, (2) MesMgBr, rt, 1 h.

Liu and Feng et al. reported a one-pot synthesis of B*-*doped PAHs from branched aryl-substituted alkynes (Scheme 194).^374^ In particular, **194.2** was obtained by heating **194.1** with boron tribromide (BBr_3_) and 2,4,6-tri(*tert*-butyl)pyridine and subsequent reaction with mesitylmagnesium bromide at rt. It was found that the bulky Brønsted base played an important role in the formation of **194.2**. In the proposed mechanism, the intermediate **194.3** was formed via a 6-*endo-dig* borylative cyclization of alkyne **194.1** in the presence of BBr_3_. Subsequently, **194.3** underwent 1,4-boron migration to afford intermediate **194.4**, followed by an electrophilic borylation. The resulting six-membered boracycle intermediate was then converted into the final **194.2** after workup with MesMgBr.

#### 5.1.3 Oxa- and Thiaphenalenes

**Scheme 195:**
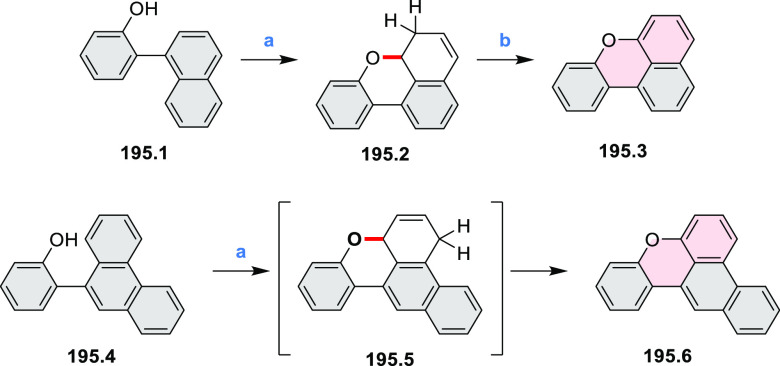
Photocyclization Reactions of Arylphenols Reagents and conditions: (a)^375^*hν*, deoxygenated H_2_O/CH_3_CN; (b) Pd/C, O_2_, toluene.

The photochemistry of benzannulated derivatives of 2-phenylphenol was investigated by Lukeman and Wang et al. (Scheme 195).^375^ Preparation of derivatives **195.1** and **195.4** was achieved by Suzuki coupling of an appropriate methoxyaryl boronic acid and aryl bromide, followed by demethylation with BBr_3_. Under dioxygen-free conditions, **195.1** was found to undergo photocyclization to dihydrobenzoxanthene **195.2**. Treatment of the latter intermediate with carbon-supported palladium in oxygenated toluene resulted in an over 90% conversion into benzo[*k*,*l*]xanthene **195.3**. Irradiation of **195.4** gave rise to a single photoproduct, which was identified as naphtho[1,2,3-*kl*]xanthene **195.6**. The primary photocyclization product **195.5** could be identified as an intermediate using NMR spectroscopy.

**Scheme 196:**
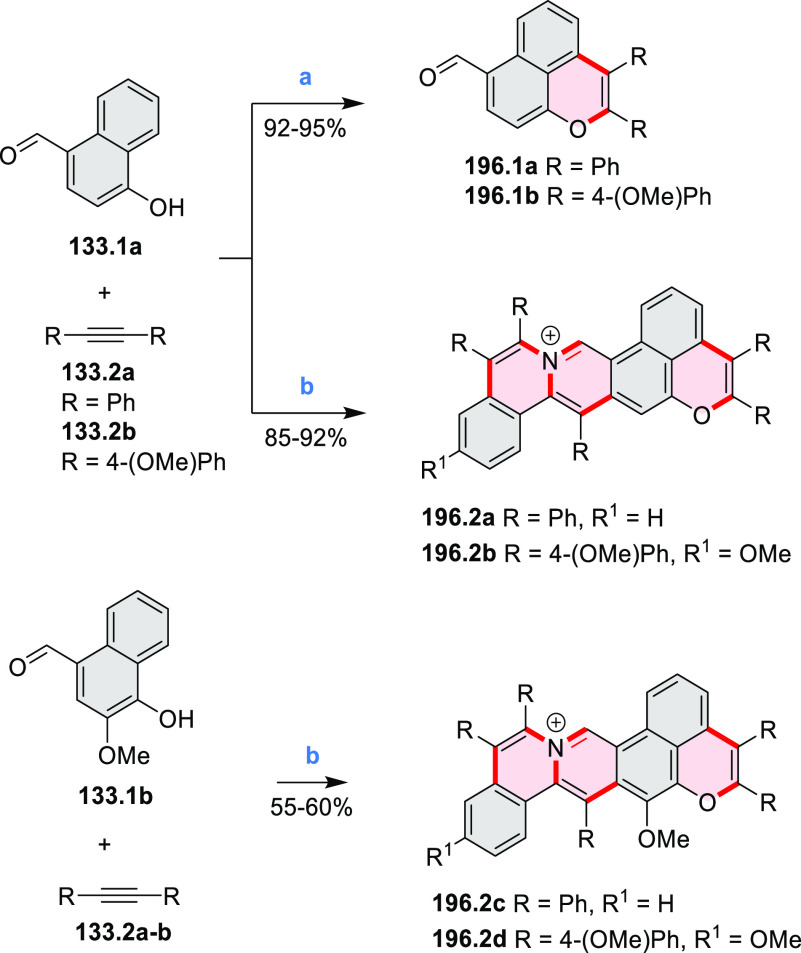
Synthesis of Pyridinium-Fused Oxaphenalenes Reagents and conditions: (a)^268^ [Cp*RhCl_2_]_2_, Cu(OAc)_2_, THF, 100 °C, 12 h; (b) [Cp*RhCl_2_]_2_, Cu(OAc)_2_, NH_4_BF_4_, THF, 150 °C, 24 h.

Pyridinium-fused oxaphenalenes **196.2a**–**d** were obtained from 4-formyl-1-naphthols **133.1a**,**b** and alkynes **133.2a**,**b** in a rhodium-catalyzed triple cyclization (Scheme 196).^268^ As the pyridinium nitrogen atoms in these products were derived from the NH_4_BF_4_ additive, pyrylium-containing pyrenoid products **133.3a**–**j** were obtained when NaBF_4_ was used instead (Scheme 133, Section 4.3). Additionally, omitting the additive and using **133.1a** and alkyne in a 1:1 stoichiometry provided products of a single cyclization **196.1a**,**b** in high yields. These oxaphenalenes were thought to be intermediates in the triple cyclizations toward **196.2a**–**d** as well as **133.3a**–**j** (see also Scheme 133, Section 4.3 for a discussion of photophysical properties of these products).

**Scheme 197:**
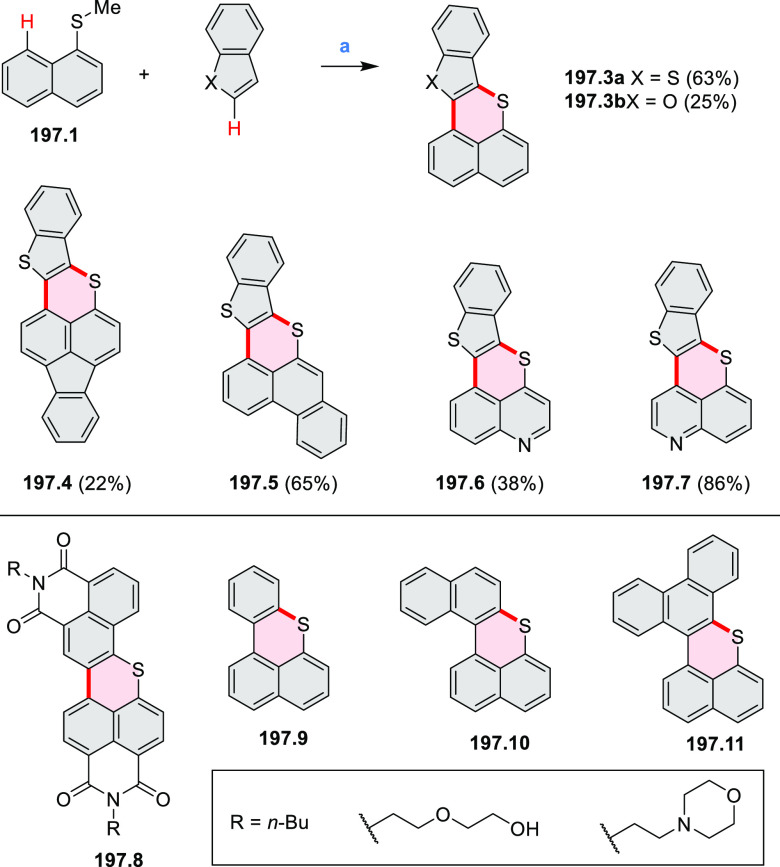
π-Extended Thiaphenalenoids Reagents and conditions: (a)^376^ [Cp*RhCl_2_]_2_ (5 mol %), AgSbF_6_ (20 mol %), 3 equiv of Ag_2_O, 1 equiv of PivOH, HFIP, 100 °C, N_2_ atmosphere, 24 h.

A one-step synthesis of benzo[*de*]thioacenes via a Rh-catalyzed *peri*-selective heteroarylation/Ag-mediated SET intramolecular cyclization sequence of 1-(methylthio)naphthalene **197.1** and its analogues was developed by You et al. in 2019 (Scheme 197).^376^ Specifically, **197.1** underwent one-shot Rh-catalyzed annulation with either benzofuran or benzothiophene in the presence of [Cp*RhCl_2_]_2_, AgSbF_6_, Ag_2_O, PivOH, and HFIP as a solvent. The reaction is applicable to the synthesis of benzo[*de*]thioacenes and showed that the benzothiophenes and naphthalenes with a variety of functional groups, as well as other carbo- and heterocyclic precursors, yielded products such as **197.4**–**7**. Related fused benzothioxanthenes were obtained by oxidative cyclization of ammonium salts (**197.8**)^377^ and via acid-mediated cyclization of sulfoxides (**197.9**–**11**, cf. Scheme 138, Section 4.3).^277^ Variously substituted **197.8** derivatives displayed large Stokes shifts and efficient singlet oxygen generation in the absence of heavy atoms in their structures (quantum yields of up to 0.82).

**Scheme 198:**
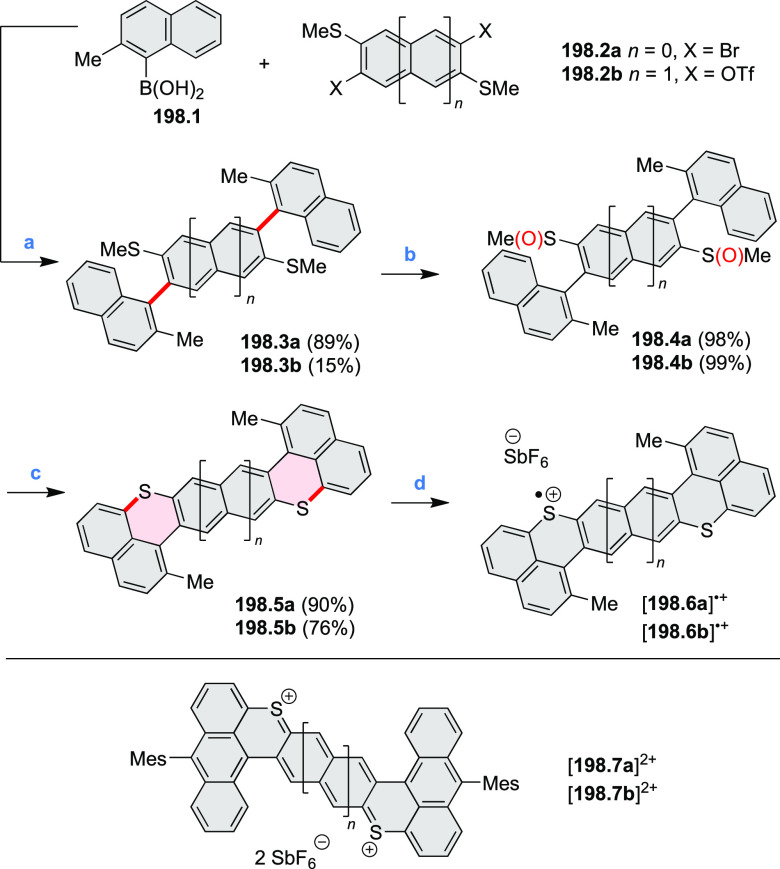
Sulfur-Doped Dibenzohepta- and Dibenzooctazethrene Reagents and conditions: (a)^378^ Pd_2_(dba)_3_, Na_2_CO_3_, SPhos, toluene/ethanol/H_2_O (10:1:1), 110 °C; (b) 30% H_2_O_2_, acetic acid/chloroform (1:1), rt; (c) (1) Eaton’s reagent, rt, (2) pyridine/H_2_O, reflux; (d) NO_2_SbF_6_, DCM/MeCN, rt.

The synthesis and characterization of sulfur-doped dibenzohepta- and dibenzooctazethrene (Scheme 198) were reported by Zhang and Wu et al. in 2020.^378^ The intermediates **198.3a**,**b** were first prepared via Suzuki coupling between 2-methylnaphthaleneboronic acid **198.1** and **198.2a**,**b** (Scheme 198). Oxidation of **198.3a**,**b** with hydrogen peroxide afforded sulfoxides **198.4a**,**b**. The two-step sequential intramolecular cyclization of **198.4a**,**b** in the presence of Eaton’s reagent followed by refluxing in pyridine gave **198.5a**,**b**. Chemical oxidation of the neutral **198.5a**,**b** with NO_2_SbF_6_ produced radical cations **198.6a**,**b**^•+^, while the oxidation of neutral **198.7a**,**b** with either 1 and 2 equiv of NOSbF_6_ gave the corresponding radical cations **198.7a**,**b**^•+^ and dication **198.7a**,**b**^2+^, respectively.

### 5.2 Diheteraphenalenes

#### 5.2.1 Pyridoacridines and Other 1,6-Diheteraphenalenoids

**Scheme 199:**
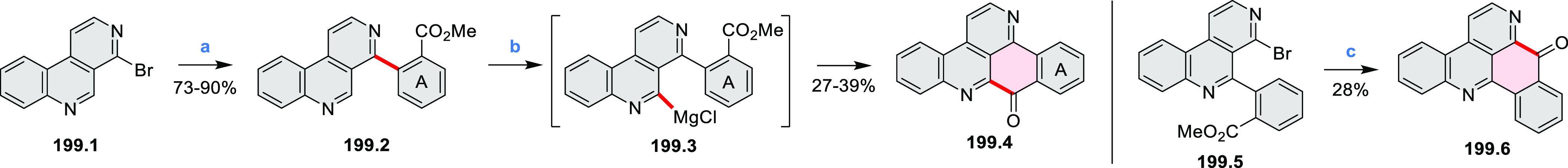
Synthesis of Ascididemin-Type Analogues (Deazaascididemin) Reagents and conditions: (a)^379^ corresponding boronic acid, KF, Pd_2_(dba)_3_, (*t*-Bu)_3_P, THF, 80 °C, 30 min; (b) TMPMgCl·LiCl, THF, 2 h at 0 °C, then 16 h, rt; (c)^380^*i*-PrMgCl·LiCl, THF, 0 °C, 2 h.

The azaoxoaporphine alkaloid deazaascididemin **199.4** as well as various ring-A-modified analogues of ascididemin-type pyridoacridine alkaloids were reported by the Bracher group in 2015 (Scheme 199).^379^ Their approach involved a Suzuki or Negishi cross-coupling reaction, followed by directed remote ring metalation and intramolecular trapping of the adjacent ester group. The protocol made it possible to introduce structural variation at a critical part of the pentacyclic ring system, which is of special pharmacological interest. The same group later reported a related strategy, which relied on the biaryl **199.5** obtained from 4-bromobenzo[*c*][2,7]naphthyridine via Negishi cross-coupling.^380^**199.5** was converted into the respective organomagnesium product by bromine–magnesium exchange with *i*-PrMgCl·LiCl, followed by intramolecular addition to the ester group which produced the expected pyridoacridone **199.6**, isomeric to **199.4**, in 28% yield.

**Scheme 200:**
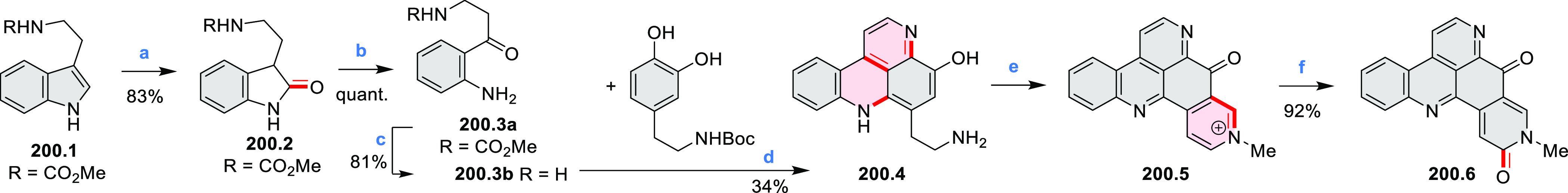
Synthesis of Amphimedine Reagents and conditions: (a)^381^ AcOH, DMSO/HCl (conc.); (b) O_2_, 1 N NaOH; (c) HBr, AcOH, 80 °C; (d) (1) Ag_2_O, CeCl_3_·7H_2_O, MeOH/AcOH (1:1), 40 °C, (2) 6 N HCl, 90 °C; (e) (CH_2_O)_*n*_, AcOH, 60 °C, 5 h; (f) K_3_[Fe(CN)_6_], aq. NaOH, 0 °C.

An improved synthesis of styelsamine D (**200.4**), deoxyamphimedine (**200.5**), and amphimedine (**200.6**) was developed by Copp et al. (Scheme 200).^381^ Coupling of kynuramine **200.3b**, which was obtained using an improved procedure, with *N-*Boc-protected 4-(2-aminoethyl)benzene-1,2-diol using a two-step sequence, afforded styelsamine D **200.4** in 34% yield. Reaction of **200.4** with paraformaldehyde afforded the two pentacyclic natural products deoxyamphimedine (**200.5**) in 48% yield and demethyldeoxyamphimedine in 52% yield. The yield of **200.5** could be improved up to 66% by increasing the amount of added paraformaldehyde. The versatility of the approach was demonstrated by the synthesis of non-natural analogues of **200.6** and **200.5**.

**Scheme 201:**
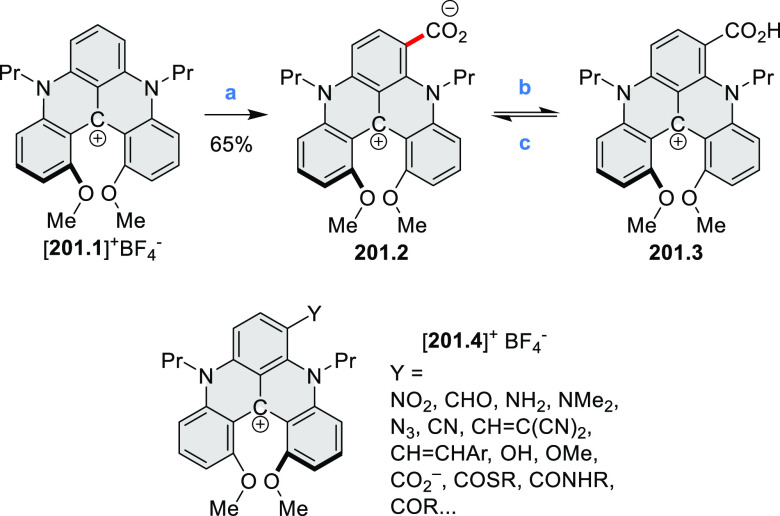
Polycyclic Heterahelicenes Reagents and conditions: (a)^382,383^ (1) DMF, POCl_3_, 90 °C, 8 h, (2) NaH_2_PO_4_, NaClO_2_, H_2_O_2_, MeCN, 60 °C, 1 h; (b) 1 M NaOH; (c) 1 M HCl.

Regioselective postfunctionalization of racemic and enantiopure cationic diaza[4]helicenes (**201.4**^+^) was described by Lacour et al. in 2016 (Scheme 201).^382,383^ Overall, more than 20 new chromophores were prepared from [**201.1**]^+^BF_4_^–^ in moderate to excellent yields (55–99%). These systems showed tunable redox and optical characteristics dependent on the nature of the Y group, with electron-donating substituents shifting the low-energy absorption band toward the far-red and NIR regions. The water-soluble zwitterionic dye with pH-dependent absorption and emission properties (**201.2**) was obtained via the Vilsmeier–Haack and Pinnick reactions of [**201.1**]^+^BF_4_^–^.^383^ Depending on its protonation state, compound **201.2** showed an efficient and reversible turn-on of electronic circular dichroism at 300 nm and of circularly polarized luminescence in the red domain.

**Scheme 202:**
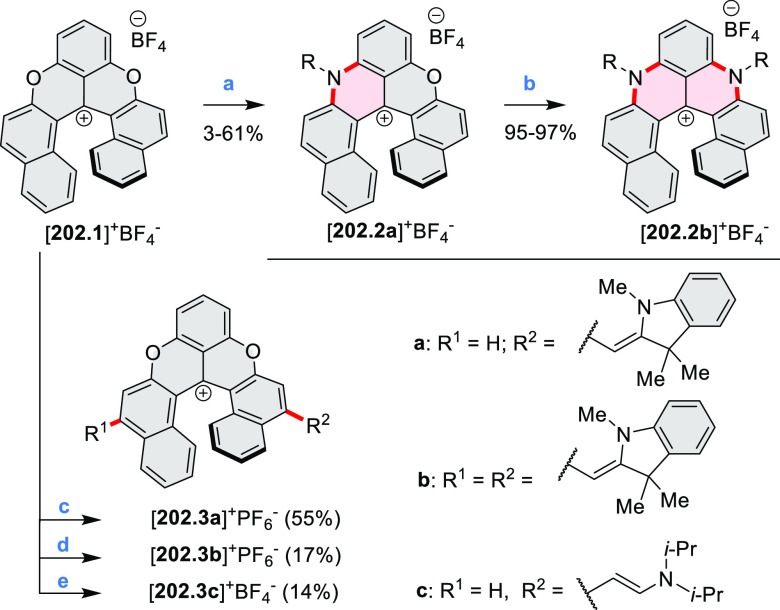
Cationic Helicenes Reagents and conditions: (a)^384^ RNH_2_ (3 equiv), PhCOOH, NMP, 60 °C, 3 h; (b) RNH_2_ (25 equiv), PhCOOH, NMP, 70 °C, 7 h; (c)^385^ 1,3,3-trimethyl-2-methyleneindoline, benzophenone, 80 °C, 21 h; (d) 1,3,3-trimethyl-2-methyleneindoline, (*i*-PrO)_3_Al, benzophenone, 80 °C, 24 h; (e) *i*-Pr_2_NEt (3.0 equiv) at 90 °C, NMP.

Sequential exchange of oxygen atoms in dioxa[6]helicene [**202.1**]^+^BF_4_^–^ with nitrogen bridges was reported in 2019 by Lacour and Poblador-Bahamonde et al. (Scheme 202).^384^ With a smaller amount of amine (3 equiv) and benzoic acid additive (1.5 equiv), it was possible to achieve preferential formation of azaoxa species [**202.2a**]^+^. Upon increasing the stoichiometry of reagents (25 equiv of amine and 12.5 equiv of benzoic acid) along with a prolonged reaction time (7 h) at 70 °C, the diaza [**202.2b**]^+^ species could be obtained from either [**202.1**]^+^BF_4_^–^ or [**202.2a**]^+^. Amines with *n*-octyl, *i*-propyl, alcohol, carboxylic acid, and ester functional groups were generally compatible with these reaction conditions, and two different R groups could be introduced using the sequential protocol.

In 2020, Bosson, Jacquemin, and Lacour et al. showed that tertiary alkyl amines can be oxidized to enamines by cationic dioxa[6]helicene, which further reacts as an electrophile and oxidant to form mono or bis donor−π–acceptor coupling products.^385^ Cationic dioxa[6]helicene **202.1** readily oxidized tertiary alkyl amines such as diisopropylethylamine into the corresponding enamine. Then, nucleophilic oxidative addition of such an in situ generated intermediate to **202.1** led to the formation of merocyanine-like products, such as **202.3c**. With 1,3,3-trimethyl-2-methyleneindoline as a nucleophile, mono- and bis-addition products of oxidative coupling **202.3a** and **202.3b** were selectively prepared. Because of the extended π-delocalization, optical properties of **202.3** displayed strong hyper- and bathochromic shifts relative to **202.1**. These salts absorbed and emitted in the far-red and NIR domains (λ_max_^abs^ of up to 791 nm, λ_max_^em^ of up to 887 nm) and had relatively strong ECD signals in the NIR range (up to |Δε| = 60 M^–1^ cm^–1^ at ca. 800 nm for the most red-shifted derivative).

**Scheme 203:**
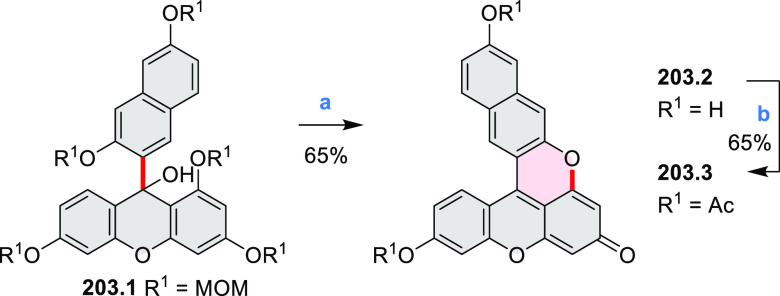
V-Shaped Dioxaphenalene Dyes Reagents and conditions: (a)^386,387^ (1) H_2_SO_4_, (2) K_2_CO_3_; (b) Ac_2_O, DMAP.

V-Shaped dioxaphenalene fluorescent dyes were described by Tsubaki et al. (Scheme 203).^386,387^ Initially, precursor **203.1** was obtained in a reaction between MOM-protected 1,3,6-trihydroxy-9*H*-xanthen-9-one and an appropriate naphthyllithium. The acid-mediated removal of the five MOM groups and intramolecular cyclization under basic conditions produced the quinoidal dioxaphenalene **203.2** in 65% yield. Subsequent treatment of **203.2** with acetic anhydride afforded the corresponding diacetate compound **203.3**. Derivatives of **203.2** containing piperidinyl groups^387^ were shown to be promising bioimaging fluorescent dyes in experiments using HeLa cells.

**Scheme 204:**
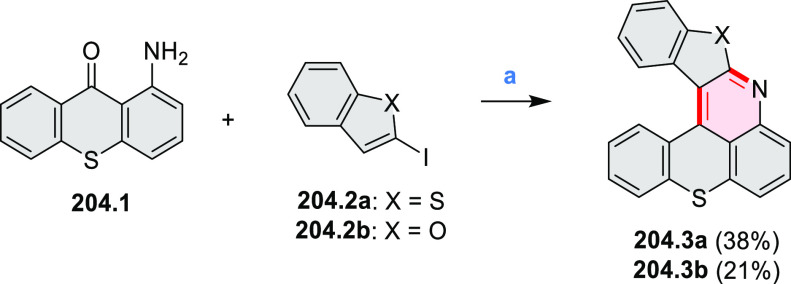
N*-*Arylation of 1-Amino-9-thioxanthone by Heteroaryl Iodides Reagents and conditions: (a)^388^ 0.2 equiv of activated Cu, 2 equiv of K_2_CO_3_, Bu_2_O, reflux, 24 h.

A one-step synthesis of azathiaphenalenes was reported by Mongin et al. in 2020, as part of their research on thioxanthone chemistry (Scheme 204).^388^ The amine **204.1**, obtained from the corresponding iodo derivative via CuI-catalyzed amination, was reacted with 2-iodobenzothiophene and 2-iodobenzofuran in the presence of activated copper (0.2 equiv) and potassium carbonate (2 equiv), yielding the corresponding hexacyclic products **204.3a**,**b**. Both compounds fluoresced in the green region of the visible spectrum, with quantum yields of ca. 0.5.

#### 5.2.2 Other Diheteraphenalenoids

**Scheme 205:**
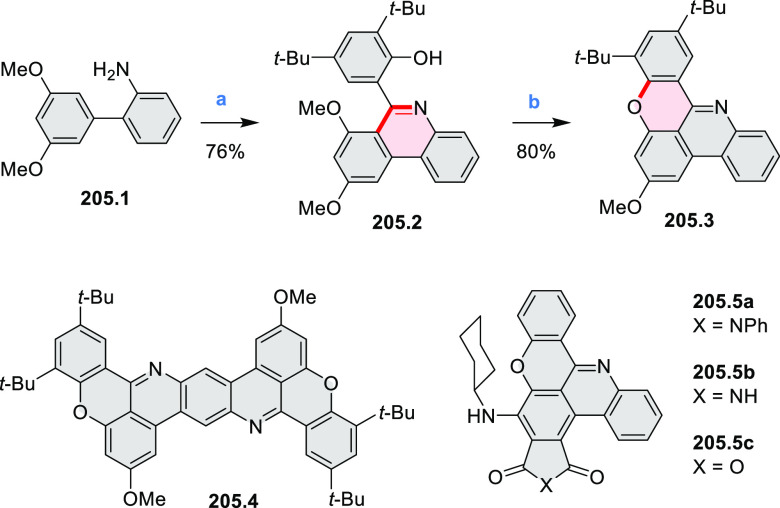
Synthesis of Oxygen- and Nitrogen-Doped Hydrocarbons Reagents and conditions: (a)^241^ 3,5-di-*tert-*butyl-2-hydroxybenzaldehyde, toluene, TFA, O_2_, 100 °C, 16 h; (b) neat, 180 °C, 24 h.

A xanthene-containing phenalenoid **205.3** was reported by Mastalerz et al., who found that **205.2**, the product of the Pictet–Spengler reaction between **205.1** and a substituted salicylaldehyde, cyclized spontaneously to give **205.3**, when heated without solvent at 180 °C (Scheme 205).^241^ This efficient transformation could also be used to make **205.4** from the corresponding diamine. In the final step, however, significantly higher reaction temperatures (300 °C) were needed. The fused systems were fluorescent in solution with a significant solvatochromism and quantum yields ranging up to 72%. An alternative multicomponent synthesis of similar chromophores, **205.5a**,**b**, was reported by Bornadiego, Díaz, and Marcos.^389^

**Scheme 206:**
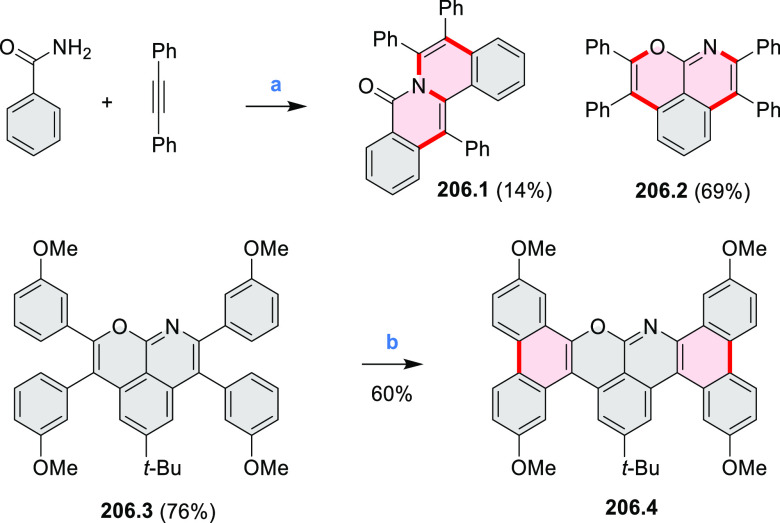
Double C–H Activation/Annulation of Benzamides with Aryl Alkynes Reagents and conditions: (a)^390^ [Cp*RhCl_2_]_2_ (5 mol %), CF_3_CO_2_Ag (30 mol %), Ag_2_O, Mg(OAc)_2_·4H_2_O, PivOH, DCE, 85 °C, under N_2_, 12 h; (b) phenyliodine(III) bis(trifluoroacetate), BF_3_·Et_2_O, DCM, N_2_, −40 °C, 2 h.

A route to helical heteroatom-doped PAHs consisting of double *ortho*-C–H activation of benzamides and subsequent N,O-double annulations with aryl alkynes was reported by Lan and You et al. in 2019 (Scheme 206).^390^ To suppress the formation of the alternative N,N-double annulation product (**206.1**), the reaction between benzamide and diphenylacetylene had to be optimized toward preferential formation of the desired N,O-annulated target **206.2**. The best result was obtained in the catalyst system of [Cp*RhCl_2_]_2_/CF_3_CO_2_Ag, in the presence of several additives. A wide scope of alkynes and *para*-substituted benzamides was demonstrated, giving the desired products in moderate to good yields. Upon treatment of one of these products, **206.3**, with PIFA and BF_3_·Et_2_O, the π-extended phenalenoid **206.4** was obtained in 60% yield. **206.4** possessed two cove regions and exhibited strong blue emission both in solution and in a PS film.

### 5.3 Higher Heteraphenalenes

#### 5.3.1 N-Doped Systems

**Scheme 207:**
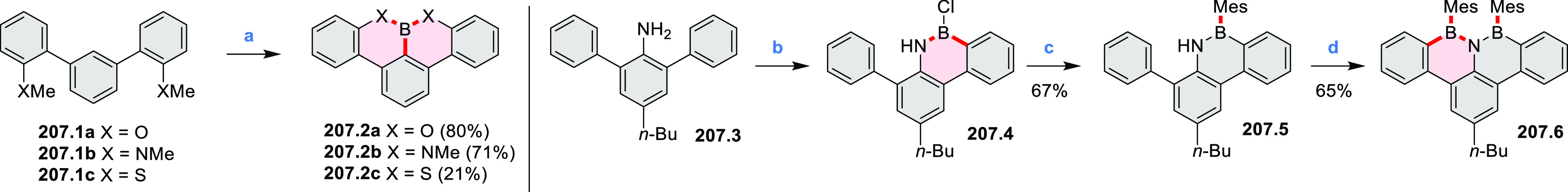
Synthesis of Boronate-Based Benzo[*fg*]tetracene Reagents and conditions: (a)^391^ BBr_3_, Brønsted bases, 120 to 200 °C, 18 h; (b)^392^ (1) BCl_3_, toluene, 0 °C to reflux, (2) AlCl_3_, reflux; (c) MesMgBr, benzene, rt; (d) (1) KHMDS, toluene, rt, (2) BCl_3_, 0 °C to rt, (3) AlCl_3_, reflux, (4) MesMgBr, benzene, rt.

A direct demethylative borylation applied to the synthesis of benzo[*fg*]tetracenes containing boronate ester, amide, and thioester substructures was reported by Hatakeyama et al. in 2016 (Scheme 207).^391^ The demethylative direct borylation of a teraryl with two methoxy groups (**207.1a**) provided the boronate-based benzo[*fg*]tetracene (**207.2a**) in one step. The key to successful synthesis was the proper choice of the boron source and Brønsted base, which facilitated the boron-mediated demethylation and successive electrophilic arene borylation. The versatile direct borylation was applied to the synthesis of benzo[*fg*]tetracenes containing boronate amide or thioester substructures (**207.2b**,**c**). The synthesis of a π-extended, hexacene analogue of **207.2a** was performed in a similar manner. The best yield (80%) for the formation of **207.2a** was obtained by using 1 equiv of BBr_3_ and 2 equiv of 2,2,6,6-tetramethylpiperidine (TMP). NaBPh_4_, acting as a noncoordinative Brønsted base, was used in the synthesis of **207.2b**, providing an improved yield of 71%. The synthesis of **207.2c** was the least efficient, despite using the harshest reaction conditions (200 °C in an autoclave). **207.2c** was observed to be air-stable but immediately decomposed in protic solvents, whereas **207.2a**,**b** showed substantial stability.

A four-step synthesis of the BNB-doped zigzag-edged benzo[*fg*]tetracene **207.6** was reported by Bettinger et al. in 2017.^392^ Preliminary experiments showed that the 2-fold addition of boron atoms to the aniline with BCl_3_ or BBr_3_ was not successful. The electrophilic borylation of one phenyl group could however be achieved using boron trichloride and aluminum trichloride. The resulting 1,2-dihydro-1,2-azaborine derivative **207.4** was too unstable to be isolated and was transformed into the mesityl derivative **207.5** using MesMgBr in diethyl ether. After deprotonation of the nitrogen center in **207.5** with KHMDS, borylation of the phenyl ring proceeded upon addition of boron trichloride and aluminum trichloride. The crude reaction product was directly transformed into the dimesityl compound **207.6** by treatment with MesMgBr. Incorporation of the BNB moiety at the zigzag periphery of the benzo[*fg*]tetracene resulted in significant bathochromic shifts of the absorption spectrum and reasonably strong fluorescence (Φ_f_ = 0.21) with a large Stokes shift (3100 cm^–1^).

**Scheme 208:**
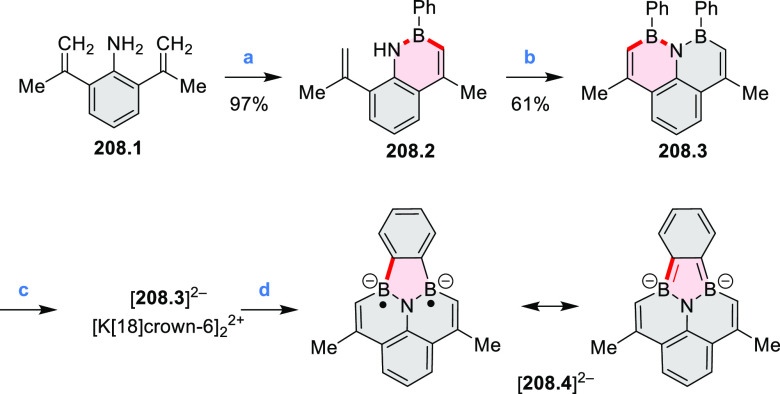
Preparation of 1,9-Dibora-9*a*-azaphenalenyl (DBAP) Anionic Species Reagents and conditions: (a)^393^ dichlorophenylborane, toluene, 0 °C to reflux; (b) (1) LiHMDS, toluene, 0 °C to rt, (2) dichlorophenylborane, 0 °C to reflux; (c) 18-crown-6, K, THF; (d) **208.3**.

A structurally related framework, **208.3**, was obtained by Zeng et al., who used a similar sequential borylation approach (Scheme 208).^393^**208.3** was chemically reduced with an excess of metallic potassium in the presence of [18]-crown-6 in anhydrous THF. The resulting salt of dianion [**208.3**]^2–^, isolated as a deep blue solid, was stable in an inert atmosphere but sensitive to moisture. Comproportionation of [**208.3**]^2–^ with an equivalent amount of neutral **208.3** produced, instead of the expected radical anion, the diamagnetic rearrangement product [**208.4**]^2–^, which was crystallographically characterized. [**208.4**]^2–^ was found to contain a completely planar BNB-doped benzo[*cd*]fluoranthene skeleton.

**Scheme 209:**
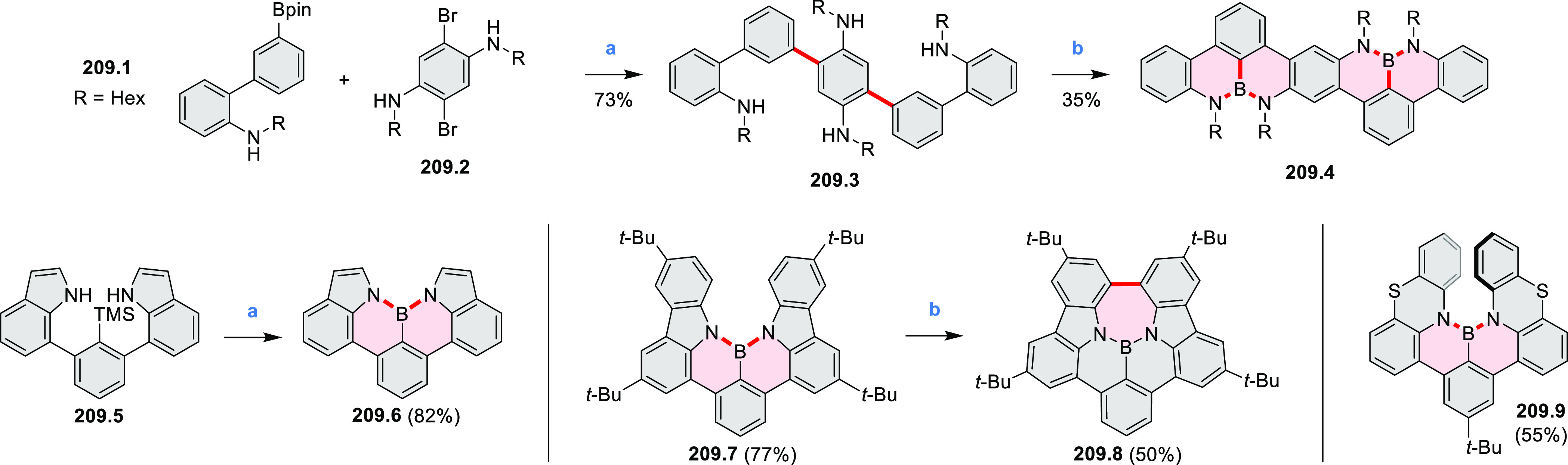
Synthesis of *NBN-*Dibenzophenalene Derivatives Reagents and conditions: (a)^394^ Cs_2_CO_3_, Pd(PPh_3_)_4_, toluene/EtOH; (b) BCl_3_, Et_3_N, *o*-DCB.

Dibenzo-fused 1,9-diaza-9*a*-boraphenalenes featuring zigzag edges with a nitrogen–boron–nitrogen bonding pattern were synthesized by Feng’s group in 2016 (Scheme 209).^394^ Quinquephenyl **209.3**, prepared via Suzuki coupling from appropriate building blocks, was treated with BCl_3_ and triethylamine at 180 °C to afford **209.4** as a yellow crystalline solid. **209.4** exhibited split emission bands at 448 nm and a high fluorescence quantum yield (Φ_PL_ = 0.83). NBN-doped phenalenoids **209.6**–**9** were synthesized by Wakamiya and Wang et al. in 2018 using a similar synthetic approach.^260^ Their syntheses consisted of a palladium-catalyzed double Suzuki coupling reaction, followed by a 2-fold electrophilic aromatic borylation. The oxidative coupling of **209.7** using DDQ led to the formation of a fully fused planar compound **209.8** in 50% yield.

**Scheme 210:**
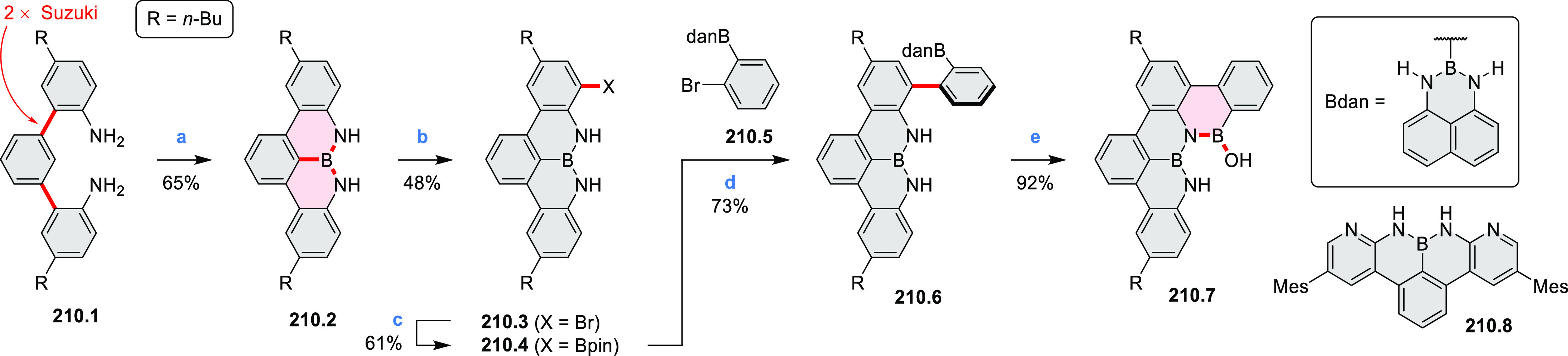
Synthesis of a Doubly BN-Doped Dibenzoperylene Derivative Reagents and conditions: (a)^395^ BBr_3_, NaBPh_4_, toluene, reflux, 18 h; (b) NBS, DCM/CHCl_3_/MeCN, 0 °C, 2 h; (c) B_2_pin_2_, KOAc, Pd(dppf)Cl_2_, 1,4-dioxane, 90 °C, 18 h; (d) Pd(PPh_3_)_4_, K_2_CO_3_, toluene/EtOH/H_2_O, reflux, 18 h; (e) degassed aq. H_2_SO_4_ (2 M), THF, 50 °C, 5 days.

In 2019, Bettinger et al. reported a series of BN-doped systems containing phenalenoid and perylenoid cores (Scheme 210).^395^ The synthetic pathway involved the diamino-substituted *m*-terphenyl **210.1**, which was subjected to electrophilic borylation with BBr_3_ in the presence of NaBPh_4_ as a noncoordinating base. Instead of the commonly used *o*-dichlorobenzene, the use of the lower-boiling toluene as the solvent was found to work well for this transformation. The resulting benzo[*f*,*g*]tetracene derivative **210.2** was singly brominated and subjected to Miyaura borylation, to give the boronate ester **210.4**, which was coupled with aryl bromide **210.5**, bearing a 1,8-naphthalenediamine-protected boryl group (Bdan). The resulting **210.6** was treated with excess degassed aqueous sulfuric acid, and the target molecule **210.7** was isolated in 92% yield. The synthesis of an analogue of **210.2** possessing two additional nitrogens **210.8** was reported by Bonifazi in 2020.^396^

**Scheme 211:**
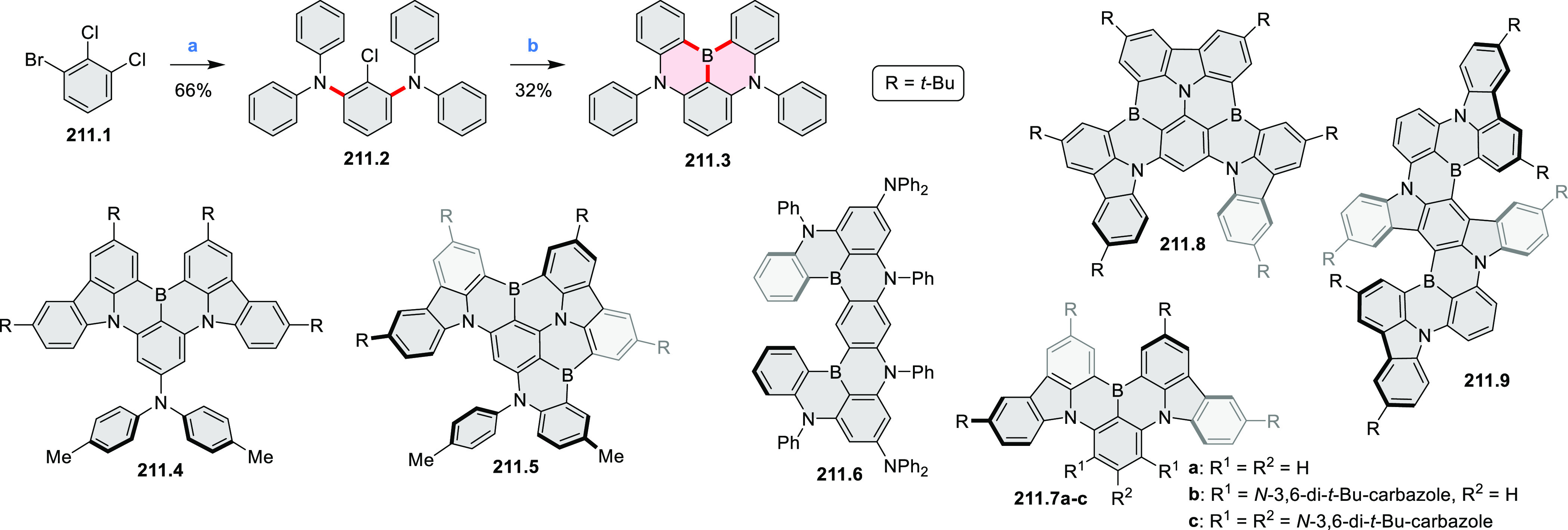
DABNA and Its Carbazole-Based Analogues Reagents and conditions: (a)^262,397^ HNPh_2_ (2.2 equiv), *t*-BuOK (2.5 equiv), (AMPHOS)_2_PdCl_2_ (1.0 mol %), *o*-xylene, 80 °C, 2 h then 120 °C, 3 h; (b) (1) *t*-BuLi (1.2 equiv), *t*-butylbenzene, 60 °C, 2 h, (2) BBr_3_ (1.2 equiv), rt, 0.5 h; EtN(*i*-Pr)_2_ (2.0 equiv), 120 °C, 3 h.

Ultrapure-blue TADF emitters, benefiting from the multiple resonance effect, were developed by Hatakeyama in 2016.^397^ The key compound **211.3** (DABNA-1) was synthesized in two steps from a commercially available starting material, 1-bromo-2,3-dichlorobenzene **211.1** (Scheme 211). The boron atom was introduced in one pot via a lithium–chloride exchange reaction of **211.2** with *tert*-butyllithium, electrophilic trapping with boron tribromide, and tandem electrophilic arene borylation to give **211.3** in 32% yield. In OLED devices, a substituted variant of **211.3** (denoted DABNA-2) exhibited pure blue emission at 467 nm with a narrow fwhm of 28 nm, CIE coordinates of (0.12, 0.13), and an IQE of ≈100%. Carbazole-based DABNA analogues **211.4**–**5** were subsequently developed by the same group,^262^ whereas the related compounds **211.6**–**8** were reported by the groups of Hatakeyama et al. and Yasuda et al.^398^ All these compounds exhibit narrowband thermally activated delayed fluorescence with emission spectra ranging from blue to red. The organic light-emitting diode devices employing these products as emitters exhibited emission with high external quantum efficiencies up to 31.8% for **211.6c**.

**Scheme 212:**
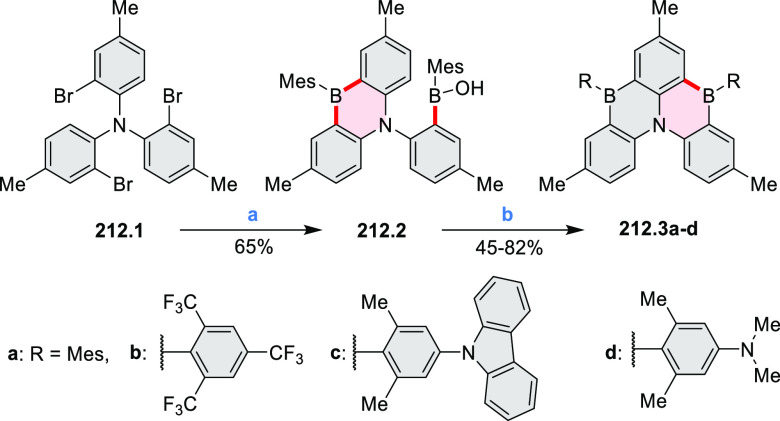
Intramolecular Borylation via Sequential B–Mes Bond Cleavage Reagents and conditions: (a)^399^*t*-BuLi and MesB(OMe)_2_, THF, −78 °C; (b) (1) BBr_3_, toluene, 110 °C, 24 h, (2) R–Li (2.4 equiv), THF or Et_2_O, −78 °C to rt, 12 h.

Benzo[4]helicenes **212.3a**–**d**, with a “reversed” BNB pattern were reported by Hatakeyama et al. and by Zysman-Colman and Wang et al. (Scheme 212).^399^ The borinic acid **212.2** was prepared from readily available **212.1** and converted into **212.3a**–**d** via borylation followed by the treatment with the corresponding phenyllithium. Small Δ*E*_S–T_ values and emission enhancement at higher temperatures supported the TADF behavior of these derivatives.

**Chart 20:**
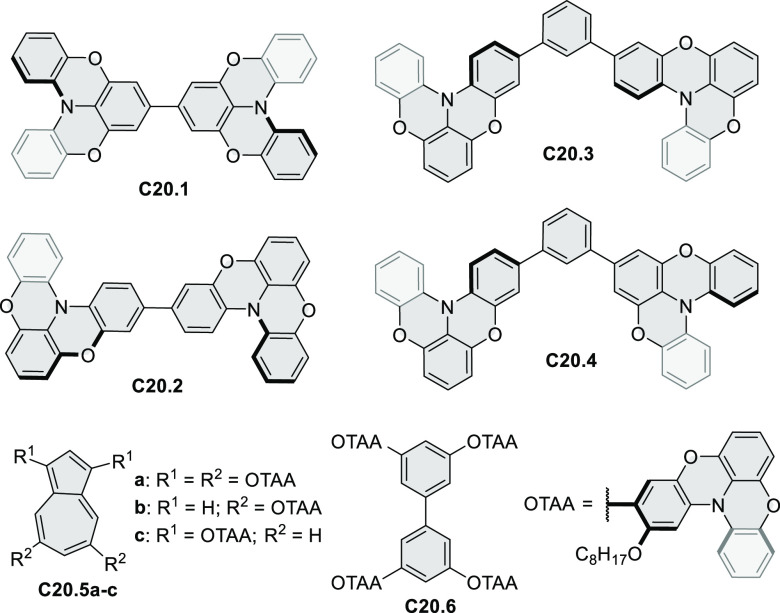
Oxygen-Bridged Triphenylamine Skeletons

Wakamiya and Murata et al. explored semiconducting properties of various structures based on oxygen-bridged triphenylamine subunits (Chart 20).^400−402^ The isomeric dimers **C20.1** and **C20.2** showed, respectively, 1D and 2D stacking patterns in the crystalline state, which influenced their charge-transporting behavior. While **C20.1** showed strong field dependency in the bulk charge mobilities, **C20.2** exhibited ambipolar charge-transporting properties with comparable hole and electron mobilities (ca. 10^–3^ cm^2^ V^–1^ s^–1^). The fluorescence spectra of amorphous films of **C20.1** and **C20.2** suggested the coexistence of a monomer-like random orientation and a π-stacking orientation. Hole-transporting materials, in which the two identical (**C20.3**) or different subunits (**C20.4**) were connected via a 1,3-phenylene linker, were reported in 2016.^401^ These compounds showed excellent amorphous stability and did not crystallize even after annealing at 160 °C. Vacuum-deposited amorphous films of **C20.3** and **C20.4** combined transparency and good hole mobility. **C20.5a**–**c** and **C20.6** were designed as hole-transporting materials for perovskite solar cells, showing better performance (PCE of 16.5% for **C20.5a**) than the commonly used Spiro-OMeTAD.^400^

**Chart 21:**
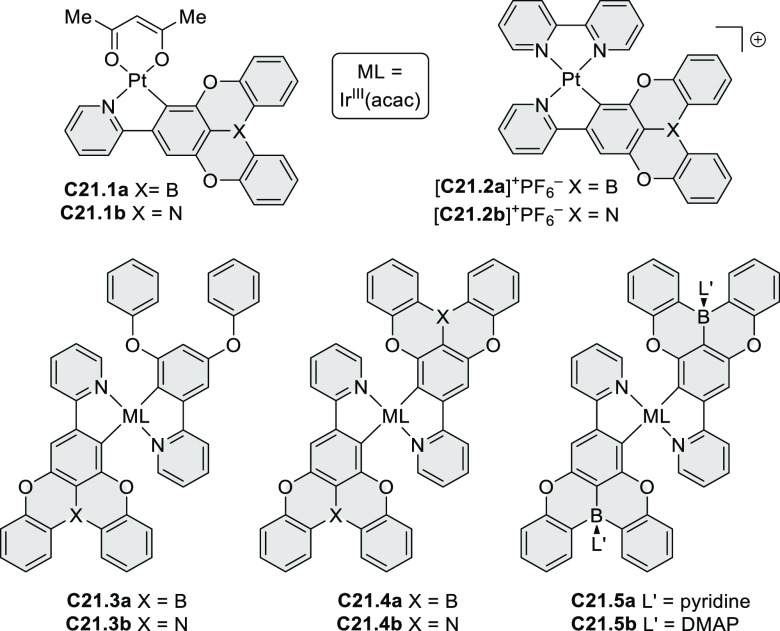
Metal Complexes with NBO-Doped Polycyclic Ligands

Phosphorescent iridium and platinum complexes with N,B,O-doped polycyclic ligands were described by Yuan et al. (Chart 21).^403,404^ Single-crystal structures indicated that the B-containing structures were more planar than their N-doped analogues, leading to different π–π-stacking and electronic characteristics. Solid-state packing could be further modified by coordination of pyridine ligands to the boron centers (**C21.5a**,**b**). More importantly, by controlling the number of embedded boron or nitrogen atoms (**C21.3a**,**b** vs **C21.4a**,**b**), solution-processed OLED devices incorporating these materials as emitting layers could achieve a phosphorescence color variation from green to deep red and showed low-efficiency roll-off and turn-on voltage. In particular, the boron derivative **C21.4a** showed good color purity with a narrow full width at half-maximum (1211 cm^–1^) and CIE coordinates (0.67, 0.31) in the deep-red region. Platinum complexes **C21.1**–**2** showed a significant dependence of intermolecular interactions in the solid state on the nature of the X heteroatom (B vs N), attributed to both steric and electronic effects. Under a hypoxic atmosphere, the N-containing neutral complex **C21.1b** displayed dual emission with both fluorescence and phosphorescence, whereas only fluorescence was observed in air. In contrast, the boron analogue **C21.1a** showed only phosphorescence at all dioxygen levels.

#### 5.3.2 Tricycloquinazolines and Related Systems

**Scheme 213:**
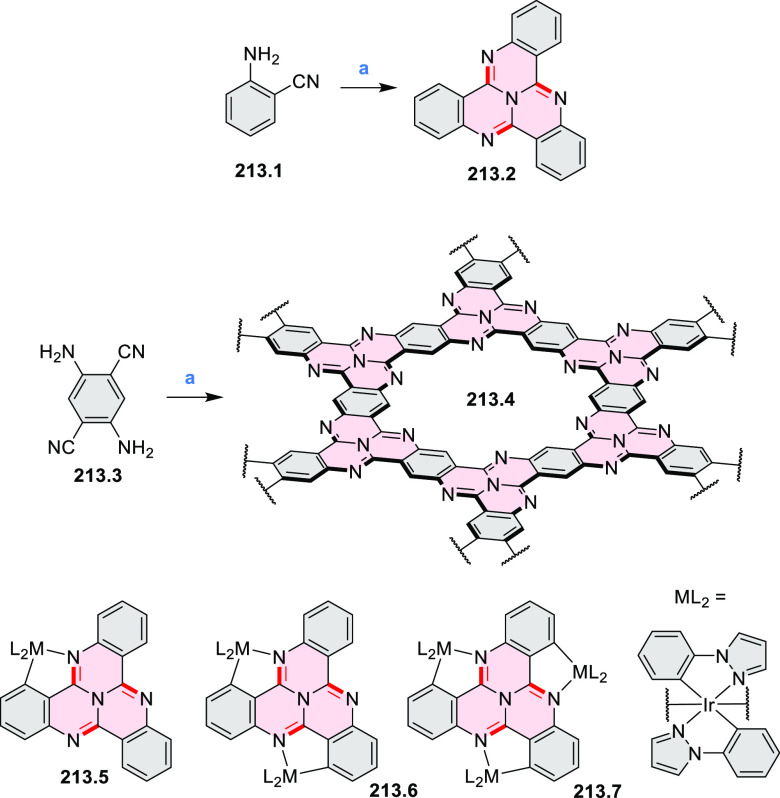
Synthesis of Porous Covalent Quinazoline Networks Reagents and conditions: (a)^405^ ZnCl_2_, 300–400 °C.

The century-old tricycloquinazoline motif **213.2** (see CR2017, Section 5.3.15.3.1) was revisited by Ruoff et al. in 2018 (Scheme 213).^405^ Using an ionothermal protocol, **213.2** was prepared in excellent yields by heating a mixture of **213.1** and 1 equiv of ZnCl_2_ at 300–350 °C in flame-sealed ampules. In the same way, heat treatment of the diamine **213.3** in the presence of 1 equiv of ZnCl_2_ at 300 to 400 °C produced covalent networks with the idealized structure **213.4**. **213.4** showed high chemical and thermal stability, a surface area greater than 1800 m^2^ g^–1^, and large pore volumes of up to 0.93 cm^3^ g^–1^. Excellent CO_2_ uptake capacity was demonstrated for these materials, reaching 7.16 mmol g^–1^ at 273 K and 1 bar.

Tricycloquinazoline was found to use a bridging ligand in a series of iridium complexes **213.5**–**7** reported by Shensky et al.^406^ Transient absorption bands observed for all three complexes in the 475–900 nm range implied the potential for reverse-saturable absorption (RSA) at those wavelengths. The presence of RSA behavior was further confirmed for all three compounds using Z-scan measurements. The enhanced triplet cross section and ratio of triplet cross section to ground-state cross section for **213.5** suggested that it might be more beneficial for RSA applications than **213.6**–**7**.

### 5.4 Phenalenoids with Nonbenzenoid *peri*-Fusion

#### 5.4.1 Cyclopenta[*cd*]phenalenes

**Scheme 214:**
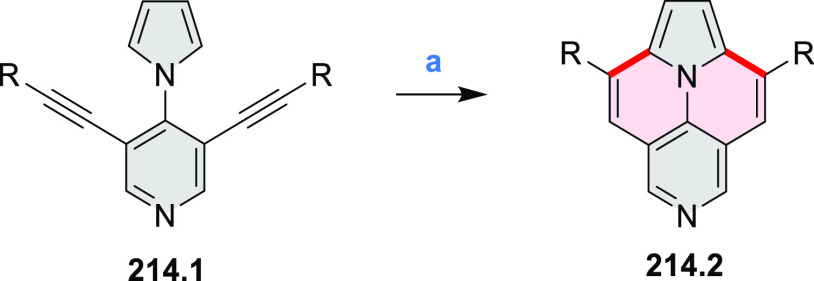
Synthesis of Azaullazines Reagents and conditions: (a)^407^*p*-TSA, xylene, 120 °C, 24 h.

**Scheme 215:**
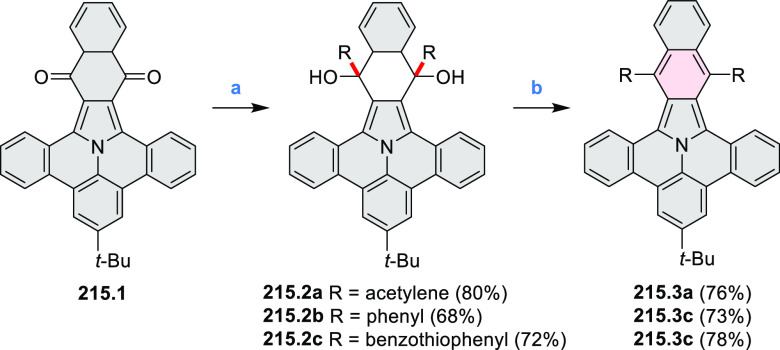
π-Extended Ullazine with a Benzoisoindole Core Reagents and conditions: (a)^408^ for **215.2a**: *n*-BuLi, triisopropylsilylacetylene, THF, 0 °C to rt, 3 days, **215.2b**: phenyllithium, THF, rt, 3 days, **215.2c**: *n*-BuLi, 2-bromobenzothiophene, THF, −78 °C to rt, 3 days; (b) SnCl_2_, DCM, rt, 3 days.

Fused and heteroatom-doped derivatives of ullazines have been explored by several research groups (see also Scheme 21, Section 2.4, and CR2017, Section 5.4.1 for earlier developments). An efficient synthesis of 6-aza-ullazines was described by Langer and co-workers in 2017 (**214.2**, Scheme 214).^407^ The procedure involved metal-free cyclization promoted by *p*-toluenesulfonic acid as the key ring-forming step. These aza derivatives showed similar absorption and emission spectra but higher oxidation potentials than the parent ullazine. Three naphtho-fused derivatives **215.3a**–**c** were synthesized by Berger and Feng et al. from the known dione **215.1** (Scheme 215).^408^ Nucleophilic addition of lithium triisopropylsilylacetylenide, phenyllithium, and benzo[*b*]thiophen-2-yllithium to **215.1** gave diols **215.2a**–**c**, which were subsequently reduced with anhydrous SnCl_2_ to yield **215.3a**–**c**. These targets had easily accessible radical cationic and anionic states, which were generated using *in situ* spectroelectrochemistry.

**Scheme 216:**
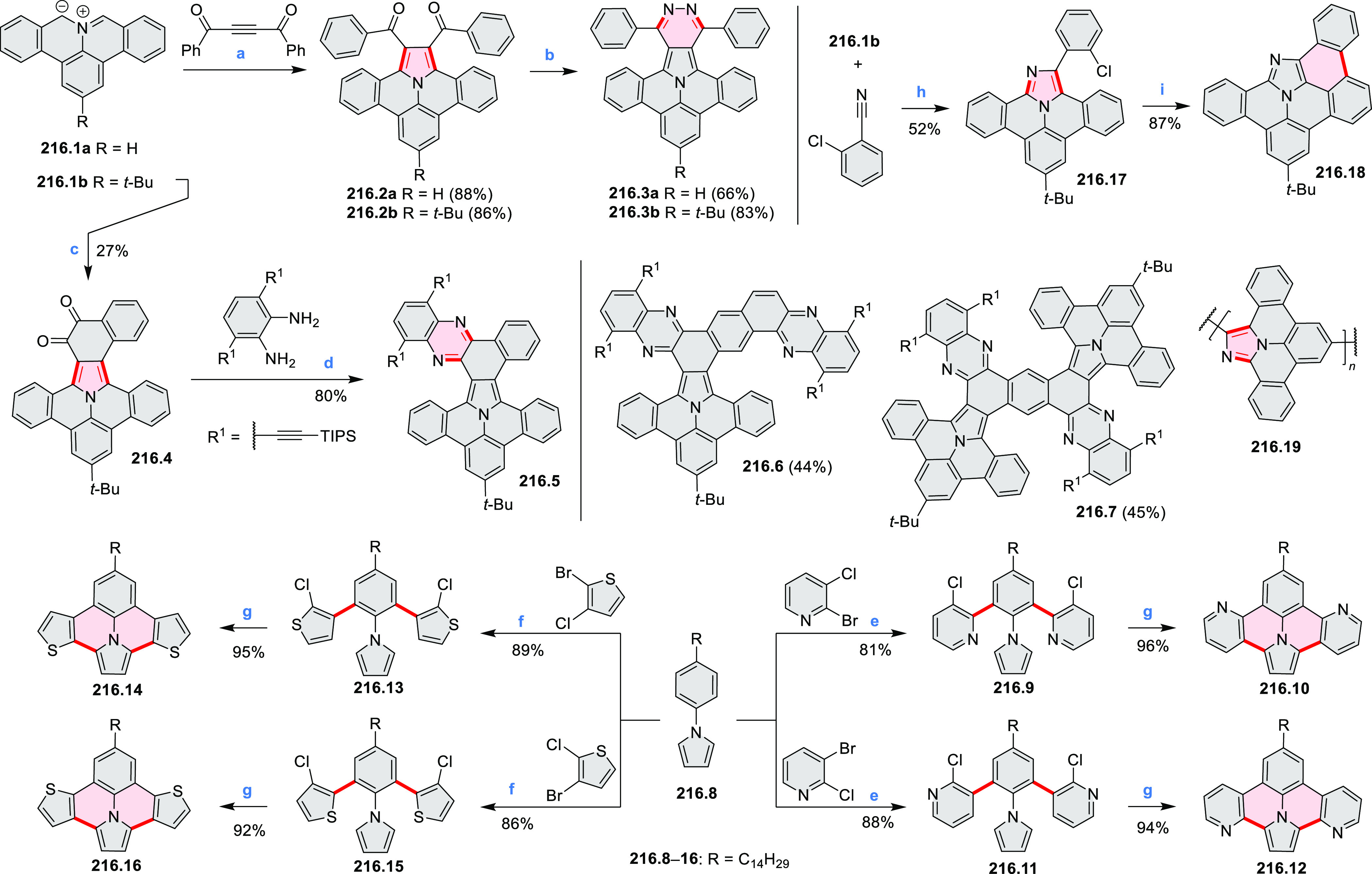
Synthetic Routes of the Ullazine Derivatives Reagents and conditions: (a)^409^ (1) 1,4-diphenylbut-2-yne-1,4-dione, DCM, 5 min, rt, (2) DDQ, toluene, rt, 3 h; (b) hydrazine, ethanol, overnight, 80 °C; (c)^410^ (1) 1,2-naphthoquinone, DCM, TEA, rt, 5 min, (2) DDQ, toluene, rt, 3 h; (d) HOAc, toluene, 100 °C, 72 h; (e)^413^ Pd(dppf)Cl_2_, Na_2_CO_3_, DMSO, 100 °C, 16 h; (f) PdCl_2_(PPh_3_)_2_, PPh_3_, K_2_CO_3_, DMF/H_2_O, 90 °C, 24 h; (g) *hν* (254 nm), decalin, 100 °C, 2 h; (h)^411^ (1) *i*-Pr_2_NEt, CsF, DMSO, 160 °C, 12 h, (2) DDQ, DCM, rt, 20 min; (i) Pd(AcO)_2_, P(*t*-Bu)_2_Me·HBF_4_, DBU, DMA, 160 °C, 12 h.

In 2019, Bunz, Feng, and Berger et al. reported ullazine derivatives (**216.3**–**7**) obtained through cycloaddition reactions of the previously reported polycyclic aromatic azomethine ylides (PAMYs, Scheme 216).^409,410^ Intermediates **216.2a**,**b** were initially obtained via 1,3-dipolar cycloaddition of PAMYs, generated in situ from the corresponding salts **216.1a**,**b** by addition of triethylamine (TEA), followed by dehydrogenation with DDQ. **216.2a**,**b** were then condensed with hydrazine to yield the final compounds **216.3a**,**b** as yellow solids.^409^ The cycloaddition reaction of PAMY **216.1b** with *ortho*-1,2-naphthoquinone and the following oxidation gave the intermediate **216.4**, which provided the helicene-like **216.5** upon condensation with *ortho*-benzenediamine. The same synthetic method was used to obtain larger fused frameworks by cycloaddition of PAMY to anthracene-1,2,6,7-tetraketone, acting as a single or double dipolarophile. Subsequent condensation with *ortho*-benzenediamine provided **216.6**–**7** with two and four [5]helicene motifs, respectively.^410^

Ito et al. reported the 1,3-dipolar cycloaddition between the PAMY derived from **216.1b** with nitriles to produce highly fused imidazole derivatives such as **216.17**.^411^ This transformation had a broad substrate scope and good functional group compatibility. Subsequent palladium-catalyzed intramolecular cyclization provided access to even more π-extended imidazoles such as **216.18**. Analogous cycloaddition chemistry was used by Palma et al. for on-surface oligomerization of cyano-substituted PAMYs into dibenzoullazine chains **216.19** on Au(111), Ag(111), and h-BN/Cu(111) surfaces.^412^

A metal-free, photochemical cyclodehydrochlorination reaction was developed by Morin et al. as an alternative route to π-extended ullazine derivatives annulated with electron-poor pyridine (**216.10** and **216.12**) and electron-rich thiophene units (**216.14** and **216.16**).^413^ The common starting material **216.8** was coupled to 2-bromo-3-chloropyridine and 3-bromo-2-chloropyridine to give the pyridyl-substituted precursors **216.9** and **216.11**. Likewise, coupling with 2-bromo-3-chlorothiophene and 3-bromo-2-chlorothiophene gave compounds **216.13** and **216.15**, respectively. When subjected to the photochemical cyclodehydrochlorination reaction, all four precursors produced the ullazine analogues **216.10**, **216.12**, **216.14**, and **216.16** in excellent yields.

**Scheme 217:**
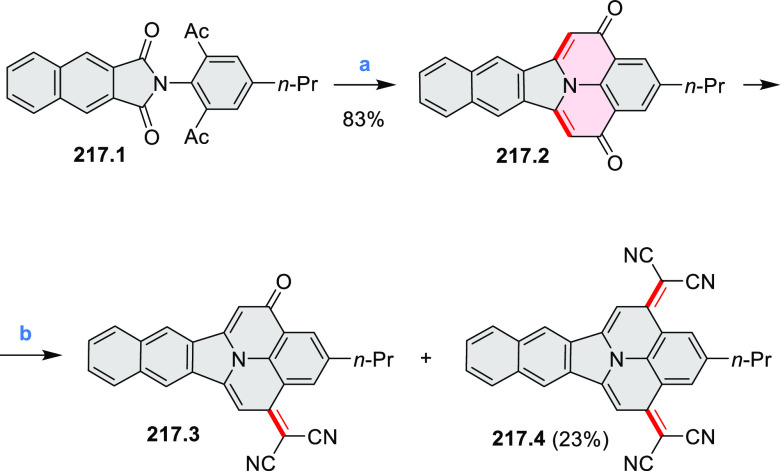
Synthesis of π-Extended Cycl[3,3,2]azines Reagents and conditions: (a)^414^ imidazole, 120 °C, 2 h; (b) AcOH, Ac_2_O, malononitrile, reflux.

A series of π-extended cycl[3,3,2]azines (**217.2**–**4**), structurally related to ullazines, were reported by Klauk, Müllen, and Li et al. in 2018 (Scheme 217).^414^ Following an established route, the initial aldol condensation was carried out on **217.1** in imidazole solution, giving **217.2** in good yields. Malononitrile was reacted with **217.2** under acidic conditions, to produce the singly and doubly condensed products, **217.3** and **217.4**. These two systems were found to be strong electron acceptors with low-lying LUMO energy levels of −3.99 and −3.95 eV, respectively. Organic thin-film transistors based on **217.4** were fabricated by vapor deposition, exhibiting extraordinarily stable *n*-type semiconductor character under ambient conditions, with the highest electron mobility of 0.06 cm^2^ V^–1^ s^–1^ retained consistently for more than 30 months.

**Scheme 218:**
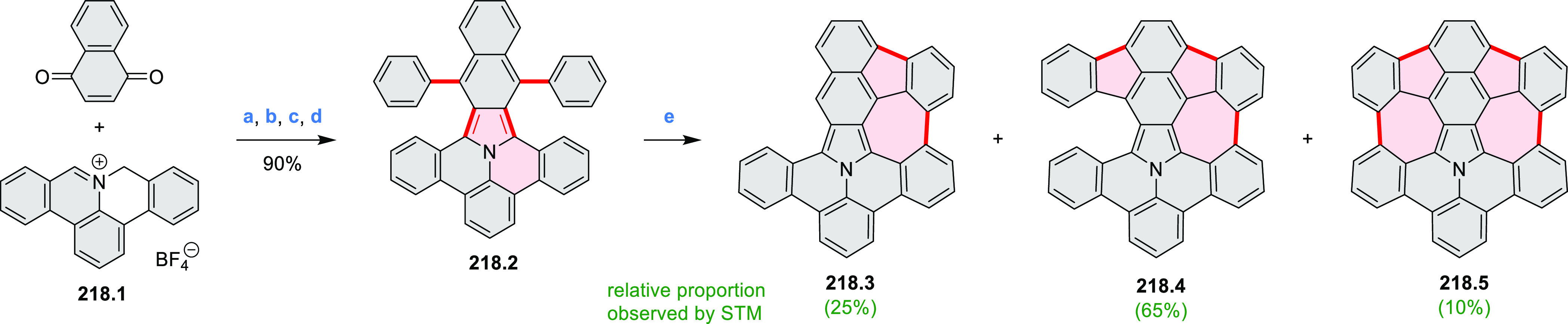
On-Surface Synthesis of Fused Azananographenes Reagents and conditions: (a)^415^ Et_3_N, DCM, rt, 5 min; (b) DDQ, toluene, rt, 3 h; (c) PhLi (excess), THF, rt, 3 days; (d) SnCl_2_, DCM, rt, 2 h; (e) 300 °C on Au(111).

In 2019, Moresco et al. reported the on-surface synthesis of a nitrogen-doped nanographene bearing five- and seven-membered rings (Scheme 218).^415^ The extended ullazine **218.2** was first synthesized in four steps from 1,4-naphthoquinone and the iminium salt **218.1** in 90% overall yield (for related cycloaddition reactions, see Scheme 221, Section 6.1.1). Afterward, compound **218.2** was deposited on an atomically clean Au(111) surface. The sample was annealed starting at 160 °C, and structural changes began to emerge after annealing to 250 °C as observed by STM. After annealing at 300 °C, three new species were observed and identified at high resolution with a CO-functionalized tip to be dephenylated, doubly cyclized species **218.3**, the triply cyclized species **218.4**, and the quadruply cyclized species **218.5** in relative abundances of 25%, 65%, and 10%, respectively.

**Scheme 219:**
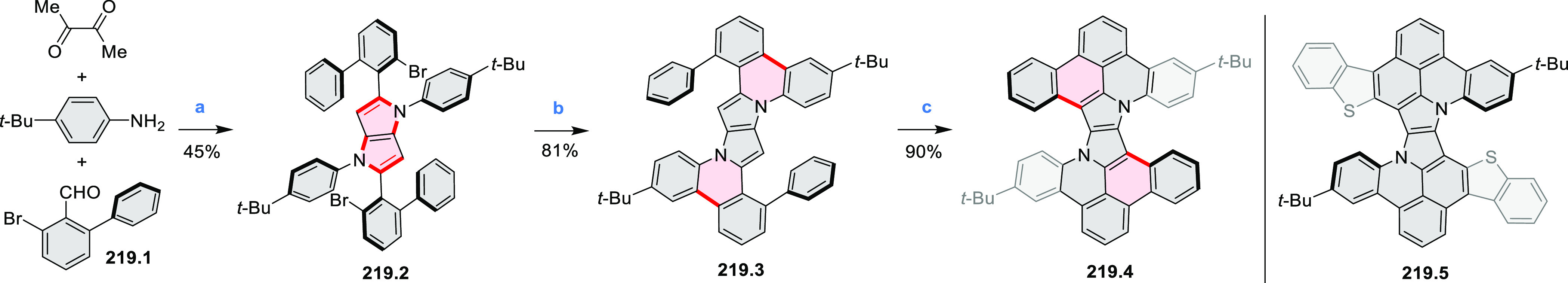
π-Extended Pyrrolopyrroles with a Double-Helicene Structure Reagents and conditions: (a)^416^*p*-TsOH, AcOH, 90 °C, 3 h; (b) Pd(AcO)_2_, PPh_3_, Cs_2_CO_3_, toluene, 120 °C, 3 h; (c) FeCl_3_, DCM, MeNO_2_, rt, 0.5 h.

Gryko and co-workers reported the synthesis of butterfly-shaped pyrrolo[3,2-*b*]pyrroles containing dual [6]helicene substructures (Scheme 219).^416^ Specifically, **219.4**–**5** were obtained in a three-step procedure, involving efficient intramolecular oxidative aromatic coupling in the final ring-forming step. These nanographenoids had nonplanar, twisted structures and were moderately emissive in solution (QY up to 32%) with observable solvatochromism.

**Scheme 220:**
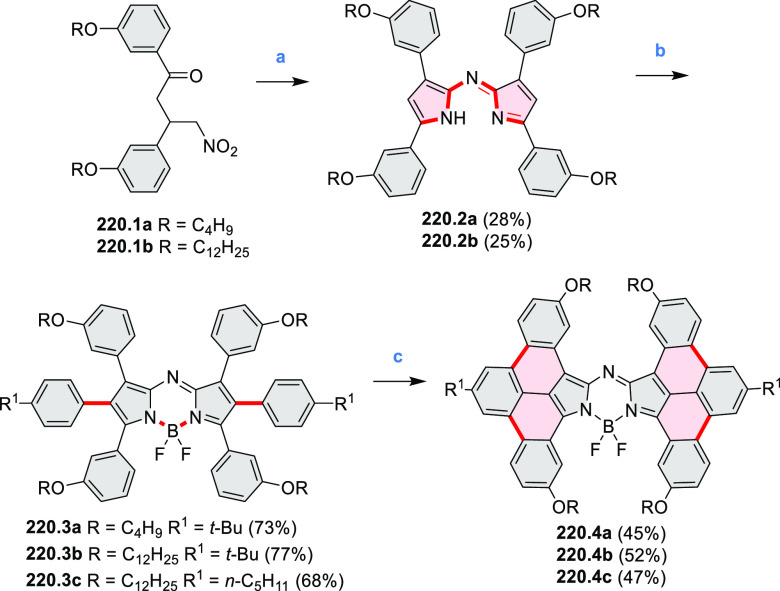
Synthesis of π-Extended Aza-BODIPYs Reagents and conditions: (a)^417^ CH_3_CO_2_NH_4_, EtOH, reflux; (b) (1) 2 equiv of Br_2_, DCM, rt, (2) 4-alkylphenylboronic acid, Pd(PPh_3_)_4_, Na_2_CO_3_, (3) Et_3_N, BF_3_·Et_2_O; (c) TfOH, DDQ, DCM, rt.

The groups of Jiao, Wang, and Pei reported in 2020 the synthesis of azadipyrromethene-based polycyclic aromatics containing 13 fused rings (Scheme 220).^417^ The installation of phenyl rings at 2,6-positions of the tetraaryl azadipyrromethenes **220.2** was achieved using Suzuki coupling, and the resulting intermediates were converted into BF_2_ complexes and subjected to oxidative ring-fusion reaction. The latter step used DDQ/TfOH as the oxidant to give the target **220.4**. In comparison with **220.3**, the fully annulated **220.4** showed a sharp absorption between 800 and 880 nm (red-shifted by more than 160 nm relative to **220.3**) and emission with a very small Stokes shift.

## 6 Nonbenzenoid Fusion

### 6.1 Circulenoids and Related Systems

#### 6.1.1 Heterofused Circulenes

**Scheme 221:**
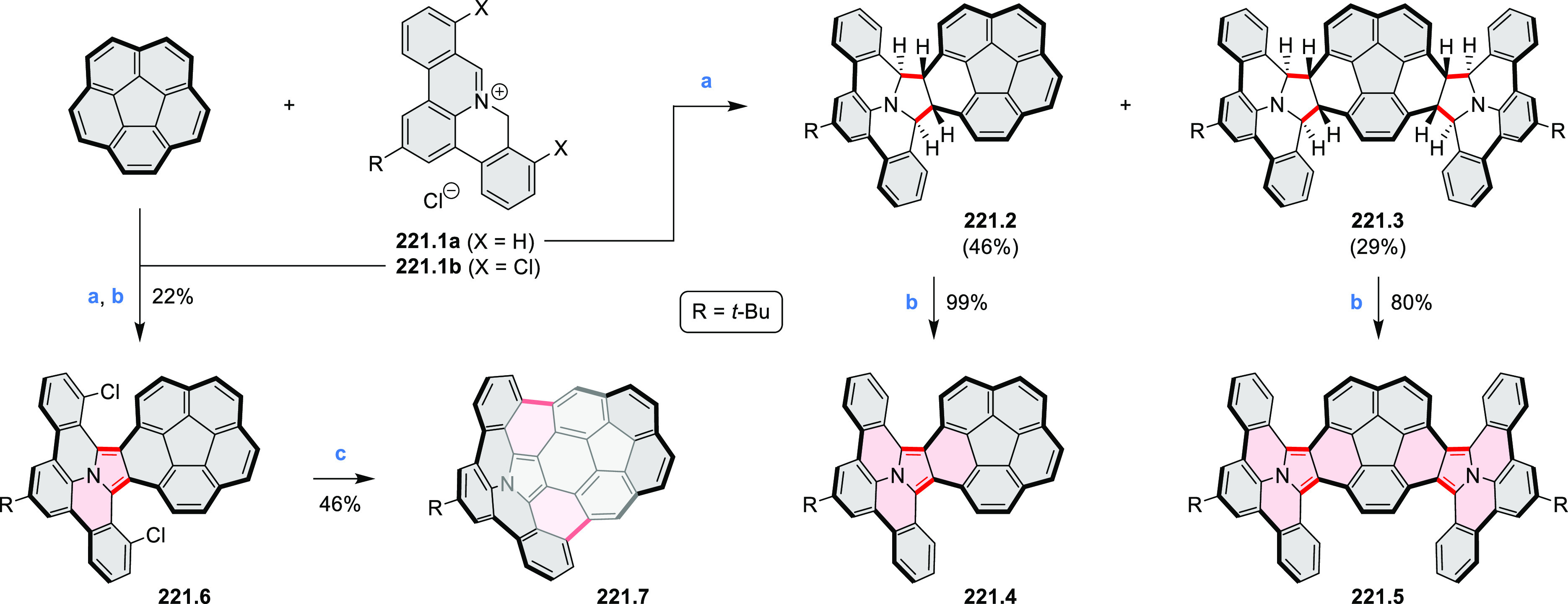
Azabuckybowl Synthesis via 1,3-Dipolar Cycloaddition of Corannulene Reagents and conditions: (a)^418,419^*i*-Pr_2_NEt, DMSO, 120–140 °C, 1–20 h; (b) DDQ (1.8–4.2 equiv), DCM, rt, 10 min–14 h; (c) Pd(OAc)_2_, *t-*Bu_2_PMe·HBF_4_, DBU, DMA, 150 °C, 24 h.

In 2017, Tokimaru, Ito, and Nozaki reported the first 1,3-dipolar cycloaddition between corannulene and a polyaromatic azomethine ylide (Scheme 221).^418^ In the presence of Hünig’s base, the iminium salt **221.1a** was deprotonated to give the corresponding azomethine ylide (the 1,3-dipole), which then reacted with corannulene (the dipolarophile). The single and double cycloadducts **221.2** and **221.3** were isolated in 46% and 29% yield, respectively. The X-ray structures of both compounds indicated the 1,3-dipole approached the rim C=C bonds of corannulene from the convex face in an *exo* manner. On DDQ-mediated dehydrogenation of **221.2**–**3**, the corresponding pyrrole-fused corannulenes **221.4**–**5** were obtained in high yields. In 2018, the same authors reported an analogous reaction sequence with the iminium salt **221.1b** bearing two chlorine atoms.^419^ The palladium-catalyzed ring closures of **221.6** afforded compound **221.7** with two new six-membered rings in 46% yield. The highly curved structure of **221.7** was evident from the large bowl depth of 4.19 Å determined from the X-ray structure. Compared with the HOMO (−5.12 eV) and LUMO (−2.52 eV) levels of the noncyclized analogue **221.4**, the additional π-conjugation in **221.7** lowered the LUMO level (−3.04 eV) but did not alter the HOMO level (−5.11 eV).

**Scheme 222:**
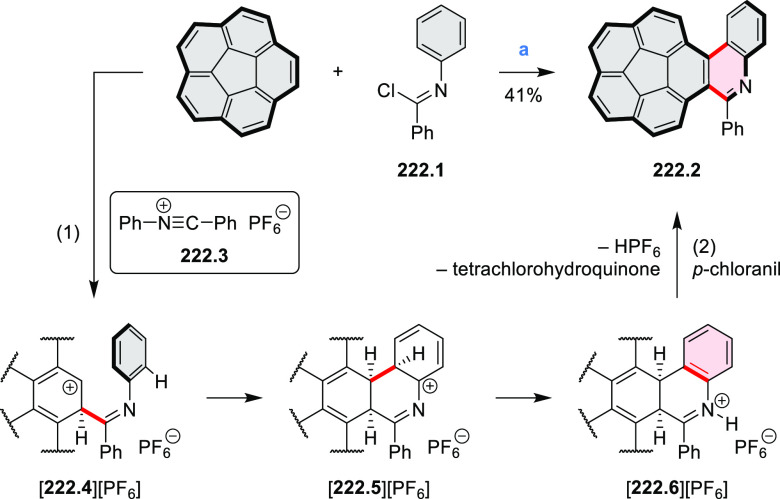
Aza-Annulative π-Extension Reaction of Corannulene Reagents and conditions: (a)^420^ (1) AgPF_6_, 1,2-DCE, 80 °C, 17 h, (2) *p*-chloranil, rt, 3 h.

In 2020, an aza-annulative π-extension (aza-APEX) reaction, applicable to unfunctionalized aromatics, was described by Itami and Ito et al. (Scheme 222).^420^ In one of the trials, a solution of corannulene, *N-*phenylbenzimidoyl chloride (**222.1**), and AgPF_6_ in 1,2-dichloroethane was heated at 80 °C, followed by addition of *p*-chloranil. The quinoline-fused corannulene derivative **222.2** was isolated in 41% yield. The authors proposed the following mechanism based on extensive DFT calculations. First, the imidoyl chloride **222.1** was converted to the nitrilium salt **222.3** upon the action of AgPF_6_. An initial π-complex formed between the nitrilium ion and the arene gave rise to the carbocationic intermediate [**222.4**]^+^. Afterward, an intramolecular C–C bond formation between the cationic center and the *N-*phenyl group generated a new six-membered ring in [**222.5**]^+^. Proton transfers assisted by PF_6_^–^ gave the rearomatized species [**222.6**]^+^, which was subsequently dehydrogenated by *p*-chloranil to produce the target product.

**Scheme 223:**
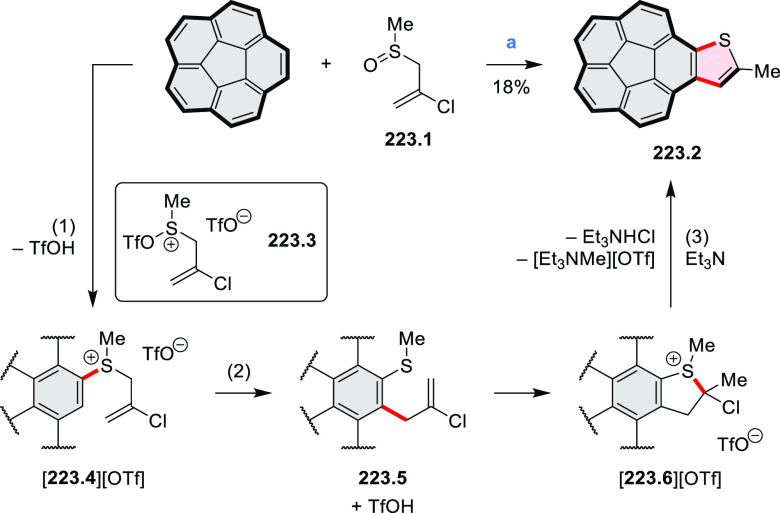
Transition-Metal-Free, One-Pot Thienannulation of Corannulene Reagents and conditions: (a)^284^ (1) Tf_2_O, 1,2-DCE, −30 °C, 20 min, then rt, 1 h, (2) microwave, 90 °C, 12 h, (3) Et_3_N, rt, 20 min, then 50 °C, 5 h.

Procter et al. reported a transition-metal-free, one-pot protocol for the thienannulation of arene substrates via 2-fold C–H functionalization (Scheme 223).^284^ In a representative example, the overall reaction between corannulene and 2-chloro-3-(methylsulfinyl)prop-1-ene (**223.1**) gave rise to the corannulene derivative **223.2** bearing an *ortho*-fused methylthiophene unit in 18% yield. Mechanistically, the sulfoxide **223.1** was first activated by triflic anhydride to become the salt **223.3**, which participated in an intermolecular interrupted Pummerer reaction with corannulene. The resulting sulfonium ion **223.4**^+^, upon microwave heating, underwent a [3,3]-sigmatropic rearrangement into the neutral intermediate **223.5**, which then spontaneously cyclized in the presence of acid to give the sulfonium species **223.6**^+^. Eventually, addition of triethylamine followed by heating effected the elimination of HCl and removal of the sulfur-bonded methyl group, thereby providing the desired thienannulation product **223.2**.

**Scheme 224:**
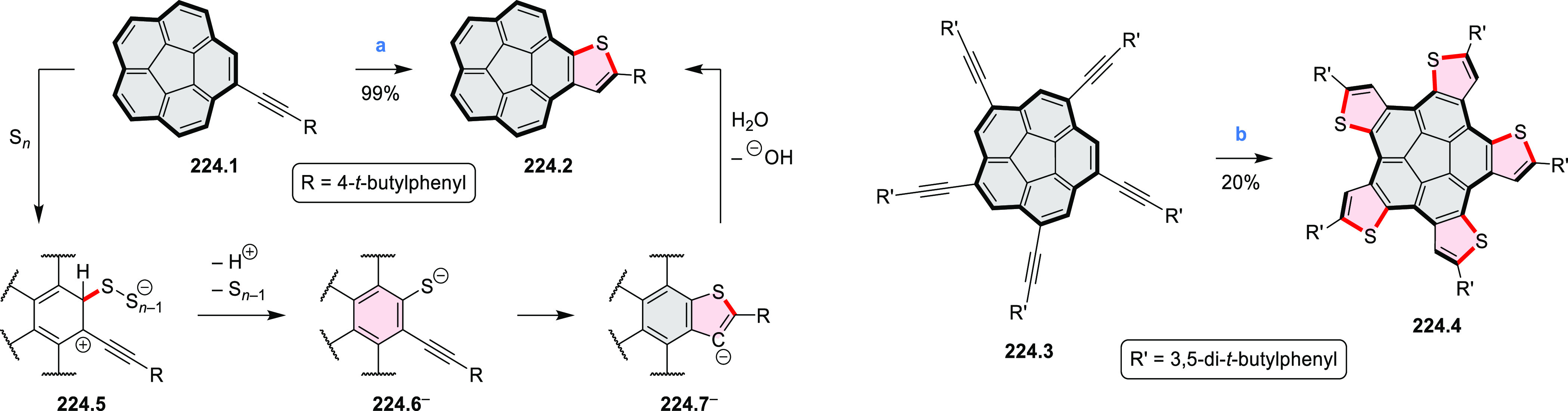
Thienannulation of Arylethynyl-Substituted Corannulenes with Elemental Sulfur Reagents and conditions: (a)^209^ S_8_ (0.5 equiv), 140 °C, 24 h; (b) S_8_ (5 equiv), DMF, 140 °C, 48 h.

Another transition-metal-free thienannulation of arylethynyl-substituted arenes using elemental sulfur was reported by Itami and Segawa et al. (Scheme 224).^209^ In the typical procedure, a solution of (4-*tert*-butylphenyl)ethynyl-substituted corannulene **224.1** in DMF was heated in the presence of elemental sulfur for 24 h. The cyclization product **224.2** bearing an *ortho*-fused thiophene unit was obtained in 99% yield. Likewise, the 5-fold thienannulation of the *C*_5_-symmetrical pentakis(arylethynyl)-substituted corannulene **224.3** furnished the corresponding pentathieno-fused derivative **224.4** in 20% yield. Based on a series of experiments, the authors proposed the following mechanism. First, the electrophilic S_*n*_ forms a C–S bond with the arene to form the zwitterion **224.5**, in which the positive charge is stabilized by both the neighboring aromatic ring and the alkyne unit. Subsequently, the elimination of elemental sulfur (S_*n*–1_) and a proton generates the rearomatized thiolate intermediate **224.6**^–^. Then, the nucleophilic sulfur anion attacks the alkyne unit, forming the carbanionic species **224.7**^–^, which eventually leads to the target product upon protonation by residual water. The authors showed that this simple procedure could be carried out on a decagram scale and could allow multiple thienannulations.

**Scheme 225:**
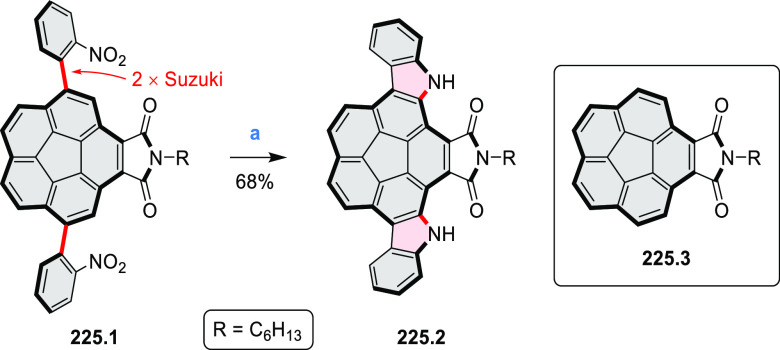
Synthesis of Diindole-Fused Corannulene Imide by Reductive Ring Closure Reagents and conditions: (a)^421^ PPh_3_, dry *o*-dichlorobenzene, 180 °C, 36 h.

In 2018, Bao et al. prepared the corannulene imide derivative **225.2** bearing two *ortho*-fused indole units via the triphenylphosphine-mediated 2-fold reductive cyclization of the bis(2-nitrophenyl)-substituted precursor **225.1** (Scheme 225).^421^ They found that the diindole-fused derivative **225.2** had an absorption onset at 588 nm, significantly red-shifted from that of the corannulene imide **225.3** without any indole fusions (437 nm). Using DFT calculations, they also determined that the diindole-fused derivative **225.2** possessed a bowl-to-bowl inversion barrier of 7.2 kcal mol^−1^, which was lower than that of **225.3** (9.8 kcal mol^−1^).

**Scheme 226:**
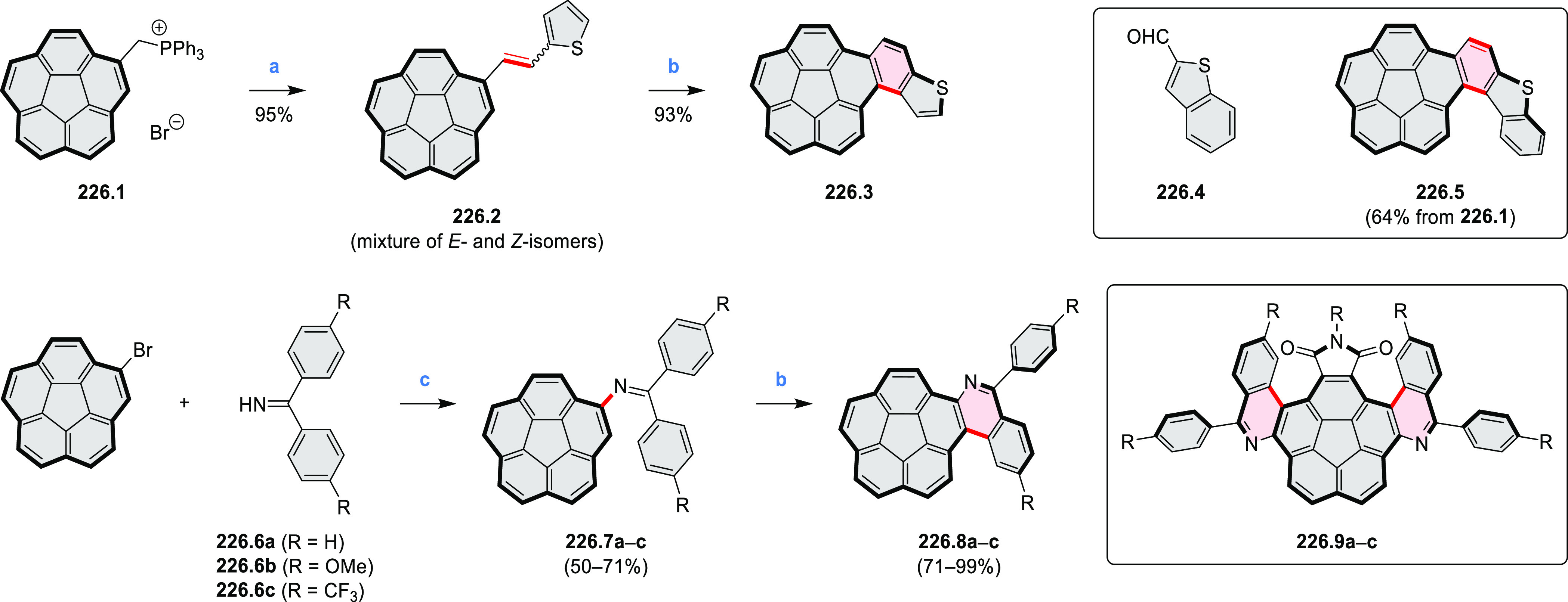
π-Extension of Corannulene via Photocyclization Reagents and conditions: (a)^422^ (1) *n*-BuLi, THF, 0 °C, 30 min, (2) 2-thiophenecarboxaldehyde, rt, 2 h; (b) I_2_, propylene oxide, medium-pressure Hg lamp (125 W), toluene; (c)^423^ Pd_2_(dba)_3_, *rac*-BINAP, *t*-BuONa, toluene, 110 °C, 12 h.

In 2016, Rajeshkumar and Stuparu reported a two-step synthesis of π-extended corannulenes (Scheme 226).^422^ First, the corannulene-based phosphonium ylide **226.1** underwent the Wittig reaction with 2-thiophenecarboxaldehyde to furnish an *E*/*Z* isomeric mixture of the stilbenoid product **226.2** in 95% yield. The Mallory reaction of **226.2** in the presence of iodine and propylene oxide gave the benzothiophene-fused corannulene derivative **226.3** in good yield. If benzo[*b*]thiophene-2-carboxaldehyde (**226.4**) was used in the first step, another corannulene derivative **226.5** bearing a fused dibenzothiophene unit could be obtained. According to the authors, this work was the first example of π-extension of the bowl-shaped corannulene nucleus via photocyclization, which served as an alternative to known methods such as metal-catalyzed cyclization and flash vacuum pyrolysis.

In 2021, Stuparu et al. reported the analogous photocyclization for the imine-containing corannulenes **226.7a**–**c**.^423^ These substrates were prepared from the Buchwald–Hartwig amination of bromocorannulene with the respective benzophenone imines **226.6a**–**c**. Subsequent photochemical cyclization of **226.7a**–**c** provided the isoquinoline-fused corannulenes **226.8a**–**c** in 71–99% yield. Both the electron-rich methoxy and electron-poor trifluoromethyl groups were tolerated in this reaction. Doubly fused corannulenes **226.9a**–**c** could also be formed from the appropriate precursors via the presumably more demanding 2-fold photocyclization in 44–99% yield. This result demonstrated the uniqueness of corannulene reactivity since the Mallory reaction is typically not applicable to *N-*aryl imines.

**Scheme 227:**
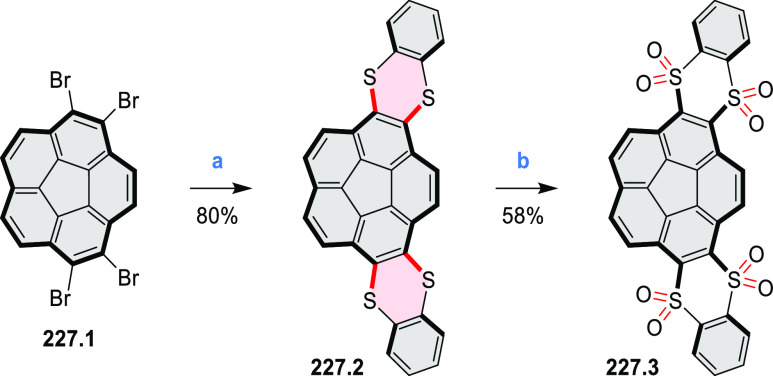
Synthesis of Doubly Fused Corannulene Tetrasulfone Reagents and conditions: (a)^424^ (1) benzene-1,2-dithiol, *t*-BuOK, DMF, rt, 1 h, (2) 60 °C, 21 h; (b) *m*-CPBA, DCM, rt, 48 h.

In 2019, a simple synthetic route to corannulene-based electron acceptors containing the sulfone functionality was presented by Stuparu et al.^424^ As shown in Scheme 227, 1,2,5,6-tetrabromocorannulene (**227.1**) was subjected to nucleophilic aromatic substitution by 1,2-benzenedithiol in the presence of a strong base. The corannulene derivative **227.2** bearing two *ortho*-fused benzodithiine units was formed in 80% yield. Upon oxidation by *m*-CPBA, compound **227.2** was converted to the corresponding tetrasulfone **227.3** in 58% yield. The reduction potentials of **227.3** and of the benchmark electron acceptor, PC_61_BM, were determined by square-wave and cyclic voltammetry. The electron affinities of the corannulene derivative **227.3** and PC_61_BM were found to be similar (0.9–1 V). The significant increase in electron-accepting capability of **227.3** relative to corannulene was attributed to the electron-withdrawing sulfone groups positioned in a rigid fused polycyclic framework.

**Scheme 228:**
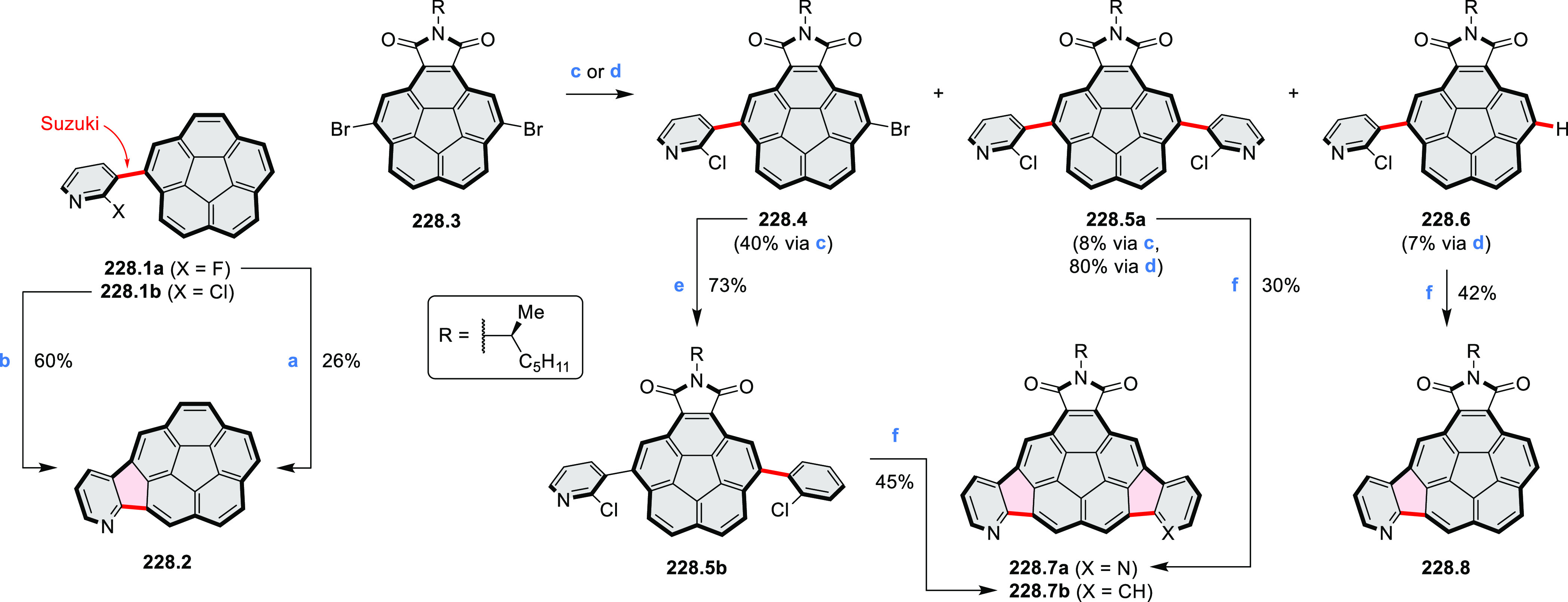
Synthesis of Azaindenocorannulenes Reagents and conditions: (a)^425^ [*i*-Pr_3_Si]^+^[CB_11_H_6_Cl_6_]^−^, (MeS)_2_SiMe_2_, chlorobenzene, microwave (225 W), 110 °C, 2 h; (b) Pd(PCy_3_)_2_Cl_2_, DBU, DMA, microwave (300 W), 160 °C, 0.5 h; (c)^426^ 2-chloropyridine-3-boronic acid (4 equiv), Pd(PPh_3_)_4_, K_2_CO_3_, THF/H_2_O (9/1), 100 °C, 1.5 h; (d) 2-chloropyridine-3-boronic acid (8 equiv), Pd(PPh_3_)_4_, K_2_CO_3_, THF/H_2_O (9/1), 100 °C, 1.5 h; (e) 2-chlorophenylboronic acid, Pd(PPh_3_)_4_, K_2_CO_3_, THF/H_2_O (9/1), 100 °C, 1.5 h; (f) Pd(PCy_3_)_2_Cl_2_, DBU, DMA, 160–180 °C, 6–7 h.

In 2018, Siegel and Baldridge et al. reported a synthetic approach toward azaindenocorannulene analogues bearing a pyridine unit.^425^ As shown in Scheme 228, 2-fluoro-3-pyridylcorannulene (**228.1a**) was subjected to silyl cation C–F activation/coupling to furnish the corresponding *peri*-fused corannulene **228.2** in 26% yield. The same product was obtainable, in 60% yield, from the palladium-catalyzed C–Cl activation/coupling of another substrate **228.1b**. In related work by the same group, three cyclization substrates **228.5a**,**b** and **228.6** were prepared from the enantiopure dibromocorannulene imide **228.3** by Suzuki–Miyaura cross-coupling.^426^ Their palladium-catalyzed ring closures successfully afforded **228.7a**,**b** and **228.8** in 30–45% yield. Each of these azaindenocorannulenes existed as two diastereomers with opposite bowl configurations. HPLC analysis of time-sequenced aliquots of **228.7b** revealed an inversion half-life of over 100 days (in diphenyl ether at 218 °C). The corresponding bowl inversion barrier exceeded 190 kcal mol^−1^, implying exceptional configurational stability.

**Scheme 229:**
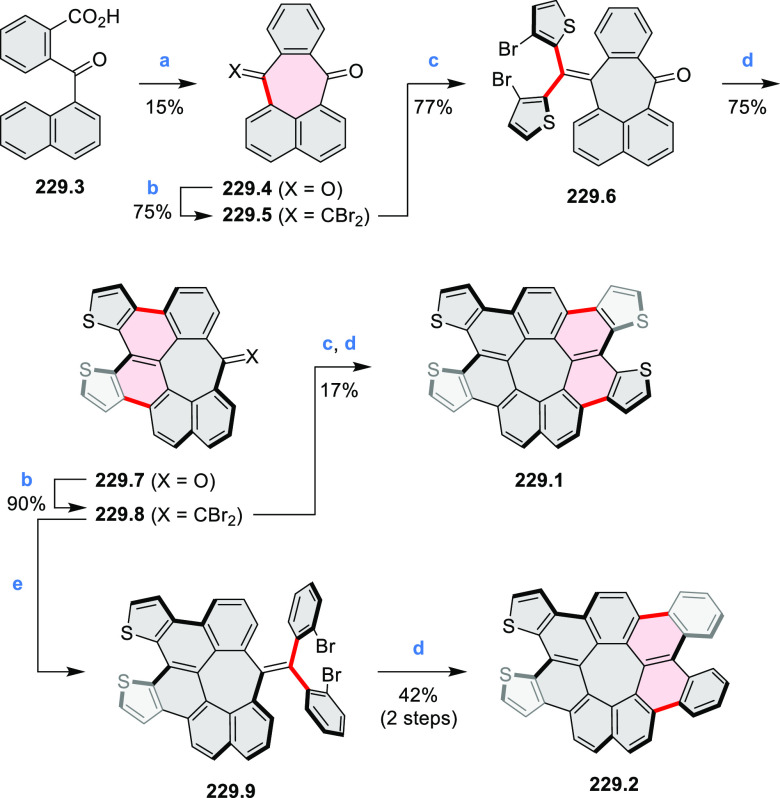
Preparation of 4-Fold Fused [7]Circulene Derivatives Bearing Four or Two Thieno Fusions Reagents and conditions: (a)^427^ NaCl, AlCl_3_, 150 °C, 0.5 h; (b) CBr_4_, PPh_3_, toluene, 80 °C, 24 h; (c) (3-bromothiophen-2-yl)boronic acid pinacol ester, Pd(PPh_3_)_4_, K_2_CO_3_, THF/H_2_O (10/1), reflux, 24 h; (d) Pd(PPh_3_)_4_, Cs_2_CO_3_, toluene, reflux, 24 h; (e) 2-bromophenylboronic acid, Pd(PPh_3_)_4_, K_2_CO_3_, THF/H_2_O (10/1), reflux, 24 h.

In 2017, Miao et al. reported the synthesis and properties of tetrabenzo[7]circulene.^427^ The authors demonstrated the flexibility of their synthetic route for accessing heterofused analogues with four and two benzo fusions replaced with thieno fusions, i.e., compounds **229.1** and **229.2**, respectively (Scheme 229). The synthesis began with the intramolecular Friedel–Crafts acylation of **229.3** to yield the diketone **229.4** bearing a seven-membered ring. On treatment with CBr_4_ and PPh_3_, one of the ketone groups in **229.4** was converted to the dibromovinylidene group in compound **229.5** in 75% yield. Surprisingly, the ketone group in **229.5** was inert to excess CBr_4_ and PPh_3_, presumably because of the steric blocking caused by the bromine atoms on the convex side of the molecule. Suzuki–Miyaura cross-coupling of the dibromide **229.5** allowed introduction of two 3-bromo-2-thienyl groups, leading to compound **229.6**. Finally, palladium-catalyzed C–H activation of **229.6** gave the doubly cyclized product **229.7** in 75% yield. The ketone group in **229.7** could smoothly react with CBr_4_ and PPh_3_ to yield the corresponding dibromovinylidene derivative **229.8** in 90% yield. A repetition of the previous cross-coupling–cyclization sequence on **229.8** offered tetrathieno[7]circulene **229.1** (17% yield over two steps). The same two-step sequence performed on **229.8**, using 2-bromophenylboronic acid as the alternative coupling partner, provided the unsymmetrical [7]circulene derivative **229.2** possessing two thieno and two benzo fusions (42% yield over two steps).

#### 6.1.2 [5]Heteracirculenoids

**Scheme 230:**
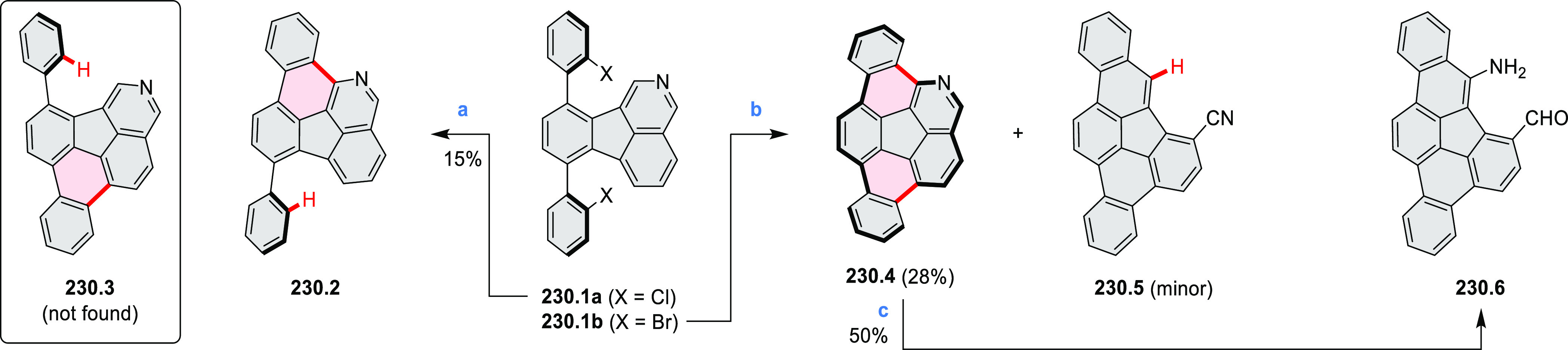
Attempted Synthesis of 5-Azadibenzo[*a*,*g*]corannulene by Solution-Phase Synthesis and Flash Vacuum Pyrolysis Reagents and conditions: (a)^428^ Pd(PCy_3_)_2_Cl_2_, DBU, DMA, 150 °C, 3 days; (b) FVP, 1000 °C; (c) silica gel, dry DCM, 24 h.

In 2017, Scott et al. reported a synthesis of the benzannulated azacorannulene derivative **230.4** (Scheme 230).^428^ They first prepared the bis(2-halophenyl)-substituted indenoisoquinoline derivatives **230.1a**,**b** using known synthetic methods. When the first substrate **230.1a** was heated with Pd(PCy_3_)_2_Cl_2_ and a strong base, single cyclization and C–Cl bond cleavage took place to give the product **230.2** in 15% yield. The cyclization was confirmed by ^1^H NMR spectroscopy to involve the pyridine nucleus. In other words, the singly cyclized isomer **230.3** was not found. For the second substrate **230.1b**, flash vacuum pyrolysis (FVP) at 1000 °C produced the desired doubly cyclized product, i.e., 5-azadibenzo[*a*,*g*]corannulene (**230.4**) in 28% yield. The nitrile **230.5** was observed as a minor product which presumably originated from the thermal opening of **230.4**. Furthermore, compound **230.4** underwent rapid hydrolysis in the presence of silica gel to yield the amino aldehyde **230.6**. Hence, the nitrogen atom in **230.4** was assumed to provide a reactive site for ring-opening reactions to release the inherent ring strain.

**Scheme 231:**
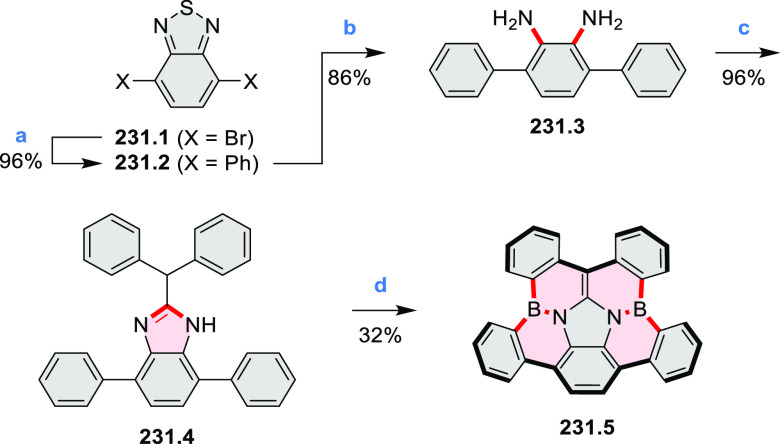
Gram-Scale, Four-Step Synthesis of B_2_N_2_ Corannulene Reagents and conditions: (a)^429^ phenylboronic acid, Pd(PPh_3_)_4_, K_2_CO_3_, THF/H_2_O (1:1), reflux, 3 days; (b) Zn, acetic acid, 40 °C, 22 h; (c) 2,2-diphenylacetic acid, 180 °C, 62 h; (d) BBr_3_, *o*-dichlorobenzene, reflux, 16 h.

A four-step synthesis reported in 2018 by Hatakeyama et al. enabled the preparation of a corannulene derivative **231.5** possessing B–N units on two of the “spoke” bonds (Scheme 231).^429^ First, the commercially available 4,7-dibromobenzo[*c*][1,2,5]thiadiazole (**231.1**) was doubly coupled with phenylboronic acid to give compound **231.2** in 96% yield. Second, sulfur extrusion of the thiadiazole ring in **231.3** using zinc metal provided the diamine **231.3** in 86% yield. Third, condensation of **231.3** with diphenylacetic acid furnished compound **231.4** bearing a new imidazole ring. In the final step, heating an *o*-dichlorobenzene solution of **231.4** with BBr_3_ at 200 °C effected the electrophilic C–H borylation to produce B_2_N_2_-containing corannulene **231.5** in 32% yield. The final product could be obtained in multigram quantities (3.6 g). According to X-ray data, **231.5** possessed a much shallower bowl depth (0.15 Å) than corannulene (0.87 Å). The authors ascribed this observation to the longer rim C–B bonds (1.54–1.58 Å) in **231.5** than the rim C–C bonds (1.45–1.47 Å) in corannulene. NICS calculations suggested the nonaromatic character of the central C_3_N_2_ ring and of all four C_4_BN rings, with NICS(0) values ranging from −2.4 to −0.4 ppm. Moreover, the BN units rendered compound **231.5** strongly blue fluorescent at 424 nm, with a 69% quantum yield. Using compound **231.5**, the authors reported the first fabrication of a corannulene-based OLED.

**Scheme 232:**
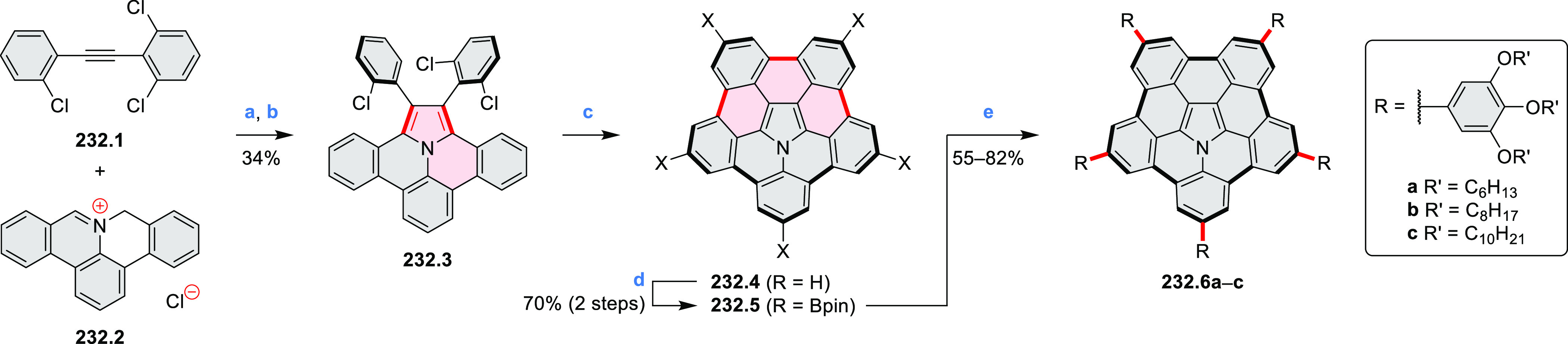
Regioselective Five-Fold C–H Functionalization of Azabuckybowl Reagents and conditions: (a)^430^ (a) *i*-Pr_2_NEt, DMSO, 100 °C, 9 h; (b) DDQ, DCM, rt, 5 min; (c) Pd(OAc)_2_, *t-*Bu_2_PMe·HBF_4_, DBU, DMA, 140 °C, 18 h; (d) B_2_pin_2_, [Ir(OMe)(cod)]_2_, 4,4′-di-*t*-butyl-2,2′-bipyridine, *t*-BuOK, THF, 85 °C, 10 days; (e) 1,2,3-trialkoxy-5-bromobenzene, Pd_2_(dba)_3_·CHCl_3_, SPhos, Cs_2_CO_3_, toluene/H_2_O (2/1), 80–85 °C, 3–5 days.

In 2018, Nozaki and Ito et al. reported the preparation and liquid-crystalline (LC) properties of 5-fold functionalized azapentabenzocorannulene derivatives.^430^ Using the methodology discussed earlier (see Scheme 221), compound **232.3** could be obtained in 34% yield. Compound **232.3** was then subjected to palladium-catalyzed 3-fold cyclization to give the unsubstituted azabuckybowl **232.4** (Scheme 232). Subsequently, the iridium-catalyzed 5-fold direct borylation of **232.4** furnished the 2,5,8,11,14-pentaborylated derivative **232.5** in 70% yield over two steps. Finally, compound **232.5** was submitted to Suzuki–Miyaura coupling with aryl bromides bearing long alkoxy substituents to yield the three pentaarylated products **232.6a**–**c** in 55–82% yield. XRD analyses of **232.6a**–**c** at 180–200 °C on cooling showed that these compounds form LC mesophases with hexagonally arranged columnar structures. The stability of these mesophases was attributed to the strong π–π stacking between the azapentabenzocorannulene cores. In the same year, Tokimaru, Ito, and Nozaki reported a conjugated azapentabenzocorannulene–corannulene hybrid **221.7** (see Scheme 221, Section 6.1.1).^419^

**Scheme 233:**
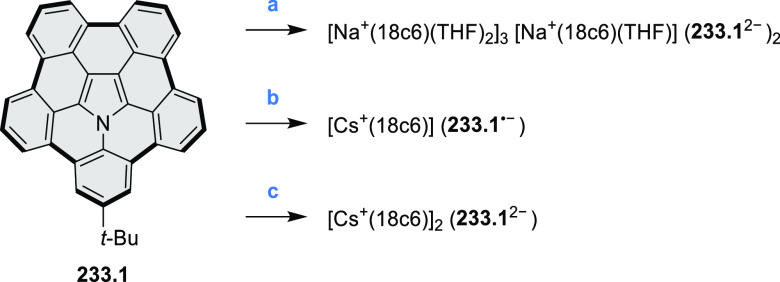
Chemical Reduction of an Azapentabenzocorannulene Derivative Reagents and conditions: (a)^431^ Na (8 equiv), 18-crown-6, THF, Ar, 25 °C, 48 h; (b) Cs (2 equiv), 18-crown-6, THF, Ar, 25 °C, 5 min; (c) Cs (5 equiv), 18-crown-6, THF, argon, 25 °C, 30 min.

In 2019, Petrukhina, Nozaki, and Ito et al. reported the chemical reduction of the known *tert*-butyl-substituted azapentabenzocorannulene **233.1** with sodium and cesium in THF in the presence of 18-crown-6 (18c6) (Scheme 233).^431^ The two-electron reduction of **233.1** with sodium metal gave the dianion **233.1**^2–^ which existed with [Na^+^(18c6)(THF)_2_] and [Na^+^(18c6)(THF)] in 2:3:1 stoichiometry in the crystal structure. The analogous reduction of **233.1** with varied amounts of cesium allowed isolation of both the radical anion and dianion salts. In the former salt [Cs^+^(18c6)](**233.1**^**•**^^–^), the 18c6-capped cesium ion was bound to the concave side of **233.1**^**•**^^–^. In the latter salt [Cs^+^(18c6)]_2_(**233.1**^2–^), two cesium ions coordinated to the concave and convex faces of **233.1**^2–^. In both cases, the binding of cesium ions took place asymmetrically at the six-membered rings of the azabuckybowl.

**Scheme 234:**
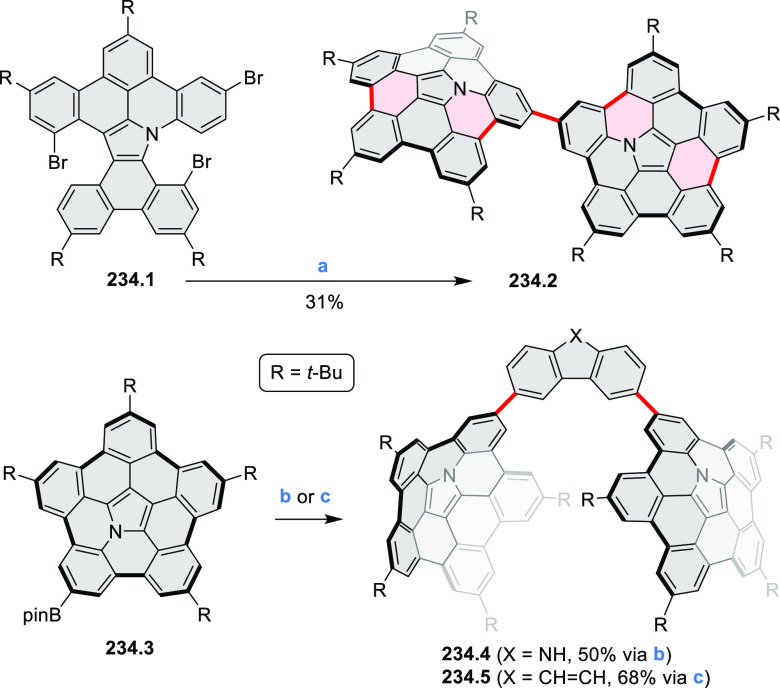
Bis(azapentabenzocorannulene)s Reagents and conditions: (a)^432^ Pd(OAc)_2_ (0.2 equiv), PCy_3_·HBF_4_ (0.4 equiv), K_2_CO_3_ (8 equiv), DMA, 130 °C, 29 h; (b)^433^ 3,6-dibromo-9*H*-carbazole, Pd(PPh_3_)_4_, Cs_2_CO_3_, THF/H_2_O (4/1), reflux, 4 h; (c) 3,6-diiodophenanthrene, Pd(PPh_3_)_4_, Cs_2_CO_3_, dioxane/H_2_O (20/3), 80 °C, 1 h.

In 2017, Shinokubo and Hiroto et al. reported the properties of azapentabenzocorannulene dimer **234.2** in which the two buckybowl units are linked through a C–C bond (Scheme 234).^432^ The synthesis involved subjecting the known polyaromatic precursor **234.1** to palladium-catalyzed intra- and intermolecular C–C bond formation using the catalyst system of Pd(OAc)_2_ (0.2 equiv) and PCy_3_·HBF_4_ (0.4 equiv). Compound **234.2** was formed in 31% yield and cocrystallized with C_60_ in 1:2 ratio. As revealed by XRD analysis, the two azabuckybowl units of **234.2** are oriented in opposite directions relative to the central C–C bond, while the concave face of each unit was coordinated to one C_60_ molecule. In solution, however, molecules of **234.2** were found to form a 1:1 complex with C_60_, while precipitation was observed at higher C_60_ concentrations. SEM images of the precipitate revealed the presence of fiber-like aggregates, resulting from the formation of one-dimensional chain-like supramolecular assemblies. The 1:1 binding ratio of **234.2** and C_60_ in the precipitate was established by variable-temperature ^1^H NMR spectroscopy using toluene-*d*_8_ as the solvent.

Shinokubo, Hiroto, and Kim et al. reported the synthesis and the host–guest chemistry of molecular tweezers bearing two azabuckybowl units connected by a carbazole or phenanthrene linker.^433^ The azabuckybowl derivative **234.3** bearing one boronate ester group was cross-coupled, in 1:2 ratio, with 3,6-dibromo-9*H*-carbazole or 3,6-diiodophenanthrene to furnish the tweezers **234.4** and **234.5**, respectively. Titration experiments with C_60_ and C_70_ as the guests were performed using fluorescence spectroscopy. For the carbazole-based tweezer **234.4** in toluene, the 1:1 association constant with C_60_ and C_70_ was 4.4 × 10^7^ M^–1^ and 7.0 × 10^8^ M^–1^, respectively. For the phenanthrene-based tweezer **234.5**, the corresponding values were 3.0 × 10^8^ (with C_60_) and 6.3 × 10^7^ M^–1^ (with C_70_), indicating an opposite binding preference. The cocrystal structure of **234.5** with C_60_ or with C_70_ indicated that, in each case, the fullerene molecule interacted with both azabuckybowl units of the same host molecule in a concave–convex manner.

**Scheme 235:**
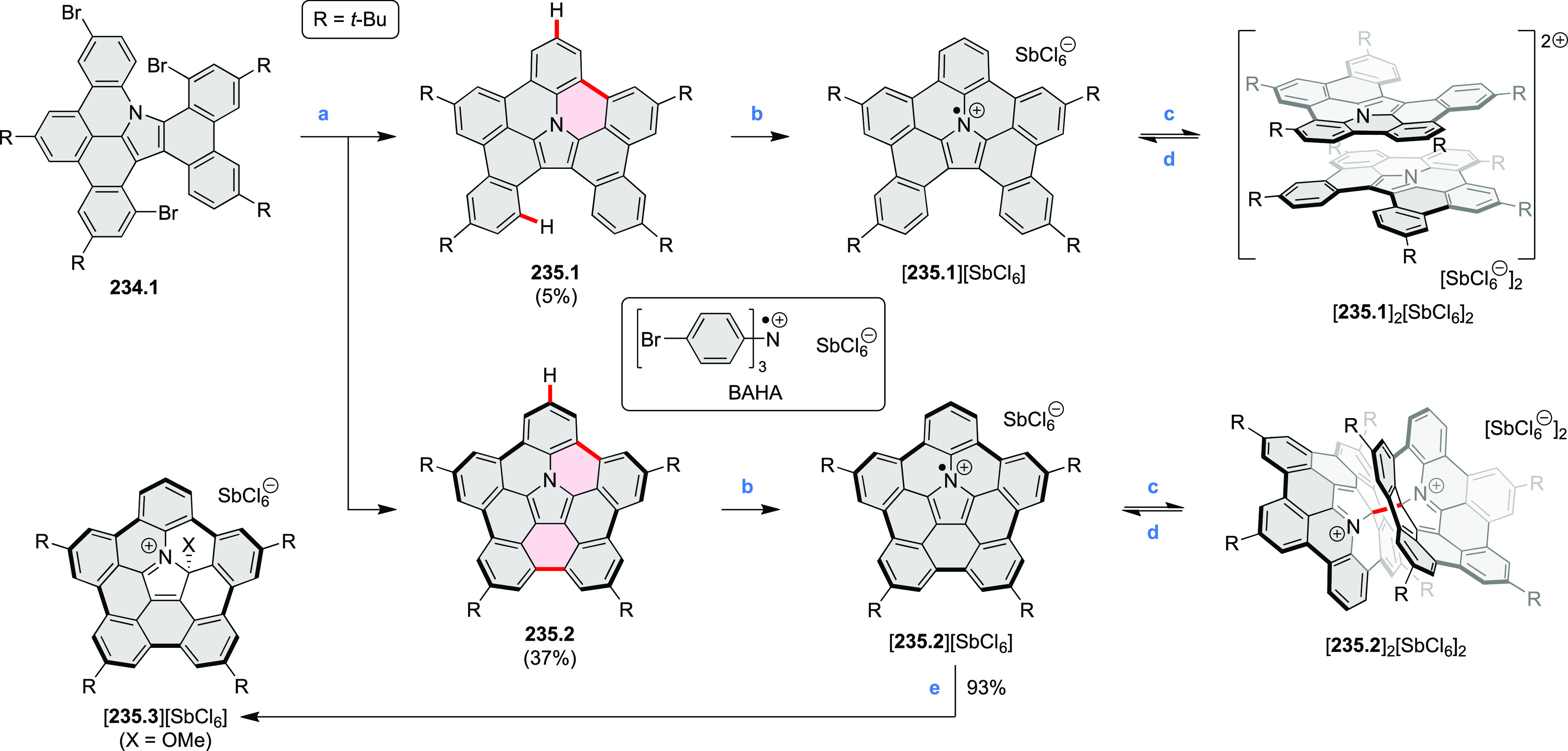
Synthesis and Reversible Dimerization of Azabuckybowl Derivatives Reagents and conditions: (a)^434^ Pd(OAc)_2_ (2 equiv), PCy_3_·HBF_4_ (4 equiv), K_2_CO_3_ (8 equiv), DMA, 130 °C, 32 h; (b) BAHA (1 equiv); (c) crystallization or cooling in solution; (d) heating in solution; (e) MeOH, DCM/toluene/Et_2_O (3:2:1) (for crystallization) or CDCl_3_ (for NMR).

In 2018, Yokoi, Hiroto, and Shinokubo described the reversible π- and σ-dimerization of azabuckybowl radical cations **235.1**^•+^ and **235.2**^•+^ (Scheme 235).^434^ Their synthesis involved subjecting the polyaromatic precursor **234.1** to the coupling conditions outlined in Scheme 234, but with excess amounts of the catalyst Pd(OAc)_2_ (2 equiv) and ligand source PCy_3_·HBF_4_ (4 equiv). In this way, the singly cyclized product **235.1** with two reductively cleaved C–Br bonds was obtained in 5% yield, in addition to the known azabuckybowl **235.2** (37%). Compound **235.1** was revealed by X-ray crystallography to adopt an essentially planar conformation. Both the planar and bowl-shaped compounds were subjected to one-electron oxidation by BAHA, generating the corresponding radical cations **235.1**^•+^ and **235.2**^•+^, which were characterized by X-ray crystallography as their SbCl_6_^–^ salts. In the former salt, a dimeric structure [**235.1**]_2_^2+^ was observed, in which two planar motifs were stacked face-to-face. The closest interplanar distance found between two α-carbon atoms of the individual pyrrole units was 3.14 Å. In the other salt, a dimeric structure [**235.2**]_2_^2+^ was observed, in which two bowl-shaped motifs were linked in a convex–convex fashion. A uniquely long C–C bond (1.64 Å) connected the two subunits via α-positions of their respective pyrrole rings. The reversible dimerization behavior of **235.1**^•+^ and **235.2**^•+^ was also probed by variable-temperature NMR, ESR, and UV–vis–NIR absorption experiments. The bowl-shaped radical cation **235.2**^•+^ could react with excess methanol to yield the methoxylated cation **235.3**^+^. However, no such conversion was observable with the planar radical cation **235.1**^•+^.

#### 6.1.3 [6]Heteracirculenoids

In two papers, Xu and Tan et al. reported the synthesis of unsubstituted trichalcogenasumanenes (**236.1a**–**c**) from 1,5,9-trisubstituted triphenylene precursors. Their approach used the triiodonium cation [**236.3**]^3+^ as the common precursor. Oxidation of 1,5,9-triiodotriphenylene (**236.2**) by *m*-CPBA in the presence of triflic acid generated the triiodonium salt [**236.3**][OTf]_3_ in 68% yield. The identity of the salt was substantiated by ^1^H, ^13^C, and ^19^F NMR spectroscopy and by MALDI–TOF mass spectrometry.^435^ Using potassium thioacetate or sulfur powder as the sulfur source and appropriate conditions, [**236.3**][OTf]_3_ could afford the sulfur-containing derivatives **236.4** or **236.5** in fair yields. Both these compounds could then be desulfurized by heating with copper powder in tetralin at 200 °C to furnish trithiasumanene (**236.1a**). Likewise, [**236.3**][OTf]_3_ was subjected to selenation with selenium powder followed by copper-mediated deselenation to give triselenasumanene (**236.1b**) in 22% overall yield. In subsequent work, tritellurasumanene (**236.1c**) was directly formed from [**236.3**][OTf]_3_, by heating with tellurium powder in the presence of 2-picoline as the base.^436^ Additionally, the synthetic versatility of the triiodonium salt was demonstrated by Xu and Tan et al. in the syntheses of the trisilasumanenes **236.6a**–**c** and the trigermasumanene **236.7** involving rhodium-catalyzed cyclodehydrogenation of Si/Ge–H and C–H bonds.^437^

**Scheme 236:**
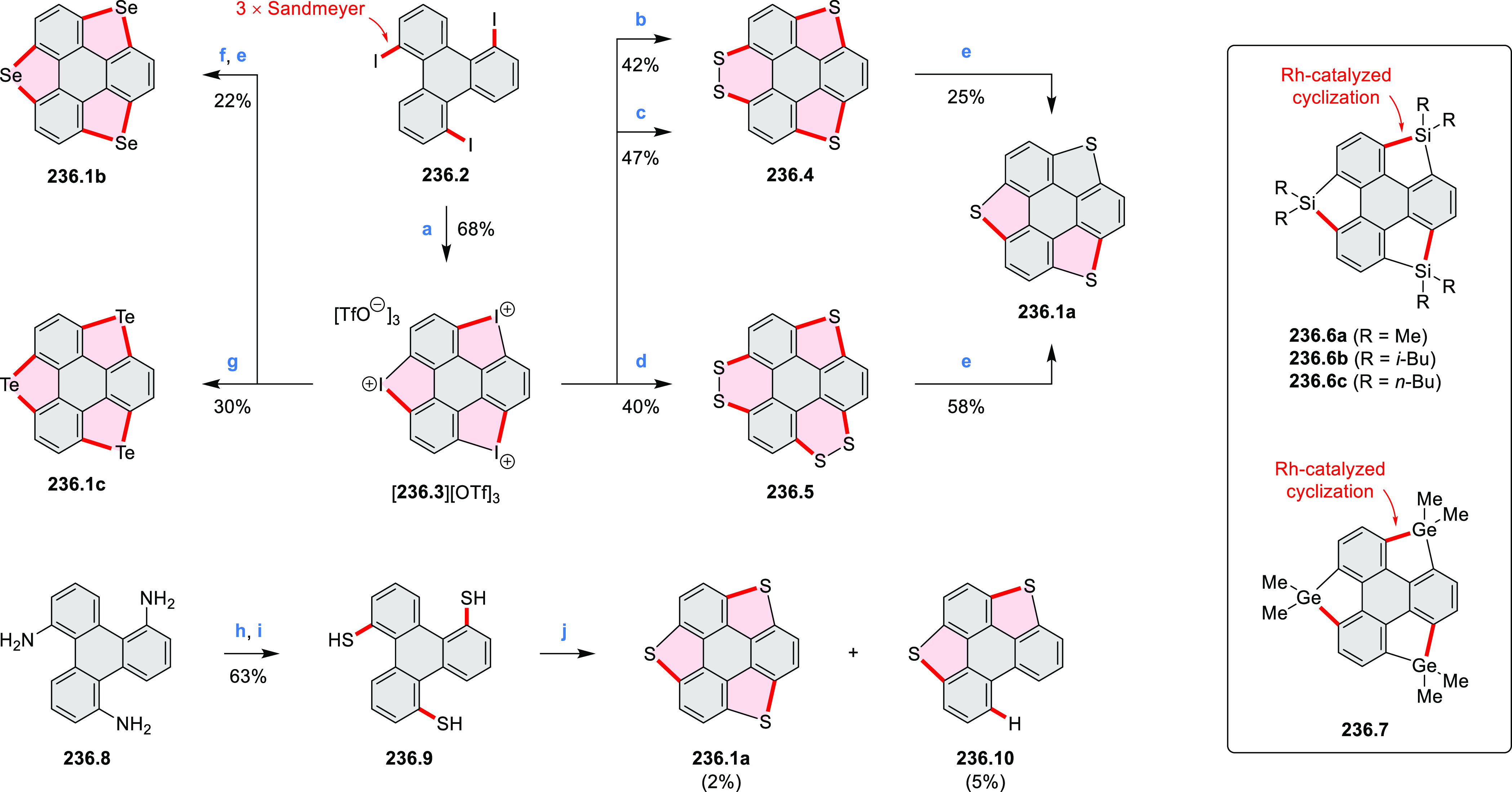
Syntheses of Unsubstituted Trichalcogenasumanenes from Triphenylene-Based Precursors Reagents and conditions: (a)^435^*m*-CPBA, TfOH, DCM, 0 °C to rt, 12 h; (b) AcSK, CuCl_2_, DMSO, 110 °C, 48 h; (c) S_8_ powder, Cs_2_CO_3_, DMSO, 140 °C, 12 h; (d) S_8_ powder, Cs_2_CO_3_, DMSO, 80 °C, 12 h; (e) Cu, tetralin, 200 °C, 1–2 h; (f) Se powder, *t*-BuOK, DMSO, 70 °C, 24 h; (g)^436^ Te powder, 2-picoline, DMSO, 125 °C, 12 h; (h)^438^ (1) HCl, NaNO_2_, H_2_O, 0 °C, (2) KSCN, FeCl_3_, 0 °C, then rt, 1 h; (i) Na_2_S·9H_2_O, EtOH/H_2_O (2/1), reflux, overnight; (j) PdCl_2_ (90 mol %), DMSO, 120 °C, 24 h.

In 2018, Xu and Tan et al. described the oxidative cyclization of 2-biphenylthiols into the corresponding dibenzothiophenes, which was achieved with PdCl_2_ as the catalyst and DMSO as both the solvent and oxidant.^438^ This set of conditions was also applicable to the 3-fold cyclization of triphenylene-1,5,9-trithiol (**236.9**) to yield trithiasumanene (**236.1a**, 2%) alongside the incompletely fused **236.10** (5%, Scheme 236). The authors ascribed the low yield of **236.1a** to the instability of the molecule under the reaction conditions. Despite the low yield, this preparation represented the first trithiasumanene synthesis using a direct “stitching” method, as opposed to desulfurative ring contractions (Schemes 236 and 240).

**Scheme 237:**
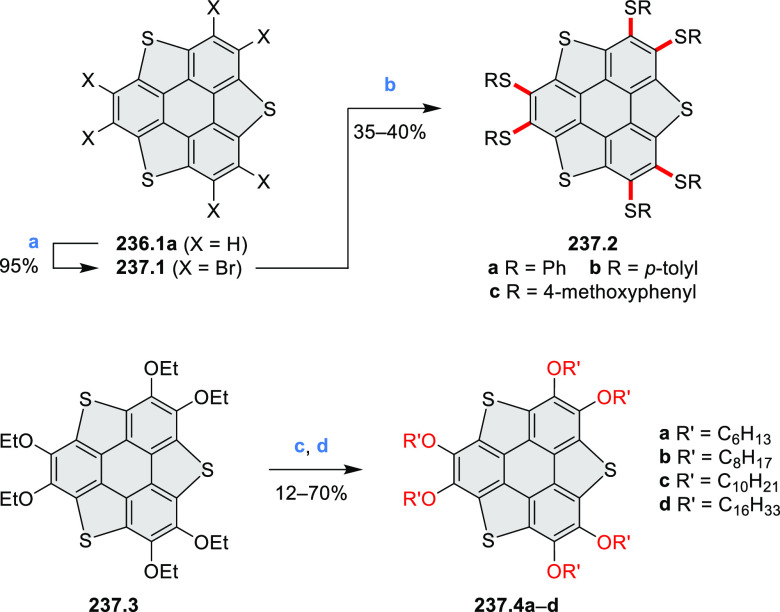
Six-Fold Functionalized Trithiasumanenes Reagents and conditions: (a)^439^ Br_2_, Fe, nitrobenzene, 80 °C, 2 days; (b) RSNa, dry 1,3-dimethyl-2-imidazolidinone, rt, 8 h; (c)^440^ BBr_3_, DCM, 0 °C to rt, then H_2_O; (d) R′Br, K_2_CO_3_, DMF, 75 °C.

Hexabromination of trithiasumanene with elemental bromine in the presence of iron powder was shown by Xie and Tan et al. to give **237.1** in high yield (Scheme 237).^439^ Subsequent 6-fold nucleophilic aromatic substitution of this compound with aryl thiolates furnished the sulfur-rich products **237.2a**–**c** in 35–40% yield. The hexakis(phenylthiol)-substituted derivative **237.2a** formed cocrystals with C_60_ and with C_70_, which were analyzed by X-ray crystallography. In the solid state, both fullerenes coordinated to the concave face of the buckybowl **237.2a** in a 1:1 stoichiometry. The six phenyl groups in **237.2a** were found to direct away from C_60_ and C_70_, lacking any interaction with the fullerene molecule in both crystal structures. The bowl depths observed in both cocrystals were 0.76 Å, in contrast to the deeper bowl depth (0.83 Å) found in the crystal of pure **237.2a**. The bowl shallowing upon complexation with fullerenes was believed to strengthen the intermolecular interaction.

In a recent report, Furukawa, Akutagawa, and Saito et al. demonstrated a new method for generation of ferroelectricity based on bowl-to-bowl inversion of trithiasumanene.^440^ The attendant dipole inversion was envisioned to induce ferroelectric response in the solid state because of the relatively low inversion barrier compared to corannulene and sumanene. Hexaalkoxytrithiasumanenes **237.4a**–**d** were prepared by applying a dealkylation–realkylation procedure to the ethoxy-substituted precursor **237.3**. The alkoxy chains were installed to enhance the flexibility and internal thermal energy of the columnar assemblies upon heating. The temperature and frequency dependence of the dielectric constants of **237.4a**–**d** was examined, alongside their ferroelectric polarization–electric field (*P*–*E*) hysteresis curves. The π-stacked assemblies of **237.4a**–**d** underwent polarization upon application of a pulse voltage, followed by a dipole relaxation pathway through bowl-to-bowl inversion in bulk.

**Scheme 238:**
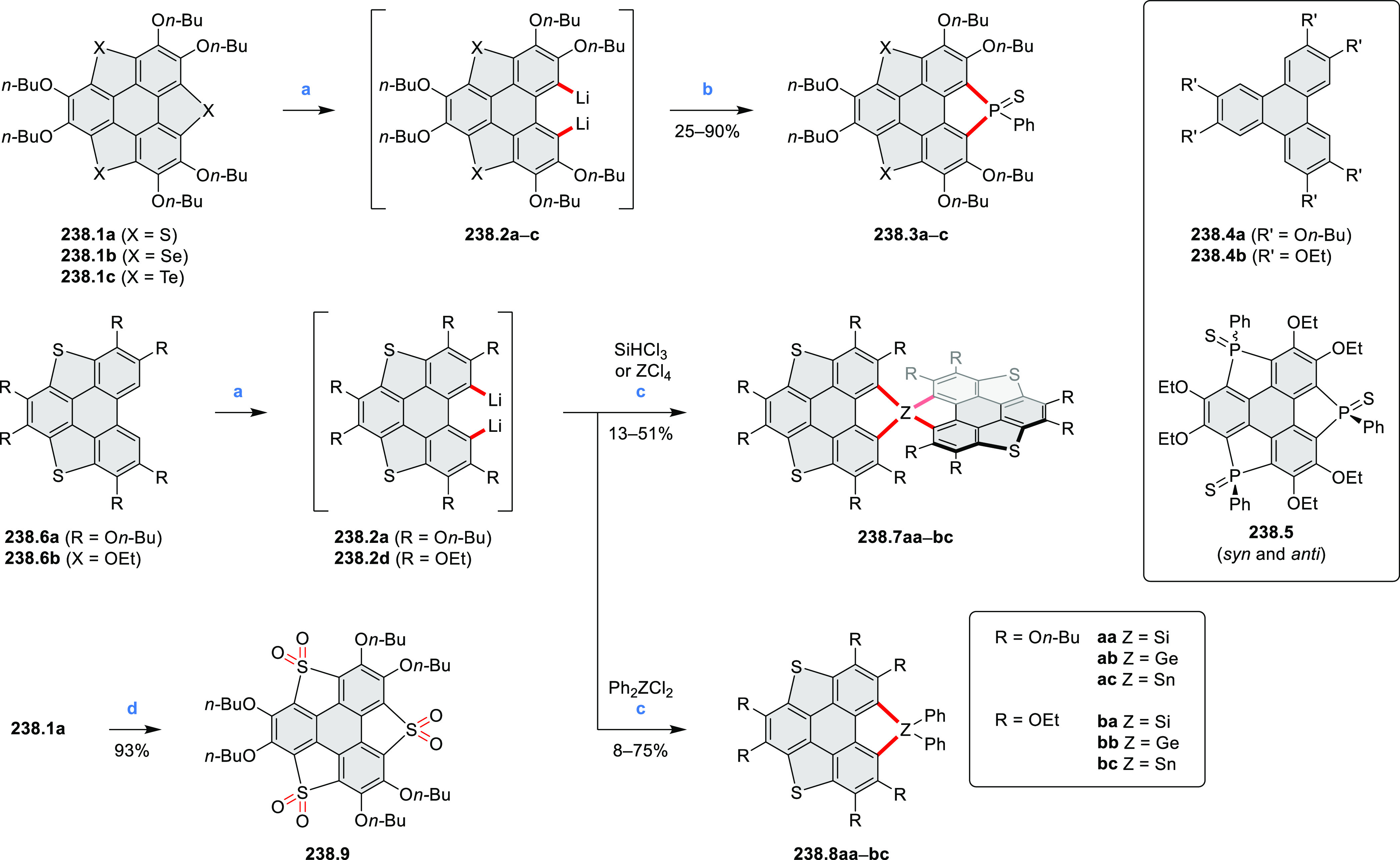
Ring-Modified Trichalcogenasumanenes Reagents and conditions: (a)^441,442^*n*-BuLi (2.2 equiv), TMEDA, hexane, −30, 60 °C or reflux, 30 min–3 h; (b) (1) PhPCl_2_, THF, −78 °C to rt, 3–4 h, (2) S powder, rt, 12.5–14 h; (c)^443^ THF, −78 °C to rt, then reflux, 12 h; (d)^444^ 30% aq. H_2_O_2_, DCM/AcOH (1/2), 60 °C, 24 h.

In 2017, Saito and Fuji et al. reported the formation of the phosphole sulfides **238.5** from the triphenylene precursor **238.4b**.^441^ Based on this method, Shao et al. proposed a general route to replace one chalcogen atom in the hexabutoxy-substituted heterasumanenes **238.1a**–**c** with a phosphorus(V) group (Scheme 238).^442^ For instance, the opening of one thiophene ring in the trithiasumanene (**238.1a**) with 2.2 equiv of *n*-BuLi generated the dilithiated intermediate **238.2a**. Sequential treatment of **238.2a** with excess PhPCl_2_ and excess sulfur powder gave rise to compound **238.3a** bearing one PhP=S group in 25% yield. This one-pot protocol was also feasible for the syntheses of the selenium-based and tellurium-based congeners, i.e., **238.3b** and **238.3c**, in 41% and 90% yield, respectively. For all three phosphorus(V)-containing sumanenes **238.3a**–**c**, the cocrystal structures with AgNO_3_ indicated coordination between Ag^+^ and the sulfur atom on the P=S functionality. The thiophene-containing derivative **238.3a** was shown to be a potential sensitive fluorescence sensor for Ag^+^, with a limit of detection as low as 0.21 μM, which is superior to the World Health Organization standard for drinking water (0.5 μM).

In 2018, Furukawa and Saito et al. reported the incorporation of group 14 elements (silicon, germanium, and tin) into the dithiasumanene structure.^443^ Compounds **238.6a**,**b** were initially treated with *n*-BuLi, and the resultant dilithiated species **238.2a**,**d** were reacted with SiHCl_3_, SiCl_4_, GeCl_4_, or SnCl_4_ to furnish the corresponding spirocyclic structures **238.7aa**–**bc** with Si, Ge, or Sn located at the spiro junction. Alternatively, the reaction between **238.2a**,**d** and Ph_2_ZCl_2_ (Z = Si, Ge, or Sn) afforded the corresponding heterasumanenes **238.8aa**–**bc**. In the solid state, the dihedral angle between sumanene subunits in **238.7ba**–**bc** ranged from 84.7° to 88.9°. As shown by CV measurements, the monomeric heterasumanenes **238.8ba**–**bc** displayed only one quasi-reversible anodic wave at *E*_1/2_ = 0.40–0.41 V, while the spirocyclic analogues **238.7ba**–**bc** showed two reversible anodic waves at *E*_1/2_ = 0.40–0.41 and 0.54–0.55 V. The inferred stability of the radical cations of **238.7ba**–**bc** was attributed to the stabilizing spiroconjugation between the two subunits. Such spiroconjugative effects were corroborated by the orbital shapes of HOMO and HOMO–1 obtained from DFT calculations.

In 2017, Shao et al. reported the oxidation of hexabutoxytrithiasumanene (**238.1a**) to the corresponding tris(*S*,*S*-dioxide) **238.9** (Scheme 238).^444^ The reaction worked when excess hydrogen peroxide was used as the oxidant, whereas the use of *m*-CPBA for the same conversion proved ineffective. Compound **238.9** displayed strong indigo fluorescence in DCM solution (λ_em_ = 463 nm) and in the solid state (λ_em_ = 478 nm). The 1:1 cocrystal of **238.9** with 2,3,6,7,10,11-hexabutoxytriphenylene (**238.4a**) showed strong yellow fluorescence in the range of 500–700 nm.

**Scheme 239:**
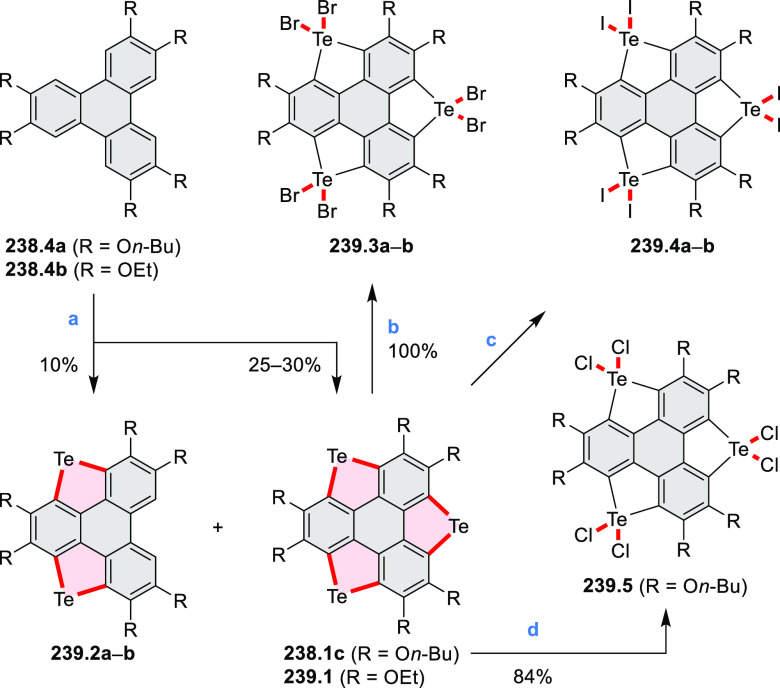
Gram-Scale, One-Pot Synthesis of a Tritellurasumanene Derivative and Its Subsequent Halogenation Reagents and conditions: (a)^445^ (1) *n*-BuLi, TMEDA, hexane, 60 °C, 3 h, (2) Te powder, ultrasound, rt, 12 h; (b) Br_2_, DCM, rt, 15 min; (c) I_2_ (yield not given); (d)^446^ FeCl_3_, DCM, MeNO_2_, rt, 2 h.

The preparation of the hexabutoxy-substituted tritellurasumanene **238.1c** mentioned above, as well as another analogue **239.1**, was described by Shao et al. earlier in 2016 (Scheme 239).^445^ Hexaalkoxytriphenylenes **238.4a**,**b** were separately hexalithiated using *n*-BuLi and subjected to ultrasound-assisted reaction with tellurium powder to afford the triply tellurated products **238.1c** and **239.1**, and the doubly tellurated products **239.2a**,**b**. These successful conversions confirmed that ultrasound could promote the surface contact between tellurium powder, which dissolves poorly in hexane, with the solvated hexalithiated intermediate. Moreover, both tritellurasumanenes could react with elemental bromine to yield the tris(*Te*,*Te*-dibromide)s **239.3a**,**b** quantitatively. Likewise, the reaction with iodine generated the tris(*Te*,*Te*-diiodide)s **239.4a**,**b** as supported by ^1^H and ^13^C NMR spectroscopy. In a later paper by the same group, the reaction of **238.1c** with FeCl_3_ in DCM/MeNO_2_ produced the tris(*Te*,*Te*-dichloride) **239.5** in 84% yield.^446^ Similar halogenations were not feasible with the trithia- and triselenasumanenes **238.1a**,**b**. Besides, the known oxone-mediated oxidative ring opening of the benzene unit in **238.1a**,**b** did not take place for the tritellurasumanene **238.1c** as inferred from the absence of any carbonyl stretch in the IR spectrum of the product mixture.

**Scheme 240:**
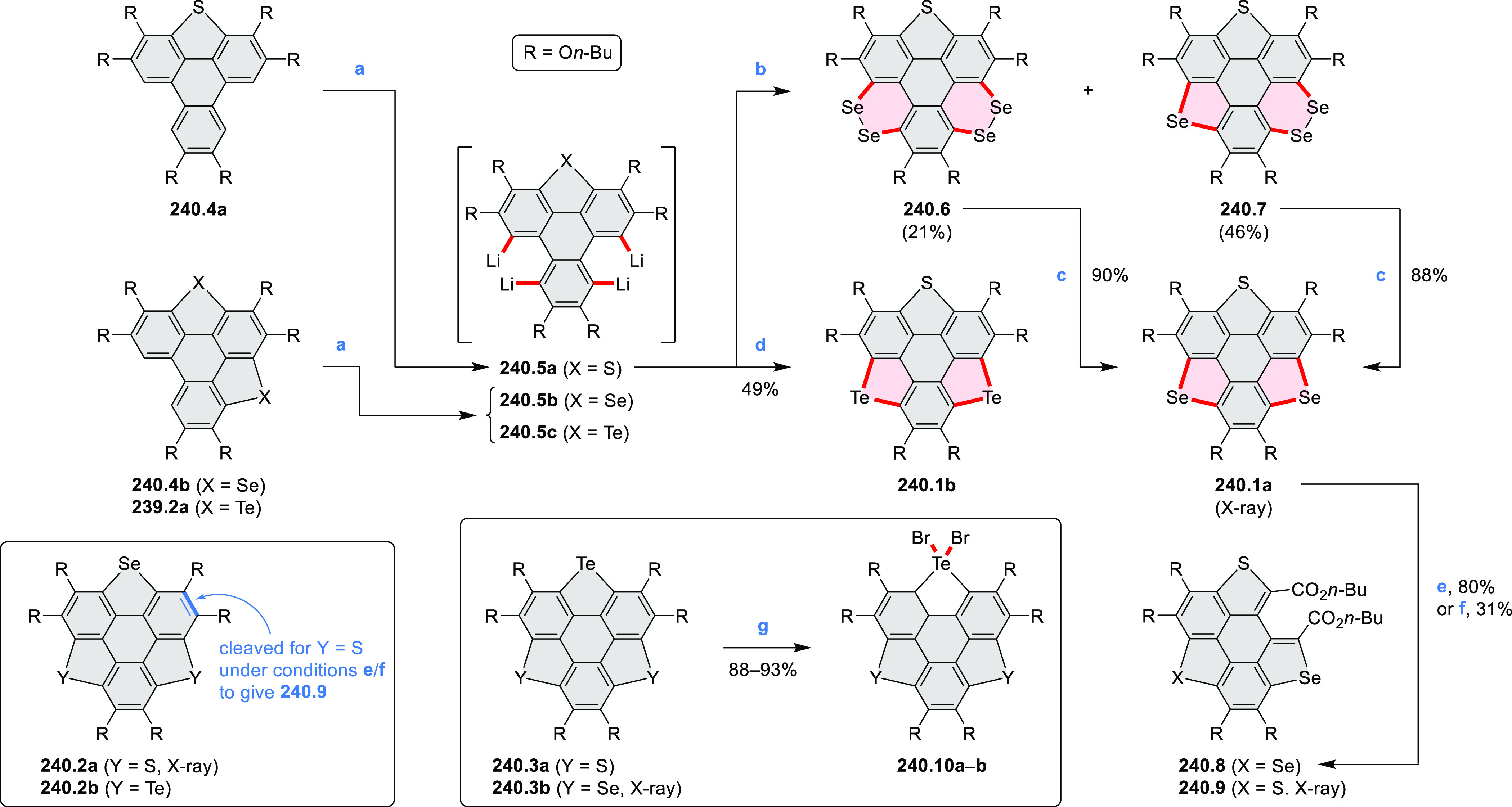
Synthesis and Reactivity of Trichalcogenasumanene Derivatives Bearing Two Different Chalcogens Reagents and conditions: (a)^447^*n*-BuLi (4, 5, or 10 equiv), TMEDA, hexane, 60 °C, 3 h; (b) Se powder (10 equiv), THF, −78 °C to rt, 8 h; (c) Cu nanopowder, 215 °C, 160 min; (d) Te powder (10 equiv), ultrasound, rt, 12 h; (e) oxone, THF/H_2_O (4/1), rt, 12 h; (f) 30% aq. H_2_O_2_, DCM/AcOH (5/1), 24 h; (g) Br_2_, DCM, rt, 15 min.

Shao et al. reported the systematic preparation of a series of six trichalcogenasumanenes **223.1a**–**3b**, each bearing two different kinds of chalcogen atoms (Scheme 240).^447^ For the preparation of the two monothia analogues, compound **240.4a** was deprotonated by excess *n*-BuLi to generate the tetralithiated species **240.5a**. A subsequent selenation–deselenation sequence successfully produced the S_1_Se_2_-sumanene derivative **240.1a**. Similarly, the ultrasound-assisted reaction between **240.5a** and tellurium powder directly afforded the S_1_Te_2_-sumanene derivative **240.1b** in 49% yield. The preparation of the monoselena (**240.2a**,**b**) and monotellura (**240.3a**,**b**) analogues employed **240.2b** and **239.2a** as the respective starting materials. According to X-ray diffraction data, the bowl depths of the S_1_Se_2_- (**240.1a**, 0.49 Å) and S_2_Se_1_-analogues (**240.2a**, 0.42 Å) were shallower than that of the S_3_-analogue (**238.1a**, 0.77 Å), whereas the Se_1_Te_2_-analogue (**240.2b**) assumed a planar geometry. Using either oxone or 30% H_2_O_2_ as the oxidant, the oxidative C–C bond cleavage of the S_1_Se_2_- and S_2_Se_1_-analogues occurred regioselectively at the benzene ring between thiophene and selenophene to yield the ring-opened diesters **240.8**–**9**. Exposure of both the S_2_Te- and Se_2_Te-analogues to elemental bromine resulted in 2-fold bromination at the tellurium atom, furnishing compounds **240.10a**,**b** (see Scheme 239).

**Scheme 241:**
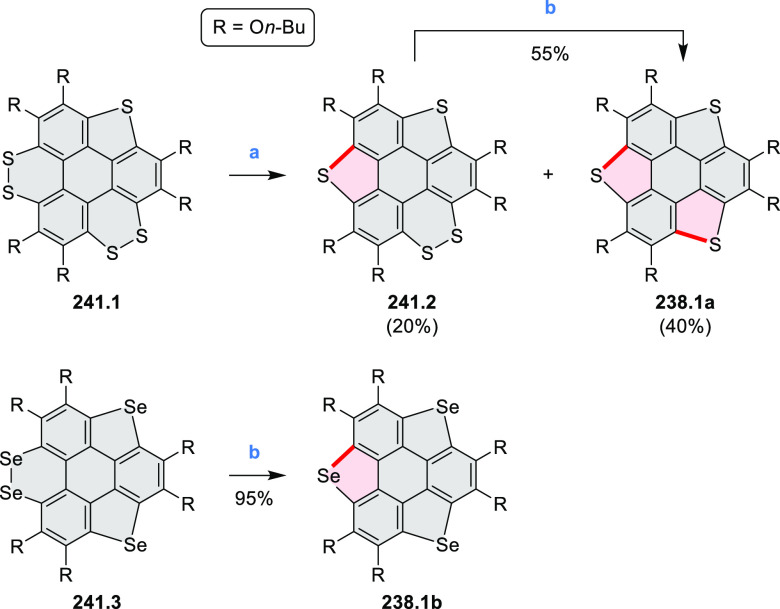
Trithiasumanene and Triselenasumanene Derivatives via Copper-Catalyzed Dechalcogenization Reagents and conditions: (a)^448^ CuI (40 mol %), 1,10-phenanthroline (80 mol %), K_2_CO_3_ (6 equiv), DMSO/H_2_O (20:1), air, 160 °C; (b) CuI (20 mol %), 1,10-phenanthroline (40 mol %), K_2_CO_3_ (3 equiv), DMSO/H_2_O (20:1), air, 160 °C.

In 2020, Wang and Shi et al. reported the copper-catalyzed dechalcogenization of aryl dichalcogenides to the corresponding aryl chalcogenides.^448^ This reaction required substoichiometric amounts of CuI as the catalyst and 1,10-phenanthroline as the ligand and involved heating in a solvent mixture of DMSO and water at 160 °C. The reaction had a broad substrate scope and was applied to the preparation of trithia- and triselenasumanene derivatives (Scheme 241). First, compound **241.1** containing two cyclic disulfide units was converted into the singly and doubly desulfurized products, i.e., **241.2** and **238.1a**, in 20% and 40% yield, respectively. Similarly, the single cyclic diselenide unit in compound **241.3** was smoothly deselenated to give the **238.1b** in 95% yield. These conditions were significantly milder than those used previously (see Schemes 236 and 240).

**Scheme 242:**
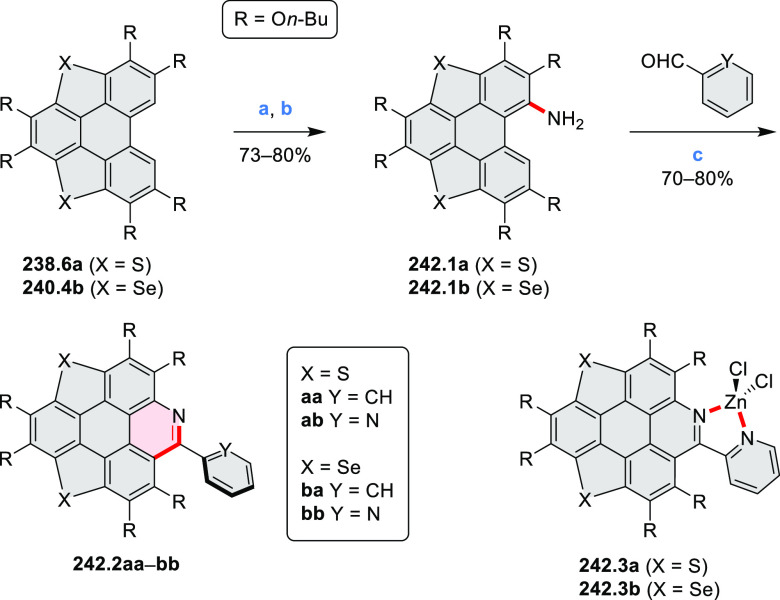
[6]Heteracirculenoids Bearing Chalcogenole and Pyridine Rings Reagents and conditions: (a)^449^*t*-BuONO, DCM, rt, 4 h; (b) Zn, AcOH/EtOH, 0 °C, 10 min, then rt, 3 h; (c) TFA, 120 °C, 12 h.

In 2018, Shao et al. described the [6]heteracirculenoids **242.2aa**–**bb** possessing two chalcogenole rings and one pyridine ring that are fused with the central benzene ring (Scheme 242).^449^ These compounds represent hybrids of trichalcogenasumanene and triazacoronene. The triphenylenodithiophene **238.6a** and triphenylenodiselenophene **240.4b** were separately subjected to a nitration–reduction sequence to give the amines **242.1a**,**b** in 73–80% yield. Subsequent Pictet–Spengler reaction with benzaldehyde or picolinaldehyde afforded the four target molecules **242.2aa**–**bb** in 70–80% yield. The protonation-dependent optical properties of the two sulfur-containing compounds **242.2aa**–**ab** could be modulated by addition of trifluoroacetic acid and triethylamine. The molecular frameworks of the two pyridyl-substituted analogues **242.2ab**,**bb** were planar in the solid state. However, the structures became bowl-shaped in the complexes **242.3a**,**b**, in which the 2,2′-bipyridine moiety was bound to a Zn^2+^ ion.

**Scheme 243:**
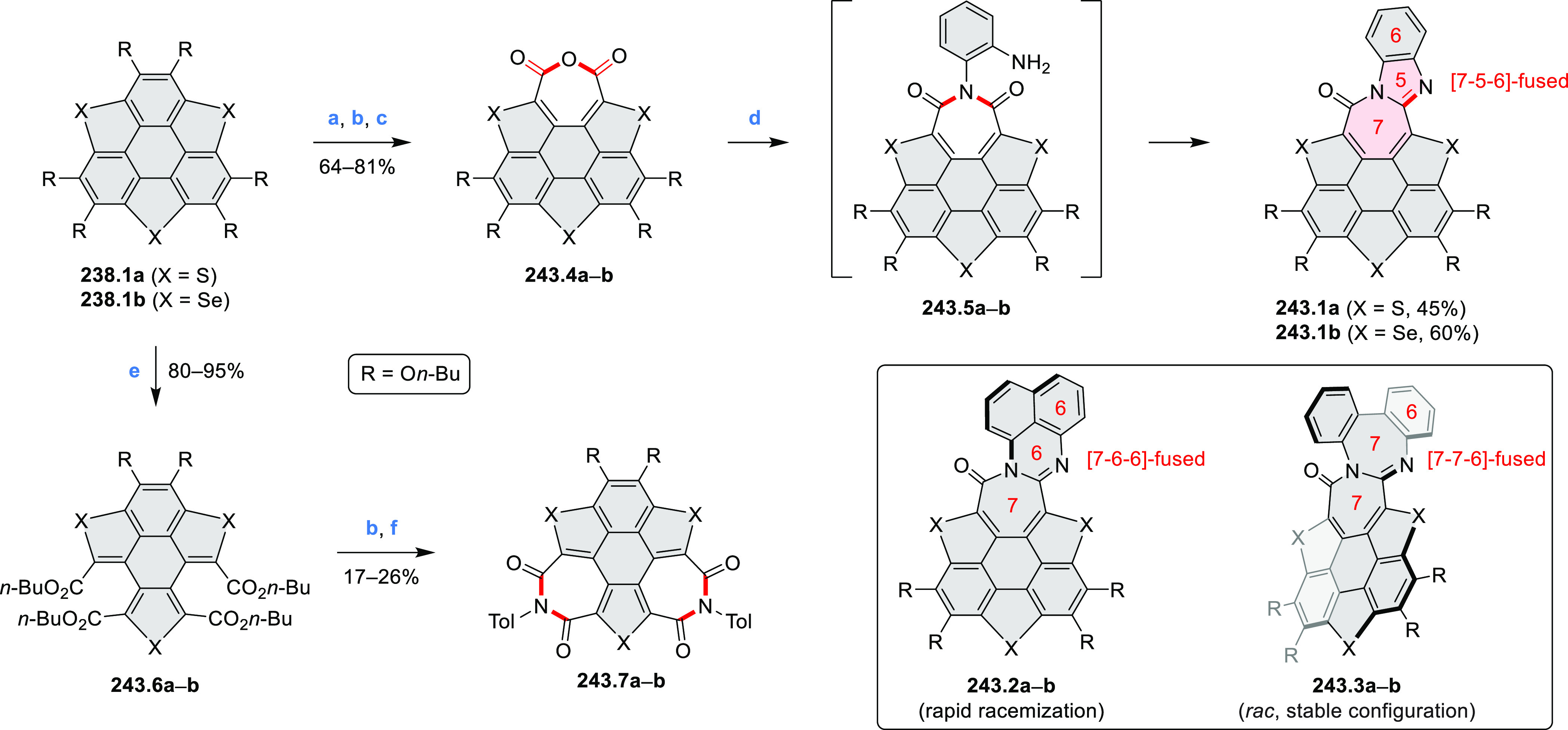
Oxidative Ring Opening of Trichalcogenasumanenes Reagents and conditions: (a)^450^ oxone, THF/H_2_O (4:1), rt, 2 h; (b) (1) NaOH, EtOH/H_2_O (10:1), reflux, 2 h, (2) aq. HCl (3 N); (c) Ac_2_O, reflux; (d) *o*-phenylenediamine, DCC, THF, reflux, 8 h; (e)^451^*t*-BuOOH (6 equiv), DCM, 40 °C, 5 h; (f) (1) *p*-toluidine, DCC, THF, reflux, 12 h.

In 2017, Shao et al. obtained a series of trichalcogenasumanene-derived amidine-containing polycycles **243.1**–**3** (Scheme 243).^450^ In the synthetic route, the trithia- and triselenasumanenes **238.1a**,**b** first underwent oxone-induced benzene ring opening to give the corresponding diesters, followed by base-promoted hydrolysis and dehydration reactions, yielding the seven-membered cyclic anhydrides **243.4a**,**b**. These intermediates were then condensed with various aromatic diamines in the presence of DCC to give the target polycycles. For instance, the double condensation with *o*-phenylenediamine led to the [7–5–6]-fused polycycles **243.1a**,**b**. Analogously, the [7–5–6]-fused (**243.2a**,**b**) and [7–7–6]-fused polycycles (**243.3a**,**b**) could be obtained using the suitable diamines. Among these three fusion types, only the last two displayed twisted molecular geometries in their X-ray crystal structures. The enantiomers of the highly twisted compound **243.3b** were separated by chiral HPLC and were found to be configurationally stable. Thin films of **243.2a**,**b** exhibited hole mobility (μ_h_) of 6 × 10^–4^ cm^2^ V^–1^ s^–1^, while those of **243.1a**,**b** and **243.3a**,**b** were poor semiconductors. Single crystals of **243.2a** could be fabricated into an OFET device with a p-type semiconducting behavior. In 2018, Shao and Sun et al. reported the use of *tert*-butyl hydroperoxide for the oxidative ring opening of two benzene rings in trithia- and triselenasumanenes (**238.1a**,**b**) to give the tetraesters **243.6a**,**b**, which could be converted to the cyclic diimides **243.7a**,**b**.^451^ Based on open-aperture Z-scan measurements, the authors concluded that **243.7a**,**b** were potentially superior to C_60_ as optical-limiting materials.

**Scheme 244:**
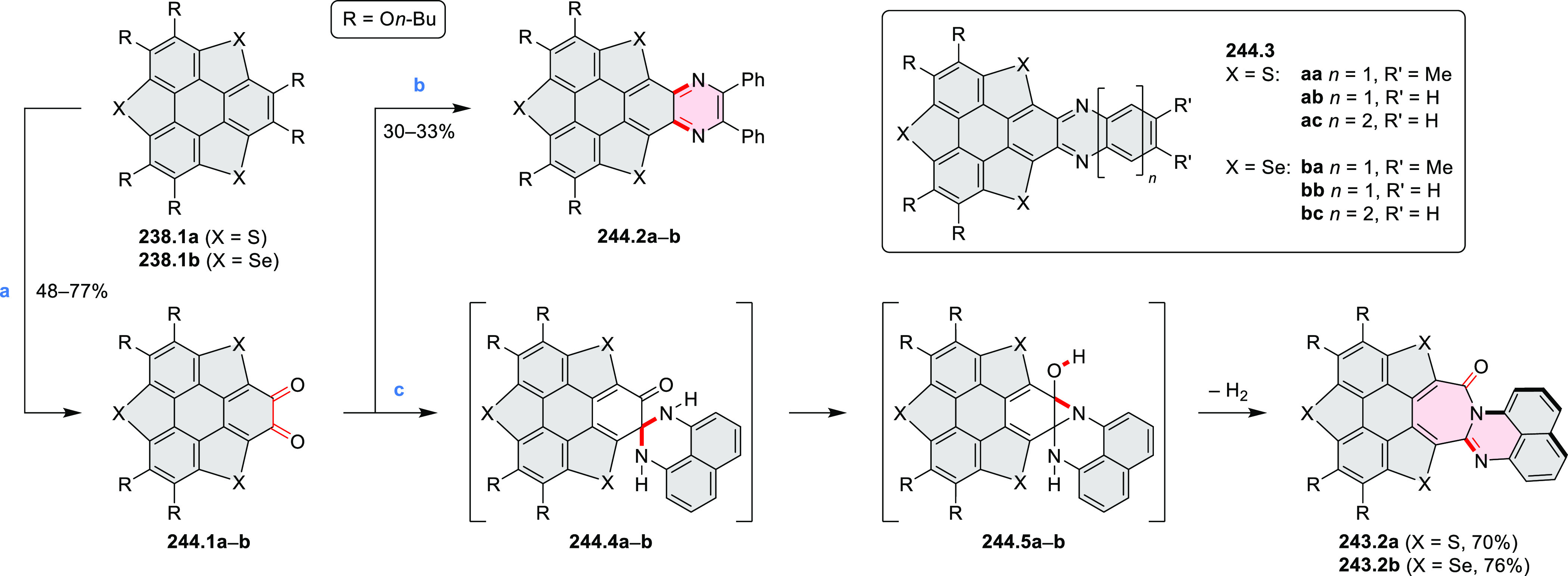
Synthesis and Reactivity of Trichalcogenasumanene-Derived *ortho*-Quinone Reagents and conditions: (a)^446^ FeCl_3_, DCM, MeNO_2_, rt, 2 h; (b) 1,2-diphenylethane-1,2-diamine, AcOH, reflux, 12 h; (c) 1,8-naphthalenediamine, AcOH, reflux, 4 h.

The same group reported the synthesis and reactivity of a trichalcogenasumanene-derived *ortho*-quinone **244.1a**,**b** (Scheme 244).^446^ On treatment of the trithia- and triselenasumanenes **238.1a**,**b** with FeCl_3_ in a mixed solvent of DCM/MeNO_2_, one of the dibutoxybenzene moieties was converted to an *ortho*-quinone unit. According to the authors, this was the first reported case of such a direct dealkylative oxidation. (The different reactivity of the tritellurasumanene **238.1c** toward FeCl_3_ has been addressed in Scheme 239.) The *ortho*-quinones **244.1a**,**b** were then condensed with 1,2-diphenylethane-1,2-diamine to afford the pyrazine-fused trichalcogenasumanene derivatives **244.2a**,**b** in 30–33% yield. Similarly, the use of aromatic *ortho*-diamines in the same reaction gave rise to a series of π-extended derivatives **244.3aa**–**bc**. Thin films of **244.3aa** and **244.3ba** behaved as a p-type semiconductor, with respective hole mobilities of 1.7 × 10^–3^ and 2.6 × 10^–3^ cm^2^ V^–^ s^–1^. Surprisingly, treatment of the *ortho*-quinone with 1,8-naphthalenediamine produced the aforementioned [7–6–6]-fused polycycles **243.2a**,**b** in 70–76% yield. The mechanism of this transformation was postulated to involve a ring rearrangement of the strained aziridine intermediate **244.5a**,**b**. The properties of **244.2a**,**b**^452^ and **244.3ba**(453) as materials were explored in subsequent work.

**Chart 22:**
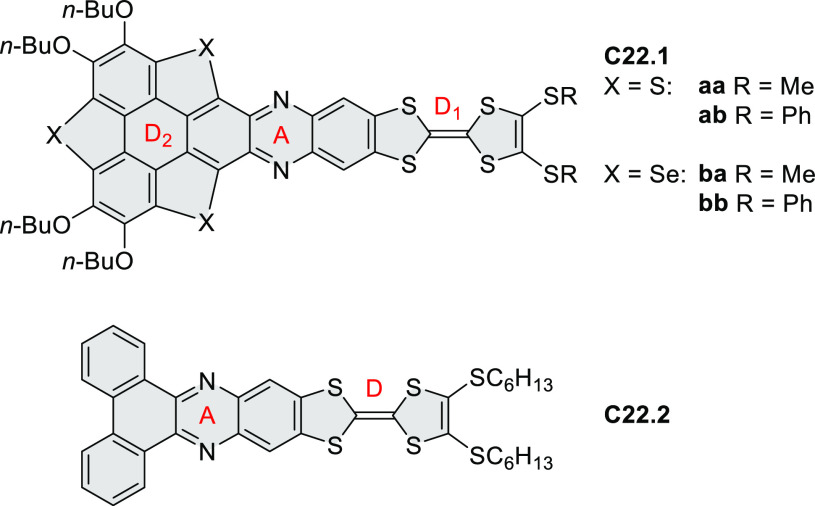
Tetrathiafulvalene-Fused Compounds with a D_1_–A–D_2_ or D–A Structure

Applying their synthetic strategy toward the pyrazine-fused trichalcogenasumanenes **244.2**, Shao et al. prepared a series of new D_1_–A–D_2_ π-systems **C22.1aa**–**bb** consisting of the electron-donating tetrathiafulvalene (D_1_) and trichalcogenasumanene (D_2_) units connected through an electron-accepting pyrazine unit (A) (Chart 22).^454^ These compounds displayed a broad absorption band (λ = 450–720 nm) as a consequence of intramolecular charge transfer (ICT) transition contributed by both D_1_ and D_2_. Femtosecond transient absorption (fs-TA) measurements were carried out under photoexcitation at λ = 385 nm. Two transient states with charge separation, CS^1^ followed by CS^2^, emerged with absorption maxima at 889 and 1105 nm, respectively. In the first state (CS^1^), the tetrathiafulvalene unit (D_1_) was in the radical cation state. Afterward, a transition from CS^1^ to the second state (CS^2^) took place via the ICT from D_2_ to D_1_^•+^. Contrary to these D_1_–A–D_2_ systems, compounds **C22.2** and **244.3ab**, possessing D–A scaffolds, exhibited only one transient charge-separated state.

**Scheme 245:**
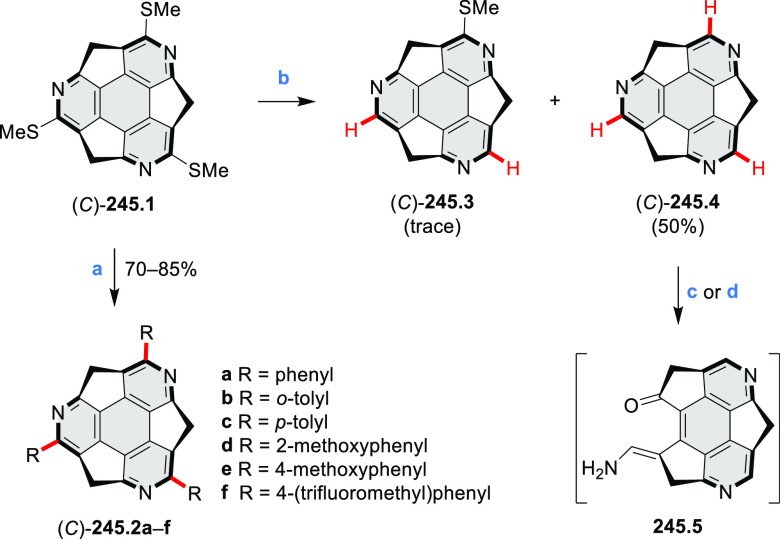
Dativizations of Enantiopure Tris(methylthio)-Substituted Triazasumanene Reagents and conditions: (a)^455^ RB(OH)_2_, Pd_2_(dba)_3_, tri(2-furyl)phosphine, copper(I) thiophene-2-carboxylate (CuTC), THF, 50 °C, 12 h; (b)^457^ Et_2_MeSiH or poly(methylhydrosiloxane) (PMHS), Pd_2_(dba)_3_, tri(2-furyl)phosphine, CuTC, THF, 50 °C, 30 min; (c) MeOH/H_2_O, pH = 2.01; (d) DCl, CD_3_OD/D_2_O.

In 2017, Sakurai et al. reported a series of *C*_3_-symmetrical triaryl-substituted triazasumanenes (*C*)-**245.2a**–**f**. These enantiopure systems were obtained by Liebeskind–Srogl arylation of the corresponding triazasumanene (*C*)-**245.1** (Scheme 245).^455^ The 3-fold reaction occurred in good yields with various 2- and 4-substituted arylboronic acids but failed with 2,6-dimethylphenylboronic acid. Based on the CD spectra of both enantiomers of **245.2f**, as well as the X-ray crystal structure of (*C*)-**245.2f**, the authors concluded that the coupling reaction was not accompanied by any racemization. Later, the same group reported the enantiopure tris(2-hydroxyphenyl)-substituted analogue of (*A*)-**245.2**, which showed dual emission in the crystalline state, caused by excited-state intramolecular proton transfer (ESIPT).^456^ The emission at 631 nm was not observable in solution. In 2018, Sakurai and Higashibayashi et al. reported the use of the same catalyst system, but with organosilanes instead of boronic acids, to induce the desulfurization of (*C*)-**245.1**.^457^ Thus, the triply desulfurized product (*C*)-**245.4** was obtained in 50% yield, accompanied by traces of the doubly desulfurized (*C*)-**245.3**. The authors found that the triazasumanene (*C*)-**245.4** was acid labile. Based on ^1^H NMR and mass spectral data, the imine cleavage was proposed to take place at a flank bond rather than a rim bond to give the hydrolysis product **245.5**.

**Scheme 246:**
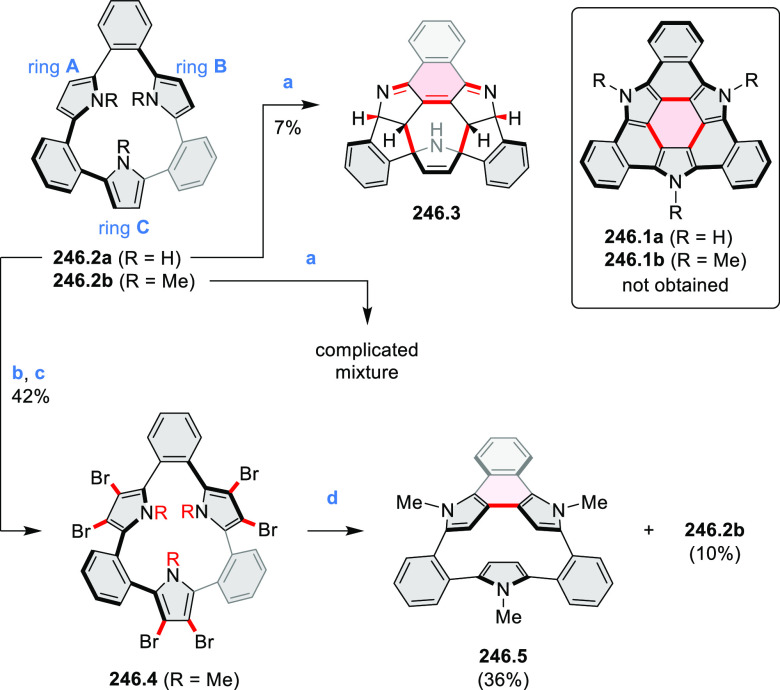
Attempted Oxidative and Reductive Fold-In Syntheses of Strained Triazasumanene Derivatives Reagents and conditions: (a)^458^ FeCl_3_, AgOTf, DCM/MeNO_2_ (10:1), rt, 24 h; (b) NBS, DMF, 0 °C, 6 h; (c) MeI, NaH, DMF, rt, 3 h; (d) Pd[P(*t*-Bu)_3_]_2_, K_3_PO_4_, toluene, 110 °C, 24 h.

In contrast to all the trichalcogenasumanenes and the C=N-containing triazasumanenes presented above, the pyrrole-containing triazasumanene skeleton is known to be highly strained. In 2017, Chen, Tanaka, and Osuka explored the construction of tribenzo-fused triazasumanenes **246.1a**,**b** via the fold-in synthesis (Scheme 246).^458^ They proposed subjecting the *ortho*-phenylene-bridged cyclic tripyrroles **246.2a,b** to 3-fold C–C bond coupling at the pyrrolic β-carbons to create the central six-membered ring. It was found that the combination of FeCl_3_ and AgOTf in DCM/MeNO_2_ transformed **246.2a** into the unexpected product **246.3** in 7% yield, instead of the desired **246.1a**. Under the same conditions, the *N*,*N*′,*N*″-trimethylated macrocycle **246.2b** produced only a complex mixture of products. The structure of **246.3** was established by X-ray crystallography to result from (i) β–β coupling of rings **A** and **B**, (ii) α–β′ addition between ring **C** and each of the rings **A** and **B**, and (iii) 1,2-hydrogen shift of the NH group in rings **A** and **B**. The alternative reductive approach using the hexabrominated cyclic tripyrrole **246.4** produced only debromination under standard Yamamoto conditions, whereas the use of Pd[P(*t*-Bu)_3_]_2_ and K_3_PO_4_ in refluxing toluene yielded the singly cyclized product **246.5** (36% yield) and the fully debrominated product **246.2b** (10% yield). The authors speculated that, after the first cyclization of **246.4**, subsequent transmetalation of the C–Pd^II^–Br to generate a further cyclized C–Pd^II^–C species was energetically disfavored due to the narrower interior space and higher rigidity of the molecule.

#### 6.1.4 [7]Heteracirculenes

**Chart 23:**
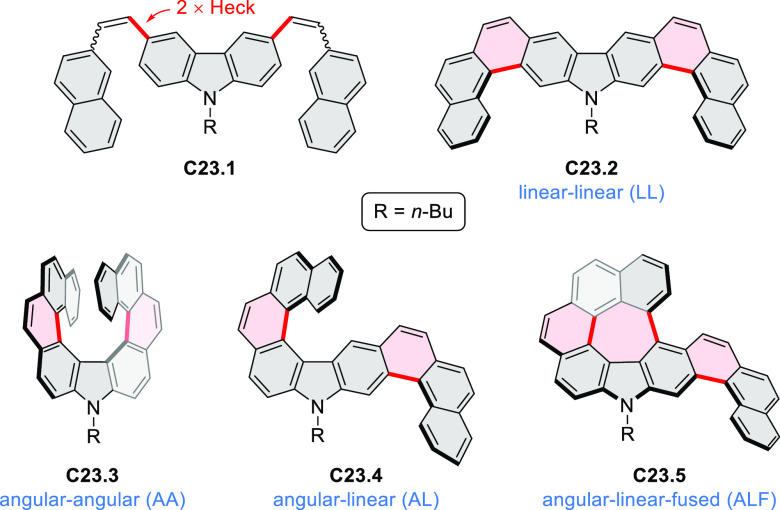
Aza[*n*]helicenes via Photochemical Cyclization

The stilbenoid substrate **C23.1** bearing two 2-naphthyl groups connected to the carbazole core via olefin linkages was explored as a precursor to aza[9]helicenes by Bedekar et al. (Chart 23).^459^ Mallory photocyclization of **C23.1**, performed using a high-pressure mercury vapor lamp in the presence of I_2_ and THF, yielded a mixture of mainly *ortho*-fused products **C23.2**–**4** containing linear (L) or angular (A) fused subsections. They were accompanied by the quasi-circulene system **C23.5**, containing a seven-membered ring fused with one pyrrole and five benzene rings. This ALF compound was proposed to originate from **C23.4** via an additional ring closure.

#### 6.1.5 [8]Heteracirculenes

**Scheme 247:**
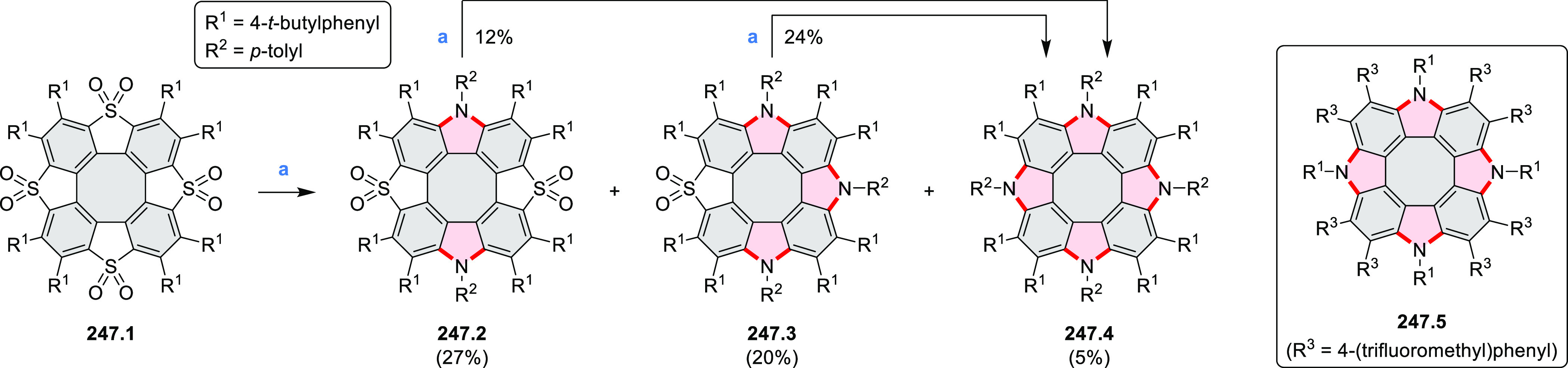
Synthesis of Tetraaza[8]circulene Derivatives via S_N_Ar Reaction Reagents and conditions: (a)^460^*p*-toluidine, KHMDS, 1,4-dioxane/toluene, 85 °C, 24 h.

In 2017, Miyake et al. reported a two-step synthesis of tetraaza[8]circulene derivatives from the corresponding tetrathia[8]circulene-based precursors (Scheme 247).^460^ The first step involved the formation of tetrathia[8]circulene octoxide, such as **247.1**, through the oxidation of sulfur atoms by *m*-CPBA. Such octoxides are negatively curved and were recently shown to exhibit AIE behavior in the solid state.^461^ In the second step, **247.1** was heated with *p*-toluidine in the presence of excess KHMDS to generate a separable mixture of the partially substituted products, **247.2** (27% yield) and **247.3** (20% yield), accompanied by the desired tetraaza[8]circulene **247.4** (5% yield). Another analogue **247.5** bearing a different substitution pattern could be formed in 25% yield without any partially substituted products.

**Scheme 248:**
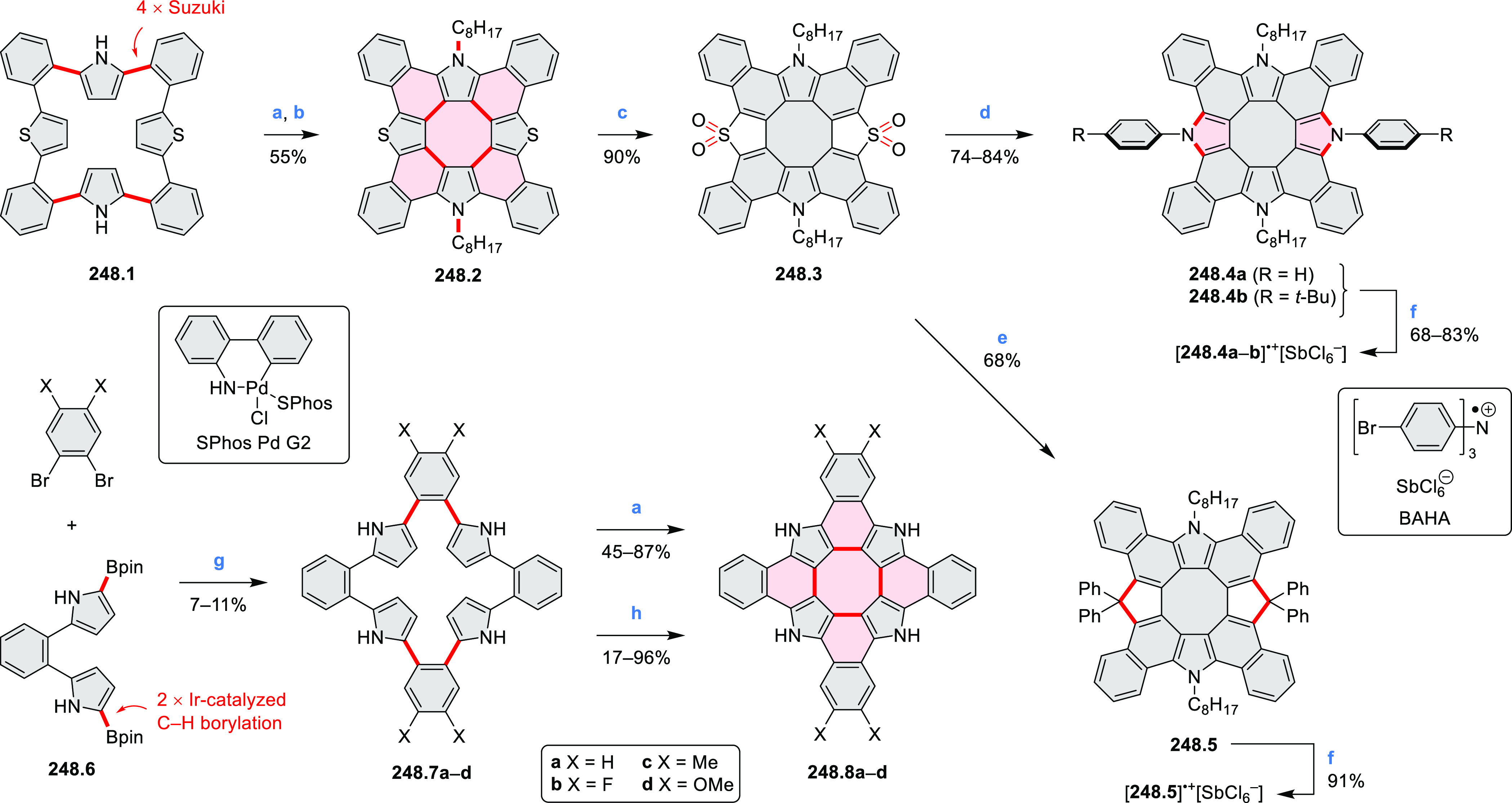
Syntheses of Tetrabenzotetraaza[8]circulenes Reagents and conditions: (a)^462^ DDQ, TfOH, DCM, rt, 10 min–6 h; (b) 1-iodooctane, NaH, DMSO, 50 °C, 38 h; (c) *m*-CPBA, CHCl_3_, reflux, 3 h; (d) aniline or 4-*tert*-butylaniline, KHMDS, dioxane, 90 °C, 24 h; (e)^463^ diphenylmethane, KHMDS, dioxane, 90 °C, 24 h; (f) BAHA, DCM, rt, 30 min; (g)^464^ SPhos Pd G2, K_3_PO_4_, THF/H_2_O, 60 °C, 12 h; (h) DDQ, Sc(OTf)_3_, toluene, darkness, reflux, 2–5 h.

In 2020, Tanaka et al. reported the synthesis and single-electron oxidation of the tetraazatetrabenzo[8]circulenes **248.4a**,**b** bearing two aryl groups on opposite pyrrole units (Scheme 248).^462^ The synthesis started with oxidative cyclization of the macrocyclic precursor **248.1**, followed by *N*,*N*′-dialkylation to introduce solubilizing groups. The resulting diazadithia[8]circulene **248.2** was subjected to *m*-CPBA oxidation to furnish the bis(*S*,*S-*dioxide) **248.3** in 90% yield. Subsequent S_N_Ar reaction with aromatic amines in the presence of KHMDS (see Scheme 247) provided the target diarylated tetraazatetrabenzo[8]circulenes **248.4a**,**b** in 74–84% yield. Chemical oxidation of these compounds with BAHA gave the corresponding radical cation salts [**248.4a**,**b**]^•+^[SbCl_6_^–^], both structurally confirmed by X-ray crystallography. The ESR spectra of both radical cations measured in DCM/toluene at 20 K show sharp signals with *g*-factors of 2.0155 and 2.0154, respectively. Unrestricted DFT calculation on **248.4a**^**•**^^+^ predicted spin delocalization over the entire [8]circulene skeleton, but not on the *N-*aryl substituents. Notably, the NIR absorptions of **248.4a**^•+^ extended to 2000 nm. An S_N_Ar reaction of **248.3** was also possible with the diphenylmethyl anion, leading ultimately to the diazatetrabenzo[8]circulene analogue **248.5** bearing two sp^3^-hybridized carbon atoms in the polycyclic framework.^463^

In 2021, the same group presented an improved synthesis of the *o*-phenylene-spaced cyclic tetrapyrroles **248.7a**–**d**.^464^ Hence, the boronate-containing dipyrrolylbenzene **248.6** underwent Suzuki–Miyaura cross-coupling with the appropriate *ortho*-dibromobenzenes to give the desired macrocycles **248.7a**–**d** in 7–11% yield. In comparison, the previous “reverse” coupling strategy suffered from lower yields attributed to the low stability of the corresponding dibromo-substituted dipyrrolylbenzene. Subsequently, two different sets of oxidative aromatic coupling conditions allowed eight-membered ring closures to afford the tetraazatetrabenzo[8]circulenes **248.8a**–**d**.

**Chart 24:**
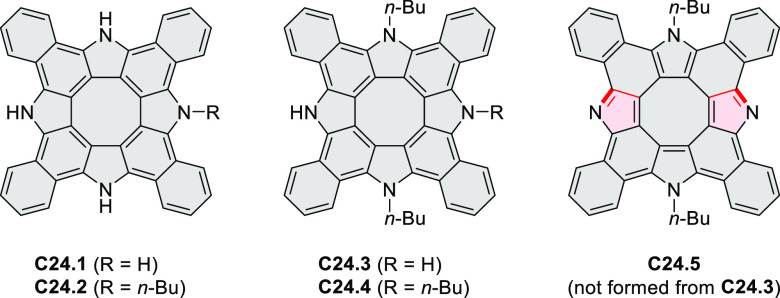
Structures of Tetraaza[8]circulene Derivatives

Controlled *N*-alkylation of the known tetrabenzotetraaza[8]circulene **C24.1** (Chart 24), performed by reacting **C24.1** with 2 or 3 equiv of 1-iodobutane and excess NaH, produced the corresponding singly, doubly, and triply *N*-butylated products **C24.2**–**4**.^465^ It was noted that double butylation occurred regioselectively on opposite pyrrole rings to yield **C24.3**. Attempts to oxidize **C24.3** using MnO_2_, NiO_2_, and FeCl_3_ to give compound **C24.5** were unsuccessful.

**Scheme 249:**
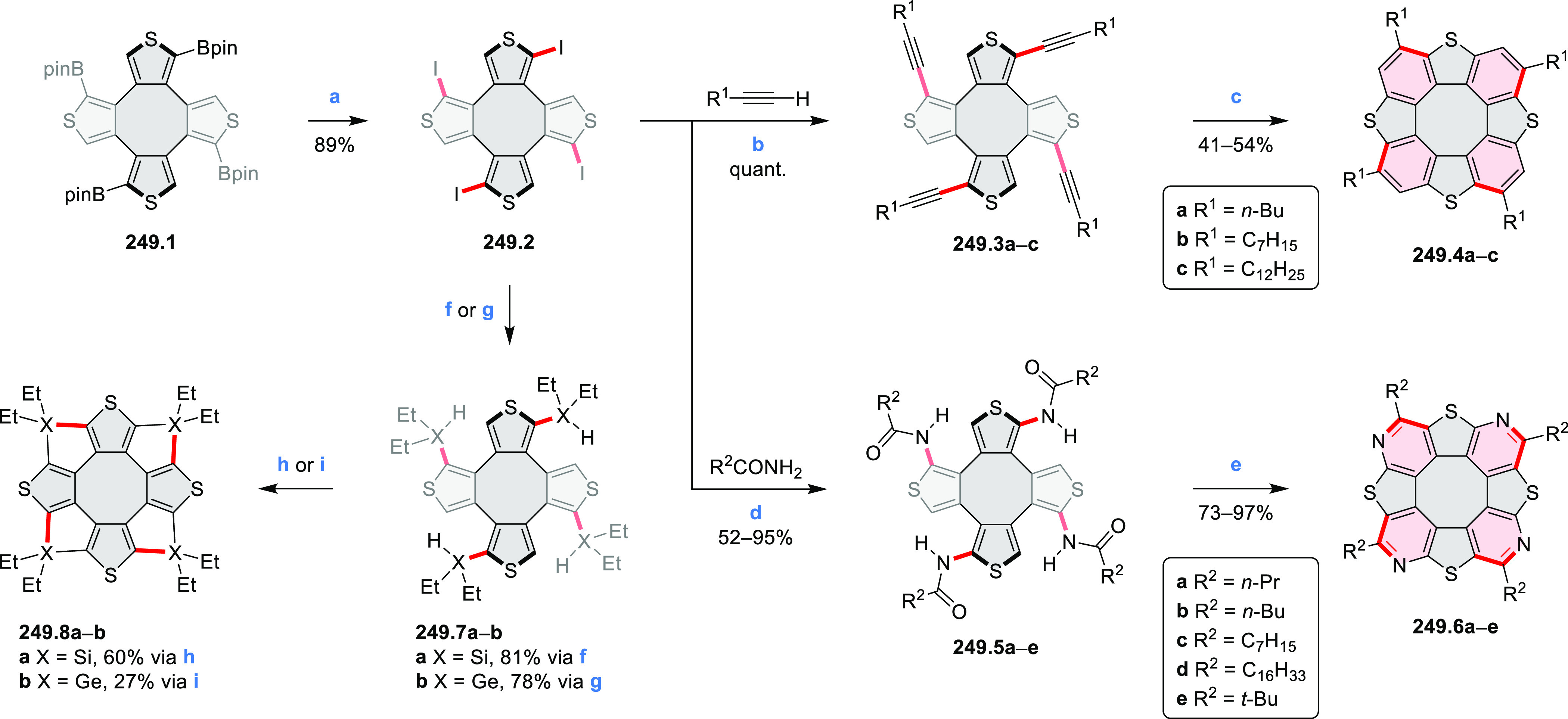
Tetrathia[8]circulenes and Their Aza-, Sila-, and Germa-Substituted Derivatives Reagents and conditions: (a)^466^ NIS, CuI, DMF, 80 °C, 3 h; (b)^467^ Pd(PPh_3_)_2_Cl_2_, CuI, Et_3_N, 60 °C, 3 h; (c) DBU, NMP, reflux, 12 h; (d)^468^ CuSO_4_·5H_2_O, K_2_CO_3_, *N*,*N′*-dimethylethylenediamine, toluene, 80 °C, 20 h; (e) POCl_3_, toluene, 120 °C, 2 h; (f)^466^ R^2^SiH_2_, Pd(*t*-Bu_3_P)_2_, Et_3_N, THF, rt, 48 h; (g)^469^ Et_2_GeH_2_, Pd(*t*-Bu_3_P)_2_, *i*-Pr_2_NEt, THF, rt, 18 h; (h)^466^ [RhCl(cod)]_2_, dppf, toluene, 140 °C, 24 h; (i)^469^ [RhCl(cod)]_2_, (C_6_F_5_)_2_PCH_2_CH_2_P(C_6_F_5_)_2_, toluene, 140 °C, 20 h.

In a series of papers, Miyake et al. demonstrated the synthetic versatility of the tetraiodo-substituted β,β-cyclooctatetrathiophene (β,β-COTh) **249.2** (Scheme 249). This compound was derived from the known tetraborate **249.1** by treatment with NIS and CuI.^466^ The 4-fold Sonogashira coupling of the tetraiodide **249.2** with aliphatic terminal alkynes gave the tetrasubstituted **249.3a**–**c** in quantitative yields.^467^ Subsequent 4-fold cycloisomerization mediated by DBU gave rise to the desired tetrathia[8]circulene derivatives **249.4a**–**c** in 41–54% yield. Alternatively, the tetraiodide was coupled with primary amides to give the tetraamides **249.5a**–**e**, which were then subjected to the Vilsmeier–Haack conditions to give the tetraazatetrathia[8]circulenes **249.6a**–**e**.^468^ Besides, the silicon- (**249.8a**)^466^ and germanium-containing (**249.8b**)^469^ hetera[8]circulenes could be obtained in two steps from the tetraiodide **249.2** by employing palladium and rhodium catalysis. The X-ray structures of **249.4a**,**6a**,**8a**,**b** showed that these polycyclic frameworks were essentially planar. Based on an isodesmic model, the association constants for the heptyl-substituted compounds **249.4b** and **249.6c** in CDCl_3_ were experimentally determined to be 58.9 and 115 M^–1^, respectively. For the tetragermatetrathia[8]circulene **249.8b**, phosphorescence at 524 nm was observable upon irradiation (λ_ex_ = 310 nm) of a frozen toluene sample at 77 K.

**Scheme 250:**
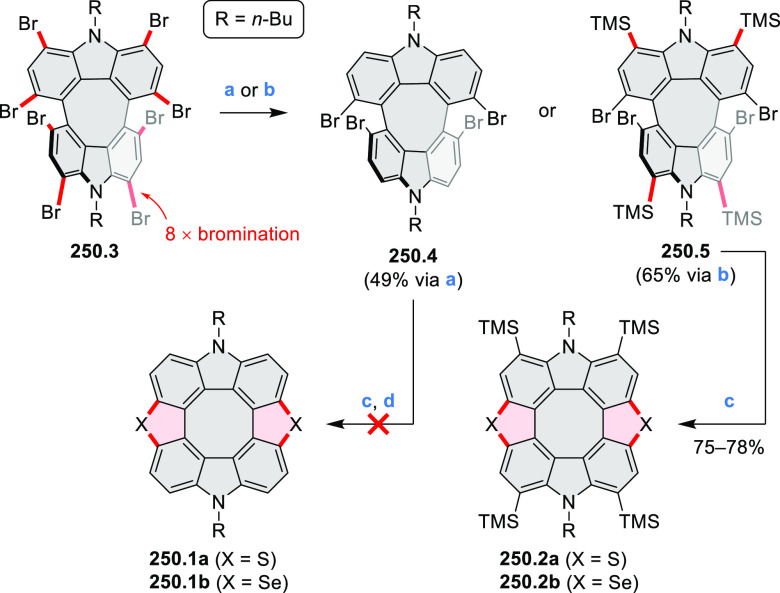
Synthesis of Diazadithia- and Diazadiselena[8]circulenes under Mild Conditions Reagents and conditions: (a)^470^ (1) *n*-BuLi, THF, −78 °C, (2) H_2_O, −78 °C, 1 h, then rt, 2 h; (b) (1) *n*-BuLi, THF, −78 °C, (2) TMSCl, −78 °C, 1 h, then rt, 2 h; (c) *n*-BuLi, THF, −78 °C, 3 h, (2) S powder or Se powder, −78 °C, 2 h, then rt, overnight; (d) Cu powder, 250 °C.

Synthetic routes toward hetera[8]circulenes **250.1**–**2** bearing two pyrrole and two chalcogenole units were explored by Wong et al. (Scheme 250).^470^*ortho*-Directed lithiation of the cyclic carbazole dimer **250.3** with *n*-BuLi, followed by addition of either water or TMSCl, furnished **250.4** and **250.5**, respectively, in fair yields. The former tetrabromide **250.4** was subjected to the known reaction sequence consisting of (i) lithiation, (ii) chalcogenization with sulfur or selenium powder, and (iii) dechalcogenization using copper powder (cf. Scheme 240). However, the desired products **250.1a**,**b** were not obtained. For the TMS-substituted tetrabromide **250.5**, however, a simple lithiation–chalcogenization sequence performed at low temperatures smoothly produced the target diazadithia- and diazadiselena[8]circulenes **250.2a**,**b** in 75–78% yield. The structures of both **250.2a**,**b** were elucidated by X-ray crystallography. In particular, the mean interior angle in the eight-membered ring was 135.0° for **250.2a** and 134.8° for **250.2b**, each almost identical to that of a regular octagon (135°).

**Scheme 251:**
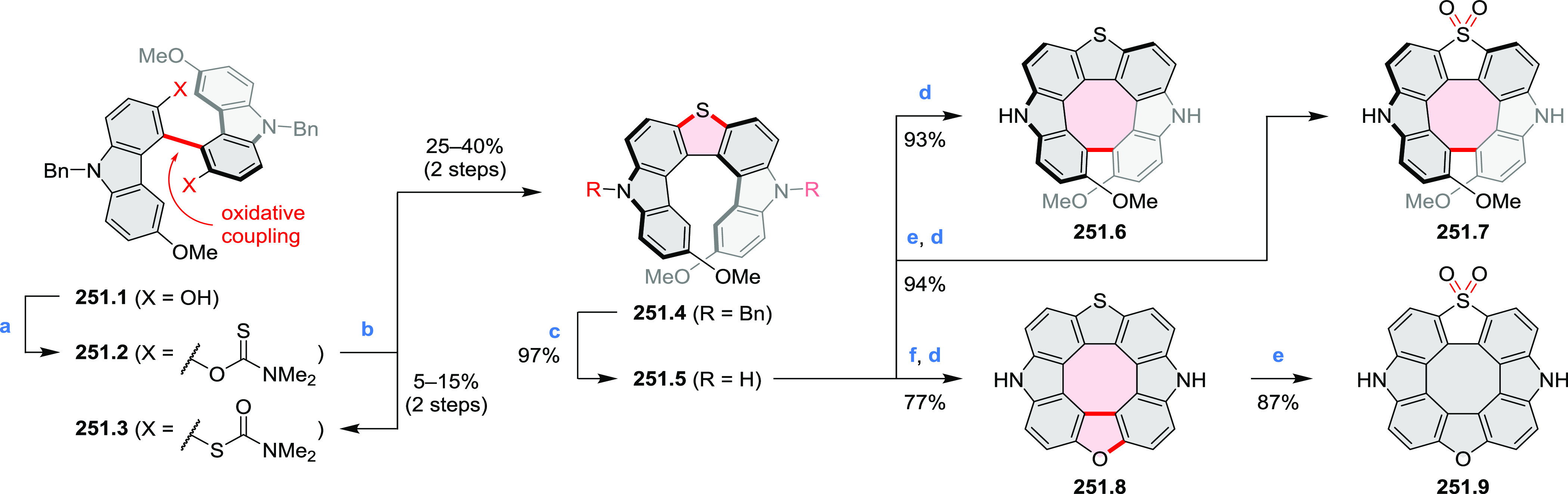
Synthesis of a Planar Diazaoxathia[8]circulene Skeleton by Compression of a Nonplanar [7]Helicene Precursor Reagents and conditions: (a)^471^ (1) NaH, DMF, 0–25 °C, 40–45 min, (2) dimethylthiocarbamoyl chloride, 85 °C, 2 h; (b) 300 °C, 25 min; (c) *t*-BuOK, O_2_, DMSO, 25 °C, 1 h; (d) chloranil, BF_3_·OEt_2_, MeCN/DCM, 25 °C, 40–45 min; (e) *m*-CPBA, THF/DCM, 25 °C, 30–45 min; (f) *n*-Bu_4_NI, BCl_3_, MeCN/DCM, 25 °C, 1 h.

In 2020, Pittelkow et al. reported the preparation of **251.8**–**9**, the first hetero[8]circulenes containing three different heterocycles (Scheme 251).^471^ The first key step in their synthesis was the thermal Newman–Kwart rearrangement of the 4,4′-bicarbazole derivative **251.2** bearing two *O*-thiocarbamate functionalities at C-3 and C-3′. The resulting diazathia[7]helicene **251.4** was debenzylated to give **251.5** with free pyrrolic NH. Intramolecular oxidative coupling of **251.5** using chloranil and BF_3_·OEt_2_ produced the eight-membered ring closure product **251.6** in 93% yield. The same cyclization could also take place after sulfone formation to give **251.7** in 94% over two steps. More remarkably, a demethylation–oxidative coupling sequence on **251.5** directly generated diazaoxathia[8]circulene **251.8** in 77% yield. The NICS(0) values calculated for the central eight-membered ring in the planar [8]circulenes **251.8** and **251.9** were respectively +7.73 and +8.50 ppm, i.e., higher than those determined for the nonplanar counterparts **251.6** and **251.7** (+7.63 and +7.41 ppm, respectively).

**Scheme 252:**
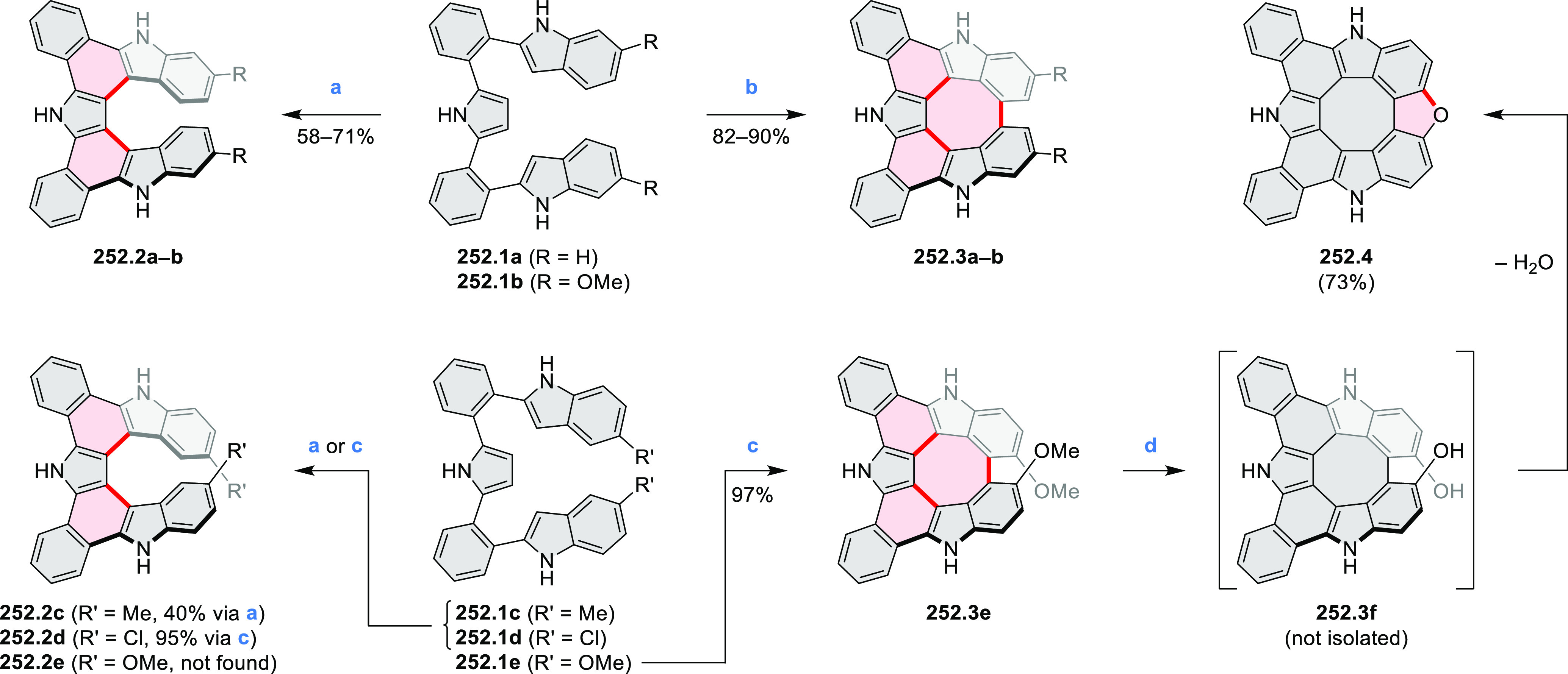
Oxidative and Dehydrative Cyclization Reactions between Pyrrole and Indole Units Reagents and conditions: (a)^472^ (1) PIFA (2.5 equiv), DCM, −78 °C, 30 min, (2) rt, 1 h; (b) DDQ (5 equiv), Sc(OTf)_3_ (5 equiv), toluene, reflux, 2–3 h; (c)^473^ PIFA (3.0–3.5 equiv), DCM, −78 °C, 3 h, (2) rt, 45 min; (d) BBr_3_, DCM, rt, 12 h.

In 2018, Tanaka and Osuka et al. showed that compounds **252.1a**–**c** underwent intramolecular oxidative coupling to different extents under two different sets of conditions (Scheme 252).^472^ Conducting the cyclization at low temperatures with PIFA as the oxidant, the three triaza[7]helicene derivatives **252.2a**–**c** resulting from a double cyclization were isolated in 40–71% yield. In contrast, the Sc(OTf)_3_-mediated cyclization of **252.1a**,**b** at a high temperature yielded the triply cyclized compounds **252.3a**,**b** bearing an additional eight-membered ring in 82–90% yield. Subsequently, the same group examined the reactivity of two additional substrates, **252.1d**–**e**, toward PIFA.^473^ The dichloro-substituted **252.1d** underwent a similar double cyclization to give the corresponding triaza[7]helicene **252.2d** in high yield. However, the dimethoxy derivative **252.1e** furnished only the triply cyclized **252.3e** (97% yield) without any sign of the double cyclization product **252.2e**. Importantly, direct formation of the triazaoxa[8]circulene derivative **252.4** was achieved by simply treating **252.3e** with BBr_3_. The diol intermediate **252.3f** was not detected even at lower reaction temperatures. This observation was attributed to the rapid cyclodehydration of **252.3f** driven by strain release.

**Scheme 253:**
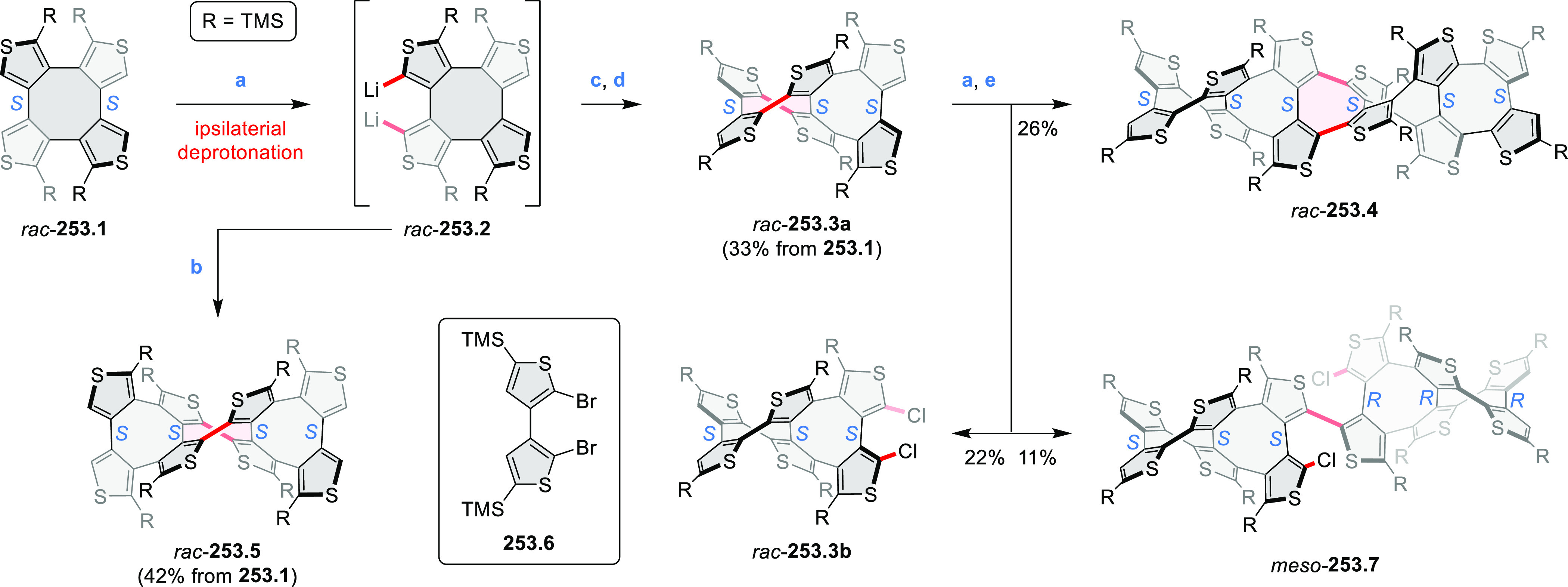
Iterative Deprotonation–Coupling Approach toward Thiophene-Based Double Helices^,^ For all the racemates, the absolute configurations of the chiral axes of depicted enantiomers are shown to facilitate discussion. Reagents and conditions: (a)^474^*n*-BuLi (2.1 equiv), THF, −78 °C, 2 h, then 60 °C, 2 h; (b) CuBr_2_, Et_2_O, −78 °C, 1 h, then rt, overnight; (c) ZnCl_2_, −78 °C, then rt; (d) **253.6**, Pd(PPh_3_)_4_, Et_2_O, 120 °C, 48 h; (d) CuCl_2_, Et_2_O, −78 °C, 2 h, then rt, 24 h.

In 2016, Wang and Rajca et al. described the synthesis and properties of a series of thiophene-based double helices *rac*-**253.3**–**5** of different lengths (Scheme 253).^474^ Following their earlier results on the regioselective deprotonation of β,β-cyclooctatetrathiophene (β,β-COTh), the racemic tetrasubstituted β,β-COTh derivative *rac*-**253.1** was exposed to *n*-BuLi (2 equiv) in THF at −78 °C for 2 h, and then heated at 60 °C for 2 h. As a result of the excellent ipsilateral selectivity, the generated dilithiated species *rac*-**253.2** had both lithium centers occupying the same bay region. Then, two different transformations were performed on *rac*-**253.2**. First, the oxidative self-coupling promoted by CuBr_2_ afforded the double helix *rac*-**253.5** in 42% yield. Second, the conversion of *rac*-**253.2** to the corresponding organozinc chloride species followed by Negishi coupling with the dibromide **253.6** furnished the shorter double helix *rac*-**253.3a** in 33% yield. This racemate was thereby submitted to ipsilateral deprotonation on the β,β-COTh moiety followed by treatment with CuCl_2_, to generate, besides the dichlorination side product *rac*-**253.3b**, two intermolecularly coupled products. The first one was the target double helix *rac*-**253.4** resulting from the formation of two C–C bonds between two reactant molecules of the same chirality. The second one, *meso*-**253.7**, originated from the C–C bond formation between a pair of enantiomers. In the latter case, chlorination occurred at the remaining lithiated sites because the second C–C bond formation was impossible. Since the yield of *rac*-**253.4** (26%) was approximately twice that of *meso*-**253.7** (11%), the first C–C bond formation was thought the be highly diastereoselective. A similar diastereoselectivity also accounted for the formation *rac*-**253.5** in reasonable yields.

**Scheme 254:**
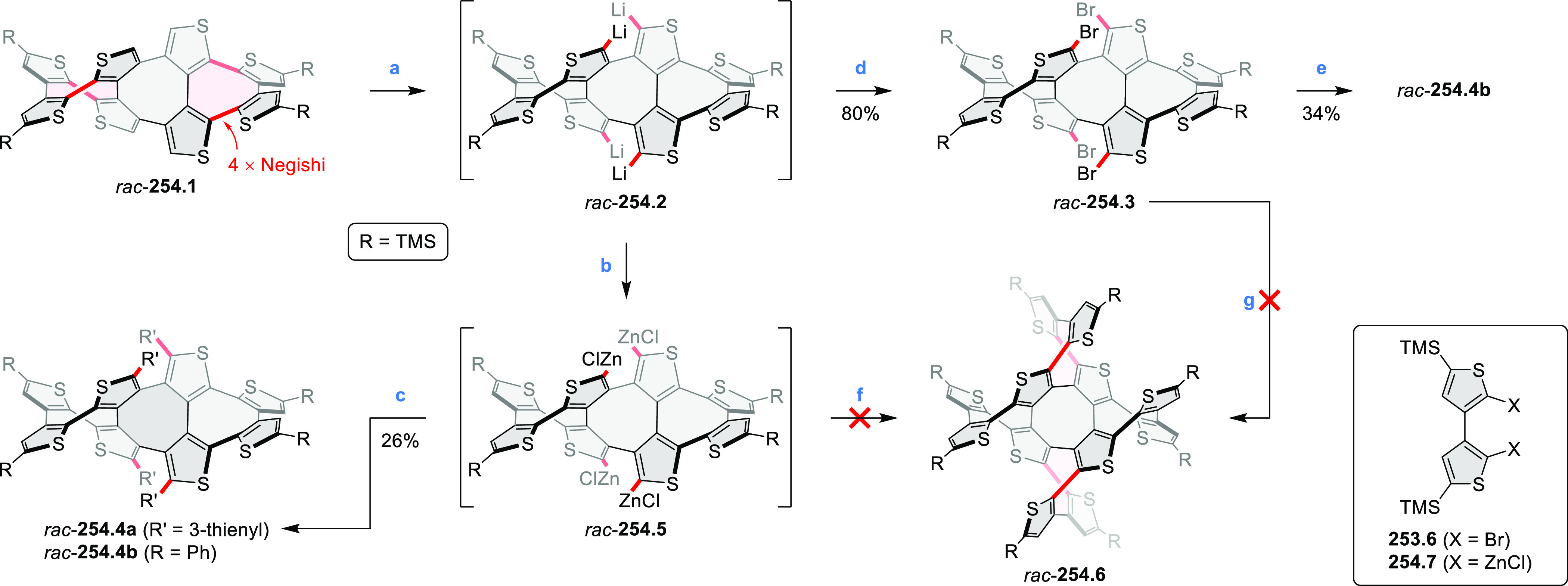
Arylation and Attempted Vertical Extension of a Thiophene-Based Double Helix Reagents and conditions: (a)^475^*n*-BuLi, THF, −78 °C, then 60 °C, 2 h; (b) ZnCl_2_, −78 °C, then rt; (c) 3-bromothiophene, Pd(PPh_3_)_4_, THF, 120 °C, 48 h; (d) C_2_Br_2_Cl_4_, −78 °C, then rt, overnight; (e) phenylboronic acid, Cs_2_CO_3_, THF/H_2_O (50:1), 100 °C; (f) **253.6**, Pd(PPh_3_)_4_, THF, 120 °C; (g) **254.7**, Pd(PPh_3_)_4_, THF, 120 °C.

Subsequently, Wang and Li et al. reported an attempted synthesis of compound **254.6** featuring a cross-dimensional double-helical structure based on the β,β-COTh core (Scheme 254).^475^ The common starting material *rac*-**254.1** was prepared via the Negishi coupling reaction between the 3,3′-bithiophene derivative **253.6** or **254.7** with the appropriate β,β-COTh precursor in 2:1 ratio in a way similar to that shown in Scheme 253. The treatment of *rac*-**254.1** with *n*-BuLi generated the tetralithiated species *rac*-**254.2**, which could be transmetalated with ZnCl_2_ and coupled with 3-bromothiophene to yield the 4-fold arylated product *rac*-**254.4a** in 26% overall yield. The tetralithiated species could also be reacted with 1,2-dibromo-1,1,2,2-tetrachloroethane to afford, in 80% overall yield, the tetrabromide **254.3**, which was then cross-coupled with phenylboronic acid to give *rac*-**254.4b** in 34% yield. However, when either *rac*-**254.5** or *rac*-**254.3** was reacted with the respective 3,3′-bithiophene **253.6** or **254.7**, the expected Negishi coupling did not take place to yield **254.6**. The authors reasoned that the formidable torsional strain in the double helical skeleton might account for this unsuccessful transformation.

**Scheme 255:**
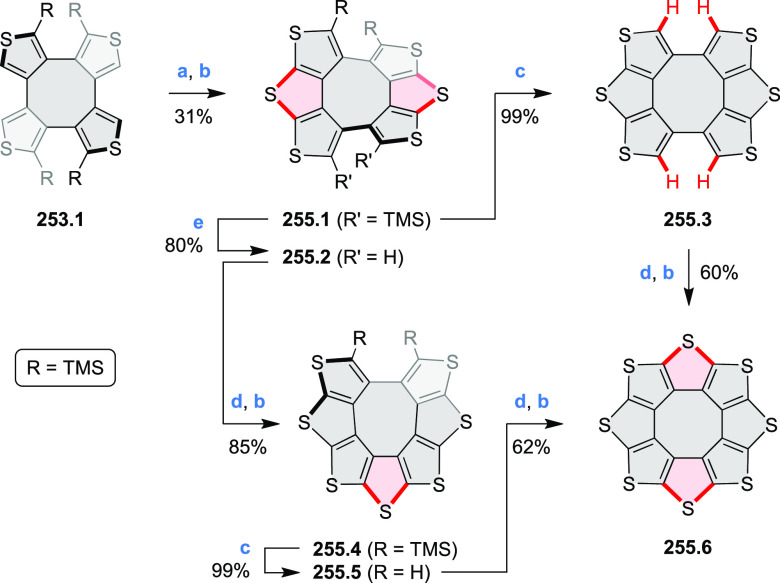
Synthesis of Sulflower and Related Systems Reagents and conditions: (a)^476^*n*-BuLi, Et_2_O, −78 °C, then 60 °C, 2 h; (b) (PhSO_2_)_2_S, −78 °C, then rt, overnight; (c) TFA, DCM, rt, monitor by TLC until complete reaction; (d) LDA (4.1 or 2.1 equiv), THF, 0 °C, 2 h; (e) TFA, DCM, rt, then aq. NaHCO_3_ right after addition of TFA.

In 2018, Wang and Shi et al. reported an alternative strategy toward the known octathia[8]circulene **255.6** (“sulflower”, cf. CR2017, Section 6.1.5) using β,β-COTh-based starting materials (Scheme 255).^476^ The tetrakis(trimethylsilyl)-substituted β,β-COTh derivative **253.1** was quadruply deprotonated by *n*-BuLi and then reacted with bis(phenylsulfonyl)sulfide to give the doubly annulated product **255.1** in 31% yield. The four TMS groups were quantitatively acidolyzed by TFA, and the resultant compound **255.3** underwent quadruple deprotonation by LDA, followed by double annulation, to yield the “sulflower” **255.6** in 60% yield. This acidolysis–deprotonation–annulation sequence could also be performed stepwise, involving the intermediacy of compounds **255.2,4**–**5**. The OFET device performances of compounds **255.3**,**5**,**6** were examined. The hole mobilities of **255.5** and **255.6** (6.8 × 10^–4^ and 2.6 × 10^–3^ cm^2^ V^–1^ s^–1^, respectively, at 80 °C) were both higher than that of “sulflower” (6.4 × 10^–4^ cm^2^ V^–1^ s^–1^ at the same temperature).

**Scheme 256:**
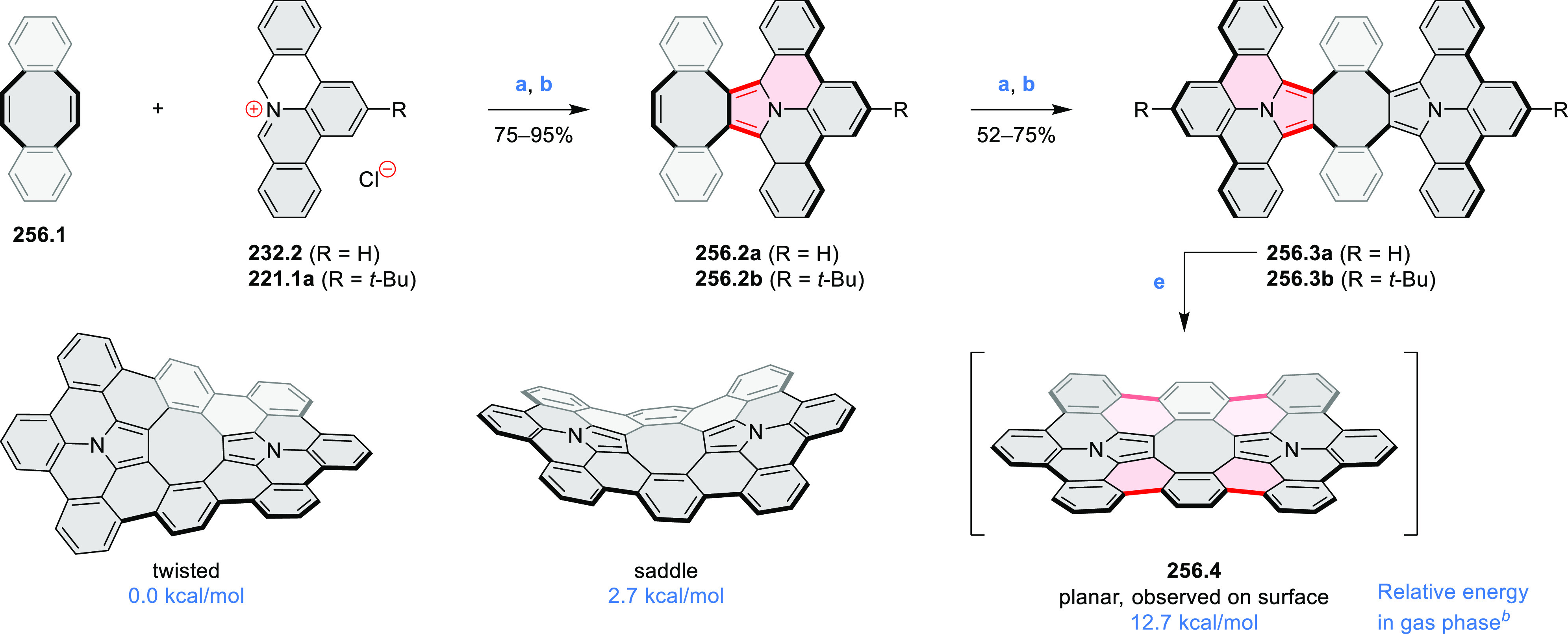
Combined In-Solution/On-Surface Approach toward a π-Extended Diaza[8]circulene Derivative^,^ Reagents and conditions: (a)^477^*i*-Pr_2_NEt, DMSO, 140 °C, 6 h–18 h; (b) DDQ, DCM, rt, 10–1 h; (e) >430 °C on Au(111). Level of theory: B3LYP/6-31G(d).

Ito, Kawai, and Foster et al. reported a combined in-solution and on-surface synthesis of a π-extended diaza[8]circulene **256.4** (Scheme 256).^477^ In their approach, based on the PAMY chemistry discussed earlier (see Schemes 221 and 232), dibenzo[*a*,*e*]cyclooctatetraene (**256.1**) reacted in a stepwise manner with the iminium salts **232.2** and **221.1a** to give the products **256.3a**,**b** with two newly fused pyrrole units. Notably, direct 2-fold cycloaddition of **256.1** proved unachievable. X-ray diffraction data showed that **256.3b** adopted a tub-shaped geometry. Conversion of **256.3a** into the diaza[8]circulene **256.4** could not be achieved in solution but was successfully realized via on-surface synthesis. Compound **256.3a** was deposited on Au(111) at rt under ultrahigh vacuum and annealed at ca. 700 K to promote cyclodehydrogenation. STM images recorded before and after the reaction showed that some of the adsorbed molecules changed from the tub conformation to a flat structure, whereas some remained unchanged. The high-resolution CO-STM images confirmed the planarity of the Au(111)-adsorbed molecule. Bond lengths in the eight-membered ring were 1.42, 1.56, and 1.69 Å, indicating a pronounced bond order variation. The eight-membered ring was believed to be highly strained judging from its distortion from the regular octagonal shape. DFT calculations on **256.4** predicted that, in the gas phase, the planar and saddle conformations were 12.7 and 2.7 kcal mol^−1^ above the most stable twisted conformation.

#### 6.1.6 Larger Circulenes and Coronoids

**Scheme 257:**
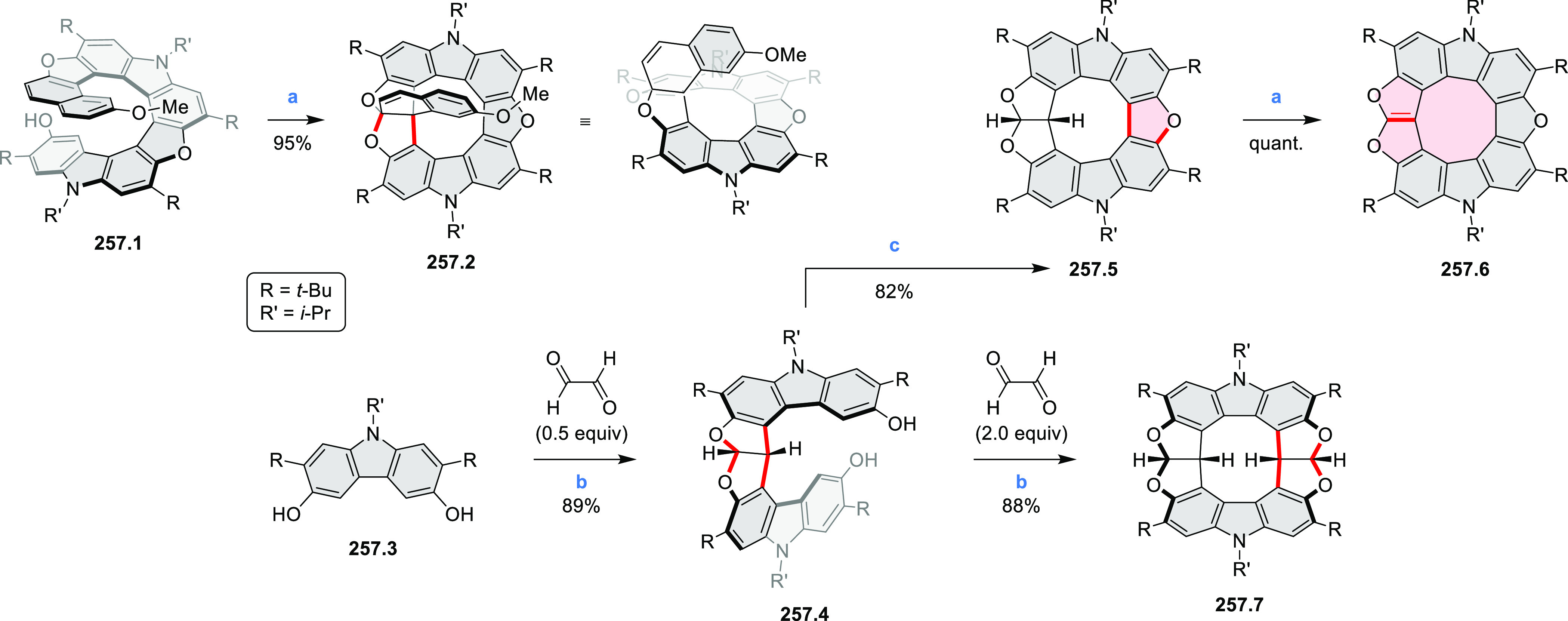
Syntheses of Derivatives of Diazatrioxa[9]circulene and Diazatetraoxa[10]circulene Reagents and conditions: (a)^478^ DDQ, DCM, rt; (b)^479^ H_2_SO_4_, AcOH, rt, 5–16 h; (c) chloranil, BF_3_·Et_2_O, DCM, rt, 45 min.

In 2020, Pittelkow and Baryshnikov et al. reported the isolation of dihydrodiazatrioxa[9]circulene **257.2**, which represented the first [*n*]circulene system with *n* > 8 (Scheme 257).^478^ Compound **257.2** was synthesized via a high-yielding oxidation of the known diazatrioxa[10]helicene **257.1** by DDQ, and was characterized by X-ray crystallography. The same group later reported the fully conjugated diazatrioxa[9]circulene.^479^ The synthesis began with the acid-catalyzed condensation of the carbazolediol **257.3** with 0.5 equiv of glyoxal to give compound **257.4** in 89% yield. Oxidation with chloranil/BF_3_·Et_2_O (cf. Scheme 251, Section 6.1.5) furnished the incompletely dehydrogenated **257.5**, which could be converted into the fully conjugated hetera[9]circulene **257.6** by subsequent quantitative oxidation with DDQ. The tetrahydro[10]circulene **257.7** could also be formed from further condensation of compound **257.4** with glyoxal. The XRD data of compound **257.6** show a planar conformation, with a significant contribution from a [9]radialene character for the nine-membered ring. Both compounds **257.6** and **257.7** show well-defined blue fluorescence at 400–500 nm. The fully conjugated hetera[9]circulene **257.6** is photolabile in aerated solvents, while the tetrahydrohetera[10]circulene is stable under similar conditions. The instability of the former compound was attributed to the intersystem crossing from the S_1_ state to the reactive T_1_ state with spin density that is localized on the α-carbon atom of the furofuran moiety.

**Scheme 258:**
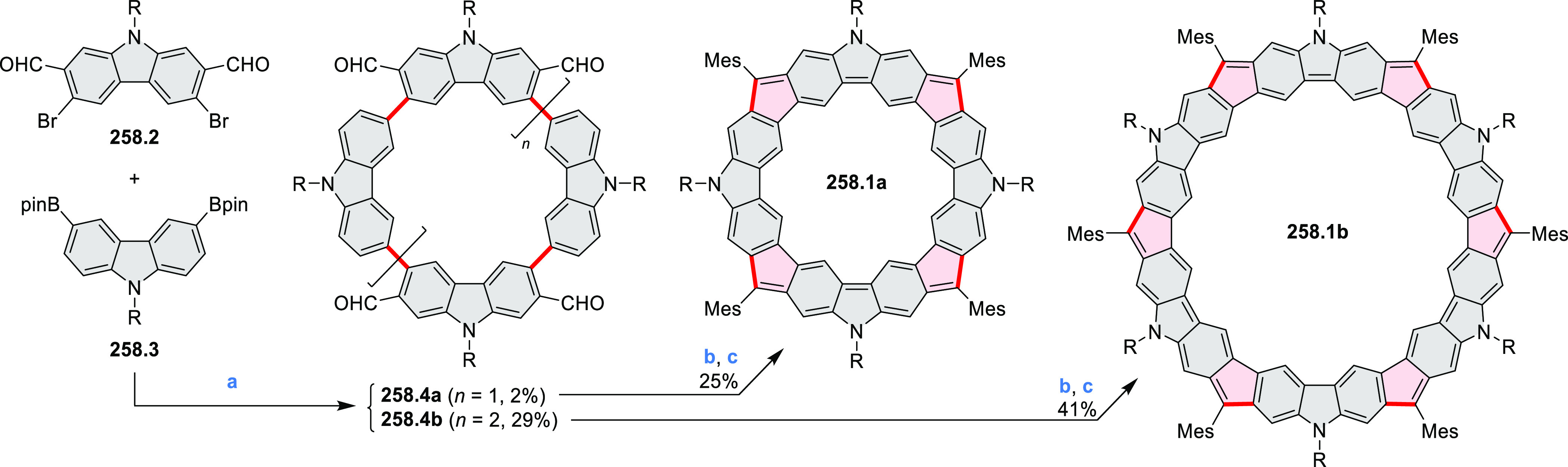
Synthesis of Carbazole-Based Macrocyclic Tetraradicaloid and Hexaradicaloid Reagents and conditions: (a)^480^ Pd_2_(dba)_3_, *t*-Bu_3_P·HBF_4_, NaHCO_3_, THF/H_2_O, 85 °C, 72 h; (b) (1) MesMgBr, THF, rt, 24 h, (2) BF_3_·OEt_2_, DCM; (c) DDQ, DCM, rt, 10–30 min.

In 2016, Wu, Casanova, Ding, and Casado et al. reported the macrocyclic tetraradicaloid **258.1a** and hexaradicaloid **258.1b**, each consisting of alternating quinoidal and aromatic carbazole units (Scheme 258).^480^ The Suzuki–Miyaura cross-coupling of the known carbazole monomers **258.2** and **258.3** followed by recycling GPC allowed the isolation of **258.4a** (2% yield) and **258.4b** (29% yield) containing four and six carbazole units, respectively. A conventional mesitylation–cyclization sequence on **258.4a** and **258.4b** led to the respective 4-fold (25% yield) and 6-fold cyclized products (41% yield) as stereoisomeric mixtures, which were then individually oxidized by DDQ to generate the target macrocycles **258.1a**,**b** in quantitative yields. Magnetic properties of **258.1a**,**b** were investigated by superconducting quantum interfering device (SQUID) measurements. The results suggested that both molecules possessed a singlet ground state, in agreement with computational predictions. The singlet–triplet energy gaps (Δ*E*_S–T_) were estimated to be −0.25 (for **258.1a**) and −0.30 kcal mol^−1^ (for **258.1b**), confirming the accessibility of the paramagnetic triplet state upon thermal excitation.

**Scheme 259:**
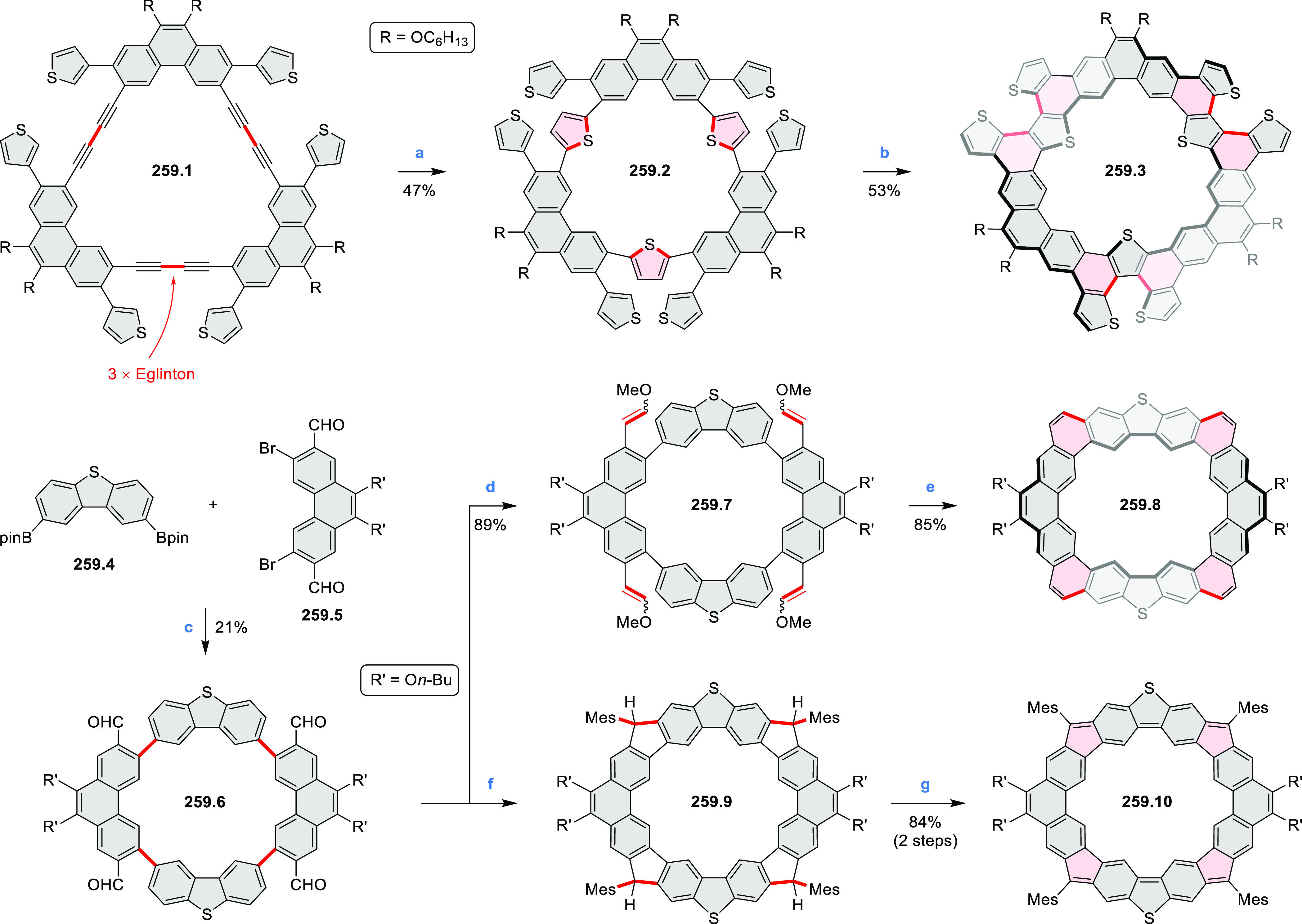
Dibenzothiophene–Phenanthrene-Based Macrocycles Reagents and conditions: (a)^481^ Na_2_S·9H_2_O, CuI, 1,10-phenanthroline, DMF, 140 °C, N_2_, 16 h; (b) FeCl_3_, DCM/MeNO_2_, bubbling with N_2_, 0 °C, 10 min; (c)^482^ Pd_2_(dba)_3_, *t*-Bu_3_P·HBF_4_, NaHCO_3_, THF/H_2_O, 80 °C, 2 days; (d) premixed [MeOCH_2_PPh_3_]^+^Cl^–^ and *t*-BuOK, THF, rt, 2 h; (e) Bi(OTf)_3_, dry 1,2-DCE, rt, 3 h; (f)^483^ (1) MesMgBr, THF, rt, overnight, (2) BF_3_·OEt_2_, DCM, 3 h; (g) DDQ, DCM, rt, 6 h.

Three examples of thiophene-containing coronoids (cycloarenes) have been recently reported (Scheme 259). In 2018, Miao et al. reported the preparation of the heterocycloarene **259.3**.^481^ The first macrocyclic intermediate, **259.1**, was obtained via the Eglinton reaction of the corresponding diyne precursor. The 1,3-diyne units in **259.1** were converted into thiophene units in **259.2** using Na_2_S, CuI, and 1,10-phenanthroline. Eventually, the action of FeCl_3_/MeNO_2_ induced the 6-fold oxidative cyclization among the thiophene moieties of **259.2**, to produce the target heterocycloarene **259.3**, which was isolated in 53% yield. This compound displayed a lowest-energy absorption maximum at 455 and a rather small Stokes shift of 0.20 eV in the emission spectrum. The broad aromatic resonances in the ^1^H NMR spectrum of **259.3** were attributed to the hindered inversion of the trithia[5]helicene units in the solution state.

The thiophene-containing analogue of octulene, **259.8**, was reported in 2020 by Wu, Lu, and Zhao.^482^ The dibenzothiophene- and phenanthrene-based building blocks (**259.4** and **259.5**) were reacted in a 1:1 ratio under Suzuki–Miyaura cross-coupling conditions. The macrocyclic product **259.6** bearing four aldehyde groups was isolated in 21% yield after recycling GPC. Next, the Wittig reaction between **259.6** and (methoxymethylene)triphenylphosphane Ph_3_P=CH(OMe) was used to install four 2-methoxyvinyl groups in high yield. The resultant compound **259.7** was submitted to Bi(OTf)_3_-catalyzed electrophilic ring closures to afford the target **259.8** in 85% yield. This molecule was predicted by DFT calculation to assume a saddle-shaped conformation. Upon complexation with C_60_ or C_70_, the fluorescence emissions of **259.8** at 466 and 495 nm were quenched. The fluorescence titration data showed a higher association constant of **259.8** with C_60_ (1.25 × 10^6^ M^–1^) than with C_70_ (9.49 × 10^5^ M^–1^) in toluene. Subsequently, Lu and Wu et al. reported the preparation of the tetraradicaloid **259.10** from the macrocycle **259.6**.^483^ Here, **259.6** was treated with mesitylmagnesium bromide followed by BF_3_·Et_2_O to give **259.9** as a stereoisomeric mixture. Dehydrogenation by DDQ provided the fully conjugated compound **259.10** in 84% yield over two steps. From the single-crystal XRD data of **259.10**, the two benzenoid rings in the dibenzothiophene unit show significant bong length alternation, indicating the dominance of the quinoidal form as shown in Scheme 259. Based on VT-EPR data, the singlet–triplet energy gap of **259.10** was determined to be −3.47 kcal mol^−1^.

**Scheme 260:**
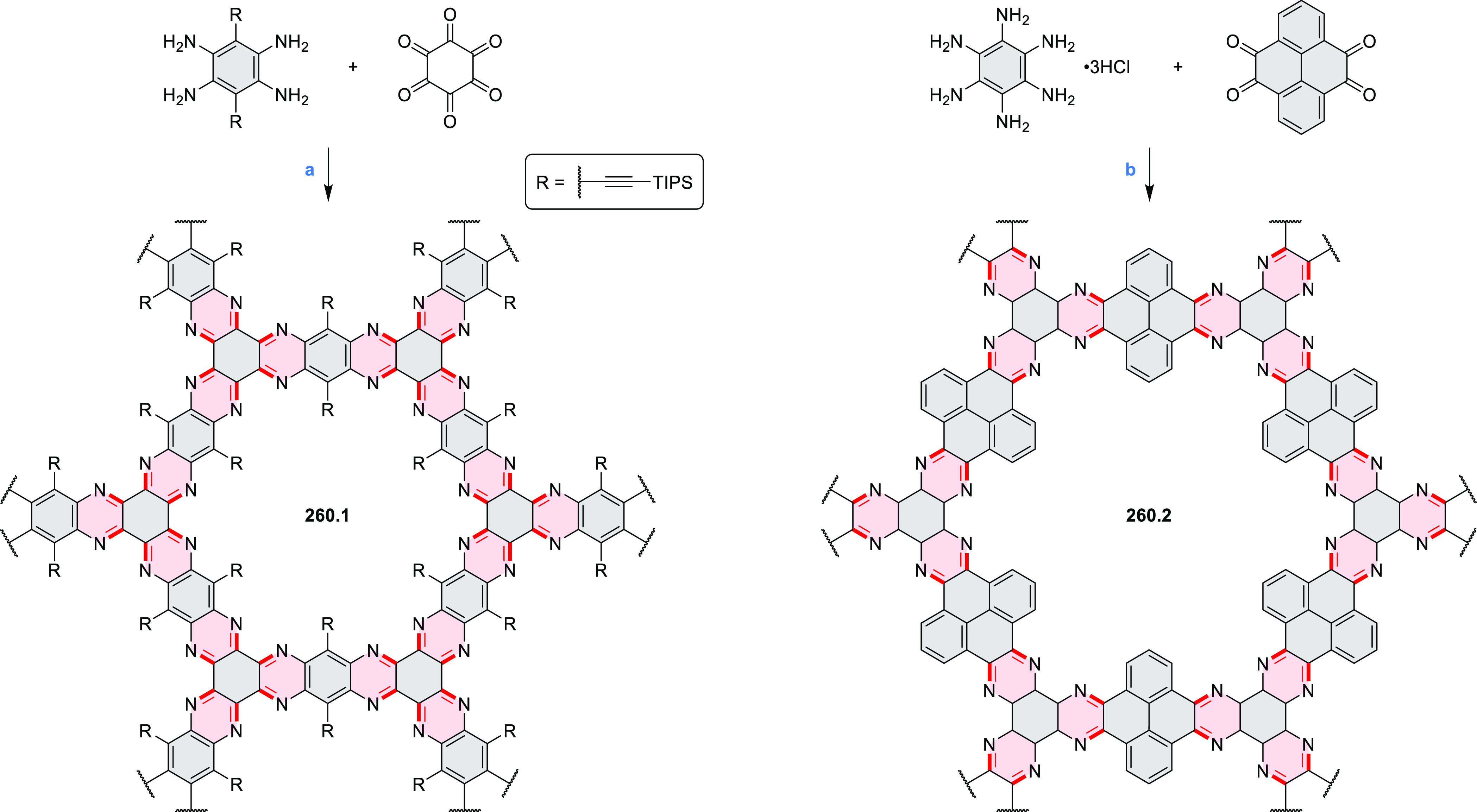
Phenazine-Fused Porous Conjugated Frameworks Reagents and conditions: (a)^484^ dioxane/AcOH (1:4), 135 °C, 7 days; (b)^485^ TfOH, 175 °C.

Heteroatom-doped coronoid substructures can be found in some of the recently reported 2D polymers obtained by condensation of aromatic components. In 2017, Mateo-Alonso et al. reported the first solvothermal synthesis of **260.1**, a conjugated mesoporous polymer (CMP) bearing fused pyrazine units (Scheme 260).^484^ This CMP was prepared from the condensation of bis(TIPS-ethynyl)-substituted benzenetetramine and hexaketocyclohexane in dioxane/acetic acid at 135 °C. According to the authors, the known, alternative ionothermal synthesis employing harsher conditions is incompatible with the TIPS groups. Using semiempirical calculations, **260.1** was predicted to assume a highly twisted framework with the bulky TIPS-ethynyl substituents being congested in the node regions. Upon sonication, the CMP **260.1** could form homogeneous dispersions in TFA, DMF, and EtOH/H_2_O (1:1). The EtOH/H_2_O dispersion was examined by atomic force microscopy (AFM) and found to consist of exfoliated layers with a mean diameter of 200 nm. In 2021, Baek, Oh et al. reported another fused aromatic framework **260.2** via the condensation of benzenehexamine trihydrochloride and 4,5,9,10-pyrenetetraone in triflic acid at 175 °C.^485^ The structure of **260.2** was supported by FT-IR spectroscopy, X-ray photoelectron spectroscopy, and elemental analysis. The isolated thin flakes of **260.2** showed remarkable electron and hole mobilities of 996 and 501 cm^2^ V^–1^ s^–1^, respectively.

### 6.2 Fused Acenaphthylene Derivatives

#### 6.2.1 Hetero[*a*]fused Acenaphthylenes

**Chart 25:**
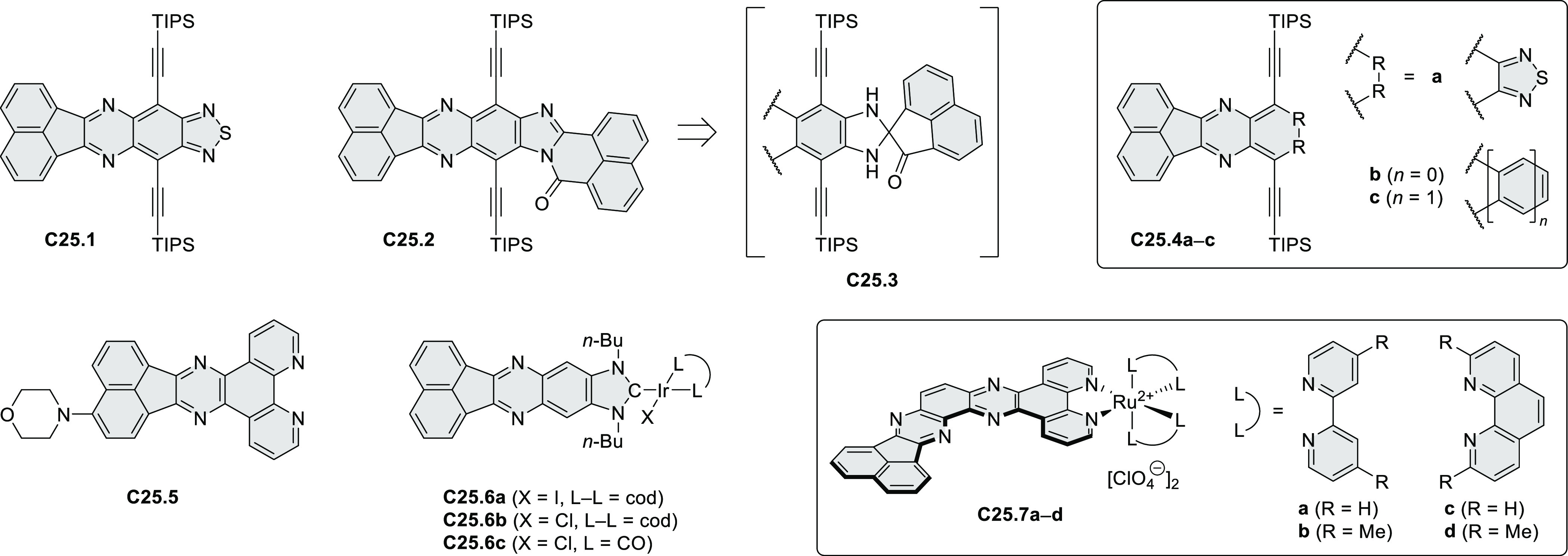
Acenaphthylene-Based Quinoxalines

**Chart 26:**
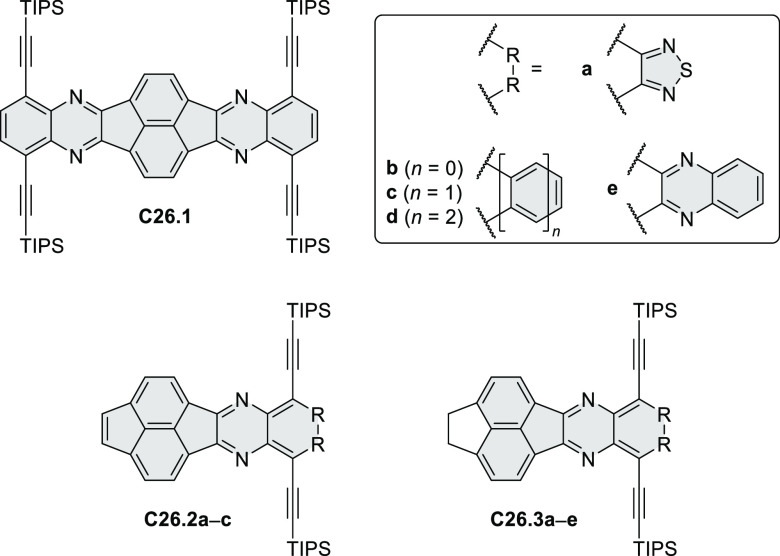
Pyracylene-Based Quinoxalines

The acenaphtho[1,2-*b*]quinoxaline structure is generally prepared via the condensation between acenaphthoquinone and aromatic *ortho*-diamines (cf. CR2017, Section 6.2.1). Recent additions to this class of heteroaromatics are summarized in Chart 25. The thiadiazole-fused compound **C25.1** was shown by Baumgarten et al. to form heat-to-head dimers with intermolecular N–S and weak N–N interactions, and stack at a distance of 3.37–3.39 Å in the solid state.^486^ Compound **C25.2** was an unexpected product characterized by Bunz et al., and was proposed to arise from the ring rearrangement of the penultimate spirocyclic species **C25.3**.^487^ In 2018, Bunz, Dreuw, Freudenburg et al. reported a series of acenaphthylene-based quinoxalines **C25.4a**–**c**.^488^ In particular, among **C25.4a**,**c** and three other pyracylene-based quinoxalines **C26.3a**–**c** tested for organic light-emitting diode (OLED) performance (see Chart 26, section 6.2.7), the green-emitter **C25.4c** had the highest luminance (5.8 × 10^3^ cd m^–2^), maximum efficiency (2.88 cd A^–1^) and efficacy (0.83 lm W^–1^). Compound **C25.5** reported in 2017 by Kothavale and Sekar consists of the electron-donating morpholine group and the electron-accepting pyrazine and phenanthroline units, and was found to exhibit negative acidochromism, i.e., a blue-shifted absorption band upon protonation of the morpholine group.^489^

The acenaphthylene–quinoxaline motif has also been introduced in ligand design (Chart 25). In 2015, Valdes, Poyatos, and Eduardo reported the iridium(I) complexes **C25.6a**–**c** bearing an N*-*heterocyclic carbene (NHC) ligand containing fused acenaphtho[1,2-*b*]quinoxaline (see also Scheme 149 in Section 4.6).^288^ In the crystal structure of **C25.6a**, the NHC ligand was positioned quasi-orthogonally to the coordination plane around the iridium center, with stacking distances of 3.47 Å between adjacent molecules. In 2016, Liu, Xing et al. reported a series of ruthenium(II) polypyridyl complexes **C25.7a**–**d**.^490^ All four complexes featured the same new bidentate ligand containing an acenaphthylene–quinoxaline terminus. The complexes were demonstrated to induce A549 cell apoptosis via a reactive oxygen species (ROS)-mediated mitochondrial dysfunction pathway.

**Scheme 261:**
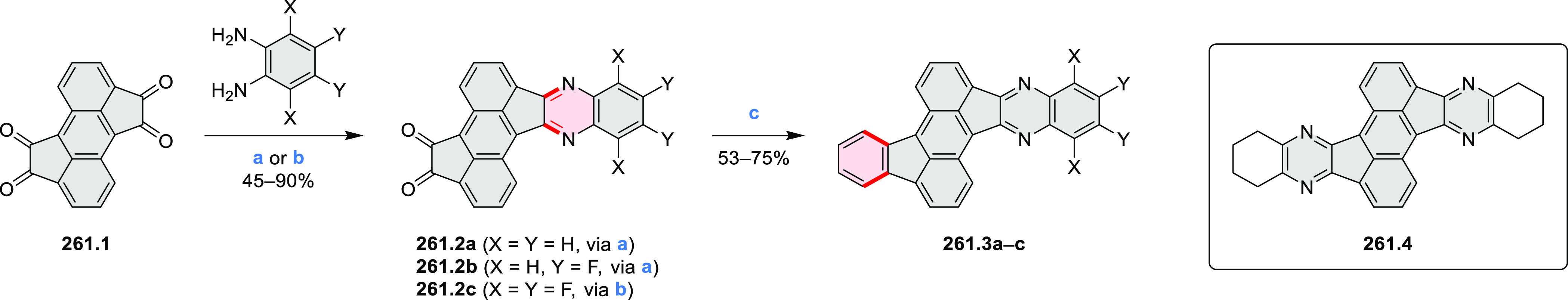
Synthesis of N*-*Doped Rubicenes Reagents and conditions: (a)^491^ pyridine, 90 °C, 16–48 h; (b) Sc(OTf)_3_, 1,2-DCE, 25 °C, 24 h; (c) (1) 1,3-butadiene, Ru_3_(CO)_12_, *t*-Bu_2_PMe·HBF_4_, *t*-BuOK, *i*-PrOH, toluene with or without DMA, 140 °C, 48 h, (2) TsOH, toluene, 90 °C, 16 h.

In 2021, Krische et al. reported a two-step synthesis of the π-extended diazarubicenes **261.3a**–**c** (Scheme 261).^491^ Quinoxalines **261.2a**–**c** were initially obtained by selective condensation of one diketone moiety of **261.1** with an appropriate *ortho*-phenylenediamine (the formation of the tetrafluoro congener **261.2c** required the presence of Sc(OTf)_3_). Compounds **261.2a**–**c** were then subjected to ruthenium-catalyzed transfer hydrogenative benzannulation with 1,3-butadiene to yield the target **261.3a**–**c**. In addition, the tetraaza-substituted rubicene **261.4** was prepared from **261.1** in two steps. The UV–vis spectra of **261.2**–**4** in chloroform were similar and resembled that of the parent rubicene. The absorption and emission peaks were rather insensitive to the variation of solvent (cyclohexane and acetonitrile), indicating the absence of charge-transfer character despite the presence of nitrogen- and/or fluorine-rich regions in these molecules.

**Scheme 262:**
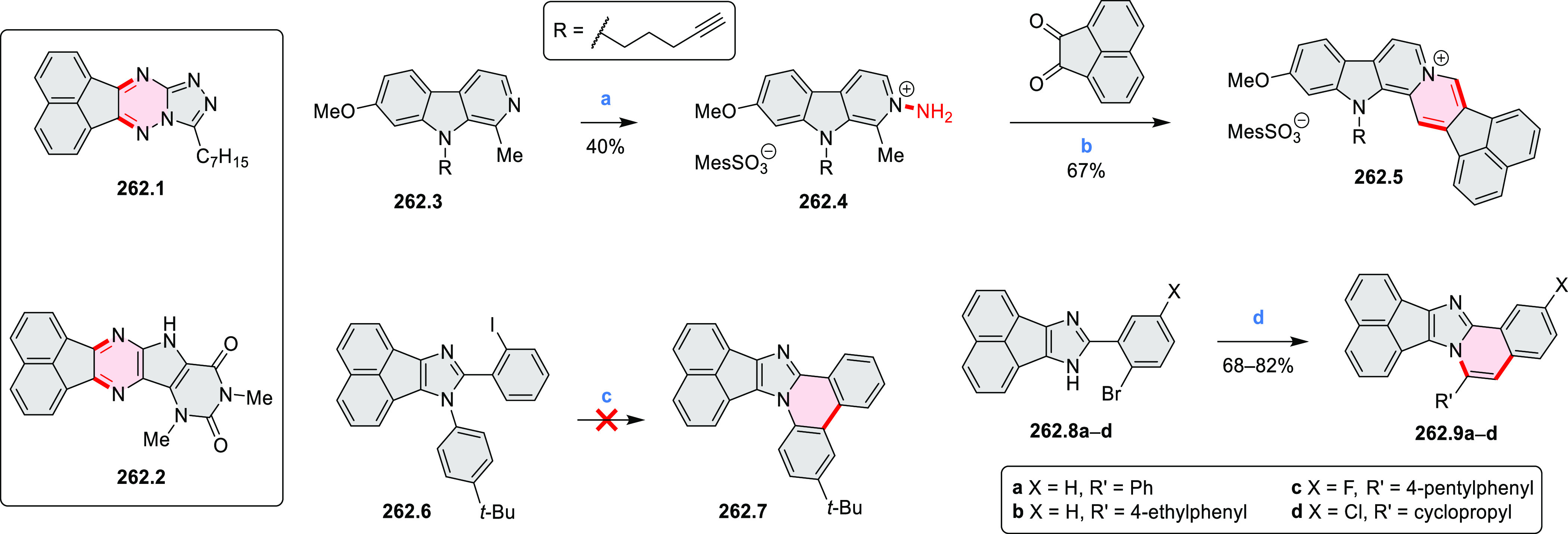
Miscellaneous Acenaphthylenes Hetero[*a*]fused with Nitrogen Heterocycles Reagents and conditions: (a)^493^ MesSO_3_NH_2_, DCM, 1 h; (b) acenaphthoquinone, NaOAc, EtOH, reflux, 1 h; (c)^333^*hν* (254 nm, 6 × 8 W), 48–72 h; (d)^494^ R′C≡CH, CuI (10 mol %), 1,10-phenanthroline (20 mol %), Cs_2_CO_3_ (1 equiv), dioxane, 100 °C, 26–32 h.

Examples of acenaphthylenes [*a*]fused to nitrogen-containing heterocycles are shown in Scheme 262. Fusco and Centore et al. reported the synthesis of compound **262.1** bearing a fused triazolotriazine unit.^342^ This nitrogen-rich polyaromatic skeleton showed a reversible reduction wave in cyclic voltammetry, corresponding to an estimated LUMO level of −3.59 eV (see Scheme 171, Section 4.7.3). In 2017, the preparation of the nitrogen-rich **262.2** from a pyrrolo[3,2-*d*]pyrimidine-based diamine was reported by Popov et al.^492^ Bellón and Alajarín et al. reported the preparation of the new acenaphtho- and indolo-fused quinolizinium salt **262.5** using the base-induced condensation of the *N-*aminopyridinium salt **262.4** with acenaphthoquinone.^493^ This transformation relied on the CH acidity of the vicinal methyl group in **262.4**.

Gang, Wang, and Liu reported a palladium-free Sonogashira coupling reaction for the efficient synthesis of a family of imidazo[2,1-*a*]isoquinolines, such as **262.9a**–**d**, from the corresponding arylated acenaphtho[1,2-*d*]imidazole precursors **262.8a**–**d** (Scheme 262).^494^ The proposed mechanism involved a cascade process of Sonogashira coupling followed by intramolecular hydroamination of the new internal alkyne unit. In another attempt to build a similar π extension on acenaphtho[1,2-*d*]imidazole, the diaryl precursor **262.6** failed to undergo the crystalline-state photochemical direct arylation to generate the fused target **262.7** (for successful examples, see Scheme 165, Section 4.7.2).^333^

**Scheme 263:**
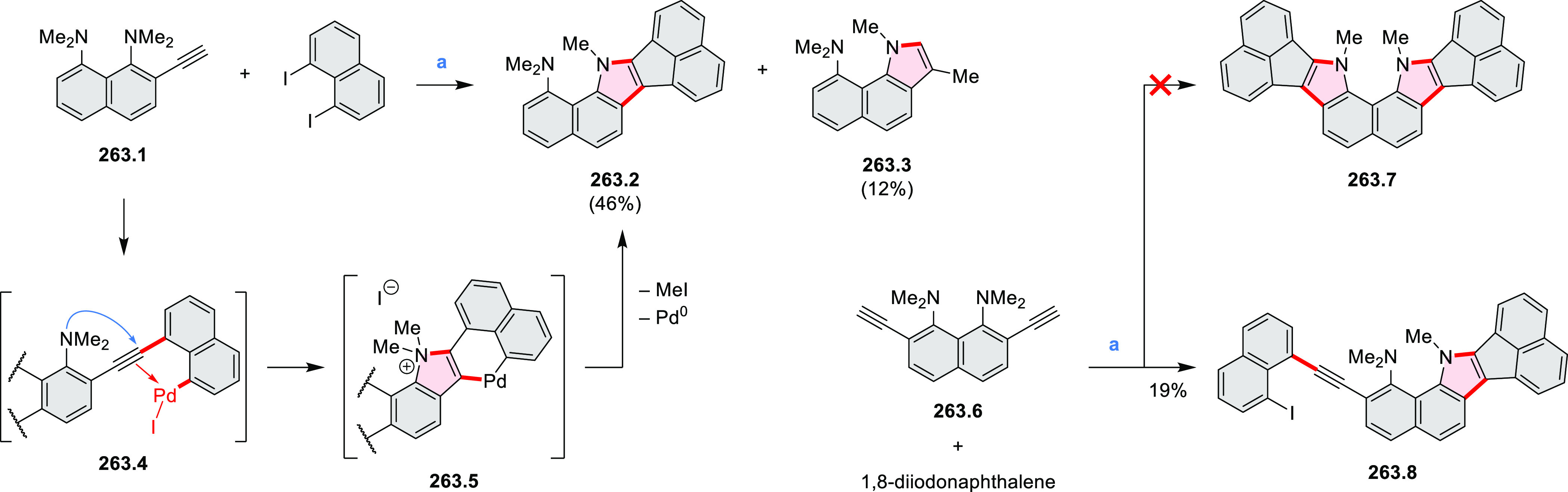
Formation of an Acenaphtho[1,2-*b*]benzo[*g*]indole Skeleton via Cascade Reaction Reagents and conditions: (a)^495^ Pd_2_(dba)_3_, CuI, PPh_3_, K_2_CO_3_, DMF, argon, 60–75 °C, 8–10 h.

In 2018, Gulevskaya et al. reported a cascade reaction between the ethynyl-substituted derivatives of the proton sponge and 1,8-diiodonaphthalene, which generated the acenaphtho[1,2-*b*]benzo[*g*]indole skeleton (Scheme 263).^495^ For instance, compound **263.1** reacted with 1,8-diiodonaphthalene under Sonogashira coupling conditions to give compound **263.2** in 46% yield. The proposed mechanism involved the intermediate **263.4**, which resulted from the initial diarylacetylene product with the remaining C–I bond further activated by palladium. The nucleophilic dimethylamino group induced a bicyclization process, resulting in the palladacycle **263.5**, which led to the final product upon reductive elimination and elimination of methyl iodide. Interestingly, when the diyne **263.6** was submitted to the same reaction with 1,8-diiodonaphthalene, the double fusion product **263.7** was not found, but compound **263.8** was obtained in 19% yield.

**Scheme 264:**
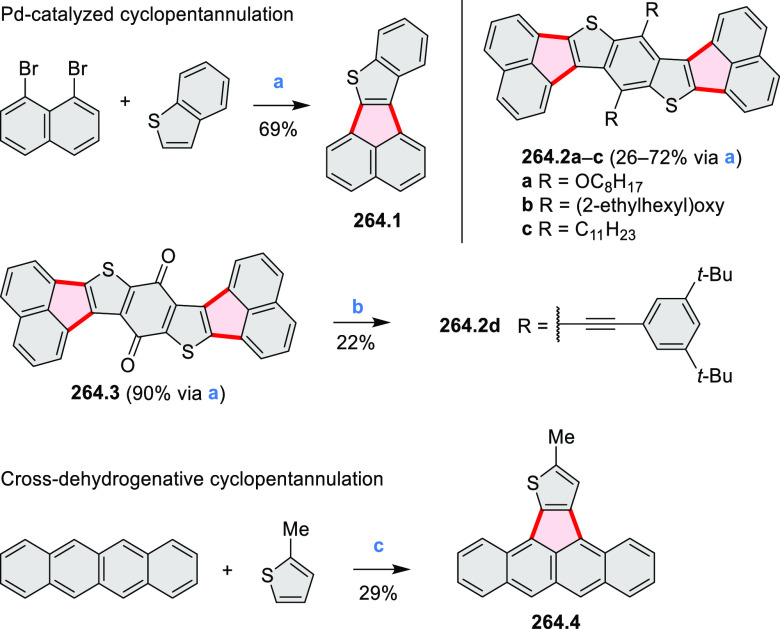
Five-Membered Ring Closures Involving a Thiophene Ring Reagents and conditions: (a)^494^ Pd(OAc)_2_, *t-*Bu_2_PMe·HBF_4_, K_2_CO_3_, DMA, 100 °C, 20 h; (b) (1) 3,5-di-*t*-butylphenylacetylene, *n*-BuLi (premixed at −78 °C for 1 h), then rt for 1 h, THF, rt, overnight, (2) SnCl_2_·H_2_O, 10% aq. HCl, 50 °C, 2 h; (c)^494^*p*-chloranil (1.5 equiv), DCM/TfOH (100/1), rt, 6 h.

In 2017, Li, Hartl, and Yang et al. reported optimized conditions for a palladium-catalyzed C–H activation reaction of benzo[*b*]thiophene with 1,8-dibromonaphthalene, which gave the acenaphthylene product **264.1** in 69% yield (Scheme 264).^496^ By applying this protocol, it was also possible to react fused dithiophenes with 1,8-dibromonaphthalene in a 1:2 ratio, to obtain compounds **264.2a**–**c** and **264.3** in 26–90% yield. Additionally, the *para*-benzoquinone **264.3** could successfully be converted to the diyne **264.2d** in 22%. When an anodic potential was applied to concentrated solutions, the neutral molecules of **264.2a,c**–**d** were found to interact with their own radical cations, to form dimers such as [**264.2a**]_2_^•+^

In 2020, Murata et al. reported a cross-dehydrogenative coupling reaction between tetracene and benzene, using *p*-chloranil as the oxidant in the presence of triflic acid, which was found to produce indeno[1,2,3-*fg*]tetracene.^497^ This method also worked when benzene is replaced with *t*-butylbenzene, fluorobenzene, anisole and 2-methylthiophene. In the last case, compound **264.4** was formed in 29% yield (Scheme 264). Notably, a solution of **264.4**, when illuminated with artificial light under air, showed negligible photodegradation as monitored by absorption spectroscopy. This contrasted with the behavior of rubrene, which is much more photolabile. In 2017, Murata and Murata et al. reported the synthesis of the doubly fused tetracene **281.3** (for synthesis, see Scheme 281, section 6.2.7),^498^ which is a π-extended analogue of **264.4**.

**Scheme 265:**
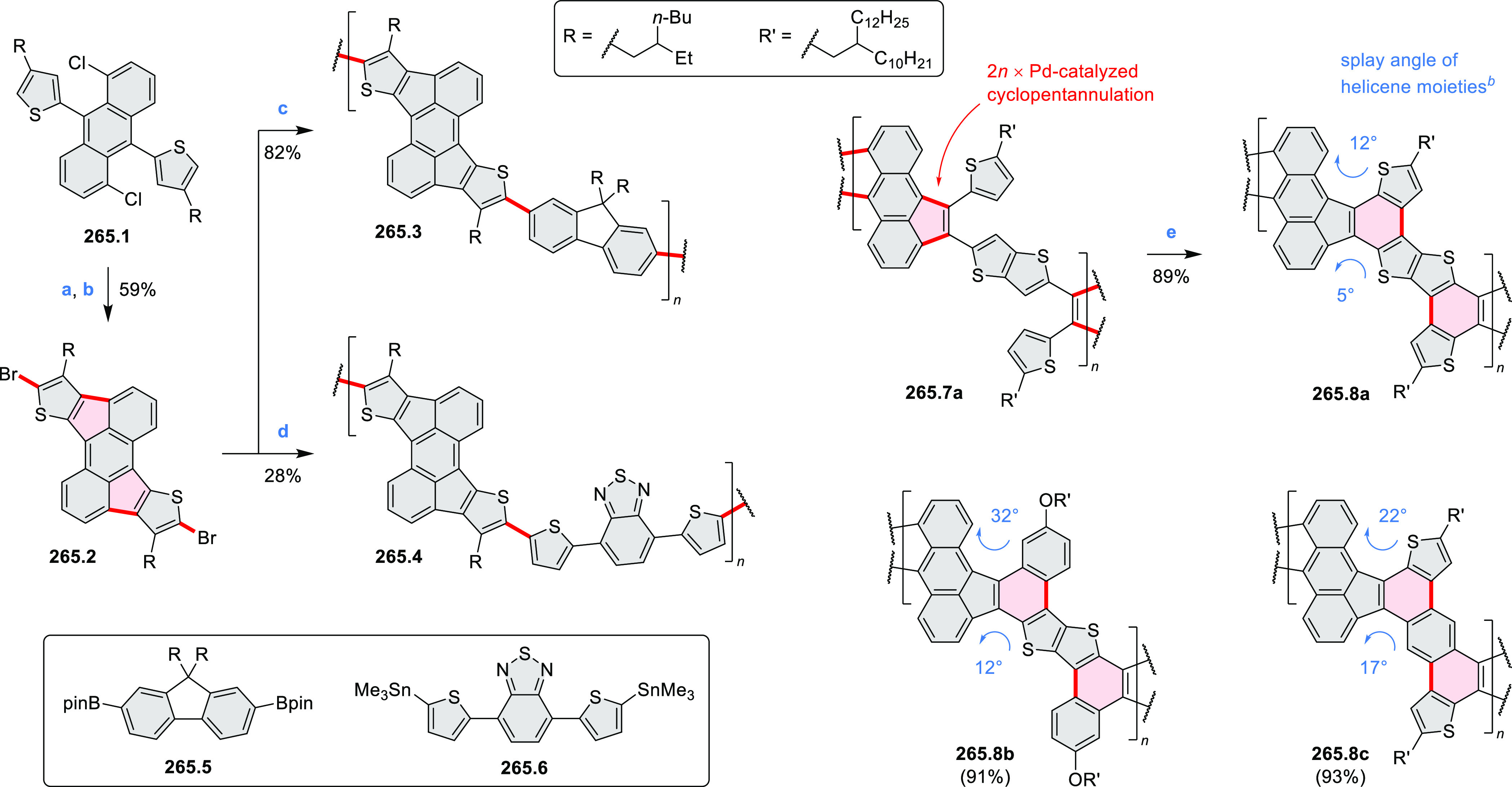
Thiophene-Fused Rubicene-Based Conjugated Polymers^,^ Reagents and conditions: (a)^502^ Pd(OAc)_2_ K_2_CO_3_, *n*-Bu_4_NHSO_4_, MeCN, reflux, 24 h; (b) NBS, THF, 0–35 °C, 12 h; (c) **265.5**, Pd(PPh_3_)_4_, K_2_CO_3_, toluene, 110 °C, microwave, 12 h; (d) **265.6**, Pd(PPh_3_)_2_Cl_2_, toluene, 110 °C, microwave, 12 h; (e)^503^ FeCl_3_, DCM/MeNO_3_, argon, rt, overnight Only one out of three possible isomeric constitutions of the repeat unit is shown for polymers **265.7a** and **265.8a**–**c**. Calculation was performed on the repeat unit of the structures shown (B3LYP/6-31G**). Alternative regioisomeric repeat units were also calculated.

In 2017, Jeng et al. reported the preparation of conjugated polymers **265.3**–**4** based on the emeraldicene core^499−501^ and examined their optoelectronic and photovoltaic properties (Scheme 265).^502^ To synthesize the monomer, compound **265.1** was subjected to palladium-catalyzed cyclopentannulation followed by dibromination to give **265.2** in an overall yield of 59%. Subsequent copolymerization via Suzuki–Miyaura coupling with **265.5** or via Stille coupling with **265.6** furnished the corresponding polymers **265.3**–**4** in 82% and 28% yield, respectively. The visible-light-transparent emeraldicene–fluorene copolymer **265.3** absorbed mostly in the UV and NIR regions. For the polymer solar cell device constructed using **265.3**, a power conversion efficiency (PCE) of 2.5% was demonstrated by the authors. When fabricated with a cross-linker additive, the device showed a greatly enhanced thermal stability.

In 2017, Plunkett et al. reported the preparation of the conjugated ladder polymers **265.8a**–**c** featuring singly or doubly thieno-fused rubicene units (Scheme 265).^503^ They were prepared from the oxidative six-membered ring closure of the corresponding copolymers (such as **265.7a**) using FeCl_3_/MeNO_2_ in 89–93% yield. Because the cyclopentannulation was not regioselective, the precursor polymer **265.7a**, as well as the cyclized product **265.8a**, contained regioisomeric repeat units. Polymer **265.8a** was essentially insoluble, except when heated in high-boiling chlorinated solvents. According to DFT calculations, the repeating units of **265.8a** are noticeably flatter than those of **265.8b**,**c**, as quantified by the smaller splay angles of the thiahelicene units in **265.8a**.

**Scheme 266:**
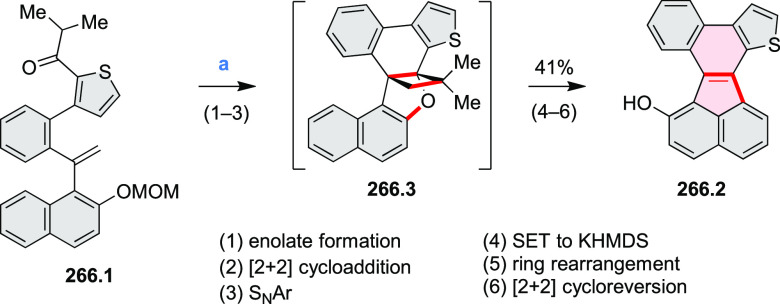
Thieno[*j*]fluoranthenes Reagents and conditions: (a)^504^ KHMDS (3 equiv), *cis*-1,2-cyclohexanediol (20 mol %), diglyme, reflux, 24 h.

In 2017, Takasu et al. reported a series of π-extended fluoranthenes through a KHMDS-promoted anionic-radical cascade.^504^ As exemplified by the thiophene-fused congener **266.2**, the precursor **266.1** was refluxed in diglyme in the presence of KHMDS (3 equiv) and *cis*-1,2-cyclohexanediol (20 mol %) to afford the doubly cyclized product in 41% yield (Scheme 266). It was suggested that KHMDS plays two roles in this cascade reaction. First, the strong base deprotonates **266.1** to give the corresponding enolate, which undergoes intramolecular [2 + 2] cycloaddition followed by nucleophilic displacement to yield the key intermediate **266.3**. Second, KHMDS functions as a one-electron reductant. It provides an electron to **266.3** via single electron transfer (SET), thereby inducing subsequent ring rearrangement of **266.3**^•–^.

#### 6.2.2 Hetero[e]fused Acenaphthylenes

**Scheme 267:**
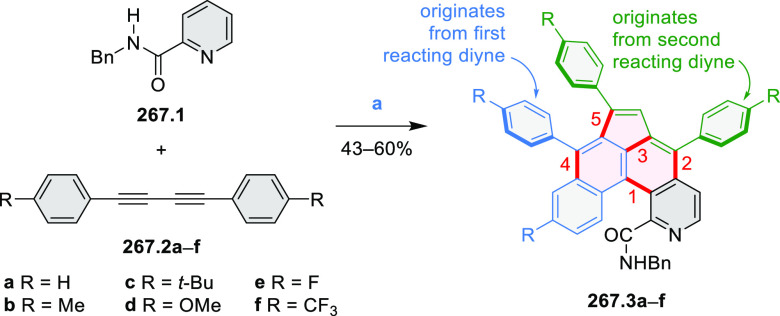
Hetero[*e*]fused Acenaphthylenes from C–H Annulation Reactions^,^ Reagents and conditions: (a)^505^ [RhCp*Cl_2_]_2_ (20 mol %), AgSbF_6_ (40 mol %), Cu(OAc)_2_ (4.0 equiv), dioxane, 130 °C, 3 h; (b)^284^ (1) Tf_2_O, 1,2-DCE, −30 °C, 20 min, then rt, 1 h, (2) microwave, 90 °C, 12 h, (3) Et_3_N, rt, 20 min, then 50 °C, 5 h. The sequence of formation of the five new C–C bonds determined by DFT calculation for **267.3a** is shown in red.

A series of fused pentacyclic compounds such as **267.3a**–**f** were prepared in an intermolecular domino C–H annulation reaction between the picolinamide **267.1** and 2 equiv of a 1,4-diaryl-1,3-butadiyne **267.2a**–**f** (Scheme 267).^505^ The reaction showed high regioselectivity, producing five new C–C bonds and four fused rings in a single operation. To proceed with improved selectivity, the transformation required [RhCp*Cl_2_]_2_ (20 mol %), AgSbF_6_ (40 mol %), and excess Cu(OAc)_2_ (4.0 equiv). This Rh^III^-catalyzed Cu^II^-assisted annulation pathway was studied using DFT calculations, and the proposed sequence of C–C bond formation is shown in Scheme 267.

**Scheme 268:**
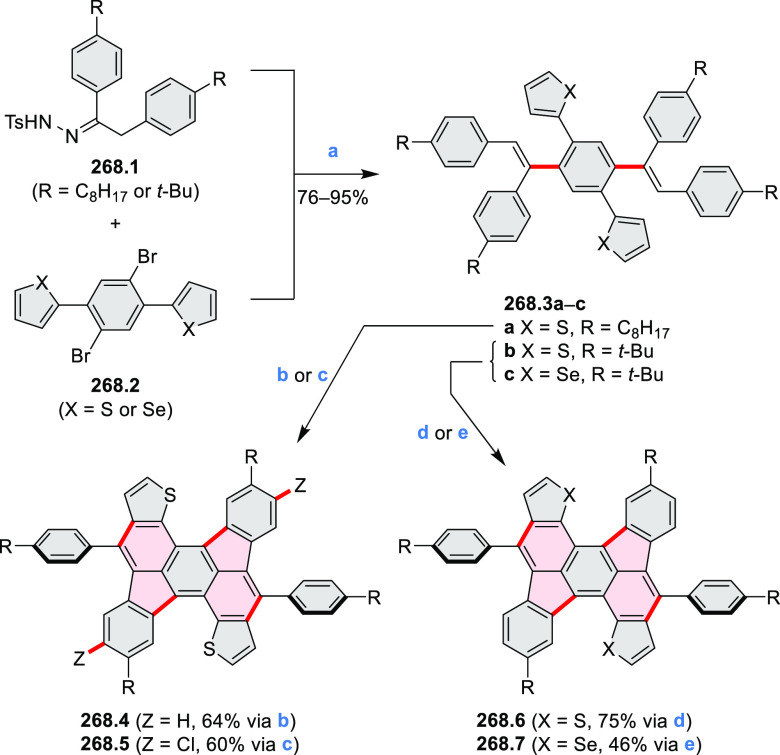
Hetero[*e*]fused Acenaphthylenes from C–H Annulation Reactions Reagents and conditions: (a)^506^ Pd(PPh_3_)_4_ (5 mol %), *t*-BuOLi (2 equiv), K_2_CO_3_ (1 equiv), KOAc (1 equiv), dioxane, N_2_, 100 °C, 24 h; (b) FeCl_3_, DCM/MeNO_2_, 0 °C; (c) FeCl_3_, DCM/MeNO_2_, rt; (d) FeCl_3_, DCM/MeNO_2_, 0 °C, 15 min, then rt, 2.5 h; (e) (1) DDQ, DCM, 0 °C, 10 min, (2) TfOH, 10 min.

Shi, Huang, and Blakey et al. recently explored the reactivity of heterocycle-substituted diolefins **268.3a**–**c** in oxidative coupling reactions (Scheme 268).^506^ These three compounds were synthesized via the known palladium-catalyzed cross-coupling of the dibromides **268.2** with the corresponding *N-*tosylhydrazones **268.1** in a 1:2 stoichiometry (76–95% yield). Compound **268.3a** underwent 4-fold cyclodehydrogenation when treated with FeCl_3_/MeNO_2_ at 0 °C, to give the dithieno-fused rubicene **268.4** in 64% yield. When the same reaction took place at rt, further aromatic chlorination occurred to give the dichloride **268.5** in 60% yield. The *tert*-butyl-substituted precursors **268.3b**,**c** were cyclized without chlorination, to furnish to the products **268.6** (using FeCl_3_/MeNO_2_) and **268.7** (using DDQ/TfOH). In the absorption spectra, a red-shift of 13 nm was observed for the selenophene-fused **268.7** relative to the thiophene-fused congener **268.6**. This phenomenon was ascribed to the greater quinoidal character of the π-system containing the selenium atom. Based on femtosecond transient absorption experiments, the accelerated intersystem crossing from the S_1_ state to the T_1_ state was confirmed for the Se-containing **268.7** in comparison to **268.6**.

**Scheme 269:**
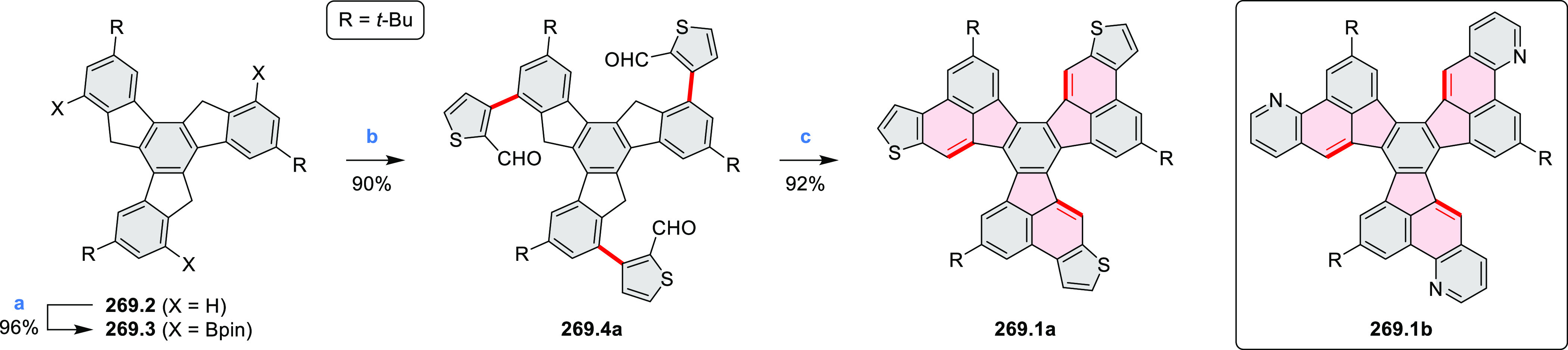
Synthesis of Heterocycle-Fused π-Extended Truxenes Reagents and conditions: (a)^507^ B_2_pin_2_, [Ir(OMe)(cod)]_2_, 4,4′-di-*t*-butyl-2,2′-bipyridine, *t*-BuOK, THF, 80 °C, 24 h; (b) 3-bromothiophene-2-carboxaldehyde, Pd_2_(dba)_3_ (10 mol %), *t-*Bu_2_PMe·HBF_4_ (17 mol %), K_2_CO_3_, THF/H_2_O (4:1), 80 °C, 16 h; (c) *t*-BuOK, THF, 60 °C, 16 h.

In 2016, Mastalerz et al. reported the preparation of a series of π-extended truxene derivatives, including two heterocycle-fused analogues **269.1a**,**b** (Scheme 269).^507^ The first key step in their work was the 3-fold iridium-catalyzed direct borylation of the tri-*tert*-butyl-substituted truxene **269.2**, which took place selectively at the least hindered aromatic sites to give **269.3** in 96% yield. Subsequent Suzuki–Miyaura cross-coupling of **269.3** with 3-bromothiophene-2-carboxaldehyde led to the trialdehyde **269.4a** in excellent yield. The second key step involved the condensation between the aldehyde and methylene groups in the presence of *t*-BuOK to give the product of 3-fold cyclization **269.1a** in 92% yield. Likewise, the pyridine-fused analogue **269.1b** was prepared from the triboronate **269.3** in 64% yield over two steps. The α-positions of the thiophene rings in **269.1a** were easily functionalizable via bromination and borylation.

#### 6.2.3 Hetero[d]fused Acenaphthylenes

**Scheme 270:**
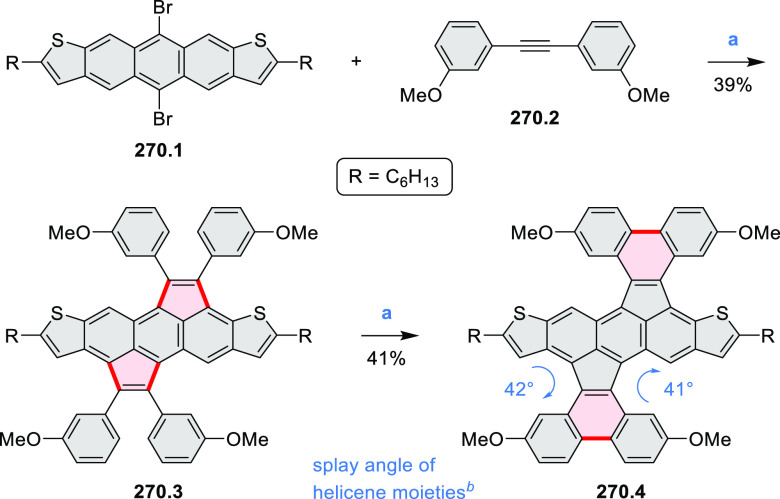
Synthesis of a Contorted Polyarene in a Cyclopentannulation–Cyclodehydrogenation Sequence^,^ Reagents and conditions: (a)^508^ Pd_2_(dba)_3_, tri(*o*-tolyl)phosphine, KOAc, LiCl, DMF/toluene, 130 °C; (b) FeCl_3_, DCM/MeNO_2_. Level of theory: B3LYP/6-311G(d,p).

In 2018, Plunkett et al. reported the two cyclopentannulated anthracenedithiophenes **270.3** and **270.4**, the latter being the cyclodehydrogenation product of the former (Scheme 270).^508^ The five-membered rings in compound **270.3** were constructed through the 2-fold palladium-catalyzed cyclopentannulation of the dibromide **270.1** with the diarylacetylene **270.2** in 39% yield. The DFT-optimized geometry of **270.3** had a contorted structure with large splay angles of roughly 40° around the (thia)[5]helicene moieties (cf. Scheme 265, Section 6.2.1). The absorption edge of **270.4** at about 830 nm was significantly bathochromically shifted relative to that of **270.3** (670 nm). Moreover, the LUMO energy of the π-system was estimated by cyclic voltammetry to drop from −3.44 to −3.70 eV upon six-membered ring closure.

#### 6.2.4 Carbazole-Based and Related Systems

**Scheme 271:**
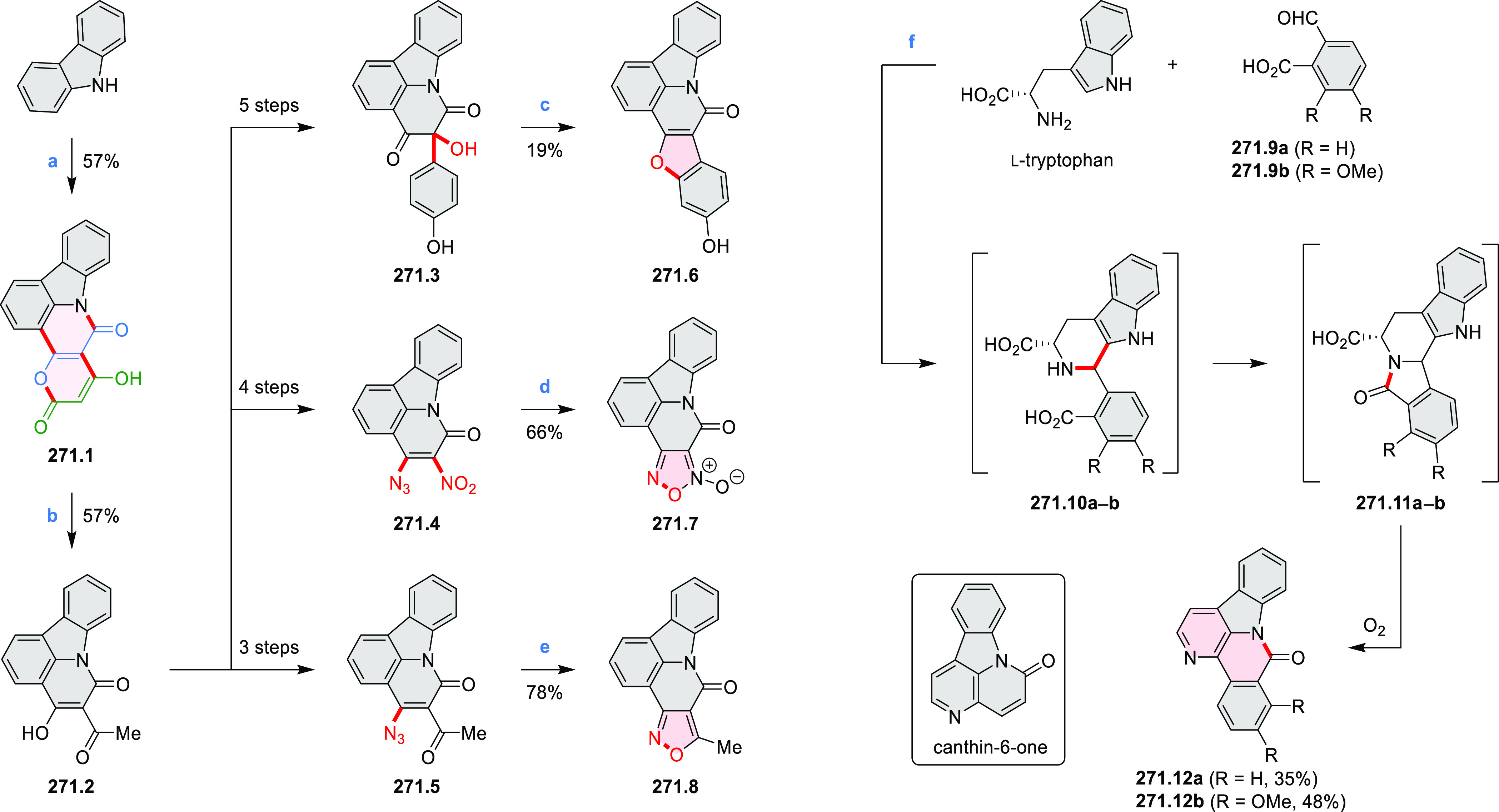
Lactam-Fused Carbazole Derivatives^,^ Reagents and conditions: (a)^509^ diethyl malonate (3 equiv), diphenyl ether, 250–260 °C, 7 h; (b) NaOH, ethylene glycol, 180 °C, 3 h; (c) P_2_O_5_, MsOH, 150 °C, 20 min; (d) DMF, reflux, 2 h; (e) bromobenzene, reflux, 3 h; (f)^510^ AcOH, reflux, air, 30–40 h. The chains of atoms in **271.1** that originate from the first and second reacting diethyl malonate molecules are shown in blue and green, respectively.

Cyclocondensation of carbazole with diethyl malonate in a 1:2 ratio was reported to yield compound **271.1** (Scheme 271).^509^ When **271.1** was heated with NaOH in ethylene glycol, the lactone ring opening followed by decarboxylation gave rise to compound **271.2**, which could be derivatized into the three compounds **271.3**–**5** in subsequent steps. Compound **271.3** underwent cyclodehydration in the presence of Eaton’s reagent to give the benzofuran-fused product **271.6** (19% yield), while the azides **271.4** and **271.5** could undergo thermal electrocyclization to give, respectively, the furazan *N-*oxide **271.7** (66% yield) and the isoxazole **271.8** (78% yield).

In 2017, Garazd et al. reported the formation of the *peri*-fused carbazole analogues **271.12a**,**b**, when a solution of l-tryptophan and 2-formylbenzoic acid (**271.9a**) or opianic acid (**271.9b**) in acetic acid was heated to reflux for 30–40 h (Scheme 271).^510^ The initial Pictet–Spengler reaction product **271.10a**,**b** was proposed to further cyclize to give the lactam **271.11a**,**b**, which furnished the observed products upon decarboxylative ring rearrangement in the presence of atmospheric oxygen. Compounds **271.12a**,**b** represent benzannulated analogues of the natural product canthin-6-one.

**Scheme 272:**
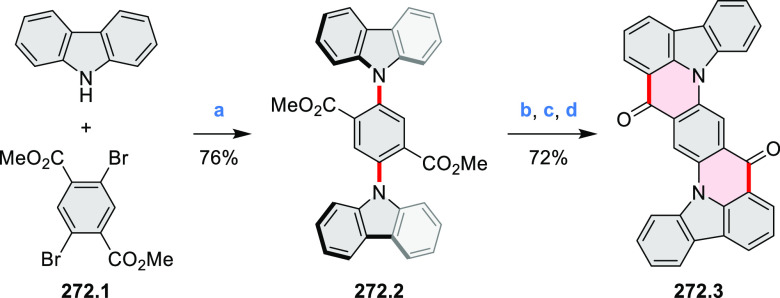
Synthesis of a Diindole-Fused Azapentacenone Reagents and conditions: (a)^511^ CuI, K_2_CO_3_, 18-crown-6, *o*-dichlorobenzene, 180 °C, N_2_, 24 h; (b) (1) NaOH, MeOH, reflux, 10 h, then aq. HCl; (c) DMF (cat.), SOCl_2_, reflux, 1 h; (d) AlCl_3_, DCM, rt, 12 h.

In 2016, Huang, Yu, and Xie et al. reported the preparation of the diindole-fused azapentacenone **272.3** using an improved procedure (Scheme 272).^511^ First, the commercially available **272.1** was subjected to the copper(I)-catalyzed Ullmann-type reaction with carbazole to give **272.2** in 76% yield. Afterward, **272.2** was hydrolyzed and activated by SOCl_2_. Subsequent 2-fold intramolecular Friedel–Crafts acylation led to the ring-closed compound **272.3** in 72% yield. Based on various data, the performance of **272.3** as an organic cathode material for lithium-ion battery was concluded to be superior to the parent azapentacenone without the two *peri*-fusions.

**Scheme 273:**
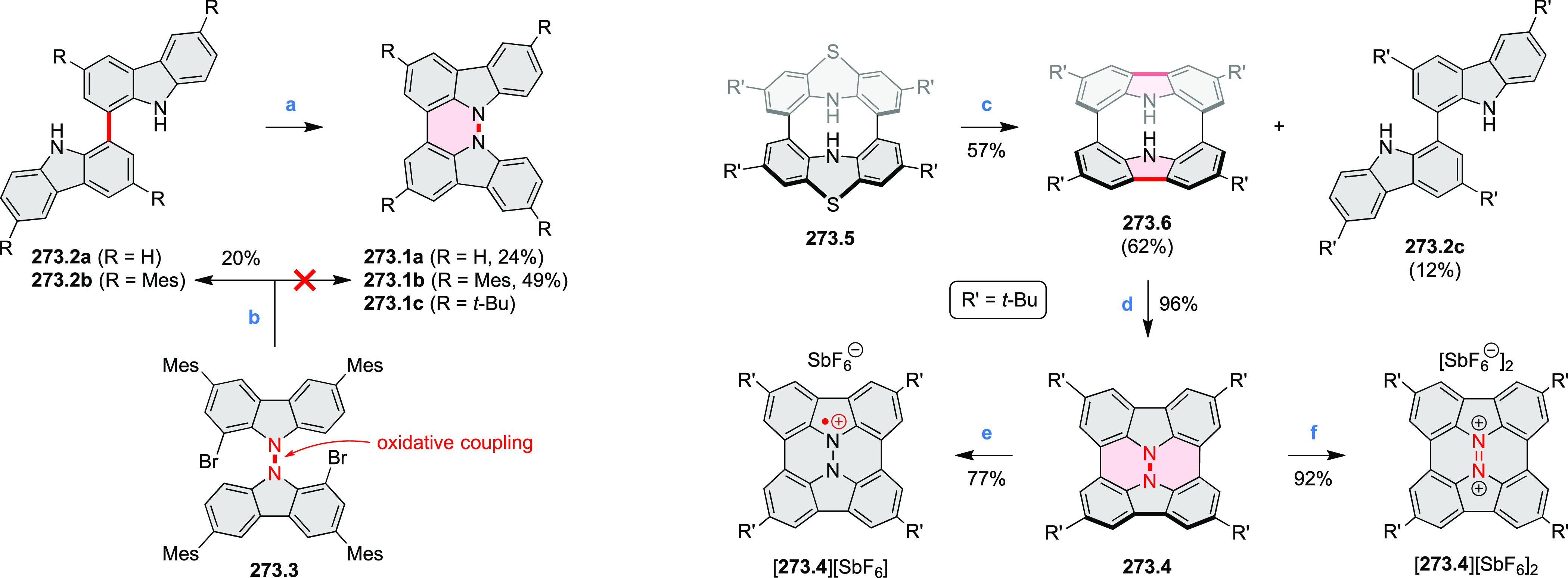
Fused Bicarbazoles Reagents and conditions: (a)^512^ [*n*-Bu_4_N][MnO_4_], pyridine, 70 °C, 24 h; (b) Ni(cod)_2_ (150 mol %), cod (150 mol %), 2,2′-bipyridyl (150 mol %), THF, 55 °C, 24 h; (c)^513^ naphthalene, Li, THF, 0 °C, 1 h; (d) (1) DDQ, DCM, 0 °C, then rt, 2 h, (2) N_2_H_4_·H_2_O, 0 °C; (e) NOSbF_6_ (1.0 equiv), DCM/MeCN, rt, 1 h; (f) NOSbF_6_ (2.1 equiv), DCM/MeCN, rt, 30 min.

In 2015, Higashibayashi et al. reported two new pyridazinodicarbazoles **273.1a**,**b**, in addition to the previously known **273.1c** (Scheme 273).^512^ Compounds **273.1a**,**b** were obtained through oxidative N–N bond formation in 1,1′-bicarbazoles **273.2a**,**b**, induced using [*n*-Bu_4_N][MnO_4_]. Interestingly, when 9,9′-bicarbazole **273.3** was subjected to the Yamamoto coupling conditions, the cyclized product **273.1b** was not obtained, but the 1,1′-bicarbazole **273.2b** was isolated in 20% yield, implying the occurrence of a reductive N–N bond cleavage. In 2016, the same group reported the synthesis and redox chemistry of the hydrazinobuckybowl **273.4**.^513^ The known compound **273.5** was reductively desulfurized using naphthalene and metallic lithium to give [2]cyclo-1,8-carbazolylene **273.6** and the further C–C bond cleavage side product **273.2c** in 62% and 12% yield, respectively. The former product underwent a DDQ-mediated N–N bond formation, followed by reductive quenching with hydrazine, to yield the hydrazinobuckybowl **273.4** almost quantitatively (for an alternative approach, see Scheme 388, Section 7.7.2). The chemical oxidation of **273.4** with 1.0 or 2.1 equiv of NOSbF_6_ led to the formation of the SbF_6_^–^ salts of the radical cation [**273.4**]^•+^ and the dication [**273.4**]^2+^, respectively. In the crystalline state, the skeletons of **273.4**, [**273.4**]^•+^, and [**273.4**]^2+^ adopted a twisted, bowl-shaped, and planar geometry, respectively.

#### 6.2.5 Carbonyl-Free Azafluoranthenes

**Scheme 274:**
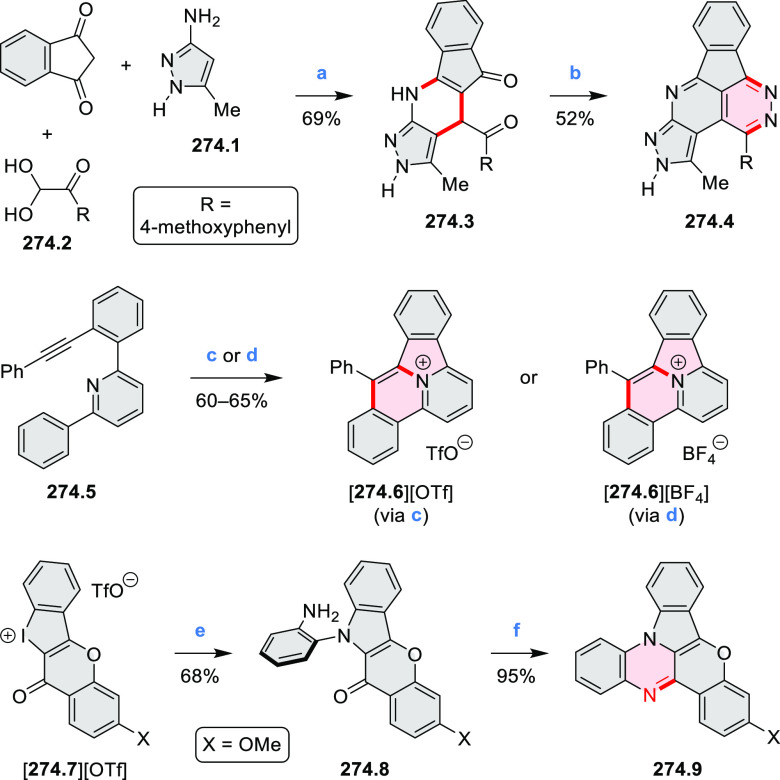
Carbonyl-Free Azafluoranthene Derivatives Reagents and conditions: (a)^514^ EtOH, reflux, 1 h; (b) N_2_H_4_·H_2_O, EtOH, reflux, 8 h; (c)^515^ Cu(OTf)_2_ (100 mol %), MeCN, 80 °C, 12 h; (d) Cu(OTf)_2_ (10 mol %), Selectfluor (1.0 equiv), MeCN, 80 °C, 12 h; (e)^517^*ortho*-phenylenediamine (2.5 equiv), Na_2_CO_3_ (3 equiv), Cu(OAc)_2_ (0.1 equiv), *i*-PrOH/ethylene glycol (9:1), argon, reflux, 16 h; (f) TsOH·H_2_O (0.1 equiv), EtOH, reflux.

In 2015, Lipson et al. reported a three-component synthesis of extended pyrazoles of the general structure **274.3** (R = aryl), obtained via cyclocondensation of 5-amino-3-methylpyrazole (**274.1**), an arylglyoxal hydrate (**274.2**), and 1,3-indanedione (Scheme 274).^514^ Subsequent condensation with hydrazine hydrate performed for the *p*-methoxyphenyl derivative was shown to provide access to the pyrazole-fused triazafluoranthene **274.4** in 52% yield.

In 2018, Patil et al. developed a library of cationic nitrogen-doped polycyclic arenes via copper-promoted intramolecular [4 + 2] cycloaddition cascade.^515^ Upon heating with an equimolar amount of Cu(OTf)_2_ in acetonitrile, **274.5** was transformed to the doubly cyclized quaternary ammonium salt [**274.6**][OTf] in 60% yield (Scheme 274). When an external oxidant (Selectfluor) was added, the use of a catalytic amount of Cu(OTf)_2_ sufficed to mediate the same bicyclization to give [**274.6**][BF_4_] in a similar yield. Substituted and fused analogues of [**274.6**][OTf] were also synthesized and were found to exhibit tunable emission wavelengths. It was later found that the outcome of the reaction depends on the oxidant; in particular, the use of a hypervalent iodine reagent led to spirocyclized products.^516^

In 2019, Wen et al. reported the double amination of heterocyclic iodonium cations with primary amines as a general method to construct polycyclic heteroaromatic fragments which might be useful in drug discovery.^517^ For instance, the reaction of the chromone-fused iodonium cation **274.7**^+^ with *ortho*-phenylenediamine in the presence of Cu(OAc)_2_ and Na_2_CO_3_ resulted in the fused indole derivative **274.8** in 68% yield (Scheme 274). Further acid-catalyzed cyclization of **274.8** produced the *peri*-fused heteroarene **274.9** in 95% yield. (See also Scheme 295, Section 6.4.2)

#### 6.2.6 Miscellaneous Azaacenaphthylenes and Azafluoranthenes

**Scheme 275:**
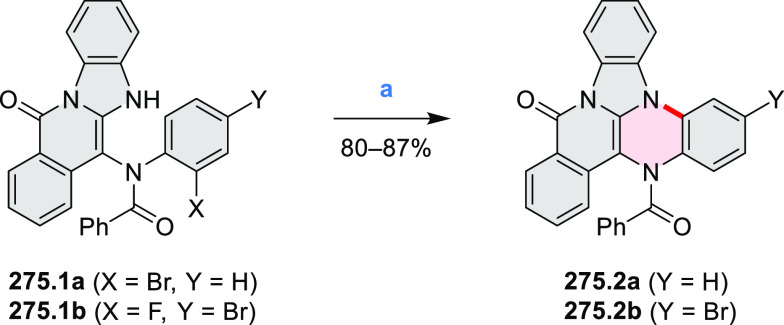
Synthesis of a Fused Benzimidazoisoquinoline–Quinoxaline Reagents and conditions: (a)^518^ Cs_2_CO_3_, CuI, DMF, microwave, 150 °C, 10 min.

In 2016, Xu and Chen et al. reported a one-pot, four-component Ugi procedure for the synthesis of a series of benzimidazoisoquinolines including **275.1a**,**b** (Scheme 275).^518^ The *ortho*-halophenyl groups in these two compounds could further react with the NH group of the benzimidazole unit in an Ullmann-type reaction. Consequently, the quinoxaline-fused products **275.2a**,**b** were isolated in 80–87% yield (for a related product with a seven-membered ring, see Scheme 295, Section 6.4.2).

**Scheme 276:**
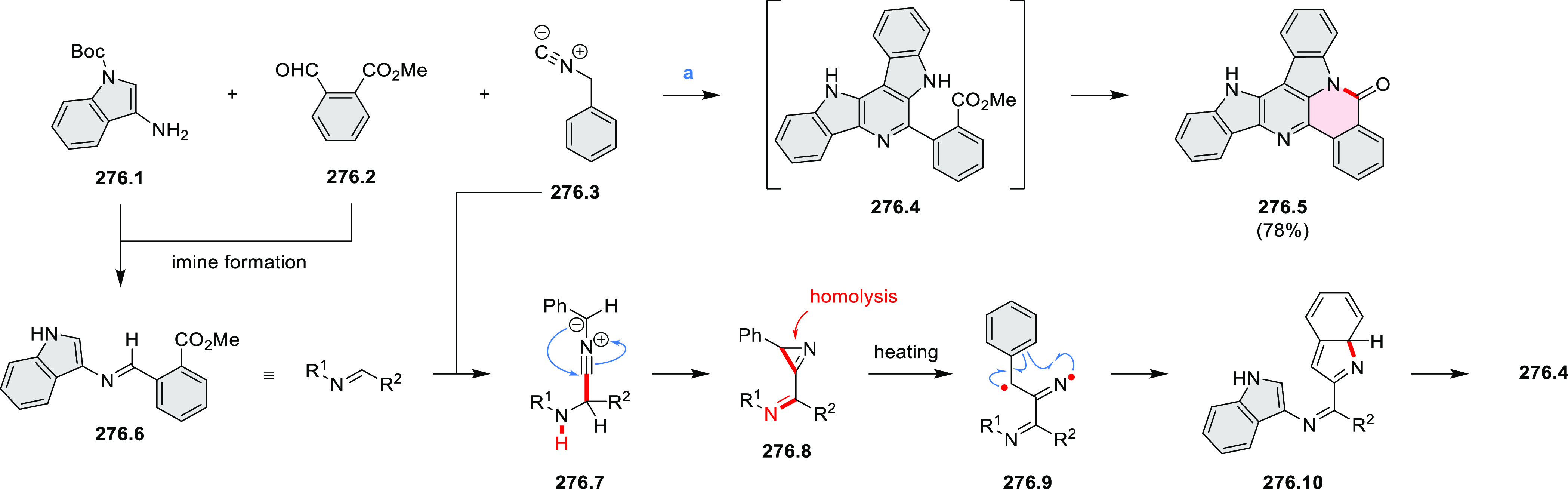
Three-Component Cascade Condensation Reagents and conditions: (a)^519^ 10% HClO_4_, MeOH, microwave, 100 °C, 10 min.

Another three-component cascade reaction, developed by Xu and Li et al. for the synthesis of pyridodiindoles, involved a condensation of the 1-Boc-protected 3-aminoindole (**276.1**), a substituted benzaldehyde, and benzyl isocyanide (**276.3**) (Scheme 276).^519^ The reaction required 10% perchloric acid in methanol under microwave heating at 100 °C. When methyl 2-formylbenzoate (**276.2**) was used as the aldehyde component, the expected aryl-substituted pyridodiindole **276.4** was found to further cyclize to give the lactam **276.5** in 78% yield. In the mechanism proposed by the authors, the imine **276.6** formed from **276.1** and **276.2** is attacked by the nucleophilic carbon atom of benzyl isocyanide to give **276.7**, which undergoes a dehydrogenative imine formation and a pseudo-Neber rearrangement to give the azirine **276.8**. Cleavage of the C–N single bond yields the intermediate **276.9**. The latter species rearranges to **276.10**, which further oxidatively cyclizes to give the penultimate intermediate **276.4**.

**Scheme 277:**
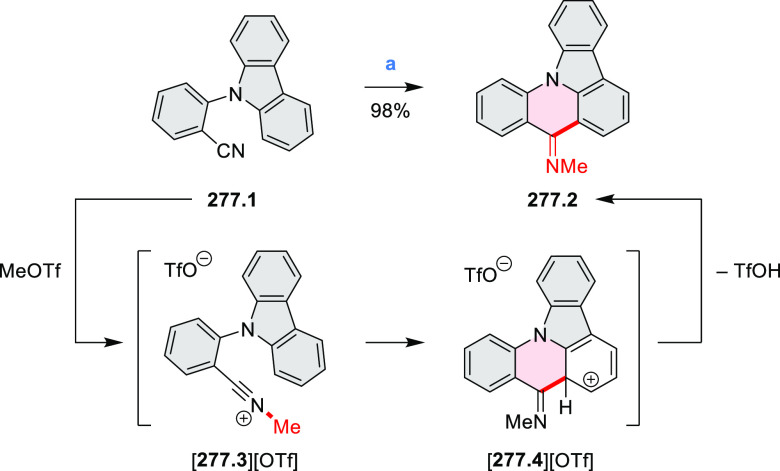
MeOTf-Induced Cyclization of *N-*(2-Cyanophenyl)carbazole Reagents and conditions: (a)^520^ MeOTf (1.2 equiv), 1,2-DCE, air, 90 °C, 24 h.

In 2019, Xi et al. reported the MeOTf-induced annulation of *N-*(2-cyanoaryl)indoles to give the corresponding indolo[1,2-*a*]indol-10-imines.^520^ When applied to the substrate *N-*(2-cyanophenyl)carbazole (**277.1**), the same protocol produced the indole-fused acridinimine **277.2** nearly quantitatively (Scheme 277). The proposed mechanism involves an initial activation of the nitrile group upon methylation. The resultant intermediate **277.3**^+^ undergoes electrophilic cyclization to give **277.4**^+^, which eventually gives the target molecule upon deprotonation.

**Scheme 278:**
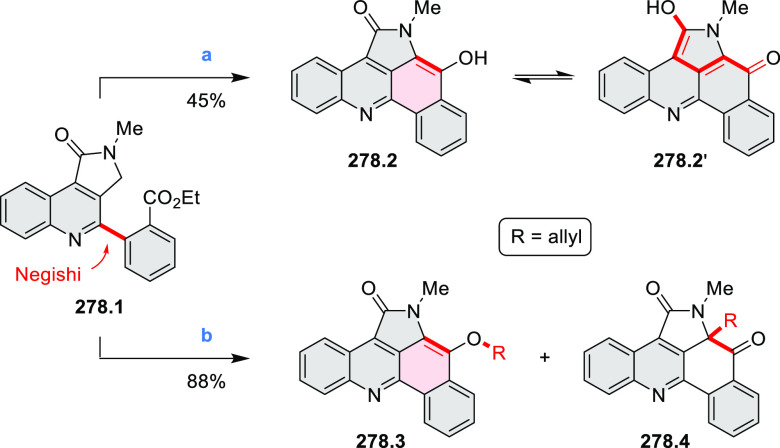
Base-Promoted Cyclization toward an Alpkinidine Analogue Reagents and conditions: (a)^521^ NaHMDS, THF, −95 °C, 10 min, then rt, 4 h; (b) (1) NaOEt, EtOH, rt, 2 h, (2) allyl bromide, 6 h.

In 2019, Tilve et al. reported the cyclization of compound **278.1** in the presence of NaHMDS to give the known compound **278.2** (45% yield), which is an analogue of the natural product alpkinidine (Scheme 278).^521^ In an attempt to increase the cyclization yield by introducing an alkyl chain, **278.1** was subjected to cyclization using sodium ethoxide following by treatment with allyl bromide. The combined yield of the *O*- and *C*-allylated polycycles **278.3**–**4** was 88%.

**Scheme 279:**
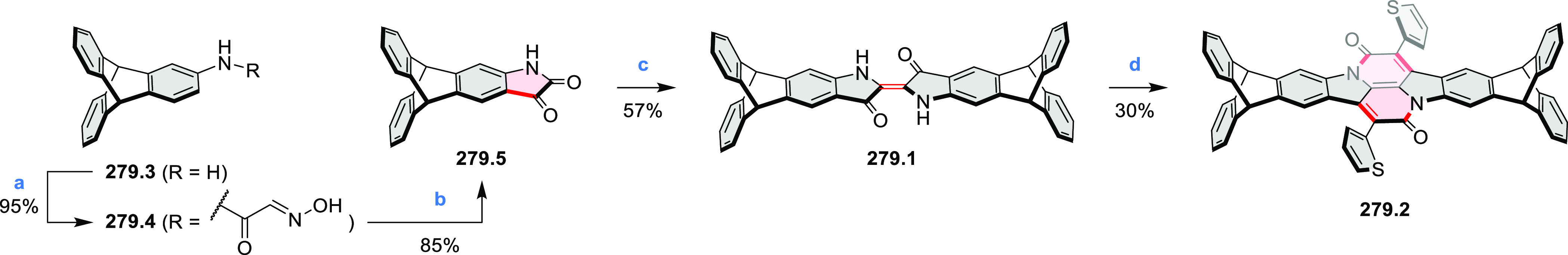
Synthesis of Triptycene-Capped Indigo Derivatives Reagents and conditions: (a)^522^ Cl_3_CCH(OH)_2_, Na_2_SO_4_, H_2_O, 50 °C, then conc. HCl, 5 min, (2) aq. NH_2_OH·HCl, 80 °C, 20 h; (b) MsOH, 50 °C, 4 h; (c) (1) PCl_5_, toluene, 100 °C, 4 h, (2) PhSH, 50 °C, 16 h; (d) 2-thiopheneacetyl chloride, *o*-xylene, 155 °C, 48 h.

In 2021, Mastalerz et al. reported the synthesis of two indigo derivatives **279.1**–**2**, both end-capped with triptycene units, in an attempt to solubilize the otherwise insoluble indigo pigment (Scheme 279).^522^ In the synthetic route, 2-aminotriptycene (**279.3**) was subjected to the sequential treatments of chloral hydrate, hydrochloric acid, and hydroxylamine. The resulting compound **279.4** underwent cyclization in MsOH to give the isatin **279.5** in 85% yield. Next, **279.5** was heated with PCl_5_ in toluene at 100 °C, followed by reduction by thiophenol in one pot to furnish the indigo derivative **279.1** in 57% yield. Further annulation was accomplished with the use of 2-thiopheneacetyl chloride, yielding the Cibalackrot analogue **279.2** in 30% yield. The solubilities of compounds **279.1** and **279.2** in DCM were determined to be 10 and 38 mmol/L. The former compound was thus 66 times more soluble than the parent indigo (0.15 mmol/L) without the end-caps.

**Scheme 280:**
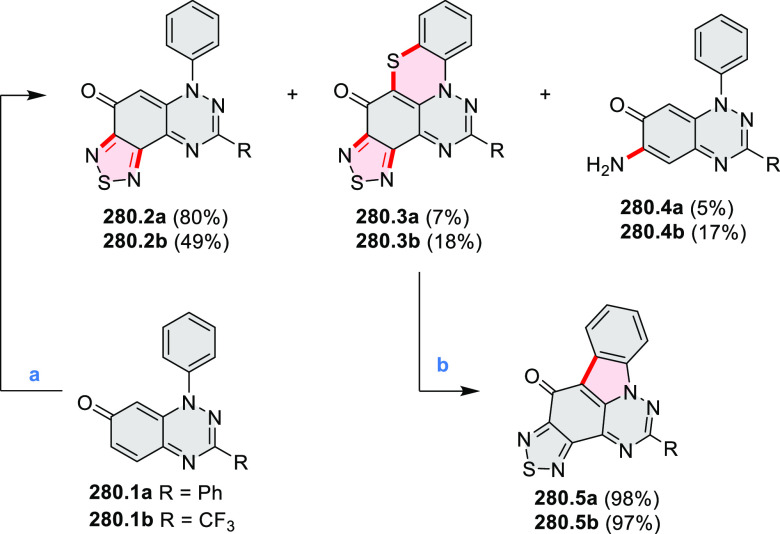
Preparation of Thiadiazolo-Fused Triazafluoranthenones Reagents and conditions: (a)^523^ S_4_N_4_, DMF, 153 °C, 7 h; (b) *m*-terphenyl, 270 °C, 3 h.

Koutentis and co-workers showed that, when heated with excess S_4_N_4_ in DMF, benzotriazinones **280.1a**,**b** produced the corresponding thiadiazole derivatives **280.2a**,**b**, accompanied by an unusual *peri*-fused product **280.3a**,**b** and a small quantity of the amine **280.4a**,**b** (Scheme 280).^523^ Thermolysis of the 1,4-thiazino-fused **280.3a**,**b** efficiently produced the ring-contracted triazafluoranthenones **280.5a**,**b**.

#### 6.2.7 Pyracylene-Based Systems

In analogy to acenaphthylene-fused quinoxalines, their pyracylene-fused analogues are prepared via diketone–diamine condensation (Chart 26). In 2017, the three polymorphs of compound **C26.1** were found by Zhang, Li et al. to exhibit different stacking modes and interlayer distances, which correlated with the trends in solid state fluorescence quantum yields.^524^ Shown in the chart are also the pyracylene-based quinoxalines **C26.2a**–**c** and **C26.3a**–**e** explored by Bunz, Dreuw, Freudenburg et al. as potential OLED emitters.^488^

In 2017, Murata, Murata et al. explored the reactivity of 5,11-bis(5-methyl-3-thienyl)tetracene (**281.1**) in oxidative pentannulation reactions (Scheme 281.1).^498^ Using one or two equivalents of *p*-chloranil in the presence of triflic acid, the singly cyclized (**281.2**) and doubly cyclized (**281.3**) products were isolated in 53% and 57% yield, respectively. Both ring closures took place regioselectively with the tetracene backbone. According to the authors, this cyclization was the first example of fusing a thiophene unit into a fully unsaturated five-membered ring using oxidative coupling. Such transformations had previously been accomplished only by palladium-catalyzed protocols (such as Schemes 264 and 265, Section 6.2.1). Experimental and theoretical data implied that the electronic structure of **281.3** had a strong antiaromatic contribution associated with the newly formed five-membered rings.

**Scheme 281:**
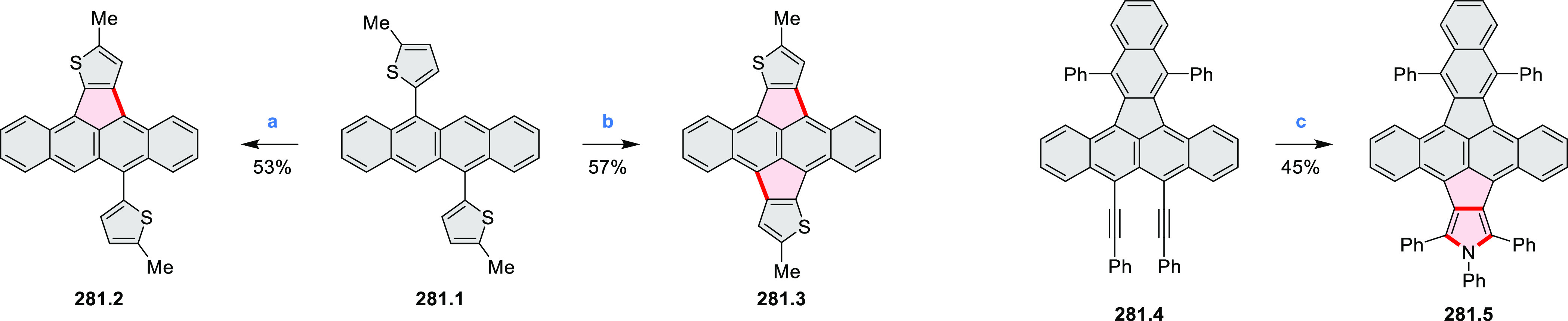
π-Extended Heterocycle-Fused Pyracylenes Reagents and conditions: (a)^498^*p*-chloranil (1.0 equiv), DCM/TfOH (100/1), 0 °C, 10 min; (b) *p*-chloranil (2.0 equiv), DCM/TfOH (100:1), 0 °C, 10 min; (c)^525^ PhNH_2_, PdCl_2_, Et_3_N, DMSO, 90 °C, 8 h.

In 2019, Hamura et al. reported a new method for construction of the benzo[5,6]indeno[1,2,3-*fg*]tetracene skeleton involving intramolecular benzoallene–alkyne cycloaddition.^525^ The resulting diacetylenes, such as the derivative **281.4**, were used as precursors to pyracylene derivatives (Scheme 281). In particular, the palladium-catalyzed bicyclization of **281.4** with aniline under previously known conditions afforded the pyrrole-fused pyracylene derivative **281.5** in 45% yield.

#### 6.2.8 Extended Thiaacenaphthylenes

**Chart 27:**
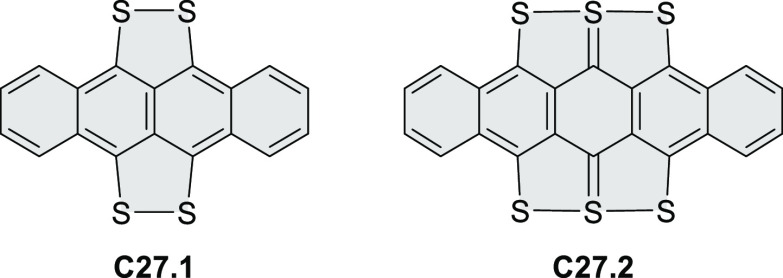
Edge-Fused Organosulfur Acenes

The known sulfur-rich acenes, tetrathiotetracene (**C27.1**) and hexathiopentacene (**C27.2**), are potential candidate cathodes in lithium-ion batteries (Chart 27, cf. CR2017, Section 6.2.8). In 2019, Long, Hu et al. developed a zone-melting chemical-vapor-transport (ZM-CVT) apparatus, which enabled the large-scale synthesis of single crystals of **C27.1**–**2** in a solvent-free, continuous manner.^526^ The lithium-ion battery performance and cycling stability of the single crystals of both acenes were compared, with hexathiopentacene **C27.2** being a better material in this respect. A two-step, three-electron lithiation mechanism was proposed for the hexathiopentacene battery instead of the typically assumed two-electron mechanism.

**Scheme 282:**
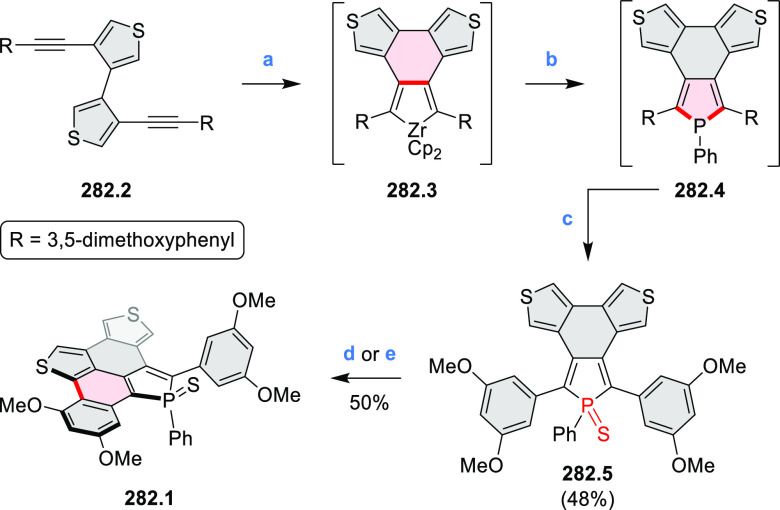
Thiophene- and Phosphole-Fused Polyarene Reagents and conditions: (a)^527^ Cp_2_ZrCl_2_, THF, −78 °C, then *n*-BuLi, overnight; (b) (1) CuI, 0 °C, 20 min, (2) PhPCl_2_, −78 °C, 15 h; (c) S_8_, DCM, rt, 5 h; (d) I_2_, propylene oxide, toluene, *hν* (mercury vapor lamp), 20 h; (e) FeCl_3_, DCM.

In 2016, Hissler, Nyulászi et al. reported the optical and electrochemical properties of the thiophene- and phosphole-fused polycyclic arene **282.1**.^527^ As shown in Scheme 282, the synthesis involved the Fagan–Nugent reaction of the diyne **282.2** with Cp_2_ZrCl_2_ and *n*-BuLi to give the zirconacycle **282.3**, followed by stepwise treatment with CuI and PhPCl_2_, to give the crude phosphole **282.4**. Subsequent oxidation with elemental sulfur gave the corresponding phosphole sulfide **282.5** in 48% overall yield. Photochemical cyclization effected the single six-membered ring closure of **282.5** to give **282.1** in 50% yield, while the doubly cyclized product was not detected. An analogue of **282.5** containing juxtaposed benzene and thiophene rings was similarly obtained.

**Scheme 283:**
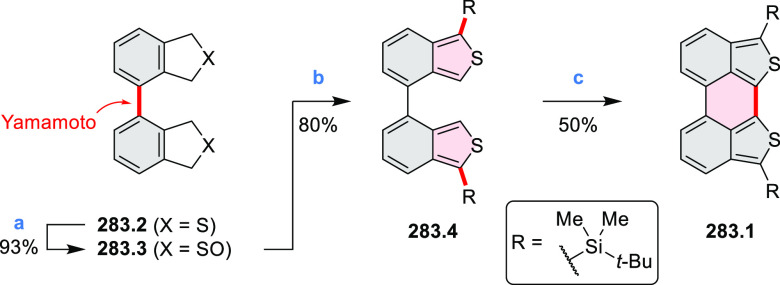
Synthesis of a Phenanthrodithiophene Derivative Reagents and conditions: (a)^528^ NaIO_4_, THF/H_2_O, rt, 12 h; (b) (1) TMEDA, THF, −80 °C, 30 min, (2) *n*-BuLi, THF, 30 min, (3) TBDMSCl, 20 min, then rt, 12 h; (c) FeCl_3_, toluene/MeNO_2_, rt,.

A synthesis of the phenanthro[9,8-*bc*:10,1-*b*′*c*′]dithiophene derivative **283.1** substituted with two bulky TBDMS groups was developed by Ooyama, Oshita et al. (Scheme 283).^528^ In the synthesis, the penultimate intermediate **283.4** originated from the sequential treatments of the bis(sulfoxide) **283.3** with TMEDA, *n*-BuLi and *tert*-butyldimethylsilyl chloride (TBDMSCl). The cyclodehydrogenation of **283.4** using FeCl_3_/MeNO_2_ gave compound **283.1** in 50% yield. This fused polyarene exhibited an intense vibronically structured absorption (λ_max_ = 598 nm) and fluorescence (λ_em_ = 613 nm), which were red-shifted relative to those the noncyclized precursor **283.4**. Notably, the Stokes shift of **283.1** (409 cm^–1^) was significantly smaller than that of **283.4** (3513 cm^–1^).

**Scheme 284:**
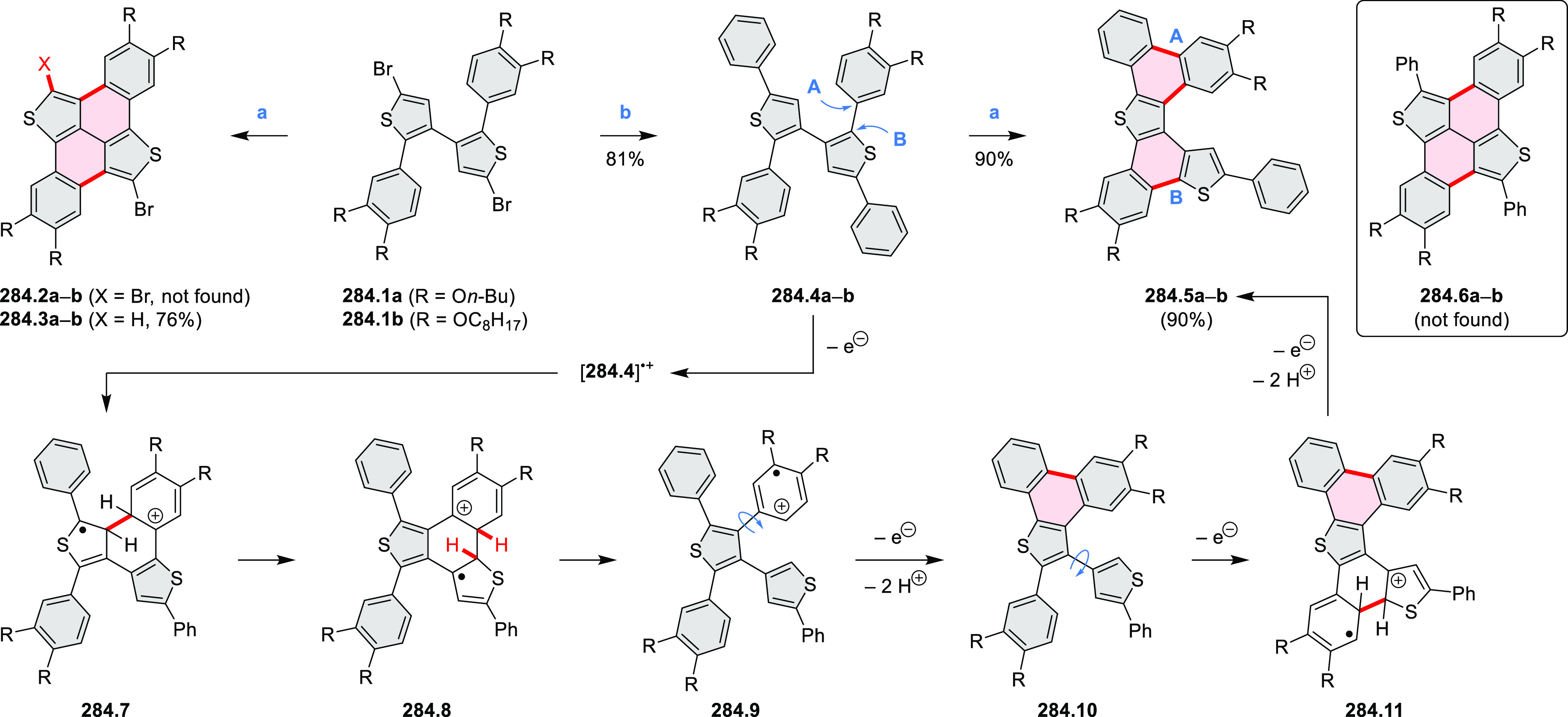
Synthesis of a Sulfur-Hybridized Pyracylene Derivative and an Unexpected Aryl Shift during Oxidative Cyclization^,^ Reagents and conditions: (a)^529^ FeCl_3_, DCM/MeNO_2_, 5 min; (b) phenylboronic acid, K_2_CO_3_, Pd(PPh_3_)_4_, THF/H_2_O, N_2_, dark, 70 °C, overnight. Two corresponding carbon atoms (**A** and **B**) of compounds **284.4** and **284.5** are labeled in blue for a better understanding.

In 2019, Huang, Zhao et al. reported the reactivity of a series of 2,2′-diaryl-substituted 3,3′-bithiophene derivatives toward FeCl_3_-mediated oxidative coupling (Scheme 284).^529^ It was found that the double oxidative cyclization of **284.1a**–**b** was indeed successful, but it furnished monobromides **284.3a**–**b**, rather than the expected dibromides **284.2a**–**b**. When the phenyl-substituted compounds **284.4a**–**b** were subjected to identical oxidative conditions, they produced unexpected rearrangement–cyclization products **284.5a**–**b**. Their identities were deduced from NMR data and further confirmed by independent synthesis. The emergence of the unexpected product was rationalized under the radical cation mechanism for oxidative coupling. Specifically, the radical cation **284.4**^•+^ was proposed to undergo C–C bond formation to give the distonic radical cation **284.7**, which then isomerized to **284.8**. Subsequent C–C bond cleavage generated **284.9**, completing an overall 1,4-shift of the 3,4-dialkoxyphenyl group. Subsequent ring closures would then lead to the observed polyaromatic skeleton of **284.5**.

#### 6.2.9 Fused Oxaacenaphthylenes

**Scheme 285:**
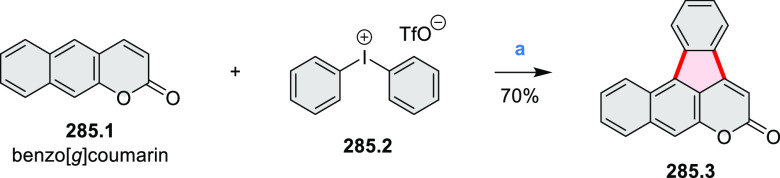
Palladium-Catalyzed Diarylation with Diaryliodonium Salts Reagents and conditions: (a)^530^ Pd(OAc)_2_ (10 mol %), DMF, 100 °C, 24 h.

In 2015, Wang, Han et al. reported a palladium-catalyzed C–H annulation reaction of mono- and disubstituted coumarin derivatives using diaryliodonium cations as coupling partners.^530^ In particular, when benzo[*g*]coumarin (**285.1**) was heated with diphenyliodonium triflate **285.2** in the presence of Pd(OAc)_2_ in DMF at 100 °C, the diarylation product **285.3** was formed in 70% yield (Scheme 285). Preliminary mechanistic studies suggested the involvement of double Pd(II)–Pd(IV) catalytic cycles.

### 6.3 Cyclopenta[*cd*]indene Systems

#### 6.3.1 Fused Pyrrolo[3,2,1-*hi*]indoles

**Scheme 286:**
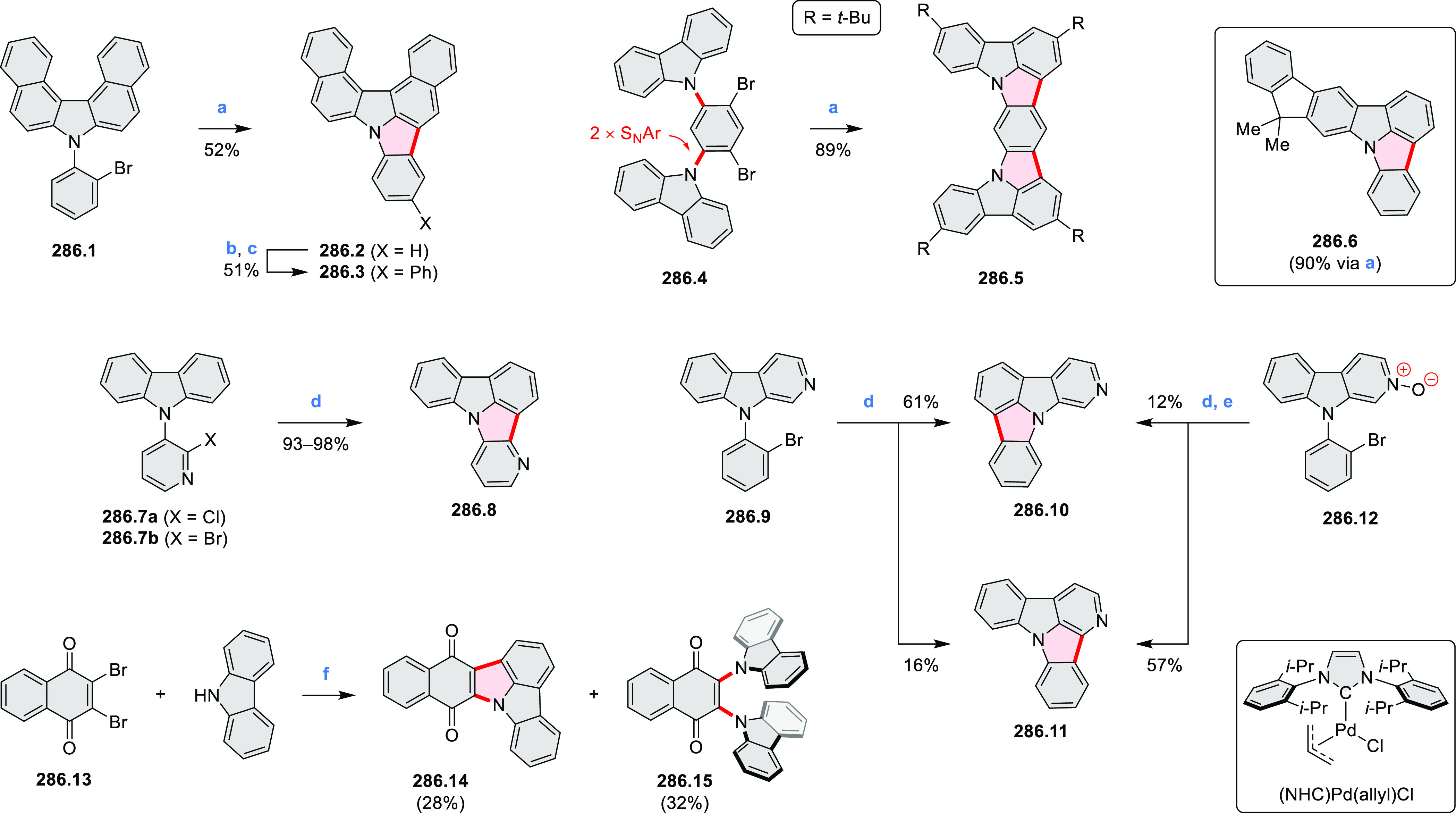
Pentannulated Carbazoles via Palladium-Catalyzed Direct Arylation Reagents and conditions: (a)^531−533^ Pd(OAc)_2_, PPh_3_, [BnNEt_3_]Cl, K_2_CO_3_, DMA, reflux, 6 h; (b) NBS, DMF, rt, 12 h; (c) diphenylamine, Pd(dba)_2_, *t*-Bu_3_P, *t*-BuONa, xylene, reflux, 12 h; (d)^534^ (NHC)Pd(allyl)Cl (5 mol %), K_2_CO_3_ (2 equiv), DMA, argon, 130 °C, 4–6 h; (e) Fe powder, AcOH, 60 °C, argon, 2 h; (f)^535^ Pd(OAc)_2_, PPh_3_, *t*-BuONa, toluene, 90 °C, 24 h.

The pyrrolo[3,2,1-*hi*]indole structure is most prevalent in its [*a*,*d*] dibenzannulated form, i.e., as the indolo[3,2,1-*jk*]carbazole structure. Two commonly used strategies to construct this extended skeleton include intramolecular direct arylation or oxidative coupling, both under palladium catalysis. Recent examples employing the former strategy are shown in Scheme 286. As reported by Lee et al., compounds **286.3**,^531^**286.5**,^532^ and **286.6**,^533^ which are sky blue to violet emitters, were synthesized from the carbazole precursors possessing *ortho*-bromoaryl groups attached to the nitrogen atoms.

Kautny showed that the use of (NHC)Pd(allyl)Cl as the catalyst granted access to a series of aza-substituted indolocarbazoles.^534^ For example, **286.8** was obtained in good yield from the 9-(2-halo-3-pyridyl)carbazoles **286.7a**–**b** using the optimized conditions. For the unsymmetrical carbazole **286.9**, the C–Br bond preferentially reacted with the benzo nucleus to give **286.10** (61% yield), with the regioisomer **286.11** (16% yield) being the minor product. Interestingly, when the *N*-oxide **286.12** was subjected to the same cyclization followed by reduction, the yields of **286.10** and **286.11** became 12% and 57%, respectively, indicating an electronic control during cyclization.

Langer et al. reported the palladium-catalyzed reaction of 2,3-dibromo-1,4-naphthoquinone (**286.13**) with carbazole to give the annulation product **286.14** in 28% yield, and the 2-fold C–N coupling product **286.15** in 32% yield.^535^

**Scheme 287:**
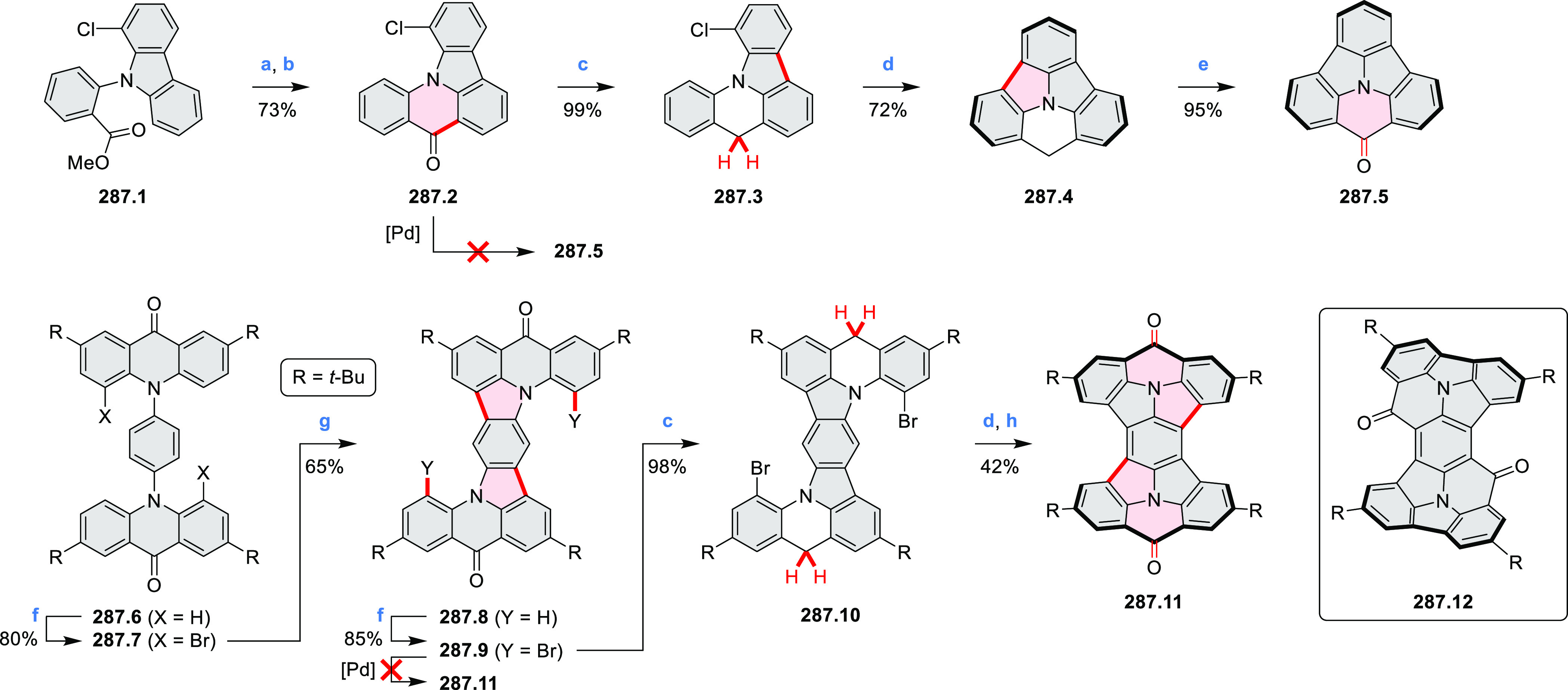
Stepwise Cyclization Approach toward Highly Fused Acridones Reagents and conditions: (a)^536^ NaOH, EtOH/H_2_O, reflux, 3 h; (b) polyphosphoric acid, 180 °C, 4 h; (c) BH_3_·THF, THF, reflux, 4 h; (d) Pd(OAc)_2_, PCy_3_·HBF_4_, K_2_CO_3_, DMA, 170 °C, 48 h; (e) Na_2_Cr_2_O_7_, AcOH, reflux, 1 h; (f)^537^ Br_2_, DCM, rt, 48 h; (g) Pd(OAc)_2_, PCy_3_·HBF_4_, K_2_CO_3_, DMA, 130 °C, 24 h; (h) DDQ, DCM/dioxane/H_2_O, 0 °C to rt, 16 h.

A route to the doubly pentannulated acridone derivative **287.5**, developed by Deng and Zhang in 2019, started with the singly fused acridone **287.2**, which was available from an intramolecular Friedel–Crafts acylation step (Scheme 287).^536^ Attempted direct conversion of **287.2** into the target molecule **287.5** was unsuccessful because of the rigidity of the fused molecular skeleton. Thus, the ketone functionality in **287.2** was quantitatively reduced by borane to furnish the more flexible molecule **287.3**, which proved susceptible to the subsequent palladium-catalyzed cyclization, yielding **287.4**. Finally, dichromate oxidation of **287.4** yielded the target ketone **287.5** in 95% yield. The bowl-shaped geometry of **287.5** was confirmed by X-ray crystallography.

In 2020, Zhou and Zhang reported an analogous approach toward the highly fused bisacridone **287.11** (Scheme 287).^537^ The synthesis began with selective 2-fold bromination of the bisacridone **287.6** to give **287.7**, followed by double cyclization. The resulting **287.8** was subjected to another dibromination step, furnishing the dibromide **287.9**. Again, the rigid molecule **287.9** could not directly cyclize to give **287.11**. Instead, the reduction–cyclization–oxidation sequence allowed isolation of the target diketone **287.11** in 41% yield over three steps. In 2021, Song and Zhang reported the isomeric nanoboat **287.12** with a lower-symmetry fusion mode.^538^ The curved, boat-like structures of both **287.11** and **287.12** were established by X-ray crystallography. The absorption profile of the latter nanoboat is bathochromically shifted from that of the former one. Based on DFT results, the HOMO levels of both molecules do not differ significantly, but the less symmetrical molecule **287.12** possesses a lower LUMO level (−2.94 eV) than does **287.11** (−2.50 eV). This difference in HOMO–LUMO gap is also reflected in the red-shifted fluorescence wavelength of **287.12** (524 nm) relative to that of **287.11** (475 nm).

**Scheme 288:**
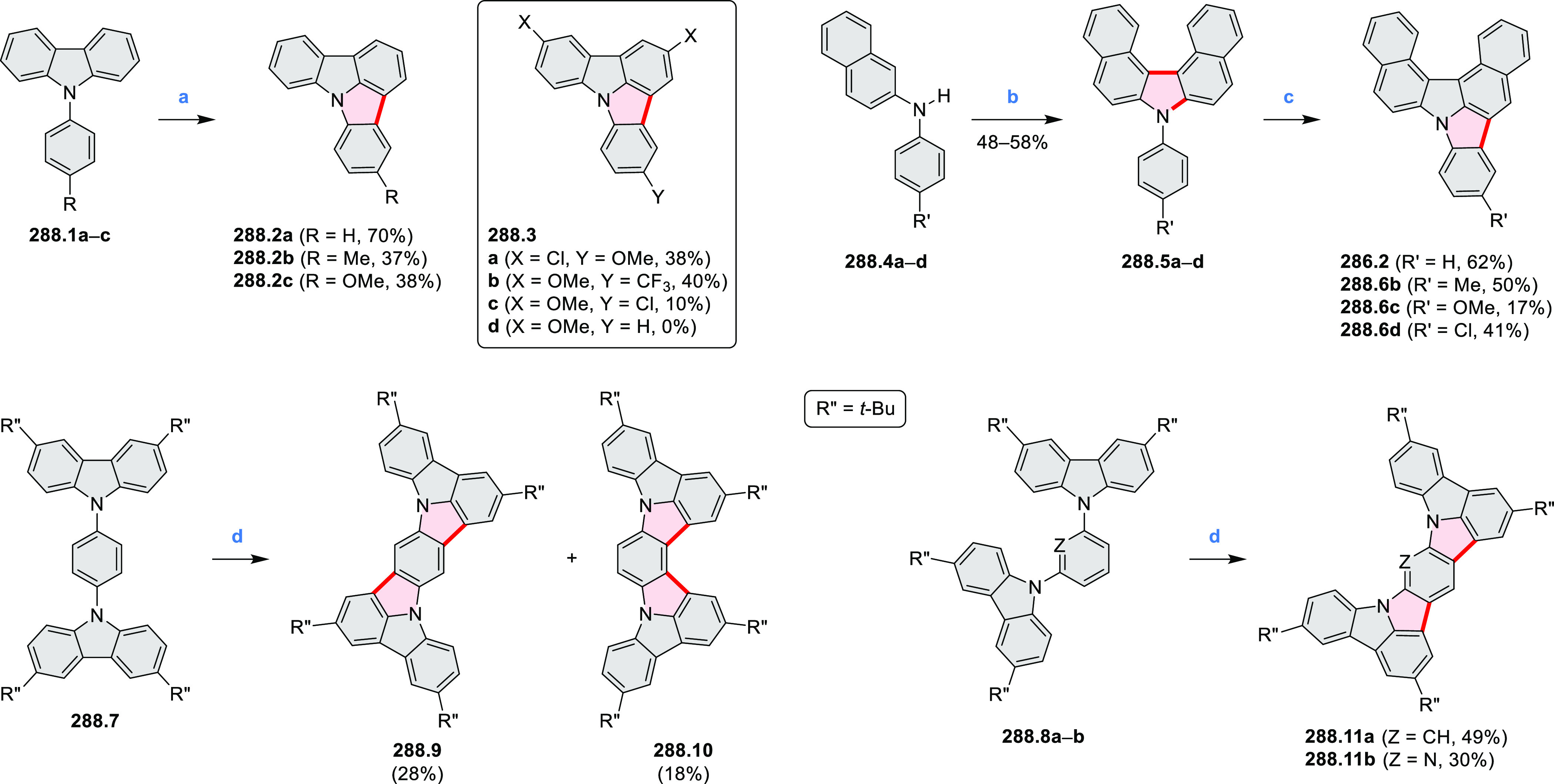
Pentannulated Carbazoles via Palladium-Catalyzed Oxidative Cyclization Reagents and conditions: (a)^539^ palladium(II) pivalate (10 mol %), Ag_2_O (1.2 equiv), CuO (1.2 equiv), pivalic acid, O_2_ (1 atm), 130 °C, 24 h; (b)^540^ Ag_2_O (1 equiv), toluene/cumene/AcOH, air, 60 °C, 2 h; (c) Pd(OAc)_2_ (10 mol %), Ag_2_O (1.2 equiv), CuO (1.2 equiv), pivalic acid, air, 130 °C, 3 days; (d)^541^ palladium(II) trifluoroacetate (30 mol %), AgOAc (6.0 equiv), pivalic acid, 160 °C, 48 h.

In 2015, Patureau et al. reported optimized conditions for the oxidative cyclization of 9-arylcarbazoles such as **288.1a**–**c** to give the corresponding indolocarbazoles **288.2a**–**c** in 38–70% yield (Scheme 288).^539^ In addition to palladium(II) pivalate, the presence of Ag_2_O, CuO, and an oxygen atmosphere was found essential to achieve the conversion. The trisubstituted products **288.3a**–**c** were also obtained from their respective precursors. However, the dimethoxy-substituted indolocarbazole **288.3d** could not be obtained under those conditions. Subsequently, the same group developed a synthesis of *N-*aryl-substituted π-extended carbazole derivatives, such as **288.5a**–**d**, directly from the corresponding aryl(2-naphthyl)amines (Scheme 288).^540^ The authors showed that a modified set of reaction conditions, with Pd(OAc)_2_ in place of palladium(II) pivalate, successfully induced pentannulation of **288.5a**–**d** to give **286.2** and **288.6b**–**d** in 17–62% isolated yield.

For oxidative cyclization of di-9-carbazolyl-substituted derivatives **288.7**–**8**, Miura et al. employed palladium(II) trifluoroacetate as the catalyst and silver(I) acetate as the oxidant (Scheme 288).^541^ The *para*-dicarbazolyl substrate **288.7** provided the isomeric doubly cyclized products **288.9** and **288.10** in 28% and 18% yield, respectively. The *meta*-dicarbazolyl-substituted benzene **288.8a** and pyridine **288.8b** afforded the corresponding double fusion products **288.11a**–**b** in 49% and 30% yield, respectively. Interestingly, the corresponding *ortho*-dicarbazolylbenzene produced a seven-membered ring under these conditions (Scheme 301, Section 6.4.4).

**Scheme 289:**
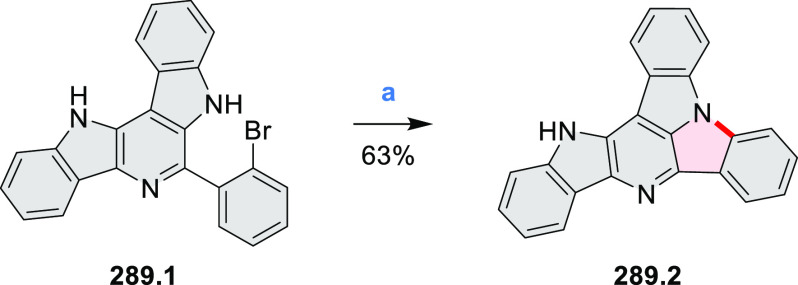
Formation of [5,5,6] Fusion via Ullmann-Type Reaction Reagents and conditions: (a)^519^ Cs_2_CO_3_, CuI, DMF, microwave, 120 °C, 10 min.

The pyridodiindole **289.1** bearing a 2-bromophenyl substituent was obtained in 85% yield during substrate screening for three-component condensation developed by Xu, Li, et al. (Scheme 289; for synthesis and mechanism, see Scheme 276, Section 6.2.6).^519^ Compound **289.1** could be cyclized in an Ullmann-type reaction under microwave heating to give the *peri*-fused compound **289.2** in 63% yield. In contrast to syntheses described earlier in this section, the formation of the 5–5–6 fusion is completed with a C–N bond formation rather than with a C–C coupling.

**Chart 28:**
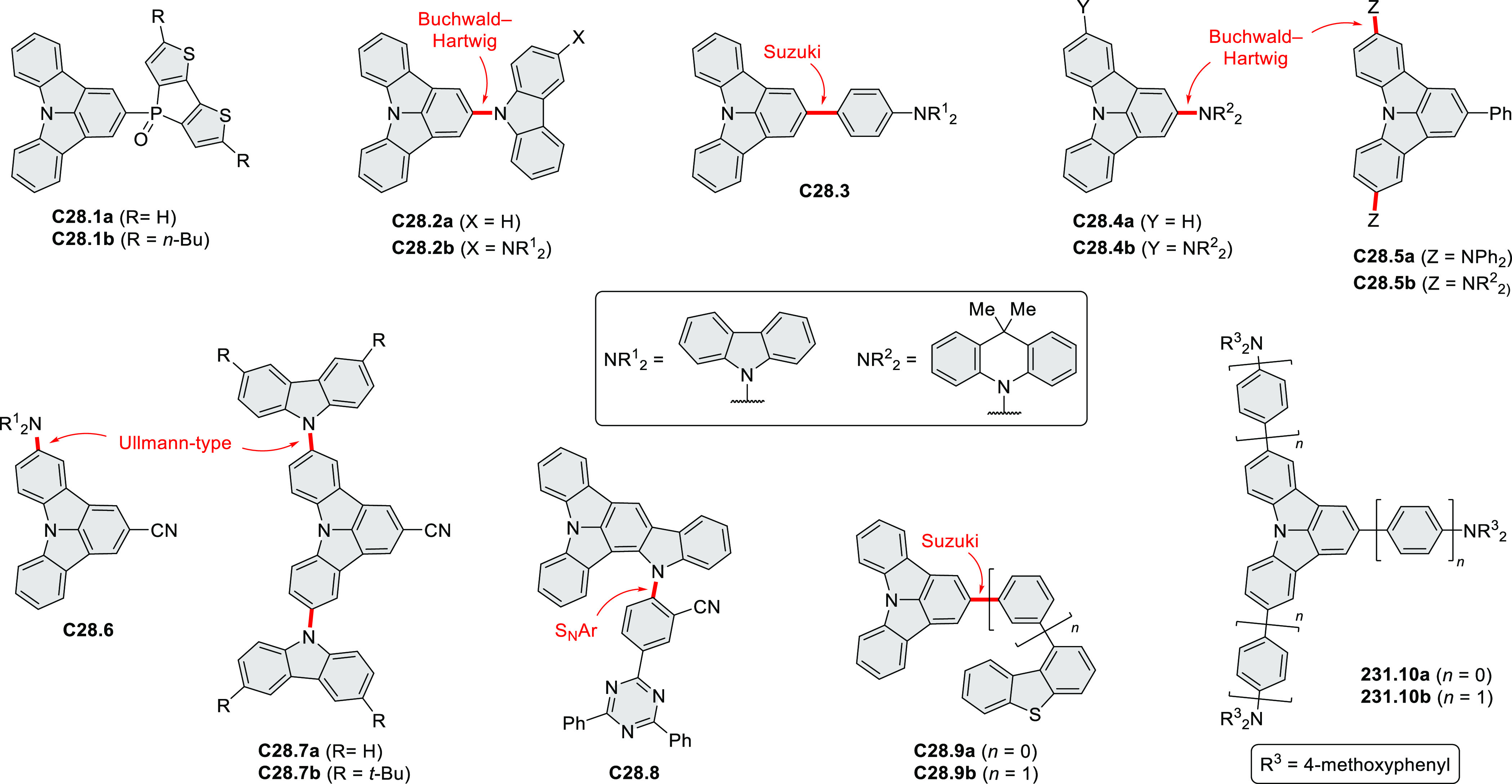
Substituted Indolocarbazoles

A range of dyes designed for optoelectronic applications have been prepared from the multistep functionalization of unsubstituted indolo[3,2,1-*jk*]carbazole (Chart 28). In 2015, Lumpi, Baumgartner et al. reported a series of *P*-substituted dithienophosphole oxides, including the indolocarbazole-containing analogues **C28.1a**–**b**.^542^ Among the reported molecules, only **C28.1a** displayed vibronically well-resolved phosphorescence, and possessed a high triplet energy (2.87 eV), making it suitable as a potential host material for phosphorescent OLED (PhOLED). Similarly, compounds **C28.2a**,**3** reported by Lumpi, Chen et al. in 2016 were also studied in terms of PhOLED applications.^543^ Compounds **C28.2b**, **C28.4a**–**b**,^544^ and **C28.5a**–**b**^545^ bearing various donor units (i.e., carbazole, carbazolylcarbazole, acridine and diphenylamino groups) were developed by Lee et al. as sky blue to deep blue TADF emitters, respectively. The same group reported that the derivative **C28.6** bearing a 2-cyanoindolo[3,2,1-*jk*]carbazole acceptor and a 9-carbazolyl donor group was a green TADF emitter,^546^ while the dicarbazolyl analogues **C28.7a**–**b** were deep blue TADF emitters.^547^ Related designs include **C28.8**, a greenish blue TADF emitter, reported by Lee, Hong et al.,^548^ and the blue phosphorescent indolocarbazole–benzothiophene conjugates **C28.9a**–**b**.^549^ The use of indolocarbazoles **231.10a**–**b** in perovskite solar cells was explored in 2019 by Wang, Jiang et al.^550^

**Chart 29:**
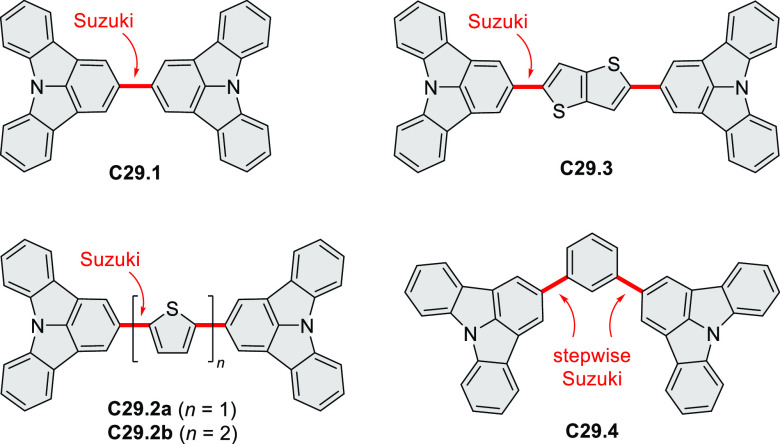
Covalently Linked Bis(indolocarbazole)s

Indolocarbazole dyads connected either via a direct C–C bond or through an aromatic linker are shown in Chart 29. The dimer **C29.1** was prepared by Lumpi, Chen et al. as part of their PhOLED study mentioned above.^543^ In 2017, Bintinger, Horkel et al. reported the use of thiophene-based linkers to construct indolocarbazole dyads such as **C29.2a**–**b** and **C29.3**, for potential application in OLED devices.^551^ Compound **C29.4** incorporating the *m*-phenylene linker, obtained by Lee et al., was used as a host for green PhOLED devices.^552^

#### 6.3.2 Fused Pyrrolo[2,1,5-*cd*]indolizines and related systems

**Scheme 290:**
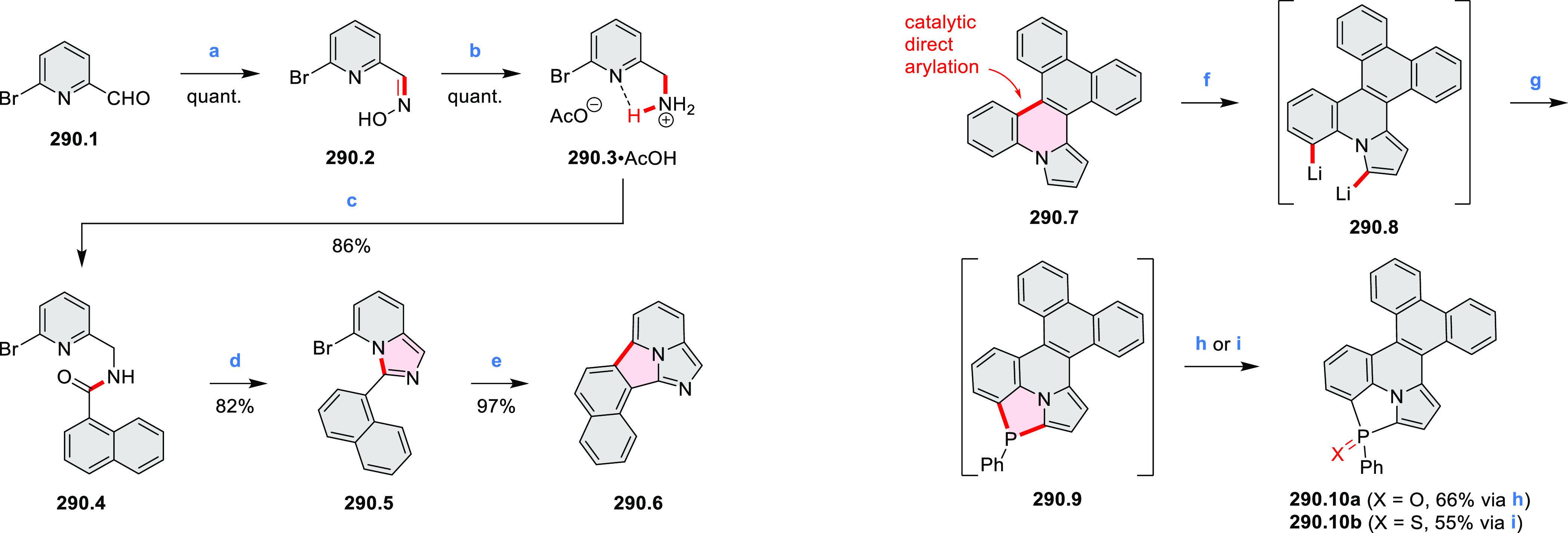
π-Extended Imidazole- and 1,3-Azaphosphole-Fused Indolizine Derivatives Reagents and conditions: (a)^553^ (HONH_3_)_2_SO_4_, NaOAc, EtOH, rt, 16 h; (b) Zn, AcOH, rt, 10 min; (c) 1-naphthylacetyl chloride, Et_3_N, DCM, 0 °C, then rt, 16 h; (d) Tf_2_O, 2-methoxypyridine; DCM, 50 °C, 16 h; (e) Pd_2_(dba)_3_ (cat.) *t*-Bu_3_P·HBF_4_ (cat.), K_2_CO_3_, DMF, 110 °C, 16 h; (f)^554^*n*-BuLi, TMEDA, THF, 0 °C, 1 h; (g) PhPCl_2_, 0 °C, 10 min, then rt, 30 min; (h) aq. H_2_O_2_ (30%), 30 min; (i) S_8_, 30 min.

In 2017, Charette et al. reported a new general synthesis of imidazo[2,1,5-*c*,*d*]indolizine fluorophores, e.g. the naphtho[*a*]-fused derivative **290.6** (Scheme 290).^553^ The synthesis started with treating the commercially available 6-bromopicolinaldehyde (**290.1**) with hydroxylammonium sulfate to provide the oxime **290.2**, followed by reduction by zinc powder to give the ammonium salt **290.3**·AcOH quantitatively over two steps. The final three steps, i.e., acylation, electrophilic cyclization, and palladium-catalyzed cyclization, provided the naphtho[*a*]-fused compound **290.6** and a range of its analogues with or without ring fusions on the [*a*] side. The last three steps had been reported earlier by the same group for the preparation of the benzo[*a*]-fused analogue. This improved method avoided the use of the expensive amine **290.3** as the starting material. Emission properties of these fluorophores could be tuned over the entire visible range by changes of substitution.

In 2018, Mathey, Duan, Chen et al. described a series of 1,3-azaphosphole oxides and phosphides, including the π-extended derivatives **290.10a**–**b**, which were prepared in a one-pot procedure from the pyrrolo[1,2-*a*]quinoline precursor **290.7**.^554^ Specifically, the bay region of the pyrrolo[1,2-*a*]quinoline skeleton was dilithiated with *n*-BuLi in the presence of TMEDA to give **290.8**. Subsequent treatment with PhPCl_2_ generated the 1,3-azaphosphole unit in the intermediate **290.9**. Eventual addition of H_2_O_2_ or elemental sulfur afforded the target oxide **290.10a** (66% yield) and sulfide **290.10b** (55% yield), respectively. Compound **290.10a** was shown to be useful as an emitting dopant in OLEDs and as a bioimaging dye.

**Scheme 291:**
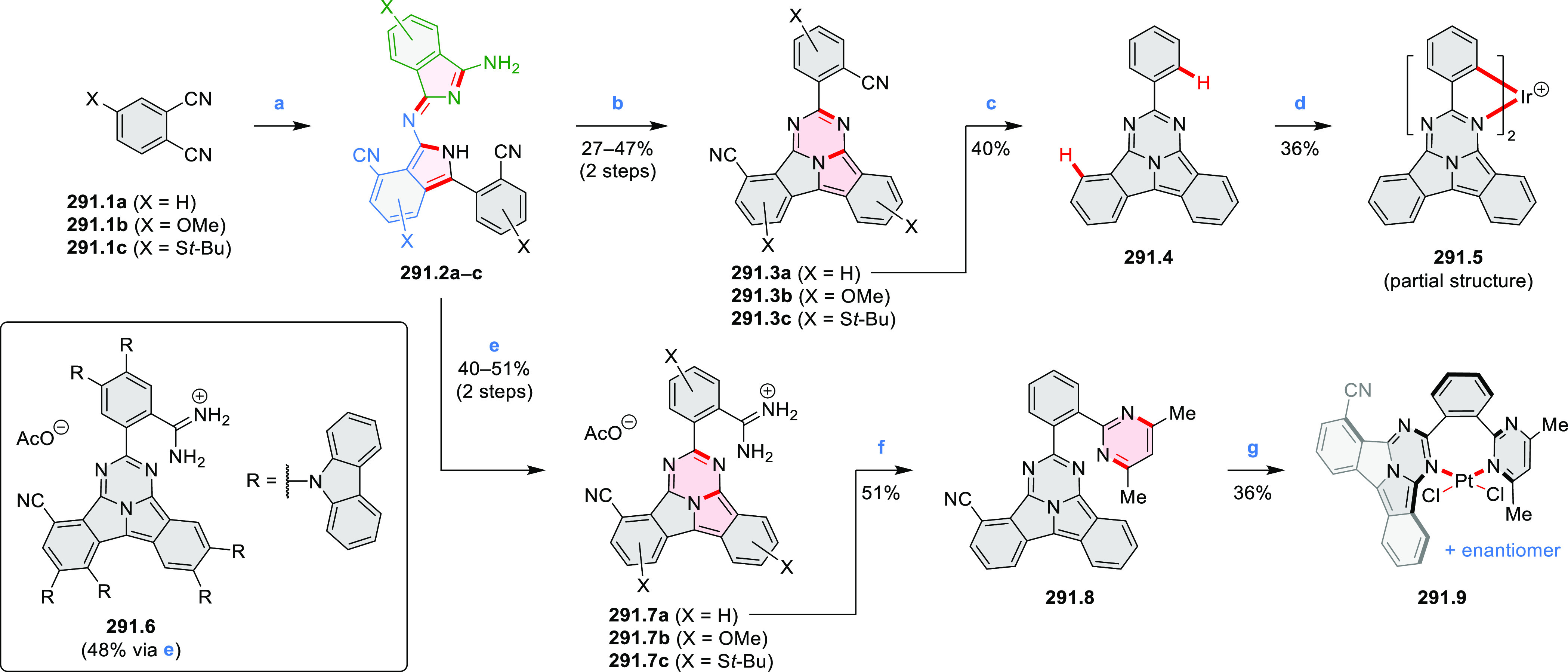
Dibenzannulated Derivatives of Triazino[2,1,6-*cd*]pyrrolizine Reagents and conditions: (a)^555^*t*-BuOK (1 equiv), DMF, 0 °C, 1 h; (b) neat, N_2_, 230 °C, 3 h; (c) [Rh(cod)Cl]_2_ (5 mol %), P(O*i*-Pr)_3_ (0.1 equiv), *i*-Pr_3_SiH (4 equiv), ethylcyclohexane, reflux, 15 h; (d) IrCl_3_, dry ethylene glycol, N_2_, reflux, 24 h; (e) EtOH/AcOH (1:1), reflux, 24 h; (f) acetylacetone, pyridine, reflux, 24 h; (g) K_2_PtCl_4_, 2-ethoxyethanol/H_2_O (3:1), N_2_, 80 °C, 24 h.

Unexpected formation of a dibenzo-fused triazino[2,1,6-*cd*]pyrrolizine ring system was reported by Li et al. (Scheme 291).^555^ When the known compound **291.2a**, obtained by trimerization of phthalonitrile (**291.1a**), was heated in a ceramic crucible at 230 °C, the “phthalorubine” **291.3a** was isolated in 47% yield. Likewise, using 4-methoxyphthlonitrile (**291.1b**) or 4-(*tert*-butylthio)phthalonitrile (**291.1c**) as the starting materials yielded phthalorubines **291.3b**–**c** of unspecified regiochemistry. Besides, the cyclization of **291.2a**–**c** could proceed in a refluxing mixture of acetic acid and ethanol, affording the amidinium salts **291.7a**–**c** in 40–51% yield. When 4,5-di(9-carbazolyl)phthalonitrile was used as the starting material, only the latter milder conditions could mediate the cyclization to give the amidinium salt **291.6**. The study was then extended to metal coordination chemistry. First, the two cyano groups in **291.3a** were reductively removed using triisopropylsilane and a rhenium(I) catalyst. The resultant compound **291.4** acted as a C,N bidentate ligand toward iridium(III), forming the mononuclear complex **291.5**. Second, **291.7a** was annulated with acetylacetone to give the pyrimidinyl-substituted derivative **291.8**, which was then reacted with K_2_PtCl_4_ to give the inherently chiral complex **291.9**.

### 6.4 *peri*-Fused Seven- and Eight-Membered Rings

#### 6.4.1 *peri*-Fused Cycloheptatrienes

**Scheme 292:**
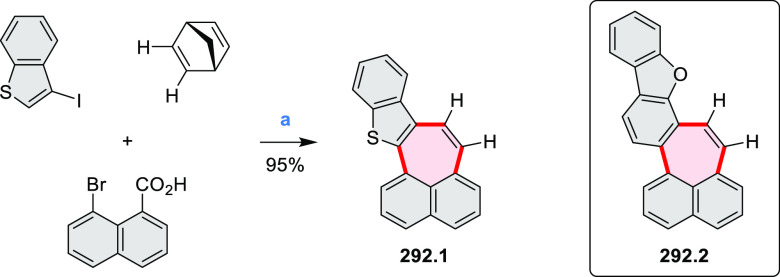
Three-Component Synthesis of Fused Cycloheptatrienes Reagents and conditions: (a)^556^ Pd(OAc)_2_ (5 mol %), PCy_3_ (15 mol %), Cs_2_CO_3_, 1,4-dioxane, 130 °C, 18 h.

In 2017, Kwong et al. reported a palladium-catalyzed three-component reaction of aryl iodide, 8-bromo-1-naphthoic acid and norbornadiene, which provided access to carbo- and heterocyclic frameworks containing seven-membered rings (Scheme 292).^556^ For example, 3-iodobenzo[*b*]thiophene could be converted into compound **292.1** in 95% yield under optimized conditions. Likewise, 4-iododibenzofuran could be transformed into **292.2** in 85% yield. On the basis of DFT calculations, the authors proposed a Pd(0)–Pd(II)–Pd(IV) catalytic cycle for the reaction.

**Scheme 293:**
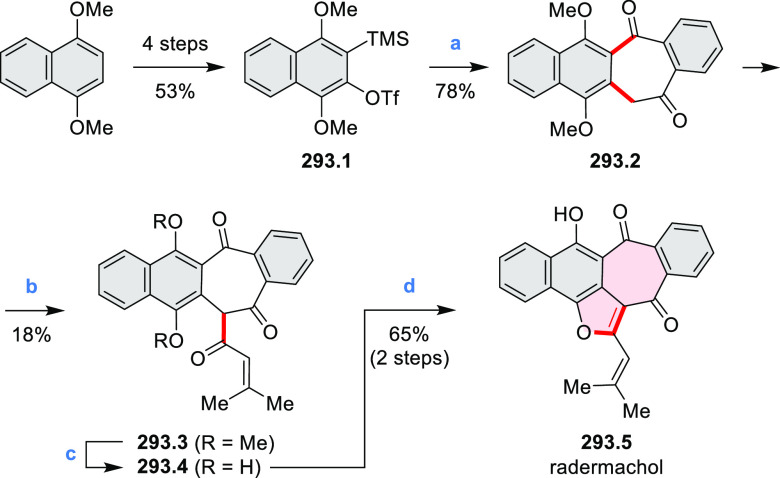
Total Synthesis of Radermachol Reagents and conditions: (a)^557^ 1,3-indanedione, CsF, MeCN, 75–80 °C, 1.5 h; (b) 3-methylcrotonoyl chloride, AlCl_3_, 55 °C, 2 h; (c) Me_3_SiI, CDCl_3_, 25 °C, 27 h; (d) TsOH, benzene, reflux, 5 h.

In 2016, Samineni, Srihari, and Mehta reported the application of a ring-expansion strategy to the total synthesis of the natural product radermachol (**293.5**, Scheme 293, for earlier work on radermachol, see CR2017, Section 6.4.1).^557^ The aryne precursor **293.1** was synthesized in four steps from 1,4-dimethoxynaphthalene. The naphthyne generated from **293.1** successfully inserted into 1,3-indanedione to give **293.2** in 78% yield. Subsequent *C-*acylation with 3-methylcrotonoyl chloride furnished **293.3**. Next, a demethylation–cyclodehydration sequence generated the furo fusion in radermachol, which was obtained in 65% yield over two steps.

#### 6.4.2 Fused Azepines and Diazepines

**Scheme 294:**
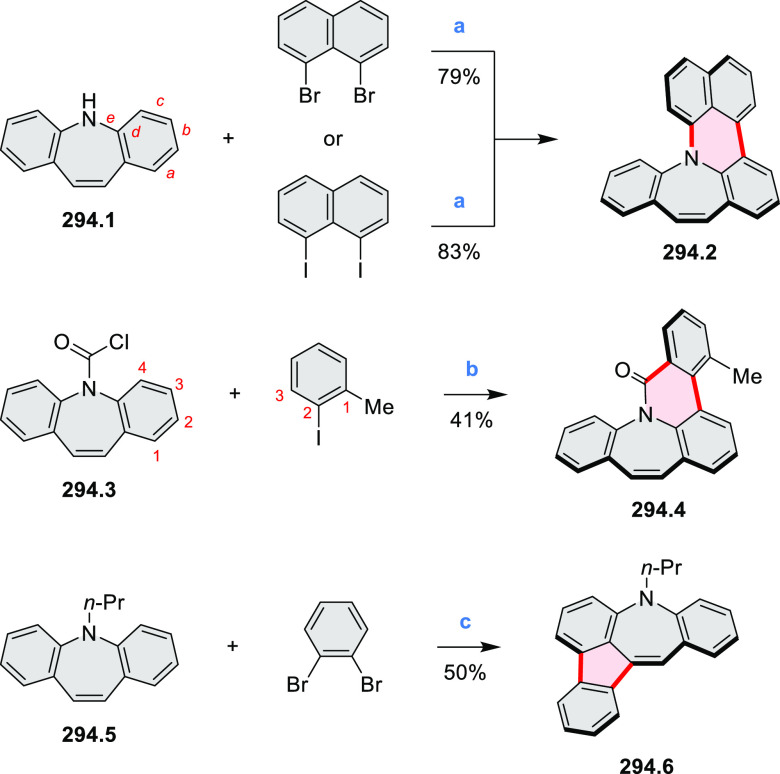
Syntheses of [*de*] and [*kl*]Fused Dibenzoazepines via Palladium-Catalyzed Annulation Reagents and conditions: (a)^76^*t*-BuONa, Pd(OAc)_2_ (3 mol %), Pd_2_(dba)_3_ (3 mol %), PCy_3_ (7 mol %), *t*-Bu_3_P (7 mol %), dry toluene, 90 °C, 10 h; (b)^558^ Pd(OAc)_2_ (10 mol %), PPh_3_ (20 mol %), norbornene (1 equiv), Cs_2_CO_3_ (4 equiv), toluene, 95 °C, 6 h; (c)^559^ PdCl(C_3_H_5_)(dppb) (2 mol %), KOAc (2 equiv), DMA, 150 °C, 72 h.

The one-pot N–H/C–H coupling reaction reported by Li et al. (for details, see Scheme 45, Section 3.1.2) was also applicable to the annulation of 5*H*-dibenzo[*b*,*f*]azepine (**294.1**).^76^ As shown in Scheme 294, the use of either 1,8-dibromo- or 1,8-diiodonaphthalene as the coupling partner under the optimized reaction conditions could lead to the [*de*]annulated dibenzoazepine **294.2** in 79–83% yield. The nonplanar geometry of **294.2** was established by X-ray crystallography. In 2016, Jiao et al. reported the palladium-catalyzed annulation of the dibenzoazepine-based carbamic chloride **294.3** with 2-iodotoluene to give **294.4** in 41% yield.^558^ In addition to the precatalyst, the phosphine ligand and the base, the addition of norbornene was necessary. DFT calculations suggested a Pd(0)–Pd(II)–Pd(IV) catalytic cycle enabled by the participation of norbornene. The two new C–C bonds were formed by C3 acylation of 2-iodotoluene, followed by C4 arylation of dibenzoazepine. As shown by Doucet, Soulé et al., dibenzo[*b*,*f*]azepines can be pentannulated using the palladium-catalyzed direct C10-arylation with aryl bromides (Scheme 294).^559^ In a particular example using the optimized conditions, the diazepine **294.5** and 1,2-dibromobenzene were heated in DMA in the presence of PdCl(C_3_H_5_)(dppb) and KOAc. The indeno-fused product **294.6** was isolated in 50% yield. The reaction proceeded via two successive C–H bond activations.

**Scheme 295:**
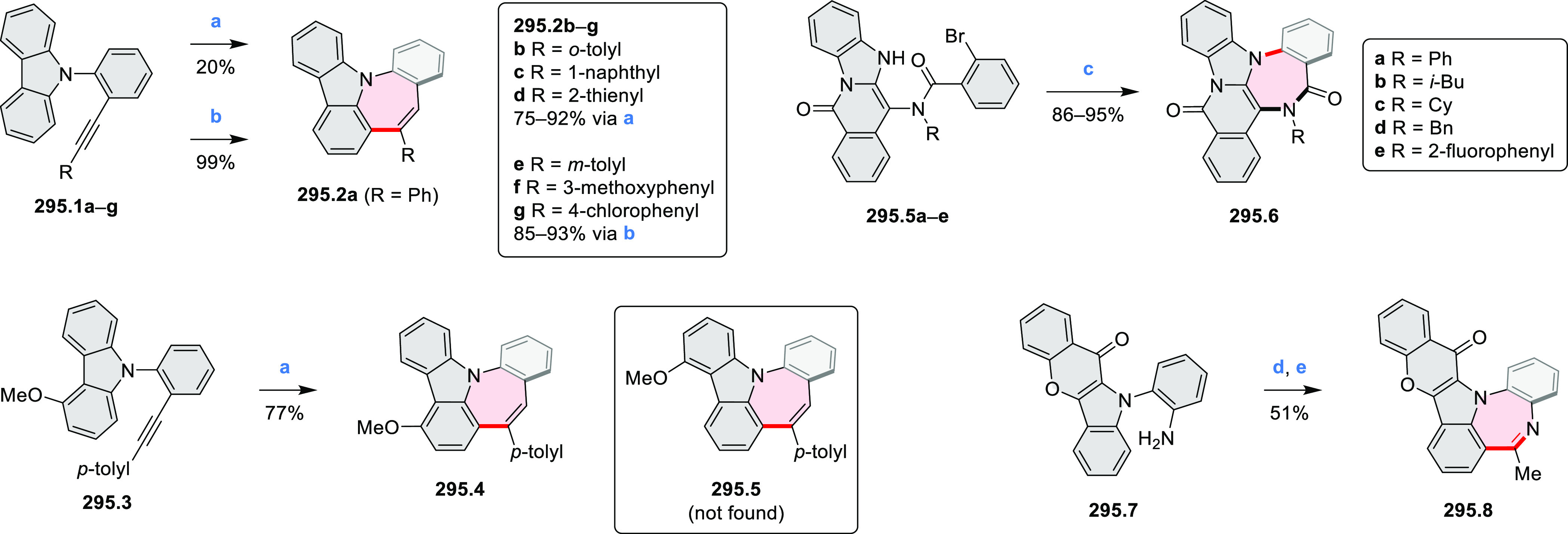
[*de*]Fused Dibenzoazepines and Analogues via Direct Cyclization Reagents and conditions: (a)^560^ AuCl(PPh_3_) (10 mol %), AgSbF_6_ (10 mol %), 1,2-DCE, 80 °C, 24 h; (b) AuCl[P(C_6_F_5_)_3_] (10 mol %), AgOTf (10 mol %), 1,2-DCE, rt, 3 h; (c)^518^ Cs_2_CO_3_, CuI, DMF, microwave, 150 °C, 10 min; (d)^517^ AcCl (1.2 equiv), Et_3_N (2.0 equiv), DCM, rt, 4 h; (e) polyphosphoric acid, POCl_3_ (10 equiv) 120 °C, 3 h.

In 2016, Shibata et al. reported the gold(I)-catalyzed cycloisomerization of 9-(2-alkynylphenyl)carbazoles, such as **295.1**–**g**, to give the a series of carbazole-fused azepine derivatives including **295.2a**–**g** (Scheme 295).^560^ In the case **295.1a**, the use of AuCl(PPh_3_) and AgSbF_6_ at a high temperature led to the cyclized product **295.2a** in 20% yield. A much higher yield (99%) could be achieved with the use of AuCl[P(C_6_F_5_)_3_] and AgOTf at rt. The authors examined the regioselectivity of this reaction with several unsymmetrical carbazoles. For example, the cycloisomerization of **295.3** occurred preferentially at the electron-rich aromatic ring to give the azepine **295.4**, while the regioisomer **295.5** could not be detected.

A different type of direct cyclization employed precursors **295.5a**–**e** obtained in the already mentioned four-component condensation reported by Xu, Chen et al. (Scheme 295, cf. Scheme 275, section 6.2.6).^518^ An Ullmann-type cyclization of the pendant 2-bromophenyl substituent led to the corresponding fused benzimidazoisoquinolines **295.6a**–**e** in 86–95% yield. Similarly, compound **295.7** (Scheme 274, Section 6.2.5) was converted into a fused diazepine derivative **295.8** in a two-step acylation sequence, as shown in Scheme 295.^517^

**Scheme 296:**
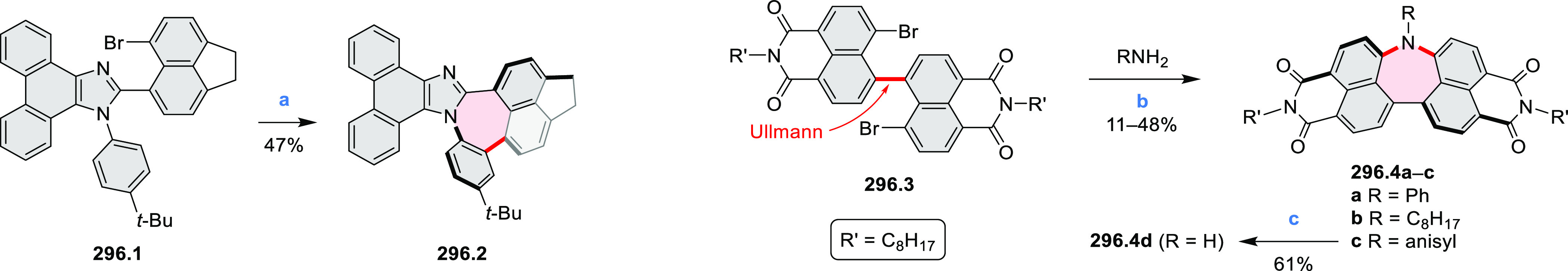
*peri*-Naphtho-Fused Azepine Derivatives via Heptannulation Reagents and conditions: (a)^561^*hν* (254 nm, 2 × 8 W), DCM, rt; (b)^562^ Pd_2_(dba)_3_·CHCl_3_, BrettPhos, *t*-BuONa, toluene, 80 °C, 9 h; (c) anisole, TFA, toluene, 70 °C, 4 h.

Phenanthroimidazole **296.1**, synthesized by Skonieczny and Gryko in a three-component condensation discussed in Section 4.7.2 (Scheme 165) was found to be susceptible to photoinduced intramolecular direct arylation of the 4-*tert*-butylphenyl ring, generating the azepine-fused product **296.2** in 47% yield (Scheme 296).^561^ This protocol worked successfully without using any sensitizer or base.

In 2019, Shinokubo, Akutagawa, Kim et al. reported a series of dinaphthoazepine diimides, such as **296.4a**–**d**, which can be considered ring-expanded analogues of perylene diimides (Scheme 296).^562^ The precursor **296.3** was prepared from the intermolecular Ullmann-type coupling of the corresponding bromoiodonaphthalene monoimide. Buchwald–Hartwig amination of **296.3** with various primary amines yielded the fused azepines **296.4a**–**c** in 11–48% yield. In particular, the 4-methoxybenzyl (anisyl) group in **296.4c** could be cleaved by excess anisole and trifluoroacetic acid to give **296.4d** in 61% yield. These fused azepines possessed flexible, nonplanar conformations, and exhibited ambipolar redox activity.

**Scheme 297:**
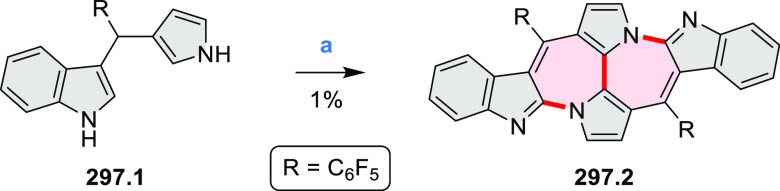
Fused Diazaheptalene via Copper-Assisted Oxidative Cyclodimerization Reagents and conditions: (a)^563^ (1) DDQ, THF, 1 h, (2) Cu(OAc)_2_, overnight.

In 2016, Anand et al. reported the oxidative cyclodimerization of the indolylpyrrolylmethane **297.1** upon successive additions of DDQ and Cu(OAc)_2_ to give the diazaheptalene derivative **297.2** in 1% yield (Scheme 297). While low-yielding, this transformation is notable for creating a sequence of six linearly fused rings of different sizes in just one synthetic step. Under identical conditions, the isomer of **297.1** containing a 2-pyrrolyl group yielded structurally different products. In the solid state, the molecules of **297.2** were planar and stacked at a distance of ca. 3.3 Å.

#### 6.4.3 Oxa- and Thia- 7-Membered Rings

**Scheme 298:**
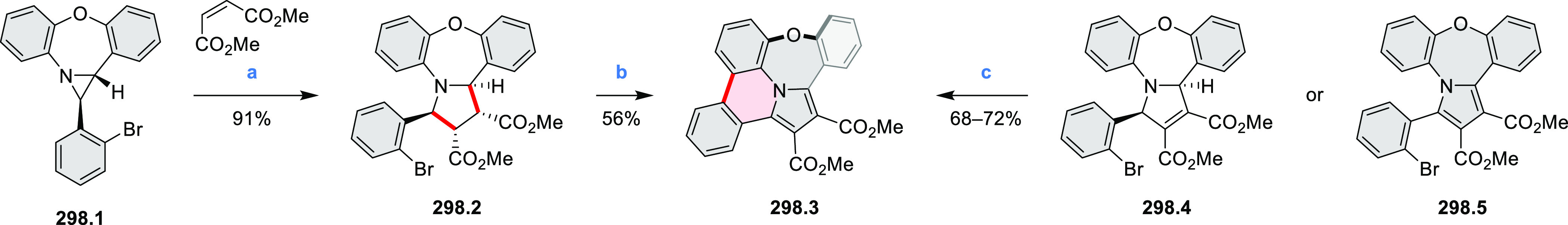
Synthesis of Indole-Fused Dibenzoxazepine via Radical Cyclization Reagents and conditions: (a)^564^ toluene, reflux, 8 h; (b) AIBN (2.2 equiv), Bu_3_SnH (0.5 equiv), toluene, 80 °C, 7 h; (c) AIBN (1.1 equiv), Bu_3_SnH (0.5 equiv), toluene, 80 °C, 4 h.

In 2017, Khlebnikov et al. reported the construction of the indole-fused dibenzoxazepine derivative **298.3** (Scheme 298).^564^ The aziridine **298.1** reported by the same group was subjected to cycloaddition with dimethyl maleate in refluxing toluene to give the pyrrolidine **298.2** stereoselectively in 91% yield. Exposure of **298.2** to AIBN and tributyltin hydride at a high temperature brought about the free radical cyclization that led to the indole-fused dibenzoxazepine **298.3** in 56% yield. When the pyrrolidine **298.2** was replaced by the previously reported pyrroline **298.4** or pyrrole **298.5**, the same radical cyclization could proceed to give the same product **298.3** in 68–72% yield using a lesser amount of AIBN and shorter reaction time.

**Scheme 299:**
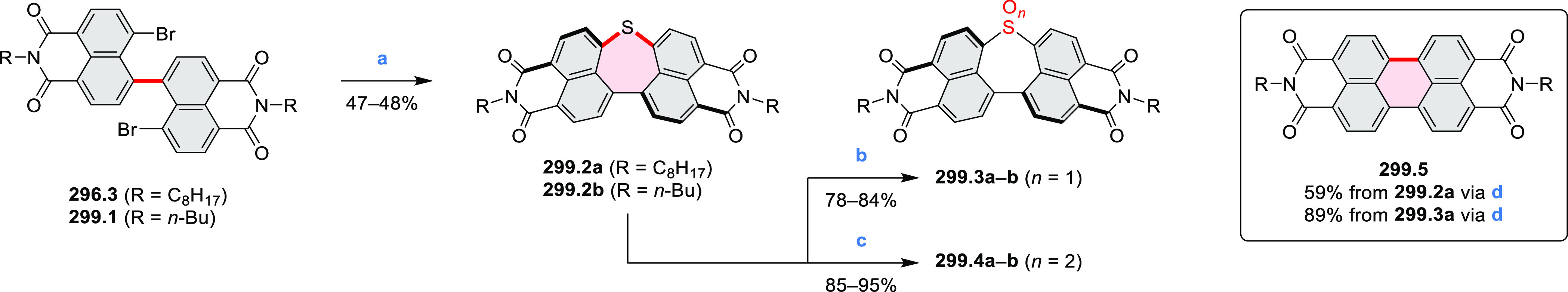
Synthesis and Reactivity of Dinaphthothiepine Diimides Reagents and conditions: (a)^565^ Na_2_S, DMF, 60 °C; 3 h; (b) *m*-CPBA, DCM, −80 °C, 30 min, then rt, 1 h; (c) aq. H_2_O_2_, Na_2_WO_4_·2H_2_O, MeN(*n*-C_8_H_17_)_3_·HSO_4_, phenylphosphonic acid, toluene/H_2_O, 50 °C; (d) (1) Co(Cp*)_2_, DCM, rt, 20 min, (2) chloranil.

In 2020, Shinokubo, Fukui, Yamada et al. reported the synthesis and properties of the dinaphthothiepine diimides **299.2a**–**b** (Scheme 299),^565^ analogous to the azepines **296.4a**–**d** mentioned above. The construction of the fused thiepine ring was accomplished by the S_N_Ar reaction of the dibromides **296.3** and **299.1** by sodium sulfide using DMF as the solvent. **299.2a**–**b** could be converted into the corresponding sulfoxides **299.3a**–**b** or sulfones **299.4a**–**b** depending on the oxidation conditions. Both **299.2a** and the sulfoxide **299.3a** could undergo sulfur extrusion upon electron injection by decamethylcobaltocene followed by quenching with chloranil to give the perylene diimide **299.5**. Besides, the same extrusion reactions could take place upon electrochemical reduction and photoirradiation, as substantiated by voltammetric and spectroscopic measurements. In contrast, photoirradiation of the sulfone **299.4a** did not lead to the PDI product **299.5**.

#### 6.4.4 Aza-, Oxa- and Thia-8-Membered Rings

**Scheme 300:**
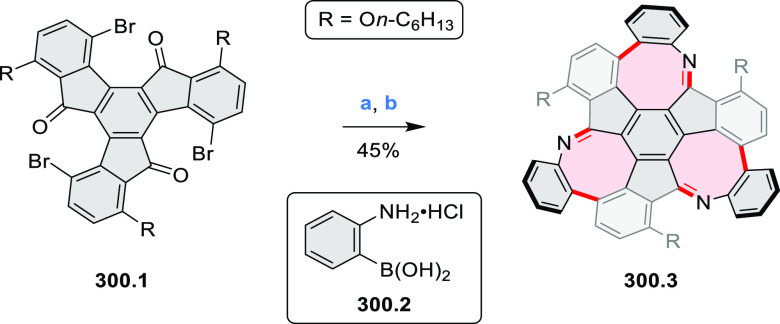
Synthesis of a Triaza Monkey Saddle Reagents and conditions: (a)^566^**300.2**, Pd_2_(dba)_3_, *t*-Bu_3_P·HBF_4_, K_2_CO_3_, THF/H_2_O (1:1), 80 °C, 48 h; (b) AcOH/CHCl_3_ (1:10), 80 °C, 18 h.

In 2020, Mastalerz et al. reported the synthesis and of the chiral monkey saddle **300.3** bearing three fused azocine rings (Scheme 300).^566^ The key intermediate in the synthesis is the tribromotruxenone **300.1**, which was first triply cross-coupled with 2-aminophenylboronic acid hydrochloride (**300.2**), followed by imine bond formation to give the triply cyclized product **300.3** in 45% yield. In the racemic single crystal, the chiral monkey saddle conformation was confirmed for both the (*R*_a_,*R*_a_,*R*_a_)- and (*S*_a_,*S*_a_,*S*_a_)-enantiomers of **300.3**. The authors managed to separate the enantiomers by chiral HPLC, and the inversion barrier and half-life of enantiopure **300.3** were found to be 113 ± 6 kJ mol^−1^ and 7.7 ± 0.1 h, respectively.

**Scheme 301:**
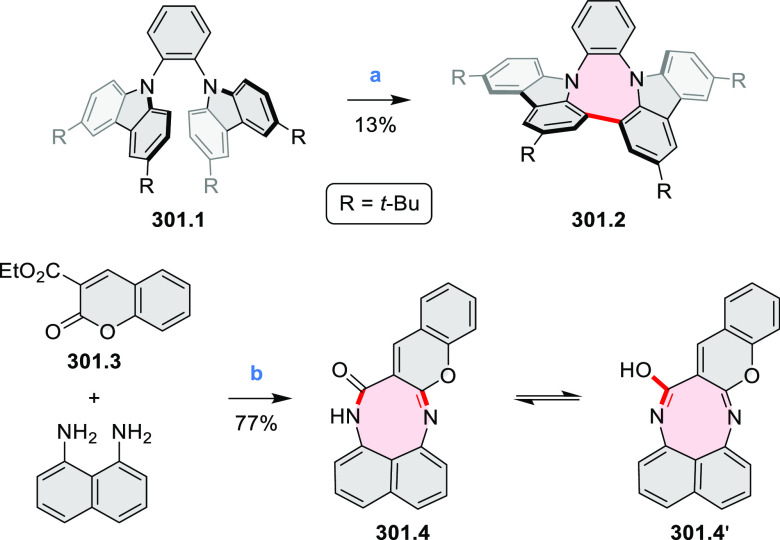
Syntheses of Fused 1,4- and 1,5-Diazocines Reagents and conditions: (a)^541^ palladium(II) trifluoroacetate (30 mol %), AgOAc (6.0 equiv), pivalic acid, 160 °C, 48 h; (b)^567^ neat, 145–150 °C.

In addition to the *meta*- and *para*-dicarbazolylbenzenes discussed above (see Scheme 288section 6.3.1), Miura et al. also tested the reactivity of the *ortho* isomer **301.1** toward the same oxidative cyclization (Scheme 301).^541^ In the latter reaction, the fused 1,4-diazocine **301.2** was isolated in 13% yield, and its twisted molecular structure was unequivocally established by X-ray crystallography. The feasibility of eight-membered ring closure was attributed to the proximity of the two carbazole units in **301.1**. A different approach to *peri*-fused diazocines was described by Deeb et al., who showed that heating an equimolar mixture of 1,8-naphthalenediamine and the ester-containing coumarin **301.3** could furnish the annulation product **301.4** which exists in equilibrium with the 1,5-diazocine tautomer **301.4**′.^567^

**Scheme 302:**
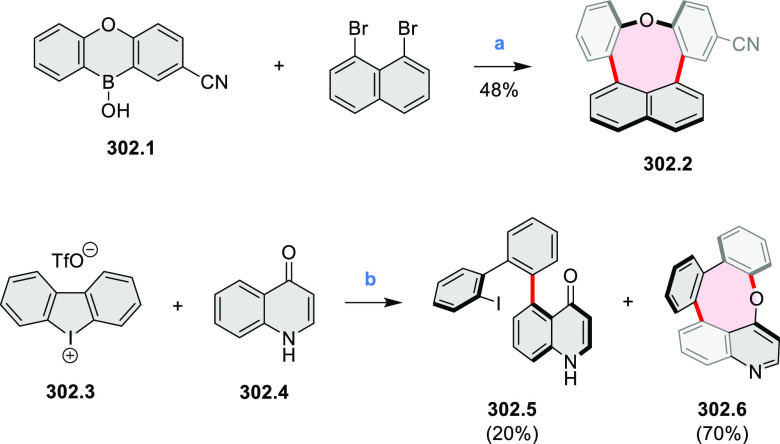
Syntheses of *peri*-Fused Oxocines via Annulative Coupling Reagents and conditions: (a)^568^ Pd_2_(dba)_3_, *t*-Bu_3_P·HBF_4_, Cs_2_CO_3_, H_2_O/*t*-amyl alcohol, 100 °C, 48 h; (b)^569^ Pd(OAc)_2_ (5 mol %), AcOH, 100 °C, 12 h.

In 2017, Igarashi, Tobisu, and Chatani reported a general, gram-scale synthesis of a series of cyclic diarylborinic acids, including the cyano-substituted analogue **302.1** (Scheme 302).^568^ Under Pd-catalyzed cross-coupling conditions, compound **302.1** underwent an unusual ring-opening annulation with 1,8-dibromonaphthalene, yielding the fused oxocine derivative **302.2** in 48% yield. Kumar et al. showed the formation of a fused oxocine ring in an iodonium-based annulation reaction (Scheme 302).^569^ When diphenyleneiodonium triflate (**302.3**) and quinolin-4(1*H*)-one (**302.4**) were heated in the presence of Pd(OAc)_2_, the C–H arylation product **302.5** and the annulation product **302.6** were isolated in yields of 20% and 70%, respectively. The identity of the oxocine **302.6** was supported by NMR and mass spectral data, and this product was believed to originate from the C–O bond formation of the minor product **302.5**.

**Scheme 303:**
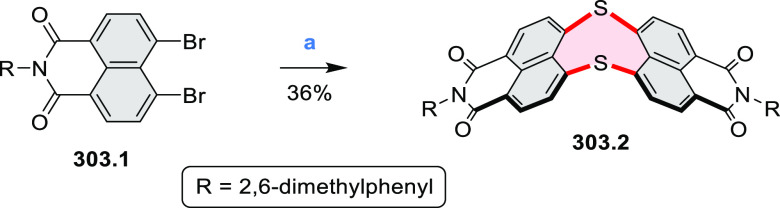
Synthesis of Dinaphthodithiocine Diimide Reagents and conditions: (a)^570^ Na_2_S (1.2 equiv), DMF, rt, 3 h.

In 2021, Shinokubo, Fukui et al. reported the preparation of the dinaphthodithiocine diimide **303.2** (Scheme 303),^570^ a larger expanded congener of thiepine diimides **299.2a**–**b**. The synthesis used the known dibromonaphthalene imide **303.1**, which was treated with Na_2_S in DMF to give the annulation product **303.2** in moderate yield. As revealed by X-ray crystallography, molecules of **303.2** assumed a nonplanar V-shaped geometry with a dihedral angle of 67° between the two naphthalene units. This conformation was shown by VT-NMR experiments and DFT calculations to be sufficiently rigid, with an inversion barrier greater than 30 kcal mol^−1^.

## 7 Macrocyclic Systems

### 7.1 [*b*]-Fused (β–β-Fused) Porphyrinoids

**Scheme 304:**
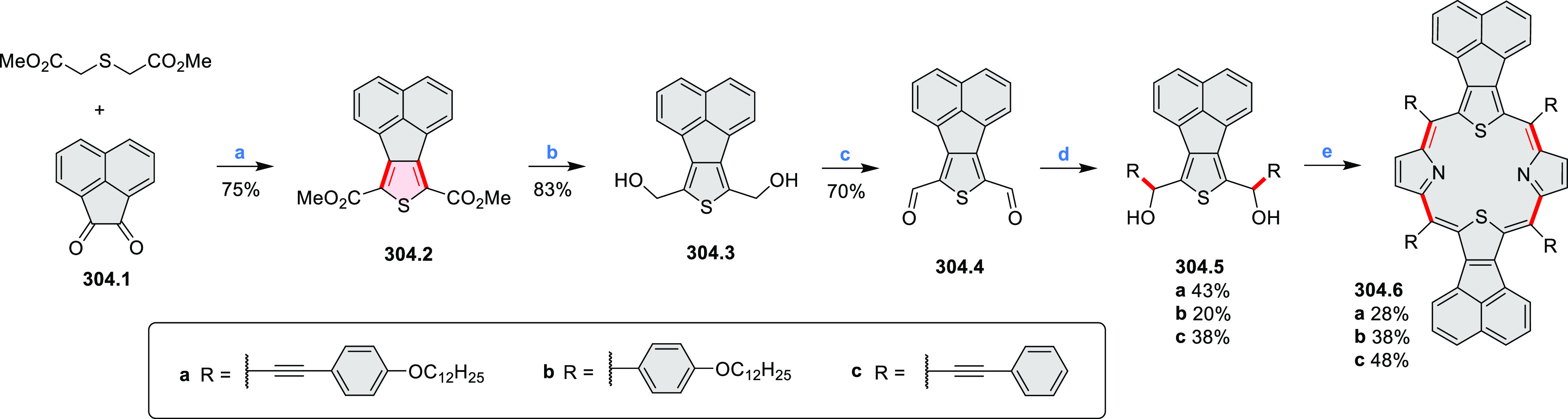
Acenaphthylene-Fused Dithiaporphyrins Reagents and conditions: (a)^571^ (1) NaOMe, THF, (2) MeOH, H_2_SO_4_; (b) LiAlH_4_, diethyl ether; (c) MnO_2_, DCM; (d) *n*-BuLi, TMEDA, **304.5a** from 1-ethynyl-4-dodecyloxybenzene; **304.5b** from 1-bromo-4-dodecyloxybenzene; **304.5c** from ethynylbenzene; (e) (1) BF_3_·Et_2_O, pyrrole, (2) DDQ.

Fusion along the β–β bonds of pyrrole subunits is one of the most frequently used methods of peripheral extension used in porphyrin chemistry. Below we summarize the most recent examples of such systems containing *peri*-fused moieties (for earlier developments, see CR2017, Section 7.1). Variously substituted acenaphthylene-fused dithiaporphyrins were reported by Hogan and Sutherland (Scheme 304).^571^ Thiophene building blocks were prepared by using the Hinsberg condensation between dimethyl 2,2′-thiodiacetate and acenaphthoquinone **304.1** to give the diester **304.2**, which was transformed into **304.5a**–**c** in three steps. Condensation of these dicarbinols with pyrrole and the subsequent oxidation by DDQ afforded acenaphthylene-fused dithiaporphyrins, **304.6a**–**c**. Compounds **304.6a** and **304.6b** displayed broad visible light spectrum absorptions extending into the near-IR range with the onsets of absorption at 1000 and 1100 nm, respectively. The curvature and low oxidation potentials of these dithiaporphyrins were reflected in their interactions with [60]fullerene.

**Scheme 305:**
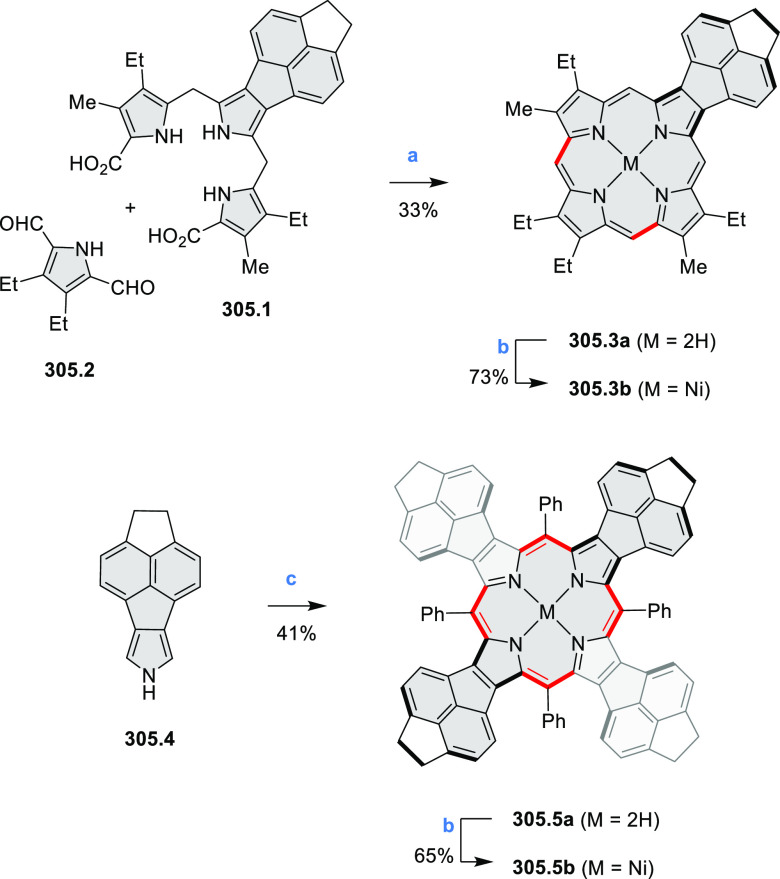
Dihydropyracyloporphyrins Reagents and conditions: (a)^572^ (1) TFA, DCM, 2 h, (2) DDQ, 1 h; (b) Ni(OAc)_2_, 16 h, reflux; (c) benzaldehyde, BF_3_·Et_2_O, CHCl_3_, N_2_, 20 min, (2) DDQ, 20 min.

In 2020, Lash and co-workers reported the incorporation of dihydropyracylene-fused subunits into extended porphyrin chromophores.^572^ The synthesis of **305.3a** was performed using the typical MacDonald condensation with tripyrrane **305.1** and dialdehyde pyrrole **305.2** (Scheme 305). Subsequent metalation afforded the nickel(II) complex **305.3b**. In addition, tetraphenyl-tetrakis(dihydropyracylo)porphyrin **305.5a** was also prepared by reacting dihydropyracylopyrrole **305.4** with benzaldehyde in the presence of BF_3_·Et_2_O, followed by oxidation with DDQ, which gave the desired product in 41% yield. The UV–vis absorption spectrum of **305.5a** did not show any significant shift in the Soret band peak compared to the tetraacenaphthoporphyrin derivative (**309.4a**, R = H, *meso*-aryl = phenyl), however, the doubly protonated **305.5a** produced a red-shift of 26 nm in the Soret band, apparently caused by the additional five membered rings.

**Scheme 306:**
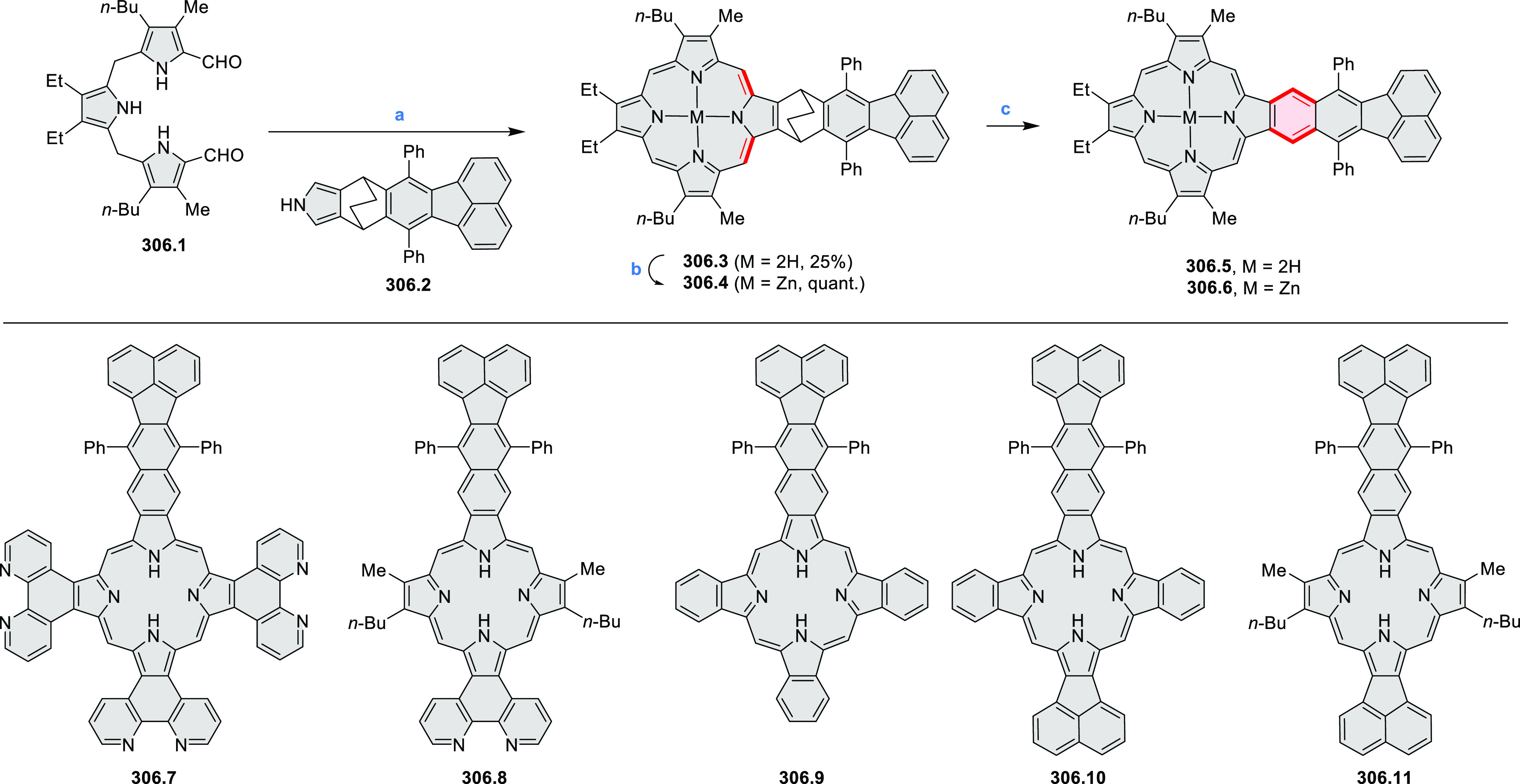
[*b*]-Condensed Porphyrins Containing “Remote” *peri-*Fusion Points Reagents and conditions: (a)^573^ (1) **306.2**, TFA, CHCl_3_, (2) Et_3_N, DDQ; (b) Zn(OAc)_2_·2H_2_O, CHCl_3_/MeOH; (c) Δ, 350 °C, reduced pressure, 0.5 to 3 h, quantitative yields.

In 2016, Okujima, Mack, and Kobayashi reported a family of porphyrins containing one fused benzofluoranthene moiety accompanied by a variety of other fused subunits (Scheme 306).^573^ The synthesis was done via 3 + 1 condensation approach fusing a tripyrrane, e.g. **306.1**, and a bicyclo[2.2.2]octadiene (BCOD) pyrrole, such as **306.2**, which afforded the corresponding BCOD-fused porphyrin, e.g. **306.3**. The π-extended porphyrins **306.5**–**11** were then obtained from the corresponding BCOD precursors via retro-Diels–Alder reactions in near-quantitative yields. The introduction of additional fused-ring moieties at β positions of pyrrole rings and variation of fusion patterns along the x or y axes of the macrocycle significantly affected the electronic structure of these porphyrins. Among the derivatives, the phenanthroline and acenaphthylene-fused porphyrins **306.7** and **306.10** had the narrowest HOMO–LUMO gaps and their lowest absorption bands (Q bands) were shifted into the red/NIR region of the spectrum (close to 700 nm).

**Scheme 307:**
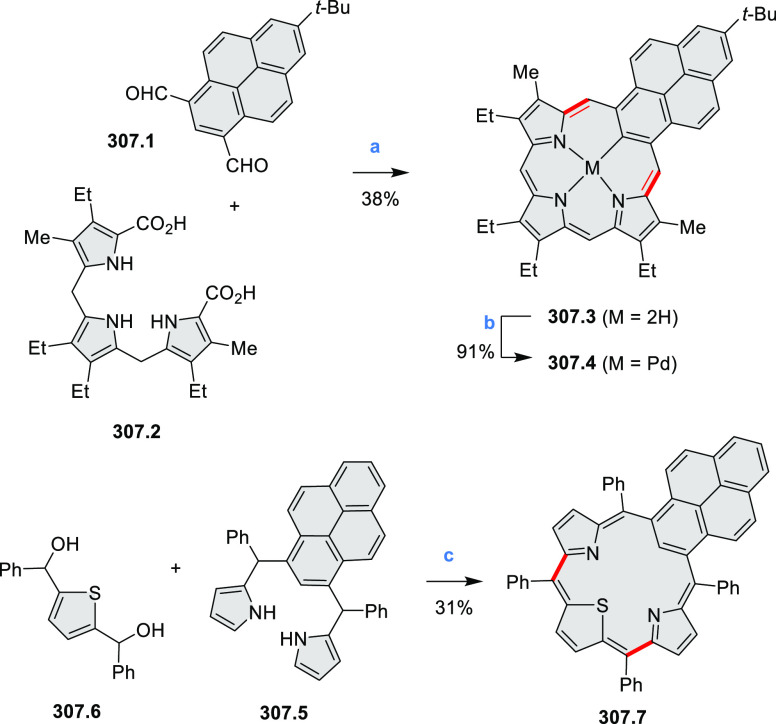
Synthesis of [*b*]-Fused Porphyrins Containing Pyrene Subunits Reagents and conditions: (a)^574^ (1) TFA, DCM, N_2_, 5 h, (2) DDQ, 3 h; (b) Pd(OAc)_2_, CHCl_3_/CH_3_CN, reflux, 2 h; (c) (1) BF_3_·Et_2_O, DCM, N_2_, 3 h, (2) DDQ, 5 h.

The “3 + 1” condensation strategy was further used by Lash and co-workers in the first syntheses of porphyrin analogues incorporating pyrene units (Scheme 307).^574^ Specifically, **307.3** was obtained via acid-catalyzed condensation of the pyrene dialdehyde **307.1** with the tripyrrane **307.2**, whereas thiapyreniporphyrin **307.7** was prepared via Lewis acid-catalyzed condensation of the thiophene dicarbinol **307.6** with the pyrene tripyrrane **307.5**. In the free base form, **307.3** and **307.7** were found to be nonaromatic, but showed significant diatropicity upon protonation, as also shown by NICS calculations and ACID plots.

**Scheme 308:**
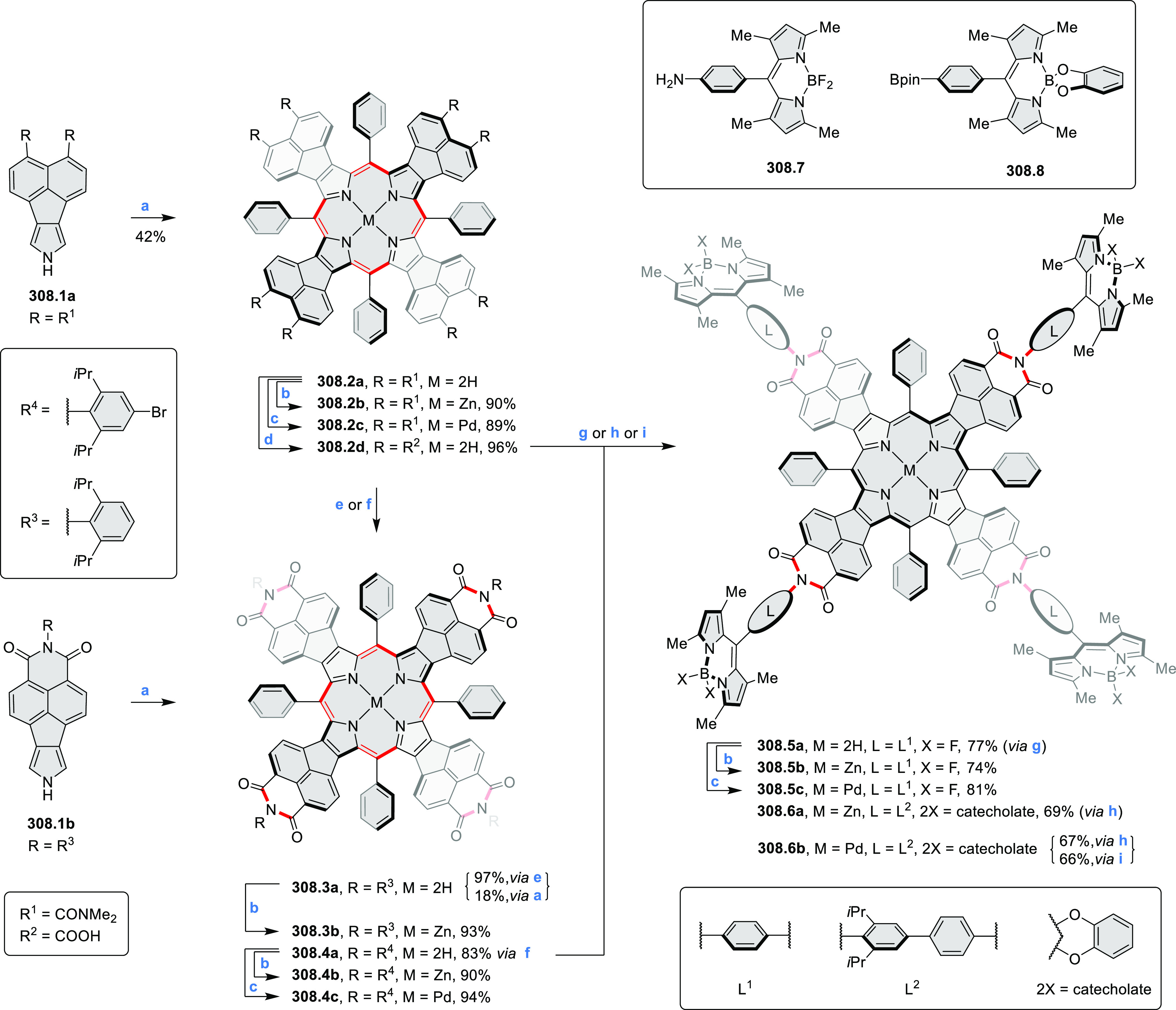
Synthesis of Donor–Acceptor π-Extended Porphyrins Reagents and conditions: (a)^22^ (1) benzaldehyde, *p*-TsOH·H_2_O, CHCl_3_/MeOH (100:1 v/v), (2) DDQ, rt; (b)^22,576^ Zn(OAc)_2_, CHCl_3_/MeOH; (c)^575,576^ Pd(OAc)_2_, CHCl_3_/MeOH; (d)^22^ HCl_conc_, reflux; (e)^22^ 2,6-diisopropylaniline, AcOH, 160 °C; (f)^576^ 4-bromo-2,6-diisopropylaniline, AcOH, 160 °C; (g)^576^**308.7**, AcOH, 160 °C; (h)^576^**308.8**, Pd(dppf)Cl_2_ (0.5 equiv), K_2_CO_3_, 120 °C, toluene/H_2_O (4:1 v/v); (i)^576^ Pd(dppf)Cl_2_ (1.5 equiv), K_2_CO_3_, 120 °C, toluene/H_2_O (4:1, v/v).

In 2016, our group reported a family of band gap-tunable pyrroles structurally related to rylene dyes which were used as building blocks for the synthesis of electron-deficient porphyrin–ryleneimide hybrids (Scheme 308).^22^ The naphthalenediamide-substituted (NDA) macrocycle, **308.2a** was synthesized from the monopyrrole **308.1a** under modified Lindsey conditions, and the porphyrin product was isolated as the trifluoroacetate salt in 42% yield. The naphthalene diimide-fused (NMI) analogue, **308.3a** could be obtained either via direct condensation of the monopyrrole **308.1b** or, more efficiently, by hydrolysis and imidization of **308.2a**. The corresponding metalloporphyrins, **308.2b**–**c** and **308.3b** were obtained from the free-base porphyrins **308.2a** and **308.3a**, respectively. The presence of electron deficient moieties in **308.3b** provided an ability to accept up to 8 electrons via electrochemical reduction and revealed reversible formation of eight charged states, characterized by remarkably red-shifted optical absorptions, extending beyond 2200 nm. The π-extended NDA-fused porphyrins were subsequently explored as potential photosensitizing agents for antimicrobial photodynamic therapy (aPDT).^575^ Their aPDT efficiency could be easily tuned by metal coordination, showing that this family of dyes might be used for treatment of infections caused by Gram-positive bacteria. NMI porphyrins were also incorporated into energy-transfer cassettes containing peripheral BODIPY units.^576^ The free base porphyrin cassette **308.5a** was obtained by condensing the octaacid **308.2d** with the amino-functionalized BODIPY **308.7**, and was converted into metal complexes **308.5b** and **308.5c** (Scheme 308). The Br-functionalized NMI metalloporphyrins **308.4a** and **308.4b** were subjected to Suzuki coupling with the catecholate-protected BODIPY **308.8** to afford the larger analogues **308.6a** and **308.6b**. The femtosecond transient absorption measurements and theoretical predications demonstrated efficient excitation energy transfer pathways in **308.5b** and **308.5c**.

**Scheme 309:**
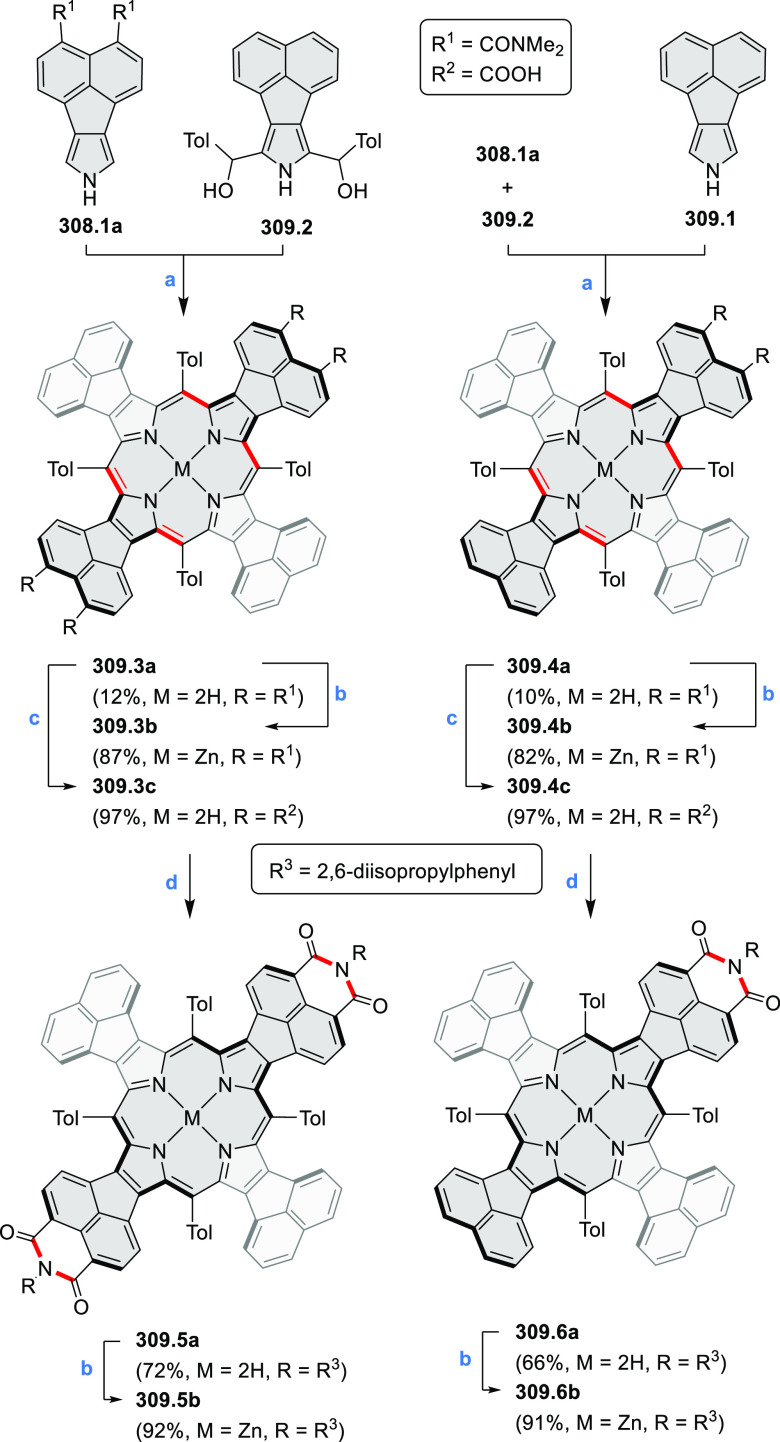
Synthesis of Donor–Acceptor Porphyrins with Mixed Ring Fusion Patterns Reagents and conditions: (a)^577^ (1) **308.1a** and **309.2** (+ **309.1**, for **309.4a**), *p*-TSA, CHCl_3_/MeOH (100:1 v/v), 1 h, (2) DDQ, 2 h; (b) Zn(OAc)_2_·2H_2_O, CHCl_3_/MeOH (3:1 v/v); (c) HCl, reflux, 24 h; (d) 2,6-diisopropylaniline, acetic acid, 20 h, reflux.

Later in 2020, we developed synthetic routes to mixed donor–acceptor porphyrins containing NDA/NMI pyrrole subunits.^577^ The approach shown in Scheme 309 was utilized to circumvent the differential reactivity of electron-rich and electron-poor pyrroles and minimizing the undesired possibilities of reaction products. **309.3a** was prepared in 12% yield by condensing the acenaphthopyrrole dicarbinol **309.2** with one equivalent of diamide pyrrole **308.1a**. Similarly, **309.4a** was obtained from a cross condensation of **308.1a**, the unsubstituted **309.1** and **309.2** in a 1:1:2 molar ratio, to afford the product in 10% yield. Subjecting the two porphyrins to acid hydrolysis followed by imidization with 2,6-diisopropylaniline gave the corresponding imides **309.5a** and **309.6a**. Free bases and zinc complexes of these porphyrins displayed distinct optical properties resulting from desymmetrization, notably more extended NIR bands, with the absorption onset approaching 1000 nm. Interestingly, despite the smaller number of electron-withdrawing NMI moieties, the HOMO–LUMO gap of all these mixed D–A porphyrins were reduced relative to those of the fully symmetrical **308.3a**–**b**.

**Scheme 310:**
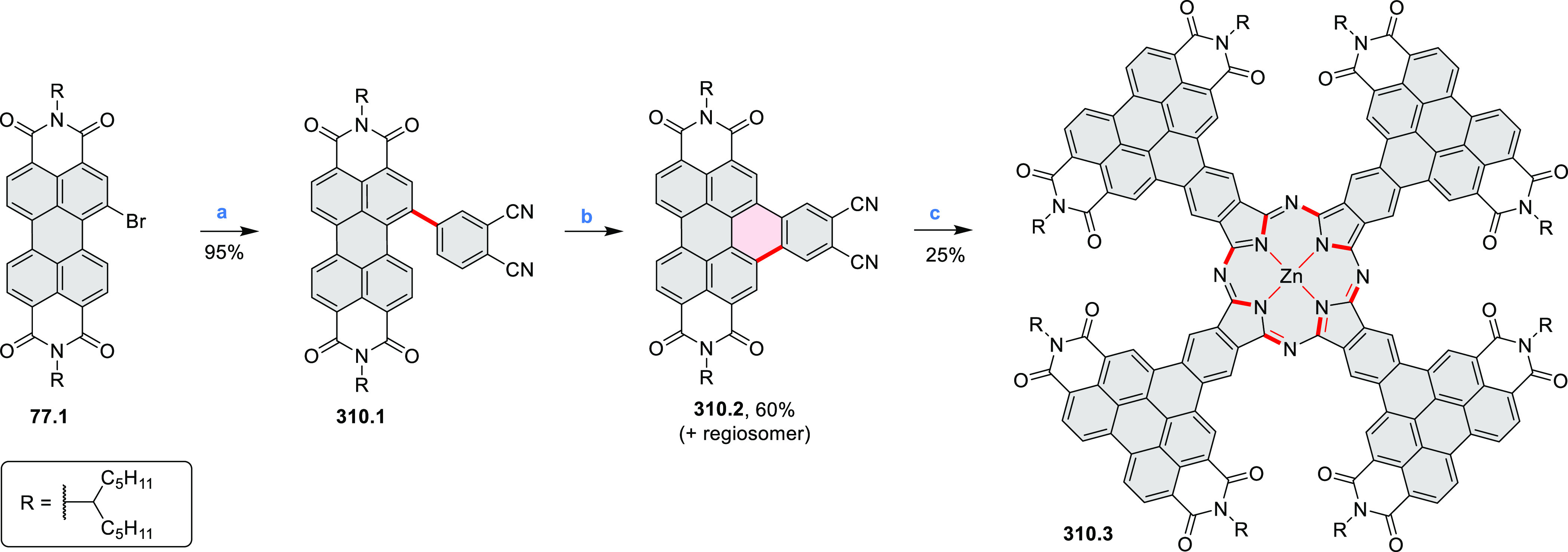
Synthesis of a Rylene-Annulated Phthalocyanine Reagents and conditions: (a)^578^ 3,4-dicyanobenzeneboronate pinacol ester, Pd(PPh_3_)_4_, K_2_CO_3_, THF/H_2_O, reflux, 12 h; (b) *hν*, I_2_, toluene, 95 °C, 48 h, Zn(OAc)_2_, DBU, *n*-hexanol, 160 °C, MW, 1 h.

In 2018, Jiang and Wang reported the synthesis of a rylene-annulated phthalocyanine by integrating electron deficient PDI subunits with the comparatively electron-rich metallophthalocyanine core.^578^ The three-step synthetic route started with the Suzuki–Miyaura cross-coupling of **77.1** with pinacol (3,4-dicyanophenyl)boronate to give **310.1** in 95% yield (Scheme 310). **310.1** was then further irradiated in toluene solution with a catalytic amount of I_2_, to afford **310.2** and its regioisomer. In the final step, zinc(II) phthalocyanine **310.3** was obtained in a microwave-assisted condensation reaction of **310.2** in the presence of zinc acetate and DBU. The four imide-bound branched alkyl chains provided good solubility for **310.3** in chloroform, THF, and o-dichlorobenzene solvents. **310.3** exhibited a nearly planar conformation and showed cage-like packing motif in the solid state. The UV–vis–NIR absorption spectrum of **310.3** displayed an absorption maximum at 785 nm and a high molar extinction coefficient of ca. 300,000 M^–1^ cm^–1^. A significant red-shift of the Q-band relative to the parent nonfused phthalocyanine was attributed to the greater π-electron delocalization in **310.3**.

### 7.2 Benzo[*cd*]-fused porphyrinoids

#### 7.2.1 Benzo[*cd*]-Fusion via *meso-*Substituent Coupling

**Scheme 311:**
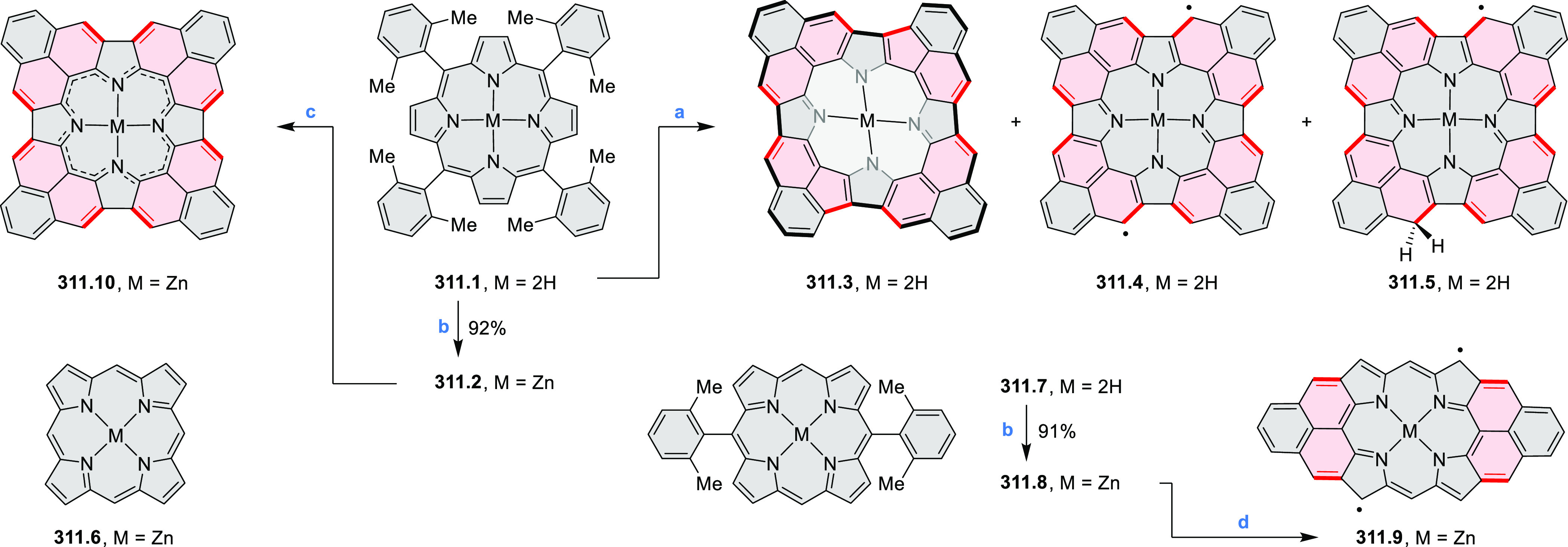
Precise Control of π-Electron Magnetism in Metal-Free Porphyrins Reagents and conditions: (a)^579^ Au(111), 295 °C; (b)^580^ Zn(OAc)_2_, THF, reflux; (c)^580^ Au(111), 300 °C; (d)^580^ Au(111), 330 °C.

In 2020, Zhuang and Wang reported a method of controlling delocalized magnetism in metal-free porphyrins by peripheral fusion of *ortho*-dimethylphenyl substituents.^579^ The three metal-free porphyrins **311.3**–**5** with different π-electron topologies were obtained from the precursor 5,10,15,20-tetrakis(2,6-dimethylphenyl)porphyrin **311.1** using on-surface synthesis on Au(111) (Scheme 311). **311.3** resulted from double methyl group cleavage followed by formation of five-membered rings, whereas **311.5** was a product of incomplete dehydrogenation. On the basis of high-resolution nc-AFM imaging, **311.3** was assigned a closed-shell structure, whereas compounds **311.4**–**5** were described as open-shell species, with two and one unpaired electrons, respectively. The π-electron magnetism in these porphyrins could be switched on/off via scanning tunneling microscope manipulation by tuning the interfacial charge transfer from the Au(111) substrate. In the same year, Fasel and co-workers examined the structural and electronic properties the three zinc porphyrins **311.6**, **311.9**, and **311.10** bearing respectively zero, two, and four *meso-*fused phenalenyl moieties.^580^ Combined experimental and theoretical data revealed a triplet ground state for **311.9** and a charge-transfer-induced open-shell character for the intrinsically closed-shell **311.10**. Formation of intramolecular- and intermolecular-fused products on Cu(111), Au(111) or Ag(110) surfaces was also demonstrated for other free-base or cobalt(II) porphyrins.^581−585^

**Scheme 312:**
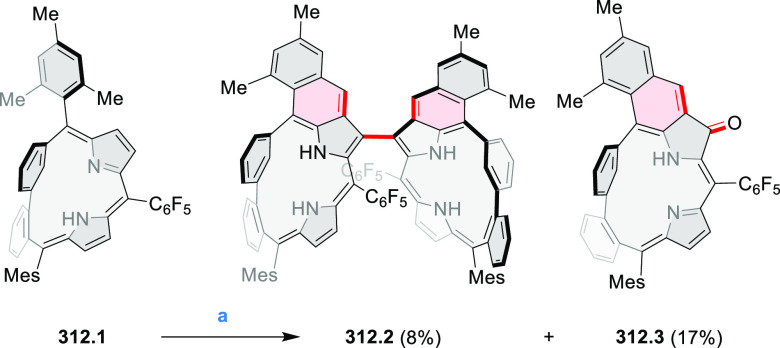
Synthesis of a β–β′-Linked *meso*-Fused Carbatriphyrin(3.1.1) Dimer Reagents and conditions: (a)^586^ PtCl_2_ (6.5 equiv), chlorobenzene, reflux.

A related benzannulation was observed by Peruncheralathan, Srinivasan et al. (Scheme 312). They reported the formation of *meso*-fused β–β′ carbaporphyrin dimer **312.2** and *meso*-fused monomer **312.3**, upon treating carbatriphyrin(3.1.1) **312.1** with PtCl_2_ under aerial conditions.^586^ The molecular structure of **312.2** was confirmed by single-crystal X-ray diffraction, which revealed the fusion of one *meso*-Mes group resulting in the formation of a 1H-benzo[*f*]indole unit. The UV–vis spectrum of **312.2** in DCM showed an intense absorption at 355 nm and a prominent band at 505 nm, while **312.3** showed relatively weaker bands between 400 and 1000 nm.

**Scheme 313:**
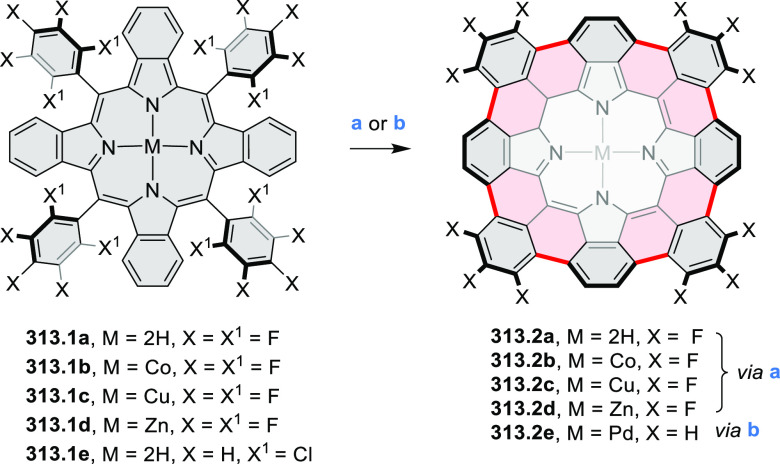
Porphyrin-Based Conical Nanocarbons Reagents and conditions: (a)^587^ gas phase, Δ*E*, N_2_; (b) Pd(PCy_3_)_2_Cl_2_ (8 equiv), DMAc/DBU (v/v 4:1), MW, N_2_, 180 °C, 4 h.

In 2020, Lungerich, Jux, Drewello, and co-workers reported a mass spectrometry-based gas-phase study of a porphyrin-based conical graphene fragment **313.2a**–**d** formed by means of 8-fold *fjord* region-selective cyclodehydrofluorination (Scheme 313).^587^ The γ–ortho cyclization and was found to depend on the choice of metal and functionalized fluorinated *meso-*aryl porphyrins. The coupling mechanism was presumed to consist of C–C nucleophilic addition followed by 1,2-elimination of HF. Attempts to perform an analogous in-solution dehydrochlorination of **313.1e** to produce the fully cyclized product **313.2e** resulted only in the formation of inseparable mixtures of partially coupled products.

**Scheme 314:**
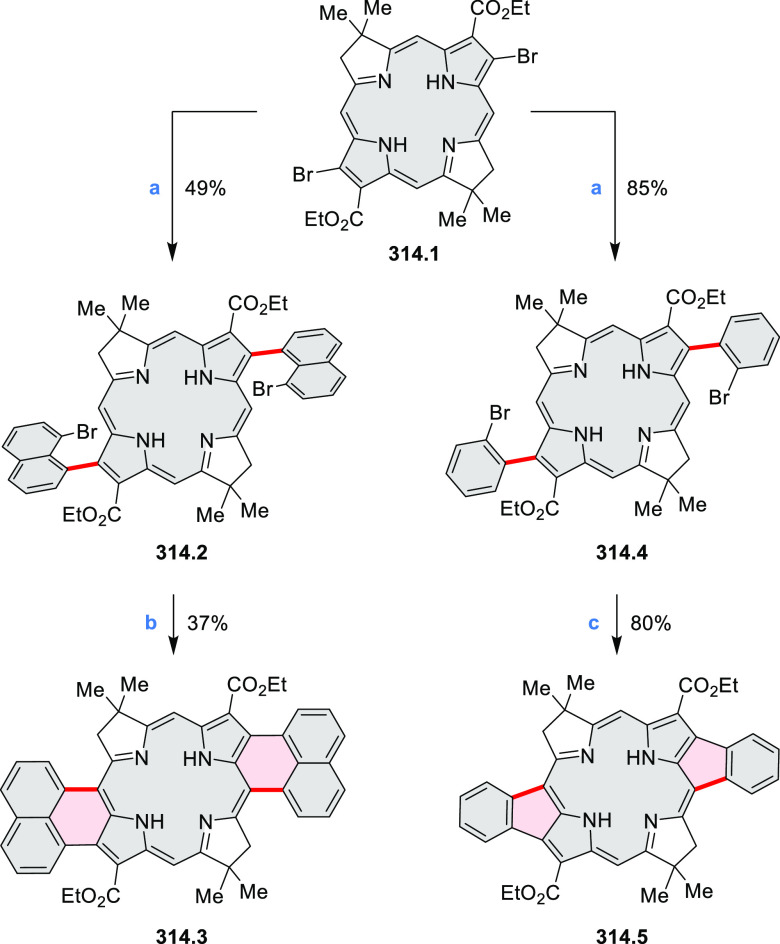
Synthesis of β,*meso-*Annulated Bacteriochlorins Reagents and conditions: (a)^588^ (2-bromophenyl)boronic acid or (8-bromonaphthalen-1-yl)boronic acid, Pd(PPh_3_)_4_, K_2_CO_3_, toluene, DMF, 100 °C; (b) Pd(PPh_3_)_4_, SPhos, K_2_CO_3_, DMF, 80 to 100 °C, 6 h; (c) Pd(PPh_3_)_4_, PCy_3_·HBF_4_, K_2_CO_3_, DMF, 110 °C, 2 h.

In 2019, Holten, Lindsey et al. reported NIR-absorbing bacteriochlorins annulated with aromatic rings (Scheme 314).^588^ Suzuki–Miyaura cross-coupling of **314.1** with (8-bromonaphthalen-1-yl)boronic acid and (2-bromophenyl)boronic acid afforded the arylated precursors **314.2** and **314.4** as mixtures of atropisomers which were used as the mixture in the next step. Palladium-catalyzed intramolecular direct arylation afforded the target β,*meso-*annulated bacteriochlorins **314.3** and **314.5** in 37% and 80% yield, respectively. The UV–vis–NIR absorption spectrum of **314.5** was significantly red-shifted compared to that of **314.3**, with the lowest-energy Q bands lying at 1033 and 913 nm, respectively. These bacteriochlorins were characterized by extremely short lowest excited singlet state lifetimes (7 ps for **314.5** and 150 ps for **314.3**) and vanishingly small fluorescence quantum yields.

**Chart 30:**
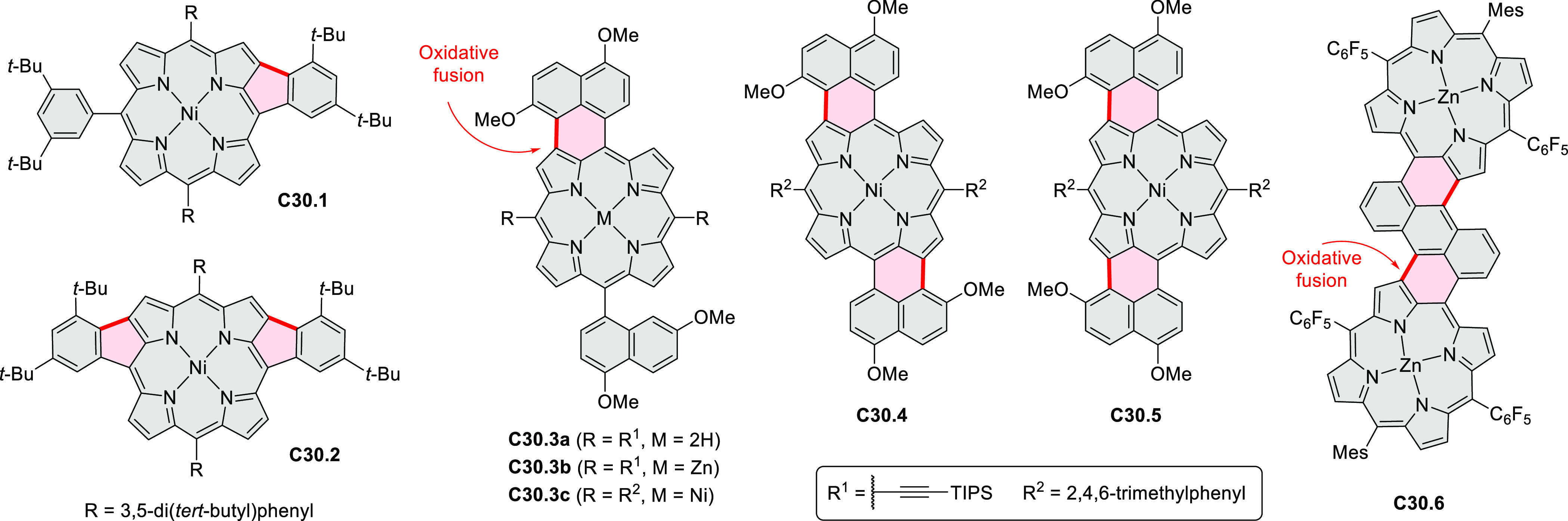
*meso*-β-Pentannulated and Benzannulated Porphyrins

Examples of naphthalene-fused porphyrins **C30.3**(589) and **C30.4**–**5**^590^ obtained using intramolecular oxidative aromatic coupling were reported by Gryko et al. (Chart 30). Prasanthkumar, Giribabu and co-workers developed a related design based on a doubly fused anthracene linker **C30.6** (Chart 30).^591^ The formation a 1:1 charge transfer complex between **C30.6** and PDI was observed spectroscopically in CHCl_3_. The face-to-face π–π stacking responsible for self-assembly of the CT complex led to further aggregation into spherical and nanorod-shaped aggregates, investigated by TEM and AFM microscopy.

#### 7.2.2 Other Benzo-Fused Systems

**Scheme 315:**
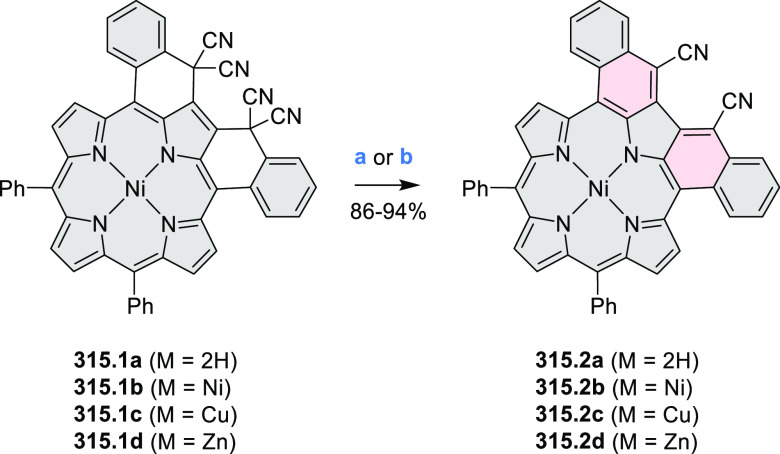
Synthesis of Cyano-Substituted π-Extended Porphyrins Reagents and conditions: (a)^592^ TBAP, DCM, glassy carbon or Pt cathode, controlled applied potential; (b) TBACN, CHCl_3_, stir, 30 min.

In 2020, Sankar and Kadish electrochemical and chemical syntheses of cyano-substituted naphtho-fused π-extended tetraphenylporphyrins from the corresponding tetracyano precursors (Scheme 315).^592^ The electrosynthetic method involved an application of a controlled reducing potential of −1.6 V or −1.7 V at a platinum electrode, followed by an oxidizing potential of 0.0 V in DCM solution, while the chemical synthesis proceeded via a cyanide anion induced electron transfer. Both approaches were found to produce the same decyanated products **315.2a**–**d** in 86–94% yields (Scheme 315). The UV–vis–NIR absorption of **315.2a**–**d** in DCM showed a significant red-shift of the lowest-energy Q bands in comparison with the parent **315.1a**–**d**.

#### 7.2.3 Pyrido[*cd*]fused Systems

**Scheme 316:**
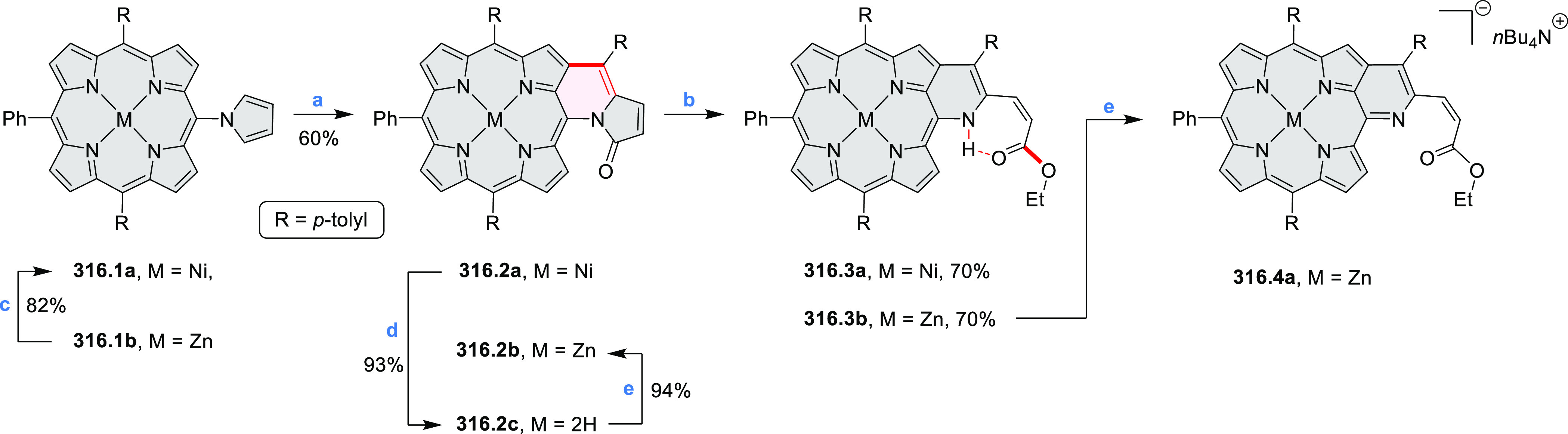
Synthesis of *meso-*Pyrrole Porphyrins Reagents and conditions: (a)^593^ (1) DCM, TFA, *p*-tolualdehyde, rt, 16 h, (2) DDQ (2 equiv); (b) EtONa (10 equiv), Ph_3_P^+^CH_2_PhCl^–^ or Et_4_N^+^Cl^–^ (10 equiv), EtOH, then addition of **316.2a** or **316.2b**, 24 h; (c) (1) TFA (10%) followed by neutralization with Et_3_N, (2) Ni(OAc)_2_, DMF, reflux, 1 h; (d) TFA/H_2_SO_4_ neutralization with Na_2_CO_3_, 2 h; (e) CHCl_3_/MeOH, Zn(OAc)_2_, 1 h; (e) THF, *n*Bu_4_N^+^F^–^, N_2_.

In 2016, Pawlicki et al. reported an indolizinone-fused porphyrin obtained via condensation between a *meso*-*N-*pyrrolyl-substituted porphyrin and an aldehyde.^593^ A transmetalation of **316.1b** yielded **316.1a**, which was condensed with *p*-tolualdehyde and oxidized with DDQ to give the lactam **316.2a** (Scheme 316). **316.2b** was obtained by a transmetalation because direct condensation was ineffective for **316.1b**. Compounds **316.2a** or **316.2b** reacted efficiently with sodium ethoxide in the presence of phosphonium (Ph_3_P^+^CH_2_PhCl^–^) or tetraethylammonium salt (Et_4_N^+^Cl^–^) to give **316.3a** or **316.3b**, respectively. Compound **316.2a** or **316.2b** showed red-shifted absorption reaching the wavelength of about 680 nm and tailing up to 700 nm. Deprotonation of **316.3b** upon titration with *n*-Bu_4_N^+^F^–^ formed **316.4a**, containing a *peri*-fused pyridine ring.

**Scheme 317:**
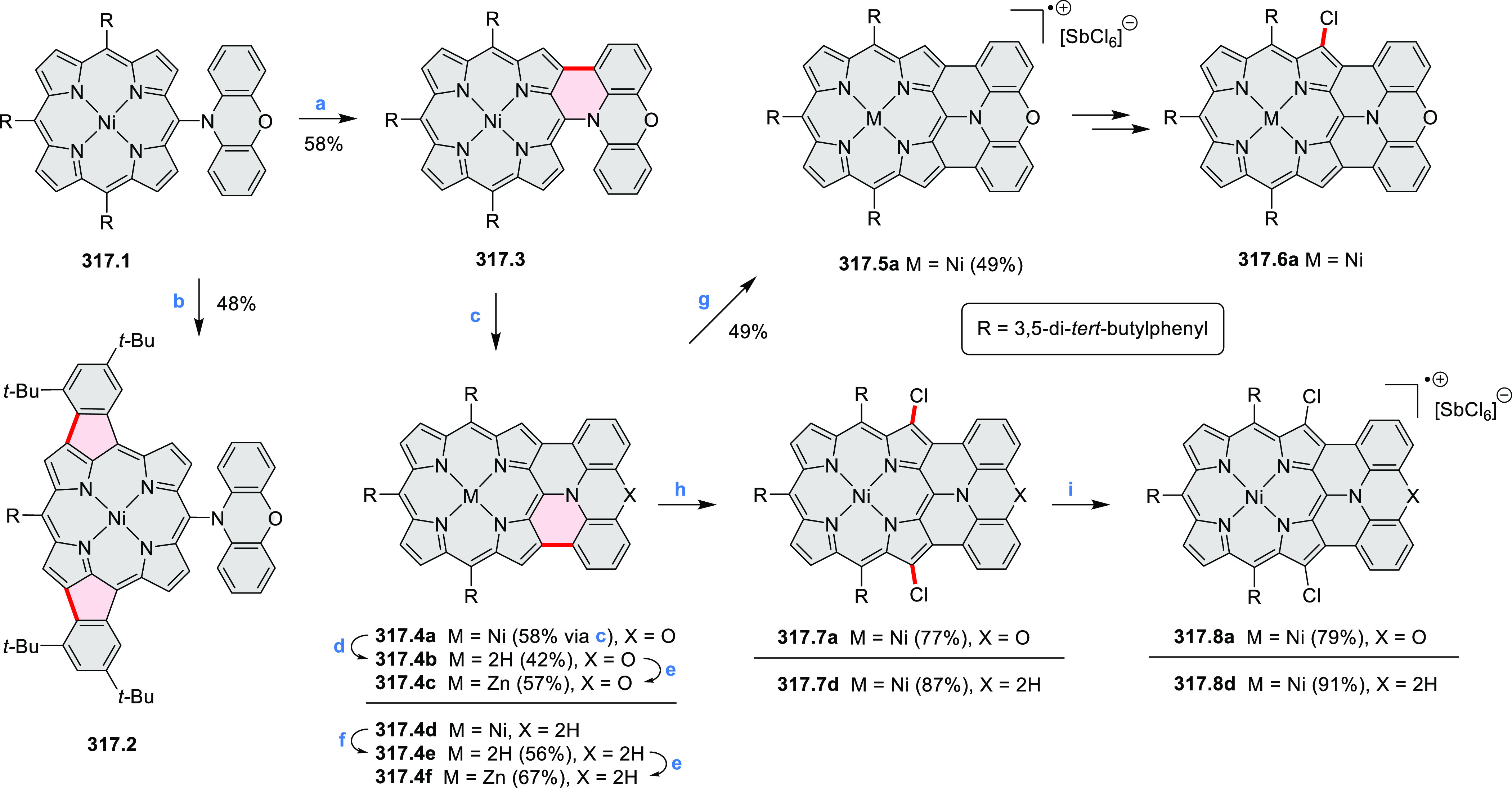
Synthesis of Diarylamine-Fused Porphyrins Reagents and conditions: (a)^594^ DDQ (10 equiv), Sc(OTf)_3_ (10 equiv), ClCH_2_CH_2_Cl/MeNO_2_ 70 °C, 1 h; (b) DDQ (10 equiv), FeCl_3_ (10 equiv), DCM/MeNO_2_, rt; (c) (1) FeCl_3_ (10 equiv), DCM/MeNO_2_, 70 °C, 1 h, (2) HCOOH, NEt_3_, Pd_2_(dba)_3_, SPhos, toluene, 120 °C, 1 h; (d) TFA/conc. H_2_SO_4_; (e) Zn(OAc)_2_·2H_2_O, DCM; (f) (1) *p*-tolylmagnesium bromide, toluene, (2) 3 M HCl_aq_; (g) BAHA (1.1 equiv), DCM, rt, 10 min; (h) 2-chloro-1,3-bis(methoxycarbonyl)guanidine (2.2 equiv), CHCl_3_, 0 °C (10 min) to rt, 2.5 h for **317.7a** and 7 h for **317.7a**; (i) BAHA (1.1 equiv), DCM, rt, 10 min for **317.7a** and 5 min for **317.7a**.

The doubly fused porphyrin **317.4a** was synthesized by Osuka et al. via stepwise oxidation of the *meso-*phenoxazinyl Ni(II) porphyrin **317.1** and was subsequently converted into the corresponding free base porphyrin **317.4b** and its zinc complex **317.4c** (Scheme 317).^594^ In contrast, the oxidation of **317.1** with DDQ and FeCl_3_ at rt resulted in the fusion of two aryl groups, yielding the doubly phenylene-fused porphyrin **317.2**. The authors also transformed the previously reported doubly diphenylamine-fused nickel(II) complex **317.4d** into the free base **317.4e** and zinc complex **317.4f**. The doubly phenoxazine-fused porphyrins exhibited features attributed to their highly planar structures, such as higher fluorescence quantum yields, formation of an offset face-to-face dimer both in solution and the solid state, and generation of a mixed-valence π-radical cation dimer upon electrochemical oxidation. The one-electron oxidation of **317.7a** and **317.7d** using BAHA gave the corresponding dichlorinated radical cations **317.8a** and **317.8d** were found to be much more stable than **317.5a** which was slowly chlorinated at the reactive β-positions.

**Scheme 318:**
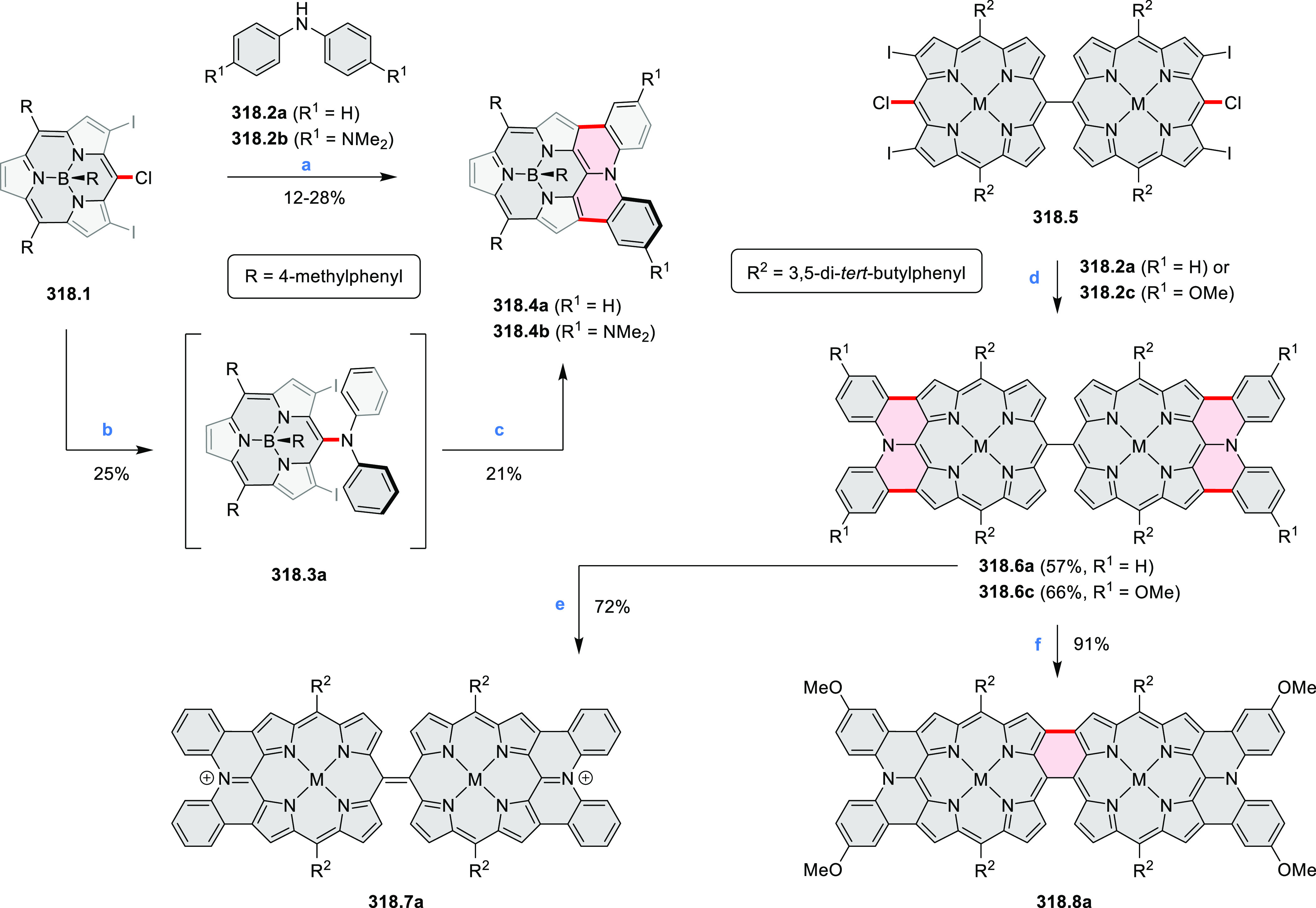
Synthesis of Diarylamine-Fused Subporphyrins and Porphyrin Dimers Reagents and conditions: (a)^595^**318.2a** or **318.2b** (3 equiv), NaO*t*-Bu (12 equiv), DMF, 100 °C, 15 min; (b)^595^**318.2a** (3 equiv), DMF, rt, 30 min; (c)^595^ NaO*t*-Bu (12 equiv), DMF, 100 °C, 10 min; (d)^596^**318.2a** or **318.2c** (2.5 equiv), NaO*t*-Bu (10 equiv), DMF, 100 °C; (e)^596^ BAHA (6.0 equiv), DCM, rt, 10 min; (f)^596^ BAHA (6.0 equiv), DCM, rt, 15 min, then H_2_NNH_2_·H_2_O.

Structurally related diarylamine-fused subporphyrins **318.4a**–**b** were synthesized by the Osuka group through a one-pot procedure involving nucleophilic aromatic substitution followed by electron-transfer-mediated intramolecular C–H/C–I coupling (Scheme 318).^595^ Of these two compounds, **318.4b** showed a more red-shifted absorption attributed to the effective conjugation between the electron-donating dimethylamino group and the subporphyrin core. The fluorescence quantum yields (Φ_f_) of **318.4a** and **318.4b** were found to be 0.21 and 0.18, respectively. In another work by Yorimitsu and Osuka, a similar synthetic strategy led to the formation of meso–meso-linked diphenylamine-fused porphyrin dimers.^596^**318.6a** and **318.6c** were prepared from **318.5** using a similar procedure. Upon oxidation with BAHA, **318.6a** and **318.6c** showed different reactivity, yielding respectively a dicationic closed-shell quinoidal dimer **318.7a** and the doubly linked porphyrin dimer **318.8a**.

**Scheme 319:**
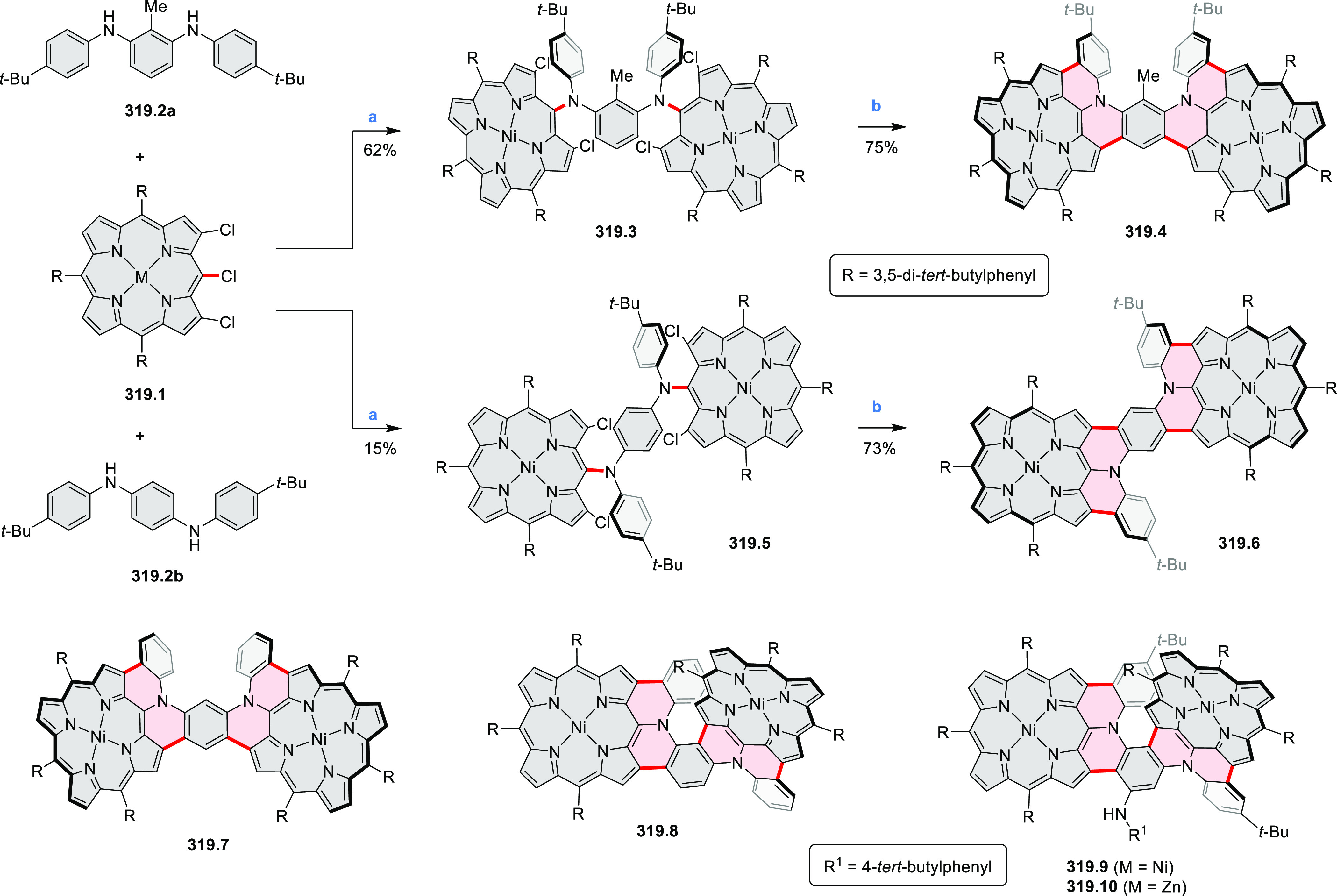
Synthesis of Diarylamine-Fused Porphyrin Dimers Reagents and conditions: (a)^597^*t*-BuONa (11 equiv), DMF, 60 °C, 10 h; (b) Pd(OAc)_2_, PCy_3_·HBF_4_, pivalic acid, K_2_CO_3_ (10 equiv), DMA, 140 °C, 13 h.

Osuka et al. reported *meso*-substituted porphyrin dimers **319.3** and **319.5**, which were synthesized in a S_N_Ar reaction of **319.1** with the corresponding phenylene-diamine **319.2a** or **319.2b** (Scheme 319).^597^ Then, palladium-catalyzed 4-fold C–H arylation conditions on **319.3** and **319.5** led to the formation of fully fused, nonplanar systems **319.4** and **319.6**. The X-ray crystal structure of **319.4** revealed that the two [4]helicene-like segments had opposite helicities, whereas in **319.6**, the corresponding two segments were homochiral. Both fused-porphyrin dimers revealed a highly reversible electrochemical oxidation and produced dication diradicals upon chemical oxidation. A related synthesis, reported later by the same authors, produced an inseparable mixture of **319.7** and its helically twisted isomer **319.8**, which could not be separated.^598^ A helically twisted nitrogen-doped fused porphyrin dimer **319.9** was however synthesized using benzene-1,3,5-triamine via *t*-BuONa-promoted one-pot reaction. Zinc complex **319.10**, prepared from **319.9**, showed fluorescence at around 650–700 nm in DCM with a quantum yield (Φ_f_) of 0.04.

**Scheme 320:**
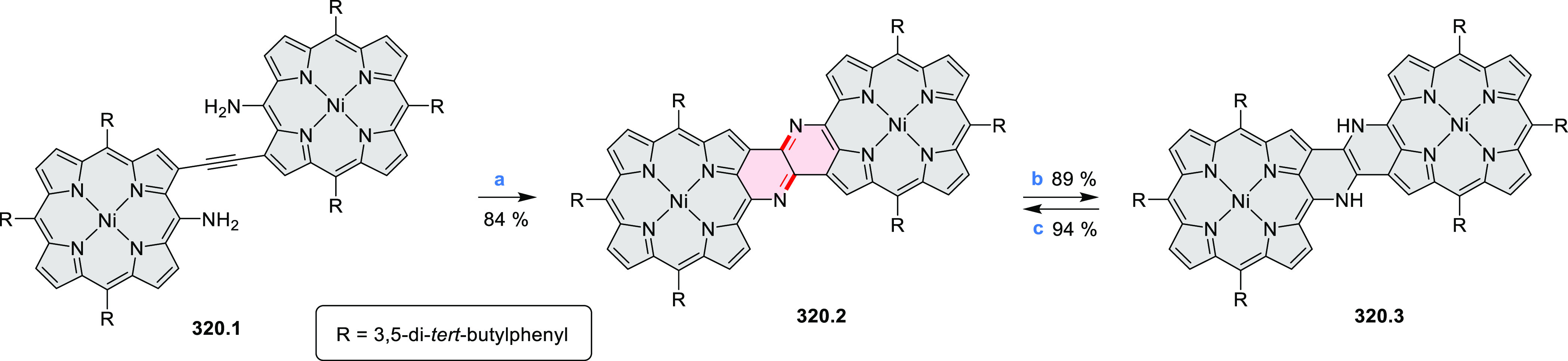
Synthesis of a 1,5-Naphthyridine-Fused Porphyrin Dimer Reagents and conditions: (a)^599^ PtCl_2_, toluene, rt, 3 h, and then, PbO_2_, DCM, rt, 1 h; (b) NaBH_4_, DCM/MeOH, rt, 15 min; (c) PbO_2_, DCM, rt, 5 min.

A synthesis of 1,5-naphthyridine-fused porphyrin dimer **320.2** was developed in 2018 by Osuka and co-workers (Scheme 320).^599^ The platinum catalyzed intramolecular cyclization of β-to-β ethynylene-bridged *meso*-amino Ni(II) porphyrin dimer **320.1** resulted in the formation of a six-membered ring via a 6-*endo*-dig cyclization and the subsequent oxidation with PbO_2_ afforded **320.2** in 84% yield. The redox active 1,4-diazabutadiene unit in **320.2** was found to be interconvertible with its reduced 1,2-diaminoethene linkage in **320.3** via reduction with NaBH_4_ and oxidation with PbO_2_. The UV–vis absorption of **320.2** in DCM showed an intense absorption band at 1011 nm in the NIR region which is drastically different than that of **320.3**, suggesting a substantial difference in their electronic interaction between the two porphyrin units via fused 1,4-diazabutadiene or 1,2-diaminoethene linkage. The electrochemical HOMO–LUMO gap **320.2** and **320.3** was found to be 0.99 and 1.8 eV, respectively.

**Scheme 321:**
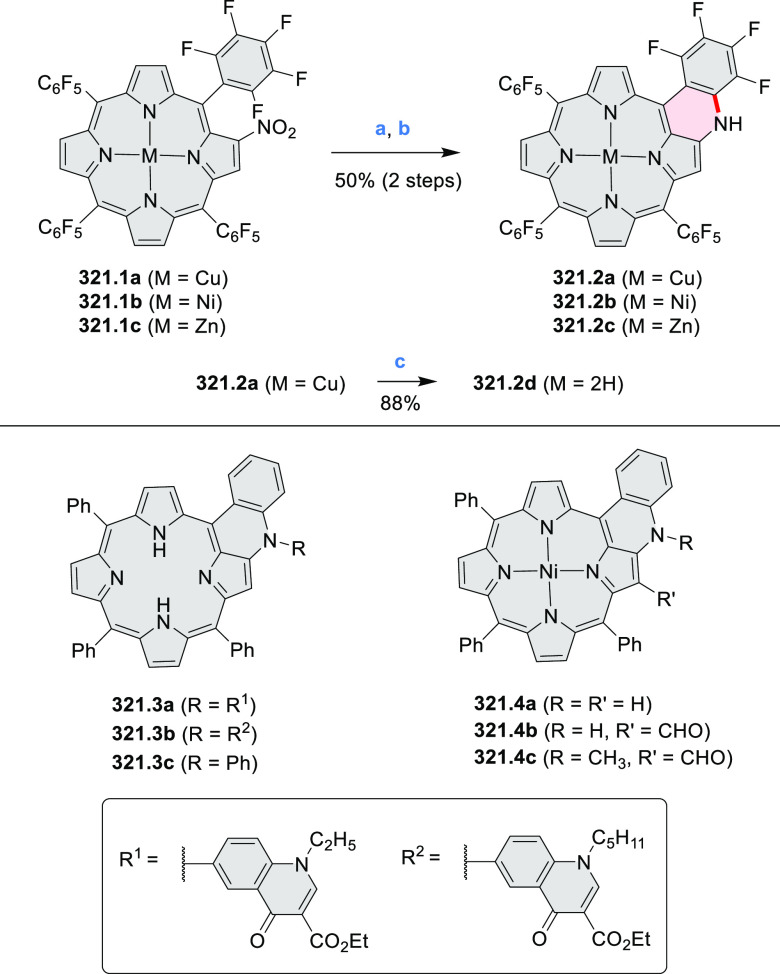
Quinolino-Fused Porphyrins Reagents and conditions: (a)^600^ NaBH_4_, 10% Pd/C, N_2_, CH_3_CN/EtOH, rt, 10 min; (b) CH_3_CN, 1 h, reflux; (c) TFA, conc. H_2_SO_4_.

In 2020, Brückner and co-workers described a route to quinoline-annulated metalloporphyrins involving an intramolecular S_N_Ar displacement of an *o*-fluorine atom on a *meso*-pentafluorophenyl group by the neighboring β-amino substituent (Scheme 321).^600^ The reduction of the nitro group in **321.1a**–**c** led to the formation of a β-aminoporphyrin, which were then directly used in the next step involving an intramolecular SNAr-type annulation reaction to afford the desired products **321.2a**–**c** in 50% isolated yields (over two steps). The free base **321.2d** was also obtained by demetalation of the copper complex. The quinoline-annulated porphyrins **321.3a**–**c**, obtained by Boechat, Neves, Cavaleiro and co-workers via oxidative cyclization of corresponding β-aminoporphyrins, were used as potential photodynamic photosensitizers.^601^ Among these, **321.3a** was found to most efficiently inactivate Gram-positive bacteria *S. aureus*, because of efficient singlet oxygen generation and the less hydrophobic character, which enhanced its affinity for bacteria. The quinoline-fused nickel(II) complex **321.4a**, obtainable via Cadogan-type cyclization, was derivatized in a variety of ways (e.g., **321.4b**–**c**^602^). An isocyanide monomer containing *N-*appended **321.4a** was copolymerized with poly(3-hexylthiophene) to afford a photovoltaic donor material.^603^ Furthermore, isatin–porphyrin conjugates were obtained via peripheral Buchwald–Hartwig amination of **321.4a**.^604^

**Scheme 322:**
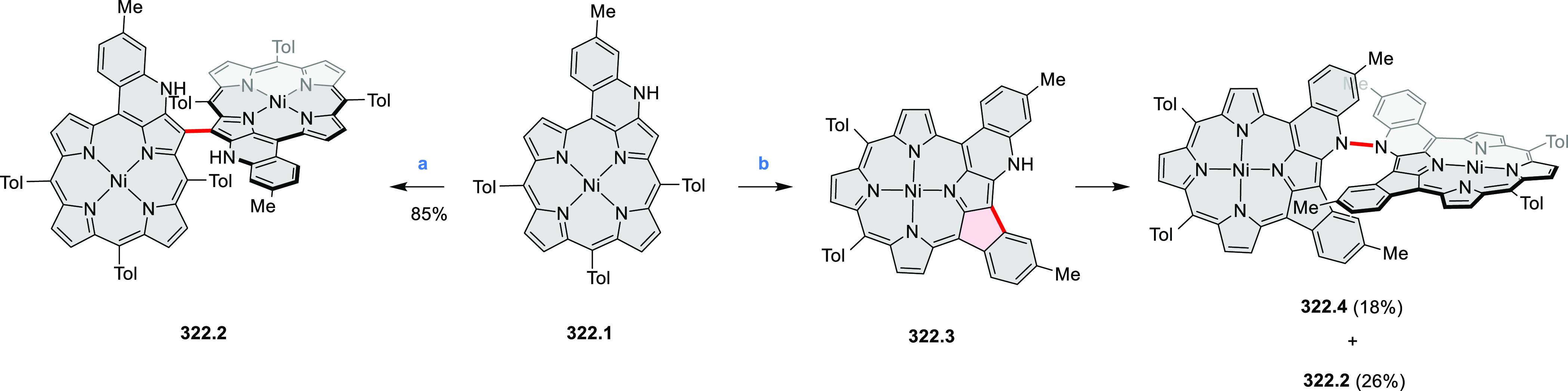
Oxidative Coupling of Enaminoporphyrin Reagents and conditions: (a)^605^ Fe(phen)_3_(PF_6_)_3_, Na_2_CO_3_, DCM, rt; (b) PIFA, Na_2_CO_3_, DCM, rt.

The porphyrin dimer **322.2** was obtained by Ruppert et al. upon subjecting **322.1** to various monoelectronic oxidants, with best results obtained when using 0.55 equiv of [Fe(phen)_3_]^3+^ in the presence of base (Scheme 322).^605^ Another porphyrin dimer, **322.4** was obtained in 18% yield upon slow and simultaneous addition of equimolar solution of **322.1** and PIFA to a suspension of sodium carbonate in DCM. Intramolecular cyclization was preferred under these conditions, leading to the formation of an intermediate **322.3**, which subsequently afforded the N–N linked dimeric product **322.4**. The UV–vis absorption spectrum of **322.4** had the lowest-energy Q-band at 675 nm, somewhat red-shifted relative to that of **322.2**.

**Scheme 323:**
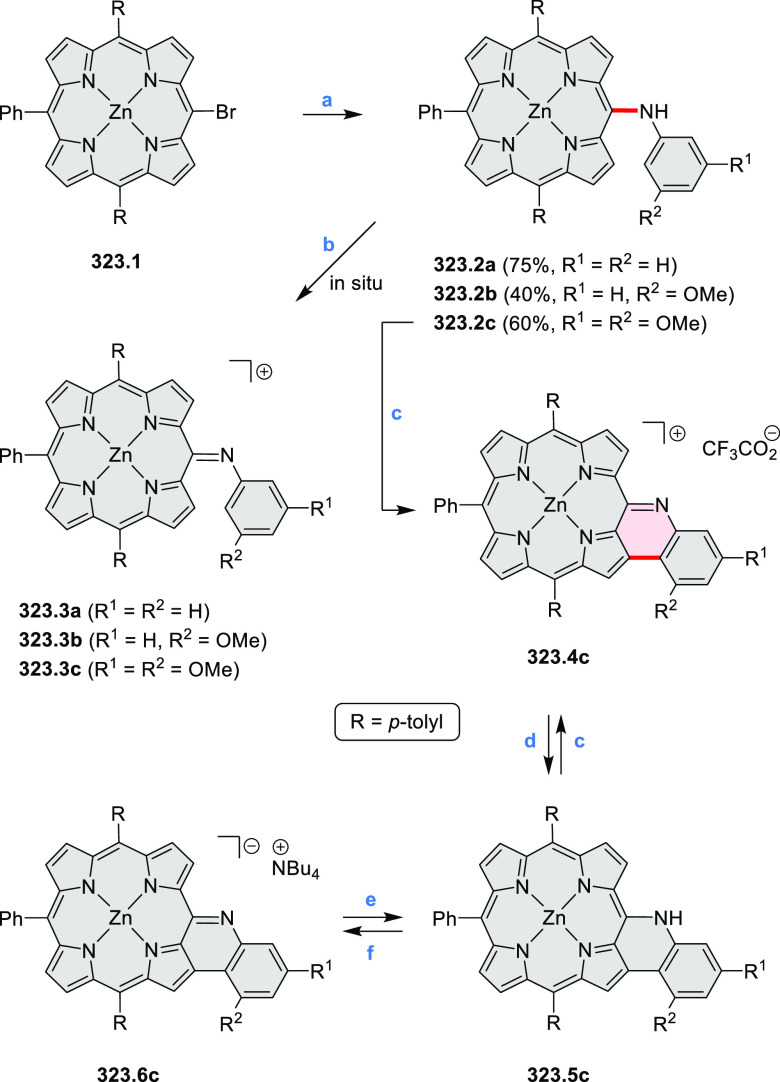
Synthesis of Fused *meso-*Aminoporphyrins Reagents and conditions: (a)^606^ arylamine (5 equiv), Pd(OAc)_2_, DPEPhos, Cs_2_CO_3_, THF, 65 °C, 3 h; (b) DDQ, DCM; (c) DDQ, DCM, TFA; (d) NaBH_4_, THF; (e) HCl; (g) DCM; (f) TBAF, THF.

In 2015, Pawlicki and Latos-Grażyński reported a NIR chromophore based on fused *meso*-aminoporphyrin, switchable using a redox process or protonation of the *meso*-nitrogen.^606^ The synthesis consisted of the Buchwald–Hartwig amination of **323.1** followed by attempted oxidative cyclization of the resulting **323.2a**–**c** (Scheme 323). The latter step was relatively more effective for **323.2c**, yielding ultimately the fused product **323.4c** (with **323.3c** as an intermediate), whereas **323.2a** and **323.2b** were only oxidized to produce the corresponding imine cations **323.3a**,**b**. **323.4c** and its reduced form **323.5c** were mutually convertible via a two-electron redox process. In addition, **323.6c** could be obtained by deprotonation of **323.5c**. Each of these species showed different electronic properties, reflected by significant changes in the absorption and fluorescence spectra.

**Scheme 324:**
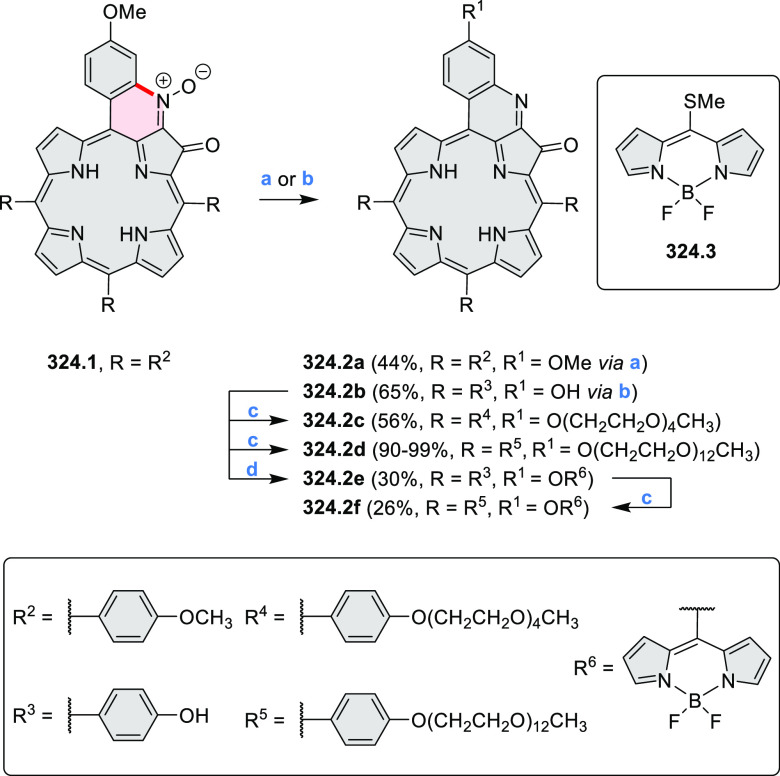
Quinoline-Annulated Porphyrins Reagents and conditions: (a)^608^ pyridine, reflux, 48 h; (b) BBr_3_, DCM; (c) Me(OCH_2_CH_2_)_4_OMs or Me(OCH_2_CH_2_)_12_OMs, Cs_2_CO_3_, DMF, 90 °C; (d) (1) Zn(OAc)_2_·2H_2_O, DCM/MeOH, (2) **324.3** (1 equiv), Na_2_CO_3_, copper(I) thiophene-2-carboxylate, CH_3_CN, 50 °C, (3) HCl.

Brückner and Zhu demonstrated the use of quinoline-annulated porphyrin derivatives as potential contrast agents for photoacoustic imaging (PAI), however, their insolubility in aqueous solutions prevented the *in vivo* assessment.^607^ To obtain water-soluble products that derivatives be useful for tumor tomography, the group employed the hydroxy-substituted **324.2b**, which was PEGylated with methyl-capped PEG-mesylates to provide **324.2c** with tetraethylene glycol chains, as well as **324.2d** with longer chains (*n* ∼ 12), or substituted with a fluorescent BODIPY tag (**324.2e**–**f**, Scheme 324).^608^**324.2c** was found to be only slightly soluble in pure water whereas **324.2d** was freely soluble in alcohols, water, serum, and PBS buffer. **324.2d** absorbed strongly within the spectroscopic window and was very weakly fluorescent. It had low acute toxicity, accumulated in the tumor site, and was excreted unaltered via renal pathways.

**Scheme 325:**
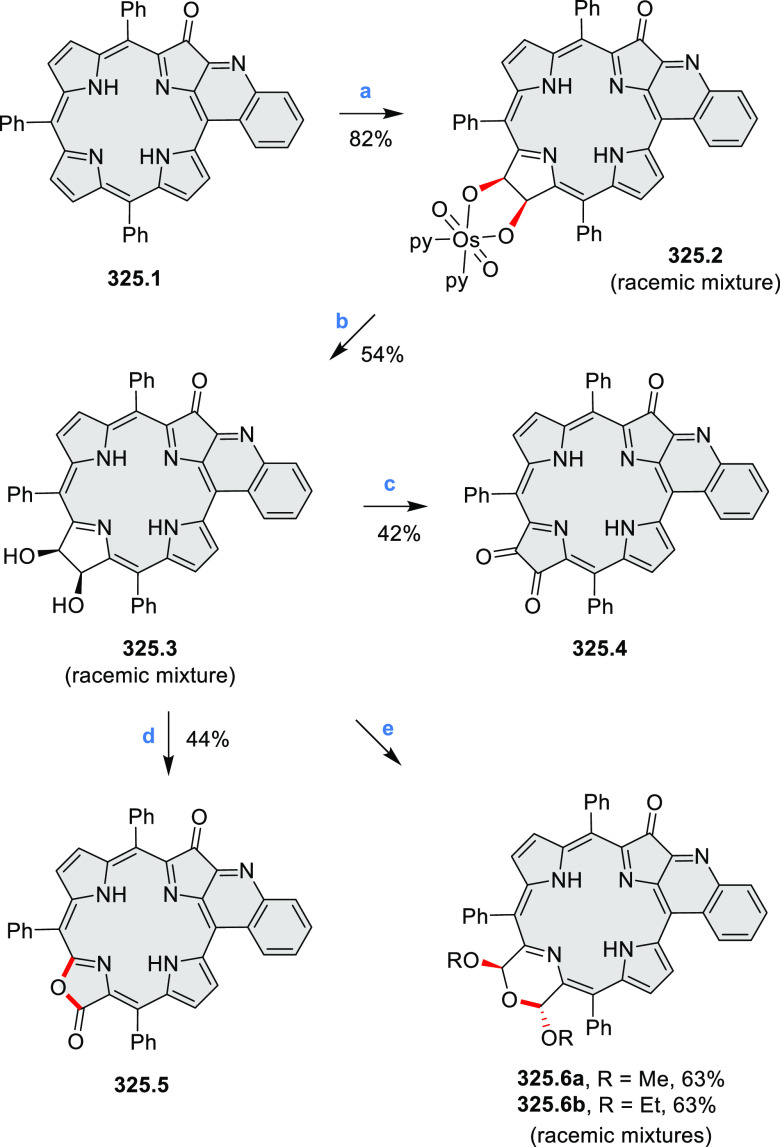
Synthesis of Quinoline-Annulated Chlorin and Derivatives Reagents and conditions: (a)^609^ OsO_4_, pyridine, CHCl_3_, rt; (b) H_2_S, CHCl_3_, rt; (c) DMP, DCM, rt; (d) DCM, CTAP; (e) NaIO_4_/silica, EtOH, or MeOH.

In 2016, Brückner described the OsO_4_-mediated dihydroxylation of quinoline-annulated porphyrins generating a quinoline-annulated dihydroxychlorin in a regioselective fashion and highlighted its importance as an effective strategy to red-shift the absorption spectra of these annulated chlorin analogs.^609^ The osmate ester **325.2** was obtained in 84% yield by reacting the quinoline-annulated porphyrin **325.1** with stoichiometric amounts of OsO_4_. **325.2** was then treated with H_2_S to afford the quinoline-annulated dihydroxychlorin **325.3** in 54% yield as a racemic mixture (Scheme 325). The oxidation of **325.3** using Dess–Martin periodinane (DMP) resulted in the formation of dione product **325.4**. The direct conversion of a pyrrole β, β′bond to a lactone moiety was made by treating **325.3** with cetyltrimethylammonium permanganate (CTAP) which yielded **325.5** as regioisomers (2-oxa-3-oxo-quinoline annulated porphyrin or 3-oxa-2-oxoquinoline annulated porphyrin). Compounds **325.6a**–**b** were obtained from the oxidative cleavage of diol **325.3** using sodium periodate. The UV–vis absorption spectrum of **325.6a** were found to be much more red-shifted than the other derivatives reaching up to 1000 nm, while **325.3** showed the lowest energy Q like band at 842 nm.

#### 7.2.4 Pyrano- and Thiopyrano[*cd*]fused Systems

**Scheme 326:**
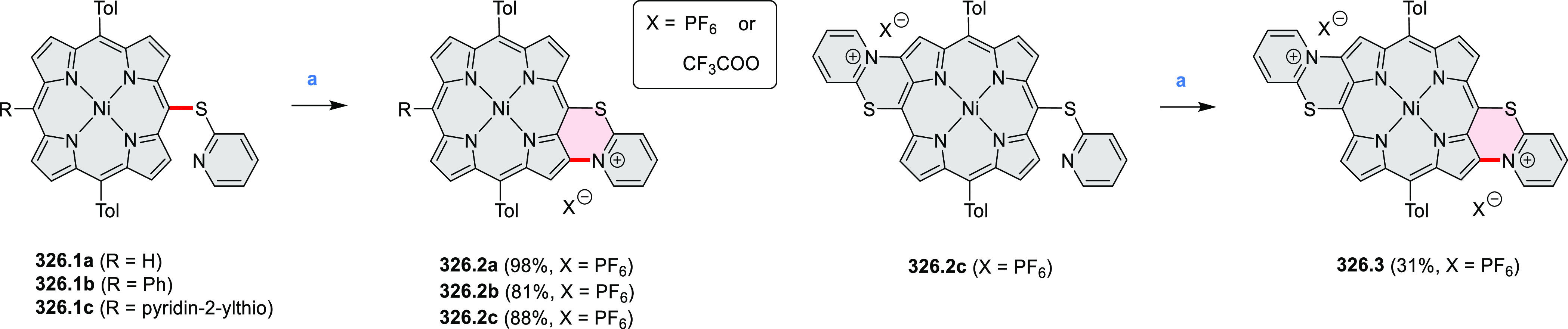
Intramolecular Oxidative C–N Coupling of Pyridin-2-ylthio-*meso-*substituted Porphyrins Reagents and conditions: (a)^610^ (1) PIFA (1 equiv), DCM, rt, (2) anion exchange: X = CF_3_COO to PF_6_.

In 2018, Devillers described chemical and electrochemical syntheses of C–N fused pyridinium-containing porphyrins from precursors bearing a pyridin-2-ylthio *meso*-substituent (Scheme 326).^610^ The chemical oxidation of nickel(II) porphyrins **326.1a**–**c** using 1.2 equiv of PIFA produced fused cationic derivatives, which were isolated as hexafluorophosphate salts **326.2a**, **326.2b**, and **326.2c** in 98%, 81%, and 88% yield, respectively. Furthermore, the chemical oxidation of **326.2c** with 1 equiv of PIFA followed by anion exchange step led to the regioselective formation of doubly C–N fused dicationic pyridinium-based porphyrin **326.3**. These singly and doubly C–N-fused products were also obtained via exhaustive electrolysis under mild conditions.

**Scheme 327:**
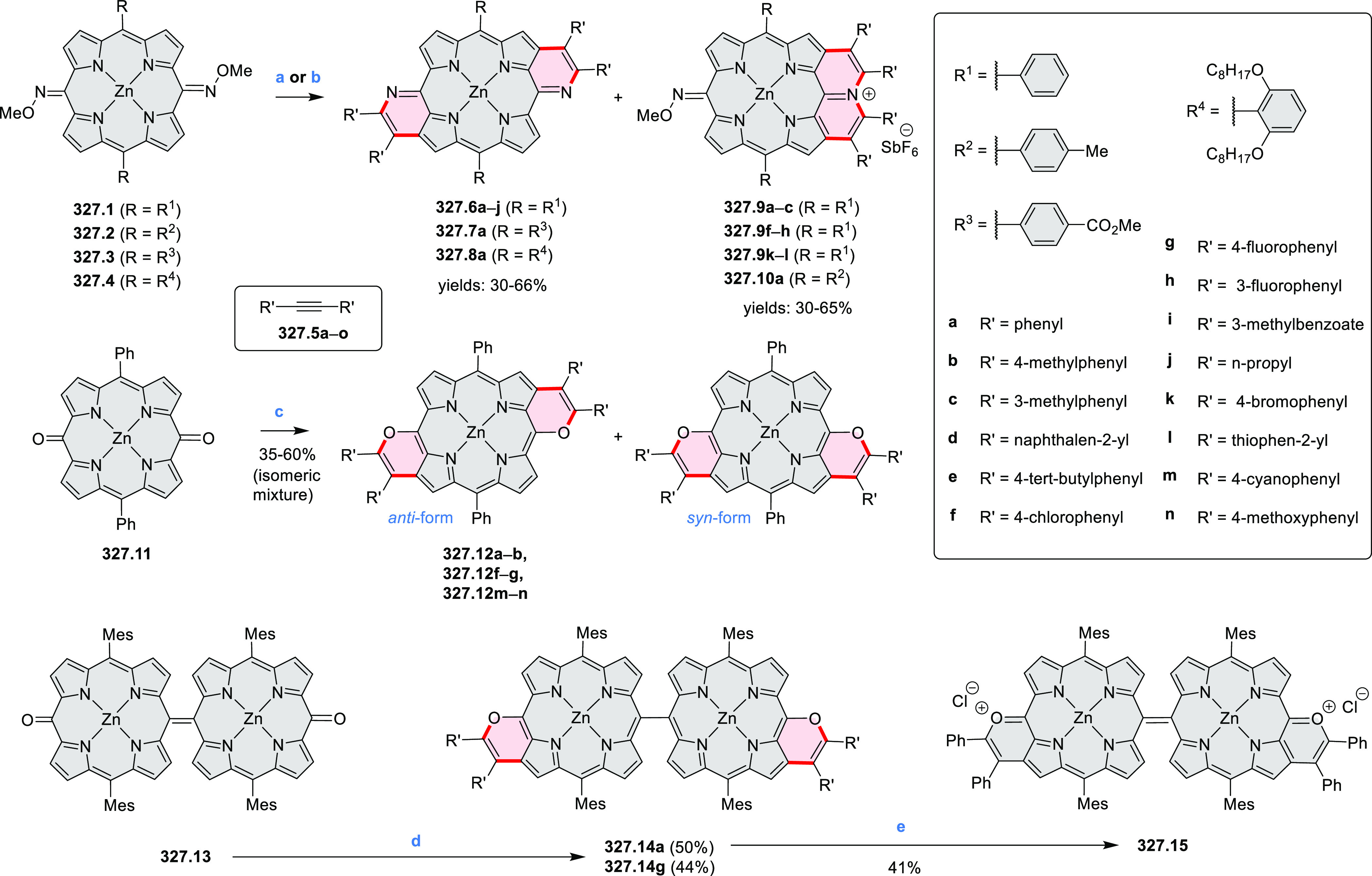
Synthesis of *meso-N*/*O*-Heteroarene-Fused Porphyrins via a [4 + 2] Oxidative Annulation Strategy Reagents and conditions: (a) Standard conditions for **348.6**–**8**: **348.5** (0.2 mmol), [Cp*RhCl_2_]_2_ (5 mol %), AgSbF_6_ (20 mol %), THF (0.5 mL), 100 °C, N_2_, 24 h; (b) standard conditions for **348.9**–**10**: **348.5** (0.2 mmol), [Cp*RhCl_2_]_2_ (5 mol %), AgSbF_6_ (20 mol %), Ag_2_O (2.0 equiv), NaSbF_6_ (2.0 equiv), DCE (0.5 mL), 120 °C, N_2_, 24 h; (c) **348.5** (0.2 mmol), [Cp*RhCl_2_]_2_ (5 mol %), AgSbF_6_ (20 mol %), Ag_2_O (2.0 equiv), DCE, or 1,4-dioxane (0.5 mL), 120 °C, N_2_, 24 h; (d) [Cp*RhCl_2_]_2_ (5 mol %), AgSbF_6_ (20 mol %), Ag_2_O (2.0 equiv), DCE, or 1,4-dioxane (0.5 mL), 120 °C, N_2_; (e) FeCl_3_ (10 equiv), DDQ (10 equiv), DCM/MeNO_2_.

In 2019, D. Wu and J. You et al. described a divergent synthesis of various *meso-N*/*O-*heteroarene-fused porphyrins via rhodium-catalyzed [4 + 2] annulation (Scheme 327).^611^ The reaction of **327.1** and diphenylacetylene in the presence of [Cp*RhCl_2_]_2_ (5 mol %), AgSbF_6_ (20 mol %), and Ag_2_O (2 equiv) afforded the doubly pyridine-fused *anti*-quinoidal porphyrin **327.6a** as a major product. In the same reaction, a high polarity product was also obtained which was identified as the pyridinium-fused **327.9a**. With the optimized reaction conditions, the scope of this reaction was extended to a variety of alkynes with either electron-donating or electron-withdrawing groups, affording the desired products **327.6**–**10** with controllable chemoselectivity and complete *anti*-selectivity.

The synthesis of *meso-O-*containing heteroarene-fused porphyrins **327.12** was carried out under similar conditions using the 5,15-dioxoporphyrin **327.11** and diphenylacetylene as the reaction substrates. The doubly pyran-fused porphyrin **327.12a** was obtained as a mixture of *syn*- and *anti*-isomers. The scope of this reaction was extended to a variety of alkynes, which typically produced regioisomeric mixtures, which could occasionally be separated chromatographically. Using this annulation approach, the doubly pyran-fused porphyrin dimers **327.14a** and **327.14g** were also prepared in 50% and 44% yields, respectively. Upon oxidation of **327.14a** with FeCl_3_ and DDQ, the doubly pyrylium-fused porphyrin dimer **327.15** was obtained, which displayed intense near-infrared (NIR) Q bands reaching up to 1300 nm in DCM. ACID and NICS(1) analyses suggested the presence of 22π aromatic conjugation in *syn*/*anti*-**327.14a**, reflecting the involvement of the pyrylium rings in π conjugation.

#### 7.2.5 Benzo-Fused Porphyrin Oligomers

**Scheme 328:**
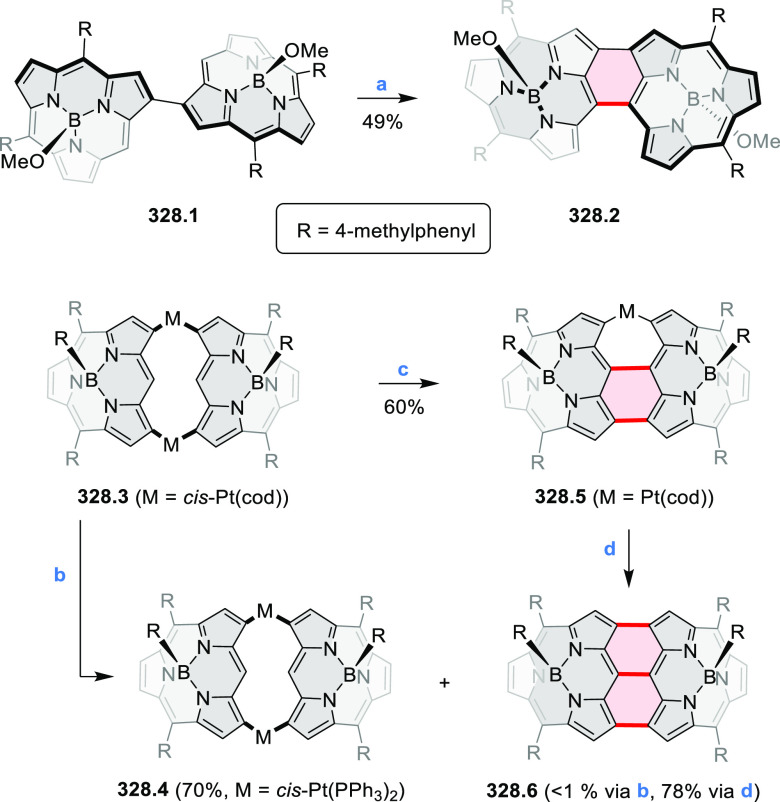
Synthesis of Doubly and Triply Fused Subporphyrin Dimers Reagents and conditions: (a)^612^ (1) NBS, CHCl_3_, 0 °C, 3 h, (2) Ni(cod)_2_, 1,5-cod, DMF, 80 °C, 3 h; (b)^613^ PPh_3_ (10 equiv), toluene, 120 °C, 3 h; (c)^613^ BAHA (4.0 equiv), DCM, rt, 1 h, then H_2_NNH_2_·H_2_O; (d)^613^ dppp (5 equiv), toluene, 120 °C, 1 h.

The preparation of a fused subporphyrin dimer **328.2** was reported in 2016 by Kim, Osuka and co-workers.^612^ The bromination of **328.1** followed by intramolecular Yamamoto coupling led exclusively to the anti diastereomer of fused subporphyrin dimer **328.2** (Scheme 328). The unobserved syn isomer was predicted by DFT calculations to have a higher energy than the anti form. The UV–vis absorption spectrum of **328.2** in DCM exhibited a broad Soret-like band and Q-like band at 641 nm with a tail reaching ca. 850 nm, considerably red-shifted relative to **328.1**, and showed a significantly smaller electrochemical HOMO–LUMO gap of 1.72 eV. The same authors reported the synthesis of triply linked subporphyrin dimer **328.6** by stepwise reductive elimination of the doubly Pt^II^-bridged subporphyrin dimer **328.3** (Scheme 328).^613^ Attempted reductive elimination of **328.3** resulted in the ligand-exchanged product **328.4** and a trace amount (<1%) of fused dimer. In contrast, treatment of **328.3** with BAHA gave the doubly fused Pt^II^-bridged dimer **328.5** in 67% yield and subsequently, further reductive elimination resulted in the formation of the desired triply linked subporphyrin dimer **328.6** in 78% yield. **328.6** had a domed structure with a positive Gaussian curvature and a bowl-depth of 1.65 Å. The UV–vis spectrum of **328.6** in DCM showed the lowest-energy absorption band at 942 nm and a small electrochemical HOMO–LUMO gap of 1.35 eV, suggesting the effective π-conjugation extending over the whole molecule.

**Scheme 329:**
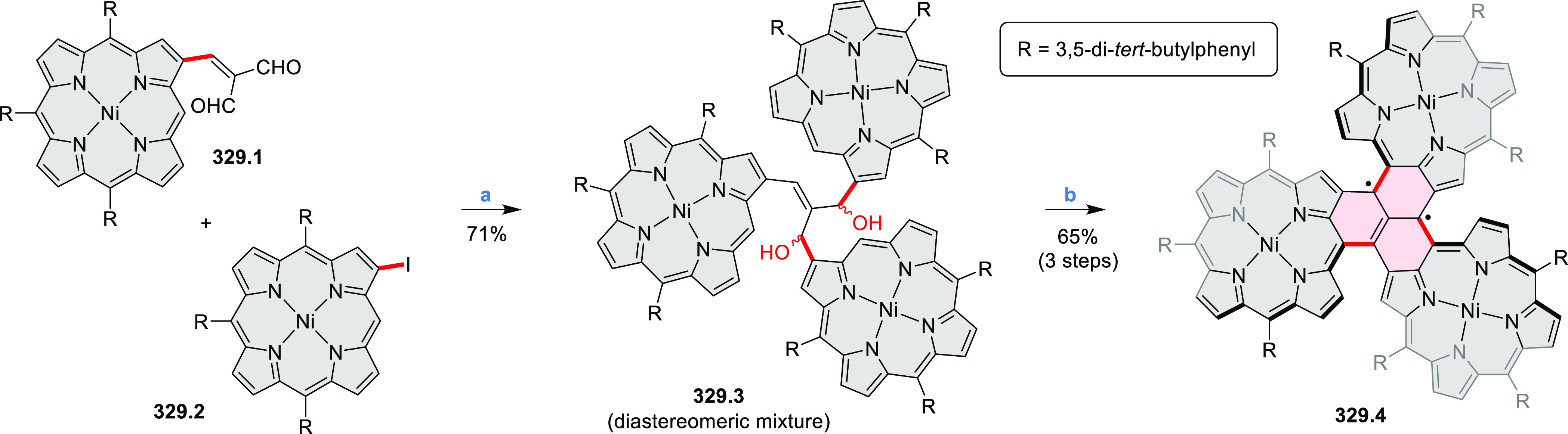
Synthesis of a Trimethylenemethane Diradical Fused with Three Porphyrins Reagents and conditions: (a)^614^*i*-PrMgCl·LiCl (1.1 equiv), THF, −40 °C, then **329.1** (0.4 equiv), −40 °C to rt, 2 h; (b) (1) 25% H_2_SO_4_, 1,2-dichloroethane, 60 °C, 11 h, (2) DDQ (10 equiv), DCM, rt, 15 min, (3) ascorbic acid (10 equiv), DCM/MeOH.

In 2018, Furukawa, Osuka et al. reported the synthesis of a trimethylenemethane (TMM) diradical fused with three nickel(II) *meso*-triarylporphyrins (Scheme 329).^614^ Dialdehyde **329.1** was reacted with two equivalents of a porphyrinylmagnesium reagent derived from **329.1**, to afford the diol **329.3** as a 4:1 mixture of diastereomers. The acid-mediated cyclization of **329.3** followed by DDQ oxidation resulted in a highly polar product which was identified as the corresponding radical cation, which was further reduced with ascorbic acid to yield the TMM diradical **329.4**. The diradical **329.4** was found be quite stable under ambient conditions because of the extensively delocalized spin density. The X-ray crystal structure of **329.4** revealed a pseudo-*C*_3_-symmetric propeller-like conformation, in which the three hetero[5]helicene moieties adopted a twisted helical conformation with the same twist direction. The lower limit of Δ*E*_ST_ from SQUID measurements was estimated for **329.4** to be +2.8 kcal mol^–1^ (*J*_1_/*k*_B_ ≈ 715 K), consistent with a theoretical value of Δ*E*_ST_ ≈ +3.0 kcal mol^–1^ (*J*_1_/*k*_B_ ≈ 750 K). These results indicated a triplet ground state, reflecting a strong ferromagnetic interaction between the spins. **329.4** displayed strong absorptions in the NIR region (700–1350 nm) and four-step redox interconversion with a narrow electrochemical gap of 0.48 V.

**Scheme 330:**
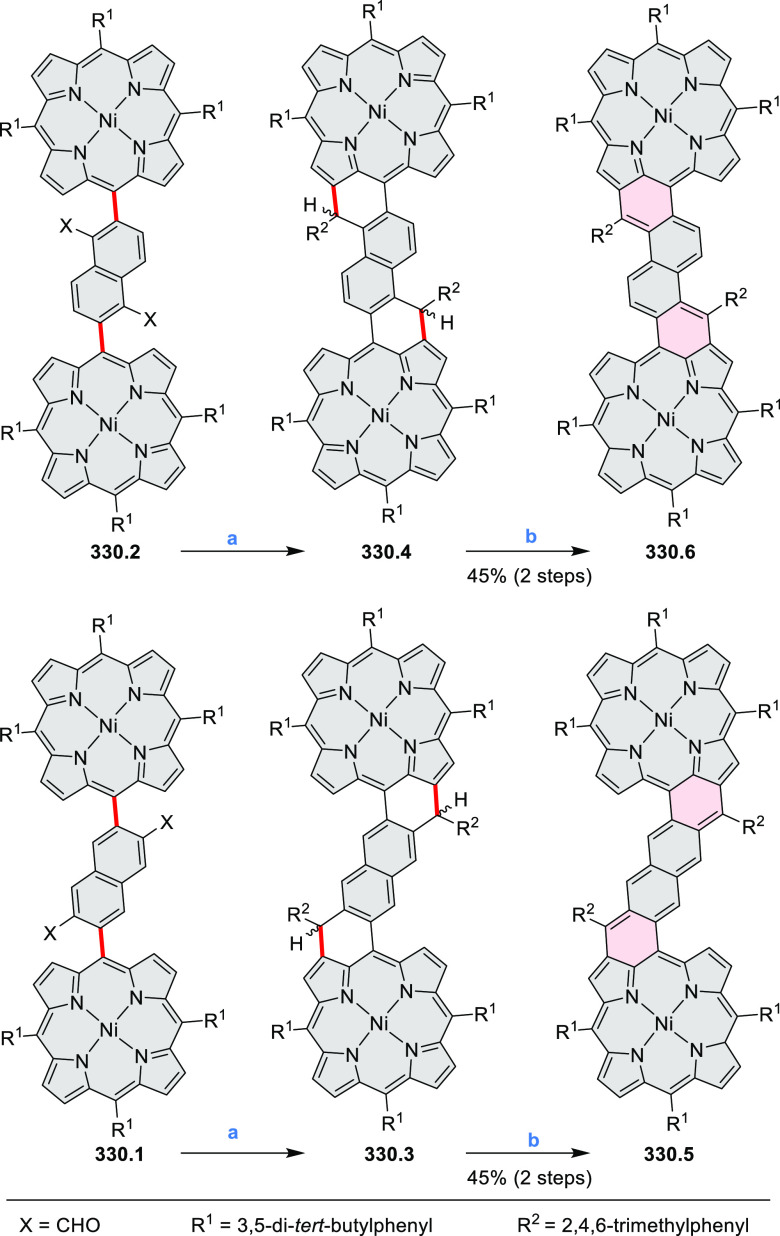
Synthesis of Naphthoquinodimethane-Bridged Porphyrin Dimers Reagents and conditions: (a)^615^ (1) 2-mesitylmagnesium bromide, dry THF, 8 h, rt, (2) BF_3_·OEt_2_, dry DCM, 5 min, rt; (b) DDQ, dry toluene, rt.

2,6-Naphthoquinodimethane- and 1,5-naphthoquinodimethane-bridged porphyrin dimers were synthesized in 2019 by Wu et al. (Scheme 330).^615^**330.1** and **330.2** were treated with 2-mesitylmagnesium bromide to give the corresponding diols, which were then subjected to BF_3_·OEt_2_ mediated Friedel–Crafts alkylation reaction to give the dihydro precursors **330.3** and **330.4**, respectively. In the subsequent step, the oxidative dehydrogenation of **330.3** and **330.4** with DDQ afforded the target product **330.5** and **330.6**, both in 45% yield over three steps. The compound **330.5** showed greater stability, which facilitated its isolation via silica gel column chromatography. **330.6** exhibited a larger diradical character, a smaller singlet–triplet energy gap (Δ*E*_ST_ = −2.60 kcal mol^–1^) and a smaller two-photon absorption (TPA) cross section value compared to the **330.5** isomer.

**Scheme 331:**
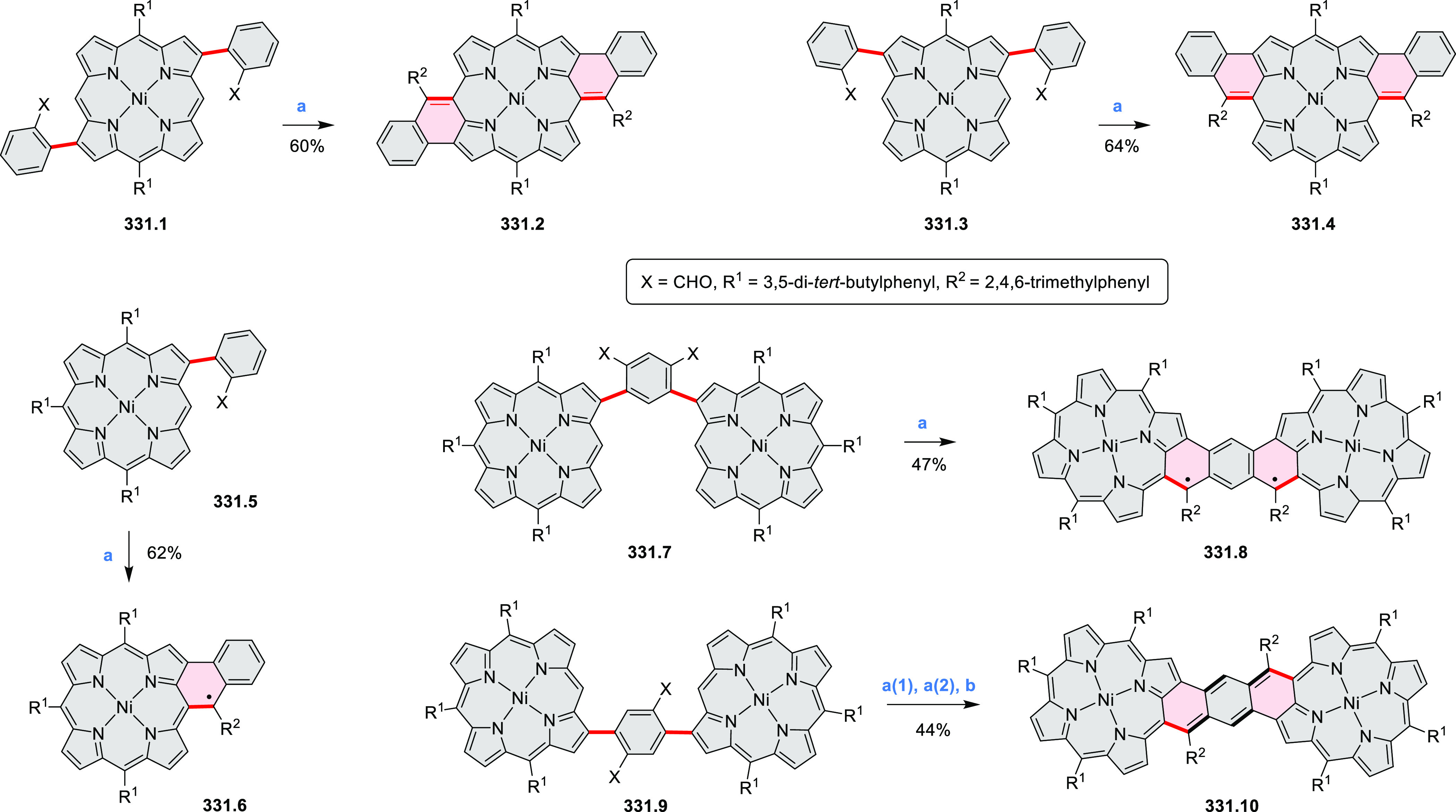
Synthesis of Singly and Doubly Naphthalene-Fused Porphyrins and Anthracene-Bridged Porphyrin Dimers Reagents and conditions: (a)^616^ (1) 2-mesitylmagnesium bromide, dry THF, rt, (2) BF_3_·OEt_2_, dry DCM, (3) DDQ, DCM; (b) *t*-BuOK, THF.

The syntheses of doubly naphthalene-fused porphyrins and *syn*- and *anti*-fused-anthracene-bridged porphyrin dimers were reported in 2021 by Song et al. (Scheme 331).^616^ In a representative example, **331.5** was reacted with 2-mesitylmagnesium bromide to provide corresponding carbinol, and intramolecular Friedel–Crafts alkylation and subsequent oxidation with DDQ afforded **331.6** in 62% yield. Similarly, other derivatives **331.2**, **331.4**, **331.8**, and **331.10**, were obtained from corresponding aldehyde containing porphyrin precursors over three steps. In the case of **331.10**, the final oxidation step was carried out using *t-*BuOK/O_2_. The monoradical **331.6** and the open-shell syn dimer **331.8** were found to be very stable: the half-life of **331.8** in 1,2-dichlorobenzene under ambient air and at 80 °C was about 28 days, despite its high-spin triplet ground-state carbon diradical character. The electrochemical HOMO–LUMO gaps of **331.6** and **331.8** were estimated to be 0.62 and 0.97 V, respectively.

#### 7.2.6 Oxobenzo- and Oxonaphtho-Fused Porphyrinoids

**Scheme 332:**
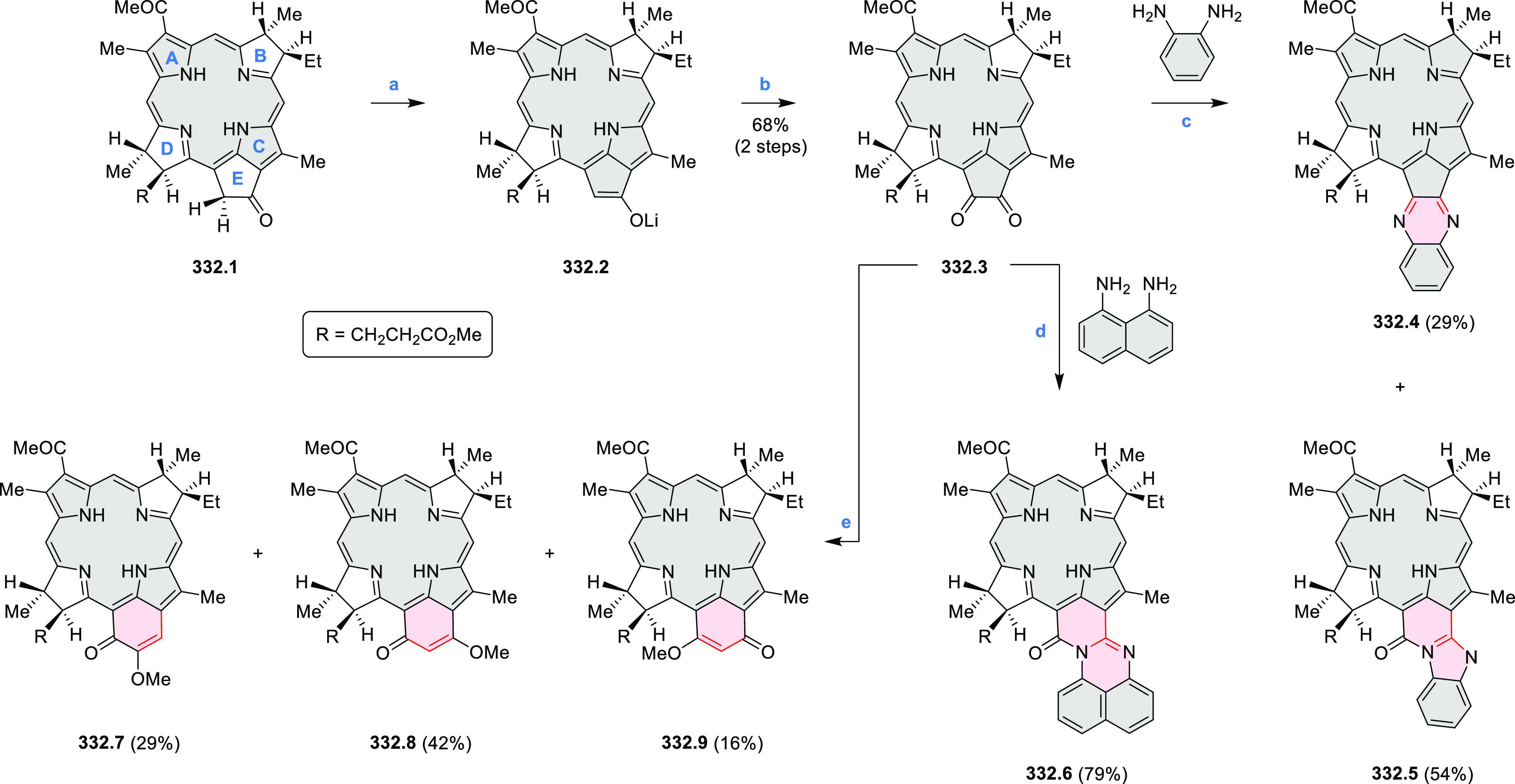
Synthesis of Bacteriochlorins Bearing Fused Quinoxaline, Benzimidazole, and Perimidine Aromatic Rings Reagents and conditions: (a)^619^ LiOH/H_2_O, THF, 24 h; (b) O_2_; (c) 1,2-phenylenediamine hydrochloride, pyridine, TFA, reflux; (d) 1,8-diaminonaphthalene hydrochloride, pyridine, TFA, reflux; (e) CH_2_N_2_, DCM, *N-*methyl-*N-*nitroso-*p*-toluenesulfonamide, rt, overnight.

Bacteriochlorophylls and their derivatives such as **332.1** possess a highly reactive β-ketoester moiety in ring E, which has been used to develop π-extended derivatives with various fused ring systems (Scheme 332).^617,618^ In 2012, Kozyrev et al. reported the synthesis of bacteriochlorins bearing fused quinoxaline, benzimidazole, and perimidine aromatic rings.^619^ The synthetic design aimed to induce large bathochromic shifts of Q_*y*_ absorption bands by simultaneously extending the conjugation on pyrrole C and introducing electron-withdrawing substituents on the opposite pyrrolic unit A. An *in situ* autoxidation of **332.1** using aqueous LiOH in THF for 24 h followed by an acidic workup and re-esterification with diazomethane, afforded the bacteriochlorin α-diketone **332.3** in 68% yield. Using **332.3** as the starting material, various highly conjugated annulated bacteriochlorins **332.4**–**6** were synthesized by condensing with diamines in the presence of pyridine/TFA. Additionally, the annulated cyclohexenenone ring systems **332.7**–**9** were also synthesized using the diazomethane ring-enlargement approach. These derivatives showed the largest bathochromic shifts, with Q_*y*_ absorptions in the range of 870–890 nm. More recently, radionuclide-containing analogues of **332.7** were investigated as PET imaging agents.^620^

**Chart 31:**
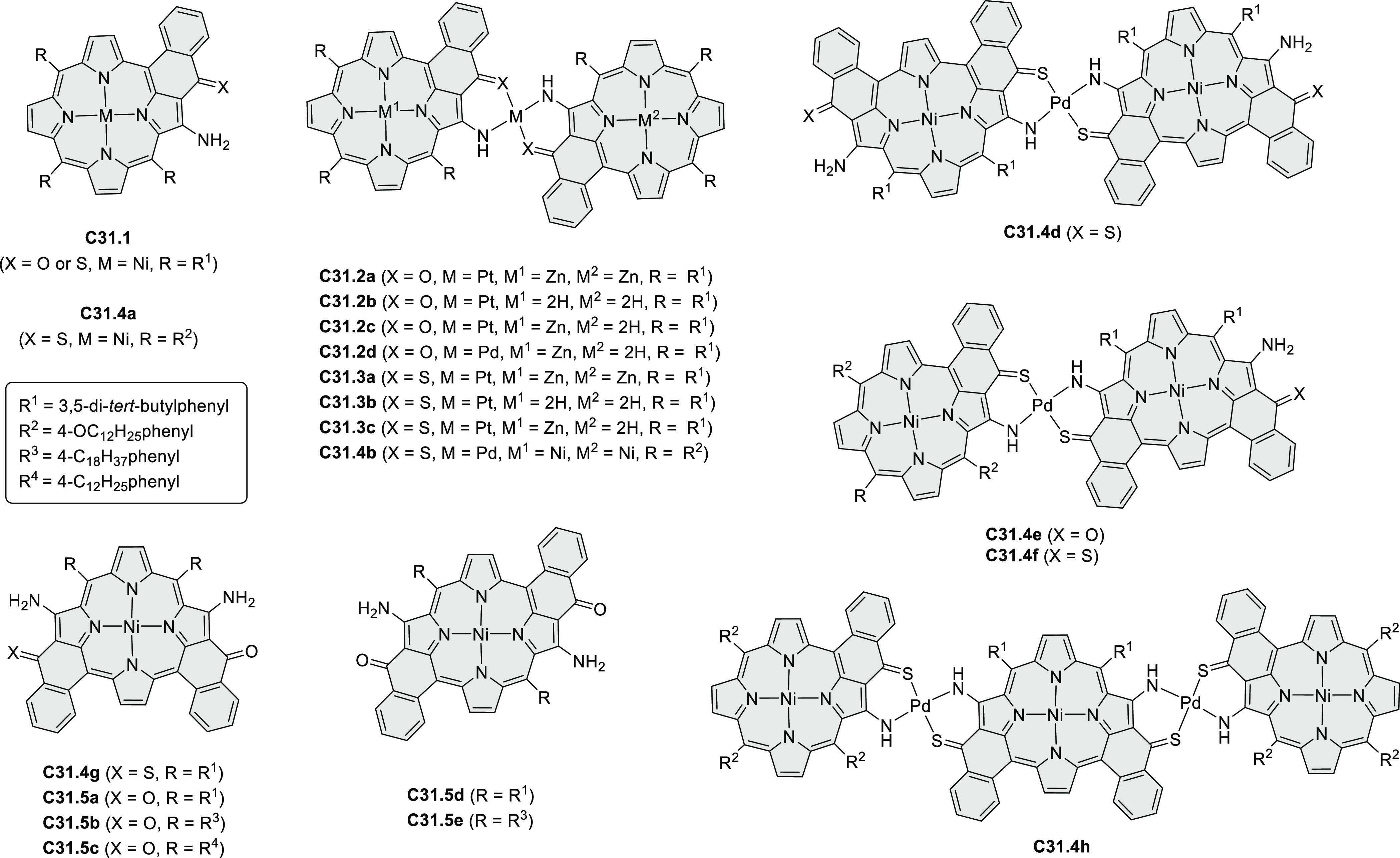
Porphyrin Oligomers Linked by Metal Ions

In a recent report, Ruppert, Harvey et al. investigated electronic communication in platinum(II)-bridged porphyrin dyads **C31.2**–**3** (Chart 31, for earlier work, see CR2017, Section 7.2.6).^621,622^ These systems revealed an extremely fast rate of S_1_ singlet energy transfer with very small S_1_ lifetimes of 2.1 ps for **C31.2b**, 0.8 ps for **C31.2a**, and 105 fs for **C31.2c**. The rate of singlet energy transfer, k_ET_(S_1_), for dyad **C31.2c** was found to be 8.3 × 10^12^ s^–1^ which was 5.5-fold faster than that of palladium(II) linked dyad **C31.2d**. In the enaminothioketone dyad **C31.3c** the S_1_ energy transfer process occurred faster than 49 fs, i.e., more rapidly than in **C31.2c** (M= Pt, X = O, 105 fs) and **C31.2d** (M= Pd, X = O, 650 fs). The rate of energy transfer for **C31.3c** (k_ET_(S_1_)≥ ∼ 15 × 10^12^ s^–1^) was found to be higher than ever reported for both natural systems and laboratory model compounds and this enhanced rate was ascribed to a significant increase in MO coupling and predominance of the Dexter mechanism.

Self-assembly of enaminoketone and enaminothioketone monomers, dimers, and trimers at a highly oriented pyrolytic graphite (HOPG)/liquid interface was explored by Kikkawa and Ruppert (Chart 31).^623^ Among all the compounds tested, the dodecyloxyphenyl-substituted monomer **C31.4a**, and dimers **C31.4b** and **C31.4f** showed the formation of highly ordered two-dimensional self-assembled structures. In a later report, nanoribbons were built from symmetrical porphyrins **C31.5a**–**c** or **C31.5d**–**e** stabilized by either hydrogen bonding between the enaminoketone fragments or by coordination of nickel(II).^624^

#### 7.2.7 Indole- and Carbazole-Based Porphyrinoids

**Scheme 333:**
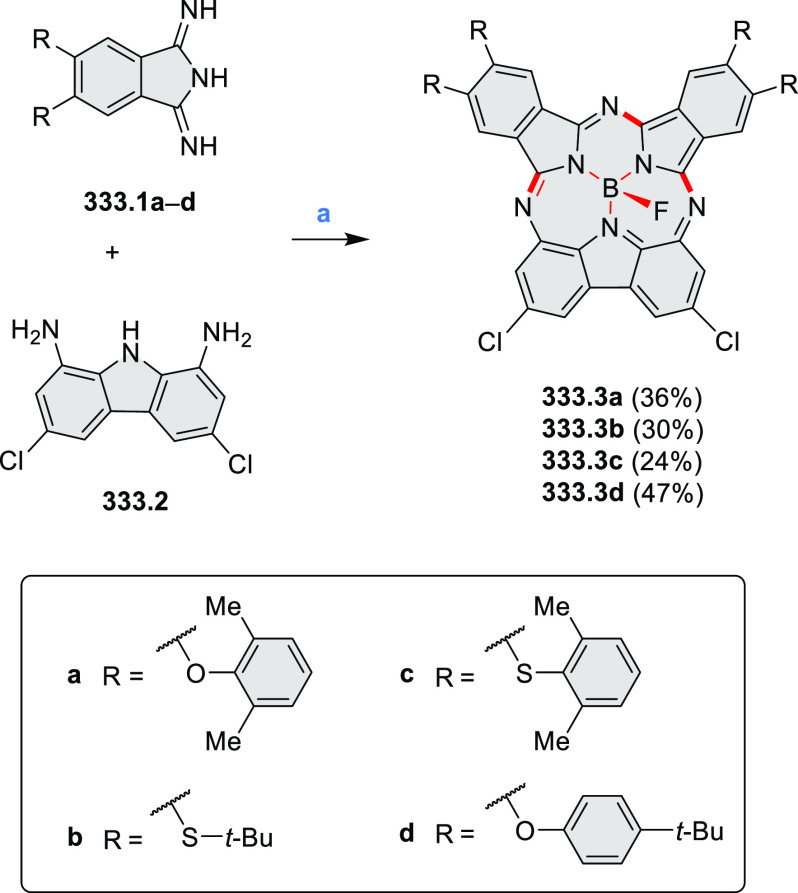
Synthesis of Boron(III) Carbazosubphthalocyanines Reagents and conditions: (a)^625^ (1) EtOH, reflux, (2) BF_3_·Et_2_O, DBU, 1-chloronaphthalene, 150 °C.

In 2019, Kobayashi and Ng reported a series of air-stable boron(III) carbazosubphthalocyanines, which can be seen as core-expanded subphthalocyanine (SubPc) analogues.^625^ The synthesis of **333.3a**–**d** was achieved by the condensation of 1,3-diiminoisoindolines **333.1a**–**d** and diamine **333.2**, followed by boron-induced complexation and cyclization (Scheme 333). The choice of BF_3_·Et_2_O instead of other Lewis acidic boron sources such as BCl_3_ and BBr_3_ was needed to obtain the desired product, stabilized by the axial fluoride ligand. These analogues represented the first examples of antiaromatic SubPc derivatives and the smallest antiaromatic azaporphyrinoids.

**Scheme 334:**
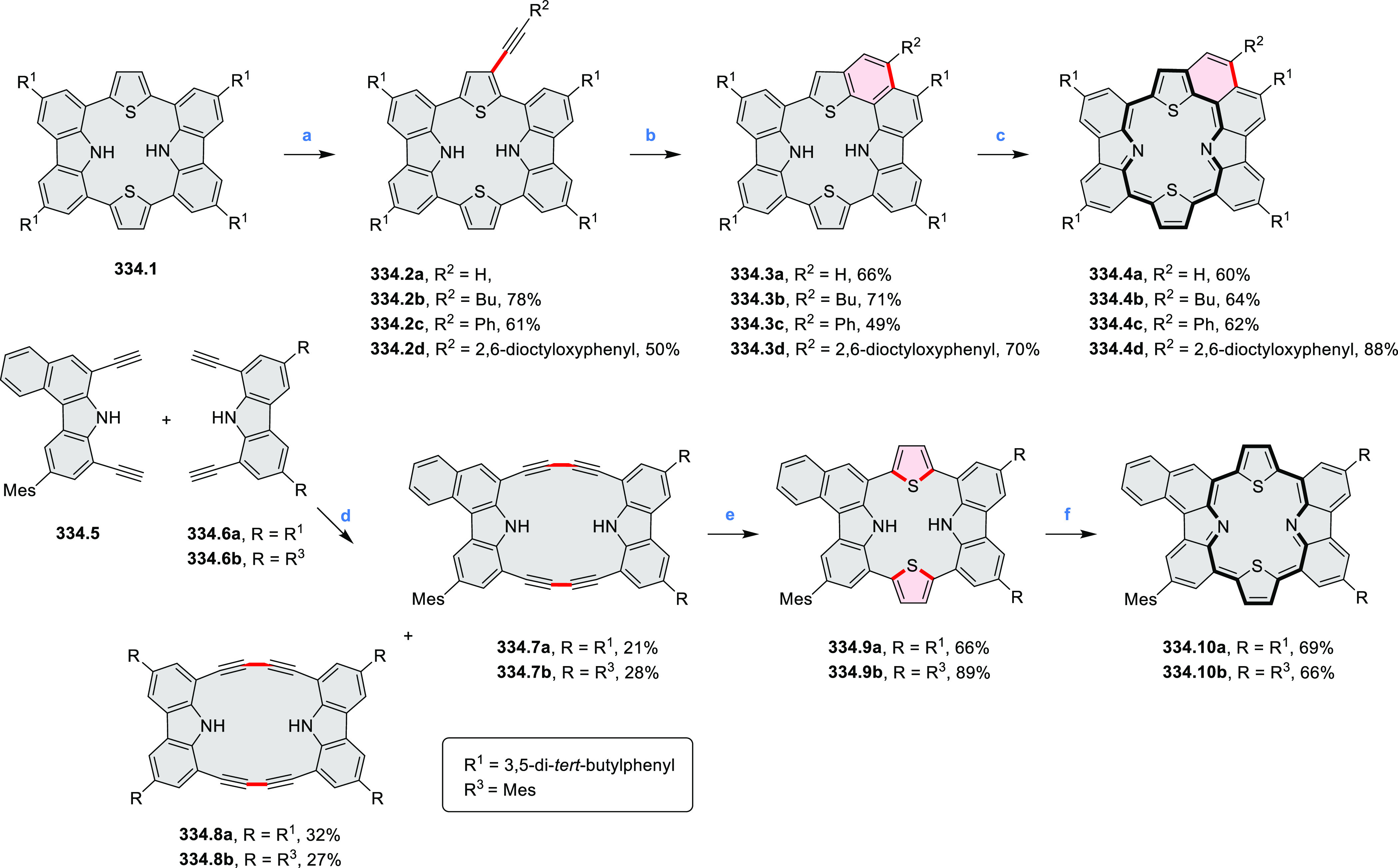
Synthesis of π-Extended Carbazole-Based Porphyrins Reagents and conditions: (a)^626^ (1) NBS, CHCl_3_, 0 °C, (2) Bu_3_SnC≡CR^2^, Pd(PPh_3_)_4_, toluene, reflux; (b) PtCl_2_, toluene, reflux; (c) MnO_2_, DCM, rt; (d) Cu(OAc)_2_·2H_2_O, pyridine, toluene, air, rt; (e) Na_2_S·9H_2_O, toluene, 2-methoxyethanol, reflux; (f) PbO_2_, DCM, rt.

In 2019, Maeda Ema et al. reported π-extended carbazole-based porphyrins containing peripherally fused benzene rings (Scheme 334).^626^ Ethynyl-substituted isophlorins **334.2a**–**d** were prepared via bromination of **334.1** with NBS followed by Stille coupling. In the next step, Pt-catalyzed intramolecular cyclization of the ethynyl substituent in **334.2a**–**d** afforded the benzo-fused isophlorins **334.3a**–**d**, which were subsequently oxidized to the porphyrin-like **334.4a**–**d**. *ortho*-Fused analogues **334.10** were synthesized via Glaser coupling of diethynylbenzocarbazole **334.5** and diethynylcarbazole **334.6**, which yielded both the unsymmetrical **334.7** and symmetrical carbazole dimer **334.8**. In the next step, the annulation reaction of **334.7a** with Na_2_S·9H_2_O provided **334.9a**, which upon further oxidation afforded **334.10a**. **334.10b** was also synthesized in the same manner. The oxidation of compounds **334.3** and **334.9** showed drastic changes in their electronic properties. The UV–vis–NIR absorption displayed intense broad NIR absorption bands at 963 and 1180 nm for **334.3a** and at 910 and 1100 nm **334.9a**. Their absorption onset reached over 1400 nm. Compounds **334.3b**–**d** displayed similar spectra while **334.9b** showed slightly red-shifted NIR bands revealing the additional fusion moieties clearly perturbed the π-electron systems of **334.3** and **334.9**.

**Scheme 335:**
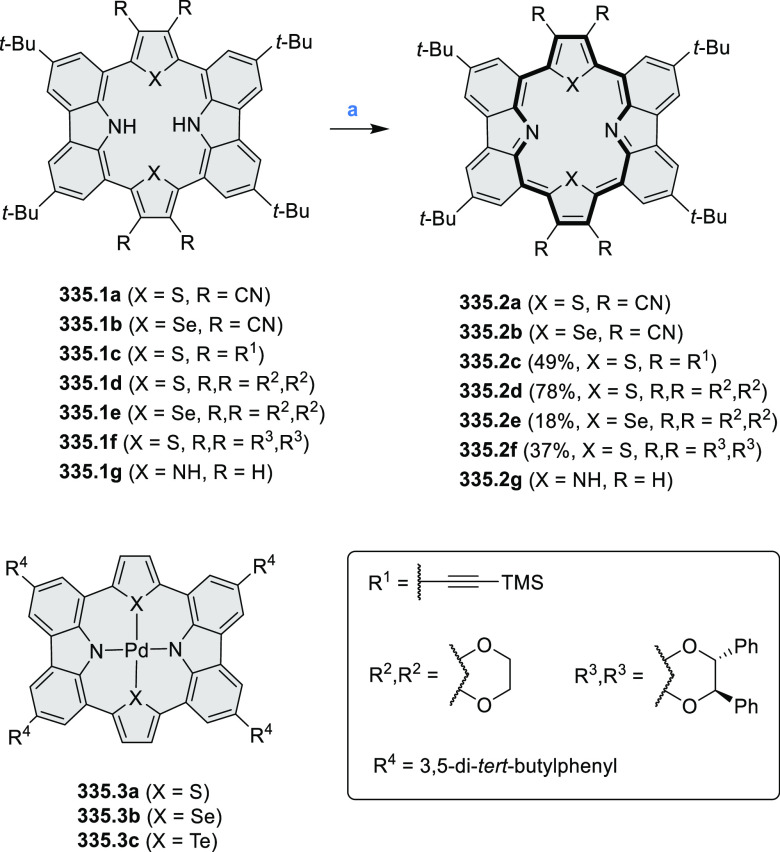
Carbazole-Based Porphyrins Reagents and conditions: (a)^627,628^ MnO_2_, DCM, rt.

Substituent effects of cyano and ethynyl groups on the photophysical properties of carbazole-based porphyrins **335.1a**–**c** were investigated by Maeda, Yoshioka et al. (Scheme 335).^627^ Additionally, thia- and selenaporphyrins **335.1d**–**e** bearing electron-donating ethylenedioxy groups were also prepared for the comparison. The introduction of the ethynyl groups into the conjugated macrocycle induced the red-shifts while that of cyano groups induced blue shifts to the lowest-energy bands. Selenaporphyrin **335.2e** displayed stronger and red-shifted absorption in the NIR region, with the lowest-energy band at 1178 nm. A chiral analogue **335.2f** displayed a CD spectrum with Cotton effects in the NIR region.^628^ Palladium complexes **335.3a**–**c** of the carbazole-based macrocycles displayed a weak antiaromatic character derived from the 20π isophlorin framework.^629^ These metal complexes exhibited weak near-infrared (NIR) absorption at 700–1000 nm.

**Scheme 336:**
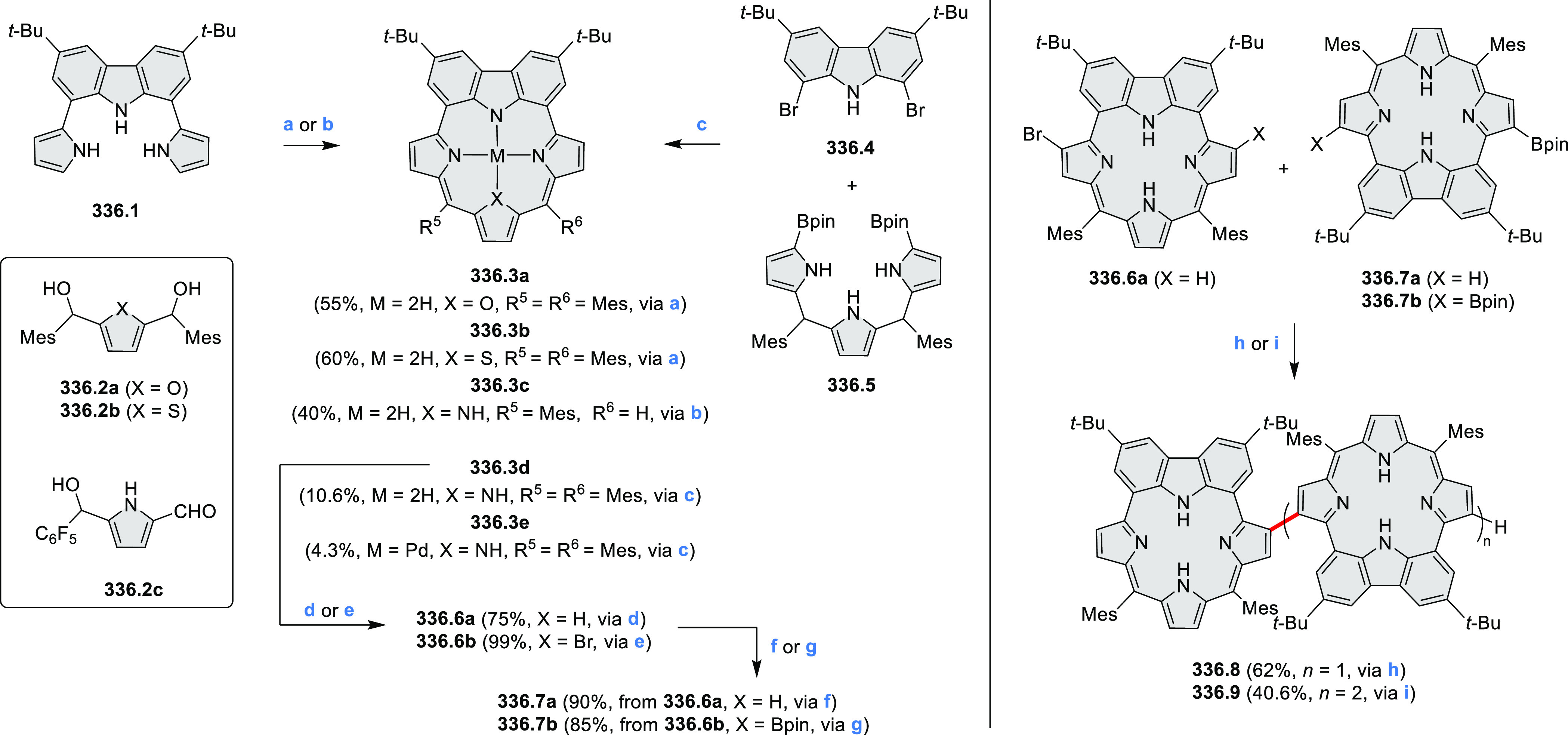
Synthesis of Asymmetric Carbazole-Based Porphyrins and Their Oligomers Reagents and conditions: (a)^631^ (1) **336.2a** or **336.2b**, *p*-TSA (0.2 equiv), DCM, rt, dark, 1 h, (2) DDQ (1 equiv), 10 min; (b)^631^ (1) **336.2c**, CF_3_COOH (1 equiv), DCM, rt, dark, 90 min, (2) DDQ (2 equiv), 10 min; (c)^630^ (1) Pd_2_(dba)_3_, SPhos, CsF, Cs_2_CO_3_, reflux, 48 h, (2) MnO_2_; (d)^630^ NBS (0.9 equiv), CHCl_3_; (e)^630^ NBS (3.7 equiv), CHCl_3_; (f)^630^ (Bpin)_2_ (2.9 equiv), Pd(dppf)Cl_2_, KOAc, 1,4-dioxane, reflux; (g)^630^ (Bpin)_2_ (9.8 equiv), Pd(dppf)Cl_2_, KOAc, 1,4-dioxane, reflux; (h)^630^**336.6a**/**336.7a** (1:1), Pd_2_(dba)_3_, PPh_3_, CsF, Cs_2_CO_3_, toluene/DMF, reflux, 48 h; (i)^630^**336.6a**/**336.7b** (1:0.5), Pd_2_(dba)_3_, PPh_3_, CsF, Cs_2_CO_3_, toluene/DMF, reflux, 48 h.

Related porphyrinoids containing a single carbazole subunit were reported in 2019 by Kim, Song et al. (Scheme 336).^630^ The palladium-catalyzed Suzuki–Miyaura cross-coupling reaction between the dibromocarbazole **336.4** and diboryltripyrrane **336.5** followed by MnO_2_ oxidation afforded the macrocycle **336.3d** along with its palladium(II) complex **336.3e** (Scheme 336). By extension of this strategy, the directly linked oligomers **336.8** and **336.9** were also synthesized from **336.6a** and **336.7a**–**b**. Later in 2020, Gokulnath and co-workers reported an alternative, higher-yielding route to **336.3a**–**c**, based on the [3 + 1] acid-catalyzed condensation,.^631^ The macrocycles **336.3a** and **336.3b** exhibited red emission with two characteristic bands from 600 to 850 nm with the fluorescence quantum yield (Φ_F_) of 7.6% and 12.8%, respectively. The furan-containing macrocycle **336.3a** showed a sensing capability toward mercury(II) ions.

**Scheme 337:**
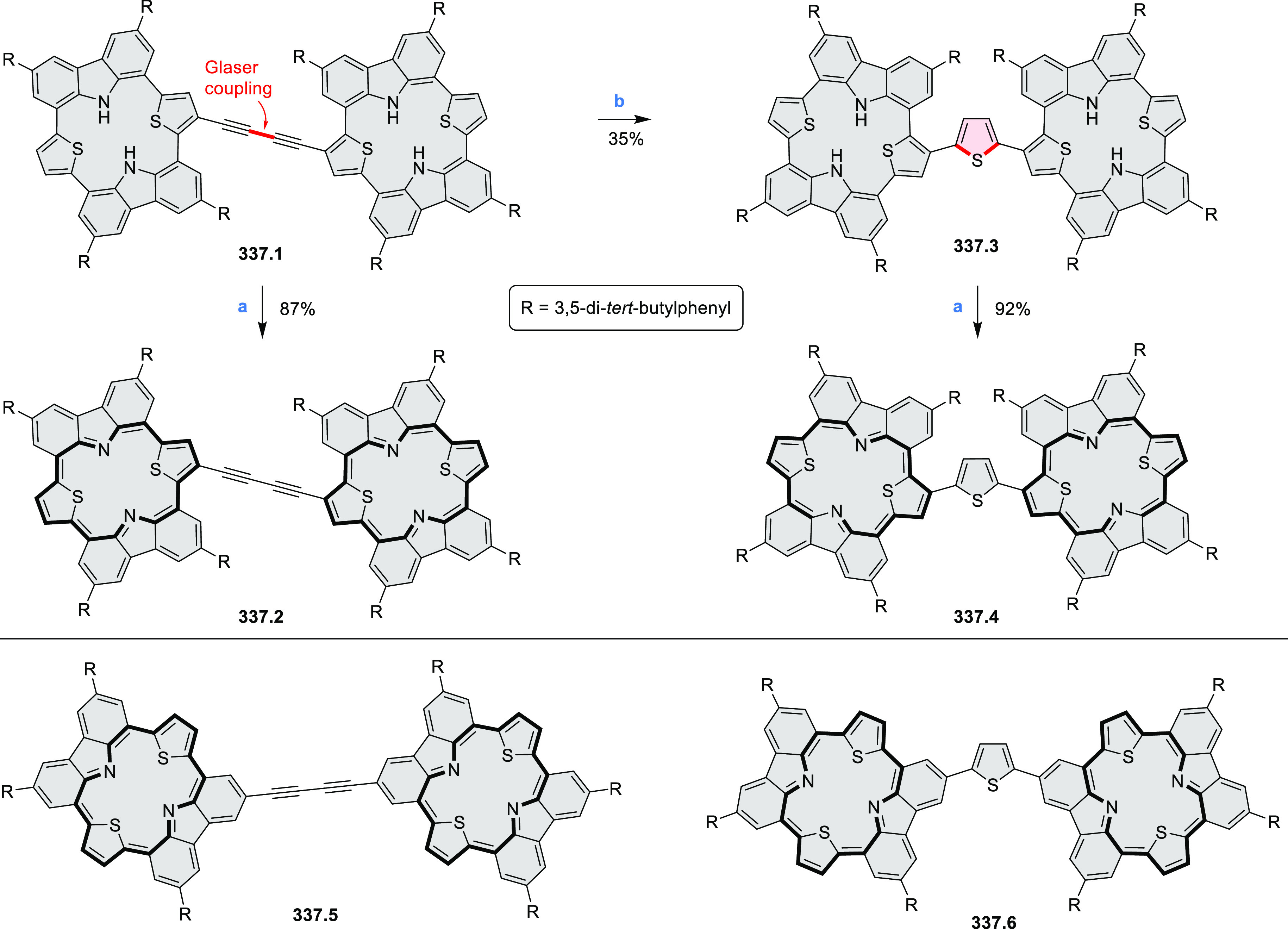
Thiophene-Bridged Carbazole-Based Diporphyrins Reagents and conditions: (a)^632^ PbO_2_, DCM; (b) Na_2_S·9H_2_O, *p*-xylene, 2-methoxyethanol, reflux.

The synthetic route to thiophene-bridged carbazole-based porphyrin dimers, reported by Maeda, Ema, and co-workers, started with the Glaser coupling reaction of ethynyl-substituted carbazole-based isophlorins **334.2a** to afford the butadiyne-bridged dimers **337.1** (Scheme 337).^632^ In the next step, the annulation reaction on **337.1** produced the thiophene-bridged dimers **337.3** in 35% yield. Finally, the oxidation of **337.1** and **337.3** with PbO_2_ afforded the carbazole-based diporphyrins **337.2** and **337.4**, respectively. Diporphyrins **337.5** and **337.6** linked at 3,3′-positions of carbazole moiety were similarly prepared. Absorption spectra indicated that the diporphyrins linked at carbazole moiety **337.5** and **337.6** had stronger intramolecular electronic interactions within the diporphyrins than those of **337.2** and **337.4**. Electrochemical HOMO–LUMO gaps of **337.5** and **337.6** were found to be 0.761, and 0.739 eV, respectively.

**Scheme 338:**
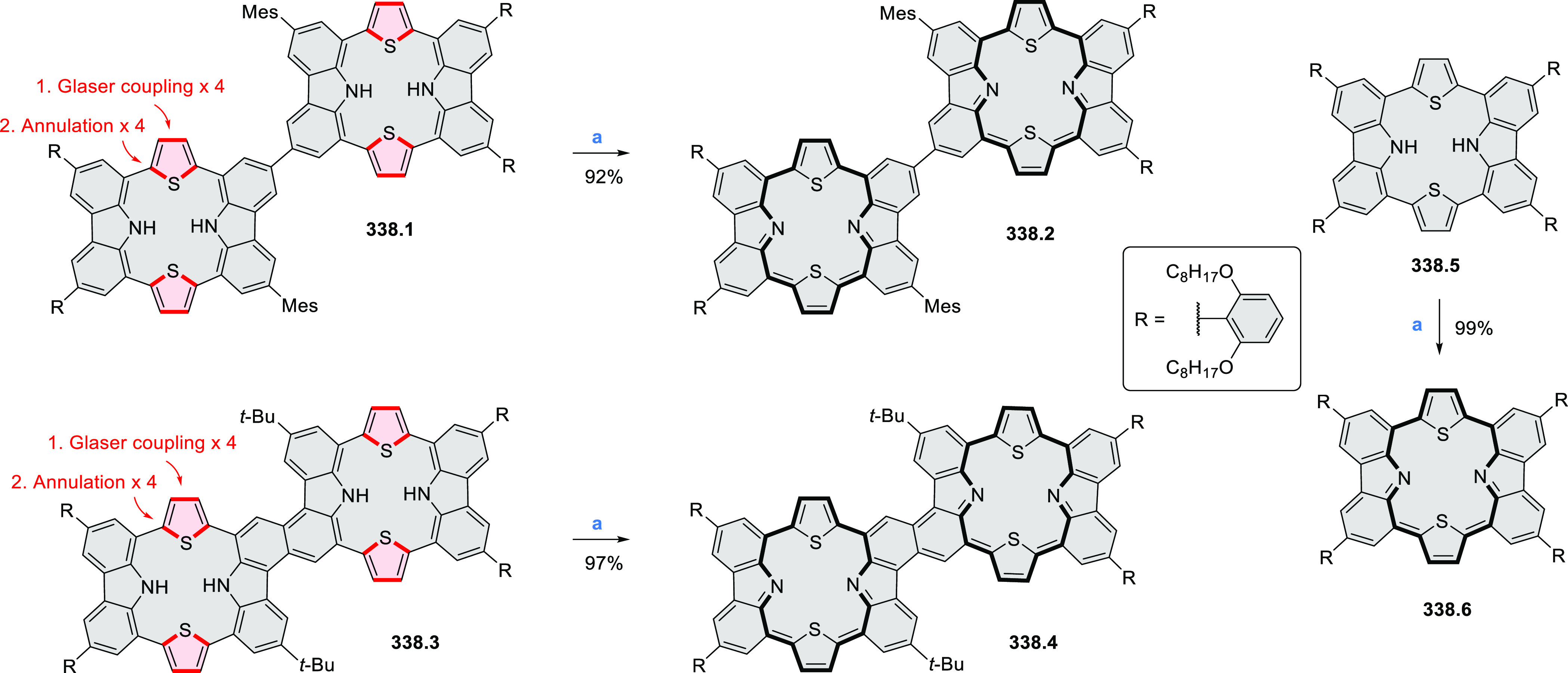
Directly Linked and Fused Carbazole-Based Diporphyrins Reagents and conditions: (a)^633^ PbO_2_, DCM.

Directly linked carbazole-based diporphyrin **338.2** and fused diporphyrin **338.4** were obtained using a similar approach, consisting of (i) Glaser coupling, (ii) annulation, and (iii) oxidation of the isophlorin porphyrin (Scheme 338).^633^ In these macrocycles, the 2,6-dioctyloxyphenyl groups were used to improve the solubility. The UV–vis–NIR absorption spectrum of **338.4** in DCM showed NIR bands at 948, 1085, and 1269 nm, bathochromically shifted in comparison with **338.2** (887, 932, 969, and 1077 nm). This red-shift was ascribed to the effective π-delocalization over the directly fused linkage between the porphyrin-like subunits in **338.4**. Cyclic voltammograms of **338.4** showed one oxidation wave at −0.012 V and two reductions at −0.535 and −0.837 V, corresponding to a narrow electrochemical gap of 0.523 eV.

**Scheme 339:**
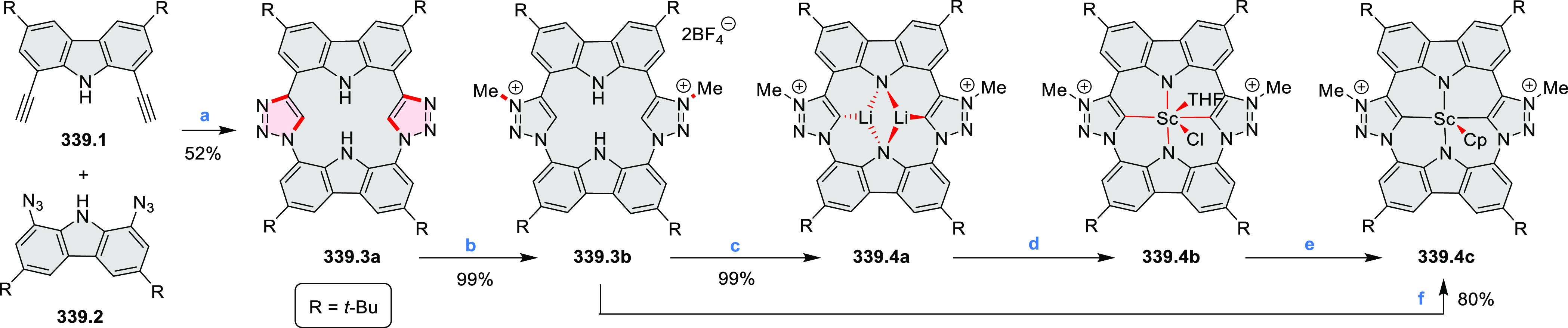
Synthesis of Carbenaporphyrins Reagents and conditions: (a)^634^ CuSO_4_·5H_2_O, tris((1-benzyl-4-triazolyl)methyl)amine, sodium ascorbate, Et_3_N, THF, Ar; (b) 2Me_3_OBF_4_, DCM; (c) LiHMDS (6.6 equiv), THF-*d*_8_; (d) ScCl_3_(THF)_3_, THF-*d*_8_; (e) LiCp, THF-*d*_8_; (f) LiHMDS, THF, LiCp.

Maulbetsch and Kunz reported a synthesis of “carbenaporphyrins” via 1,3-dipolar cycloaddition (Scheme 339).^634^ The azide precursor **339.2** was obtained from a Sandmeyer-type reaction and subjected to copper-catalyzed 1,3-dipolar cycloaddition with **339.1**, to produce the macrocycle **339.3a**. The latter system had a nonplanar geometry in the solid state, with the carbazole and triazole planes tilted in opposite directions to avoid steric congestion of C–H and N–H bonds. **339.3a** was alkylated by Meerwein’s salt to yield **339.3b** in nearly quantitative yield. Deprotonation of **339.3b** with LiHMDS led to the lithium complex **339.4a**. Then, transmetalation with ScCl_3_ resulted in the desired scandium complex **339.4b**, which exhibited orange fluorescence. The latter species could be further converted into the Cp derivative **339.4c** via ligand exchange.

### 7.3 Naphtho[2,1,8,7-*cdef*]-fused porphyrinoids

#### 7.3.1 Monoporphyrinoids

**Scheme 340:**
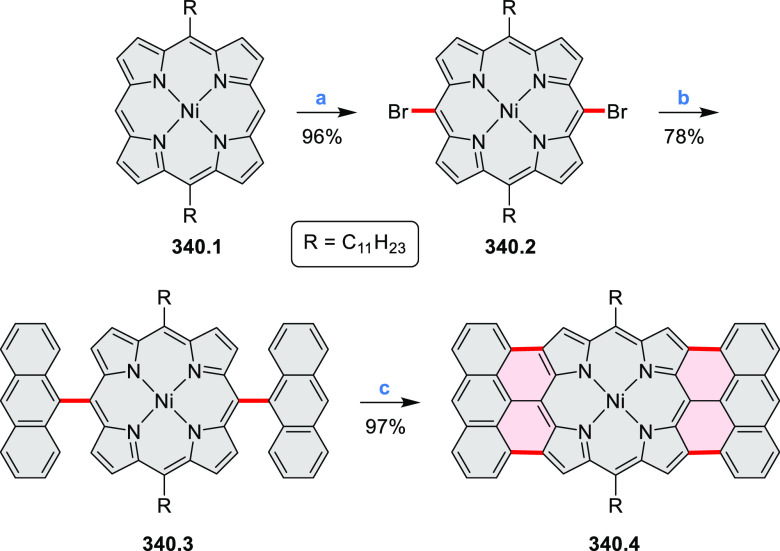
Synthesis of Bisanthracene-Fused Porphyrins Reagents and conditions: (a)^635^ NBS, DCM/MeOH (10:1, v/v); (b) 9-anthraceneboronic acid, Pd(PPh_3_)_4_, K_2_CO_3_, toluene/DMF (5:1, v/v), 120 °C; (c) TfOH, DDQ, DCM.

In 2020, Mai reported a doubly anthracene-fused porphyrin **340.4** as a photocatalyst visible light-induced aerobic oxidation of amines to imines under ambient conditions (Scheme 340).^635^ In the final step of their synthesis, cyclodehydrogenation of the anthryl-substituted porphyrin **340.3** using DDQ and a catalytic amount of triflic acid produced **340.4** in a high yield of 97%. These cyclodehydrogenation conditions were more efficient than employing FeCl_3_ and catalytic AgOTf/DDQ. UV–vis–NIR absorption spectrum of **340.4** in THF displayed a large red-shift in the lowest-energy wavelength band compared to **340.3**, with Soret-band absorption at 565 nm and Q-bands in the NIR region (850 to 1150 nm).

**Scheme 341:**
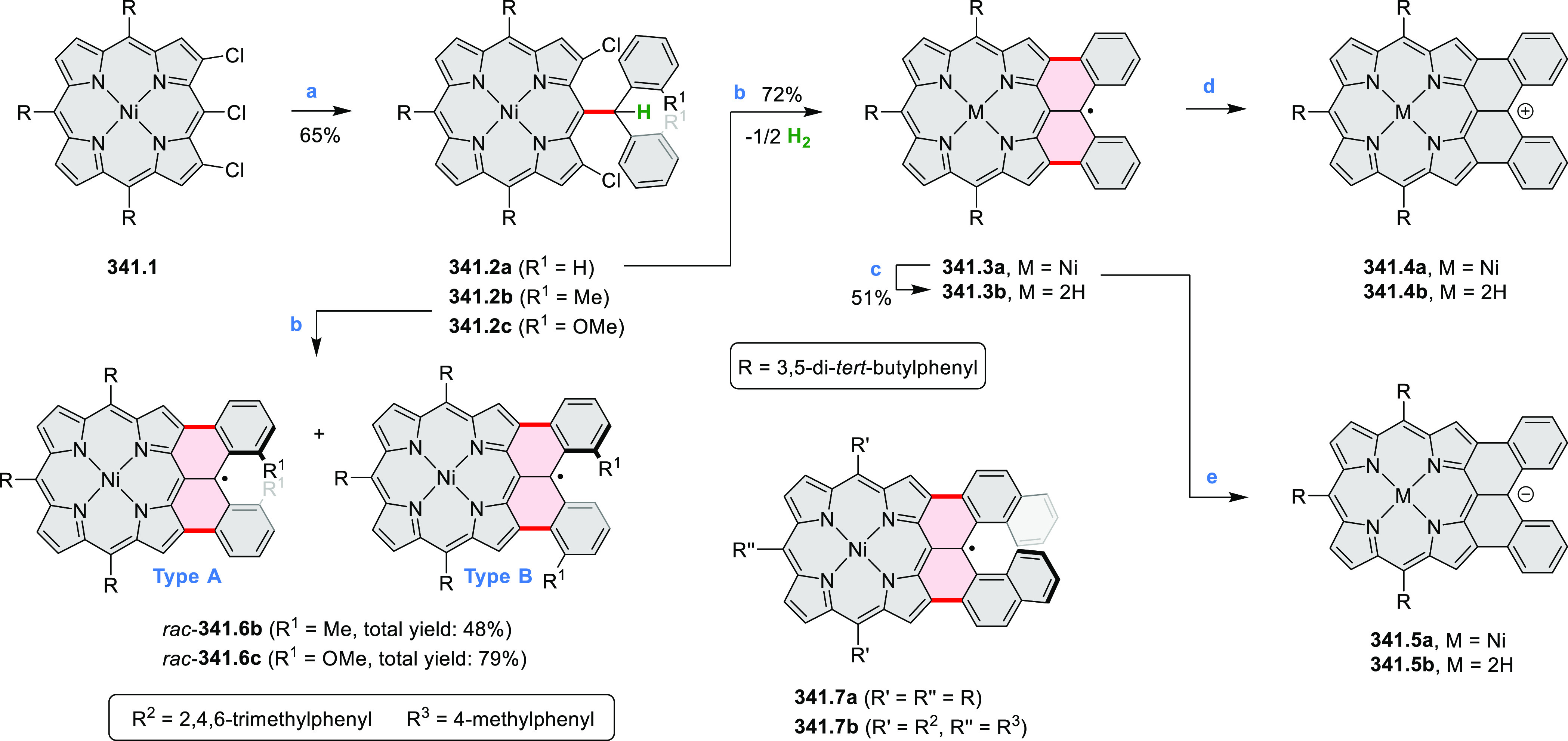
Synthesis of Diphenylmethane-Fused Porphyrins Reagents and conditions: (a)^636,637^ (2-R^1^-C_6_H_4_)_2_CHLi (2.0 equiv), THF, −98 °C to rt, 2 h; (b)^636,637^ Pd(OAc)_2_, PCy_3_·HBF_4_, K_2_CO_3_ (5.0 equiv), toluene, reflux, overnight; (c)^636^ H_2_SO_4_/CF_3_CO_2_H, 0 °C to rt, 45 min; (d)^636^ BAHA, CDCl_3_, rt; (e)^636^ cobaltocene, [D_8_]THF, rt.

Diphenylmethane-fused porphyrin **341.3a**, reported in 2016 by Osuka et al., was obtained in a two-step reaction sequence beginning with a S_N_Ar reaction of **341.1** with diphenylmethyllithium which produced **341.2** in 65% yield (Scheme 341).^636^ Then, a 2-fold palladium-catalyzed intramolecular C–H arylation of **341.2** afforded **341.3a** with a doubly fused structure with a loss of one hydrogen atom at the diphenylmethane segment. Based on spectroscopic evidence, compound **341.3a** was identified as the neutral porphyrin radical, which was found to be very stable under ambient conditions. Demetalation of **341.3a** resulted in the freebase radical **341.3b**, which was likewise air- and moisture-stable. The UV–vis–NIR absorption spectra of these compounds in DCM displayed a relatively weak Soret-like band at 481 nm and Q-like bands at 582 and 620 nm for **341.3a**, and a broadened Soret-like band at 475 nm with Q-like bands at 564 and 633 nm for **341.3b**, along with several weak and broad bands reaching up to 1500 nm. The observed low-energy broad absorption bands in the UV–vis–NIR were indicative of the presence of radical character in these porphyrins. Excited-state dynamics of the radical porphyrins were studied by femtosecond transient absorption (TA) measurements, which showed two decay components with lifetimes of 0.5 and 8 ps for **341.3a**, and 0.3 and 9 ps for **341.3b**, suggesting the lowest excited state relaxation to the open-shell ground state. In ^1^H NMR titration experiments, chemical oxidation and reduction of **341.3a** (and **341.3b**) led to the generation of cationic **341.4a**–**b** and anionic species **341.5a**–**b** with perturbed electronic properties.

Later in 2017, Furukawa, Osuka et al. reported helically chiral analogues of these diphenylmethane-fused porphyrins.^637^ The preparation of *rac*-**341.6b**–**c** and *rac*-**341.7a**–**b** from more sterically hindered diarylmethane derivatives, containing either ortho substituents, or additional benzene rings. The formation of *rac*-**341.6b**–**c** was accompanied by partial rearrangement (type B products, Scheme 341). The crystallographic analysis of *rac*-**341.7a** revealed a fully conjugated framework containing a [6]helicene unit. Experimental bond lengths as well as DFT calculations indicated that the radical was stabilized by full delocalization over the whole fused π-system.

**Scheme 342:**
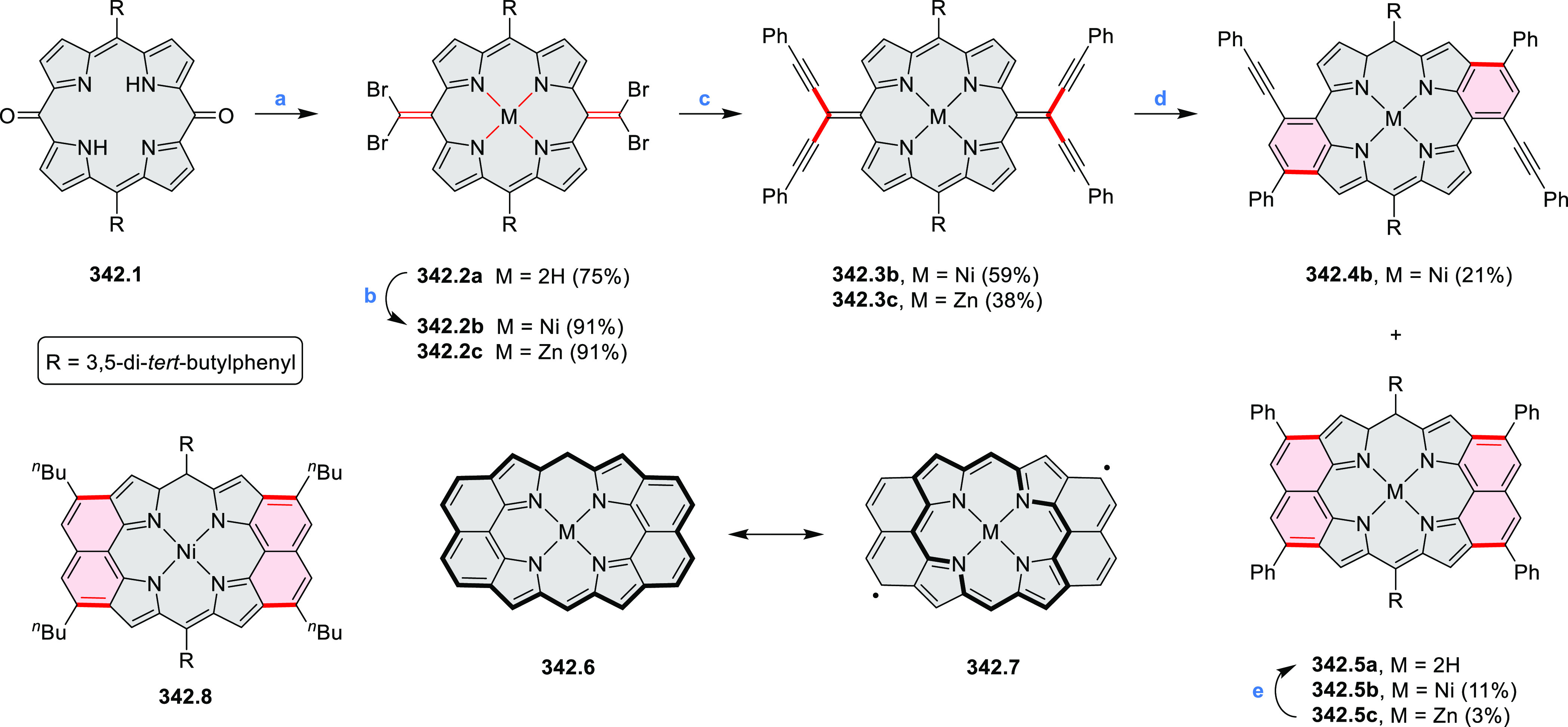
Synthesis of Di-*peri-*dinaphthoporphyrin Porphyrins Reagents and conditions: (a)^638^ CBr_4_, PPh_3_, toluene, 80 °C, 12 h; (b) M = Ni: Ni(acac)_2_, toluene, reflux, 3 h; M = Zn: Zn(OAc)_2_·2H_2_O, DCM, MeOH, rt, 1 h; (c) tributyl(phenylethynyl)tin, Pd_2_(dba)_3_, P(2-furyl)_3_, toluene, 80 °C, 1 h; (d) PtCl_2_ or Pt(MeCN)_2_Cl_2_, toluene, 90 °C, 12 h; (e) HCl, DCM, quantitative yield.

In 2016, Tanaka, Kim, Osuka et al. developed a synthesis of di-*peri-*dinaphthoporphyrin (Scheme 342).^638^ Initially, **342.2a** was obtained by the Ramirez olefination of **342.1** with tetrabromomethane in the presence of triphenylphosphine, which was then metalated with a nickel salt to give the **342.2b** complex. Then, **342.2b** was subjected to Stille coupling with 8 equiv of tributyl(phenylethynyl)tin to yield the quinodimethane-type porphyrin **342.3b**. The treatment of **342.3b** with 0.6 equiv of PtCl_2_ afforded a 2-fold cyclized product **342.4b** in 21% yield, whereas when 2 equiv of PtCl_2_ was used, the 4-fold cyclization product **342.5b** was formed in low yield along with many side products. This complex reaction mixture hampered the isolation of **342.5b** in a pure form. Compound **342.5b** could instead be obtained in up to 11% yield using 8 equiv of a different platinum reagent, Pt(MeCN)_2_Cl_2_.

Synthesis of the free base porphyrin **342.5a** was instead achieved starting from the **342.2c** precursor by first converting to **342.3c**, then cycloisomerization with 2 equiv of the platinum reagent and subsequent demetalation of **342.5c**. The UV–vis spectrum of **342.5b** exhibited a sharp Soret-like band at 522 nm and a red-shifted band at 897 nm with a very weak absorption tail reaching to around 1300 nm. Such weak absorption tails were referred to the presence of NIR dark states, supporting the 24 π antiaromatic circuit as the dominant resonance contributor in **342.6**. This conclusion was also supported by the combined spectroscopic investigations from ^1^H NMR, cyclic voltammetry, and DFT calculations. An alkyl-substituted di-*peri-*dinaphthoporphyrin **342.8** was obtained following a similar synthetic pathway.^639^**342.8** exhibited slightly shorter intermolecular packing distance in the solid state (ca. 3.402 Å) and showed blue-shifted absorption relative to its phenyl-substituted analogue.

**Scheme 343:**
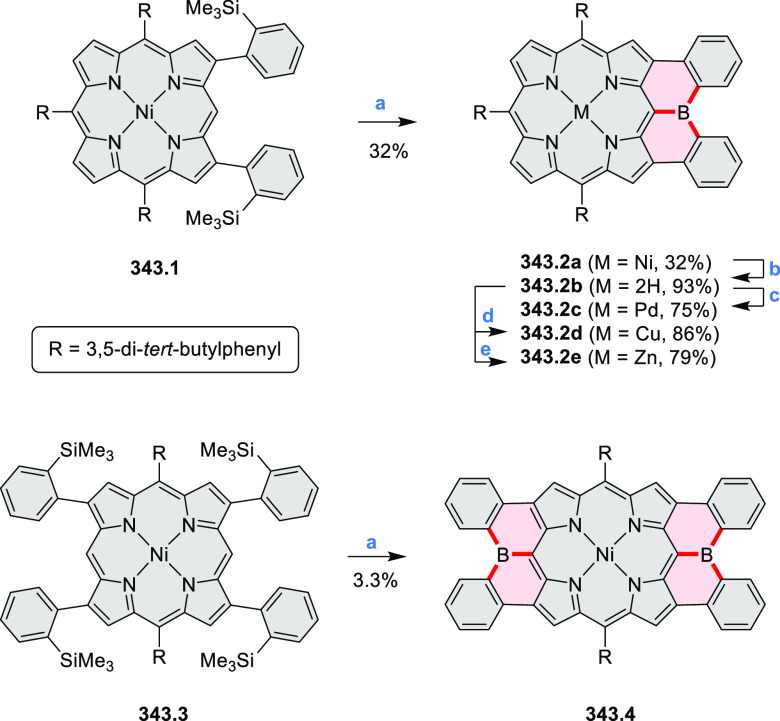
Synthesis of Diphenylborane-Fused Porphyrins Reagents and conditions: (a)^640^ BBr_3_, *o*-dichlorobenzene, 80 °C; (b) TFA/H_2_SO_4_/DCM, 0 °C; (c) PdCl_2_(PhCN)_2_, PhCN, 140 °C; (d) Cu(OAc)_2_, DCM/MeOH, rt; (e) Zn(OAc)_2_·2H_2_O, DCM/MeOH, rt.

Mono- and bis(diphenylborane)-fused porphyrins were obtained through a sequence of (i) Si–B exchange reaction, (ii) intramolecular bora-Friedel–Crafts reaction, and (iii) ring-closing Si–B exchange (Scheme 343).^640^ The mono- (diphenylborane)-fused nickel(II) porphyrin, **343.2a** underwent demetalation under strongly acidic conditions to afford **343.2b**, which was further metalated with Pd, Cu, and Zn salts, yielding **343.2a**–**e**. The bis(diphenylborane)-fused porphyrin **343.4** was synthesized from **343.3** in a similar manner. Absorption spectra of mono and bis(diphenylborane)-fused porphyrins **343.2a**–**e** and **343.4** showed significant red-shifts compared to the reference porphyrin with no boron-fusion. This effect was ascribed to extended electronic interactions between the empty p-orbital of the boron atom of the fused diphenylborane group and the porphyrin π-network. The addition of pyridine at the boron center in the porphyrin disrupted of the electronic interaction of the boron atom as indicated by UV–vis titration experiments. Compound **343.2e** interacted with Lewis bases, displaying regioselective binding of DMAP at the boron atom and 3,5-difluoropyridine at the zinc center.

**Scheme 344:**
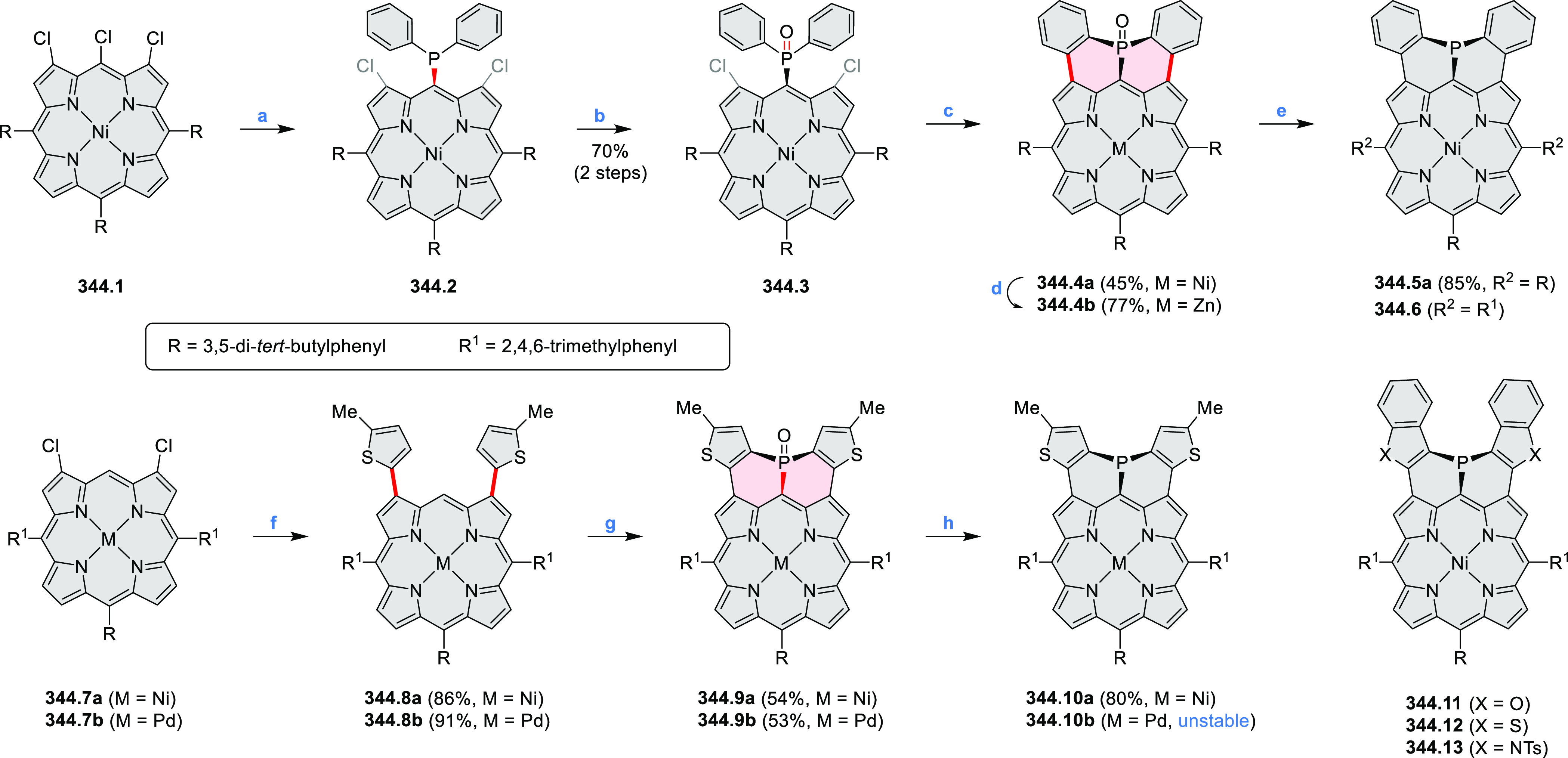
Synthesis of Diphenylphosphine-Fused Porphyrins Reagents and conditions: (a)^642^ LiPPh_2_, THF, rt; (b)^642^ H_2_O_2_, DCM, rt; (c)^642^ Pd(OAc)_2_, PCy_3_·HBF_4_, *t*-BuCO_2_H, K_2_CO_3_, DMA, 120 °C; (d)^642^ conc. H_2_SO_4_, TFA, 0 °C, and then, Zn(OAc)_2_·2H_2_O, DCM/MeOH, rt; (e)^642,643^ HSiCl_3_, toluene, reflux; (f)^643^ 5-methyl-2-thienylzinc chloride lithium chloride, Pd_2_(dba)_3_, RuPhos, THF, reflux; (g)^643^ PBr_3_, ZnI_2_, *o*-dichlorobenzene, 180 °C; then, H_2_O_2_; (h)^643^ HSiCl_3_, toluene, rt.

A triphenylsilane-fused Ni(II) porphyrin in which the *meso*-linked silicon atom was embedded in a triply linked skeleton was subsequently reported by the same group.^641^ The synthesis of phosphorus analogues, summarized in Scheme 344, involved a sequence of nucleophilic aromatic substitution, oxidation, and palladium-catalyzed intramolecular cyclization.^642^ In the latter step, double cyclization via intramolecular C–H arylation of **344.3** using palladium–pivalic acid catalytic system gave the doubly cyclized product **344.4a** in 45% yield. The success of this cyclization could be attributed to the electron-deficient nature of the phenyl groups at the diphenylphosphine oxide moiety. **344.5a** was obtained by the reduction of **344.4a** with HSiCl_3_ in 85% yield. The zinc(II) complex of the diphenylphosphine-fused porphyrin **344.5a** could not be obtained because of its unstable nature. In the solid state, **344.4a** and **344.5a** showed planar structures. **344.5a** formed antiparallel face-to-face dimer stabilized by P–Ni bonds, while **344.4b** formed an analogous dimer stabilized by O–Zn bonds. The embedded P = O and P centers acted as an electron-accepting and an electron-donating unit, respectively, which perturbed the optical and electronic properties of nickel(II) porphyrins. According to an NMR spectroscopic investigation in CDCl_3_, the dimerization was entropy-driven, with a large entropy gain of Δ*S*_D_ = 207 J K^–1^ mol^–1^. Later in 2017, the same group synthesized a mesityl-substituted derivative **344.6** and heterocycle-containing analogues **344.7**–**13** with a goal of stabilizing a planar phosphorus(III) center embedded in a fused aromatic skeleton.^643^

**Scheme 345:**
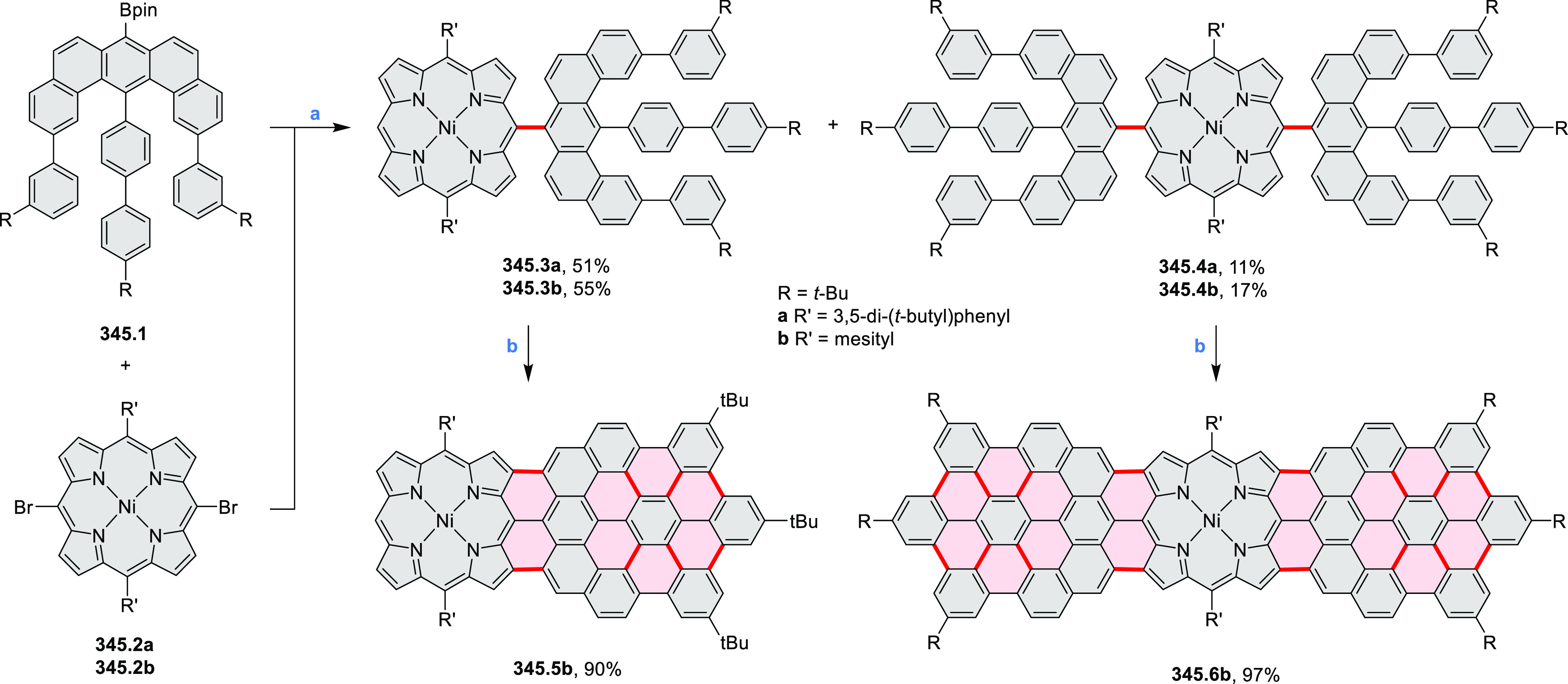
Synthesis of Triply Fused Porphyrin–Nanographene Conjugates Reagents and conditions: (a)^644^ (1) Pd(PPh_3_)_4_, K_2_CO_3_, toluene/DMF (1:1, v/v), 110 °C, 36 h, (2) Pd(PPh_3_)_4_, triethylamine, formic acid, toluene, 100 °C, 2 h; (b) DDQ, DCM/triflic acid (100:1, v/v), rt, 10 h.

In 2018, Müllen, Narita et al. reported the synthesis of the β-, *meso-*, β-triply fused porphyrin-nanographene conjugates **345.5b** and **345.6b** by fusing porphyrin with π-extended HBCs having two extra K-regions.^644^ These π-extended electronic networks were expected to facilitate the electron delocalization over the porphyrin and HBC units, thus narrowing the HOMO–LUMO gap and producing large red-shifts in the UV–vis–NIR absorption spectra. Boronate **345.1** was subjected to a Suzuki coupling reaction with **345.2**–**b**, which afforded the corresponding one- and 2-fold coupling products **345.3a**–**b** and **345.4a**–**b** (Scheme 345). The mesityl-substituted **345.3b** and **345.4b** underwent oxidative cyclodehydrogenation with DDQ/TfOH to provide the triply fused porphyrin-nanographene conjugates **345.5b** (90%) and **345.6b** (97%), respectively. The attempts to obtain the corresponding free bases using trifluoroacetic acid and sulfuric acid resulted in decomposition. The UV–vis–NIR absorption of **345.5b** in THF showed the maximum absorption peak at 866 nm with the tail extended up to around 1000 nm, while **345.6b** showed largely red-shifted absorption with the tail extending to approximately 1400 nm upon fusion of another nanographene unit to the porphyrin core. The planar structure of **345.6b** was validated by STM measurements which also revealed a self-assembled network at the interface of 1,2,4-trichlorobenzene (TCB) and highly oriented pyrolytic graphite (HOPG).

#### 7.3.2 Porphyrin Tapes

**Scheme 346:**
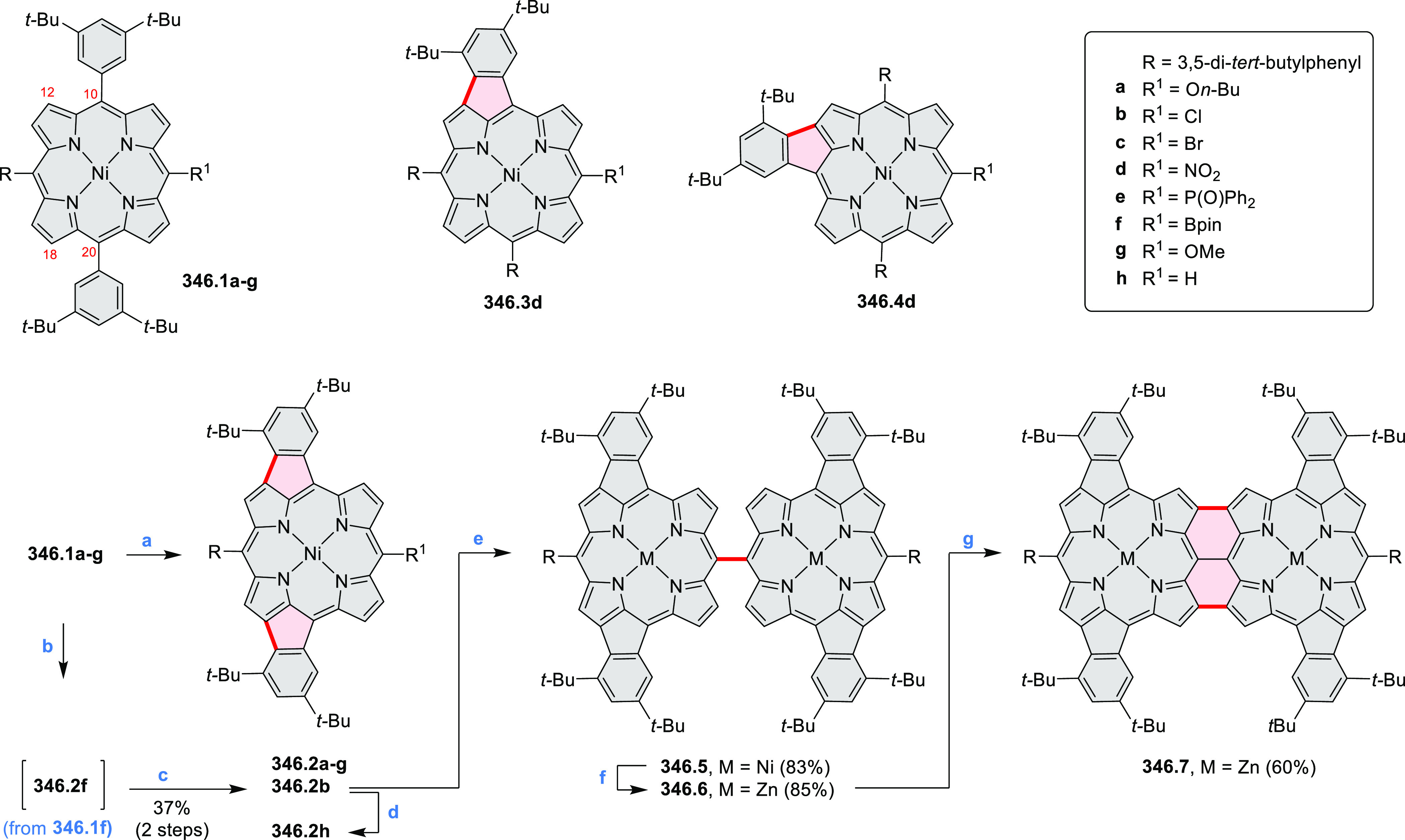
Synthesis of Doubly and Quadruply Phenylene-Fused Porphyrins Reagents and conditions: (a)^645^ DDQ, FeCl_3_, DCM/MeNO_2_, rt; (b) DDQ, FeCl_3_, DCM/MeNO_2_, rt, 4 h; (c) NCS, CuCl, DMF/toluene, 80 °C, 1 h; (d) NEt_3_/HCOOH, Pd_2_(dba)_3_, SPhos, toluene, 120 °C, 13 h; (e) Ni(cod)_2_, DMF, 100 °C, 9 h; (f) (1) TFA/conc. H_2_SO_4_, 0 °C, 30 min, (2) Zn(OAc)_2_·2H_2_O, DCM, MeOH, rt, 1 h; (g) DDQ, Sc(OTf)_3_, toluene, 70 °C, 1 h.

In 2016, Yorimitsu, Kim, Osuka et al. described a regioselective oxidative fusion of 10,15,20-triaryl nickel(II)-porphyrins bearing electron-withdrawing substituents at the 5-position (Scheme 346).^645^ Upon oxidation with DDQ and FeCl_3_, fusion of the 10- and 20-aryl groups at the 12- and 18-positions was observed, with selectivities dependent on the character of the meso substituents. The *meso*–*meso* linked diporphyrin **346.5** was obtained by Ni(0)-mediated reductive homocoupling of **346.2c**. The zinc complex **346.6** prepared via demetalation of **346.5** was subjected to the oxidative fusion reaction with DDQ and Sc(OTf)_3_ which afforded **346.7** in 60% yield. This quadruply phenylene-fused diporphyrin displayed red-shifted absorption bands in the near-infrared region, smaller optical and electrochemical HOMO–LUMO gap, and a larger TPA cross section value than the diporphyrin analogues lacking phenylene fusion. In 2020, Jux et al. reported similar cyclizations on the symmetrically substituted nickel(II) *meso*-tetra(3,5-di(*tert*-butyl)phenyl)porphyrin.^646^ The singly or doubly fused products, **C30.1** or **C30.2**, could be obtained by varying the amount of the FeCl_3_ oxidant and reaction time (Chart 30).

**Chart 32:**
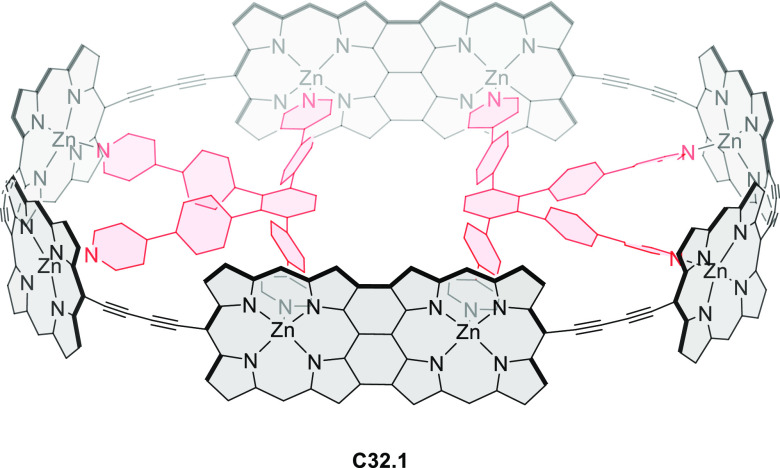
Porphyrin Nanobelt from Triply Linked Porphyrin Tapea

A fully π-conjugated macrocycle containing triply linked porphyrin tape units was reported by Anderson and co-workers.^647^ A K-shaped tetrapyridyl template was employed to synthesize the cyclic porphyrin octamer **C32.1** containing six butadiyne links and two β,meso,β-fused connections (Chart 32). The formation of the nanoring **C32.1** with 2:2 complex of porphyrin tetramer precursor and the template was confirmed by ^1^H diffusion NMR spectroscopy of the complex. The UV–vis–NIR spectra of **C32.1** nanoring showed the lowest absorption energy band at 1304 nm indicating the π-conjugation is extended around the whole macrocycle; with a dominant contribution of S_0_ → S_2_ transitions, as predicted by TD-DFT calculations. NMR-monitored titrations with oxidants revealed that **C32.1** exhibited global antiaromatic and aromatic ring currents, in the [**C32.1**]^4+^ and [**C32.1**]^6+^ oxidation states, respectively.

a *meso*-Aryl rings and double bonds are omitted for clarity (*meso*-aryl = 3,5-bis(trihexylsilyl)phenyl).

**Scheme 347:**
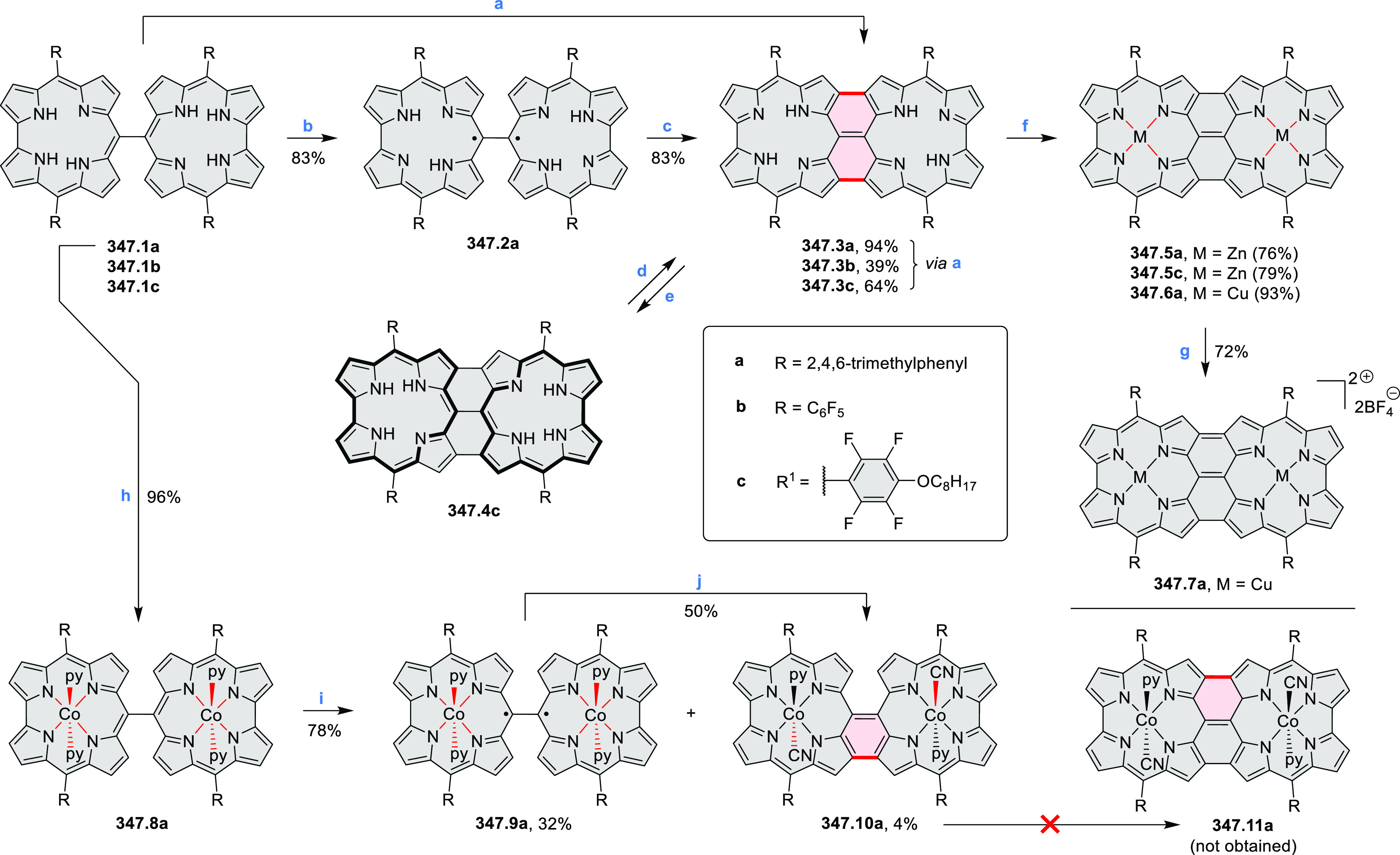
Synthesis of Triply Linked Corrole Dimers Reagents and conditions: (a)^612^ DDQ (3.5 equiv), CHCl_3_ (ca. 0.03 mM), reflux, 30 min; (b)^648^*p*-chloranil (1 equiv), CHCl_3_, reflux, 15 min; (c)^648^ DDQ (2 equiv), CHCl_3_, reflux, 30 min; (d) NaBH_4_, THF/MeOH; (e) air; (f)^612,649^ M = Zn: Zn(OAc)_2_·2H_2_O, CHCl_3_, reflux, 20 h; M = Cu: Cu(acac)_2_ (30 equiv), toluene/MeOH, reflux, 20 h; (g)^649^ AgBF_4_ (2 equiv), DCM/EtNO_2_, rt, 5 min; (h)^648^ Co(OAc)_2_·2H_2_O, pyridine, 100 °C, 15 min; (i)^648^ DDQ (3.5 equiv), CHCl_3_, reflux, 30 min; (j) *p*-chloranil (1 equiv), CHCl_3_, reflux, 3.5 h.

Triply linked corrole dimers were described in 2016 by Tanaka, Kim, Osuka et al.^612^ The synthesis was achieved by intramolecular oxidative fusion reaction of **347.1a**–**c** with DDQ, which gave the target dimers **347.3a**–**c** in 39% yield (Scheme 347). High-dilution conditions were essential to avoid undesirable intermolecular oxidative couplings. The UV–vis–NIR spectrum of the fused dimer **347.3a** in DCM exhibited split Soret-like bands at 520 and 546 nm and a broad band at 653 nm along with a weak absorption tail extending into the NIR region. It was found that the molar absorption coefficient of **347.3a** was distinctly weaker than that of the *meso-*meso linked corrole dimer **347.1a**, presumably because of the nonaromatic nature of the former species. Metalation of **347.3a**,**c** gave the corresponding zinc(II) fused dimers **347.5a**,**c** which were also characterized as nonaromatic complexes. **347.3c** was reduced with NaBH_4_ to give **347.4c**, characterized by corrole-like oxidation level, which however reverted to **347.3c** under aerobic conditions. Spectroscopic investigations involving ^1^H NMR, UV–vis absorption spectroscopy and DFT calculations indicated the recovery of moderate aromaticity in **347.4c** upon reduction.

It was subsequently found that the radical dimer **347.2a** was formed from **347.1a** upon using mild oxidant, *p*-chloranil, in refluxing CHCl_3_ with a short reaction time (Scheme 347).^648^ This diradical dimer could be further oxidized with DDQ, producing **347.3a** in 83% yield. **347.2a** was found be stable under ambient conditions, which was ascribed largely to the strong intramolecular antiferromagnetic interaction of the two spins, with the experimental spin-exchange integral *J*_S–T_ value of −2.19 kcal·mol^–1^. The UV–vis absorption spectrum of **347.2a** in DCM exhibited a Soret-like band at 386 nm and a broad band around 520 nm along with a broad NIR band with a maximum at 981 nm, in agreement with the diradical structure.

The copper complex **347.6a** was subsequently obtained from **347.3a** in a reaction with Cu(acac)_2_ in toluene under reflux conditions (Scheme 347).^649^ Oxidation of **347.6a** with 2 equiv of AgBF_4_ afforded **347.7a**, which was characterized as a dicationic bis-copper(II) species. Oxidation of the meso–meso linked cobalt corrole dimer **347.8a** containing two axially coordinated pyridines with DDQ resulted in the formation of two products **347.9a** and **347.10a**.^648^ The major product **317** was found to be a meso–*meso-*linked cobalt corrole dimer and the minor one **347.10a** was a fused dimer bearing pyridine and cyanide as axial ligands. An attempt to obtain the triply fused cobalt corrole dimer **347.11a** from **347.10a** was not successful. The authors proposed that the cyanide anion could be derived from the decomposed DDQ, which was later supported by a ligand exchange reaction with aqueous NaCN. A SQUID measurement on **347.9a** revealed a strong antiferromagnetic interaction with *J*_S–T_ = −1.76 kcal·mol^–1^, suggesting a similar singlet diradical character as in **347.2a**. The UV–vis–NIR absorption spectrum of **347.9a** in DCM(containing 1% pyridine) was found to be similar to that of **347.2a**, with a broad NIR band at 1115 nm, whereas the fused corrole dimer **347.10a** displayed panchromatic absorption with a weak absorption tail reaching up to 1500 nm.

**Scheme 348:**
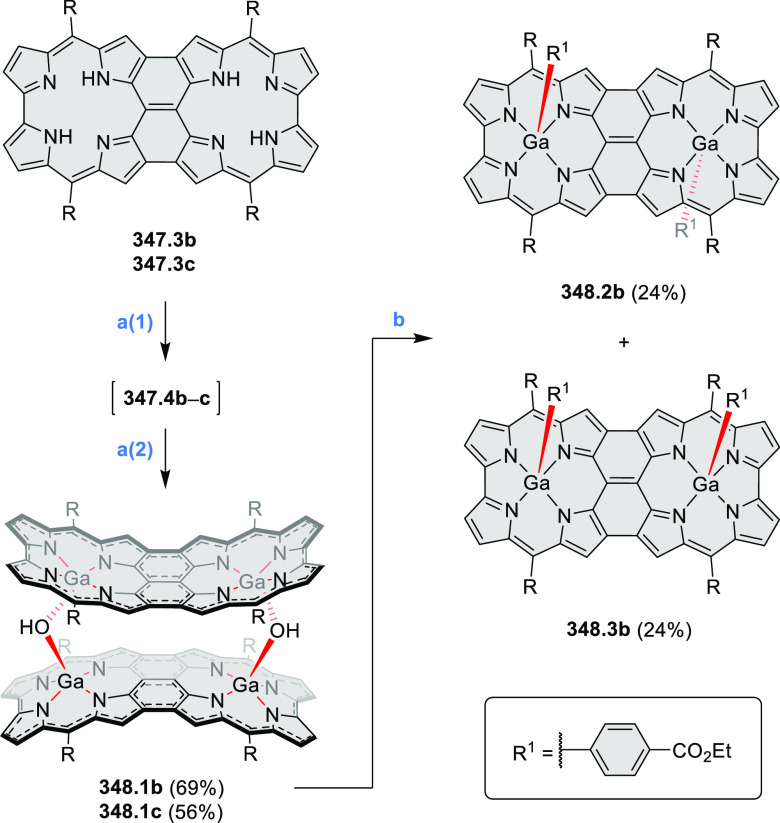
Synthesis of Face-to-Face Dimers and Monomeric Gallium(III) Corrole Tapes Reagents and conditions: (a)^650^ (1) NaBH_4_, THF/MeOH, (2) GaCl_3_ (50 equiv), pyridine, reflux, workup; (b) R^1^ZnI·LiCl (20 equiv), TMSCl (40 equiv), THF, reflux.

Gallium(III) complexes of these triply linked corrole dimers were later found to assemble into unique face-to-face π-dimers (Scheme 348).^650^**348.1b**–**c** were obtained from **347.3b**–**c** in two steps, via initial reduction with NaBH_4_ followed by metalation with GaCl_3_ in pyridine. In the solid state, **348.1b** was found to be a dimeric, with two Ga(III) corrole dimers bridged by apically coordinated hydroxy groups. The ^1^H NMR spectra of these dimers showed very broad peaks, indicating their open-shell character, which was further confirmed by ESR spectroscopy. SQUID measurements indicated the structures **348.1b** and **348.1c** to be singlet diradicals with spin-exchange integral values (*J*_S-T_) of −475 cm^–1^ (−684 K) and −447 cm^–1^ (−643 K), respectively. The two radicals were antiferromagnetically coupled by through-space interaction between the two corrole tapes, which was apparently reflected in the short face-to-face distance (3.24 Å). In line with its diradical character, **348.1b** showed a strong Soret-like band at 611 nm along with weak and broad bands with peaks at 1900 nm and exhibited a narrow electrochemical band gap of 0.37 V. Dimer **348.1b** underwent nucleophilic addition to form the diamagnetic monomers **348.2b** and **348.3b** upon reacting with *para*-ethoxycarbonylphenyl zinc iodide lithium chloride complex.

**Scheme 349:**
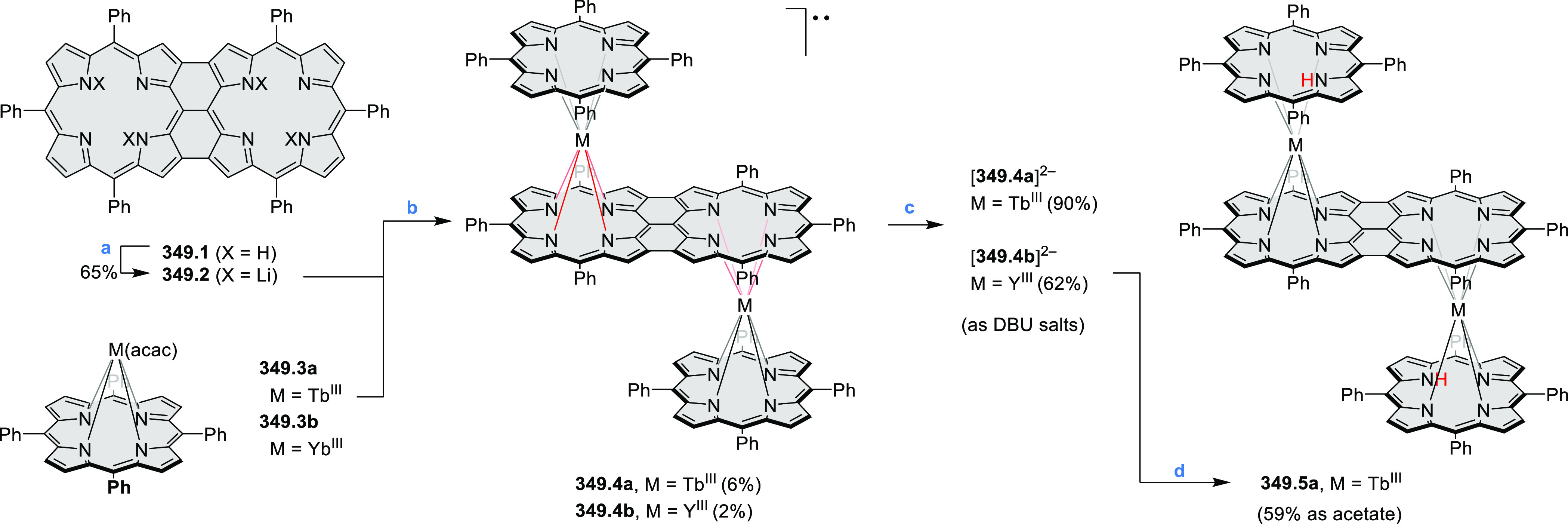
Synthesis of Triple Decker Complexes Containing a Triply Linked Diporphyrin Reagents and conditions: (a)^651^ LiHMDS (6.6 equiv), 1,2,4-trichlorobenzene 120 °C, 1 h; (b) **349.3a** (2 equiv) or **349.3a** (2 equiv), 1,2,4-trichlorobenzene 120 °C, 2 h; (c) DBU (116 equiv), DCM, rt, 1 h; (d) CH_3_COOH (excess), DCM, rt, N_2_, 1 h

In 2019, Ogawa reported the synthesis of dinuclear triple-decker metal complexes with a fused diporphyrin and two tetraphenylporphyrin ligands in their neutral, dianionic, and diprotonated forms (Scheme 349).^651^**349.2**, obtained via lithiation of **349.1**, was reacted with **349.3a** to yield the terbium(III) complex **349.4a** along with a mixture of quadruple-decker and quintuple-decker complexes. The Yb(III) complex **349.4b** was obtained via a similar synthetic protocol. The neutral forms of these metal complexes had large open shell biradical characters, with the experimental spin-exchange integral *J* = −1.4 kcal mol^–1^ determined for **349.3b** using the magnetic susceptibility measurements. These neutral complexes possessed very small HOMO–LUMO gaps (0.33–0.36 eV) and showed multiple redox states.

**Scheme 350:**
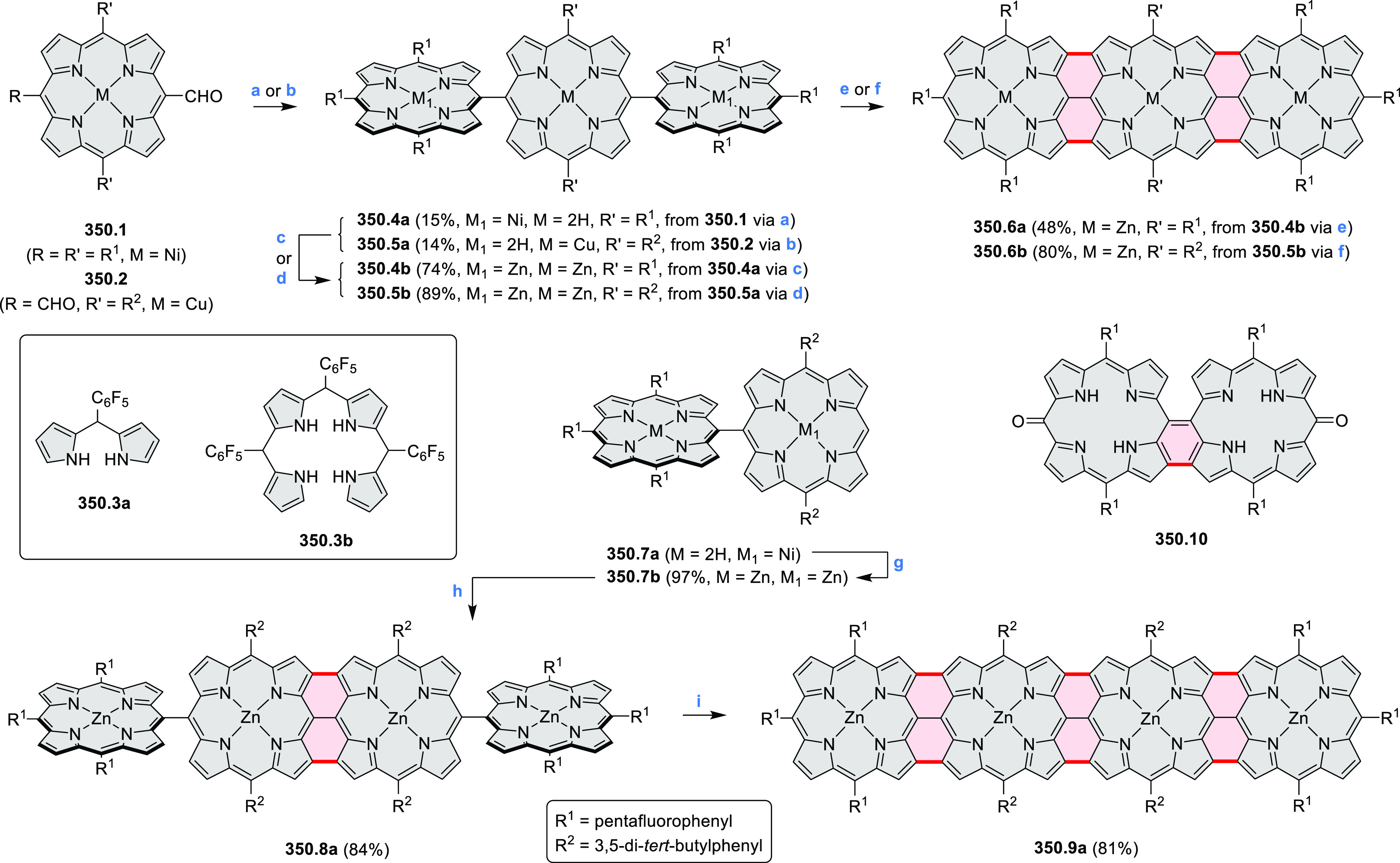
Synthesis of Porphyrin Tapes Reagents and conditions: (a)^652^ (1) **350.3a**, CH_3_SO_3_H (15 mol %), (2) DDQ (3 equiv), DCM, 0 °C, 2 h, then rt 15 min; (b) (1) **350.3b**, *p*-TsOH·H_2_O (1 equiv), (2) DDQ (6 equiv), DCM, 0 °C, 2 h, then rt 15 min; (c) (1) H_2_SO_4_, TFA, rt, 5 days, (2) Zn(OAc)_2_·2H_2_O (60 equiv), CHCl_3_/MeOH, 50 °C, o/n; (d) (1) H_2_SO_4_, TFA, 0 °C, 30 min, (2) Zn(OAc)_2_·2H_2_O (60 equiv), CHCl_3_/MeOH, 50 °C, o/n; (e) DDQ (8 equiv), Sc(OTf)_3_ (8 equiv), toluene, 100 °C, 5 h; (f) DDQ (8 equiv), Sc(OTf)_3_ (8 equiv), toluene, 80 °C, 4 h; (g) H_2_SO_4_, TFA, 0 °C, 30 min, (2) Zn(OAc)_2_·2H_2_O (40 equiv), CHCl_3_/MeOH, 50 °C, o/n; (h) DDQ (5 equiv), Sc(OTf)_3_ (5 equiv), toluene, rt, 3 h; (i) DDQ (12 equiv), Sc(OTf)_3_ (12 equiv), toluene, 80 °C, 3 h.

The preparation of electron-deficient hybrid porphyrin tapes, ADA and AAA comprising *meso*-3,5-di-*tert*-butylphenyl-substituted Zn(II)-porphyrins (D) and *meso*-pentafluorophenyl-substituted Zn(II)-porphyrins (A) was described by Kim, Osuka et al.^652^ The syntheses were achieved via cross-condensation of *meso*-formyl porphyrins **350.1**–**2** with oligopyrromethanes **350.3a**–**b** (Scheme 350). For instance, acid-catalyzed condensation of *mono*-formyl porphyrin **350.1** and 5-(pentafluorophenyl) dipyrromethane **350.3a** followed by the DDQ oxidation afforded the trimeric nickel(II) porphyrin **350.4a**, which was subsequently converted into the zinc(II) complex **350.4b**. In the subsequent step, the oxidative fusion of **350.4b** using the combination of DDQ and Sc(OTf)_3_ resulted in the formation of the desired AAA-type porphyrin tape **350.6a** in 48% yield. ADA and ADDA porphyrin tapes, **350.6b** and **350.9a**, were similarly prepared. The UV–vis–NIR spectrum of **350.9a** displayed a significant bathochromic shift (Q-like bands at 1400 and 1657 nm) and a large value of TPA cross-section of 1400 GM.

The singly fused diporphyrin quinone **350.10** was reported by Yamashita, Sugiura et al.^653^ The compound was obtained in low yield in a tandem PIFA-induced oxidation of a 5,15-diarylporphyrin along with the expected monomeric dioxoporphodimethane. **350.10** displayed strong panchromatic absorption between 300 and 1000 nm, with a large maximum at 724 nm, and possessed a significantly reduced electrochemical HOMO–LUMO gap relative to the monomeric quinone.^653^

**Scheme 351:**
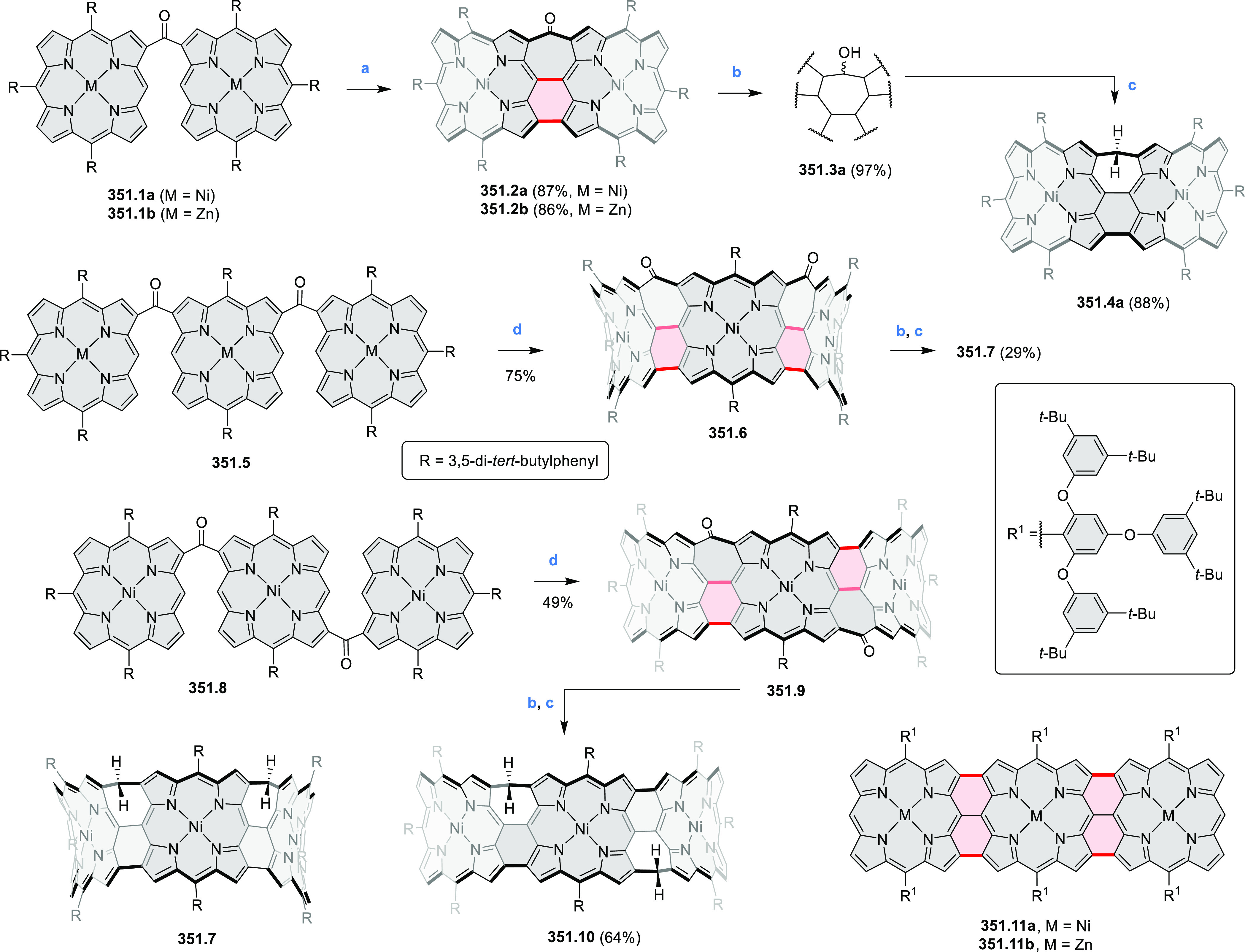
Synthesis of Carbonyl- and Methylene-Containing Porphyrin Arch Tapes Reagents and conditions: (a)^654^ DDQ/Sc(OTf)_3_ (5 equiv), toluene, 60 C, 2–3 h; (b) NaBH_4_, CeCl_3_, DCM/MeOH, −80 °C; (c) (1) HBF_4_·OEt_2_, DCM, rt, 10 min, (2) BH_3_·NEt_3_, rt, 10 min; (d) DDQ/Sc(OTf)_3_ (15 equiv), toluene, 60 °C, 5 h.

Osuka et al. introduced seven membered rings into porphyrin tapes to produce significant curvature of the π system.^654^ Carbonyl-containing arch-tapes were obtained from the bridged porphyrin dimers **351.1** or trimers **351.5** and **351.8** via an intramolecular oxidative fusion using DDQ and Sc(OTf)_3_ (Scheme 351). Subsequently, the Luche reduction with NaBH_4_ and CeCl_3_ produced the carbinol **351.3a**, which was directly subjected to ionic hydrogenation reaction with HBF_4_·Et_2_O and BH_3_·NEt_3_ to afford the methylene-containing dimer **351.4a** in 88% yield. The same two-step procedure was applied in the syntheses of **351.7** and **351.10**. Because of their curved shape, these arch-porphyrin tapes were found to be highly soluble. In comparison with the planar trimers **351.11a**–**b**, containing bulky *meso*-substituents to prevent aggregation, UV–vis–NIR absorption spectra of carbonyl-containing arch tapes were most red-shifted, with lowest-energy Q bands at 1260 and 1254 nm and electrochemical HOMO–LUMO gaps of 0.87 and 0.85 eV for **351.5** and **351.8**, respectively. The methylene-bridged arch tape **351.7** exhibited strong association with C_60_ in toluene, with an association constant of (1.5 ± 0.4) × 10^7^ M^–1^ at 25 °C.

**Scheme 352:**
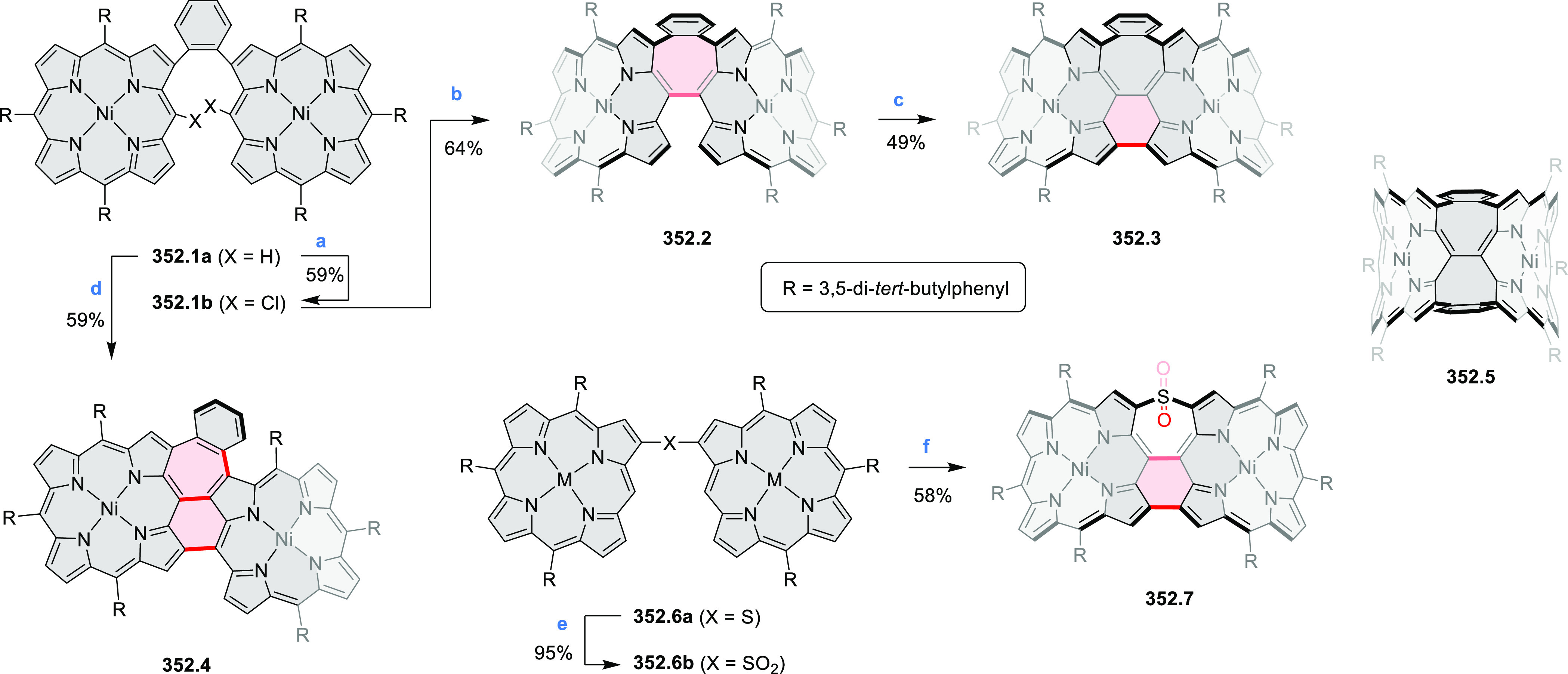
Synthesis of 1,2-Phenylene- and Sulfone-Containing Porphyrin Arch-Tape Dimers Reagents and conditions: (a)^655^ Palau′Chlor, CHCl_3_, rt, 6 h; (b)^655^ Ni(cod)_2_, 1,5-cyclooctadiene, DMF, 100 °C, 9 h; (c)^655^ DDQ, Sc(OTf)_3_, toluene, 80 °C, 2 h; (d)^655^ DDQ, Sc(OTf)_3_, toluene, 60 °C, 1 h; (e)^656^ H_2_O_2_, NaWO_4_·2H_2_O, MeN(*n*Oct)_3_·HSO_4_, PhP(O)(OH)_2_, toluene, 50 °C, 13 h; (f)^656^ AuCl_3_, AgOTf, (CHCl_2_)_2_, 80 °C, 30 min.

Later in 2018, Osuka reported the synthesis of singly and doubly 1,2-phenylene-expanded porphyrin arch-tape dimers **352.2**–**5** (Scheme 352).^655^ The oxidative approach described above was not successful with the *ortho*-phenylene precursor **352.1a**, which produced a rearranged product **352.4** when treated with DDQ and Sc(OTf)_3_. The structure of this product was consistent with an oxidation-induced 1,2-migration of the phenylene bridge to the neighboring β-position. Nickel(0)-mediated reductive coupling of the chlorinated precursor **352.1b** produced **352.2**, which upon oxidation with DDQ and Sc(OTf)_3_ in the subsequent step furnished the desired arch-tape dimer **352.3**. Interestingly the doubly 1,2-phenylene-expanded arch-tape dimer **352.5** could be obtained using the oxidative method at slightly higher temperature. X-ray crystallographic investigations revealed that the embedded eight-membered rings in the phenylene-containing dimers produced even more contorted structures than the carbonyl-containing porphyrin tapes bearing seven-membered rings. The Q-band of the absorption spectrum became more red-shifted with the increasing molecular contortion and the electrochemical HOMO–LUMO gaps became smaller, reaching respectively 1294 nm and 0.77 eV in **352.3**. In subsequent work, the same authors prepared sulfone-containing arch-tape dimers which also exhibited large molecular contortion.^656^ The synthesis of **352.3** was achieved by first converting the bridging sulfur unit in **352.6a** into the sulfone. The resulting **352.6b** was then subjected to the oxidative fusion reaction using AuCl_3_ and AgOTf instead of DDQ and Sc(OTf)_3_, to provide the target **352.7** in 58% yield.

**Scheme 353:**
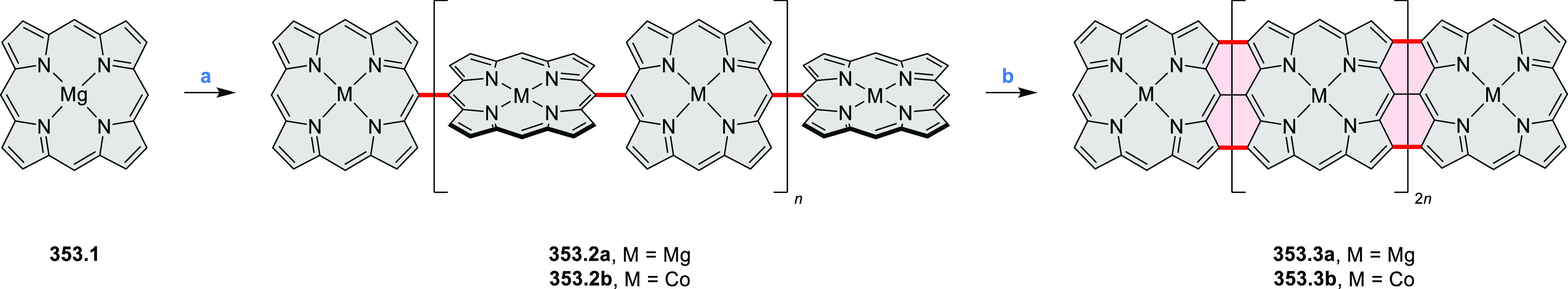
Electropolymerization of Metalloporphyrins Reagents and conditions: (a)^658^ electrochemical potential, *E*_app_ = 0.35 V, CH_3_CN; (b)^658,660^*E*_app_ > 0.65 V, in TBAPF_6_/CH_3_CN.

*meso*-Unsubstituted porphyrins can be oxidatively coupled using electrochemical methods to produce benzo-fused dimers.^657^ Two-step electropolymerization unsubstituted magnesium porphine **353.1** in acetonitrile solutions produced films, which were proposed to contain triply linked porphine tapes **353.3a** (Scheme 353).^658^ The addition of water or a strong proton accepting additive such as lutidine to the magnesium porphyrin solution during the course of its electrooxidation was found to strongly accelerate the rate of film growth. This lutidine addition increased the efficiency of this process without modifying the redox properties and electric conductivity of the obtained polyporphine films. The intermediate magnesium(II) polyporphine **353.2a** could be transmetalated to the corresponding cobalt(II) species **353.2b**,^659^ which was electrochemically converted into the fused polymer **353.3b**.^660^ In comparison to **353.2b**, **353.3b** was found to be more stable toward potential cycling in an aqueous matrix, and was thus employed for sensing of sulfite in aqueous solutions. Structurally related polymeric porphyrin tapes were also obtained by using oxidative chemical vapor deposition (oCVD)^661−666^ and were utilized in photocatalysis^667^ and ammonia sensing.^668^

**Scheme 354:**
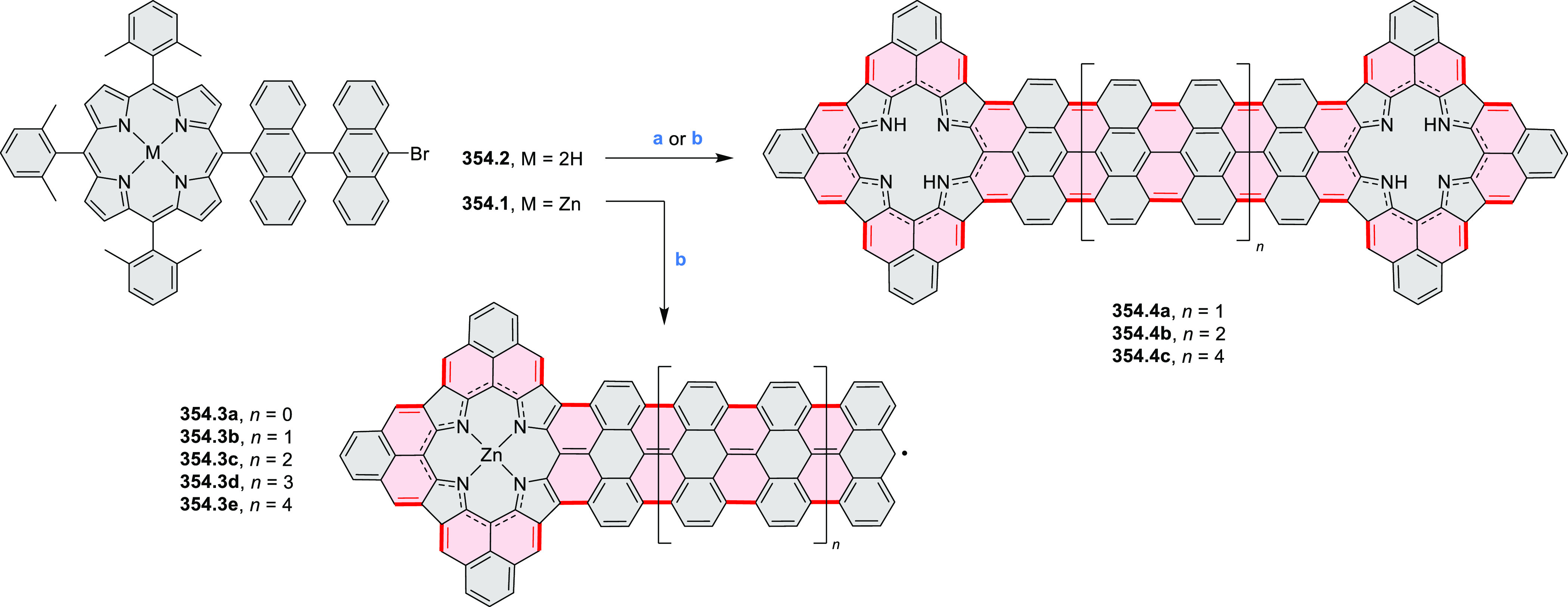
On-Surface Synthesis of Singly and Doubly Porphyrin-Capped Graphene Nanoribbon Segments Reagents and conditions: (a)^669^ 200–350 °C on the Au(111) surface; (b)^670^ 10,10′-dibromo-9,9′-bianthracene, 200–350 °C, Au(111).

In 2020, Bottari, Fasel, Torres et al. reported an on-surface synthesis of a structurally precise porphyrin–GNR hybrid **354.4a**. The two-step annealing process used by the authors consisted of Ullmann-type coupling and subsequent cyclodehydrogenation of the free-base porphyrin precursor **354.2** bearing a bromodianthryl moiety (Scheme 354).^669^ The atomically precise structure of the triply fused hybrid was characterized by STM and nc-AFM methods. The electronic properties were investigated by scanning tunneling spectroscopy (STS) in combination with DFT calculations, which revealed a low electronic gap of 0.4 eV. Later in 2021, the same authors reported on-surface synthesis of GNRs with increased lengths fused at either one or both of their termini with a porphyrin macrocycle (**354.3a**–**e**, **354.4b**–**c**).^670^ Combined high-resolution STM, STS and DFT data revealed weak hybridization of the electronic states of the GNR and the porphyrin moieties despite the high degree of conjugation in these hybrids.

### 7.4 [*cd*]-Fused porphyrinoids with Five- and Seven-membered rings

#### 7.4.1 Indeno[1,2,3-*cd*]porphyrins

**Scheme 355:**
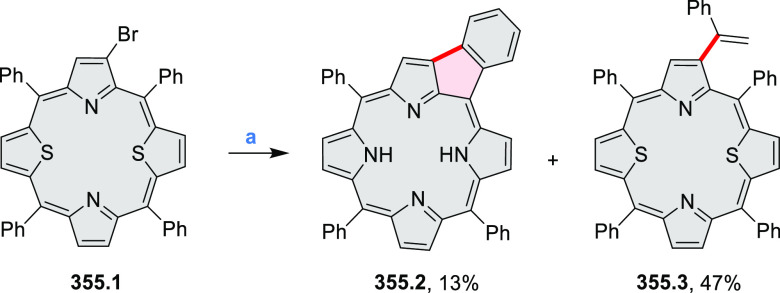
Synthesis of β-Substituted Dithiaporphyrins by a Heck Reaction Reagents and conditions: (a)^671^ styrene, Pd(OAc)_2_, K_2_CO_3_, PPh_3_, DMF/toluene (1:1, v/v), reflux, 1 h.

Indeno[1,2,3-*cd*]-fused porphyrins have been typically obtained by either CDA-based cyclizations or on-surface synthesis (cf. CR2017, Section 7.4.2). CDA type reactions may occasionally occur spontaneously, as evidenced by a recent report by Cavaleiro, de Souza, Neves et al., who observed formation of **355.2** under Heck-type conditions (Scheme 355).^671^ The UV–vis spectrum of **355.2** exhibited a broad and split Soret band, which was ascribed to the high deformation of the macrocycle caused by its five-membered ring.

**Scheme 356:**
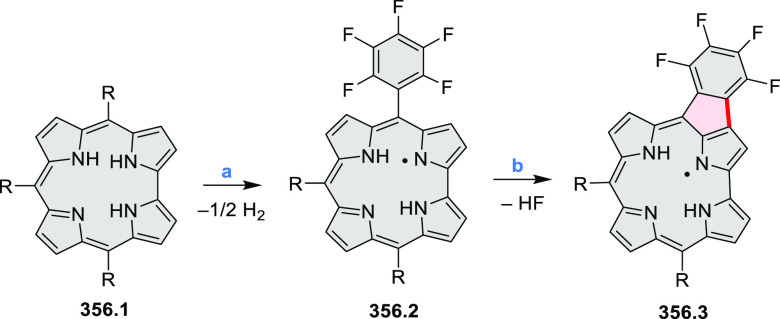
On-Surface Site-Selective Cyclization of Corrole Radicals Reagents and conditions: (a)^672^ Ag(111), 330 K; (b) Ag(111), 430 K.

On-surface synthesis of stable corrole radicals on Ag(111) was achieved via site-specific dehydrogenation of a pyrrole N–H bond in 5,10,15-tris(pentafluorophenyl)corrole **356.1** (Scheme 356).^672^ The process was triggered by annealing at 330 K under ultrahigh-vacuum conditions. It was found that surface-adsorbed corrole radicals were stable up to 430 K. At higher temperatures radical-cascade reactions caused site-selective ring closure in **356.2**, which produced an extended π-conjugated corrole system **356.3**. Above 550 K, formation of covalently coupled corroles on Ag(111) was revealed.

**Scheme 357:**
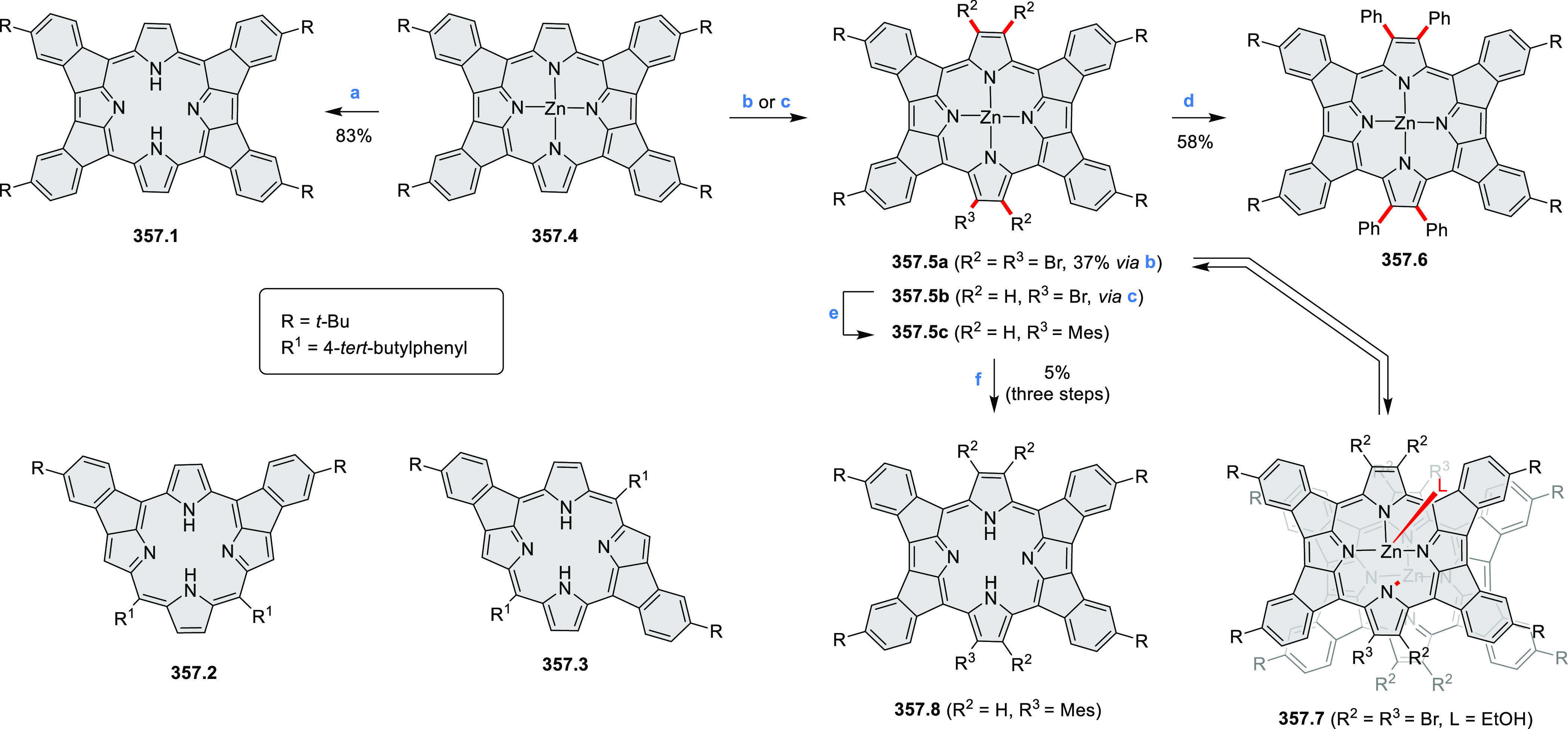
Synthesis of Quadruply Ring-Fused Porphyrinsa Reagents and conditions: (a)^673^ TFA, CHCl_3_; (b)^674^ NBS (5 equiv), CHCl_3_; (c)^675^ NBS (1.9 equiv), CHCl_3_; (d)^674^ (1) phenylboronic acid, Pd(PPh_3_)_4_, K_2_CO_3_, (2) TFA, NaHCO_3_, (3) Zn(OAc)_2_·2H_2_O; (e)^675^ 2,4,6-trimethylphenylboronic acid, Pd(PPh_3_)_4_, K_2_CO_3_; (f)^675^ TFA, CHCl_3_.

Free bases of the multiply fused porphyrins **357.1**–**3** were studied in 2017, Ishizuka, Kojima et al., who found a significant difference in the first and second protonation constants, which was ascribed to the structural rigidity of these macrocycles (Scheme 357).^673^ In particular, the first protonation of the quadruply fused porphyrin **357.1** (QFP) proceeded smoothly with TFA as the acid source, whereas the second protonation required a large excess of the acid, with the corresponding equilibrium constants of *K*_1_ = (1.3 ± 0.1) × 10^5^ M^–1^ and *K*_2_ = 7.3 ± 0.3 M^–1^, respectively. Br- and Ph-functionalized QFP derivatives **357.5a** and **357.6** were reported show red-shifts in their absorption spectra, caused by substitution effects.^674^ Additionally **357.5a** underwent unusual ligand-induced dimerization process, which involved dissociation of one of the nonfused pyrroles. This dimerization caused hypsochromic shifts of absorption bands, resulting from H-type aggregation of the two porphyrins. NH tautomerization behavior was studied for the unsymmetrically substituted free base **357.8**, which was obtained in three steps from **357.4**.^675^ Δ*G*^‡298^ for tautomerization of **357.8** was found to be larger than that of free base tetraphenylporphyrin, and the difference was ascribed to the more severe steric congestion of the NH protons in the deformed QFP core. On the basis of kinetic and thermodynamic analyses, NH tautomerism in **357.8** was proposed to involve dissociation of a π-stacked dimer stabilized by dipole–dipole interactions.

#### 7.4.2 Other Cyclopenta-Fused Systems

**Scheme 358:**
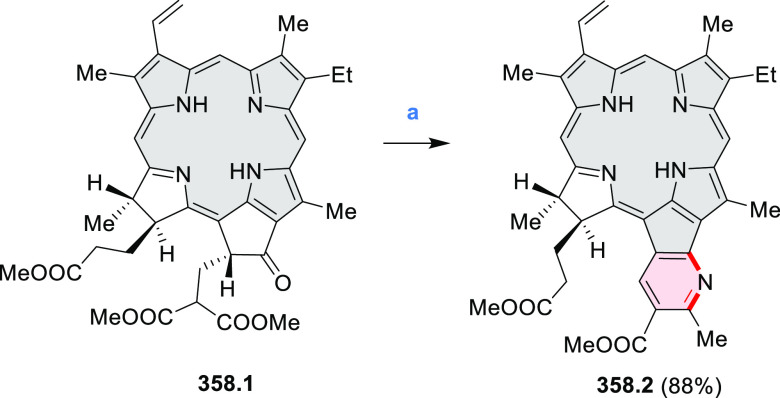
Synthesis of π-Extended Derivatives from Enone-Substituted Chlorins Reagents and conditions: (a)^676^ NH_4_OAc, acetic acid, N_2_, reflux.

π-Extended cyclopenta-fused systems can be accessed by chemical modification of chlorophyll derivatives. In a recent example, pyridine-fused chlorins such as **358.2** were elaborated via conventional condensation chemistry (Scheme 358).^676^ In the final step, **358.1** was reacted with ammonium acetate in acetic acid to close the heterocyclic ring. These pyridine fused chlorophylls showed significant bathochromic shifted absorption in the near-infrared region (748–766 nm) with unusually broadened Q bands, as well as small electrochemical HOMO–LUMO gaps (1.89–2.02 eV).

**Scheme 359:**
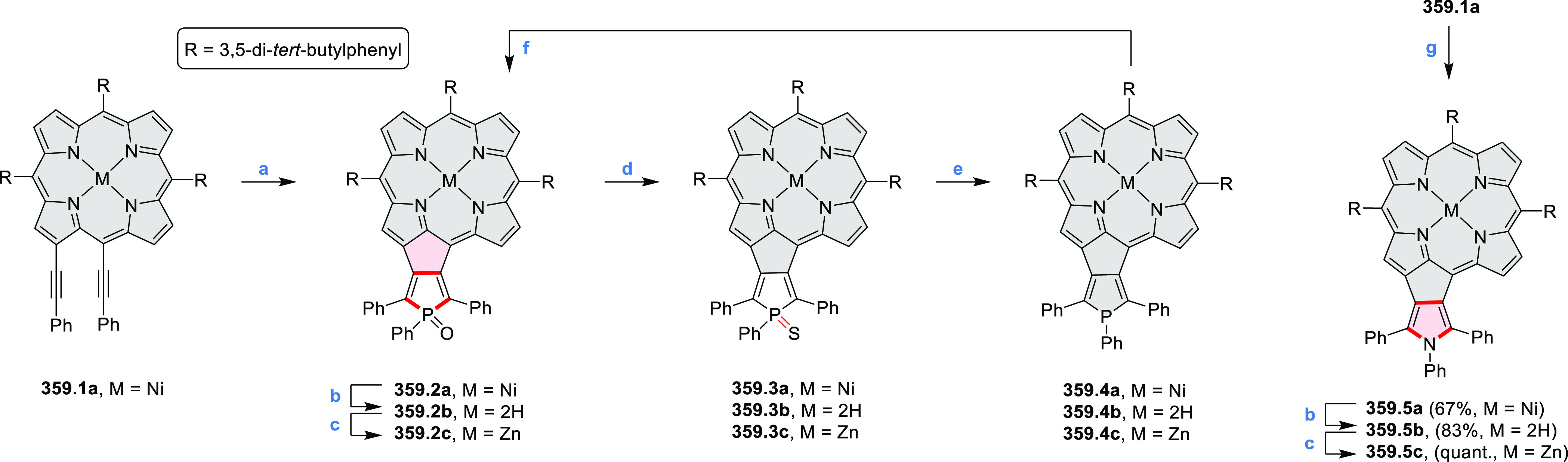
Phosphole-Fused and Pyrrole-Fused Dehydropurpurins Reagents and conditions: (a)^677^ (1) Ti(O*i*-Pr)_4_−*i*-PrMgCl, (2) PhPCl_2_, (3) H_2_O_2_ or S_8_, Et_2_O; (b) conc. H_2_SO_4_, CF_3_COOH; (c) Zn(OAc)_2_·2H_2_O, DCM/MeOH; (d) Lawesson’s reagent, toluene; (e) P(NMe_2_)_3_, toluene; (f) air; (g)^678^ aniline, PdCl_2_, Et_3_N, toluene/DMSO.

In 2019, Higashino and Imahori described a synthesis of phosphole-fused dehydropurpurins via titanium-mediated [2 + 2+1] cyclization.^677^ The reaction of bis(alkynyl)porphyrin **359.1a** with excess Ti(O*i*-Pr)_4_ and *i*-PrMgCl produced the phosphole-fused product **359.2a** in 62% yield (Scheme 359). The latter species was converted into a range of derivatives, including the trivalent **359.4a**–**c**, which were obtained by reacting **359.3a**–**c** with P(NMe_2_)_3_ in toluene solution. These phospholes were gradually reoxidized under air to the respective oxides **359.2a**–**c**, probably owing to the electron-rich nature of the porphyrin core. Dehydropurpurins **359.4a**–**c** showed diminished absorption (and slightly red-shifted Q-bands) and smaller fluorescence quantum yields (Φ_f_) relative to the P = O derivatives, a difference potentially caused by the 24π antiaromatic character of the phosphole-containing π-system of the porphyrin. Later in 2020, the same group synthesized the pyrrole-fused dehydropurpurins **359.5a**–**c** via similar [2 + 2+1] cyclization strategy.^678^ The antiaromatic 24π-electron conjugation in these pyrrole-fused 7,8-dehydropurpurins appeared to be more significant than in their phosphole counterparts.

**Scheme 360:**
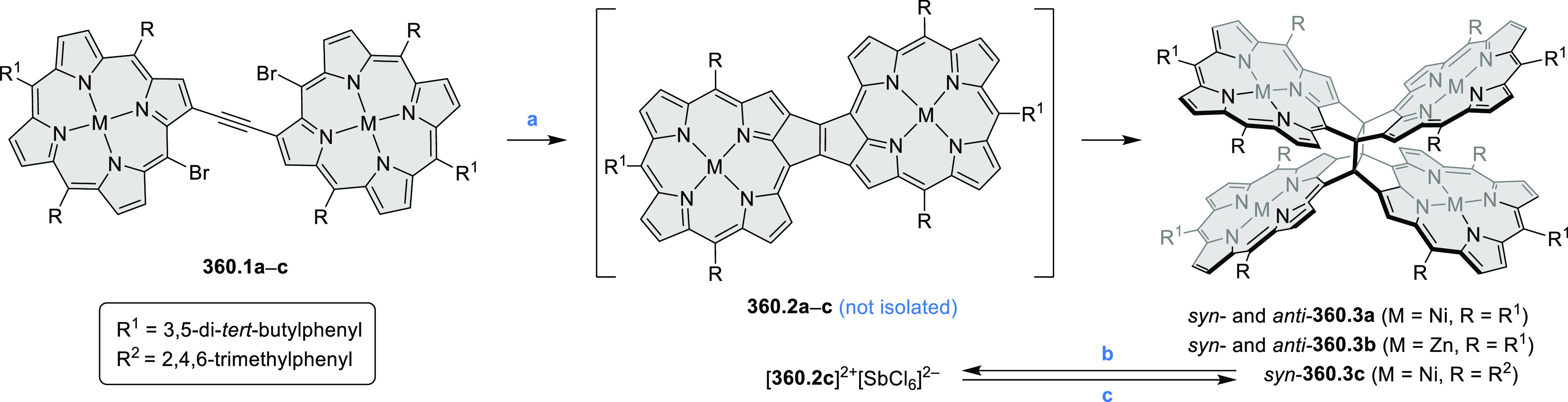
Synthesis of Etheno-Fused Diporphyrin Dications Reagents and conditions: (a)^679^ Ni(cod)_2_, 2,2′-bipyridyl, THF; (b)^680^ BAHA (4 equiv), DCM, rt; (c)^680^ CoCp_2_ (4 equiv), DCM, rt.

In 2018, Shinokubo et al. attempted the synthesis of **360.2a** using the nickel-mediated tandem double cyclization of ethynylene-linked dibromodiporphyrins **360.1a** (Scheme 360).^679^ However, presumably because of the high reactivity of **360.2a**, it underwent a thermal [2 + 2] cycloaddition at the fused C–C double bond to afford the X-shaped cyclobutane-linked tetraporphyrin **360.3a** as a mixture of *syn* and *anti* isomers. Spectroscopic investigations indicated that the formation of these X-shaped tetraporphyrins might involve a thermally activated triplet state of **360.2a** in a thermal [2 + 2] cycloaddition reaction. It was later found that a differently substituted tetraporphyrin **360.3c**, obtained regioselectively in the syn form, underwent two-step four-electron oxidation with BAHA.^680^ Subsequent spontaneous ring opening produced two molecules of the etheno-fused diporphyrin dication [**360.2c**]^2+^, which was found to be nearly planar in the solid state. Reduction of the latter species with excess of cobaltocene regenerated the cyclobutane-linked tetraporphyrin **360.3c**. This redox-mediated cyclobutane cycloreversion could also be induced electrochemically and produced large hysteresis in the cyclic voltammogram. The unique reactivity of the etheno-fused diporphyrins **360.2a**–**c** was attributed to the contribution of antiaromaticity to their macrocyclic conjugation.

#### 7.4.3 Fused 7-Membered Rings

**Scheme 361:**
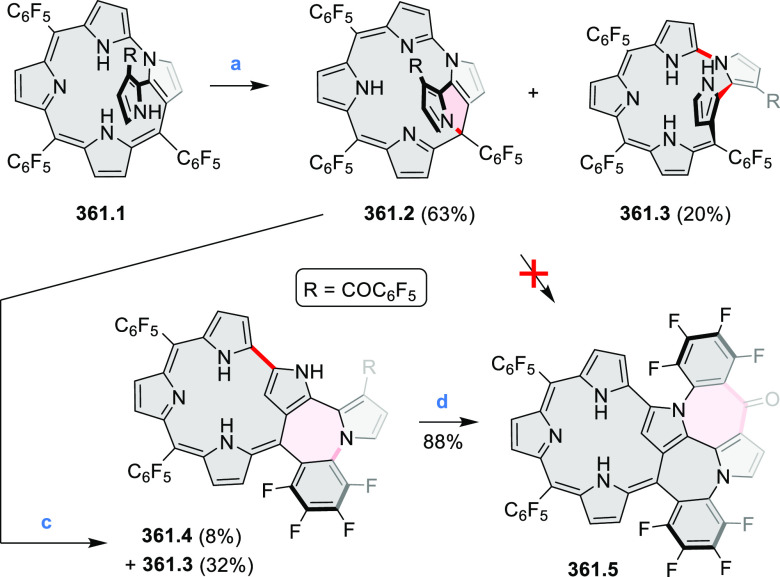
Synthesis of Singly and Doubly N-C_Ar_-Fused Confused Corroles Reagents and conditions: (a)^681^ DDQ (1 equiv), DCM; (b) NBS, DCM; (c) toluene, reflux; (d) DMF, 30 °C.

An oxidative fusion reaction of the pyrrole-substituted norrole **361.1**, reported in 2016 by Furuta, Xie et al., produced in the N–C_meso_-fused pyrrolyl isonorrole **361.2** accompanied by pentaphyrin **361.3** (Scheme 361).^681^ Upon refluxing in toluene for 10 h, **361.2** underwent skeletal rearrangement to the azepine-fused system **361.4**, again accompanied by **361.3**. Subsequent azepine fusion, yielding the doubly annulated product **361.5**, occurred when **361.4** was stirred in DMF for 4 h at 30 °C. This reaction was facilitated by the basic nature of DMF solvent, which induced deprotonation of the NH group of **361.4** followed by its nucleophilic attack at the C_6_F_5_ moiety. Bathochromic shifts of Q-bands observed for the peripherally annulated products **361.4** and **361.5** relative to **361.2** were explained by the recovery of conjugation in the two former compounds. Dimeric copper complexes based on the extended frameworks of **361.4**–**5** were subsequently reported by the same authors.^682^

**Scheme 362:**
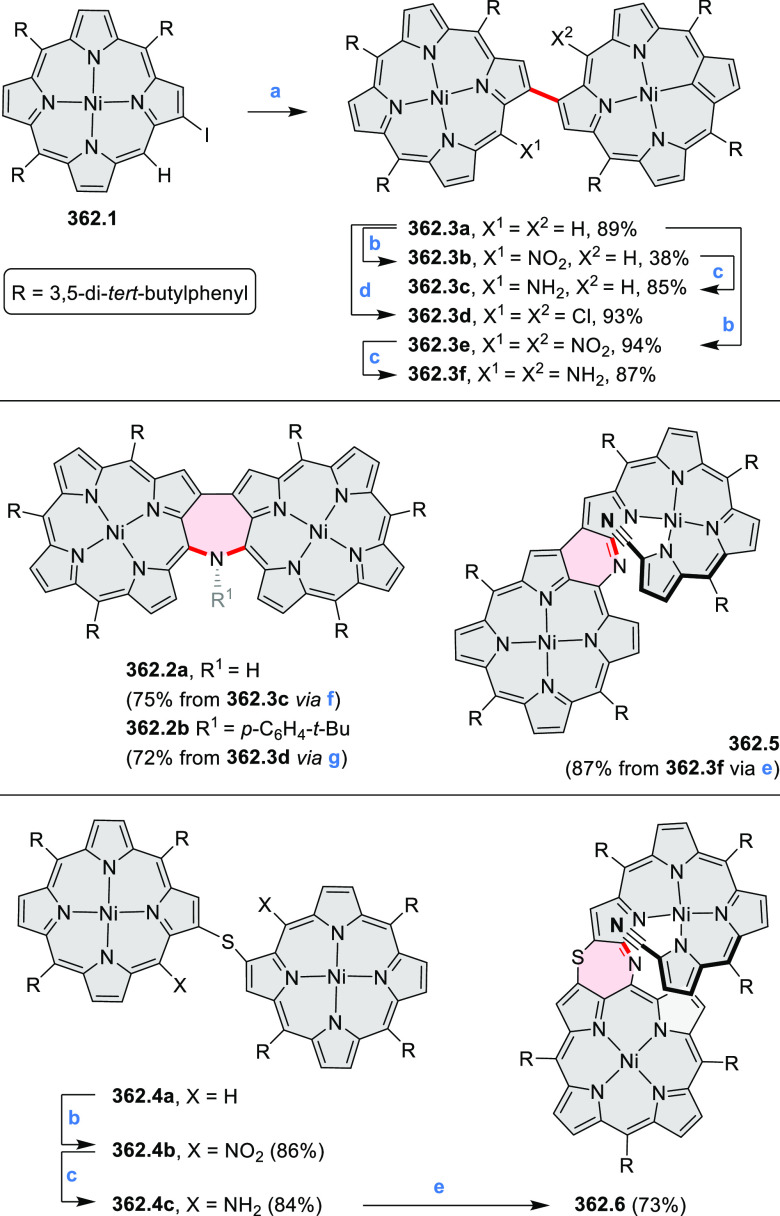
Synthesis of β -to- β-Linked *meso-*Aminoporphyrin Dimers Reagents and conditions: (a)^683^ Ni(cod)_2_, DMF, 100 °C; (b)^683,684^ I_2_, AgNO_2_, DCM/MeCN, rt; (c)^683,684^ NaBH_4_, Pd/C, DCM/MeOH, rt; (d)^683^ PhICl_2_, CHCl_3_, 0 °C; (e)^684^ PbO_2_, DCM, rt; (f)^683^ PbO_2_, DCM, rt, and then, SnCl_2_, DCM, rt; (g)^683^ 4-*tert*-butylaniline, Pd-PEPPSI-IPr, NaO*t*-Bu, toluene, 120 °C.

Osuka et al. reported the synthesis of an azepine-fused nickel(II) porphyrin dimer via oxidative amination of β–β linked Ni(II) porphyrin dimer (Scheme 362).^683^ The synthesis of the amine precursor **362.3c** was achieved in 3 steps starting from the β-iodoporphyrin **362.1**. Oxidative fusion of **362.3c** with PbO_2_, followed by treatment with SnCl_2_, afforded **362.2a** in 75% yield. **362.2b** was obtained through a different route by performing a 2-fold Buchwald–Hartwig amination on **362.3d** with 4-*tert*-butylaniline. Oxidation of **362.2a** gave successively a stable neutral aminyl radical and a nitrenium ion, whereas **362.2b** afforded a formal nitrenium dication in a single-step, with the two-electron oxidation mainly taking place on the bridging nitrogen.

The same authors later showed that the oxidation of the dimer **362.3f** carrying two *meso*-amino groups gave a hybrid product containing a helical tetrapyrrin fused to an intact porphyrin macrocycle.^684^ The oxidation was proposed to involve a *meso*-aminyl radical intermediate, in which the α-carbon of the cleaved macrocycle formed a N–C(α) bond with the nitrogen atom of the other porphyrin segment. Similarly, a sulfur-bridged analogue **362.6** was also synthesized from **362.4c**. **362.5** and **362.6** displayed intense NIR absorption bands at 1200–1400 nm and reversible redox processes. The more red-shifted absorption and the greater ease of first reduction suggested a more effective π-conjugation through the direct β–β linkage in **362.5** in comparison with **362.6**. The helical structure of the tetrapyrrin-fused Ni(II) porphyrins enabled chiral resolution into stable enantiomers.

**Scheme 363:**
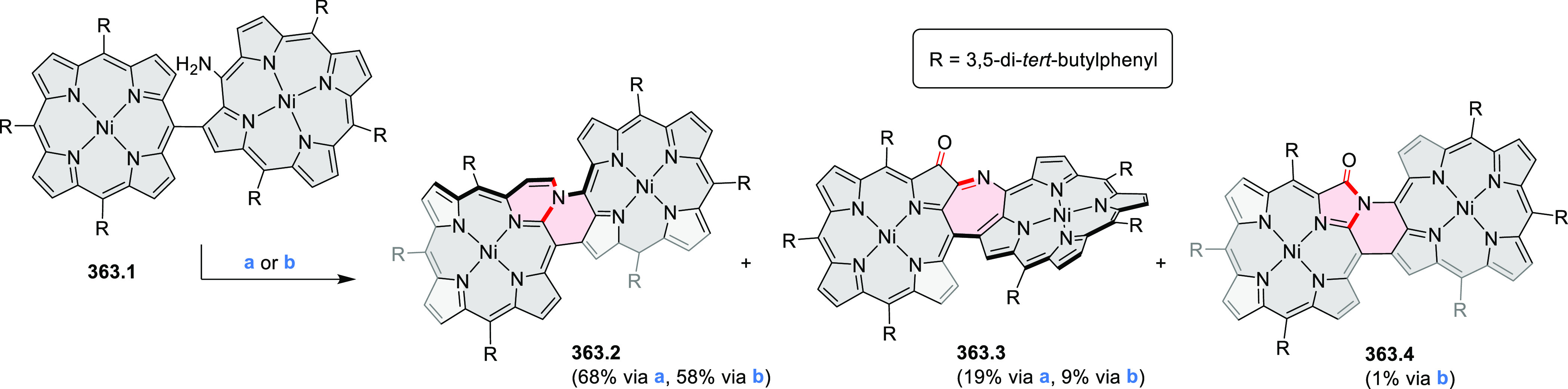
Oxidative Reactivity of *meso-*Amino *meso-*to-β-Linked Nickel(II) Aminoporphyrin Reagents and conditions: (a)^685^ PbO_2_ (100 equiv), DCM, rt, 12 h; (b) MnO_2_ (100 equiv), DCM, rt, 16 h.

Oxidation of the β–*meso*-linked porphyrin dimer **363.1** resulted in the formation of three differently fused porphyrin dimers **363.2**, **363.3**, and **363.4** (Scheme 363).^685^ Specifically, the use of PbO_2_ in DCM at rt gave **363.2** and **363.3** in yields of 68 and 19%, respectively, whereas oxidation with MnO_2_ produced **363.4** was obtained in 1% yield along with **363.2** (58%) and **363.3** (9%). The UV–vis–NIR absorption spectra showed perturbed optical properties with a large red-shift in the lowest-energy Q-like band (937 nm) for **363.3**. The electrochemical gaps were found to be in the order of **363.4** > **363.2** > **363.3**.

### 7.5 Porphyrinoids with Polycyclic Subunits

#### 7.5.1 Porphyrinoids with Benzannulated Bipyrrole Units

**Scheme 364:**
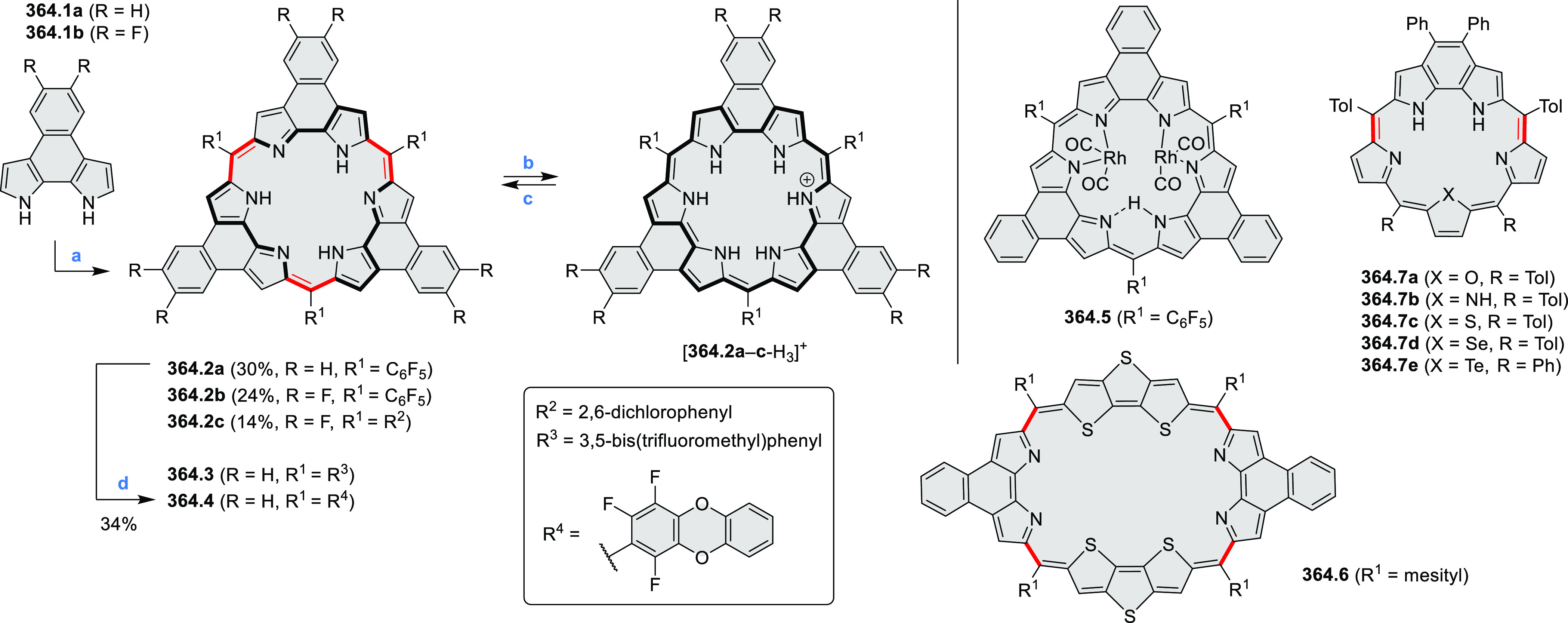
Naphthobipyrrole Macrocycles Reagents and conditions: (a)^687^ appropriate aldehyde, TFA, DCM, (2) DDQ, TEA; (b)^687^ TFA, cobaltocene, THF-*d*_8_; (c)^687^ TEA; (d)^690^ catechol, K_2_CO_3_, NMP, 100 °C, 6 h.

Benzo and naphthobipyrrole fragments have been introduced into porphyrinoid frameworks as a means of rigidifying the macrocycle (for earlier work, see CR2017, Section 7.5.1). The effect of this rigidification is most apparent in expanded macrocycles, e.g. sapphyrins (**364.2a**–**e**),^686^ rosarins,^687^ or rubyrins.^688^ Peripherally substituted antiaromatic naphthorosarins **364.2a**–**c** were obtained by Byon, Sessler, Lee et al. from the corresponding naphthobipyrroles **364.1** (Scheme 364).^687^**364.2c**, bearing both peripheral fluorine and meso-2,6-dichlorophenyl substituents, was reduced to a stable 25 π-electron radical species upon treating with TFA. Subsequent formation of the 26 π-electron trication, [**364.2c**-H_3_]^+^, was found to be slower than in the case of [**364.2a**-H_3_]^+^ or [**364.2b**-H_3_]^+^. This behavior of naphthorosarins is contrasted with the chemistry of the analogous naphthorubyrin, which prefers 26 π-electron conjugation and could not be oxidized to the corresponding 24 π-electron state.^688^ The properties of naphthorosarins can be further tuned by substitution: the electron-deficient **364.3** was found to form a cofacially stacked noncovalent dimer,^689^ whereas **364.2a** was found to undergo nucleophilic substitution with catechol to yield **364.4**.^690^

In a naphthorosarin rhodium complex **364.5**, prepared by Sessler, Lee and co-workers, the macrocycle maintained its planarity, but the two rhodium ions reside on opposite sides of the molecular plane, resulting in inherent chirality.^691^ The rhodium(I)–rhodium(I) distance was found to be 3.143 Å. The UV–vis absorption spectrum of **364.5** showed multiple absorption bands between 416 and 496 nm, along with bands at 623 and 653 nm, which was distinct to that of the free base **364.2a**. Cyclic voltammogram measurements revealed that upon metalation, the tendency to undergo reduction or oxidation was less pronounced than in the free base naphthorosarin and consequently, reduction to the corresponding 26 π-aromatic form became more difficult.

A core-modified rigid [32]octaphyrin(1.0.1.0.1.0.1.0) **364.6** derived from rigid naphthobipyrrole and dithienothiophene (DTT) precursors were reported by Zhang, Kim, Sessler et al.^692^ The X-ray crystallographic analysis of revealed the macrocycle was essentially a planar system and further spectroscopic investigations indicated a weakly 32 π-electron antiaromatic or nonaromatic character of **364.6**. **364.6** underwent proton-coupled two-electron reduction to produce the aromatic 34 π-electron state, in the presence of HCl and other hydrogen halides. The intermediate 33 π-electron radical species were observed upon addition of acids (such as TFA) that are less redox active than HCl to solutions of **364.6**.

#### 7.5.2 Cyclooctatetraene-Fused Systems

**Scheme 365:**
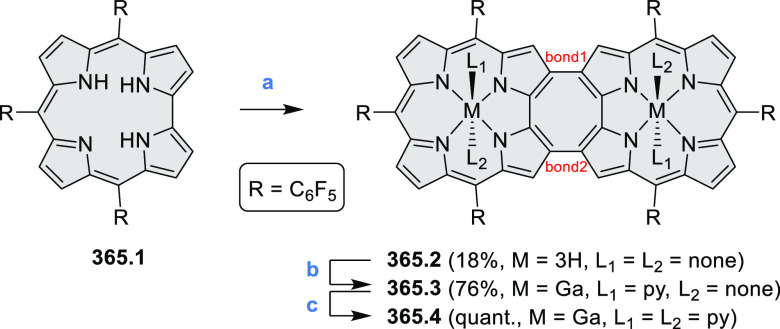
Synthesis of a Cyclooctatetraene-Bridged Gallium(III) Corrole Dimer Reagents and conditions: (a) 1,2,4-trichlorobenzene, reflux, 24 h; (b) (1) GaCl_3_, pyridine, N_2_, (2) DCM, *n*-heptane; (c) pyridine.

A modified synthetic procedure to obtain cyclooctatetraene (COT) fused corrole dimer was reported by Z. Gross et al. (Scheme 365).^693^**365.1** was heated in 1,2,4-trichlorobenzene at 200 °C for 24 h in air which allowed the isolation of corrole dimer **365.2** in 18% yield. Upon treating **365.2** with GaCl_3_ in pyridine under inert conditions, **365.3** was obtained, wherein one pyridine ligand was coordinated to each metal center. Upon recrystallization in pyridine, the tetrapyridine complex **365.4** was obtained in quantitative yield. The fusion of the two corrole units in **365.2**–**4** was found to produce significant distortion of bond lengths in the COT ring. Extensive π delocalization through the COT bridge in these systems was reflected in their absorption spectra, which contained low-energy bands reaching into the near-infrared (λ_max_ at 720–724 nm).

#### 7.5.3 Thiophene-Fused Systems

**Chart 33:**
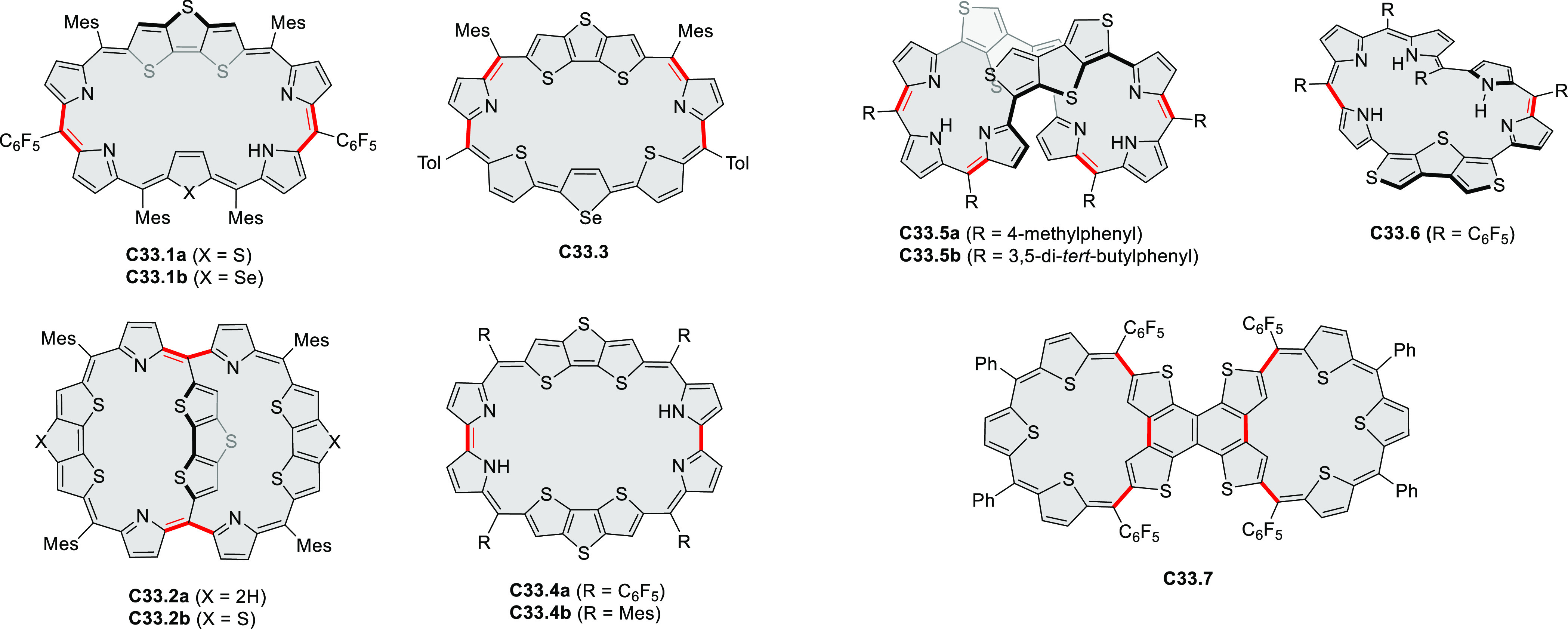
Porphyrinoids Containing Polycyclic Thiophene-Based Subunits

In 2016, Chandrashekar and co-workers reported Möbius-aromatic [32]π heptaphyrins **C33.1a**–**b**, which were obtained via acid-catalyzed cross-condensation of two modified tripyrranes with pentafluorobenzaldehyde (Chart 33).^694^ Proton NMR studies indicated weak Möbius aromaticity at 298 K; however, upon lowering the temperature, the molecule adopted a [4n]π Möbius conformation with a strong diatropic ring current. This aromaticity was further supported by the ACID plots obtained from DFT calculations. Similarly designed, planar [30]π heptaphyrin **C33.3** and [34]π octaphyrins **C33.4a**–**b** were later reported to be aromatic in freebase and protonated forms.^695^ The more expanded dithienothiophene-containing [40]π nonaphyrin was found to adopt a twisted figure-eight conformation.^696^

Bicyclic dithienothiophene-bridged [34]octaphyrins **C33.2a**–**b** were reported to form Baird-type aromatic triplet states.^697^ The synthetic route to these macrocycles involved an acid-catalyzed condensation reaction involving a diformyl dithienothiophene as the source of the bridging unit. The absorption spectrum of **C33.2a** in DCM showed two B-like bands at 501 and 605 nm, which suggested two competing π-conjugation pathways, containing [26]π and [34]π electronic-circuits, within the same nonplanar framework. Two-electron electrooxidation of **C33.2a** led to the generation of a 40π-electron triplet species, showing features consistent with global Baird aromaticity. Later in 2019, Park, Kim and co-workers reported a detailed study on the significant changes in the macrocyclic aromaticity of **C33.2b** upon C_60_ complexation.^698^ The binding ability of the bowl-shaped macrocycle with C_60_ was monitored with spectroscopic titration, which confirmed 1:1 binding stoichiometry in a toluene solution with an association constant of about 1.8 × 10^3^ M^–1^. ^1^H NMR spectroscopy also indicated a host–guest complexation occurred by effective π–π interactions between the bowl-shaped macrocycle and fullerene. Furthermore, femtosecond transient absorption measurements revealed that formation of the photoinduced charge-separated state and the triplet excited-state populations of the bowl-shaped and rigid expanded porphyrin could be controlled by a simple complexation with C_60_.

In 2017, Higashino, Imahori et al. reported the synthesis of thiophene-fused dithiaoctaphyrins **C33.5a**–**b**.^699^ These systems were characterized by coexistence of a macrocyclic [36]π-electron circuit and noncyclic cross-conjugation. In a related [28]hexaphyrin **C33.6**, switching between these two types of conjugation was realized via a change in the topology of the π system.^700^ A naphthalene-fused dimer **C33.7** of an antiaromatic expanded isophlorin was reported by Kögerler, Anand et al. in 2017.^701^**C33.7** was obtained from atypical [3 + 2] acid-catalyzed condensation followed by a rare β–β oxidative dehydrogenation which occurred between the adjacent two ring-inverted thiophene rings. Fused dimer **C33.7** exhibited marginal peripheral aromaticity rather than strong global diatropicity or paratropicity and weak electronic communication as a result of cross-conjugation.

#### 7.5.4 Systems with Acene and Heteroacene Subunits

**Scheme 366:**
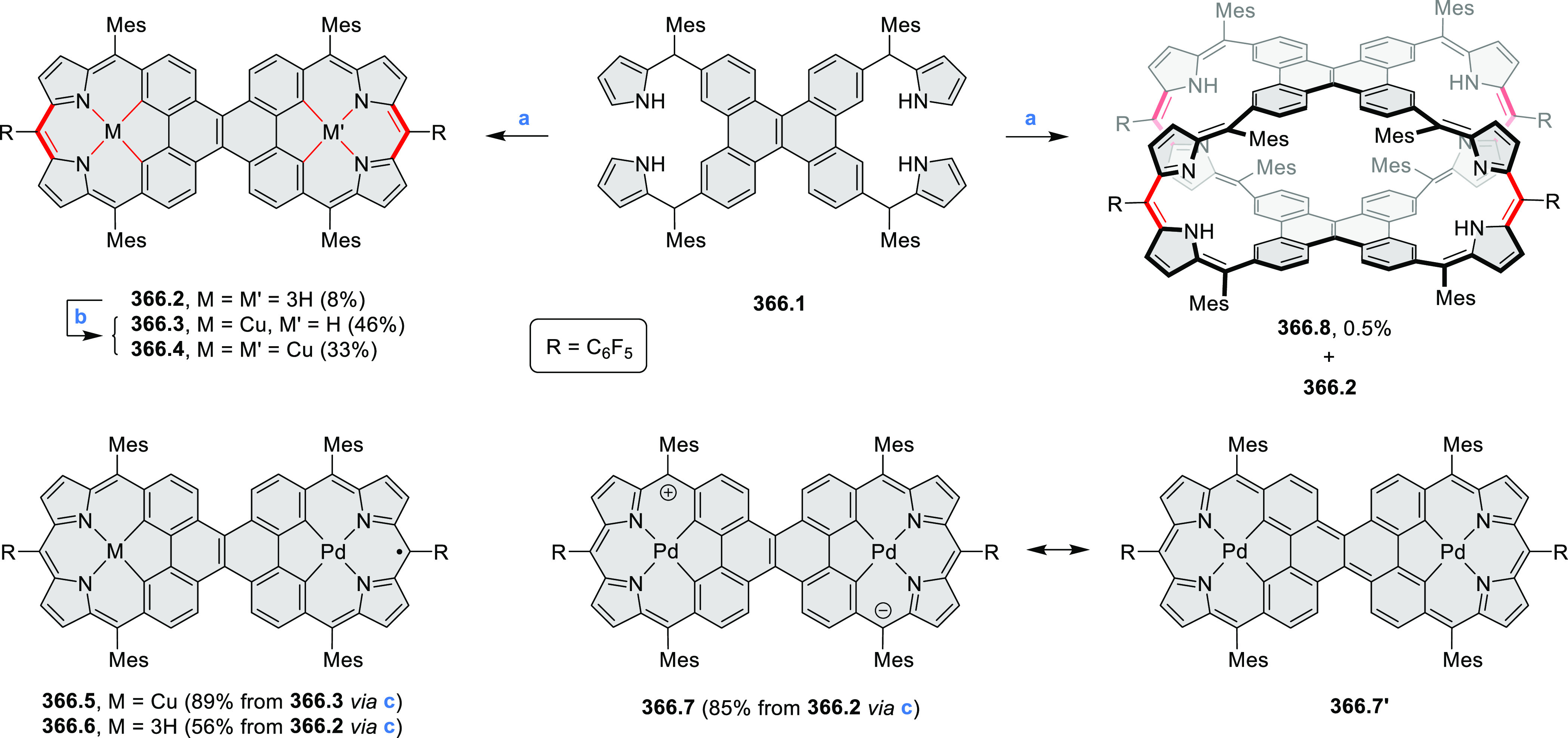
Synthesis of Dibenzo[*g*,*p*]chrysene-Fused Bisdicarbacorrole Reagents and conditions: (a)^702,704^ (1) pentafluorobenzaldehyde, BF_3_·OEt_2_, DCM, rt, (2) DDQ, rt; (b) Cu(OAc)_2_·H_2_O (73.8 equiv), CHCl_3_, reflux, 48 h; (c)^702,703^ Pd(PhCN)_2_Cl_2_ (3 equiv for **366.5**; 2 equiv for **366.6**; 15 equiv for **366.7**), PhCN, 180 °C.

A bis-“dicarbacorrole” **366.2** obtained by incorporating a dibenzo[*g*,*p*]chrysene moiety into a macrocyclic structure was reported in 2017 by Kim, Sessler, and co-workers (Scheme 366).^702^ This ligand system possesses two contracted corrole-like cores and stabilized higher oxidation states of metal ions. **366.2** was obtained in 8% yield in an acid catalyzed condensation reaction between **366.1** and pentafluorobenzaldehyde, followed by oxidation with DDQ. The trianionic cores in **366.2** were used for copper and palladium coordination to obtain mono- and bis-metal complexes, **366.3**–**7**.^702,703^ Compounds **366.2** and **366.4** were both antiaromatic, each containing two formally 16 π-electron dicarbacorrole subunits, whereas the mixed Cu/Pd complex **366.5** was an organic π radical, consisting of a fused 15 π-electron nonaromatic subunit and a 16 π-electron antiaromatic subunit. Formation of these organic radicals was ascribed to one-electron transfer from the ligand backbone to the Pd center. The UV–vis–NIR absorption spectra of bis-Pd **366.7** in toluene displayed an intense NIR absorption band at ca. 1420 nm, whereas **366.2** and **366.4** were characterized by very weak or negligible NIR absorptions over 1000 nm. A detailed spectroscopic analysis revealed a closed-shell ground state for the bis-Pd complex **366.7**, whereas the mono-Pd complex **366.6** existed as stable monoradical. The electronic structure of **366.7** was described as quinoidal with zwitterionic contributions such as **366.7’**.^703^

In parallel work, the authors utilized the **366.1** to prepare a fully conjugated three-dimensional expanded carbaporphyrin.^704^ An acid catalyzed [2 + 4] condensation reaction with pentafluorobenzaldehyde in DCM followed by DDQ oxidation resulted in the formation of the target product **366.8** in 0.5% yield along with the more favored product **366.2**. The neutral form of this cage **366.8** was nonaromatic, but it gained a global aromatic character upon protonation, which was inferred from the large negative NICS value (−11.63) and diatropic ring current observed in an ACID plot. In addition, the size of the cavity in **366.8** increased upon protonation to ca. 143 Å^3^.

**Scheme 367:**
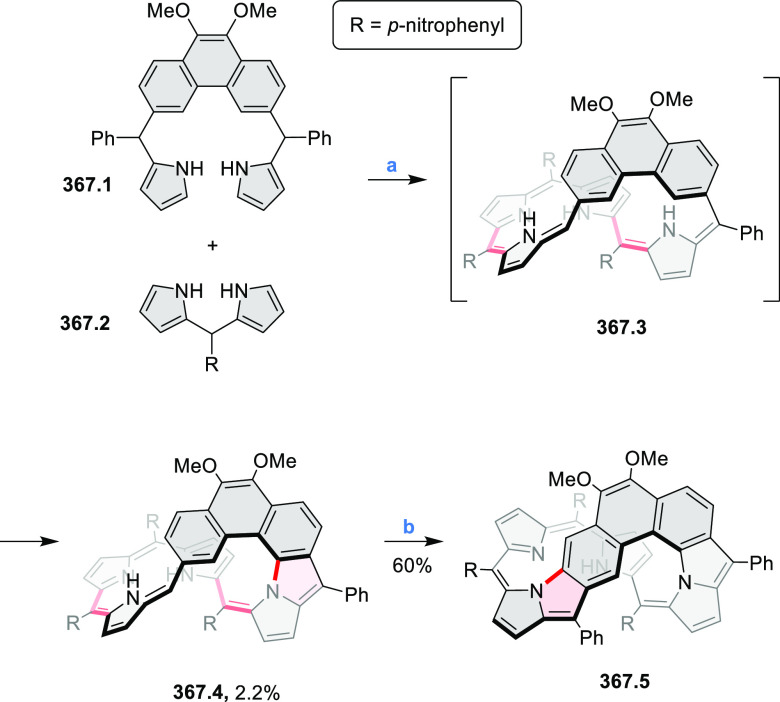
Expanded Carbaporphyrins Incorporating Aza-[5]helicene Motifs Reagents and conditions: (a)^705^ (1) *p*-nitrobenzaldehyde, BF_3_·OEt_2_, CHCl_3_, 2 h, (2) DDQ, 10 min; (b) BF_3_·OEt_2_, toluene, triethylamine, reflux, 90 min, (2) triethylamine.

The phenanthrene-containing porphyrinoid **367.3** was obtained by Szyszko, Latos-Grażyński, and co-workers as an intermediate during the acid-catalyzed condensation reaction between **367.1** and **367.2** (Scheme 367).^705^**367.3** underwent subsequent intramolecular oxidative ring fusion to yield the “helicenophyrin” **367.4**, which was shown to adopt a figure-of-eight conformation in the solid state. **367.4** appeared to be relatively difficult to protonate, but it formed a monoanion upon treatment with TBAF. When treated with BF_3_·OEt_2_, **367.4** gave the doubly fused macrocyclic product **367.5** in 60% yield, instead of an expected boron complex. The X-ray structure revealed that this macrocycle **367.5** incorporated an *ortho*-fused system of seven aromatic rings, forming an S-shaped helicene ribbon. Compounds **367.4**–**5** were both chiral and could be separated into enantiomers using chiral HPLC.

**Scheme 368:**
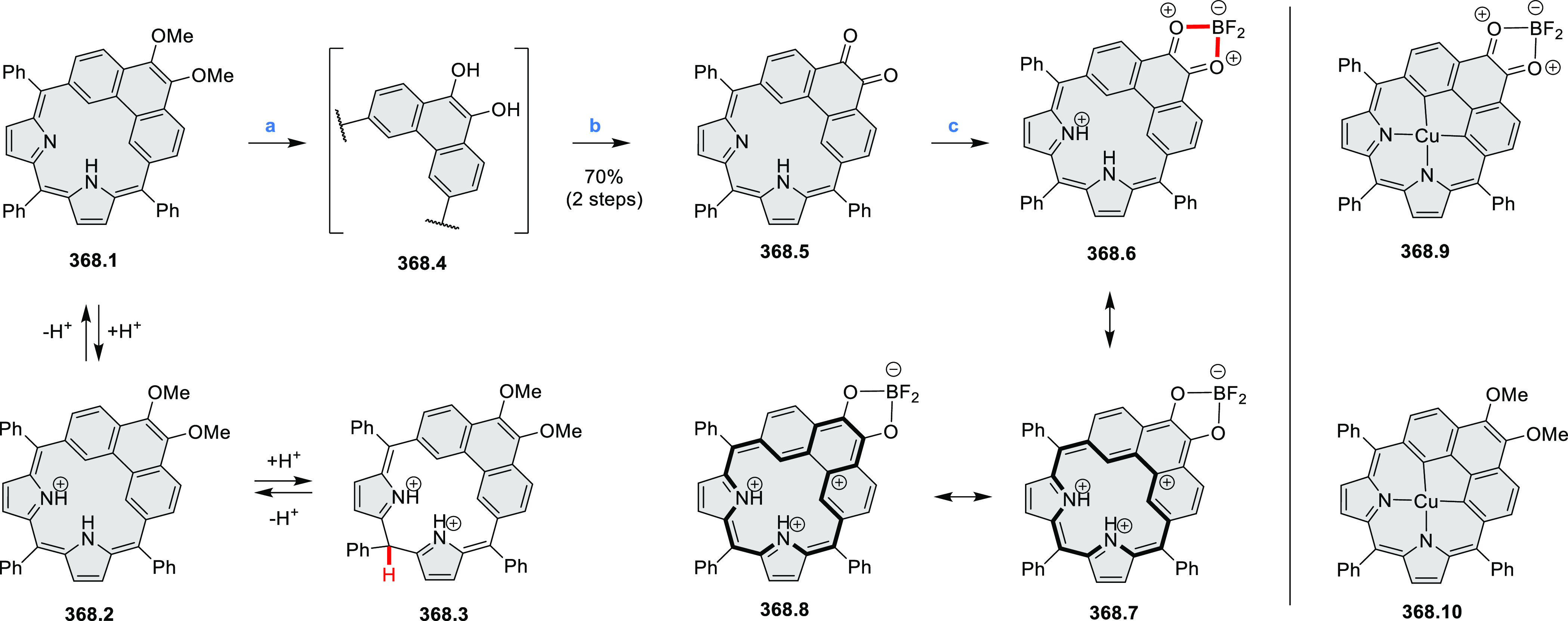
Phenanthriporphyrin Reagents and conditions: (a)^706^ H_2_SO_4_, 0 to 40 °C, 28 h; (b) air oxidation; (c) HBF_4_·Et_2_O.

**Scheme 369:**
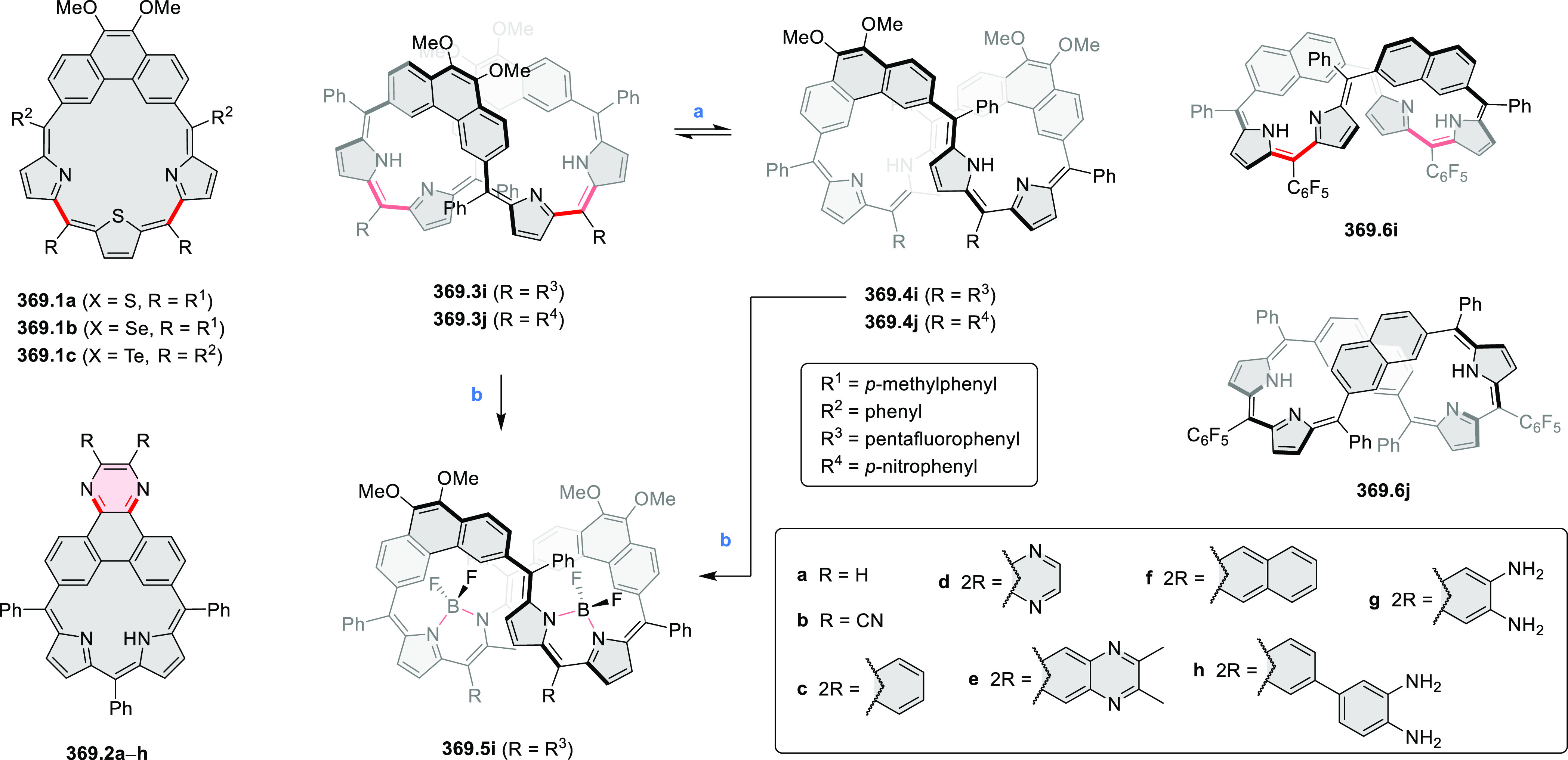
Phenanthrene- and Naphthalene-Containing Porphyrinoid Macrocycles Reagents and conditions: (a) Δ, amine; (b) (1) BF_3_·OEt_2_, TEA, toluene, reflux, 3 h, (2) TEA.

Later in 2018, Latos-Grażyński and co-workers explored the reactivity of phenanthrene-containing porphyrinoids **368.1** and **368.5** toward protic and Lewis acids (Scheme 368).^706^ When titrated with HBF_4_·Et_2_O, **368.1** underwent two-step protonation, yielding ultimately the *meso*-protonated dication **368.3**. Reaction of **368.1** with sulfuric acid (85%) at 40 °C produced the intermediate **368.4**, which underwent spontaneous oxidation during workup to give **368.5** in 70% yield. The latter species formed two well-defined species, a monocation and a trication, when treated with HBF_4_·Et_2_O. Upon long exposure to tetrafluoroboric acid, **368.5** underwent borylation at the carbonyl oxygen atoms, forming the aromatic BF_2_ derivative **368.6**, which was identified by XRD analysis. These phenanthrene-containing macrocycles acted as trianionic ligands and could be transformed into copper(III) complexes **368.9**–**10**.^707^ The dione **368.5** was subsequently transformed into π-extended phenanthriporphyrins **369.2a**–**h** via condensation with aliphatic and aromatic amines (Scheme 369).^708^

Phenanthrene and naphthalene building blocks was similarly used for the synthesis of phenanthrisapphyrins **369.1a**–**c**^709^ and larger macrocycles^710−712^ (Scheme 369). Diphenanthrioctaphyrins **369.3i** and **369.4i** were obtained as conformationally locked macrocycles in a single macrocyclization reaction.^710^ The UV–vis absorption of **369.3i** exhibited three intense bands at 255, 374, and 458 nm and a weaker and broad absorption extending up to 900 nm, while the low-energy bands for **369.4i** were slightly blue-shifted. Both **369.3i** and **369.4i** adopted a figure-eight shape, differing in the relative alignment of the phenanthrene moieties. DFT calculations showed that the **369.4i** was more stable than **369.3i** by 6.6 kcal mol^−1^. Interconversion between these two species could be induced by hydrogen bond acceptors such as amines. Both **369.3i** and **369.4i** produced the same bisboron(III) complex **369.5i** with a conformation corresponding to that found in **369.4i**. A similar conformational dichotomy was subsequently observed in a di-2,7-naphthihexaphyrin(1.1.1.1.1.1), which formed two conformationally locked stereoisomers **369.6i** and **369.6j**, which could be interconverted using acid–base chemistry under either kinetic or thermodynamic control.^712^

**Scheme 370:**
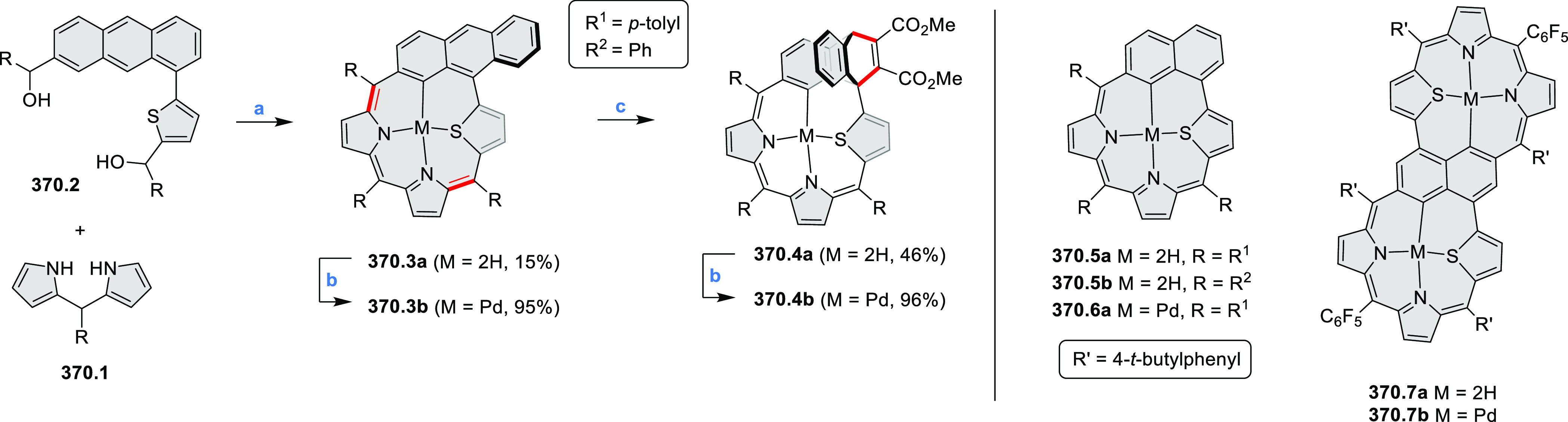
Anthracene- and Naphthalene-Based Carbaporphyrins Reagents and conditions: (a)^713^ (1) BF_3_·OEt_2_, DCM, 1 h, (2) *p*-chloranil, 20 min; (b) Pd(OAc)_2_, DCM/CH_3_CN; (c) dimethyl acetylenedicarboxylate, *o*-DCB, 145 °C.

Anthriporphyrin **370.3a** was synthesized by Cho and co-workers from the anthracene–thiophene dicarbinol **370.2** and dipyrromethane **370.1** (Scheme 370).^713^ The anthracene unit in **370.3a** reacted with dimethyl acetylenedicarboxylate to produce the Diels–Alder phlorin-like adduct **370.4a**. For each macrocycle, the corresponding palladium complex, **370.3b** or **370.4b**, was obtained under standard conditions. Naphthalene-containing analogues **370.5a**,**b**^714^ and the bismacrocyclic systems **370.7a**(715) were obtained in a similar way. In each case, the corresponding palladium(II) complex could be obtained.

**Scheme 371:**
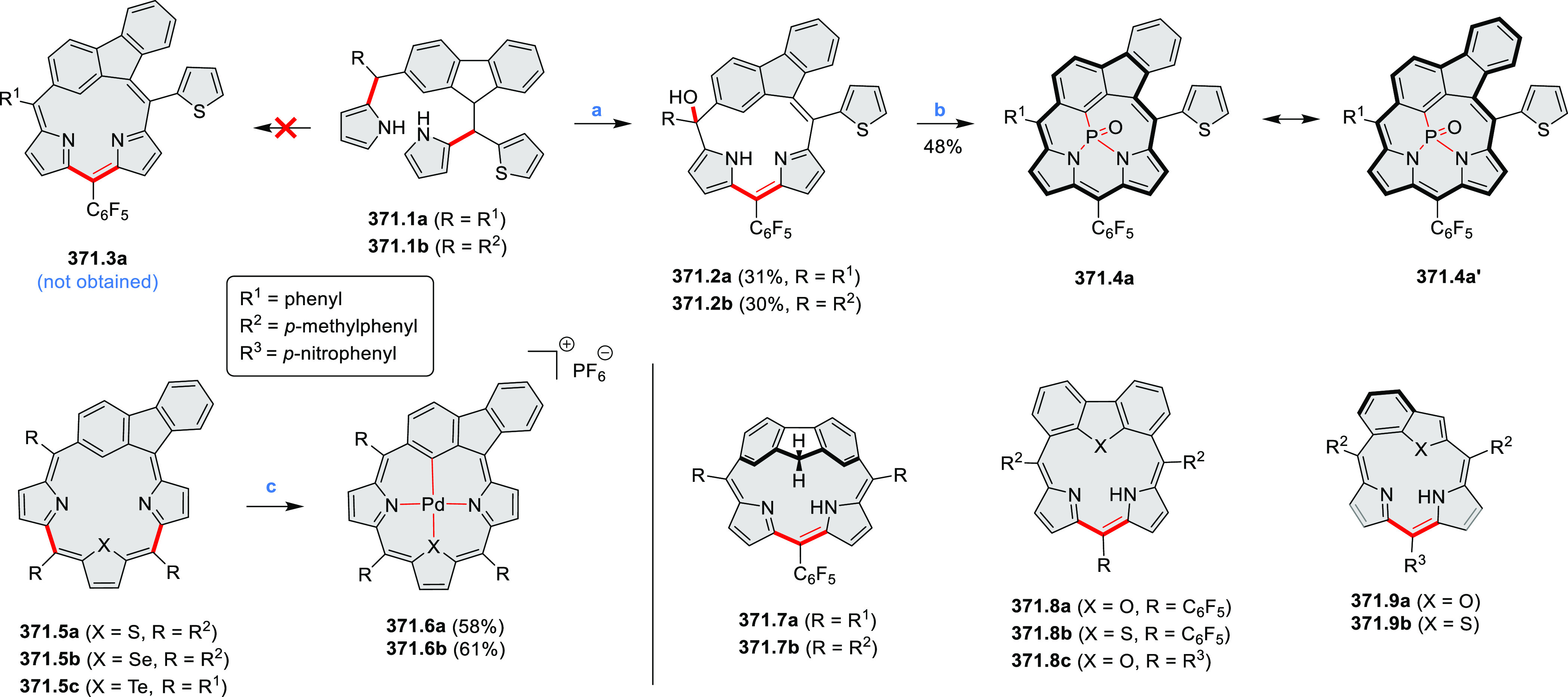
Hybrid Porphyrinoid Macrocycles Reagents and conditions: (a)^716^ (1) BF_3_·OEt_2_, C_6_F_5_CHO, (2) DDQ; (b)^716^ PCl_3_, TEA, toluene, reflux, 1 h; (c)^717^ PdCl_2_, CHCl_3_/CH_3_CN, reflux.

In 2019, Ravikanth et al. reported *meso*-fused carbatriphyrins(2.1.1) based on a fused fluorene motif (Scheme 371).^716^ Condensation of pentafluorobenzaldehyde and the fluorene-based tripyrranes **371.1a**,**b** produced the phlorin-like **371.2a**,**b** rather than the expected fully conjugated **371.3a**. However, the phosphorus(V) complex **371.4a** was obtained in 48% yield by treating the phlorin **371.2a** with PCl_3_ in toluene/triethylamine. **371.4a** showed features consistent with macrocyclic aromaticity, with the dominant 18-electron conjugation pathway. In a similar way, *meso*-fused heterabenziporphyrins **371.5a**–**c** and their palladium complexes **371.6a**,**b** were obtained by the same group.^717^ These systems showed weak absorptions in the NIR range, with a progressive red-shift observed in analogues containing heavier chalcogen heteroatoms. Related designs from the Ravikanth group include the strained fluorenophyrins **371.7a**,**b**,^718^ dibenzofuran/dibenzothiophene-based macrocycles **371.8a**–**c**,^719^ and their contracted benzofuran/benzothiophene analogues **371.9a**,**b**.^720^

**Scheme 372:**
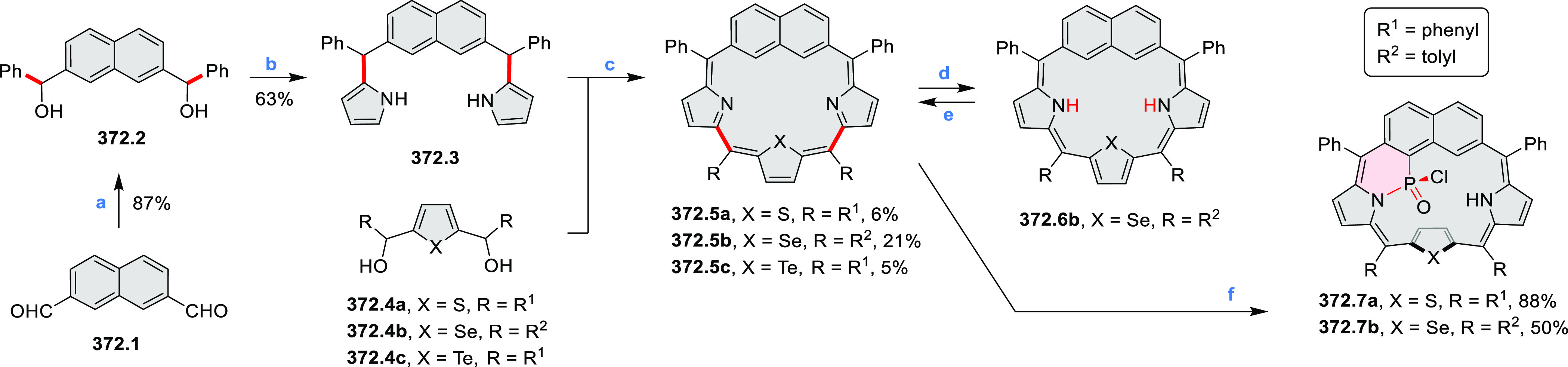
Synthesis of 28-Hetero-2,7-naphthiporphyrins Reagents and conditions: (a)^721^ (1) phenylmagnesium bromide, THF, (2) H_3_O^+^; (b) (1) pyrrole, BF_3_·OEt_2_, CHCl_3_, N_2_, reflux, 48 h, (2) triethylamine; (c) (1) BF_3_·OEt_2_, CHCl_3_, (2) DDQ, 15 min; (d) Zn(Hg), toluene, N_2_; (e) O_2_; (f) PCl_3_, TEA, toluene, reflux, 80 min.

2,7-Naphthiporphyrins **372.5a**–**c** containing various chalcogens in the coordination core were obtained by Szyszko, Latos-Grażyński, and co-workers from the tripyrrane analogue **372.3** (Scheme 372).^721^ These porphyrinoids had a larger coordination cavity than *m*-benziporphyrins and showed an ability to form organophosphorus(V) complexes **372.7a**,**b** when treated with excess phosphorus(III) trichloride in the presence of triethylamine. In these complexes the macrocycle was formally reduced relative to its oxidation state in **372.5a**–**c**. The reduced free base **372.6b** could be reversibly generated from the Se analogue **372.5b**.

#### 7.5.5 Systems with Macrocyclic Subunits

**Chart 34:**
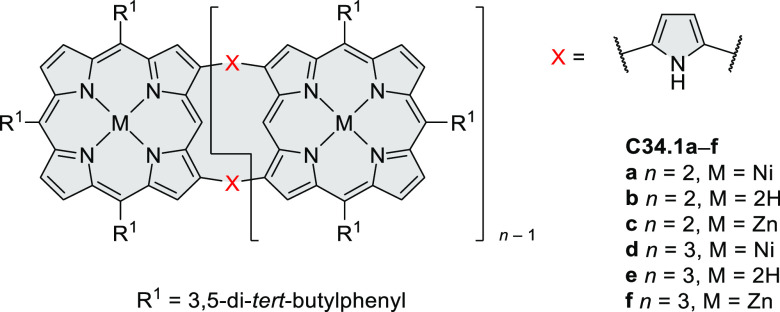
Porphyrin–Pyrrolylene Supermacrocycles

Large supermacrocycles based on porphyrin subunits are typically highly nonplanar and show limited π-conjugation. A relatively high degree of coplanarity between subunits is displayed by 2,5-pyrrolylene-linked cyclic porphyrin dimers **C34.1a**,**b** and trimers **C34.1d**–**c**, obtained by Song et al. via Suzuki–Miyaura coupling of 2,5-diborylpyrrole with appropriate 3,7-dibromoporphyrins (Chart 34).^722^**C34.1a** adopted a bent geometry, in which the pyrrole nitrogens were pointing inward, whereas **C34.1d** showed a more coplanar triangular molecular shape with a reverse alignment of pyrrole bridges. Zinc complexes **C34.1c** and **C34.1f** were also prepared from the corresponding free base oligomers. The UV–vis absorption spectra showed a split Soret band at λ_max_ = 419 and 496 nm for **C34.1c** and at λ_max_ = 416 and 510 nm for **C34.1f** and a long tailing Q-band indicating strong electronic interactions in the cyclic oligomers. The cyclic trimer **C34.1f** displayed ultrafast excitation energy transfer (690 fs) mediated by through-bond electronic interaction via the pyrrole linkage. A similar synthetic approach was further utilized to make several new analogues of cyclic porphyrin oligomers incorporating 6,6″-terpyridylene bridging units.^723^

**Chart 35:**
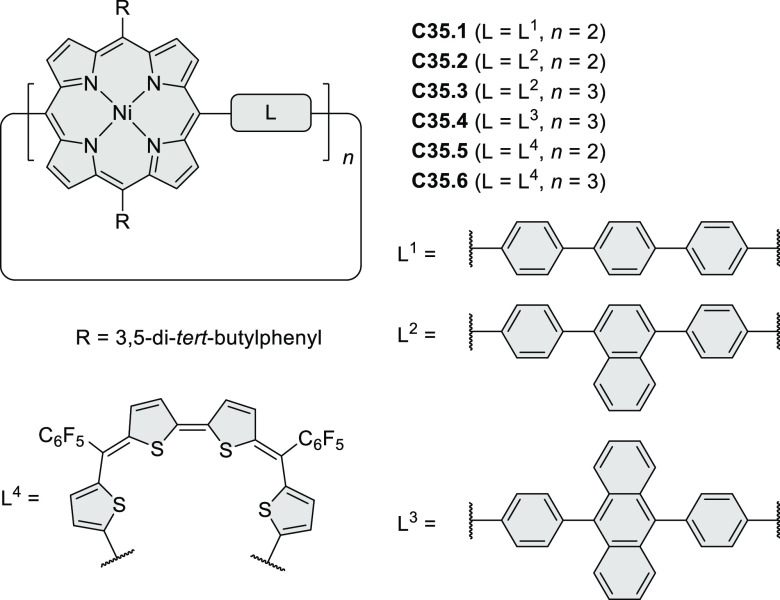
Porphyrin Nanohoops and Nanorings

The synthesis of radially π-conjugated porphyrinylene/phenylene nanohoops **C35.1**–**4** was reported by Meyer, Delius, and co-workers (Chart 35).^724^ The X-ray structure of **C35.1** exhibited an oval-shaped nanohoop structure with an average diameter of ca. 13.2 Å. DFT calculations predicted the ring strain of 54 kcal mol^–1^ for **C35.1**. The UV–vis absorption spectrum of **C35.1** showed bathochromic shifts of Soret and Q bands relative to immediate unstrained precursors. The observed red-shifts were ascribed to the narrowing of the HOMO/LUMO gap with increasing ring strain. For the π-extended analogues **C35.2**–**4**, a slight blue-shift of absorption features was indeed observed. The small nanohoop **C35.1** was found to accommodate C_60_ and C_70_ with binding constants of ca. 3 × 10^8^ M^–1^ and ca. 2 × 10^7^ M^–1^, respectively. The naphthalene-containing nanohoop **C35.3** was found to bind more strongly with C_70_ by a factor of 5.

In 2019, the Wu group reported a template-free synthesis of porphyrin-based macrocycles containing aromatic porphyrins strapped with quinoidal oligothiophene units (Chart 35).^725^ The cyclic porphyrin dimer **C35.5** and trimer **C35.6** were obtained via Yamamoto coupling reaction followed by DDQ oxidation. The solid-state structure of dimer **C35.5** showed a cyclophane-like distorted geometry. Theoretical calculations, including ACID, NICS, and ICSS, indicated the local aromatic character of the porphyrin and nearby thiophene rings. The electrochemical HOMO–LUMO gaps of **C35.5** and **C35.6** were estimated to be 1.52 and 1.35 eV, respectively. The solid-state structure of the dication of **C35.5** exhibited a chair-shaped conformation facilitating p-orbital overlap between the porphyrin, thiophene, and bithiophene units. The dications of **C35.5** and **C35.6** were both found to show global aromaticity with a dominant 54 π and 82 π conjugation pathway, respectively.

**Scheme 373:**
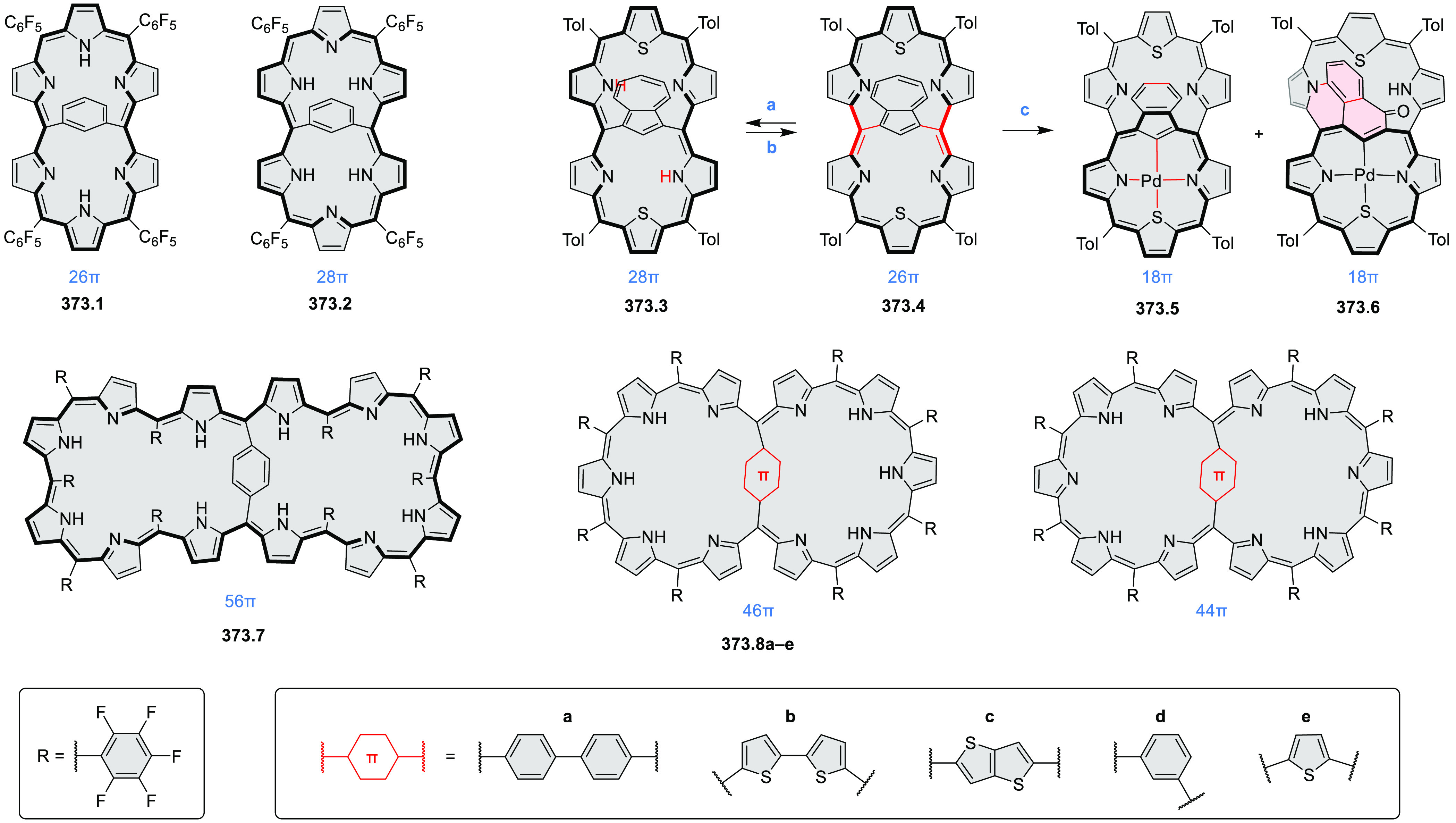
Internally Strapped Porphyrinoid Macrocycles Reagents and conditions: (a)^732^ Zn/H^+^; (b) O_2_; (c) Pd(OAc)_2_, DMF.

Bismacrocyclic structures, constructed by strapping a large porphyrinoid ring with a π-conjugated subunit, can exhibit unusual aromaticity resulting from the coexistence of multiple conjugation pathways (for earlier work, see CR2017, Section 7.5.5). Internally 1,3-phenylene-strapped [26]π- and [28]π-hexaphyrins, **373.1** and **373.2**, exhibit Hückel aromaticity and antiaromaticity, respectively, in their ground states (Scheme 373).^726^ The excited-singlet-state aromaticity of these hexaphyrins was evaluated based on femtosecond transient absorption measurements, and it was found that the aromaticity reversal takes place in the lowest singlet excited states of [26]-and [28]hexaphyrins where **373.1** becomes antiaromatic and **373.2** could be assumed to be aromatic. Subsequently, this excited-state aromaticity reversal study was investigated by using time-resolved IR spectroscopy, and the results were rationalized in terms of the aromaticity-driven change in the molecular structures and IR spectral features.^727^ An even larger system, dodecaphyrin **373.7**, was reported by the Osuka group.^728^ This 56 π-electron macrocycle could be reversibly oxidized to its 54- and 52-electron congeners in a stepwise manner. The 52 π-electron macrocycle was metalated with palladium(II), yielding a quadruply twisted structure. The same group reported a range of bismacrocyclic [46]decaphyrins **373.8a**–**e** strapped with a variety of aromatic subunits.^729,730^ A structurally related three-dimensional cage structure consisting of thiophene units was reported by the Wu group.^731^

An azulene-strapped hexaphyrin **373.4**, reported by the Latos-Grażyński group, is an aromatic 26 π-electron system, and its absorption spectrum showed intense bands at 455 and 565 nm along with a NIR band at 1038 nm.^732^ The chemical reduction of **373.4** with zinc powder in the presence of gaseous HCl produced the highly antiaromatic 28π-electron macrocycle **373.3**. A mononuclear palladium(II) complex obtained from **373.4** upon insertion of palladium(II) in DCM/MeCN retained the aromaticity of the original macrocycle. In DMF, however, palladium insertion produced a range of products, including the two rearranged species **373.5** and **373.6**. Each of these systems showed a predominance of an 18π-electron pathway restricted to the Pd-containing macrocycle.

**Chart 36:**
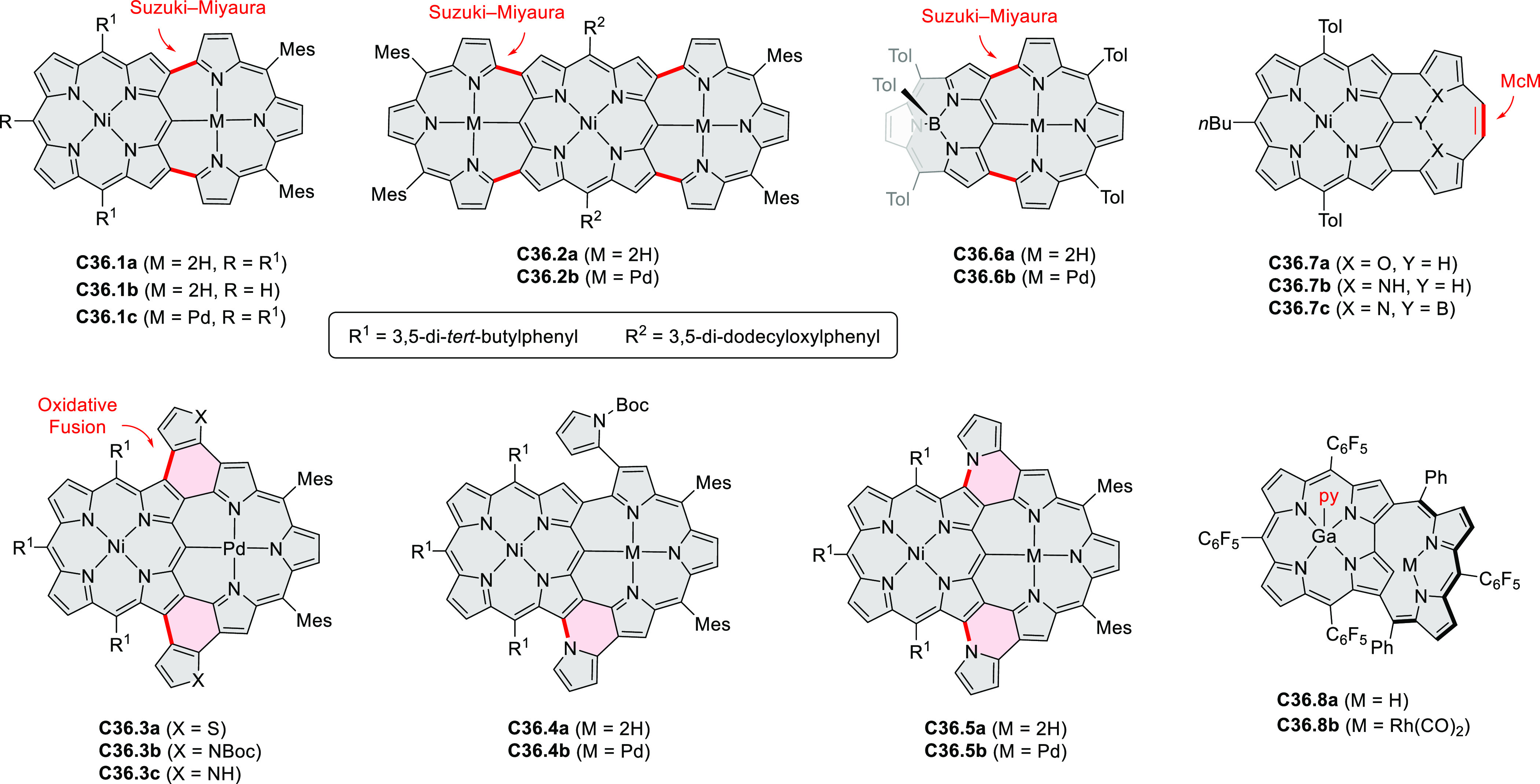
Edge-Sharing π-Extension in Porphyrinoids with Multiple Cavities

“Earring” porphyrins **C36.1**, **C36.2**, and **C36.6**, containing tripyrrin loops fused to the macrocyclic core, were developed by Song and Kim et al. (Chart 36).^733,734^ The synthetic strategy, based on Suzuki–Miyaura coupling, was subsequently extended to develop the heterole-fused derivatives **C36.3**–**5**.^735^ These porphyrinoids possess multiple coordination cavities and can accommodate two or three metal ions per molecule, with meso carbons acting as organometallic donors in the earring loops. The UV–vis–NIR absorption spectra of the metalated complexes **C36.1c**, **C36.2b**, and **C36.6b** were significantly red-shifted, reaching up to 1400–1500 nm in DCM. Even larger red-shifts, reaching up to 2200 nm, were observed for the metal complexes **C36.3a** and **C36.3c**. In line with these observations, the electrochemical HOMO–LUMO gaps of **C36.1c**, **C36.2b**, **C36.3a**, and **C36.3c** were 0.99, 0.88, 0.80, and 0.78 eV, respectively.

A similar design was employed in the synthesis of hybrid macrocycles **C36.7a**,**b**, reported by Pawlicki et al., in which the triphyrin(2.1.1)-like loop was closed via the McMurry reaction.^736^ The loop in **C36.7b** accommodated boron(III), yielding the boron(III) complex **C36.7c** with a direct B–C bond. Boron coordination strongly affected the optical properties of **C36.7c**, which showed a strong absorption at 807 nm. Lateral π-extension of corrole by attaching a dipyrrin loop produced the hybrid bismacrocycle **C36.8a** containing a nonplanar corrorin-like subunit.^737^ The electronic absorption spectra of **C36.8a** and its rhodium complex **C36.8b** showed weak broad Q-like bands in the 500–800 nm region. **C36.8a** was also found to be emissive, with a low quantum yield of 1.3%.

**Scheme 374:**
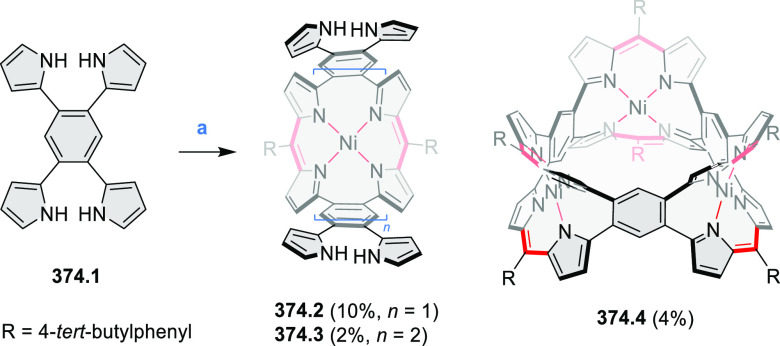
Synthesis of a Porphyrin(2.1.2.1)-Based Nanobelt Reagents and conditions: (a)^738^ (1) RCHO, BF_3_·Et_2_O (5–70 mol %), (2) DDQ, (3) Ni(OAc)_2_.

In 2019, Yamada and co-workers reported the synthesis of porphyrin(2.1.2.1)-based nanobelt **374.4** via condensation reaction of 1,2,4,5-tetra(pyrrol-2-yl)benzene **374.1** and arylaldehyde (Scheme 374).^738^ This condensation reaction with multiple-reactive site containing precursor **374.1** resulted in the formation of different products **374.2**–**4**, depending upon the amount of acid and reaction time. The solid-state structure of **374.4** showed a belt-shaped structure with *C*_3*h*_ symmetry, in which the porphyrin moieties maintained the arch-shaped structures similar to those in **374.2**. The absorption spectrum of **374.4** in DCM showed a maximum at 512 nm, while **374.3** showed red-shifted absorption at 558 nm. Cyclic voltammetry revealed that **374.4** had an ability to both release and accept five electrons, indicating that the shape of the nanobelt stabilized multicationic and multianionic states. The concave-shaped cavity of **374.4** acted as a receptor capable of cooperative binding of two C_60_ molecules, yielding a 1:2 supramolecular complex.

**Scheme 375:**
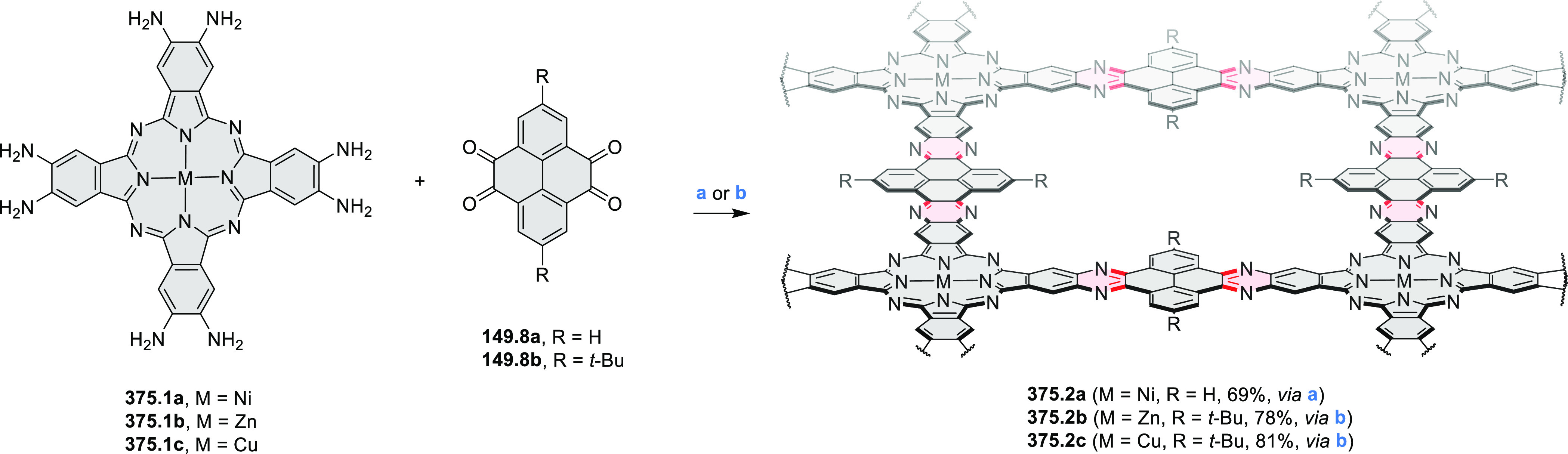
Phthalocyanine-Based Pyrazine-Linked Conjugated Two-Dimensional Covalent Organic Frameworks Reagents and conditions: (a)^739^ dimethylacetamide/*o*-dichlorobenzene (1:1, v/v), H_2_SO_4_, 202 °C, 10 days; (b)^740^ 1-methyl-2-pyrrolidone/mesitylene (2:1, v/v), *p*-TSA (3.5 M), 150 °C, 3 days.

Well-ordered two-dimensional polymers with porous frameworks can be obtained by dione–diamine condensations (cf. Scheme 260, Section 6.1.6, and Scheme 393, Section 7.7.2). This approach was successfully applied for the synthesis of phthalocyanine-based COFs **375.2a**–**c** by Mirica et al. (Scheme 375).^739^ and by Dong and Feng et al.^740^ The synthetic procedure of Mirica et al. involved the reaction of **375.1a** and **149.8a** in a DMAc/*o*-DCB mixture in the presence of sulfuric acid for 10 days, which afforded the desired COF material **375.2a**. This synthesis was also achieved by using microwave heating, which significantly reduced the reaction time, albeit at the expense of slightly diminished crystallinity. The structure of **375.2a** featured square apertures with a side length of 2.2 nm and showed high chemical and thermal stability. The intrinsic bulk conductivity of the **375.2a** reached 2.51 × 10^–3^ S/m and increased by 3 orders of magnitude with iodine doping. Upon integration into chemiresistive devices, this conductive COF showed excellent responses to various reducing and oxidizing gases, including NH_3_, H_2_S, NO, and NO_2_ with limits of detection at the ppb level. The synthetic procedure described by Dong and Feng et al.^740^ involved condensation of the reactants with *p*-TSA as the catalyst in a heated NMP/mesitylene mixture. The resultant compounds **375.2b**–**c** were found to be p-type semiconductors, both with a band gap of ∼1.2 eV and relatively similar electronic properties. It was subsequently found that **375.2b** exhibited enhanced conductivity upon molecular iodine doping, with the carrier mobility reaching ∼22 cm^2^ V^–1^ s^–1^.^741^

### 7.6 Internally Fused Porphyrinoids

**Scheme 376:**
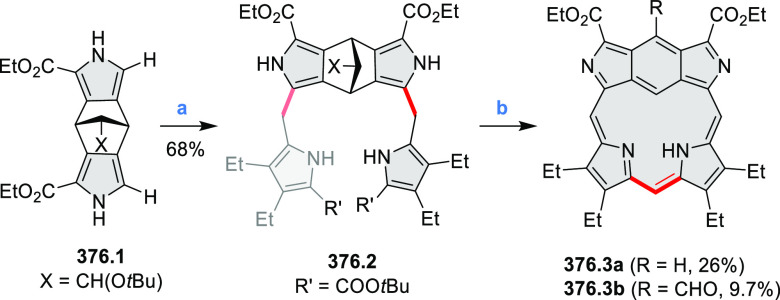
Benzene-Fused Doubly N*-*Confused Porphyrins Reagents and conditions: (a)^742^*tert*-butyl 5-acetoxymethyl-3,4-diethylpyrrole-2-carboxylate, catalytic *p*-TsOH·H_2_O, AcOH, rt, 3 h; (b) TFA, N_2_, CH_2_(OMe)_2_, DCM, *p*-chloranil.

Doubly N-confused porphyrins containing an internally fused benzene ring were reported in 2020 by Uno, Mack, and co-workers (Scheme 376).^742^ When the bicyclo-fused bis(dipyrromethane) **376.2** was condensed with methylal under acidic conditions, a mixture of **376.3a** and **376.3b** was obtained in moderate yields. The proposed mechanism involved acid-induced skeletal opening of the bicyclo[2.2.1]heptadiene ring to give an intermediate with a formyl group attached to the sp^3^ carbon. ^1^H NMR spectra, X-ray structures, and theoretical calculations indicated the coexistence of both macrocyclic 18π and local 6π aromaticity in these systems, in spite of the presence of the formally antiaromatic 2,6-diaza-*s*-indacene subunit in the macrocycle.

**Scheme 377:**
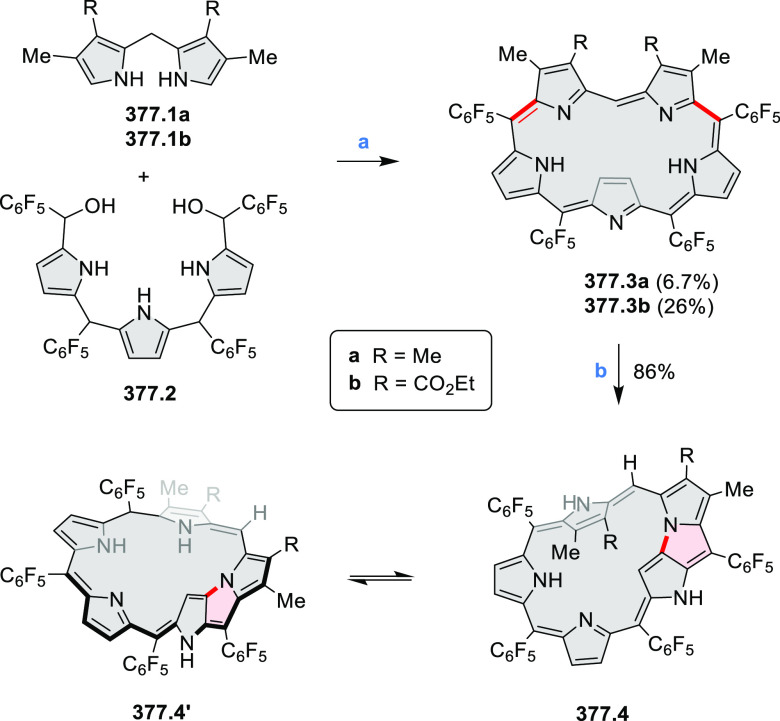
N*-*Fused Pentaphyrins and Their Reactivity Reagents and conditions: (a)^743^ (1) *p*-TsOH·H_2_O, DCM, N_2_, 90 min, (2) DDQ, 1 h; (b) CH_3_OH, 25 °C, 4 h.

In 2017, Yoneda and Neya reported the synthesis of [22]pentaphyrins **377.3a**,**b** containing β,β-disubstituted pyrroles flanking a single free meso position (Scheme 377).^743^ The substitution of an electron-withdrawing group at the periphery in **377.3b** suppressed its oxidative decomposition relative to **377.3a**. **377.3a** and **377.3b** showed a roughly planar nonfused macrocyclic structure in the solid state and displayed strong 22 π-electron Hückel aromaticity. In methanol solution, compound **377.3b** underwent an N-fusion reaction to give N-fused pentaphyrin **377.4**, which exhibited solvent-dependent polymorphism, yielding either Hückel- or Möbius-type conformations in the solid state. Spectroscopic and theoretical evidence suggested that the Hückel conformer **377.4** was nonaromatic, whereas the Möbius conformer **377.4′** had a Möbius aromatic character.

**Scheme 378:**
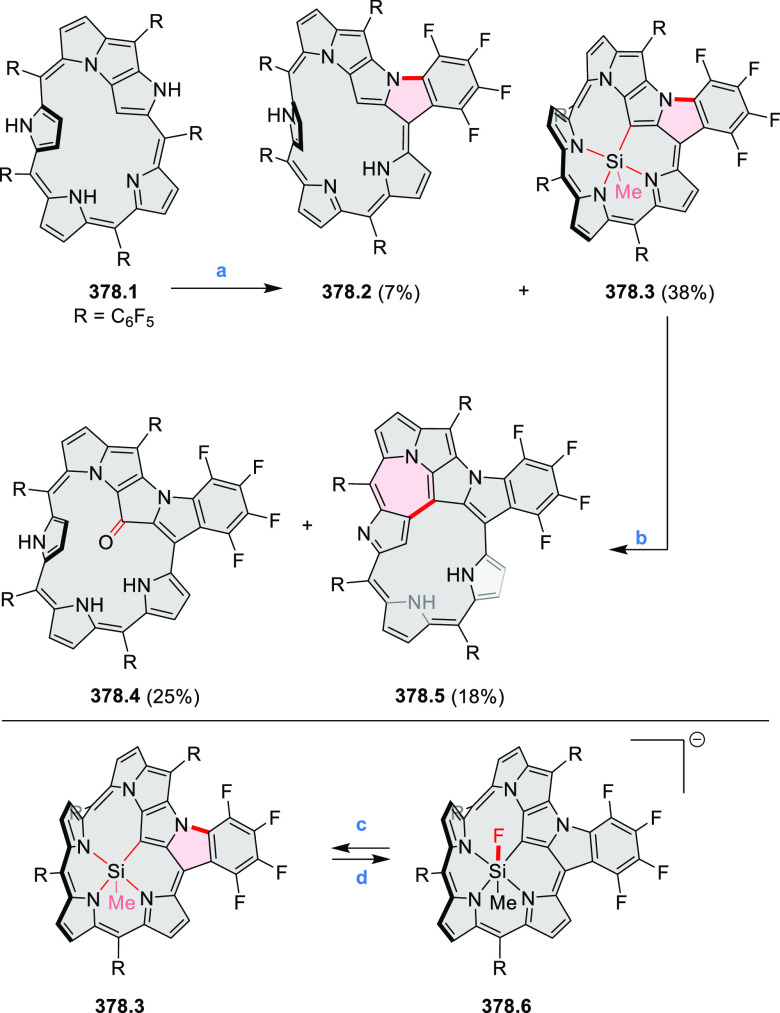
Synthesis of Doubly N-Fused [24]Pentaphyrin and Its Reactivity Reagents and conditions: (a)^744^ MeSiCl_3_ (100 equiv), DIPEA (300 equiv), DMF, 80 °C, 14 h; (b) KHCO_3_ (4 equiv), excess aq. H_2_O_2_, THF, rt, 45 min; (c) TBAF, CD_2_Cl_2_; (d) H_3_O^+^.

Osuka et al. reported the synthesis of doubly N-fused [24]pentaphyrin **378.2** and its silicon complex **378.3**, which were obtained in 6% and 38% yield, respectively, upon treating the singly fused **378.1** with MeSiCl_3_ and *N*,*N*-diisopropylethylamine in DMF at 80 °C (Scheme 378).^744^ The X-ray crystallographic analyses of these doubly fused structures revealed twisted geometries with a Möbius structure exhibiting effective macrocyclic π-conjugation. Upon addition of a fluoride ion, the silicon complex **378.3** afforded a monoanionic hexacoordinate Si^IV^ complex **378.6**, which could be converted back into **378.3** when treated with acid. Additionally, **378.3** was found to undergo Tamao–Fleming oxidation, which resulted in the formation of the nonaromatic β-keto doubly N-fused pentaphyrin **378.4** and Hückel-antiaromatic triply fused [24]pentaphyrin **378.5** in moderate yields.

**Scheme 379:**
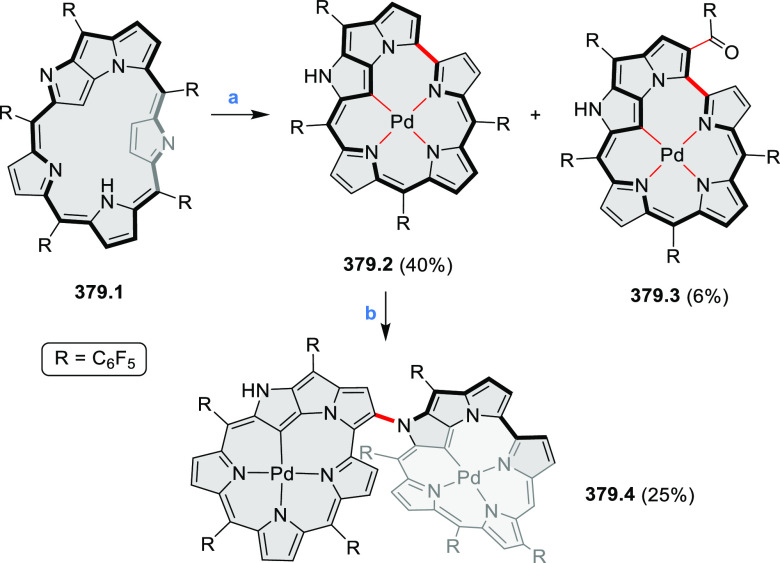
Palladium Insertion-Triggered Reactivity of N*-*Fused Pentaphyrin Reagents and conditions: (a)^745^ Pd(CF_3_CO_2_)_2_ (5 equiv), THF, reflux; (b) DDQ (1.2 equiv), Sc(OTf)_3_ (0.7 equiv), toluene, rt.

Metalation of the N-fused [22]pentaphyrin(1.1.1.1.1) **379.1** with palladium(II) trifluoroacetate in refluxing THF under aerobic conditions afforded two complexes of N-fused [22]pentaphyrin(1.1.1.1.0) **379.2** and **379.3**, formed by extrusion of one of the meso carbons (Scheme 379).^745^ X-ray crystallographic analyses revealed fairly planar structures for both species, and the structural features indicated that the carbonyl moiety in **379.3** was conjugated to the sapphyrin core. Oxidation of **379.2** with DDQ and Sc(OTf)_3_ led to the formation of the heterodimer **379.4**, in which two sapphyrin units were linked via a C–N bond. The UV–vis absorption spectrum of **379.4** in DCM exhibited Soret-like bands at 457 and 521 nm and Q-like bands at 638, 697, and 799 nm. Cyclic voltammograms showed two pseudoreversible oxidations and two reversible reduction waves for **379.4** with an electrochemical HOMO–LUMO gap of 1.63 eV.

**Scheme 380:**
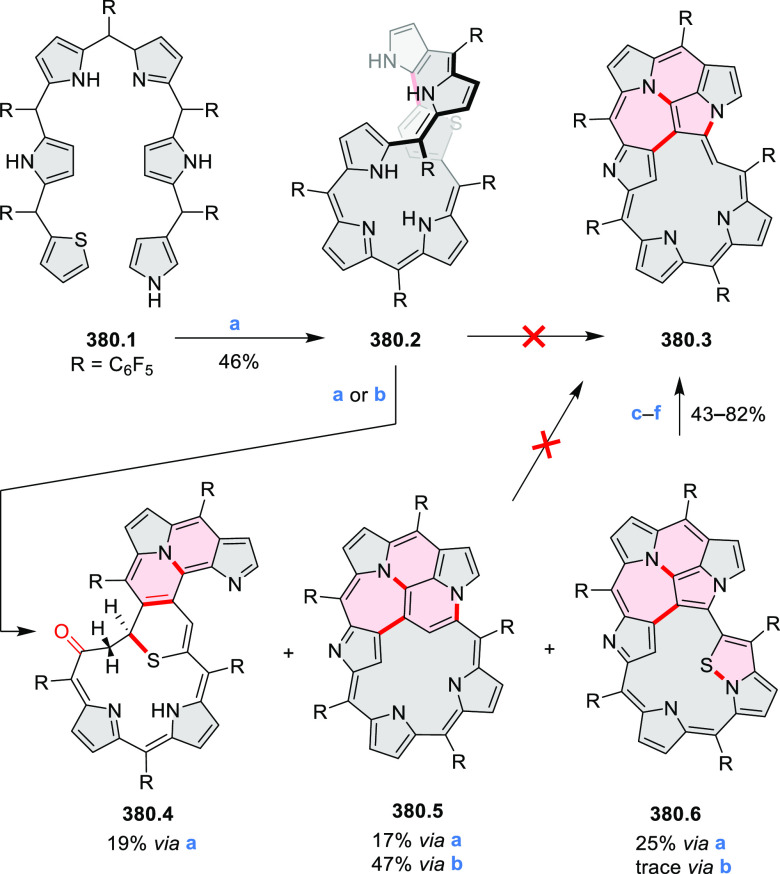
Oxidative Reactivity and Ring Fusion in Twisted Thianorhexaphyrin Reagents and conditions: (a)^746^ DDQ, DCM; (b) *p*-chloranil, DCM; (c) O_2_, Et_3_N/MeCN; (d) H_2_O_2_/AcOH, THF; (e) *m*-CPBA, DCM; (f) PPh_3_, DCM.

In 2019, Furuta and Xie et al. reported the synthesis of a thianorhexaphyrin **380.2**, which showed unprecedented reactivity with oxidants accompanied by multiple N–C fusions and skeletally rearranged products (Scheme 380).^746^**380.2** was prepared in 46% yield from **380.1** upon reacting it with 4 equiv of DDQ under high dilution conditions. Upon further addition of DDQ (2 equiv), three different multiply fused products were obtained, including the extensively fused **380.5**–**6**. The unusual pyrrolo[1,2]isothiazole moiety in **380.6** could be desulfurized under a variety of conditions, to produce pyrrolizine species **380.3**. The formally 26 π-electron macrocycle **380.2** had a nonaromatic character, while **380.3**, **380.5**, and **380.6** showed apparent globally aromatic features according to calculations.

**Scheme 381:**
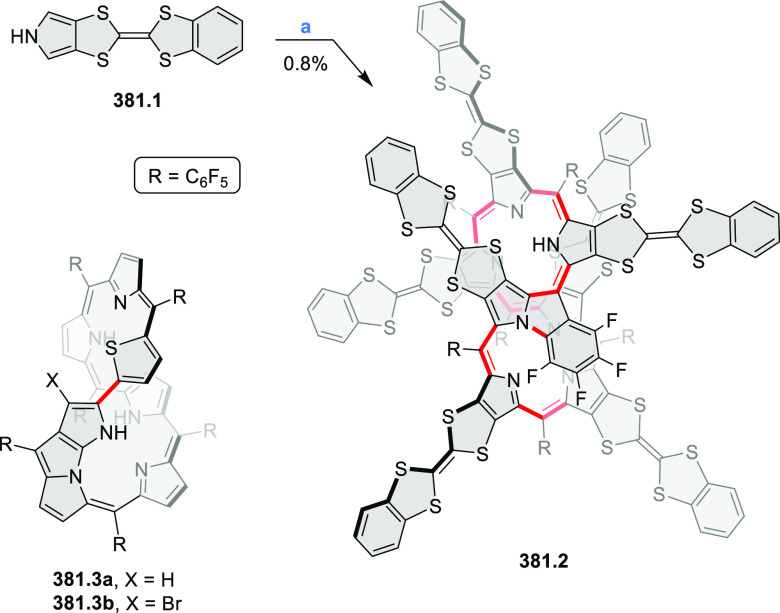
N-Fused Heptaphyrins Reagents and conditions: (a)^747^ (1) pentafluorobenzaldehyde, BF_3_·H_2_O, DCM, 3 h, rt, (2) DDQ, 1 h, rt.

The N-fused heptaphyrin **381.2** was isolated as a byproduct in a macrocyclization reaction between the benzo-tetrathiafulvalene-fused pyrrole **381.1** and pentafluorobenzaldehyde (Scheme 381).^747^ The X-ray crystal structure revealed that the central macrocyclic core exists in a highly strained, nonplanar figure-of-eight conformation. The electronic spectrum of **381.2** extended up to 1600 nm, revealing the intrinsic intramolecular charge transfer (CT) character of the chromophore. A fused thiaheptaphyrin(1.1.1.1.1.1.0) **381.3a**, structurally related to **380.2**, was obtained by sequential dehydrogenation of a heptapyrromethane analogue (Scheme 381).^748^ In the solid state, the brominated derivative **381.3b** was found to contain a pyrrolo[3,2-*b*]pyrrolizine fragment and to adopt a figure-eight-like conformation. The UV–vis–NIR spectrum of **381.3a** in DCM displayed absorption maxima at 428, 653, and 808 nm and a broad low-energy band at 950–1350 nm.

**Scheme 382:**
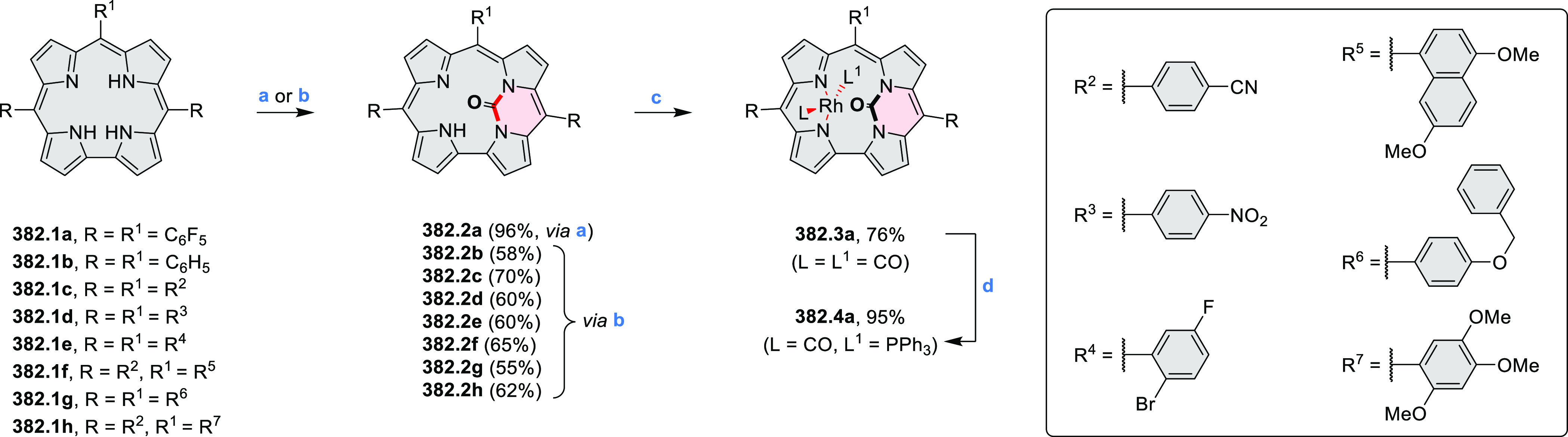
Synthesis of *N*^21^,*N*^22^-Carbamide-corroles Reagents and conditions: (a)^749^ COCl_2_, toluene, 0 °C (10 min), then pyridine, toluene, N_2_, reflux (15 min); (b)^751^ (NH_4_)_2_CO_3_ (excess), CHCl_2_, pyridine, 110 °C, 3 h; (c)^750^ Rh_2_(CO)_4_Cl_2_, LiHMDS, benzene, reflux; (d)^750^**382.3a**, PPh_3_, benzene.

An internally bridged, chiral corrole **382.2a** was obtained by Goldberg and Gross et al. upon treating **382.1a** with phosgene (Scheme 382).^749^ The XRD analysis showed that the corrole ring in **382.2a** was severely twisted to minimize steric repulsion between the carbonyl group and the *N*-pyrrole proton. The same group later reported the rhodium(I) complexes **382.3a** and **382.4a**, which were utilized as cyclopropanation catalysts, with **382.4a** showing a higher catalytic reactivity than **382.3a**.^750^ In 2017, Biswal, Kar, and co-workers reported modified synthetic protocol to synthesize further *N*^21^,*N*^22^-carbamide-corrole derivatives **382.2b**–**h**.^751^ The latter compounds were obtained by treating **382.1b**–**h** with ammonium carbonate in the presence of pyridine, which was found to act as both a solvent and a weak base.

**Scheme 383:**
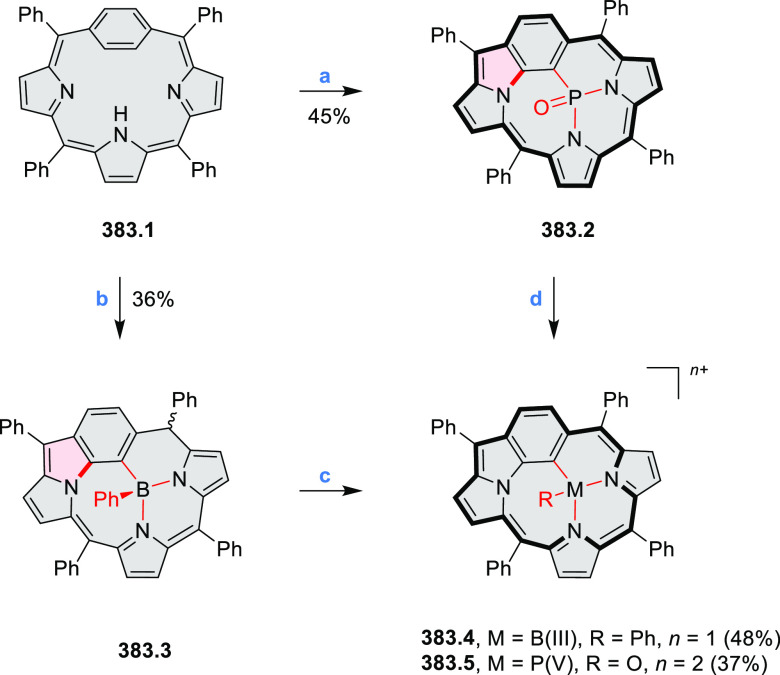
Synthesis of Boron(III) and Phosphorus(V) N-Fused *p*-Benziporphyrin Reagents and conditions: (a)^752^ PCl_3_, Et_3_N, H_2_O, toluene, 3 h; (b) PhBCl_2_, Et_3_N, toluene, 5 h; (c) Br_2_, CHCl_3_; (d) Br_2_, DCM, or DDQ/MeOH

Internal N-fusion was induced inside *para*-benziporphyrin upon insertion of boron(III) and phosphorus(V).^752^ Reactions of **383.1** with PCl_3_ or PhBCl_2_ in a boiling toluene/Et_3_N mixture afforded, respectively, **383.2** and two stereoisomers of **383.3** (Scheme 383). Protonation of **383.2** occurred at one of the meso positions, also leading to stereoisomerism. Further, the oxidation of **383.2** and **383.3** with an excess of bromine in strictly anhydrous conditions led to two-electron oxidation and the formation of aromatic complexes **383.4** and **383.5**, respectively.

### 7.7 *peri-*Fused Cyclophanes

#### 7.7.1 Pyridine-Fused Cyclophanes

**Scheme 384:**
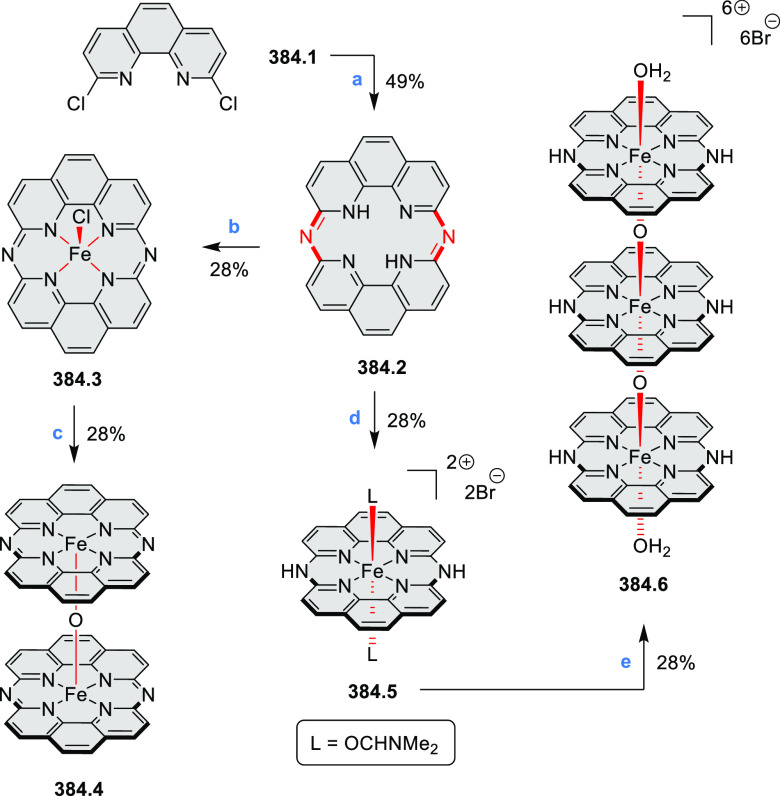
Bisphenanthroline *peri-*Fused Cyclophanes and Their Complexes Reagents and conditions: (a)^753^ NH_3_, 280–300 °C, 36 h, (b)^753^ FeCl_3_, N(*n*-Bu)_3_, DMF, 170 °C, 36 h, (c)^753^ EtOH, 48 h, Soxhlet, (d)^754^ FeBr_2_, DMF; (e)^754^ H_2_O under air, rt.

Coordination chemistry of the previously reported bisphenanthroline macrocycle **384.2** (cf. CR2017, Section 7.7.1) was explored by Surendranath et al. (Scheme 384).^753^ The poorly soluble iron(III) complex **384.3**, obtained by metalation with anhydrous FeCl_3_, contained an apical chloride ligand and retained the deprotonated peripheral nitrogens characteristic of the free-base ligand. **384.3** and the corresponding μ-oxo complex, **384.4**, were found to be good models of Fe–N–C electrocatalysts. In particular, the electrochemical and catalytic properties of **384.3** showed a high selectivity for the four-electron reduction of O_2_ in both acidic and alkaline media. In parallel work, Nabae et al.^754^ reported a related blue-colored complex **384.5**, which was proposed to contain iron(II), a peripherally protonated bisphenanthroline ligand, and two axially coordinated DMF molecules. Upon heating this product in water at 85 °C, the triple-decker complex **384.6** was isolated and characterized crystallographically. The fourteen-membered macrocycle in **384.6** had relatively strong Fe–N bonds with an average bond distance of 1.90 Å, which is markedly shorter than that in porphyrin complexes (2.0 Å).

**Scheme 385:**
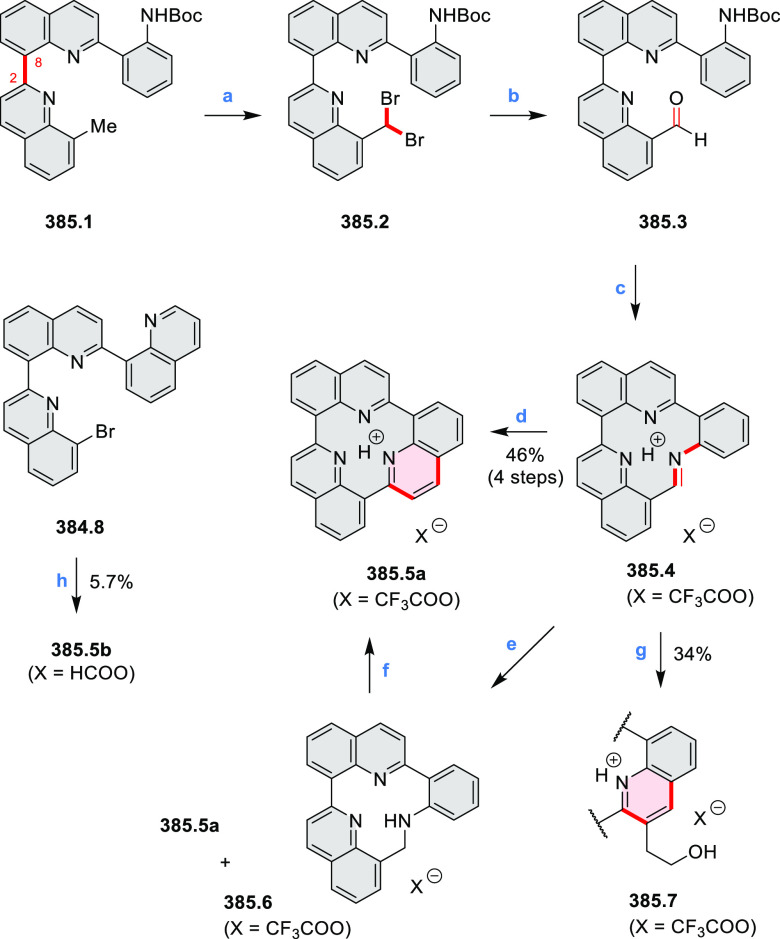
Shape-Persistent Macrocycles Containing *peri*-Condensed Pyridine Rings Reagents and conditions: (a)^755^ AIBN, NBS (2.5 equiv), CCl_4_, 77 °C, 5 h; (b) Na_2_CO_3_, 1,4-dioxane, 80 °C, 14 h; (c) TFA (10 equiv), dichloroethane, 70 °C, 18 h; (d) *n*-butyl vinyl ether (10 equiv), MeCN, 45 °C, 25 h; (e) *n*-butyl vinyl ether (10 equiv), degassed CH_3_CN, rt; (f) air; (g) 2,3-dihydrofuran (50 equiv), MeOH, 25 °C, 24 h; (h) *n*BuLi, THF, −78 to 25 °C, 16 h.

In 2019, Kumagai et al. reported the synthesis of “TriQuinoline”, a macrocycle consisting of three quinoline units concatenated at the 2- and 8-positions in a head-to-tail fashion (Scheme 385).^755^ In an initial synthetic approach, **385.8** was employed to promote nucleophilic cyclization, followed by spontaneous oxidative aromatization which afforded the TriQuinoline **385.5b** in rather poor yield (5.7%). In an alternative strategy, 2-fold benzylic radical bromination on **385.1** resulted in the dibromide product **385.2**, which upon exposure to aqueous Na_2_CO_3_ at 80 °C afforded the aldehyde **385.3**. In the subsequent step, a key intermediate product was obtained upon removal of the *N-*Boc group with trifluoroacetic acid, which afforded the TFA salt of cyclic imine **385.4**. The final step involved an unusual uninterrupted Povarov reaction, which furnished the desired **385.5a**. **385.5a** was found to be an efficient DNA intercalator for the inhibition of topoisomerase I activity. **385.5a** formed supramolecular binary and ternary complexes with [12]cycloparaphenylene (CPP) and coronene, stabilized by π stacking and CH···π interactions.

**Scheme 386:**
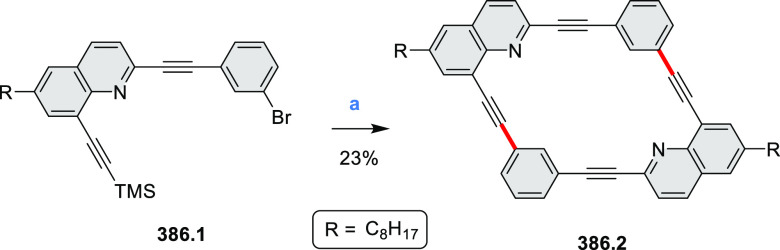
Shape-Persistent Macrocycles Containing *peri*-Condensed Pyridine Rings Reagents and conditions: (a)^756^ (1) KOH, MeOH/THF (2:1 v/v), rt, 24 h, (2) Pd(PPh_3_)_4_, CuI, NEt_3_/THF (1:2, v/v), 50 °C, 24 h.

Synthesis of a shape-persistent *m*-diethynylene-phenylene bridged macrocycle incorporating two quinoline moieties was described by Shimasaki and Shibata et al.^756^**386.2** was obtained in 23% yield from **386.1** by deprotection of the TMS group with KOH followed by cyclodimerization in a high-dilution Sonogashira cross-coupling reaction (Scheme 386). X-ray crystal structure revealed that **386.2** adopts an almost planar *C*_2*h*_ symmetrical parallelogram structure. Fluorescence of **386.2** had a maximum at 420 nm with a quantum yield of 47%.

**Chart 37:**
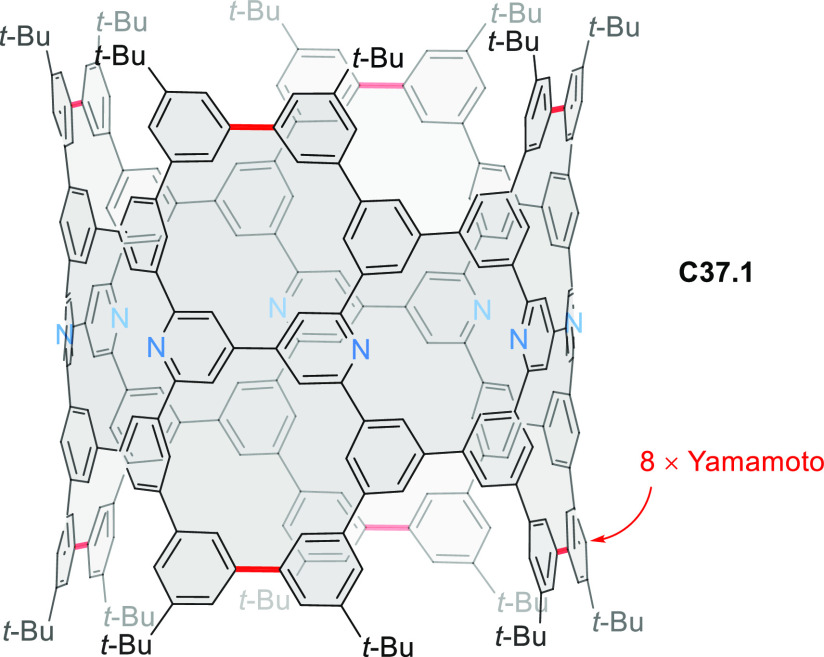
Nitrogen-Doped Phenine Nanotubes

In 2020, Isobe and co-workers reported the bottom-up synthesis of a nitrogen-doped phenine nanotube with a discrete, rigid structure by combining eight 2,4,6-trisubstituted pyridine units with thirty-two 1,3,5-trisubstituted benzene units (Chart 37).^757^ The target nanotube fragment **C37.1** was obtained by forming eight aryl–aryl bonds along the edges of the tube in a Yamamoto-type coupling reaction. **C37.1** has a porous structure based on the graphitic lattice of (12,12)-carbon nanotube with a length index of *t*_f_ = 7.0. The UV–vis absorption spectrum of N-doped macromolecule showed absorptions reaching up to 378 nm, corresponding to an optical gap of 3.28 eV, and the fluorescence quantum yield of ca. 16%.

#### 7.7.2 Pyrrole-Fused and Related Cyclophanes

**Scheme 387:**
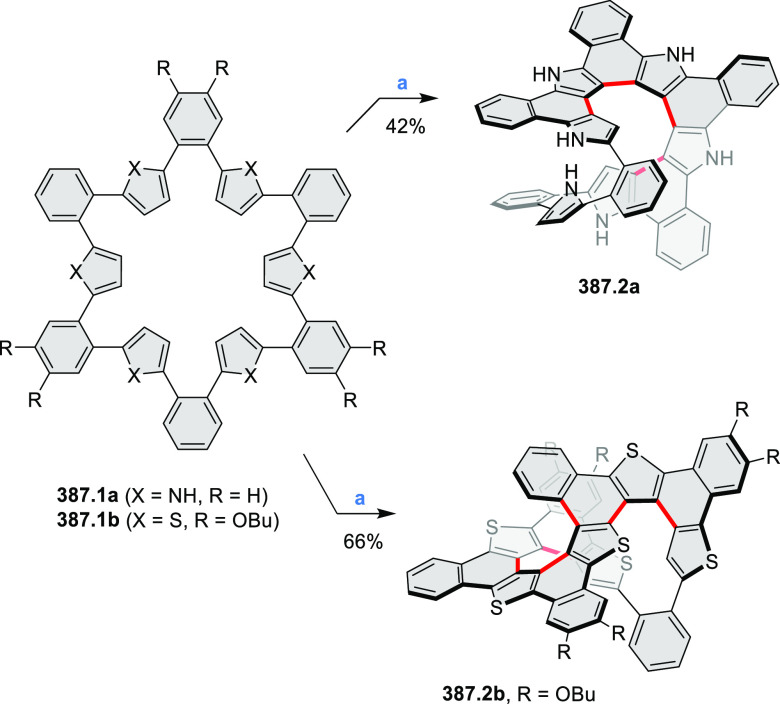
Oxidative Fusion Reactions of *ortho*-Phenylene-Bridged Cyclic Hexapyrrole and Hexathiophene Reagents and conditions: (a)^758^ DDQ, Sc(OTf)_3_, toluene, reflux.

Fold-in syntheses of macrocyclic tetrabenzopentaaza[9]helicene and pentabenzohexathia[9]/[5]helicene were reported in 2017 by Tanaka, Kim, and Osuka et al.^758^ Oxidative fusion reaction of the 1,2-phenylene-bridged cyclic hexapyrrole **387.1a** with 5 equiv of DDQ and Sc(OTf)_3_ gave a pale-yellow compound **387.2a** in 4% yield (Scheme 387). Regioselectivity in this oxidation was high, producing only the 4-fold fusion product **387.2a** even upon increasing temperature or increasing oxidant equivalents. Similarly, by oxidation of **387.1b** with 8 equiv of DDQ and Sc(OTf)_3_, a thiophene-based system **387.2b** was obtained in 66% yield. On the basis of X-ray crystallographic analysis and spectroscopic studies, the structure was identified as the double helicene product **387.2b** resulting from a 5-fold oxidative fusion along with an unexpected 1,2-aryl shift. **387.2a** exhibited well-defined emission at 427 and 453 nm with a high fluorescence quantum yield, Φ_f_ = 0.31. In contrast, **387.2b** emitted very weakly with Φ_f_ = 0.018. Both macrocycles were chiral and could be separated into enantiomers.

**Scheme 388:**
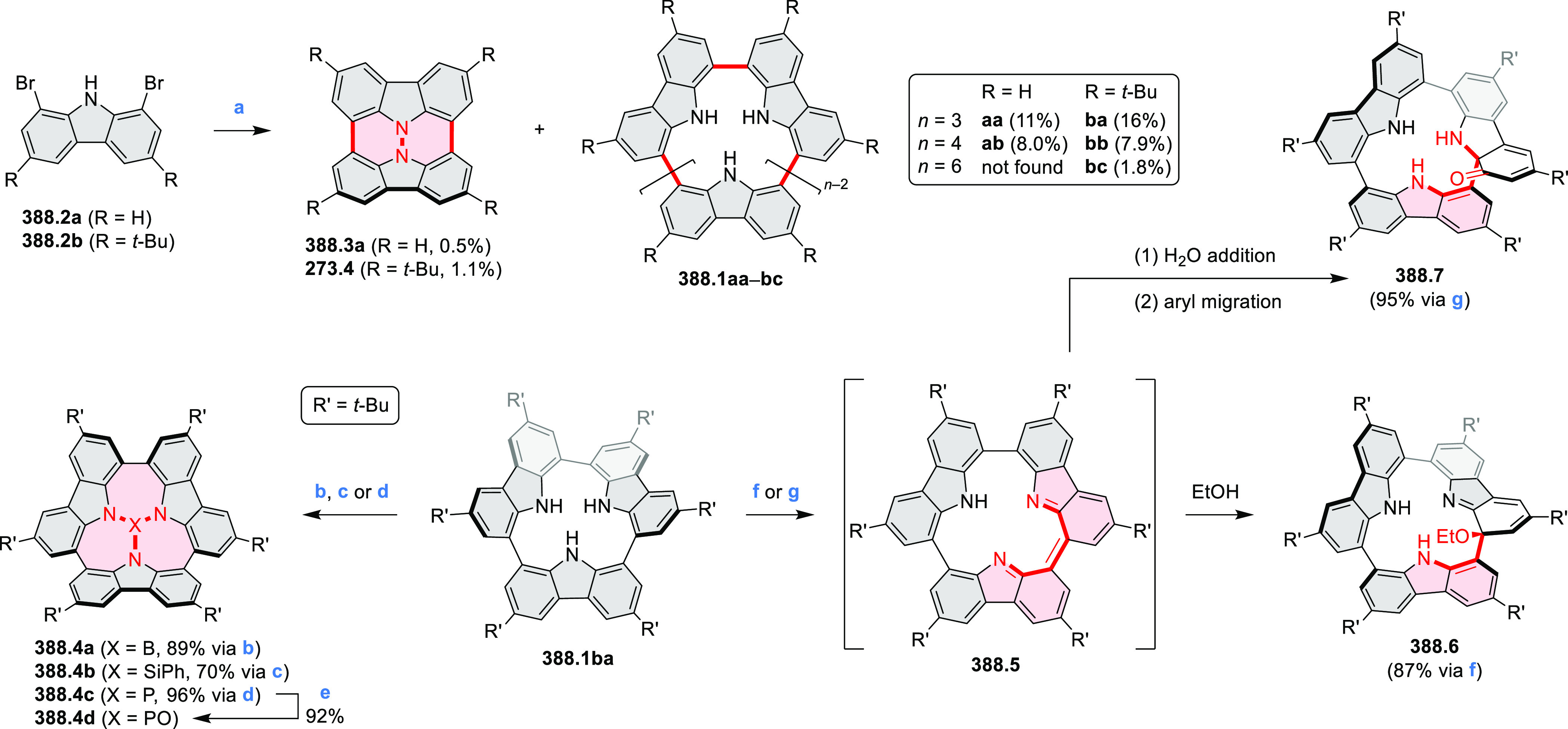
Synthesis and Oxidation of [*n*]Cyclo-1,8-carbazolylenes Reagents and conditions: (a)^759^ slow addition of **388.2** over 1 h to Ni(cod)_2_ (250 mol %), cod (250 mol %), 2,2′-bipyridyl (150 mol %), THF, 80 °C, then, 80 °C, 1 h; (b) *n*-BuLi, toluene, −80 °C, then 0 °C, 30 min, (2) BBr_3_, −80 °C, then rt, 3 h; (c) PhSiCl_3_, Et_3_N, 1,2-DCE, 90 °C, 12 h; (d) PCl_3_, Et_3_N, 1,2-DCE, 90 °C, 16 h; (e) *m*-CPBA, DCM, 30 min; (f)^760^ DDQ (1.5 equiv), CHCl_3_ stabilized by 0.3–1.0% EtOH, rt; (g) DDQ (2.0 equiv), EtOH-free CHCl_3_, rt, 95%.

In 2017, Higashibayashi et al. reported the isolation of the members in the [*n*]cyclo-1,8-carbozolylene family **388.1aa**–**bc** (Scheme 388).^759^ In their procedure, each of the 2,8-dibromo carbazole derivatives **388.2a**,**b** was slowly added to a THF solution of Ni(cod)_2_, cod, and 2,2′-bipyridyl at 80 °C. The reductive cyclooligomerization of **388.2a** produced the macrocyclic trimer (11%) and tetramer (8.0%), whereas **388.2b** yielded the macrocyclic trimer, tetramer, and hexamer (16%, 7.9%, and 1.8%, respectively). The absence of the cyclic hexamer in the former case was ascribed to the lack of solubilizing substituents. The corresponding hydrazinobuckybowls **388.3a** and **273.4** were isolated in each case as a minor side product. [3]Cyclo-1,8-carbazolylene **388.1ba** was further used as a tridentate ligand for boron, silicon, and phosphorus, affording the corresponding flake-shaped complexes **388.4a**–**d**. In 2017, the same group attempted to prepare the macrocycle **388.5** via the DDQ-mediated dehydrogenation of the [3]cyclo-1,8-carbozolylene **388.1ba** (Scheme 388).^760^ When ethanol-stabilized chloroform was used as the solvent, the α-ethoxyimine structure **388.6** (87% yield) was isolated. In contrast, the use of ethanol-free chloroform gave the ring-rearranged ketone **388.7** (95% yield), which was shown by DFT calculations to be 6.6 kcal mol^−1^ more stable than the presumed α-hydroxyimine intermediate. These dearomatized products were proposed to arise from the desired **388.5**, which was too unstable to be isolated.

**Chart 38:**
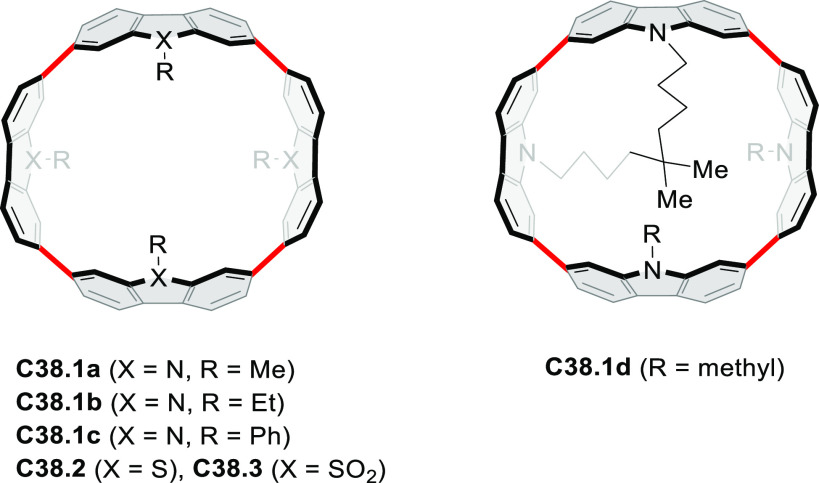
[4]Cyclo(2,7)carbazoles and Related Systems

In 2016, Suzuki et al. described the synthesis of three N-substituted tetracyclo(2,7-carbazoles) to investigate macrocyclic ring currents inside nanohoops.^761^**C38.1a**, **C38.1c**, and **C38.1d** were obtained using a platinum-mediated cyclooligomerization strategy (Chart 38). The X-ray crystal structure of **C38.1a** revealed the alternant conformation of the macrocycle, which was also predicted to be most stable by DFT calculations. **C38.1d** contained a 5,5-dimethylnonane bridge between two neighboring *anti*-carbazoles, which acted as a covalently bound probe for determination of NMR shielding inside the macrocycle. Accordingly, the methyl groups of the bridge were found to resonate at −2.70 ppm. Cyclocarbazole **C38.1b** was also employed in an OFET device by Poriel et al. and was found to produce a hole mobility μ_FE_ of 1.1 × 10^–5^ cm^2^ V^–1^ s^–1^.^762^ Analogous tetramers of 3,7-dibenzo[*b*,*d*]thiophene (**C38.2**) and 3,7-dibenzo[*b*,*d*]thiophene-5,5-dioxide (**C38.3**) were similarly obtained by Yamago et al.^763^**C38.3** was characterized by the single-crystal X-ray structure which revealed the *anti*-orientations with respect to the adjacent DBTO unit. The fluorescence spectra of **C38.2** and **C38.3** showed emission maxima at 510 nm (Φ_F_ = 0.21) and at 429 and 529 nm (Φ_f_ = 0.41), respectively.

**Scheme 389:**
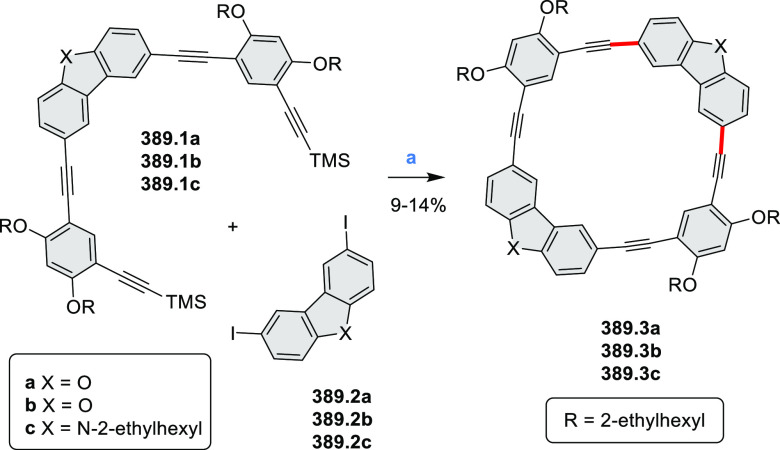
*peri-*Fused Dibenzofuran-, Dibenzothiophene-, and Carbazole-acetylene Macrocycles Reagents and conditions: (a)^764^ (1) TBAF, THF, rt, 1 h, (2) **389.2a**, **389.2b**, or **389.2c**, Pd(PPh_3_)_4_, CuI, NEt_3_/THF (1:1 v/v), rt, 24 h.

In 2018, Shimasaki and Shibata reported the synthesis of fully conjugated macrocycles composed of two *m*-diethynylene-phenylene-bridged dibenzofuran, dibenzothiophene, and carbazole units.^764^ The Sonogashira cross-coupling reaction between the diiodide precursors **389.2a**–**c** and the deprotected diethynyl derivatives **389.1a**–**c** under high dilution conditions afforded the desired macrocycles **389.3a**–**c** in moderate yields (Scheme 389). The three compounds showed UV absorptions with onsets below 400 nm and were fluorescent, with Φ_F_ of up to 0.57 for **389.3a**.

**Scheme 390:**
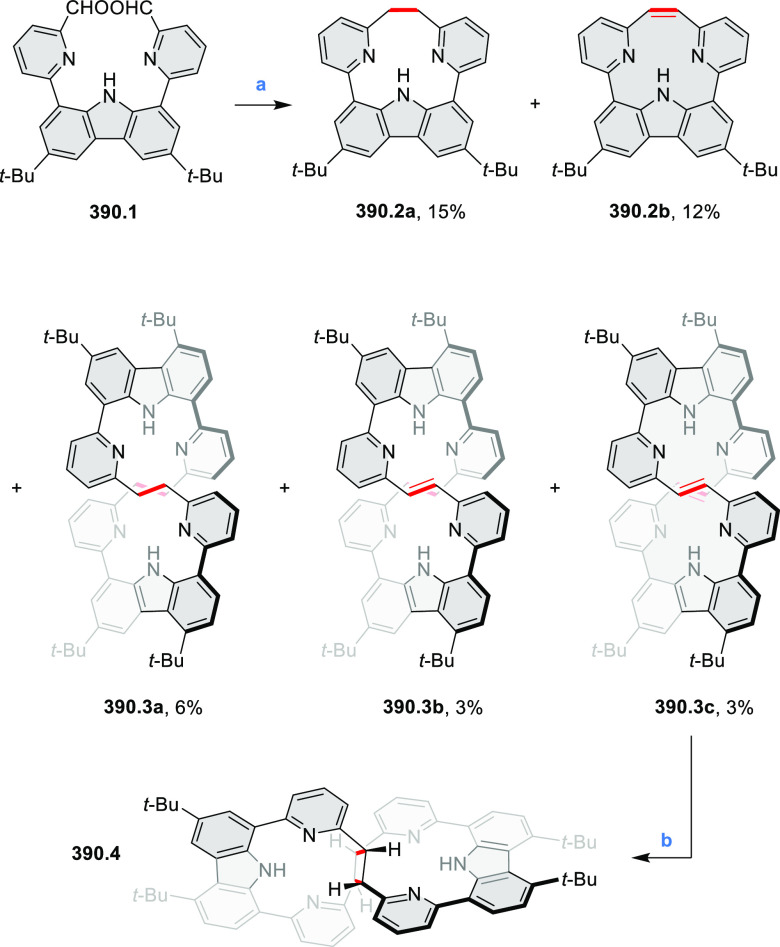
Synthesis of a Carbazole-Based Cyclophane Reagents and conditions: (a)^765^ TiCl_4_, Zn, pyridine, dioxane, reflux; (b) CHCl_3_ or CDCl_3_, 365 nm, 72 h.

Hybrid cyclophanes consisting of carbazole and pyridine units were reported in 2019 by Pawlicki et al.^765^ The dialdehyde precursor **390.1** was subjected to the low valent titanium coupling in 1,4-dioxane which resulted in products of both intramolecular and intermolecular cyclization (Scheme 390). **390.2a** and **390.2b** were obtained in 15% and 12% yield. In this reaction, cyclodimers **390.3a**–**c** were also isolated as minor products. Upon UV irradiation of **390.3c** in air-free CDCl_3_ for 72 h, an intramolecular cyclobutane-linked product **390.4** was obtained as a result of close proximity of the two vinylene bridges, which allowed the [2 + 2] photodimerization. In contrast to the helically twisted **390.3c**, compound **390.4** was found to be silent in the CD experiments in line with its achiral structure.

**Scheme 391:**
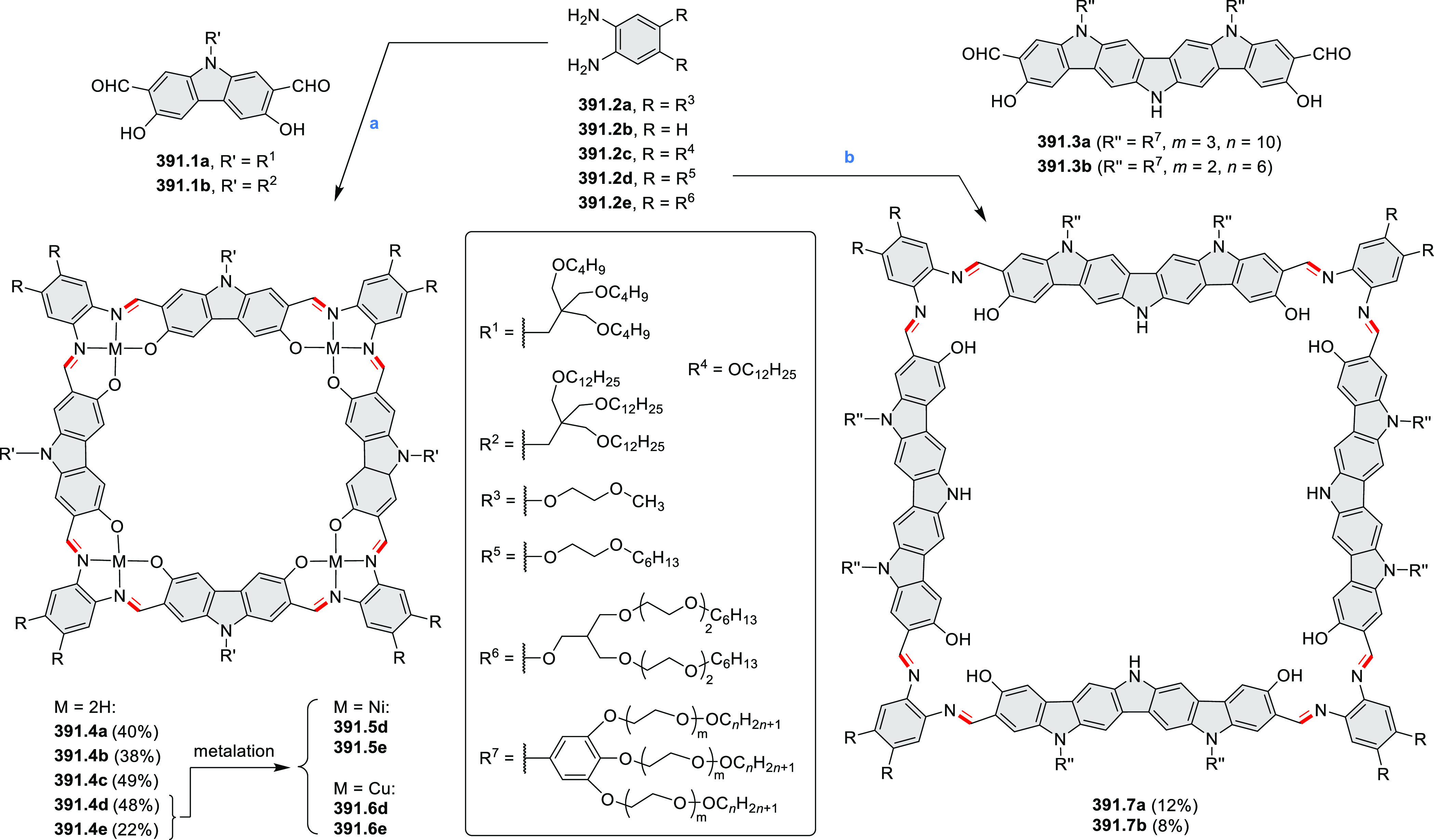
Synthesis of an Imine-Bridged Carbazole-Based Cyclophane Reagents and conditions: (a)^766^ EtOH, reflux, 48 h; (b)^767^ EtOH, CH_3_CN, reflux, 36.5 to 38 h.

In 2015, Tanaka et al. reported the synthesis of mesogenic macrocycles composed of four bis(salicylidene)-*o*-phenylenediamine (salphen) moieties alternating with four carbazoles.^766^**391.4e** was synthesized in 22% yield in a 4:4 condensation reaction between the carbazole **391.1b** and *o*-phenylenediamine **391.2e** (Scheme 391). A similar cyclization approach was used to synthesize the other macrocycles **391.4a**–**d**. An X-ray crystallographic analysis of **391.4a** revealed a highly symmetric structure where the inner cavity size was ca. 9 Å. Metalated derivatives **391.5**–**6** self-assembled into columnar liquid-crystalline phases showing high fluidity over a wide range of temperatures. Later in 2017, the same authors reported the synthesis of expanded macrocycles containing diindolocarbazole units, which were design to retain the geometry of their carbazole-based congeners.^767^ The structure of the giant macrocycle **391.7b** was unambiguously confirmed using STM, which showed a hollow square structure with a diagonal of 2.5 nm. **391.7a** formed a Col_r_ thermotropic phase stable over a range of 16–213 °C.

**Scheme 392:**
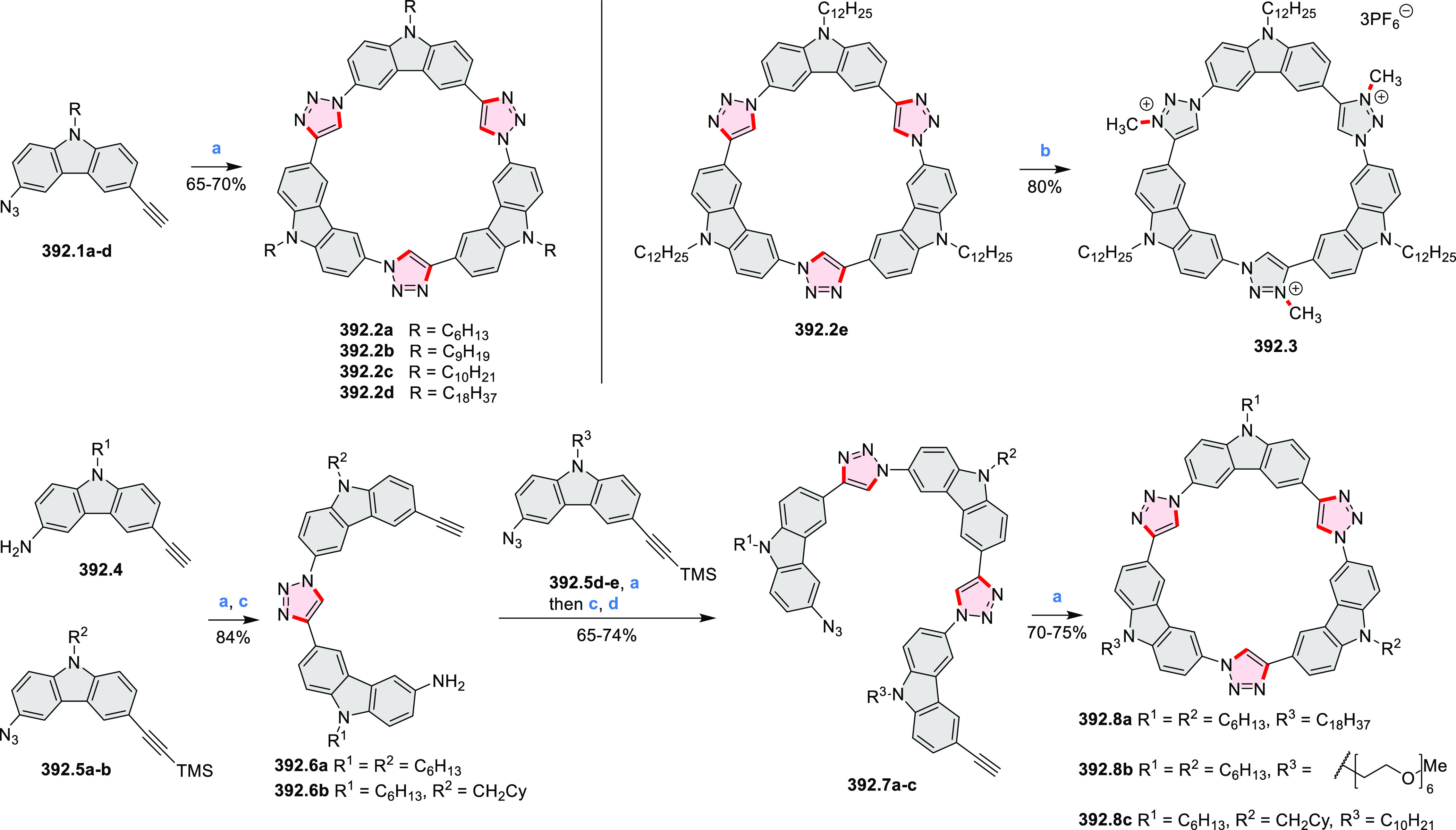
Synthesis of Tricarbazolotriazolophane Macrocycles Reagents and conditions: (a)^768,769^ CuSO_4_, sodium ascorbate, TBTA, 2:1:1 THF/EtOH/H_2_O, 55–70 °C, 6–7 h; (b)^772^ (1) Me_3_OBF_4_, DCM, rt, 4 d, (2) NH_4_PF_6_, H_2_O/acetone, rt, 2 h; (c)^769^ K_2_CO_3_, 2:1 THF/MeOH, rt, 1 h; (d) (1) NaNO_2_, TsOH, THF/H_2_O, 0 °C, 30 min, (2) NaN_3_, 0 °C to rt, 1.5 h.

Trimeric macrocycles with alternating carbazole and triazole units can be formed via CuAAC cyclooligomerization (Scheme 392). Following the first description of these structures by Flood and co-workers (see CR2017, Section 7.7.2), a recent development was the study of their self-assembly in the presence of anionic guests.^768^ A combination of anion binding by triazoles acting as C–H hydrogen bond donors and macrocycle stacking caused the formation of a 3:2 host–guest complex of **392.2b** and a bisulfate anion in CHCl_3_. A hydrogen-bonded bisulfate dimer was thus bound inside a stack of three **392.2b** molecules. Addition of MeCN to the medium weakened host–host interaction and allowed the 2:2 complex to form instead. A solid-state structure of **392.2a** cocrystallized with NBu_4_HSO_4_ revealed 2:2:2 assemblies of **392.2a** with HSO_4_^–^ and NBu_4_^+^.

A stepwise synthesis of tricarbazolotriazolophanes was also performed, which allowed us to prepare compounds bearing dissimilar N-substituents on the carbazole groups.^769^ The low reactivity of TMS-capped alkynes in the CuAAC reaction and the use of arylamine as a precursor to the triazole reactive handle enabled the synthesis of dimers **392.6a**,**b** followed by open trimers **392.7a**–**c**. These precursors were cyclized in the final step, providing the macrocycles **392.8a**–**c** with two or three different N-substituents. On the graphite surface, the products tended to assemble into rings of six molecules each, with different substitution patterns resulting in different 2D polymorphs. These assemblies were stabilized by hydrogen bonding interactions between the triazole N atoms and the carbazole C–H units on the outside of the macrocycle, and their self-organization behavior was further studied using STM imaging and computational methods.^770,771^

Nakamura and co-workers demonstrated enhancement of the anion affinity of a tricarbazolotriazolophane by *N*-alkylation of its triazoles.^772^ This was performed via treatment with a trimethyloxonium salt, providing the tricationic product **392.3** in 80% yield. Halide binding in DCM was consistent with 1:1 association and was enhanced by 2 orders of magnitude in the alkylated receptor **392.3** compared to **392.2**. Anion affinity order was I^–^ > Br^–^ ≈ Cl^–^, reflecting the better size match of I^–^ with the macrocycle cavity.

**Scheme 393:**
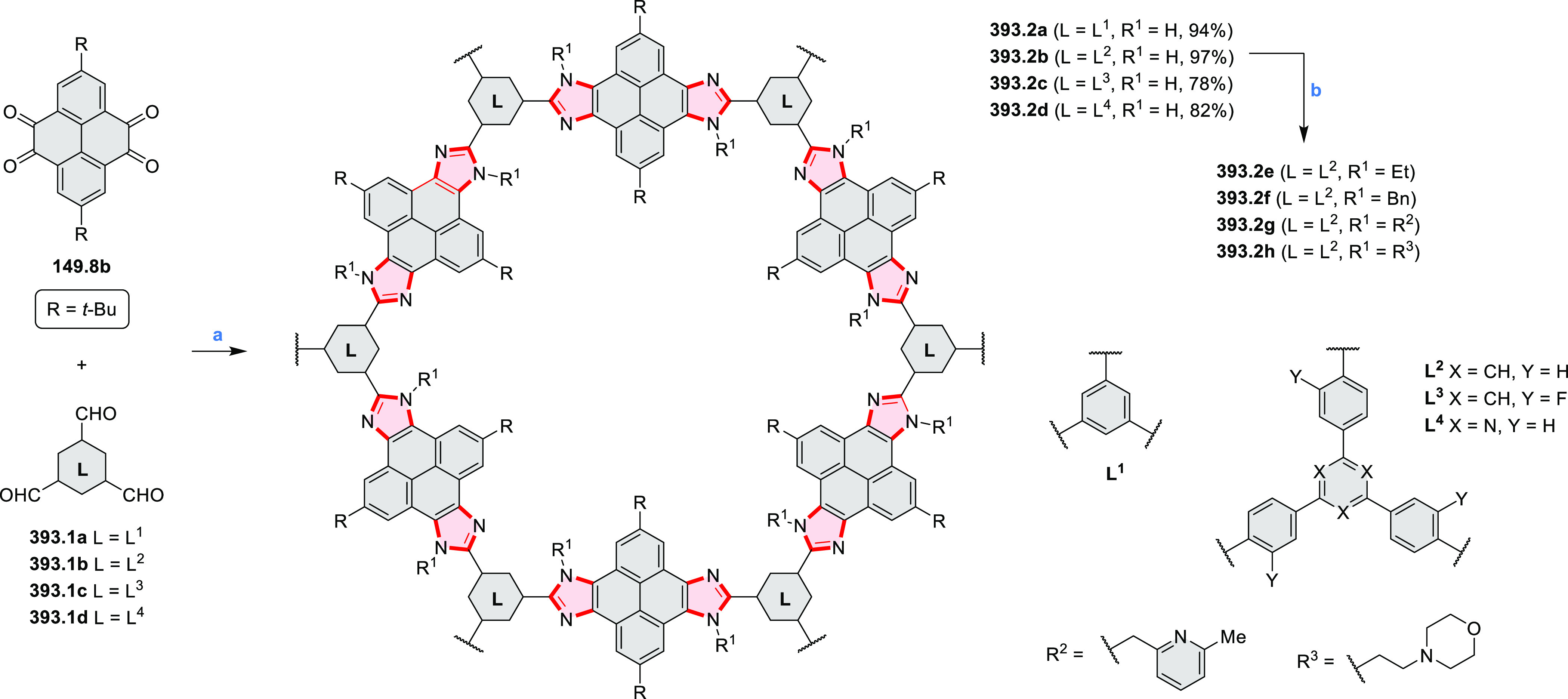
Shape-Persistent Macrocycles Containing *peri-*Condensed Pyridine Rings Reagents and conditions: (a)^331^ NH_4_OAc, dioxane/mesitylene (1:4, v/v) 150 °C, 5 days; (b) R^1^-Br, NaH, THF, 65 °C, 2 days.

Ding and Wang reported pyrenodiimidazole-based COFs **393.2a**–**d**, which were obtained using the Debus–Radziszewski reaction under solvothermal conditions from the tetraone **149.8b**, ammonium acetate, and the corresponding trialdehydes **393.1a**–**d** (Scheme 393, cf. Scheme 165, Section 4.7.2).^331^ Their structures were proposed to contain hexagonal lattices of cyclophane-like pores. These imidazole-linked COFs were found be insoluble in common solvents and showed high thermal stability (up to 400 °C) and chemical inertness toward strong acids and bases. These imidazole-linked COFs were further postmodified via *N*-alkylation to yield **393.2e**–**h** which could be used as functional materials. BET surface areas determined for these systems using nitrogen adsorption–desorption measurements were in the rage of ca. 500 to 800 m^2^ g^–1^.

#### 7.7.3 Sulfur-Containing *peri-*Fused Cyclophanes

**Chart 39:**
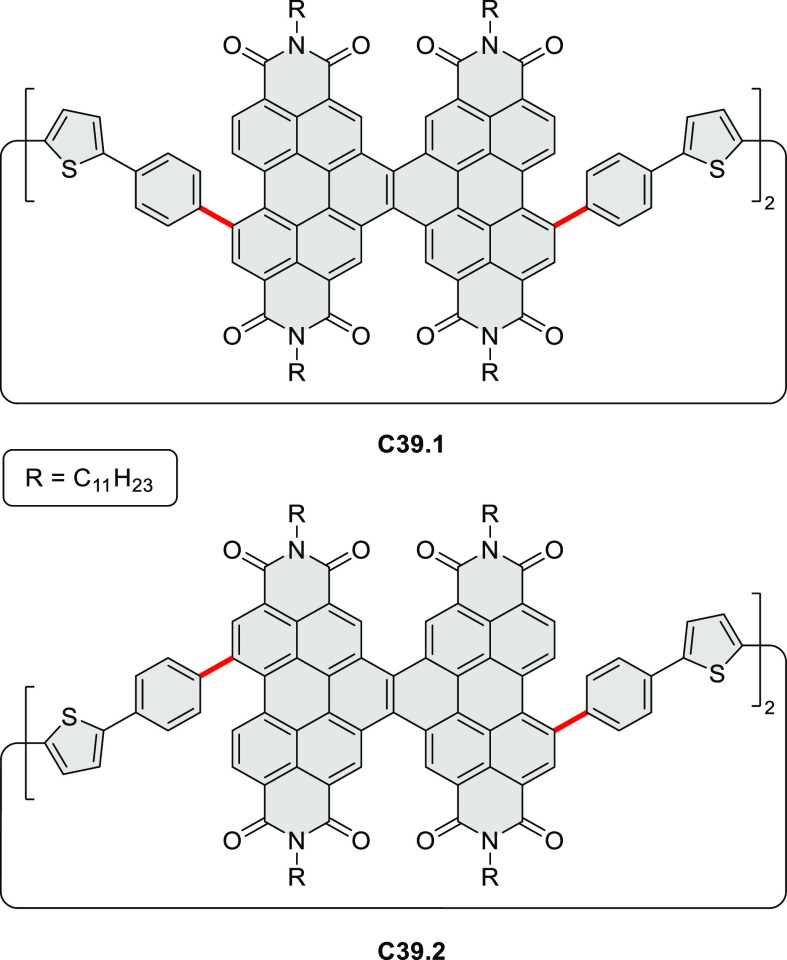
Sulfur-Containing *peri-*Fused Macrocycles

In 2018, the Nuckolls group described two large, isomeric macrocycles **C39.1** (“cis”) and **C39.2** (“trans”) consisting of helical PDI ribbons connected via phenylene–thiophene linkers (Chart 39).^773^ These systems were synthesized from regioisomerically pure PDI building blocks, which were assembled into cyclodimers using the platinacycle method. The macrocycles were explored as n-type charge-transport materials in OFET devices. The cis isomer **C39.1** exhibited electron mobility of ∼4.1 × 10^–3^ cm^2^ V^–1^ s^–1^, which was four times greater than that of the *trans*-isomer **C39.2** (∼9.9 × 10^–4^ cm^2^ V^–1^ s^–1^). This difference in mobility was ascribed to the greater flexibility of the former species, which enhanced intermolecular contacts in thin films.

**Scheme 394:**
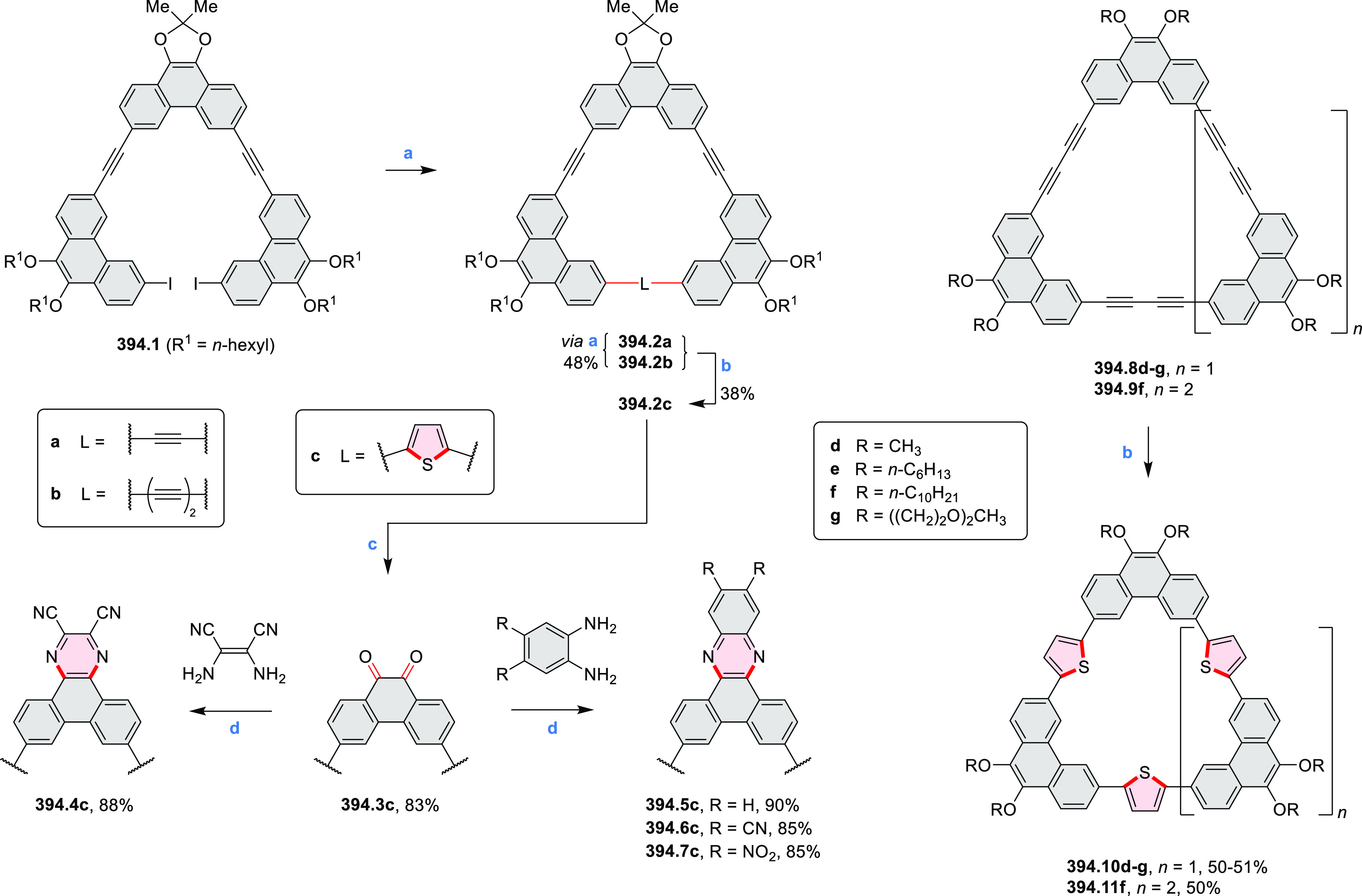
Sulfur-Containing *peri-*Fused Macrocycles Reagents and conditions: (a)^775^ acetylene, Pd(PPh_3_)_4_, CuI, Et_3_N, THF, 70 °C, 3 h; (b)^774,775^ Na_2_S·H_2_O, toluene, 2-methoxyethanol, 150 °C, 12 h; (c)^775^ TFA, DCM, H_2_O, rt, 12 h; (d)^775^*p*-TsOH, AcOH, EtOH, DCM, 110 °C, 12 h.

Phenanthrylene–thienylene macrocycles **394.10**–**11** were obtained by Mazal and co-workers via sulfide-mediated annulation of the corresponding butadiynylene precursors **394.8**–**9** (Scheme 394).^774^ The cyclotetramer **394.11f** was more easily oxidizable than the corresponding cyclotrimer and formed a stable radical cation upon treatment with DDQ. Self-association of these macrocycles due to π–π stacking was quantified using ^1^H NMR; the association constant was determined to be 291 ± 33 M^–1^ for **394.11f**. Later in 2018, the same group reported another series of fully conjugated donor–acceptor (D–A) phenanthrylene–thienylene structures as shape-persistent macrocycles (Scheme 394).^775^ The open-chain diiodo compound **394.1** was used as the precursor for Sonogashira coupling reaction with acetylene gas, which afforded two macrocyclic products in a combined yield of 48%. The resulting inseparable products **394.2a** and **394.2b** were directly used as a mixture in the next step, in which the butadiynylene linker of **394.2b** was selectively transformed into the 2,5-thienylene moiety. To obtain the D–A macrocycles, the acetonide protection of the quinone functionality was removed by trifluoroacetic acid (TFA) in a two-phase DCM/water system, yielding the quinone **394.3c**. The latter quinone was subjected to condensation reactions with diamines to produce peripherally extended macrocycles **394.4c**–**7c**.

**Scheme 395:**
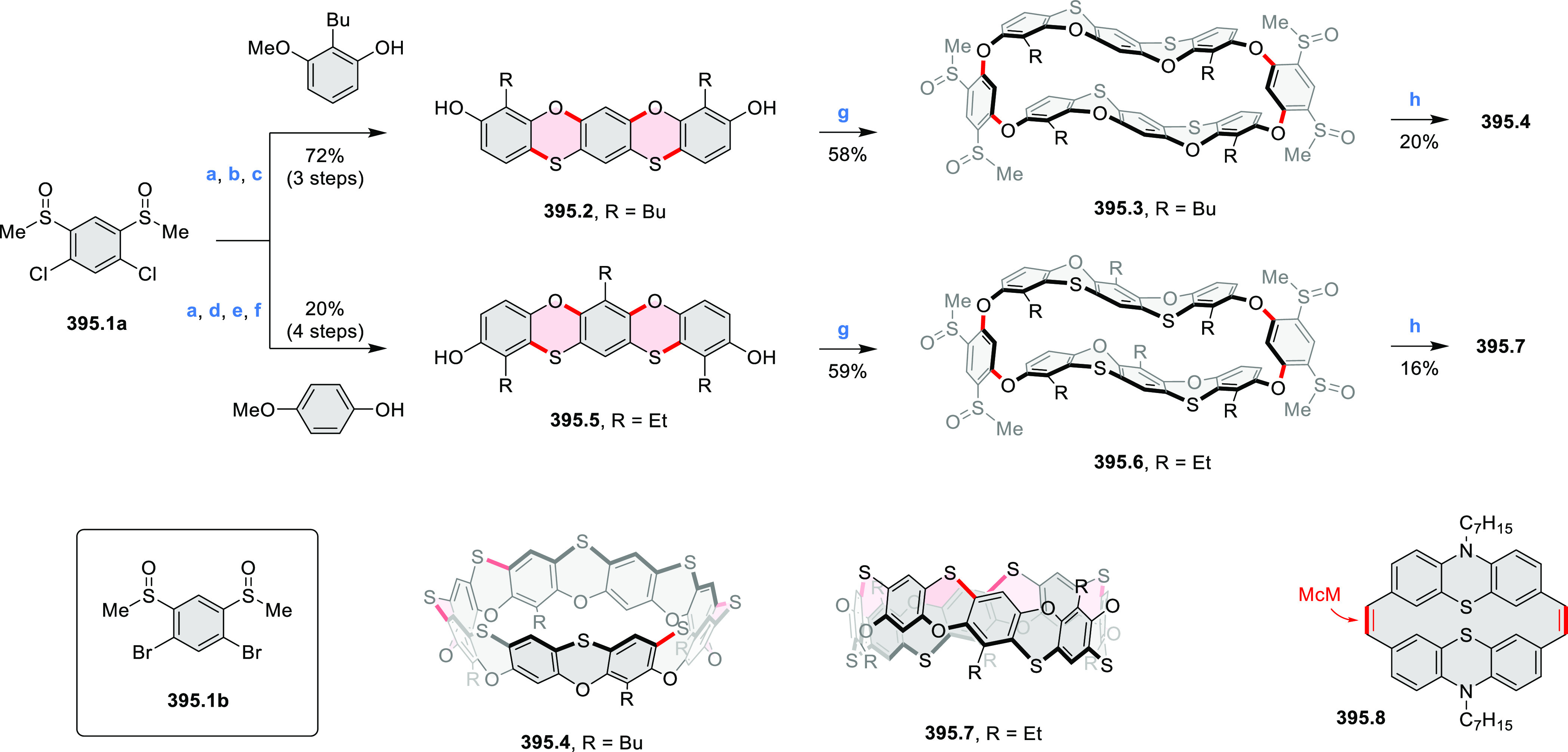
Phenoxathiin- and Phenazine-Containing Cyclophanes Reagents and conditions: (a)^777^ Cs_2_CO_3_, DMSO, 120 °C, 26 h; (b) (1) Tf_2_O, dichloroethane, rt, 1 h, (2) pyridine, rt, 10 h; (c) BBr_3_, DCM, −15 °C to rt, 20 h; (d) (1) CF_3_SO_3_H, P_2_O_5_, rt, 26 h, (2) pyridine/H_2_O (1:1, v/v), 105 °C, 18 h; (e) (1) *n*BuLi, THF, −5 °C, 1 h, (2) CH_3_CH_2_I, rt, 24 h; (f) BBr_3_, DCM, −10 °C to rt, 20 h; (g) **395.1b**, CuI, Cs_2_CO_3_, *N*,*N-*dimethylglycine, DMAc, 150 °C, 48 h; (h) (1) CF_3_SO_3_H, 80 °C, 48 h, (2) pyridine/H_2_O (1:1, v/v), 105 °C, 15 h.

Cyclophanes containing phenothiazine and related subunits were previously reported by several groups (cf. CR2017, Section 7.7.3). More recently the known system **395.8** was explored as an antimicrobial agent, capable of inhibiting both bacterial and fungal pathogens (Scheme 395).^776^ In 2020, Jiang, Zhu, and co-workers reported the synthesis of phenoxathiin-based molecular belts.^777^ The biphenolic building blocks **395.2** and **395.5** were obtained over a series of steps starting from **395.1a**. Ullmann-type cyclization of **395.2** or **395.5** with the dibromoarene **395.1b** followed by an intramolecular Friedel–Crafts reaction resulted in the formation of cyclo[8]phenoxathiins **395.4** and **395.7**, respectively. **395.4** had a bowl-like geometry and was found to form a capsule-like 2:1 complex with C_60_, stabilized by C–H···S hydrogen bonds. The complex was stable not only in the solid state but also in solution, with a high formation constant β_2_ = 3.6 × 10^9^ M^–2^. In the crystal, **395.7** formed a ring-in-ring supramolecular complex with [2,2]paracyclophane, characterized by a distorted octagonal conformation.

**Scheme 396:**
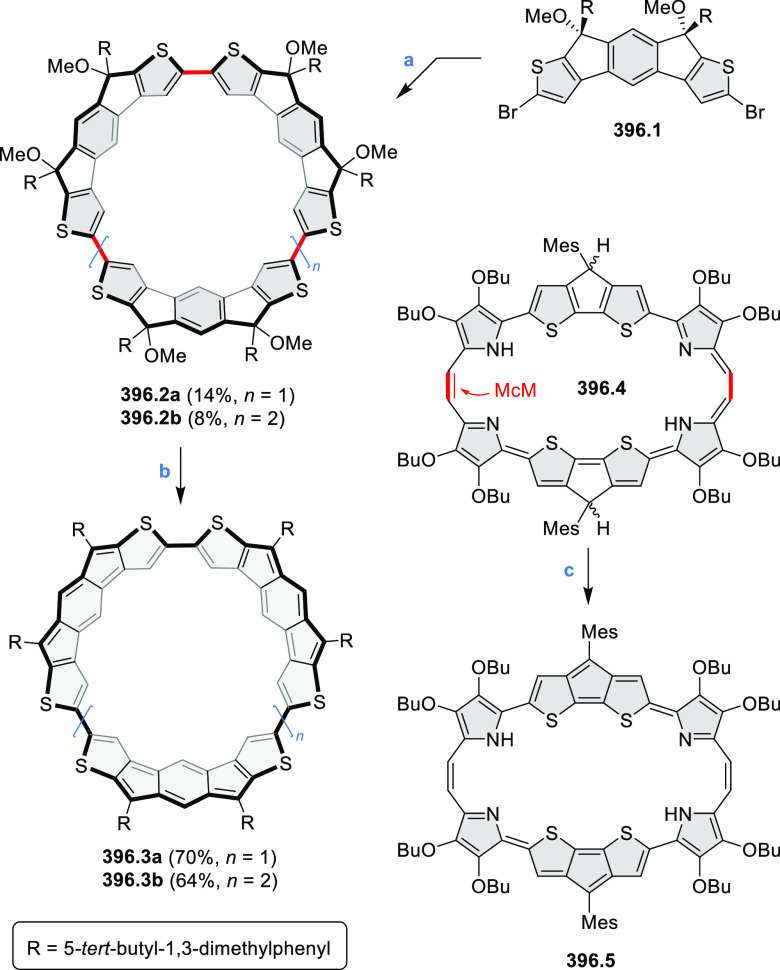
Sulfur-Containing *peri-*Fused Macrocycles Reagents and conditions: (a)^778^ Ni(cod)_2_, THF, 65 °C; (b)^778^ SnCl_2_, DCM, rt; (c)^779^*p*-chloranil, rt, 2 h.

*peri*-Fused macrocycles, cyclotrimer **396.3a** and cyclotetramer **396.3b**, were reported by the Wu group (Scheme 396).^778^ These macrocycles were synthesized via Yamamoto coupling of the dibromo monomer **396.1** followed by the reduction with SnCl_2_ to afford the fully conjugated products. These macrocycle products exhibited good stability under ambient conditions, and the attachment of bulky 4-*tert*-butyl-2,6-dimethylphenyl groups improved the solubility. NMR investigations indicated the closed-shell ground-state electronic structure for both macrocycles, with 36 π- and 48 π-electron antiaromatic conjugation pathways in **396.3a** and **396.3b**, respectively. The dications of **396.3a** and **396.3b** obtained by oxidation with NO[SbF_6_] were characterized as globally aromatic open-shell singlet diradical dications. The singlet–triplet energy gaps of [**396.3a**]^2+^ and [**396.3b**]^2+^ were estimated to be −2.90 and −2.60 kcal mol^–1^, respectively.

In another report by the Wu group, the dihydro-expanded porphycene **396.4**, obtained via McMurry coupling, was subjected to further oxidative dehydrogenation to produce the cyclopentabithiophene-bridged macrocycle **396.5**.^779^ ESR and SQUID measurements revealed that **396.5** had an open-shell singlet ground state with a small tetraradicaloid contribution and a small singlet–triplet energy gap (Δ*E*_S–T_) of −0.92 kcal mol^–1^. The cyclic voltammogram of **396.5** displayed four oxidation waves at −0.64, −0.54, −0.09, and +0.22 V and one reduction at −1.45 V (vs Fc/Fc^+^) with a small electrochemical HOMO–LUMO energy gap of 0.81 eV. Despite these features, the macrocycle exhibited very good stability under ambient conditions.

#### 7.7.4 Oxygen-Containing Cyclophanes

**Scheme 397:**
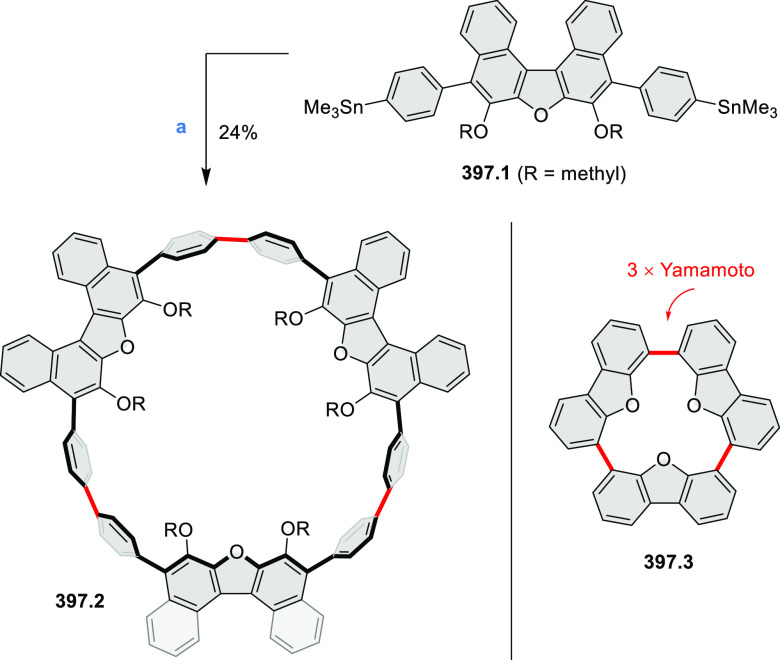
Oxygen-Containing *peri-*Fused Macrocycles Reagents and conditions: (a)^780^ (1) PtCl_2_(cod), THF, reflux, 2 days, (2) PPh_3_, toluene, reflux, 3 h.

Preparation of the furan-fused [12]cyclo-*p*-phenylene macrocycle **397.2** was reported in 2017 by Tsubaki et al.^780^ The target cyclic compound **397.2** consisting of dinaphthofuran units and biphenylene units was synthesized from **397.1** using the platinacycle method (Scheme 397). The UV–vis spectrum of **397.2** in DCM exhibited a maximum at 361 nm and was similar to that of **397.1**, reflecting the weak electronic communication between the dinaphthofuran fragments. The fluorescence quantum yield of compound **397.2** was 7% with the emission maximum at 439 nm. In 2016, Higashibayashi et al. reported the synthesis of [3]cyclo-4,6-dibenzofuranylene **397.3** by Ni(0)-mediated reductive coupling of 4,6-dibromodibenzofuran.^781^ The flake-shaped *C*_2_ symmetric structure for this cyclotrimer **397.3** was found to be the most stable conformer as predicted by the DFT calculations.

#### 7.7.5 Heteroatom-Bridged Cyclophanes

**Chart 40:**
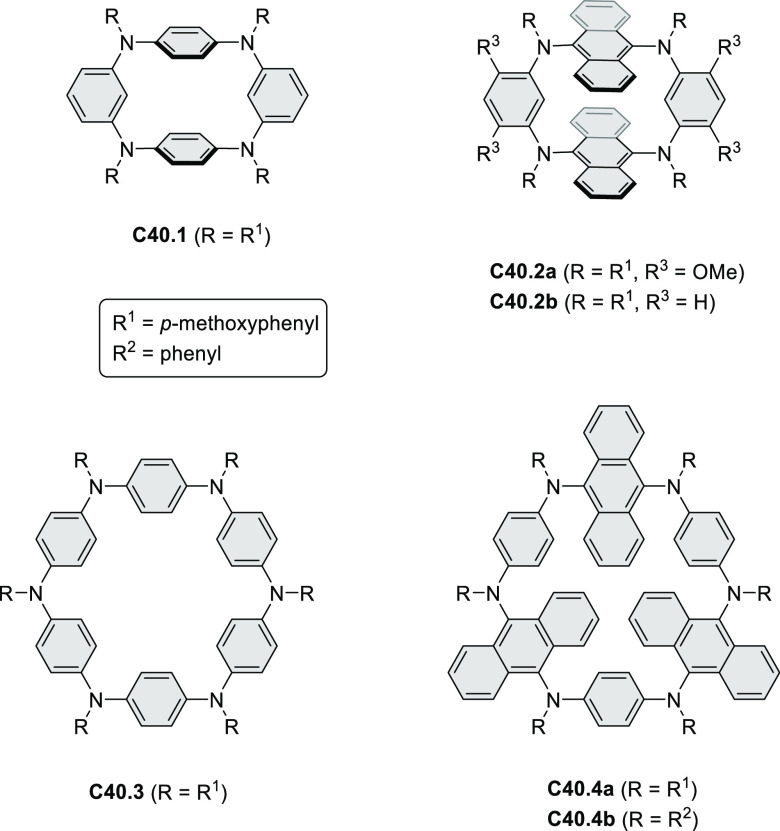
Polycyclic Arylamine Cyclophanes

In 2017, Ito and co-workers investigated redox properties of the known tetraazacyclophanes **C40.1** and newly synthesized tetraazacyclophanes **C40.2a**,**b** containing 9,10-anthracenylene moieties (Chart 40).^782^**C40.2a** and **C40.2b** were obtained in 14% and 8% yield from a Buchwald–Hartwig reaction. The dication of **C40.2a** was prepared upon addition of 2 equiv of AgSbF_6_ and was isolated as an air-stable salt, while the dication of **C40.2b** was too unstable to be isolated. X-ray crystallographic analysis of the dications of **C40.1** and **C40.2a** revealed different types of structural deformation caused by steric demand of constituent aromatic moieties, which led to different spin density distributions. The singlet–triplet energy gap (Δ*E*_S–T_) values for the dications of **C40.1** and **C40.2a** were determined to be 0.3 kcal mol^–1^ and −1.0 kcal mol^–1^, respectively, by SQUID measurements, indicating, respectively, a ground-state triplet and singlet for these two species. Later in the same year, Sakamaki, Iwanaga, and Ito reported two larger macrocyclic derivatives hexaaza[1_6_]paracyclophane, **C40.3** and **C40.4**.^783^ The authors were able to isolate the stable trication salt of **C40.4a** and found that the three segmented phenylenediamine-based radical spins were found to be mutually antiferromagnetically coupled with *J*/*k*_B_ ≃ −74 K, leading to a typical spin-frustrated three-spin system.

**Scheme 398:**
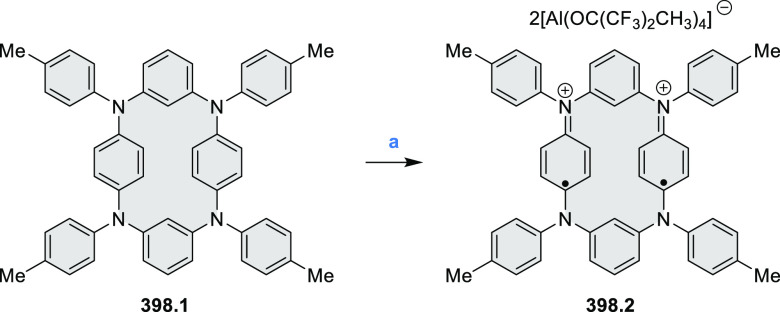
Oxidation of a Tetraazacyclophane to a Diradical Dication Reagents and conditions: (a)^784^ 2 equiv of Ag[Al(OC(CF_3_)_2_CH_3_)], DCM, rt, 1 day.

A related diradical dication salt **398.2** was prepared by Wang and Rajca et al. via oxidation of tetraazacyclophane **398.1** with an Ag(I) aluminate reagent (Scheme 398).^784^ The product proved to be thermally stable up to 180 °C and could be stored under ambient conditions. It had a triplet ground state with a singlet–triplet energy gap of approximately 0.5 kcal mol^–1^. In the solid state, **398.2** formed 1D chains with contacts between the peripheral *p*-tolyl groups. Neighboring chains were largely separated by the aluminate anions. These assemblies were found to possess intrachain antiferromagnetic coupling of *J*′/*k* = −5.4 K, which was associated with the intermolecular C···C contacts, including π–π interactions.

**Scheme 399:**
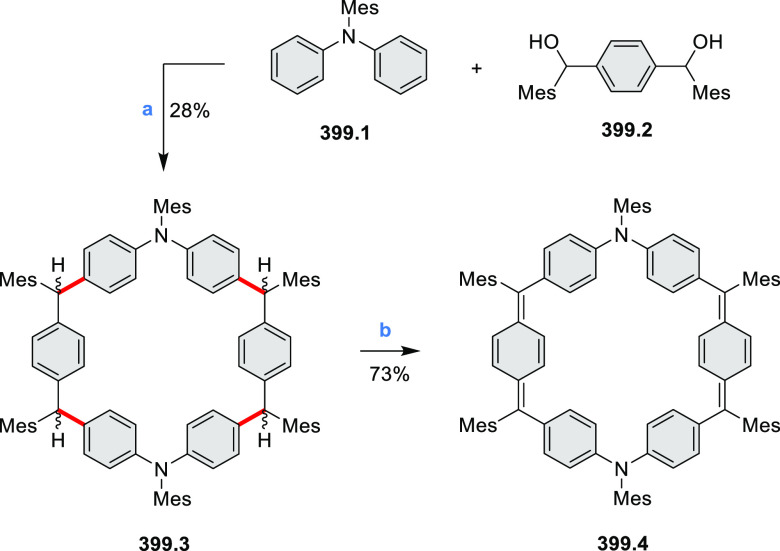
Polycyclic Arylamine Cyclophanes Reagents and conditions: (a)^785^ BF_3_·OEt_2_, DCM; (b) DDQ, toluene.

In 2019, Chi and co-workers reported the synthesis of a hybrid macrocycle **399.4**, containing alternating *para*-quinodimethane and triphenylamine subunits (Scheme 399). The initial precursor **399.3** was obtained in a Lewis acid-mediated intermolecular Friedel–Crafts alkylation between the triarylamine **399.1** and the diol **399.2** in a dilute DCM solution (Scheme 399). Subsequently, oxidative dehydrogenation of **399.3** with DDQ in toluene afforded **399.4** in 73% yield. This macrocycle showed moderate 32 π-electron global antiaromaticity, reflecting the participation of the bridging nitrogen atoms in π-conjugation. The dication and tetracation of **399.4** showed an open-shell diradical character with mall singlet–triplet gaps of −1.07 and −2.96 kcal mol^–1^, respectively. These two species were globally aromatic and antiaromatic, respectively, with [30]- and [28]annulene structures.

**Scheme 400:**
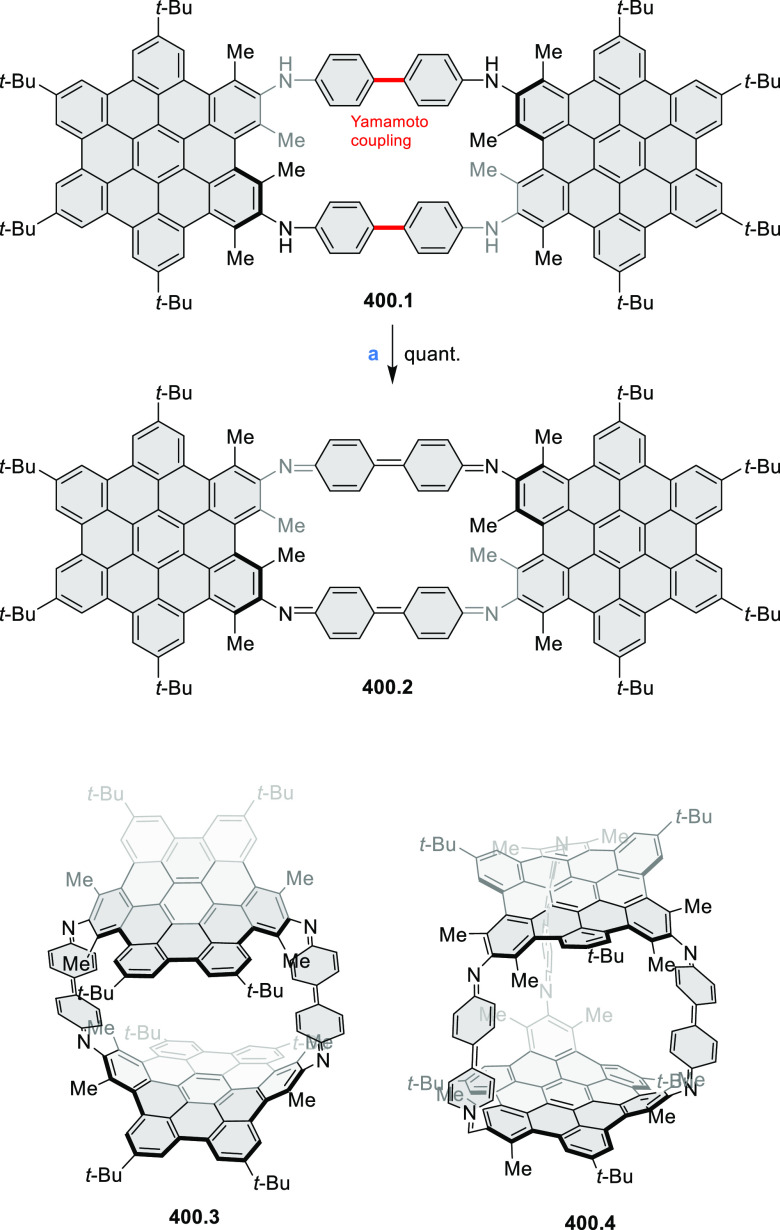
Conjugated Macrocyclic Hexabenzocoronene Dimers Reagents and conditions: (a)^786^ PbO_2_, CDCl_2_CDCl_2_, rt, 5 min.

Macrocycles consisting of two hexabenzocoronene-based units connected by benzidine linkers were described by Isobe, Wu, and co-workers. The macrocyclization step was a double Yamamoto coupling of aryl bromide precursors in the presence of Ni(cod)_2_, which provided **400.1** and related molecules in 7–15% yields. Conjugated final products **400.2**–**4** were obtained from these precursors in quantitative yields upon oxidation with PbO_2_ (Scheme 400).^786^ Steric crowding in the dimethyl-substituted bay regions of **400.1**–**2** induced chirality in their hexabenzocoronene blocks. Only the homochiral dimers of **400.1** were obtained in the Yamamoto cyclodimerization from a racemic starting material. Compound **400.1** was separated into enantiomers by chiral HPLC and used to prepare optically pure samples of **400.2**. Compounds **400.2**–**4** possessed oligoradicaloid characters due to the quinoidal structure of the oxidized benzidine linkers. Weak and broad low-energy absorption bands characteristic of such structures emerged in the final oxidative step. Their ESR signals were weak at rt but increased upon heating, revealing Δ*E*_S-T_ close to 7 kcal mol^−1^ in all cases. The hydrogenated precursors of **400.3**–**4** selectively encapsulated fullerene C_70_ via interactions with their concave surfaces.

**Scheme 401:**
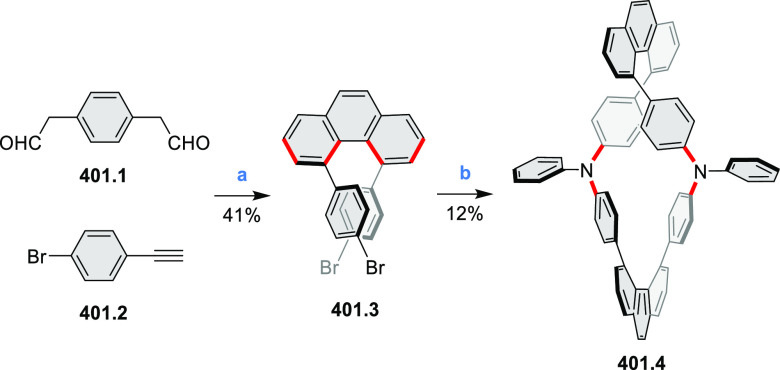
Twisted Macrocycle with a Nitrogen Bridge Reagents and conditions: (a)^787^ BF_3_·Et_2_O, MS4 Å, 10:1 DCM/HFIP, rt, 3 h; (b) aniline, *t*-BuONa, Pd_2_(dba)_3_, P(*t*-Bu)_3_, toluene, 90 °C, 16 h.

The synthesis of helically twisted macrocycles, including the nitrogen-bridged derivative **401.4**, was reported by Itami and co-workers (Scheme 401).^787^ Based on a previously reported method, condensation of *p*-phenylenediacetaldehyde **401.1** with 1-bromo-4-ethynylbenzene **401.2** led to the sterically congested phenanthrene **401.3**. This compound was then coupled with aniline under Buchwald–Hartwig amination conditions to provide the cyclic dimer **401.4** in 12% yield. The nitrogen linker in **401.4** was the smallest linker in a series of compounds prepared via a similar approach. This resulted in a particularly high configurational stability with a racemization barrier of 31.7 kcal mol^−1^ according to computational analysis. Emission of **401.4** (λ_em_ = 466 nm, Φ = 41%) was bathochromically shifted relative to the analogues with aromatic linkers.

**Scheme 402:**
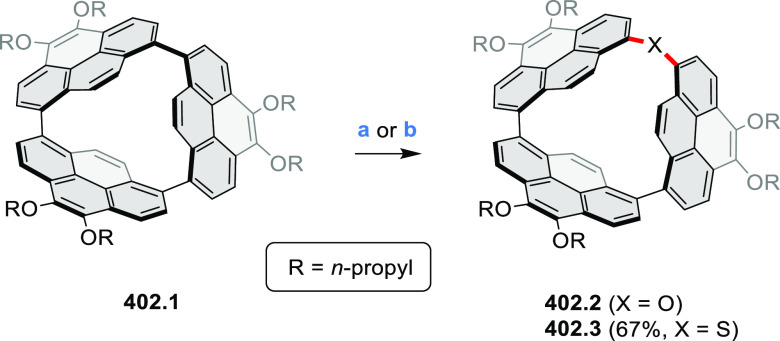
Oxygen-Containing *peri-*Fused Macrocycles Reagents and conditions: (a) O_2_, *hν*; (b) Na_2_S·9H_2_O, DMF, 80 °C.

The formation of oxygen- and sulfur-bridged cyclopyrenylene macrocycles was reported in 2019 by Aratani and Yamada et al. (Scheme 402).^788^ While performing emission studies of the cyclopyrenylene trimer **402.1**, the authors observed a spectral change under ambient light in DCM, with a clear isosbestic point and a new peak emerging at 480 nm. The change was found to result in quantitative formation of the cyclic ether **402.2**, containing an oxygen bridge between two pyrene units. Excitation of the cyclopyrenylene trimer at 515 nm resulted in singlet dioxygen ^1^O_2_* (^1^Δ_g_) sensitization with a quantum yield of 0.33 at 298 K, yielding ultimately the oxygen insertion product. When treated with Na_2_S·9H_2_O, **402.1** was also transformed into the cyclic thioether **402.3**, providing the latter product in 67% conversion yield. The fluorescence quantum yields (Φ_f_) for **402.2** and **402.3** were found to be 0.54 and 0.44, respectively.

**Chart 41:**
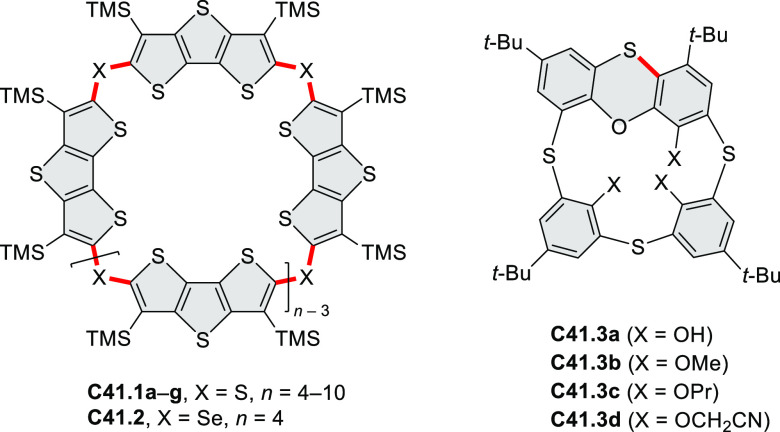
Miscellaneous Heteroatom-Bridged Cyclophanes

Synthesis of a series of thiacalix[*n*]dithienothiophenes **C41.1a**–**g** (*n* = 4–10) and selenacalix[4]dithienothiophene **C41.2** were reported by Hasegawa and co-workers (Chart 41).^789,790^ The sulfur-containing macrocycles were prepared through a Pd-catalyzed coupling reaction of an appropriate dibromo precursor with tributylstannyl sulfide. The combined yield of the cyclic homologues **C41.1a**–**g** was as high as 75% when toluene solvent was used. Formation of larger macrocycles was found to be suppressed in DMF, whereas the highest conversion was found for reactions performed in a 1:1 mixture of DMF/toluene. An XRD analysis showed that **C41.1a** formed a puckered quadrilateral conformation with a large square cavity with a diagonal distance of 12.8 Å, and the shortest distance between facing walls was found to be 8.6 Å. Compound **C41.1b** showed an envelope-shaped pentagonal geometry with diagonal S···S distances in the range of 13.2–15.6 Å. UV–vis absorption spectra showed a red-shifted band for the larger macrocycles, which is attributed to the conjugation between the DTT units of each macrocycle through the sulfide linkers. Cyclic voltammograms showed reversible multielectron redox processes with low oxidation potentials, reflecting efficient electronic delocalization in these systems. Binding studies of these macrocycles with C_60_ revealed that **C41.1a** formed a Janus-head complex with two molecules of C_60_, whereas **C41.1b** and **C41.1c** formed stable 1:1 complexes with C_60_. The related selenium derivative **C41.2** was prepared in 29% yield from the palladium-catalyzed coupling of 2,5-dibromodithienothiophene derivatives with (*n*Bu_3_Sn)_2_Se. In this reaction only the tetrameric macrocycle was obtained, in contrast to the synthesis of thiacalix[*n*]dithienothiophenes. The cyclic tetramer **C41.2** underwent similar complexation with C_60_ to produce 1:2 adducts in the solid state.

Lhoták and co-workers reported the phenoxathiin-based thiacalix[4]arene **C41.3a**, which was accessed via an acid-catalyzed rearrangement of a spirodienone derivative, obtained by oxidation of thia[4]calixarene with chloramine-T.^791−793^ The inherently chiral **C41.3a** was alkylated, which led to the configurationally stable derivatives **C41.3b**,**c** with stereoselective formation of a partial cone conformer, which was determined using NMR and XRD analyses.

## 8 Conclusions

The field of polycyclic heteroaromatics covers a wide array of diverse yet interrelated topics. The research presented in this review is unified by a shared interest in two-dimensionally extended π-conjugation as a means of creating new properties in heterocyclic systems. Developments of the last five years, summarized herein, have expanded the repertoire of synthetic methods and spawned a plethora of new molecules. These achievements are of high relevance from the fundamental viewpoint, contributing to our understanding of physical properties of aromatic compounds and providing access to previously unknown structural motifs. These new structures often display unusual features, such as intrinsic curvature, three-dimensional aromaticity, or open-shell character. At the same time, by altering ring fusion and heteroatom placement in PHA molecules, it is possible to tune very precisely their electronic properties and solid-state behavior. In consequence, practically useful characteristics can be engendered even in structurally simple motifs, with clear benefits for materials science.

Future developments in the field will likely be driven by a continuing search for novel structural motifs. Here, introduction of seven-membered and larger rings may be seen as a particularly promising strategy toward such new structures. Unusual two-dimensional fusion patterns based on enlarged rings are easily accessible in pyrrole-based chemistry, notably among porphyrinoids, but can also be elaborated by modifications of benzenoid structures. Apart from changing the topology of the fused framework, large rings can induce out-of-plane distortions of the π-electron surface and may yield systems with pronounced negative curvature. Likewise, one can anticipate increasing interest in positively and neutrally curved heteroaromatics, which may be obtained using methods developed for carbocyclic targets. While much of the chemistry of heterocyclic nanographene analogues has been based on nitrogen, exploration of other heteroatoms, notably boron and oxygen but also some of the less-studied elements such as selenium or tellurium, is likely to have a significant impact on further evolution of the field, especially as a route toward functional materials.

Elaboration of new PHA motifs is often challenged by synthetic difficulties, and further progress will thus undoubtedly depend on the availability of efficient ring-forming reactions. Cyclizations based on oxidative, reductive, and transition-metal-catalyzed protocols are among the most fundamental methods available in the synthetic toolbox, and their further refinements will have a direct impact not only on the scope of structures that can be made but also on their large-scale availability. Annulative approaches, which enable multicomponent assembly of complicated targets, are an important alternative to direct cyclizations and will play an increasing role in heteroaromatic chemistry. Unique preparative opportunities have been offered by the remarkable development of on-surface synthesis that occurred over the past decade. While these methods currently do not give access to bulk quantities of products, they can provide unique chemical selectivities. Given the possibility of structural analysis on the single-molecule level, on-surface synthesis will surely remain an important tool of chemical discovery.

With the growing availability of polycyclic heteroaromatics, their use in diverse branches of organic chemistry and materials science will continue to expand. Because of their anisotropic shapes and structural rigidity, PHA motifs are suitable components for 1D and 2D polymers, framework materials, and supramolecular assemblies, such as molecular cages. They can also serve as functional platforms for the development of novel fluorophores, NIR dyes, charge-transfer assemblies, open-shell organics, and other systems relying on extensive π-electron conjugation. This broad range of applications has been one of the driving forces of ongoing research and will likely shape the field for years to come.

## Abbreviations

18c6: 18-crown-6
Ac: acetyl
acac: acetylacetonate
ACID: anisotropy of induced current density
AFM: atomic force microscopy
AIBN: azobis(isobutyronitrile)
AIE: aggregation-induced emission
AM: air mass (coefficient)
BAHA: tris(4-bromophenyl)aminium hexachloroantimonate
BHJ: bulk heterojunction
BINAP: 2,2′-bis(diphenylphosphino)-1,1′-binaphthyl
BODIPY: 4,4-difluoro-4-bora-3*a*,4*a*-diaza-*s*-indacene
Bpin: 4,4,5,5-tetramethyl-1,3,2-dioxaborolan-2-yl
bpy: 2,2′-bipyridyl
BrettPhos: 2-(dicyclohexylphosphino)-3,6-dimethoxy-2′,4′,6′-triisopropyl-1,1′-biphenyl
CAN: ceric ammonium nitrate
CD: circular dichroism
CDA: catalytic direct arylation
CIE: Commission International de l’Eclairage
CMP: conjugated mesoporous polymer
cod: 1,5-cyclooctadiene
COF: covalent organic framework
Col_h_: columnar hexagonal (phase)
COT: cyclooctatetraene
COTh: cyclooctatetrathiophene
*m*-CPBA: *m*-chloroperoxybenzoic acid
CPP: cycloparaphenylene
CR2017: Part 1 of this Review^1^
CT: charge transfer
CTAB: cetyltrimethylammonium bromide
CTAP: cetyltrimethylammonium permanganate
CuAAC: copper-catalyzed azide–alkyne cycloaddition
CV: cyclic voltammetry
DABCO: 1,4-diazabicyclo[2.2.2]octane
dba: *trans*,*trans*-dibenzylideneacetone
DBAP: dibenzo-9*a*-azaphenalene
(*o*-)DCB: (*ortho*-)dichlorobenzene
DCC: dicyclohexylcarbodiimide
DCE: 1,2-dichloroethane
DCM: dichloromethane
DDQ: 2,3-dichloro-5,6-dicyano-1,4-benzoquinone
DFT: density functional theory
DIC: dichloroisocyanuric acid
dipp: 2,6-di(isopropyl)phenyl
DIPEA: *N*,*N-*diisopropylethylamine
DMA, DMAc: *N*,*N-*dimethylacetamide
DMAP: 4-dimethylaminopyridine
DME: dimethoxyethane
DMF: *N*,*N-*dimethylformamide
DMSO: dimethyl sulfoxide
DMT: dimercaptotriazine
DOSY: diffusion-ordered spectroscopy
DPP: diphenylphosphine
dppb: 1,1-bis(diphenylphosphino)butane
dppf: 1,1′-bis(diphenylphosphino)ferrocene
dppp: 1,1-bis(diphenylphosphino)propane
DSC: differential scanning calorimetry
ECL: electrogenerated chemiluminescence
ESIPT: excited-state intramolecular proton transfer
ESR: electron spin resonance
Fc: ferrocene
FET: field-effect transistor
FF: fill factor
FT-IR: Fourier-transform infrared (spectroscopy)
FVP: flash vacuum pyrolysis
fwhm: full width at half-maximum
GNR: graphene nanoribbon
GPC: gel permeation chromatography
HBC: hexa-*peri*-hexabenzocoronene
*c*-HBC: hexa-cata-hexabenzocoronene
HFIP: 1,1,1,3,3,3-hexafluoro-2-propanol
HOMO: highest occupied molecular orbital
HOPG: highly ordered pyrolytic graphite
HPHAC: hexapyrrolohexaazacoronene
HPLC: high-performance liquid chromatography
ICT: intramolecular charge transfer
Ir(ppy)_3_: iridium(III) fac-tris(2-phenylpyridine)
ITO: indium tin oxide
KHMDS: potassium bis(trimethylsilyl)amide
LB: Langmuir–Blodgett (films)
LC: liquid crystal
LiHMDS: lithium bis(trimethylsilyl)amide
LUMO: lowest unoccupied molecular orbital
MALDI: matrix-assisted laser desorption ionization
MCD: magnetic circular dichroism
MIDA: *N-*methyliminodiacetic acid boronate
MO: molecular orbital
MOM: methoxymethyl
MW: microwave (heating)
NaHMDS: sodium bis(trimethylsilyl)amide
NAP: N*-*annulated perylene
NBS: *N-*bromosuccinimide
nc-AFM: noncontact atomic force microscopy
NDI: naphthalene diimide
NHC: N*-*heterocyclic carbene
NICS: nucleus-independent chemical shift
NIR: near-infrared
NIS: *N-*iodosuccinimide
NMI: naphthalene monoimide
NMP: *N-*methylpyrrolidone
NMR: nuclear magnetic resonance
NTDA: naphthalenetetracarboxylic dianhydride
OFET: organic field-effect transistor
OLED: organic light-emitting diode
OPA: one-photon absorption
OPV: organic photovoltaic(s)
OSC: organic solar cell
OTFT: organic thin-film transistor
PAH: polycyclic aromatic hydrocarbon
Palau′Chlor: 2-chloro-1,3-bis(methoxycarbonyl)guanidine
PAMY: polycyclic aromatic azomethine ylide
PC_(61)_BM: [6,6]-phenyl-C_61_-butyric acid methyl ester
PC_71_BM: [6,6]-phenyl-C_71_-butyric acid methyl ester
PCE: (photovoltaic) power conversion efficiency
Pd-PEPPSI-IPr: (1,3-bis(2,6-diisopropylphenyl)imidazolidene)(3-chloropyridyl) palladium(II) dichloride
PDBT-T1: poly[[5,10-bis(5-octyl-2-thienyl)dithieno[2,3-*d*:2′,3′-*d*′]benzo[1,2-*b*:4,5-*b*′]dithiophene-2,7-diyl]-2,5-thiophenediyl[5,7-bis(2-ethylhexyl)-4,8-dioxo-4*H*,8*H*-benzo[1,2-*c*:4,5-*c*′]dithiophene-1,3-diyl]-2,5-thiophenediyl]
PDI: perylene diimide
PDT: photodynamic therapy
PET: photoinduced electron transfer
PHA: polycyclic heteroaromatic (system)
phen: 1,10-phenanthroline
PhOLED: phosphorescent organic light-emitting diode
PIFA: phenyliodine bis(trifluoroacetate)
PLED: polymer light-emitting diode
PLQY: photoluminescence quantum yield
PMMA: poly(methyl methacrylate)
PPA: polyphosphoric acid
Ppy: 2-phenylpyridine
PS: polystyrene
PTB7: poly[[4,8-bis[(2-ethylhexyl)oxy]benzo[1,2-*b*:4,5-*b*′]dithiophene-2,6-diyl][3-fluoro-2-[(2-ethylhexyl)carbonyl]thieno[3,4-*b*]thiophenediyl]]
PTB7-Th: poly[4,8-bis(5-(2-ethylhexyl)thiophen-2-yl)benzo[1,2-*b*;4,5-*b*′]dithiophene-2,6-diyl-*alt*-(4-(2-ethylhexyl)-3-fluorothieno[3,4-*b*]thiophene-)-2-carboxylate-2–6-diyl)]
QFP: quadruply fused porphyrin
RE: reductive elimination
room temperature: rt
RuPhos: 2-dicyclohexylphosphino-2′,6′-diisopropoxybiphenyl
SCE: standard calomel electrode
SF: singlet fission
SPhos: 2-dicyclohexylphosphino-2′,6′-dimethoxybiphenyl
Spiro-OMeTAD: 2,2′,7,7′-tetrakis(*N*,*N-*di-*p*-methoxyphenylamine)-9,9′-spirobifluorene
SQUID: superconducting quantum interference device
STM: scanning tunneling microscopy
STS: scanning tunneling spectroscopy
SubPc: subphthalocyanine
TADF: thermally activated delayed fluorescence
TAT: 7,8,15,16-tetraazaterrylene
TBA: tetrabutylammonium (cation)
TBAP: tetrabutylammonium perchlorate
TBDMS: *tert*-butyldimethylsilyl
TBP: 2,4,6-tri(*tert*-butyl)pyridine
TBTA: tris[(1-benzyl-1*H*-1,2,3-triazol-4-yl)methyl]amine
TBTQ: tribenzotriquinacene
TCB: 1,2,4-trichlorobenzene
TCNE: tetracyanoethylene
TCNQ: tetracyanoquinodimethane
TEA: triethylamine
TEM: transition electron microscopy
Tf: trifluoromethanesulfonyl
TFE: 2,2,2-trifluoroethanol
TFT: thin-film transistor
THF: tetrahydrofuran
TIBS: tri-*iso*-butylsilyl
Tip: 2,4,6-triisopropylphenyl
TIPS: triisopropylsilyl
TFA: trifluoroacetic acid
TMEDA: tetramethylethylenediamine
TMP: 2,2,6,6-tetramethylpiperidinyl-magnesium chloride lithium chloride complex (Knochel–Hauser base)
TMSCl: trimethylsilyl chloride
TOF: time-of-flight
Tol: *p*-Tol, *para*-tolyl
TPA: two-photon absorption
TTF: tetrathiafulvalene
*p*-TSA: *p*-toluenesulfonic acid
XRD: X-ray diffraction

## References

[ref1] Stępień M.; Gońka E.; Żyła M.; Sprutta N. Heterocyclic Nanographenes and Other Polycyclic Heteroaromatic Compounds: Synthetic Routes, Properties, and Applications. Chem. Rev. 2017, 117, 3479–3716. 10.1021/acs.chemrev.6b00076. 27258218

[ref2] Ammon F.; Sauer S. T.; Lippert R.; Lungerich D.; Reger D.; Hampel F.; Jux N. Unexpected Formation of [5]Helicenes from Hexaarylbenzenes Containing Pyrrole Moieties. Org. Chem. Front. 2017, 4, 861–870. 10.1039/C7QO00112F.

[ref3] Reger D.; Schöll K.; Hampel F.; Maid H.; Jux N. Pyridinic Nanographenes by Novel Precursor Design. Chem. - Eur. J. 2021, 27, 1984–1989. 10.1002/chem.202004983. 33225488 PMC7898602

[ref4] Schulze M.; Philipp M.; Waigel W.; Schmidt D.; Würthner F. Library of Azabenz-Annulated Core-Extended Perylene Derivatives with Diverse Substitution Patterns and Tunable Electronic and Optical Properties. J. Org. Chem. 2016, 81, 8394–8405. 10.1021/acs.joc.6b01573. 27568658

[ref5] Greßies S.; Ito M.; Sakai M.; Osaki H.; Kim J. H.; Gensch T.; Daniliuc C.; Ando N.; Yamaguchi S.; Glorius F. Twofold C-H Activation Enables Synthesis of a Diazacoronene-Type Fluorophore with Near Infrared Emission Through Isosteric Replacement. Chem. - Eur. J. 2021, 27, 2753–2759. 10.1002/chem.202004080. 33085826

[ref6] Delaney C.; Ó Máille G. M.; Twamley B.; Draper S. M. One-Pot, High-Yielding, Oxidative Cyclodehydrogenation Route for N-Doped Nanographene Synthesis. Org. Lett. 2016, 18, 88–91. 10.1021/acs.orglett.5b03312. 26683976

[ref7] Wecławski M. K.; Jakešová M.; Charyton M.; Demitri N.; Koszarna B.; Oppelt K.; Sariciftci S.; Gryko D. T.; Głowacki E. D. Biscoumarin-Containing Acenes as Stable Organic Semiconductors for Photocatalytic Oxygen Reduction to Hydrogen Peroxide. J. Mater. Chem. A 2017, 5, 20780–20788. 10.1039/C7TA05882A. PMC11976386 40213317

[ref8] Wecławski M. K.; Deperasińska I.; Banasiewicz M.; Young D. C.; Leniak A.; Gryko D. T. Building Molecular Complexity from Quinizarin: Conjoined Coumarins and Coronene Analogs. Chem. - Asian J. 2019, 14, 1763–1770. 10.1002/asia.201800757. 30022613

[ref9] Zhao Y.; Zhang Q.; Chen K.; Gao H.; Qi H.; Shi X.; Han Y.; Wei J.; Zhang C. Triphenothiazinyl Triazacoronenes: Donor-Acceptor Molecular Graphene Exhibiting Multiple Fluorescence and Electrogenerated Chemiluminescence Emissions. J. Mater. Chem. C 2017, 5, 4293–4301. 10.1039/C7TC00314E.

[ref10] Liu B.; Shi D.; Yang Y.; Liu D.; Li M.; Liu E.; Wang X.; Zhang Q.; Yang M.; Li J.; et al. Triazacoronene Derivatives with Three *Peri* -Benzopyrano Extensions: Synthesis, Structure, and Properties: Triazacoronene Derivatives with Three *Peri* -Benzopyrano Extensions: Synthesis, Structure, and Properties. Eur. J. Org. Chem. 2018, 2018, 869–873. 10.1002/ejoc.201701386.

[ref11] Sun Y.-X.; Wang X.-G.; Shen G.-D.; Yang T.; Yang Y.-H.; Li J.; Yang M.-Y.; Sun H.-M.; Wei J.-F. A 6π Azaelectrocyclization Strategy towards the 1,5,9-Triazacoronenes. Adv. Synth. Catal. 2020, 362, 1651–1656. 10.1002/adsc.201901433.

[ref12] Lee J.; Buyukcakir O.; Kwon T.; Coskun A. Energy Band-Gap Engineering of Conjugated Microporous Polymers via Acidity-Dependent in Situ Cyclization. J. Am. Chem. Soc. 2018, 140, 10937–10940. 10.1021/jacs.8b05978. 30089358

[ref13] Duan R.; Schollmeyer D.; Müllen K.; Li C. From Anthraquinone to Heterocoronene as Stable Red Chromophore. J. Mater. Chem. C 2018, 6, 1334–1337. 10.1039/C7TC05073A.

[ref14] De J.; Bala I.; Gupta S. P.; Pandey U. K.; Pal S. K. High Hole Mobility and Efficient Ambipolar Charge Transport in Heterocoronene-Based Ordered Columnar Discotics. J. Am. Chem. Soc. 2019, 141, 18799–18805. 10.1021/jacs.9b09126. 31682432

[ref15] Wijesinghe L. P.; Perera S. D.; Larkin E.; O Maille G. M.; Conway-Kenny R.; Lankage B. S.; Wang L.; Draper S. M. [2 + 2 + 2] Cyclotrimerisation as a Convenient Route to 6N-Doped Nanographenes: A Synthetic Introduction to Hexaazasuperbenzenes. RSC Adv. 2017, 7, 24163–24167. 10.1039/C7RA02648J.

[ref16] Oki K.; Takase M.; Kobayashi N.; Uno H. Synthesis and Characterization of Peralkylated Pyrrole-Fused Azacoronene. J. Org. Chem. 2021, 86, 5102–5109. 10.1021/acs.joc.0c03042. 33621082

[ref17] Sasaki Y.; Takase M.; Mori S.; Uno H. Synthesis and Properties of NitroHPHAC: The First Example of Substitution Reaction on HPHAC. Molecules 2020, 25, 2486 10.3390/molecules25112486. PMC7321187 32471200

[ref18] Uno H.; Ishiwata M.; Muramatsu K.; Takase M.; Mori S.; Okujima T. Oxidation Behavior of 1,3-Dihydrothieno[3,4-a]HPHAC. Bull. Chem. Soc. Jpn. 2019, 92, 973–981. 10.1246/bcsj.20190022.

[ref19] Sasaki Y.; Takase M.; Okujima T.; Mori S.; Uno H. Synthesis and Redox Properties of Pyrrole- and Azulene-Fused Azacoronene. Org. Lett. 2019, 21, 1900–1903. 10.1021/acs.orglett.9b00515. 30835482

[ref20] Oki K.; Takase M.; Mori S.; Shiotari A.; Sugimoto Y.; Ohara K.; Okujima T.; Uno H. Synthesis, Structures, and Properties of Core-Expanded Azacoronene Analogue: A Twisted π-System with Two N-Doped Heptagons. J. Am. Chem. Soc. 2018, 140, 10430–10434. 10.1021/jacs.8b06079. 30068084

[ref21] Oki K.; Takase M.; Mori S.; Uno H. Synthesis and Isolation of Antiaromatic Expanded Azacoronene via Intramolecular Vilsmeier-Type Reaction. J. Am. Chem. Soc. 2019, 141, 16255–16259. 10.1021/jacs.9b09260. 31577141

[ref22] Zhylitskaya H.; Cybińska J.; Chmielewski P.; Lis T.; Stępień M. Bandgap Engineering in π-Extended Pyrroles. a Modular Approach to Electron-Deficient Chromophores with Multi-Redox Activity. J. Am. Chem. Soc. 2016, 138, 11390–11398. 10.1021/jacs.6b07826. 27533895

[ref23] Żyła-Karwowska M.; Zhylitskaya H.; Cybińska J.; Lis T.; Chmielewski P. J.; Stepień M. An Electron-Deficient Azacoronene Obtained by Radial π Extension. Angew. Chem., Int. Ed. 2016, 55, 14658–14662. 10.1002/anie.201608400. 27739647

[ref24] Navakouski M.; Zhylitskaya H.; Chmielewski P. J.; Lis T.; Cybińska J.; Stepień M. Stereocontrolled Synthesis of Chiral Heteroaromatic Propellers with Small Optical Bandgaps. Angew. Chem., Int. Ed. 2019, 58, 4929–4933. 10.1002/anie.201900175. 30714666

[ref25] Navakouski M.; Zhylitskaya H.; Chmielewski P. J.; Żła-Karwowska M.; Stępień M. Electrophilic Aromatic Coupling of Hexapyrrolylbenzenes. A Mechanistic Analysis. J. Org. Chem. 2020, 85, 187–194. 10.1021/acs.joc.9b02556. 31694376

[ref26] Sasaki Y.; Takase M.; Kobayashi N.; Mori S.; Ohara K.; Okujima T.; Uno H. Radially π-Extended Pyrrole-Fused Azacoronene: A Series of Crystal Structures of HPHAC with Various Oxidation States. J. Org. Chem. 2021, 86, 4290–4295. 10.1021/acs.joc.0c02825. 33522813

[ref27] Moshniaha L.; Żyła-Karwowska M.; Chmielewski P. J.; Lis T.; Cybińska J.; Gońka E.; Oschwald J.; Drewello T.; Rivero S. M.; Casado J.; et al. Aromatic Nanosandwich Obtained by σ-Dimerization of a Nanographenoid π-Radical. J. Am. Chem. Soc. 2020, 142, 3626–3635. 10.1021/jacs.9b13942. 31997634 PMC7467677

[ref28] Wang X.-Y.; Richter M.; He Y.; Björk J.; Riss A.; Rajesh R.; Garnica M.; Hennersdorf F.; Weigand J. J.; Narita A.; et al. Exploration of Pyrazine-Embedded Antiaromatic Polycyclic Hydrocarbons Generated by Solution and on-Surface Azomethine Ylide Homocoupling. Nat. Commun. 2017, 8, 1948 10.1038/s41467-017-01934-1. 29208962 PMC5717246

[ref29] Wang X.-Y.; Narita A.; Zhang W.; Feng X.; Müllen K. Synthesis of Stable Nanographenes with OBO-Doped Zigzag Edges Based on Tandem Demethylation-Electrophilic Borylation. J. Am. Chem. Soc. 2016, 138, 9021–9024. 10.1021/jacs.6b04092. 27374883

[ref30] Cloke R. R.; Marangoni T.; Nguyen G. D.; Joshi T.; Rizzo D. J.; Bronner C.; Cao T.; Louie S. G.; Crommie M. F.; Fischer F. R. Site-Specific Substitutional Boron Doping of Semiconducting Armchair Graphene Nanoribbons. J. Am. Chem. Soc. 2015, 137, 8872–8875. 10.1021/jacs.5b02523. 26153349

[ref31] Carbonell-Sanromà E.; Garcia-Lekue A.; Corso M.; Vasseur G.; Brandimarte P.; Lobo-Checa J.; de Oteyza D. G.; Li J.; Kawai S.; Saito S.; et al. Electronic Properties of Substitutionally Boron-Doped Graphene Nanoribbons on a Au(111) Surface. J. Phys. Chem. C 2018, 122, 16092–16099. 10.1021/acs.jpcc.8b03748.

[ref32] Min Y.; Dou C.; Tian H.; Liu J.; Wang L. A Disk-Type Polyarene Containing Four B-N Units. Chem. Commun. 2019, 55, 3638–3641. 10.1039/C9CC00769E. 30849151

[ref33] Dosso J.; Tasseroul J.; Fasano F.; Marinelli D.; Biot N.; Fermi A.; Bonifazi D. Synthesis and Optoelectronic Properties of Hexa-Peri-Hexabenzoborazinocoronene. Angew. Chem., Int. Ed. 2017, 56, 4483–4487. 10.1002/anie.201700907. 28323375

[ref34] Fresta E.; Dosso J.; Cabanillas-González J.; Bonifazi D.; Costa R. D. Origin of the Exclusive Ternary Electroluminescent Behavior of BN-Doped Nanographenes in Efficient Single-Component White Light-Emitting Electrochemical Cells. Adv. Funct. Mater. 2020, 30, 1906830 10.1002/adfm.201906830.

[ref35] Dosso J.; Battisti T.; Ward B. D.; Demitri N.; Hughes C. E.; Williams P. A.; Harris K. D. M.; Bonifazi D. Boron-Nitrogen-Doped Nanographenes: A Synthetic Tale from Borazine Precursors. Chem. - Eur. J. 2020, 26, 6608–6621. 10.1002/chem.201905794. 32023358

[ref36] Matsui K.; Oda S.; Yoshiura K.; Nakajima K.; Yasuda N.; Hatakeyama T. One-Shot Multiple Borylation toward BN-Doped Nanographenes. J. Am. Chem. Soc. 2018, 140, 1195–1198. 10.1021/jacs.7b10578. 29120174

[ref37] Guo X.; Yuan Z.; Zhu Y.; Li Z.; Huang R.; Xia Z.; Zhang W.; Li Y.; Wang J. A Nitrogen-Doped Hexapole [7]Helicene versus Its All-Carbon Analogue. Angew. Chem., Int. Ed. 2019, 58, 16966–16972. 10.1002/anie.201907972. 31407458

[ref38] Reger D.; Haines P.; Heinemann F. W.; Guldi D. M.; Jux N. Oxa[7]Superhelicene: A π-Extended Helical Chromophore Based on Hexa-Peri-Hexabenzocoronenes. Angew. Chem., Int. Ed. 2018, 57, 5938–5942. 10.1002/anie.201800585. 29508521

[ref39] Wade J.; Brandt J. R.; Reger D.; Zinna F.; Amsharov K. Y.; Jux N.; Andrews D. L.; Fuchter M. J. 500-Fold Amplification of Small Molecule Circularly Polarised Luminescence through Circularly Polarised FRET. Angew. Chem., Int. Ed. 2021, 60, 222–227. 10.1002/anie.202011745. PMC7839560 33030274

[ref40] Dong R.; Pfeffermann M.; Skidin D.; Wang F.; Fu Y.; Narita A.; Tommasini M.; Moresco F.; Cuniberti G.; Berger R.; et al. Persulfurated Coronene: A New Generation of “Sulflower. J. Am. Chem. Soc. 2017, 139, 2168–2171. 10.1021/jacs.6b12630. 28128953

[ref41] Yin J.; Hu Y.; Zhang D.; Li X.; Jin W. Synthesis and Characterization of Symmetrical Sulfur-Fused Polycyclic Aromatic Hydrocarbons with Controlled Shapes. Tetrahedron 2017, 73, 5794–5799. 10.1016/j.tet.2017.08.020.

[ref42] Qiao X.; Li Q.; Schaugaard R. N.; Noffke B. W.; Liu Y.; Li D.; Liu L.; Raghavachari K.; Li L. Well-Defined Nanographene-Rhenium Complex as an Efficient Electrocatalyst and Photocatalyst for Selective CO2 Reduction. J. Am. Chem. Soc. 2017, 139, 3934–3937. 10.1021/jacs.6b12530. 28271885

[ref43] Endres A. H.; Schaffroth M.; Paulus F.; Reiss H.; Wadepohl H.; Rominger F.; Krämer R.; Bunz U. H. F. Coronene-Containing N-Heteroarenes: 13 Rings in a Row. J. Am. Chem. Soc. 2016, 138, 1792–1795. 10.1021/jacs.5b12642. 26808212

[ref44] Kojima M.; Tamoto A.; Aratani N.; Yamada H. Rearrangement of an Aniline Linked Perylene Bisimide under Acidic Conditions and Visible to Near-Infrared Emission from the Intramolecular Charge-Transfer State of Its Fused Derivatives. Chem. Commun. 2017, 53, 5698–5701. 10.1039/C7CC01520H. 28447685

[ref45] Yang X.; Rominger F.; Mastalerz M. Cata-Condensed Heteroannulated Coronenes via Selective Bromination of Diarenoperylenes as the Key Step. Org. Lett. 2018, 20, 7270–7273. 10.1021/acs.orglett.8b03181. 30394096

[ref46] Davy N. C.; Man G.; Kerner R. A.; Fusella M. A.; Purdum G. E.; Sezen M.; Rand B. P.; Kahn A.; Loo Y.-L. Contorted Hexabenzocoronenes with Extended Heterocyclic Moieties Improve Visible-Light Absorption and Performance in Organic Solar Cells. Chem. Mater. 2016, 28, 673–681. 10.1021/acs.chemmater.5b04503.

[ref47] Urieta-Mora J.; Krug M.; Alex W.; Perles J.; Fernández I.; Molina-Ontoria A.; Guldi D. M.; Martín N. Homo and Hetero Molecular 3D Nanographenes Employing a Cyclooctatetraene Scaffold. J. Am. Chem. Soc. 2020, 142, 4162–4172. 10.1021/jacs.9b10203. 31859500

[ref48] Liu Y.; Marszalek T.; Müllen K.; Pisula W.; Feng X. Derivatizing Tribenzothiophene-Fused Hexa-Peri-Hexabenzocoronenes with Tunable Optoelectronic Properties. Chem. - Asian J. 2016, 11, 2107–2112. 10.1002/asia.201600753. 27348404

[ref49] Dusold C.; Haines P.; Platzer B.; Guldi D. M.; Hirsch A. Helically and Linearly Fused Rylenediimide-Hexabenzocoronenes. Chem. - Eur. J. 2021, 27, 6511–6521. 10.1002/chem.202005235. 33492668 PMC8252035

[ref50] Gu X.; Wang H.; Roose J.; He Z.; Zhou Y.; Yan Y.; Cai Y.; Shi H.; Zhang Y.; Sung H. H. Y.; et al. A Luminescent Nitrogen-Containing Polycyclic Aromatic Hydrocarbon Synthesized by Photocyclodehydrogenation with Unprecedented Regioselectivity. Chem. - Eur. J. 2015, 21, 17973–17980. 10.1002/chem.201503147. 26490877

[ref51] Raouafi S.; Aloui F. New Functional Benzo[Ghi]Perylene Derivatives: Synthesis and Photophysical Properties. J. Mol. Struct. 2019, 1196, 685–690. 10.1016/j.molstruc.2019.07.022.

[ref52] Raouafi S.; Aloui F. Synthesis and Photophysical Properties of New Nitrile Grafted Benzo[Ghi]Perylene Derivatives. J. Mol. Struct. 2019, 1195, 153–160. 10.1016/j.molstruc.2019.05.100.

[ref53] Pigulski B.; Ximenis M.; Shoyama K.; Würthner F. Synthesis of Polycyclic Aromatic Hydrocarbons by Palladium-Catalysed [3 + 3] Annulation. Org. Chem. Front. 2020, 7, 2925–2930. 10.1039/D0QO00968G.

[ref54] Tasior M.; Deperasińska I.; Morawska K.; Banasiewicz M.; Vakuliuk O.; Kozankiewicz B.; Gryko D. T. Vertically π-Expanded Coumarin - Synthesis via the Scholl Reaction and Photophysical Properties. Phys. Chem. Chem. Phys. 2014, 16, 18268–18275. 10.1039/C4CP02003K. 25058341

[ref55] Xue W.; Wang D.; Li C.; Zhai Z.; Wang T.; Liang Y.; Zhang Z. π-Expanded Coumarins: One-Pot Photo Synthesis of 5 *H* -Benzo[12,1]Tetrapheno[7,6,5- *Cde* ]Chromen-5-Ones and Photophysical Properties. J. Org. Chem. 2020, 85, 3689–3698. 10.1021/acs.joc.9b03327. 31973520

[ref56] Rosenberg M.; Santella M.; Bogh S. A.; Muñoz A. V.; Andersen H. O. B.; Hammerich O.; Bora I.; Lincke K.; Laursen B. W. Extended Triangulenium Ions: Syntheses and Characterization of Benzo-Bridged Dioxa- and Diazatriangulenium Dyes. J. Org. Chem. 2019, 84, 2556–2567. 10.1021/acs.joc.8b02978. 30694674

[ref57] Lewis B. W.; Bisballe N.; Santella M.; Summers P. A.; Vannier J.-B.; Kuimova M. K.; Laursen B. W.; Vilar R. Assessing The Key Photophysical Properties of Triangulenium Dyes for DNA Binding by Alteration of the Fluorescent Core. Chem. - Eur. J. 2021, 27, 2523–2536. 10.1002/chem.202003875. 33105523

[ref58] Escande A.; Crossley D. L.; Cid J.; Cade I. A.; Vitorica-Yrezabal I.; Ingleson M. J. Inter- and Intra-Molecular C-H Borylation for the Formation of PAHs Containing Triarylborane and Indole Units. Dalton Trans. 2016, 45, 17160–17167. 10.1039/C6DT03526D. 27722680

[ref59] Del Grosso A.; Ayuso Carrillo J.; Ingleson M. J. Regioselective Electrophilic Borylation of Haloarenes. Chem. Commun. 2015, 51, 2878–2881. 10.1039/C4CC10153G. 25582820

[ref60] Farrell J. M.; Schmidt D.; Grande V.; Würthner F. Synthesis of a Doubly Boron-Doped Perylene through NHC-Borenium Hydroboration/C-H Borylation/Dehydrogenation. Angew. Chem., Int. Ed. 2017, 56, 11846–11850. 10.1002/anie.201706346. 28741895

[ref61] Farrell J. M.; Mützel C.; Bialas D.; Rudolf M.; Menekse K.; Krause A.-M.; Stolte M.; Würthner F. Tunable Low-LUMO Boron-Doped Polycyclic Aromatic Hydrocarbons by General One-Pot C-H Borylations. J. Am. Chem. Soc. 2019, 141, 9096–9104. 10.1021/jacs.9b04675. 31117551

[ref62] Farrell J. M.; Grande V.; Schmidt D.; Würthner F. A Highly Warped Heptagon-Containing Sp^2^ Carbon Scaffold via Vinylnaphthyl Π-Extension. Angew. Chem., Int. Ed. 2019, 58, 16504–16507. 10.1002/anie.201909975. PMC6900032 31507020

[ref63] Radtke J.; Schickedanz K.; Bamberg M.; Menduti L.; Schollmeyer D.; Bolte M.; Lerner H.-W.; Wagner M. Selective Access to Either a Doubly Boron-Doped Tetrabenzopentacene or an Oxadiborepin from the Same Precursor. Chem. Sci. 2019, 10, 9017–9027. 10.1039/C9SC03115D. 32874487 PMC7442282

[ref64] Yi Z.; Okuda H.; Koyama Y.; Seto R.; Uchida S.; Sogawa H.; Kuwata S.; Takata T. Exact Helical Polymer Synthesis by a Two-Point-Covalent-Linking Protocol between C2-Chiral Spirobifluorene and C2- or Cs-Symmetric Anthraquinone Monomers. Chem. Commun. 2015, 51, 10423–10426. 10.1039/C5CC02086G. 26028037

[ref65] Tran M. N.; Rarig R.-A. F.; Chenoweth D. M. Synthesis and Properties of Lysosome-Specific Photoactivatable Probes for Live-Cell Imaging. Chem. Sci. 2015, 6, 4508–4512. 10.1039/C5SC01601K. 28496967 PMC5299591

[ref66] Tran M. N.; Chenoweth D. M. Photoelectrocyclization as an Activation Mechanism for Organelle-Specific Live-Cell Imaging Probes. Angew. Chem., Int. Ed. 2015, 54, 6442–6446. 10.1002/anie.201502403. PMC4485560 25950154

[ref67] Zhang S.; Liu Z.; Fang Q. Synthesis, Structures, and Optoelectronic Properties of Pyrene-Fused Thioxanthenes. Org. Lett. 2017, 19, 1382–1385. 10.1021/acs.orglett.7b00276. 28234489

[ref68] Sato K.; Seki Y.; Suga S.; Ikeda Y.; Yamaguchi M. Reinvestigation of the Photoreaction of 1,4-Bis(2,4,6-Triphenylpyridinio)Benzene: Synthesis of a Diazonia Derivative of Hexabenzoperylene by Multiple Photocyclization. J. Photochem. Photobiol., A 2016, 331, 8–16. 10.1016/j.jphotochem.2016.06.022.

[ref69] Park Y. S.; Dibble D. J.; Kim J.; Lopez R. C.; Vargas E.; Gorodetsky A. A. Synthesis of Nitrogen-Containing Rubicene and Tetrabenzopentacene Derivatives. Angew. Chem., Int. Ed. 2016, 55, 3352–3355. 10.1002/anie.201510320. 26834003

[ref70] Tokuo K.; Sakai H.; Sakanoue T.; Takenobu T.; Araki Y.; Wada T.; Hasobe T. Control of the Electrochemical and Photophysical Properties of N-Substituted Benzo[Ghi]Perylene Derivatives. Mater. Chem. Front. 2017, 1, 2299–2308. 10.1039/C7QM00301C.

[ref71] Evoniuk C. J.; Gomes G.; dos P.; Hill S. P.; Fujita S.; Hanson K.; Alabugin I. V. Coupling N-H Deprotonation, C-H Activation, and Oxidation: Metal-Free C(Sp3)-H Aminations with Unprotected Anilines. J. Am. Chem. Soc. 2017, 139, 16210–16221. 10.1021/jacs.7b07519. 29037029

[ref72] Langhals H.; Reichherzer S.; Mayer P.; Polborn K. A Three-Step Synthesis of 1,7-Diazaperylene and Derivatives. Synthesis 2021, 53, 713–722. 10.1055/s-0040-1707293.

[ref73] Okamoto T.; Kumagai S.; Fukuzaki E.; Ishii H.; Watanabe G.; Niitsu N.; Annaka T.; Yamagishi M.; Tani Y.; Sugiura H.; et al. Robust, High-Performance n-Type Organic Semiconductors. Sci. Adv. 2020, 6, eaaz0632 10.1126/sciadv.aaz0632. 32494668 PMC7195148

[ref74] Kumagai S.; Ishii H.; Watanabe G.; Annaka T.; Fukuzaki E.; Tani Y.; Sugiura H.; Watanabe T.; Kurosawa T.; Takeya J.; et al. Cooperative Aggregations of Nitrogen-Containing Perylene Diimides Driven by Rigid and Flexible Functional Groups. Chem. Mater. 2020, 32, 9115–9125. 10.1021/acs.chemmater.0c01888.

[ref75] Sawada T.; Makita T.; Yamamura A.; Sasaki M.; Yoshimura Y.; Hayakawa T.; Okamoto T.; Watanabe S.; Kumagai S.; Takeya J. Low-Voltage Complementary Inverters Using Solution-Processed, High-Mobility Organic Single-Crystal Transistors Fabricated by Polymer-Blend Printing. Appl. Phys. Lett. 2020, 117, 033301 10.1063/5.0006651.

[ref76] Zagranyarski Y.; Skabeev A.; Ma Y.; Müllen K.; Li C. Facile Synthesis of Annulated Heterocyclic Benzo[Kl]Acridine Derivatives via One-Pot N-H/C-H Coupling. Org. Chem. Front. 2016, 3, 1520–1523. 10.1039/C6QO00371K.

[ref77] Dorđevic L.; Valentini C.; Demitri N.; Meziere C.; Allain M.; Salle M.; Folli A.; Murphy D.; Manas-Valero S.; Coronado E.; Bonifazi D.; et al. O-Doped Nanographenes: A Pyrano/Pyrylium Route Towards Semiconducting Cationic Mixed-Valence Complexes. Angew. Chem., Int. Ed. 2020, 59, 4106–4114. 10.1002/anie.201914025. 31889372

[ref78] Okamoto Y.; Tanioka M.; Muranaka A.; Miyamoto K.; Aoyama T.; Ouyang X.; Kamino S.; Sawada D.; Uchiyama M. Stable Thiele’s Hydrocarbon Derivatives Exhibiting Near-Infrared Absorption/Emission and Two-Step Electrochromism. J. Am. Chem. Soc. 2018, 140, 17857–17861. 10.1021/jacs.8b11092. 30507181

[ref79] Harada M.; Tanioka M.; Muranaka A.; Aoyama T.; Kamino S.; Uchiyama M. A Remarkably Air-Stable Quinodimethane Radical Cation. Chem. Commun. 2020, 56, 9565–9568. 10.1039/D0CC04025H. 32691766

[ref80] Hirono A.; Sakai H.; Hasobe T. Synthesis and Electrochemical and Photophysical Properties of Azaterrylene Derivatives. Chem. - Asian J. 2019, 14, 1754–1762. 10.1002/asia.201801410. 30378763

[ref81] Hirono A.; Sakai H.; Kochi S.; Sato T.; Hasobe T. Electrochemical Properties and Excited-State Dynamics of Azaperylene Derivatives. J. Phys. Chem. B 2020, 124, 9921–9930. 10.1021/acs.jpcb.0c07532. 33085485

[ref82] Stassen D.; Demitri N.; Bonifazi D. Extended O-Doped Polycyclic Aromatic Hydrocarbons. Angew. Chem., Int. Ed. 2016, 55, 5947–5951. 10.1002/anie.201509517. PMC5071652 27062492

[ref83] Katayama T.; Nakatsuka S.; Hirai H.; Yasuda N.; Kumar J.; Kawai T.; Hatakeyama T. Two-Step Synthesis of Boron-Fused Double Helicenes. J. Am. Chem. Soc. 2016, 138, 5210–5213. 10.1021/jacs.6b01674. 27077723

[ref84] Wang X.-Y.; Wang X.-C.; Narita A.; Wagner M.; Cao X.-Y.; Feng X.; Müllen K. Synthesis, Structure, and Chiroptical Properties of a Double [7]Heterohelicene. J. Am. Chem. Soc. 2016, 138, 12783–12786. 10.1021/jacs.6b08664. 27658004

[ref85] Zhou Z.; Wang X.-Y.; Wei Z.; Müllen K.; Petrukhina M. A. Charging OBO-Fused Double [5]Helicene with Electrons. Angew. Chem., Int. Ed. 2019, 58, 14969–14973. 10.1002/anie.201908658. PMC6916263 31430019

[ref86] Ikeda N.; Oda S.; Matsumoto R.; Yoshioka M.; Fukushima D.; Yoshiura K.; Yasuda N.; Hatakeyama T. Solution-Processable Pure Green Thermally Activated Delayed Fluorescence Emitter Based on the Multiple Resonance Effect. Adv. Mater. 2020, 32, 2004072 10.1002/adma.202004072. 32864797

[ref87] Scholz A. S.; Massoth J. G.; Bursch M.; Mewes J.-M.; Hetzke T.; Wolf B.; Bolte M.; Lerner H.-W.; Grimme S.; Wagner M. BNB-Doped Phenalenyls: Modular Synthesis, Optoelectronic Properties, and One-Electron Reduction. J. Am. Chem. Soc. 2020, 142, 11072–11083. 10.1021/jacs.0c03118. 32464052

[ref88] Kaehler T.; Bolte M.; Lerner H.-W.; Wagner M. Introducing Perylene as a New Member to the Azaborine Family. Angew. Chem., Int. Ed. 2019, 58, 11379–11384. 10.1002/anie.201905823. 31141261

[ref89] Sun K.; Sugawara K.; Lyalin A.; Ishigaki Y.; Uosaki K.; Taketsugu T.; Suzuki T.; Kawai S. Heterocyclic Ring-Opening of Nanographene on Au(111). Angew. Chem., Int. Ed. 2021, 60, 9427–9432. 10.1002/anie.202017137. 33576120

[ref90] Sakamaki D.; Kumano D.; Yashima E.; Seki S. A Double Hetero[4]Helicene Composed of Two Phenothiazines: Synthesis, Structural Properties, and Cationic States. Chem. Commun. 2015, 51, 17237–17240. 10.1039/C5CC07680C. 26459965

[ref91] Shindo Y.; Nomura S.; Saikawa Y.; Nakata M.; Tanaka K.; Hanaya K.; Sugai T.; Higashibayashi S. Synthesis and Properties of Hydrazine-Embedded Biphenothiazines and Application of Hydrazine-Embedded Heterocyclic Compounds to Fluorescence Cell Imaging. Asian J. Org. Chem. 2018, 7, 1797–1801. 10.1002/ajoc.201800364.

[ref92] Zhu G.; Song Y.; Zhang Q.; Ding W.; Chen X.; Wang Y.; Zhang G. Modulating the Properties of Buckybowls Containing Multiple Heteroatoms. Org. Chem. Front. 2021, 8, 727–735. 10.1039/D0QO01452D.

[ref93] Gupta R. K.; Pathak S. K.; Pradhan B.; Rao D. S. S.; Prasad S. K.; Achalkumar A. S. Self-Assembly of Luminescent N-Annulated Perylene Tetraesters into Fluid Columnar Phases. Soft Matter 2015, 11, 3629–3636. 10.1039/C5SM00463B. 25812168

[ref94] Gupta R. K.; Pradhan B.; Pathak S. K.; Gupta M.; Pal S. K.; Ammathnadu Sudhakar A. Perylo[1,12- *b*, *c*, *d* ] Thiophene Tetraesters: A New Class of Luminescent Columnar Liquid Crystals. Langmuir 2015, 31, 8092–8100. 10.1021/acs.langmuir.5b01187. 26077109

[ref95] Gupta R. K.; Pathak S. K.; Pradhan B.; Gupta M.; Pal S. K.; Sudhakar A. A. Bay-Annulated Perylene Tetraesters: A New Class of Discotic Liquid Crystals. ChemPhysChem 2016, 17, 859–872. 10.1002/cphc.201501028. 26748430

[ref96] Gupta R. K.; Das D.; Gupta M.; Pal S. K.; Iyer P. K.; Achalkumar A. S. Electroluminescent Room Temperature Columnar Liquid Crystals Based on Bay-Annulated Perylene Tetraesters. J. Mater. Chem. C 2017, 5, 1767–1781. 10.1039/C6TC04166C.

[ref97] Gupta R. K.; Shankar Rao D. S.; Prasad S. K.; Achalkumar A. S. Columnar Self-Assembly of Electron-Deficient Dendronized Bay-Annulated Perylene Bisimides. Chem. - Eur. J. 2018, 24, 3566–3575. 10.1002/chem.201705290. 29283196

[ref98] Gupta R. K.; Achalkumar A. S. Microwave-Assisted Method for the Synthesis of Perylene Ester Imides as a Gateway Toward Unsymmetrical Perylene Bisimides. J. Org. Chem. 2018, 83, 6290–6300. 10.1021/acs.joc.8b00161. 29772902

[ref99] Chintala S. M.; Petroff II J. T.; Barnes A.; McCulla R. D. Photodeoxygenation of Phenanthro[4,5-Bcd]Thiophene S-Oxide, Triphenyleno[1,12-Bcd]Thiophene S-Oxide and Perylo[1,12-Bcd]Thiophene S-Oxide. J. Sulfur Chem. 2019, 40, 503–515. 10.1080/17415993.2019.1615065.

[ref100] Xing L.; Jiang W.; Huang Z.; Liu J.; Song H.; Zhao W.; Dai J.; Zhu H.; Wang Z.; Weiss P. S.; et al. Steering Two-Dimensional Porous Networks with σ-Hole Interactions of Br···S and Br···Br. Chem. Mater. 2019, 31, 3041–3048. 10.1021/acs.chemmater.9b01126.

[ref101] Madhu S.; Evans H. A.; Doan-Nguyen V. V. T.; Labram J. G.; Wu G.; Chabinyc M. L.; Seshadri R.; Wudl F. Infinite Polyiodide Chains in the Pyrroloperylene-Iodine Complex: Insights into the Starch-Iodine and Perylene-Iodine Complexes. Angew. Chem., Int. Ed. 2016, 55, 8032–8035. 10.1002/anie.201601585. 27239781

[ref102] Santoni M.-P.; Santoro A.; Salerno T. M. G.; Puntoriero F.; Nastasi F.; Di Pietro M. L.; Galletta M.; Campagna S. Photoinduced Charge Separation in a Donor-Spacer-Acceptor Dyad with N-Annulated Perylene Donor and Methylviologen Acceptor. ChemPhysChem 2015, 16, 3147–3150. 10.1002/cphc.201500615. 26331881

[ref103] Rocard L.; Hatych D.; Chartier T.; Cauchy T.; Hudhomme P. Original Suzuki-Miyaura Coupling Using Nitro Derivatives for the Synthesis of Perylenediimide-Based Multimers. Eur. J. Org. Chem. 2019, 2019, 7635–7643. 10.1002/ejoc.201901319.

[ref104] Ma Y.; Shi Z.; Zhang A.; Li J.; Wei X.; Jiang T.; Li Y.; Wang X. Self-Assembly, Optical and Electrical Properties of Five Membered O- or S-Heterocyclic Annulated Perylene Diimides. Dyes Pigm. 2016, 135, 41–48. 10.1016/j.dyepig.2016.06.027.

[ref105] Li W.; Wang Q.; Ma Y.; Jiang T.; Zhu Y.; Shao Y.; Sun C.; Wu J. Photophysical Property, Electronic Structure and Solid-State Packing of O-Heterocyclic Annulated Perylene Diimide. Pigm. Resin Technol. 2019, 48, 256–262. 10.1108/PRT-04-2018-0034.

[ref106] Zhang F.; Huang X.; Wei X.; Ren H.; Jiang T.; Li X.; Wu J.; Ma Y. Synthesis and Properties of Bay Unilaterally Extended and Mono-Substituted Perylene Diimides. J. Chem. Res. 2020, 44, 60–66. 10.1177/1747519819886502.

[ref107] Deng Q.; Zhou E.; Huang Y.; Qing W.; Zhai H.; Liu Z.; Wei Z. Chalcogen-Substitution Modulated Supramolecular Chirality and Gas Sensing Properties in Perylenediimides. Chem. Commun. 2019, 55, 4379–4382. 10.1039/C9CC01443H. 30916083

[ref108] Chen L.; Xia P.; Du T.; Deng Y.; Xiao Y. Catalyst-Free One-Pot Synthesis of Unsymmetrical Five- and Six-Membered Sulfur-Annulated Heterocyclic Perylene Diimides for Electron-Transporting Property. Org. Lett. 2019, 21, 5529–5532. 10.1021/acs.orglett.9b01847. 31246035

[ref109] Li G.; Li D.; Liu X.; Xu H.; Zhang J.; Wang S.; Liu Z.; Tang B. Novel Dithiano-Thieno Fused Perylene Diimides: Synthesis, Characterization and Application in Organic Thin-Film Transistors (OTFTs). Chem. Commun. 2019, 55, 9661–9664. 10.1039/C9CC04133H. 31342019

[ref110] Wang R.; Li G.; Zhou Y.; Hao P.; Shang Q.; Wang S.; Zhang Y.; Li D.; Yang S.; Zhang Q.; et al. Facile Syntheses, Characterization, and Physical Properties of Sulfur-Decorated Pyran-Annulated Perylene Diimides. Asian J. Org. Chem. 2018, 7, 702–706. 10.1002/ajoc.201800011.

[ref111] Li X.; Wang H.; Nakayama H.; Wei Z.; Schneider J. A.; Clark K.; Lai W.-Y.; Huang W.; Labram J. G.; de Alaniz J. R.; et al. Multi-Sulfur-Annulated Fused Perylene Diimides for Organic Solar Cells with Low Open-Circuit Voltage Loss. ACS Appl. Energy Mater. 2019, 2, 3805–3814. 10.1021/acsaem.9b00492.

[ref112] Li X.; Wang H.; Schneider J. A.; Wei Z.; Lai W.-Y.; Huang W.; Wudl F.; Zheng Y. Catalyst-Free One-Step Synthesis of Ortho-Tetraaryl Perylene Diimides for Efficient OPV Non-Fullerene Acceptors. J. Mater. Chem. C 2017, 5, 2781–2785. 10.1039/C7TC00263G.

[ref113] Yao Z.; Zhang M.; Li R.; Yang L.; Qiao Y.; Wang P. A Metal-Free N-Annulated Thienocyclopentaperylene Dye: Power Conversion Efficiency of 12% for Dye-Sensitized Solar Cells. Angew. Chem., Int. Ed. 2015, 54, 5994–5998. 10.1002/anie.201501195. 25820975

[ref114] Wang J.; Wu H.; Jin L.; Zhang J.; Yuan Y.; Wang P. A Perylene-Based Polycyclic Aromatic Hydrocarbon Electron Donor for a Highly Efficient Solar Cell Dye. ChemSusChem 2017, 10, 2962–2967. 10.1002/cssc.201700916. 28631405

[ref115] Wang J.; Xie X.; Weng G.; Yuan Y.; Zhang J.; Wang P. A Low-Energy-Gap Thienochrysenocarbazole Dye for Highly Efficient Mesoscopic Titania Solar Cells: Understanding the Excited State and Charge Carrier Dynamics. ChemSusChem 2018, 11, 1460–1466. 10.1002/cssc.201800186. 29570953

[ref116] Xie X.; Sun D.; Wei Y.; Yuan Y.; Zhang J.; Ren Y.; Wang P. Thienochrysenocarbazole Based Organic Dyes for Transparent Solar Cells with over 10% Efficiency. J. Mater. Chem. A 2019, 7, 11338–11346. 10.1039/C9TA03115D.

[ref117] Yao Z.; Liao X.; Gao K.; Lin F.; Xu X.; Shi X.; Zuo L.; Liu F.; Chen Y.; Jen A. K.-Y. Dithienopicenocarbazole-Based Acceptors for Efficient Organic Solar Cells with Optoelectronic Response Over 1000 Nm and an Extremely Low Energy Loss. J. Am. Chem. Soc. 2018, 140, 2054–2057. 10.1021/jacs.7b13239. 29377679

[ref118] Gupta R. K.; Ulla H.; Satyanarayan M. N.; Sudhakar A. A. A Perylene-Triazine-Based Star-Shaped Green Light Emitter for Organic Light Emitting Diodes: A Perylene-Triazine-Based Star-Shaped Green Light Emitter for Organic Light Emitting Diodes. Eur. J. Org. Chem. 2018, 2018, 1608–1613. 10.1002/ejoc.201800161.

[ref119] Hendsbee A. D.; Sun J.-P.; Law W. K.; Yan H.; Hill I. G.; Spasyuk D. M.; Welch G. C. Synthesis, Self-Assembly, and Solar Cell Performance of N-Annulated Perylene Diimide Non-Fullerene Acceptors. Chem. Mater. 2016, 28, 7098–7109. 10.1021/acs.chemmater.6b03292.

[ref120] Vespa M.; Cann J. R.; Dayneko S. V.; Melville O. A.; Hendsbee A. D.; Zou Y.; Lessard B. H.; Welch G. C. Synthesis of a Perylene Diimide Dimer with Pyrrolic N-H Bonds and N-Functionalized Derivatives for Organic Field-Effect Transistors and Organic Solar Cells. Eur. J. Org. Chem. 2018, 2018, 4592–4599. 10.1002/ejoc.201801055.

[ref121] Cann J. R.; Cabanetos C.; Welch G. C. Synthesis of Molecular Dyads and Triads Based Upon N-Annulated Perylene Diimide Monomers and Dimers. Eur. J. Org. Chem. 2018, 2018, 6933–6943. 10.1002/ejoc.201801383.

[ref122] Nazari M.; Cieplechowicz E.; Welsh T. A.; Welch G. C. A Direct Comparison of Monomeric vs. Dimeric and Non-Annulated vs. N-Annulated Perylene Diimide Electron Acceptors for Organic Photovoltaics. New J. Chem. 2019, 43, 5187–5195. 10.1039/C8NJ06491A.

[ref123] Cann J.; Farahat M. E.; Welch G. C. Hybrid Tetrameric Perylene Diimide Assemblies. ChemSusChem 2021, 14, 3511 10.1002/cssc.202002784. 33496067

[ref124] You F.; Zhou X.; Huang H.; Liu Y.; Liu S.; Shao J.; Zhao B.; Qin T.; Huang W. N-Annulated Perylene Diimide Derivatives as Non-Fullerene Acceptors for Solution-Processed Solar Cells with an Open-Circuit Voltage of up to 1.14 V. New J. Chem. 2018, 42, 15079–15087. 10.1039/C8NJ02566E.

[ref125] Sun D.; Meng D.; Cai Y.; Fan B.; Li Y.; Jiang W.; Huo L.; Sun Y.; Wang Z. Non-Fullerene-Acceptor-Based Bulk-Heterojunction Organic Solar Cells with Efficiency over 7%. J. Am. Chem. Soc. 2015, 137, 11156–11162. 10.1021/jacs.5b06414. 26278192

[ref126] Meng D.; Sun D.; Zhong C.; Liu T.; Fan B.; Huo L.; Li Y.; Jiang W.; Choi H.; Kim T.; et al. High-Performance Solution-Processed Non-Fullerene Organic Solar Cells Based on Selenophene-Containing Perylene Bisimide Acceptor. J. Am. Chem. Soc. 2016, 138, 375–380. 10.1021/jacs.5b11149. 26652276

[ref127] Cann J.; Dayneko S.; Sun J.-P.; Hendsbee A. D.; Hill I. G.; Welch G. C. N-Annulated Perylene Diimide Dimers: Acetylene Linkers as a Strategy for Controlling Structural Conformation and the Impact on Physical, Electronic, Optical and Photovoltaic Properties. J. Mater. Chem. C 2017, 5, 2074–2083. 10.1039/C6TC05107C.

[ref128] Cann J. R.; Cabanetos C.; Welch G. C. Spectroscopic Engineering toward Near-Infrared Absorption of Materials Containing Perylene Diimide. ChemPlusChem 2017, 82, 1359–1364. 10.1002/cplu.201700502. 31957186

[ref129] Payne A.-J.; Li S.; Dayneko S. V.; Risko C.; Welch G. C. An Unsymmetrical Non-Fullerene Acceptor: Synthesis via Direct Heteroarylation, Self-Assembly, and Utility as a Low Energy Absorber in Organic Photovoltaic Cells. Chem. Commun. 2017, 53, 10168–10171. 10.1039/C7CC05836E. 28852753

[ref130] McAfee S. M.; Dayneko S. V.; Hendsbee A. D.; Josse P.; Blanchard P.; Cabanetos C.; Welch G. C. Applying Direct Heteroarylation Synthesis to Evaluate Organic Dyes as the Core Component in PDI-Based Molecular Materials for Fullerene-Free Organic Solar Cells. J. Mater. Chem. A 2017, 5, 11623–11633. 10.1039/C7TA00318H.

[ref131] McAfee S. M.; Dayneko S. V.; Josse P.; Blanchard P.; Cabanetos C.; Welch G. C. Simply Complex: The Efficient Synthesis of an Intricate Molecular Acceptor for High-Performance Air-Processed and Air-Tested Fullerene-Free Organic Solar Cells. Chem. Mater. 2017, 29, 1309–1314. 10.1021/acs.chemmater.6b04862.

[ref132] Hendsbee A. D.; Dayneko S. V.; Pells J. A.; Cann J. R.; Welch G. C. N-Annulated Perylene Diimide Dimers: The Effect of Thiophene Bridges on Physical, Electronic, Optical, and Photovoltaic Properties. Sustain. Energy Fuels 2017, 1, 1137–1147. 10.1039/C7SE00056A.

[ref133] Payne A.-J.; Rice N. A.; McAfee S. M.; Li S.; Josse P.; Cabanetos C.; Risko C.; Lessard B. H.; Welch G. C. Donor or Acceptor? How Selection of the Rylene Imide End Cap Impacts the Polarity of π-Conjugated Molecules for Organic Electronics. ACS Appl. Energy Mater. 2018, 1, 4906–4916. 10.1021/acsaem.8b00929.

[ref134] Welsh T. A.; Laventure A.; Baumgartner T.; Welch G. C. Dithienophosphole-Based Molecular Electron Acceptors Constructed Using Direct (Hetero)Arylation Cross-Coupling Methods. J. Mater. Chem. C 2018, 6, 2148–2154. 10.1039/C7TC05631A.

[ref135] Breukelaar W. B.; McAfee S. M.; Welch G. C. Exploiting Direct Heteroarylation Polymerization Homocoupling Defects for the Synthesis of a Molecular Dimer. New J. Chem. 2018, 42, 1617–1621. 10.1039/C7NJ04285J.

[ref136] Welsh T. A.; Laventure A.; Alahmadi A. F.; Zhang G.; Baumgartner T.; Zou Y.; Jäkle F.; Welch G. C. Borane Incorporation in a Non-Fullerene Acceptor To Tune Steric and Electronic Properties and Improve Organic Solar Cell Performance. ACS Appl. Energy Mater. 2019, 2, 1229–1240. 10.1021/acsaem.8b01793.

[ref137] Laventure A.; Stanzel S.; Payne A.-J.; Lessard B. H.; Welch G. C. A Ring Fused N-Annulated PDI Non-Fullerene Acceptor for High Open Circuit Voltage Solar Cells Processed from Non-Halogenated Solvents. Synth. Met. 2019, 250, 55–62. 10.1016/j.synthmet.2019.02.010.

[ref138] Welsh T. A.; Payne A.-J.; Welch G. C. Synthesis of Aromatic Imide Tetramers Relevant to Organic Electronics by Direct (Hetero)Arylation. New J. Chem. 2019, 43, 9333–9337. 10.1039/C9NJ01553A.

[ref139] Payne A.-J.; Song J.; Sun Y.; Welch G. C. A Tetrameric Perylene Diimide Non-Fullerene Acceptor via Unprecedented Direct (Hetero)Arylation Cross-Coupling Reactions. Chem. Commun. 2018, 54, 11443–11446. 10.1039/C8CC06446F. 30250948

[ref140] Koenig J. D. B.; Laventure A.; Welch G. C. Harnessing Direct (Hetero)Arylation in Pursuit of a Saddle-Shaped Perylene Diimide Tetramer. ACS Appl. Energy Mater. 2019, 2, 8939–8945. 10.1021/acsaem.9b01978.

[ref141] Fan W.; Liang N.; Meng D.; Feng J.; Li Y.; Hou J.; Wang Z. A High Performance Three-Dimensional Thiophene-Annulated Perylene Dye as an Acceptor for Organic Solar Cells. Chem. Commun. 2016, 52, 11500–11503. 10.1039/C6CC05810H. 27709212

[ref142] Luo Z.; Liu T.; Cheng W.; Wu K.; Xie D.; Huo L.; Sun Y.; Yang C. A Three-Dimensional Thiophene-Annulated Perylene Bisimide as a Fullerene-Free Acceptor for a High Performance Polymer Solar Cell with the Highest PCE of 8.28% and a VOC over 1.0 V. J. Mater. Chem. C 2018, 6, 1136–1142. 10.1039/C7TC05261H.

[ref143] Li G.; Wang S.; Liu T.; Hao P.; Liu Z.; Li F.; Yang L.-M.; Zhang Y.; Li D.; Yang S.; et al. Non-Fullerene Acceptor Engineering with Three-Dimensional Thiophene/Selenophene-Annulated Perylene Diimides for High Performance Polymer Solar Cells. J. Mater. Chem. C 2018, 6, 12601–12607. 10.1039/C8TC04926B.

[ref144] Qu J.; Mu Z.; Lai H.; Xie M.; Liu L.; Lu W.; Chen W.; He F. Effect of the Molecular Configuration of Perylene Diimide Acceptors on Charge Transfer and Device Performance. ACS Appl. Energy Mater. 2018, 1, 833–840. 10.1021/acsaem.7b00277.

[ref145] Liang Y.; Deng P.; Wang Z.; Guo Z.; Lei Y. Novel Perylene Diimide Acceptor for Nonfullerene Organic Solar Cells. Funct. Mater. Lett. 2019, 12, 1950022 10.1142/S179360471950022X.

[ref146] Wu F.; Luo Z.; Zhu L.; Chen C.; Lu H.; Chen Z.; Tang J.; Yang C. Sulfur-Annulated Perylenediimide as an Interfacial Material Enabling Inverted Perovskite Solar Cells with over 20% Efficiency and High Fill Factors Exceeding 83%. J. Mater. Chem. A 2019, 7, 21176–21181. 10.1039/C9TA07349C.

[ref147] Li M.; Wang H.; Liu Y.; Zhou Y.; Lu H.; Song J.; Bo Z. Perylene Diimide Acceptor with Two Planar Arms and a Twisted Core for High Efficiency Polymer Solar Cells. Dyes Pigm. 2020, 175, 108186 10.1016/j.dyepig.2020.108186.

[ref148] Meng D.; Fu H.; Xiao C.; Meng X.; Winands T.; Ma W.; Wei W.; Fan B.; Huo L.; Doltsinis N. L.; et al. Three-Bladed Rylene Propellers with Three-Dimensional Network Assembly for Organic Electronics. J. Am. Chem. Soc. 2016, 138, 10184–10190. 10.1021/jacs.6b04368. 27440216

[ref149] Hu M.; Zhao X.; Zhu G.; Zhang Y.; Yuan Z.; Wang L.; Huang X.; Hu Y.; Chen Y. Seleno Twisted Benzodiperylenediimides: Facile Synthesis and Excellent Electron Acceptors for Additive-Free Organic Solar Cells. Chem. Commun. 2019, 55, 703–706. 10.1039/C8CC09367A. 30566143

[ref150] Ma Z.; Fu H.; Meng D.; Jiang W.; Sun Y.; Wang Z. Isomeric N-Annulated Perylene Diimide Dimers for Organic Solar Cells. Chem. - Asian J. 2018, 13, 918–923. 10.1002/asia.201800058. 29443455

[ref151] Guo Y.; Ma Z.; Niu X.; Zhang W.; Tao M.; Guo Q.; Wang Z.; Xia A. Bridge-Mediated Charge Separation in Isomeric N-Annulated Perylene Diimide Dimers. J. Am. Chem. Soc. 2019, 141, 12789–12796. 10.1021/jacs.9b05723. 31334641

[ref152] Buendıa J.; Greciano E. E.; Sánchez L. Influence of Axial and Point Chirality in the Chiral Self-Assembly of Twin *N* -Annulated Perylenecarboxamides. J. Org. Chem. 2015, 80, 12444–12452. 10.1021/acs.joc.5b02309. 26584232

[ref153] Greciano E. E.; Sánchez L. Seeded Supramolecular Polymerization in a Three-Domain Self-Assembly of an N-Annulated Perylenetetracarboxamide. Chem. - Eur. J. 2016, 22, 13724–13730. 10.1002/chem.201602344. 27534518

[ref154] Buendia J.; Calbo J.; Orti E.; Sanchez L. Flexible Chirality in Self-Assembled N-Annulated Perylenedicarboxamides. Small 2017, 13, 1603880 10.1002/smll.201603880. 28388003

[ref155] Greciano E. E.; Calbo J.; Ortí E.; Sánchez L. N-Annulated Perylene Bisimides to Bias the Differentiation of Metastable Supramolecular Assemblies into J- and H-Aggregates. Angew. Chem., Int. Ed. 2020, 59, 17517–17524. 10.1002/anie.202005837. 32537822

[ref156] Qin R.; Guo D.; Ma H.; Yang J.; Jiang Y.; Liu H.; Liu Z.; Song J.; Qin C. Effect of Molecular Structures of Donor Monomers of Polymers on Photovoltaic Properties. ACS Omega 2019, 4, 19177–19182. 10.1021/acsomega.9b02476. 31763541 PMC6868602

[ref157] Liu X.; Kim Y. J.; Ha Y. H.; Zhao Q.; Park C. E.; Kim Y.-H. Effects of Alkyl Chain Length on the Optoelectronic Properties and Performance of Pyrrolo-Perylene Solar Cells. ACS Appl. Mater. Interfaces 2015, 7, 8859–8867. 10.1021/acsami.5b01444. 25836743

[ref158] Sung M. J.; Yoon S.; Kwon S.-K.; Kim Y.-H.; Chung D. S. Synthesis of Phenanthro[1,10,9,8- *Cdefg* ]Carbazole-Based Conjugated Polymers for Green-Selective Organic Photodiodes. ACS Appl. Mater. Interfaces 2016, 8, 31172–31178. 10.1021/acsami.6b12410. 27934249

[ref159] Đorđević L.; Milano D.; Demitri N.; Bonifazi D. O-Annulation to Polycyclic Aromatic Hydrocarbons: A Tale of Optoelectronic Properties from Five- to Seven-Membered Rings. Org. Lett. 2020, 22, 4283–4288. 10.1021/acs.orglett.0c01331. 32429668

[ref160] Fujimoto K.; Izawa S.; Takahashi A.; Inuzuka T.; Sanada K.; Sakamoto M.; Nakayama Y.; Hiramoto M.; Takahashi M. Curved Perylene Diimides Fused with Seven-Membered Rings. Chem. - Asian J. 2021, 16, 690–695. 10.1002/asia.202100066. 33491273

[ref161] Zink-Lorre N.; Doncel-Giménez A.; Font-Sanchis E.; Calbo J.; Sastre-Santos Á.; Ortí E.; Fernández-Lázaro F. Diels-Alder Reaction on Perylenediimides: Synthesis and Theoretical Study of Core-Expanded Diimides. Org. Chem. Front. 2019, 6, 2860–2871. 10.1039/C9QO00682F.

[ref162] Goujon A.; Rocard L.; Cauchy T.; Hudhomme P. An Imine Photocyclization as an Alternative to the Pictet-Spengler Reaction for the Synthesis of AzaBenzannulated Perylenediimide Dyes. J. Org. Chem. 2020, 85, 7218–7224. 10.1021/acs.joc.0c00600. 32352293

[ref163] Hao L.; Jiang W.; Wang Z. Integration of Nitrogen into Coronene Bisimides. Tetrahedron 2012, 68, 9234–9239. 10.1016/j.tet.2012.08.084.

[ref164] Schulze M.; Steffen A.; Würthner F. Near-IR Phosphorescent Ruthenium(II) and Iridium(III) Perylene Bisimide Metal Complexes. Angew. Chem., Int. Ed. 2015, 54, 1570–1573. 10.1002/anie.201410437. 25504675

[ref165] Shi J.; Fan J.; Qu Z.; Wang S.; Wang Y. Solution Concentration-Dependent Tunable Emission in Cyclometalated Iridium Complex Bearing Perylene Diimide (PDI) Ligand: From Visible to near-Infrared Emission. Dyes Pigm. 2018, 154, 263–268. 10.1016/j.dyepig.2018.03.011.

[ref166] Wang C.-S.; Sun Q.; García F.; Wang C.; Yoshikai N. Robust Cobalt Catalyst for Nitrile/Alkyne [2 + 2+2] Cycloaddition: Synthesis of Polyarylpyridines and Their Mechanochemical Cyclodehydrogenation to Nitrogen-Containing Polyaromatics**. Angew. Chem., Int. Ed. 2021, 60, 9627–9634. 10.1002/anie.202017220. 33559370

[ref167] Wei H.; Qiu T.; Huang X.; Zhou J.; Guo J.; Jiang C.; Luo S.; Zeng Z.; Wu J. A Facile Approach toward 1,2-Diazabenzo[Ghi]Perylene Derivatives: Structures and Electronic Properties. Chem. Commun. 2017, 53, 6740–6743. 10.1039/C7CC03362A. 28589189

[ref168] Hahn U.; Maisonhaute E.; Nierengarten J.-F. Twisted N-Doped Nano-Graphenes: Synthesis, Characterization, and Resolution. Angew. Chem., Int. Ed. 2018, 57, 10635–10639. 10.1002/anie.201805852. 29905402

[ref169] Chen Z.; Li J.; Li M.; Chen C.; Xu S.; Tang X.; Chen L.; Chen R.; Huang W. Synthesis and Application of Perylene-Embedded Benzoazoles for Small-Molecule Organic Solar Cells. Org. Lett. 2018, 20, 6376–6379. 10.1021/acs.orglett.8b02650. 30295498

[ref170] Benniston A. C.; He X.; Lemmetyinen H.; Tkachenko N. V. Charge Transfer Properties of a Donor-Acceptor Dyad Based on an Expanded Acridinium Cation. RSC Adv. 2013, 3, 4995 10.1039/c3ra22813d.

[ref171] He X.; Benniston A. C.; Lemmetyinen H.; Tkachenko N. V. Charge Shift/Recombination and Triplet Formation in a Molecular Dyad Based on a Borondipyrromethene (Bodipy) and an Expanded Acridinium Cation. ChemPhotoChem. 2018, 2, 277–282. 10.1002/cptc.201700184.

[ref172] Zhong H.; Wu C.-H.; Li C.-Z.; Carpenter J.; Chueh C.-C.; Chen J.-Y.; Ade H.; Jen A. K.-Y. Rigidifying Nonplanar Perylene Diimides by Ring Fusion Toward Geometry-Tunable Acceptors for High-Performance Fullerene-Free Solar Cells. Adv. Mater. 2016, 28, 951–958. 10.1002/adma.201504120. 26638861

[ref173] Yang L.; Gu W.; Lv L.; Chen Y.; Yang Y.; Ye P.; Wu J.; Hong L.; Peng A.; Huang H. Triplet Tellurophene-Based Acceptors for Organic Solar Cells. Angew. Chem., Int. Ed. 2018, 57, 1096–1102. 10.1002/anie.201712011. 29215780

[ref174] Wang L.; Hu M.; Zhang Y.; Yuan Z.; Hu Y.; Zhao X.; Chen Y. Single-Strand and Ladder-Type Polymeric Acceptors Based on Regioisomerically-Pure Perylene Diimides towards All-Polymer Solar Cells. Polymer 2019, 162, 108–115. 10.1016/j.polymer.2018.12.041.

[ref175] Hartnett P. E.; Matte H. S. S. R.; Eastham N. D.; Jackson N. E.; Wu Y.; Chen L. X.; Ratner M. A.; Chang R. P. H.; Hersam M. C.; Wasielewski M. R.; et al. Ring-Fusion as a Perylenediimide Dimer Design Concept for High-Performance Non-Fullerene Organic Photovoltaic Acceptors. Chem. Sci. 2016, 7, 3543–3555. 10.1039/C5SC04956C. 29997846 PMC6007210

[ref176] Liu X.-P.; Xue L.-W.; Wei Q.; Liang M.; Deng K.; Zhang Z.-J.; Jiang P. Seeing Modulability Self-Assembled Monolayers of π-Conjugated Perylene Derivatives by Scanning Tunneling Microscopy. J. Phys. Chem. C 2016, 120, 18607–18615. 10.1021/acs.jpcc.6b04975.

[ref177] Cai Z.; Vázquez R. J.; Zhao D.; Li L.; Lo W.; Zhang N.; Wu Q.; Keller B.; Eshun A.; Abeyasinghe N.; et al. Two Photon Absorption Study of Low-Bandgap, Fully Conjugated Perylene Diimide-Thienoacene-Perylene Diimide Ladder-Type Molecules. Chem. Mater. 2017, 29, 6726–6732. 10.1021/acs.chemmater.7b01512.

[ref178] Cai Z.; Zhao D.; Sharapov V.; Awais M. A.; Zhang N.; Chen W.; Yu L. Enhancement in Open-Circuit Voltage in Organic Solar Cells by Using Ladder-Type Nonfullerene Acceptors. ACS Appl. Mater. Interfaces 2018, 10, 13528–13533. 10.1021/acsami.8b01308. 29589434

[ref179] Carlotti B.; Cai Z.; Kim H.; Sharapov V.; Madu I. K.; Zhao D.; Chen W.; Zimmerman P. M.; Yu L.; Goodson T. Charge Transfer and Aggregation Effects on the Performance of Planar vs Twisted Nonfullerene Acceptor Isomers for Organic Solar Cells. Chem. Mater. 2018, 30, 4263–4276. 10.1021/acs.chemmater.8b01047.

[ref180] Li X.; Wu K.; Zheng L.; Deng Y.; Tan S.; Chen H. Synthesis and Characterization of Novel Benzodithiophene-Fused Perylene Diimide Acceptors: Regulate Photovoltaic Performance via Structural Isomerism. Dyes Pigm. 2019, 168, 59–67. 10.1016/j.dyepig.2019.04.027.

[ref181] Tian Z.; Guo Y.; Li J.; Li C.; Li W. Benzodithiophene-Fused Perylene Bisimides as Electron Acceptors for Non-Fullerene Organic Solar Cells with High Open-Circuit Voltage. ChemPhysChem 2019, 20, 2696–2701. 10.1002/cphc.201900309. 31012986

[ref182] Yang J.; Chen F.; Ran H.; Hu J.-Y.; Xiao B.; Tang A.; Wang X.; Zhou E. Design and Synthesis of a Novel N-Type Polymer Based on Asymmetric Rylene Diimide for the Application in All-Polymer Solar Cells. Macromol. Rapid Commun. 2018, 39, 1700715 10.1002/marc.201700715. 29292584

[ref183] Li S.; Liu W.; Li C.-Z.; Lau T.-K.; Lu X.; Shi M.; Chen H. A Non-Fullerene Acceptor with a Fully Fused Backbone for Efficient Polymer Solar Cells with a High Open-Circuit Voltage. J. Mater. Chem. A 2016, 4, 14983–14987. 10.1039/C6TA07368A.

[ref184] Ziffer M. E.; Jo S. B.; Zhong H.; Ye L.; Liu H.; Lin F.; Zhang J.; Li X.; Ade H. W.; Jen A. K.-Y.; et al. Long-Lived, Non-Geminate, Radiative Recombination of Photogenerated Charges in a Polymer/Small-Molecule Acceptor Photovoltaic Blend. J. Am. Chem. Soc. 2018, 140, 9996–10008. 10.1021/jacs.8b05834. 30008210

[ref185] Zhang J.; Li Y.; Huang J.; Hu H.; Zhang G.; Ma T.; Chow P. C. Y.; Ade H.; Pan D.; Yan H. Ring-Fusion of Perylene Diimide Acceptor Enabling Efficient Nonfullerene Organic Solar Cells with a Small Voltage Loss. J. Am. Chem. Soc. 2017, 139, 16092–16095. 10.1021/jacs.7b09998. 29112393

[ref186] Wu Q.; Zhao D.; Yang J.; Sharapov V.; Cai Z.; Li L.; Zhang N.; Neshchadin A.; Chen W.; Yu L. Propeller-Shaped Acceptors for High-Performance Non-Fullerene Solar Cells: Importance of the Rigidity of Molecular Geometry. Chem. Mater. 2017, 29, 1127–1133. 10.1021/acs.chemmater.6b04287.

[ref187] Sun H.; Song X.; Xie J.; Sun P.; Gu P.; Liu C.; Chen F.; Zhang Q.; Chen Z.-K.; Huang W. PDI Derivative through Fine-Tuning the Molecular Structure for Fullerene-Free Organic Solar Cells. ACS Appl. Mater. Interfaces 2017, 9, 29924–29931. 10.1021/acsami.7b08282. 28795560

[ref188] Liu J.; Ma L.-K.; Sheong F. K.; Zhang L.; Hu H.; Zhang J.-X.; Zhang J.; Li Z.; Ma C.; Han X.; et al. Carboxylate Substitution Position Influencing Polymer Properties and Enabling Non-Fullerene Organic Solar Cells with High Open Circuit Voltage and Low Voltage Loss. J. Mater. Chem. A 2018, 6, 16874–16881. 10.1039/C8TA04935A.

[ref189] Shi Q.; Andreansky E. S.; Marder S. R.; Blakey S. B. Synthesis and C-H Functionalization Chemistry of Thiazole-Semicoronenediimides (TsCDIs) and -Coronenediimides (TCDIs). J. Org. Chem. 2017, 82, 10139–10148. 10.1021/acs.joc.7b01604. 28885844

[ref190] Shi Q.; Zhang S.; Zhang J.; Oswald V. F.; Amassian A.; Marder S. R.; Blakey S. B. KOtBu-Initiated Aryl C-H Iodination: A Powerful Tool for the Synthesis of High Electron Affinity Compounds. J. Am. Chem. Soc. 2016, 138, 3946–3949. 10.1021/jacs.5b12259. 26996823

[ref191] Yu Y.; Xue N.; Xiao C.; Ravva M. K.; Guo Y.; Wu L.; Zhang L.; Li Z.; Yue W.; Wang Z. Effect of Conjugation Length on the Properties of Fused Perylene Diimides with Variable Isoindigos. J. Mater. Chem. C 2019, 7, 12263–12269. 10.1039/C9TC04078A.

[ref192] Witalewska M.; Wrona-Piotrowicz A.; Makal A.; Zakrzewski J. Polycyclic Aromatic *N* -Ethoxycarbonyl Thioamide *S* -Oxides and Their Triflic Acid Promoted Cyclization to Fluorescent Thiophene Imine-Fused Arenes. J. Org. Chem. 2018, 83, 1933–1939. 10.1021/acs.joc.7b02867. 29359552

[ref193] Aksakal N. E.; Bayar M.; Dumrul H.; Atilla D.; Chumakov Y.; Yuksel F. Structural and Optical Properties of New Naphthalene and Perylene Imide Imidazoles. Polycyclic Aromat. Compd. 2019, 39, 363–373. 10.1080/10406638.2017.1327871.

[ref194] Volland M.; Zhou P.; Wibmer L.; Häner R.; Decurtins S.; Liu S.-X.; Guldi D. M. Nanographene Favors Electronic Interactions with an Electron Acceptor Rather than an Electron Donor in a Planar Fused Push-Pull Conjugate. Nanoscale 2019, 11, 1437–1441. 10.1039/C8NR06961A. 30608494

[ref195] Inan D.; Dubey R. K.; Westerveld N.; Bleeker J.; Jager W. F.; Grozema F. C. Substitution Effects on the Photoinduced Charge-Transfer Properties of Novel Perylene-3,4,9,10-Tetracarboxylic Acid Derivatives. J. Phys. Chem. A 2017, 121, 4633–4644. 10.1021/acs.jpca.7b03806. 28558214 PMC5483891

[ref196] Xie J.; Chen W.; Wang Z.; Jie K. C. W.; Liu M.; Zhang Q. Synthesis and Exploration of Ladder-Structured Large Aromatic Dianhydrides as Organic Cathodes for Rechargeable Lithium-Ion Batteries. Chem. - Asian J. 2017, 12, 868–876. 10.1002/asia.201700070. 28221009

[ref197] Schönamsgruber J.; Hirsch A. Benz-Bisimidazole-Bridged Perylenes - Linearly Expanded Chromophores: Benz-Bisimidazole-Bridged Perylenes. Eur. J. Org. Chem. 2015, 2015, 2167–2174. 10.1002/ejoc.201403561.

[ref198] Chen L.; Zhang K.; Tang C.; Zheng Q.; Xiao Y. Controllable and Stepwise Synthesis of Soluble Ladder-Conjugated Bis(Perylene Imide) Fluorenebisimidazole as a Multifunctional Optoelectronic Material. J. Org. Chem. 2015, 80, 1871–1877. 10.1021/jo5028529. 25574830

[ref199] Wang Q.; Qi J.; Qiao W.; Wang Z. Y. Soluble Ladder Conjugated Polypyrrones: Synthesis, Characterization and Application in Photodetectors. Dyes Pigm. 2015, 113, 160–164. 10.1016/j.dyepig.2014.08.010.

[ref200] Miletić T.; Fermi A.; Orfanos I.; Avramopoulos A.; De Leo F.; Demitri N.; Bergamini G.; Ceroni P.; Papadopoulos M. G.; Couris S.; et al. Tailoring Colors by O Annulation of Polycyclic Aromatic Hydrocarbons. Chem. - Eur. J. 2017, 23, 2363–2378. 10.1002/chem.201604866. 27897357 PMC5324668

[ref201] de Echegaray P.; Mancheño M. J.; Arrechea-Marcos I.; Juárez R.; López-Espejo G.; López Navarrete J. T.; Ramos M. M.; Seoane C.; Ortiz R. P.; Segura J. L. Synthesis of Perylene Imide Diones as Platforms for the Development of Pyrazine Based Organic Semiconductors. J. Org. Chem. 2016, 81, 11256–11267. 10.1021/acs.joc.6b02214. 27791365

[ref202] Yuan K.; Kahan R. J.; Si C.; Williams A.; Kirschner S.; Uzelac M.; Zysman-Colman E.; Ingleson M. J. The Synthesis of Brominated-Boron-Doped PAHs by Alkyne 1,1-Bromoboration: Mechanistic and Functionalisation Studies. Chem. Sci. 2020, 11, 3258–3267. 10.1039/C9SC05404A. 34122833 PMC8157679

[ref203] Zhang G.; Rominger F.; Zschieschang U.; Klauk H.; Mastalerz M. Facile Synthetic Approach to a Large Variety of Soluble Diarenoperylenes. Chem. - Eur. J. 2016, 22, 14840–14845. 10.1002/chem.201603336. 27428573

[ref204] Suzuki Y.; Yamada K.; Watanabe K.; Kochi T.; Ie Y.; Aso Y.; Kakiuchi F. Synthesis of Dibenzo[ *h*, *Rst* ]Pentaphenes and Dibenzo[ *Fg*, *Qr* ]Pentacenes by the Chemoselective C-O Arylation of Dimethoxyanthraquinones. Org. Lett. 2017, 19, 3791–3794. 10.1021/acs.orglett.7b01666. 28703598

[ref205] Li B.; Peng W.; Luo S.; Jiang C.; Guo J.; Xie S.; Hu Y.; Zhang Y.; Zeng Z. Diagonally π-Extended Perylene-Based Bis(Heteroacene) for Chiroptical Activity and Integrating Luminescence with Carrier-Transporting Capability. Org. Lett. 2019, 21, 1417–1421. 10.1021/acs.orglett.9b00152. 30762373

[ref206] Fujikawa T.; Mitoma N.; Wakamiya A.; Saeki A.; Segawa Y.; Itami K. Synthesis, Properties, and Crystal Structures of π-Extended Double [6]Helicenes: Contorted Multi-Dimensional Stacking Lattice. Org. Biomol. Chem. 2017, 15, 4697–4703. 10.1039/C7OB00987A. 28516991

[ref207] Fujikawa T.; Segawa Y.; Itami K. Laterally π-Extended Dithia[6]Helicenes with Heptagons: Saddle-Helix Hybrid Molecules. J. Org. Chem. 2017, 82, 7745–7749. 10.1021/acs.joc.7b01540. 28686025

[ref208] Wu J.; He D.; Wang Y.; Su F.; Guo Z.; Lin J.; Zhang H.-J. Selective Ortho-π-Extension of Perylene Diimides for Rylene Dyes. Org. Lett. 2018, 20, 6117–6120. 10.1021/acs.orglett.8b02557. 30252493

[ref209] Meng L.; Fujikawa T.; Kuwayama M.; Segawa Y.; Itami K. Thiophene-Fused π-Systems from Diarylacetylenes and Elemental Sulfur. J. Am. Chem. Soc. 2016, 138, 10351–10355. 10.1021/jacs.6b06486. 27501373

[ref210] Zeng C.; Xiao C.; Feng X.; Zhang L.; Jiang W.; Wang Z. Electron-Transporting Bis(Heterotetracenes) with Tunable Helical Packing. Angew. Chem., Int. Ed. 2018, 57, 10933–10937. 10.1002/anie.201805614. 29901845

[ref211] Zeng C.; Meng D.; Jiang W.; Wang Z. Synthesis of Isomeric Perylenodithiophene Diimides. Org. Lett. 2018, 20, 6606–6609. 10.1021/acs.orglett.8b02983. 30296103

[ref212] Su F.; Chen S.; Mo X.; Wu K.; Wu J.; Lin W.; Lin Z.; Lin J.; Zhang H.-J.; Wen T.-B. Trisulfur Radical Anion-Triggered Stitching Thienannulation: Rapid Access to Largely π-Extended Thienoacenes. Chem. Sci. 2020, 11, 1503–1509. 10.1039/C9SC05332H. PMC8148024 34084379

[ref213] Cabral M. G. B.; Pereira de Oliveira Santos D. M.; Bentaleb A.; Hillard E. A.; Cristiano R.; Gallardo H.; Durola F.; Bock H. Columnar Liquid-Crystalline Dibenzopentacenodithiophenes by Photocyclization. Chem. - Eur. J. 2016, 22, 8043–8047. 10.1002/chem.201600773. 27141916

[ref214] Daigle M.; Picard-Lafond A.; Soligo E.; Morin J.-F. Regioselective Synthesis of Nanographenes by Photochemical Cyclodehydrochlorination. Angew. Chem., Int. Ed. 2016, 55, 2042–2047. 10.1002/anie.201509130. 26693659

[ref215] Tokoro Y.; Oishi A.; Fukuzawa S. Twisted Polycyclic Aromatic Systems Prepared by Annulation of Bis(Arylethynyl)Arenes with Biphenylboronic Acids. Chem. - Eur. J. 2016, 22, 13908–13915. 10.1002/chem.201602474. 27515137

[ref216] Regar R.; Mishra R.; Mondal P. K.; Sankar J. Metal-Free Annulation at the *Ortho* - and *Bay* -Positions of Perylene Bisimide Leading to Lateral π-Extension with Strong NIR Absorption. J. Org. Chem. 2018, 83, 9547–9552. 10.1021/acs.joc.8b01303. 30033732

[ref217] Regar R.; Sekhar A. R.; Mishra R.; Sankar J. Bay- and Ortho- Ring Annulated Perylenediimides: Synthesis and Their Panchromatic Absorption. Indian J. Chem. Sect. B Org. Chem. Med. Chem. 2018, 57B, 308–313.

[ref218] Pfeifer D.; Klimant I.; Borisov S. M. Ultrabright Red-Emitting Photostable Perylene Bisimide Dyes: New Indicators for Ratiometric Sensing of High PH or Carbon Dioxide. Chem. - Eur. J. 2018, 24, 10711–10720. 10.1002/chem.201800867. 29738607 PMC6099519

[ref219] Zhang L.; Song I.; Ahn J.; Han M.; Linares M.; Surin M.; Zhang H.-J.; Oh J. H.; Lin J. π-Extended Perylene Diimide Double-Heterohelicenes as Ambipolar Organic Semiconductors for Broadband Circularly Polarized Light Detection. Nat. Commun. 2021, 12, 142 10.1038/s41467-020-20390-y. 33420007 PMC7794514

[ref220] Wu J.; He D.; Zhang L.; Liu Y.; Mo X.; Lin J.; Zhang H. Direct Synthesis of Large-Scale Ortho-Iodinated Perylene Diimides: Key Precursors for Functional Dyes. Org. Lett. 2017, 19, 5438–5441. 10.1021/acs.orglett.7b02718. 28956614

[ref221] Chang H.; Liu H.; Dmitrieva E.; Chen Q.; Ma J.; He P.; Liu P.; Popov A. A.; Cao X.-Y.; Wang X.-Y.; et al. Furan-Containing Double Tetraoxa[7]Helicene and Its Radical Cation. Chem. Commun. 2020, 56, 15181–15184. 10.1039/D0CC06970A. 33216069

[ref222] Hamzehpoor E.; Perepichka D. F. Crystal Engineering of Room Temperature Phosphorescence in Organic Solids. Angew. Chem., Int. Ed. 2020, 59, 9977–9981. 10.1002/anie.201913393. 31725174

[ref223] Haedler A. T.; Meskers S. C. J.; Zha R. H.; Kivala M.; Schmidt H.-W.; Meijer E. W. Pathway Complexity in the Enantioselective Self-Assembly of Functional Carbonyl-Bridged Triarylamine Trisamides. J. Am. Chem. Soc. 2016, 138, 10539–10545. 10.1021/jacs.6b05184. 27462007

[ref224] Valera J. S.; Sánchez-Naya R.; Ramírez F. J.; Zafra J. L.; Gómez R.; Casado J.; Sánchez L. Solvent-Directed Helical Stereomutation Discloses Pathway Complexity on N-Heterotriangulene-Based Organogelators. Chem. - Eur. J. 2017, 23, 11141–11146. 10.1002/chem.201702391. 28590076

[ref225] Valera J. S.; Gómez R.; Sánchez L. Tunable Energy Landscapes to Control Pathway Complexity in Self-Assembled N-Heterotriangulenes: Living and Seeded Supramolecular Polymerization. Small 2018, 14, 1702437 10.1002/smll.201702437. 29141117

[ref226] Dorca Y.; Valera J. S.; Cerdá J.; Aragó J.; Gómez R.; Ortí E.; Sánchez L. Synergy of Axial and Point Chirality to Construct Helical N-Heterotriangulene-Based Supramolecular Polymers. ChemNanoMat 2018, 4, 781–784. 10.1002/cnma.201800186.

[ref794] Dorca Y.; Cerdá J.; Aragó J.; Orti E.; Sánchez L. Flipping Motion To Bias the Organized Supramolecular Polymerization of N-Heterotriangulenes. Chem. Mater. 2019, 31, 7024–7032. 10.1021/acs.chemmater.9b01653.

[ref227] Kato S.; Matsuoka T.; Suzuki S.; Asano M. S.; Yoshihara T.; Tobita S.; Matsumoto T.; Kitamura C. Synthesis, Structures, and Properties of Neutral and Radical Cationic S,C,C-Bridged Triphenylamines. Org. Lett. 2020, 22, 734–738. 10.1021/acs.orglett.9b04575. 31887050

[ref228] Karjule N.; Munavvar F. M.; Nithyanandhan J. Heterotriangulene-Based Unsymmetrical Squaraine Dyes: Synergistic Effects of Donor Moieties and out-of-Plane Branched Alkyl Chains on Dye Cell Performance. J. Mater. Chem. A 2016, 4, 18910–18921. 10.1039/C6TA08651A.

[ref229] Fingerle M.; Bettinger H. F. Embedding a Boroxazine Ring into a Nanographene Scaffold by a Concise Bottom-up Synthetic Strategy. Chem. Commun. 2020, 56, 3847–3850. 10.1039/D0CC00471E. 32091056

[ref230] Kitamoto Y.; Suzuki T.; Miyata Y.; Kita H.; Funaki K.; Oi S. The First Synthesis and X-Ray Crystallographic Analysis of an Oxygen-Bridged Planarized Triphenylborane. Chem. Commun. 2016, 52, 7098–7101. 10.1039/C6CC02440H. 27161278

[ref231] Kitamoto Y.; Oda K.; Ogino K.; Hiyama K.; Kita H.; Hattori T.; Oi S. Synthesis of an Azadioxa-Planar Triphenylborane and Investigation of Its Structural and Photophysical Properties. Chem. Commun. 2021, 57, 2297–2300. 10.1039/D0CC08331C. 33533350

[ref232] Yamamura M.; Nabeshima T. Relationship between the Bowl-Shaped Geometry of Phosphangulene and an Axial Group on the Phosphorus Atom. Bull. Chem. Soc. Jpn. 2016, 89, 42–49. 10.1246/bcsj.20150288.

[ref233] Yamamura M.; Sukegawa K.; Nabeshima T. Tuning the Depth of Bowl-Shaped Phosphine Hosts: Capsule and Pseudo-Cage Architectures in Host-Guest Complexes with C60 Fullerene. Chem. Commun. 2015, 51, 12080–12083. 10.1039/C5CC04194E. 26120943

[ref234] Yamamura M.; Sukegawa K.; Okada D.; Yamamoto Y.; Nabeshima T. Chiroptical Switching Caused by Crystalline/Liquid Crystalline Phase Transition of a Chiral Bowl-Shaped Molecule. Chem. Commun. 2016, 52, 4585–4588. 10.1039/C6CC00995F. 26948812

[ref235] Heskia A.; Maris T.; Wuest J. D. Foiling Normal Patterns of Crystallization by Design. Polymorphism of Phosphangulene Chalcogenides. Cryst. Growth Des. 2019, 19, 5390–5406. 10.1021/acs.cgd.9b00907.

[ref236] Heskia A.; Maris T.; Wuest J. D. Putting Fullerenes in Their Place: Cocrystallizing C60 and C70 with Phosphangulene Chalcogenides. Cryst. Growth Des. 2019, 19, 5418–5428. 10.1021/acs.cgd.9b00954.

[ref237] Heskia A.; Maris T.; Wuest J. D. Bis(Phosphangulene)Iminium Salts. Holding on to Fullerenes with Phangs. Cryst. Growth Des. 2020, 20, 1319–1327. 10.1021/acs.cgd.9b01568.

[ref238] Heskia A.; Maris T.; Aguiar P. M.; Wuest J. D. Building Large Structures with Curved Aromatic Surfaces by Complexing Metals with Phosphangulene. J. Am. Chem. Soc. 2019, 141, 18740–18753. 10.1021/jacs.9b08179. 31657550

[ref239] Yamamura M.; Hasegawa T.; Nabeshima T. Synthesis of Phosphorus-Centered and Chalcogen-Bridged Concave Molecules: Modulation of Bowl Geometries and Packing Structures by Changing Bridging Atoms. Org. Lett. 2016, 18, 816–819. 10.1021/acs.orglett.6b00105. 26836024

[ref240] Nakatsuka S.; Gotoh H.; Kinoshita K.; Yasuda N.; Hatakeyama T. Divergent Synthesis of Heteroatom-Centered 4,8,12-Triazatriangulenes. Angew. Chem., Int. Ed. 2017, 56, 5087–5090. 10.1002/anie.201701246. 28370999

[ref241] Elbert S. M.; Reinschmidt M.; Baumgärtner K.; Rominger F.; Mastalerz M. Benzopyrano-Fused N-Heterocyclic Polyaromatics: Benzopyrano-Fused N-Heterocyclic Polyaromatics. Eur. J. Org. Chem. 2018, 2018, 532–536. 10.1002/ejoc.201701796.

[ref242] Yokoyama S.; Hirose T.; Matsuda K. Photochemical Cleavage of the Axial Group Attached to the Central Carbon Atom of Triazatriangulene. Chem. Lett. 2015, 44, 76–78. 10.1246/cl.140897.

[ref243] Shaikh A. C.; Veleta J. M.; Moutet J.; Gianetti T. L. Trioxatriangulenium (TOTA+) as a Robust Carbon-Based Lewis Acid in Frustrated Lewis Pair Chemistry. Chem. Sci. 2021, 12, 4841–4849. 10.1039/D0SC05893A. 34168760 PMC8179643

[ref244] Shivalingam A.; Izquierdo M. A.; Marois A. L.; Vyšniauskas A.; Suhling K.; Kuimova M. K.; Vilar R. The Interactions between a Small Molecule and G-Quadruplexes Are Visualized by Fluorescence Lifetime Imaging Microscopy. Nat. Commun. 2015, 6, 8178 10.1038/ncomms9178. 26350962 PMC4579598

[ref245] Shivalingam A.; Vyšniauskas A.; Albrecht T.; White A. J. P.; Kuimova M. K.; Vilar R. Trianguleniums as Optical Probes for G-Quadruplexes: A Photophysical, Electrochemical, and Computational Study. Chem. - Eur. J. 2016, 22, 4129–4139. 10.1002/chem.201504099. 26880483 PMC4991273

[ref246] Wallabregue A.; Moreau D.; Sherin P.; Moneva Lorente P.; Jarolímová Z.; Bakker E.; Vauthey E.; Gruenberg J.; Lacour J. Selective Imaging of Late Endosomes with a PH-Sensitive Diazaoxatriangulene Fluorescent Probe. J. Am. Chem. Soc. 2016, 138, 1752–1755. 10.1021/jacs.5b09972. 26799309

[ref247] Delgado I. H.; Pascal S.; Besnard C.; Voci S.; Bouffier L.; Sojic N.; Lacour J. C-Functionalized Cationic Diazaoxatriangulenes: Late-Stage Synthesis and Tuning of Physicochemical Properties. Chem. - Eur. J. 2018, 24, 10186–10195. 10.1002/chem.201801486. PMC6099254 29698563

[ref248] Rosenberg M.; Rostgaard K. R.; Liao Z.; Madsen A. Ø.; Martinez K. L.; Vosch T.; Laursen B. W. Design, Synthesis, and Time-Gated Cell Imaging of Carbon-Bridged Triangulenium Dyes with Long Fluorescence Lifetime and Red Emission. Chem. Sci. 2018, 9, 3122–3130. 10.1039/C8SC00089A. 29780456 PMC5932597

[ref249] Feng B.-B.; Liu J.-Q.; Wang X.-S. Cu(OAc)2-Catalyzed Aerobic Oxidative Dehydrogenation Coupling: Synthesis of Heptacyclic Quinolizino[3,4,5,6-Kla]Perimidines. J. Org. Chem. 2017, 82, 1817–1822. 10.1021/acs.joc.6b02644. 28029790

[ref250] Xie J.; Shi K.; Cai K.; Zhang D.; Wang J.-Y.; Pei J.; Zhao D. A NIR Dye with High-Performance n-Type Semiconducting Properties. Chem. Sci. 2016, 7, 499–504. 10.1039/C5SC03045E. 29861996 PMC5952312

[ref251] Kotwica K.; Bujak P.; Data P.; Krzywiec W.; Wamil D.; Gunka P. A.; Skorka L.; Jaroch T.; Nowakowski R.; Pron A.; et al. Soluble Flavanthrone Derivatives: Synthesis, Characterization, and Application to Organic Light-Emitting Diodes. Chem. - Eur. J. 2016, 22, 7978–7986. 10.1002/chem.201600513. 27106658

[ref252] Kotwica K.; Bujak P.; Skorka L.; Jaroch T.; Nowakowski R. Luminophore from Forgotten Dye: Di(Alkylthiophene) Derivative of Benzo[h]Benz[5,6]Acridino[2,1,9,8-Klmna]Acridine. Synth. Met. 2017, 232, 117–122. 10.1016/j.synthmet.2017.08.002.

[ref253] Zhu G.; Zhang G. Access to a Phthalazine Derivative Through an Angular Cis-Quinacridone. J. Org. Chem. 2021, 86, 1198–1203. 10.1021/acs.joc.0c02344. 33284013

[ref254] Yang Y.; Liu D.; Song M.; Shi D.; Liu B.; Cheng K.; Lu Y.; Liu H.; Yang M.; Wang W.; et al. Facile Synthesis of π-Extended Viologens: Electron-Deficient Polycyclic Aza-Aromatics. Chem. - Eur. J. 2017, 23, 7409–7413. 10.1002/chem.201700018. 28397294

[ref255] Yang S.; Bristow J. C.; Addicoat M. A.; Wallis J. D. One Step Conversion of 1,5-Bis(Dimethylamino)Naphthalene to Salts of “Back to Back” Bis-Acridine Derivatives. New J. Chem. 2020, 44, 9621–9625. 10.1039/D0NJ00757A.

[ref256] Šebera J.; Sebechlebská T.; Nováková Lachmanová Š.; Gasior J.; Moreno Garcia P.; Mészáros G.; Valášek M.; Kolivoška V.; Hromadová M. Investigation of the Charge Transport in Model Single Molecule Junctions Based on Expanded Bipyridinium Molecular Conductors. Electrochim. Acta 2019, 301, 267–273. 10.1016/j.electacta.2019.01.132.

[ref257] Imran M.; Wehrmann C. M.; Chen M. S. Open-Shell Effects on Optoelectronic Properties: Antiambipolar Charge Transport and Anti-Kasha Doublet Emission from a *N* -Substituted Bisphenalenyl. J. Am. Chem. Soc. 2020, 142, 38–43. 10.1021/jacs.9b10677. 31854979

[ref258] Taublaender M. J.; Glöcklhofer F.; Marchetti-Deschmann M.; Unterlass M. M. Green and Rapid Hydrothermal Crystallization and Synthesis of Fully Conjugated Aromatic Compounds. Angew. Chem., Int. Ed. 2018, 57, 12270–12274. 10.1002/anie.201801277. PMC6485404 29897647

[ref259] Skonieczny K.; Jażwiński J.; Gryko D. T. The Synthesis of Imidazo[1,2-f]Phenanthridines, Phenanthro-[9,10-d]Imidazoles, and Phenanthro[9’,10’:4,5]Imidazo[1,2-f]-Phenanthridines via Intramolecular Oxidative Aromatic Coupling. Synthesis 2017, 49, 4651–4662. 10.1055/s-0036-1589053.

[ref260] Yang D.-T.; Nakamura T.; He Z.; Wang X.; Wakamiya A.; Peng T.; Wang S. Doping Polycyclic Arenes with Nitrogen-Boron-Nitrogen (NBN) Units. Org. Lett. 2018, 20, 6741–6745. 10.1021/acs.orglett.8b02850. 30350666

[ref261] Pati P. B.; Jin E.; Kim Y.; Kim Y.; Mun J.; Kim S. J.; Kang S. J.; Choe W.; Lee G.; Shin H.-J.; et al. Unveiling 79-Year-Old Ixene and Its BN-Doped Derivative. Angew. Chem., Int. Ed. 2020, 59, 14891–14895. 10.1002/anie.202004049. 32410277

[ref262] Oda S.; Kumano W.; Hama T.; Kawasumi R.; Yoshiura K.; Hatakeyama T. Carbazole-Based DABNA Analogues as Highly Efficient Thermally Activated Delayed Fluorescence Materials for Narrowband Organic Light-Emitting Diodes. Angew. Chem., Int. Ed. 2021, 60, 2882–2886. 10.1002/anie.202012891. 33180369

[ref263] Zhang Y.; Hanifi D.; Alvarez S.; Antonio F.; Pun A.; Klivansky L. M.; Hexemer A.; Ma B.; Liu Y. Charge Transport Anisotropy in *n* -Type Disk-Shaped Triphenylene-Tris(Aroyleneimidazole)s. Org. Lett. 2011, 13, 6528–6531. 10.1021/ol202814y. 22082278

[ref264] Zhang Y.; Hanifi D. A.; Fernández-Liencres M. P.; Klivansky L. M.; Ma B.; Navarro A.; Liu Y. Understanding Electron Transport in Disk-Shaped Triphenylene-Tris(Naphthaleneimidazole)s through Structural Modification and Theoretical Investigation. ACS Appl. Mater. Interfaces 2017, 9, 20010–20019. 10.1021/acsami.7b03795. 28534391

[ref265] Menke E. H.; Lami V.; Vaynzof Y.; Mastalerz M. π-Extended Rigid Triptycene-Trisaroylenimidazoles as Electron Acceptors. Chem. Commun. 2016, 52, 1048–1051. 10.1039/C5CC07238G. 26595168

[ref266] Menke E. H.; Leibold D.; Lami V.; Hofstetter Y. J.; Mastalerz M.; Vaynzof Y. Triptycene-Trisaroyleneimidazoles as Non-Fullerene Acceptors - Influence of Side-Chains on Solubility, Device Morphology and Performance. Org. Electron. 2017, 47, 211–219. 10.1016/j.orgel.2017.05.004.

[ref267] Vladimir A.; Mikhail F.; Amsharov K. Alumina-Promoted Oxodefluorination. RSC Adv. 2020, 10, 10879–10882. 10.1039/D0RA01369B. PMC9050430 35492952

[ref268] Chen X.; Yan L.; Liu Y.; Yang Y.; You J. Switchable Cascade C-H Annulation to Polycyclic Pyryliums and Pyridiniums: Discovering Mitochondria-Targeting Fluorescent Probes. Chem. Commun. 2020, 56, 15080–15083. 10.1039/D0CC06997C. 33206731

[ref269] Berezin A.; Biot N.; Battisti T.; Bonifazi D. Oxygen-Doped Zig-Zag Molecular Ribbons. Angew. Chem., Int. Ed. 2018, 57, 8942–8946. 10.1002/anie.201803282. 29732664

[ref270] Wetherby A. E.; Benson S. D.; Weinert C. S. Reaction of Bis(Bis(Trimethylsilyl)Amido)Mercury(II) with 3,3′-Disubstituted Binaphthols: Cyclization via an Intramolecular Electrophilic Aromatic Substitution Reaction. Inorg. Chim. Acta 2007, 360, 1977–1986. 10.1016/j.ica.2006.10.005.

[ref271] Sciutto A.; Fermi A.; Folli A.; Battisti T.; Beames J. M.; Murphy D. M.; Bonifazi D. Customizing Photoredox Properties of PXX-Based Dyes through Energy Level Rigid Shifts of Frontier Molecular Orbitals. Chem. - Eur. J. 2018, 24, 4382–4389. 10.1002/chem.201705620. 29232478

[ref272] Sciutto A.; Berezin A.; Lo Cicero M.; Miletić T.; Stopin A.; Bonifazi D. Tailored Synthesis of N-Substituted Peri-Xanthenoxanthene Diimide (PXXDI) and Monoimide (PXXMI) Scaffolds. J. Org. Chem. 2018, 83, 13787–13798. 10.1021/acs.joc.8b02076. 30354131

[ref273] Wehrmann C. M.; Charlton R. T.; Chen M. S. A Concise Synthetic Strategy for Accessing Ambient Stable Bisphenalenyls toward Achieving Electroactive Open-Shell π-Conjugated Materials. J. Am. Chem. Soc. 2019, 141, 3240–3248. 10.1021/jacs.8b13300. 30689950

[ref274] Boonloed A.; Weber G. L.; Ramzy K. M.; Dias V. R.; Remcho V. T. Centrifugal Partition Chromatography: A Preparative Tool for Isolation and Purification of Xylindein from Chlorociboria Aeruginosa. J. Chromatogr. A 2016, 1478, 19–25. 10.1016/j.chroma.2016.11.026. 27919517

[ref275] Giesbers G.; Van Schenck J.; Gutierrez S. V.; Robinson S.; Ostroverkhova O. Fungi-Derived Pigments for Sustainable Organic (Opto)Electronics. MRS Adv. 2018, 3, 3459–3464. 10.1557/adv.2018.446.

[ref276] Giesbers G.; Van Schenck J.; Quinn A.; Van Court R.; Vega Gutierrez S. M.; Robinson S. C.; Ostroverkhova O. Xylindein: Naturally Produced Fungal Compound for Sustainable (Opto)Electronics. ACS Omega 2019, 4, 13309–13318. 10.1021/acsomega.9b01490. 31460459 PMC6704441

[ref277] Moon S.; Nishii Y.; Miura M. Thioether-Directed Peri-Selective C-H Arylation under Rhodium Catalysis: Synthesis of Arene-Fused Thioxanthenes. Org. Lett. 2019, 21, 233–236. 10.1021/acs.orglett.8b03675. 30575404

[ref278] Hertz V. M.; Lerner H.-W.; Wagner M. Ru-Catalyzed Benzannulation Leads to Luminescent Boron-Containing Polycyclic Aromatic Hydrocarbons. Org. Lett. 2015, 17, 5240–5243. 10.1021/acs.orglett.5b02604. 26468670

[ref279] Crossley D. L.; Kahan R. J.; Endres S.; Warner A. J.; Smith R. A.; Cid J.; Dunsford J. J.; Jones J. E.; Vitorica-Yrezabal I.; Ingleson M. J. A Modular Route to Boron Doped PAHs by Combining Borylative Cyclisation and Electrophilic C-H Borylation. Chem. Sci. 2017, 8, 7969–7977. 10.1039/C7SC02793A. 29568443 PMC5853289

[ref280] Nagahora N.; Hirano K.; Yasuda A.; Sasamori T.; Shioji K.; Okuma K. The Electronic Structure of Thioxanthylium Scaffolds. Heterocycles 2021, 102, 451–464. 10.3987/COM-20-14372.

[ref281] John A.; Bolte M.; Lerner H.-W.; Wagner M. A Vicinal Electrophilic Diborylation Reaction Furnishes Doubly Boron-Doped Polycyclic Aromatic Hydrocarbons. Angew. Chem., Int. Ed. 2017, 56, 5588–5592. 10.1002/anie.201701591. 28402016

[ref282] Yu Z.; Zhang Y.; Tang J.; Zhang L.; Liu Q.; Li Q.; Gao G.; You J. Ir-Catalyzed Cascade C-H Fusion of Aldoxime Ethers and Heteroarenes: Scope and Mechanisms. ACS Catal. 2020, 10, 203–209. 10.1021/acscatal.9b04274.

[ref283] Mocanu A.; Szücs R.; Caytan E.; Roisnel T.; Dorcet V.; Bouit P.-A.; Nyulászi L.; Hissler M. Synthesis, Optical, and Redox Properties of Regioisomeric Benzoheterocycles-Fused Pyrene. J. Org. Chem. 2019, 84, 957–962. 10.1021/acs.joc.8b02884. 30584837

[ref284] Yan J.; Pulis A. P.; Perry G. J. P.; Procter D. J. Metal-Free Synthesis of Benzothiophenes by Twofold C-H Functionalization: Direct Access to Materials-Oriented Heteroaromatics. Angew. Chem., Int. Ed. 2019, 58, 15675–15679. 10.1002/anie.201908319. 31479175

[ref285] Wang Z.; Li J.; Zhang S.; Wang Q.; Dai G.; Liu B.; Zhu X.; Li Z.; Kolodziej C.; McCleese C.; et al. Stable 2D Bisthienoacenes: Synthesis, Crystal Packing, and Photophysical Properties. Chem. - Eur. J. 2018, 24, 14442–14447. 10.1002/chem.201802411. 29969163

[ref286] Zhou C.; Gu W.; Zhang G.; Liu L.; Lv A.; Zhang L. Isomeric Pyrenodithiophenediones and Their Derivatives: Synthesis, Reactivity, and Device Performance. J. Org. Chem. 2019, 84, 5936–5942. 10.1021/acs.joc.9b00579. 30931568

[ref287] Hossain M. A.; Yamashita K.; Hirabayashi K.; Shimizu T.; Goto K.; Sugiura K. Thiophene-Fused Dinaphthopentaphenes: Versatile Applications of 1,2-Bis(Pyren-2-Yl)Aromatics in the Synthesis of π-Expanded Molecules. ChemistrySelect 2017, 2, 4343–4348. 10.1002/slct.201700152.

[ref288] Valdés H.; Poyatos M.; Peris E. Postmodification of the Electronic Properties by Addition of π-Stacking Additives in N-Heterocyclic Carbene Complexes with Extended Polyaromatic Systems. Inorg. Chem. 2015, 54, 3654–3659. 10.1021/acs.inorgchem.5b00250. 25781501

[ref289] Valdés H.; Poyatos M.; Peris E. A Nanosized Janus Bis-N-Heterocyclic Carbene Ligand Based on a Quinoxalinophenanthrophenazine Core, and Its Coordination to Iridium. Organometallics 2015, 34, 1725–1729. 10.1021/acs.organomet.5b00208.

[ref290] Wang Z.; Gu P.; Liu G.; Yao H.; Wu Y.; Li Y.; Rakesh G.; Zhu J.; Fu H.; Zhang Q. A Large Pyrene-Fused N-Heteroacene: Fifteen Aromatic Six-Membered Rings Annulated in One Row. Chem. Commun. 2017, 53, 7772–7775. 10.1039/C7CC03898D. 28650017

[ref291] Mastalerz M.; Ueberricke L.; Ciubotaru I.; Ghalami F.; Mildner F.; Rominger F.; Elstner M. Di- and Tetracyano Substituted Pyrene-Fused Pyrazaacenes -Aggregation in the Solid State. Chem. - Eur. J. 2020, 26, 11634–11642. 10.1002/chem.202002382. 32459010 PMC7540477

[ref292] Ma B.-B.; Feng G.-F.; Zhao P.-C.; Chang F.-F.; Huang W. Asymmetric Imidazole/Pyrene/Pyrazine Based D-π-A Compounds Showing Visualized Acidichromism and near-Infrared Electrochromism. Tetrahedron Lett. 2015, 56, 6912–6914. 10.1016/j.tetlet.2015.10.100.

[ref293] Gu P.-Y.; Zhang J.; Long G.; Wang Z.; Zhang Q. Solution-Processable Thiadiazoloquinoxaline-Based Donor-Acceptor Small Molecules for Thin-Film Transistors. J. Mater. Chem. C 2016, 4, 3809–3814. 10.1039/C5TC03222A.

[ref294] Li Y.; Zhang C.; Gu P.; Wang Z.; Li Z.; Li H.; Lu J.; Zhang Q. Nonvolatile Tri-State Resistive Memory Behavior of a Stable Pyrene-Fused N-Heteroacene with Ten Linearly-Annulated Rings. Chem. - Eur. J. 2018, 24, 7845–7851. 10.1002/chem.201801146. 29572988

[ref295] Gu P.-Y.; Wang Z.; Liu G.; Yao H.; Wang Z.; Li Y.; Zhu J.; Li S.; Zhang Q. Synthesis, Full Characterization, and Field Effect Transistor Behavior of a Stable Pyrene-Fused N-Heteroacene with Twelve Linearly Annulated Six-Membered Rings. Chem. Mater. 2017, 29, 4172–4175. 10.1021/acs.chemmater.7b01318.

[ref296] Lee S. H.; Paredes M. S. V.; Rappenecker T. J.; Robins K. A.; Lee D.-C. Optimized Synthesis of Thermally Stable Axially Modified Pyrazine-Acene Nanoribbon with Gelation Properties. New J. Chem. 2020, 44, 3604–3611. 10.1039/C9NJ06303J.

[ref297] Cortizo-Lacalle D.; Pertegás A.; Melle-Franco M.; Bolink H. J.; Mateo-Alonso A. Pyrene-Fused Bisphenazinothiadiazoles with Red to NIR Electroluminescence. Org. Chem. Front. 2017, 4, 876–881. 10.1039/C7QO00227K.

[ref298] Li Y.; Wang Z.; Zhang C.; Gu P.; Chen W.; Li H.; Lu J.; Zhang Q. Thiadizoloquinoxaline-Based N-Heteroacenes as Active Elements for High-Density Data-Storage Device. ACS Appl. Mater. Interfaces 2018, 10, 15971–15979. 10.1021/acsami.8b05178. 29682969

[ref299] Hu B.-L.; Zhang K.; An C.; Schollmeyer D.; Pisula W.; Baumgarten M. Layered Thiadiazoloquinoxaline-Containing Long Pyrene-Fused N-Heteroacenes. Angew. Chem., Int. Ed. 2018, 57, 12375–12379. 10.1002/anie.201803230. 30070417

[ref300] Cortizo-Lacalle D.; Gozalvez C.; Melle-Franco M.; Mateo-Alonso A. A Thiadiazole-Capped Nanoribbon with 18 Linearly Fused Rings. Nanoscale 2018, 10, 11297–11301. 10.1039/C8NR03516D. 29892730

[ref301] Sahoo P. K.; Giri C.; Haldar T. S.; Puttreddy R.; Rissanen K.; Mal P. Mechanochemical Synthesis, Photophysical Properties, and X-Ray Structures of N-Heteroacenes. Eur. J. Org. Chem. 2016, 2016, 1283–1291. 10.1002/ejoc.201600005.

[ref302] Fratczak E. Z.; Makowski T.; Moustafa R. M.; El-Assaad T. H.; Moneta M. E.; Uznanski P.; Kaafarani B. R. Spectroscopic Characterization of the Structural Properties of Quinoxalinophenanthrophenazine Thin Films. J. Mater. Chem. C 2018, 6, 781–789. 10.1039/C7TC04757F.

[ref303] Maass F.; Stein A.; Kohl B.; Hahn L.; Gade L. H.; Mastalerz M.; Tegeder P. Substrate-Directed Growth of N-Heteropolycyclic Molecules on a Metal Surface. J. Phys. Chem. C 2016, 120, 2866–2873. 10.1021/acs.jpcc.5b12080.

[ref304] Kohl B.; Baumgaertner K.; Rominger F.; Mastalerz M. Quinoxalinophenanthrophenazines (QPPs) and Hexabenzoovalenes (HBOs) - Proving the Solubility Enhancement by Triptycene End-Capping. Eur. J. Org. Chem. 2019, 2019, 4891–4896. 10.1002/ejoc.201900819.

[ref305] Kohl B.; Rominger F.; Mastalerz M. Crystal Structures of a Molecule Designed Not To Pack Tightly. Chem. - Eur. J. 2015, 21, 17308–17313. 10.1002/chem.201502847. 26450149

[ref306] Kohl B.; Bohnwagner M. V.; Rominger F.; Wadepohl H.; Dreuw A.; Mastalerz M. Attractive Dispersion Interactions Versus Steric Repulsion of Tert-Butyl Groups in the Crystal Packing of a D3h-Symmetric Tris(Quinoxalinophenanthrophenazine). Chem. - Eur. J. 2016, 22, 646–655. 10.1002/chem.201503863. 26626549

[ref307] Ueberricke L.; Holub D.; Kranz J.; Rominger F.; Elstner M.; Mastalerz M. Triptycene End-Capped Quinoxalinophenanthrophenazines (QPPs): Influence of Substituents and Conditions on Aggregation in the Solid State. Chem. - Eur. J. 2019, 25, 11121–11134. 10.1002/chem.201902002. 31210369

[ref308] Ueberricke L.; Wieland S.; Rominger F.; Mastalerz M. Triptycene End-Capped Quinoxalinophenanthrophenazines with Aromatic Substituents - Synthesis, Characterization, and Single-Crystal Structure Analysis. Org. Mater. 2019, 01, 050–062. 10.1055/s-0039-1700847.

[ref309] Ueberricke L.; Ghalami F.; Zhang W.-S.; Rao V.; Rominger F.; Schröder R. R.; Elstner M.; Mastalerz M. Isostructural Charge-Transfer Cocrystals Based on Triptycene End-Capped Quinoxalinophenanthrophenazine. Cryst. Growth Des. 2021, 21, 1329–1341. 10.1021/acs.cgd.0c01619.

[ref310] Ueberricke L.; Benke B. P.; Kirschbaum T.; Hahn S.; Rominger F.; Bunz U. H. F.; Mastalerz M. Synthesis and Optoelectronic Properties of a Quinoxalino-Phenanthrophenazine (QPP) Extended Tribenzotriquinacene (TBTQ). Chem. - Eur. J. 2021, 27, 2043–2049. 10.1002/chem.202003666. 32954544 PMC7898691

[ref311] Hu B.-L.; An C.; Wagner M.; Ivanova G.; Ivanova A.; Baumgarten M. Three-Dimensional Pyrene-Fused N-Heteroacenes. J. Am. Chem. Soc. 2019, 141, 5130–5134. 10.1021/jacs.9b01082. 30860825 PMC6727623

[ref312] Li K.; Xu Z.; Deng H.; Zhou Z.; Dang Y.; Sun Z. Dimeric Cycloparaphenylenes with a Rigid Aromatic Linker. Angew. Chem., Int. Ed. 2021, 60, 7649–7653. 10.1002/anie.202016995. 33427356

[ref313] Cortizo-Lacalle D.; Mora-Fuentes J. P.; Strutyński K.; Saeki A.; Melle-Franco M.; Mateo-Alonso A. Monodisperse N-Doped Graphene Nanoribbons Reaching 7.7 Nanometers in Length. Angew. Chem., Int. Ed. 2018, 57, 703–708. 10.1002/anie.201710467. PMC5768023 29193535

[ref314] Song X.; Zhao J.; Zhang W.; Chen L. Novel N-Channel Organic Semiconductor Based on Pyrene-Phenazine Fused Monoimide and Bisimides. Chin. Chem. Lett. 2018, 29, 331–335. 10.1016/j.cclet.2017.09.015.

[ref315] Min Y.; Dou C.; Tian H.; Geng Y.; Liu J.; Wang L. N-Type Azaacenes Containing B-N Units. Angew. Chem., Int. Ed. 2018, 57, 2000–2004. 10.1002/anie.201712986. 29288570

[ref316] Wang C.-Z.; Feng X.; Elsegood M. R. J.; Warwick T. G.; Teat S. J.; Redshaw C.; Bi Y.-S.; Yamato T. Pyrene-Fused Pyrazaacenes with Eight Rectilinearly Arranged Aromatic Rings. Asian J. Org. Chem. 2019, 8, 155–160. 10.1002/ajoc.201800608.

[ref317] Mora-Fuentes J. P.; Papadopoulos I.; Thiel D.; Álvarez-Boto R.; Cortizo-Lacalle D.; Clark T.; Melle-Franco M.; Guldi D. M.; Mateo-Alonso A. Singlet Fission in Pyrene-Fused Azaacene Dimers. Angew. Chem., Int. Ed. 2020, 59, 1113–1117. 10.1002/anie.201911529. PMC7687256 31647593

[ref318] Jiang M.; Li X.; Sun L.; Niu X.; Liang Q.; Cai X.; Huang J.; Ling J.; Mo Y. Special Photophysical Properties of Poly(2,11-Diquinoxalinopyrene)s. Chin. J. Polym. Sci. 2017, 35, 1097–1109. 10.1007/s10118-017-1961-2.

[ref319] Chu T.; Ju X.; Han X.; Du H.; Zhang Y.; Zhao J.; Zhang J. Synthesis and Electrochromic Properties of Cross-Linked and Soluble Conjugated Polymers Based on 5, 8, 14, 17-Tetrabromoquinoxaline[2’, 3′:9,10]Phenanthro[4,5-Abc]Phenazine as the Multifunctionalized Acceptor Unit. Org. Electron. 2019, 73, 43–54. 10.1016/j.orgel.2019.06.004.

[ref320] Chu T.; Yue H.; Zhao Y.; Du H.; Zhang Y.; Han X.; Zhao J.; Zhang J. Synthesis and Characterization of D-A Type Conjugated Electrochromic Polymers with Cross-Linked Structure Employing a Novel and Multi-Functionalized Molecular as the Acceptor Unit. J. Electroanal. Chem. 2019, 848, 113276 10.1016/j.jelechem.2019.113276.

[ref321] Jiang L.; Papageorgiou A. C.; Oh S. C.; Saglam Ö.; Reichert J.; Duncan D. A.; Zhang Y.-Q.; Klappenberger F.; Guo Y.; Allegretti F.; et al. Synthesis of Pyrene-Fused Pyrazaacenes on Metal Surfaces: Toward One-Dimensional Conjugated Nanostructures. ACS Nano 2016, 10, 1033–1041. 10.1021/acsnano.5b06340. 26651905

[ref322] Yu X.; Wan J.; Yuan C.; Guo N.; Shen Y.; Li J. Tetraazatetraoxodecacene and Tetraazatetrathiodecacene: Synthesis, Crystal Structures, Linear and Third-Order Nonlinear Optical Properties. Dyes Pigm. 2019, 161, 130–136. 10.1016/j.dyepig.2018.07.038.

[ref323] Marco A. B.; Gozalvez C.; Olano M.; Sun X.; Atxabal A.; Melle-Franco M.; Hueso L. E.; Mateo-Alonso A. Bis(Triisopropylsilylethynyl)-Substituted Pyrene-Fused Tetraazaheptacene: Synthesis and Properties. Phys. Chem. Chem. Phys. 2016, 18, 11616–11619. 10.1039/C5CP07656K. 26910505

[ref324] Pan H.; Song T.; Yin X.; Jin P.; Xiao J. Synthesis, Crystal Analysis, and Optoelectronic Properties of Diazole-Functionalized Acenes and Azaacenes. Chem. - Eur. J. 2018, 24, 6572–6579. 10.1002/chem.201705657. 29341382

[ref325] Antonicelli G.; Gozalvez C.; Atxabal A.; Melle-Franco M.; Hueso L. E.; Mateo-Alonso A. K-Conjugated Dibenzoazahexacenes. Org. Lett. 2016, 18, 4694–4697. 10.1021/acs.orglett.6b02332. 27602601

[ref326] Yuan Y.; Lo K.-C.; Szeto L.; Chan W.-K. Synthesis of Pyrazinopyrazine-Fused Azaacenes through Direct Condensation Reactions between Quinoxalinediamine and Diketones. J. Org. Chem. 2020, 85, 6372–6379. 10.1021/acs.joc.9b03504. 32312048

[ref327] Wu Z.-H.; Sun W.-J.; Tian H.-H.; Yu Z.-F.; Guo R.-X.; Shao X.; Zhang H.-L. 9,10-Imide-Pyrene-Fused Pyrazaacenes (IPPA) as N-Type Doping Materials for High-Performance Nonvolatile Organic Field Effect Transistor Memory Devices. Adv. Electron. Mater. 2019, 5, 1800598 10.1002/aelm.201800598.

[ref328] Riaño A.; Carini M.; Melle-Franco M.; Mateo-Alonso A. Mechanically Interlocked Nitrogenated Nanographenes. J. Am. Chem. Soc. 2020, 142, 20481–20488. 10.1021/jacs.0c10345. 33213145

[ref329] Baumgärtner K.; Kirschbaum T.; Krutzek F.; Dreuw A.; Rominger F.; Mastalerz M. K-Region-Extended [ *c* ]-Heteroannulated Pyrenes. Chem. - Eur. J. 2017, 23, 17817–17822. 10.1002/chem.201703988. 28977702

[ref330] Chen W.; Long G.; Kanehira K.; Zhang M.; Michinobu T.; Liu M.; Zhang Q. A Direct Method to Access Substituted Pyreno[4,5-c:9,10-c’] Difuran and Its Analogues. Asian J. Org. Chem. 2018, 7, 2213–2217. 10.1002/ajoc.201800122.

[ref331] Wang P.-L.; Ding S.-Y.; Zhang Z.-C.; Wang Z.-P.; Wang W. Constructing Robust Covalent Organic Frameworks via Multicomponent Reactions. J. Am. Chem. Soc. 2019, 141, 18004–18008. 10.1021/jacs.9b10625. 31682437

[ref332] Liu Y.-L.; Yang L.; Guo Y.-Q.; Xu G.-Q.; Qu B.; Fu Y. Synthesis and Configurational Character Study of Novel Structural Isomers Based on Pyrene-Imidazole. Molecules 2019, 24, 2293 10.3390/molecules24122293. PMC6630678 31226827

[ref333] Skonieczny K.; Gryko D. T. Light-Induced Direct Arylation in the Solid Crystalline State as a Strategy Towards π-Expanded Imidazoles. Chem. - Asian J. 2016, 11, 2513–2517. 10.1002/asia.201600752. 27452918

[ref334] Karthik S.; Ajantha J.; Nagaraja C. M.; Easwaramoorthi S.; Gandhi T. Synthesis and Photophysics of Extended π-Conjugated Systems of Substituted 10-Aryl-Pyrenoimidazoles. Org. Biomol. Chem. 2016, 14, 10255–10266. 10.1039/C6OB01760F. 27747363

[ref335] Ibáñez S.; Guerrero A.; Poyatos M.; Peris E. Fluorescent Pyrene-Based Bis-Azole Compounds: Synthesis and Photophysical Analysis. Chem. - Eur. J. 2015, 21, 10566–10575. 10.1002/chem.201501179. 26096615

[ref336] Mardanya S.; Karmakar S.; Bar M.; Baitalik S. Pyrene-Biimidazole Based Ru(II) and Os(II) Complexes as Highly Efficient Probes for the Visible and near-Infrared Detection of Cyanide in Aqueous Media. Dalton Trans. 2015, 44, 21053–21072. 10.1039/C5DT03766B. 26592760

[ref337] Mardanya S.; Karmakar S.; Mondal D.; Baitalik S. Homo- and Heterobimetallic Ruthenium(II) and Osmium(II) Complexes Based on a Pyrene-Biimidazolate Spacer as Efficient DNA-Binding Probes in the Near-Infrared Domain. Inorg. Chem. 2016, 55, 3475–3489. 10.1021/acs.inorgchem.5b02912. 27011117

[ref338] Mardanya S.; Mondal D.; Baitalik S. Bimetallic Ru(II) and Os(II) Complexes Based on a Pyrene-Bisimidazole Spacer: Synthesis, Photophysics, Electrochemistry and Multisignalling DNA Binding Studies in the near Infrared Region. Dalton Trans. 2017, 46, 17010–17024. 10.1039/C7DT03355A. 29184930

[ref339] Wen H.; Gong X.; Jia Z.; Han P.; Lin B.; Ye S.; Sun Y.; Zhang X.; Yang H. Conjugated Polymers Constructed by a Novel Pyrene-Fused Polycyclic Building Block and Their Applications as Organic Electronic Materials. Dyes Pigm. 2016, 130, 16–23. 10.1016/j.dyepig.2016.03.009.

[ref340] Shahrokhi F.; Zhao Y. Self-Condensation of Pyrene-4,5-Dione: An Approach To Generate Functional Organic Fluorophores. Org. Lett. 2019, 21, 9306–9310. 10.1021/acs.orglett.9b03297. 31668081

[ref341] Fan M.; Shen Y.; Zheng Y.; Yu X.; Li M.; Xu L.; Wang J.; Yang L.; Li J. Pyridazine-Containing Diazatwistanthracene and Tetraazatwisttetracene: Synthesis, Crystal Structures and Third Order Non-linear Optical Properties. ChemistrySelect 2019, 4, 2810–2814. 10.1002/slct.201804065.

[ref342] Fusco S.; Maglione C.; Velardo A.; Piccialli V.; Liguori R.; Peluso A.; Rubino A.; Centore R. N-Rich Fused Heterocyclic Systems: Synthesis, Structure, Optical and Electrochemical Characterization. Eur. J. Org. Chem. 2016, 2016, 1772–1780. 10.1002/ejoc.201501283.

[ref343] Zheng Y.; Zhao L.; Zhang Y.; Wang C.; Wang K.; Qi D.; Jiang J. Novel Chiral Binaphthalene-Linked Pyrenes. Synthesis, Structure, and Spectroscopy. Dyes Pigm. 2017, 141, 245–250. 10.1016/j.dyepig.2017.02.031.

[ref344] Lv B.; Xiao J.; Zhou J.; Zhang X.; Duan J.; Su W.; Zhao J. Synthesis, Crystal Analyses, Physical Properties, and Electroluminescent Behavior of Unsymmetrical Heterotwistacenes. ACS Appl. Mater. Interfaces 2016, 8, 18998–19003. 10.1021/acsami.6b07304. 27383556

[ref345] Jin P.; Tian F.; Han Y.; Wang L.; Zhao X.; Xiao J. Dimesitylboryl-Decorated Azaarene: Synthesis, Enhanced Stability and Optoelectronic Property. Chem. - Asian J. 2019, 14, 4395–4399. 10.1002/asia.201901349. 31709746

[ref346] Cheung K. Y.; Watanabe K.; Segawa Y.; Itami K. Synthesis of a Zigzag Carbon Nanobelt. Nat. Chem. 2021, 13, 255–259. 10.1038/s41557-020-00627-5. 33495606

[ref347] Chen H.; Gui S.; Zhang Y.; Liu Z.; Miao Q. Synthesis of a Hydrogenated Zigzag Carbon Nanobelt. CCS Chem. 2021, 3, 613 10.31635/ccschem.020.202000189.

[ref348] Tian F.; Song T.; Wang T.; Xiao J.; Zhao X. 11,16-Di-Tert-Butyl-9,18-Diphenylbenzo[Kl]Benzo[8,9]Triphenyleno [2,3-b]Xanthene: Synthesis, Photophysics, Self-Assembly and Electroluminescent Properties. Asian J. Org. Chem. 2019, 8, 399–403. 10.1002/ajoc.201900048.

[ref349] Liu Z.; Wang W.; Xu W.; Chen H.; Zhang X.; Ren T.; Wang X.; Zhao J.; Xiao J. Synthesis, Characterization and Photocurrent Behavior of Asymmetrical Heterotwistacenes. Dyes Pigm. 2015, 115, 143–148. 10.1016/j.dyepig.2014.12.025.

[ref350] Pan H.; Duan J.; Zhai G.; Jin P.; Zhao X.; Jiang L.; Xiao J. Synthesis, Optoelectronic and Self-Assembly Properties of Diazadioxaacene Derivatives. Chem. - Asian J. 2017, 12, 2121–2126. 10.1002/asia.201700700. 28632320

[ref351] Song T.; Han Y.; Jin P.; Li X.; Song Y.; Xiao J. The Enhanced Two-Photon Absorption Behavior of Twistfuranacenes to Phenylacetylene-Functionalized Twistacenes. J. Mater. Chem. C 2019, 7, 6344–6351. 10.1039/C9TC01568J.

[ref352] Duan J.; Gu P.; Xiao J.; Shen X.; Liu X.; Yi Y.; Zhang Q. Synthesis, Physical Properties and Memory Device Application of a Twelve-Ring Fused Twistheteroacene. Chem. - Asian J. 2017, 12, 638–642. 10.1002/asia.201700048. 28117935

[ref353] Verbitskiy E. V.; Cheprakova E. M.; Makarova N. I.; Dorogan I. V.; Metelitsa A. V.; Minkin V. I.; Slepukhin P. A.; Svalova T. S.; Ivanova A. V.; Kozitsina A. N.; et al. Heteroacenes Bearing the Pyrimidine Scaffold: Synthesis, Photophysical and Electrochemical Properties. Eur. J. Org. Chem. 2016, 2016, 1420–1428. 10.1002/ejoc.201501450.

[ref354] Zhao K.-Q.; Jing M.; An L.-L.; Du J.-Q.; Wang Y.-H.; Hu P.; Wang B.-Q.; Monobe H.; Heinrich B.; Donnio B. Facile Transformation of 1-Aryltriphenylenes into Dibenzo[Fg,Op]Tetracenes by Intramolecular Scholl Cyclodehydrogenation: Synthesis, Self-Assembly, and Charge Carrier Mobility of Large π-Extended Discogens. J. Mater. Chem. C 2017, 5, 669–682. 10.1039/C6TC04530H.

[ref355] Moriguchi T.; Higashi M.; Yakeya D.; Jalli V.; Tsuge A.; Okauchi T.; Nagamatsu S.; Takashima W. Synthesis, Characterization and Air Stable Semiconductor Properties of Thiophene-Condensed Pyrene Derivatives. J. Mol. Struct. 2017, 1127, 413–418. 10.1016/j.molstruc.2016.07.104.

[ref356] Moriguchi T.; Yakeya D.; Tsuge A.; Jalli V. Synthesis of Three New Thiophene Condensed Pyrene Derivatives, Crystal Structure and Evaluation of Their Photophysical Properties. J. Mol. Struct. 2018, 1157, 348–354. 10.1016/j.molstruc.2017.12.081.

[ref357] Uryu M.; Hiraga T.; Koga Y.; Saito Y.; Murakami K.; Itami K. Synthesis of Polybenzoacenes: Annulative Dimerization of Phenylene Triflate by Twofold C-H Activation. Angew. Chem., Int. Ed. 2020, 59, 6551–6554. 10.1002/anie.202001211. 32119161

[ref358] Lv B.; Shen X.; Xiao J.; Duan J.; Wang X.; Yi Y. Synthesis, Single Crystal, and Physical Properties of Asymmetrical Thiophene/Selenophene-Fused Twistacenes. Chem. - Asian J. 2015, 10, 2677–2682. 10.1002/asia.201500733. 26282451

[ref359] Tang S.; Zhang L.; Ruan H.; Zhao Y.; Wang X. A Magnetically Robust Triplet Ground State Sulfur-Hydrocarbon Diradical Dication. J. Am. Chem. Soc. 2020, 142, 7340–7344. 10.1021/jacs.0c02141. 32270677

[ref360] Shi X.; Lee S.; Son M.; Zheng B.; Chang J.; Jing L.; Huang K.-W.; Kim D.; Chi C. Pro-Aromatic Bisphenaleno-Thieno[3,2-b]Thiophene versus Anti-Aromatic Bisindeno-Thieno[3,2-b]Thiophene: Different Ground-State Properties and Applications in Field-Effect Transistors. Chem. Commun. 2015, 51, 13178–13180. 10.1039/C5CC04243G. 26199107

[ref361] Wang Q.; Huang K.; Cai S.; Liu C.; Jiao X.; He S.; Zhao L.; Zeng X. Synthesis of Near-Infrared Fluorescent Rhodamines via an SNArH Reaction and Their Biological Applications. Org. Biomol. Chem. 2018, 16, 7163–7169. 10.1039/C8OB01701H. 30246856

[ref362] El-Shaieb K. M.; Mohamed A. H.; Abdel-Latif F. F. Investigation of the Reactivity of 4-Amino-5-Hydrazineyl-4H-1,2, 4-Triazole-3-Thiol towards Some Selected Carbonyl Compounds: Synthesis of Novel Triazolotriazine-, Triazolotetrazine-, and Triazolopthalazine Derivatives. Z. Naturforsch., B: J. Chem. Sci. 2019, 74, 847–855. 10.1515/znb-2019-0140.

[ref363] Shiina Y.; Karasaki H.; Mori S.; Kobayashi N.; Furuta H.; Shimizu S. A Novel Isoindole-Containing Polyaromatic Hydrocarbon Unexpectedly Formed during the Synthesis of Meso-2,6-Dichlorophenyl-Substituted Tribenzosubporphyrin. J. Porphyrins Phthalocyanines 2016, 20, 1049–1054. 10.1142/S1088424616500541.

[ref364] Peng S.; Liu S.; Zhang S.; Cao S.; Sun J. Synthesis of Polyheteroaromatic Compounds via Rhodium-Catalyzed Multiple C-H Bond Activation and Oxidative Annulation. Org. Lett. 2015, 17, 5032–5035. 10.1021/acs.orglett.5b02510. 26439472

[ref365] Chow C. H. E.; Han Y.; Phan H.; Wu J. Nitrogen-Doped Heptazethrene and Octazethrene Diradicaloids. Chem. Commun. 2019, 55, 9100–9103. 10.1039/C9CC04564C. 31297496

[ref366] Arikawa S.; Shimizu A.; Shintani R. Azoniadibenzo[a, j]Phenalenide: A Polycyclic Zwitterion with Singlet Biradical Character. Angew. Chem., Int. Ed. 2019, 58, 6415–6419. 10.1002/anie.201902006. 30869185

[ref367] Yan C.; Shang R.; Nakamoto M.; Yamamoto Y.; Adachi Y. The Substituent Effect of Bridged Triarylamine Helicenes on Light-Emitting and Charge Transfer Properties. Chem. Lett. 2020, 49, 457–460. 10.1246/cl.200089.

[ref368] Min H.; Park I. S.; Yasuda T. Cis-Quinacridone-Based Delayed Fluorescence Emitters: Seemingly Old but Renewed Functional Luminogens. Angew. Chem., Int. Ed. 2021, 60, 7643–7648. 10.1002/anie.202016914. 33511749

[ref369] Wu H.; Zhang Z.; Ma N.; Liu Q.; Liu T.; Zhang G. Synthesis of Acridines from *o* -Aminoaryl Ketones and Arylboronic Acids by Copper Trifluoroacetate-Mediated Relay Reactions. J. Org. Chem. 2018, 83, 12880–12886. 10.1021/acs.joc.8b01828. 30232875

[ref370] Raju S.; Annamalai P.; Chen P.-L.; Liu Y.-H.; Chuang S.-C. Iptycenes with an Acridinone Motif Developed through [4 + 2] Cycloaddition of Tethered Naphthalene and Iminoquinone via a Radical Reaction. Chem. Commun. 2017, 53, 6247–6250. 10.1039/C7CC03030D. 28540943

[ref371] Prinzisky C.; Meyenburg I.; Jacob A.; Heidelmeier B.; Schröder F.; Heimbrodt W.; Sundermeyer J. Optical and Electrochemical Properties of Anthraquinone Imine Based Dyes for Dye-Sensitized Solar Cells. Eur. J. Org. Chem. 2016, 2016, 756–767. 10.1002/ejoc.201501309.

[ref372] Hertz V. M.; Ando N.; Hirai M.; Bolte M.; Lerner H.-W.; Yamaguchi S.; Wagner M. Steric Shielding vs Structural Constraint in a Boron-Containing Polycyclic Aromatic Hydrocarbon. Organometallics 2017, 36, 2512–2519. 10.1021/acs.organomet.6b00800.

[ref373] Hertz V. M.; Massoth J. G.; Bolte M.; Lerner H.-W.; Wagner M. En Route to Stimuli-Responsive Boron-, Nitrogen-, and Sulfur-Doped Polycyclic Aromatic Hydrocarbons. Chem. - Eur. J. 2016, 22, 13181–13188. 10.1002/chem.201602406. 27514699

[ref374] Zhang J.-J.; Tang M.-C.; Fu Y.; Low K.-H.; Ma J.; Yang L.; Weigand J. J.; Liu J.; Yam V. W.-W.; Feng X. One-Pot Synthesis of Boron-Doped Polycyclic Aromatic Hydrocarbons via 1,4-Boron Migration. Angew. Chem., Int. Ed. 2021, 60, 2833–2838. 10.1002/anie.202011237. PMC7898796 33112494

[ref375] Lukeman M.; Simon H.; Wan P.; Wang Y.-H. Photocyclization and Photoaddition Reactions of Arylphenols via Intermediate Quinone Methides. J. Org. Chem. 2015, 80, 11281–11293. 10.1021/acs.joc.5b01580. 26496171

[ref376] Yang S.; Cheng R.; Zhang M.; Bin Z.; You J. Rh/Ag-Mediated *Peri* -Selective Heteroarylation/Single Electron Transfer Annulation Cascade of 1-(Methylthio)Naphthalenes and Analogues: Road Less Traveled to Benzo[ *de* ]Thioacenes. ACS Catal. 2019, 9, 6188–6193. 10.1021/acscatal.9b01426.

[ref377] Zhang L.; Huang Z.; Dai D.; Xiao Y.; Lei K.; Tan S.; Cheng J.; Xu Y.; Liu J.; Qian X. Thio-Bisnaphthalimides as Heavy-Atom-Free Photosensitizers with Efficient Singlet Oxygen Generation and Large Stokes Shifts: Synthesis and Properties. Org. Lett. 2016, 18, 5664–5667. 10.1021/acs.orglett.6b02902. 27750427

[ref378] Chow C. H. E.; Phan H.; Zhang X.; Wu J. Sulfur-Doped (Dibenzo)Heptazethrene and (Dibenzo)Octazethrene Diradicaloids. J. Org. Chem. 2020, 85, 234–240. 10.1021/acs.joc.9b02796. 31711284

[ref379] Plodek A.; König M.; Bracher F. Synthesis of the Azaoxoaporphine Alkaloid Sampangine and Ascididemin-Type Pyridoacridines through TMPMgCl·LiCl-Mediated Ring Closure. Eur. J. Org. Chem. 2015, 2015, 1302–1308. 10.1002/ejoc.201403502.

[ref380] Melzer B. C.; Plodek A.; Bracher F. Functionalization of 4-Bromobenzo[c][2,7]Naphthyridine via Regioselective Direct Ring Metalation. A Novel Approach to Analogues of Pyridoacridine Alkaloids. Beilstein J. Org. Chem. 2019, 15, 2304–2310. 10.3762/bjoc.15.222. 31598182 PMC6774065

[ref381] Khalil I. M.; Barker D.; Copp B. R. Bioinspired Syntheses of the Pyridoacridine Marine Alkaloids Demethyldeoxyamphimedine, Deoxyamphimedine, and Amphimedine. J. Org. Chem. 2016, 81, 282–289. 10.1021/acs.joc.5b02312. 26642369

[ref382] Delgado I. H.; Pascal S.; Wallabregue A.; Duwald R.; Besnard C.; Guénée L.; Nançoz C.; Vauthey E.; Tovar R. C.; Lunkley J. L.; et al. Functionalized Cationic [4]Helicenes with Unique Tuning of Absorption, Fluorescence and Chiroptical Properties up to the Far-Red Range. Chem. Sci. 2016, 7, 4685–4693. 10.1039/C6SC00614K. 29348909 PMC5772034

[ref383] Pascal S.; Besnard C.; Zinna F.; Di Bari L.; Le Guennic B.; Jacquemin D.; Lacour J. Zwitterionic [4]Helicene: A Water-Soluble and Reversible PH-Triggered ECD/CPL Chiroptical Switch in the UV and Red Spectral Regions. Org. Biomol. Chem. 2016, 14, 4590–4594. 10.1039/C6OB00752J. 27139039

[ref384] Labrador G. M.; Besnard C.; Bürgi T.; Poblador-Bahamonde A. I.; Bosson J.; Lacour J. Stereochemical Significance of O to N Atom Interchanges within Cationic Helicenes: Experimental and Computational Evidence of near Racemization to Remarkable Enantiospecificity. Chem. Sci. 2019, 10, 7059–7067. 10.1039/C9SC02127B. 31588273 PMC6676467

[ref385] Bosson J.; Labrador G. M.; Besnard C.; Jacquemin D.; Lacour J. Chiral Near-Infrared Fluorophores by Self-Promoted Oxidative Coupling of Cationic Helicenes with Amines/Enamines. Angew. Chem., Int. Ed. 2021, 60, 8733–8738. 10.1002/anie.202016643. 33481294

[ref386] Yamagami A.; Ishimura H.; Katori A.; Kuramochi K.; Tsubaki K. Syntheses and Properties of the V-Shaped Dimeric Xanthene Dyes. Org. Biomol. Chem. 2016, 14, 10963–10972. 10.1039/C6OB01967F. 27824208

[ref387] Yamagami A.; Kawano K.; Futaki S.; Kuramochi K.; Tsubaki K. Syntheses and Properties of Second-Generation V-Shaped Xanthene Dyes with Piperidino Groups. Tetrahedron 2017, 73, 7061–7066. 10.1016/j.tet.2017.10.064.

[ref388] Mokhtari Brikci-Nigassa N.; Nauton L.; Moreau P.; Mongin O.; Duval R. E.; Picot L.; Thiéry V.; Souab M.; Baratte B.; Ruchaud S.; et al. Functionalization of 9-Thioxanthone at the 1-Position: From Arylamino Derivatives to [1]Benzo(Thio)Pyrano[4,3,2-de]Benzothieno[2,3-b]Quinolines of Biological Interest. Bioorg. Chem. 2020, 94, 103347 10.1016/j.bioorg.2019.103347. 31810757

[ref389] Bornadiego A.; Díaz J.; Marcos C. F. Tandem Synthesis of 4-Aminoxanthones Is Controlled by a Water-Assisted Tautomerization: A General Straightforward Reaction. Org. Biomol. Chem. 2019, 17, 1410–1422. 10.1039/C8OB02527D. 30667448

[ref390] Li J.; Liu J.; Yin J.; Zhang Y.; Han W.; Lan J.; Wu D.; Bin Z.; You J. Double Ortho-C-H Activation/Annulation of Benzamides with Aryl Alkynes: A Route to Double-Helical Polycyclic Heteroaromatics. J. Org. Chem. 2019, 84, 15697–15705. 10.1021/acs.joc.9b02339. 31656071

[ref391] Numano M.; Nagami N.; Nakatsuka S.; Katayama T.; Nakajima K.; Tatsumi S.; Yasuda N.; Hatakeyama T. Synthesis of Boronate-Based Benzo[ *Fg* ]Tetracene and Benzo[ *Hi* ]Hexacene via Demethylative Direct Borylation. Chem. - Eur. J. 2016, 22, 11574–11577. 10.1002/chem.201602753. 27321480

[ref392] Fingerle M.; Maichle-Mössmer C.; Schundelmeier S.; Speiser B.; Bettinger H. F. Synthesis and Characterization of a Boron-Nitrogen-Boron Zigzag-Edged Benzo[Fg]Tetracene Motif. Org. Lett. 2017, 19, 4428–4431. 10.1021/acs.orglett.7b01873. 28812899

[ref393] Wei H.; Liu Y.; Gopalakrishna T. Y.; Phan H.; Huang X.; Bao L.; Guo J.; Zhou J.; Luo S.; Wu J.; et al. B-N-B Bond Embedded Phenalenyl and Its Anions. J. Am. Chem. Soc. 2017, 139, 15760–15767. 10.1021/jacs.7b07375. 29025264

[ref394] Wang X.; Zhang F.; Schellhammer K. S.; Machata P.; Ortmann F.; Cuniberti G.; Fu Y.; Hunger J.; Tang R.; Popov A. A.; et al. Synthesis of NBN-Type Zigzag-Edged Polycyclic Aromatic Hydrocarbons: 1,9-Diaza-9a-Boraphenalene as a Structural Motif. J. Am. Chem. Soc. 2016, 138, 11606–11615. 10.1021/jacs.6b04445. 27541867

[ref395] Fingerle M.; Stocker S.; Bettinger H. F. New Synthesis of a Dibenzoperylene Motif Featuring a Doubly Boron-Nitrogen-Doped Bay Region. Synthesis 2019, 51, 4147–4152. 10.1055/s-0039-1690687.

[ref396] Tasseroul J.; Lorenzo-Garcia M. M.; Dosso J.; Simon F.; Velari S.; De Vita A.; Tecilla P.; Bonifazi D. Probing Peripheral H-Bonding Functionalities in BN-Doped Polycyclic Aromatic Hydrocarbons. J. Org. Chem. 2020, 85, 3454–3464. 10.1021/acs.joc.9b03202. 32027511

[ref397] Hatakeyama T.; Shiren K.; Nakajima K.; Nomura S.; Nakatsuka S.; Kinoshita K.; Ni J.; Ono Y.; Ikuta T. Ultrapure Blue Thermally Activated Delayed Fluorescence Molecules: Efficient HOMO-LUMO Separation by the Multiple Resonance Effect. Adv. Mater. 2016, 28, 2777–2781. 10.1002/adma.201505491. 26865384

[ref398] aKondo Y.; Yoshiura K.; Kitera S.; Nishi H.; Oda S.; Gotoh H.; Sasada Y.; Yanai M.; Hatakeyama T. Narrowband Deep-Blue Organic Light-Emitting Diode Featuring an Organoboron-Based Emitter. Nature Photonics 2019, 13 (10), 678–682. 10.1038/s41566-019-0476-5.

[ref399] aOda S.; Kawakami B.; Kawasumi R.; Okita R.; Hatakeyama T. Multiple Resonance Effect-Induced Sky-Blue Thermally Activated Delayed Fluorescence with a Narrow Emission Band. Org. Lett. 2019, 21 (23), 9311–9314. 10.1021/acs.orglett.9b03342. 31613109

[ref400] Nishimura H.; Ishida N.; Shimazaki A.; Wakamiya A.; Saeki A.; Scott L. T.; Murata Y. Hole-Transporting Materials with a Two-Dimensionally Expanded π-System around an Azulene Core for Efficient Perovskite Solar Cells. J. Am. Chem. Soc. 2015, 137, 15656–15659. 10.1021/jacs.5b11008. 26651163

[ref401] Nishimura H.; Hasegawa Y.; Wakamiya A.; Murata Y. Development of Transparent Organic Hole-Transporting Materials Using Partially Oxygen-Bridged Triphenylamine Skeletons. Chem. Lett. 2017, 46, 817–820. 10.1246/cl.170164.

[ref402] Nishimura H.; Fukushima T.; Wakamiya A.; Murata Y.; Kaji H. The Influence of Quasiplanar Structures of Partially Oxygen-Bridged Triphenylamine Dimers on the Properties of Their Bulk Films. Bull. Chem. Soc. Jpn. 2016, 89, 726–732. 10.1246/bcsj.20160031.

[ref403] Li Q.; Shi C.; Huang M.; Wei X.; Yan H.; Yang C.; Yuan A. B- and N-Embedded Color-Tunable Phosphorescent Iridium Complexes and B-N Lewis Adducts with Intriguing Structural and Optical Changes. Chem. Sci. 2019, 10, 3257–3263. 10.1039/C8SC04252G. 30996910 PMC6429608

[ref404] Shi C.; Li F.; Li Q.; Zhao W.; Cao Y.; Zhao Q.; Yuan A. B- and N-Embedded π-Conjugation Units Tuning Intermolecular Interactions and Optical Properties of Platinum(II) Complexes. Inorg. Chem. 2021, 60, 525–534. 10.1021/acs.inorgchem.0c03078. 33378182

[ref405] Buyukcakir O.; Yuksel R.; Jiang Y.; Lee S. H.; Seong W. K.; Chen X.; Ruoff R. S. Synthesis of Porous Covalent Quinazoline Networks (CQNs) and Their Gas Sorption Properties. Angew. Chem., Int. Ed. 2019, 58, 872–876. 10.1002/anie.201813075. 30456920

[ref406] Shensky W. M.; Ferry M. J.; O’Donnell R. M.; Ensley T. R.; Shi J. Nonlinear Optical Characterization of Multinuclear Iridium Compounds Containing Tricycloquinazoline. Appl. Opt. 2017, 56, B179–B183. 10.1364/AO.56.00B179. 28157880

[ref407] Boldt S.; Parpart S.; Villinger A.; Ehlers P.; Langer P. Synthesis and Properties of Aza-Ullazines. Angew. Chem., Int. Ed. 2017, 56, 4575–4578. 10.1002/anie.201701347. 28332753

[ref408] Richter M.; Schellhammer K. S.; Machata P.; Cuniberti G.; Popov A.; Ortmann F.; Berger R.; Müllen K.; Feng X. Polycyclic Heteroaromatic Hydrocarbons Containing a Benzoisoindole Core. Org. Chem. Front. 2017, 4, 847–852. 10.1039/C7QO00180K.

[ref409] Richter M.; Fu Y.; Dmitrieva E.; Weigand J. J.; Popov A.; Berger R.; Liu J.; Feng X. Polycyclic Aromatic Hydrocarbons Containing A Pyrrolopyridazine Core. ChemPlusChem 2019, 84, 613–618. 10.1002/cplu.201900031. 31944030

[ref410] Richter M.; Hahn S.; Dmitrieva E.; Rominger F.; Popov A.; Bunz U. H. F.; Feng X.; Berger R. Helical Ullazine-Quinoxaline-Based Polycyclic Aromatic Hydrocarbons. Chem. - Eur. J. 2019, 25, 1345–1352. 10.1002/chem.201804751. 30397968

[ref411] Li Q.-Q.; Ochiai K.; Lee C.-A.; Ito S. Synthesis of π-Extended Imidazoles by 1,3-Dipolar Cycloaddition of Polycyclic Aromatic Azomethine Ylides with Nitriles. Org. Lett. 2020, 22, 6132–6137. 10.1021/acs.orglett.0c02203. 32663016

[ref412] Riss A.; Richter M.; Paz A. P.; Wang X.-Y.; Raju R.; He Y.; Ducke J.; Corral E.; Wuttke M.; Seufert K.; et al. Polycyclic Aromatic Chains on Metals and Insulating Layers by Repetitive [3 + 2] Cycloadditions. Nat. Commun. 2020, 11, 1–8. 10.1038/s41467-020-15210-2. 32753628 PMC7403408

[ref413] Miao D.; Aumaitre C.; Morin J.-F. Photochemical Synthesis of π-Extended Ullazine Derivatives as New Electron Donors for Efficient Conjugated D-A Polymers. J. Mater. Chem. C 2019, 7, 3015–3024. 10.1039/C8TC05288C.

[ref414] Skabeev A.; Zschieschang U.; Zagranyarski Y.; Klauk H.; Müllen K.; Li C. Carbonyl-Functionalized Cyclazines as Colorants and Air-Stable n-Type Semiconductors. Org. Lett. 2018, 20, 1409–1412. 10.1021/acs.orglett.8b00183. 29446956

[ref415] Skidin D.; Eisenhut F.; Richter M.; Nikipar S.; Krüger J.; Ryndyk D. A.; Berger R.; Cuniberti G.; Feng X.; Moresco F. On-Surface Synthesis of Nitrogen-Doped Nanographenes with 5–7 Membered Rings. Chem. Commun. 2019, 55, 4731–4734. 10.1039/C9CC00276F. 30942792

[ref416] Krzeszewski M.; Kodama T.; Espinoza E. M.; Vullev V. I.; Kubo T.; Gryko D. T. Nonplanar Butterfly-Shaped π-Expanded Pyrrolopyrroles. Chem. - Eur. J. 2016, 22, 16478–16488. 10.1002/chem.201603282. 27659591

[ref417] Sheng W.; Zheng Y.-Q.; Wu Q.; Chen K.; Li M.; Jiao L.; Hao E.; Wang J.-Y.; Pei J. Synthesis, Characterization, and Tunable Semiconducting Properties of Aza-BODIPY Derived Polycyclic Aromatic Dyes. Sci. China: Chem. 2020, 63, 1240–1245. 10.1007/s11426-020-9807-7.

[ref418] Tokimaru Y.; Ito S.; Nozaki K. Synthesis of Pyrrole-Fused Corannulenes: 1,3-Dipolar Cycloaddition of Azomethine Ylides to Corannulene. Angew. Chem., Int. Ed. 2017, 56, 15560–15564. 10.1002/anie.201707087. 29071787

[ref419] Tokimaru Y.; Ito S.; Nozaki K. A Hybrid of Corannulene and Azacorannulene: Synthesis of a Highly Curved Nitrogen-Containing Buckybowl. Angew. Chem., Int. Ed. 2018, 57, 9818–9822. 10.1002/anie.201805678. 29878551

[ref420] Kawahara K. P.; Matsuoka W.; Ito H.; Itami K. Synthesis of Nitrogen-Containing Polyaromatics by Aza-Annulative π-Extension of Unfunctionalized Aromatics. Angew. Chem., Int. Ed. 2020, 59, 6383–6388. 10.1002/anie.201913394. 32011794

[ref421] Saha M.; Bao Y.-H.; Zhou C. A Diindole-Fused Corannulene Imide Derivative: Synthesis and Properties. Chem. Lett. 2018, 47, 1383–1386. 10.1246/cl.180680.

[ref422] Rajeshkumar V.; Stuparu M. C. A Photochemical Approach to Aromatic Extension of the Corannulene Nucleus. Chem. Commun. 2016, 52, 9957–9960. 10.1039/C6CC04910A. 27440449

[ref423] Ghosh A.; Csókás D.; Budanović M.; Webster R. D.; Pápai I.; Stuparu M. C. Synthesis of Azahelicenes through Mallory Reaction of Imine Precursors: Corannulene Substrates Provide an Exception to the Rule in Oxidative Photocyclizations of Diarylethenes. Chem. Sci. 2021, 12, 3977–3983. 10.1039/D0SC06730J. 34163668 PMC8179518

[ref424] Barát V.; Budanović M.; Halilovic D.; Huh J.; Webster R. D.; Mahadevegowda S. H.; Stuparu M. C. A General Approach to Non-Fullerene Electron Acceptors Based on the Corannulene Motif. Chem. Commun. 2019, 55, 3113–3116. 10.1039/C9CC00327D. 30789623

[ref425] Wang Y.; Allemann O.; Balaban T. S.; Vanthuyne N.; Linden A.; Baldridge K. K.; Siegel J. S. Chiral Atropisomeric Indenocorannulene Bowls: Critique of the Cahn-Ingold-Prelog Conception of Molecular Chirality. Angew. Chem., Int. Ed. 2018, 57, 6470–6474. 10.1002/anie.201801325. 29656583

[ref426] Tian X.; Roch L. M.; Vanthuyne N.; Xu J.; Baldridge K. K.; Siegel J. S. Azaindenocorannulenes: Synthesis, Properties, and Chirality. Org. Lett. 2019, 21, 3510–3513. 10.1021/acs.orglett.9b00718. 30995051

[ref427] Gu X.; Li H.; Shan B.; Liu Z.; Miao Q. Synthesis, Structure, and Properties of Tetrabenzo[7]Circulene. Org. Lett. 2017, 19, 2246–2249. 10.1021/acs.orglett.7b00714. 28421768

[ref428] Tsefrikas V. M.; Greene A. K.; Scott L. T. 5-Azadibenzo[*a*,*g*]Corannulene. Org. Chem. Front. 2017, 4, 688–698. 10.1039/C6QO00831C.

[ref429] Nakatsuka S.; Yasuda N.; Hatakeyama T. Four-Step Synthesis of B_2_N_2_-Embedded Corannulene. J. Am. Chem. Soc. 2018, 140, 13562–13565. 10.1021/jacs.8b08197. 30251839

[ref430] Nagano T.; Nakamura K.; Tokimaru Y.; Ito S.; Miyajima D.; Aida T.; Nozaki K. Functionalization of Azapentabenzocorannulenes by Fivefold C-H Borylation and Cross-Coupling Arylation: Application to Columnar Liquid-Crystalline Materials. Chem. - Eur. J. 2018, 24, 14075–14078. 10.1002/chem.201803676. 30043435

[ref431] Zhou Z.; Wei Z.; Tokimaru Y.; Ito S.; Nozaki K.; Petrukhina M. A. Stepwise Reduction of Azapentabenzocorannulene. Angew. Chem., Int. Ed. 2019, 58, 12107–12111. 10.1002/anie.201906748. 31251429

[ref432] Yokoi H.; Hiroto S.; Sakamaki D.; Seki S.; Shinokubo H. Supramolecular Assemblies of a Nitrogen-Embedded Buckybowl Dimer with C60. Chem. Sci. 2018, 9, 819–824. 10.1039/C7SC04453D. 29629149 PMC5872494

[ref433] Takeda M.; Hiroto S.; Yokoi H.; Lee S.; Kim D.; Shinokubo H. Azabuckybowl-Based Molecular Tweezers as C _60_ and C _70_ Receptors. J. Am. Chem. Soc. 2018, 140, 6336–6342. 10.1021/jacs.8b02327. 29660980

[ref434] Yokoi H.; Hiroto S.; Shinokubo H. Reversible σ-Bond Formation in Bowl-Shaped π-Radical Cations: The Effects of Curved and Planar Structures. J. Am. Chem. Soc. 2018, 140, 4649–4655. 10.1021/jacs.8b00798. 29542920

[ref435] Tan Q.; Zhou D.; Zhang T.; Liu B.; Xu B. Iodine-Doped Sumanene and Its Application for the Synthesis of Chalcogenasumanenes and Silasumanenes. Chem. Commun. 2017, 53, 10279–10282. 10.1039/C7CC05885C. 28868542

[ref436] Jiang M.; Guo J.; Liu B.; Tan Q.; Xu B. Synthesis of Tellurium-Containing π-Extended Aromatics with Room-Temperature Phosphorescence. Org. Lett. 2019, 21, 8328–8333. 10.1021/acs.orglett.9b03106. 31560555

[ref437] Zhou D.; Gao Y.; Liu B.; Tan Q.; Xu B. Synthesis of Silicon and Germanium-Containing Heterosumanenes via Rhodium-Catalyzed Cyclodehydrogenation of Silicon/Germanium-Hydrogen and Carbon-Hydrogen Bonds. Org. Lett. 2017, 19, 4628–4631. 10.1021/acs.orglett.7b02254. 28832161

[ref438] Zhang T.; Deng G.; Li H.; Liu B.; Tan Q.; Xu B. Cyclization of 2-Biphenylthiols to Dibenzothiophenes under PdCl_2_/DMSO Catalysis. Org. Lett. 2018, 20, 5439–5443. 10.1021/acs.orglett.8b02347. 30106302

[ref439] Liu Y.-M.; Xia D.; Li B.-W.; Zhang Q.-Y.; Sakurai T.; Tan Y.-Z.; Seki S.; Xie S.-Y.; Zheng L.-S. Functional Sulfur-Doped Buckybowls and Their Concave-Convex Supramolecular Assembly with Fullerenes. Angew. Chem., Int. Ed. 2016, 55, 13047–13051. 10.1002/anie.201606383. 27650894

[ref440] Furukawa S.; Wu J.; Koyama M.; Hayashi K.; Hoshino N.; Takeda T.; Suzuki Y.; Kawamata J.; Saito M.; Akutagawa T. Ferroelectric Columnar Assemblies from the Bowl-to-Bowl Inversion of Aromatic Cores. Nat. Commun. 2021, 12, 768 10.1038/s41467-021-21019-4. 33536427 PMC7859410

[ref441] Furukawa S.; Suda Y.; Kobayashi J.; Kawashima T.; Tada T.; Fujii S.; Kiguchi M.; Saito M. Triphosphasumanene Trisulfide: High Out-of-Plane Anisotropy and Janus-Type π-Surfaces. J. Am. Chem. Soc. 2017, 139, 5787–5792. 10.1021/jacs.6b12119. 28387119

[ref442] Wang S.; Yan C.; Shang J.; Wang W.; Yuan C.; Zhang H.-L.; Shao X. Doping Sumanene with Both Chalcogens and Phosphorus(V): One-Step Synthesis, Coordination, and Selective Response Toward Ag^I^. Angew. Chem., Int. Ed. 2019, 58, 3819–3823. 10.1002/anie.201813070. 30672088

[ref443] Furukawa S.; Hayashi K.; Yamagishi K.; Saito M. Synthesis and Properties of Spiro-Type Heterasumanenes Containing Group 14 Elements as Bridging Atoms. Mater. Chem. Front. 2018, 2, 929–934. 10.1039/C7QM00590C.

[ref444] Hou X.; Zhu Y.; Qin Y.; Chen L.; Li X.; Zhang H.-L.; Xu W.; Zhu D.; Shao X. Tris(*S*,*S*-Dioxide)-Trithiasumanene: Strong Fluorescence and Cocrystal with 1,2,6,7,10,11-Hexabutoxytriphenylene. Chem. Commun. 2017, 53, 1546–1549. 10.1039/C6CC09531C. 28094368

[ref445] Wang S.; Li X.; Hou X.; Sun Y.; Shao X. Tritellurasumanene: Ultrasound Assisted One-Pot Synthesis and Extended Valence Adducts with Bromine. Chem. Commun. 2016, 52, 14486–14489. 10.1039/C6CC08170C. 27904895

[ref446] Sun Y.; Li X.; Sun C.; Shen H.; Hou X.; Lin D.; Zhang H.-L.; Di C.; Zhu D.; Shao X. Trichalcogenasumanene Ortho-Quinones: Synthesis, Properties, and Transformation into Various Heteropolycycles. Angew. Chem., Int. Ed. 2017, 56, 13470–13474. 10.1002/anie.201707397. 28834589

[ref447] Wang S.; Shang J.; Yan C.; Wang W.; Yuan C.; Zhang H.-L.; Shao X. Trichalcogenasumanenes Containing Various Chalcogen Atoms: Synthesis, Structure, Properties, and Chemical Reactivity. Org. Chem. Front. 2019, 6, 263–272. 10.1039/C8QO01220B.

[ref448] Wang Y.; Deng J.; Chen J.; Cao F.; Hou Y.; Yang Y.; Deng X.; Yang J.; Wu L.; Shao X.; et al. Dechalcogenization of Aryl Dichalcogenides to Synthesize Aryl Chalcogenides via Copper Catalysis. ACS Catal. 2020, 10, 2707–2712. 10.1021/acscatal.9b04931.

[ref449] Geng R.; Hou X.; Sun Y.; Yan C.; Wu Y.; Zhang H.-L.; Shao X. Driving π-Plane to π-Bowl through Lateral Coordination at Room Temperature. Mater. Chem. Front. 2018, 2, 1456–1461. 10.1039/C8QM00168E.

[ref450] Hou X.; Li X.; Sun C.; Chen L.; Sun Y.; Liu Z.; Zhang H.-L.; Shao X. Dissecting Trichalcogenasumanenes: π-Bowl to Planar, Invertible Curvature, and Chiral Polycycles. Chem. - Eur. J. 2017, 23, 14375–14383. 10.1002/chem.201703469. 28758338

[ref451] Hou X.; Sun J.; Liu Z.; Yan C.; Song W.; Zhang H.-L.; Zhou S.; Shao X. Opening Two Benzene Rings on Trichalcogenasumanenes toward High Performance Organic Optical-Limiting Materials. Chem. Commun. 2018, 54, 10981–10984. 10.1039/C8CC05480K. 30209446

[ref452] Sun J.; Sun Y.; Yan C.; Lin D.; Xie Z.; Zhou S.; Yuan C.; Zhang H.-L.; Shao X. Remarkable Nonlinear Optical Response of Pyrazine-Fused Trichalcogenasumanenes and Their Application for Optical Power Limiting. J. Mater. Chem. C 2018, 6, 13114–13119. 10.1039/C8TC04778B.

[ref453] Fu B.; Wang C.; Sun Y.; Yao J.; Wang Y.; Ge F.; Yang F.; Liu Z.; Dang Y.; Zhang X.; et al. A “Phase Separation” Molecular Design Strategy Towards Large-Area 2D Molecular Crystals. Adv. Mater. 2019, 31, 1901437 10.1002/adma.201901437. 31268577

[ref454] Liu L.; Yan C.; Li Y.; Liu Z.; Yuan C.; Zhang H.-L.; Shao X. Tetrathiafulvalene-Fused Heterabuckybowl: Protonation-Induced Electron Transfer and Self-Sensitized Photooxidation. Chem. - Eur. J. 2020, 26, 7083–7091. 10.1002/chem.201905732. 32073723

[ref455] Kaewmati P.; Tan Q.; Higashibayashi S.; Yakiyama Y.; Sakurai H. Synthesis of Triaryltriazasumanenes. Chem. Lett. 2017, 46, 146–148. 10.1246/cl.160978.

[ref456] Kaewmati P.; Yakiyama Y.; Ohtsu H.; Kawano M.; Haesuwannakij S.; Higashibayashi S.; Sakurai H. Tris(2-Hydroxyphenyl)Triazasumanene: Bowl-Shaped Excited-State Intramolecular Proton Transfer (ESIPT) Fluorophore Coupled with Aggregation-Induced Enhanced Emission (AIEE). Mater. Chem. Front. 2018, 2, 514–519. 10.1039/C7QM00530J.

[ref457] Tan Q.; Kaewmati P.; Higashibayashi S.; Kawano M.; Yakiyama Y.; Sakurai H. Triazasumanene: An Isoelectronic Heteroanalogue of Sumanene. Bull. Chem. Soc. Jpn. 2018, 91, 531–537. 10.1246/bcsj.20170384.

[ref458] Chen F.; Tanaka T.; Osuka A. Exploring the “Fold-in” Strategy toward the Construction of a Highly-Strained Triazasumanene Skeleton. Chem. Commun. 2017, 53, 2705–2708. 10.1039/C7CC00329C. 28197583

[ref459] Upadhyay G. M.; Talele H. R.; Bedekar A. V. Synthesis and Photophysical Properties of Aza[n]Helicenes. J. Org. Chem. 2016, 81, 7751–7759. 10.1021/acs.joc.6b01395. 27439443

[ref460] Nagata Y.; Kato S.; Miyake Y.; Shinokubo H. Synthesis of Tetraaza[8]Circulenes from Tetrathia[8]Circulenes through an SNAr-Based Process. Org. Lett. 2017, 19, 2718–2721. 10.1021/acs.orglett.7b01074. 28489399

[ref461] Murase H.; Nagata Y.; Akahori S.; Shinokubo H.; Miyake Y. Aggregation-Induced Emission in Tetrathia[8]Circulene Octaoxides via Restriction of the Dynamic Motion of Their Negatively Curved π-Frameworks. Chem. - Asian J. 2020, 15, 3873–3877. 10.1002/asia.202001129. 32975006

[ref462] Matsuo Y.; Tanaka T.; Osuka A. Highly Stable Radical Cations of N,N’-Diarylated Tetrabenzotetraaza[8]Circulene. Chem. - Eur. J. 2020, 26, 8144–8152. 10.1002/chem.202001934. 32342540

[ref463] Matsuo Y.; Tanaka T.; Osuka A. Diazadimethano[8]Circulene: Synthesis, Structure, Properties, and Isolation of Stable Radical Cation. Chem. Lett. 2020, 49, 959–962. 10.1246/cl.200336.

[ref464] Morimoto Y.; Chen F.; Matsuo Y.; Kise K.; Tanaka T.; Osuka A. Improved Synthesis of Ortho-Phenylene-Bridged Cyclic Tetrapyrroles and Oxidative Fusion Reactions Toward Substituted Tetraaza[8]Circulenes. Chem. - Asian J. 2021, 16, 648–655. 10.1002/asia.202001459. 33428328

[ref465] Chen F.; Hong Y. S.; Kim D.; Tanaka T.; Osuka A. Sequential N-Alkylations of Tetrabenzotetraaza[8]Circulene as a Tool To Tune Its Optical Properties. ChemPlusChem 2017, 82, 1048–1051. 10.1002/cplu.201600537. 31961605

[ref466] Serizawa Y.; Akahori S.; Kato S.; Sakai H.; Hasobe T.; Miyake Y.; Shinokubo H. Synthesis of Tetrasilatetrathia[8]Circulenes by a Fourfold Intramolecular Dehydrogenative Silylation of C-H Bonds. Chem. - Eur. J. 2017, 23, 6948–6952. 10.1002/chem.201700729. 28370827

[ref467] Kato S.; Akahori S.; Serizawa Y.; Lin X.; Yamauchi M.; Yagai S.; Sakurai T.; Matsuda W.; Seki S.; Shinokubo H.; et al. Systematic Synthesis of Tetrathia[8]Circulenes: The Influence of Peripheral Substituents on the Structures and Properties in Solution and Solid States. J. Org. Chem. 2020, 85, 62–69. 10.1021/acs.joc.9b01655. 31401831

[ref468] Akahori S.; Sasamori T.; Shinokubo H.; Miyake Y. Enthalpically and Entropically Favorable Self-Assembly: Synthesis of C4h-Symmetric Tetraazatetrathia[8]Circulenes by Regioselective Introduction of Pyridine Rings. Chem. - Eur. J. 2021, 27, 5675–5682. 10.1002/chem.202005077. 33300177

[ref469] Akahori S.; Sakai H.; Hasobe T.; Shinokubo H.; Miyake Y. Synthesis and Photodynamics of Tetragermatetrathia[8]Circulene. Org. Lett. 2018, 20, 304–307. 10.1021/acs.orglett.7b03764. 29281294

[ref470] Xiong X.; Deng C.-L.; Li Z.; Peng X.-S.; Wong H. N. C. Quasi-Planar Diazadithio and Diazodiseleno[8]Circulenes: Synthesis, Structures and Properties. Org. Chem. Front. 2017, 4, 682–687. 10.1039/C6QO00662K.

[ref471] Lousen B.; Pedersen S. K.; Bols P.; Hansen K. H.; Pedersen M. R.; Hammerich O.; Bondarchuk S.; Minaev B.; Baryshnikov G. V.; Ågren H.; et al. Compressing a Non-Planar Aromatic Heterocyclic [7]Helicene to a Planar Hetero[8]Circulene. Chem. - Eur. J. 2020, 26, 4935–4940. 10.1002/chem.201905339. 32052498

[ref472] Chen F.; Tanaka T.; Mori T.; Osuka A. Synthesis, Structures, and Optical Properties of Azahelicene Derivatives and Unexpected Formation of Azahepta[8]Circulenes. Chem. - Eur. J. 2018, 24, 7489–7497. 10.1002/chem.201800617. 29533480

[ref473] Matsuo Y.; Chen F.; Kise K.; Tanaka T.; Osuka A. Facile Synthesis of Fluorescent Hetero[8]Circulene Analogues with Tunable Solubilities and Optical Properties. Chem. Sci. 2019, 10, 11006–11012. 10.1039/C9SC05087F. 32110354 PMC7012041

[ref474] Zhang S.; Liu X.; Li C.; Li L.; Song J.; Shi J.; Morton M.; Rajca S.; Rajca A.; Wang H. Thiophene-Based Double Helices: Syntheses, X-Ray Structures, and Chiroptical Properties. J. Am. Chem. Soc. 2016, 138, 10002–10010. 10.1021/jacs.6b05709. 27440376

[ref475] Li B.; Zhang S.; Li L.; Ma Z.; Li C.; Xu L.; Wang H. All-Thiophene-Based Double Helix: Synthesis, Crystal Structure, Chiroptical Property and Arylation. ACS Omega 2018, 3, 16014–16020. 10.1021/acsomega.8b02492. 31458239 PMC6644213

[ref476] Li L.; Zhao S.; Li B.; Xu L.; Li C.; Shi J.; Wang H. From Saddle-Shaped to Planar Cyclic Oligothienoacenes: Stepped-Cyclization and Their Applications in OFETs. Org. Lett. 2018, 20, 2181–2185. 10.1021/acs.orglett.8b00482. 29600862

[ref477] Nakamura K.; Li Q.-Q.; Krejčí O.; Foster A. S.; Sun K.; Kawai S.; Ito S. On-Surface Synthesis of a π-Extended Diaza[8]Circulene. J. Am. Chem. Soc. 2020, 142, 11363–11369. 10.1021/jacs.0c02534. 32413264

[ref478] Pedersen S. K.; Eriksen K.; Karaush-Karmazin N. N.; Minaev B.; Ågren H.; Baryshnikov G. V.; Pittelkow M. Anti-Aromatic versus Induced Paratropicity: Synthesis and Interrogation of a Dihydro-Diazatrioxa[9]Circulene with a Proton Placed Directly above the Central Ring. Angew. Chem., Int. Ed. 2020, 59, 5144–5150. 10.1002/anie.201913552. 31961984

[ref479] Pedersen S. K.; Eriksen K.; Ågren H.; Minaev B. F.; Karaush-Karmazin N. N.; Hammerich O.; Baryshnikov G. V.; Pittelkow M. A Fully Conjugated Planar Heterocyclic [9]Circulene. J. Am. Chem. Soc. 2020, 142, 14058–14063. 10.1021/jacs.0c05898. 32787263

[ref480] Das S.; Herng T. S.; Zafra J. L.; Burrezo P. M.; Kitano M.; Ishida M.; Gopalakrishna T. Y.; Hu P.; Osuka A.; Casado J.; et al. Fully Fused Quinoidal/Aromatic Carbazole Macrocycles with Poly-Radical Characters. J. Am. Chem. Soc. 2016, 138, 7782–7790. 10.1021/jacs.6b04539. 27248181

[ref481] Yang Y.; Chu M.; Miao Q. From Phenanthrylene Butadiynylene Macrocycles to S-Heterocycloarenes. Org. Lett. 2018, 20, 4259–4262. 10.1021/acs.orglett.8b01668. 29953241

[ref482] Yang L.; Zhang N.; Han Y.; Zou Y.; Qiao Y.; Chang D.; Zhao Y.; Lu X.; Wu J.; Liu Y. A Sulfur-Containing Hetero-Octulene: Synthesis, Host-Guest Properties, and Transistor Applications. Chem. Commun. 2020, 56, 9990–9993. 10.1039/D0CC04289G. 32724946

[ref483] Lu X.; An D.; Han Y.; Zou Y.; Qiao Y.; Zhang N.; Chang D.; Wu J.; Liu Y. A Cyclopenta-Fused Dibenzo[b, d]Thiophene-Co-Phenanthrene Macrocyclic Tetraradicaloid. Chem. Sci. 2021, 12, 3952–3957. 10.1039/D0SC06185A. 34163665 PMC8179481

[ref484] Marco A. B.; Cortizo-Lacalle D.; Perez-Miqueo I.; Valenti G.; Boni A.; Plas J.; Strutyński K.; De Feyter S.; Paolucci F.; Montes M.; et al. Twisted Aromatic Frameworks: Readily Exfoliable and Solution-Processable Two-Dimensional Conjugated Microporous Polymers. Angew. Chem., Int. Ed. 2017, 56, 6946–6951. 10.1002/anie.201700271. PMC5485174 28318084

[ref485] Mahmood J.; Lee E. K.; Noh H.-J.; Ahmad I.; Seo J.-M.; Im Y.-K.; Jeon J.-P.; Kim S.-J.; Oh J. H.; Baek J.-B. Fused Aromatic Network with Exceptionally High Carrier Mobility. Adv. Mater. 2021, 33, 2004707 10.1002/adma.202004707. 33470474

[ref486] An C.; Zhou S.; Baumgarten M. Condensed Derivatives of Thiadiazoloquinoxaline as Strong Acceptors. Cryst. Growth Des. 2015, 15, 1934–1938. 10.1021/acs.cgd.5b00105.

[ref487] Biegger P.; Schaffroth M.; Brödner K.; Tverskoy O.; Rominger F.; Bunz U. H. F. Bisalkynylated 3,6-Diiminocyclohexa-1,4-Diene-1,4-Diamine. Chem. Commun. 2015, 51, 14844–14847. 10.1039/C5CC05427C. 26221848

[ref488] Ganschow M.; Koser S.; Hodecker M.; Rominger F.; Freudenberg J.; Dreuw A.; Bunz U. H. F. Azaacenes Bearing Five-Membered Rings. Chem. - Eur. J. 2018, 24, 13667–13675. 10.1002/chem.201802900. 29947438

[ref489] Kothavale S.; Sekar N. Novel Pyrazino-Phenanthroline Based Rigid Donor-π-Acceptor Compounds: A Detail Study of Optical Properties, Acidochromism, Solvatochromism and Structure-Property Relationship. Dyes Pigm. 2017, 136, 31–45. 10.1016/j.dyepig.2016.08.032.

[ref490] Zhang C.; Zeng C.-C.; Lai S.-H.; Xing D.-G.; Li W.; Han B.-J.; Liu Y.-J. Synthesis, Cytotoxicity in Vitro, Apoptosis, Cell Cycle Arrest and Comet Assay of Asymmetry Ruthenium(II) Complexes. Polyhedron 2016, 106, 115–124. 10.1016/j.poly.2015.12.058.

[ref491] Shuler W. G.; Parvathaneni S. P.; Rodriguez J. B.; Lewis T. N.; Berges A. J.; Bardeen C. J.; Krische M. J. Synthesis and Photophysical Properties of Soluble N-Doped Rubicenes via Ruthenium-Catalyzed Transfer Hydrogenative Benzannulation. Chem. - Eur. J. 2021, 27, 4898–4902. 10.1002/chem.202100134. 33576516

[ref492] Tkachenko Yu. N.; Popov L. D.; Pozharskii A. F.; Borodkin S. A.; Levchenkov S. I. Nitro Derivatives of Pyrrolo[3,2-d]Pyrimidine-2,4-Diones: Synthesis of Amines and New Polynuclear Heterocycles Based Thereon. Russ. J. Org. Chem. 2017, 53, 1564–1572. 10.1134/S1070428017100128.

[ref493] Rodríguez-Sanz A.; Sánchez-Alonso P.; Bellón T.; Alajarín R.; Martínez-Cabeza V.; Selgas R.; Vaquero J. J.; Álvarez-Builla J. Synthesis and Biological Evaluation of Pyridazino[1’,6’:1,2]Pyrido[3,4-b]Indolinium and Pyridazino[1,6-a]Benzimidazolium Salts as Anti-Inflammatory Agents. Eur. J. Med. Chem. 2015, 93, 83–92. 10.1016/j.ejmech.2015.01.060. 25659754

[ref494] Gang M.-Y.; Liu J.-Q.; Wang X.-S. CuI-Catalyzed Sonogashira Reaction for the Efficient Synthesis of 1H-Imidazo[2,1-a]Isoquinoline Derivatives. Tetrahedron 2017, 73, 4698–4705. 10.1016/j.tet.2017.06.043.

[ref495] Filatova E. A.; Gulevskaya A. V.; Pozharskii A. F.; Suslonov V. V. The Sonogashira Coupling of 2- and 4-Ethynyl Derivatives of Proton Sponge with 1,8-Diiodonaphthalene: Novel Cascade Transformations into Naphtho[1,2-k]Fluoranthenes and Acenaphtho[1,2-b]Benzo[g]Indoles. Tetrahedron 2018, 74, 165–173. 10.1016/j.tet.2017.11.058.

[ref496] Wu H.; Fang R.; Tao J.; Wang D.; Qiao X.; Yang X.; Hartl F.; Li H. Diacenaphthylene-Fused Benzo[1,2-b:4,5-B’]Dithiophenes: Polycyclic Heteroacenes Containing Full-Carbon Five-Membered Aromatic Rings. Chem. Commun. 2017, 53, 751–754. 10.1039/C6CC09184A. 27995232

[ref497] Murata M.; Togo M.; Mishima D.; Harada A.; Muraoka M. Benzo- and Thieno-Annulated Tetracenes: A One-Pot Synthesis via Cross-Dehydrogenative Annulation. Org. Lett. 2020, 22, 4160–4163. 10.1021/acs.orglett.0c01244. 32383601

[ref498] Chaolumen Murata M.; Wakamiya A.; Murata Y. Dithieno-Fused Polycyclic Aromatic Hydrocarbon with a Pyracylene Moiety: Strong Antiaromatic Contribution to the Electronic Structure. Org. Lett. 2017, 19, 826–829. 10.1021/acs.orglett.6b03819. 28133961

[ref499] Smet M.; Van Dijk J.; Dehaen W. An Improved Synthesis of Substituted Rubicenes Providing Access to Heterocyclic Rubicene Analogues. Synlett 1999, 1999, 495–497. 10.1055/s-1999-2624.

[ref500] Mohebbi A. R.; Wudl F. Electron-Accepting Dithiarubicene (Emeraldicene) and Derivatives Prepared by Unprecedented Nucleophilic Hydrogen Substitution by Alkyllithium Reagents. Chem. - Eur. J. 2011, 17, 2642–2646. 10.1002/chem.201002608. 21264966

[ref501] Wang M.; Mohebbi A. R.; Sun Y.; Wudl F. Ribbons, Vesicles, and Baskets: Supramolecular Assembly of a Coil-Plate-Coil Emeraldicene Derivative. Angew. Chem., Int. Ed. 2012, 51, 6920–6924. 10.1002/anie.201201796. 22674681

[ref502] Chao Y.-C.; Yeh S.-C.; Hsu H.-L.; Jiang B.-H.; Sun K.-H.; Chen C.-T.; Chen C.-P.; Jeng R.-J. Visibly Transparent Conjugated Polymers Based on Non-Alternant Cyclopenta-Fused Emeraldicene for Polymer Solar Cells. Org. Electron. 2017, 49, 114–122. 10.1016/j.orgel.2017.06.049.

[ref503] Bheemireddy S. R.; Hautzinger M. P.; Li T.; Lee B.; Plunkett K. N. Conjugated Ladder Polymers by a Cyclopentannulation Polymerization. J. Am. Chem. Soc. 2017, 139, 5801–5807. 10.1021/jacs.6b12916. 28371580

[ref504] Ogawa N.; Yamaoka Y.; Yamada K.; Takasu K. Synthesis of π-Extended Fluoranthenes via a KHMDS-Promoted Anionic-Radical Reaction Cascade. Org. Lett. 2017, 19, 3327–3330. 10.1021/acs.orglett.7b01538. 28590747

[ref505] Martínez Á. M.; Alonso I.; Rodríguez N.; Gómez Arrayás R.; Carretero J. C. Rhodium-Catalyzed Copper-Assisted Intermolecular Domino C-H Annulation of 1,3-Diynes with Picolinamides: Access to Pentacyclic π-Extended Systems. Chem. - Eur. J. 2019, 25, 5733–5742. 10.1002/chem.201900162. 30734965

[ref506] Shi Q.; Shi X.; Feng C.; Wu Y.; Zheng N.; Liu J.; Wu X.; Chen H.; Peng A.; Li J.; et al. Synthetic Routes for Heteroatom-Containing Alkylated/Arylated Polycyclic Aromatic Hydrocarbons. Angew. Chem., Int. Ed. 2021, 60, 2924–2928. 10.1002/anie.202014108. 33107179

[ref507] Zhang G.; Rominger F.; Mastalerz M. Fused π-Extended Truxenes via a Threefold Borylation as the Key Step. Chem. - Eur. J. 2016, 22, 3084–3093. 10.1002/chem.201504621. 26833764

[ref508] Bheemireddy S. R.; Hussain W. A.; Uddin A.; Du Y.; Hautzinger M. P.; Kevorkian P. V.; Petrie F. A.; Plunkett K. N. Cyclopentannulation and Cyclodehydrogenation of Isomerically Pure 5,11-Dibromo-Anthradithiophenes Leading to Contorted Aromatics. Chem. Commun. 2018, 54, 14140–14143. 10.1039/C8CC07327A. 30500004

[ref509] Stadlbauer W.; Dang V. H.; Guttenberger N. 5-Unsubstituted Pyrido[3,2,1-Jk]Carbazol-6-Ones: Syntheses, Substitution, and Cyclization Reactions. J. Heterocycl. Chem. 2015, 52, 114–123. 10.1002/jhet.1994.

[ref510] Sen’ko O. A.; Dybenko A. G.; Garazd M. M.; Kartsev V. G. Synthesis of Benzoannelated Canthin-6-One Analogs. Chem. Nat. Compd. 2017, 53, 523–528. 10.1007/s10600-017-2037-9.

[ref511] Zhao J.; Li R.; Ai W.; Dong D.; Li J.; Chen L.; Xie L.; Yu T.; Huang W. Pi-Extended Diindole-Fused Azapentacenone: Synthesis, Characterization, and Photophysical and Lithium-Storage Properties. Chem. - Asian J. 2016, 11, 1382–1387. 10.1002/asia.201501366. 26717256

[ref512] Pandit P.; Nakamura T.; Higashibayashi S. Synthesis and Acid-Responsive Electron-Transfer Disproportionation of Non- and Tetramesityl-Substituted 1,1’,9,9’-Bicarbazole. Chem. Lett. 2015, 44, 1336–1338. 10.1246/cl.150557.

[ref513] Higashibayashi S.; Pandit P.; Haruki R.; Adachi S.; Kumai R. Redox-Dependent Transformation of a Hydrazinobuckybowl between Curved and Planar Geometries. Angew. Chem., Int. Ed. 2016, 55, 10830–10834. 10.1002/anie.201605340. 27491494

[ref514] Petrova O. N.; Lipson V. V.; Zamigailo L. L.; Shirobokova M. G.; Musatov V. I.; Baumer V. N.; Sofronov D. S. Synthesis and Chemical Properties of 4-Aroyl-3-Methyl-4,10-Dihydroindeno[1,2-b]Pyrazolo-[4,3-e]Pyridin-5-Ones. Russ. J. Org. Chem. 2015, 51, 1597–1605. 10.1134/S1070428015110147.

[ref515] Mule R. D.; Shaikh A. C.; Gade A. B.; Patil N. T. A New Class of N-Doped Ionic PAHs via Intramolecular [4 + 2]-Cycloaddition between Arylpyridines and Alkynes. Chem. Commun. 2018, 54, 11909–11912. 10.1039/C8CC05743E. 30283940

[ref516] Shaikh A. C.; Banerjee S.; Mule R. D.; Bera S.; Patil N. T. External Oxidant-Dependent Reactivity Switch in Copper-Mediated Intramolecular Carboamination of Alkynes: Access to a Different Class of Fluorescent Ionic Nitrogen-Doped Polycyclic Aromatic Hydrocarbons. J. Org. Chem. 2019, 84, 4120–4130. 10.1021/acs.joc.9b00120. 30813732

[ref517] Zhu D.; Wu Z.; Liang L.; Sun Y.; Luo B.; Huang P.; Wen S. Heterocyclic Iodoniums as Versatile Synthons to Approach Diversified Polycyclic Heteroarenes. RSC Adv. 2019, 9, 33170–33179. 10.1039/C9RA07288H. PMC9073335 35529157

[ref518] Liao W.-L.; Li S.-Q.; Wang J.; Zhang Z.-Y.; Yang Z.-W.; Xu D.; Xu C.; Lan H.-T.; Chen Z.-Z.; Xu Z.-G. An Efficient and Facile Method for the Synthesis of Benzimidazoisoquinoline Derivatives via a Multicomponent Reaction. ACS Comb. Sci. 2016, 18, 65–69. 10.1021/acscombsci.5b00145. 26634875

[ref519] Chen Z.-Z.; Li S.-Q.; Zhang Y.-J.; Tang D.-Y.; Meng J.-P.; Lei J.; Li H.-Y.; Xu Z.-G. Synthesis of Pyridodiindoles with Anticancer Activity by a Three-Component Cascade Condensation. Org. Lett. 2018, 20, 7811–7815. 10.1021/acs.orglett.8b03245. 30512962

[ref520] Zou S.; Liu Y.; Xi C. Concise and Efficient Synthesis of Indole-Indolone Scaffolds through MeOTf-Induced Annulation of N-(2-Cyanoaryl)Indoles. ACS Omega 2019, 4, 18734–18740. 10.1021/acsomega.9b02679. 31737835 PMC6854827

[ref521] Volvoikar P. S.; Tilve S. G.; Zubkov F. I. A Concise Approach for the Synthesis of the ABCD Ring System of Alpkinidine. ChemistrySelect 2019, 4, 7187–7189. 10.1002/slct.201900357.

[ref522] Benke B. P.; Hertwig L.; Yang X.; Rominger F.; Mastalerz M. Triptycene End-Capped Indigo Derivatives - Turning Insoluble Pigments to Soluble Dyes. Eur. J. Org. Chem. 2021, 2021, 72–76. 10.1002/ejoc.202001362. PMC7821156 33510580

[ref523] Zissimou G. A.; Kourtellaris A.; Manoli M.; Koutentis P. A. Redox Active Quinoidal 1,2,4-Benzotriazines. J. Org. Chem. 2018, 83, 9391–9402. 10.1021/acs.joc.8b01311. 29940730

[ref524] Gu P.-Y.; Liu G.; Zhao J.; Aratani N.; Ye X.; Liu Y.; Yamada H.; Nie L.; Zhang H.; Zhu J.; et al. Understanding the Structure-Determining Solid Fluorescence of an Azaacene Derivative. J. Mater. Chem. C 2017, 5, 8869–8874. 10.1039/C7TC03089D.

[ref525] Kitamura K.; Asahina K.; Adachi K.; Hamura T. Intramolecular Benzoallene-Alkyne Cycloaddition Initiated by Site-Selective SN2’ Reaction of Epoxytetracene En Route to π-Extended Pyracylene. Chem. Commun. 2019, 55, 11021–11024. 10.1039/C9CC05500B. 31478039

[ref526] Hu P.; He X.; Ng M.-F.; Ye J.; Zhao C.; Wang S.; Tan K.; Chaturvedi A.; Jiang H.; Kloc C.; et al. Trisulfide-Bond Acenes for Organic Batteries. Angew. Chem., Int. Ed. 2019, 58, 13513–13521. 10.1002/anie.201906301. 31317598

[ref527] Delaunay W.; Szucs R.; Pascal S.; Mocanu A.; Bouit P.-A.; Nyulászi L.; Hissler M. Synthesis and Electronic Properties of Polycyclic Aromatic Hydrocarbons Doped with Phosphorus and Sulfur. Dalton Trans. 2016, 45, 1896–1903. 10.1039/C5DT04154F. 26646633

[ref528] Ooyama Y.; Enoki T.; Aoyama S.; Ohshita J. Synthesis and Optical and Electrochemical Properties of a Phenanthrodithiophene (Fused-Bibenzo[c]Thiophene) Derivative. Org. Biomol. Chem. 2017, 15, 7302–7307. 10.1039/C7OB01695F. 28819667

[ref529] Liu S.; Huang C.; Zhang J.; Tian S.; Li C.; Fu N.; Wang L.; Zhao B.; Huang W. Synthesis of Sulfur-Hybridized Pyracylene and the Unexpected Phenyl Shift Mediated Rearrangement of Scholl Reaction. Eur. J. Org. Chem. 2019, 2019, 3061–3070. 10.1002/ejoc.201900344.

[ref530] Wu X.; Yang Y.; Han J.; Wang L. Palladium Catalyzed C-I and Vicinal C-H Dual Activation of Diaryliodonium Salts for Diarylation: Synthesis of 4,5-Benzocoumarins. Org. Lett. 2015, 17, 5654–5657. 10.1021/acs.orglett.5b02938. 26523622

[ref531] Patil V. V.; Lee K. H.; Lee J. Y. Dibenzo[c, g]Indolo[3,2,1-Jk]Carbazole as a New Chromophore for Blue Organic Light-Emitting Diodes. J. Mater. Chem. C 2019, 7, 14301–14305. 10.1039/C9TC04783B.

[ref532] Lee H. L.; Chung W. J.; Lee J. Y. Narrowband and Pure Violet Organic Emitter with a Full Width at Half Maximum of 14 Nm and y Color Coordinate of Below 0.02. Small 2020, 16, 1907569 10.1002/smll.201907569. 32162765

[ref533] Venunath Patil V.; Hyung Lee K.; Yeob Lee J. A Novel Fluorene-Indolocarbazole Hybrid Chromophore to Assemble High Efficiency Deep-Blue Fluorescent Emitters with Extended Device Lifetime. J. Mater. Chem. C 2020, 8, 3051–3057. 10.1039/C9TC06434F.

[ref534] Kader T.; Stöger B.; Fröhlich J.; Kautny P. Azaindolo[3,2,1-Jk]Carbazoles: New Building Blocks for Functional Organic Materials. Chem. - Eur. J. 2019, 25, 4412–4425. 10.1002/chem.201805578. 30620787 PMC6491992

[ref535] Do H. H.; Tran H. Q.; Ohlendorf L.; Ngo T. N.; Dang T. T.; Ehlers P.; Villinger A.; Langer P. One-Pot Palladium-Catalyzed Synthesis of Benzo[b]Carbazolediones. Synlett 2015, 26, 2429–2433. 10.1055/s-0035-1560212.

[ref536] Deng N.; Zhang G. Nitrogen-Centered Concave Molecules with Double Fused Pentagons. Org. Lett. 2019, 21, 5248–5251. 10.1021/acs.orglett.9b01861. 31247791

[ref537] Zhou L.; Zhang G. A Nanoboat with Fused Concave N-Heterotriangulene. Angew. Chem., Int. Ed. 2020, 59, 8963–8968. 10.1002/anie.202002869. 32150655

[ref538] Song Y.; Zhang G. Effect of Fusion Manner of Concave Molecules on the Properties of Resulting Nanoboats. Org. Lett. 2021, 23, 491–496. 10.1021/acs.orglett.0c04008. 33403857

[ref539] Jones A. W.; Louillat-Habermeyer M.-L.; Patureau F. W. Strained Dehydrogenative Ring Closure of Phenylcarbazoles. Adv. Synth. Catal. 2015, 357, 945–949. 10.1002/adsc.201401136.

[ref540] Rank C. K.; Jones A. W.; Wall T.; Martino-Fumo P. D.; Schröck S.; Gerhards M.; Patureau F. W. An Intermolecular C-H Oxidizing Strategy to Access Highly Fused Carbazole Skeletons from Simple Naphthylamines. Chem. Commun. 2019, 55, 13749–13752. 10.1039/C9CC05240B. 31663087

[ref541] Taniguchi T.; Itai Y.; Nishii Y.; Tohnai N.; Miura M. Construction of Nitrogen-Containing Polycyclic Aromatic Compounds by Intramolecular Oxidative C-H/C-H Coupling of Bis(9H-Carbazol-9-Yl)Benzenes and Their Properties. Chem. Lett. 2019, 48, 1160–1163. 10.1246/cl.190494.

[ref542] Puntscher H.; Kautny P.; Stöger B.; Tissot A.; Hametner C.; Hagemann H. R.; Fröhlich J.; Baumgartner T.; Lumpi D. Structure-Property Studies of P-Triarylamine-Substituted Dithieno[3,2-b:2’,3′-d]Phospholes. RSC Adv. 2015, 5, 93797–93807. 10.1039/C5RA13651B.

[ref543] Kautny P.; Wu Z.; Eichelter J.; Horkel E.; Stöger B.; Chen J.; Ma D.; Fröhlich J.; Lumpi D. Indolo[3,2,1-Jk]Carbazole Based Planarized CBP Derivatives as Host Materials for PhOLEDs with Low Efficiency Roll-Off. Org. Electron. 2016, 34, 237–245. 10.1016/j.orgel.2016.04.036.

[ref544] Seo J.-A.; Im Y.; Han S. H.; Lee C. W.; Lee J. Y. Unconventional Molecular Design Approach of High-Efficiency Deep Blue Thermally Activated Delayed Fluorescent Emitters Using Indolocarbazole as an Acceptor. ACS Appl. Mater. Interfaces 2017, 9, 37864–37872. 10.1021/acsami.7b09351. 28980471

[ref545] Patil V. V.; Lee K. H.; Lee J. Y. Universal Blue Emitters for High Efficiency Thermally Activated Delayed Fluorescence and Fluorescent Organic Light-Emitting Diodes. Dyes Pigm. 2020, 174, 108070 10.1016/j.dyepig.2019.108070.

[ref546] Im Y.; Han S. H.; Lee J. Y. CN Substituted Indolocarbazole as a Core Structure of Exciton Harvesting and Lifetime Extending Host for Green Thermally Activated Delayed Fluorescent Emitter. Dyes Pigm. 2019, 164, 233–236. 10.1016/j.dyepig.2019.01.024.

[ref547] Im Y.; Han S. H.; Lee J. Y. Deep Blue Thermally Activated Delayed Fluorescent Emitters Using CN-Modified Indolocarbazole as an Acceptor and Carbazole-Derived Donors. J. Mater. Chem. C 2018, 6, 5012–5017. 10.1039/C8TC00546J.

[ref548] Konidena R. K.; Lee K. H.; Lee J. Y.; Hong W. P. 15H-Diindolo[2,3-b:1’,2’,3′-Lm]Carbazole: A Novel Rigid Donor for Highly Efficient Thermally Activated Delayed Fluorescence Emitters. J. Mater. Chem. C 2019, 7, 8037–8044. 10.1039/C9TC02195G.

[ref549] Im Y.; Han S. H.; Lee J. Y. Dibenzothiophene and Indolocarbazole Cored Bipolar Hosts for Blue Phosphorescent Organic Light-Emitting Diodes. Org. Electron. 2018, 62, 560–565. 10.1016/j.orgel.2018.06.031.

[ref550] Ma X.-J.; Zhu X.-D.; Wang K.-L.; Igbari F.; Yuan Y.; Zhang Y.; Gao C.-H.; Jiang Z.-Q.; Wang Z.-K.; Liao L.-S. Planar Starburst Hole-Transporting Materials for Highly Efficient Perovskite Solar Cells. Nano Energy 2019, 63, 103865 10.1016/j.nanoen.2019.103865.

[ref551] Holzer B.; Bintinger J.; Lumpi D.; Choi C.; Kim Y.; Stöger B.; Hametner C.; Marchetti-Deschmann M.; Plasser F.; Horkel E.; et al. Color Fine-Tuning of Optical Materials Through Rational Design. ChemPhysChem 2017, 18, 549–563. 10.1002/cphc.201601204. 27959475

[ref552] Im Y.; Han S. H.; Lee J. Y. Bipolar Type Indolocarbazole Host for Green Phosphorescent Organic Light-Emitting Diodes. J. Ind. Eng. Chem. 2018, 66, 381–386. 10.1016/j.jiec.2018.06.004.

[ref553] Lévesque É.; Bechara W. S.; Constantineau-Forget L.; Pelletier G.; Rachel N. M.; Pelletier J. N.; Charette A. B. General C-H Arylation Strategy for the Synthesis of Tunable Visible Light-Emitting Benzo[a]Imidazo[2,1,5-c, d]Indolizine Fluorophores. J. Org. Chem. 2017, 82, 5046–5067. 10.1021/acs.joc.6b02928. 28441020

[ref554] Wu D.; Chen L.; Ma S.; Luo H.; Cao J.; Chen R.; Duan Z.; Mathey F. Synthesis of 1,3-Azaphospholes with Pyrrolo[1,2-a]Quinoline Skeleton and Their Optical Applications. Org. Lett. 2018, 20, 4103–4106. 10.1021/acs.orglett.8b01663. 29931983

[ref555] Zheng W.; Zhao Y.; Zhuang W.-H.; Wu J.-J.; Wang F.-Z.; Li C.-H.; Zuo J.-L. Phthalorubines: Fused-Ring Compounds Synthesized from Phthalonitrile. Angew. Chem., Int. Ed. 2018, 57, 15384–15389. 10.1002/anie.201807281. 30251399

[ref556] Fu W. C.; Wang Z.; Chan W. T. K.; Lin Z.; Kwong F. Y. Regioselective Synthesis of Polycyclic and Heptagon-Embedded Aromatic Compounds through a Versatile π-Extension of Aryl Halides. Angew. Chem., Int. Ed. 2017, 56, 7166–7170. 10.1002/anie.201703551. 28510348

[ref557] Samineni R.; Srihari P.; Mehta G. Versatile Route to Benzoannulated Medium-Ring Carbocycles via Aryne Insertion into Cyclic 1,3-Diketones: Application to a Synthesis of Radermachol. Org. Lett. 2016, 18, 2832–2835. 10.1021/acs.orglett.6b01078. 27268522

[ref558] Li X.; Pan J.; Song S.; Jiao N. Pd-Catalyzed Dehydrogenative Annulation Approach for the Efficient Synthesis of Phenanthridinones. Chem. Sci. 2016, 7, 5384–5389. 10.1039/C6SC01148A. 30155191 PMC6020758

[ref559] Li H.; Roisnel T.; Soulé J.-F.; Doucet H. Convenient Access to C10- and C11-(Di)Arylated Dibenzo[b, f]Azepines via Palladium-Catalyzed C-H Bonds Cleavages. Adv. Synth. Catal. 2019, 361, 791–802. 10.1002/adsc.201801366.

[ref560] Ito M.; Kawasaki R.; Kanyiva K. S.; Shibata T. Construction of a Polycyclic Conjugated System Containing a Dibenzazepine Moiety by Cationic Gold(I)-Catalyzed Cycloisomerization. Eur. J. Org. Chem. 2016, 2016, 5234–5237. 10.1002/ejoc.201601147.

[ref561] Skonieczny K.; Gryko D. T. Photochemical Conversion of Phenanthro[9,10-d]Imidazoles into π-Expanded Heterocycles. J. Org. Chem. 2015, 80, 5753–5763. 10.1021/acs.joc.5b00714. 25938658

[ref562] Hayakawa S.; Kawasaki A.; Hong Y.; Uraguchi D.; Ooi T.; Kim D.; Akutagawa T.; Fukui N.; Shinokubo H. Inserting Nitrogen: An Effective Concept To Create Nonplanar and Stimuli-Responsive Perylene Bisimide Analogues. J. Am. Chem. Soc. 2019, 141, 19807–19816. 10.1021/jacs.9b09556. 31746597

[ref563] Gadekar S. C.; Reddy B. K.; Panchal S. P.; Anand V. G. Metal Assisted Cyclomerization of Benzodipyrrins into Expanded Norroles, Aza-Heptalene and Acyclic Dimers. Chem. Commun. 2016, 52, 4565–4568. 10.1039/C5CC10356H. 26939925

[ref564] Petrovskii P. P.; Tomashenko O. A.; Novikov M. S.; Khlebnikov A. F.; Stoeckli-Evans H. Synthesis, Crystal Structure, and Photophysical Properties of Dimethyl 7-Oxa-2a1-Azabenzo[b]Cyclopenta[Pq]Pleiadene-1,2-Dicarboxylate - Novel Fused Oxazapolycyclic Skeleton. Chem. Heterocycl. Compd. 2017, 53, 909–912. 10.1007/s10593-017-2144-3.

[ref565] Hayakawa S.; Matsuo K.; Yamada H.; Fukui N.; Shinokubo H. Dinaphthothiepine Bisimide and Its Sulfoxide: Soluble Precursors for Perylene Bisimide. J. Am. Chem. Soc. 2020, 142, 11663–11668. 10.1021/jacs.0c04096. 32543842

[ref566] Kirschbaum T.; Rominger F.; Mastalerz M. An Isosteric Triaza Analogue of a Polycyclic Aromatic Hydrocarbon Monkey Saddle. Chem. - Eur. J. 2020, 26, 14560–14564. 10.1002/chem.202002826. 32539193 PMC7756504

[ref567] Hassan A. Y.; Sarg M. T.; El Deeb M. A.; Bayoumi A. H.; El Rabeb S. I. Facile Synthesis and Anticancer Activity Study of Novel Series of Substituted and Fused Coumarin Derivatives. J. Heterocycl. Chem. 2018, 55, 1426–1443. 10.1002/jhet.3179.

[ref568] Igarashi T.; Tobisu M.; Chatani N. Catalytic Double Carbon-Boron Bond Formation for the Synthesis of Cyclic Diarylborinic Acids as Versatile Building Blocks for π-Extended Heteroarenes. Angew. Chem., Int. Ed. 2017, 56, 2069–2073. 10.1002/anie.201612535. 28111913

[ref569] Mehra M. K.; Sharma S.; Rangan K.; Kumar D. Substrate or Solvent-Controlled PdII-Catalyzed Regioselective Arylation of Quinolin-4(1H)-Ones Using Diaryliodonium Salts: Facile Access to Benzoxocine and Aaptamine Analogues. Eur. J. Org. Chem. 2020, 2020, 2409–2413. 10.1002/ejoc.202000013.

[ref570] Tanaka Y.; Tajima K.; Fukui N.; Shinokubo H. Dinaphtho[1,8-Bc:1’,8’-Fg][1,5]Dithiocine Bisimide. Asian J. Org. Chem. 2021, 10, 541–544. 10.1002/ajoc.202000722.

[ref571] Bromby A. D.; Hogan D. T.; Sutherland T. C. Core Expanded, 21,23-Dithiadiacenaphtho[1,2-c]Porphyrin Interactions with [60]Fullerene. New J. Chem. 2017, 41, 4802–4805. 10.1039/C6NJ03353A.

[ref572] Rauen P. J.; Lash T. D. Dihydropyracyloporphyrins. Tetrahedron Lett. 2020, 61, 152662 10.1016/j.tetlet.2020.152662.

[ref573] Okujima T.; Mack J.; Nakamura J.; Kubheka G.; Nyokong T.; Zhu H.; Komobuchi N.; Ono N.; Yamada H.; Uno H.; et al. Synthesis, Characterization, and Electronic Structures of Porphyrins Fused with Polycyclic Aromatic Ring Systems. Chem. - Eur. J. 2016, 22, 14730–14738. 10.1002/chem.201602213. 27558988

[ref574] Gao R.; AbuSalim D. I.; Lash T. D. Pyreniporphyrins: Porphyrin Analogues That Incorporate a Polycyclic Aromatic Hydrocarbon Subunit within the Macrocyclic Framework. J. Org. Chem. 2017, 82, 6680–6688. 10.1021/acs.joc.7b00829. 28574261

[ref575] Galstyan A.; Maurya Y. K.; Zhylitskaya H.; Bae Y. J.; Wu Y.-L.; Wasielewski M. R.; Lis T.; Dobrindt U.; Stępień M. π-Extended Donor-Acceptor Porphyrins and Metalloporphyrins for Antimicrobial Photodynamic Inactivation. Chem. - Eur. J. 2020, 26, 8262–8266. 10.1002/chem.201905372. 31968144 PMC7384002

[ref576] Janiga E.; Kim G.; Chmielewski P. J.; Lis T.; Kim D.; Stępień M. Porphyrin-Ryleneimide Hybrids: Low-Bandgap Acceptors in Energy-Transfer Cassettes. Chem. - Asian J. 2020, 15, 2854–2858. 10.1002/asia.202000762. 32667127

[ref577] Kumar S.; Maurya Y. K.; Kang S.; Chmielewski P.; Lis T.; Cybińska J.; Kim D.; Stępień M. Porphyrin-Ryleneimide Hybrids: Tuning of Visible and Near-Infrared Absorption by Chromophore Desymmetrization. Org. Lett. 2020, 22, 7202–7207. 10.1021/acs.orglett.0c02544. 32857521 PMC7506948

[ref578] Feng J.; Fu L.; Huang T.; Geng H.; Jiang W.; Wang Z. Rylene Annulated Phthalocyanine: A Fully Conjugated Block for the Construction of a Supramolecular Two-Dimensional Framework. Chem. Commun. 2018, 54, 7294–7297. 10.1039/C8CC01216D. 29717308

[ref579] Zhao Y.; Jiang K.; Li C.; Liu Y.; Xu C.; Zheng W.; Guan D.; Li Y.; Zheng H.; Liu C.; et al. Precise Control of π-Electron Magnetism in Metal-Free Porphyrins. J. Am. Chem. Soc. 2020, 142, 18532–18540. 10.1021/jacs.0c07791. 32959653

[ref580] Sun Q.; Mateo L. M.; Robles R.; Ruffieux P.; Lorente N.; Bottari G.; Torres T.; Fasel R. Inducing Open-Shell Character in Porphyrins through Surface-Assisted Phenalenyl π-Extension. J. Am. Chem. Soc. 2020, 142, 18109–18117. 10.1021/jacs.0c07781. 32985889

[ref581] Xiang F.; Gemeinhardt A.; Schneider M. A. Competition between Dehydrogenative Organometallic Bonding and Covalent Coupling of an Unfunctionalized Porphyrin on Cu(111). ACS Nano 2018, 12, 1203–1210. 10.1021/acsnano.7b06998. 29336554

[ref582] Shu C.-H.; Xie Y.-L.; Wang A.; Shi K.-J.; Zhang W.-F.; Li D.-Y.; Liu P.-N. On-Surface Reactions of Aryl Chloride and Porphyrin Macrocycles via Merging Two Reactive Sites into a Single Precursor. Chem. Commun. 2018, 54, 12626–12629. 10.1039/C8CC07652A. 30351327

[ref583] Seufert K.; McBride F.; Jaekel S.; Wit B.; Haq S.; Steiner A.; Poli P.; Persson M.; Raval R.; Grill L. Porphine Homocoupling on Au(111). J. Phys. Chem. C 2019, 123, 16690–16698. 10.1021/acs.jpcc.9b02770.

[ref584] Xiang F.; Lu Y.; Wang Z.; Ju H.; Di Filippo G.; Li C.; Liu X.; Leng X.; Zhu J.; Wang L.; et al. On-Surface Synthesis of Chiral π-Conjugate Porphyrin Tapes by Substrate-Regulated Dehydrogenative Coupling. J. Phys. Chem. C 2019, 123, 23007–23013. 10.1021/acs.jpcc.9b06025.

[ref585] Yin C.; Peng Z.; Liu D.; Song H.; Zhu H.; Chen Q.; Wu K. Selective Intramolecular Dehydrocyclization of Co-Porphyrin on Au(111). Molecules 2020, 25, 3766 10.3390/molecules25173766. PMC7503656 32824933

[ref586] Murugavel M.; Adinarayana B.; Das M.; Peruncheralathan S.; Palepu N. R.; Srinivasan A. PtCl2Mediated Peripheral Transformation of Carbatriphyrin(3.1.1) into a Meso-Fused β-B’ Dimer and Its Monomer Analogue. Chem. Commun. 2020, 56, 12809–12812. 10.1039/D0CC05309K. 32966387

[ref587] Lungerich D.; Hitzenberger J. F.; Ruppel M.; Döpper T.; Witt M.; Ivanović-Burmazović I.; Görling A.; Jux N.; Drewello T. Gas-Phase Transformation of Fluorinated Benzoporphyrins to Porphyrin-Embedded Conical Nanocarbons. Chem. - Eur. J. 2020, 26, 12180–12187. 10.1002/chem.202002638. 32578918 PMC7540561

[ref588] Fujita H.; Jing H.; Krayer M.; Allu S.; Veeraraghavaiah G.; Wu Z.; Jiang J.; Diers J. R.; Magdaong N. C. M.; Mandal A. K.; et al. Annulated Bacteriochlorins for Near-Infrared Photophysical Studies. New J. Chem. 2019, 43, 7209–7232. 10.1039/C9NJ01113G.

[ref589] Hooper R. W.; Zhang A.; Koszelewski D.; Lewtak J. P.; Koszarna B.; Levy C. J.; Gryko D. T.; Stillman M. J. Differential Quenching of the Angular Momentum of the B and Q Bands of a Porphyrin as a Result of Extended Ring π-Conjugation. J. Porphyrins Phthalocyanines 2018, 22, 1111–1128. 10.1142/S1088424618501110.

[ref590] Lewtak J. P.; Koszarna B.; Charyton M. K.; Gryko D. T. Extending a Porphyrin Chromophore via Fusion with Naphthalene. J. Porphyrins Phthalocyanines 2020, 24, 448–455. 10.1142/S1088424619501530.

[ref591] Achary B. S.; Gokulnath S.; Ghosh S.; Mrinalini M.; Prasanthkumar S.; Giribabu L. Unprecedented Charge-Transfer Complex of Fused Diporphyrin as Near-Infrared Absorption-Induced High-Aspect-Ratio Nanorods. Chem. - Asian J. 2016, 11, 3498–3502. 10.1002/asia.201601363. 27781413

[ref592] Osterloh W. R.; Kumar S.; Chaudhri N.; Fang Y.; Sankar M.; Kadish K. M. Facile Heterogeneous and Homogeneous Anion Induced Electrosynthesis: An Efficient Method for Obtaining π-Extended Porphyrins. Inorg. Chem. 2020, 59, 16737–16746. 10.1021/acs.inorgchem.0c02770. 33143408

[ref593] Hurej K.; Stawski W.; Latos-Grażyński L.; Pawlicki M. Meso-N-Pyrrole as a Versatile Substituent Influencing the Optical Properties of Porphyrin. Chem. - Asian J. 2016, 11, 3329–3333. 10.1002/asia.201601210. 27727507

[ref594] Fukui N.; Cha W.; Shimizu D.; Oh J.; Furukawa K.; Yorimitsu H.; Kim D.; Osuka A. Highly Planar Diarylamine-Fused Porphyrins and Their Remarkably Stable Radical Cations. Chem. Sci. 2017, 8, 189–199. 10.1039/C6SC02721K. 28451165 PMC5308406

[ref595] Kise K.; Hong Y.; Fukui N.; Shimizu D.; Kim D.; Osuka A. Diarylamine-Fused Subporphyrins: Proof of Twisted Intramolecular Charge Transfer (TICT) Mechanism. Chem. - Eur. J. 2018, 24, 8306–8310. 10.1002/chem.201801576. 29667245

[ref596] Fukui N.; Yorimitsu H.; Osuka A. Meso-Meso-Linked Diarylamine-Fused Porphyrin Dimers. Chem. - Eur. J. 2016, 22, 18476–18483. 10.1002/chem.201604301. 27859737

[ref597] Kato K.; Osuka A. Meta- and Para-Phenylenediamine-Fused Porphyrin Dimers: Synthesis and Magnetic Interactions of Their Dication Diradicals. Angew. Chem., Int. Ed. 2019, 58, 8546–8550. 10.1002/anie.201901939. 30980458

[ref598] Kato K.; Osuka A. Helically Twisted Benzene-1,3,5-Triamine-Fused Porphyrin Dimers. Chem. Lett. 2020, 49, 517–520. 10.1246/cl.200115.

[ref599] Fujimoto K.; Osuka A. A 1,5-Naphthyridine-Fused Porphyrin Dimer: Intense NIR Absorption and Facile Redox Interconversion with Its Reduced Congener. Chem. - Eur. J. 2018, 24, 6530–6533. 10.1002/chem.201800854. 29536577

[ref600] Thuita D.; Guberman-Pfeffer M. J.; Brückner C. SNAr Reaction Toward the Synthesis of Fluorinated Quinolino[2,3,4-at]Porphyrins. Eur. J. Org. Chem. 2021, 2021, 318–323. 10.1002/ejoc.202001347.

[ref601] Batalha P. N.; Gomes A. T. P. C.; Forezi L. S. M.; Costa L.; de Souza M. C. B. V.; Boechat F. d. C. S.; Ferreira V. F.; Almeida A.; Faustino M. A. F.; Neves M. G. P. M. S.; Cavaleiro J. A. S.; et al. Synthesis of New Porphyrin/4-Quinolone Conjugates and Evaluation of Their Efficiency in the Photoinactivation of Staphylococcus Aureus. RSC Adv. 2015, 5, 71228–71239. 10.1039/C5RA11070J.

[ref602] Ahmad Dar T.; Mandeep Sankar M. Synthesis, Spectral and Electrochemical Redox Properties of N-Methyl Fused Nickel(II) Porphyrin. J. Porphyrins Phthalocyanines 2018, 22, 1106–1110. 10.1142/S1088424618501109.

[ref603] Chevrier M.; Richeter S.; Coulembier O.; Surin M.; Mehdi A.; Lazzaroni R.; Evans R. C.; Dubois P.; Clément S. Expanding the Light Absorption of Poly(3-Hexylthiophene) by End-Functionalization with π-Extended Porphyrins. Chem. Commun. 2016, 52, 171–174. 10.1039/C5CC06290J. 26506849

[ref604] Pereira A. M. V. M.; Lacerda P. S. S.; Silva B. N. M.; Neves M. G. P. M. S.; Silva A. M. S.; Silva B. V.; da Silva F. C.; Ferreira V. F.; Pinto A. C.; Cavaleiro J. A. S. One-Pot Synthesis of New Isatin-Porphyrin Conjugates by the Palladium Buchwald-Hartwig Methodology Involving β-Aminoporphyrinatonickel(II) and 3-Ketal Isatin Derivatives. Dyes Pigm. 2017, 139, 247–254. 10.1016/j.dyepig.2016.12.010.

[ref605] Jimenez A. J.; Mesa N. S.; Pereira A. M. V. M.; Jean M.; Vincent B.; Jeandon C.; Gisselbrecht J.-P.; Ruppert R. Oxidative Coupling of an Enaminoporphyrin: C-C, N-N Linkages or Both?. Asian J. Org. Chem. 2015, 4, 1294–1300. 10.1002/ajoc.201500279.

[ref606] Pawlicki M.; Hurej K.; Kwiecińska K.; Szterenberg L.; Latos-Grażyński L. A Fused Meso-Aminoporphyrin: A Switchable near-IR Chromophore. Chem. Commun. 2015, 51, 11362–11365. 10.1039/C5CC01231G. 25777739

[ref607] Abuteen A.; Zanganeh S.; Akhigbe J.; Samankumara L. P.; Aguirre A.; Biswal N.; Braune M.; Vollertsen A.; Röder B.; Brückner C.; et al. The Evaluation of NIR-Absorbing Porphyrin Derivatives as Contrast Agents in Photoacoustic Imaging. Phys. Chem. Chem. Phys. 2013, 15, 18502–18509. 10.1039/c3cp52193a. 24071709 PMC5056905

[ref608] Luciano M.; Erfanzadeh M.; Zhou F.; Zhu H.; Bornhütter T.; Röder B.; Zhu Q.; Brückner C. In Vivo Photoacoustic Tumor Tomography Using a Quinoline-Annulated Porphyrin as NIR Molecular Contrast Agent. Org. Biomol. Chem. 2017, 15, 972–983. 10.1039/C6OB02640K. 28059409 PMC5302001

[ref609] Akhigbe J.; Yang M.; Luciano M.; Brückner C. Quinoline-Annulated Chlorins and Chlorin-Analogs. J. Porphyrins Phthalocyanines 2016, 20, 265–273. 10.1142/S1088424616500036.

[ref610] Berthelot M.; Hoffmann G.; Bousfiha A.; Echaubard J.; Roger J.; Cattey H.; Romieu A.; Lucas D.; Fleurat-Lessard P.; Devillers C. H. Oxidative C-N Fusion of Pyridinyl-Substituted Porphyrins. Chem. Commun. 2018, 54, 5414–5417. 10.1039/C8CC01375F. 29726882

[ref611] Li C.; Zhu L.; Liang W.; Su R.; Yin J.; Hu Y.; Lan Y.; Wu D.; You J. An Unusual [4 + 2] Fusion Strategy to Forge *Meso-N*/*O* -Heteroarene-Fused (Quinoidal) Porphyrins with Intense near-Infrared Q-Bands. Chem. Sci. 2019, 10, 7274–7280. 10.1039/C9SC01596E. 31588297 PMC6686728

[ref612] Ooi S.; Tanaka T.; Park K. H.; Kim D.; Osuka A. Triply Linked Corrole Dimers. Angew. Chem., Int. Ed. 2016, 55, 6535–6539. 10.1002/anie.201601864. 27037528

[ref613] Okuda Y.; Fukui N.; Kim J.; Kim T.; Jiang H.-W.; Copley G.; Kitano M.; Kim D.; Osuka A. A Meso-Meso β-β β-β Triply Linked Subporphyrin Dimer. Angew. Chem., Int. Ed. 2017, 56, 12317–12321. 10.1002/anie.201707123. 28744951

[ref614] Kato K.; Furukawa K.; Osuka A. A Stable Trimethylenemethane Triplet Diradical Based on a Trimeric Porphyrin Fused π-System. Angew. Chem., Int. Ed. 2018, 57, 9491–9494. 10.1002/anie.201804644. 29858542

[ref615] Zhang H.; Kim J.; Phan H.; Herng T. S.; Gopalakrishna T. Y.; Zeng W.; Ding J.; Kim D.; Wu J. 2,6-/1,5-Naphthoquinodimethane Bridged Porphyrin Dimer Diradicaloids. J. Porphyrins Phthalocyanines 2020, 24, 220–229. 10.1142/S1088424619500998.

[ref616] Wang K.; Liu P.; Zhang F.; Xu L.; Zhou M.; Nakai A.; Kato K.; Furukawa K.; Tanaka T.; Osuka A.; et al. A Robust Porphyrin-Stabilized Triplet Carbon Diradical. Angew. Chem., Int. Ed. 2021, 60, 7002–7006. 10.1002/anie.202015356. 33393192

[ref617] Kozyrev A. N.; Alderfer J. L.; Srikrishnan T.; Pandey R. K. Synthesis of Novel Laterally-Bridged Pyropheophorbide a Dimers. J. Chem. Soc., Perkin Trans. 1 1998, 837–838. 10.1039/a800609a.

[ref618] Kozyrev A. N.; Suresh V.; Das S.; Senge M. O.; Shibata M.; Dougherty T. J.; Pandey R. K. Syntheses and Spectroscopic Studies of Novel Chlorins with Fused Quinoxaline or Benzimidazole Ring Systems and the Related Dimers with Extended Conjugation1. Tetrahedron 2000, 56, 3353–3364. 10.1016/S0040-4020(00)00256-8.

[ref619] Kozyrev A.; Ethirajan M.; Chen P.; Ohkubo K.; Robinson B. C.; Barkigia K. M.; Fukuzumi S.; Kadish K. M.; Pandey R. K. Synthesis, Photophysical and Electrochemistry of Near-IR Absorbing Bacteriochlorins Related to Bacteriochlorophyll *a*. J. Org. Chem. 2012, 77, 10260–10271. 10.1021/jo301895p. 23082726 PMC3526342

[ref620] Marko A. J.; Dukh M.; Patel N. J.; Missert J. R.; Ohulchanskyy T.; Tabaczynski W. A.; Ohkubo K.; Fukuzumi S.; Yao R.; Sajjad M.; et al. A Pyropheophorbide Analogue Containing a Fused Methoxy Cyclohexenone Ring System Shows Promising Cancer-Imaging Ability. ChemMedChem 2019, 14, 1503–1513. 10.1002/cmdc.201900352. 31343840

[ref621] Dekkiche H.; Buisson A.; Langlois A.; Karsenti P.-L.; Ruhlmann L.; Ruppert R.; Harvey P. Metal Linkage Effects on Ultrafast Energy Transfer. Chem. - Eur. J. 2016, 22, 10484–10493. 10.1002/chem.201601322. 27333487

[ref622] Dekkiche H.; Buisson A.; Langlois A.; Karsenti P.-L.; Ruhlmann L.; Harvey P. D.; Ruppert R. Ultrafast Singlet Energy Transfer in Porphyrin Dyads. Inorg. Chem. 2016, 55, 10329–10336. 10.1021/acs.inorgchem.6b01594. 27709919

[ref623] Carvalho M.-A.; Dekkiche H.; Karmazin L.; Sanchez F.; Vincent B.; Kanesato M.; Kikkawa Y.; Ruppert R. Synthesis and Study at a Solid/Liquid Interface of Porphyrin Dimers Linked by Metal Ions. Inorg. Chem. 2017, 56, 15081–15090. 10.1021/acs.inorgchem.7b02422. 29193969

[ref624] Carvalho M.-A.; Dekkiche H.; Nagasaki M.; Kikkawa Y.; Ruppert R. Coordination-Driven Construction of Porphyrin Nanoribbons at a Highly Oriented Pyrolytic Graphite (HOPG)/Liquid Interface. J. Am. Chem. Soc. 2019, 141, 10137–10141. 10.1021/jacs.9b02145. 31184484

[ref625] Chan J. Y. M.; Kawata T.; Kobayashi N.; Ng D. K. P. Boron(III) Carbazosubphthalocyanines: Core-Expanded Antiaromatic Boron(III) Subphthalocyanine Analogues. Angew. Chem., Int. Ed. 2019, 58, 2272–2277. 10.1002/anie.201811420. 30600889

[ref626] Maeda C.; Tanaka Y.; Shirakawa T.; Ema T. Synthesis and Electronic Properties of π-Expanded Carbazole-Based Porphyrins. Chem. Commun. 2019, 55, 10162–10165. 10.1039/C9CC05079E. 31389426

[ref627] Maeda C.; Kurihara K.; Masuda M.; Yoshioka N. Effects of Cyano, Ethynyl and Ethylenedioxy Groups on the Photophysical Properties of Carbazole-Based Porphyrins. Org. Biomol. Chem. 2015, 13, 11286–11291. 10.1039/C5OB01824B. 26416478

[ref628] Maeda C.; Ema T. Chiral Carbazole-Based Porphyrins Showing Absorption and Circular Dichroism in the near-Infrared Region. J. Porphyrins Phthalocyanines 2020, 24, 247–251. 10.1142/S1088424619500937.

[ref629] Maeda C.; Takaishi K.; Ema T. Palladium Complexes of Carbazole-Based Chalcogenaisophlorins: Synthesis, Structure, and Solid-State NIR Absorption Spectra. ChemPlusChem 2017, 82, 1368–1371. 10.1002/cplu.201700430. 31957237

[ref630] Wu T.; Kim T.; Yin B.; Wang K.; Xu L.; Zhou M.; Kim D.; Song J. Carbazole-Containing Porphyrinoid and Its Oligomers. Chem. Commun. 2019, 55, 11454–11457. 10.1039/C9CC06114B. 31490520

[ref631] Kalaiselvan A.; Vamsi Krishna I. S.; Nambiar A. P.; Edwin A.; Reddy V. S.; Gokulnath S. Carbazole-Based Porphyrins: Synthesis, Structure-Photophysical Property Correlations, and Mercury Ion Sensing. Org. Lett. 2020, 22, 4494–4499. 10.1021/acs.orglett.0c01500. 32406691

[ref632] Maeda C.; Takata M.; Honsho A.; Ema T. Intramolecular Electronic Coupling in the Thiophene-Bridged Carbazole-Based Diporphyrin. Org. Lett. 2016, 18, 6070–6073. 10.1021/acs.orglett.6b03054. 27934383

[ref633] Maeda C.; Shirakawa T.; Ema T. Synthesis and Electronic Properties of Carbazole-Based Core-Modified Diporphyrins Showing near Infrared Absorption. Chem. Commun. 2020, 56, 15048–15051. 10.1039/D0CC06289H. 33196711

[ref634] Maulbetsch T.; Kunz D. Carbenaporphyrins: No Longer Missing Ligands in N-Heterocyclic Carbene Chemistry. Angew. Chem., Int. Ed. 2021, 60, 2007–2012. 10.1002/anie.202013434. PMC7898644 33078891

[ref635] Zhang P.; Yu C.; Yin Y.; Droste J.; Klabunde S.; Hansen M. R.; Mai Y. Bis-Anthracene Fused Porphyrin as an Efficient Photocatalyst: Facile Synthesis and Visible-Light-Driven Oxidative Coupling of Amines. Chem. - Eur. J. 2020, 26, 16497–16503. 10.1002/chem.202003398. 32720370

[ref636] Kato K.; Cha W.; Oh J.; Furukawa K.; Yorimitsu H.; Kim D.; Osuka A. Spontaneous Formation of an Air-Stable Radical upon the Direct Fusion of Diphenylmethane to a Triarylporphyrin. Angew. Chem., Int. Ed. 2016, 55, 8711–8714. 10.1002/anie.201602683. 27257973

[ref637] Kato K.; Furukawa K.; Mori T.; Osuka A. Porphyrin-Based Air-Stable Helical Radicals. Chem. - Eur. J. 2018, 24, 572–575. 10.1002/chem.201705291. 29194817

[ref638] Umetani M.; Naoda K.; Tanaka T.; Lee S.-K.; Oh J.; Kim D.; Osuka A. Synthesis of Di-Peri-Dinaphthoporphyrins by PtCl2-Mediated Cyclization of Quinodimethane-Type Porphyrins. Angew. Chem., Int. Ed. 2016, 55, 6305–6309. 10.1002/anie.201601303. 27073133

[ref639] Umetani M.; Naoda K.; Tanaka T.; Osuka A. Synthesis and Antiaromatic Character of Alkyl-Substituted Di-Peri-Dinaphthoporphyrin Ni(II) Complex. J. Porphyrins Phthalocyanines 2017, 21, 850–856. 10.1142/S1088424617500808.

[ref640] Fujimoto K.; Oh J.; Yorimitsu H.; Kim D.; Osuka A. Directly Diphenylborane-Fused Porphyrins. Angew. Chem., Int. Ed. 2016, 55, 3196–3199. 10.1002/anie.201511981. 26821874

[ref641] Kato K.; Kim J. O.; Yorimitsu H.; Kim D.; Osuka A. Triphenylsilane-Fused Porphyrins. Chem. - Asian J. 2016, 11, 1738–1746. 10.1002/asia.201600424. 27124659

[ref642] Fujimoto K.; Kasuga Y.; Fukui N.; Osuka A. Diphenylphosphine-Oxide-Fused and Diphenylphosphine-Fused Porphyrins: Synthesis, Tunable Electronic Properties, and Formation of Cofacial Dimers. Chem. - Eur. J. 2017, 23, 6741–6745. 10.1002/chem.201700909. 28397373

[ref643] Fujimoto K.; Osuka A. Effective Stabilization of a Planar Phosphorus(III) Center Embedded in a Porphyrin-Based Fused Aromatic Skeleton. Chem. Sci. 2017, 8, 8231–8239. 10.1039/C7SC03882H. 29568471 PMC5857933

[ref644] Chen Q.; Brambilla L.; Daukiya L.; Mali K. S.; De Feyter S.; Tommasini M.; Müllen K.; Narita A. Synthesis of Triply Fused Porphyrin-Nanographene Conjugates. Angew. Chem., Int. Ed. 2018, 57, 11233–11237. 10.1002/anie.201805063. 29984483

[ref645] Fukui N.; Lee S.-K.; Kato K.; Shimizu D.; Tanaka T.; Lee S.; Yorimitsu H.; Kim D.; Osuka A. Regioselective Phenylene-Fusion Reactions of Ni(II)-Porphyrins Controlled by an Electron-Withdrawing Meso-Substituent. Chem. Sci. 2016, 7, 4059–4066. 10.1039/C5SC04748J. 30155049 PMC6013929

[ref646] Martin M. M.; Oleszak C.; Hampel F.; Jux N. Oxidative Cyclodehydrogenation Reactions with Tetraarylporphyrins. Eur. J. Org. Chem. 2020, 2020, 6758–6762. 10.1002/ejoc.202001174.

[ref647] Kopp S. M.; Gotfredsen H.; Deng J.-R.; Claridge T. D. W.; Anderson H. L. Global Aromaticity in a Partially Fused 8-Porphyrin Nanoring. J. Am. Chem. Soc. 2020, 142, 19393–19401. 10.1021/jacs.0c09973. 33125228

[ref648] Ooi S.; Adinarayana B.; Shimizu D.; Tanaka T.; Osuka A. Stable Meso-Meso-Linked 2NH-Corrole Radical Dimers as a Key Intermediate to Corrole Tape. Angew. Chem., Int. Ed. 2020, 59, 9423–9427. 10.1002/anie.202002976. 32170827

[ref649] Ooi S.; Tanaka T.; Ikeue T.; Yamasumi K.; Ueta K.; Shimizu D.; Ishida M.; Furuta H.; Osuka A. Bis-Copper(II) Complex of Triply-Linked Corrole Dimer and Its Dication. Chem. - Asian J. 2019, 14, 1771–1776. 10.1002/asia.201801467. 30376217

[ref650] Ooi S.; Shimizu D.; Furukawa K.; Tanaka T.; Osuka A. Stable Face-to-Face Singlet Diradicaloids: Triply Linked Corrole Dimer Gallium(III) Complexes with Two μ-Hydroxo-Bridges. Angew. Chem., Int. Ed. 2018, 57, 14916–14920. 10.1002/anie.201810200. 30239078

[ref651] Lee S.; Yamashita K.; Sakata N.; Hirao Y.; Ogawa K.; Ogawa T. Stable Singlet Biradicals of Rare-Earth-Fused Diporphyrin-Triple-Decker Complexes with Low Energy Gaps and Multi-Redox States. Chem. - Eur. J. 2019, 10.1002/chem.201805927. 30609157

[ref652] Mori H.; Kim T.; Kim D.; Osuka A. Trimeric and Tetrameric Electron-Deficient Porphyrin Tapes. Chem. - Asian J. 2016, 11, 1454–1463. 10.1002/asia.201600241. 26991968

[ref653] Yamashita K.; Hirano D.; Fujimaki K.; Sugiura K. Synthesis of Porphyrinquinone and Doubly-Fused Diporphyrin Quinone Through Oxidation of Diarylporphyrins Using a Hypervalent Iodine Compound. Chem. - Asian J. 2020, 15, 3037–3043. 10.1002/asia.202000781. 32749058

[ref654] Fukui N.; Kim T.; Kim D.; Osuka A. Porphyrin Arch-Tapes: Synthesis, Contorted Structures, and Full Conjugation. J. Am. Chem. Soc. 2017, 139, 9075–9088. 10.1021/jacs.7b05332. 28622724

[ref655] Fukui N.; Osuka A. Singly and Doubly 1,2-Phenylene-Inserted Porphyrin Arch-Tape Dimers: Synthesis and Highly Contorted Structures. Angew. Chem., Int. Ed. 2018, 57, 6304–6308. 10.1002/anie.201802494. 29577532

[ref656] Fukui N.; Osuka A. Singly and Doubly Sulfone-Inserted Porphyrin Arch-Tape Dimers. Bull. Chem. Soc. Jpn. 2018, 91, 1131–1137. 10.1246/bcsj.20180103. 29577532

[ref657] Dimé A. K. D.; Devillers C. H.; Cattey H.; Lucas D. Versatile Redox Reactivity of Triaryl-Meso-Substituted Ni(II) Porphyrin. Dalton Trans. 2014, 43, 14554–14564. 10.1039/C4DT00221K. 24828141

[ref658] Konev D. V.; Devillers C. H.; Lizgina K. V.; Baulin V. E.; Vorotyntsev M. A. Electropolymerization of Non-Substituted Mg(II) Porphine: Effects of Proton Acceptor Addition. J. Electroanal. Chem. 2015, 737, 235–242. 10.1016/j.jelechem.2014.09.018.

[ref659] Rolle S. D.; Konev D. V.; Devillers C. H.; Lizgina K. V.; Lucas D.; Stern C.; Herbst F.; Heintz O.; Vorotyntsev M. A. Efficient Synthesis of a New Electroactive Polymer of Co(II) Porphine by in-Situ Replacement of Mg(II) inside Mg(II) Polyporphine Film. Electrochim. Acta 2016, 204, 276–286. 10.1016/j.electacta.2016.03.039.

[ref660] Rolle S. D.; Devillers C. H.; Fournier S.; Heintz O.; Gibault H.; Lucas D. A Glassy Carbon Electrode Modified by a Triply-Fused-like Co(II) Polyporphine and Its Ability for Sulphite Oxidation and Detection. New J. Chem. 2018, 42, 8180–8189. 10.1039/C7NJ04370H.

[ref661] Bengasi G.; Baba K.; Frache G.; Desport J.; Gratia P.; Heinze K.; Boscher N. D. Conductive Fused Porphyrin Tapes on Sensitive Substrates by a Chemical Vapor Deposition Approach. Angew. Chem., Int. Ed. 2019, 58, 2103–2108. 10.1002/anie.201814034. PMC6582438 30556943

[ref662] Baba K.; Bengasi G.; El Assad D.; Grysan P.; Lentzen E.; Heinze K.; Frache G.; Boscher N. D. Conductive Directly Fused Poly(Porphyrin) Coatings by Oxidative Chemical Vapour Deposition - From Single- to Triple-Fused. Eur. J. Org. Chem. 2019, 2019, 2368–2375. 10.1002/ejoc.201900045.

[ref663] Bengasi G.; Baba K.; Back O.; Frache G.; Heinze K.; Boscher N. D. Reactivity of Nickel(II) Porphyrins in OCVD Processes—Polymerisation, Intramolecular Cyclisation and Chlorination. Chem. - Eur. J. 2019, 25, 8313–8320. 10.1002/chem.201900793. 30939219 PMC6771558

[ref664] Bengasi G.; Desport J. S.; Baba K.; Cosas Fernandes J. P.; De Castro O.; Heinze K.; Boscher N. D. Molecular Flattening Effect to Enhance the Conductivity of Fused Porphyrin Tape Thin Films. RSC Adv. 2020, 10, 7048–7057. 10.1039/C9RA09711B. PMC9049719 35493879

[ref665] Bengasi G.; Quétu L.; Baba K.; Ost A.; Fernandes J. P. C.; Grysan P.; Heinze K.; Boscher N. D. Constitution and Conductivity of Metalloporphyrin Tapes. Eur. J. Inorg. Chem. 2020, 2020, 1938–1945. 10.1002/ejic.202000243.

[ref666] Baba K.; Bengasi G.; Loyer F.; Fernandes J. P. C.; El Assad D.; De Castro O.; Boscher N. D. Fused Metalloporphyrin Thin Film with Tunable Porosity via Chemical Vapor Deposition. ACS Appl. Mater. Interfaces 2020, 12, 37732–37740. 10.1021/acsami.0c09630. 32692925 PMC7472432

[ref667] Huerta-Flores A. M.; Bengasi G.; Baba K.; Boscher N. D. Fused Porphyrin Thin Films as Heterogeneous Visible-Light Active Photocatalysts with Well-Defined Active Metal Sites for Hydrogen Generation. ACS Appl. Energy Mater. 2020, 3, 9848–9855. 10.1021/acsaem.0c01545.

[ref668] Bengasi G.; Meunier-Prest R.; Baba K.; Kumar A.; Pellegrino A. L.; Boscher N. D.; Bouvet M. Molecular Engineering of Porphyrin-Tapes/Phthalocyanine Heterojunctions for a Highly Sensitive Ammonia Sensor. Adv. Electron. Mater. 2020, 6, 2000812 10.1002/aelm.202000812.

[ref669] Mateo L. M.; Sun Q.; Liu S.-X.; Bergkamp J. J.; Eimre K.; Pignedoli C. A.; Ruffieux P.; Decurtins S.; Bottari G.; Fasel R.; et al. On-Surface Synthesis and Characterization of Triply Fused Porphyrin-Graphene Nanoribbon Hybrids. Angew. Chem., Int. Ed. 2020, 59, 1334–1339. 10.1002/anie.201913024. 31729821

[ref670] Mateo L. M.; Sun Q.; Eimre K.; Pignedoli C. A.; Torres T.; Fasel R.; Bottari G. On-Surface Synthesis of Singly and Doubly Porphyrin-Capped Graphene Nanoribbon Segments. Chem. Sci. 2021, 12, 247–252. 10.1039/D0SC04316H. PMC8178705 34163593

[ref671] Forezi L. d. S. M.; Gomes A. T. P. C.; Neves M. G. P. M. S.; Ferreira V. F.; Boechat F. d. C. S.; de Souza M. C. B. V.; Cavaleiro J. A. S. Synthesis of β-Substituted Meso-Tetraaryl-21,23-Dithiaporphyrins by Heck Reaction. Eur. J. Org. Chem. 2015, 2015, 5909–5913. 10.1002/ejoc.201500812.

[ref672] Tebi S.; Paszkiewicz M.; Aldahhak H.; Allegretti F.; Gonglach S.; Haas M.; Waser M.; Deimel P. S.; Aguilar P. C.; Zhang Y.-Q.; et al. On-Surface Site-Selective Cyclization of Corrole Radicals. ACS Nano 2017, 11, 3383–3391. 10.1021/acsnano.7b00766. 28212484

[ref673] Saegusa Y.; Ishizuka T.; Shiota Y.; Yoshizawa K.; Kojima T. Acid-Base Properties of a Freebase Form of a Quadruply Ring-Fused Porphyrin—Stepwise Protonation Induced by Rigid Ring-Fused Structure. J. Org. Chem. 2017, 82, 322–330. 10.1021/acs.joc.6b02419. 27966350

[ref674] Saegusa Y.; Ishizuka T.; Kojima T. Substituent Effects at the β-Positions of the Nonfused Pyrroles in a Quadruply Fused Porphyrin on the Structure and Optical and Electrochemical Properties. Inorg. Chem. 2018, 57, 1106–1115. 10.1021/acs.inorgchem.7b02551. 29360352

[ref675] Saegusa Y.; Ishizuka T.; Shiota Y.; Yoshizawa K.; Kojima T. NH Tautomerism of a Quadruply Fused Porphyrin: Rigid Fused Structure Delays the Proton Transfer. J. Phys. Chem. B 2018, 122, 316–327. 10.1021/acs.jpcb.7b10945. 29224345

[ref676] Li J.; He N.; Liu Y.; Zhang Z.; Zhang X.; Han X.; Gai Y.; Liu Y.; Yin J.; Wang J. Synthesis and Photophysical Properties of Novel Pyridine Fused Chlorophyll a Derivatives. Dyes Pigm. 2017, 146, 189–198. 10.1016/j.dyepig.2017.07.005.

[ref677] Higashino T.; Nishimura I.; Imahori H. Phosphole-Fused Dehydropurpurins via Titanium-Mediated [2 + 2+1] Cyclization Strategy. Chem. - Eur. J. 2019, 25, 13816–13823. 10.1002/chem.201903269. 31393036

[ref678] Higashino T.; Nishimura I.; Imahori H. Unique Role of Heterole-Fused Structures in Aromaticity and Physicochemical Properties of 7,8-Dehydropurpurins. Chem. - Eur. J. 2020, 26, 12043–12049. 10.1002/chem.202001361. 32338789

[ref679] Nagai T.; Takiguchi A.; Ueda M.; Oda K.; Hiroto S.; Shinokubo H. X-Shaped Cyclobutane-Linked Tetraporphyrins through a Thermal [2 + 2] Cycloaddition of Etheno-Fused Diporphyrins. J. Am. Chem. Soc. 2018, 140, 8392–8395. 10.1021/jacs.8b04673. 29925230

[ref680] Miyagawa K.; Hisaki I.; Fukui N.; Shinokubo H. Redox-Induced Reversible [2 + 2] Cycloaddition of an Etheno-Fused Diporphyrin. Chem. Sci. 2021, 12, 5224–5229. 10.1039/D1SC00438G. 34168775 PMC8179634

[ref681] Li M.; Wei P.; Ishida M.; Li X.; Savage M.; Guo R.; Ou Z.; Yang S.; Furuta H.; Xie Y. Macrocyclic Transformations from Norrole to Isonorrole and an N-Confused Corrole with a Fused Hexacyclic Ring System Triggered by a Pyrrole Substituent. Angew. Chem., Int. Ed. 2016, 55, 3063–3067. 10.1002/anie.201510879. 26822959

[ref682] Kumar Maurya Y.; Wei P.; Shimada T.; Yamasumi K.; Mori S.; Furukawa K.; Kusaba H.; Ishihara T.; Xie Y.; Ishida M.; Furuta H.; et al. Chiral Interlocked Corrole Dimers Directly Linked at Inner Carbon Atoms of Confused Pyrrole Rings. Chem. - Asian J. 2021, 16, 743–747. 10.1002/asia.202100083. 33624937

[ref683] Fujimoto K.; Shimizu D.; Osuka A. Porphyrin-Stabilized Nitrenium Dication. Chem. - Eur. J. 2018, 25, 521–525. 10.1002/chem.201805491. 30402895

[ref684] Fujimoto K.; Shimizu D.; Mori T.; Li Y.; Zhou M.; Song J.; Osuka A. Selective Formation of Helical Tetrapyrrin-Fused Porphyrins by Oxidation of β-to-β Linked Meso-Aminoporphyrin Dimers. Chem. - Eur. J. 2018, 25, 1711–1715. 10.1002/chem.201805659. 30537047

[ref685] Li Y.; Zhou M.; Xu L.; Zhou B.; Rao Y.; Nie H.; Gu T.; Zhou J.; Liang X.; Yin B.; et al. Simultaneous Implementation of N-Heterocycle-Fused Bridge and Modified Pyrrole Unit on Ni(II) Porphyrin Dimers. Org. Lett. 2020, 22, 6001–6005. 10.1021/acs.orglett.0c02084. 32692918

[ref686] Isar P.; Ravikanth M. Dibenzoylbenzodipyrroles: Key Precursors for the Synthesis of Fused *Meso* -Aryl Sapphyrins. J. Org. Chem. 2020, 85, 7287–7296. 10.1021/acs.joc.0c00663. 32403925

[ref687] Firmansyah D.; Hong S.-J.; Dutta R.; He Q.; Bae J.; Jo H.; Kim H.; Ok K. M.; Lynch V. M.; Byon H. R.; et al. Trapping of Stable [4n+1] π-Electron Species from Peripherally Substituted, Conformationally Rigid, Antiaromatic Hexaphyrins. Chem. - Eur. J. 2019, 25, 3525–3531. 10.1002/chem.201900022. 30684359

[ref688] Ishida M.; Furuyama T.; Lim J. M.; Lee S.; Zhang Z.; Ghosh S. K.; Lynch V. M.; Lee C.-H.; Kobayashi N.; Kim D.; et al. Structural, Photophysical, and Magnetic Circular Dichroism Studies of Three Rigidified Meso-Pentafluorophenyl-Substituted Hexaphyrin Analogues. Chem. - Eur. J. 2017, 23, 6682–6692. 10.1002/chem.201700759. 28317197

[ref689] Kim G.; Dutta R.; Cha W.-Y.; Hong S.-J.; Oh J.; Firmansyah D.; Jo H.; Ok K. M.; Lee C.-H.; Kim D. Noncovalent Intermolecular Interaction in Cofacially Stacked 24π Antiaromatic Hexaphyrin Dimer. Chem. - Eur. J. 2020, 26, 16434–16440. 10.1002/chem.202002884. 32557895

[ref690] Dutta R.; Chandra B.; Hong S.-J.; Park Y.; Jung Y. M.; Lee C.-H. Post Synthetic Modification of Planar Antiaromatic Hexaphyrin (1.0.1.0.1.0) by Regio-Selective, Sequential SNAr. Molecules 2021, 26, 1025 10.3390/molecules26041025. 33672044 PMC7919474

[ref691] Samala S.; Dutta R.; He Q.; Park Y.; Chandra B.; Lynch V. M.; Jung Y. M.; Sessler J. L.; Lee C.-H. A Robust Bis-Rhodium(I) Complex of π-Extended Planar, Anti-Aromatic Hexaphyrin[1.0.1.0.1.0]. Chem. Commun. 2020, 56, 758–761. 10.1039/C9CC09221H. 31845684

[ref692] Sarma T.; Kim G.; Sen S.; Cha W.-Y.; Duan Z.; Moore M. D.; Lynch V. M.; Zhang Z.; Kim D.; Sessler J. L. Proton-Coupled Redox Switching in an Annulated π-Extended Core-Modified Octaphyrin. J. Am. Chem. Soc. 2018, 140, 12111–12119. 10.1021/jacs.8b06938. 30180553

[ref693] Bhowmik S.; Kosa M.; Mizrahi A.; Fridman N.; Saphier M.; Stanger A.; Gross Z. The Planar Cyclooctatetraene Bridge in Bis-Metallic Macrocycles: Isolating or Conjugating?. Inorg. Chem. 2017, 56, 2287–2296. 10.1021/acs.inorgchem.6b02944. 28182414

[ref694] Ghosh A.; Chaudhary A.; Srinivasan A.; Suresh C. H.; Chandrashekar T. K. [32]π Fused Core-Modified Heptaphyrin with Möbius Aromaticity. Chem. - Eur. J. 2016, 22, 3942–3946. 10.1002/chem.201505116. 26781367

[ref695] Dash S.; Ghosh A.; Chandrashekar T. K. Dithienothiophene Fused 30π Heptaphyrin and 34π Octaphyrins: Syntheses, Characterization and Spectral Properties. J. Porphyrins Phthalocyanines 2020, 24, 98–104. 10.1142/S1088424619500974.

[ref696] Ghosh A.; Srinivasan A.; Suresh C. H.; Chandrashekar T. K. [40]π Fused and Nonfused Core-Modified Nonaphyrins: Syntheses and Structural Diversity. Chem. - Eur. J. 2016, 22, 11152–11155. 10.1002/chem.201602245. 27272458

[ref697] Cha W.-Y.; Kim T.; Ghosh A.; Zhang Z.; Ke X.-S.; Ali R.; Lynch V. M.; Jung J.; Kim W.; Lee S.; et al. Bicyclic Baird-Type Aromaticity. Nat. Chem. 2017, 9, 1243–1248. 10.1038/nchem.2834. 29168483

[ref698] Cha W.-Y.; Ahn A.; Kim T.; Oh J.; Ali R.; Park J. S.; Kim D. Changes in Macrocyclic Aromaticity and Formation of a Charge-Separated State by Complexation of Expanded Porphyrin and C60. Chem. Commun. 2019, 55, 8301–8304. 10.1039/C9CC03928G. 31250853

[ref699] Higashino T.; Kumagai A.; Imahori H. Thiophene-Fused Dithiaoctaphyrins: π-System Switching between Cross-Conjugated and Macrocyclic π-Networks. Chem. Commun. 2017, 53, 5091–5094. 10.1039/C7CC01273J. 28304031

[ref700] Higashino T.; Kumagai A.; Sakaki S.; Imahori H. Reversible π-System Switching of Thiophene-Fused Thiahexaphyrins by Solvent and Oxidation/Reduction. Chem. Sci. 2018, 9, 7528–7539. 10.1039/C8SC02448K. 30319753 PMC6179095

[ref701] Reddy B. K.; Rawson J.; Gadekar S. C.; Kögerler P.; Anand V. G. A Naphthalene-Fused Dimer of an Anti-Aromatic Expanded Isophlorin. Chem. Commun. 2017, 53, 8211–8214. 10.1039/C7CC04050D. 28681895

[ref702] Ke X.-S.; Hong Y.; Tu P.; He Q.; Lynch V. M.; Kim D.; Sessler J. L. Hetero Cu(III)-Pd(II) Complex of a Dibenzo[g, p]Chrysene-Fused Bis-Dicarbacorrole with Stable Organic Radical Character. J. Am. Chem. Soc. 2017, 139, 15232–15238. 10.1021/jacs.7b09167. 28965390

[ref703] Ke X.-S.; Hong Y.; Lynch V. M.; Kim D.; Sessler J. L. Metal-Stabilized Quinoidal Dibenzo[g, p]Chrysene-Fused Bis-Dicarbacorrole System. J. Am. Chem. Soc. 2018, 140, 7579–7586. 10.1021/jacs.8b02718. 29787675

[ref704] Ke X.-S.; Kim T.; He Q.; Lynch V. M.; Kim D.; Sessler J. L. Three-Dimensional Fully Conjugated Carbaporphyrin Cage. J. Am. Chem. Soc. 2018, 140, 16455–16459. 10.1021/jacs.8b11158. 30452259

[ref705] Szyszko B.; Przewoznik M.; Białek M. J.; Białońska A.; Chmielewski P. J.; Cichos J.; Latos-Grazyński L. Helicenophyrins: Expanded Carbaporphyrins Incorporating Aza[5]Helicene and Heptacyclic S-Shaped Aza[5]Helicene Motifs. Angew. Chem., Int. Ed. 2018, 57, 4030–4034. 10.1002/anie.201800879. 29451346

[ref706] Kupietz K.; Białek M. J.; Białońska A.; Szyszko B.; Latos-Grazyński L. Aromaticity Control via Modifications of a Macrocyclic Frame: 5,6-Dimethoxyphenanthriporphyrin and 5,6-Dioxophenanthriporphyrin. Org. Chem. Front. 2018, 5, 3068–3076. 10.1039/C8QO00751A.

[ref707] Kupietz K.; Białek M. J.; Białońska A.; Szyszko B.; Latos-Grazyński L. Organocopper(III) Phenanthriporphyrin—Exocyclic Transformations. Inorg. Chem. 2019, 58, 1451–1461. 10.1021/acs.inorgchem.8b02997. 30600994

[ref708] Szyszko B.; Drózdz D.; Sarwa A.; Mucha S. G.; Białońska A.; Białek M. J.; Matczyszyn K.; Latos-Grazyński L. An Exocyclic π-System Extension of the Phenanthriporphyrin Framework: Towards Azaaceneporphyrinoids. Org. Chem. Front. 2020, 7, 1430–1436. 10.1039/D0QO00436G.

[ref709] Szyszko B.; Małecki M.; Berlicka A.; Białek M. J.; Białońska A.; Kupietz K.; Pacholska-Dudziak E.; Latos-Grazyński L. Incorporation of a Phenanthrene Subunit into a Sapphyrin Framework: Synthesis of Expanded Aceneporphyrinoids. Chem. - Eur. J. 2016, 22, 7602–7608. 10.1002/chem.201600606. 27098207

[ref710] Szyszko B.; Chmielewski P. J.; Przewoznik M.; Białek M. J.; Kupietz K.; Białońska A.; Latos-Grazyński L. Diphenanthrioctaphyrin(1.1.1.0.1.1.1.0): Conformational Switching Controls the Stereochemical Dynamics of the Topologically Chiral System. J. Am. Chem. Soc. 2019, 141, 6060–6072. 10.1021/jacs.9b01357. 30895778

[ref711] Szyszko B.; Przewoznik M.; Białek M. J.; Białońska A.; Chmielewski P. J.; Latos-Grazyński L. Conformation-Dependent Response to the Protonation of Diphenanthrioctaphyrin(1.1.1.0.1.1.1.0): A Route to Pseudorotaxane-Like Structures. Chem. - Eur. J. 2020, 26, 8555–8566. 10.1002/chem.202000940. 32203626

[ref712] Szyszko B.; Rymut P.; Matviyishyn M.; Białońska A.; Latos-Grazyński L. Kinetic versus Thermodynamic Control Over Multiple Conformations of Di-2,7-Naphthihexaphyrin(1.1.1.1.1.1). Angew. Chem., Int. Ed. 2020, 59, 20137–20146. 10.1002/anie.202008518. 33462869

[ref713] Aslam A. S.; Hong J.-H.; Shin J.-H.; Cho D.-G. Synthesis of a Phlorin from a Meso-Fused Anthriporphyrin by a Diels-Alder Strategy. Angew. Chem., Int. Ed. 2017, 56, 16247–16251. 10.1002/anie.201709026. 29098755

[ref714] Hong J.-H.; Aslam A. S.; Ishida M.; Mori S.; Furuta H.; Cho D.-G. 2-(Naphthalen-1-yl)Thiophene as a New Motif for Porphyrinoids: Meso-Fused Carbaporphyrin. J. Am. Chem. Soc. 2016, 138, 4992–4995. 10.1021/jacs.6b01063. 26906064

[ref715] Hong J.-H.; Aslam A. S.; Cho B.; Ko M.-S.; Kim Y.; Heo J.; Cho D.-G. Carbaporphyrin Dimers That Bear a Rigid Naphthalene Motif as an Internal Strap. Org. Lett. 2021, 23, 1846–1850. 10.1021/acs.orglett.1c00252. 33577339

[ref716] Kumar A.; Thorat K. G.; Sinha A.; Butcher R. J.; Ravikanth M. Meso-Fused Carbatriphyrins(2.1.1) and Its Organo Phosphorus(V) Complex. J. Org. Chem. 2019, 84, 9067–9074. 10.1021/acs.joc.9b01015. 31264867

[ref717] Kumar A.; Laxman K.; Ravikanth M. Antiaromatic Carbaporphyrinoids: Fluorene as a Fused Motif toward the Synthesis of Meso-Fused Heterobenziporphyrins. Org. Lett. 2019, 21, 8726–8730. 10.1021/acs.orglett.9b03329. 31618042

[ref718] Kumar A.; Thorat K. G.; Ravikanth M. Synthesis and Studies of Strained Fluorenophyrins. J. Org. Chem. 2019, 84, 10321–10327. 10.1021/acs.joc.9b01486. 31331168

[ref719] Kumar A.; Rajeswara Rao M.; Lee W.-Z.; Ravikanth M. Hybrid Macrocycles of Subporphyrins and Triphyrins. Org. Lett. 2017, 19, 5924–5927. 10.1021/acs.orglett.7b02919. 29052997

[ref720] Kumar A.; Thorat K. G.; Ravikanth M. Benzofuran-/Benzothiophene-Incorporated NIR-Absorbing Triphyrins(2.1.1). Org. Lett. 2018, 20, 4871–4874. 10.1021/acs.orglett.8b02012. 30067043

[ref721] Szyszko B.; Matviyishyn M.; Hirka S.; Pacholska-Dudziak E.; Białońska A.; Latos-Grazyński L. 28-Hetero-2,7-Naphthiporphyrins: Horizontal Expansion of the m-Benziporphyrin Macrocycle. Org. Lett. 2019, 21, 7009–7014. 10.1021/acs.orglett.9b02587. 31423794

[ref722] Rao Y.; Kim J. O.; Kim W.; Zhong G.; Yin B.; Zhou M.; Shinokubo H.; Aratani N.; Tanaka T.; Liu S.; et al. β-to-β 2,5-Pyrrolylene-Linked Cyclic Porphyrin Oligomers. Chem. - Eur. J. 2016, 22, 8801–8804. 10.1002/chem.201601306. 27124728

[ref723] Yin B.; Liang X.; Zhu W.; Xu L.; Zhou M.; Song J. β to β Terpyridylene-Bridged Porphyrin Nanorings. Chin. Chem. Lett. 2018, 29, 99–101. 10.1016/j.cclet.2017.05.003.

[ref724] Xu Y.; Gsänger S.; Minameyer M. B.; Imaz I.; Maspoch D.; Shyshov O.; Schwer F.; Ribas X.; Drewello T.; Meyer B.; et al. Highly Strained, Radially π-Conjugated Porphyrinylene Nanohoops. J. Am. Chem. Soc. 2019, 141, 18500–18507. 10.1021/jacs.9b08584. 31710474

[ref725] Ren L.; Gopalakrishna T. Y.; Park I.-H.; Han Y.; Wu J. Porphyrin/Quinoidal-Bithiophene-Based Macrocycles and Their Dications: Template-Free Synthesis and Global Aromaticity. Angew. Chem., Int. Ed. 2020, 59, 2230–2234. 10.1002/anie.201911269. 31692181

[ref726] Sung Y. M.; Oh J.; Kim W.; Mori H.; Osuka A.; Kim D. Switching between Aromatic and Antiaromatic 1,3-Phenylene-Strapped [26]- and [28]Hexaphyrins upon Passage to the Singlet Excited State. J. Am. Chem. Soc. 2015, 137, 11856–11859. 10.1021/jacs.5b04047. 26340352

[ref727] Oh J.; Sung Y. M.; Mori H.; Park S.; Jorner K.; Ottosson H.; Lim M.; Osuka A.; Kim D. Unraveling Excited-Singlet-State Aromaticity via Vibrational Analysis. Chem. 2017, 3, 870–880. 10.1016/j.chempr.2017.09.005.

[ref728] Soya T.; Mori H.; Osuka A. Quadruply Twisted Hückel-Aromatic Dodecaphyrin. Angew. Chem., Int. Ed. 2018, 57, 15882–15886. 10.1002/anie.201811433. 30324734

[ref729] Soya T.; Mori H.; Hong Y.; Koo Y. H.; Kim D.; Osuka A. Internally 2,5-Thienylene-Bridged [46]Decaphyrin: (Annuleno)Annulene Network Consisting of Möbius Aromatic Thia[28]Hexaphyrins and Strong Hückel Aromaticity of Its Protonated Form. Angew. Chem., Int. Ed. 2017, 56, 3232–3236. 10.1002/anie.201700607. 28252243

[ref730] Soya T.; Osuka A. Internally Bridged Hückel Aromatic [46]Decaphyrins: (Doubly-Twisted-Annuleno)Doubly-Twisted-Annulene Variants. Chem. - Eur. J. 2019, 25, 5173–5176. 10.1002/chem.201900819. 30850993

[ref731] Ni Y.; Gopalakrishna T. Y.; Phan H.; Kim T.; Herng T. S.; Han Y.; Tao T.; Ding J.; Kim D.; Wu J. 3D Global Aromaticity in a Fully Conjugated Diradicaloid Cage at Different Oxidation States. Nat. Chem. 2020, 12, 242–248. 10.1038/s41557-019-0399-2. 31959959

[ref732] Białek M.ł J.; Latos-Grazynski L.ła. Aromaticity Switching via Azulene Transformations in Azulene-Bridged A,D-Dithiahexaphyrin. Chem. Commun. 2018, 54, 1837–1840. 10.1039/C7CC08754C. 29296986

[ref733] Rao Y.; Kim T.; Park K. H.; Peng F.; Liu L.; Liu Y.; Wen B.; Liu S.; Kirk S. R.; Wu L.; et al. π-Extended “Earring” Porphyrins with Multiple Cavities and Near-Infrared Absorption. Angew. Chem., Int. Ed. 2016, 55, 6438–6442. 10.1002/anie.201600955. 27038255

[ref734] Guan H.; Zhou M.; Yin B.; Xu L.; Song J. Synthesis and Characterization of π-Extended “Earring” Subporphyrins. Beilstein J. Org. Chem. 2018, 14, 1956–1960. 10.3762/bjoc.14.170. 30202449 PMC6122274

[ref735] Wu L.; Li F.; Rao Y.; Wen B.; Xu L.; Zhou M.; Tanaka T.; Osuka A.; Song J. Synthesis, Structures, and Near-IR Absorption of Heterole-Fused Earring Porphyrins. Angew. Chem., Int. Ed. 2019, 58, 8124–8128. 10.1002/anie.201903446. 30983101

[ref736] Stawski W.; Hurej K.; Skonieczny J.; Pawlicki M. Organoboron Complexes in Edge-Sharing Macrocycles: The Triphyrin(2.1.1)-Tetraphyrin(1.1.1.1) Hybrid. Angew. Chem., Int. Ed. 2019, 58, 10946–10950. 10.1002/anie.201904819. 31141278

[ref737] Basumatary B.; Ramana Reddy R. V.; Rahul; Sankar J. The Curious Case of a Parasitic Twin of the Corroles. Angew. Chem., Int. Ed. 2018, 57, 5052–5056. 10.1002/anie.201801555. 29504712

[ref738] Xue S.; Kuzuhara D.; Aratani N.; Yamada H. Synthesis of a Porphyrin(2.1.2.1) Nanobelt and Its Ability To Bind Fullerene. Org. Lett. 2019, 21, 2069–2072. 10.1021/acs.orglett.9b00329. 30875231

[ref739] Meng Z.; Stolz R. M.; Mirica K. A. Two-Dimensional Chemiresistive Covalent Organic Framework with High Intrinsic Conductivity. J. Am. Chem. Soc. 2019, 141, 11929–11937. 10.1021/jacs.9b03441. 31241936

[ref740] Wang M.; Ballabio M.; Wang M.; Lin H.-H.; Biswal B. P.; Han X.; Paasch S.; Brunner E.; Liu P.; Chen M.; et al. Unveiling Electronic Properties in Metal-Phthalocyanine-Based Pyrazine-Linked Conjugated Two-Dimensional Covalent Organic Frameworks. J. Am. Chem. Soc. 2019, 141, 16810–16816. 10.1021/jacs.9b07644. 31557002

[ref741] Wang M.; Wang M.; Lin H.-H.; Ballabio M.; Zhong H.; Bonn M.; Zhou S.; Heine T.; Cánovas E.; Dong R.; et al. High-Mobility Semiconducting Two-Dimensional Conjugated Covalent Organic Frameworks with p-Type Doping. J. Am. Chem. Soc. 2020, 142, 21622–21627. 10.1021/jacs.0c10482. 33332109

[ref742] Uno H.; Muramatsu K.; Hiraoka S.; Tahara H.; Hirose M.; Tamura E.; Shiraishi T.; Mack J.; Kobayashi N.; Mori S.; et al. Synthesis and Aromaticity of Benzene-Fused Doubly N-Confused Porphyrins. Chem. - Eur. J. 2020, 26, 5701–5708. 10.1002/chem.202000339. 32147874

[ref743] Yoneda T.; Hoshino T.; Neya S. Stable Non-Fused [22]Pentaphyrins and A Fused [24]Pentaphyrin Displaying Crystal Polymorphism between Hückel and Möbius Structures. Chem. - Asian J. 2017, 12, 405–409. 10.1002/asia.201601689. 28028937

[ref744] Ishida S.; Kim J. O.; Kim D.; Osuka A. Doubly N-Fused [24]Pentaphyrin Silicon Complex and Its Fluorosilicate: Enhanced Möbius Aromaticity in the Fluorosilicate. Chem. - Eur. J. 2016, 22, 16554–16561. 10.1002/chem.201603598. 27706861

[ref745] Nakai A.; Yoneda T.; Tanaka T.; Osuka A. PdII Insertion-Triggered Meso-Carbon Extrusion of N-Fused Pentaphyrin to Form N-Fused Sapphyrin PdII Complexes. Chem. Commun. 2021, 57, 3034–3037. 10.1039/D1CC00518A. 33624682

[ref746] Li Q.; Ishida M.; Kai H.; Gu T.; Li C.; Li X.; Baryshnikov G.; Liang X.; Zhu W.; Ågren H.; et al. Skeletal Rearrangement of Twisted Thia-Norhexaphyrin: Multiply Annulated Polypyrrolic Aromatic Macrocycles. Angew. Chem., Int. Ed. 2019, 58, 5925–5929. 10.1002/anie.201900010. 30843636

[ref747] Jana A.; Ishida M.; Furuta H. Benzo-Tetrathiafulvalene- (BTTF-) Annulated Expanded Porphyrins: Potential Next-Generation Multielectron Reservoirs. Chem. - Eur. J. 2021, 27, 4466–4472. 10.1002/chem.202005021. 33347663

[ref748] Fu Y.; Liu X.; Li C.; Tong Z.; Baryshnikov G.; Ågren H.; Li Q.; Xie Y. Rational Synthesis of 5,5,5-Tricyclic Fused Thia-Heptaphyrin (1.1.1.1.1.1.0) From a Helical Oligopyrrin Hybrid. Chem. - Asian J. 2020, 15, 1285–1289. 10.1002/asia.202000100. 32128999

[ref749] Saltsman I.; Goldberg I.; Gross Z. One-Step Conversions of a Simple Corrole into Chiral and Amphiphilic Derivatives. Tetrahedron Lett. 2003, 44, 5669–5673. 10.1016/S0040-4039(03)01356-X.

[ref750] Saltsman I.; Simkhovich L.; Balazs Y.; Goldberg I.; Gross Z. Synthesis, Spectroscopy, and Structures of New Rhodium(I) and Rhodium(III) Corroles and Catalysis Thereby. Inorg. Chim. Acta 2004, 357, 3038–3046. 10.1016/j.ica.2004.05.002.

[ref751] Patra S. K.; Sahu K.; Patra B.; Sahoo D. K.; Mondal S.; Mukherjee P.; Biswal H. S.; Kar S. Synthesis of Urea Derivatives via Reductive Carbon Dioxide Fixation into Contracted Porphyrin Analogues. Green Chem. 2017, 19, 5772–5776. 10.1039/C7GC02798B.

[ref752] Idec A.; Pawlicki M.; Latos-Grazyński L. Three-Stage Aromaticity Switching in Boron(III) and Phosphorus(V) N-Fused p-Benziporphyrin. Chem. - Eur. J. 2019, 25, 200–204. 10.1002/chem.201804983. 30277286

[ref753] Marshall-Roth T.; Libretto N. J.; Wrobel A. T.; Anderton K. J.; Pegis M. L.; Ricke N. D.; Voorhis T. V.; Miller J. T.; Surendranath Y. A Pyridinic Fe-N4Macrocycle Models the Active Sites in Fe/N-Doped Carbon Electrocatalysts. Nat. Commun. 2020, 11, 5283 10.1038/s41467-020-18969-6. 33077736 PMC7572418

[ref754] Moriya M.; Takahama R.; Kamoi K.; Ohyama J.; Kawashima S.; Kojima R.; Okada M.; Hayakawa T.; Nabae Y. Fourteen-Membered Macrocyclic Fe Complexes Inspired by FeN _4_ -Center-Embedded Graphene for Oxygen Reduction Catalysis. J. Phys. Chem. C 2020, 124, 20730–20735. 10.1021/acs.jpcc.0c05536.

[ref755] Adachi S.; Shibasaki M.; Kumagai N. TriQuinoline. Nat. Commun. 2019, 10, 3820 10.1038/s41467-019-11818-1. 31444349 PMC6707140

[ref756] Shimasaki T.; Kuroda R.; Akao M.; Akimoto T.; Ishikawa T.; Iwanaga T.; Teramoto N.; Shibata M. Synthesis and Properties of a Conjugated Macrocyclic Molecule Incorporating Two Quinoline Moieties. Chem. Lett. 2019, 48, 133–136. 10.1246/cl.180888.

[ref757] Ikemoto K.; Yang S.; Naito H.; Kotani M.; Sato S.; Isobe H. A Nitrogen-Doped Nanotube Molecule with Atom Vacancy Defects. Nat. Commun. 2020, 11, 1807 10.1038/s41467-020-15662-6. 32286324 PMC7156684

[ref758] Chen F.; Tanaka T.; Hong Y. S.; Mori T.; Kim D.; Osuka A. Closed Pentaaza[9]Helicene and Hexathia[9]/[5]Helicene: Oxidative Fusion Reactions of Ortho-Phenylene-Bridged Cyclic Hexapyrroles and Hexathiophenes. Angew. Chem., Int. Ed. 2017, 56, 14688–14693. 10.1002/anie.201708429. 28948686

[ref759] Yamamoto K.; Pandit P.; Higashibayashi S. Non-Planar [n]Cyclo-1,8-Carbazolylenes (N = 3,4,6) and [3]Cyclo-1,8-Carbazolylenyl B, P, PO, SiPh Complexes. Chem. - Eur. J. 2017, 23, 14011–14016. 10.1002/chem.201702853. 28733988

[ref760] Yasui M.; Ohtsu H.; Kawano M.; Hanaya K.; Sugai T.; Higashibayashi S. Dearomative Oxidative Rearrangement of [3]Cyclo-1,8-Carbazolylene. Chem. Lett. 2018, 47, 1357–1359. 10.1246/cl.180637.

[ref761] Kuroda Y.; Sakamoto Y.; Suzuki T.; Kayahara E.; Yamago S. Tetracyclo(2,7-Carbazole)s: Diatropicity and Paratropicity of Inner Regions of Nanohoops. J. Org. Chem. 2016, 81, 3356–3363. 10.1021/acs.joc.6b00425. 27015266

[ref762] Lucas F.; Sicard L.; Jeannin O.; Rault-Berthelot J.; Jacques E.; Quinton C.; Poriel C. [4]Cyclo-N-Ethyl-2,7-Carbazole: Synthesis, Structural, Electronic and Charge Transport Properties. Chem. - Eur. J. 2019, 25, 7740–7748. 10.1002/chem.201901066. 30946486

[ref763] Kayahara E.; Zhai X.; Yamago S. Synthesis and Physical Properties of [4]Cyclo-3,7-Dibenzo[b, d]Thiophene and Its S,S-Dioxide. Can. J. Chem. 2017, 95, 351–356. 10.1139/cjc-2016-0474.

[ref764] Shimasaki T.; Okajima S.; Ishikawa R.; Kawaguchi S.; Akimoto T.; Asano N.; Iwanaga T.; Watanabe M.; Teramoto N.; Shibata M. Synthesis and Properties of Fully Conjugated Macrocycles Composed of M-Diethynylene-Phenylene-Bridged Two Dibenzofuran, Dibenzothiophene and Carbazole Units. Tetrahedron 2018, 74, 2454–2465. 10.1016/j.tet.2018.03.072.

[ref765] Klajn J.; Stawski W.; Chmielewski P. J.; Cybińska J.; Pawlicki M. A Route to a Cyclobutane-Linked Double-Looped System via a Helical Macrocycle. Chem. Commun. 2019, 55, 4558–4561. 10.1039/C9CC01201J. 30931464

[ref766] Kawano S.; Ishida Y.; Tanaka K. Columnar Liquid-Crystalline Metallomacrocycles. J. Am. Chem. Soc. 2015, 137, 2295–2302. 10.1021/ja510585u. 25658844

[ref767] Kawano S.; Kato M.; Soumiya S.; Nakaya M.; Onoe J.; Tanaka K. Columnar Liquid Crystals from a Giant Macrocycle Mesogen. Angew. Chem., Int. Ed. 2018, 57, 167–171. 10.1002/anie.201709542. 29131500

[ref768] Dobscha J. R.; Debnath S.; Fadler R. E.; Fatila E. M.; Pink M.; Raghavachari K.; Flood A. H. Host-Host Interactions Control Self-Assembly and Switching of Triple and Double Decker Stacks of Tricarbazole Macrocycles Co-Assembled with Anti-Electrostatic Bisulfate Dimers. Chem. - Eur. J. 2018, 24, 9841–9852. 10.1002/chem.201800827. 29665108

[ref769] Dobscha J. R.; Castillo H. D.; Li Y.; Fadler R. E.; Taylor R. D.; Brown A. A.; Trainor C. Q.; Tait S. L.; Flood A. H. Sequence-Defined Macrocycles for Understanding and Controlling the Build-up of Hierarchical Order in Self-Assembled 2D Arrays. J. Am. Chem. Soc. 2019, 141, 17588–17600. 10.1021/jacs.9b06410. 31503483 PMC7461245

[ref770] Debnath S.; Yang J.; Ortoleva P.; Raghavachari K. Understanding the Origin of 2D Self-Assembly of Tricarbazole Macrocycles: An Integrated Quantum Mechanical/Molecular Dynamics Study. J. Phys. Chem. C 2019, 123, 17616–17623. 10.1021/acs.jpcc.9b04290.

[ref771] Castillo H. D.; Yang J.; Debnath S.; Dobscha J. R.; Trainor C. Q.; Mortensen R. D.; Raghavachari K.; Flood A. H.; Ortoleva P. J.; Tait S. L. Solution-Mediated Annealing Pathways Are Critical for Supramolecular Ordering of Complex Macrocycles at Surfaces. J. Phys. Chem. C 2020, 124, 6689–6699. 10.1021/acs.jpcc.9b11867.

[ref772] Jin S.; Kato S.; Nakamura Y. Synthesis, Self-Association, and Anion Recognition of Conjugated Macrocycles Composed of Carbazole and Triazolium Moieties. Chem. Lett. 2016, 45, 869–871. 10.1246/cl.160400.

[ref773] Ball M. L.; Zhang B.; Xu Q.; Paley D. W.; Ritter V. C.; Ng F.; Steigerwald M. L.; Nuckolls C. Influence of Molecular Conformation on Electron Transport in Giant, Conjugated Macrocycles. J. Am. Chem. Soc. 2018, 140, 10135–10139. 10.1021/jacs.8b06565. 30063344

[ref774] Phulwale B. V.; Mishra S. K.; Nečas M.; Mazal C. Phenanthrylene-Butadiynylene and Phenanthrylene-Thienylene Macrocycles: Synthesis, Structure, and Properties. J. Org. Chem. 2016, 81, 6244–6252. 10.1021/acs.joc.6b00814. 27398717

[ref775] Phulwale B. V.; Mishra S. K.; Mazal C. Synthesis and Properties of π-Conjugated Donor-Acceptor Macrocycles Derived from Phenanthrylene Building Blocks. Tetrahedron 2018, 74, 3616–3623. 10.1016/j.tet.2018.05.025.

[ref776] Kanagalatha R.; Rajakumar P.; Senthamil Selvi C.S.; Mohan N. Synthesis and Antimicrobicidal Activity of Phenothiazinophanes: A New Class of Permanent Fluorescence Sensing Stilbenophanes. Asian J. Chem. 2015, 27, 4373–4378. 10.14233/ajchem.2015.19128.

[ref777] Xie J.; Li X.; Wang S.; Li A.; Jiang L.; Zhu K. Heteroatom-Bridged Molecular Belts as Containers. Nat. Commun. 2020, 11, 3348 10.1038/s41467-020-17134-3. 32620853 PMC7335211

[ref778] Li G.; Gopalakrishna T. Y.; Phan H.; Herng T. S.; Ding J.; Wu J. From Open-Shell Singlet Diradicaloid to Closed-Shell Global Antiaromatic Macrocycles. Angew. Chem., Int. Ed. 2018, 57, 7166–7170. 10.1002/anie.201803949. 29673072

[ref779] Rana A.; Hong Y.; Gopalakrishna T. Y.; Phan H.; Herng T. S.; Yadav P.; Ding J.; Kim D.; Wu J. Stable Expanded Porphycene-Based Diradicaloid and Tetraradicaloid. Angew. Chem., Int. Ed. 2018, 57, 12534–12537. 10.1002/anie.201807411. 30088323

[ref780] Shouda T.; Nakanishi K.; Sasamori T.; Tokitoh N.; Kuramochi K.; Tsubaki K. Synthesis and Structures of Zigzag Shaped [12]Cyclo-p-Phenylene Composed of Dinaphthofuran Units and Biphenyl Units. J. Org. Chem. 2017, 82, 7850–7855. 10.1021/acs.joc.7b01053. 28675033

[ref781] Higashibayashi S. Synthesis of Flake-Shaped [3]Cyclo-4,6-Dibenzofuranylene. Chem. Lett. 2018, 47, 95–96. 10.1246/cl.170925.

[ref782] Kurata R.; Sakamaki D.; Ito A. Tetraaza[1.1.1.1]m, p, m, p-Cyclophane Diradical Dications Revisited: Tuning Spin States by Confronted Arenes. Org. Lett. 2017, 19, 3115–3118. 10.1021/acs.orglett.7b01229. 28571315

[ref783] Kurata R.; Sakamaki D.; Uebe M.; Kinoshita M.; Iwanaga T.; Matsumoto T.; Ito A. Isolable Triradical Trication of Hexaaza[1 _6_ ]Paracyclophane with Embedded 9,10-Anthrylenes: A Frustrated Three-Spin System. Org. Lett. 2017, 19, 4371–4374. 10.1021/acs.orglett.7b02088. 28783366

[ref784] Wang W.; Chen C.; Shu C.; Rajca S.; Wang X.; Rajca A. S = 1 Tetraazacyclophane Diradical Dication with Robust Stability: A Case of Low-Temperature One-Dimensional Antiferromagnetic Chain. J. Am. Chem. Soc. 2018, 140, 7820–7826. 10.1021/jacs.8b02415. 29863339

[ref785] Dong S.; Gopalakrishna T. Y.; Han Y.; Chi C. Cyclobis(7,8-(*Para* -quinodimethane)-4,4’-triphenylamine) and Its Cationic Species Showing Annulene-Like Global (Anti)Aromaticity. Angew. Chem., Int. Ed. 2019, 58, 11742–11746. 10.1002/anie.201905763. 31225671

[ref786] Li G.; Matsuno T.; Han Y.; Phan H.; Wu S.; Jiang Q.; Zou Y.; Isobe H.; Wu J. Benzidine/Quinoidal-Benzidine-Linked, Superbenzene-Based Π-Conjugated Chiral Macrocycles and Cyclophanes. Angew. Chem., Int. Ed. 2020, 59, 9727–9735. 10.1002/anie.202002447. 32187780

[ref787] Li Y.; Yagi A.; Itami K. Synthesis of Highly Twisted, Nonplanar Aromatic Macrocycles Enabled by an Axially Chiral 4,5-Diphenylphenanthrene Building Block. J. Am. Chem. Soc. 2020, 142, 3246–3253. 10.1021/jacs.9b13549. 31986018

[ref788] Kurosaki R.; Hayashi H.; Suzuki M.; Jiang J.; Hatanaka M.; Aratani N.; Yamada H. A Remarkably Strained Cyclopyrenylene Trimer That Undergoes Metal-Free Direct Oxygen Insertion into the Biaryl C-C σ-Bond. Chem. Sci. 2019, 10, 6785–6790. 10.1039/C9SC01777A. 31391899 PMC6640197

[ref789] Inoue R.; Hasegawa M.; Nishinaga T.; Yoza K.; Mazaki Y. Efficient Synthesis, Structure, and Complexation Studies of Electron-Donating Thiacalix[ *n* ]Dithienothiophene. Angew. Chem., Int. Ed. 2015, 54, 2734–2738. 10.1002/anie.201410970. 25641269

[ref790] Hasegawa M.; Takahashi K.; Inoue R.; Haga S.; Mazaki Y. Selenacalix[4]Dithienothiophene: Synthesis, Structure, and Complexation of a Cyclic Tetramer of Selenide-Bridging Dithienothiophene. Chem. - Asian J. 2019, 14, 1647–1650. 10.1002/asia.201801105. 30079592

[ref791] Landovský T.; Dvořáková H.; Eigner V.; Babor M.; Krupička M.; Kohout M.; Lhoták P. Chemoselective Oxidation of Phenoxathiin-Based Thiacalix[4]Arene and the Stereoselective Alkylation of Products. New J. Chem. 2018, 42, 20074–20086. 10.1039/C8NJ04690E.

[ref792] Vrzal L.; Kratochvílová-Šimánová M.; Landovský T.; Polívková K.; Budka J.; Dvořáková H.; Lhoták P. Application of RDC Enhanced NMR Spectroscopy in Structural Analysis of Thiacalix[4]Arene Derivatives. Org. Biomol. Chem. 2015, 13, 9610–9618. 10.1039/C5OB01424G. 26257213

[ref793] Landovský T.; Tichotová M.; Vrzal L.; Budka J.; Eigner V.; Dvořáková H.; Lhoták P. Structure Elucidation of Phenoxathiin-Based Thiacalix[4]Arene Conformations Using NOE and RDC Data. Tetrahedron 2018, 74, 902–907. 10.1016/j.tet.2018.01.020.

